# Supplementary material for: Genome-wide characterization of human minisatellite VNTRs: population-specific alleles and gene expression differences
Source: Nucleic Acids Res. 2021 Apr 13;49(8):4308–24. doi: 10.1093/nar/gkab224 (PMC8096271; doi:10.1093/nar/gkab224)
Supplement: gkab224_Supplemental_Files [file Reference_TRs.pdf › Reference_TRs.pdf]

TRid	chr	start	end	pattern	array	copy_number	Upstream	3prime	Intron	Exon	5prime	Downstream	CpG	TFBS	DNAse	Gene
182168468	chr1	99040	99115	33	76	2.303030	no	no	yes	no	no	no	no	no	no	yes
182168552	chr1	367479	367812	179	334	1.932961	no	no	yes	no	no	no	no	no	no	yes
182168705	chr1	714325	714426	50	102	2.040000	no	no	yes	no	no	no	no	no	no	yes
182168742	chr1	811234	811268	18	35	1.944444	no	no	yes	no	no	no	no	yes	no	yes
182168749	chr1	832658	832711	25	54	2.160000	no	no	yes	no	no	no	no	yes	no	yes
182168755	chr1	841914	841941	14	28	2.000000	no	yes	yes	no	no	no	no	no	yes	yes
182168756	chr1	844361	844403	22	43	1.954545	no	yes	yes	no	no	yes	no	no	no	yes
182168763	chr1	866415	867192	49	778	15.897959	no	yes	yes	no	no	yes	yes	yes	yes	yes
182168771	chr1	870168	870208	21	41	2.047619	yes	no	yes	no	yes	no	yes	yes	yes	yes
182168777	chr1	887236	887272	19	37	2.052632	no	no	no	no	no	no	no	no	no	no
182168793	chr1	904472	904743	60	272	4.533333	yes	no	no	no	no	no	yes	yes	yes	no
182168796	chr1	904971	905006	18	36	2.055556	no	no	yes	no	no	no	yes	yes	no	yes
182168797	chr1	905013	905114	28	102	3.642857	no	no	yes	no	no	no	yes	yes	no	yes
182168798	chr1	906875	907014	68	140	2.058824	no	no	yes	no	no	no	no	yes	yes	yes
182168805	chr1	908728	908812	39	85	2.200000	no	no	yes	no	no	no	no	yes	no	yes
182168807	chr1	908921	909899	45	979	22.000000	no	no	yes	no	no	no	yes	yes	no	yes
182168812	chr1	915597	915677	41	81	1.975610	no	no	yes	no	no	yes	no	yes	yes	yes
182168815	chr1	916351	916432	28	82	2.928571	no	no	no	no	no	yes	no	yes	no	no
182168820	chr1	930078	930152	21	75	3.571429	yes	no	yes	no	no	no	no	yes	no	yes
182168821	chr1	933962	934962	28	1001	35.750000	no	no	yes	no	no	no	yes	yes	no	yes
182168822	chr1	936235	936468	39	234	5.813953	no	no	yes	no	no	yes	yes	yes	no	yes
182168825	chr1	939399	939508	49	110	2.265306	yes	no	yes	yes	no	yes	no	yes	no	yes
182168827	chr1	947655	947744	45	90	2.000000	yes	no	yes	no	no	no	no	yes	no	yes
182168828	chr1	948647	948677	11	31	2.818182	no	no	yes	no	no	no	no	no	no	yes

182168833	chr1	950137	950265	70	129	1.885714	no	no	yes	no	no	no	no	yes	yes	yes
182168835	chr1	950307	950349	22	43	2.045455	no	no	yes	no	no	no	no	yes	yes	yes
182168846	chr1	951960	951995	19	36	2.000000	no	no	yes	no	no	no	no	yes	no	yes
182168848	chr1	956763	956895	65	133	2.046154	no	no	yes	yes	yes	no	no	yes	no	yes
182168853	chr1	964543	964844	38	302	8.105263	no	no	yes	no	no	yes	yes	yes	no	yes
182168856	chr1	968882	968909	14	28	2.000000	no	no	yes	no	no	no	no	no	no	yes
182168857	chr1	969334	969385	26	52	2.000000	no	no	yes	no	no	no	no	yes	yes	yes
182168863	chr1	969722	969765	22	44	2.000000	no	no	yes	no	no	no	no	yes	yes	yes
182168864	chr1	969760	969790	16	31	1.937500	no	no	yes	no	no	no	no	yes	no	yes
182168866	chr1	969959	970182	55	224	4.072727	yes	no	yes	no	no	no	no	yes	no	yes
182168872	chr1	976687	978181	32	1495	47.724998	yes	no	yes	no	no	yes	yes	yes	yes	yes
182168877	chr1	978296	978326	15	31	2.066667	no	no	yes	no	no	no	yes	yes	yes	yes
182168878	chr1	978367	978403	16	37	2.312500	no	no	yes	no	no	no	yes	yes	yes	yes
182168883	chr1	988687	989365	23	679	29.750000	no	no	no	no	no	no	no	yes	no	no
182168885	chr1	992884	993152	26	269	10.423077	no	no	no	no	no	no	no	yes	yes	no
182168887	chr1	996255	996867	66	613	9.313433	no	no	no	no	yes	no	no	yes	yes	yes
182168888	chr1	997611	997731	39	121	3.102564	no	no	no	no	yes	no	no	yes	yes	yes
182168889	chr1	998744	998784	14	41	2.928571	yes	no	no	no	no	yes	yes	yes	yes	no
182168890	chr1	999274	999312	21	39	1.904762	no	no	no	yes	yes	no	yes	yes	yes	yes
182168899	chr1	1015324	1015388	34	65	1.911765	no	no	no	no	no	yes	no	yes	yes	no
182168909	chr1	1019470	1019509	15	40	2.666667	yes	no	no	no	no	no	yes	yes	yes	no
182168913	chr1	1019933	1019990	28	58	2.107143	yes	no	no	no	no	no	yes	yes	yes	no
182168915	chr1	1025747	1025784	20	38	1.900000	no	no	yes	no	no	no	no	yes	no	yes
182168916	chr1	1028472	1029145	61	674	11.065574	no	no	yes	no	no	no	yes	yes	no	yes
182168917	chr1	1029366	1029446	28	81	2.806452	no	no	yes	no	no	no	no	yes	no	yes

182168925	chr1	1033758	1033850	45	93	2.111111	yes	no	yes	no	no	no	yes	yes	yes	yes
182168931	chr1	1040981	1041125	75	145	1.946667	no	no	yes	no	no	no	yes	yes	yes	yes
182168932	chr1	1041679	1041868	47	190	4.042553	no	no	yes	yes	no	yes	yes	yes	no	yes
182168935	chr1	1049043	1049197	84	155	1.857143	no	yes	yes	yes	no	no	yes	yes	no	yes
182168936	chr1	1052454	1052659	67	206	3.042253	no	yes	yes	no	no	no	no	yes	yes	yes
182168939	chr1	1052588	1052860	92	273	2.968085	no	yes	yes	no	no	no	no	yes	yes	yes
182168940	chr1	1053180	1053227	14	48	3.428571	no	yes	yes	no	no	no	no	yes	no	yes
182168941	chr1	1053207	1053290	37	84	2.270270	no	yes	yes	no	no	no	no	yes	no	yes
182168942	chr1	1055952	1055993	17	42	2.470588	no	yes	no	no	no	no	no	no	no	yes
182168946	chr1	1056319	1056530	82	212	2.585366	no	no	no	no	no	yes	yes	yes	yes	no
182168948	chr1	1056449	1056720	52	272	5.370370	no	no	no	no	no	yes	yes	yes	yes	no
182168953	chr1	1059629	1059683	13	55	4.307693	yes	no	no	no	no	no	yes	yes	yes	no
182168966	chr1	1064844	1065322	30	479	16.031250	no	no	yes	no	no	no	no	yes	no	yes
182168968	chr1	1067533	1067565	12	33	2.750000	no	no	yes	no	no	no	yes	yes	yes	yes
182168969	chr1	1068729	1068834	39	106	2.717949	no	no	yes	no	no	no	yes	yes	yes	yes
182168970	chr1	1069033	1069111	18	79	4.388889	no	no	yes	no	no	no	yes	yes	yes	yes
182168976	chr1	1075587	1075721	44	135	3.044445	no	no	no	no	no	no	no	no	no	no
182168977	chr1	1075853	1079429	30	3577	119.366669	no	no	no	no	no	no	yes	yes	yes	no
182168978	chr1	1080637	1081029	32	393	12.281250	no	no	no	no	no	yes	yes	yes	yes	no
182168979	chr1	1084543	1084640	51	98	1.960784	yes	no	yes	no	no	no	yes	yes	yes	yes
182168984	chr1	1087943	1087975	15	33	2.200000	yes	no	yes	no	no	no	yes	yes	no	yes
182168986	chr1	1089526	1089563	20	38	1.900000	no	no	yes	no	no	yes	no	yes	no	yes
182168987	chr1	1091690	1091821	38	132	3.400000	no	no	yes	no	yes	no	no	yes	yes	yes
182168989	chr1	1092310	1092345	18	36	2.000000	yes	no	yes	no	no	no	yes	yes	no	yes
182168990	chr1	1092682	1092733	22	52	2.363636	yes	no	yes	no	yes	no	no	yes	no	yes

182169004	chr1	1108048	1109117	95	1070	11.577319	no	no	yes	no	no	no	no	yes	no	yes
182169006	chr1	1112369	1112679	31	311	10.032258	no	no	yes	no	no	no	no	yes	yes	yes
182169009	chr1	1115477	1115745	79	269	3.345238	no	no	yes	no	no	no	yes	yes	no	yes
182169014	chr1	1120999	1121073	25	75	3.000000	no	no	no	no	no	no	no	no	no	no
182169017	chr1	1126558	1126747	27	190	7.037037	no	no	no	no	no	no	no	no	no	no
182169018	chr1	1128694	1128759	32	66	2.062500	no	no	no	no	no	no	no	yes	yes	no
182169020	chr1	1134209	1134296	42	88	2.095238	no	no	no	no	no	no	no	yes	no	no
182169021	chr1	1134740	1134857	59	118	2.033898	no	no	no	no	no	no	no	yes	yes	no
182169022	chr1	1136067	1136116	23	50	2.173913	yes	no	no	no	no	no	no	no	yes	no
182169023	chr1	1139071	1140713	61	1643	26.934425	no	no	yes	no	no	no	no	yes	no	yes
182169030	chr1	1142038	1142155	39	118	3.025641	no	no	yes	no	no	no	no	no	no	yes
182169031	chr1	1143243	1143479	47	237	5.042553	no	yes	no	no	no	no	no	yes	no	yes
182169036	chr1	1147127	1147449	36	323	9.027778	no	no	no	no	no	no	no	no	no	no
182169037	chr1	1148842	1149068	45	227	5.044445	no	no	no	no	no	no	no	yes	no	no
182169039	chr1	1149632	1149699	35	68	1.942857	no	no	no	no	no	no	no	no	no	no
182169041	chr1	1151817	1151885	31	69	2.225806	no	no	no	no	no	no	no	no	no	no
182169042	chr1	1152391	1153586	43	1196	27.813953	no	no	no	no	no	no	no	yes	yes	no
182169045	chr1	1156351	1156496	72	146	2.027778	no	no	no	no	no	no	no	yes	yes	no
182169047	chr1	1157286	1157347	20	62	3.150000	no	no	no	no	no	no	no	yes	yes	no
182169049	chr1	1157327	1157428	9	102	11.300000	no	no	no	no	no	no	no	yes	no	no
182169052	chr1	1157422	1157500	42	79	1.904762	no	no	no	no	no	no	no	yes	yes	no
182169053	chr1	1164198	1164242	23	45	1.956522	no	no	no	no	no	no	yes	yes	yes	no
182169055	chr1	1166509	1166560	12	52	4.333333	yes	no	no	no	no	no	no	yes	yes	no
182169057	chr1	1168010	1168133	35	124	3.542857	yes	no	no	no	no	yes	no	yes	yes	no
182169062	chr1	1170468	1170577	49	110	2.320000	no	no	no	no	no	yes	no	yes	yes	no

182169071	chr1	1176112	1176395	83	284	3.614458	no	no	yes	no	yes	no	no	yes	yes	yes
182169073	chr1	1176504	1176549	18	46	2.555556	no	no	yes	no	no	no	no	yes	yes	yes
182169079	chr1	1177131	1177197	22	67	3.045455	no	no	yes	no	no	no	no	yes	yes	yes
182169083	chr1	1181592	1181629	18	38	2.111111	no	no	yes	no	no	yes	no	yes	yes	yes
182169084	chr1	1184191	1184227	17	37	2.176471	yes	no	yes	no	no	yes	no	yes	no	yes
182169085	chr1	1184527	1184566	21	40	2.000000	yes	no	yes	no	no	yes	no	yes	no	yes
182169086	chr1	1184656	1184980	108	325	3.009259	yes	no	yes	yes	no	yes	no	yes	no	yes
182169089	chr1	1192362	1192866	24	505	20.814816	no	no	yes	no	no	no	no	yes	no	yes
182169093	chr1	1197857	1197887	7	31	4.428571	no	yes	no	no	no	yes	no	yes	no	yes
182169094	chr1	1198070	1198176	50	107	2.200000	no	no	no	no	no	yes	no	yes	yes	no
182169096	chr1	1201779	1202830	38	1052	27.684210	no	no	no	no	no	yes	no	yes	yes	no
182169097	chr1	1204248	1204297	24	50	2.083333	no	no	yes	no	no	no	no	yes	yes	yes
182169098	chr1	1211042	1211301	43	260	6.046512	no	no	no	no	no	yes	no	no	yes	no
182169103	chr1	1214189	1214236	20	48	2.400000	yes	no	no	no	no	no	no	yes	yes	no
182169110	chr1	1222923	1223153	50	231	4.673077	no	no	yes	no	no	no	yes	yes	yes	yes
182169112	chr1	1223130	1223189	25	60	2.444444	no	no	yes	no	no	no	yes	yes	yes	yes
182169115	chr1	1225687	1225780	29	94	3.241379	no	no	yes	no	no	no	no	yes	yes	yes
182169118	chr1	1227284	1227499	29	216	7.448276	no	no	yes	no	yes	no	no	yes	no	yes
182169120	chr1	1229064	1229199	27	136	5.148148	yes	no	yes	no	no	no	no	yes	yes	yes
182169122	chr1	1240551	1240603	25	53	2.160000	no	no	no	no	no	no	no	yes	no	no
182169128	chr1	1244879	1244914	19	36	1.947368	yes	no	yes	no	no	no	no	yes	yes	yes
182169129	chr1	1246874	1246902	13	29	2.307692	yes	no	no	no	no	no	yes	yes	no	no
182169130	chr1	1248055	1249687	66	1633	24.757576	no	no	no	no	no	yes	no	yes	yes	no
182169131	chr1	1258433	1258474	22	42	2.000000	no	no	yes	no	no	no	no	yes	yes	yes
182169139	chr1	1259304	1259394	39	91	2.384615	no	no	yes	no	no	no	yes	yes	yes	yes

182169140	chr1	1259918	1259951	17	34	2.058824	no	no	yes	no	no	no	no	yes	no	yes
182169141	chr1	1260409	1260504	49	96	2.000000	no	no	yes	no	no	no	no	yes	yes	yes
182169146	chr1	1267525	1267650	64	126	2.015625	no	no	yes	no	no	yes	no	yes	no	yes
182169147	chr1	1269100	1269412	79	313	3.962025	no	no	yes	no	no	no	no	yes	no	yes
182169148	chr1	1270802	1270891	44	90	2.068182	no	no	yes	no	no	no	no	yes	no	yes
182169150	chr1	1272609	1272717	40	109	2.725000	no	no	yes	no	no	no	no	yes	yes	yes
182169152	chr1	1275584	1275616	16	33	2.062500	no	no	yes	no	no	no	no	no	yes	yes
182169155	chr1	1279130	1279200	35	71	2.028571	no	yes	yes	no	no	no	no	yes	no	yes
182169163	chr1	1284106	1284226	53	121	2.283019	no	no	yes	no	no	no	no	yes	yes	yes
182169164	chr1	1284216	1284314	40	99	2.487805	no	no	yes	no	no	no	no	yes	yes	yes
182169168	chr1	1284494	1284683	28	190	6.785714	no	no	yes	no	no	no	no	yes	no	yes
182169169	chr1	1288033	1288099	34	67	2.000000	no	yes	yes	yes	no	no	yes	yes	no	yes
182169170	chr1	1288182	1290253	18	2072	111.818184	no	no	yes	no	no	no	yes	yes	yes	yes
182169171	chr1	1290984	1291017	17	34	2.000000	no	no	yes	no	no	yes	yes	yes	no	yes
182169172	chr1	1293732	1293796	20	65	3.250000	no	no	yes	no	no	yes	yes	yes	yes	yes
182169173	chr1	1293996	1294036	15	41	2.800000	no	no	yes	no	no	yes	yes	yes	yes	yes
182169174	chr1	1296327	1296434	53	108	2.037736	yes	no	yes	yes	yes	yes	no	yes	no	yes
182169176	chr1	1296807	1296838	16	32	2.000000	yes	no	yes	no	no	yes	yes	yes	no	yes
182169178	chr1	1297063	1297807	46	745	16.543478	yes	no	yes	no	no	yes	yes	yes	no	yes
182169181	chr1	1298477	1298555	39	79	2.051282	yes	no	yes	no	no	yes	no	yes	no	yes
182169182	chr1	1299411	1299479	29	69	2.379310	yes	no	yes	no	yes	yes	no	yes	no	yes
182169183	chr1	1303504	1303541	20	38	2.050000	yes	no	yes	no	yes	no	no	yes	yes	yes
182169184	chr1	1303587	1303754	61	168	2.686567	yes	no	yes	no	yes	no	no	yes	yes	yes
182169185	chr1	1307934	1308089	52	156	2.928571	yes	no	yes	no	no	no	yes	yes	yes	yes
182169187	chr1	1308179	1308209	11	31	2.818182	yes	no	yes	no	no	no	yes	yes	yes	yes

182169189	chr1	1310761	1310893	52	133	2.557692	no	yes	yes	no	no	yes	no	yes	no	yes
182169191	chr1	1314517	1314598	22	82	3.772727	yes	no	yes	no	yes	yes	no	yes	no	yes
182169192	chr1	1315132	1315209	36	78	2.166667	yes	no	yes	no	yes	yes	no	yes	no	yes
182169199	chr1	1322537	1322587	22	51	2.363636	no	no	yes	no	no	yes	no	no	no	yes
182169200	chr1	1322623	1322664	17	42	2.529412	no	no	yes	no	no	yes	no	no	no	yes
182169204	chr1	1323098	1323132	18	35	2.000000	no	yes	yes	yes	yes	no	no	yes	no	yes
182169207	chr1	1324755	1324799	24	45	1.916667	yes	yes	no	no	no	no	yes	yes	yes	yes
182169209	chr1	1329540	1329582	13	43	3.307692	no	no	no	no	no	yes	no	yes	yes	no
182169210	chr1	1329690	1329719	15	30	2.000000	no	no	no	no	no	yes	no	yes	yes	no
182169211	chr1	1329803	1329840	20	38	1.900000	no	no	no	no	no	yes	no	yes	no	no
182169216	chr1	1333720	1333761	15	42	2.800000	no	no	no	yes	no	no	no	yes	yes	yes
182169218	chr1	1337837	1337944	33	108	3.272727	no	no	yes	no	no	yes	no	yes	yes	yes
182169222	chr1	1339866	1340052	39	187	4.775000	yes	no	yes	yes	yes	no	yes	no	no	yes
182169228	chr1	1341175	1341234	22	60	2.727273	yes	no	yes	no	no	yes	no	yes	yes	yes
182169231	chr1	1341540	1341572	10	33	3.300000	no	no	yes	no	no	yes	no	yes	yes	yes
182169233	chr1	1342858	1343053	97	196	2.020618	no	no	yes	no	no	no	no	yes	no	yes
182169234	chr1	1344938	1345063	38	126	3.250000	no	no	yes	no	no	no	no	yes	yes	yes
182169236	chr1	1349975	1351458	40	1484	37.099998	yes	no	no	no	no	no	yes	yes	yes	no
182169238	chr1	1355387	1355454	28	68	2.500000	yes	no	yes	yes	yes	no	yes	yes	no	yes
182169244	chr1	1356309	1356519	110	211	1.945455	yes	no	yes	no	no	no	no	yes	yes	yes
182169249	chr1	1360904	1360972	34	69	2.029412	no	no	yes	no	no	no	no	yes	yes	yes
182169250	chr1	1360976	1361026	26	51	2.038461	no	no	yes	no	no	no	no	yes	yes	yes
182169252	chr1	1361034	1361078	24	45	1.958333	no	no	yes	no	no	no	no	yes	yes	yes
182169253	chr1	1361123	1361162	21	40	1.904762	no	no	yes	no	no	no	no	yes	yes	yes
182169257	chr1	1361182	1361237	27	56	2.148148	no	no	yes	no	no	no	no	yes	yes	yes

182169270	chr1	1366220	1366265	22	46	2.090909	no	no	no	no	no	no	no	yes	yes	no
182169276	chr1	1366344	1366400	28	57	2.107143	no	no	no	no	no	no	no	yes	yes	no
182169277	chr1	1366411	1366457	24	47	1.958333	no	no	no	no	no	no	no	yes	no	no
182169283	chr1	1366541	1366577	16	37	2.312500	no	no	no	no	no	no	no	yes	no	no
182169284	chr1	1366660	1366726	35	67	2.000000	no	no	no	no	no	no	no	yes	yes	no
182169292	chr1	1367001	1367256	100	256	2.650000	no	no	no	no	no	no	no	yes	yes	no
182169299	chr1	1367484	1367531	24	48	2.000000	no	no	no	no	no	no	no	yes	yes	no
182169304	chr1	1382231	1382905	39	675	17.450001	no	no	no	no	no	no	no	yes	no	no
182169307	chr1	1387137	1387178	21	42	2.000000	no	yes	no	no	yes	yes	no	yes	yes	yes
182169309	chr1	1391600	1391654	18	55	3.055556	yes	no	yes	no	yes	yes	no	yes	no	yes
182169316	chr1	1407575	1407621	24	47	1.958333	yes	no	no	no	no	no	no	yes	no	no
182169324	chr1	1420348	1420376	13	29	2.230769	no	yes	yes	yes	no	no	yes	yes	no	yes
182169327	chr1	1420662	1420788	54	127	2.363636	no	no	yes	no	no	no	no	yes	no	yes
182169331	chr1	1427959	1428209	85	251	2.943182	no	no	no	no	no	yes	no	yes	yes	no
182169335	chr1	1431051	1431170	45	120	2.688889	no	no	yes	no	no	no	no	yes	yes	yes
182169338	chr1	1433011	1433076	16	66	4.125000	no	no	yes	no	no	no	no	yes	no	yes
182169339	chr1	1439711	1439755	21	45	2.238095	no	yes	no	yes	no	no	yes	yes	yes	yes
182169342	chr1	1442839	1442939	32	101	3.156250	no	yes	no	no	no	yes	no	yes	no	yes
182169344	chr1	1444347	1444450	50	104	2.080000	no	no	no	no	no	no	no	no	no	no
182169345	chr1	1445170	1445294	41	125	3.023809	no	no	no	no	no	no	no	no	no	no
182169356	chr1	1461590	1461786	39	197	5.051282	yes	no	yes	no	no	no	no	yes	yes	yes
182169373	chr1	1479373	1479448	26	76	2.923077	no	no	yes	no	no	no	no	yes	yes	yes
182169378	chr1	1479838	1479873	14	36	2.571429	no	no	yes	no	no	no	no	yes	yes	yes
182169383	chr1	1482041	1482081	17	41	2.411765	no	no	yes	no	no	no	yes	yes	no	yes
182169384	chr1	1481830	1482059	85	230	2.705882	no	no	yes	no	no	no	yes	yes	yes	yes

182169385	chr1	1487547	1487650	54	104	1.925926	no	no	yes	no	no	no	no	yes	yes	yes
182169388	chr1	1497155	1497217	31	63	2.032258	no	yes	no	no	no	yes	no	no	no	yes
182169405	chr1	1511880	1511917	15	38	2.533333	yes	no	no	no	no	no	yes	yes	yes	no
182169406	chr1	1512481	1512523	13	43	3.214286	yes	no	yes	no	no	no	yes	yes	yes	yes
182169409	chr1	1518003	1518039	15	37	2.466667	no	no	yes	no	no	yes	no	yes	no	yes
182169412	chr1	1518214	1518278	33	65	1.969697	no	no	yes	no	no	yes	no	yes	no	yes
182169414	chr1	1518352	1518389	14	38	2.714286	no	no	yes	no	no	yes	no	yes	yes	yes
182169416	chr1	1518578	1518624	18	47	2.550000	no	no	yes	no	no	yes	no	yes	yes	yes
182169419	chr1	1518706	1518879	75	174	2.373333	no	no	yes	no	no	yes	no	yes	yes	yes
182169424	chr1	1528365	1528444	39	80	2.051282	no	no	yes	no	no	no	no	yes	no	yes
182169430	chr1	1535042	1535095	19	54	2.842105	no	yes	no	no	no	yes	no	yes	no	yes
182169431	chr1	1535145	1535182	18	38	2.166667	no	yes	no	no	no	yes	no	yes	no	yes
182169436	chr1	1540218	1540274	25	57	2.320000	no	no	yes	no	no	no	yes	yes	yes	yes
182169439	chr1	1540643	1540682	14	40	2.866667	yes	no	no	no	no	yes	yes	yes	yes	no
182169443	chr1	1543588	1543831	25	244	9.880000	no	no	yes	no	no	yes	no	yes	yes	yes
182169449	chr1	1554142	1554407	44	266	6.045455	no	no	yes	no	no	no	yes	yes	no	yes
182169452	chr1	1559207	1559300	47	94	2.000000	no	no	yes	no	no	no	no	yes	yes	yes
182169453	chr1	1562446	1562656	77	211	2.730769	no	yes	yes	no	no	no	no	yes	yes	yes
182169455	chr1	1564387	1564411	12	25	2.083333	no	yes	yes	no	no	no	no	yes	yes	yes
182169464	chr1	1571479	1571508	15	30	2.000000	no	no	yes	no	no	no	no	yes	no	yes
182169487	chr1	1594370	1594453	43	84	1.953488	no	no	no	no	no	no	no	yes	yes	no
182169495	chr1	1597454	1597491	15	38	2.533333	no	no	no	no	no	yes	no	yes	yes	no
182169498	chr1	1600302	1600339	19	38	2.000000	yes	yes	no	no	no	no	yes	yes	yes	yes
182169501	chr1	1604654	1604766	40	113	2.829268	no	no	no	no	no	no	no	no	no	no
182169506	chr1	1608035	1608111	18	77	4.444445	no	no	no	no	no	no	no	no	no	no

182169512	chr1	1615889	1615914	12	26	2.166667	yes	yes	yes	no	yes	no	yes	yes	yes	yes
182169513	chr1	1621504	1621553	26	50	1.923077	no	no	yes	no	no	no	no	yes	no	yes
182169514	chr1	1621789	1621816	13	28	2.153846	no	no	yes	no	no	no	no	yes	no	yes
182169515	chr1	1623375	1623460	42	86	2.047619	yes	yes	yes	yes	yes	no	yes	no	no	yes
182169517	chr1	1627439	1627604	51	166	3.333333	yes	yes	yes	yes	no	yes	yes	yes	yes	yes
182169519	chr1	1627823	1627955	36	133	3.694444	yes	yes	yes	yes	no	yes	yes	yes	yes	yes
182169521	chr1	1629847	1629871	7	25	3.571429	no	no	yes	no	no	yes	yes	yes	yes	yes
182169529	chr1	1650304	1650330	12	27	2.250000	no	no	yes	no	no	no	no	no	no	yes
182169531	chr1	1651314	1652350	108	1037	9.629311	no	no	yes	no	no	no	no	no	no	yes
182169536	chr1	1665068	1665115	24	48	2.041667	no	yes	yes	no	yes	no	no	yes	yes	yes
182169547	chr1	1678984	1679018	18	35	1.944444	no	no	yes	no	no	no	no	yes	yes	yes
182169552	chr1	1683742	1683786	21	45	2.142857	no	no	yes	no	no	no	no	yes	yes	yes
182169558	chr1	1685757	1685794	18	38	2.166667	no	no	yes	no	no	no	no	no	yes	yes
182169566	chr1	1692348	1692424	28	77	2.821429	no	no	yes	no	no	no	yes	yes	yes	yes
182169568	chr1	1693192	1693225	14	34	2.428571	yes	no	no	no	no	no	yes	yes	yes	no
182169577	chr1	1696852	1697023	77	172	2.230769	no	no	no	no	no	no	no	yes	yes	no
182169579	chr1	1699599	1699638	9	40	4.444445	yes	no	no	no	no	no	no	no	no	no
182169588	chr1	1717573	1718634	483	1062	2.212679	no	no	yes	no	no	yes	no	no	no	yes
182169590	chr1	1718972	1719165	108	194	1.814815	no	no	yes	no	no	yes	no	no	no	yes
182169593	chr1	1721247	1721536	112	290	2.663717	no	no	yes	no	no	yes	no	no	yes	yes
182169609	chr1	1748582	1748665	43	84	1.953488	no	no	no	no	no	no	no	yes	no	no
182169616	chr1	1759265	1759379	57	115	2.017544	yes	no	yes	no	no	no	no	yes	yes	yes
182169617	chr1	1760682	1760730	24	49	2.041667	no	no	yes	no	no	yes	no	yes	no	yes
182169620	chr1	1765455	1765487	9	33	3.666667	no	no	yes	no	no	no	no	no	no	yes
182169628	chr1	1771090	1771129	18	40	2.277778	no	no	yes	no	no	no	no	yes	no	yes

182169631	chr1	1778225	1778260	17	36	2.176471	no	no	yes	no	no	yes	yes	yes	yes	yes
182169636	chr1	1785737	1785772	10	36	3.700000	no	yes	no	no	no	no	no	no	no	yes
182169652	chr1	1826861	1826921	27	61	2.333333	no	no	yes	no	no	no	no	yes	yes	yes
182169678	chr1	1872530	1872606	37	77	2.081081	no	no	yes	no	no	no	no	yes	no	yes
182169686	chr1	1894756	1894832	36	77	2.166667	no	no	no	no	no	no	no	yes	yes	no
182169691	chr1	1898218	1898274	29	57	1.965517	no	no	no	no	no	no	no	yes	yes	no
182169693	chr1	1898604	1898693	21	90	4.380952	no	no	no	no	no	no	no	yes	yes	no
182169706	chr1	1909151	1909194	19	44	2.368421	no	no	no	no	no	no	yes	yes	yes	no
182169707	chr1	1909246	1909303	18	58	3.222222	no	no	no	no	no	no	no	yes	yes	no
182169710	chr1	1919189	1919227	9	39	4.333333	yes	no	no	yes	yes	no	yes	yes	no	yes
182169712	chr1	1919317	1919353	16	37	2.312500	yes	no	no	no	no	no	yes	yes	yes	no
182169713	chr1	1919424	1919594	78	171	2.205128	yes	no	no	no	no	no	yes	yes	yes	no
182169718	chr1	1924343	1924398	27	56	2.148148	no	no	yes	yes	yes	no	no	no	no	yes
182169720	chr1	1927109	1927134	8	26	3.250000	no	no	yes	no	yes	no	no	yes	no	yes
182169721	chr1	1928089	1928375	33	287	8.696970	yes	no	yes	no	no	no	no	yes	yes	yes
182169724	chr1	1929385	1929454	23	70	3.040000	no	no	yes	no	no	no	no	yes	yes	yes
182169733	chr1	1934206	1935602	34	1397	42.184212	no	no	yes	no	no	no	no	yes	no	yes
182169742	chr1	1938423	1938475	22	53	2.409091	no	no	yes	no	no	no	no	yes	no	yes
182169747	chr1	1944169	1944341	76	173	2.276316	no	no	yes	yes	no	no	yes	yes	yes	yes
182169751	chr1	1945860	1945943	39	84	2.205128	no	no	yes	no	no	yes	no	yes	no	yes
182169759	chr1	1955019	1955089	18	71	3.944444	no	no	yes	no	yes	yes	yes	yes	yes	yes
182169766	chr1	1968886	1969023	69	138	2.014493	no	no	yes	no	no	no	no	no	no	yes
182169774	chr1	1978899	1979943	58	1045	18.017241	no	no	yes	no	no	no	yes	yes	yes	yes
182169775	chr1	1980048	1980496	73	449	6.178082	no	no	yes	no	no	no	yes	no	yes	yes
182169776	chr1	1980830	1980869	16	40	2.500000	no	no	yes	no	no	no	yes	yes	yes	yes

182169777	chr1	1981392	1982441	35	1050	29.944445	no	no	yes	no	no	no	yes	no	yes	yes
182169780	chr1	1984574	1984697	39	124	3.150000	no	no	yes	no	yes	no	no	yes	no	yes
182169782	chr1	1988817	1988873	20	57	2.850000	no	no	yes	no	no	no	no	yes	yes	yes
182169784	chr1	1989748	1989850	52	103	2.000000	no	no	yes	no	no	no	no	yes	no	yes
182169791	chr1	2001024	2001153	60	130	2.213115	no	no	yes	no	no	no	no	no	no	yes
182169800	chr1	2016288	2016319	15	32	2.133333	yes	no	no	no	no	no	no	no	no	no
182169806	chr1	2019274	2019303	11	30	2.818182	yes	no	no	no	no	no	yes	yes	yes	no
182169808	chr1	2028302	2028396	22	95	4.318182	yes	no	yes	no	no	no	yes	no	yes	yes
182169809	chr1	2031598	2031738	35	141	4.028572	no	no	no	no	no	yes	no	no	yes	no
182169823	chr1	2042030	2042152	66	123	1.909091	no	no	no	no	no	no	no	yes	no	no
182169825	chr1	2042141	2042207	23	67	2.840000	no	no	no	no	no	no	no	yes	no	no
182169827	chr1	2043338	2043400	27	63	2.333333	no	no	no	no	no	no	yes	yes	yes	no
182169838	chr1	2050219	2050262	17	44	2.588235	yes	no	no	no	no	no	yes	yes	yes	no
182169846	chr1	2070725	2070821	41	97	2.365854	no	no	yes	no	no	no	no	no	no	yes
182169847	chr1	2080322	2080365	18	44	2.421053	no	no	yes	no	no	no	no	yes	no	yes
182169850	chr1	2082818	2082948	66	131	2.030303	no	no	yes	no	no	no	no	no	no	yes
182169852	chr1	2085550	2085823	56	274	4.928571	no	no	yes	no	no	no	no	no	no	yes
182169854	chr1	2091923	2091947	11	25	2.272727	no	no	yes	no	no	no	no	yes	no	yes
182169855	chr1	2094355	2094795	49	441	9.000000	no	no	yes	no	no	no	yes	yes	no	yes
182169859	chr1	2097556	2097678	66	123	1.878788	no	no	yes	no	no	no	no	yes	no	yes
182169862	chr1	2099023	2099115	24	93	3.875000	no	no	yes	no	no	no	no	yes	no	yes
182169866	chr1	2106392	2106875	58	484	8.322034	no	no	yes	no	no	no	no	no	yes	yes
182169868	chr1	2107705	2107740	15	36	2.400000	no	no	yes	no	no	no	no	yes	no	yes
182169870	chr1	2107745	2107972	54	228	4.407407	no	no	yes	no	no	no	no	yes	no	yes
182169876	chr1	2110022	2110306	95	285	3.000000	no	no	yes	no	no	no	no	no	no	yes

182169877	chr1	2110574	2110609	19	36	1.947368	no	no	yes	no	no	no	no	no	no	yes
182169878	chr1	2110783	2110846	34	64	1.911765	yes	no	yes	no	no	no	no	yes	yes	yes
182169880	chr1	2113218	2113246	13	29	2.230769	no	no	yes	no	no	no	no	no	no	yes
182169886	chr1	2121433	2121501	22	69	3.136364	no	no	yes	no	no	no	no	yes	yes	yes
182169892	chr1	2130904	2130981	38	78	2.105263	no	no	yes	no	no	no	no	yes	yes	yes
182169895	chr1	2133462	2133567	36	106	2.972973	yes	no	yes	no	no	no	yes	yes	yes	yes
182169899	chr1	2141863	2142496	42	634	15.069767	no	no	yes	no	yes	no	no	no	no	yes
182169900	chr1	2146732	2146782	26	51	1.961538	no	no	yes	no	no	yes	no	no	yes	yes
182169901	chr1	2146903	2146943	19	41	2.157895	no	no	yes	no	no	yes	no	no	no	yes
182169909	chr1	2150312	2150337	13	26	2.000000	no	no	yes	no	no	no	no	no	no	yes
182169911	chr1	2155030	2155071	18	42	2.333333	no	no	yes	no	no	no	no	yes	no	yes
182169918	chr1	2155633	2155756	64	124	1.937500	no	no	yes	no	no	no	no	yes	no	yes
182169920	chr1	2162870	2162974	35	105	3.057143	no	no	yes	no	no	no	no	yes	no	yes
182169921	chr1	2165874	2165954	41	81	1.975610	no	no	yes	no	no	no	no	yes	yes	yes
182169923	chr1	2166540	2166740	59	201	3.403226	no	no	yes	no	no	no	no	yes	yes	yes
182169929	chr1	2172839	2173021	45	183	4.066667	no	no	yes	no	no	no	yes	yes	no	yes
182169934	chr1	2175349	2175515	93	167	1.827957	no	no	yes	no	no	no	no	yes	no	yes
182169936	chr1	2175554	2175585	15	32	2.133333	no	no	yes	no	no	no	no	yes	no	yes
182169938	chr1	2177107	2177197	48	91	1.958333	no	no	yes	no	no	no	no	yes	no	yes
182169939	chr1	2177871	2177949	25	79	3.160000	no	no	yes	no	no	no	no	yes	no	yes
182169941	chr1	2180317	2180546	125	230	1.840000	no	no	yes	no	no	no	no	yes	yes	yes
182169946	chr1	2182088	2182124	15	37	2.466667	no	no	yes	no	yes	yes	no	yes	no	yes
182169953	chr1	2183402	2183529	34	128	3.631579	no	no	yes	no	no	yes	no	yes	no	yes
182169959	chr1	2189599	2189663	28	65	2.321429	no	yes	yes	no	yes	no	yes	yes	yes	yes
182169967	chr1	2204888	2205033	42	146	3.441860	no	no	yes	no	no	no	yes	yes	yes	yes

182169972	chr1	2205027	2205183	81	157	1.975309	no	no	yes	no	no	no	yes	yes	yes	yes
182169974	chr1	2207111	2207142	15	32	2.133333	no	no	yes	no	no	no	no	yes	yes	yes
182169981	chr1	2213587	2213671	30	85	2.833333	yes	no	yes	no	no	no	no	yes	yes	yes
182169983	chr1	2213731	2213875	66	145	2.212121	no	no	yes	no	no	no	no	yes	yes	yes
182169998	chr1	2228145	2228208	24	64	2.750000	yes	no	no	no	no	no	yes	yes	no	no
182170003	chr1	2228467	2228526	31	60	1.967742	no	no	no	no	yes	no	yes	yes	no	yes
182170005	chr1	2228957	2229025	37	69	1.918919	no	no	no	yes	no	no	yes	yes	yes	yes
182170012	chr1	2249810	2249846	19	37	2.052632	no	no	yes	no	no	no	no	yes	yes	yes
182170013	chr1	2253222	2253338	29	117	4.034483	no	no	yes	no	no	no	no	no	no	yes
182170017	chr1	2256183	2256298	59	116	2.000000	no	no	yes	no	no	no	no	yes	yes	yes
182170020	chr1	2261780	2262599	70	820	11.516854	no	no	yes	no	no	no	no	yes	no	yes
182170025	chr1	2269776	2270031	36	256	7.111111	no	no	yes	no	no	no	no	yes	no	yes
182170026	chr1	2272819	2272871	22	53	2.409091	no	no	yes	no	no	no	no	yes	yes	yes
182170027	chr1	2273214	2273379	83	166	2.000000	no	no	yes	no	no	no	no	yes	yes	yes
182170029	chr1	2277631	2277815	71	185	2.633803	no	no	yes	no	no	no	no	yes	no	yes
182170032	chr1	2277729	2277906	90	178	2.044445	no	no	yes	no	no	no	no	yes	no	yes
182170035	chr1	2277965	2278031	32	67	2.125000	no	no	yes	no	no	no	no	yes	no	yes
182170036	chr1	2280564	2282544	43	1981	45.977272	no	no	yes	no	no	no	yes	yes	no	yes
182170041	chr1	2289468	2289636	84	169	2.047619	no	no	yes	no	no	no	no	yes	no	yes
182170042	chr1	2292268	2292296	15	29	1.933333	no	no	yes	no	no	no	no	no	no	yes
182170045	chr1	2302169	2302284	58	116	2.000000	no	no	yes	no	no	no	no	yes	yes	yes
182170046	chr1	2308442	2308473	15	32	2.133333	no	yes	no	no	no	no	no	no	no	yes
182170047	chr1	2309635	2309872	39	238	6.121951	no	yes	no	no	no	no	no	no	no	yes
182170049	chr1	2314786	2314831	20	46	2.300000	yes	no	no	no	no	no	yes	yes	no	no
182170052	chr1	2315673	2315718	24	46	1.958333	no	yes	no	no	no	no	no	yes	yes	yes

182170053	chr1	2315721	2315750	15	30	2.000000	no	yes	no	no	no	no	no	yes	yes	yes
182170055	chr1	2317155	2317199	22	45	2.045455	no	no	yes	no	no	no	no	yes	no	yes
182170059	chr1	2317361	2317398	19	38	2.000000	no	no	yes	no	no	no	no	no	yes	yes
182170064	chr1	2325192	2325230	16	39	2.437500	no	no	yes	no	no	yes	no	yes	no	yes
182170076	chr1	2329966	2330005	18	40	2.222222	no	no	yes	no	no	no	no	yes	no	yes
182170077	chr1	2331795	2332031	17	237	14.529411	no	no	yes	no	no	yes	yes	yes	yes	yes
182170079	chr1	2333394	2333432	20	39	2.050000	no	no	yes	no	no	no	no	yes	no	yes
182170086	chr1	2345975	2346214	26	240	9.074074	no	no	yes	no	no	no	no	yes	no	yes
182170089	chr1	2347008	2347099	37	92	2.513514	no	no	yes	no	no	no	no	no	no	yes
182170095	chr1	2348656	2348824	90	169	1.877778	no	no	yes	no	no	no	no	yes	no	yes
182170097	chr1	2348849	2348895	20	47	2.350000	no	no	yes	no	no	no	no	yes	no	yes
182170106	chr1	2373256	2373291	18	36	2.111111	no	no	yes	no	no	no	no	no	yes	yes
182170108	chr1	2384040	2384181	72	142	1.986111	no	no	yes	no	no	no	no	yes	no	yes
182170111	chr1	2393802	2393841	18	40	2.277778	yes	no	yes	no	no	no	no	yes	yes	yes
182170112	chr1	2398172	2398324	49	153	3.122449	no	no	yes	no	no	no	no	yes	no	yes
182170125	chr1	2427769	2427841	17	73	4.294117	no	no	yes	no	no	no	no	yes	no	yes
182170126	chr1	2435455	2435492	7	38	5.428571	no	no	yes	no	no	no	no	no	no	yes
182170129	chr1	2437566	2437594	15	29	1.933333	no	no	yes	no	no	no	no	yes	no	yes
182170130	chr1	2440391	2440733	69	343	5.000000	no	no	yes	no	no	no	yes	yes	yes	yes
182170131	chr1	2441213	2441249	10	37	3.700000	no	no	yes	no	no	no	no	no	no	yes
182170132	chr1	2443275	2443342	34	68	2.000000	no	no	yes	no	no	no	no	yes	yes	yes
182170133	chr1	2446730	2446792	18	63	3.555556	no	no	yes	no	no	no	no	no	no	yes
182170135	chr1	2448698	2448764	33	67	2.030303	no	no	yes	no	no	no	no	no	yes	yes
182170137	chr1	2450469	2450511	16	43	2.647059	no	no	yes	no	no	no	no	no	no	yes
182170143	chr1	2451413	2451456	23	44	1.913043	no	no	yes	no	no	no	no	no	yes	yes

182170144	chr1	2452945	2453081	68	137	2.029412	no	no	yes	no	no	no	no	yes	yes	yes
182170145	chr1	2453942	2454012	25	71	2.840000	no	no	yes	no	no	no	no	yes	no	yes
182170146	chr1	2459276	2459737	16	462	28.875000	no	no	yes	no	no	no	no	yes	no	yes
182170149	chr1	2465566	2465607	14	42	3.000000	no	no	yes	no	no	no	no	yes	no	yes
182170150	chr1	2467715	2467776	31	62	2.000000	no	no	yes	no	no	no	no	yes	no	yes
182170151	chr1	2468470	2468564	48	95	1.979167	no	no	yes	no	no	no	no	yes	no	yes
182170153	chr1	2478342	2478470	42	129	3.071429	yes	no	yes	no	no	no	yes	yes	yes	yes
182170156	chr1	2481143	2481184	10	42	4.200000	no	no	yes	no	no	no	no	yes	yes	yes
182170157	chr1	2482293	2482367	39	75	1.974359	no	no	yes	no	no	no	no	yes	no	yes
182170158	chr1	2483753	2484138	22	386	17.652174	no	no	yes	no	no	no	yes	yes	no	yes
182170161	chr1	2485505	2485717	42	213	5.071429	no	no	yes	no	no	no	no	no	no	yes
182170162	chr1	2491732	2492062	165	331	2.006061	no	no	yes	no	no	yes	no	yes	no	yes
182170164	chr1	2494985	2495064	25	80	3.200000	yes	no	yes	no	no	no	no	yes	no	yes
182170165	chr1	2497164	2497198	17	35	2.058824	yes	no	yes	no	no	no	no	yes	no	yes
182170166	chr1	2497618	2497653	15	36	2.400000	yes	no	yes	no	no	no	no	yes	no	yes
182170167	chr1	2500775	2500875	20	101	5.000000	no	yes	yes	no	no	no	no	no	no	yes
182170168	chr1	2503020	2503056	14	37	2.642857	no	yes	yes	no	no	no	no	yes	yes	yes
182170169	chr1	2506878	2506925	11	48	4.363636	no	no	no	no	no	no	no	yes	yes	no
182170173	chr1	2511742	2511968	48	227	4.875000	no	no	yes	no	no	yes	no	yes	no	yes
182170176	chr1	2515957	2515994	20	38	1.950000	yes	no	yes	no	no	no	no	yes	no	yes
182170178	chr1	2519379	2519475	28	97	3.500000	yes	no	yes	no	no	no	no	no	yes	yes
182170180	chr1	2522690	2523010	99	321	3.272727	no	no	yes	no	no	no	no	yes	yes	yes
182170182	chr1	2526289	2526364	37	76	2.081081	yes	no	yes	no	no	no	yes	yes	yes	yes
182170184	chr1	2527694	2527737	16	44	2.750000	no	no	no	no	no	no	no	yes	yes	no
182170186	chr1	2529783	2529840	16	58	3.555556	yes	no	yes	no	no	no	yes	yes	no	yes

182170188	chr1	2530929	2530994	29	66	2.275862	yes	no	yes	no	no	no	no	yes	yes	yes
182170191	chr1	2537037	2537082	22	46	2.090909	no	no	yes	no	no	no	no	yes	no	yes
182170197	chr1	2537095	2537198	46	104	2.347826	no	no	yes	no	no	no	no	yes	yes	yes
182170205	chr1	2547170	2547211	19	42	2.210526	no	yes	no	no	no	no	no	yes	no	yes
182170206	chr1	2548250	2548277	12	28	2.333333	no	no	no	no	no	yes	no	yes	yes	no
182170210	chr1	2555526	2555570	18	45	2.500000	yes	no	yes	no	yes	no	no	yes	no	yes
182170212	chr1	2559737	2559784	19	48	2.500000	yes	yes	yes	no	no	yes	no	yes	yes	yes
182170214	chr1	2562260	2562290	14	31	2.214286	no	yes	yes	no	no	yes	no	yes	no	yes
182170216	chr1	2562908	2562949	20	42	2.200000	no	yes	yes	no	no	no	no	yes	no	yes
182170219	chr1	2585233	2585487	33	255	7.764706	no	no	no	no	no	yes	no	yes	yes	no
182170221	chr1	2586798	2586863	14	66	4.600000	yes	yes	yes	yes	yes	no	yes	yes	no	yes
182170223	chr1	2587613	2587653	19	41	2.210526	yes	no	yes	no	no	no	no	yes	yes	yes
182170224	chr1	2592278	2592645	23	368	15.884615	no	no	yes	no	no	yes	no	yes	no	yes
182170225	chr1	2595380	2595462	43	83	1.930233	no	no	yes	no	no	no	no	no	no	yes
182170226	chr1	2595806	2595927	40	122	3.050000	no	no	yes	no	no	no	yes	no	no	yes
182170229	chr1	2604019	2604156	73	138	1.904110	no	no	yes	yes	no	no	no	yes	no	yes
182170230	chr1	2604567	2604598	14	32	2.285714	no	no	yes	no	no	no	no	no	no	yes
182170231	chr1	2604626	2604668	15	43	2.866667	no	no	yes	no	no	no	no	no	no	yes
182170244	chr1	2628680	2628739	30	60	2.000000	no	no	yes	no	no	no	yes	yes	no	yes
182170248	chr1	2630473	2630519	18	47	2.611111	yes	no	yes	no	no	no	no	yes	no	yes
182170254	chr1	2630773	2631073	98	301	3.019048	no	no	yes	no	no	no	no	yes	yes	yes
182170261	chr1	2632769	2632834	31	66	2.129032	no	no	yes	no	no	no	no	yes	yes	yes
182170272	chr1	2640999	2641265	137	267	1.970803	no	yes	no	no	no	yes	no	yes	yes	yes
182170277	chr1	2648709	2648809	51	101	2.000000	no	no	yes	no	no	no	no	no	no	yes
182170339	chr1	2777970	2778008	19	39	2.052632	no	no	yes	no	no	no	no	no	no	yes

182170341	chr1	2784957	2785022	30	66	2.200000	no	no	yes	no	no	yes	no	no	no	yes
182170342	chr1	2785134	2785284	75	151	2.013333	no	no	yes	no	no	yes	no	yes	no	yes
182170350	chr1	2811030	2811067	14	38	2.714286	no	no	no	no	no	yes	no	yes	no	no
182170351	chr1	2811548	2811586	19	39	2.052632	no	no	no	no	no	yes	no	yes	no	no
182170352	chr1	2812074	2812106	10	33	3.181818	no	no	yes	no	no	no	no	no	no	yes
182170358	chr1	2819302	2819374	34	73	2.176471	no	no	no	no	no	no	no	no	no	no
182170361	chr1	2819575	2819610	14	36	2.571429	no	no	no	no	no	no	no	yes	no	no
182170365	chr1	2819683	2819727	18	45	2.500000	no	no	no	no	no	no	no	yes	no	no
182170370	chr1	2820227	2820388	17	162	9.529411	no	no	no	no	no	no	no	yes	no	no
182170372	chr1	2820588	2820749	84	162	1.940476	no	no	no	no	no	no	no	no	no	no
182170375	chr1	2825039	2825134	31	96	3.125000	no	no	no	no	no	no	no	no	no	no
182170377	chr1	2827753	2827803	22	51	2.363636	no	no	no	no	no	no	no	no	no	no
182170392	chr1	2848786	2849744	352	959	2.744318	no	no	no	no	no	no	no	no	yes	no
182170394	chr1	2851920	2852971	52	1052	20.230770	no	no	no	no	no	no	no	yes	yes	no
182170400	chr1	2863231	2863476	138	246	1.826087	no	no	no	no	no	no	no	no	yes	no
182170401	chr1	2871324	2871565	39	242	6.205128	no	no	no	no	no	no	no	yes	no	no
182170406	chr1	2877523	2877635	49	113	2.306123	no	no	no	no	no	no	no	no	yes	no
182170407	chr1	2882217	2882285	36	69	1.916667	no	no	no	no	no	no	no	no	no	no
182170411	chr1	2887412	2887462	27	51	1.962963	no	no	no	no	no	no	no	no	no	no
182170414	chr1	2893151	2893200	19	50	2.631579	no	no	no	no	no	no	no	no	no	no
182170415	chr1	2893707	2893745	21	39	1.904762	no	no	no	no	no	no	no	yes	no	no
182170416	chr1	2894695	2894744	14	50	3.642857	no	no	no	no	no	no	no	no	yes	no
182170428	chr1	2899700	2899821	64	122	1.921875	no	no	no	no	no	no	no	yes	no	no
182170429	chr1	2900338	2900375	17	38	2.235294	no	no	no	no	no	no	no	yes	yes	no
182170430	chr1	2902321	2902951	28	631	22.193548	no	no	no	no	no	no	no	no	no	no

182170435	chr1	2914709	2914777	31	69	2.225806	no	no	no	no	no	no	no	yes	no	no
182170439	chr1	2916167	2916203	17	37	2.235294	no	no	no	no	no	no	no	yes	yes	no
182170441	chr1	2917056	2917091	18	36	2.055556	no	no	no	no	no	no	no	yes	no	no
182170442	chr1	2921281	2921348	32	68	2.125000	no	no	no	no	no	no	no	no	yes	no
182170443	chr1	2921417	2922039	42	623	14.833333	no	no	no	no	no	no	no	yes	yes	no
182170449	chr1	2930160	2930244	26	85	3.178571	no	no	no	no	no	no	no	yes	no	no
182170451	chr1	2932288	2932339	20	52	2.600000	no	no	no	no	no	no	no	no	no	no
182170453	chr1	2933517	2933609	31	93	3.032258	no	no	no	no	no	no	no	no	no	no
182170454	chr1	2936957	2936990	16	34	2.125000	no	no	no	no	no	no	no	no	no	no
182170455	chr1	2937963	2938004	14	42	3.000000	no	no	no	no	no	no	no	yes	no	no
182170458	chr1	2938162	2938200	20	39	1.950000	no	no	no	no	no	no	no	yes	no	no
182170470	chr1	2945341	2945382	18	42	2.444444	no	no	no	no	no	no	no	no	no	no
182170472	chr1	2947418	2947509	46	92	2.000000	no	no	no	no	no	no	no	yes	yes	no
182170473	chr1	2949525	2950064	49	540	11.020409	no	no	no	no	no	no	no	yes	yes	no
182170476	chr1	2950050	2950083	16	34	2.125000	no	no	no	no	no	no	no	yes	no	no
182170482	chr1	2959999	2960238	30	240	7.967742	no	no	no	no	no	yes	yes	no	no	no
182170483	chr1	2960522	2960921	35	400	11.485714	no	no	no	no	yes	yes	no	no	no	yes
182170490	chr1	2966717	2966800	42	84	2.000000	no	no	yes	no	no	no	no	yes	no	yes
182170491	chr1	2970811	2970863	25	53	2.120000	no	no	no	no	no	no	no	no	no	no
182170493	chr1	2974168	2974291	61	124	2.032787	no	no	no	no	no	no	no	no	no	no
182170494	chr1	2974537	2974791	22	255	11.608696	no	no	no	no	no	no	no	yes	yes	no
182170497	chr1	2976503	2977987	20	1485	73.099998	no	no	no	no	no	no	no	no	no	no
182170500	chr1	2980994	2981083	15	90	6.000000	no	no	no	no	no	no	no	no	no	no
182170504	chr1	2986258	2986374	63	117	1.920635	no	no	no	no	no	no	no	yes	no	no
182170506	chr1	2987616	2987652	10	37	3.700000	no	no	no	no	no	no	no	no	yes	no

182170522	chr1	3016355	3016666	103	312	2.938053	no	no	no	no	no	no	no	yes	no	no
182170523	chr1	3019655	3019818	80	164	2.050000	no	no	no	no	no	no	no	yes	no	no
182170528	chr1	3029051	3029500	64	450	7.031250	no	no	no	no	no	no	no	yes	no	no
182170529	chr1	3036600	3036651	24	52	2.291667	no	no	no	no	no	no	no	no	no	no
182170530	chr1	3037751	3037894	71	144	2.028169	no	no	no	no	no	no	no	yes	yes	no
182170531	chr1	3039406	3039451	16	46	2.823529	no	no	no	no	no	no	no	yes	yes	no
182170533	chr1	3039658	3039698	15	41	2.733333	no	no	no	no	no	no	no	no	no	no
182170535	chr1	3039735	3039766	15	32	2.133333	no	no	no	no	no	no	no	no	no	no
182170542	chr1	3042615	3042708	19	94	5.000000	no	no	no	no	no	no	no	yes	no	no
182170545	chr1	3051720	3051751	13	32	2.461539	no	no	no	no	no	no	no	no	no	no
182170551	chr1	3069315	3069368	28	54	1.928571	yes	no	yes	no	no	no	yes	yes	no	yes
182170553	chr1	3074790	3074814	13	25	1.923077	no	no	yes	no	no	no	no	yes	no	yes
182170554	chr1	3079318	3079405	46	88	1.913043	no	no	yes	no	no	no	no	no	no	yes
182170555	chr1	3082208	3082248	15	41	2.733333	no	no	yes	no	no	no	no	no	no	yes
182170557	chr1	3086479	3086549	20	71	3.550000	no	no	yes	no	no	no	no	yes	no	yes
182170558	chr1	3087169	3087301	16	133	8.312500	no	no	yes	no	no	no	no	yes	no	yes
182170563	chr1	3088751	3088800	26	50	2.038461	no	no	yes	no	no	no	no	no	no	yes
182170566	chr1	3109906	3110592	39	687	17.450001	no	no	yes	no	no	no	no	no	no	yes
182170573	chr1	3114402	3114497	40	96	2.450000	no	no	yes	no	no	no	no	yes	no	yes
182170577	chr1	3117970	3118169	112	200	1.812500	no	no	yes	no	no	no	no	yes	yes	yes
182170582	chr1	3118203	3118245	22	43	1.954545	no	no	yes	no	no	no	no	yes	no	yes
182170584	chr1	3124127	3124162	19	36	1.947368	no	no	yes	no	no	no	no	yes	no	yes
182170585	chr1	3128669	3128721	23	53	2.304348	no	no	yes	no	no	yes	no	no	yes	yes
182170592	chr1	3134025	3134061	18	37	2.055556	yes	no	yes	no	no	no	no	yes	yes	yes
182170597	chr1	3148198	3148258	19	61	3.210526	no	no	yes	no	no	no	no	no	no	yes

182170599	chr1	3150872	3151055	28	184	6.517241	no	no	yes	no	no	no	no	no	no	yes
182170601	chr1	3153246	3153276	16	31	1.937500	no	no	yes	no	no	no	no	yes	no	yes
182170603	chr1	3158653	3158778	47	126	2.750000	no	no	yes	no	no	no	no	yes	no	yes
182170605	chr1	3162862	3163340	48	479	9.979167	no	no	yes	no	no	no	no	no	no	yes
182170606	chr1	3165155	3165946	24	792	33.041668	no	no	yes	no	no	no	no	yes	yes	yes
182170607	chr1	3167538	3168370	59	833	14.046875	no	no	yes	no	no	no	no	yes	no	yes
182170610	chr1	3173944	3173969	12	26	2.166667	no	no	yes	no	no	no	no	no	no	yes
182170616	chr1	3181153	3181213	20	61	2.954545	no	no	yes	no	no	no	no	yes	yes	yes
182170619	chr1	3181957	3182011	20	55	2.714286	no	no	yes	no	no	no	no	yes	no	yes
182170621	chr1	3185436	3185576	28	141	5.035714	no	no	yes	no	no	no	no	yes	no	yes
182170622	chr1	3189306	3189489	57	184	3.228070	no	no	yes	no	no	no	no	no	no	yes
182170623	chr1	3190156	3190305	24	150	6.250000	no	no	yes	no	no	no	no	yes	no	yes
182170624	chr1	3192998	3193156	31	159	5.129032	no	no	yes	no	no	no	no	yes	yes	yes
182170625	chr1	3194564	3194752	21	189	9.000000	no	no	yes	no	no	no	yes	yes	no	yes
182170628	chr1	3202336	3202394	10	59	5.900000	no	no	yes	no	no	no	no	no	no	yes
182170629	chr1	3202575	3202635	27	61	2.259259	no	no	yes	no	no	no	no	no	no	yes
182170631	chr1	3210478	3210514	16	37	2.312500	no	no	yes	no	no	no	no	no	no	yes
182170635	chr1	3210736	3211034	153	299	1.954248	no	no	yes	no	no	no	no	no	no	yes
182170637	chr1	3211794	3211828	18	35	1.944444	no	no	yes	no	no	no	no	no	no	yes
182170638	chr1	3212590	3212695	35	106	3.028571	no	no	yes	no	no	no	no	yes	no	yes
182170639	chr1	3215347	3215466	62	120	1.951613	no	no	yes	no	no	no	no	no	no	yes
182170640	chr1	3216599	3216647	23	49	2.173913	no	no	yes	no	no	no	no	no	no	yes
182170641	chr1	3219675	3219726	22	52	2.363636	no	no	yes	no	no	no	no	yes	yes	yes
182170643	chr1	3224439	3224548	39	110	2.820513	no	no	yes	no	no	no	no	yes	no	yes
182170645	chr1	3231371	3231449	39	79	2.051282	no	no	yes	no	no	no	yes	yes	yes	yes

182170651	chr1	3247697	3247726	14	30	2.142857	no	no	yes	no	no	no	yes	yes	yes	yes
182170652	chr1	3251307	3251404	50	98	1.960000	no	no	yes	no	no	no	no	yes	yes	yes
182170653	chr1	3255827	3255946	30	120	4.000000	no	no	yes	no	no	no	no	no	yes	yes
182170656	chr1	3261770	3261806	19	37	1.947368	no	no	yes	no	no	no	no	no	yes	yes
182170657	chr1	3262726	3262924	102	199	1.950980	no	no	yes	no	no	no	no	no	no	yes
182170661	chr1	3265876	3265915	20	40	2.000000	no	no	yes	no	no	no	no	yes	no	yes
182170665	chr1	3270932	3270974	14	43	3.071429	no	no	yes	no	no	no	no	yes	no	yes
182170668	chr1	3276660	3276726	25	67	2.680000	no	no	yes	no	no	no	no	no	no	yes
182170672	chr1	3277718	3277782	18	65	3.611111	no	no	yes	no	no	no	no	yes	no	yes
182170678	chr1	3281502	3281802	88	301	3.420455	no	no	yes	no	no	no	no	yes	no	yes
182170680	chr1	3283545	3283770	56	226	4.035714	no	no	yes	no	no	no	no	yes	no	yes
182170683	chr1	3286839	3286996	39	158	4.051282	no	no	yes	no	no	no	no	yes	no	yes
182170686	chr1	3287223	3287893	47	671	14.102041	no	no	yes	no	no	no	yes	yes	no	yes
182170690	chr1	3292564	3292611	19	48	2.526316	no	no	yes	no	no	no	no	yes	no	yes
182170691	chr1	3294115	3294214	33	100	3.030303	no	no	yes	no	no	no	no	yes	no	yes
182170693	chr1	3297916	3298014	43	99	2.302325	no	no	yes	no	no	no	no	no	no	yes
182170695	chr1	3298781	3298810	15	30	2.000000	no	no	yes	no	no	no	no	no	no	yes
182170696	chr1	3299084	3300545	61	1462	23.967213	no	no	yes	no	no	no	no	no	yes	yes
182170701	chr1	3303779	3304305	172	527	2.994565	no	no	yes	no	no	no	no	yes	no	yes
182170702	chr1	3304487	3304624	71	138	1.943662	no	no	yes	no	no	no	no	yes	no	yes
182170703	chr1	3305064	3305116	26	53	2.038461	no	no	yes	no	no	no	no	no	no	yes
182170717	chr1	3316547	3316864	104	318	3.047619	no	no	yes	no	no	no	no	yes	yes	yes
182170726	chr1	3318519	3318555	19	37	1.947368	no	no	yes	no	no	no	no	yes	no	yes
182170729	chr1	3321297	3321412	30	116	3.750000	no	no	yes	no	no	no	no	yes	no	yes
182170731	chr1	3321660	3321699	18	40	2.222222	no	no	yes	no	no	no	no	yes	no	yes

182170735	chr1	3321989	3322034	20	46	2.300000	no	no	yes	no	no	no	no	yes	no	yes
182170741	chr1	3324162	3324186	12	25	2.083333	no	no	yes	no	no	no	no	yes	no	yes
182170742	chr1	3324718	3324765	22	48	2.181818	no	no	yes	no	no	no	no	yes	no	yes
182170749	chr1	3336311	3336358	18	48	2.777778	no	no	yes	no	no	no	no	yes	no	yes
182170752	chr1	3336999	3337262	90	264	2.956522	no	no	yes	no	no	no	no	yes	no	yes
182170754	chr1	3338056	3338095	16	40	2.500000	no	no	yes	no	no	no	no	yes	no	yes
182170755	chr1	3341062	3341127	22	66	3.000000	no	no	yes	no	no	no	no	yes	no	yes
182170757	chr1	3345715	3345826	43	112	2.604651	no	no	yes	no	no	no	no	yes	no	yes
182170758	chr1	3349840	3349876	18	37	2.055556	no	no	yes	no	no	no	no	yes	yes	yes
182170763	chr1	3355067	3355119	20	53	2.650000	no	no	yes	no	no	no	no	no	yes	yes
182170764	chr1	3361709	3362045	33	337	10.000000	no	no	yes	no	no	no	no	no	no	yes
182170766	chr1	3362815	3362853	20	39	1.950000	no	no	yes	no	no	no	no	yes	no	yes
182170772	chr1	3370795	3370841	20	47	2.400000	no	no	yes	no	no	no	no	no	no	yes
182170774	chr1	3373920	3373953	10	34	3.400000	yes	no	yes	no	yes	no	no	no	no	yes
182170776	chr1	3379067	3380113	14	1047	74.470589	no	no	yes	no	no	no	no	no	no	yes
182170777	chr1	3381147	3381184	18	38	2.111111	no	no	yes	no	no	no	no	no	no	yes
182170778	chr1	3383592	3383712	63	121	1.920635	no	no	yes	no	no	no	no	no	no	yes
182170786	chr1	3394310	3394338	10	29	2.900000	no	no	yes	no	no	no	yes	yes	no	yes
182170788	chr1	3403007	3403092	31	86	2.774194	no	no	yes	no	no	no	no	yes	no	yes
182170798	chr1	3408724	3408888	86	165	1.941860	no	no	yes	no	no	no	yes	yes	no	yes
182170800	chr1	3408906	3408987	43	82	1.906977	no	no	yes	no	no	no	yes	yes	yes	yes
182170804	chr1	3409091	3409205	58	115	2.017241	no	no	yes	no	no	no	yes	yes	yes	yes
182170814	chr1	3424790	3424820	12	31	2.538461	yes	no	yes	no	no	no	no	yes	yes	yes
182170815	chr1	3426606	3426640	18	35	1.944444	no	no	yes	no	no	yes	no	yes	no	yes
182170817	chr1	3428147	3428340	48	194	4.041667	no	no	yes	no	no	no	no	no	no	yes

182170822	chr1	3430944	3430983	21	40	1.904762	no	yes	no	yes	no	no	no	no	yes	yes
182170824	chr1	3433420	3433647	39	228	5.974359	no	no	yes	no	no	no	no	no	no	yes
182170826	chr1	3434393	3434429	19	37	2.000000	no	yes	no	no	no	yes	no	yes	no	yes
182170827	chr1	3435652	3435712	30	61	2.033333	no	yes	no	no	no	yes	no	yes	no	yes
182170829	chr1	3444817	3444898	42	82	1.976190	no	no	no	no	no	no	no	yes	no	no
182170832	chr1	3452748	3452780	15	33	2.200000	no	no	no	no	no	no	no	yes	no	no
182170835	chr1	3461580	3461618	20	39	2.000000	no	no	yes	no	no	no	no	yes	no	yes
182170838	chr1	3470095	3470159	32	65	2.031250	no	no	yes	no	no	no	no	yes	no	yes
182170845	chr1	3470341	3470426	45	86	1.933333	no	no	yes	no	no	no	no	yes	yes	yes
182170850	chr1	3470504	3470584	20	81	4.050000	no	no	yes	no	no	no	no	yes	no	yes
182170852	chr1	3470615	3470669	26	55	2.115385	yes	no	yes	no	no	no	no	yes	yes	yes
182170860	chr1	3470956	3471134	96	179	1.927083	yes	no	yes	no	no	no	no	yes	yes	yes
182170865	chr1	3474191	3474243	20	53	2.700000	no	yes	yes	no	no	yes	no	yes	yes	yes
182170867	chr1	3477293	3477347	23	55	2.375000	no	no	yes	no	no	no	no	yes	yes	yes
182170868	chr1	3477814	3477901	42	88	2.119048	no	yes	yes	yes	no	no	no	yes	yes	yes
182170870	chr1	3484319	3484389	21	71	3.200000	no	no	no	no	no	no	no	yes	no	no
182170873	chr1	3492037	3492150	47	114	2.416667	no	no	yes	no	no	yes	no	yes	yes	yes
182170874	chr1	3493136	3493422	21	287	13.666667	no	no	yes	no	yes	no	no	yes	no	yes
182170875	chr1	3500479	3500566	39	88	2.256410	no	no	yes	no	no	no	yes	yes	yes	yes
182170876	chr1	3501991	3502114	39	124	3.275000	no	no	yes	no	no	no	no	yes	yes	yes
182170877	chr1	3503464	3503502	11	39	3.545455	no	no	yes	no	no	no	no	yes	yes	yes
182170878	chr1	3503667	3503742	35	76	2.171429	no	no	yes	no	no	no	no	yes	yes	yes
182170879	chr1	3504102	3504161	28	60	2.142857	no	no	yes	no	no	no	no	yes	no	yes
182170880	chr1	3505037	3505082	17	46	2.705882	no	no	yes	no	no	no	no	yes	yes	yes
182170881	chr1	3510320	3510654	36	335	9.205129	no	no	yes	no	no	no	no	yes	yes	yes

182170887	chr1	3518661	3518705	23	45	2.000000	no	no	yes	no	no	no	no	yes	no	yes
182170888	chr1	3520537	3520658	32	122	3.787879	no	no	yes	no	no	no	no	yes	no	yes
182170889	chr1	3530413	3530449	18	37	2.055556	no	no	yes	no	no	no	no	yes	yes	yes
182170890	chr1	3531805	3531851	21	47	2.238095	yes	no	yes	no	no	no	no	yes	yes	yes
182170891	chr1	3535425	3535467	18	43	2.388889	no	no	yes	no	no	no	no	yes	yes	yes
182170894	chr1	3544178	3544284	36	107	2.972222	no	no	yes	no	no	no	no	yes	yes	yes
182170896	chr1	3546499	3546897	48	399	8.520833	no	no	yes	no	no	no	no	yes	no	yes
182170899	chr1	3546847	3546986	36	140	3.972973	no	no	yes	no	no	no	no	yes	no	yes
182170900	chr1	3551729	3551760	14	32	2.285714	no	no	yes	no	no	no	no	yes	no	yes
182170902	chr1	3559112	3559136	9	25	2.777778	no	no	yes	no	no	no	no	no	no	yes
182170906	chr1	3562300	3562371	34	72	2.176471	no	no	yes	no	no	no	no	no	no	yes
182170913	chr1	3564876	3564985	52	110	2.134615	no	no	yes	no	no	no	no	yes	yes	yes
182170914	chr1	3568347	3568416	32	70	2.187500	no	no	yes	no	no	no	no	no	no	yes
182170915	chr1	3570189	3570339	76	151	1.986842	no	no	yes	no	no	no	no	yes	yes	yes
182170916	chr1	3571403	3571571	87	169	1.954023	no	no	yes	no	no	no	no	yes	no	yes
182170922	chr1	3574033	3574070	15	38	2.533333	no	no	yes	no	no	no	no	yes	no	yes
182170928	chr1	3576651	3577125	229	475	2.091304	no	no	yes	no	no	no	no	yes	no	yes
182170929	chr1	3578746	3578928	91	183	2.010989	no	no	yes	no	no	no	no	no	no	yes
182170930	chr1	3580729	3580775	19	47	2.473684	no	no	yes	no	no	no	no	yes	no	yes
182170931	chr1	3583262	3583932	18	671	36.958332	no	no	yes	no	no	no	yes	no	no	yes
182170934	chr1	3585084	3585124	19	41	2.157895	no	no	yes	no	no	no	no	yes	yes	yes
182170937	chr1	3585573	3585606	17	34	2.000000	no	no	yes	no	no	no	no	yes	no	yes
182170940	chr1	3585858	3585942	45	85	1.911111	no	no	yes	no	no	no	no	yes	no	yes
182170943	chr1	3586179	3586228	21	50	2.380952	no	no	yes	no	no	no	no	yes	yes	yes
182170946	chr1	3587841	3588571	20	731	36.549999	no	no	yes	no	no	no	no	yes	yes	yes

182170949	chr1	3590822	3590903	35	82	2.371428	no	no	yes	no	no	no	no	yes	yes	yes
182170950	chr1	3591596	3591929	66	334	5.060606	no	no	yes	no	no	no	no	yes	yes	yes
182170951	chr1	3596424	3596737	44	314	7.136364	no	no	yes	no	no	no	no	no	no	yes
182170952	chr1	3604328	3604356	12	29	2.416667	no	no	yes	no	no	no	no	yes	no	yes
182170958	chr1	3605319	3605351	17	33	1.941176	no	no	yes	no	no	no	no	yes	no	yes
182170964	chr1	3612272	3612362	45	91	2.022222	yes	no	no	no	no	no	no	yes	no	no
182170965	chr1	3613812	3613929	30	118	4.000000	no	no	no	no	no	no	no	yes	yes	no
182170966	chr1	3616392	3616481	45	90	2.000000	no	no	no	no	no	no	no	no	no	no
182170968	chr1	3616552	3616601	26	50	1.923077	no	no	no	no	no	no	no	no	no	no
182170972	chr1	3625149	3625189	17	41	2.470588	yes	no	no	yes	no	no	yes	yes	no	yes
182170974	chr1	3627683	3627725	21	43	2.047619	no	no	yes	no	no	no	no	yes	no	yes
182170975	chr1	3628612	3628650	19	39	2.052632	no	yes	no	no	no	no	no	yes	yes	yes
182170976	chr1	3633108	3633155	25	48	1.960000	no	no	yes	no	yes	yes	no	no	no	yes
182170977	chr1	3636229	3636470	77	242	3.142857	yes	no	yes	no	yes	no	no	yes	no	yes
182170978	chr1	3639690	3639845	60	156	2.600000	yes	no	yes	no	no	no	no	no	no	yes
182170980	chr1	3640355	3640801	37	447	12.108109	yes	no	yes	no	no	no	yes	yes	yes	yes
182170982	chr1	3641209	3641387	96	179	1.864583	no	no	yes	no	no	no	no	yes	no	yes
182170984	chr1	3643529	3644433	51	905	17.764706	no	no	yes	no	no	no	yes	yes	no	yes
182170985	chr1	3645294	3645555	38	262	6.894737	no	no	yes	no	no	no	yes	yes	no	yes
182170986	chr1	3649111	3649191	42	81	1.952381	no	no	yes	no	no	no	no	yes	yes	yes
182170992	chr1	3649786	3649825	17	40	2.352941	no	no	yes	no	no	no	yes	yes	no	yes
182170994	chr1	3651959	3652002	20	44	2.200000	yes	no	yes	no	no	no	yes	yes	no	yes
182170995	chr1	3653643	3653671	15	29	1.933333	yes	no	yes	no	no	no	no	yes	no	yes
182171007	chr1	3668946	3668997	26	52	2.000000	yes	no	yes	no	no	no	no	yes	yes	yes
182171008	chr1	3670183	3670272	45	90	2.000000	no	no	yes	no	no	no	no	no	no	yes

182171011	chr1	3674990	3675354	24	365	15.160000	no	no	yes	no	no	no	no	yes	yes	yes
182171016	chr1	3676407	3676462	28	56	2.107143	no	no	yes	no	no	no	no	no	no	yes
182171017	chr1	3676447	3676503	21	57	2.727273	no	no	yes	no	no	no	no	no	no	yes
182171022	chr1	3681820	3681970	73	151	2.068493	yes	no	yes	no	no	no	no	no	no	yes
182171026	chr1	3689738	3689776	19	39	2.052632	yes	no	yes	no	no	no	no	yes	no	yes
182171027	chr1	3693666	3695003	35	1338	38.083332	no	no	yes	no	no	no	no	no	no	yes
182171029	chr1	3697150	3697201	18	52	2.888889	yes	no	yes	no	no	no	no	yes	no	yes
182171030	chr1	3699027	3699115	22	89	4.045455	no	no	yes	no	no	no	no	yes	no	yes
182171031	chr1	3699710	3699748	21	39	1.952381	no	no	yes	no	no	no	no	yes	yes	yes
182171033	chr1	3702461	3702492	14	32	2.285714	no	no	yes	no	no	no	no	yes	no	yes
182171034	chr1	3705745	3705781	12	37	3.166667	no	no	yes	no	no	no	no	yes	no	yes
182171036	chr1	3707809	3707839	7	31	4.428571	no	yes	yes	no	no	no	no	yes	no	yes
182171038	chr1	3713610	3713648	15	39	2.600000	no	no	yes	no	no	no	no	yes	no	yes
182171039	chr1	3714443	3714481	15	39	2.600000	yes	no	yes	no	no	no	no	no	yes	yes
182171040	chr1	3715255	3715371	38	117	3.050000	yes	no	yes	no	no	no	no	yes	no	yes
182171041	chr1	3719583	3719618	17	36	2.176471	no	no	yes	no	no	no	no	yes	no	yes
182171047	chr1	3722767	3722867	48	101	2.104167	no	no	yes	no	no	no	no	yes	no	yes
182171051	chr1	3725591	3725643	27	53	1.962963	no	no	yes	no	no	no	no	yes	no	yes
182171055	chr1	3727567	3727597	16	31	2.000000	no	yes	yes	no	no	no	no	yes	yes	yes
182171061	chr1	3734889	3734919	10	31	3.100000	no	yes	no	no	no	yes	no	yes	yes	yes
182171062	chr1	3735383	3735408	9	26	2.888889	no	yes	no	no	no	yes	no	no	yes	yes
182171063	chr1	3739214	3740189	36	976	27.222221	no	no	yes	no	yes	yes	no	yes	yes	yes
182171064	chr1	3740242	3740314	36	73	2.027778	no	no	yes	no	yes	no	no	yes	yes	yes
182171076	chr1	3761968	3762000	17	33	1.941176	no	no	yes	no	no	no	no	no	no	yes
182171079	chr1	3772468	3772672	99	205	2.101010	yes	no	no	no	no	yes	yes	yes	yes	no

182171081	chr1	3772952	3772976	12	25	2.083333	no	no	yes	no	yes	no	yes	yes	no	yes
182171083	chr1	3788655	3788737	44	83	1.909091	no	no	yes	no	no	no	no	yes	yes	yes
182171084	chr1	3791192	3791218	14	27	1.928571	no	no	yes	no	no	no	no	yes	yes	yes
182171099	chr1	3822231	3822266	16	36	2.250000	no	no	yes	no	no	yes	no	yes	no	yes
182171101	chr1	3823091	3823119	12	29	2.416667	no	yes	yes	no	no	no	no	yes	no	yes
182171102	chr1	3824789	3825174	22	386	17.280001	no	no	yes	no	no	no	no	no	no	yes
182171106	chr1	3826654	3826719	34	66	1.941176	no	no	yes	yes	no	no	no	no	no	yes
182171107	chr1	3827996	3828082	42	87	2.095238	no	no	yes	no	no	no	no	yes	yes	yes
182171108	chr1	3832208	3832309	17	102	6.111111	yes	no	yes	no	no	no	no	yes	yes	yes
182171109	chr1	3832293	3832558	24	266	10.812500	no	no	yes	no	no	no	no	yes	yes	yes
182171110	chr1	3834253	3834508	53	256	4.830189	no	no	yes	no	no	yes	no	no	no	yes
182171111	chr1	3835179	3835260	38	82	2.157895	no	no	yes	no	no	no	no	no	no	yes
182171113	chr1	3846526	3846561	15	36	2.466667	no	no	yes	no	no	no	no	no	no	yes
182171124	chr1	3872615	3872681	17	67	3.941176	no	no	yes	no	no	yes	no	no	no	yes
182171127	chr1	3874051	3875054	78	1004	12.871795	no	no	yes	no	no	no	yes	no	no	yes
182171128	chr1	3877830	3877880	18	51	2.833333	no	no	yes	no	no	no	no	no	no	yes
182171129	chr1	3880716	3880768	17	53	3.117647	no	no	yes	no	no	no	no	no	no	yes
182171130	chr1	3883806	3883835	15	30	2.000000	no	yes	yes	no	no	yes	no	no	no	yes
182171131	chr1	3885496	3885571	18	76	4.315790	no	no	no	no	no	yes	no	no	yes	no
182171132	chr1	3891247	3891340	46	94	2.043478	no	no	yes	no	yes	no	no	yes	yes	yes
182171133	chr1	3891819	3892682	46	864	18.782608	no	no	yes	no	yes	no	yes	yes	yes	yes
182171135	chr1	3895505	3895591	44	87	2.000000	no	no	yes	no	yes	no	no	no	no	yes
182171141	chr1	3899742	3900028	153	287	1.882353	yes	no	yes	no	no	no	yes	yes	yes	yes
182171148	chr1	3904438	3904538	52	101	1.942308	no	no	yes	no	no	no	no	yes	yes	yes
182171149	chr1	3907428	3907483	19	56	3.000000	no	no	yes	no	no	no	no	yes	yes	yes

182171152 chr1 3910043 3910128 44 86 1.954545 no no yes no no no
no yes yes yes
182171156 chr1 3920608 3920651 22 44 2.000000 no no no no no no
no no no no
182171160 chr1 3925367 3925516 50 150 3.000000 no no no no no no
no yes no no
182171162 chr1 3926657 3926696 18 40 2.333333 no no no no no no
no yes no no
182171174 chr1 3946062 3946089 14 28 2.000000 no no yes no no yes
no no no yes
182171175 chr1 3946163 3946334 27 172 6.370370 no no yes no yes yes
no no no yes
182171181 chr1 3959102 3959128 11 27 2.454545 no no no no no no
no no no no
182171185 chr1 3973360 3973854 45 495 11.088889 no no no no no no
no no no no
182171189 chr1 3985537 3985592 18 56 3.111111 no no yes no no no
no no no yes
182171193 chr1 3987703 3988060 191 358 1.895288 no no yes no no no
no yes yes yes
182171197 chr1 3992560 3992597 17 38 2.235294 no no yes no no no
no yes no yes
182171201 chr1 3999537 3999947 59 411 6.983051 no no yes no no yes
no no yes yes
182171203 chr1 4000920 4000952 17 33 1.941176 no no yes no no no
no yes no yes
182171211 chr1 4007575 4007711 69 137 2.000000 no no yes no no no
no yes yes yes
182171215 chr1 4008581 4008745 60 165 2.750000 no no yes no no no
no no no yes
182171223 chr1 4018255 4018304 26 50 1.923077 no no yes no no no
no no no yes
182171225 chr1 4019587 4019611 8 25 3.125000 yes no no no no no
no no no no
182171227 chr1 4020326 4020374 24 49 2.041667 yes no no no no no
no no no no
182171228 chr1 4020855 4020883 15 29 2.000000 no no no no no no
no no no no
182171243 chr1 4047259 4047290 15 32 2.200000 no no no no no no
no no yes no
182171260 chr1 4062509 4062549 16 41 2.687500 no no no no no no
no no no no
182171263 chr1 4069015 4069069 24 55 2.291667 no no no no no no
no no no no
182171264 chr1 4073330 4073375 27 46 1.777778 no no no no no no
no no no no
182171266 chr1 4077240 4078872 174 1633 9.247422 no no no no no no
no no no no
182171273 chr1 4086396 4086483 38 88 2.315789 no no no no no no
no no no no

182171275	chr1	4087821	4087855	17	35	2.117647	no	no	no	no	no	no	no	no	yes	no
182171276	chr1	4088238	4088268	16	31	1.937500	no	no	no	no	no	no	no	no	no	no
182171277	chr1	4091614	4091650	17	37	2.176471	no	no	no	no	no	no	no	no	no	no
182171280	chr1	4099040	4099107	35	68	1.942857	no	no	no	no	no	no	no	no	no	no
182171284	chr1	4102450	4102501	26	52	2.000000	no	no	no	no	no	no	no	no	no	no
182171287	chr1	4108018	4108074	25	57	2.280000	no	no	no	no	no	no	no	no	no	no
182171297	chr1	4129706	4130002	60	297	4.919355	no	no	no	no	no	no	no	yes	no	no
182171298	chr1	4133260	4133293	11	34	3.090909	no	no	no	no	no	no	no	yes	no	no
182171304	chr1	4144343	4144594	110	252	2.290909	no	no	no	no	no	no	no	yes	no	no
182171309	chr1	4160611	4160645	10	35	3.500000	no	no	no	no	no	no	no	no	no	no
182171310	chr1	4162147	4162179	14	33	2.357143	no	no	no	no	no	no	no	yes	yes	no
182171313	chr1	4165105	4165159	27	55	2.111111	no	no	no	no	no	no	no	no	no	no
182171314	chr1	4165193	4165233	21	41	2.000000	no	no	no	no	no	no	no	no	no	no
182171319	chr1	4180839	4180869	16	31	1.937500	no	no	no	no	no	no	no	no	no	no
182171332	chr1	4221427	4221578	77	152	1.974026	no	no	no	no	no	no	no	no	no	no
182171380	chr1	4323774	4323810	14	37	2.642857	no	no	no	no	no	no	no	no	no	no
182171383	chr1	4327519	4327706	94	188	2.000000	no	no	no	no	no	no	no	no	no	no
182171386	chr1	4331688	4332170	20	483	23.692308	no	no	no	no	no	no	no	yes	yes	no
182171389	chr1	4332354	4333581	29	1228	42.193550	no	no	no	no	no	no	no	no	no	no
182171395	chr1	4343421	4343710	20	290	15.000000	no	no	no	no	no	no	no	no	yes	no
182171405	chr1	4355753	4355894	40	142	3.550000	no	no	no	no	no	no	no	no	no	no
182171409	chr1	4368910	4368956	12	47	4.083333	no	no	no	no	no	no	no	yes	no	no
182171412	chr1	4369109	4369301	108	193	1.805556	no	no	no	no	no	no	no	yes	no	no
182171417	chr1	4372679	4372712	17	34	2.058824	no	no	no	no	no	no	no	no	no	no
182171428	chr1	4376783	4376812	16	30	1.937500	no	no	no	no	no	no	no	no	no	no

182171438	chr1	4402209	4402266	22	58	2.636364	no	no	no	no	no	no	no	no	no	no
182171447	chr1	4423605	4423760	64	156	2.446154	no	no	yes	no	no	no	no	yes	no	yes
182171450	chr1	4425957	4426138	69	182	2.661972	no	no	no	no	no	no	no	yes	yes	no
182171465	chr1	4429698	4429722	12	25	2.083333	no	no	no	no	no	no	no	no	no	no
182171474	chr1	4460206	4460244	20	39	1.950000	no	no	no	no	no	no	no	no	no	no
182171475	chr1	4463138	4463162	12	25	2.083333	no	no	no	no	no	no	no	yes	no	no
182171486	chr1	4473511	4473566	29	56	1.965517	no	no	no	no	no	no	no	no	no	no
182171488	chr1	4480856	4480888	17	33	1.941176	no	no	no	no	yes	no	no	no	no	yes
182171490	chr1	4482037	4482209	82	173	2.121951	no	no	no	no	yes	no	no	yes	yes	yes
182171492	chr1	4496166	4496219	20	54	2.800000	no	no	no	no	no	no	no	yes	no	no
182171499	chr1	4496605	4496666	33	62	1.909091	no	no	no	no	no	no	no	yes	yes	no
182171504	chr1	4501101	4501145	22	45	2.045455	no	no	no	no	no	no	no	no	no	no
182171505	chr1	4502851	4502948	43	98	2.279070	no	no	no	no	no	no	no	no	no	no
182171514	chr1	4513604	4513650	21	47	2.285714	no	no	no	no	no	no	no	no	yes	no
182171516	chr1	4515232	4515269	11	38	3.454545	no	no	no	no	no	no	no	no	no	no
182171524	chr1	4533792	4533840	19	49	2.631579	no	no	no	no	no	no	no	no	no	no
182171534	chr1	4560643	4560689	24	47	1.958333	no	no	no	no	no	no	no	no	no	no
182171538	chr1	4576752	4576813	33	62	1.909091	no	no	yes	no	no	no	no	no	no	yes
182171541	chr1	4585537	4585565	12	29	2.416667	no	no	yes	no	no	yes	no	yes	no	yes
182171547	chr1	4593720	4593757	14	38	2.714286	no	yes	no	no	no	no	no	no	no	yes
182171548	chr1	4595469	4595511	13	43	3.307692	no	no	no	no	no	no	no	no	no	no
182171553	chr1	4601188	4601392	71	205	2.901408	no	no	no	no	no	no	no	no	no	no
182171556	chr1	4616390	4616545	40	156	3.900000	no	no	no	no	no	no	no	yes	no	no
182171558	chr1	4616990	4617114	66	125	1.893939	no	no	no	no	no	no	no	no	no	no
182171559	chr1	4617659	4617709	15	51	3.235294	no	no	no	no	no	no	no	no	no	no

182171570	chr1	4619123	4619159	16	37	2.375000	no	no	no	no	no	no	no	no	no	no
182171573	chr1	4632534	4633461	7	928	133.000000	no	no	no	no	no	no	no	no	yes	no
182171574	chr1	4636151	4636197	24	47	1.958333	no	no	no	no	no	no	no	yes	no	no
182171575	chr1	4636479	4636514	18	36	2.000000	no	no	no	no	no	no	no	yes	no	no
182171577	chr1	4644287	4644520	37	234	6.243902	no	no	no	no	no	no	no	no	no	no
182171580	chr1	4644462	4644585	65	124	1.923077	no	no	no	no	no	no	no	no	no	no
182171584	chr1	4648971	4649737	46	767	16.782608	no	no	no	no	no	no	no	yes	yes	no
182171587	chr1	4654696	4654727	16	32	2.000000	yes	no	no	no	yes	no	yes	yes	no	yes
182171588	chr1	4657987	4658076	42	90	2.142857	no	no	yes	no	no	no	no	yes	yes	yes
182171591	chr1	4666055	4666262	52	208	4.000000	no	no	yes	no	no	no	no	no	no	yes
182171592	chr1	4666476	4667022	32	547	17.085714	no	no	yes	no	no	no	yes	yes	yes	yes
182171595	chr1	4671548	4671611	18	64	3.526316	no	no	yes	no	no	no	no	no	no	yes
182171600	chr1	4682667	4682869	87	203	2.333333	no	no	yes	no	no	no	no	yes	no	yes
182171601	chr1	4687792	4687833	8	42	5.250000	no	no	yes	no	no	no	no	no	no	yes
182171602	chr1	4692221	4692254	13	34	2.615385	no	no	yes	no	no	no	no	no	no	yes
182171603	chr1	4696915	4696993	27	79	2.892857	no	no	yes	no	no	no	no	yes	no	yes
182171605	chr1	4702034	4702208	57	175	3.070175	no	no	yes	no	no	no	no	no	no	yes
182171609	chr1	4709331	4709442	59	112	1.898305	no	no	yes	no	no	no	no	no	no	yes
182171615	chr1	4710388	4710419	16	32	2.000000	yes	no	yes	no	no	no	no	yes	no	yes
182171618	chr1	4714220	4714263	15	44	2.933333	no	no	yes	no	no	no	no	yes	no	yes
182171621	chr1	4722845	4722891	25	47	1.960000	no	no	yes	no	no	no	no	no	no	yes
182171622	chr1	4726139	4726242	36	104	2.888889	no	no	yes	no	no	no	no	no	no	yes
182171625	chr1	4737474	4737510	17	37	2.235294	no	no	yes	no	no	no	no	no	no	yes
182171627	chr1	4743930	4743957	9	28	3.111111	no	no	yes	no	no	no	no	no	no	yes
182171633	chr1	4749997	4750048	24	52	2.166667	no	no	yes	no	no	no	no	yes	no	yes

182171636	chr1	4750206	4750251	16	46	2.777778	no	no	yes	no	no	no	no	yes	yes	yes
182171639	chr1	4750495	4750672	90	178	1.977778	no	no	yes	no	no	no	no	yes	no	yes
182171641	chr1	4752147	4752214	18	68	3.736842	no	no	yes	no	no	no	no	yes	yes	yes
182171657	chr1	4765475	4765517	20	43	2.150000	no	no	yes	no	no	no	no	no	no	yes
182171661	chr1	4768538	4768569	16	32	2.062500	no	no	yes	no	no	no	no	yes	no	yes
182171663	chr1	4778019	4778052	17	34	2.000000	no	no	yes	no	no	yes	no	yes	no	yes
182171668	chr1	4781085	4781127	21	43	2.047619	no	no	yes	no	no	no	no	no	no	yes
182171686	chr1	4823481	4823571	34	91	2.638889	no	no	no	no	no	no	no	no	no	no
182171687	chr1	4826587	4826761	95	175	1.842105	no	no	no	no	no	no	no	yes	no	no
182171688	chr1	4829797	4829859	32	63	1.968750	no	no	no	no	no	no	no	no	no	no
182171689	chr1	4832133	4832187	28	55	1.964286	no	no	no	no	no	no	no	no	no	no
182171695	chr1	4854990	4855064	26	75	2.884615	no	no	no	no	no	no	no	no	no	no
182171697	chr1	4856839	4856870	12	32	2.666667	no	no	no	no	no	no	no	no	no	no
182171706	chr1	4906611	4906664	28	54	1.928571	no	no	no	no	no	no	no	no	no	no
182171708	chr1	4928360	4928418	29	59	2.034483	no	no	no	no	no	no	no	yes	no	no
182171712	chr1	4938470	4938510	22	41	1.909091	no	no	no	no	no	no	no	no	no	no
182171715	chr1	4939312	4939462	81	151	1.913580	no	no	no	no	no	no	no	yes	yes	no
182171718	chr1	4939656	4939804	82	149	1.865854	no	no	no	no	no	no	no	yes	yes	no
182171727	chr1	4946379	4946727	73	349	4.814815	no	no	no	no	no	no	no	yes	no	no
182171733	chr1	4947257	4947289	17	33	1.941176	no	no	no	no	no	no	no	no	no	no
182171755	chr1	5008621	5008794	76	174	2.315789	no	no	no	no	no	no	no	yes	no	no
182171758	chr1	5009031	5009075	22	45	2.045455	no	no	no	no	no	no	no	no	no	no
182171776	chr1	5069504	5069550	25	47	1.960000	no	no	no	no	no	no	no	no	no	no
182171777	chr1	5070089	5070123	14	35	2.500000	no	no	no	no	no	no	no	no	no	no
182171786	chr1	5075980	5076054	31	75	2.451613	no	no	no	no	no	no	no	no	no	no

182171797	chr1	5088746	5088796	9	51	5.666667	no	no	yes	no	no	no	no	no	no	yes
182171799	chr1	5090745	5090885	71	141	2.000000	no	no	no	no	yes	no	no	no	no	yes
182171802	chr1	5098417	5098455	20	39	1.950000	no	no	no	no	no	no	no	no	no	no
182171805	chr1	5111718	5111848	45	131	2.911111	no	no	no	no	no	no	no	yes	yes	no
182171806	chr1	5112300	5112520	12	221	18.833334	no	no	no	no	no	no	no	no	no	no
182171809	chr1	5120690	5120803	52	114	2.192308	no	no	no	no	no	no	no	no	no	no
182171810	chr1	5120773	5120940	89	168	1.887640	no	no	no	no	no	no	no	no	no	no
182171819	chr1	5129646	5129708	26	63	2.481482	no	no	no	no	no	no	no	no	no	no
182171824	chr1	5137022	5137193	8	172	21.500000	no	no	no	no	no	no	no	yes	no	no
182171826	chr1	5140618	5140709	46	92	2.000000	no	no	no	no	no	no	no	no	no	no
182171829	chr1	5146794	5146850	16	57	3.562500	no	no	no	no	no	no	no	yes	no	no
182171831	chr1	5155276	5155312	19	37	1.947368	no	no	no	no	no	no	no	no	no	no
182171845	chr1	5189919	5189978	25	60	2.370370	no	no	no	no	no	no	no	no	no	no
182171851	chr1	5205702	5205743	22	42	1.954545	no	no	no	no	no	no	no	no	no	no
182171858	chr1	5212134	5212244	22	111	5.045455	no	no	no	no	no	no	no	yes	no	no
182171864	chr1	5216736	5216768	16	33	2.062500	no	no	no	no	no	no	no	yes	no	no
182171878	chr1	5237493	5237527	17	35	2.117647	no	no	no	no	no	no	no	no	no	no
182171879	chr1	5241189	5241244	28	56	2.000000	no	no	no	no	no	no	no	no	no	no
182171881	chr1	5244293	5244362	36	70	1.944444	no	no	no	no	no	no	no	no	no	no
182171900	chr1	5302111	5302172	32	62	2.062500	no	no	yes	no	no	no	no	no	no	yes
182171902	chr1	5303973	5304283	51	311	6.117647	no	no	yes	no	no	no	no	yes	no	yes
182171907	chr1	5306772	5306820	25	49	2.040000	no	no	yes	no	no	no	no	yes	no	yes
182171912	chr1	5320007	5320119	42	113	2.714286	no	no	no	no	no	no	no	no	no	no
182171918	chr1	5334034	5334778	30	745	24.833334	no	no	no	no	no	no	no	no	no	no
182171919	chr1	5338234	5338271	20	38	1.950000	no	no	no	no	no	no	no	no	no	no

182171921	chr1	5343658	5343766	17	109	6.333333	no	no	no	no	no	no	no	no	no	no
182171925	chr1	5347227	5347308	43	82	1.953488	no	no	no	no	no	no	no	yes	no	no
182171928	chr1	5348953	5349017	23	65	2.826087	no	no	no	no	no	no	no	yes	yes	no
182171931	chr1	5359916	5359958	21	43	2.142857	no	no	no	no	no	no	no	no	no	no
182171933	chr1	5375050	5375087	20	38	1.900000	no	no	no	no	no	no	no	yes	no	no
182171934	chr1	5380963	5381020	29	58	2.000000	no	no	no	no	no	no	no	no	no	no
182171935	chr1	5382496	5382524	15	29	1.933333	no	no	no	no	no	no	no	no	no	no
182171942	chr1	5394639	5394688	26	50	2.000000	no	no	no	no	no	no	no	yes	no	no
182171958	chr1	5404624	5405026	208	403	2.000000	no	no	no	no	no	no	no	yes	yes	no
182171968	chr1	5417230	5417271	22	42	1.909091	no	no	no	no	no	no	no	no	no	no
182171972	chr1	5428664	5428695	16	32	2.125000	no	no	no	no	no	no	no	yes	yes	no
182171979	chr1	5430341	5430491	35	151	4.342857	no	no	no	no	no	no	no	no	yes	no
182171989	chr1	5444213	5444258	18	46	2.578947	no	no	no	no	no	no	no	no	no	no
182171990	chr1	5444764	5444796	15	33	2.200000	no	no	no	no	no	no	no	yes	no	no
182171994	chr1	5470155	5470225	17	71	4.176471	no	no	no	no	no	no	no	yes	no	no
182172003	chr1	5490701	5490748	18	48	2.666667	no	no	yes	no	yes	yes	no	no	yes	yes
182172005	chr1	5497923	5497986	30	64	2.200000	no	no	no	no	no	no	no	yes	no	no
182172015	chr1	5500765	5500850	30	86	2.866667	no	no	no	no	no	no	no	yes	yes	no
182172017	chr1	5509812	5509845	7	34	4.857143	no	no	no	no	no	no	no	yes	no	no
182172018	chr1	5511651	5511692	22	42	1.909091	no	no	no	no	no	no	no	no	no	no
182172026	chr1	5557319	5557344	12	26	2.166667	no	no	no	no	no	no	no	no	no	no
182172037	chr1	5577473	5577505	15	33	2.200000	no	no	yes	no	no	no	no	no	no	yes
182172041	chr1	5578121	5578158	20	38	1.900000	no	no	yes	no	no	no	no	yes	no	yes
182172046	chr1	5582785	5582853	10	69	6.727273	no	no	yes	no	no	no	no	no	no	yes
182172052	chr1	5600965	5600997	16	33	2.062500	no	no	yes	no	no	no	no	yes	no	yes

182172054	chr1	5601626	5601952	61	327	5.403226	no	no	yes	no	no	no	no	yes	no	yes
182172060	chr1	5606354	5606414	32	61	1.937500	no	no	yes	no	no	no	no	no	no	yes
182172062	chr1	5612233	5612301	33	69	2.090909	no	no	yes	no	no	no	no	yes	no	yes
182172063	chr1	5620232	5620256	12	25	2.083333	no	no	yes	no	no	no	no	no	no	yes
182172067	chr1	5632738	5632831	28	94	3.344828	no	no	yes	no	no	no	no	no	no	yes
182172069	chr1	5633567	5634078	94	512	5.595745	no	no	yes	no	no	no	no	yes	no	yes
182172075	chr1	5651528	5651609	41	82	2.000000	no	no	yes	no	no	no	no	yes	no	yes
182172077	chr1	5653698	5653771	32	74	2.312500	no	no	yes	no	no	no	no	no	no	yes
182172093	chr1	5662106	5662251	36	146	4.055555	no	no	yes	no	no	no	no	no	no	yes
182172096	chr1	5667579	5668251	100	673	6.794117	no	no	yes	no	yes	no	no	yes	yes	yes
182172100	chr1	5680909	5680961	23	53	2.304348	no	no	no	no	no	no	no	no	yes	no
182172104	chr1	5688783	5688830	22	48	2.181818	no	no	no	no	no	no	no	yes	yes	no
182172105	chr1	5691866	5691934	25	69	2.730769	no	no	no	no	no	no	no	no	no	no
182172112	chr1	5701732	5701832	51	101	1.980392	no	no	no	no	no	no	no	no	no	no
182172120	chr1	5711995	5712433	54	439	8.017858	no	no	no	no	no	no	yes	no	no	no
182172123	chr1	5713524	5713712	63	189	3.000000	no	no	no	no	no	no	no	no	yes	no
182172124	chr1	5717645	5717705	31	61	1.967742	no	no	no	no	no	no	no	yes	no	no
182172127	chr1	5733456	5733709	28	254	9.071428	no	no	no	no	no	no	yes	no	no	no
182172130	chr1	5736250	5736294	20	45	2.300000	no	no	no	no	no	no	no	no	no	no
182172132	chr1	5736293	5736338	17	46	2.882353	no	no	no	no	no	no	no	no	no	no
182172148	chr1	5738449	5738484	18	36	2.000000	no	no	no	no	no	no	no	no	no	no
182172155	chr1	5746660	5746686	9	27	3.000000	no	no	no	no	no	no	no	yes	no	no
182172176	chr1	5767902	5767966	19	65	3.421053	no	no	no	no	no	no	no	yes	no	no
182172196	chr1	5803978	5804153	94	176	1.872340	no	no	no	no	no	no	no	yes	no	no
182172197	chr1	5804531	5804595	32	65	2.031250	no	no	no	no	no	no	no	no	no	no

182172204	chr1	5808776	5808834	27	59	2.259259	no	no	no	no	no	no	no	yes	no	no
182172205	chr1	5808818	5808852	18	35	1.944444	no	no	no	no	no	no	no	yes	no	no
182172208	chr1	5808890	5809063	82	174	2.195122	no	no	no	no	no	no	no	yes	no	no
182172214	chr1	5816487	5817645	24	1159	48.360001	no	no	no	no	no	no	no	yes	yes	no
182172229	chr1	5831475	5831559	45	85	1.933333	no	no	no	no	no	no	no	yes	yes	no
182172231	chr1	5833185	5833266	25	82	3.280000	no	no	no	no	no	no	no	no	no	no
182172232	chr1	5836094	5836433	168	340	2.023809	no	no	no	no	no	no	no	no	no	no
182172239	chr1	5865377	5865474	50	98	1.960000	no	no	yes	no	yes	no	no	no	yes	yes
182172251	chr1	5873797	5873886	46	90	2.000000	yes	no	yes	no	yes	no	no	yes	no	yes
182172256	chr1	5874033	5874089	15	57	3.800000	yes	no	yes	no	no	yes	no	yes	no	yes
182172258	chr1	5879147	5879187	19	41	2.157895	no	no	yes	no	no	yes	no	no	no	yes
182172261	chr1	5879776	5879889	18	114	6.200000	no	no	yes	no	no	no	no	yes	no	yes
182172265	chr1	5882157	5882399	27	243	9.000000	no	no	yes	no	yes	no	no	yes	no	yes
182172266	chr1	5883249	5883417	55	169	3.072727	yes	no	yes	no	no	no	no	no	no	yes
182172267	chr1	5883589	5883685	45	97	2.155555	yes	no	yes	no	no	no	no	no	no	yes
182172268	chr1	5884418	5885424	49	1007	20.959183	no	no	yes	no	no	no	no	yes	yes	yes
182172269	chr1	5886738	5886773	18	36	2.000000	no	yes	yes	no	no	no	no	yes	no	yes
182172270	chr1	5887921	5887959	18	39	2.166667	no	no	yes	no	no	no	no	no	no	yes
182172272	chr1	5895828	5895994	55	167	3.036364	no	no	yes	no	no	no	no	yes	no	yes
182172273	chr1	5907679	5907738	30	60	2.000000	no	no	yes	no	no	no	no	no	no	yes
182172274	chr1	5911643	5911684	13	42	3.230769	no	no	yes	no	no	no	no	no	no	yes
182172278	chr1	5915203	5915232	7	30	4.285714	no	no	yes	no	no	no	no	yes	yes	yes
182172280	chr1	5915579	5915640	32	62	1.937500	no	no	yes	no	no	no	no	yes	yes	yes
182172284	chr1	5934498	5934533	17	36	2.117647	no	no	yes	no	no	no	no	yes	no	yes
182172288	chr1	5939384	5939548	81	165	2.061728	no	no	yes	no	no	no	no	yes	yes	yes

182172292	chr1	5949815	5949916	53	102	1.943396	no	no	yes	no	no	no	no	yes	no	yes
182172293	chr1	5950064	5950088	13	25	1.923077	no	no	yes	no	no	no	no	yes	no	yes
182172294	chr1	5952555	5952626	20	72	3.600000	no	no	yes	no	no	no	no	no	no	yes
182172303	chr1	5957417	5957779	52	363	6.980769	no	no	yes	no	no	no	no	no	no	yes
182172308	chr1	5959655	5959990	121	336	2.772358	no	no	yes	no	no	no	no	yes	yes	yes
182172316	chr1	5977874	5977910	12	37	3.166667	no	no	yes	no	no	no	no	yes	yes	yes
182172322	chr1	5988745	5988891	49	147	3.000000	no	no	yes	no	no	no	no	no	yes	yes
182172323	chr1	5990632	5990667	13	36	2.923077	yes	no	yes	no	no	no	no	no	no	yes
182172324	chr1	5991922	5991969	7	48	6.857143	yes	no	yes	no	no	no	yes	yes	yes	yes
182172332	chr1	6004922	6005488	40	567	14.325000	no	no	yes	no	no	no	no	yes	yes	yes
182172335	chr1	6005886	6006762	40	877	22.560976	no	no	yes	no	no	no	no	yes	yes	yes
182172339	chr1	6007501	6007538	11	38	3.454545	no	yes	yes	no	no	no	no	yes	yes	yes
182172340	chr1	6009390	6009434	22	45	2.045455	no	no	yes	no	no	no	no	no	no	yes
182172344	chr1	6011211	6011510	79	300	3.800000	no	no	yes	no	no	no	no	yes	no	yes
182172357	chr1	6021467	6022546	44	1080	24.520834	no	no	yes	no	no	no	no	yes	yes	yes
182172360	chr1	6025462	6025508	23	47	2.043478	yes	no	yes	no	no	no	no	yes	yes	yes
182172361	chr1	6025829	6025982	17	154	9.058824	yes	no	yes	no	no	no	no	yes	no	yes
182172371	chr1	6032629	6032665	11	37	3.333333	no	no	yes	no	no	no	no	no	no	yes
182172377	chr1	6040335	6040377	15	43	2.866667	no	no	yes	no	no	no	no	yes	yes	yes
182172378	chr1	6043252	6043320	35	69	1.971429	no	no	yes	no	no	no	no	no	no	yes
182172379	chr1	6047798	6047838	21	41	1.952381	no	no	yes	no	no	no	no	yes	no	yes
182172380	chr1	6058976	6059004	15	29	1.933333	no	no	yes	no	no	no	no	yes	no	yes
182172381	chr1	6061315	6061406	37	92	2.486486	no	no	yes	no	no	no	no	yes	no	yes
182172383	chr1	6064181	6064218	19	38	2.000000	no	no	yes	no	no	no	no	no	no	yes
182172385	chr1	6068774	6068812	20	39	1.950000	no	no	yes	no	no	no	no	yes	no	yes

182172389	chr1	6071636	6071734	34	99	2.885714	no	no	yes	no	no	no	yes	yes	yes	yes
182172391	chr1	6071728	6071913	18	186	10.050000	no	no	yes	no	no	no	yes	yes	yes	yes
182172394	chr1	6073256	6073284	15	29	2.000000	yes	no	yes	no	no	yes	no	yes	yes	yes
182172397	chr1	6086820	6086933	55	114	2.072727	yes	no	yes	no	no	yes	no	no	yes	yes
182172404	chr1	6096737	6096861	64	125	1.984375	no	yes	yes	yes	no	yes	no	yes	yes	yes
182172405	chr1	6099424	6099455	17	32	1.941176	no	yes	no	no	no	yes	no	yes	yes	yes
182172409	chr1	6108487	6108539	19	53	2.750000	no	no	yes	no	no	no	no	yes	no	yes
182172418	chr1	6119748	6119809	20	62	3.100000	no	no	yes	no	no	no	no	no	no	yes
182172424	chr1	6126192	6126233	21	42	2.047619	no	no	yes	no	no	no	no	yes	yes	yes
182172425	chr1	6126226	6126557	113	332	2.921739	no	no	yes	no	no	no	no	yes	yes	yes
182172437	chr1	6144455	6144495	20	41	2.200000	yes	no	yes	no	no	no	no	no	yes	yes
182172438	chr1	6148301	6148346	16	46	2.875000	no	no	yes	no	no	no	no	yes	yes	yes
182172441	chr1	6150922	6151033	57	112	1.982456	no	no	yes	yes	no	no	no	no	no	yes
182172444	chr1	6155051	6155212	40	162	4.000000	no	no	yes	no	no	no	no	yes	yes	yes
182172445	chr1	6155877	6155906	8	30	3.750000	no	no	yes	no	no	no	no	no	no	yes
182172446	chr1	6156682	6156811	59	130	2.203390	no	no	yes	no	no	no	no	no	no	yes
182172450	chr1	6159981	6160529	179	549	3.047872	no	no	yes	no	no	no	no	no	no	yes
182172455	chr1	6170492	6170528	19	37	1.947368	no	no	yes	no	no	no	no	no	no	yes
182172461	chr1	6175857	6175887	15	31	2.066667	no	no	yes	no	no	no	no	yes	no	yes
182172462	chr1	6175969	6176006	18	38	2.111111	no	no	yes	no	no	no	no	no	no	yes
182172466	chr1	6189168	6189254	20	87	4.350000	no	no	yes	no	no	no	no	yes	yes	yes
182172476	chr1	6203871	6203909	18	39	2.166667	yes	no	yes	no	no	yes	no	yes	no	yes
182172477	chr1	6206108	6206139	17	32	1.941176	yes	yes	yes	no	yes	no	yes	yes	yes	yes
182172478	chr1	6206558	6206592	18	35	1.944444	yes	yes	yes	yes	no	no	yes	yes	no	yes
182172484	chr1	6235681	6235711	10	31	3.100000	yes	no	yes	no	no	no	yes	yes	no	yes

182172486	chr1	6243887	6243916	16	30	1.937500	yes	no	no	no	no	no	no	yes	no	no
182172488	chr1	6244959	6245049	49	91	1.938776	no	no	yes	no	no	no	no	yes	no	yes
182172490	chr1	6249705	6249746	23	42	1.956522	no	no	no	yes	no	no	yes	yes	no	yes
182172494	chr1	6258776	6258822	23	47	2.043478	no	no	yes	no	no	no	no	yes	yes	yes
182172495	chr1	6259887	6259973	43	87	2.023256	no	no	yes	no	no	no	no	yes	yes	yes
182172496	chr1	6260758	6260789	14	32	2.357143	no	no	yes	no	no	no	yes	yes	yes	yes
182172499	chr1	6265188	6265348	42	161	3.833333	no	no	yes	no	no	no	no	no	yes	yes
182172502	chr1	6271599	6271648	21	50	2.380952	no	no	yes	no	no	no	no	yes	yes	yes
182172508	chr1	6299452	6299598	47	147	3.127660	no	no	yes	no	no	no	no	yes	no	yes
182172511	chr1	6300462	6300842	118	381	3.170543	no	no	yes	no	no	no	no	yes	yes	yes
182172528	chr1	6314740	6314974	80	235	2.939759	no	no	yes	no	no	no	no	yes	no	yes
182172538	chr1	6333704	6333735	8	32	4.125000	no	no	yes	no	no	no	no	no	no	yes
182172548	chr1	6354600	6354894	30	295	9.935484	no	no	yes	no	no	no	no	yes	yes	yes
182172550	chr1	6356392	6356476	19	85	4.473684	no	no	yes	no	no	no	no	yes	no	yes
182172560	chr1	6381205	6381247	22	43	1.954545	no	no	yes	no	no	no	no	no	no	yes
182172561	chr1	6386073	6386107	16	35	2.187500	yes	no	yes	no	no	no	no	yes	yes	yes
182172565	chr1	6393476	6393512	17	37	2.176471	yes	no	no	no	yes	no	yes	yes	yes	yes
182172569	chr1	6399638	6399677	20	40	2.000000	no	no	no	no	no	no	no	yes	no	no
182172588	chr1	6423664	6423693	14	30	2.142857	no	no	yes	no	no	no	no	yes	yes	yes
182172596	chr1	6436896	6437040	72	145	2.013889	yes	no	yes	no	no	no	no	yes	no	yes
182172598	chr1	6447121	6447254	19	134	7.000000	yes	no	yes	no	no	no	yes	yes	yes	yes
182172604	chr1	6460711	6460752	21	42	2.047619	no	yes	yes	no	no	yes	yes	yes	no	yes
182172608	chr1	6463922	6463982	24	61	2.583333	yes	no	yes	no	yes	yes	no	yes	no	yes
182172609	chr1	6465329	6465374	23	46	2.000000	yes	no	yes	no	no	yes	no	yes	no	yes
182172613	chr1	6475173	6475286	24	114	4.653846	no	no	yes	no	no	no	no	yes	yes	yes

182172626	chr1	6505231	6505320	34	90	2.647059	no	no	yes	no	no	no	no	yes	no	yes
182172649	chr1	6544513	6544544	12	32	2.666667	no	no	yes	no	no	no	no	no	yes	yes
182172650	chr1	6547920	6547995	24	76	3.120000	no	yes	yes	no	no	no	no	yes	no	yes
182172663	chr1	6582645	6582687	21	43	2.047619	no	no	yes	no	no	yes	no	yes	no	yes
182172668	chr1	6586900	6586925	9	26	2.888889	yes	yes	yes	no	no	no	no	yes	no	yes
182172673	chr1	6602730	6602764	18	35	1.944444	no	no	yes	yes	no	no	yes	yes	yes	yes
182172681	chr1	6613202	6613226	7	25	3.571429	yes	no	yes	no	no	no	yes	yes	yes	yes
182172682	chr1	6614297	6614336	21	40	1.904762	no	no	yes	no	no	no	yes	yes	yes	yes
182172683	chr1	6620582	6620606	12	25	2.083333	no	no	yes	no	no	no	no	no	no	yes
182172686	chr1	6625121	6625176	19	56	3.000000	yes	yes	yes	no	yes	no	yes	yes	yes	yes
182172689	chr1	6641036	6641081	17	46	2.705882	no	no	yes	no	no	no	no	yes	no	yes
182172692	chr1	6649933	6649958	10	26	2.600000	no	no	yes	no	no	no	no	no	no	yes
182172703	chr1	6670302	6670343	19	42	2.210526	no	no	yes	no	no	yes	no	no	no	yes
182172708	chr1	6679413	6679447	16	35	2.187500	no	no	yes	no	no	no	no	no	no	yes
182172711	chr1	6686252	6686285	16	34	2.125000	no	no	yes	no	no	no	no	yes	yes	yes
182172722	chr1	6698934	6698976	20	43	2.150000	no	no	yes	no	no	no	no	no	no	yes
182172742	chr1	6721534	6721574	20	41	2.050000	no	no	no	no	no	no	no	no	no	no
182172763	chr1	6747206	6747262	25	57	2.280000	no	no	no	no	no	no	no	no	yes	no
182172765	chr1	6748673	6748733	15	61	4.066667	no	no	no	no	no	no	no	yes	no	no
182172774	chr1	6751476	6751505	15	30	2.000000	no	no	no	no	no	no	no	yes	no	no
182172783	chr1	6767482	6767511	12	30	2.461539	yes	no	no	no	no	no	no	no	no	no
182172784	chr1	6768633	6768665	17	33	1.941176	no	yes	no	no	no	no	no	no	no	yes
182172795	chr1	6784690	6784731	22	42	1.909091	yes	no	no	no	yes	no	yes	yes	yes	yes
182172798	chr1	6793026	6793078	23	53	2.304348	no	no	yes	no	no	no	no	yes	no	yes
182172799	chr1	6793067	6793091	11	25	2.272727	no	no	yes	no	no	no	no	yes	no	yes

182172806 chr1 6807859 6807906 16 48 3.000000 no no yes no no no
no yes yes yes
182172807 chr1 6814716 6814747 16 32 2.000000 no no yes no no no
no no no yes
182172809 chr1 6825225 6825263 16 39 2.470588 no no yes no no no
no yes no yes
182172810 chr1 6825469 6825497 15 29 2.000000 no no yes no no no
no yes yes yes
182172814 chr1 6834247 6834288 19 42 2.210526 no no yes no no yes
no no no yes
182172818 chr1 6836037 6836076 20 40 2.000000 no no yes no no no
no no no yes
182172821 chr1 6836573 6836619 10 47 4.583333 no no yes no no no
no yes no yes
182172823 chr1 6838564 6838593 15 30 2.000000 no no yes no no no
no no no yes
182172833 chr1 6868350 6868391 21 42 2.000000 no no yes no no no
no yes yes yes
182172834 chr1 6871847 6871881 18 35 1.944444 no yes yes no no yes
no yes no yes
182172835 chr1 6871980 6872017 19 38 2.052632 no yes yes no no yes
no yes yes yes
182172843 chr1 6911486 6911604 30 119 3.843750 no no yes no no no
no yes yes yes
182172849 chr1 6926297 6926356 30 60 2.033333 no no yes no no no
no yes no yes
182172853 chr1 6926643 6926688 19 46 2.421053 no no yes no no no
no yes no yes
182172858 chr1 6929998 6930104 52 107 2.057692 no no yes no no no
no no no yes
182172862 chr1 6937805 6937860 24 56 2.458333 no no yes no no no
no yes no yes
182172865 chr1 6943166 6943223 8 58 7.250000 no no yes no no no
no yes no yes
182172867 chr1 6962801 6962839 18 39 2.166667 no no yes no no no
no yes yes yes
182172872 chr1 6964129 6964340 112 212 1.937500 no no yes no no no
no no no yes
182172879 chr1 6979733 6979892 53 160 3.018868 no no yes no no no
no no no yes
182172882 chr1 6983938 6983974 18 37 2.055556 no no yes no no no
no no no yes
182172886 chr1 6984103 6984223 31 121 4.000000 no no yes no no no
no no no yes
182172891 chr1 7001138 7001237 50 100 2.000000 no no yes no no no
no yes no yes
182172901 chr1 7051664 7051727 24 64 2.666667 no no yes no no no
no no no yes
182172903 chr1 7055869 7056020 75 152 2.026667 no no yes no no no
no no no yes

182172909	chr1	7064265	7064301	16	37	2.312500	no	no	yes	no	no	no	no	no	no	yes
182172917	chr1	7101892	7101932	20	41	2.100000	no	no	yes	no	no	no	no	yes	no	yes
182172922	chr1	7103716	7103759	15	44	3.000000	no	no	yes	no	no	no	no	yes	yes	yes
182172934	chr1	7121227	7121286	27	60	2.214286	no	no	yes	no	no	no	no	yes	no	yes
182172963	chr1	7148151	7148192	18	42	2.388889	no	no	yes	no	no	no	no	yes	no	yes
182172967	chr1	7148237	7148299	23	63	2.739130	no	no	yes	no	no	no	no	yes	no	yes
182172974	chr1	7173879	7173918	21	40	2.000000	no	no	yes	no	no	no	no	no	no	yes
182172980	chr1	7177436	7177524	40	89	2.250000	no	no	yes	no	no	no	no	yes	no	yes
182172994	chr1	7222704	7222740	19	37	1.947368	no	no	yes	no	no	no	no	no	no	yes
182173009	chr1	7245966	7246047	43	82	1.906977	no	no	yes	no	no	no	no	yes	yes	yes
182173015	chr1	7264110	7264142	15	33	2.200000	no	no	yes	no	no	no	no	yes	yes	yes
182173032	chr1	7284826	7284873	19	48	2.526316	no	no	yes	no	no	no	no	yes	no	yes
182173052	chr1	7337480	7337729	29	250	8.655172	no	no	yes	no	no	no	no	yes	no	yes
182173063	chr1	7347395	7347502	55	108	1.963636	no	no	yes	no	no	no	no	no	no	yes
182173068	chr1	7362456	7362508	27	53	1.962963	no	no	yes	no	no	no	no	yes	no	yes
182173070	chr1	7365417	7365454	20	38	1.950000	no	no	yes	no	no	no	no	no	no	yes
182173087	chr1	7404908	7404964	21	57	2.772727	no	no	yes	no	no	no	no	no	no	yes
182173096	chr1	7428051	7428088	18	38	2.111111	no	no	yes	no	no	no	no	no	yes	yes
182173104	chr1	7430098	7430425	153	328	2.198718	no	no	yes	no	no	no	no	yes	no	yes
182173110	chr1	7434034	7434127	34	94	2.742857	no	no	yes	no	no	no	no	no	yes	yes
182173118	chr1	7456984	7457026	16	43	2.750000	no	no	yes	no	no	no	no	yes	yes	yes
182173123	chr1	7457043	7457106	28	64	2.285714	no	no	yes	no	no	no	no	yes	no	yes
182173126	chr1	7457483	7457551	35	69	1.971429	no	no	yes	no	no	no	no	no	no	yes
182173137	chr1	7479641	7479672	15	32	2.133333	no	no	yes	no	no	no	no	yes	yes	yes
182173152	chr1	7488482	7488546	28	65	2.392857	no	no	yes	no	no	no	no	yes	no	yes

182173156	chr1	7488546	7488617	35	72	2.114286	no	no	yes	no	no	no	no	yes	no	yes
182173181	chr1	7498778	7499615	75	838	11.250000	no	no	yes	no	no	no	no	yes	no	yes
182173189	chr1	7499495	7499851	185	357	2.005405	no	no	yes	no	no	no	no	yes	no	yes
182173193	chr1	7499924	7499957	17	34	2.000000	no	no	yes	no	no	no	no	yes	no	yes
182173196	chr1	7502897	7502927	14	31	2.214286	no	no	yes	no	no	no	no	no	no	yes
182173197	chr1	7504887	7505134	83	248	2.987952	no	no	yes	no	no	no	no	no	no	yes
182173198	chr1	7506427	7506486	13	60	4.500000	no	no	yes	no	no	no	no	no	no	yes
182173201	chr1	7506679	7506844	77	166	2.215190	no	no	yes	no	no	no	no	yes	no	yes
182173211	chr1	7506954	7507140	91	187	2.098901	no	no	yes	no	no	no	no	yes	no	yes
182173220	chr1	7514955	7515220	38	266	7.026316	no	no	yes	no	no	no	no	yes	no	yes
182173226	chr1	7531530	7531559	15	30	2.000000	no	no	yes	no	no	no	no	yes	no	yes
182173228	chr1	7534945	7534999	25	55	2.200000	no	no	yes	no	no	no	no	no	no	yes
182173230	chr1	7536115	7536167	21	53	2.523809	no	no	yes	no	no	no	no	yes	no	yes
182173236	chr1	7548349	7549304	358	956	2.672222	no	no	yes	no	no	no	no	yes	no	yes
182173238	chr1	7550058	7550102	22	45	2.090909	no	no	yes	no	no	no	no	yes	yes	yes
182173242	chr1	7551311	7551371	32	61	1.906250	no	no	yes	no	no	no	no	yes	yes	yes
182173249	chr1	7586114	7586146	17	33	1.941176	no	no	yes	no	no	no	no	yes	yes	yes
182173269	chr1	7613733	7613793	29	61	2.137931	no	no	yes	no	no	no	no	no	yes	yes
182173272	chr1	7613832	7614019	99	188	1.929293	no	no	yes	no	no	no	no	no	no	yes
182173276	chr1	7631991	7632044	10	54	5.400000	no	no	yes	no	no	no	no	no	no	yes
182173278	chr1	7641887	7641922	11	36	3.272727	no	no	yes	no	no	no	no	yes	yes	yes
182173279	chr1	7645973	7646026	24	54	2.291667	no	no	yes	no	no	no	no	yes	yes	yes
182173281	chr1	7646651	7646766	45	116	2.565217	no	no	yes	no	no	no	no	yes	no	yes
182173292	chr1	7680888	7680943	20	56	2.800000	no	no	yes	no	no	no	yes	yes	yes	yes
182173293	chr1	7685006	7685189	45	184	4.088889	no	no	yes	no	no	no	no	no	no	yes

182173296	chr1	7692505	7692546	12	42	3.500000	no	no	yes	no	no	yes	no	no	no	yes
182173297	chr1	7704019	7704047	14	29	2.142857	no	no	yes	no	no	no	no	yes	yes	yes
182173299	chr1	7704781	7704829	15	49	3.187500	no	no	yes	no	no	no	yes	yes	no	yes
182173301	chr1	7705510	7705570	32	61	1.906250	no	no	yes	no	no	no	yes	yes	yes	yes
182173302	chr1	7709065	7709090	8	26	3.250000	no	no	yes	no	no	no	no	no	no	yes
182173304	chr1	7725874	7726014	51	141	2.862745	no	no	yes	no	no	no	no	no	no	yes
182173308	chr1	7742359	7742404	16	46	2.937500	no	no	yes	no	no	no	no	no	no	yes
182173327	chr1	7784385	7784428	21	44	2.095238	yes	no	yes	no	no	no	yes	yes	no	yes
182173330	chr1	7810556	7810598	21	43	2.047619	no	no	yes	yes	yes	yes	no	yes	yes	yes
182173334	chr1	7829874	7830126	54	253	4.685185	no	no	yes	yes	no	no	no	no	no	yes
182173346	chr1	7859237	7859268	10	32	3.200000	no	no	no	no	no	no	no	no	no	no
182173353	chr1	7864877	7865546	57	670	11.929825	no	no	no	no	no	no	no	no	no	no
182173377	chr1	7894130	7894163	16	34	2.125000	no	no	no	no	no	no	no	yes	no	no
182173386	chr1	7902990	7903365	9	376	42.222221	no	no	no	no	no	no	no	yes	yes	no
182173395	chr1	7914209	7914258	17	50	2.941176	no	no	no	no	no	no	no	no	yes	no
182173410	chr1	7938023	7938063	17	41	2.411765	no	no	yes	no	no	no	no	yes	no	yes
182173416	chr1	7942091	7942192	45	102	2.266667	yes	no	yes	no	no	no	no	yes	yes	yes
182173420	chr1	7944180	7944243	33	64	1.969697	no	no	no	no	no	no	no	no	no	no
182173423	chr1	7949911	7949952	19	42	2.210526	no	no	no	no	no	no	no	no	yes	no
182173436	chr1	7961851	7961885	18	35	1.944444	yes	no	yes	no	no	no	yes	yes	yes	yes
182173438	chr1	7971255	7971289	16	35	2.187500	no	yes	yes	no	no	yes	no	yes	yes	yes
182173444	chr1	7988017	7988060	22	44	2.000000	no	no	no	no	no	no	no	no	yes	no
182173462	chr1	8016832	8016866	18	35	1.944444	no	no	yes	no	no	no	no	yes	yes	yes
182173467	chr1	8027079	8027209	68	131	1.955882	yes	yes	no	no	no	no	no	yes	yes	yes
182173475	chr1	8040364	8040425	30	62	2.066667	no	no	yes	no	no	no	no	yes	no	yes

182173496	chr1	8062102	8062144	20	43	2.150000	no	no	yes	no	no	no	no	yes	yes	yes
182173499	chr1	8064767	8064799	13	33	2.538461	no	no	yes	no	no	no	no	no	no	yes
182173506	chr1	8073336	8073378	14	43	3.066667	no	no	yes	no	no	no	no	yes	yes	yes
182173511	chr1	8115534	8115615	38	82	2.184211	no	no	yes	no	no	no	no	yes	no	yes
182173517	chr1	8127187	8127219	12	33	2.833333	no	no	no	no	no	no	no	yes	yes	no
182173531	chr1	8157549	8157580	14	32	2.285714	no	no	no	no	no	no	no	no	no	no
182173535	chr1	8161669	8161711	19	43	2.263158	no	no	no	no	no	no	no	no	yes	no
182173537	chr1	8163467	8163520	16	54	3.437500	no	no	no	no	no	no	no	yes	no	no
182173549	chr1	8175648	8175698	26	51	1.961538	no	no	no	no	no	no	no	no	no	no
182173556	chr1	8191050	8191075	12	26	2.166667	no	no	yes	no	no	no	no	yes	no	yes
182173564	chr1	8211315	8211342	7	28	4.000000	no	no	yes	no	no	no	no	yes	yes	yes
182173569	chr1	8215889	8215938	26	50	1.923077	no	no	no	no	no	yes	no	yes	yes	no
182173572	chr1	8219481	8219526	22	46	2.090909	no	no	yes	no	no	no	no	no	no	yes
182173580	chr1	8232335	8232379	22	45	2.045455	no	no	no	no	no	no	no	yes	yes	no
182173582	chr1	8233721	8233761	20	41	2.100000	no	no	no	no	no	no	no	no	no	no
182173625	chr1	8272601	8272645	17	45	2.666667	no	no	no	no	no	no	no	no	no	no
182173634	chr1	8284013	8284053	21	41	1.952381	no	no	no	no	no	no	no	no	yes	no
182173637	chr1	8301121	8301976	28	856	30.678572	no	no	no	no	no	no	no	yes	no	no
182173638	chr1	8302718	8302768	24	51	2.125000	no	no	no	no	no	no	no	yes	yes	no
182173653	chr1	8321670	8321723	29	54	1.931034	no	no	yes	no	no	no	no	yes	yes	yes
182173657	chr1	8331248	8331471	39	224	5.697674	no	no	yes	no	no	no	no	no	no	yes
182173658	chr1	8332419	8332455	14	37	2.785714	no	no	yes	no	no	no	no	no	no	yes
182173660	chr1	8333224	8333272	24	49	2.125000	no	no	yes	no	no	no	no	no	no	yes
182173669	chr1	8347933	8347960	14	28	2.000000	no	no	no	no	no	no	no	yes	yes	no
182173673	chr1	8358581	8358640	15	60	4.000000	no	no	yes	yes	no	no	no	yes	yes	yes

182173674	chr1	8359769	8359811	18	43	2.388889	no	no	yes	yes	no	no	no	yes	yes	yes
182173677	chr1	8361027	8361073	24	47	1.958333	no	no	yes	yes	no	yes	no	yes	yes	yes
182173684	chr1	8374747	8374776	15	30	2.000000	no	no	yes	no	no	no	no	yes	no	yes
182173685	chr1	8375499	8375837	28	339	12.107142	no	no	yes	no	no	no	no	yes	yes	yes
182173691	chr1	8392337	8392367	16	31	1.937500	no	no	yes	no	no	no	no	no	no	yes
182173699	chr1	8423717	8423754	16	38	2.437500	yes	no	yes	no	no	no	yes	yes	yes	yes
182173703	chr1	8428787	8428820	17	34	2.000000	no	no	yes	no	no	no	no	no	no	yes
182173704	chr1	8432393	8432476	30	84	2.800000	no	no	yes	no	no	no	no	yes	no	yes
182173705	chr1	8432939	8432969	12	31	2.583333	no	no	yes	no	no	no	no	no	no	yes
182173711	chr1	8448948	8448983	14	36	2.571429	no	no	yes	no	no	no	no	yes	yes	yes
182173712	chr1	8450443	8450479	18	37	2.055556	no	no	yes	no	no	no	no	yes	yes	yes
182173713	chr1	8467234	8467277	23	44	1.913043	no	no	yes	no	no	no	no	yes	no	yes
182173715	chr1	8470661	8470718	28	58	2.071429	no	no	yes	no	no	no	no	yes	yes	yes
182173720	chr1	8481474	8481510	16	37	2.375000	no	no	yes	no	no	no	no	no	no	yes
182173722	chr1	8482842	8482880	19	39	2.105263	no	no	yes	no	no	no	no	yes	no	yes
182173735	chr1	8500123	8500165	15	43	2.933333	no	no	yes	no	no	no	no	no	no	yes
182173764	chr1	8526169	8526286	57	118	2.105263	yes	no	yes	no	no	no	no	yes	yes	yes
182173765	chr1	8526635	8526690	24	56	2.360000	yes	no	yes	no	no	no	no	yes	yes	yes
182173773	chr1	8533867	8533895	13	29	2.230769	no	no	yes	no	no	no	no	yes	no	yes
182173776	chr1	8540070	8540110	19	41	2.157895	no	no	yes	no	no	no	no	no	no	yes
182173778	chr1	8545634	8545683	18	50	2.833333	no	no	yes	no	no	no	no	yes	no	yes
182173787	chr1	8554706	8554918	35	213	6.085714	no	no	yes	no	no	no	no	no	no	yes
182173788	chr1	8557032	8557070	19	39	2.052632	no	no	yes	no	no	no	no	no	no	yes
182173799	chr1	8595444	8595556	45	113	2.604167	no	no	yes	no	no	no	no	no	no	yes
182173804	chr1	8601565	8601783	111	219	1.972973	no	no	yes	no	no	no	no	no	yes	yes

182173810	chr1	8612746	8612781	17	36	2.117647	no	no	yes	no	no	no	no	no	no	yes
182173812	chr1	8615986	8616018	15	33	2.266667	no	no	yes	no	no	no	no	no	no	yes
182173819	chr1	8635209	8635254	24	46	1.916667	no	no	yes	no	no	no	no	no	no	yes
182173825	chr1	8646056	8646149	46	94	2.043478	no	no	yes	no	no	no	no	no	yes	yes
182173826	chr1	8647243	8647282	20	40	2.000000	no	no	yes	no	no	no	no	yes	no	yes
182173831	chr1	8656233	8656258	12	26	2.166667	no	no	no	yes	yes	no	no	no	no	yes
182173836	chr1	8660046	8660079	18	34	1.944444	no	no	yes	no	no	no	no	yes	no	yes
182173846	chr1	8684674	8684723	25	50	2.000000	no	no	yes	no	no	no	no	yes	yes	yes
182173853	chr1	8702412	8702447	15	36	2.466667	no	no	yes	no	no	no	no	yes	yes	yes
182173858	chr1	8710268	8710332	34	65	1.911765	no	no	yes	no	no	no	no	no	no	yes
182173876	chr1	8757673	8757711	20	39	1.950000	no	no	yes	no	no	no	no	no	yes	yes
182173889	chr1	8777853	8777900	21	48	2.333333	no	no	yes	no	no	no	no	yes	no	yes
182173900	chr1	8813076	8813119	22	44	2.000000	no	no	yes	no	no	no	no	yes	no	yes
182173920	chr1	8845863	8845933	36	71	1.972222	no	no	yes	no	no	no	no	yes	no	yes
182173923	chr1	8849212	8849240	13	29	2.230769	yes	no	no	no	no	no	no	yes	no	no
182173934	chr1	8879133	8879170	14	38	2.714286	yes	no	yes	no	yes	no	yes	yes	yes	yes
182173956	chr1	8904386	8904427	15	42	2.800000	no	no	no	no	no	no	no	yes	no	no
182173986	chr1	8949568	8949593	13	26	2.000000	no	no	yes	no	no	no	no	no	yes	yes
182173990	chr1	8955612	8955663	23	52	2.260870	no	no	yes	no	no	no	no	no	no	yes
182173997	chr1	8975146	8975180	12	35	2.916667	no	no	no	no	no	yes	no	yes	no	no
182173998	chr1	8976114	8976212	51	99	1.941176	no	no	no	no	no	no	no	no	no	no
182174011	chr1	8984580	8984613	10	34	3.500000	no	no	no	no	no	no	no	yes	no	no
182174012	chr1	8990119	8990214	40	96	2.425000	no	no	no	no	no	no	no	yes	yes	no
182174023	chr1	9016657	9016716	24	60	2.500000	no	no	yes	no	no	no	no	no	no	yes
182174027	chr1	9018747	9018779	17	33	2.000000	no	no	yes	no	no	no	no	yes	no	yes

182174030	chr1	9024948	9024982	11	35	3.181818	no	no	yes	yes	no	no	no	yes	no	yes
182174037	chr1	9037257	9037342	45	86	1.911111	no	yes	no	no	no	no	no	yes	yes	yes
182174038	chr1	9039715	9039758	21	44	2.095238	no	no	yes	no	yes	yes	yes	yes	yes	yes
182174039	chr1	9042534	9042600	29	67	2.310345	no	no	yes	no	no	no	no	yes	no	yes
182174044	chr1	9051192	9051253	26	62	2.423077	no	no	yes	no	no	no	no	yes	no	yes
182174061	chr1	9071877	9071964	28	88	3.142857	yes	no	yes	no	no	no	no	yes	yes	yes
182174109	chr1	9154945	9156003	41	1059	25.860466	no	no	yes	no	no	no	no	yes	no	yes
182174114	chr1	9160012	9160094	36	83	2.305556	no	no	yes	no	no	no	no	yes	no	yes
182174116	chr1	9161955	9161995	18	41	2.333333	no	no	yes	no	no	no	no	yes	no	yes
182174133	chr1	9201836	9201873	15	38	2.533333	no	no	no	no	no	no	no	yes	yes	no
182174147	chr1	9231999	9232034	18	36	2.000000	no	no	no	no	no	no	no	yes	no	no
182174153	chr1	9234702	9234762	15	61	4.133333	yes	no	no	no	no	no	yes	yes	yes	no
182174156	chr1	9250350	9250571	38	222	5.842105	no	no	yes	no	no	no	no	yes	yes	yes
182174163	chr1	9258279	9258323	22	45	2.045455	no	no	yes	no	no	no	no	yes	yes	yes
182174166	chr1	9258339	9258533	105	195	1.933333	no	no	yes	no	no	no	no	yes	yes	yes
182174172	chr1	9258699	9258854	79	156	2.037975	no	no	yes	no	no	no	no	yes	no	yes
182174179	chr1	9259288	9259353	28	66	2.464286	no	no	yes	no	no	no	no	no	no	yes
182174187	chr1	9259581	9259802	123	222	1.804878	no	no	yes	no	no	no	no	yes	yes	yes
182174189	chr1	9259788	9259855	33	68	2.060606	no	no	yes	no	no	no	no	yes	no	yes
182174205	chr1	9260539	9260748	97	210	2.195876	no	no	yes	no	no	no	no	yes	yes	yes
182174210	chr1	9275652	9275746	42	95	2.261905	no	no	no	no	no	no	no	yes	yes	no
182174214	chr1	9280366	9280451	18	86	4.777778	no	no	no	no	no	no	no	yes	yes	no
182174217	chr1	9282485	9282528	23	44	1.913043	no	no	no	no	no	no	no	yes	yes	no
182174224	chr1	9293118	9293162	24	45	1.916667	no	no	yes	no	no	no	yes	yes	yes	yes
182174228	chr1	9296523	9296573	26	51	1.961538	no	no	yes	no	no	no	no	yes	yes	yes

182174229	chr1	9296595	9296632	14	38	2.714286	no	no	yes	no	no	no	no	yes	yes	yes
182174237	chr1	9309262	9309306	22	45	2.045455	no	no	yes	no	no	no	no	yes	yes	yes
182174239	chr1	9310074	9310230	54	157	2.907408	no	no	yes	no	no	no	no	yes	yes	yes
182174248	chr1	9319611	9319652	18	42	2.388889	no	no	yes	no	no	no	no	yes	yes	yes
182174249	chr1	9324054	9324083	15	30	2.000000	no	no	yes	no	no	no	no	no	no	yes
182174256	chr1	9337389	9337430	20	42	2.150000	no	no	yes	no	no	no	no	no	no	yes
182174261	chr1	9350004	9350075	29	72	2.482759	no	no	yes	no	no	no	no	yes	yes	yes
182174265	chr1	9352820	9352861	20	42	2.150000	no	no	yes	no	no	no	no	yes	yes	yes
182174266	chr1	9353172	9353229	29	58	2.000000	no	no	yes	no	no	no	no	yes	yes	yes
182174273	chr1	9372748	9372787	21	40	1.952381	no	no	no	no	no	no	no	yes	no	no
182174277	chr1	9384647	9384692	15	46	3.066667	no	no	no	no	no	no	no	yes	no	no
182174283	chr1	9407151	9407202	25	52	2.080000	no	no	no	no	no	no	no	no	yes	no
182174298	chr1	9417110	9417170	29	61	2.172414	no	no	no	no	no	no	no	yes	yes	no
182174302	chr1	9424535	9424566	16	32	2.000000	no	no	no	no	no	yes	no	yes	yes	no
182174306	chr1	9445699	9445744	22	46	2.090909	no	no	no	no	no	no	no	no	no	no
182174332	chr1	9483627	9483667	19	41	2.157895	no	no	no	no	no	no	no	no	yes	no
182174335	chr1	9492345	9492384	20	40	2.000000	no	no	no	no	no	no	no	yes	yes	no
182174338	chr1	9502302	9502381	34	80	2.400000	no	no	yes	no	no	no	no	no	no	yes
182174371	chr1	9536533	9536580	23	48	2.130435	no	no	no	no	no	no	no	yes	no	no
182174378	chr1	9549304	9549536	29	233	8.000000	no	no	yes	no	no	no	no	yes	yes	yes
182174390	chr1	9560692	9560732	18	41	2.277778	no	no	yes	no	no	no	no	yes	yes	yes
182174391	chr1	9561926	9561977	26	52	2.000000	no	no	yes	no	no	no	no	yes	no	yes
182174396	chr1	9570667	9570702	18	36	2.000000	no	no	yes	no	no	no	no	no	no	yes
182174401	chr1	9585006	9585043	20	38	1.900000	no	yes	no	no	no	no	no	no	no	yes
182174405	chr1	9588785	9588841	29	57	2.034483	yes	no	no	no	no	no	yes	yes	yes	no

182174407	chr1	9588833	9588910	8	78	9.750000	yes	no	no	no	no	no	yes	yes	no	no
182174408	chr1	9605265	9605426	54	162	3.000000	yes	no	yes	no	no	yes	no	yes	yes	yes
182174409	chr1	9608880	9608959	39	80	2.102564	no	no	yes	no	no	no	no	no	no	yes
182174422	chr1	9628783	9628821	18	39	2.166667	no	no	no	no	no	no	no	yes	yes	no
182174434	chr1	9643441	9643507	25	67	2.680000	no	no	no	no	no	no	no	yes	yes	no
182174469	chr1	9681850	9681965	56	116	2.071429	no	no	yes	no	no	no	no	yes	yes	yes
182174487	chr1	9710669	9710726	28	58	2.071429	no	no	yes	no	no	no	no	no	no	yes
182174490	chr1	9715800	9715843	22	44	2.000000	yes	no	yes	no	no	no	yes	yes	yes	yes
182174495	chr1	9721084	9721125	22	42	1.909091	no	no	yes	yes	no	no	no	yes	yes	yes
182174499	chr1	9731848	9731883	19	36	1.947368	no	yes	no	yes	yes	no	no	yes	yes	yes
182174500	chr1	9732076	9732115	21	40	1.952381	no	no	yes	no	no	no	no	yes	no	yes
182174503	chr1	9761500	9761535	12	36	2.923077	no	no	yes	no	no	no	no	no	no	yes
182174504	chr1	9762518	9762628	37	111	3.000000	no	no	yes	no	no	no	yes	no	no	yes
182174506	chr1	9762594	9762891	108	298	2.759259	no	no	yes	no	no	no	yes	no	no	yes
182174511	chr1	9768178	9768873	31	696	22.677420	no	no	yes	no	no	no	no	yes	yes	yes
182174518	chr1	9776799	9776902	22	104	4.666667	no	no	yes	no	no	no	no	yes	no	yes
182174535	chr1	9798126	9798184	18	59	3.388889	no	no	yes	no	no	no	no	yes	no	yes
182174558	chr1	9840252	9840287	15	36	2.400000	no	no	no	no	no	no	no	yes	yes	no
182174567	chr1	9858965	9858990	11	26	2.363636	no	no	yes	no	no	no	no	yes	no	yes
182174569	chr1	9863050	9863156	52	107	2.057692	no	no	yes	no	no	no	no	yes	no	yes
182174573	chr1	9871361	9871388	11	28	2.545455	no	no	yes	no	no	no	no	yes	no	yes
182174574	chr1	9875468	9875511	23	44	1.913043	no	no	yes	no	no	no	no	yes	yes	yes
182174576	chr1	9879214	9879251	13	38	2.866667	no	no	yes	no	no	no	no	yes	no	yes
182174578	chr1	9890953	9890993	20	41	2.050000	no	no	yes	no	no	no	no	no	no	yes
182174607	chr1	9928317	9928347	15	31	2.133333	no	yes	yes	no	no	no	no	yes	no	yes

182174635	chr1	9984376	9984419	18	44	2.500000	no	yes	yes	no	no	yes	no	no	no	yes
182174659	chr1	10009861	10009901	16	41	2.625000	no	no	yes	no	no	no	no	no	no	yes
182174667	chr1	10020961	10020992	14	32	2.357143	no	no	no	no	no	no	no	yes	yes	no
182174686	chr1	10031642	10031694	25	53	2.120000	no	no	no	no	no	no	no	yes	yes	no
182174687	chr1	10031869	10031899	16	31	1.937500	yes	no	no	no	no	no	no	no	no	no
182174693	chr1	10037395	10037434	15	40	2.687500	no	no	yes	no	no	no	no	yes	no	yes
182174705	chr1	10071680	10071707	12	28	2.333333	no	no	yes	no	no	no	no	no	no	yes
182174717	chr1	10095141	10095165	13	25	1.923077	no	no	yes	no	no	no	no	no	no	yes
182174718	chr1	10097464	10097539	38	76	2.000000	no	no	yes	no	no	no	no	no	no	yes
182174729	chr1	10104700	10104904	107	205	1.925234	no	no	yes	no	no	no	no	no	no	yes
182174732	chr1	10107298	10107348	21	51	2.428571	no	no	yes	yes	no	no	no	yes	no	yes
182174752	chr1	10126503	10126542	18	40	2.222222	no	no	yes	no	no	no	no	yes	no	yes
182174754	chr1	10133055	10133113	24	59	2.458333	no	no	yes	no	no	no	no	no	yes	yes
182174778	chr1	10169348	10169378	15	31	2.133333	no	no	yes	no	no	no	no	yes	yes	yes
182174780	chr1	10174937	10174984	20	48	2.450000	no	no	yes	no	no	no	no	no	no	yes
182174791	chr1	10186869	10186938	37	70	1.918919	no	no	no	no	no	no	no	yes	yes	no
182174799	chr1	10190511	10190568	29	58	2.000000	no	no	no	no	no	no	no	yes	no	no
182174838	chr1	10278165	10278197	13	33	2.538461	yes	no	yes	no	no	no	no	no	no	yes
182174840	chr1	10281333	10281360	13	28	2.153846	no	no	yes	no	no	no	no	yes	no	yes
182174850	chr1	10322903	10322945	13	43	3.214286	no	no	yes	no	no	no	no	yes	no	yes
182174857	chr1	10352017	10352319	61	303	5.000000	yes	no	yes	no	no	no	no	yes	yes	yes
182174868	chr1	10366420	10366552	30	133	4.433333	no	no	yes	no	no	no	no	no	no	yes
182174873	chr1	10374549	10374592	22	44	2.000000	no	no	yes	no	no	no	no	yes	yes	yes
182174887	chr1	10404094	10404141	18	48	2.666667	no	no	yes	no	no	no	no	yes	no	yes
182174889	chr1	10405426	10405483	30	58	1.933333	no	no	yes	no	no	no	no	no	no	yes

182174890	chr1	10407981	10408017	18	37	2.055556	no	no	yes	no	no	no	no	yes	no	yes
182174924	chr1	10490274	10490365	44	92	2.090909	no	no	yes	no	no	no	no	yes	no	yes
182174931	chr1	10505531	10505557	11	27	2.454545	no	no	yes	no	no	no	no	no	no	yes
182174932	chr1	10507053	10507113	26	61	2.346154	no	no	yes	no	no	no	no	yes	no	yes
182174934	chr1	10511233	10511298	35	66	1.914286	no	no	yes	no	no	no	no	yes	yes	yes
182174935	chr1	10511573	10511618	24	46	1.916667	no	no	yes	no	no	no	no	yes	yes	yes
182174939	chr1	10514083	10514133	15	51	3.400000	no	no	yes	no	no	no	no	yes	yes	yes
182174941	chr1	10516384	10516427	21	44	2.095238	no	no	yes	no	no	no	no	yes	yes	yes
182174948	chr1	10549840	10549876	19	37	1.947368	no	no	yes	no	no	no	no	no	no	yes
182174950	chr1	10554360	10554392	15	33	2.200000	no	no	yes	no	no	no	no	yes	yes	yes
182174952	chr1	10565956	10565996	20	41	2.050000	no	no	yes	no	no	no	no	no	yes	yes
182174967	chr1	10581753	10581792	18	40	2.222222	no	no	yes	no	no	no	no	no	no	yes
182174974	chr1	10590187	10590248	20	62	3.100000	no	no	yes	no	no	no	no	no	no	yes
182174977	chr1	10605684	10605725	21	42	2.000000	no	no	yes	no	no	no	no	no	no	yes
182174986	chr1	10629813	10629845	9	33	3.666667	no	no	no	yes	no	no	no	yes	yes	yes
182174994	chr1	10639080	10639166	27	87	3.222222	yes	no	no	yes	no	no	yes	yes	yes	yes
182174995	chr1	10639226	10639278	24	53	2.208333	yes	yes	no	yes	no	no	yes	yes	yes	yes
182174998	chr1	10642348	10642388	21	41	1.952381	no	no	yes	no	no	no	no	yes	no	yes
182174999	chr1	10643133	10643169	17	37	2.176471	no	no	yes	yes	no	no	no	yes	no	yes
182175001	chr1	10651322	10651353	12	32	2.666667	yes	yes	yes	no	no	no	no	yes	no	yes
182175003	chr1	10657271	10657311	21	41	2.047619	no	no	yes	no	no	yes	no	yes	yes	yes
182175005	chr1	10659303	10659349	23	47	2.043478	no	no	yes	no	no	no	no	yes	no	yes
182175006	chr1	10659390	10659424	13	35	2.692308	no	no	yes	no	no	no	no	yes	no	yes
182175016	chr1	10662116	10662214	49	99	2.061224	yes	no	yes	no	no	no	no	yes	no	yes
182175023	chr1	10662721	10662762	17	42	2.470588	no	no	yes	no	no	no	no	yes	no	yes

182175024	chr1	10662995	10663027	18	33	1.944444	no	no	yes	no	no	no	no	yes	yes	yes
182175026	chr1	10676529	10676582	20	54	2.714286	no	no	yes	no	no	no	no	no	yes	yes
182175032	chr1	10694677	10694722	15	46	3.200000	yes	no	yes	no	no	no	yes	yes	yes	yes
182175040	chr1	10707657	10707687	16	31	2.000000	no	no	yes	no	no	no	no	no	no	yes
182175047	chr1	10713289	10713320	16	32	2.000000	no	no	yes	no	no	no	no	no	no	yes
182175050	chr1	10715472	10716312	21	841	40.238094	no	no	yes	no	no	no	no	no	no	yes
182175051	chr1	10722167	10722210	22	44	2.090909	no	no	yes	no	no	no	no	no	no	yes
182175053	chr1	10729087	10729131	22	45	2.136364	no	no	yes	no	no	no	no	yes	no	yes
182175056	chr1	10744345	10744731	24	387	16.125000	no	no	yes	no	no	no	no	yes	yes	yes
182175057	chr1	10747638	10747665	13	28	2.153846	no	no	yes	no	no	no	no	no	no	yes
182175058	chr1	10748224	10748329	54	106	1.962963	no	no	yes	no	no	no	no	yes	no	yes
182175062	chr1	10767093	10767223	64	131	2.046875	no	no	yes	no	no	no	no	yes	no	yes
182175071	chr1	10777665	10777720	26	56	2.153846	no	no	yes	no	no	no	no	yes	yes	yes
182175077	chr1	10778038	10778178	72	141	2.097222	no	no	yes	no	no	no	no	yes	no	yes
182175088	chr1	10792315	10792346	14	32	2.285714	no	no	yes	no	no	no	no	no	yes	yes
182175093	chr1	10809572	10809657	39	86	2.275000	no	no	no	no	no	no	no	no	no	no
182175095	chr1	10819674	10819718	20	45	2.300000	no	no	no	no	no	no	no	yes	yes	no
182175098	chr1	10824382	10824454	36	73	2.027778	no	no	no	no	no	no	no	no	yes	no
182175106	chr1	10835367	10835398	15	32	2.200000	no	no	no	no	no	no	no	yes	no	no
182175111	chr1	10853052	10853140	45	89	1.977778	no	no	no	no	no	no	no	yes	no	no
182175117	chr1	10853433	10853687	111	255	2.303571	no	no	no	no	no	no	no	yes	no	no
182175128	chr1	10866304	10866354	12	51	4.250000	no	no	no	no	no	no	no	yes	no	no
182175132	chr1	10873347	10873462	29	116	4.000000	no	no	no	no	no	no	no	yes	yes	no
182175158	chr1	10915900	10915926	14	27	1.928571	no	no	no	no	no	no	no	no	no	no
182175164	chr1	10920135	10920195	31	61	2.000000	no	no	no	no	no	no	no	no	yes	no

182175178	chr1	10936183	10936236	20	54	2.700000	no	no	no	no	no	no	no	yes	no	no
182175188	chr1	10969306	10969330	10	25	2.500000	no	no	yes	no	no	no	no	yes	no	yes
182175195	chr1	10981455	10981486	14	32	2.285714	no	no	yes	no	no	no	no	no	no	yes
182175197	chr1	10981484	10981556	38	73	2.026316	no	no	yes	no	no	no	no	no	no	yes
182175218	chr1	11007858	11007883	8	26	3.250000	no	no	no	no	no	no	no	no	no	no
182175245	chr1	11040535	11040721	81	187	2.308642	no	no	yes	no	no	no	no	yes	no	yes
182175252	chr1	11059894	11059926	16	33	2.125000	yes	no	no	yes	no	no	yes	yes	no	yes
182175273	chr1	11097785	11097832	25	48	1.920000	no	no	yes	no	no	no	no	no	no	yes
182175274	chr1	11099940	11100067	20	128	6.400000	yes	no	yes	no	no	no	yes	yes	yes	yes
182175282	chr1	11112625	11112858	78	234	3.012820	no	no	yes	yes	yes	no	no	no	no	yes
182175291	chr1	11128831	11128869	20	39	1.950000	no	no	yes	yes	no	no	no	no	no	yes
182175292	chr1	11130616	11130667	21	52	2.476191	no	no	no	yes	no	yes	no	no	no	yes
182175311	chr1	11173911	11173938	13	28	2.153846	no	no	yes	no	no	no	no	yes	no	yes
182175312	chr1	11182382	11182416	18	35	1.944444	no	no	yes	no	no	no	no	yes	yes	yes
182175324	chr1	11221697	11221750	23	54	2.347826	no	no	yes	no	no	no	no	no	no	yes
182175331	chr1	11246273	11246300	13	28	2.153846	no	no	yes	no	no	no	no	yes	yes	yes
182175350	chr1	11273046	11273095	26	50	1.923077	yes	no	no	no	no	no	no	yes	yes	no
182175378	chr1	11329075	11329177	52	103	1.980769	no	no	no	no	no	no	no	yes	no	no
182175383	chr1	11336984	11337020	15	37	2.437500	no	no	no	no	no	no	no	yes	yes	no
182175384	chr1	11337135	11337176	23	42	1.913043	no	no	no	no	no	no	no	yes	yes	no
182175396	chr1	11338533	11338563	14	31	2.214286	no	no	no	no	no	no	no	yes	no	no
182175402	chr1	11343035	11343063	15	29	2.000000	no	no	no	no	no	no	no	yes	no	no
182175437	chr1	11413804	11413967	42	164	4.071429	no	no	no	no	no	no	no	yes	yes	no
182175452	chr1	11440095	11440162	27	68	2.518518	no	no	no	no	no	no	no	no	yes	no
182175456	chr1	11441856	11441906	19	51	2.684211	no	no	no	no	no	no	no	yes	no	no

182175471	chr1	11481427	11481461	17	35	2.117647	no	yes	yes	no	no	no	no	yes	no	yes
182175472	chr1	11487334	11487371	19	38	2.000000	no	no	yes	no	no	no	no	yes	no	yes
182175475	chr1	11494329	11494357	14	29	2.071429	no	no	yes	no	no	no	no	yes	no	yes
182175481	chr1	11522524	11522999	11	476	43.272728	no	no	yes	no	no	no	no	no	yes	yes
182175482	chr1	11523419	11523692	30	274	9.161290	no	no	yes	no	no	no	no	yes	no	yes
182175486	chr1	11524662	11524970	41	309	7.658536	no	no	yes	no	no	no	no	no	no	yes
182175491	chr1	11528640	11528796	72	157	2.180556	no	no	yes	no	no	no	no	yes	yes	yes
182175504	chr1	11561841	11561984	42	144	3.488372	no	no	no	no	no	no	no	yes	no	no
182175518	chr1	11593399	11593436	20	38	1.950000	no	no	no	no	no	no	no	yes	no	no
182175524	chr1	11618257	11618307	20	51	2.650000	no	no	no	no	no	no	no	no	no	no
182175535	chr1	11641683	11641760	37	78	2.108108	no	yes	yes	no	no	no	no	yes	no	yes
182175536	chr1	11642646	11642687	21	42	2.000000	no	yes	yes	no	no	no	no	yes	yes	yes
182175547	chr1	11654823	11654875	20	53	2.650000	yes	no	yes	no	yes	no	yes	yes	yes	yes
182175561	chr1	11682757	11682796	20	40	2.000000	no	no	yes	no	no	no	no	yes	yes	yes
182175564	chr1	11684574	11684611	20	38	1.950000	no	no	yes	no	no	no	no	yes	yes	yes
182175568	chr1	11691468	11691492	13	25	1.923077	yes	no	no	no	yes	no	yes	yes	yes	yes
182175574	chr1	11702163	11702278	57	116	2.105263	no	no	yes	no	no	no	no	yes	yes	yes
182175585	chr1	11728966	11729006	15	41	2.733333	no	no	no	no	no	no	no	no	no	no
182175589	chr1	11730247	11730400	61	154	2.500000	no	no	no	no	no	no	no	yes	yes	no
182175613	chr1	11770200	11770238	19	39	2.105263	no	no	yes	no	no	no	no	no	yes	yes
182175619	chr1	11791452	11791499	22	48	2.181818	no	no	yes	no	no	no	no	yes	yes	yes
182175622	chr1	11806286	11806320	18	35	2.000000	yes	yes	no	yes	yes	no	yes	yes	no	yes
182175628	chr1	11829526	11829813	32	288	9.000000	yes	no	yes	no	no	yes	no	yes	no	yes
182175640	chr1	11839092	11839132	19	41	2.157895	no	no	yes	no	no	yes	no	yes	no	yes
182175642	chr1	11847729	11847758	11	30	2.727273	no	yes	yes	no	yes	yes	no	no	no	yes

182175653	chr1	11869524	11869559	12	36	3.000000	no	no	no	no	no	no	no	no	no	no
182175654	chr1	11869976	11870016	10	41	4.300000	no	no	no	no	no	no	no	no	no	no
182175656	chr1	11874604	11874648	25	45	1.920000	no	no	no	no	no	no	no	no	yes	no
182175667	chr1	11895601	11895636	18	36	2.000000	no	no	no	no	no	no	no	yes	no	no
182175677	chr1	11899756	11899788	14	33	2.357143	no	no	no	no	no	no	no	no	no	no
182175685	chr1	11909549	11909600	10	52	5.200000	no	no	yes	no	no	yes	no	yes	no	yes
182175706	chr1	11931413	11931454	19	42	2.210526	no	no	no	no	no	no	no	yes	yes	no
182175720	chr1	11944756	11944799	19	44	2.315789	no	no	yes	no	no	no	no	yes	yes	yes
182175721	chr1	11944788	11944827	15	40	2.666667	no	no	yes	no	no	no	no	yes	yes	yes
182175722	chr1	11945479	11945524	16	46	2.875000	no	no	yes	no	no	no	no	no	no	yes
182175723	chr1	11945511	11945548	12	38	3.166667	no	no	yes	no	no	no	no	no	no	yes
182175735	chr1	11960830	11960871	23	42	1.913043	no	no	yes	no	no	no	no	yes	yes	yes
182175741	chr1	11966024	11966061	20	38	1.900000	no	no	yes	no	no	no	no	yes	yes	yes
182175743	chr1	11966396	11966546	43	151	3.558140	no	yes	yes	no	no	yes	no	yes	no	yes
182175751	chr1	11971080	11971157	42	78	1.928571	yes	no	yes	no	no	no	no	yes	no	yes
182175753	chr1	11971200	11971240	19	41	2.210526	yes	no	yes	no	no	no	no	no	no	yes
182175762	chr1	11972575	11972665	46	91	2.043478	no	yes	yes	no	no	no	no	yes	no	yes
182175764	chr1	11980231	11980266	15	36	2.400000	yes	no	no	no	yes	no	yes	yes	yes	yes
182175765	chr1	11981088	11981153	32	66	2.062500	yes	no	yes	no	no	no	no	yes	no	yes
182175775	chr1	12007301	12007329	13	29	2.230769	no	no	yes	no	no	no	no	yes	no	yes
182175789	chr1	12031766	12031797	15	32	2.133333	no	yes	no	yes	no	no	no	yes	yes	yes
182175800	chr1	12046862	12046900	14	39	2.687500	no	no	no	no	no	no	no	yes	yes	no
182175801	chr1	12046896	12046937	14	42	3.000000	no	no	no	no	no	no	no	yes	no	no
182175813	chr1	12053856	12053936	32	81	2.531250	no	no	no	no	no	no	no	yes	yes	no
182175820	chr1	12073525	12073583	18	59	3.388889	no	no	yes	no	no	no	no	yes	no	yes

182175827	chr1	12076698	12076734	19	37	1.947368	no	no	yes	no	no	no	no	yes	yes	yes
182175828	chr1	12079904	12079939	17	36	2.117647	no	no	yes	no	no	yes	no	yes	no	yes
182175829	chr1	12081945	12082057	26	113	4.346154	no	no	yes	no	no	no	no	yes	yes	yes
182175833	chr1	12086443	12086514	32	72	2.242424	no	no	yes	no	no	no	no	no	no	yes
182175853	chr1	12128114	12128142	14	29	2.071429	no	no	yes	no	no	no	no	no	no	yes
182175857	chr1	12134522	12134562	22	41	1.909091	no	no	yes	no	no	no	no	yes	yes	yes
182175862	chr1	12144947	12145021	38	75	1.973684	no	no	no	no	no	yes	no	yes	no	no
182175869	chr1	12166729	12166770	15	42	2.800000	yes	no	no	no	no	no	no	yes	yes	no
182175873	chr1	12172922	12172954	18	33	1.944444	no	no	yes	no	no	no	no	yes	no	yes
182175877	chr1	12184405	12184517	58	113	1.982759	no	no	yes	no	no	no	no	yes	yes	yes
182175880	chr1	12192228	12192295	28	68	2.428571	no	no	yes	no	no	yes	no	yes	yes	yes
182175888	chr1	12230662	12230828	85	167	1.964706	no	no	yes	no	no	no	no	yes	yes	yes
182175894	chr1	12238230	12238287	31	58	1.935484	no	no	yes	no	no	no	no	no	no	yes
182175895	chr1	12239850	12239961	56	112	2.000000	no	no	yes	no	no	no	no	no	yes	yes
182175896	chr1	12242260	12242437	46	178	4.043478	no	no	yes	no	no	no	no	no	no	yes
182175899	chr1	12247492	12247522	11	31	2.818182	no	no	yes	no	no	no	no	no	no	yes
182175907	chr1	12263526	12263618	45	93	2.066667	no	no	yes	no	no	no	no	no	no	yes
182175911	chr1	12273224	12273253	15	30	2.000000	no	no	yes	no	no	no	no	no	no	yes
182175919	chr1	12299507	12299683	88	177	2.011364	no	no	yes	no	no	no	no	no	no	yes
182175921	chr1	12310769	12310937	8	169	21.125000	yes	no	yes	no	no	no	no	yes	no	yes
182175924	chr1	12321101	12321134	15	34	2.266667	no	no	yes	no	no	no	no	yes	no	yes
182175926	chr1	12325980	12326015	14	36	2.571429	no	no	yes	no	no	no	no	no	no	yes
182175927	chr1	12326990	12327049	32	60	1.906250	no	no	yes	no	no	no	no	no	yes	yes
182175928	chr1	12334329	12334363	16	35	2.187500	no	no	yes	no	no	no	no	no	no	yes
182175942	chr1	12380199	12380277	29	79	2.724138	no	no	yes	no	no	no	no	yes	no	yes

182175952	chr1	12416437	12416464	14	28	2.000000	no	no	yes	no	no	no	no	no	yes	yes
182175953	chr1	12424556	12424612	30	57	1.900000	no	no	yes	no	no	no	no	no	no	yes
182175956	chr1	12432577	12432613	18	37	2.055556	no	no	yes	no	no	no	no	no	no	yes
182175957	chr1	12433259	12433297	16	39	2.437500	no	no	yes	no	no	no	no	yes	no	yes
182175964	chr1	12446532	12446580	18	49	2.722222	no	no	yes	no	no	no	no	yes	no	yes
182175969	chr1	12460601	12460690	48	90	1.937500	no	no	yes	no	no	no	no	yes	no	yes
182175970	chr1	12463275	12463304	15	30	2.000000	no	no	yes	no	no	no	no	yes	no	yes
182175974	chr1	12490553	12490883	41	331	8.073171	no	no	yes	no	no	no	no	yes	yes	yes
182175976	chr1	12504936	12504962	7	27	3.857143	no	no	yes	no	no	no	no	no	yes	yes
182175982	chr1	12519277	12519310	17	34	2.058824	no	no	no	no	no	no	no	yes	no	no
182175983	chr1	12526137	12526180	11	44	4.000000	no	no	no	no	no	no	no	yes	no	no
182175986	chr1	12529709	12529737	15	29	1.933333	no	no	no	no	no	no	no	yes	no	no
182175998	chr1	12552387	12552530	74	144	1.945946	no	no	no	no	no	no	no	yes	yes	no
182176001	chr1	12571105	12571137	18	33	1.944444	no	no	yes	no	no	no	no	yes	no	yes
182176005	chr1	12583117	12583178	26	62	2.384615	no	no	yes	no	no	no	no	yes	no	yes
182176011	chr1	12586963	12587032	23	70	3.043478	no	no	yes	no	no	no	no	yes	yes	yes
182176031	chr1	12609660	12609697	21	38	1.904762	no	no	yes	no	no	no	no	yes	no	yes
182176033	chr1	12614758	12614835	36	78	2.166667	no	no	yes	no	no	no	no	yes	yes	yes
182176034	chr1	12615135	12615212	25	78	3.120000	no	no	yes	no	no	no	no	yes	yes	yes
182176037	chr1	12623167	12623212	20	46	2.350000	no	no	no	no	no	no	no	yes	no	no
182176041	chr1	12628088	12628122	13	35	2.769231	no	no	no	no	no	no	no	no	no	no
182176058	chr1	12658208	12658247	22	40	1.909091	no	no	yes	no	no	no	no	yes	yes	yes
182176060	chr1	12658640	12658802	42	163	3.930233	no	no	yes	no	no	no	no	yes	yes	yes
182176070	chr1	12688135	12688203	25	69	2.760000	no	no	no	no	no	no	no	no	no	no
182176078	chr1	12715202	12715279	37	78	2.108108	yes	no	no	no	no	no	no	yes	yes	no

182176080	chr1	12717171	12717212	21	42	2.000000	no	no	yes	no	no	no	no	no	no	yes
182176082	chr1	12722991	12723022	12	32	2.750000	no	no	yes	no	no	no	no	no	no	yes
182176091	chr1	12762157	12762583	213	427	2.004695	no	yes	no	no	no	yes	no	yes	no	yes
182176106	chr1	12809514	12809542	12	29	2.416667	no	no	no	no	no	no	no	no	no	no
182176160	chr1	12915541	12915674	20	134	6.700000	yes	no	no	no	no	no	no	no	no	no
182176201	chr1	13073162	13073214	21	53	2.523809	no	no	yes	no	no	no	no	no	no	yes
182176210	chr1	13090578	13090619	18	42	2.333333	no	no	no	no	no	no	no	no	no	no
182176264	chr1	13221310	13221785	237	476	2.037815	no	no	no	no	no	yes	no	no	no	no
182176306	chr1	13283514	13283570	29	57	2.034483	no	no	yes	no	no	no	no	no	no	yes
182176319	chr1	13308960	13309026	21	67	3.238095	no	no	no	no	no	yes	no	no	no	no
182176359	chr1	13344750	13344789	11	40	3.538461	yes	no	yes	no	no	no	no	no	no	yes
182176364	chr1	13370267	13370379	58	113	1.948276	no	no	yes	no	no	no	no	no	no	yes
182176381	chr1	13399817	13399846	13	30	2.307692	no	no	no	no	no	no	no	no	no	no
182176388	chr1	13412022	13412082	26	61	2.346154	no	no	yes	no	no	no	no	no	no	yes
182176389	chr1	13412636	13412693	30	58	1.933333	no	no	yes	no	no	no	no	no	no	yes
182176411	chr1	13433516	13433569	18	54	3.111111	no	no	no	no	no	no	no	yes	no	no
182176446	chr1	13482138	13482184	21	47	2.333333	no	no	yes	no	no	no	no	no	no	yes
182176449	chr1	13483918	13483960	20	43	2.150000	no	no	yes	no	no	no	no	no	no	yes
182176460	chr1	13502856	13502895	19	40	2.105263	no	no	yes	no	no	no	no	no	no	yes
182176468	chr1	13513563	13513601	21	39	1.904762	no	no	yes	yes	yes	no	yes	yes	yes	yes
182176469	chr1	13513797	13513825	15	29	2.000000	no	no	yes	no	yes	no	yes	yes	no	yes
182176470	chr1	13516614	13516647	16	34	2.125000	no	no	no	no	no	yes	no	yes	no	no
182176472	chr1	13522114	13522305	32	192	6.000000	no	no	no	no	no	no	no	yes	yes	no
182176513	chr1	13578779	13578820	21	42	2.000000	no	no	no	no	no	no	no	no	no	no
182176515	chr1	13588568	13588686	47	119	2.510204	no	no	yes	no	no	no	no	no	no	yes

182176521	chr1	13601967	13602077	17	111	6.500000	no	no	yes	no	no	no	no	no	no	yes
182176522	chr1	13603071	13603108	20	38	1.900000	no	no	yes	no	no	no	no	no	no	yes
182176523	chr1	13605151	13605282	62	132	2.129032	no	no	yes	no	no	no	no	yes	yes	yes
182176528	chr1	13612087	13612127	21	41	1.952381	no	no	yes	no	no	no	no	yes	no	yes
182176536	chr1	13636079	13636176	38	98	2.564103	no	no	no	no	no	no	no	no	no	no
182176566	chr1	13683901	13683955	24	55	2.291667	no	no	no	no	no	no	no	no	no	no
182176571	chr1	13697114	13697603	260	490	1.900000	yes	no	no	no	no	no	no	yes	no	no
182176581	chr1	13773020	13773066	23	47	2.043478	no	no	yes	no	no	no	no	no	no	yes
182176582	chr1	13778376	13778407	14	32	2.357143	no	no	yes	no	no	no	no	yes	no	yes
182176586	chr1	13782229	13782269	21	41	1.952381	no	no	yes	yes	no	no	no	yes	yes	yes
182176591	chr1	13824001	13824042	19	42	2.263158	no	yes	no	no	no	yes	no	no	no	yes
182176610	chr1	13915109	13915150	16	42	2.625000	no	no	yes	no	no	no	no	yes	no	yes
182176611	chr1	13915882	13915914	18	33	1.944444	no	no	yes	no	no	no	no	no	no	yes
182176616	chr1	13935943	13935974	14	32	2.285714	no	no	yes	no	no	no	no	no	no	yes
182176620	chr1	13950081	13950109	14	29	2.142857	no	no	yes	no	no	no	no	no	no	yes
182176627	chr1	13960844	13960882	15	39	2.600000	no	no	yes	no	no	no	no	yes	no	yes
182176628	chr1	13961231	13961282	25	52	2.080000	no	no	yes	no	no	no	no	yes	no	yes
182176631	chr1	13966164	13966216	18	53	2.944444	no	no	yes	no	no	no	no	yes	no	yes
182176638	chr1	13981349	13981391	23	43	1.913043	no	no	yes	no	no	no	no	yes	no	yes
182176651	chr1	14013265	14013304	20	40	2.050000	no	no	yes	no	no	no	no	no	no	yes
182176657	chr1	14040036	14040091	16	56	3.500000	no	no	yes	no	no	no	no	yes	no	yes
182176670	chr1	14070356	14070380	13	25	1.923077	no	no	yes	no	no	no	no	no	no	yes
182176675	chr1	14081199	14081231	17	33	1.941176	no	no	yes	no	no	no	no	no	no	yes
182176683	chr1	14092532	14092625	40	94	2.350000	no	no	yes	no	no	no	no	no	no	yes
182176686	chr1	14102295	14102589	148	295	2.000000	no	no	yes	no	no	no	no	no	no	yes

182176694	chr1	14127782	14127810	13	29	2.230769	no	no	yes	no	no	no	no	no	no	yes
182176701	chr1	14148975	14149000	13	26	2.000000	no	no	yes	no	no	no	no	no	no	yes
182176708	chr1	14176424	14176449	9	26	2.888889	no	no	yes	no	no	no	no	yes	no	yes
182176709	chr1	14187819	14187851	17	33	2.000000	no	no	yes	no	no	no	no	no	no	yes
182176711	chr1	14195202	14195236	17	35	2.058824	no	no	yes	no	no	no	no	no	no	yes
182176714	chr1	14201010	14201047	17	38	2.235294	no	no	yes	no	no	no	no	no	no	yes
182176725	chr1	14250813	14250930	52	118	2.283019	no	no	yes	no	no	no	no	yes	no	yes
182176736	chr1	14258140	14258174	11	35	3.181818	no	no	yes	no	no	no	no	no	no	yes
182176738	chr1	14262390	14262426	15	37	2.437500	no	no	yes	no	no	no	no	no	no	yes
182176746	chr1	14295509	14295694	93	186	2.043011	no	no	yes	no	no	no	no	yes	yes	yes
182176751	chr1	14320328	14320360	15	33	2.266667	no	no	yes	no	no	no	no	no	no	yes
182176755	chr1	14334848	14334889	22	42	1.909091	no	no	yes	no	no	no	no	yes	yes	yes
182176759	chr1	14339089	14339128	18	40	2.222222	no	no	yes	no	no	no	no	no	no	yes
182176767	chr1	14369405	14369431	12	27	2.250000	no	no	yes	no	no	no	no	no	no	yes
182176770	chr1	14395155	14395211	26	57	2.192308	no	no	yes	no	no	no	no	no	no	yes
182176773	chr1	14402167	14402214	20	48	2.400000	no	no	yes	no	no	no	no	no	no	yes
182176779	chr1	14413123	14413174	22	52	2.347826	no	no	yes	no	no	no	no	yes	no	yes
182176784	chr1	14416947	14417011	20	65	3.350000	no	no	yes	no	no	no	no	no	no	yes
182176794	chr1	14435723	14435899	35	177	5.057143	no	no	yes	no	no	no	no	yes	yes	yes
182176811	chr1	14478173	14478230	28	58	2.071429	no	no	yes	no	no	no	no	no	no	yes
182176817	chr1	14480899	14480958	31	60	1.935484	no	no	yes	no	no	no	no	no	no	yes
182176839	chr1	14515956	14515980	11	25	2.272727	no	no	yes	no	no	no	no	yes	no	yes
182176852	chr1	14551967	14552010	18	44	2.444444	no	no	yes	no	no	no	no	yes	no	yes
182176855	chr1	14558917	14558971	25	55	2.240000	no	no	yes	no	no	no	no	no	no	yes
182176861	chr1	14572584	14572626	21	43	2.047619	no	no	yes	no	no	no	no	no	no	yes

182176866	chr1	14590689	14590729	22	41	1.954545	no	no	yes	no	no	no	no	yes	yes	yes
182176873	chr1	14598736	14598779	15	44	2.933333	no	yes	yes	no	yes	no	yes	yes	yes	yes
182176874	chr1	14604366	14604418	22	53	2.409091	no	no	yes	no	no	no	no	no	no	yes
182176880	chr1	14639237	14639261	13	25	1.923077	no	no	yes	no	no	no	no	no	no	yes
182176887	chr1	14655144	14655197	22	54	2.454545	no	no	yes	no	no	no	no	no	yes	yes
182176888	chr1	14657824	14657901	40	78	1.975000	no	no	yes	no	no	no	no	no	yes	yes
182176891	chr1	14662877	14662982	43	106	2.534884	no	no	yes	no	no	no	no	no	no	yes
182176894	chr1	14670114	14670153	17	40	2.352941	no	no	yes	no	no	no	no	yes	no	yes
182176896	chr1	14675230	14675295	34	66	2.000000	no	no	yes	no	no	no	no	no	no	yes
182176897	chr1	14678816	14678852	16	37	2.312500	no	no	yes	no	no	no	no	no	no	yes
182176903	chr1	14711386	14711425	21	40	1.904762	no	no	yes	no	no	no	no	no	no	yes
182176908	chr1	14716154	14716195	18	42	2.444444	no	no	yes	no	no	no	no	yes	no	yes
182176920	chr1	14753200	14753235	19	36	1.947368	no	no	yes	no	no	no	no	no	no	yes
182176924	chr1	14775623	14775686	32	64	2.000000	no	no	yes	no	yes	no	no	yes	yes	yes
182176930	chr1	14788756	14788821	23	66	3.000000	no	no	yes	no	no	no	no	yes	yes	yes
182176933	chr1	14793649	14793822	87	174	2.000000	no	no	yes	no	no	no	no	yes	yes	yes
182176945	chr1	14831155	14831211	24	57	2.360000	no	no	yes	no	no	no	no	no	no	yes
182176955	chr1	14844925	14845038	55	114	2.072727	no	no	yes	no	no	no	no	yes	no	yes
182176964	chr1	14868034	14868074	22	41	1.909091	no	no	yes	no	no	no	no	yes	no	yes
182176970	chr1	14874537	14874579	18	43	2.388889	no	no	yes	no	no	no	no	yes	no	yes
182176976	chr1	14888539	14888610	38	72	1.947368	no	no	yes	no	no	no	no	no	no	yes
182176977	chr1	14892369	14892409	19	41	2.210526	no	no	yes	no	no	no	no	no	no	yes
182176979	chr1	14894140	14894204	24	65	2.680000	no	no	yes	no	no	no	no	yes	no	yes
182176983	chr1	14899676	14899712	15	37	2.466667	no	no	yes	no	no	no	no	no	no	yes
182176996	chr1	14921394	14921430	15	37	2.466667	no	no	yes	no	no	no	no	yes	yes	yes

182176999	chr1	14932651	14932697	23	47	2.086957	no	no	yes	no	no	no	no	no	no	yes
182177002	chr1	14936310	14936347	19	38	2.000000	no	no	yes	no	no	no	no	no	no	yes
182177003	chr1	14937072	14937133	31	62	2.000000	no	no	yes	no	no	no	no	no	no	yes
182177004	chr1	14939605	14939649	19	45	2.421053	no	no	yes	no	no	no	no	yes	no	yes
182177008	chr1	14947480	14947519	18	40	2.222222	no	no	yes	no	no	no	no	yes	yes	yes
182177014	chr1	14954331	14954388	30	58	1.933333	no	no	yes	no	no	no	no	yes	yes	yes
182177016	chr1	14958303	14958499	80	197	2.462500	no	no	yes	no	no	no	no	yes	no	yes
182177019	chr1	14977330	14977368	20	39	2.000000	no	no	yes	no	no	no	no	yes	no	yes
182177021	chr1	14982340	14982364	12	25	2.083333	no	no	yes	no	no	no	no	no	no	yes
182177028	chr1	15014733	15014835	51	103	2.019608	no	no	yes	no	no	no	no	yes	yes	yes
182177038	chr1	15036234	15036427	103	194	1.883495	no	no	yes	no	no	no	no	no	no	yes
182177039	chr1	15036569	15036618	20	50	2.500000	no	no	yes	no	no	no	no	no	no	yes
182177040	chr1	15038410	15039163	55	754	13.836364	no	no	yes	no	no	no	no	yes	yes	yes
182177047	chr1	15048540	15048911	14	372	26.571428	no	no	yes	no	no	no	yes	yes	no	yes
182177051	chr1	15050828	15050869	14	42	3.000000	no	no	yes	no	no	no	no	no	no	yes
182177056	chr1	15059865	15059906	22	42	1.954545	no	no	yes	no	no	no	no	no	no	yes
182177057	chr1	15063691	15063913	55	223	4.054545	no	no	yes	no	no	no	no	no	no	yes
182177062	chr1	15083676	15083708	12	33	2.750000	no	no	yes	no	no	no	no	yes	yes	yes
182177063	chr1	15089212	15089251	21	40	1.952381	no	no	yes	no	no	no	no	yes	no	yes
182177065	chr1	15091110	15091148	20	39	1.950000	no	no	yes	no	no	no	no	no	no	yes
182177071	chr1	15093215	15093243	15	29	1.933333	no	no	yes	no	no	no	no	yes	no	yes
182177076	chr1	15101847	15101902	28	56	2.071429	no	no	yes	no	no	no	no	no	yes	yes
182177079	chr1	15103503	15103578	35	76	2.200000	no	no	yes	no	no	no	no	yes	no	yes
182177093	chr1	15168089	15168117	13	29	2.230769	no	no	yes	no	no	yes	no	no	no	yes
182177100	chr1	15184000	15184055	25	56	2.320000	no	no	yes	no	no	no	no	no	no	yes

182177108	chr1	15197542	15197597	26	56	2.153846	no	no	yes	no	no	no	no	yes	no	yes
182177111	chr1	15201398	15201439	22	42	1.909091	no	no	yes	no	no	no	no	no	no	yes
182177114	chr1	15206261	15206428	91	168	1.901099	no	no	yes	no	no	no	no	yes	no	yes
182177151	chr1	15284905	15284956	23	52	2.347826	no	no	yes	no	no	no	no	yes	yes	yes
182177164	chr1	15299539	15299573	17	35	2.058824	no	no	yes	no	no	no	no	no	no	yes
182177176	chr1	15321800	15321846	14	47	3.357143	no	no	yes	no	no	no	no	yes	no	yes
182177180	chr1	15328559	15328583	13	25	1.923077	no	yes	yes	no	yes	yes	no	no	yes	yes
182177182	chr1	15331359	15331520	60	162	2.700000	no	no	yes	no	no	no	no	no	no	yes
182177184	chr1	15331637	15331770	12	134	11.166667	no	no	yes	no	no	no	no	no	no	yes
182177185	chr1	15333315	15333359	16	45	2.812500	no	no	yes	no	no	yes	no	yes	no	yes
182177196	chr1	15362163	15362214	26	52	2.000000	yes	no	yes	no	no	no	no	yes	no	yes
182177198	chr1	15362813	15362971	82	159	1.939024	no	no	yes	no	no	no	no	yes	yes	yes
182177199	chr1	15364994	15365025	15	32	2.133333	no	no	yes	no	no	yes	no	yes	yes	yes
182177205	chr1	15377353	15377469	56	117	2.089286	no	no	yes	no	no	no	no	yes	yes	yes
182177206	chr1	15383350	15383388	16	39	2.437500	yes	yes	yes	no	no	no	no	no	no	yes
182177207	chr1	15383962	15384008	18	47	2.611111	no	yes	yes	no	no	no	no	yes	yes	yes
182177220	chr1	15405940	15405971	7	32	4.571429	no	no	yes	no	yes	no	no	no	yes	yes
182177222	chr1	15419891	15419924	17	34	2.000000	no	no	yes	no	no	no	no	yes	no	yes
182177225	chr1	15426515	15426557	22	43	1.954545	no	no	yes	no	no	no	no	no	yes	yes
182177235	chr1	15441605	15441650	23	46	2.043478	no	no	yes	no	no	no	no	yes	no	yes
182177241	chr1	15447486	15447627	71	142	2.000000	no	yes	no	no	no	yes	no	yes	no	yes
182177244	chr1	15448887	15448921	18	35	1.944444	no	yes	no	no	no	no	no	yes	no	yes
182177256	chr1	15489226	15489264	16	39	2.437500	no	no	yes	no	no	no	no	yes	yes	yes
182177259	chr1	15495022	15495063	21	42	2.047619	no	no	yes	no	no	yes	no	no	no	yes
182177264	chr1	15501010	15501043	17	34	2.000000	no	no	yes	no	no	no	no	no	yes	yes

182177267	chr1	15501374	15501440	23	67	3.000000	no	no	yes	no	no	no	no	yes	no	yes
182177269	chr1	15504899	15504943	23	45	2.000000	no	no	yes	no	no	no	no	no	no	yes
182177276	chr1	15524257	15524304	18	48	2.631579	yes	no	yes	no	yes	no	yes	yes	yes	yes
182177280	chr1	15524599	15524896	159	298	1.911950	yes	no	yes	no	yes	no	yes	yes	yes	yes
182177287	chr1	15546337	15546390	26	54	2.076923	no	no	yes	no	no	no	no	no	no	yes
182177297	chr1	15561856	15561903	16	48	2.941176	no	no	yes	no	no	no	no	no	no	yes
182177337	chr1	15631196	15631220	10	25	2.500000	no	no	yes	no	no	no	no	yes	no	yes
182177355	chr1	15676342	15676426	25	85	3.366667	no	no	no	no	no	no	no	yes	no	no
182177364	chr1	15683498	15683535	18	38	2.166667	yes	no	no	no	no	no	no	yes	yes	no
182177367	chr1	15683838	15684092	137	255	1.861314	yes	no	no	no	no	no	yes	yes	no	no
182177371	chr1	15684503	15684556	12	54	4.583333	yes	yes	no	no	yes	no	yes	yes	no	yes
182177372	chr1	15685541	15685590	21	50	2.428571	no	no	yes	no	no	no	no	yes	yes	yes
182177377	chr1	15693354	15693391	19	38	2.000000	no	no	yes	no	no	no	no	no	yes	yes
182177379	chr1	15697587	15697626	17	40	2.352941	no	no	yes	no	no	no	no	yes	no	yes
182177395	chr1	15718667	15718691	11	25	2.272727	no	no	yes	no	no	no	no	yes	no	yes
182177401	chr1	15732228	15732257	14	30	2.142857	no	no	yes	no	no	no	no	yes	yes	yes
182177403	chr1	15739292	15739331	21	40	1.904762	no	yes	no	yes	no	yes	no	yes	yes	yes
182177404	chr1	15741075	15741112	20	38	1.900000	no	yes	yes	no	no	no	no	yes	no	yes
182177405	chr1	15744092	15744123	17	32	1.941176	no	no	yes	no	no	no	no	yes	no	yes
182177409	chr1	15750375	15750414	21	40	1.904762	yes	no	no	no	no	no	no	yes	no	no
182177421	chr1	15765445	15765488	22	44	2.000000	no	no	yes	no	no	yes	no	yes	yes	yes
182177437	chr1	15789590	15789671	30	82	2.733333	no	no	no	no	no	no	no	no	yes	no
182177441	chr1	15789906	15789938	12	33	2.750000	no	no	no	no	no	no	no	yes	no	no
182177449	chr1	15799873	15799922	16	50	3.125000	no	no	no	no	no	no	no	yes	no	no
182177467	chr1	15837295	15837361	33	67	2.060606	no	no	yes	no	no	no	yes	yes	yes	yes

182177474	chr1	15842788	15842835	24	48	2.041667	no	no	yes	no	no	no	no	no	no	yes
182177476	chr1	15843095	15843136	23	42	1.913043	no	no	yes	no	no	no	no	no	no	yes
182177492	chr1	15860391	15860494	52	104	2.000000	no	no	yes	no	no	no	no	yes	yes	yes
182177494	chr1	15866279	15866311	14	33	2.357143	no	no	yes	no	no	no	no	yes	no	yes
182177495	chr1	15868156	15868191	8	36	4.500000	no	no	yes	no	no	no	no	no	no	yes
182177497	chr1	15875604	15875635	14	32	2.285714	yes	no	yes	no	no	no	no	no	no	yes
182177518	chr1	15904745	15904792	25	48	1.920000	no	no	yes	no	no	no	no	yes	no	yes
182177525	chr1	15926423	15926466	16	44	2.705882	no	no	yes	no	no	no	no	no	no	yes
182177526	chr1	15934179	15934229	21	51	2.476191	no	no	no	yes	no	no	no	yes	yes	yes
182177534	chr1	15945138	15945172	9	35	3.888889	yes	no	no	yes	yes	yes	no	yes	no	yes
182177536	chr1	15947521	15947642	64	122	1.937500	yes	no	yes	no	no	no	no	yes	no	yes
182177544	chr1	15961838	15961866	13	29	2.230769	no	no	yes	no	no	no	no	no	no	yes
182177547	chr1	15971351	15971603	19	253	13.285714	no	no	yes	no	no	no	no	yes	no	yes
182177561	chr1	15993408	15993472	31	65	2.096774	no	yes	no	no	no	no	no	no	no	yes
182177563	chr1	16009485	16009533	18	49	2.684211	no	no	no	no	no	yes	no	yes	no	no
182177564	chr1	16017596	16017639	18	44	2.444444	no	no	yes	no	yes	no	no	yes	yes	yes
182177567	chr1	16021848	16021886	15	39	2.600000	yes	no	yes	no	yes	yes	no	yes	yes	yes
182177583	chr1	16074301	16074521	120	221	1.883333	yes	no	no	no	no	no	no	yes	yes	no
182177584	chr1	16076202	16076240	20	39	1.950000	no	no	no	no	no	no	no	yes	yes	no
182177590	chr1	16081929	16081962	10	34	3.400000	no	no	no	no	no	no	no	no	yes	no
182177592	chr1	16083603	16083645	10	43	4.300000	no	no	no	no	no	no	no	yes	yes	no
182177605	chr1	16098643	16098674	16	32	2.000000	no	no	no	no	no	no	no	no	no	no
182177606	chr1	16098988	16099024	15	37	2.600000	no	no	no	no	no	no	no	no	no	no
182177620	chr1	16143425	16143449	11	25	2.272727	no	no	yes	no	no	no	no	yes	no	yes
182177621	chr1	16147247	16147294	23	48	2.086957	yes	no	yes	no	no	no	no	yes	yes	yes

182177623	chr1	16157910	16157983	33	74	2.393939	no	no	no	no	no	yes	no	yes	no	no
182177628	chr1	16165360	16165400	8	41	5.125000	no	no	no	no	no	no	no	yes	no	no
182177633	chr1	16166382	16166427	24	46	1.916667	no	no	no	no	no	no	no	yes	yes	no
182177639	chr1	16179194	16179233	18	40	2.277778	no	no	no	no	no	no	no	yes	yes	no
182177661	chr1	16202319	16202386	20	68	3.400000	no	no	yes	no	no	no	no	yes	yes	yes
182177670	chr1	16216364	16216392	15	29	1.933333	no	no	yes	no	no	yes	no	yes	yes	yes
182177676	chr1	16221523	16221897	202	375	1.886139	no	no	yes	no	no	no	no	yes	no	yes
182177682	chr1	16225209	16225382	57	174	3.052632	no	no	no	no	yes	no	no	no	no	yes
182177690	chr1	16264599	16264631	16	33	2.062500	no	no	yes	no	no	no	no	no	no	yes
182177700	chr1	16277035	16277066	17	32	1.941176	no	no	yes	no	no	no	no	no	no	yes
182177703	chr1	16278730	16278770	14	41	2.866667	no	no	yes	no	no	no	no	no	yes	yes
182177729	chr1	16325493	16325529	20	37	1.950000	no	no	yes	no	no	no	no	no	yes	yes
182177750	chr1	16355701	16355736	13	36	2.769231	no	no	yes	no	no	no	no	no	no	yes
182177764	chr1	16371394	16371427	14	34	2.428571	no	no	yes	no	no	no	no	yes	no	yes
182177772	chr1	16383977	16384015	19	39	2.052632	no	no	yes	no	no	no	no	no	no	yes
182177778	chr1	16397803	16397838	15	36	2.400000	no	yes	yes	no	no	yes	no	yes	yes	yes
182177807	chr1	16440538	16440585	20	48	2.400000	yes	no	no	no	no	no	yes	yes	yes	no
182177812	chr1	16452760	16452796	18	37	2.166667	no	no	yes	no	no	yes	no	no	yes	yes
182177841	chr1	16498904	16498956	18	53	3.000000	no	no	yes	no	no	no	yes	yes	no	yes
182177843	chr1	16499332	16499374	17	43	2.529412	yes	no	no	no	no	no	yes	yes	yes	no
182177865	chr1	16557728	16557876	12	149	12.500000	no	no	no	no	no	no	no	no	no	no
182177919	chr1	16692434	16692475	13	42	3.307692	yes	no	no	no	yes	no	yes	no	no	yes
182177921	chr1	16693152	16693187	16	36	2.250000	yes	no	no	no	yes	no	yes	no	no	yes
182177922	chr1	16694002	16694055	21	54	2.571429	no	no	yes	no	yes	no	yes	no	no	yes
182177957	chr1	16790158	16790309	78	152	1.987180	no	no	yes	no	no	no	no	no	no	yes

182177982	chr1	16921727	16921759	17	33	2.000000	yes	no	yes	no	no	no	no	yes	yes	yes
182177988	chr1	16939366	16939412	22	47	2.136364	no	no	yes	no	no	no	no	no	no	yes
182177994	chr1	16946498	16946552	18	55	3.055556	yes	no	yes	no	no	yes	no	no	no	yes
182178000	chr1	16957750	16957789	19	40	2.157895	no	no	yes	no	no	no	no	no	no	yes
182178016	chr1	16975400	16975478	24	79	3.291667	yes	no	yes	no	no	yes	no	yes	yes	yes
182178019	chr1	16985804	16985834	13	31	2.384615	no	no	yes	no	no	yes	no	no	no	yes
182178020	chr1	16986943	16987011	36	69	1.972222	no	no	yes	yes	yes	no	no	yes	yes	yes
182178029	chr1	16997850	16997891	17	42	2.529412	no	no	yes	no	no	no	no	no	no	yes
182178036	chr1	17011731	17011773	20	43	2.250000	no	no	no	yes	yes	no	yes	yes	no	yes
182178048	chr1	17036674	17036799	32	126	3.937500	no	no	yes	no	no	no	no	no	no	yes
182178060	chr1	17071591	17071618	14	28	2.000000	no	no	yes	no	no	no	no	no	no	yes
182178061	chr1	17079613	17079684	12	72	6.000000	no	no	yes	no	no	no	no	yes	yes	yes
182178095	chr1	17163550	17163604	27	55	2.074074	no	no	no	no	no	no	no	no	no	no
182178100	chr1	17166192	17166245	28	54	1.928571	no	no	no	no	no	no	no	yes	no	no
182178103	chr1	17181865	17181908	23	44	1.956522	no	no	no	no	no	no	no	yes	yes	no
182178108	chr1	17194556	17194617	31	62	2.032258	no	yes	yes	no	no	no	no	no	yes	yes
182178116	chr1	17208374	17208699	37	326	8.891891	no	no	yes	no	no	no	no	yes	yes	yes
182178121	chr1	17217006	17217080	27	75	2.777778	no	no	yes	no	no	no	no	no	no	yes
182178122	chr1	17223354	17223390	18	37	2.111111	yes	no	yes	no	no	no	no	no	no	yes
182178125	chr1	17229207	17229240	10	34	3.400000	no	no	yes	no	no	no	no	yes	no	yes
182178130	chr1	17235568	17235695	60	128	2.150000	no	no	yes	no	no	no	no	yes	yes	yes
182178134	chr1	17238945	17238976	16	32	2.000000	yes	no	yes	no	no	no	no	no	no	yes
182178135	chr1	17239557	17239588	16	32	2.062500	yes	no	yes	no	no	no	no	yes	yes	yes
182178136	chr1	17241452	17241986	36	535	14.861111	no	no	yes	no	no	no	no	yes	yes	yes
182178137	chr1	17242822	17242850	12	29	2.416667	no	no	yes	no	no	no	no	no	no	yes

182178139	chr1	17246150	17246181	12	32	2.666667	no	no	no	no	no	yes	no	yes	yes	no
182178141	chr1	17250724	17250775	23	52	2.260870	no	no	yes	no	no	no	no	yes	yes	yes
182178146	chr1	17266722	17266754	17	33	1.941176	no	no	no	yes	no	no	no	no	no	yes
182178152	chr1	17279236	17279264	13	29	2.230769	no	no	yes	no	no	no	no	yes	yes	yes
182178162	chr1	17293292	17293346	28	55	2.035714	no	no	no	no	no	no	no	yes	no	no
182178165	chr1	17299080	17299118	20	39	1.950000	no	no	no	no	no	no	no	no	no	no
182178188	chr1	17329068	17329108	12	41	3.500000	no	no	yes	no	no	no	no	yes	no	yes
182178190	chr1	17335201	17335236	18	36	2.055556	no	no	yes	no	no	yes	no	yes	no	yes
182178196	chr1	17343206	17343253	22	48	2.227273	no	no	yes	no	no	no	no	no	no	yes
182178202	chr1	17351952	17352066	27	115	4.259259	no	no	yes	no	no	no	no	yes	no	yes
182178216	chr1	17381658	17381683	12	26	2.166667	no	no	yes	no	no	no	no	no	no	yes
182178219	chr1	17397649	17397706	29	58	2.000000	no	no	yes	no	no	no	no	yes	yes	yes
182178220	chr1	17398296	17398389	48	94	1.979167	no	no	yes	no	no	no	no	yes	no	yes
182178221	chr1	17401874	17401923	20	50	2.550000	no	no	no	no	no	yes	no	no	yes	no
182178280	chr1	17508922	17508968	25	47	1.920000	no	no	no	no	no	no	no	no	no	no
182178316	chr1	17557692	17557732	21	41	2.000000	no	no	yes	no	no	no	no	no	no	yes
182178334	chr1	17624280	17624384	52	105	2.019231	no	no	yes	no	no	no	no	yes	yes	yes
182178335	chr1	17627519	17627552	14	34	2.428571	no	no	yes	no	no	no	no	yes	no	yes
182178337	chr1	17630859	17631176	105	318	3.028037	no	no	yes	no	no	no	no	yes	yes	yes
182178340	chr1	17634576	17634617	8	42	5.250000	no	no	yes	no	no	no	no	yes	no	yes
182178342	chr1	17639543	17639584	23	42	1.913043	yes	no	yes	no	no	no	no	yes	yes	yes
182178344	chr1	17641622	17641671	20	50	2.500000	no	no	yes	no	no	no	no	no	no	yes
182178346	chr1	17648453	17648491	20	39	2.000000	no	no	yes	no	no	no	no	yes	no	yes
182178349	chr1	17666682	17666881	65	200	3.090909	no	no	yes	no	no	no	no	no	no	yes
182178351	chr1	17674534	17674565	13	32	2.461539	no	no	yes	no	no	no	no	yes	no	yes

182178359	chr1	17683073	17683458	28	386	13.785714	no	no	yes	no	no	no	no	yes	no	yes
182178365	chr1	17702624	17702662	21	39	1.904762	no	no	no	no	no	no	no	no	no	no
182178376	chr1	17717263	17717378	39	116	2.974359	yes	no	no	no	no	no	no	yes	no	no
182178381	chr1	17725958	17726019	31	62	2.000000	no	no	yes	no	no	no	no	yes	no	yes
182178385	chr1	17727791	17727848	28	58	2.071429	no	no	yes	no	no	no	no	no	no	yes
182178391	chr1	17743259	17743299	15	41	2.733333	no	no	yes	no	no	no	no	no	yes	yes
182178394	chr1	17746582	17746636	25	55	2.240000	no	no	yes	no	no	no	no	no	no	yes
182178395	chr1	17748843	17748885	19	43	2.368421	no	no	yes	no	no	no	no	yes	no	yes
182178396	chr1	17748871	17749071	98	201	2.081633	no	no	yes	no	no	no	no	yes	no	yes
182178402	chr1	17757609	17757650	21	42	2.142857	no	no	yes	no	no	no	no	no	yes	yes
182178403	chr1	17759615	17759650	18	36	2.000000	no	no	yes	no	no	no	no	yes	no	yes
182178404	chr1	17763836	17763877	21	42	2.000000	no	no	yes	no	no	no	no	yes	no	yes
182178406	chr1	17779184	17779233	17	50	3.000000	no	no	yes	no	no	no	no	no	no	yes
182178409	chr1	17786511	17786540	12	30	2.500000	no	no	yes	no	no	no	no	no	no	yes
182178430	chr1	17854040	17854085	16	46	2.937500	no	no	no	no	no	no	no	no	no	no
182178450	chr1	17884799	17884839	22	41	1.909091	no	no	no	no	no	no	no	yes	no	no
182178455	chr1	17885873	17885932	24	60	2.500000	no	no	no	no	no	no	no	yes	no	no
182178456	chr1	17887587	17887615	11	29	2.636364	no	no	no	no	no	no	no	no	no	no
182178466	chr1	17933894	17933961	33	68	2.060606	no	no	no	no	no	no	no	yes	no	no
182178467	chr1	17935587	17935615	15	29	1.933333	no	no	no	no	no	no	no	yes	yes	no
182178469	chr1	17935701	17935755	12	55	4.583333	no	no	no	no	no	no	no	no	no	no
182178470	chr1	17935753	17935789	19	37	1.947368	no	no	no	no	no	no	no	no	no	no
182178472	chr1	17935773	17935830	28	58	2.142857	no	no	no	no	no	no	no	no	no	no
182178473	chr1	17935825	17935910	24	86	3.583333	no	no	no	no	no	no	no	no	no	no
182178488	chr1	17950382	17950417	15	36	2.400000	no	no	no	no	no	no	no	no	no	no

182178492	chr1	17966862	17966900	21	39	2.000000	no	no	no	no	no	no	no	yes	no	no
182178496	chr1	17970819	17970848	15	30	2.000000	no	no	no	no	no	no	no	yes	yes	no
182178499	chr1	17980816	17980967	55	152	2.763636	no	no	no	no	no	no	no	no	no	no
182178523	chr1	18035715	18035756	9	42	4.666667	no	no	yes	no	no	no	no	no	no	yes
182178526	chr1	18039542	18039592	24	51	2.125000	no	no	yes	no	no	no	no	no	no	yes
182178527	chr1	18041273	18041342	35	70	2.000000	no	no	yes	no	no	no	no	yes	yes	yes
182178534	chr1	18059101	18059135	18	35	1.944444	no	no	no	no	no	no	no	no	no	no
182178539	chr1	18068208	18068264	22	57	2.590909	no	yes	no	no	no	no	no	no	no	yes
182178542	chr1	18083104	18083148	17	45	2.578947	no	no	no	no	no	no	no	no	no	no
182178544	chr1	18087818	18087931	57	114	2.000000	no	no	no	no	no	no	no	no	no	no
182178548	chr1	18094439	18094473	17	35	2.117647	no	no	no	no	no	no	no	no	no	no
182178557	chr1	18097691	18097743	27	53	2.037037	no	no	no	no	no	no	no	no	no	no
182178558	chr1	18098303	18098339	19	37	2.000000	no	no	no	no	no	no	no	no	no	no
182178564	chr1	18101755	18101795	20	41	2.050000	no	no	no	no	no	no	no	no	no	no
182178565	chr1	18103672	18103721	14	50	3.571429	no	no	no	no	no	no	no	yes	no	no
182178569	chr1	18103764	18103966	34	203	6.264706	no	no	no	no	no	no	no	yes	yes	no
182178576	chr1	18103996	18104154	78	159	2.141026	no	no	no	no	no	no	no	yes	no	no
182178579	chr1	18104234	18104301	20	68	3.400000	no	no	no	no	no	no	no	yes	no	no
182178586	chr1	18108072	18108115	9	44	5.000000	no	no	no	no	yes	no	yes	yes	no	yes
182178588	chr1	18110979	18111003	8	25	3.125000	no	yes	yes	no	no	no	yes	yes	no	yes
182178593	chr1	18118874	18118932	29	59	2.034483	no	no	yes	no	no	no	no	no	no	yes
182178597	chr1	18119110	18119165	24	56	2.333333	no	no	yes	no	no	no	no	yes	no	yes
182178598	chr1	18121744	18121780	19	37	1.947368	no	no	yes	no	no	no	no	no	no	yes
182178602	chr1	18129313	18129349	19	37	1.947368	no	no	yes	no	no	no	no	yes	no	yes
182178604	chr1	18132228	18132259	7	32	4.571429	no	no	yes	no	no	no	no	yes	no	yes

182178605	chr1	18132292	18132318	13	27	2.076923	no	no	yes	no	no	no	no	yes	yes	yes
182178613	chr1	18154814	18154850	11	37	3.454545	no	no	yes	no	no	no	no	yes	no	yes
182178614	chr1	18156283	18156349	34	67	1.970588	no	no	yes	no	no	no	no	no	no	yes
182178616	chr1	18164372	18164404	15	33	2.200000	no	no	yes	no	no	no	no	no	no	yes
182178617	chr1	18171301	18171331	14	31	2.214286	no	no	yes	no	no	no	no	no	yes	yes
182178621	chr1	18205097	18205128	16	32	2.062500	no	no	yes	no	no	no	no	no	yes	yes
182178629	chr1	18242164	18242229	33	66	2.000000	no	no	yes	no	no	no	no	yes	yes	yes
182178640	chr1	18281124	18281213	28	90	3.357143	no	no	yes	no	no	no	no	yes	no	yes
182178650	chr1	18292141	18292170	15	30	2.000000	no	no	yes	no	no	no	no	no	no	yes
182178657	chr1	18315703	18315910	89	208	2.347826	no	no	yes	no	no	no	no	yes	no	yes
182178660	chr1	18330496	18330694	48	199	4.145833	no	no	yes	no	no	no	no	no	no	yes
182178668	chr1	18349622	18349657	15	36	2.400000	no	no	yes	no	no	no	no	no	no	yes
182178669	chr1	18350938	18350977	19	40	2.157895	no	no	yes	no	no	no	no	yes	no	yes
182178674	chr1	18357118	18357164	23	47	2.043478	no	no	yes	no	no	no	no	yes	no	yes
182178677	chr1	18357350	18357393	23	44	1.913043	no	no	yes	no	no	no	no	yes	no	yes
182178693	chr1	18377685	18377771	46	87	1.934783	no	no	yes	no	no	yes	no	no	no	yes
182178694	chr1	18383909	18383955	22	47	2.181818	no	no	no	no	no	no	no	no	no	no
182178706	chr1	18400125	18400154	15	30	2.000000	no	no	no	no	no	no	no	no	yes	no
182178709	chr1	18413598	18413630	10	33	3.400000	no	no	no	no	no	no	no	no	no	no
182178715	chr1	18439352	18439390	18	39	2.277778	no	no	no	no	no	no	no	no	no	no
182178734	chr1	18472392	18472505	35	114	3.216216	no	no	no	no	no	no	no	yes	no	no
182178735	chr1	18472517	18472570	27	54	2.000000	no	no	no	no	no	no	no	yes	no	no
182178737	chr1	18474462	18474524	12	63	5.416667	no	no	no	no	no	no	no	no	no	no
182178741	chr1	18474637	18474672	18	36	2.000000	no	no	no	no	no	no	no	no	no	no
182178765	chr1	18511475	18511528	25	54	2.160000	no	no	no	no	no	no	no	yes	no	no

182178771	chr1	18523599	18523686	45	88	1.977778	no	no	no	no	no	no	no	yes	no	no
182178788	chr1	18571118	18571150	14	33	2.357143	no	no	no	no	no	no	no	no	no	no
182178790	chr1	18572629	18572659	12	31	2.666667	no	no	no	no	no	no	no	yes	yes	no
182178793	chr1	18576629	18576660	15	32	2.133333	no	no	no	no	no	no	no	yes	no	no
182178794	chr1	18576773	18576810	12	38	3.250000	no	no	no	no	no	no	no	no	no	no
182178812	chr1	18597954	18598310	70	357	5.040541	no	no	no	no	no	no	no	yes	no	no
182178817	chr1	18602441	18602799	83	359	4.380952	no	no	no	no	no	no	no	yes	yes	no
182178818	chr1	18604721	18605102	19	382	20.157894	no	no	no	no	no	no	no	no	no	no
182178822	chr1	18617149	18617186	19	38	2.052632	no	no	no	no	no	no	no	no	no	no
182178834	chr1	18626153	18626279	69	127	1.927536	no	no	no	no	no	no	no	no	no	no
182178837	chr1	18626647	18626766	49	120	2.448980	no	no	no	no	no	no	no	no	no	no
182178840	chr1	18631154	18631183	8	30	3.750000	yes	no	no	no	yes	no	yes	yes	yes	yes
182178841	chr1	18634432	18634465	18	34	1.944444	no	no	no	yes	no	no	no	yes	yes	yes
182178847	chr1	18638062	18638098	18	37	2.055556	no	no	yes	no	no	no	no	yes	no	yes
182178848	chr1	18640893	18640937	16	45	2.875000	no	no	yes	no	no	no	yes	yes	no	yes
182178849	chr1	18642129	18642169	17	41	2.470588	no	no	yes	no	no	no	no	yes	no	yes
182178850	chr1	18646868	18646906	14	39	2.733333	no	no	yes	no	no	no	yes	yes	yes	yes
182178853	chr1	18653859	18653896	19	38	2.000000	no	no	yes	no	no	no	no	yes	no	yes
182178855	chr1	18656550	18656602	22	53	2.409091	no	no	yes	no	no	no	no	no	no	yes
182178862	chr1	18662417	18662457	8	41	5.125000	no	no	yes	no	no	no	no	yes	no	yes
182178874	chr1	18685745	18685783	20	39	2.000000	no	no	yes	no	no	no	no	no	no	yes
182178877	chr1	18689403	18689454	26	52	2.000000	no	no	yes	no	no	no	no	no	no	yes
182178885	chr1	18701309	18701455	48	147	3.104167	no	no	yes	no	no	no	no	yes	no	yes
182178893	chr1	18707820	18707860	20	41	2.050000	no	no	yes	no	no	no	no	no	yes	yes
182178894	chr1	18712127	18712176	17	50	2.941176	no	no	yes	no	no	no	no	yes	no	yes

182178897	chr1	18720569	18720668	33	100	3.030303	no	no	yes	no	no	no	no	yes	no	yes
182178898	chr1	18721082	18721124	23	43	1.956522	no	no	yes	no	no	no	no	no	no	yes
182178899	chr1	18723181	18723205	9	25	2.777778	no	no	yes	no	no	no	no	yes	yes	yes
182178900	chr1	18725910	18725950	20	41	2.050000	no	no	yes	no	no	no	no	yes	yes	yes
182178903	chr1	18726497	18726556	21	60	2.857143	no	no	yes	no	no	no	no	yes	yes	yes
182178905	chr1	18727057	18727089	17	33	1.941176	no	no	yes	no	no	no	no	yes	no	yes
182178908	chr1	18727289	18727341	21	53	2.523809	no	no	yes	no	no	no	no	yes	no	yes
182178910	chr1	18728324	18728425	51	102	2.000000	no	no	yes	no	no	no	no	yes	no	yes
182178911	chr1	18734387	18734500	55	114	2.072727	no	no	yes	no	no	no	no	no	no	yes
182178914	chr1	18737368	18737404	17	37	2.176471	no	no	yes	no	no	no	no	no	no	yes
182178920	chr1	18751099	18751127	15	29	1.933333	no	no	no	no	no	no	no	no	yes	no
182178923	chr1	18771206	18771262	27	57	2.111111	no	no	no	no	no	no	no	yes	no	no
182178945	chr1	18801427	18802212	397	786	2.010076	no	no	no	no	no	no	no	yes	no	no
182178950	chr1	18802354	18802410	28	57	2.178571	no	no	no	no	no	no	no	yes	no	no
182178952	chr1	18802436	18802477	22	42	1.954545	no	no	no	no	no	no	no	yes	no	no
182178956	chr1	18818410	18818468	21	59	2.857143	no	no	no	no	no	no	no	yes	no	no
182178960	chr1	18825226	18825831	266	606	2.277153	no	no	no	no	no	no	no	yes	no	no
182178963	chr1	18826032	18826084	27	53	1.962963	no	no	no	no	no	no	no	yes	no	no
182178976	chr1	18857938	18857972	9	35	3.888889	no	no	yes	no	no	no	no	yes	no	yes
182178989	chr1	18865249	18865282	10	34	3.272727	no	no	no	no	no	no	no	yes	no	no
182178993	chr1	18895340	18895683	70	344	4.957143	no	no	yes	no	no	no	no	yes	yes	yes
182179006	chr1	18951581	18951646	23	66	2.869565	no	no	yes	no	no	no	no	yes	yes	yes
182179008	chr1	18956234	18956290	28	57	2.107143	yes	no	no	yes	no	no	yes	yes	no	yes
182179010	chr1	18957880	18957908	14	29	2.142857	no	no	no	no	no	no	no	yes	yes	no
182179035	chr1	19008345	19008388	17	44	2.588235	no	no	no	no	no	no	no	no	yes	no

182179037	chr1	19011632	19011702	26	71	2.730769	no	no	no	no	no	no	no	yes	yes	no
182179043	chr1	19029621	19029661	7	41	5.857143	no	no	no	no	no	no	no	yes	yes	no
182179045	chr1	19030183	19030255	23	73	3.166667	no	no	no	no	no	no	no	yes	yes	no
182179053	chr1	19052573	19052705	22	133	6.045455	no	no	no	no	no	no	no	yes	yes	no
182179062	chr1	19072381	19072546	28	166	6.035714	no	no	no	no	yes	no	no	yes	yes	yes
182179065	chr1	19096627	19096652	12	26	2.166667	yes	no	yes	yes	yes	no	no	yes	no	yes
182179066	chr1	19098922	19098957	19	36	1.947368	no	no	yes	no	no	no	no	yes	no	yes
182179068	chr1	19117998	19118030	11	33	3.090909	no	no	yes	no	yes	no	no	yes	no	yes
182179080	chr1	19138266	19138305	20	40	2.000000	no	no	yes	no	no	no	no	yes	no	yes
182179081	chr1	19138935	19138976	20	42	2.100000	no	no	yes	no	no	no	no	no	no	yes
182179085	chr1	19143456	19143492	7	37	5.285714	no	no	yes	no	no	no	no	no	no	yes
182179089	chr1	19169878	19169906	15	29	1.933333	no	no	yes	no	no	no	no	yes	yes	yes
182179090	chr1	19178341	19178393	27	53	2.000000	yes	no	yes	no	no	no	no	no	no	yes
182179094	chr1	19193244	19193277	17	34	2.000000	no	no	yes	no	no	no	no	yes	no	yes
182179097	chr1	19198733	19198759	14	27	1.928571	no	no	yes	no	no	no	no	yes	yes	yes
182179099	chr1	19198932	19198970	20	39	1.950000	no	no	yes	no	no	no	no	yes	yes	yes
182179101	chr1	19234870	19234924	27	55	2.074074	no	no	yes	no	no	no	no	no	no	yes
182179106	chr1	19238395	19238426	15	32	2.133333	no	no	yes	no	no	no	no	yes	no	yes
182179107	chr1	19241869	19241902	14	34	2.428571	no	no	yes	no	no	no	no	yes	yes	yes
182179111	chr1	19254891	19254932	19	42	2.210526	no	no	yes	no	no	no	no	no	no	yes
182179121	chr1	19288269	19288306	16	38	2.500000	no	no	yes	no	no	no	no	yes	no	yes
182179137	chr1	19313280	19313346	36	67	1.916667	no	no	yes	no	no	no	no	yes	yes	yes
182179138	chr1	19314282	19314507	119	226	1.915966	no	no	yes	no	no	no	no	yes	yes	yes
182179142	chr1	19324565	19324597	17	33	1.941176	yes	no	yes	no	no	no	no	yes	yes	yes
182179149	chr1	19337707	19337870	82	164	2.000000	no	no	no	no	no	yes	no	yes	yes	no

182179150	chr1	19343836	19343953	59	118	2.033898	no	no	yes	no	no	no	no	yes	yes	yes
182179154	chr1	19358445	19358503	29	59	2.034483	no	no	yes	no	no	no	no	no	no	yes
182179158	chr1	19367870	19367902	13	33	2.538461	no	no	yes	no	no	no	no	yes	yes	yes
182179159	chr1	19371305	19371353	20	49	2.450000	no	no	yes	no	no	no	no	no	no	yes
182179160	chr1	19378385	19378462	31	78	2.516129	no	no	yes	no	no	no	no	no	no	yes
182179171	chr1	19429054	19429091	20	38	1.950000	no	no	yes	no	no	no	no	no	no	yes
182179172	chr1	19430711	19430739	12	29	2.416667	no	no	yes	no	no	no	no	yes	yes	yes
182179178	chr1	19449794	19449842	22	49	2.227273	no	no	yes	no	no	no	no	no	no	yes
182179184	chr1	19458260	19458331	34	72	2.147059	no	no	yes	no	no	no	no	no	no	yes
182179187	chr1	19468556	19468599	15	44	2.933333	no	no	yes	no	no	no	no	yes	yes	yes
182179194	chr1	19486007	19486032	10	26	2.600000	yes	no	yes	no	no	no	yes	yes	yes	yes
182179211	chr1	19514812	19514889	39	78	2.000000	no	no	yes	no	no	no	no	yes	no	yes
182179217	chr1	19527095	19527125	15	31	2.133333	no	no	yes	no	no	no	no	yes	no	yes
182179225	chr1	19528080	19528111	15	32	2.133333	no	no	yes	no	no	no	no	no	no	yes
182179258	chr1	19604595	19604630	12	36	3.083333	no	no	yes	no	no	no	no	no	yes	yes
182179264	chr1	19614384	19614426	16	43	2.812500	no	no	yes	no	no	no	no	no	no	yes
182179272	chr1	19636340	19636364	10	25	2.500000	no	no	yes	no	no	no	no	no	no	yes
182179280	chr1	19647851	19647915	24	65	2.708333	no	no	yes	no	no	no	no	yes	yes	yes
182179282	chr1	19650772	19650816	18	45	2.500000	no	no	yes	no	no	no	no	yes	yes	yes
182179288	chr1	19657026	19657057	12	32	2.666667	no	no	no	yes	no	yes	no	yes	yes	yes
182179311	chr1	19698702	19698738	16	37	2.312500	no	no	yes	no	no	no	no	yes	no	yes
182179337	chr1	19734345	19734396	22	52	2.363636	no	no	yes	no	no	no	no	yes	yes	yes
182179341	chr1	19743605	19743659	19	55	2.894737	no	no	yes	no	no	no	no	yes	no	yes
182179347	chr1	19745030	19745136	49	107	2.224490	no	no	yes	no	no	no	no	no	no	yes
182179356	chr1	19758410	19758448	20	39	1.950000	no	no	yes	no	no	no	no	yes	yes	yes

182179364	chr1	19787676	19787719	14	44	3.214286	no	no	yes	no	no	no	no	yes	no	yes
182179366	chr1	19791309	19791344	18	36	2.000000	no	no	yes	no	no	no	no	no	no	yes
182179389	chr1	19845216	19845252	17	37	2.352941	no	no	no	no	no	no	no	yes	no	no
182179396	chr1	19855050	19855102	25	53	2.200000	no	no	no	no	no	no	no	no	no	no
182179403	chr1	19861885	19861923	21	39	1.904762	no	no	no	no	no	no	no	yes	no	no
182179409	chr1	19874065	19874173	15	109	7.266667	no	no	no	no	no	no	no	no	no	no
182179413	chr1	19882723	19882775	22	53	2.500000	no	no	yes	yes	no	no	yes	yes	yes	yes
182179418	chr1	19904208	19904240	17	33	1.941176	no	no	yes	no	no	no	no	no	no	yes
182179424	chr1	19926423	19926514	42	92	2.190476	no	no	no	no	no	no	no	yes	no	no
182179476	chr1	20075220	20075252	11	33	3.000000	no	no	yes	no	no	no	no	no	no	yes
182179491	chr1	20134107	20134217	59	111	1.932203	no	no	no	no	no	no	no	no	no	no
182179502	chr1	20148743	20148780	16	38	2.375000	no	yes	no	no	no	no	no	no	no	yes
182179503	chr1	20149256	20149443	94	188	2.000000	no	yes	no	no	no	no	no	yes	yes	yes
182179512	chr1	20165756	20165827	36	72	2.027778	no	no	yes	no	no	no	no	yes	yes	yes
182179523	chr1	20179403	20179506	53	104	2.000000	no	no	no	no	no	no	no	yes	no	no
182179528	chr1	20179854	20179897	14	44	3.066667	no	no	no	no	no	no	no	yes	yes	no
182179529	chr1	20179883	20179960	41	78	1.902439	no	no	no	no	no	no	no	yes	yes	no
182179531	chr1	20184103	20184144	22	42	1.909091	no	no	no	no	no	yes	no	yes	yes	no
182179533	chr1	20185997	20186029	10	33	3.300000	yes	no	yes	no	no	no	yes	yes	yes	yes
182179538	chr1	20205810	20205835	13	26	2.000000	no	no	no	no	no	no	no	no	no	no
182179540	chr1	20213266	20213292	14	27	1.928571	no	no	no	no	no	no	no	yes	yes	no
182179547	chr1	20254911	20255330	30	420	14.064516	no	no	no	no	no	no	no	no	yes	no
182179553	chr1	20274651	20274684	17	34	2.000000	no	no	yes	no	no	no	no	yes	no	yes
182179562	chr1	20292096	20292153	27	58	2.259259	no	no	yes	no	no	no	no	yes	no	yes
182179588	chr1	20362990	20363056	37	67	1.918919	no	no	yes	no	no	no	no	no	no	yes

182179591	chr1	20366407	20366519	60	113	1.916667	no	no	yes	no	no	no	yes	yes	yes	yes
182179597	chr1	20382492	20382540	18	49	2.722222	no	no	yes	no	no	no	no	no	no	yes
182179602	chr1	20409878	20409939	20	62	3.000000	no	no	yes	no	no	no	no	yes	no	yes
182179618	chr1	20443843	20443874	13	32	2.461539	no	no	no	no	no	no	no	no	no	no
182179627	chr1	20454816	20454856	18	41	2.277778	no	no	no	no	no	no	no	yes	no	no
182179642	chr1	20483396	20483441	18	46	2.611111	no	yes	no	no	yes	no	no	yes	no	yes
182179644	chr1	20485834	20485862	15	29	1.933333	no	no	no	no	yes	no	yes	yes	yes	yes
182179646	chr1	20485946	20485977	14	32	2.285714	no	no	no	no	yes	no	yes	yes	yes	yes
182179649	chr1	20486239	20486270	11	32	2.909091	yes	no	no	no	no	no	yes	yes	yes	no
182179674	chr1	20553764	20553804	18	41	2.277778	no	no	no	yes	no	no	yes	yes	yes	yes
182179675	chr1	20555712	20555755	23	44	1.913043	no	no	no	no	no	yes	no	yes	yes	no
182179685	chr1	20573265	20573353	24	89	3.653846	no	no	no	no	no	no	no	no	no	no
182179690	chr1	20574055	20574160	38	106	2.794872	no	no	no	no	no	no	no	yes	no	no
182179699	chr1	20586020	20586044	11	25	2.272727	no	no	no	no	no	no	no	no	no	no
182179703	chr1	20590426	20590475	26	50	1.923077	no	no	yes	no	no	no	no	yes	yes	yes
182179707	chr1	20597877	20597907	16	31	1.937500	no	no	yes	no	no	no	no	no	yes	yes
182179719	chr1	20623369	20623467	47	99	2.106383	no	no	no	no	no	no	no	yes	no	no
182179740	chr1	20670678	20670731	27	54	2.000000	no	no	yes	no	no	no	no	no	yes	yes
182179746	chr1	20682111	20682212	29	102	3.517241	no	no	yes	no	no	no	no	no	yes	yes
182179751	chr1	20693801	20693839	10	39	3.818182	no	no	yes	no	no	no	no	yes	yes	yes
182179766	chr1	20704141	20704168	12	28	2.333333	no	no	yes	no	no	no	no	no	yes	yes
182179772	chr1	20710866	20710901	18	36	2.000000	no	no	yes	no	no	no	no	yes	yes	yes
182179778	chr1	20713855	20713904	21	50	2.380952	no	no	yes	no	no	no	no	yes	no	yes
182179785	chr1	20721503	20721557	22	55	2.500000	no	yes	no	no	yes	no	no	no	no	yes
182179786	chr1	20722896	20722927	15	32	2.266667	no	no	yes	yes	yes	no	no	no	no	yes

182179789	chr1	20746024	20746067	23	44	1.913043	no	no	yes	no	no	no	no	no	no	yes
182179803	chr1	20779183	20779227	21	45	2.190476	no	no	yes	no	no	yes	no	yes	no	yes
182179816	chr1	20803795	20803828	18	34	1.944444	no	no	no	no	no	no	no	yes	no	no
182179817	chr1	20805819	20805843	12	25	2.083333	no	no	no	no	no	yes	no	no	no	no
182179818	chr1	20805932	20805979	23	48	2.086957	no	no	no	no	no	yes	no	no	no	no
182179823	chr1	20811852	20811883	14	32	2.357143	no	no	yes	no	no	no	no	no	no	yes
182179854	chr1	20821591	20821825	43	235	5.500000	no	no	yes	no	no	no	no	yes	no	yes
182179859	chr1	20841858	20841946	43	89	2.116279	no	no	yes	no	no	yes	no	yes	no	yes
182179865	chr1	20857648	20857700	19	53	2.789474	no	no	yes	no	no	no	no	no	no	yes
182179875	chr1	20886132	20886172	22	41	1.909091	no	no	yes	no	no	no	no	yes	no	yes
182179882	chr1	20897751	20897790	21	40	1.904762	no	no	yes	no	no	no	no	no	no	yes
182179884	chr1	20903827	20903895	34	69	2.058824	no	no	yes	no	no	no	no	yes	yes	yes
182179885	chr1	20905970	20906010	19	41	2.157895	no	no	yes	no	no	no	no	no	no	yes
182179889	chr1	20910787	20910822	17	36	2.117647	no	no	yes	no	no	no	no	no	no	yes
182179896	chr1	20938558	20938622	34	65	1.911765	no	no	yes	no	no	no	no	no	no	yes
182179898	chr1	20941712	20941744	9	33	3.666667	yes	no	yes	yes	no	no	no	no	no	yes
182179908	chr1	20956152	20956182	15	31	2.066667	no	no	yes	no	no	no	no	no	no	yes
182179916	chr1	20964364	20964407	21	44	2.095238	no	no	yes	no	no	no	no	no	no	yes
182179920	chr1	20977053	20977097	24	45	1.916667	no	no	yes	no	no	no	no	no	no	yes
182179927	chr1	20996981	20997028	23	48	2.086957	no	no	yes	no	no	no	no	no	no	yes
182179929	chr1	20998668	20998779	56	112	2.000000	no	no	yes	no	no	no	no	yes	no	yes
182179930	chr1	20999672	20999700	13	29	2.230769	no	no	yes	no	no	no	no	yes	yes	yes
182179932	chr1	21009123	21009155	14	33	2.428571	no	no	yes	no	no	no	no	no	no	yes
182179947	chr1	21032783	21032812	13	30	2.307692	no	no	yes	no	no	no	no	no	no	yes
182179952	chr1	21040637	21040677	21	41	2.047619	no	no	yes	no	no	no	no	no	no	yes

182179972	chr1	21062554	21062597	18	44	2.444444	no	no	yes	no	no	no	no	yes	no	yes
182179974	chr1	21064171	21064246	38	76	2.000000	no	no	yes	no	no	yes	no	yes	yes	yes
182179976	chr1	21066295	21066339	18	45	2.500000	no	no	yes	no	no	no	no	yes	no	yes
182180012	chr1	21143309	21143394	45	86	1.955556	no	no	yes	no	no	no	no	yes	no	yes
182180019	chr1	21153224	21153259	14	36	2.533333	no	no	yes	no	no	no	no	no	yes	yes
182180020	chr1	21154749	21154792	16	44	2.750000	no	no	yes	no	no	no	no	no	yes	yes
182180024	chr1	21158977	21159014	20	38	1.900000	no	no	yes	no	no	no	no	no	no	yes
182180031	chr1	21176098	21176132	18	35	2.000000	yes	no	yes	no	yes	no	no	yes	yes	yes
182180043	chr1	21189894	21189984	29	91	3.137931	no	no	no	no	no	no	no	yes	yes	no
182180045	chr1	21190346	21190376	16	31	1.937500	no	no	no	no	no	no	no	no	no	no
182180046	chr1	21190885	21191052	17	168	9.882353	no	no	no	no	no	no	no	yes	yes	no
182180057	chr1	21206879	21206924	22	46	2.090909	no	no	no	no	no	no	no	no	no	no
182180070	chr1	21266140	21266267	36	128	3.555556	no	no	yes	no	no	no	no	yes	yes	yes
182180076	chr1	21282780	21282830	22	51	2.363636	no	no	yes	no	no	no	no	no	no	yes
182180084	chr1	21301004	21301087	41	84	2.048780	no	no	yes	no	no	no	no	yes	yes	yes
182180088	chr1	21307821	21307875	22	55	2.500000	no	no	yes	no	no	no	no	yes	yes	yes
182180170	chr1	21425039	21425085	20	47	2.350000	no	no	yes	no	no	no	no	no	no	yes
182180172	chr1	21426588	21426632	15	45	3.000000	no	no	yes	no	no	no	no	no	no	yes
182180175	chr1	21432690	21432725	16	36	2.250000	no	no	no	no	no	no	no	no	no	no
182180200	chr1	21479861	21483571	1559	3711	2.400510	no	yes	yes	yes	no	yes	no	no	no	yes
182180222	chr1	21509288	21509363	40	76	1.900000	yes	no	no	no	no	no	yes	yes	yes	no
182180223	chr1	21510093	21510126	15	34	2.400000	no	no	yes	no	no	no	no	yes	yes	yes
182180228	chr1	21516209	21516236	10	28	2.800000	no	no	yes	no	no	no	no	no	no	yes
182180235	chr1	21532518	21532555	17	38	2.235294	no	no	yes	no	no	no	no	no	no	yes
182180237	chr1	21538746	21538773	14	28	2.000000	no	no	yes	no	no	no	no	yes	no	yes

182180238	chr1	21540273	21540303	16	31	1.937500	no	no	yes	no	no	no	no	yes	no	yes
182180244	chr1	21552532	21552571	19	40	2.157895	no	no	yes	no	no	no	no	yes	no	yes
182180246	chr1	21554358	21554405	18	48	2.631579	no	no	yes	no	no	no	no	no	no	yes
182180247	chr1	21554398	21554476	15	79	5.400000	no	no	yes	no	no	no	no	no	no	yes
182180253	chr1	21560809	21560839	15	31	2.066667	no	no	yes	no	no	no	no	no	no	yes
182180258	chr1	21568790	21568860	31	71	2.290323	no	no	yes	no	no	no	no	yes	no	yes
182180262	chr1	21572700	21572737	17	38	2.235294	no	no	yes	no	no	no	no	yes	no	yes
182180266	chr1	21576754	21576791	19	38	2.000000	no	no	yes	no	no	no	no	no	no	yes
182180267	chr1	21578008	21578057	22	50	2.272727	no	yes	no	no	no	yes	no	no	no	yes
182180279	chr1	21610311	21610347	19	37	2.000000	no	no	yes	no	no	no	no	yes	no	yes
182180281	chr1	21622467	21622510	23	44	2.000000	no	no	yes	no	yes	yes	yes	yes	yes	yes
182180287	chr1	21636393	21636425	13	33	2.571429	no	no	yes	no	no	no	no	yes	no	yes
182180299	chr1	21651409	21651446	14	38	2.714286	no	no	yes	no	yes	no	no	yes	yes	yes
182180300	chr1	21651741	21651775	18	35	2.000000	no	no	yes	no	yes	no	no	yes	yes	yes
182180304	chr1	21653583	21653682	52	100	1.923077	yes	no	yes	no	no	no	no	yes	yes	yes
182180311	chr1	21662577	21662609	16	33	2.062500	no	no	yes	no	no	no	no	yes	no	yes
182180312	chr1	21668271	21668322	17	52	3.176471	yes	no	yes	no	no	no	no	yes	no	yes
182180320	chr1	21696818	21696846	14	29	2.071429	no	no	yes	no	no	no	no	no	no	yes
182180323	chr1	21707385	21707414	14	30	2.142857	no	no	yes	no	no	no	no	yes	yes	yes
182180335	chr1	21723672	21723703	13	32	2.461539	no	no	yes	no	no	yes	no	no	no	yes
182180336	chr1	21727350	21727386	19	37	1.947368	yes	no	yes	no	no	yes	no	no	no	yes
182180337	chr1	21734820	21734847	14	28	2.000000	no	no	yes	no	no	no	no	yes	no	yes
182180347	chr1	21746053	21746158	52	106	2.038461	no	no	yes	no	no	no	no	no	no	yes
182180349	chr1	21755247	21755284	20	38	1.950000	no	no	yes	no	no	no	no	no	no	yes
182180356	chr1	21763821	21763861	12	41	3.416667	no	no	yes	no	no	no	no	yes	yes	yes

182180358	chr1	21768648	21768678	16	31	1.937500	no	no	yes	no	no	yes	no	yes	yes	yes
182180374	chr1	21781046	21781088	15	43	2.875000	no	no	yes	no	no	no	no	yes	no	yes
182180391	chr1	21801975	21802016	20	42	2.200000	no	no	no	no	no	no	no	no	no	no
182180400	chr1	21815095	21815122	10	28	2.800000	no	no	yes	no	no	no	no	yes	yes	yes
182180409	chr1	21822559	21822586	14	28	2.000000	no	yes	yes	no	yes	yes	no	yes	yes	yes
182180422	chr1	21872866	21872902	14	37	2.642857	yes	no	yes	yes	no	no	no	yes	yes	yes
182180428	chr1	21895932	21895964	15	33	2.200000	no	no	no	yes	yes	no	no	yes	no	yes
182180432	chr1	21905237	21905285	10	49	4.818182	no	no	yes	no	no	no	no	yes	no	yes
182180438	chr1	21937103	21937144	21	42	2.000000	no	no	yes	no	no	no	yes	yes	no	yes
182180445	chr1	21937346	21937425	36	80	2.243243	yes	no	no	no	no	no	yes	yes	yes	no
182180473	chr1	22005887	22005918	8	32	4.000000	no	yes	yes	no	no	no	no	yes	no	yes
182180505	chr1	22046270	22046318	24	49	2.208333	no	no	yes	no	no	no	no	no	no	yes
182180515	chr1	22068469	22068498	13	30	2.307692	no	no	yes	no	yes	no	no	no	yes	yes
182180527	chr1	22115798	22115827	15	30	2.000000	no	no	no	no	no	no	no	yes	yes	no
182180529	chr1	22125854	22125890	19	37	1.947368	no	no	yes	no	no	no	no	no	no	yes
182180530	chr1	22136619	22136643	8	25	3.125000	no	no	yes	no	no	no	no	no	no	yes
182180534	chr1	22141260	22141301	19	42	2.210526	no	no	yes	no	no	no	no	yes	no	yes
182180535	chr1	22142379	22142411	10	33	3.300000	yes	no	yes	no	no	no	no	yes	yes	yes
182180537	chr1	22142994	22143031	20	38	1.900000	no	no	yes	no	yes	no	yes	yes	yes	yes
182180550	chr1	22159701	22159726	12	26	2.166667	no	no	no	no	no	no	no	no	no	no
182180555	chr1	22166830	22166867	16	38	2.470588	no	no	no	no	no	no	no	yes	no	no
182180558	chr1	22172719	22172752	17	34	2.000000	no	no	no	no	no	no	no	yes	no	no
182180565	chr1	22185590	22185627	19	38	2.105263	no	no	no	no	no	no	no	yes	no	no
182180566	chr1	22188242	22188281	21	40	2.047619	no	no	no	no	no	no	no	no	no	no
182180570	chr1	22205736	22205778	20	43	2.150000	no	no	no	no	no	no	no	yes	no	no

182180577	chr1	22226682	22226737	23	56	2.478261	no	no	no	no	no	no	no	yes	no	no
182180584	chr1	22230763	22231530	202	768	3.855769	no	no	no	no	no	no	no	yes	no	no
182180597	chr1	22260298	22260365	32	68	2.125000	no	no	no	no	no	no	no	yes	no	no
182180601	chr1	22260971	22261161	23	191	8.250000	no	no	no	no	no	no	no	yes	no	no
182180604	chr1	22261539	22261577	21	39	1.952381	no	no	no	no	no	no	no	no	yes	no
182180605	chr1	22264207	22264237	15	31	2.066667	no	no	no	no	no	no	no	yes	no	no
182180607	chr1	22265390	22265455	34	66	1.941176	yes	no	no	no	no	no	no	yes	no	no
182180614	chr1	22272233	22272262	16	30	1.937500	no	no	no	no	no	no	no	yes	no	no
182180615	chr1	22272273	22272301	15	29	1.933333	no	no	no	no	no	no	no	yes	no	no
182180621	chr1	22279924	22280021	37	98	2.648649	no	no	no	no	no	no	no	yes	no	no
182180640	chr1	22330914	22330974	31	61	2.032258	no	no	no	no	no	no	no	yes	no	no
182180643	chr1	22333899	22334115	86	217	2.523256	no	no	no	no	no	no	no	yes	no	no
182180644	chr1	22336106	22336140	18	35	1.944444	no	no	no	no	no	no	no	yes	no	no
182180649	chr1	22342568	22342604	14	37	2.642857	no	no	no	no	no	no	no	yes	no	no
182180657	chr1	22350958	22350982	11	25	2.272727	no	no	no	no	no	no	no	yes	yes	no
182180664	chr1	22354354	22354397	20	44	2.250000	no	no	no	no	no	no	no	no	no	no
182180668	chr1	22366610	22366644	18	35	1.944444	yes	no	no	no	no	no	no	yes	no	no
182180682	chr1	22394329	22394379	19	51	2.684211	no	no	no	no	no	no	no	no	no	no
182180685	chr1	22399878	22399931	28	54	1.964286	no	no	no	no	no	no	no	yes	no	no
182180690	chr1	22402926	22402953	14	28	2.000000	no	no	no	no	no	no	no	yes	yes	no
182180697	chr1	22412917	22412958	12	42	3.500000	no	no	no	no	no	no	no	yes	no	no
182180709	chr1	22431160	22431199	20	40	2.000000	no	no	yes	no	no	no	no	yes	no	yes
182180717	chr1	22433638	22433676	20	39	1.950000	no	no	yes	no	no	no	no	no	no	yes
182180732	chr1	22508127	22508171	23	45	1.956522	no	no	yes	yes	no	no	no	yes	no	yes
182180733	chr1	22521796	22521826	16	31	1.937500	no	no	yes	no	no	no	no	yes	no	yes

182180735	chr1	22532042	22532105	30	64	2.133333	no	no	no	no	no	yes	no	yes	no	no
182180737	chr1	22535577	22535624	20	48	2.400000	no	no	no	no	no	no	no	yes	no	no
182180741	chr1	22557155	22557217	34	63	1.911765	no	no	no	no	no	no	no	yes	no	no
182180743	chr1	22566079	22566116	13	38	3.000000	no	no	yes	no	no	no	no	no	no	yes
182180744	chr1	22570249	22570301	20	53	2.650000	no	no	yes	no	no	no	no	yes	no	yes
182180747	chr1	22570288	22570366	38	79	2.131579	no	no	yes	no	no	no	no	yes	no	yes
182180754	chr1	22587256	22587294	20	39	1.950000	no	no	yes	no	no	no	no	yes	no	yes
182180756	chr1	22589073	22589101	15	29	1.933333	no	no	no	yes	no	no	no	yes	yes	yes
182180761	chr1	22597513	22597557	20	45	2.250000	no	no	yes	no	no	no	no	no	no	yes
182180772	chr1	22622781	22622818	19	38	2.052632	no	no	no	no	no	no	no	yes	no	no
182180781	chr1	22644065	22644094	15	30	2.000000	no	no	no	yes	no	no	no	no	yes	yes
182180793	chr1	22672680	22672720	20	41	2.050000	no	no	no	no	no	no	no	yes	no	no
182180796	chr1	22679666	22679704	13	39	3.000000	no	no	no	no	no	no	no	no	yes	no
182180817	chr1	22733764	22733993	57	230	4.035088	no	no	yes	no	no	no	no	yes	no	yes
182180819	chr1	22737391	22737448	31	58	1.903226	no	no	yes	no	no	no	no	yes	yes	yes
182180820	chr1	22741128	22741218	45	91	2.022222	no	no	yes	no	no	no	no	yes	yes	yes
182180829	chr1	22752492	22752569	21	78	3.761905	no	no	yes	no	no	no	no	no	no	yes
182180830	chr1	22754796	22754979	21	184	8.761905	no	no	yes	no	no	no	no	yes	yes	yes
182180836	chr1	22778715	22778743	13	29	2.230769	no	no	yes	no	no	no	no	no	no	yes
182180837	chr1	22779332	22779413	43	82	1.953488	no	no	yes	no	no	no	no	no	yes	yes
182180839	chr1	22782688	22782716	13	29	2.230769	no	no	yes	no	no	no	no	yes	no	yes
182180841	chr1	22785128	22785156	13	29	2.230769	no	no	yes	no	no	no	no	no	no	yes
182180845	chr1	22791168	22791212	21	45	2.190476	no	no	yes	no	no	no	no	yes	no	yes
182180849	chr1	22793940	22793985	23	46	2.000000	no	no	yes	no	no	no	no	yes	no	yes
182180868	chr1	22862925	22862976	21	52	2.476191	no	no	yes	no	no	yes	no	no	no	yes

182180874	chr1	22878055	22878272	78	218	2.807692	no	no	yes	no	no	no	no	yes	yes	yes
182180883	chr1	22910581	22910617	16	37	2.312500	no	no	yes	no	no	no	no	no	yes	yes
182180885	chr1	22913327	22913351	7	25	3.571429	no	yes	yes	no	no	no	no	no	no	yes
182180888	chr1	22924285	22924318	13	34	2.692308	no	no	no	no	no	no	no	no	no	no
182180946	chr1	23018809	23018865	16	57	3.562500	yes	no	no	no	no	no	no	yes	yes	no
182180957	chr1	23062779	23062824	13	46	3.333333	no	no	yes	no	no	no	no	no	no	yes
182180962	chr1	23102903	23102953	10	51	4.916667	no	no	yes	no	no	no	no	yes	yes	yes
182180974	chr1	23116588	23116652	30	65	2.166667	no	no	yes	no	no	no	no	yes	no	yes
182180987	chr1	23140107	23140188	41	82	2.000000	yes	no	yes	no	no	no	no	no	no	yes
182180988	chr1	23140777	23140809	16	33	2.062500	no	yes	yes	no	no	no	no	no	no	yes
182180994	chr1	23150216	23150258	23	43	1.913043	no	no	yes	no	no	no	no	no	yes	yes
182181000	chr1	23166998	23167034	16	37	2.312500	no	no	yes	no	no	no	no	yes	yes	yes
182181009	chr1	23176679	23176726	23	48	2.130435	no	no	yes	no	no	no	no	no	no	yes
182181014	chr1	23183979	23184027	24	49	2.041667	no	no	no	no	no	no	no	yes	no	no
182181017	chr1	23187881	23187906	13	26	2.000000	no	no	no	no	no	no	no	yes	yes	no
182181023	chr1	23191979	23192006	13	28	2.153846	no	yes	no	no	no	no	no	no	no	yes
182181025	chr1	23202393	23202441	23	49	2.130435	no	no	no	no	no	no	no	yes	yes	no
182181032	chr1	23218897	23218945	26	49	1.923077	no	no	no	no	no	no	no	yes	no	no
182181035	chr1	23219264	23219293	10	30	3.000000	no	no	no	no	no	no	no	no	yes	no
182181085	chr1	23305778	23305831	27	54	2.111111	no	yes	yes	no	no	no	no	no	no	yes
182181087	chr1	23310930	23310958	12	29	2.416667	no	no	no	yes	yes	no	no	yes	no	yes
182181088	chr1	23313505	23313544	22	40	1.909091	no	no	yes	no	no	yes	no	no	no	yes
182181090	chr1	23316548	23316590	21	43	2.047619	no	no	yes	no	no	no	no	yes	no	yes
182181091	chr1	23320261	23320289	15	29	1.933333	no	no	yes	no	no	no	no	yes	yes	yes
182181100	chr1	23337087	23337128	21	42	2.095238	no	no	yes	no	no	no	no	no	no	yes

182181118	chr1	23360346	23360384	16	39	2.437500	no	yes	no	no	no	no	no	no	no	yes
182181132	chr1	23380636	23380680	14	45	3.200000	no	no	no	no	no	yes	no	yes	yes	no
182181133	chr1	23384843	23384867	13	25	1.923077	no	no	yes	no	no	no	no	yes	yes	yes
182181149	chr1	23402977	23403033	27	57	2.111111	no	no	yes	no	no	yes	no	yes	no	yes
182181151	chr1	23407921	23407953	10	33	3.300000	no	no	yes	no	no	yes	no	no	no	yes
182181161	chr1	23430766	23430919	38	154	4.052631	no	no	yes	no	no	no	no	yes	no	yes
182181166	chr1	23447822	23447866	21	45	2.190476	no	no	yes	no	no	no	no	yes	yes	yes
182181167	chr1	23448038	23448067	11	30	2.727273	no	no	yes	no	no	no	no	yes	yes	yes
182181170	chr1	23455502	23455543	21	42	2.000000	no	no	yes	no	no	yes	no	no	no	yes
182181179	chr1	23484232	23484267	17	36	2.117647	yes	no	yes	no	no	no	yes	yes	yes	yes
182181181	chr1	23485422	23485461	21	40	1.904762	yes	no	no	no	no	no	no	yes	yes	no
182181215	chr1	23523028	23523059	16	32	2.000000	no	no	yes	no	no	no	no	yes	no	yes
182181219	chr1	23530618	23530647	16	30	1.937500	no	no	no	yes	no	no	yes	yes	yes	yes
182181220	chr1	23530975	23531003	14	29	2.071429	no	no	no	no	yes	no	yes	yes	yes	yes
182181227	chr1	23539979	23540016	20	38	1.900000	no	no	no	no	no	no	no	yes	yes	no
182181232	chr1	23548128	23548157	12	30	2.583333	no	no	no	no	no	yes	no	yes	no	no
182181237	chr1	23555636	23555667	16	32	2.000000	no	no	no	no	no	no	no	yes	yes	no
182181239	chr1	23557619	23557668	24	50	2.083333	no	no	no	no	no	yes	no	yes	yes	no
182181245	chr1	23568479	23568524	17	46	2.764706	no	no	no	no	no	no	yes	yes	yes	no
182181246	chr1	23568599	23568630	16	32	2.000000	no	no	no	no	no	no	no	yes	yes	no
182181270	chr1	23636341	23636368	14	28	2.000000	no	no	yes	no	no	no	no	yes	yes	yes
182181301	chr1	23672124	23672155	15	32	2.133333	no	no	yes	no	no	no	no	yes	no	yes
182181308	chr1	23680279	23680305	12	27	2.250000	no	no	no	no	no	yes	no	yes	no	no
182181334	chr1	23703581	23703925	34	345	10.175000	no	no	no	no	no	no	no	yes	no	no
182181339	chr1	23710713	23711314	48	602	12.562500	no	no	yes	no	no	no	no	no	no	yes

182181362	chr1	23761710	23761753	22	44	2.000000	no	yes	yes	no	no	no	no	yes	no	yes
182181377	chr1	23783289	23783430	42	142	3.428571	no	no	yes	no	no	no	no	no	no	yes
182181393	chr1	23828349	23828391	17	43	2.647059	no	no	yes	no	no	no	no	yes	no	yes
182181418	chr1	23856135	23856170	19	36	1.947368	no	no	yes	no	no	no	no	yes	no	yes
182181422	chr1	23860248	23860280	17	33	1.941176	no	no	yes	no	no	no	no	no	no	yes
182181424	chr1	23865304	23865424	60	121	2.016667	no	no	yes	no	no	no	no	yes	yes	yes
182181431	chr1	23875971	23876005	17	35	2.058824	no	no	yes	no	no	no	no	no	no	yes
182181450	chr1	23906005	23906050	24	46	1.916667	no	no	yes	no	no	no	no	yes	yes	yes
182181458	chr1	23916237	23916275	16	39	2.437500	no	no	no	no	no	no	no	yes	no	no
182181461	chr1	23920151	23920182	11	32	2.909091	no	no	no	no	no	no	no	yes	yes	no
182181498	chr1	23979279	23979311	16	33	2.062500	yes	no	yes	no	no	no	no	yes	yes	yes
182181516	chr1	24007087	24007120	17	34	2.058824	no	no	no	no	no	yes	no	no	no	no
182181517	chr1	24008298	24008350	27	53	1.962963	no	no	no	no	yes	no	no	no	no	yes
182181526	chr1	24023123	24023154	16	32	2.000000	no	no	no	no	no	no	no	yes	no	no
182181552	chr1	24052114	24052147	17	34	2.000000	no	no	yes	no	no	no	no	no	no	yes
182181555	chr1	24062993	24063042	19	50	2.571429	no	no	yes	no	no	no	no	no	no	yes
182181557	chr1	24068172	24068213	10	42	4.200000	no	no	yes	no	no	no	no	no	yes	yes
182181568	chr1	24096698	24096756	18	59	3.277778	no	no	yes	no	no	no	no	yes	yes	yes
182181575	chr1	24111895	24111950	20	56	2.727273	no	no	yes	no	no	no	no	yes	yes	yes
182181582	chr1	24120317	24120350	17	34	2.000000	no	yes	no	no	no	no	no	yes	yes	yes
182181583	chr1	24122368	24122417	24	50	2.083333	no	no	yes	no	no	no	no	no	no	yes
182181585	chr1	24128497	24128537	22	41	1.909091	no	no	yes	no	no	no	no	yes	no	yes
182181589	chr1	24146517	24146579	10	63	6.400000	no	no	no	no	no	no	no	no	no	no
182181602	chr1	24166177	24166222	8	46	5.750000	no	no	yes	no	no	no	no	yes	no	yes
182181604	chr1	24166213	24166253	17	41	2.411765	no	no	yes	no	no	no	no	yes	no	yes

182181605	chr1	24166397	24166441	22	45	2.090909	no	no	yes	no	no	no	no	yes	no	yes
182181606	chr1	24168179	24168225	25	47	1.920000	no	no	yes	no	no	no	no	yes	no	yes
182181610	chr1	24173613	24173663	25	51	2.040000	no	no	yes	no	no	no	no	yes	no	yes
182181613	chr1	24182413	24182455	21	43	2.190476	no	no	yes	no	no	no	no	no	no	yes
182181616	chr1	24184698	24184726	15	29	1.933333	no	no	yes	no	no	no	no	yes	no	yes
182181619	chr1	24187287	24187316	7	30	4.285714	yes	no	yes	no	yes	no	yes	yes	yes	yes
182181630	chr1	24198014	24198097	39	84	2.153846	no	no	no	no	no	no	no	yes	yes	no
182181633	chr1	24203112	24203197	17	86	5.058824	no	no	yes	no	no	no	no	yes	no	yes
182181648	chr1	24224926	24224956	15	31	2.066667	no	no	no	no	no	no	no	no	no	no
182181656	chr1	24238067	24238110	18	44	2.421053	no	no	no	no	no	no	no	yes	yes	no
182181657	chr1	24239389	24239416	14	28	2.000000	no	no	no	no	no	no	no	no	no	no
182181660	chr1	24258786	24258851	29	66	2.275862	no	no	no	no	no	no	no	no	no	no
182181684	chr1	24315012	24315053	20	42	2.100000	no	no	yes	no	no	no	no	no	no	yes
182181685	chr1	24319215	24319255	21	41	2.000000	yes	no	yes	no	no	no	no	yes	no	yes
182181688	chr1	24335205	24335255	20	51	2.550000	no	no	yes	no	no	no	no	no	yes	yes
182181689	chr1	24342392	24342429	17	38	2.235294	no	no	yes	no	no	yes	no	no	no	yes
182181691	chr1	24344958	24345327	17	370	22.058823	no	no	yes	no	no	no	no	yes	yes	yes
182181693	chr1	24345323	24345356	14	34	2.428571	no	no	yes	no	no	no	no	yes	yes	yes
182181695	chr1	24352538	24352590	26	53	2.076923	no	no	yes	no	no	no	no	yes	no	yes
182181696	chr1	24353469	24353516	24	48	2.000000	no	no	yes	no	no	no	no	yes	no	yes
182181700	chr1	24363748	24363772	12	25	2.083333	no	no	yes	no	no	no	no	yes	yes	yes
182181701	chr1	24366461	24366497	20	37	1.950000	no	no	yes	no	no	no	no	yes	yes	yes
182181704	chr1	24380750	24380780	16	31	1.937500	yes	no	yes	no	no	no	no	no	no	yes
182181713	chr1	24417151	24417266	57	116	2.035088	yes	no	yes	no	no	no	no	yes	yes	yes
182181716	chr1	24437576	24437611	16	36	2.312500	no	no	yes	no	no	no	no	yes	no	yes

182181719	chr1	24447556	24447601	25	46	1.880000	no	no	yes	no	no	no	no	no	no	yes
182181722	chr1	24462944	24463017	15	74	4.777778	no	no	yes	no	no	no	no	no	no	yes
182181736	chr1	24498476	24498509	15	34	2.266667	no	no	yes	no	no	no	no	no	no	yes
182181740	chr1	24503302	24503334	15	33	2.200000	yes	no	yes	no	no	no	yes	yes	yes	yes
182181745	chr1	24511360	24511467	30	108	3.600000	no	no	yes	no	no	no	no	yes	no	yes
182181748	chr1	24517077	24517122	19	46	2.421053	no	no	yes	no	no	no	no	yes	no	yes
182181753	chr1	24522153	24522194	22	42	1.909091	no	no	yes	no	no	no	no	no	no	yes
182181767	chr1	24545185	24545247	33	63	1.939394	no	no	yes	no	no	no	no	no	no	yes
182181770	chr1	24547985	24548018	16	34	2.125000	no	no	yes	no	no	no	no	no	no	yes
182181776	chr1	24557389	24557459	26	71	2.807692	no	no	yes	no	no	no	no	yes	yes	yes
182181780	chr1	24568747	24568780	17	34	2.058824	no	no	yes	no	no	no	no	yes	no	yes
182181790	chr1	24581766	24581801	17	36	2.117647	no	no	yes	no	no	no	no	no	yes	yes
182181798	chr1	24597517	24597562	18	46	2.555556	no	no	yes	no	no	no	no	yes	no	yes
182181815	chr1	24632333	24632361	15	29	1.933333	yes	no	yes	no	no	no	no	no	yes	yes
182181816	chr1	24633891	24633949	29	59	2.034483	no	no	yes	no	no	no	no	no	no	yes
182181824	chr1	24649674	24649715	19	42	2.210526	yes	no	yes	no	no	yes	no	yes	no	yes
182181825	chr1	24650437	24650462	13	26	2.000000	no	no	yes	no	no	yes	no	no	yes	yes
182181840	chr1	24673601	24673633	16	33	2.062500	no	no	no	no	no	yes	no	no	yes	no
182181856	chr1	24696780	24696830	23	51	2.217391	no	no	no	no	no	no	no	yes	no	no
182181870	chr1	24707821	24707869	23	49	2.130435	no	no	yes	no	no	no	no	yes	yes	yes
182181952	chr1	24804478	24804509	15	32	2.200000	no	no	yes	no	no	no	no	no	no	yes
182181957	chr1	24813398	24813436	20	39	2.000000	no	no	yes	no	no	no	no	no	no	yes
182181970	chr1	24829600	24829646	23	47	2.043478	no	no	yes	no	no	no	no	no	yes	yes
182181994	chr1	24838889	24838935	15	47	3.133333	no	no	yes	no	no	no	no	no	no	yes
182181996	chr1	24849266	24849302	15	37	2.466667	no	no	no	no	no	no	no	yes	yes	no

182182001	chr1	24855985	24856022	20	38	1.900000	no	no	no	no	no	no	no	no	no	no
182182004	chr1	24863406	24863527	39	122	3.128205	no	no	no	no	no	no	no	no	yes	no
182182005	chr1	24863528	24863570	22	43	1.954545	no	no	no	no	no	no	no	no	yes	no
182182006	chr1	24864212	24864243	12	32	2.666667	no	no	no	no	no	no	no	yes	yes	no
182182012	chr1	24867600	24867629	7	30	4.285714	no	no	no	no	no	no	no	no	yes	no
182182016	chr1	24873498	24873667	72	170	2.480000	no	no	no	no	no	no	no	yes	no	no
182182019	chr1	24880556	24880599	13	44	3.384615	no	no	no	no	no	no	no	no	no	no
182182025	chr1	24887502	24887576	26	75	2.884615	no	no	no	no	no	no	no	yes	no	no
182182029	chr1	24900910	24900942	17	33	1.941176	no	yes	no	no	no	yes	no	yes	no	yes
182182032	chr1	24907620	24908338	25	719	28.760000	no	no	yes	no	no	no	yes	yes	no	yes
182182033	chr1	24917168	24917206	20	39	1.950000	no	no	yes	no	no	no	no	yes	no	yes
182182034	chr1	24918227	24918260	14	34	2.428571	no	no	yes	no	no	no	no	yes	no	yes
182182040	chr1	24925117	24925149	17	33	1.941176	no	no	yes	no	no	no	no	yes	no	yes
182182042	chr1	24947227	24947255	15	29	1.933333	no	no	yes	no	no	no	no	yes	no	yes
182182045	chr1	24948538	24948667	68	130	1.955882	no	no	yes	no	no	no	no	yes	no	yes
182182048	chr1	24956442	24956475	18	34	1.944444	no	no	yes	no	no	no	no	no	no	yes
182182064	chr1	25002126	25002515	30	390	13.166667	no	no	no	no	no	no	no	no	no	no
182182068	chr1	25005802	25005881	40	80	2.000000	no	no	no	no	no	no	no	no	yes	no
182182070	chr1	25012820	25012850	16	31	1.937500	no	no	no	no	no	no	no	no	no	no
182182078	chr1	25019821	25019956	58	136	2.379310	no	no	no	no	no	no	no	yes	no	no
182182082	chr1	25020004	25020256	86	253	2.941860	no	no	no	no	no	no	no	yes	yes	no
182182086	chr1	25025365	25025400	16	36	2.250000	no	no	no	no	no	no	no	no	no	no
182182100	chr1	25036461	25036500	9	40	4.444445	no	no	no	no	no	no	no	yes	yes	no
182182109	chr1	25044885	25044985	23	101	4.416667	no	no	yes	no	no	yes	no	yes	yes	yes
182182114	chr1	25054276	25054486	97	211	2.234694	no	no	yes	no	no	no	no	no	no	yes

182182119	chr1	25072022	25072061	12	40	3.333333	no	no	yes	no	no	no	no	yes	no	yes
182182123	chr1	25090850	25090884	15	35	2.333333	no	no	yes	no	no	no	no	yes	yes	yes
182182125	chr1	25094915	25094965	25	51	2.040000	no	no	yes	no	no	no	no	yes	no	yes
182182132	chr1	25108711	25108873	84	163	1.952381	no	no	yes	no	no	no	no	no	no	yes
182182137	chr1	25133963	25134015	23	53	2.347826	no	no	no	no	no	no	no	no	no	no
182182138	chr1	25135960	25136018	30	59	1.966667	no	no	no	no	no	no	no	yes	no	no
182182148	chr1	25146529	25146602	22	74	3.363636	no	no	no	no	no	no	no	yes	yes	no
182182153	chr1	25154614	25154670	21	57	2.772727	no	no	no	no	no	no	no	yes	no	no
182182158	chr1	25164442	25164470	15	29	1.933333	no	no	no	no	no	no	no	no	no	no
182182167	chr1	25179432	25179495	31	64	2.064516	no	no	no	no	no	no	no	yes	yes	no
182182177	chr1	25193662	25193699	20	38	2.000000	no	no	no	no	no	no	no	yes	no	no
182182180	chr1	25194177	25194417	102	241	2.382353	no	no	no	no	no	no	no	yes	yes	no
182182192	chr1	25222621	25222652	14	32	2.285714	no	yes	no	no	no	yes	no	yes	no	yes
182182199	chr1	25257045	25257078	17	34	2.000000	no	no	yes	no	no	no	no	yes	yes	yes
182182225	chr1	25344697	25344742	24	46	1.916667	no	no	yes	no	no	no	no	no	no	yes
182182229	chr1	25359098	25359171	25	74	2.960000	no	no	yes	no	no	no	no	no	no	yes
182182233	chr1	25369112	25369150	21	39	1.904762	no	no	yes	no	no	no	no	no	no	yes
182182254	chr1	25451536	25451573	13	38	3.076923	no	no	yes	no	no	no	no	no	no	yes
182182259	chr1	25455744	25455777	16	34	2.125000	no	no	yes	no	no	no	no	yes	no	yes
182182262	chr1	25471454	25471488	16	35	2.250000	no	no	yes	no	no	no	no	no	yes	yes
182182265	chr1	25482969	25482998	14	30	2.142857	no	no	yes	no	no	no	no	no	no	yes
182182266	chr1	25492792	25492827	12	36	3.000000	no	no	yes	no	no	no	no	no	no	yes
182182278	chr1	25529334	25529492	87	159	1.873563	no	no	no	no	no	no	no	yes	yes	no
182182280	chr1	25531190	25531282	46	93	2.021739	no	no	no	no	no	no	no	yes	no	no
182182285	chr1	25543515	25543552	17	38	2.294118	yes	no	no	no	no	no	yes	yes	yes	no

182182288	chr1	25556157	25556238	40	82	2.050000	no	no	yes	no	no	no	no	no	yes	yes
182182290	chr1	25560189	25560254	22	66	3.000000	no	no	yes	no	no	no	no	no	yes	yes
182182293	chr1	25577184	25577210	14	27	1.928571	no	no	no	no	no	no	no	no	no	no
182182297	chr1	25586511	25586604	40	94	2.350000	no	no	yes	no	no	no	no	yes	no	yes
182182300	chr1	25590829	25591044	33	216	6.545455	no	no	no	no	no	yes	no	no	yes	no
182182306	chr1	25595030	25595175	76	146	1.921053	no	no	no	no	no	no	no	yes	no	no
182182330	chr1	25604933	25604980	11	48	4.416667	no	no	no	no	no	no	no	no	no	no
182182333	chr1	25614445	25614473	9	29	3.333333	no	no	no	no	no	no	no	yes	yes	no
182182335	chr1	25616731	25616761	13	31	2.384615	yes	no	no	no	no	no	no	yes	no	no
182182336	chr1	25617481	25617524	19	44	2.400000	yes	no	no	no	yes	no	yes	yes	no	yes
182182346	chr1	25642224	25642264	20	41	2.050000	no	no	yes	no	no	no	no	yes	yes	yes
182182350	chr1	25655780	25655815	12	36	3.000000	no	no	yes	no	no	no	no	no	yes	yes
182182351	chr1	25655914	25656019	49	106	2.183673	no	no	yes	no	no	no	no	no	no	yes
182182352	chr1	25656784	25656815	12	32	2.666667	no	no	yes	no	no	no	no	yes	yes	yes
182182360	chr1	25663999	25664032	17	34	2.000000	no	no	yes	no	no	no	no	no	yes	yes
182182361	chr1	25669964	25670002	18	39	2.166667	no	no	yes	no	no	no	no	no	yes	yes
182182365	chr1	25678972	25679061	42	90	2.190476	no	no	yes	no	no	no	no	yes	no	yes
182182371	chr1	25685625	25685700	38	76	2.000000	yes	no	yes	no	no	no	no	no	no	yes
182182394	chr1	25759169	25759204	17	36	2.176471	no	yes	yes	no	no	no	no	yes	yes	yes
182182408	chr1	25767426	25768846	570	1421	2.536752	no	no	yes	no	no	no	no	yes	no	yes
182182415	chr1	25769607	25769661	24	55	2.291667	no	no	yes	no	no	no	no	yes	yes	yes
182182416	chr1	25773217	25773260	16	44	2.750000	no	no	yes	no	no	no	no	yes	no	yes
182182418	chr1	25787094	25787213	55	120	2.181818	no	no	no	no	no	yes	no	yes	yes	no
182182425	chr1	25797100	25797172	28	73	2.607143	no	no	no	no	no	no	no	yes	yes	no
182182426	chr1	25798218	25798260	16	43	2.687500	no	no	no	no	no	no	no	yes	yes	no

182182433	chr1	25805623	25805676	23	54	2.347826	no	no	yes	no	no	no	no	yes	no	yes
182182434	chr1	25808290	25808371	15	82	5.466667	no	no	yes	no	no	no	no	no	no	yes
182182455	chr1	25875398	25875422	8	25	3.125000	no	no	no	no	no	no	yes	yes	yes	no
182182456	chr1	25876855	25876902	26	48	1.923077	yes	no	no	no	no	no	no	yes	no	no
182182460	chr1	25880593	25880717	65	125	1.953846	no	no	no	no	no	no	no	yes	yes	no
182182472	chr1	25922480	25922518	21	39	1.904762	no	no	no	no	no	no	no	yes	no	no
182182494	chr1	25955815	25955854	19	40	2.105263	no	no	no	no	no	no	no	yes	no	no
182182516	chr1	26006771	26006927	60	157	2.639344	no	no	no	no	no	yes	no	yes	yes	no
182182517	chr1	26006888	26006999	37	112	3.027027	no	no	no	no	no	yes	no	yes	yes	no
182182526	chr1	26048760	26048798	20	39	1.950000	no	yes	no	no	no	no	no	yes	no	yes
182182529	chr1	26056160	26056202	17	43	2.647059	no	no	yes	no	no	no	no	yes	no	yes
182182533	chr1	26065003	26065041	11	39	3.583333	no	no	yes	no	no	no	no	yes	no	yes
182182557	chr1	26118774	26118824	26	51	1.961538	no	no	yes	no	no	no	no	yes	no	yes
182182560	chr1	26129413	26129461	24	49	2.041667	no	no	no	no	no	no	no	yes	yes	no
182182571	chr1	26147092	26147128	13	37	2.785714	no	no	no	no	no	no	no	no	no	no
182182575	chr1	26149316	26149490	88	175	2.034091	no	no	no	no	no	no	no	no	no	no
182182580	chr1	26162121	26162146	12	26	2.166667	no	yes	no	no	no	no	no	yes	yes	yes
182182582	chr1	26164266	26164297	12	32	2.666667	no	no	no	yes	no	yes	yes	yes	yes	yes
182182584	chr1	26167929	26167966	15	38	2.666667	no	no	yes	no	no	no	no	no	no	yes
182182585	chr1	26168337	26168388	28	52	1.928571	no	no	yes	no	no	no	no	no	no	yes
182182590	chr1	26189463	26189525	34	63	1.941176	no	yes	no	yes	no	yes	no	yes	no	yes
182182601	chr1	26208873	26208964	23	92	3.920000	yes	no	no	no	no	no	no	no	no	no
182182602	chr1	26210241	26210442	90	202	2.244444	no	yes	no	no	no	no	no	yes	yes	yes
182182620	chr1	26241069	26241109	21	41	1.952381	no	no	yes	no	no	no	no	no	no	yes
182182635	chr1	26282317	26282417	24	101	4.208333	no	no	no	yes	yes	yes	no	yes	no	yes

182182648	chr1	26316405	26316429	13	25	1.923077	no	no	yes	no	no	no	no	yes	yes	yes
182182650	chr1	26318689	26318741	21	53	2.523809	yes	yes	yes	no	no	no	no	yes	yes	yes
182182656	chr1	26328159	26328202	16	44	2.750000	no	no	yes	no	no	yes	no	yes	yes	yes
182182664	chr1	26344748	26345283	153	536	3.503268	no	no	yes	yes	no	no	no	yes	no	yes
182182670	chr1	26353878	26353930	25	53	2.160000	no	no	yes	no	no	no	no	yes	no	yes
182182678	chr1	26371112	26371150	16	39	2.437500	yes	no	yes	no	no	no	no	yes	yes	yes
182182699	chr1	26391101	26391143	16	43	2.812500	no	no	no	no	no	no	no	yes	yes	no
182182716	chr1	26410659	26410700	16	42	2.625000	yes	no	no	no	no	no	no	yes	yes	no
182182718	chr1	26417324	26417355	16	32	2.000000	no	no	yes	no	no	no	no	yes	yes	yes
182182724	chr1	26433086	26433122	19	37	1.947368	no	yes	yes	no	no	no	no	yes	yes	yes
182182732	chr1	26440834	26440881	20	48	2.400000	no	no	yes	no	no	no	no	no	no	yes
182182776	chr1	26529114	26529148	14	35	2.571429	yes	no	no	no	no	no	no	yes	no	no
182182777	chr1	26529136	26529175	20	40	2.050000	yes	no	no	no	no	no	no	yes	no	no
182182778	chr1	26529607	26529651	24	45	1.916667	yes	no	no	no	no	no	yes	yes	yes	no
182182779	chr1	26532695	26532732	20	38	1.900000	no	no	yes	no	no	no	no	yes	yes	yes
182182782	chr1	26544320	26544486	62	167	2.725806	no	no	yes	no	no	no	no	yes	yes	yes
182182786	chr1	26545562	26545614	18	53	2.944444	yes	no	yes	no	no	no	no	yes	yes	yes
182182868	chr1	26675800	26675859	31	60	1.935484	no	no	no	no	no	no	no	no	no	no
182182883	chr1	26685049	26685101	26	53	2.038461	no	no	no	no	no	no	no	yes	yes	no
182182885	chr1	26685762	26685804	19	43	2.315789	no	no	no	no	no	no	no	yes	no	no
182182891	chr1	26695949	26696018	30	70	2.366667	yes	no	yes	no	yes	no	yes	yes	yes	yes
182182897	chr1	26697418	26697446	15	29	1.933333	no	no	yes	yes	no	no	yes	yes	no	yes
182182898	chr1	26701406	26701454	25	49	1.960000	no	no	yes	no	no	no	no	yes	yes	yes
182182905	chr1	26713200	26713232	15	33	2.266667	no	no	yes	no	no	no	no	yes	no	yes
182182913	chr1	26731482	26731513	16	32	2.062500	no	no	no	yes	no	yes	no	yes	no	yes

182182918	chr1	26739622	26739653	16	32	2.000000	no	no	yes	no	no	no	no	yes	yes	yes
182182921	chr1	26750728	26750763	17	36	2.117647	no	no	yes	no	no	no	no	yes	no	yes
182182937	chr1	26798751	26798789	19	39	2.157895	no	no	no	no	no	yes	no	yes	no	no
182182963	chr1	26816273	26816325	25	53	2.120000	yes	no	no	no	no	no	no	yes	yes	no
182182986	chr1	26829722	26829765	21	44	2.095238	no	no	yes	no	no	no	no	yes	no	yes
182182987	chr1	26830467	26830557	22	91	4.173913	no	no	yes	no	no	no	no	no	no	yes
182183020	chr1	26873510	26873564	28	55	1.964286	no	no	no	no	no	no	no	yes	no	no
182183022	chr1	26879236	26879276	21	41	1.952381	no	yes	no	no	no	yes	no	yes	no	yes
182183056	chr1	26942666	26942694	12	29	2.416667	no	no	no	yes	no	no	no	yes	no	yes
182183061	chr1	26952508	26952589	41	82	2.000000	no	no	yes	no	no	no	no	yes	no	yes
182183070	chr1	26965395	26965450	28	56	2.000000	no	no	no	no	no	no	no	yes	no	no
182183080	chr1	26974258	26974319	32	62	1.968750	no	no	no	no	no	no	no	no	no	no
182183082	chr1	26976791	26976835	20	45	2.250000	no	no	no	no	no	no	no	yes	no	no
182183094	chr1	27008463	27008537	38	75	2.000000	no	no	yes	no	no	no	no	yes	yes	yes
182183115	chr1	27040048	27040075	7	28	4.000000	no	no	no	no	no	no	no	no	no	no
182183128	chr1	27060997	27061025	15	29	1.933333	no	no	no	no	no	no	no	no	no	no
182183151	chr1	27077020	27077048	15	29	1.933333	no	no	no	no	no	no	no	yes	no	no
182183153	chr1	27077046	27077084	19	39	2.052632	no	no	no	no	no	no	no	yes	no	no
182183186	chr1	27107566	27107627	33	62	1.909091	no	no	yes	no	no	no	no	yes	no	yes
182183239	chr1	27191975	27192012	18	38	2.111111	no	no	no	no	no	no	no	yes	no	no
182183272	chr1	27234686	27234739	28	54	1.964286	yes	no	no	no	yes	no	yes	yes	yes	yes
182183285	chr1	27253328	27253389	30	62	2.066667	no	no	yes	no	no	no	no	yes	yes	yes
182183312	chr1	27283459	27283499	18	41	2.277778	no	no	yes	no	no	no	no	no	no	yes
182183325	chr1	27297072	27297099	12	28	2.333333	no	yes	no	yes	no	no	no	yes	no	yes
182183326	chr1	27307587	27307626	22	40	1.909091	no	yes	yes	no	no	yes	no	yes	yes	yes

182183336	chr1	27342817	27342902	36	86	2.388889	yes	no	yes	no	no	no	no	yes	no	yes
182183339	chr1	27348160	27348227	10	68	6.800000	no	no	yes	no	no	yes	no	yes	no	yes
182183341	chr1	27353494	27353535	12	42	3.384615	no	no	yes	no	no	yes	no	yes	no	yes
182183343	chr1	27356112	27356386	96	275	2.864583	yes	no	yes	no	no	yes	no	yes	no	yes
182183345	chr1	27357843	27357871	15	29	1.933333	yes	no	no	yes	yes	no	no	yes	no	yes
182183347	chr1	27365627	27365665	11	39	3.500000	yes	no	yes	no	no	no	no	yes	yes	yes
182183349	chr1	27373591	27373880	72	290	4.013699	yes	no	yes	no	yes	no	no	yes	no	yes
182183363	chr1	27404815	27404848	12	34	2.833333	no	yes	no	no	no	no	no	yes	yes	yes
182183366	chr1	27408849	27408884	17	36	2.117647	no	no	yes	no	no	no	no	no	no	yes
182183380	chr1	27450804	27450844	15	41	2.733333	no	no	yes	no	no	no	no	yes	no	yes
182183405	chr1	27489253	27489541	96	289	3.059406	no	no	yes	no	no	no	no	yes	yes	yes
182183432	chr1	27529000	27529050	20	51	2.650000	no	no	yes	no	no	no	no	yes	yes	yes
182183444	chr1	27548859	27548890	9	32	3.555556	no	no	no	yes	no	no	no	yes	yes	yes
182183446	chr1	27555468	27555492	13	25	1.923077	no	no	yes	no	no	no	no	yes	yes	yes
182183454	chr1	27563083	27563364	118	282	2.423729	no	no	yes	no	no	no	no	yes	no	yes
182183458	chr1	27586313	27586343	16	31	1.937500	no	no	yes	no	no	no	no	no	no	yes
182183461	chr1	27592284	27592399	48	116	2.458333	no	no	yes	no	no	no	no	yes	yes	yes
182183462	chr1	27593169	27593204	17	36	2.176471	no	no	yes	no	no	no	no	yes	yes	yes
182183464	chr1	27597347	27597387	20	41	2.050000	no	no	yes	no	no	no	no	yes	yes	yes
182183496	chr1	27660010	27660046	8	37	4.625000	no	no	no	no	no	yes	yes	yes	no	no
182183497	chr1	27664902	27664940	19	39	2.052632	no	no	no	no	no	no	no	no	yes	no
182183500	chr1	27668915	27669248	12	334	27.833334	yes	no	yes	yes	no	no	no	yes	yes	yes
182183503	chr1	27672285	27672365	41	81	2.024390	yes	no	yes	no	no	no	no	yes	yes	yes
182183508	chr1	27676837	27676867	14	31	2.285714	no	no	yes	no	no	no	no	yes	no	yes
182183539	chr1	27734809	27734860	25	52	2.080000	no	no	yes	no	no	no	no	yes	no	yes

182183564	chr1	27773077	27773115	12	39	3.250000	yes	no	no	no	no	no	no	yes	yes	no
182183642	chr1	27916195	27916231	19	37	1.947368	no	no	no	no	no	no	no	no	yes	no
182183646	chr1	27916744	27916868	42	125	2.931818	no	no	no	no	no	no	no	yes	yes	no
182183656	chr1	27924129	27924181	27	53	2.000000	no	no	no	no	no	no	no	no	no	no
182183657	chr1	27924890	27924934	22	45	2.090909	no	no	no	no	no	no	no	no	no	no
182183662	chr1	27941872	27941936	29	65	2.241379	no	no	yes	no	no	no	no	yes	yes	yes
182183667	chr1	27960570	27960611	20	42	2.150000	yes	no	yes	no	no	no	no	yes	no	yes
182183669	chr1	27973908	27973939	17	32	1.941176	no	yes	no	no	no	no	no	yes	yes	yes
182183676	chr1	27995756	27995794	20	39	2.000000	no	no	yes	no	no	no	no	no	no	yes
182183699	chr1	28069596	28069629	18	34	1.944444	no	no	yes	no	no	no	no	yes	no	yes
182183706	chr1	28082529	28082597	34	69	2.029412	no	no	yes	no	no	no	no	no	no	yes
182183711	chr1	28087374	28087414	15	41	2.733333	no	no	yes	no	no	no	no	yes	no	yes
182183719	chr1	28106802	28106836	16	35	2.250000	no	no	no	no	no	no	no	no	no	no
182183721	chr1	28111558	28111598	18	41	2.277778	no	no	no	no	no	no	no	yes	yes	no
182183757	chr1	28175926	28176034	52	109	2.096154	no	no	yes	no	no	no	no	yes	no	yes
182183765	chr1	28193798	28193842	23	45	1.956522	no	no	no	no	yes	no	no	yes	yes	yes
182183766	chr1	28194212	28194363	44	152	3.454545	yes	no	no	no	no	no	no	no	no	no
182183793	chr1	28260104	28260180	32	77	2.468750	no	no	yes	no	no	no	yes	yes	yes	yes
182183809	chr1	28292343	28292395	20	53	2.650000	no	no	no	no	no	no	no	no	no	no
182183817	chr1	28299659	28299705	21	47	2.285714	no	no	no	no	no	no	no	no	no	no
182183876	chr1	28366055	28366099	22	45	2.045455	no	no	no	no	no	no	no	yes	yes	no
182183883	chr1	28370936	28370968	17	33	1.941176	no	no	yes	no	no	no	no	yes	yes	yes
182183896	chr1	28394283	28394324	21	42	2.047619	no	no	yes	no	no	no	no	yes	no	yes
182183904	chr1	28408610	28408667	22	58	2.681818	no	no	yes	no	no	no	no	no	no	yes
182183905	chr1	28409876	28409909	16	34	2.125000	no	no	yes	no	no	no	no	yes	yes	yes

182183911	chr1	28411854	28411933	24	80	3.416667	no	no	yes	no	no	no	no	yes	no	yes
182183921	chr1	28442144	28442183	20	40	2.000000	no	no	yes	no	no	no	no	yes	no	yes
182183948	chr1	28484464	28484607	20	144	7.400000	no	no	yes	no	no	no	no	no	no	yes
182183956	chr1	28491803	28491837	14	35	2.500000	no	no	yes	no	no	no	no	yes	no	yes
182183958	chr1	28493910	28493946	9	37	4.222222	no	no	yes	no	no	no	no	no	no	yes
182183965	chr1	28506079	28506112	16	34	2.125000	yes	yes	yes	no	yes	no	no	yes	no	yes
182183969	chr1	28518094	28518144	21	51	2.428571	yes	no	yes	no	yes	no	yes	yes	yes	yes
182183993	chr1	28553060	28553098	14	39	2.785714	yes	yes	no	no	yes	no	yes	yes	yes	yes
182184010	chr1	28581993	28582037	24	45	1.916667	yes	no	yes	no	yes	no	yes	yes	yes	yes
182184086	chr1	28702088	28702124	18	37	2.055556	no	no	yes	no	no	no	no	no	yes	yes
182184093	chr1	28719809	28719837	14	29	2.071429	no	no	no	no	no	yes	no	yes	yes	no
182184100	chr1	28728366	28728509	72	144	2.000000	no	no	no	no	no	no	no	yes	no	no
182184104	chr1	28749626	28749663	19	38	2.000000	no	no	yes	no	no	no	no	yes	yes	yes
182184116	chr1	28766422	28766467	21	46	2.190476	no	no	yes	no	no	no	no	no	yes	yes
182184119	chr1	28769233	28769273	21	41	2.000000	no	yes	no	no	no	yes	no	no	no	yes
182184132	chr1	28777938	28777962	13	25	1.923077	no	no	no	no	no	no	no	yes	no	no
182184143	chr1	28794727	28794768	20	42	2.150000	no	no	no	no	no	no	no	no	no	no
182184154	chr1	28830993	28831025	16	33	2.062500	no	no	yes	no	no	no	no	yes	yes	yes
182184155	chr1	28832081	28832121	14	41	2.928571	no	no	yes	no	no	no	no	no	no	yes
182184165	chr1	28847011	28847070	12	60	5.000000	no	no	yes	no	no	no	no	no	no	yes
182184188	chr1	28877230	28877254	13	25	1.923077	no	no	no	no	yes	no	no	yes	yes	yes
182184195	chr1	28887876	28887909	18	34	1.944444	no	no	yes	no	no	no	no	yes	yes	yes
182184202	chr1	28897666	28897702	18	37	2.055556	no	no	yes	no	no	no	no	yes	no	yes
182184208	chr1	28906665	28906691	14	27	1.928571	no	no	yes	no	no	no	no	no	yes	yes
182184212	chr1	28910638	28910668	13	31	2.384615	no	no	yes	no	no	no	no	yes	yes	yes

182184215	chr1	28919809	28919846	8	38	4.777778	no	no	yes	no	no	no	no	yes	no	yes
182184217	chr1	28919862	28919897	18	36	2.000000	no	no	yes	no	no	no	no	yes	no	yes
182184240	chr1	28968441	28968555	56	115	2.053571	no	no	yes	no	no	no	no	no	no	yes
182184250	chr1	28981843	28981890	22	48	2.181818	no	no	yes	no	no	yes	no	no	no	yes
182184265	chr1	29006089	29006126	20	38	1.950000	no	no	yes	no	no	no	no	no	no	yes
182184274	chr1	29028133	29028168	18	36	2.000000	no	no	yes	no	no	no	no	no	no	yes
182184276	chr1	29035748	29035791	22	44	2.045455	no	no	yes	no	no	no	no	no	no	yes
182184277	chr1	29039205	29039236	16	32	2.000000	yes	no	yes	no	no	no	no	no	no	yes
182184297	chr1	29069581	29069611	15	31	2.066667	no	yes	yes	no	no	yes	no	no	no	yes
182184298	chr1	29073976	29074029	24	54	2.250000	no	no	yes	no	no	yes	no	yes	no	yes
182184315	chr1	29115069	29115105	13	37	3.076923	no	no	yes	no	no	yes	no	no	no	yes
182184318	chr1	29123930	29123968	11	39	3.416667	yes	no	no	no	no	no	yes	yes	yes	no
182184350	chr1	29148845	29148879	12	35	2.916667	no	no	no	yes	yes	no	no	yes	no	yes
182184352	chr1	29149165	29149211	18	47	2.611111	no	no	no	yes	yes	no	no	yes	no	yes
182184356	chr1	29162006	29162043	15	38	2.600000	no	no	yes	no	no	no	no	yes	no	yes
182184362	chr1	29176447	29176471	12	25	2.083333	no	no	yes	no	no	no	no	yes	yes	yes
182184370	chr1	29207004	29207041	12	38	3.071429	yes	no	yes	no	no	no	no	yes	no	yes
182184373	chr1	29223548	29223635	44	88	2.000000	yes	no	yes	no	yes	no	no	yes	yes	yes
182184383	chr1	29236549	29236591	23	43	1.913043	yes	no	no	no	yes	no	yes	yes	yes	yes
182184385	chr1	29237551	29237589	14	39	2.857143	no	no	yes	no	no	no	yes	yes	yes	yes
182184386	chr1	29237848	29237898	26	51	2.000000	no	no	yes	no	no	no	yes	yes	no	yes
182184387	chr1	29238062	29238096	12	35	2.916667	no	no	yes	no	no	no	yes	yes	yes	yes
182184389	chr1	29244426	29244450	13	25	1.923077	no	no	yes	no	no	no	no	yes	no	yes
182184390	chr1	29245660	29245692	11	33	3.000000	no	no	yes	no	no	no	no	yes	no	yes
182184391	chr1	29249157	29249204	22	48	2.181818	no	no	yes	no	no	no	no	yes	yes	yes

182184394	chr1	29266444	29266543	50	100	2.040000	no	no	yes	no	no	no	no	no	no	yes
182184399	chr1	29270183	29270233	17	51	3.000000	no	no	yes	no	no	no	no	no	no	yes
182184400	chr1	29271961	29272051	45	91	2.022222	no	no	yes	no	no	no	no	yes	yes	yes
182184411	chr1	29283604	29283631	12	28	2.333333	yes	no	yes	no	no	no	no	yes	yes	yes
182184420	chr1	29305396	29305424	15	29	1.933333	no	yes	no	yes	no	no	no	no	no	yes
182184422	chr1	29319799	29319836	20	38	2.000000	yes	no	yes	no	no	no	no	yes	no	yes
182184426	chr1	29329035	29329081	20	47	2.350000	no	no	no	no	no	yes	no	no	no	no
182184429	chr1	29341971	29342015	17	45	2.611111	no	no	yes	no	no	no	no	yes	yes	yes
182184436	chr1	29348570	29348714	71	145	2.084507	no	no	yes	no	no	no	no	yes	no	yes
182184439	chr1	29358807	29358861	18	55	3.166667	no	no	no	no	no	no	no	no	yes	no
182184441	chr1	29365206	29365367	71	162	2.281690	no	no	no	no	no	no	no	yes	no	no
182184445	chr1	29381900	29381933	15	34	2.333333	no	no	no	no	no	no	no	no	no	no
182184446	chr1	29389554	29389585	15	32	2.200000	no	no	no	no	no	no	no	yes	no	no
182184450	chr1	29393891	29393975	44	85	1.977273	no	no	no	no	no	no	no	yes	yes	no
182184454	chr1	29409938	29409962	13	25	1.923077	no	no	no	no	no	no	no	no	no	no
182184456	chr1	29415033	29415074	21	42	2.000000	no	no	no	no	no	no	no	no	no	no
182184463	chr1	29453761	29453828	29	68	2.344828	no	no	no	no	no	no	no	yes	yes	no
182184465	chr1	29454136	29454175	18	40	2.222222	no	no	no	no	no	no	no	yes	yes	no
182184466	chr1	29454959	29454996	16	38	2.375000	no	no	no	no	no	no	no	yes	no	no
182184467	chr1	29467846	29467887	22	42	1.954545	no	no	no	no	no	no	no	no	no	no
182184469	chr1	29482200	29482231	16	32	2.000000	no	no	no	no	no	no	no	yes	no	no
182184472	chr1	29482259	29482319	21	61	2.904762	no	no	no	no	no	no	no	yes	no	no
182184473	chr1	29483446	29483474	15	29	1.933333	no	no	no	no	no	no	no	no	no	no
182184474	chr1	29487369	29487404	15	36	2.400000	no	no	no	no	no	yes	no	yes	no	no
182184478	chr1	29504942	29504988	24	47	2.000000	no	no	no	no	no	no	no	no	no	no

182184482	chr1	29516255	29516292	20	38	1.950000	no	no	no	no	no	no	no	no	no	no
182184487	chr1	29522648	29522698	18	51	2.833333	no	no	no	no	no	no	no	yes	yes	no
182184493	chr1	29535661	29535712	23	52	2.260870	no	no	no	no	no	no	no	no	no	no
182184497	chr1	29541361	29541411	19	51	2.684211	no	no	no	no	no	no	no	no	no	no
182184498	chr1	29545707	29545754	24	48	2.000000	no	no	no	no	no	no	no	no	yes	no
182184501	chr1	29548818	29549128	140	311	2.251748	no	no	no	no	no	no	no	no	yes	no
182184511	chr1	29554435	29554675	121	241	2.008265	no	no	no	no	no	no	no	yes	yes	no
182184520	chr1	29563698	29563733	17	36	2.176471	no	no	no	no	no	no	no	yes	yes	no
182184527	chr1	29593583	29593614	16	32	2.000000	no	no	no	no	no	no	no	no	no	no
182184543	chr1	29641193	29641254	27	62	2.407408	no	no	no	no	no	no	no	no	no	no
182184549	chr1	29671626	29671703	32	78	2.437500	no	no	no	no	no	no	no	yes	no	no
182184557	chr1	29686721	29686939	45	219	4.866667	no	no	no	no	no	no	no	no	no	no
182184560	chr1	29698206	29698253	19	48	2.526316	no	no	no	no	no	no	no	yes	yes	no
182184575	chr1	29727193	29727914	28	722	25.806452	no	no	no	no	no	no	no	no	no	no
182184578	chr1	29734644	29734684	20	41	2.050000	no	no	no	no	no	no	no	no	no	no
182184579	chr1	29735764	29735814	25	51	2.120000	no	no	no	no	no	no	no	no	no	no
182184580	chr1	29738080	29738125	15	46	3.133333	no	no	no	no	no	no	no	no	no	no
182184583	chr1	29747312	29747348	17	37	2.235294	no	no	no	no	no	no	no	no	yes	no
182184587	chr1	29761587	29761644	30	58	1.933333	no	no	yes	no	no	no	no	no	no	yes
182184589	chr1	29767028	29767057	10	30	3.000000	no	no	yes	no	no	no	no	no	no	yes
182184595	chr1	29770650	29770868	71	219	2.987500	no	no	yes	no	no	no	no	no	no	yes
182184596	chr1	29772656	29772693	17	38	2.352941	no	no	yes	no	no	no	no	no	no	yes
182184605	chr1	29794570	29794681	31	112	3.545455	no	no	no	no	no	no	no	no	no	no
182184613	chr1	29820809	29820907	50	99	2.000000	no	no	no	no	no	no	no	no	no	no
182184622	chr1	29847400	29847460	31	61	1.967742	no	no	no	no	no	no	no	yes	no	no

182184624	chr1	29859212	29859453	49	242	4.901961	no	no	no	no	no	no	no	no	no	no
182184628	chr1	29876336	29876406	25	71	2.840000	no	no	no	no	no	no	no	no	no	no
182184631	chr1	29880444	29880485	20	42	2.100000	no	no	no	no	no	no	no	yes	no	no
182184643	chr1	29915286	29915314	15	29	1.933333	no	no	no	no	no	no	no	no	no	no
182184657	chr1	29937227	29937260	16	34	2.125000	no	no	no	no	no	no	no	no	no	no
182184659	chr1	29947770	29947820	27	51	1.962963	no	no	no	no	no	no	no	no	no	no
182184661	chr1	29948689	29948747	30	59	1.966667	no	no	no	no	no	no	no	yes	yes	no
182184664	chr1	29962891	29963466	31	576	18.593750	no	no	no	no	no	no	no	yes	yes	no
182184665	chr1	29963795	29963826	15	32	2.133333	no	no	no	no	no	no	no	no	no	no
182184675	chr1	29991379	29991430	22	52	2.409091	no	no	no	no	no	no	no	yes	no	no
182184676	chr1	30006661	30006701	21	41	1.952381	no	no	no	no	no	no	no	yes	no	no
182184679	chr1	30008773	30008833	28	61	2.178571	no	no	no	no	no	no	no	yes	yes	no
182184681	chr1	30011159	30011193	12	35	2.916667	no	no	no	no	no	no	no	yes	no	no
182184683	chr1	30011556	30011589	16	34	2.125000	no	no	no	no	no	no	no	no	no	no
182184692	chr1	30025494	30025527	17	34	2.117647	no	no	yes	no	no	no	no	no	no	yes
182184700	chr1	30056112	30056153	18	42	2.500000	no	no	no	no	no	no	no	no	no	no
182184702	chr1	30062299	30062335	19	37	1.947368	no	no	no	no	no	no	no	no	no	no
182184704	chr1	30066266	30066311	22	46	2.136364	no	no	no	no	no	no	no	no	no	no
182184707	chr1	30080274	30080352	23	79	3.434783	no	no	no	no	no	no	no	yes	no	no
182184708	chr1	30082175	30082249	29	75	2.586207	no	no	no	no	no	no	no	no	no	no
182184709	chr1	30088454	30088482	13	29	2.230769	no	no	no	no	no	no	no	no	no	no
182184716	chr1	30101647	30101704	22	58	2.636364	no	no	no	no	no	no	no	yes	yes	no
182184719	chr1	30108050	30108120	31	71	2.290323	no	no	no	no	no	no	no	no	no	no
182184724	chr1	30130430	30130519	45	90	2.000000	no	no	no	no	no	no	no	yes	no	no
182184727	chr1	30162192	30162221	14	30	2.142857	no	no	no	no	no	no	no	no	no	no

182184740	chr1	30191397	30191654	42	258	6.166667	no	no	no	no	no	no	no	yes	yes	no
182184742	chr1	30197092	30197153	27	62	2.296296	no	no	no	no	no	no	no	no	no	no
182184750	chr1	30198303	30198419	60	117	1.983333	no	no	no	no	no	no	no	yes	no	no
182184764	chr1	30216658	30216723	28	66	2.392857	no	no	no	no	no	no	no	yes	yes	no
182184769	chr1	30225142	30225435	148	294	2.006757	no	no	no	no	no	no	no	yes	no	no
182184772	chr1	30234592	30234641	18	50	2.777778	no	no	no	no	no	no	no	no	no	no
182184773	chr1	30244270	30244305	16	36	2.250000	no	no	no	no	no	no	no	no	no	no
182184780	chr1	30283532	30283573	20	42	2.100000	no	no	no	no	no	no	no	no	no	no
182184782	chr1	30283609	30283770	74	162	2.202703	no	no	no	no	no	no	no	no	no	no
182184785	chr1	30283846	30283933	37	88	2.378378	no	no	no	no	no	no	no	no	no	no
182184790	chr1	30302333	30302357	13	25	1.923077	no	no	no	no	no	no	no	no	no	no
182184793	chr1	30312078	30312113	17	36	2.117647	no	no	no	no	no	no	no	no	no	no
182184794	chr1	30314628	30314676	17	49	2.882353	no	no	no	no	no	no	no	no	no	no
182184796	chr1	30335122	30335165	16	44	2.812500	no	no	no	no	no	no	no	no	no	no
182184808	chr1	30356414	30356457	17	44	2.588235	no	no	no	no	no	no	no	no	no	no
182184811	chr1	30360541	30360565	13	25	1.923077	no	no	no	no	no	no	no	no	no	no
182184815	chr1	30366354	30366420	25	67	2.730769	no	no	no	no	no	no	no	no	no	no
182184819	chr1	30375544	30376099	54	556	10.272727	no	no	no	no	no	no	no	no	no	no
182184826	chr1	30385358	30385383	11	26	2.363636	no	no	no	no	no	no	no	no	yes	no
182184828	chr1	30398177	30398616	107	440	4.090090	no	no	no	no	no	no	no	no	no	no
182184830	chr1	30398930	30398956	14	27	1.928571	no	no	no	no	no	no	no	no	no	no
182184834	chr1	30409675	30409795	55	121	2.196429	no	no	yes	no	yes	no	no	no	yes	yes
182184843	chr1	30461502	30461947	107	446	4.162162	no	no	no	no	no	no	no	yes	yes	no
182184849	chr1	30464605	30464645	21	41	1.952381	no	no	no	no	no	no	no	yes	no	no
182184855	chr1	30468458	30468497	18	40	2.222222	no	no	no	no	no	no	no	no	no	no

182184858	chr1	30477235	30477277	22	43	2.000000	no	no	no	no	no	no	no	no	no	no
182184863	chr1	30479176	30479205	15	30	2.000000	no	no	no	no	no	no	no	no	no	no
182184871	chr1	30485674	30485706	16	33	2.062500	no	no	no	no	no	no	no	yes	no	no
182184873	chr1	30488289	30488369	40	81	2.025000	no	no	no	no	no	no	no	yes	no	no
182184892	chr1	30508147	30508175	13	29	2.230769	no	no	no	no	no	no	no	no	no	no
182184897	chr1	30523892	30523934	19	43	2.315789	no	no	no	no	no	no	no	no	no	no
182184898	chr1	30523977	30524039	31	63	2.032258	no	no	no	no	no	no	no	no	no	no
182184918	chr1	30541037	30541085	9	49	5.444445	no	no	no	no	no	no	no	no	no	no
182184936	chr1	30598067	30598246	29	180	6.413793	no	no	no	no	no	no	no	yes	yes	no
182184943	chr1	30609745	30609898	76	154	2.078947	no	no	no	no	no	no	no	no	no	no
182184947	chr1	30621673	30621749	34	77	2.352941	no	no	no	no	no	no	no	no	yes	no
182184953	chr1	30629634	30629735	36	102	2.833333	no	no	no	no	no	no	no	no	no	no
182184954	chr1	30631205	30631229	12	25	2.083333	no	no	no	no	no	no	no	no	no	no
182184959	chr1	30644803	30644844	20	42	2.200000	no	no	no	no	no	no	no	yes	no	no
182184962	chr1	30645429	30645491	31	63	2.064516	no	no	no	no	no	no	no	no	no	no
182184966	chr1	30649717	30649849	67	133	1.985075	no	no	no	no	no	no	no	yes	yes	no
182184969	chr1	30650309	30650349	12	41	3.307692	no	no	no	no	no	no	no	no	no	no
182184974	chr1	30651598	30651948	124	351	2.911290	no	no	no	no	no	no	no	yes	yes	no
182184979	chr1	30656157	30656194	20	38	1.900000	no	no	no	no	no	no	no	yes	yes	no
182184980	chr1	30659023	30659087	34	65	1.911765	no	no	no	no	no	no	no	yes	yes	no
182184983	chr1	30660994	30661047	29	54	1.896552	no	no	no	no	no	no	no	no	no	no
182184984	chr1	30667522	30667557	16	36	2.250000	no	no	no	no	no	no	no	no	no	no
182184985	chr1	30671061	30671090	11	30	2.727273	no	no	no	no	no	no	no	no	no	no
182184994	chr1	30685637	30685685	14	49	3.500000	no	no	no	no	no	no	no	yes	yes	no
182184996	chr1	30689997	30690050	29	54	1.931034	no	no	no	no	no	no	no	yes	no	no

182185004	chr1	30712606	30712630	13	25	1.923077	no	yes	no	no	no	no	no	yes	no	yes
182185007	chr1	30713378	30713435	16	58	3.625000	no	yes	no	no	no	yes	no	yes	yes	yes
182185011	chr1	30718452	30718680	129	229	1.806202	yes	yes	yes	no	no	no	yes	yes	yes	yes
182185014	chr1	30727877	30727986	54	110	2.037037	no	no	no	no	no	no	no	yes	yes	no
182185018	chr1	30740398	30740430	17	33	1.941176	no	no	yes	no	no	no	no	yes	yes	yes
182185025	chr1	30773148	30773453	37	306	8.270270	no	no	no	no	no	no	yes	yes	no	no
182185027	chr1	30775808	30775859	27	52	2.037037	no	no	no	no	no	no	no	yes	no	no
182185034	chr1	30782399	30782436	16	38	2.375000	no	no	no	no	no	no	no	yes	yes	no
182185037	chr1	30791237	30791289	24	53	2.208333	no	no	no	no	no	no	no	yes	no	no
182185042	chr1	30793535	30793566	16	32	2.000000	no	no	no	no	no	no	no	yes	no	no
182185056	chr1	30807388	30807426	20	39	2.000000	no	no	no	no	no	no	yes	yes	no	no
182185081	chr1	30869210	30869249	21	40	1.952381	no	no	no	no	no	yes	no	yes	yes	no
182185084	chr1	30874029	30874064	18	36	2.000000	no	no	yes	no	no	no	no	yes	yes	yes
182185091	chr1	30894421	30894453	12	33	2.692308	no	no	yes	no	no	no	no	yes	no	yes
182185095	chr1	30900797	30900833	20	37	1.900000	no	no	yes	no	no	no	no	yes	no	yes
182185104	chr1	30907743	30907770	10	28	2.800000	no	no	yes	no	no	no	no	yes	yes	yes
182185106	chr1	30908501	30908548	18	48	2.631579	no	no	no	yes	no	no	yes	yes	no	yes
182185107	chr1	30908587	30908626	19	40	2.210526	no	no	no	no	yes	no	yes	yes	yes	yes
182185109	chr1	30908643	30908687	24	45	1.916667	no	no	no	no	yes	no	yes	yes	yes	yes
182185111	chr1	30915103	30915149	15	47	3.000000	no	no	no	no	no	no	no	yes	no	no
182185121	chr1	30926917	30927026	38	110	2.947368	no	no	no	no	no	no	no	no	no	no
182185135	chr1	30958275	30958299	12	25	2.083333	no	no	yes	no	no	no	no	no	no	yes
182185138	chr1	30970619	30970771	77	153	1.987013	no	no	yes	no	no	no	no	yes	yes	yes
182185145	chr1	30981706	30981751	12	46	3.769231	no	no	yes	no	no	no	no	no	no	yes
182185148	chr1	30995475	30995518	17	44	2.588235	yes	no	yes	no	no	no	no	no	no	yes

182185151	chr1	31003956	31004017	30	62	2.066667	no	no	yes	no	no	no	no	no	no	yes
182185156	chr1	31012525	31012559	19	35	1.947368	no	no	yes	no	no	no	no	yes	yes	yes
182185163	chr1	31032326	31032352	13	27	2.076923	no	no	yes	no	no	no	no	yes	no	yes
182185179	chr1	31050167	31050211	22	45	2.045455	yes	no	yes	no	no	no	no	no	no	yes
182185213	chr1	31090010	31090047	7	38	5.428571	no	no	no	no	no	no	no	no	no	no
182185226	chr1	31108693	31108733	17	41	2.529412	yes	no	no	no	no	no	no	no	no	no
182185232	chr1	31109151	31109192	17	42	2.529412	yes	no	no	no	no	no	no	no	no	no
182185248	chr1	31117289	31117325	15	37	2.533333	no	no	no	no	no	no	no	no	no	no
182185261	chr1	31118064	31118106	20	43	2.150000	no	no	no	no	no	no	no	no	no	no
182185263	chr1	31118128	31118167	20	40	2.000000	no	no	no	no	no	no	no	no	no	no
182185274	chr1	31134333	31134369	15	37	2.466667	no	no	no	no	no	no	no	yes	no	no
182185296	chr1	31155176	31155210	15	35	2.333333	no	no	no	no	no	no	no	yes	no	no
182185297	chr1	31155513	31155564	21	52	2.476191	no	no	no	no	no	no	yes	yes	yes	no
182185316	chr1	31186813	31186840	14	28	2.000000	no	no	yes	no	no	no	no	yes	yes	yes
182185317	chr1	31190511	31190552	21	42	2.047619	no	no	yes	no	no	no	no	yes	no	yes
182185342	chr1	31214519	31214547	14	29	2.071429	no	no	yes	no	no	no	no	yes	no	yes
182185346	chr1	31214537	31214589	26	53	2.038461	no	no	yes	no	no	no	no	yes	no	yes
182185354	chr1	31235876	31235903	14	28	2.000000	no	no	yes	no	no	no	no	no	no	yes
182185367	chr1	31263080	31263123	11	44	4.181818	no	no	yes	no	no	yes	no	no	no	yes
182185368	chr1	31272255	31272289	16	35	2.187500	no	no	yes	no	no	no	no	no	no	yes
182185374	chr1	31313397	31313434	13	38	2.923077	no	no	yes	no	no	no	no	no	no	yes
182185391	chr1	31364149	31364187	12	39	3.250000	no	yes	no	yes	no	no	no	yes	no	yes
182185410	chr1	31404434	31404470	19	37	2.000000	no	no	no	no	no	no	no	no	no	no
182185413	chr1	31413154	31413200	17	47	2.764706	yes	no	yes	no	no	no	yes	yes	yes	yes
182185415	chr1	31413853	31413888	18	36	2.055556	no	yes	yes	no	yes	no	yes	yes	yes	yes

182185418	chr1	31415878	31415910	16	33	2.062500	no	yes	yes	no	no	no	no	yes	yes	yes
182185422	chr1	31430617	31430666	20	50	2.500000	no	no	yes	no	no	no	no	no	yes	yes
182185423	chr1	31431092	31431133	22	42	1.909091	no	no	yes	no	no	no	no	yes	no	yes
182185424	chr1	31431745	31432232	9	488	54.222221	no	no	yes	no	no	no	no	yes	yes	yes
182185425	chr1	31434213	31434247	17	35	2.058824	no	yes	no	no	no	no	no	yes	yes	yes
182185437	chr1	31469831	31469926	32	96	3.000000	no	no	no	no	no	no	no	yes	no	no
182185462	chr1	31522633	31522674	18	42	2.333333	no	yes	yes	no	no	yes	no	yes	no	yes
182185463	chr1	31533004	31533028	12	25	2.083333	no	no	yes	no	no	no	no	no	no	yes
182185470	chr1	31548963	31548994	16	32	2.062500	no	no	yes	no	no	no	no	yes	yes	yes
182185480	chr1	31569740	31569829	45	90	2.000000	no	no	yes	no	no	no	no	yes	no	yes
182185481	chr1	31569929	31569969	21	41	2.047619	no	no	yes	no	no	no	no	yes	yes	yes
182185490	chr1	31580492	31580536	24	45	1.916667	no	no	yes	no	no	no	no	yes	yes	yes
182185511	chr1	31641238	31641283	24	46	2.000000	no	no	yes	no	no	no	no	yes	no	yes
182185519	chr1	31680795	31680890	47	96	2.042553	no	no	yes	no	no	no	no	no	yes	yes
182185521	chr1	31683706	31683750	24	45	1.958333	yes	no	yes	yes	yes	yes	no	no	no	yes
182185522	chr1	31683800	31683829	15	30	2.066667	yes	no	yes	no	no	yes	no	yes	no	yes
182185526	chr1	31699461	31699506	18	46	2.555556	no	no	yes	no	no	no	no	yes	yes	yes
182185529	chr1	31706453	31706493	18	41	2.388889	no	no	no	no	no	no	no	yes	yes	no
182185534	chr1	31709028	31709076	21	49	2.333333	no	no	no	no	no	no	no	yes	no	no
182185537	chr1	31723163	31723192	12	30	2.500000	no	no	no	no	no	no	no	no	no	no
182185540	chr1	31734808	31734849	22	42	1.909091	no	no	yes	no	no	no	no	yes	yes	yes
182185542	chr1	31742638	31742673	18	36	2.000000	no	no	yes	no	no	no	no	yes	no	yes
182185544	chr1	31764054	31764098	24	45	1.958333	yes	no	yes	no	yes	no	yes	yes	no	yes
182185545	chr1	31764352	31764407	29	56	1.965517	yes	no	yes	no	no	no	yes	yes	yes	yes
182185546	chr1	31765382	31765440	28	59	2.107143	yes	no	no	no	no	no	no	yes	yes	no

182185547	chr1	31769183	31769215	17	33	1.941176	no	no	no	no	no	no	no	yes	yes	no
182185551	chr1	31786069	31786098	16	30	1.937500	no	no	no	no	no	no	no	yes	no	no
182185596	chr1	31859711	31859748	19	38	2.000000	no	no	yes	no	no	no	no	yes	no	yes
182185611	chr1	31892958	31893055	28	98	3.500000	no	no	yes	no	no	no	no	no	no	yes
182185626	chr1	31929749	31929781	17	33	2.000000	no	no	yes	no	no	no	no	yes	no	yes
182185627	chr1	31936526	31936558	17	33	1.941176	no	no	yes	no	no	no	no	yes	yes	yes
182185630	chr1	31938195	31938250	19	56	2.904762	yes	no	yes	no	yes	no	yes	yes	no	yes
182185632	chr1	31944186	31944214	14	29	2.071429	no	no	yes	no	no	no	no	yes	yes	yes
182185665	chr1	32013111	32013150	16	40	2.529412	yes	no	no	no	no	no	no	yes	yes	no
182185666	chr1	32017348	32017578	117	231	1.974359	no	no	yes	no	no	no	no	no	no	yes
182185695	chr1	32077754	32077809	23	56	2.434783	no	no	yes	no	no	no	no	no	no	yes
182185698	chr1	32081051	32081157	53	107	2.018868	no	no	yes	no	no	no	no	yes	no	yes
182185749	chr1	32161571	32161613	22	43	1.954545	no	no	yes	no	no	no	no	no	no	yes
182185755	chr1	32168984	32169033	25	50	2.000000	no	no	yes	no	no	no	no	no	no	yes
182185761	chr1	32195956	32196009	22	54	2.454545	no	yes	no	no	no	no	no	yes	no	yes
182185762	chr1	32204120	32204148	8	29	3.625000	no	no	yes	no	no	yes	no	yes	yes	yes
182185775	chr1	32227819	32227858	12	40	3.333333	no	no	yes	no	no	no	no	yes	no	yes
182185791	chr1	32242398	32242487	28	90	3.214286	yes	no	no	no	no	no	no	yes	no	no
182185837	chr1	32274267	32274295	15	29	1.933333	no	no	yes	yes	no	no	no	yes	no	yes
182185846	chr1	32282816	32282863	17	48	2.823529	no	no	yes	no	no	no	no	yes	no	yes
182185848	chr1	32284260	32284342	19	83	4.578948	no	no	yes	no	no	no	no	no	no	yes
182185853	chr1	32292339	32292405	35	67	1.971429	no	no	yes	no	no	no	yes	yes	yes	yes
182185862	chr1	32299404	32299446	17	43	2.588235	no	no	yes	no	no	no	no	yes	yes	yes
182185863	chr1	32301096	32301166	35	71	2.028571	no	no	yes	no	no	no	no	no	yes	yes
182185874	chr1	32334305	32334346	19	42	2.315789	no	yes	no	no	no	yes	no	yes	yes	yes

182185895	chr1	32357273	32357309	16	37	2.312500	no	no	yes	no	no	no	no	yes	no	yes
182185897	chr1	32360659	32360683	13	25	1.923077	no	no	yes	no	no	yes	no	yes	no	yes
182185899	chr1	32361294	32361339	25	46	1.840000	no	yes	yes	no	yes	yes	no	yes	no	yes
182185912	chr1	32398419	32398452	16	34	2.125000	no	no	no	no	no	no	no	yes	no	no
182185913	chr1	32401043	32401080	16	38	2.375000	no	no	no	no	no	no	no	yes	yes	no
182185915	chr1	32408265	32408296	17	32	1.941176	no	no	no	no	no	no	no	no	no	no
182185922	chr1	32421703	32421738	17	36	2.176471	yes	no	no	no	no	no	no	no	no	no
182185931	chr1	32430952	32430982	14	31	2.214286	no	no	no	no	no	no	no	no	no	no
182185957	chr1	32471033	32471082	12	50	4.166667	no	no	no	yes	no	no	no	yes	no	yes
182185972	chr1	32500418	32500453	8	36	4.625000	no	no	yes	no	no	no	no	yes	yes	yes
182185973	chr1	32500698	32500747	26	50	2.000000	no	no	yes	no	no	no	yes	yes	yes	yes
182185975	chr1	32500833	32500876	23	44	1.913043	no	no	yes	no	no	no	yes	yes	yes	yes
182185995	chr1	32539767	32539809	18	43	2.400000	no	no	yes	no	no	no	yes	yes	yes	yes
182185997	chr1	32545204	32545241	18	38	2.111111	no	no	yes	no	no	no	no	no	no	yes
182186037	chr1	32600098	32600139	23	42	1.913043	no	yes	yes	yes	no	yes	no	yes	yes	yes
182186040	chr1	32607919	32607955	16	37	2.312500	no	no	yes	no	no	no	no	no	no	yes
182186069	chr1	32658881	32659093	110	213	2.045455	no	no	yes	no	no	no	no	yes	yes	yes
182186127	chr1	32741721	32741752	17	32	1.941176	yes	no	no	no	no	no	yes	yes	yes	no
182186130	chr1	32747010	32747047	20	38	1.900000	no	no	yes	no	no	no	no	no	no	yes
182186142	chr1	32786489	32786537	23	49	2.130435	no	no	yes	no	yes	yes	no	yes	no	yes
182186163	chr1	32805207	32805299	16	93	5.823529	no	no	yes	no	no	no	no	yes	no	yes
182186169	chr1	32823764	32823804	13	41	3.071429	no	no	yes	no	no	no	no	no	no	yes
182186177	chr1	32844771	32844838	28	68	2.428571	no	no	yes	no	no	no	no	no	no	yes
182186186	chr1	32870389	32870420	14	32	2.285714	no	no	yes	no	no	yes	no	yes	yes	yes
182186191	chr1	32876244	32876275	16	32	2.000000	no	no	no	no	no	no	no	yes	yes	no

182186192 chr1 32878261 32878297 19 37 2.000000 no no no no no
no no yes no no
182186195 chr1 32882739 32882772 14 34 2.500000 no no no no no
no no yes yes no
182186215 chr1 32934054 32934088 18 35 1.944444 no no yes no no
no no yes yes yes
182186220 chr1 32947272 32947345 27 74 2.814815 no no yes no no
no no no no yes
182186231 chr1 32964933 32964971 15 39 2.600000 yes no no no no
no yes yes yes no
182186264 chr1 32986475 32986511 17 37 2.176471 yes no no no no
no no no no no
182186268 chr1 32996037 32996080 23 44 1.913043 no no yes no no
no no no no yes
182186286 chr1 33035949 33035984 15 36 2.466667 no no yes no no
no no yes no yes
182186316 chr1 33092242 33092336 16 95 5.937500 yes no yes no no
yes no no no yes
182186338 chr1 33160726 33160754 13 29 2.230769 no no yes no no
no no no no yes
182186344 chr1 33174105 33174141 14 37 2.714286 no no yes no no
no no no no yes
182186347 chr1 33175002 33175050 18 49 2.722222 no no yes no no
no no no no yes
182186353 chr1 33180946 33180998 17 53 3.055556 no no yes no no
yes yes yes yes yes
182186373 chr1 33221827 33221879 21 53 2.523809 no no no no no
no no no no no
182186374 chr1 33224503 33224540 17 38 2.235294 no no no no no
no no yes no no
182186384 chr1 33250746 33250903 74 158 2.162162 no no no no no
no no yes no no
182186389 chr1 33256540 33256590 25 51 2.040000 yes no no no
yes no yes yes yes yes
182186391 chr1 33256661 33256702 23 42 1.913043 no no yes no no
no yes yes no yes
182186392 chr1 33256904 33256944 18 41 2.277778 no no yes no no
no yes yes yes yes
182186394 chr1 33268168 33268218 24 51 2.125000 no no yes no no
no no yes no yes
182186396 chr1 33270441 33270474 15 34 2.266667 yes no yes no no
no no no no yes
182186397 chr1 33271701 33271830 43 130 3.023256 no no yes no
no no no no yes yes
182186402 chr1 33296349 33296377 15 29 1.933333 no no yes no no
no no yes yes yes
182186403 chr1 33299334 33299372 15 39 2.600000 no yes no no no
no no yes no yes
182186404 chr1 33300284 33300315 17 32 1.941176 no yes no no no
no no no no yes

182186405	chr1	33304383	33304422	19	40	2.105263	no	no	no	no	no	no	no	no	yes	no
182186424	chr1	33318839	33318877	21	39	1.952381	no	no	yes	no	no	no	no	no	yes	yes
182186428	chr1	33349796	33349843	25	48	2.000000	yes	no	yes	no	yes	yes	yes	yes	no	yes
182186437	chr1	33371390	33371546	19	157	8.263158	no	no	yes	no	no	yes	no	no	no	yes
182186439	chr1	33372327	33372398	37	72	2.000000	no	no	no	yes	yes	no	no	no	no	yes
182186443	chr1	33379287	33379378	46	92	2.065217	no	no	yes	no	no	no	no	yes	yes	yes
182186447	chr1	33386737	33386779	22	43	1.954545	no	no	yes	no	no	no	no	yes	no	yes
182186448	chr1	33386767	33386812	20	46	2.300000	no	no	yes	no	no	no	no	yes	no	yes
182186450	chr1	33386807	33386872	24	66	2.750000	no	no	yes	no	no	no	no	yes	yes	yes
182186454	chr1	33392172	33392212	20	41	2.050000	no	no	yes	no	no	no	no	yes	no	yes
182186456	chr1	33404561	33404609	25	49	2.000000	no	no	yes	no	no	no	no	no	no	yes
182186457	chr1	33408331	33408378	25	48	1.920000	no	no	yes	no	no	no	no	no	yes	yes
182186466	chr1	33424164	33424218	26	55	2.115385	no	no	yes	no	no	no	no	yes	no	yes
182186467	chr1	33426798	33426851	22	54	2.454545	no	no	yes	no	no	no	no	no	no	yes
182186475	chr1	33445347	33445390	22	44	2.000000	no	no	no	no	no	no	no	yes	yes	no
182186483	chr1	33472866	33472916	27	51	1.925926	no	no	yes	no	no	no	no	yes	yes	yes
182186485	chr1	33476302	33476350	24	49	2.041667	no	no	yes	no	no	no	no	no	no	yes
182186486	chr1	33476713	33476785	37	73	1.972973	no	no	yes	no	no	no	no	no	no	yes
182186488	chr1	33481823	33481859	18	37	2.055556	no	no	yes	no	no	no	no	no	no	yes
182186490	chr1	33494524	33494752	84	229	2.726191	no	no	no	yes	no	no	no	yes	yes	yes
182186495	chr1	33499332	33499379	24	48	2.083333	no	no	no	no	no	no	no	no	yes	no
182186506	chr1	33516040	33516077	19	38	2.000000	no	yes	no	no	no	no	no	yes	yes	yes
182186513	chr1	33549729	33549832	52	104	2.000000	no	no	yes	no	no	no	no	no	no	yes
182186516	chr1	33576866	33576895	15	30	2.000000	no	no	yes	no	no	no	no	yes	no	yes
182186517	chr1	33577670	33577711	19	42	2.210526	no	no	yes	no	no	no	no	yes	no	yes

182186522	chr1	33599922	33599957	12	36	3.000000	no	yes	yes	no	yes	no	no	yes	yes	yes
182186523	chr1	33613443	33613504	31	62	2.000000	no	no	yes	no	no	no	no	yes	no	yes
182186524	chr1	33619292	33619338	19	47	2.473684	no	no	yes	no	no	no	no	no	no	yes
182186525	chr1	33627693	33627732	20	40	2.050000	no	no	yes	no	no	no	no	yes	yes	yes
182186532	chr1	33656139	33656182	20	44	2.200000	no	no	yes	no	no	no	no	no	no	yes
182186533	chr1	33663182	33663217	16	36	2.250000	no	no	yes	no	no	no	no	no	no	yes
182186542	chr1	33689013	33689133	44	121	2.739130	no	no	yes	no	no	no	no	yes	no	yes
182186562	chr1	33740283	33740332	24	50	2.083333	no	no	yes	no	no	no	no	yes	yes	yes
182186563	chr1	33743242	33743283	23	42	1.913043	no	no	yes	yes	no	no	no	yes	no	yes
182186567	chr1	33756691	33756755	33	65	1.969697	no	no	yes	no	no	no	no	no	no	yes
182186571	chr1	33763200	33763236	13	37	2.846154	no	no	yes	no	no	no	no	yes	yes	yes
182186572	chr1	33763963	33764004	20	42	2.100000	no	no	yes	no	no	no	no	no	no	yes
182186574	chr1	33768655	33768702	25	48	1.920000	no	no	yes	no	no	no	no	no	no	yes
182186579	chr1	33794742	33795222	34	481	14.147058	no	no	yes	no	no	no	no	no	no	yes
182186584	chr1	33849800	33849835	18	36	2.111111	no	no	yes	no	no	no	no	no	no	yes
182186590	chr1	33882806	33882846	20	41	2.050000	no	no	yes	no	no	no	no	no	no	yes
182186596	chr1	33892117	33892156	20	40	2.050000	no	no	yes	no	no	no	no	no	no	yes
182186602	chr1	33932648	33932682	18	35	1.944444	no	no	yes	no	no	no	no	no	no	yes
182186613	chr1	33952837	33952868	16	32	2.000000	no	no	yes	no	no	no	no	no	no	yes
182186632	chr1	34007207	34007248	20	42	2.100000	no	no	yes	no	no	no	no	no	no	yes
182186633	chr1	34008974	34009010	18	37	2.055556	no	no	yes	no	no	no	no	no	no	yes
182186634	chr1	34009017	34009052	12	36	3.083333	no	no	yes	no	no	no	no	no	no	yes
182186639	chr1	34027841	34027902	18	62	3.555556	no	no	yes	no	no	no	no	no	no	yes
182186640	chr1	34032010	34032049	21	40	1.952381	no	no	yes	no	no	no	no	no	no	yes
182186641	chr1	34034082	34034119	20	38	1.900000	no	no	yes	no	no	no	no	yes	no	yes

182186644	chr1	34044504	34044557	23	54	2.347826	no	no	yes	no	no	no	no	yes	yes	yes
182186646	chr1	34048971	34048999	15	29	1.933333	no	no	yes	no	no	no	no	no	no	yes
182186655	chr1	34061983	34062011	13	29	2.230769	no	no	yes	no	no	no	no	no	no	yes
182186656	chr1	34066361	34066403	17	43	2.529412	no	no	yes	no	no	no	no	no	yes	yes
182186660	chr1	34078283	34078325	17	43	2.647059	no	no	yes	no	no	no	no	no	no	yes
182186672	chr1	34108242	34108305	34	64	1.911765	no	no	yes	no	no	no	no	no	no	yes
182186673	chr1	34108682	34108717	17	36	2.176471	no	no	yes	no	no	no	no	yes	no	yes
182186678	chr1	34122522	34122691	82	170	2.073171	no	no	yes	no	no	no	no	no	no	yes
182186682	chr1	34126776	34126836	19	61	3.200000	no	no	yes	no	no	no	no	yes	no	yes
182186686	chr1	34136931	34136967	17	37	2.235294	no	no	yes	no	no	no	no	yes	no	yes
182186687	chr1	34137640	34137669	10	30	3.000000	no	no	yes	no	no	no	no	no	no	yes
182186693	chr1	34151769	34151814	24	46	1.916667	no	no	yes	no	no	no	no	yes	no	yes
182186695	chr1	34156195	34156232	19	38	2.052632	no	no	yes	no	no	no	no	yes	no	yes
182186696	chr1	34159074	34159110	18	37	2.055556	no	no	yes	no	no	no	no	no	no	yes
182186706	chr1	34189121	34189171	14	51	3.714286	no	no	yes	no	no	no	no	yes	yes	yes
182186712	chr1	34189530	34189567	20	38	1.950000	no	no	yes	no	no	no	no	no	no	yes
182186714	chr1	34196377	34196403	12	27	2.250000	no	no	yes	no	no	no	no	no	no	yes
182186721	chr1	34204450	34204511	32	62	1.968750	no	no	yes	no	no	no	no	no	no	yes
182186737	chr1	34247417	34247621	20	205	10.750000	no	no	no	no	no	no	no	no	no	no
182186740	chr1	34266316	34266346	15	31	2.066667	no	no	no	no	no	no	no	yes	yes	no
182186743	chr1	34274458	34274509	24	52	2.166667	no	no	no	no	no	no	no	no	no	no
182186746	chr1	34275186	34275502	69	317	4.608696	no	no	no	no	no	no	no	yes	no	no
182186747	chr1	34283768	34283809	20	42	2.100000	no	no	no	no	no	no	no	no	no	no
182186772	chr1	34344228	34344263	13	36	3.000000	no	no	no	no	no	no	no	no	no	no
182186776	chr1	34363676	34363702	14	27	1.928571	no	no	no	no	no	no	no	no	yes	no

182186778	chr1	34372365	34372400	16	36	2.312500	no	no	no	no	no	no	no	no	no	no
182186784	chr1	34388635	34388729	37	95	2.538461	no	no	no	no	no	no	no	no	no	no
182186794	chr1	34416553	34416587	16	35	2.187500	no	no	no	no	no	no	no	no	no	no
182186798	chr1	34430448	34430472	13	25	1.923077	no	no	no	no	no	no	no	no	no	no
182186799	chr1	34433438	34433475	16	38	2.375000	no	no	no	no	no	no	no	yes	no	no
182186810	chr1	34480090	34480132	21	43	2.095238	no	no	no	no	no	no	no	yes	no	no
182186811	chr1	34480389	34480423	17	35	2.117647	no	no	no	no	no	no	no	no	no	no
182186813	chr1	34483023	34483063	10	41	4.100000	no	no	no	no	no	no	no	no	no	no
182186817	chr1	34499098	34499144	20	47	2.350000	no	no	no	no	no	no	no	no	no	no
182186820	chr1	34506021	34506203	62	183	2.951613	no	no	no	no	no	no	no	no	no	no
182186849	chr1	34609531	34609577	17	47	2.777778	no	no	no	no	no	no	no	no	no	no
182186852	chr1	34614534	34614565	15	32	2.133333	no	no	no	no	no	no	no	no	no	no
182186863	chr1	34652757	34652792	15	36	2.466667	no	no	yes	no	no	no	no	no	no	yes
182186874	chr1	34719366	34719411	24	46	1.916667	no	no	yes	no	no	no	no	no	no	yes
182186885	chr1	34741493	34741757	132	265	2.007576	no	no	yes	no	no	no	no	no	no	yes
182186886	chr1	34741803	34741837	9	35	3.888889	no	no	yes	no	no	no	no	no	no	yes
182186887	chr1	34745370	34745400	14	31	2.214286	no	no	yes	no	no	no	no	no	no	yes
182186888	chr1	34748989	34749020	12	32	2.666667	no	no	yes	no	no	no	no	no	no	yes
182186891	chr1	34752742	34752768	13	27	2.076923	no	no	yes	no	no	no	no	yes	no	yes
182186895	chr1	34777608	34777985	62	378	6.096774	no	no	yes	no	no	no	no	yes	no	yes
182186896	chr1	34785862	34785893	16	32	2.062500	no	yes	yes	no	no	no	no	yes	no	yes
182186910	chr1	34827997	34828029	16	33	2.062500	no	no	yes	no	no	no	no	no	yes	yes
182186911	chr1	34830001	34830072	35	72	2.057143	no	no	yes	no	no	no	no	no	no	yes
182186913	chr1	34832491	34832538	23	48	2.130435	no	no	yes	no	no	no	no	yes	no	yes
182186922	chr1	34867421	34867499	41	79	1.951220	no	no	yes	no	no	no	no	yes	no	yes

182186939	chr1	34914437	34914480	17	44	2.588235	no	no	yes	no	no	no	no	no	no	yes
182186941	chr1	34928963	34928997	14	35	2.571429	no	no	yes	no	no	no	no	yes	yes	yes
182186969	chr1	34991192	34991240	24	49	2.041667	no	no	yes	no	no	no	no	no	no	yes
182186970	chr1	34992600	34992901	27	302	11.214286	no	no	yes	no	no	no	no	no	no	yes
182186973	chr1	34994994	34995092	31	99	3.156250	no	no	yes	no	no	no	no	no	no	yes
182186976	chr1	35001459	35001497	16	39	2.500000	no	no	yes	no	no	no	no	no	no	yes
182187028	chr1	35099185	35099222	19	38	2.000000	no	no	yes	no	no	no	no	no	no	yes
182187073	chr1	35184233	35184270	11	38	3.454545	no	yes	yes	no	no	no	no	yes	no	yes
182187081	chr1	35192781	35192843	30	63	2.100000	no	no	no	yes	no	no	no	yes	yes	yes
182187098	chr1	35206451	35206513	22	63	2.863636	no	no	no	no	no	no	no	no	no	no
182187101	chr1	35207654	35207701	18	48	2.666667	no	no	no	no	no	no	no	no	no	no
182187124	chr1	35219286	35219326	18	41	2.388889	no	no	no	no	no	no	no	no	no	no
182187166	chr1	35260188	35260590	35	403	11.657143	no	no	no	no	no	no	no	no	no	no
182187174	chr1	35269381	35269415	11	35	3.181818	no	no	yes	no	no	no	no	yes	yes	yes
182187199	chr1	35290340	35290368	15	29	1.933333	no	no	yes	no	no	no	no	no	no	yes
182187208	chr1	35307474	35307518	21	45	2.142857	no	no	yes	no	no	no	no	no	no	yes
182187215	chr1	35312633	35312710	24	78	3.269231	no	no	yes	no	no	no	no	no	no	yes
182187218	chr1	35313797	35313840	20	44	2.200000	no	no	yes	no	no	no	no	no	no	yes
182187232	chr1	35349912	35349982	36	71	2.000000	yes	no	yes	no	no	no	no	no	no	yes
182187240	chr1	35355747	35355814	34	68	2.000000	no	no	yes	no	no	no	no	yes	no	yes
182187248	chr1	35374889	35374921	17	33	2.000000	no	no	yes	no	no	no	no	yes	yes	yes
182187249	chr1	35377264	35377297	16	34	2.125000	no	no	yes	no	no	no	no	no	no	yes
182187257	chr1	35382214	35382279	28	66	2.357143	no	no	yes	no	no	no	no	no	no	yes
182187260	chr1	35382401	35382443	22	43	2.000000	no	no	yes	no	no	no	no	no	no	yes
182187264	chr1	35391657	35391683	12	27	2.250000	yes	no	yes	no	no	no	no	yes	no	yes

182187277	chr1	35436655	35436700	19	46	2.421053	no	no	yes	no	no	no	no	no	no	yes
182187279	chr1	35437304	35437339	18	36	2.166667	no	no	yes	no	no	no	no	yes	no	yes
182187282	chr1	35451090	35451119	14	30	2.142857	no	no	yes	no	no	yes	no	yes	no	yes
182187301	chr1	35498515	35498567	28	53	1.928571	no	no	yes	no	no	no	no	no	no	yes
182187302	chr1	35499753	35499793	18	41	2.277778	no	no	yes	no	no	no	no	yes	no	yes
182187324	chr1	35547010	35547111	50	102	2.100000	no	no	yes	no	no	no	no	yes	no	yes
182187325	chr1	35547212	35547263	28	52	1.928571	no	no	yes	no	no	no	no	no	no	yes
182187326	chr1	35548403	35548458	27	56	2.148148	no	no	yes	no	no	no	no	no	no	yes
182187328	chr1	35557975	35558030	21	56	2.666667	yes	no	yes	no	yes	no	yes	yes	no	yes
182187330	chr1	35574047	35574076	15	30	2.000000	yes	no	yes	yes	no	no	yes	yes	yes	yes
182187331	chr1	35574201	35574242	20	42	2.150000	yes	no	yes	yes	no	no	yes	yes	yes	yes
182187333	chr1	35576187	35576239	22	53	2.545455	no	no	yes	no	no	no	no	yes	yes	yes
182187334	chr1	35576857	35576931	37	75	2.027027	yes	no	yes	no	no	no	yes	yes	yes	yes
182187335	chr1	35576929	35577052	17	124	7.294117	yes	no	yes	no	no	no	yes	yes	no	yes
182187340	chr1	35586653	35586703	17	51	3.000000	no	no	yes	no	no	no	no	no	no	yes
182187344	chr1	35589418	35589459	22	42	1.909091	no	no	yes	no	no	no	no	yes	no	yes
182187345	chr1	35590294	35590323	14	30	2.142857	no	no	yes	no	no	no	no	yes	no	yes
182187347	chr1	35590849	35591061	43	213	5.000000	no	no	yes	no	no	no	no	yes	yes	yes
182187352	chr1	35603658	35603690	15	33	2.266667	no	no	yes	no	no	no	no	yes	yes	yes
182187361	chr1	35640560	35640778	109	219	2.009174	no	no	yes	no	no	no	no	yes	yes	yes
182187395	chr1	35691489	35691566	35	78	2.228571	no	no	no	no	no	no	no	no	yes	no
182187403	chr1	35717921	35717952	16	32	2.000000	no	no	yes	no	no	no	no	yes	yes	yes
182187414	chr1	35738354	35738412	27	59	2.185185	yes	no	yes	no	no	no	no	no	no	yes
182187415	chr1	35739415	35739468	24	54	2.250000	no	yes	no	yes	no	no	no	no	no	yes
182187423	chr1	35754146	35754175	15	30	2.000000	no	no	yes	no	no	no	no	yes	no	yes

182187425	chr1	35756534	35756566	17	33	1.941176	no	no	yes	no	no	no	no	yes	no	yes
182187427	chr1	35766376	35766436	28	61	2.178571	no	no	yes	no	no	no	no	no	no	yes
182187444	chr1	35790846	35790891	24	46	1.916667	no	no	no	no	no	no	no	yes	no	no
182187446	chr1	35792150	35792183	13	34	2.615385	no	no	no	no	no	no	no	no	no	no
182187461	chr1	35803061	35803088	13	28	2.153846	no	no	no	no	no	no	no	no	no	no
182187465	chr1	35808460	35808484	7	25	3.571429	no	no	yes	no	no	no	yes	yes	yes	yes
182187474	chr1	35821712	35821752	19	41	2.157895	no	no	yes	no	no	no	no	no	no	yes
182187475	chr1	35827705	35827748	15	44	3.000000	no	no	yes	no	no	no	no	no	no	yes
182187480	chr1	35833793	35833821	12	29	2.416667	no	no	yes	no	no	no	no	yes	no	yes
182187487	chr1	35843431	35843495	20	65	3.350000	no	no	yes	no	no	no	no	no	no	yes
182187488	chr1	35843833	35843865	12	33	2.833333	no	no	yes	no	no	no	no	no	no	yes
182187496	chr1	35853412	35853478	31	67	2.218750	yes	no	yes	no	no	no	no	no	no	yes
182187509	chr1	35880036	35880074	11	39	3.500000	no	no	yes	no	no	no	no	yes	no	yes
182187510	chr1	35880224	35880283	28	60	2.142857	no	no	yes	no	no	no	no	yes	no	yes
182187511	chr1	35888993	35889128	44	136	3.090909	no	no	yes	no	no	no	no	yes	yes	yes
182187513	chr1	35891475	35891503	14	29	2.071429	no	no	yes	no	no	no	no	yes	no	yes
182187514	chr1	35907404	35907442	20	39	1.950000	no	no	yes	no	no	no	no	no	no	yes
182187517	chr1	35932884	35932926	23	43	1.913043	no	no	yes	no	no	no	no	yes	no	yes
182187529	chr1	35963366	35963392	14	27	1.928571	no	no	yes	no	no	no	no	yes	no	yes
182187530	chr1	35963673	35963714	22	42	2.000000	no	no	yes	no	no	no	no	no	no	yes
182187532	chr1	35966356	35966384	15	29	1.933333	yes	no	yes	no	no	no	no	no	no	yes
182187541	chr1	35971029	35971170	79	142	1.860759	no	no	yes	no	no	no	no	no	no	yes
182187548	chr1	35990686	35990738	17	53	3.117647	no	no	yes	no	no	no	no	no	no	yes
182187584	chr1	36059253	36059286	18	34	1.944444	no	yes	no	no	no	no	no	no	no	yes
182187585	chr1	36064829	36064871	23	43	1.956522	no	yes	no	no	no	no	no	no	no	yes

182187586	chr1	36066598	36066642	11	45	3.923077	no	yes	no	no	no	no	no	no	no	yes
182187588	chr1	36072081	36072155	37	75	2.027027	no	yes	no	no	no	no	no	no	no	yes
182187591	chr1	36077343	36077396	23	54	2.478261	no	no	no	no	no	no	no	no	no	no
182187600	chr1	36086375	36086510	50	136	2.705882	yes	no	yes	no	no	no	no	no	no	yes
182187602	chr1	36088904	36088933	15	30	2.000000	no	no	no	yes	no	yes	yes	yes	yes	yes
182187606	chr1	36099082	36099123	19	42	2.263158	no	no	no	yes	no	no	no	yes	yes	yes
182187611	chr1	36106930	36106970	15	41	2.733333	no	no	yes	no	no	no	no	no	no	yes
182187614	chr1	36113246	36113298	25	53	2.160000	no	no	yes	no	no	no	no	no	no	yes
182187628	chr1	36151997	36152035	19	39	2.052632	no	no	yes	no	no	no	no	yes	yes	yes
182187629	chr1	36157022	36157057	16	36	2.375000	yes	no	yes	no	no	no	no	yes	yes	yes
182187635	chr1	36168605	36168630	9	26	2.888889	no	no	yes	no	no	no	no	yes	no	yes
182187670	chr1	36217361	36217397	17	37	2.176471	no	no	no	no	no	no	no	yes	yes	no
182187682	chr1	36231032	36231082	26	51	2.000000	no	no	yes	no	no	no	no	no	yes	yes
182187706	chr1	36283617	36283720	52	104	2.000000	no	no	yes	no	no	no	no	yes	yes	yes
182187720	chr1	36319747	36319790	19	44	2.421053	yes	yes	no	yes	no	no	no	yes	no	yes
182187721	chr1	36319788	36319960	90	173	1.922222	yes	yes	no	yes	no	no	no	yes	no	yes
182187725	chr1	36320497	36320605	54	109	2.018518	yes	yes	no	yes	no	no	no	yes	yes	yes
182187726	chr1	36320845	36320902	16	58	3.812500	no	no	yes	no	no	yes	yes	yes	yes	yes
182187730	chr1	36339088	36339123	13	36	2.769231	no	no	no	no	no	yes	no	yes	yes	no
182187750	chr1	36403641	36403748	53	108	2.075472	yes	no	no	no	no	no	no	yes	no	no
182187761	chr1	36412593	36412629	9	37	4.111111	no	no	no	no	no	no	no	yes	no	no
182187769	chr1	36443793	36443834	23	42	1.913043	no	no	yes	no	no	no	no	yes	no	yes
182187774	chr1	36447444	36447556	61	113	1.901639	no	no	yes	no	no	no	no	yes	no	yes
182187775	chr1	36449061	36449105	21	45	2.142857	no	no	yes	no	no	no	no	yes	no	yes
182187789	chr1	36493556	36493609	25	54	2.240000	no	no	no	no	no	no	no	yes	no	no

182187803	chr1	36514709	36514849	40	141	3.500000	no	no	no	no	no	no	no	yes	yes	no
182187807	chr1	36521427	36522161	40	735	18.400000	no	no	no	no	no	no	no	yes	no	no
182187809	chr1	36524493	36524553	25	61	2.520000	no	no	no	no	no	no	no	yes	no	no
182187813	chr1	36531581	36531670	35	90	2.666667	no	no	no	no	no	no	no	no	no	no
182187816	chr1	36543884	36543960	30	77	2.666667	no	no	no	no	no	no	no	no	no	no
182187817	chr1	36543953	36544370	34	418	12.222222	no	no	no	no	no	no	no	no	no	no
182187819	chr1	36544628	36545156	26	529	20.586206	no	no	no	no	no	no	no	no	no	no
182187826	chr1	36562179	36562244	31	66	2.161290	no	no	no	no	no	no	no	no	no	no
182187830	chr1	36568427	36568485	23	59	2.565217	no	no	no	no	no	no	no	yes	no	no
182187831	chr1	36568619	36568658	17	40	2.352941	no	no	no	no	no	no	no	yes	no	no
182187834	chr1	36569224	36569296	37	73	2.000000	no	no	no	no	no	no	no	yes	no	no
182187836	chr1	36571095	36572198	62	1104	17.652174	no	no	no	no	no	no	no	yes	yes	no
182187847	chr1	36604400	36604456	30	57	1.900000	no	no	no	no	no	no	no	no	no	no
182187855	chr1	36613684	36613809	50	126	2.520000	no	no	no	no	no	no	no	yes	no	no
182187859	chr1	36613984	36614030	20	47	2.350000	no	no	no	no	no	no	no	yes	no	no
182187865	chr1	36616389	36616454	31	66	2.193548	no	no	no	no	no	no	no	no	no	no
182187867	chr1	36617391	36617450	31	60	1.935484	no	no	no	no	no	no	no	yes	no	no
182187868	chr1	36619637	36619669	15	33	2.200000	no	no	no	no	no	no	no	no	no	no
182187870	chr1	36620268	36620301	14	34	2.428571	no	no	no	no	no	no	no	yes	no	no
182187881	chr1	36673496	36673682	37	187	5.054054	no	no	no	no	no	no	no	no	yes	no
182187889	chr1	36688289	36688352	34	64	1.911765	no	no	no	no	no	no	no	yes	no	no
182187893	chr1	36688479	36689072	18	594	33.444443	no	no	no	no	no	no	no	yes	no	no
182187906	chr1	36700599	36700667	32	69	2.218750	no	no	no	no	no	no	no	no	no	no
182187909	chr1	36712064	36712104	15	41	2.687500	no	no	no	no	no	no	no	no	no	no
182187911	chr1	36715911	36716044	66	134	2.030303	no	no	no	no	no	no	no	no	yes	no

182187914	chr1	36740599	36740775	80	177	2.209877	no	no	no	no	no	no	no	no	yes	no
182187916	chr1	36743799	36743995	98	197	2.010204	no	no	no	no	no	no	no	yes	yes	no
182187918	chr1	36752523	36752547	12	25	2.083333	no	no	no	no	no	no	no	no	no	no
182187919	chr1	36754895	36754927	17	33	1.941176	no	no	no	no	no	no	no	yes	yes	no
182187920	chr1	36756021	36756049	10	29	2.900000	no	no	no	no	no	no	no	no	no	no
182187933	chr1	36769290	36769393	36	104	2.888889	yes	no	no	no	yes	no	no	no	no	yes
182187934	chr1	36776752	36776784	17	33	1.941176	no	no	no	no	no	yes	no	no	no	no
182187936	chr1	36778926	36778957	12	32	2.666667	no	no	no	no	no	no	no	yes	yes	no
182187938	chr1	36778966	36779019	22	54	2.500000	no	no	no	no	no	no	no	yes	yes	no
182187940	chr1	36792808	36792851	16	44	2.750000	no	no	no	no	no	no	no	no	no	no
182187941	chr1	36792937	36793011	38	75	1.973684	no	no	no	no	no	no	no	yes	no	no
182187952	chr1	36809329	36809383	20	55	2.750000	no	no	yes	no	no	no	no	no	no	yes
182187954	chr1	36810197	36810251	19	55	2.947368	no	no	yes	no	no	no	no	no	no	yes
182187960	chr1	36814974	36815009	16	36	2.250000	no	no	yes	no	no	no	no	yes	no	yes
182187963	chr1	36823782	36823825	22	44	2.000000	no	no	yes	no	no	no	no	yes	no	yes
182187968	chr1	36848043	36848084	21	42	2.000000	no	no	yes	no	no	no	no	yes	yes	yes
182187974	chr1	36879120	36879155	18	36	2.000000	no	no	yes	no	no	no	no	no	no	yes
182187975	chr1	36882976	36883014	21	39	1.952381	no	no	yes	no	no	no	no	yes	yes	yes
182187976	chr1	36883771	36883796	10	26	2.600000	no	no	yes	no	no	no	no	no	no	yes
182187991	chr1	36929365	36929389	11	25	2.272727	no	no	yes	no	no	no	no	no	no	yes
182187992	chr1	36942625	36942780	56	156	2.785714	no	no	yes	no	no	no	no	no	yes	yes
182187993	chr1	36944472	36944515	18	44	2.500000	no	no	yes	no	no	no	no	no	no	yes
182187994	chr1	36948785	36948809	8	25	3.125000	no	no	yes	no	no	no	no	yes	no	yes
182187996	chr1	36950080	36950116	13	37	3.000000	no	no	yes	no	no	no	no	no	no	yes
182187997	chr1	36950904	36950943	20	40	2.000000	no	no	yes	no	no	no	no	yes	no	yes

182188002	chr1	36958974	36959017	23	44	1.913043	no	no	yes	no	no	no	no	no	no	yes
182188008	chr1	36987195	36987339	46	145	3.152174	no	no	yes	no	no	no	no	no	no	yes
182188023	chr1	37034284	37034320	16	37	2.375000	yes	no	no	no	yes	no	yes	yes	yes	yes
182188026	chr1	37034579	37034612	16	34	2.125000	yes	no	no	no	no	no	yes	yes	yes	no
182188028	chr1	37039730	37039770	20	41	2.050000	no	no	no	no	no	no	no	yes	no	no
182188032	chr1	37048687	37048743	28	57	2.035714	no	no	no	no	no	no	no	no	no	no
182188037	chr1	37062601	37062691	17	91	5.411765	no	no	no	no	no	no	no	no	no	no
182188056	chr1	37105706	37105737	16	32	2.000000	no	no	no	no	no	no	no	no	no	no
182188058	chr1	37118933	37118970	18	38	2.222222	no	no	no	no	no	no	no	no	no	no
182188061	chr1	37128258	37128352	31	95	3.064516	no	no	no	no	no	no	no	yes	yes	no
182188062	chr1	37131180	37131223	16	44	2.750000	no	no	no	no	no	no	no	no	no	no
182188067	chr1	37132675	37132721	25	47	1.960000	yes	no	no	no	no	no	no	no	no	no
182188068	chr1	37136492	37136536	16	45	2.812500	no	no	no	no	no	yes	no	yes	no	no
182188074	chr1	37150317	37150357	17	41	2.411765	no	no	no	no	no	no	no	no	no	no
182188076	chr1	37155225	37155253	15	29	1.933333	no	no	no	no	yes	no	no	no	no	yes
182188080	chr1	37168207	37168284	26	78	2.928571	no	no	yes	no	no	no	no	no	no	yes
182188082	chr1	37176519	37176564	16	46	2.875000	no	no	yes	no	no	no	no	no	yes	yes
182188091	chr1	37217015	37217046	14	32	2.285714	no	no	yes	no	no	no	no	yes	no	yes
182188099	chr1	37256194	37256233	20	40	2.050000	no	no	yes	no	no	no	no	no	yes	yes
182188104	chr1	37273787	37273824	19	38	2.052632	no	no	yes	no	no	no	no	yes	yes	yes
182188110	chr1	37288382	37288452	30	71	2.366667	no	no	yes	no	no	no	no	no	no	yes
182188115	chr1	37307350	37307416	26	67	2.615385	no	no	yes	no	no	no	no	yes	no	yes
182188116	chr1	37309132	37309260	65	129	1.984615	no	no	yes	no	no	no	no	yes	no	yes
182188118	chr1	37311130	37311193	28	64	2.285714	no	no	yes	no	no	no	no	no	no	yes
182188119	chr1	37311743	37311788	24	46	2.041667	no	no	yes	no	no	no	no	no	yes	yes

182188127	chr1	37319074	37319356	106	283	2.747663	yes	no	yes	no	no	no	no	yes	yes	yes
182188134	chr1	37319316	37319576	144	261	1.812500	yes	no	yes	no	no	no	no	yes	yes	yes
182188137	chr1	37323186	37323296	18	111	6.277778	no	no	yes	no	no	no	no	no	no	yes
182188140	chr1	37332906	37332947	22	42	2.000000	no	no	no	no	no	no	no	no	no	no
182188145	chr1	37352951	37352996	19	46	2.421053	no	no	yes	no	no	no	no	yes	no	yes
182188156	chr1	37388035	37388092	19	58	3.052632	no	no	yes	no	no	no	no	yes	no	yes
182188157	chr1	37391745	37391778	12	34	2.769231	no	no	yes	no	no	no	no	yes	no	yes
182188170	chr1	37426483	37426526	20	44	2.200000	no	no	yes	no	no	no	no	no	no	yes
182188184	chr1	37434806	37434854	23	49	2.173913	no	no	yes	no	no	no	no	yes	yes	yes
182188213	chr1	37474706	37474760	25	55	2.200000	yes	no	yes	no	no	no	no	yes	no	yes
182188214	chr1	37491122	37491163	22	42	1.909091	no	no	no	no	no	no	no	no	no	no
182188225	chr1	37502107	37502162	19	56	2.947368	no	no	yes	no	no	no	no	yes	no	yes
182188226	chr1	37502440	37502484	23	45	2.000000	no	no	yes	no	no	no	no	yes	yes	yes
182188251	chr1	37555009	37555041	16	33	2.062500	yes	no	no	no	no	no	no	yes	no	no
182188254	chr1	37583230	37583270	20	41	2.050000	yes	no	yes	no	yes	no	no	yes	no	yes
182188255	chr1	37588631	37588671	19	41	2.157895	no	no	yes	no	no	no	no	no	no	yes
182188270	chr1	37645282	37645345	24	64	2.666667	no	no	no	no	no	no	no	no	no	no
182188274	chr1	37650810	37650855	19	46	2.421053	no	no	no	no	no	no	no	no	no	no
182188281	chr1	37669591	37669857	70	267	3.928571	no	no	no	no	no	no	no	yes	no	no
182188296	chr1	37719669	37719746	34	78	2.294118	no	no	yes	no	no	yes	no	no	no	yes
182188315	chr1	37753639	37753739	16	101	6.312500	yes	no	yes	no	no	no	yes	yes	no	yes
182188337	chr1	37794340	37794380	22	41	1.954545	yes	no	no	yes	no	no	yes	yes	no	yes
182188341	chr1	37806546	37806579	16	34	2.125000	yes	no	yes	no	no	no	no	yes	yes	yes
182188362	chr1	37870013	37870106	46	94	2.043478	no	no	yes	no	yes	no	no	no	no	yes
182188419	chr1	37932941	37932977	14	37	2.642857	no	no	yes	no	no	no	no	yes	yes	yes

182188429	chr1	37940324	37940348	13	25	1.923077	no	no	yes	no	no	yes	no	yes	yes	yes
182188454	chr1	38022087	38022115	15	29	1.933333	no	no	yes	no	no	no	no	yes	no	yes
182188457	chr1	38029586	38029616	16	31	2.000000	no	no	no	no	no	no	no	yes	yes	no
182188459	chr1	38033004	38033045	17	42	2.470588	no	no	no	no	no	no	no	no	no	no
182188464	chr1	38036319	38036369	16	51	3.176471	no	no	no	no	no	no	no	no	no	no
182188467	chr1	38041857	38041894	16	38	2.437500	no	no	no	no	no	no	no	yes	no	no
182188468	chr1	38044583	38044623	20	41	2.050000	no	yes	no	no	no	no	yes	yes	yes	yes
182188470	chr1	38046209	38046261	26	53	2.076923	no	no	no	yes	no	no	yes	yes	no	yes
182188475	chr1	38053090	38053137	24	48	2.000000	no	no	yes	no	no	no	no	yes	yes	yes
182188476	chr1	38054322	38054364	23	43	1.956522	no	no	yes	no	no	no	no	no	no	yes
182188479	chr1	38060032	38060080	20	49	2.450000	no	no	yes	no	no	no	no	yes	yes	yes
182188488	chr1	38081057	38081115	20	59	2.950000	yes	no	yes	no	no	no	no	yes	no	yes
182188492	chr1	38093672	38093713	22	42	1.909091	no	no	yes	no	no	no	no	yes	no	yes
182188495	chr1	38100280	38100324	22	45	2.045455	no	no	yes	no	no	no	no	no	yes	yes
182188501	chr1	38115624	38115655	12	32	2.666667	no	no	yes	no	no	no	no	yes	no	yes
182188502	chr1	38118595	38118631	18	37	2.166667	no	no	yes	no	no	no	no	no	no	yes
182188503	chr1	38133615	38133640	13	26	2.000000	no	no	yes	no	no	no	no	no	no	yes
182188505	chr1	38134169	38134271	22	103	4.772727	no	no	yes	no	no	no	no	yes	no	yes
182188508	chr1	38137378	38137425	16	48	3.000000	no	no	yes	no	no	no	no	yes	yes	yes
182188510	chr1	38140398	38140429	16	32	2.000000	no	no	yes	no	no	no	no	yes	no	yes
182188514	chr1	38160736	38160769	11	34	3.090909	no	no	yes	no	no	no	no	no	no	yes
182188521	chr1	38168910	38168949	14	40	2.866667	no	no	no	no	no	no	no	no	no	no
182188538	chr1	38249100	38249129	14	30	2.142857	no	no	no	no	no	no	no	no	no	no
182188544	chr1	38260517	38260572	29	56	1.965517	no	no	no	no	no	no	no	yes	no	no
182188549	chr1	38267727	38267755	10	29	2.900000	no	no	no	no	no	no	no	yes	yes	no

182188553	chr1	38272690	38272735	22	46	2.090909	no	no	no	no	no	no	no	yes	yes	no
182188558	chr1	38301724	38301774	20	51	2.550000	no	no	no	no	no	no	no	no	no	no
182188576	chr1	38342506	38342582	24	77	3.208333	no	no	no	no	no	no	no	no	no	no
182188578	chr1	38345964	38346008	24	45	1.916667	no	no	no	no	no	no	no	no	no	no
182188587	chr1	38374414	38374460	12	47	3.916667	no	no	no	no	no	no	no	no	no	no
182188593	chr1	38416234	38416265	17	32	1.941176	no	no	no	no	no	no	no	yes	no	no
182188599	chr1	38453680	38453836	79	157	1.987342	no	no	no	no	no	no	no	no	no	no
182188608	chr1	38471190	38471222	16	33	2.062500	no	no	no	no	no	no	no	no	no	no
182188610	chr1	38480277	38480328	24	52	2.166667	no	no	yes	no	no	no	no	no	no	yes
182188617	chr1	38497480	38497522	17	43	2.529412	no	no	yes	no	no	no	no	yes	no	yes
182188627	chr1	38529995	38530651	29	657	22.827587	no	no	no	no	no	no	no	yes	yes	no
182188634	chr1	38538755	38538816	25	62	2.500000	no	no	no	no	no	no	no	no	no	no
182188645	chr1	38559816	38559868	25	53	2.120000	no	no	no	no	no	no	no	yes	no	no
182188647	chr1	38565976	38566028	25	53	2.200000	no	no	no	no	no	no	no	no	no	no
182188651	chr1	38584995	38585030	16	36	2.250000	no	no	no	no	no	no	no	yes	no	no
182188659	chr1	38599731	38599763	17	33	1.941176	no	no	no	no	no	no	no	no	no	no
182188668	chr1	38616272	38616304	17	33	1.941176	no	no	no	no	no	no	no	yes	no	no
182188672	chr1	38620735	38620793	24	59	2.500000	no	no	no	no	no	no	no	yes	no	no
182188675	chr1	38625902	38625953	24	52	2.291667	no	no	no	no	no	no	no	no	no	no
182188679	chr1	38628113	38628154	21	42	2.000000	no	no	no	no	no	no	no	no	no	no
182188691	chr1	38655052	38655104	27	53	1.962963	no	no	no	no	no	no	no	yes	no	no
182188692	chr1	38667278	38667324	24	47	2.000000	no	no	no	no	no	no	no	no	no	no
182188695	chr1	38686902	38686926	13	25	1.923077	no	no	no	no	no	no	no	no	no	no
182188698	chr1	38696268	38696390	56	123	2.196429	no	no	no	no	no	no	no	no	no	no
182188704	chr1	38720979	38721025	14	47	3.533333	no	no	no	no	no	no	no	yes	no	no

182188729	chr1	38819274	38819303	11	30	2.727273	no	no	no	no	no	no	no	yes	yes	no
182188737	chr1	38849365	38849405	19	41	2.157895	no	no	yes	no	no	no	no	yes	no	yes
182188780	chr1	38919160	38919200	21	41	2.000000	no	yes	yes	yes	no	no	no	yes	yes	yes
182188781	chr1	38921468	38921500	17	33	2.000000	no	no	yes	no	no	no	no	no	no	yes
182188783	chr1	38927027	38927061	16	35	2.187500	yes	no	yes	no	no	yes	no	no	no	yes
182188810	chr1	38971440	38971468	14	29	2.071429	no	no	no	no	no	no	no	yes	no	no
182188863	chr1	39043690	39043755	32	66	2.125000	no	no	no	no	no	no	no	yes	yes	no
182188867	chr1	39044247	39044279	10	33	3.300000	no	no	no	no	no	no	no	no	no	no
182188901	chr1	39077114	39077158	19	45	2.350000	no	no	no	no	no	no	no	no	yes	no
182188909	chr1	39084246	39084310	18	65	3.611111	no	no	no	yes	no	no	no	yes	no	yes
182188920	chr1	39103361	39103398	19	38	2.000000	no	yes	yes	no	no	yes	no	yes	yes	yes
182188932	chr1	39133507	39133561	22	55	2.500000	no	no	yes	no	no	no	no	yes	no	yes
182188934	chr1	39136028	39136075	24	48	2.000000	no	no	yes	no	no	no	no	no	no	yes
182188947	chr1	39149100	39149158	23	59	2.565217	no	no	yes	no	no	no	no	no	yes	yes
182188953	chr1	39158661	39158685	12	25	2.083333	yes	no	yes	no	no	no	no	yes	yes	yes
182188954	chr1	39159507	39159538	16	32	2.062500	no	no	yes	no	no	no	no	yes	yes	yes
182188957	chr1	39166355	39166434	40	80	2.000000	no	no	yes	no	no	no	no	yes	no	yes
182188964	chr1	39176674	39176705	16	32	2.000000	no	no	yes	no	no	no	no	no	yes	yes
182188965	chr1	39178018	39178057	20	40	2.000000	no	no	yes	no	no	no	no	yes	yes	yes
182188977	chr1	39190333	39190369	9	37	4.200000	no	no	yes	no	no	no	no	yes	no	yes
182188989	chr1	39210909	39210986	33	78	2.363636	no	no	yes	no	no	no	no	yes	no	yes
182188990	chr1	39214610	39214638	15	29	1.933333	yes	no	yes	no	no	no	no	yes	no	yes
182188993	chr1	39221648	39221674	12	27	2.250000	no	no	yes	no	no	no	no	yes	yes	yes
182189022	chr1	39270094	39270124	13	31	2.384615	no	no	yes	no	no	no	no	yes	yes	yes
182189023	chr1	39274199	39274300	35	102	2.921053	no	no	yes	no	no	no	no	no	no	yes

182189025	chr1	39276354	39276430	38	77	2.052632	no	no	yes	no	no	no	no	yes	yes	yes
182189026	chr1	39280218	39280255	19	38	2.000000	no	no	yes	no	no	no	no	no	no	yes
182189030	chr1	39294709	39294737	15	29	1.933333	no	no	yes	no	no	no	no	no	yes	yes
182189035	chr1	39296731	39296866	42	136	3.333333	no	no	yes	no	no	no	no	no	no	yes
182189039	chr1	39302803	39302837	17	35	2.117647	no	no	yes	no	no	no	no	no	no	yes
182189081	chr1	39413244	39413879	39	636	16.307692	no	no	yes	yes	no	no	no	yes	yes	yes
182189083	chr1	39413820	39413941	60	122	2.033333	no	no	yes	yes	no	no	no	yes	yes	yes
182189091	chr1	39435817	39435854	18	38	2.166667	no	yes	yes	no	no	no	no	no	no	yes
182189096	chr1	39446238	39446279	9	42	4.888889	no	no	yes	no	no	no	no	no	no	yes
182189097	chr1	39446333	39446366	14	34	2.500000	no	no	yes	no	no	no	no	no	no	yes
182189099	chr1	39451247	39451293	24	47	1.958333	no	no	yes	no	no	no	no	yes	yes	yes
182189107	chr1	39485562	39485598	12	37	3.083333	no	yes	no	yes	no	no	no	yes	yes	yes
182189115	chr1	39522785	39522824	14	40	2.857143	no	no	yes	no	no	no	no	no	yes	yes
182189132	chr1	39561629	39561662	15	34	2.266667	no	yes	yes	no	yes	yes	no	yes	no	yes
182189151	chr1	39595357	39595385	11	29	2.636364	no	no	no	no	no	no	no	no	yes	no
182189175	chr1	39634987	39635019	14	33	2.357143	no	no	yes	no	no	no	no	no	no	yes
182189176	chr1	39639684	39639718	11	35	3.181818	yes	no	no	no	no	no	yes	yes	yes	no
182189181	chr1	39650819	39650866	18	48	2.666667	no	no	no	no	no	no	no	no	no	no
182189184	chr1	39671994	39672025	9	32	3.555556	no	no	no	yes	no	no	yes	yes	yes	yes
182189190	chr1	39680707	39680736	14	30	2.142857	no	yes	no	no	no	no	no	yes	no	yes
182189212	chr1	39707028	39707061	17	34	2.058824	no	no	yes	no	no	no	no	yes	no	yes
182189221	chr1	39740336	39740360	13	25	1.923077	no	no	yes	no	no	no	no	yes	no	yes
182189224	chr1	39744037	39744108	31	72	2.322581	no	no	yes	no	no	no	no	yes	no	yes
182189229	chr1	39788493	39788527	13	35	2.692308	yes	no	no	no	yes	no	yes	yes	no	yes
182189255	chr1	39819456	39819597	20	142	7.100000	no	no	no	no	no	no	no	yes	yes	no

182189262	chr1	39835658	39835705	23	48	2.086957	no	no	no	no	no	no	no	no	yes	no
182189311	chr1	39919680	39919752	27	73	2.703704	no	no	no	no	no	no	no	yes	yes	no
182189320	chr1	39954832	39954883	18	52	2.888889	yes	no	no	no	no	no	yes	yes	no	no
182189327	chr1	39968360	39968400	22	41	1.954545	no	yes	no	yes	no	no	no	yes	yes	yes
182189348	chr1	40005911	40005963	26	53	2.038461	no	no	no	no	no	no	no	no	yes	no
182189376	chr1	40057769	40057805	19	37	1.947368	no	no	yes	no	no	no	no	yes	yes	yes
182189378	chr1	40067592	40067632	21	41	1.952381	no	yes	no	yes	no	yes	no	yes	yes	yes
182189382	chr1	40074346	40074400	23	55	2.391304	no	no	yes	no	no	no	no	yes	no	yes
182189403	chr1	40108655	40108698	22	44	2.000000	no	no	no	no	no	no	no	no	no	no
182189409	chr1	40132579	40132658	30	80	2.666667	yes	no	no	no	no	no	no	no	no	no
182189410	chr1	40132964	40133008	20	45	2.250000	no	yes	no	no	no	no	yes	no	yes	yes
182189413	chr1	40134740	40134866	68	127	1.867647	no	no	no	no	no	no	no	no	no	no
182189415	chr1	40134852	40134904	18	53	2.894737	no	no	no	no	no	no	no	no	no	no
182189426	chr1	40143961	40144092	26	132	5.076923	no	no	no	no	no	no	no	yes	yes	no
182189441	chr1	40178011	40178047	17	37	2.176471	no	no	yes	no	no	yes	no	no	no	yes
182189451	chr1	40200922	40201040	51	119	2.352941	no	no	yes	no	no	no	no	no	yes	yes
182189460	chr1	40226836	40226869	16	34	2.125000	no	no	yes	no	no	no	no	no	yes	yes
182189469	chr1	40257723	40257767	19	45	2.368421	yes	no	no	no	yes	no	yes	yes	yes	yes
182189493	chr1	40311419	40311466	21	48	2.272727	no	no	yes	no	no	yes	no	yes	yes	yes
182189499	chr1	40322337	40322374	12	38	3.076923	no	no	no	no	no	no	no	no	no	no
182189514	chr1	40344378	40344415	13	38	2.923077	yes	no	no	no	no	no	no	yes	yes	no
182189530	chr1	40380123	40380162	21	40	1.904762	no	no	yes	no	no	no	no	yes	yes	yes
182189531	chr1	40387589	40387626	9	38	4.222222	no	no	yes	no	no	no	no	yes	yes	yes
182189537	chr1	40397218	40397244	14	27	1.928571	no	no	yes	no	no	no	no	yes	yes	yes
182189542	chr1	40429715	40429775	24	61	2.541667	no	no	no	no	no	no	no	no	no	no

182189549	chr1	40442266	40442332	35	67	1.914286	no	no	yes	no	no	no	no	no	no	yes
182189570	chr1	40488887	40488923	17	37	2.176471	yes	no	yes	no	no	no	no	no	no	yes
182189571	chr1	40488994	40489028	18	35	2.000000	yes	no	yes	no	no	no	no	no	no	yes
182189580	chr1	40494577	40494743	66	167	2.521739	no	no	yes	no	no	no	no	no	no	yes
182189598	chr1	40542913	40542950	20	38	1.900000	no	no	yes	no	no	no	no	yes	no	yes
182189602	chr1	40553046	40553076	15	31	2.066667	no	no	no	no	no	no	no	no	no	no
182189657	chr1	40649555	40649613	29	59	2.034483	no	no	yes	no	no	no	no	yes	no	yes
182189674	chr1	40698763	40698815	28	53	1.928571	no	no	yes	no	no	no	no	yes	no	yes
182189688	chr1	40749762	40749827	23	66	2.869565	no	no	yes	no	no	yes	no	yes	no	yes
182189689	chr1	40753890	40753922	17	33	1.941176	yes	no	yes	no	no	yes	no	yes	yes	yes
182189695	chr1	40763102	40763317	32	216	6.750000	no	no	yes	no	no	yes	no	yes	no	yes
182189698	chr1	40783752	40783785	16	34	2.187500	yes	no	no	no	no	no	yes	yes	yes	no
182189707	chr1	40817362	40817553	47	192	4.085106	no	no	yes	no	no	no	no	no	no	yes
182189708	chr1	40835385	40835411	14	27	1.928571	no	no	yes	no	no	no	no	no	yes	yes
182189714	chr1	40857068	40857128	27	61	2.259259	no	no	no	no	no	no	no	yes	no	no
182189741	chr1	40896839	40896870	10	32	3.200000	no	no	no	no	no	no	no	yes	no	no
182189753	chr1	40920549	40920612	31	64	2.064516	no	no	no	no	no	no	no	yes	no	no
182189784	chr1	40979621	40979679	26	59	2.296296	yes	no	no	no	no	no	yes	yes	yes	no
182189786	chr1	40986562	40986611	25	50	2.000000	no	no	yes	no	no	no	no	yes	yes	yes
182189788	chr1	40997624	40997655	15	32	2.133333	no	no	yes	no	no	no	no	no	yes	yes
182189791	chr1	41000871	41000930	26	60	2.307692	yes	yes	yes	no	no	no	no	no	no	yes
182189792	chr1	41014052	41014080	14	29	2.142857	yes	no	no	no	no	no	no	yes	yes	no
182189793	chr1	41014068	41014106	20	39	2.000000	yes	no	no	no	no	no	no	yes	yes	no
182189801	chr1	41044431	41044477	24	47	1.958333	no	no	yes	no	no	yes	no	yes	yes	yes
182189811	chr1	41073873	41073918	23	46	2.000000	no	no	yes	no	no	no	no	no	no	yes

182189815	chr1	41085152	41085180	15	29	1.933333	no	no	yes	no	no	no	no	no	no	yes
182189821	chr1	41116720	41116764	22	45	2.045455	no	no	yes	no	no	no	no	no	no	yes
182189831	chr1	41155421	41155533	48	113	2.354167	no	no	yes	no	no	no	no	yes	yes	yes
182189837	chr1	41170752	41170794	17	43	2.529412	no	no	yes	no	no	no	no	no	no	yes
182189845	chr1	41182875	41182912	19	38	2.000000	no	no	yes	no	no	no	no	no	no	yes
182189850	chr1	41200494	41200537	20	44	2.200000	no	no	yes	no	no	no	no	no	no	yes
182189857	chr1	41214082	41214122	12	41	3.416667	no	no	yes	no	no	no	no	no	yes	yes
182189860	chr1	41233188	41233234	15	47	3.133333	no	no	yes	no	no	no	no	no	no	yes
182189864	chr1	41245278	41245320	18	43	2.388889	no	no	yes	no	no	no	no	no	no	yes
182189866	chr1	41247718	41247761	11	44	3.916667	no	no	yes	no	no	no	no	no	no	yes
182189872	chr1	41279537	41279563	11	27	2.454545	no	no	yes	no	no	no	no	yes	no	yes
182189874	chr1	41290940	41291008	20	69	3.428571	no	no	yes	no	no	no	no	yes	no	yes
182189879	chr1	41315756	41315831	40	76	1.900000	no	no	yes	no	no	no	no	yes	yes	yes
182189880	chr1	41323397	41323437	19	41	2.157895	no	no	yes	no	no	no	no	yes	yes	yes
182189882	chr1	41325418	41325450	16	33	2.062500	no	no	yes	no	no	no	no	yes	no	yes
182189884	chr1	41328958	41329598	65	641	9.969231	no	no	yes	no	no	no	no	yes	yes	yes
182189893	chr1	41361521	41361555	18	35	1.944444	yes	no	no	no	no	no	yes	yes	no	no
182189899	chr1	41392129	41392182	28	54	2.000000	no	no	no	no	no	no	no	yes	yes	no
182189908	chr1	41413783	41413822	15	40	2.666667	no	no	no	no	no	no	no	yes	no	no
182189915	chr1	41424298	41424346	11	49	4.500000	no	no	no	no	no	no	no	yes	no	no
182189920	chr1	41425758	41425796	14	39	2.687500	no	no	no	no	no	no	no	yes	no	no
182189924	chr1	41426043	41426350	44	308	7.000000	no	no	no	no	no	no	no	no	no	no
182189931	chr1	41467403	41467433	13	31	2.384615	yes	no	no	no	no	no	no	no	no	no
182189932	chr1	41469921	41469961	22	41	1.954545	no	no	no	no	no	no	no	no	yes	no
182189935	chr1	41482636	41482676	20	41	2.050000	no	no	yes	no	no	no	no	yes	yes	yes

182189938	chr1	41489515	41490018	99	504	5.212121	no	no	no	no	no	no	no	yes	no	no
182189942	chr1	41506586	41506631	23	46	2.000000	no	yes	no	no	no	no	no	no	no	yes
182189943	chr1	41506884	41506920	13	37	2.846154	no	yes	no	no	no	no	no	no	no	yes
182189944	chr1	41511010	41511054	24	45	1.916667	no	no	no	yes	yes	no	no	no	no	yes
182189946	chr1	41521600	41521637	20	38	1.900000	no	no	yes	no	no	no	no	yes	yes	yes
182189960	chr1	41527620	41528005	219	386	1.808219	no	no	yes	no	no	no	no	no	no	yes
182189971	chr1	41528865	41528938	26	74	2.888889	no	no	yes	no	no	no	no	no	no	yes
182189980	chr1	41529616	41529651	17	36	2.176471	no	no	yes	no	no	no	no	no	no	yes
182189982	chr1	41530634	41530670	18	37	2.055556	no	no	yes	no	no	no	no	yes	no	yes
182189983	chr1	41531011	41532347	16	1337	83.388885	no	no	yes	no	no	no	no	yes	no	yes
182189988	chr1	41561990	41562026	19	37	2.000000	no	no	yes	no	no	no	no	yes	no	yes
182189991	chr1	41562480	41562605	42	126	3.047619	no	no	yes	no	no	no	no	no	no	yes
182190022	chr1	41639006	41639035	11	30	2.727273	no	no	yes	no	no	no	no	yes	yes	yes
182190025	chr1	41653054	41653086	14	33	2.357143	no	no	yes	no	no	no	no	yes	no	yes
182190032	chr1	41662148	41662202	20	55	2.750000	no	no	yes	no	yes	no	yes	yes	yes	yes
182190034	chr1	41662202	41662269	34	68	2.029412	no	no	yes	no	yes	no	yes	yes	yes	yes
182190043	chr1	41688183	41688253	23	71	3.086957	no	no	yes	no	no	no	no	no	no	yes
182190046	chr1	41703749	41703798	25	50	2.040000	no	no	yes	no	no	no	no	no	no	yes
182190054	chr1	41742140	41742193	28	54	1.928571	no	no	yes	no	no	no	no	yes	no	yes
182190055	chr1	41743113	41743148	7	36	5.285714	no	no	yes	no	no	no	no	no	no	yes
182190056	chr1	41744434	41744466	17	33	1.941176	no	no	yes	no	no	no	no	no	yes	yes
182190057	chr1	41744737	41744823	30	87	2.900000	no	no	yes	no	no	no	no	no	no	yes
182190061	chr1	41751889	41751950	25	62	2.520000	no	no	yes	no	no	no	no	yes	no	yes
182190068	chr1	41761068	41761098	16	31	1.937500	no	no	yes	no	no	no	no	no	no	yes
182190079	chr1	41761475	41761514	20	40	2.100000	no	no	yes	no	no	no	no	yes	no	yes

182190080	chr1	41761576	41761605	12	30	2.500000	no	no	yes	no	no	no	no	yes	no	yes
182190082	chr1	41772159	41772196	19	38	2.052632	no	no	yes	no	no	no	no	no	no	yes
182190086	chr1	41783981	41784011	16	31	1.937500	no	no	yes	no	no	no	no	no	no	yes
182190087	chr1	41789505	41789548	21	44	2.095238	no	no	yes	no	no	no	no	yes	yes	yes
182190095	chr1	41824355	41824404	25	50	2.000000	no	no	yes	no	no	no	no	no	no	yes
182190098	chr1	41829573	41829615	20	43	2.200000	no	no	yes	no	no	no	no	no	no	yes
182190105	chr1	41854334	41854371	17	38	2.235294	no	no	yes	no	no	no	no	no	no	yes
182190106	chr1	41869187	41869235	19	49	2.631579	no	no	yes	no	no	no	no	yes	yes	yes
182190108	chr1	41877874	41877915	14	42	3.000000	no	no	yes	no	no	no	no	no	no	yes
182190110	chr1	41881159	41881193	18	35	1.944444	no	no	yes	no	no	no	no	yes	yes	yes
182190120	chr1	41888707	41888765	31	59	1.935484	no	no	yes	no	no	no	no	yes	yes	yes
182190128	chr1	41899177	41899216	20	40	2.100000	no	no	yes	no	no	no	no	no	yes	yes
182190130	chr1	41914544	41914581	18	38	2.166667	no	no	yes	no	no	no	no	yes	no	yes
182190134	chr1	41921913	41921952	15	40	2.666667	no	no	yes	no	no	no	no	no	no	yes
182190144	chr1	41939928	41939965	16	38	2.352941	no	no	yes	no	no	no	no	no	no	yes
182190148	chr1	41952531	41952567	19	37	2.000000	no	no	yes	no	no	no	no	yes	no	yes
182190153	chr1	41985034	41985070	12	37	3.083333	no	no	yes	no	no	no	no	yes	no	yes
182190162	chr1	42031160	42031188	14	29	2.071429	no	no	yes	no	no	no	no	yes	yes	yes
182190163	chr1	42033212	42033244	17	33	1.941176	no	no	yes	no	no	no	no	no	no	yes
182190164	chr1	42039422	42039448	12	27	2.250000	no	no	yes	no	no	no	no	no	no	yes
182190172	chr1	42069677	42069716	20	40	2.050000	no	no	no	no	no	no	no	no	no	no
182190190	chr1	42114166	42114217	27	52	1.925926	no	no	no	no	no	no	no	no	no	no
182190204	chr1	42146221	42146261	16	41	2.562500	no	no	no	no	no	no	no	yes	yes	no
182190207	chr1	42150040	42150079	21	40	1.904762	no	no	no	no	no	no	no	no	no	no
182190211	chr1	42165612	42165645	17	34	2.000000	yes	no	no	no	no	no	no	yes	yes	no

182190214	chr1	42166249	42166284	17	36	2.176471	no	no	no	no	no	no	no	yes	yes	no
182190218	chr1	42166481	42166508	12	28	2.333333	no	no	no	no	no	no	no	yes	no	no
182190220	chr1	42166546	42166603	24	58	2.416667	no	no	no	no	no	no	no	no	yes	no
182190226	chr1	42166629	42167660	319	1032	3.134831	no	no	no	no	no	no	no	no	yes	no
182190231	chr1	42181737	42181823	44	87	1.977273	no	no	yes	no	no	no	no	yes	no	yes
182190237	chr1	42221965	42222046	24	82	3.416667	no	no	yes	no	no	no	no	yes	no	yes
182190256	chr1	42281382	42281445	34	64	1.970588	no	no	yes	no	no	no	no	no	no	yes
182190258	chr1	42290207	42290243	19	37	1.947368	no	no	yes	no	no	no	no	yes	no	yes
182190261	chr1	42292016	42292112	35	97	2.771429	no	no	yes	no	no	no	no	yes	no	yes
182190262	chr1	42293116	42293232	60	117	1.950000	no	no	yes	no	no	no	no	no	no	yes
182190265	chr1	42308209	42308241	14	33	2.428571	no	no	yes	no	no	no	no	no	yes	yes
182190266	chr1	42308566	42308689	66	124	1.893939	no	no	yes	no	no	no	no	no	no	yes
182190267	chr1	42311315	42311364	21	50	2.428571	no	no	yes	no	no	no	no	yes	yes	yes
182190271	chr1	42320522	42320670	50	149	3.019608	no	no	yes	no	no	no	no	yes	no	yes
182190276	chr1	42324015	42324178	57	164	2.881356	no	no	yes	no	no	no	no	no	no	yes
182190277	chr1	42324148	42324260	41	113	2.761905	no	no	yes	no	no	no	no	no	no	yes
182190290	chr1	42361401	42361432	13	32	2.461539	no	no	no	no	no	no	no	no	no	no
182190299	chr1	42384471	42384614	32	144	4.500000	no	no	yes	no	no	no	no	no	no	yes
182190319	chr1	42439783	42439846	31	64	2.064516	no	no	yes	no	no	no	no	no	yes	yes
182190320	chr1	42440101	42440139	16	39	2.437500	no	no	yes	no	no	no	no	yes	yes	yes
182190323	chr1	42441548	42441589	22	42	2.000000	no	no	yes	no	no	no	no	no	no	yes
182190325	chr1	42447712	42447748	17	37	2.176471	no	no	yes	no	no	no	no	no	no	yes
182190333	chr1	42488177	42488212	12	36	3.000000	no	no	yes	no	no	no	no	no	no	yes
182190340	chr1	42499784	42499824	21	41	1.952381	no	no	yes	no	no	yes	no	no	no	yes
182190351	chr1	42539593	42539717	62	125	2.016129	no	no	yes	no	no	no	no	no	no	yes

182190354	chr1	42544976	42545012	19	37	1.947368	no	no	yes	no	no	no	no	no	no	yes
182190361	chr1	42557405	42557439	14	35	2.466667	no	no	yes	no	no	no	no	yes	yes	yes
182190362	chr1	42557608	42557644	16	37	2.375000	no	no	yes	no	no	no	no	yes	no	yes
182190364	chr1	42557630	42557714	32	85	2.687500	no	no	yes	no	no	no	no	yes	no	yes
182190370	chr1	42565789	42565817	15	29	2.000000	yes	no	yes	no	no	no	no	no	no	yes
182190384	chr1	42620140	42620175	16	36	2.375000	no	no	yes	no	no	no	no	yes	no	yes
182190420	chr1	42739138	42739167	14	30	2.142857	no	no	yes	no	no	no	no	yes	yes	yes
182190422	chr1	42747241	42747278	19	38	2.000000	no	no	yes	yes	yes	yes	no	yes	yes	yes
182190423	chr1	42748695	42748732	20	38	1.900000	yes	no	yes	no	yes	no	no	no	no	yes
182190433	chr1	42754777	42754825	27	49	1.962963	no	no	yes	no	no	yes	no	no	no	yes
182190443	chr1	42788551	42788576	10	26	2.600000	no	no	yes	no	no	no	no	no	no	yes
182190454	chr1	42808550	42808588	18	39	2.277778	no	no	yes	no	no	no	no	no	no	yes
182190455	chr1	42808606	42808712	36	107	2.972973	no	no	yes	no	no	no	no	no	no	yes
182190466	chr1	42845282	42845345	33	64	2.000000	no	no	yes	no	no	yes	no	yes	yes	yes
182190467	chr1	42845432	42845484	25	53	2.160000	no	no	yes	no	no	yes	no	yes	yes	yes
182190470	chr1	42845510	42845550	18	41	2.333333	no	no	yes	no	no	yes	no	yes	no	yes
182190476	chr1	42853446	42853498	26	53	2.038461	no	no	no	no	no	yes	no	yes	yes	no
182190478	chr1	42855183	42855219	17	37	2.235294	no	no	no	no	no	no	no	no	no	no
182190501	chr1	42932250	42932285	13	36	2.923077	no	no	yes	no	no	no	no	yes	yes	yes
182190513	chr1	42968929	42969019	45	91	2.022222	no	no	yes	no	no	no	no	yes	no	yes
182190532	chr1	43002098	43002150	19	53	2.789474	no	no	no	no	no	no	no	yes	no	no
182190551	chr1	43055819	43055848	12	30	2.500000	no	no	no	no	no	no	no	yes	no	no
182190552	chr1	43057206	43057248	23	43	1.913043	no	no	no	no	no	no	no	yes	yes	no
182190553	chr1	43057651	43057704	27	54	2.000000	no	no	no	no	no	no	no	yes	no	no
182190554	chr1	43059015	43059077	33	63	1.969697	no	no	no	no	no	no	no	yes	yes	no

182190557	chr1	43062605	43062643	18	39	2.166667	no	no	no	no	no	no	no	yes	yes	no
182190560	chr1	43068397	43068422	9	26	2.888889	no	no	no	no	no	no	yes	yes	yes	no
182190598	chr1	43125035	43125067	17	33	1.941176	no	no	no	no	no	no	no	yes	no	no
182190603	chr1	43129015	43129068	28	54	1.928571	no	no	no	no	no	no	no	yes	no	no
182190607	chr1	43147845	43147909	25	65	2.680000	yes	yes	yes	no	yes	no	yes	yes	yes	yes
182190611	chr1	43154081	43154148	29	68	2.344828	no	no	yes	no	no	no	no	no	no	yes
182190617	chr1	43154349	43154453	36	105	3.000000	no	no	yes	no	no	no	no	yes	no	yes
182190622	chr1	43165254	43165284	16	31	1.937500	no	no	yes	no	no	no	no	no	yes	yes
182190624	chr1	43172564	43172651	24	88	3.640000	yes	no	yes	no	yes	no	no	yes	yes	yes
182190647	chr1	43254051	43254079	15	29	1.933333	no	no	yes	yes	no	no	no	no	no	yes
182190656	chr1	43268570	43268600	14	31	2.214286	no	no	yes	no	no	no	no	no	no	yes
182190657	chr1	43270288	43270333	24	46	1.916667	yes	no	yes	no	no	no	no	yes	no	yes
182190658	chr1	43273064	43273108	21	45	2.142857	no	no	no	yes	no	yes	yes	yes	no	yes
182190667	chr1	43308302	43308504	50	203	4.060000	yes	no	yes	no	no	no	no	yes	no	yes
182190673	chr1	43340813	43340867	27	55	2.037037	no	no	yes	no	no	no	no	yes	yes	yes
182190678	chr1	43349075	43349122	24	48	2.000000	yes	no	yes	no	no	no	yes	yes	yes	yes
182190681	chr1	43352410	43352439	11	30	2.666667	no	no	yes	no	no	no	no	no	yes	yes
182190686	chr1	43366275	43366323	24	49	2.125000	no	no	yes	no	yes	no	no	yes	yes	yes
182190688	chr1	43367820	43367868	10	49	4.900000	yes	no	yes	no	no	no	no	yes	yes	yes
182190692	chr1	43376277	43376329	26	53	2.038461	no	no	no	no	no	no	no	no	no	no
182190697	chr1	43396711	43396747	17	37	2.235294	no	no	yes	no	no	no	no	no	no	yes
182190699	chr1	43423319	43424184	42	866	21.000000	no	no	yes	no	no	no	no	yes	yes	yes
182190702	chr1	43432484	43432524	22	41	1.909091	no	yes	yes	yes	no	yes	no	yes	no	yes
182190703	chr1	43453161	43453185	13	25	1.923077	yes	yes	yes	no	yes	yes	no	yes	yes	yes
182190705	chr1	43453835	43453873	7	39	5.714286	yes	yes	no	no	yes	no	yes	yes	yes	yes

182190712	chr1	43462795	43462829	18	35	1.944444	no	no	no	no	no	no	no	no	no	no
182190721	chr1	43491292	43491329	9	38	4.300000	no	no	no	no	no	no	no	no	yes	no
182190743	chr1	43530676	43530715	19	40	2.105263	yes	no	yes	no	no	no	no	yes	yes	yes
182190745	chr1	43531228	43531287	33	60	1.939394	no	no	yes	no	no	no	yes	yes	yes	yes
182190746	chr1	43531980	43532036	23	57	2.478261	no	no	yes	no	no	no	no	yes	yes	yes
182190754	chr1	43559590	43559979	162	390	2.457317	no	no	yes	no	no	no	no	yes	yes	yes
182190768	chr1	43597099	43597138	12	40	3.230769	yes	no	yes	no	no	no	no	yes	yes	yes
182190769	chr1	43597143	43597192	26	50	1.923077	yes	no	yes	no	no	no	no	yes	yes	yes
182190774	chr1	43597250	43597307	28	58	2.142857	yes	no	yes	no	no	no	no	yes	yes	yes
182190776	chr1	43597413	43597450	20	38	1.950000	yes	no	yes	no	no	no	no	yes	no	yes
182190779	chr1	43597493	43597530	15	38	2.600000	yes	no	yes	no	no	no	no	yes	no	yes
182190780	chr1	43597551	43597579	15	29	1.933333	yes	no	yes	no	no	no	no	yes	no	yes
182190782	chr1	43604404	43604449	24	46	1.916667	no	no	yes	no	no	no	no	yes	yes	yes
182190783	chr1	43612901	43612937	19	37	1.947368	yes	no	yes	no	no	no	no	yes	no	yes
182190786	chr1	43618165	43618239	38	75	1.973684	no	no	yes	no	no	no	no	yes	yes	yes
182190788	chr1	43622846	43622906	16	61	3.875000	no	yes	no	no	no	yes	no	no	yes	yes
182190798	chr1	43633086	43633228	62	143	2.306452	no	no	no	no	no	no	no	no	no	no
182190803	chr1	43651989	43652057	30	69	2.300000	no	no	yes	no	no	no	no	yes	yes	yes
182190810	chr1	43665311	43665346	17	36	2.117647	yes	no	yes	no	no	no	no	no	no	yes
182190814	chr1	43671932	43671978	23	47	2.086957	no	no	yes	no	no	no	no	yes	no	yes
182190817	chr1	43704771	43704806	8	36	4.500000	yes	yes	yes	no	no	no	no	yes	yes	yes
182190818	chr1	43710651	43710679	14	29	2.071429	no	no	yes	no	no	no	no	no	yes	yes
182190849	chr1	43741435	43741473	20	39	1.950000	no	no	yes	no	no	no	no	no	yes	yes
182190861	chr1	43770010	43770075	33	66	2.000000	no	no	yes	no	no	no	no	yes	no	yes
182190864	chr1	43770268	43770303	12	36	3.166667	no	no	yes	no	no	no	no	yes	no	yes

182190867	chr1	43773560	43773605	23	46	2.000000	no	no	yes	no	no	yes	no	yes	no	yes
182190872	chr1	43787458	43787492	15	35	2.333333	no	no	yes	no	no	no	no	yes	no	yes
182190878	chr1	43806319	43806349	16	31	1.937500	no	no	yes	no	no	no	no	no	yes	yes
182190899	chr1	43872291	43872343	25	53	2.120000	no	no	yes	no	no	no	no	no	no	yes
182190907	chr1	43904677	43904731	21	55	2.565217	no	no	yes	no	no	no	no	yes	yes	yes
182190913	chr1	43911488	43911692	28	205	7.321429	no	no	yes	no	no	no	no	no	no	yes
182190916	chr1	43916189	43916215	13	27	2.076923	no	no	yes	no	no	no	no	yes	no	yes
182190930	chr1	43939246	43939353	17	108	6.588235	no	no	yes	no	no	no	no	yes	yes	yes
182190934	chr1	43945847	43945878	13	32	2.461539	no	no	yes	no	no	no	no	yes	yes	yes
182190938	chr1	43979255	43979286	16	32	2.000000	yes	yes	yes	no	yes	yes	yes	yes	yes	yes
182190947	chr1	44020240	44020316	38	77	2.026316	no	no	yes	no	no	no	no	no	no	yes
182190949	chr1	44023686	44023748	30	63	2.100000	no	no	yes	no	no	no	no	no	no	yes
182190955	chr1	44034623	44034652	14	30	2.142857	no	no	yes	no	no	no	no	yes	no	yes
182190956	chr1	44035015	44035056	17	42	2.470588	no	no	yes	no	no	no	no	yes	no	yes
182190959	chr1	44041804	44041853	22	50	2.363636	no	no	yes	no	no	yes	no	no	no	yes
182190970	chr1	44074190	44074222	15	33	2.266667	no	no	yes	no	no	no	no	yes	no	yes
182190973	chr1	44081245	44081280	14	36	2.571429	no	no	yes	no	no	no	no	no	yes	yes
182190986	chr1	44090913	44090974	22	62	2.818182	no	no	yes	no	no	no	no	yes	yes	yes
182190998	chr1	44103327	44103354	13	28	2.153846	no	yes	no	no	yes	yes	yes	yes	no	yes
182191009	chr1	44117430	44117512	28	83	2.966667	yes	no	no	no	no	no	no	no	no	no
182191010	chr1	44118490	44118533	18	44	2.444444	yes	no	no	no	no	no	no	no	no	no
182191011	chr1	44119085	44119113	15	29	1.933333	no	no	yes	no	no	no	no	no	no	yes
182191012	chr1	44119110	44119242	33	133	4.030303	no	no	yes	no	no	no	no	no	no	yes
182191022	chr1	44138449	44138507	24	59	2.458333	no	no	yes	no	no	no	no	no	no	yes
182191024	chr1	44138944	44140978	42	2035	48.477272	no	no	no	yes	no	no	no	no	no	yes

182191035	chr1	44146787	44146820	17	34	2.000000	no	no	no	no	no	no	no	no	no	no
182191068	chr1	44207223	44207272	21	50	2.380952	no	no	no	no	no	no	no	no	no	no
182191070	chr1	44211743	44211783	12	41	3.307692	no	no	no	no	no	no	no	no	no	no
182191076	chr1	44239783	44239820	9	38	4.222222	no	no	yes	no	no	no	no	yes	yes	yes
182191079	chr1	44241866	44241930	25	65	2.680000	no	yes	yes	yes	no	no	no	no	yes	yes
182191081	chr1	44251144	44251176	15	33	2.200000	no	no	yes	no	no	no	no	no	no	yes
182191082	chr1	44259264	44259295	8	32	4.000000	no	no	yes	no	no	no	no	yes	no	yes
182191086	chr1	44266153	44266181	13	29	2.230769	no	no	yes	no	no	no	no	no	no	yes
182191089	chr1	44281849	44281879	16	31	1.937500	no	no	yes	no	no	no	no	no	no	yes
182191097	chr1	44313466	44313520	27	55	2.037037	no	no	yes	no	no	no	no	yes	yes	yes
182191098	chr1	44319829	44319864	17	36	2.117647	yes	no	yes	no	no	no	no	no	no	yes
182191101	chr1	44331415	44331472	28	58	2.107143	no	no	yes	no	no	no	no	no	no	yes
182191111	chr1	44359541	44359569	15	29	1.933333	no	no	no	no	no	no	no	yes	yes	no
182191112	chr1	44364286	44364318	8	33	4.000000	no	no	no	no	no	no	no	no	no	no
182191124	chr1	44405190	44405226	16	37	2.312500	yes	no	no	no	yes	no	no	yes	yes	yes
182191127	chr1	44405389	44405431	15	43	2.866667	no	no	no	no	yes	no	no	yes	yes	yes
182191129	chr1	44415597	44415633	16	37	2.375000	no	no	yes	no	no	no	no	no	no	yes
182191131	chr1	44418738	44418996	136	259	1.926471	no	no	yes	no	no	no	no	yes	no	yes
182191132	chr1	44423775	44423803	13	29	2.230769	no	no	yes	no	no	no	no	yes	yes	yes
182191133	chr1	44431068	44431112	18	45	2.500000	no	no	yes	no	no	no	no	no	no	yes
182191134	chr1	44434110	44434162	28	53	1.928571	no	no	yes	no	no	no	no	no	yes	yes
182191149	chr1	44486028	44486066	18	39	2.166667	no	no	yes	no	no	no	no	yes	yes	yes
182191160	chr1	44502244	44502279	19	36	2.000000	no	no	yes	no	no	no	no	no	yes	yes
182191162	chr1	44506444	44506478	17	35	2.058824	no	no	yes	no	no	no	no	no	no	yes
182191166	chr1	44510266	44510328	32	63	2.031250	no	no	yes	no	no	no	no	yes	yes	yes

182191168	chr1	44513392	44513429	14	38	2.714286	no	no	yes	no	no	no	no	no	no	yes
182191169	chr1	44516633	44516668	18	36	2.055556	no	no	yes	no	no	no	no	yes	no	yes
182191198	chr1	44576033	44576166	69	134	1.971014	no	no	yes	no	no	no	no	no	no	yes
182191200	chr1	44577297	44577412	36	116	3.216216	no	no	yes	no	no	no	no	no	yes	yes
182191208	chr1	44608405	44608442	21	38	1.904762	no	no	yes	no	no	no	no	yes	yes	yes
182191211	chr1	44617962	44618002	19	41	2.210526	no	no	yes	no	no	no	no	yes	no	yes
182191212	chr1	44622542	44622591	26	50	1.923077	no	no	yes	no	no	no	no	no	yes	yes
182191213	chr1	44622867	44622924	28	58	2.071429	no	no	yes	no	no	no	no	no	yes	yes
182191214	chr1	44624663	44624700	15	38	2.600000	no	no	yes	no	no	no	no	yes	no	yes
182191219	chr1	44636814	44636849	18	36	2.055556	no	no	yes	no	no	yes	no	yes	yes	yes
182191220	chr1	44650082	44650116	15	35	2.333333	no	yes	yes	no	no	yes	no	no	yes	yes
182191225	chr1	44675075	44675119	23	45	1.956522	yes	no	yes	no	no	no	no	yes	yes	yes
182191263	chr1	44775779	44775821	21	43	2.047619	yes	yes	yes	no	yes	no	yes	yes	yes	yes
182191283	chr1	44816378	44816947	29	570	19.931034	no	no	no	no	no	yes	no	yes	no	no
182191289	chr1	44823202	44823244	20	43	2.150000	no	no	yes	yes	no	no	no	yes	no	yes
182191292	chr1	44832904	44832929	13	26	2.000000	no	no	yes	no	no	no	no	yes	no	yes
182191308	chr1	44893906	44893935	14	30	2.142857	no	no	yes	no	no	no	no	no	no	yes
182191314	chr1	44909150	44909190	21	41	1.952381	no	no	yes	no	no	no	no	no	no	yes
182191331	chr1	44933753	44933805	17	53	3.176471	no	no	yes	no	no	no	no	no	yes	yes
182191340	chr1	44940413	44940455	20	43	2.150000	no	no	yes	no	no	yes	no	yes	no	yes
182191361	chr1	44964073	44964104	16	32	2.000000	no	no	yes	no	no	no	no	no	no	yes
182191372	chr1	44981190	44981227	19	38	2.052632	no	no	yes	no	no	no	no	no	no	yes
182191381	chr1	45017369	45017405	20	37	1.950000	no	yes	no	no	no	no	no	yes	yes	yes
182191385	chr1	45027370	45027402	17	33	1.941176	no	no	yes	no	no	no	no	no	no	yes
182191400	chr1	45057010	45057054	21	45	2.142857	no	no	yes	no	no	no	no	yes	no	yes

182191401	chr1	45060632	45060672	21	41	1.952381	no	no	yes	no	no	no	no	yes	no	yes
182191414	chr1	45086374	45086412	20	39	1.950000	no	no	yes	no	no	no	no	no	no	yes
182191415	chr1	45090409	45090443	17	35	2.058824	no	no	yes	no	no	no	no	no	no	yes
182191430	chr1	45116381	45116478	51	98	1.941176	no	no	yes	no	no	no	no	yes	yes	yes
182191437	chr1	45132051	45132080	15	30	2.000000	no	no	yes	no	no	no	no	no	no	yes
182191455	chr1	45171807	45171864	29	58	2.000000	no	no	yes	no	no	no	no	yes	no	yes
182191464	chr1	45193908	45193945	10	38	3.800000	no	no	yes	no	no	no	no	no	no	yes
182191466	chr1	45203360	45203396	18	37	2.111111	no	no	yes	no	no	no	no	yes	no	yes
182191477	chr1	45225808	45225874	11	67	6.083333	no	no	yes	no	no	no	no	yes	no	yes
182191495	chr1	45256967	45257002	17	36	2.117647	no	no	yes	no	no	no	no	yes	no	yes
182191513	chr1	45303673	45303826	71	154	2.169014	yes	no	yes	no	no	no	yes	yes	yes	yes
182191534	chr1	45362675	45362730	29	56	1.965517	no	no	yes	no	no	no	no	no	no	yes
182191546	chr1	45384364	45384437	8	74	9.250000	no	no	yes	no	no	no	no	yes	no	yes
182191552	chr1	45386404	45386466	25	63	2.538461	no	no	yes	no	no	no	no	no	no	yes
182191571	chr1	45412652	45412691	20	40	2.000000	no	no	yes	no	no	no	no	no	no	yes
182191574	chr1	45416270	45416313	20	44	2.200000	no	no	yes	no	no	yes	no	no	no	yes
182191591	chr1	45454415	45454456	17	42	2.555556	no	no	yes	no	no	no	no	yes	no	yes
182191603	chr1	45477769	45477846	38	78	2.052632	no	no	yes	no	no	no	no	no	no	yes
182191616	chr1	45499350	45499480	65	131	2.015385	yes	no	yes	yes	yes	no	no	yes	yes	yes
182191628	chr1	45523569	45523881	155	313	2.019355	yes	no	no	no	no	no	no	yes	yes	no
182191636	chr1	45527893	45527934	22	42	1.909091	no	no	no	no	no	no	no	yes	no	no
182191639	chr1	45532092	45532135	21	44	2.095238	no	yes	no	no	no	no	no	yes	no	yes
182191660	chr1	45564519	45564571	26	53	2.038461	no	no	yes	no	no	no	no	yes	yes	yes
182191678	chr1	45603028	45603070	22	43	1.954545	no	no	yes	no	no	yes	no	yes	yes	yes
182191681	chr1	45623608	45623658	21	51	2.428571	yes	no	yes	yes	yes	no	no	yes	yes	yes

182191689	chr1	45638546	45638586	18	41	2.277778	no	no	yes	no	no	no	no	no	no	yes
182191696	chr1	45660392	45660429	7	38	5.375000	yes	no	no	no	yes	yes	no	yes	no	yes
182191708	chr1	45688282	45688367	43	86	2.023256	yes	no	yes	no	no	no	no	yes	yes	yes
182191712	chr1	45695608	45695645	18	38	2.111111	no	no	yes	no	no	no	no	yes	no	yes
182191740	chr1	45728350	45728379	15	30	2.066667	no	no	yes	no	no	no	no	no	no	yes
182191751	chr1	45746785	45746817	14	33	2.357143	no	no	yes	no	no	no	no	yes	no	yes
182191778	chr1	45790352	45790504	67	153	2.343284	no	no	yes	no	no	no	no	no	no	yes
182191782	chr1	45790592	45790642	22	51	2.318182	no	no	yes	no	no	no	no	no	no	yes
182191806	chr1	45856002	45856029	13	28	2.153846	no	no	yes	no	no	no	no	no	yes	yes
182191828	chr1	45911415	45911447	16	33	2.062500	no	no	yes	no	no	no	no	no	no	yes
182191844	chr1	45945621	45945694	23	74	3.208333	no	no	yes	no	no	no	no	yes	yes	yes
182191862	chr1	45987763	45987816	26	54	2.076923	no	no	yes	no	no	no	no	no	no	yes
182191867	chr1	46005671	46005705	17	35	2.058824	yes	no	yes	no	no	no	no	no	no	yes
182191868	chr1	46009896	46009927	13	32	2.461539	no	no	yes	no	no	no	no	no	no	yes
182191869	chr1	46029134	46029170	18	37	2.055556	yes	yes	yes	no	no	no	no	yes	no	yes
182191877	chr1	46063121	46063150	15	30	2.000000	no	no	yes	no	no	no	no	yes	yes	yes
182191880	chr1	46068035	46068090	17	56	3.411765	no	no	yes	no	no	no	no	yes	no	yes
182191897	chr1	46121637	46121678	21	42	2.000000	no	no	yes	no	no	no	no	no	no	yes
182191924	chr1	46192679	46192709	16	31	1.937500	yes	no	yes	no	no	yes	no	yes	no	yes
182191925	chr1	46198349	46198377	11	29	2.636364	yes	no	yes	no	yes	no	no	yes	yes	yes
182191936	chr1	46209831	46209882	24	52	2.166667	no	no	yes	no	no	no	no	no	no	yes
182191940	chr1	46222207	46222241	14	35	2.500000	no	no	no	no	no	yes	no	yes	no	no
182191975	chr1	46245298	46245346	24	49	2.041667	no	no	no	no	no	no	no	yes	yes	no
182191977	chr1	46247346	46247560	75	215	2.921053	yes	no	yes	no	no	no	yes	yes	yes	yes
182191983	chr1	46256730	46256773	23	44	1.913043	no	no	yes	no	no	no	no	yes	no	yes

182191991	chr1	46306232	46306267	17	36	2.117647	no	no	yes	no	no	no	no	yes	yes	yes
182192028	chr1	46352475	46352515	15	41	2.866667	no	no	yes	no	no	no	no	yes	no	yes
182192034	chr1	46358891	46358936	24	46	1.916667	no	no	yes	no	no	no	no	no	no	yes
182192044	chr1	46374791	46374840	8	50	6.222222	no	no	no	no	no	no	no	yes	no	no
182192063	chr1	46402197	46402269	36	73	2.027778	no	yes	yes	yes	no	no	no	yes	yes	yes
182192068	chr1	46407312	46407354	20	43	2.200000	no	no	yes	no	no	no	no	yes	yes	yes
182192073	chr1	46423873	46423914	16	42	2.750000	no	no	no	no	no	no	no	no	no	no
182192075	chr1	46425239	46425287	25	49	2.000000	no	no	no	no	no	no	no	yes	yes	no
182192085	chr1	46453270	46453304	10	35	3.500000	no	no	yes	no	no	no	no	yes	no	yes
182192086	chr1	46453602	46453740	34	139	4.088235	no	no	yes	no	no	no	no	yes	no	yes
182192087	chr1	46459056	46459093	19	38	2.105263	no	no	yes	no	no	no	no	no	yes	yes
182192101	chr1	46474374	46474399	8	26	3.250000	no	no	yes	no	no	no	no	yes	no	yes
182192102	chr1	46475324	46475356	17	33	1.941176	no	no	yes	no	no	no	no	no	no	yes
182192107	chr1	46485299	46485339	21	41	1.952381	no	no	no	no	no	no	no	yes	yes	no
182192109	chr1	46488868	46488900	14	33	2.357143	no	no	no	no	no	no	no	yes	no	no
182192111	chr1	46507036	46507070	19	35	1.947368	no	no	no	yes	no	no	no	yes	yes	yes
182192113	chr1	46522987	46523039	27	53	1.962963	no	no	no	no	no	no	no	yes	yes	no
182192115	chr1	46533586	46533659	36	74	2.055556	no	no	yes	no	yes	no	no	yes	yes	yes
182192119	chr1	46539283	46539361	37	79	2.135135	no	no	yes	no	no	no	no	no	no	yes
182192123	chr1	46548601	46548638	20	38	1.900000	no	no	yes	no	no	no	no	yes	no	yes
182192124	chr1	46557593	46557626	16	34	2.125000	no	yes	yes	no	yes	yes	no	yes	yes	yes
182192136	chr1	46609103	46609136	16	34	2.125000	no	yes	yes	no	no	yes	no	yes	no	yes
182192146	chr1	46637157	46637201	21	45	2.142857	no	no	yes	no	no	no	no	no	no	yes
182192153	chr1	46652376	46652487	55	112	2.036364	no	no	yes	no	yes	yes	no	yes	yes	yes
182192156	chr1	46671840	46671881	21	42	2.000000	yes	no	yes	no	yes	no	no	no	no	yes

182192158	chr1	46681762	46681793	17	32	1.941176	no	no	yes	no	no	no	no	yes	no	yes
182192159	chr1	46691534	46691572	20	39	1.950000	no	no	yes	no	no	no	no	no	no	yes
182192162	chr1	46693513	46693565	21	53	2.523809	no	no	yes	no	no	no	no	no	yes	yes
182192164	chr1	46696495	46696568	35	74	2.114286	no	no	yes	yes	yes	no	no	no	yes	yes
182192166	chr1	46707812	46707843	17	32	1.941176	no	no	yes	no	no	no	no	yes	no	yes
182192179	chr1	46739588	46739641	26	54	2.076923	no	no	no	no	no	no	no	yes	no	no
182192182	chr1	46757410	46757446	16	37	2.312500	yes	no	no	no	no	no	no	yes	yes	no
182192214	chr1	46842598	46842638	21	41	1.952381	no	no	no	no	no	yes	no	no	no	no
182192230	chr1	46931232	46931273	23	42	1.913043	no	no	yes	no	no	yes	no	yes	yes	yes
182192252	chr1	47024002	47024042	7	41	5.750000	no	no	yes	no	no	no	no	yes	yes	yes
182192253	chr1	47032987	47033019	17	33	1.941176	no	no	yes	no	no	no	no	yes	yes	yes
182192254	chr1	47036937	47036982	22	46	2.090909	no	no	yes	no	no	no	no	no	no	yes
182192255	chr1	47045293	47045324	16	32	2.000000	no	no	yes	no	no	no	no	no	no	yes
182192257	chr1	47052774	47052822	20	49	2.450000	no	no	no	no	no	no	no	no	no	no
182192272	chr1	47118402	47118517	55	116	2.109091	no	no	yes	no	no	yes	no	no	no	yes
182192273	chr1	47118673	47118722	22	50	2.318182	no	no	yes	no	no	yes	no	no	no	yes
182192283	chr1	47143151	47143204	28	54	1.964286	no	no	yes	no	no	no	no	no	no	yes
182192291	chr1	47163847	47163905	20	59	2.950000	no	no	yes	no	no	yes	no	no	no	yes
182192293	chr1	47164852	47164891	20	40	2.000000	no	no	yes	no	no	no	no	yes	no	yes
182192299	chr1	47184439	47184480	22	42	1.909091	no	no	yes	no	no	no	no	yes	yes	yes
182192306	chr1	47223076	47223107	16	32	2.125000	no	no	yes	no	no	yes	no	yes	no	yes
182192307	chr1	47223709	47223752	21	44	2.095238	no	no	yes	no	no	yes	no	yes	no	yes
182192309	chr1	47225413	47225440	7	28	4.000000	yes	no	yes	no	no	no	yes	yes	no	yes
182192310	chr1	47230060	47230107	22	48	2.272727	yes	yes	yes	no	no	no	no	yes	yes	yes
182192311	chr1	47230515	47230562	24	48	2.000000	yes	yes	yes	no	no	no	no	yes	yes	yes

182192338	chr1	47293708	47293754	19	47	2.600000	no	no	yes	no	no	no	no	yes	no	yes
182192339	chr1	47293736	47293778	22	43	1.954545	no	no	yes	no	no	no	no	yes	no	yes
182192352	chr1	47315748	47315782	18	35	1.944444	no	no	no	no	no	no	no	yes	no	no
182192364	chr1	47336440	47336495	29	56	1.965517	no	no	yes	no	no	no	no	yes	no	yes
182192367	chr1	47338496	47338591	20	96	5.000000	no	no	yes	no	no	no	no	yes	no	yes
182192385	chr1	47364637	47364687	23	51	2.304348	no	no	yes	no	no	no	no	no	no	yes
182192407	chr1	47415903	47415938	15	36	2.400000	yes	no	no	no	no	no	no	yes	yes	no
182192408	chr1	47423595	47423622	13	28	2.153846	no	no	no	no	no	no	no	yes	no	no
182192416	chr1	47435707	47435738	15	32	2.200000	yes	no	no	no	no	no	no	yes	yes	no
182192417	chr1	47438433	47438466	18	34	1.944444	no	no	no	yes	no	no	yes	yes	yes	yes
182192422	chr1	47447594	47447642	13	49	3.769231	no	no	no	no	no	no	no	yes	no	no
182192425	chr1	47458466	47458491	13	26	2.000000	no	no	no	no	no	no	no	no	no	no
182192434	chr1	47467986	47468075	46	90	1.956522	no	no	no	no	no	no	no	no	no	no
182192437	chr1	47472584	47472620	15	37	2.533333	no	no	no	no	no	no	no	no	no	no
182192444	chr1	47482912	47482971	30	60	2.033333	no	no	no	no	no	yes	no	yes	yes	no
182192459	chr1	47521833	47521881	25	49	1.960000	no	no	no	no	no	no	no	yes	yes	no
182192460	chr1	47528516	47528550	18	35	1.944444	no	no	no	no	no	no	no	no	no	no
182192462	chr1	47533176	47533212	19	37	2.000000	no	no	no	no	no	no	no	yes	yes	no
182192463	chr1	47533627	47533699	39	73	1.923077	no	no	no	no	no	no	no	yes	yes	no
182192465	chr1	47544612	47544643	16	32	2.000000	no	no	no	no	no	no	no	no	no	no
182192474	chr1	47571677	47571736	31	60	1.935484	no	no	no	no	no	no	no	yes	no	no
182192483	chr1	47588266	47588291	12	26	2.166667	no	no	no	no	no	no	no	yes	yes	no
182192488	chr1	47591378	47591450	31	73	2.354839	no	no	no	no	no	no	no	no	no	no
182192519	chr1	47688251	47688288	18	38	2.111111	yes	no	no	no	no	no	no	yes	no	no
182192522	chr1	47689772	47689835	24	64	2.708333	no	no	yes	no	no	no	no	no	no	yes

182192527	chr1	47694433	47694486	18	54	2.947368	no	no	yes	no	no	no	no	no	no	yes
182192534	chr1	47708428	47708632	28	205	7.321429	no	no	no	no	no	no	no	yes	no	no
182192539	chr1	47730151	47730183	16	33	2.187500	no	no	no	no	no	no	no	no	no	no
182192541	chr1	47731831	47732010	89	180	2.022472	no	no	no	no	no	no	no	no	no	no
182192544	chr1	47736839	47736882	17	44	2.588235	no	no	no	no	no	no	no	no	no	no
182192554	chr1	47771051	47771116	27	66	2.444444	no	no	yes	no	no	no	no	no	no	yes
182192555	chr1	47777204	47777228	12	25	2.083333	no	no	yes	no	no	no	no	no	no	yes
182192559	chr1	47802210	47802248	18	39	2.166667	no	no	yes	no	no	no	no	no	no	yes
182192565	chr1	47818239	47818270	17	32	1.941176	no	no	yes	no	no	no	no	no	no	yes
182192569	chr1	47827700	47827892	64	193	3.000000	no	no	yes	no	no	no	no	yes	yes	yes
182192579	chr1	47856384	47856420	20	37	1.950000	no	no	yes	no	no	no	no	no	no	yes
182192586	chr1	47883301	47883359	29	59	2.034483	no	no	yes	no	no	no	no	yes	no	yes
182192609	chr1	47949094	47949129	19	36	1.947368	no	no	yes	no	no	no	no	no	no	yes
182192611	chr1	47953061	47953091	16	31	1.937500	no	no	yes	no	no	no	no	no	no	yes
182192614	chr1	47965195	47965250	22	56	2.590909	no	no	yes	no	no	no	no	yes	yes	yes
182192625	chr1	47997370	47997398	15	29	1.933333	yes	no	no	no	yes	no	yes	yes	no	yes
182192628	chr1	47997589	47997626	12	38	3.250000	yes	no	no	no	no	no	yes	yes	yes	no
182192629	chr1	48001329	48001381	22	53	2.409091	no	no	no	no	no	no	no	no	no	no
182192632	chr1	48011306	48011357	27	52	1.925926	no	no	no	no	no	no	no	no	no	no
182192634	chr1	48016471	48016506	16	36	2.250000	no	no	no	no	no	no	no	no	no	no
182192636	chr1	48032914	48032960	18	47	2.611111	no	no	no	no	no	no	no	no	no	no
182192638	chr1	48037505	48037554	26	50	1.961538	no	no	no	no	no	no	no	no	yes	no
182192641	chr1	48053257	48053339	34	83	2.470588	yes	no	yes	no	no	no	no	no	no	yes
182192644	chr1	48079117	48079180	30	64	2.133333	no	no	yes	no	no	no	no	no	no	yes
182192649	chr1	48085958	48086056	33	99	3.000000	no	no	yes	no	no	no	no	no	yes	yes

182192665	chr1	48157443	48157503	33	61	1.909091	no	no	yes	no	no	no	no	no	no	yes
182192673	chr1	48183161	48183196	14	36	2.571429	yes	no	yes	no	no	no	no	no	no	yes
182192696	chr1	48221498	48221569	34	72	2.117647	no	no	no	no	no	no	no	no	no	no
182192697	chr1	48225260	48225467	103	208	2.019418	no	no	yes	no	no	no	no	yes	no	yes
182192699	chr1	48226962	48227006	18	45	2.500000	no	no	yes	no	no	yes	no	yes	no	yes
182192702	chr1	48227211	48227353	71	143	2.126760	no	no	yes	no	no	yes	no	yes	no	yes
182192703	chr1	48227362	48227589	71	228	3.263889	no	no	yes	no	no	yes	no	yes	no	yes
182192704	chr1	48227649	48227689	20	41	2.150000	no	no	yes	no	no	yes	no	yes	no	yes
182192726	chr1	48244712	48244847	22	136	6.125000	no	no	yes	no	no	no	no	no	no	yes
182192728	chr1	48262696	48262741	24	46	2.000000	no	no	no	no	yes	no	no	no	no	yes
182192742	chr1	48307345	48307413	28	69	2.535714	no	no	yes	no	no	no	no	no	no	yes
182192751	chr1	48347524	48347556	13	33	2.615385	no	no	yes	no	no	no	no	no	no	yes
182192753	chr1	48358322	48358378	28	57	2.035714	no	no	yes	no	no	no	no	no	no	yes
182192761	chr1	48379784	48379945	77	162	2.129870	no	no	yes	no	no	no	no	no	no	yes
182192762	chr1	48381546	48381586	21	41	1.952381	no	no	yes	no	no	no	no	no	no	yes
182192780	chr1	48411314	48411355	23	42	1.956522	no	no	yes	no	no	yes	no	no	no	yes
182192816	chr1	48444976	48445008	16	33	2.125000	no	no	yes	no	no	no	no	no	no	yes
182192818	chr1	48445874	48445907	15	34	2.266667	no	no	yes	no	no	no	no	no	no	yes
182192825	chr1	48463319	48463352	16	34	2.125000	no	no	yes	no	no	no	no	no	no	yes
182192841	chr1	48483304	48484065	27	762	28.838709	no	no	no	no	no	no	no	no	no	no
182192845	chr1	48489112	48489155	21	44	2.095238	no	no	no	no	no	no	no	yes	no	no
182192850	chr1	48521459	48521538	37	80	2.236842	no	no	no	no	no	no	no	no	no	no
182192860	chr1	48549454	48549496	12	43	3.583333	no	no	yes	no	no	no	no	no	no	yes
182192863	chr1	48552030	48552081	19	52	2.750000	yes	no	yes	no	no	no	no	no	no	yes
182192872	chr1	48573659	48573696	20	38	1.900000	no	no	yes	no	no	no	no	no	no	yes

182192873	chr1	48577780	48577819	13	40	3.076923	no	no	yes	no	no	no	no	no	yes	yes
182192875	chr1	48582264	48582304	18	41	2.388889	no	no	yes	no	no	no	no	yes	no	yes
182192895	chr1	48608642	48608681	19	40	2.105263	no	no	yes	no	no	no	no	no	no	yes
182192899	chr1	48625137	48625200	28	64	2.285714	no	no	yes	no	no	no	no	no	no	yes
182192907	chr1	48673633	48673688	27	56	2.074074	no	no	yes	no	no	no	no	yes	no	yes
182192915	chr1	48699905	48699952	14	48	3.333333	no	no	yes	no	no	no	no	no	no	yes
182192923	chr1	48711717	48711783	32	67	2.093750	no	no	yes	no	no	no	no	no	no	yes
182192933	chr1	48742405	48742441	19	37	1.947368	no	no	yes	no	no	no	no	no	no	yes
182192934	chr1	48746421	48746460	21	40	1.904762	no	no	yes	no	no	no	no	no	no	yes
182192942	chr1	48787452	48787496	23	45	2.000000	no	no	yes	no	no	no	no	no	no	yes
182192943	chr1	48787597	48787628	13	32	2.538461	no	no	yes	no	no	no	no	no	no	yes
182192949	chr1	48810095	48810123	8	29	3.625000	no	no	yes	no	no	no	no	no	no	yes
182192951	chr1	48816103	48816139	10	37	3.700000	no	no	yes	no	no	no	no	no	no	yes
182192952	chr1	48818011	48818223	106	213	2.009434	no	yes	yes	yes	no	no	no	yes	no	yes
182192963	chr1	48870051	48870087	18	37	2.055556	no	no	yes	no	no	no	no	yes	no	yes
182192979	chr1	48918267	48918302	16	36	2.250000	no	no	yes	no	no	no	no	yes	yes	yes
182192984	chr1	48926853	48926883	15	31	2.066667	no	no	yes	no	no	no	no	no	no	yes
182192987	chr1	48928994	48929048	29	55	1.931034	no	no	yes	no	no	no	no	no	no	yes
182192991	chr1	48932978	48933011	18	34	1.944444	no	no	yes	no	no	no	no	no	no	yes
182192996	chr1	48951303	48951345	19	43	2.263158	no	no	yes	no	no	no	no	yes	yes	yes
182192997	chr1	48956919	48956948	15	30	2.000000	no	no	yes	no	no	no	no	no	no	yes
182193004	chr1	48965061	48965105	23	45	1.956522	no	no	yes	no	no	no	no	no	no	yes
182193008	chr1	48969073	48969113	20	41	2.050000	no	no	yes	no	no	no	no	no	no	yes
182193018	chr1	49018276	49018341	32	66	2.093750	no	no	yes	no	no	no	no	no	no	yes
182193022	chr1	49042136	49042161	13	26	2.000000	no	no	yes	no	no	no	no	no	no	yes

182193023	chr1	49050595	49050663	22	69	3.136364	no	no	yes	no	no	no	no	no	no	yes
182193032	chr1	49115380	49115454	38	75	1.973684	no	no	yes	no	no	no	no	no	no	yes
182193038	chr1	49123972	49124001	15	30	2.000000	no	no	yes	no	no	no	no	no	no	yes
182193041	chr1	49134992	49135025	13	34	2.615385	no	no	yes	no	no	no	no	no	no	yes
182193049	chr1	49170136	49170334	27	199	7.392857	no	no	yes	no	no	no	no	no	no	yes
182193053	chr1	49176133	49176171	18	39	2.166667	no	no	yes	no	no	no	no	yes	no	yes
182193055	chr1	49183846	49183883	20	38	1.900000	no	no	yes	no	no	no	no	yes	no	yes
182193057	chr1	49192245	49192280	12	36	3.083333	no	no	yes	no	no	no	no	no	no	yes
182193070	chr1	49233765	49233793	13	29	2.307692	no	no	yes	no	no	no	no	no	no	yes
182193074	chr1	49243019	49243080	24	62	2.583333	no	no	yes	no	no	no	no	no	no	yes
182193076	chr1	49245142	49245195	12	54	4.500000	no	no	yes	no	no	no	no	no	no	yes
182193083	chr1	49296623	49296655	17	33	1.941176	no	no	yes	no	no	no	no	no	no	yes
182193090	chr1	49316916	49316953	19	38	2.052632	no	no	yes	no	no	no	no	no	no	yes
182193102	chr1	49377964	49378094	26	131	5.038462	no	no	yes	no	no	no	no	no	no	yes
182193109	chr1	49393254	49393282	15	29	1.933333	no	no	yes	no	no	no	no	no	no	yes
182193112	chr1	49395727	49395875	50	149	2.980000	no	no	yes	no	no	no	no	no	no	yes
182193115	chr1	49396331	49396386	26	56	2.153846	no	no	yes	no	no	no	no	no	no	yes
182193116	chr1	49397348	49397383	18	36	2.000000	no	no	yes	no	no	no	no	no	no	yes
182193147	chr1	49521123	49521158	13	36	2.769231	no	no	yes	no	no	no	no	no	no	yes
182193154	chr1	49540719	49540758	19	40	2.105263	no	no	yes	no	no	no	no	no	no	yes
182193176	chr1	49588902	49588944	22	43	2.000000	no	no	yes	no	no	no	no	yes	no	yes
182193181	chr1	49607063	49607114	25	52	2.080000	no	no	yes	no	no	no	no	no	no	yes
182193189	chr1	49634775	49634840	32	66	2.062500	no	no	yes	no	no	no	no	no	no	yes
182193194	chr1	49665961	49666017	29	57	2.000000	no	no	yes	no	no	no	no	no	no	yes
182193203	chr1	49696040	49696078	19	39	2.052632	no	no	yes	no	no	no	no	no	no	yes

182193211	chr1	49720171	49720202	7	32	4.571429	no	no	yes	no	no	no	no	no	no	yes
182193225	chr1	49777507	49777535	13	29	2.230769	no	no	yes	no	no	no	no	no	no	yes
182193236	chr1	49817573	49817609	19	37	1.947368	no	no	yes	no	no	no	no	yes	no	yes
182193242	chr1	49825295	49825333	20	39	1.950000	no	no	yes	no	no	no	no	no	no	yes
182193245	chr1	49839744	49839802	31	59	1.903226	no	no	yes	no	no	no	no	no	no	yes
182193246	chr1	49841950	49842020	36	71	1.972222	no	no	yes	no	no	no	no	no	no	yes
182193248	chr1	49842465	49842530	32	66	2.062500	no	no	yes	no	no	no	no	no	no	yes
182193266	chr1	49887133	49887259	56	127	2.298246	no	no	yes	no	no	no	no	no	no	yes
182193290	chr1	49950566	49950608	18	43	2.388889	no	no	yes	no	no	no	no	no	no	yes
182193293	chr1	49960910	49960946	19	37	1.947368	no	no	yes	no	no	no	no	no	no	yes
182193298	chr1	49976401	49976442	23	42	1.913043	no	no	yes	no	no	no	no	no	no	yes
182193299	chr1	49977146	49977388	23	243	10.541667	no	no	yes	no	no	no	no	no	no	yes
182193303	chr1	50001007	50001062	26	56	2.153846	no	no	yes	no	no	no	no	no	no	yes
182193304	chr1	50001185	50001219	17	35	2.058824	no	no	yes	no	no	no	no	no	no	yes
182193306	chr1	50001418	50001476	18	59	3.277778	no	no	yes	no	no	no	no	no	no	yes
182193309	chr1	50001466	50001530	30	65	2.166667	no	no	yes	no	no	no	no	no	no	yes
182193313	chr1	50002942	50002973	10	32	3.200000	no	no	yes	no	no	no	no	no	no	yes
182193317	chr1	50018114	50018152	20	39	2.000000	no	no	yes	no	no	yes	no	no	no	yes
182193321	chr1	50023920	50023952	15	33	2.200000	yes	no	no	no	yes	no	yes	yes	yes	yes
182193328	chr1	50065324	50065376	26	53	2.038461	no	no	yes	no	no	no	no	no	no	yes
182193336	chr1	50086741	50086771	16	31	2.000000	no	no	yes	no	no	no	no	no	yes	yes
182193339	chr1	50101461	50101502	15	42	2.933333	no	no	yes	no	no	no	no	no	no	yes
182193347	chr1	50115021	50115085	18	65	3.550000	no	yes	yes	no	no	no	no	no	no	yes
182193352	chr1	50127657	50127686	15	30	2.000000	no	no	yes	no	no	no	no	yes	yes	yes
182193357	chr1	50138350	50138418	35	69	1.971429	no	no	yes	no	no	no	no	no	no	yes

182193358	chr1	50150146	50150183	21	38	1.952381	no	no	yes	no	no	no	no	no	no	yes
182193359	chr1	50152850	50152887	19	38	2.000000	no	no	yes	no	no	no	no	no	no	yes
182193361	chr1	50155976	50156126	64	151	2.500000	no	no	yes	no	no	no	no	no	yes	yes
182193375	chr1	50200427	50200468	22	42	1.954545	no	no	yes	no	no	no	no	no	no	yes
182193376	chr1	50200501	50200543	16	43	2.687500	no	no	yes	no	no	no	no	no	no	yes
182193381	chr1	50246363	50246396	15	34	2.266667	no	no	yes	no	no	no	no	no	yes	yes
182193385	chr1	50250549	50250594	15	46	3.125000	no	no	yes	no	no	no	no	no	no	yes
182193388	chr1	50256142	50256231	45	90	2.000000	no	yes	yes	no	no	no	no	no	no	yes
182193399	chr1	50287545	50287584	19	40	2.105263	no	no	yes	no	no	no	no	no	no	yes
182193400	chr1	50289167	50289205	19	39	2.052632	no	no	yes	no	no	no	no	no	no	yes
182193403	chr1	50294375	50294404	11	30	2.666667	no	no	yes	no	no	yes	no	yes	no	yes
182193407	chr1	50304388	50304434	24	47	1.958333	no	no	yes	no	no	no	no	no	no	yes
182193411	chr1	50327013	50327086	37	74	2.000000	no	no	no	no	no	yes	no	no	no	no
182193414	chr1	50332944	50332970	12	27	2.250000	no	no	no	no	no	no	no	yes	no	no
182193416	chr1	50341097	50341137	10	41	4.000000	no	no	no	no	no	no	no	no	yes	no
182193417	chr1	50349124	50349178	24	55	2.333333	no	no	no	no	no	no	no	yes	no	no
182193444	chr1	50394734	50394798	33	65	2.000000	no	no	no	no	no	no	no	no	no	no
182193451	chr1	50417991	50418033	18	43	2.368421	no	yes	no	no	no	no	no	yes	no	yes
182193453	chr1	50420521	50420569	17	49	2.882353	no	no	yes	no	no	no	yes	yes	yes	yes
182193454	chr1	50424748	50424785	18	38	2.111111	no	no	yes	no	no	no	yes	yes	yes	yes
182193455	chr1	50426005	50426056	26	52	2.000000	no	no	no	no	no	yes	no	yes	yes	no
182193475	chr1	50487055	50487101	20	47	2.350000	no	no	yes	no	no	no	no	no	no	yes
182193479	chr1	50490717	50490760	22	44	2.045455	no	no	yes	no	no	no	no	yes	yes	yes
182193481	chr1	50513527	50513590	29	64	2.233333	no	no	yes	no	no	no	no	no	no	yes
182193482	chr1	50517146	50517181	18	36	2.000000	no	no	yes	no	no	no	no	no	no	yes

182193485	chr1	50526443	50526480	20	38	1.950000	no	no	yes	no	no	no	no	no	no	yes
182193488	chr1	50530063	50530094	17	32	1.941176	no	no	yes	no	no	no	no	no	no	yes
182193490	chr1	50534849	50534909	30	61	2.033333	no	no	yes	no	no	no	no	no	no	yes
182193491	chr1	50535313	50535351	11	39	3.545455	no	no	yes	no	no	no	no	no	no	yes
182193496	chr1	50551288	50551333	22	46	2.136364	no	no	yes	no	no	no	no	no	no	yes
182193501	chr1	50555217	50555297	30	81	2.700000	no	no	yes	no	no	no	no	no	no	yes
182193507	chr1	50555935	50556016	39	82	2.102564	no	no	yes	no	no	no	no	no	no	yes
182193509	chr1	50558203	50558269	25	67	2.680000	no	no	yes	no	no	no	no	no	no	yes
182193512	chr1	50573812	50573840	15	29	1.933333	no	no	yes	no	no	no	no	no	yes	yes
182193515	chr1	50580554	50580592	21	39	1.904762	no	no	yes	no	no	no	no	no	no	yes
182193533	chr1	50632453	50632494	22	42	1.909091	no	no	yes	no	no	no	no	no	no	yes
182193534	chr1	50632985	50633013	15	29	2.000000	no	no	yes	no	no	no	no	no	no	yes
182193541	chr1	50653988	50654023	16	36	2.250000	no	no	yes	no	no	no	no	no	no	yes
182193551	chr1	50678274	50678315	20	42	2.150000	no	no	yes	no	no	no	no	no	yes	yes
182193552	chr1	50678434	50678462	15	29	2.000000	no	no	yes	no	no	no	no	no	no	yes
182193555	chr1	50680318	50680395	39	78	2.000000	no	no	yes	no	no	no	no	no	no	yes
182193558	chr1	50687239	50687275	19	37	2.000000	no	no	yes	no	no	no	no	no	no	yes
182193567	chr1	50706667	50706704	11	38	3.454545	yes	no	yes	no	no	no	no	no	no	yes
182193572	chr1	50709556	50709630	38	75	1.973684	no	no	yes	no	no	no	no	no	no	yes
182193587	chr1	50739254	50739385	32	132	4.125000	no	no	yes	no	no	no	no	yes	no	yes
182193588	chr1	50739382	50739433	26	52	2.000000	no	no	yes	no	no	no	no	yes	no	yes
182193611	chr1	50805004	50805046	23	43	1.913043	no	no	yes	no	no	no	no	no	no	yes
182193622	chr1	50819821	50819849	14	29	2.071429	no	no	yes	no	no	no	no	no	no	yes
182193625	chr1	50819870	50819927	31	58	1.903226	no	no	yes	no	no	no	no	yes	no	yes
182193628	chr1	50823382	50823419	20	38	1.900000	no	no	yes	no	no	no	no	no	no	yes

182193640	chr1	50838359	50838430	38	72	1.921053	no	no	yes	no	no	no	no	no	no	yes
182193641	chr1	50838409	50838454	24	46	1.916667	no	no	yes	no	no	no	no	no	no	yes
182193645	chr1	50844924	50844969	22	46	2.090909	no	no	yes	no	no	no	no	yes	no	yes
182193646	chr1	50845736	50845774	14	39	2.857143	yes	no	yes	no	no	no	no	no	no	yes
182193648	chr1	50849576	50849631	30	56	1.933333	no	no	yes	no	no	no	no	no	no	yes
182193649	chr1	50849638	50849693	24	56	2.320000	no	no	yes	no	no	no	no	no	no	yes
182193654	chr1	50883194	50883236	23	43	1.956522	no	no	yes	no	no	no	no	no	no	yes
182193663	chr1	50900879	50901012	66	134	2.030303	no	no	yes	no	no	no	no	no	no	yes
182193676	chr1	50931262	50931336	39	75	1.923077	no	no	yes	no	no	no	no	yes	no	yes
182193680	chr1	50941748	50941794	23	47	2.043478	no	no	yes	no	no	no	no	no	no	yes
182193690	chr1	50959925	50959966	12	42	3.500000	yes	no	no	no	yes	no	yes	yes	no	yes
182193695	chr1	50965977	50966085	53	109	2.056604	no	no	yes	no	no	no	no	yes	no	yes
182193763	chr1	51088590	51088647	23	58	2.565217	no	no	no	no	no	no	no	yes	yes	no
182193773	chr1	51118423	51118485	32	63	1.968750	no	no	yes	no	no	no	no	no	no	yes
182193800	chr1	51176067	51176115	19	49	2.578947	no	no	no	no	no	no	no	no	no	no
182193805	chr1	51185197	51185232	17	36	2.117647	no	no	no	no	no	no	no	no	no	no
182193818	chr1	51206756	51206793	14	38	2.666667	no	no	yes	no	no	no	no	no	no	yes
182193828	chr1	51218049	51218092	10	44	4.250000	no	no	yes	no	no	no	no	no	no	yes
182193837	chr1	51236366	51236394	12	29	2.416667	no	yes	no	no	yes	no	yes	yes	yes	yes
182193841	chr1	51242472	51242504	17	33	1.941176	no	no	yes	no	no	no	no	yes	no	yes
182193855	chr1	51270626	51270801	90	176	1.966667	no	no	yes	no	no	no	no	no	no	yes
182193860	chr1	51280484	51280604	63	121	1.984127	no	no	no	no	no	no	no	yes	yes	no
182193864	chr1	51296027	51296061	18	35	1.944444	no	yes	yes	yes	no	no	no	yes	no	yes
182193873	chr1	51322223	51322261	18	39	2.166667	yes	no	yes	no	yes	no	no	yes	yes	yes
182193879	chr1	51344282	51344354	35	73	2.114286	no	no	yes	no	no	no	no	yes	no	yes

182193898	chr1	51402963	51403022	27	60	2.214286	no	no	yes	no	no	yes	no	no	no	yes
182193921	chr1	51463616	51463647	17	32	1.941176	no	no	yes	no	no	yes	no	yes	no	yes
182193931	chr1	51506813	51506858	20	46	2.300000	no	no	yes	no	no	no	no	yes	yes	yes
182193938	chr1	51519378	51519416	14	39	2.866667	yes	no	yes	no	no	no	no	yes	yes	yes
182193939	chr1	51522191	51522215	13	25	1.923077	no	no	yes	no	no	no	no	no	no	yes
182193941	chr1	51523101	51523139	19	39	2.105263	no	no	yes	no	no	no	no	no	no	yes
182193943	chr1	51526585	51526629	22	45	2.045455	no	no	yes	no	no	no	no	yes	yes	yes
182193949	chr1	51532975	51533041	18	67	3.777778	no	no	yes	no	no	no	no	yes	no	yes
182193961	chr1	51556803	51556858	22	56	2.545455	no	no	yes	no	no	no	no	no	no	yes
182193964	chr1	51558839	51558882	20	44	2.200000	no	no	yes	no	no	no	no	yes	yes	yes
182193996	chr1	51614222	51614305	40	84	2.100000	no	no	yes	no	yes	no	no	no	no	yes
182194000	chr1	51616997	51617024	11	28	2.545455	yes	no	no	no	yes	no	yes	yes	yes	yes
182194016	chr1	51662305	51662356	20	52	2.700000	no	no	yes	no	no	no	no	no	no	yes
182194021	chr1	51692618	51692659	22	42	1.909091	no	no	yes	no	no	no	no	yes	no	yes
182194041	chr1	51715054	51715082	15	29	2.000000	no	no	yes	no	no	no	no	no	no	yes
182194043	chr1	51721232	51721268	19	37	2.000000	no	no	yes	no	no	no	no	no	no	yes
182194046	chr1	51729809	51729833	9	25	2.777778	yes	yes	yes	no	yes	no	yes	yes	no	yes
182194047	chr1	51729841	51729881	21	41	1.952381	yes	yes	yes	no	yes	no	yes	yes	no	yes
182194054	chr1	51745372	51745406	18	35	2.000000	yes	yes	yes	no	no	no	no	yes	no	yes
182194057	chr1	51757593	51757633	17	41	2.411765	no	no	yes	no	no	yes	no	no	no	yes
182194062	chr1	51776362	51776390	15	29	1.933333	no	no	yes	no	no	no	no	no	no	yes
182194063	chr1	51786903	51786936	18	34	1.944444	no	no	yes	no	no	no	no	yes	yes	yes
182194066	chr1	51790985	51791037	26	53	2.038461	no	yes	yes	yes	yes	no	no	yes	no	yes
182194067	chr1	51792823	51792862	20	40	2.050000	no	no	yes	no	no	no	no	yes	no	yes
182194070	chr1	51804412	51805074	30	663	22.100000	no	no	yes	no	no	no	no	no	no	yes

182194072	chr1	51823131	51823212	43	82	1.930233	no	no	yes	no	no	no	no	no	no	yes
182194075	chr1	51840308	51840358	18	51	2.833333	no	no	no	yes	yes	no	no	yes	no	yes
182194076	chr1	51840380	51840432	21	53	2.523809	no	no	no	yes	yes	no	no	yes	no	yes
182194095	chr1	51859736	51859783	25	48	1.920000	yes	yes	yes	no	no	no	no	yes	no	yes
182194098	chr1	51866943	51866979	19	37	1.947368	no	no	yes	no	no	no	no	yes	no	yes
182194099	chr1	51867235	51867267	17	33	2.000000	no	no	yes	no	no	no	no	yes	yes	yes
182194100	chr1	51870781	51870810	14	30	2.142857	no	no	yes	no	no	no	no	yes	no	yes
182194101	chr1	51870802	51870851	23	50	2.260870	no	no	yes	no	no	no	no	yes	no	yes
182194107	chr1	51891256	51891334	38	79	2.078947	no	no	no	no	no	no	no	no	no	no
182194111	chr1	51906838	51906900	18	63	3.611111	no	no	no	no	no	no	no	yes	no	no
182194117	chr1	51935176	51935221	25	46	1.960000	no	no	yes	no	no	no	no	no	no	yes
182194118	chr1	51935872	51935912	21	41	1.952381	no	no	yes	no	no	no	no	yes	no	yes
182194123	chr1	51941872	51941911	20	40	2.000000	no	no	yes	no	no	no	no	yes	yes	yes
182194130	chr1	51958014	51958058	23	45	2.000000	no	no	yes	no	no	no	no	yes	yes	yes
182194131	chr1	51960513	51960547	13	35	2.692308	no	no	yes	no	no	no	no	yes	yes	yes
182194149	chr1	51989869	51990006	74	138	1.878378	no	no	yes	no	no	no	no	yes	yes	yes
182194159	chr1	52014359	52014436	23	78	3.434783	no	no	no	no	no	no	no	no	no	no
182194193	chr1	52064478	52064514	19	37	1.947368	no	no	yes	no	no	no	no	yes	no	yes
182194195	chr1	52070282	52070351	17	70	4.176471	no	no	yes	no	no	no	no	no	no	yes
182194230	chr1	52091195	52091236	21	42	2.000000	no	no	no	no	no	yes	no	yes	yes	no
182194245	chr1	52114068	52114146	42	79	1.904762	no	no	no	no	no	no	no	no	no	no
182194246	chr1	52115118	52115157	20	40	2.000000	no	no	no	no	no	no	no	yes	no	no
182194255	chr1	52121237	52121267	16	31	1.937500	no	no	no	no	no	no	no	no	no	no
182194265	chr1	52139828	52139868	20	41	2.050000	no	no	no	no	no	no	no	yes	no	no
182194273	chr1	52162112	52162154	18	43	2.388889	no	no	yes	no	no	yes	no	no	no	yes

182194274	chr1	52162788	52162829	18	42	2.333333	no	no	yes	no	yes	no	no	no	no	yes
182194276	chr1	52164554	52164633	42	80	1.904762	no	no	yes	no	no	no	no	yes	no	yes
182194299	chr1	52206219	52206281	30	63	2.100000	no	no	yes	no	no	no	no	no	no	yes
182194317	chr1	52254553	52254591	20	39	1.950000	no	no	yes	no	no	yes	no	no	no	yes
182194326	chr1	52277191	52277259	18	69	3.947368	no	no	yes	no	no	no	no	no	no	yes
182194330	chr1	52282347	52282388	21	42	2.095238	no	no	yes	no	no	no	no	no	no	yes
182194332	chr1	52295196	52295258	32	63	1.968750	yes	no	yes	no	no	no	no	no	no	yes
182194337	chr1	52304591	52304628	18	38	2.111111	no	no	yes	no	no	no	no	no	no	yes
182194353	chr1	52346343	52346393	26	51	2.000000	no	yes	yes	no	no	no	no	no	no	yes
182194361	chr1	52370908	52370949	20	42	2.200000	no	no	no	no	no	no	no	no	no	no
182194374	chr1	52401738	52401770	16	33	2.062500	no	no	yes	no	no	no	no	yes	no	yes
182194380	chr1	52410120	52410171	16	52	3.250000	no	no	yes	no	no	no	no	no	no	yes
182194390	chr1	52436913	52436958	21	46	2.190476	no	no	no	yes	yes	no	no	yes	yes	yes
182194411	chr1	52484567	52484611	24	45	1.916667	no	no	yes	no	no	no	no	no	no	yes
182194414	chr1	52494791	52494842	26	52	2.076923	no	no	yes	no	no	no	no	yes	no	yes
182194421	chr1	52516320	52516349	14	30	2.142857	no	no	yes	no	no	no	no	yes	yes	yes
182194422	chr1	52517427	52517473	24	47	1.958333	no	no	yes	no	no	no	no	yes	no	yes
182194423	chr1	52522275	52522307	14	33	2.357143	no	no	yes	no	no	no	no	no	no	yes
182194425	chr1	52525892	52525962	30	71	2.366667	yes	no	yes	yes	yes	yes	no	yes	yes	yes
182194427	chr1	52529458	52529504	24	47	1.958333	no	no	yes	no	no	no	no	no	no	yes
182194428	chr1	52531389	52531415	12	27	2.250000	no	no	yes	no	no	no	no	yes	yes	yes
182194445	chr1	52569968	52570010	18	43	2.444444	no	no	no	no	no	no	no	yes	yes	no
182194464	chr1	52620193	52620243	19	51	2.684211	no	no	no	no	no	no	no	no	no	no
182194505	chr1	52687463	52687551	44	89	2.022727	no	yes	no	no	no	yes	no	yes	no	yes
182194538	chr1	52726473	52726508	18	36	2.000000	no	no	no	no	yes	no	yes	yes	yes	yes

182194557	chr1	52774762	52774820	29	59	2.034483	no	no	yes	no	no	no	no	no	no	yes
182194575	chr1	52795985	52796017	17	33	1.941176	no	no	yes	no	no	no	no	yes	no	yes
182194601	chr1	52842122	52842194	38	73	1.947368	yes	no	no	no	no	no	no	yes	yes	no
182194603	chr1	52842585	52842612	9	28	3.111111	yes	no	no	no	yes	no	no	yes	no	yes
182194616	chr1	52860670	52860703	17	34	2.000000	no	no	yes	no	no	no	no	yes	yes	yes
182194632	chr1	52875893	52875919	14	27	1.928571	no	no	yes	no	no	no	no	no	yes	yes
182194646	chr1	52898719	52898788	35	70	2.000000	no	no	yes	no	yes	yes	no	yes	yes	yes
182194659	chr1	52947462	52947515	23	54	2.434783	no	no	yes	no	no	no	no	yes	yes	yes
182194661	chr1	52955044	52955085	18	42	2.333333	no	no	yes	no	no	no	no	no	no	yes
182194663	chr1	52957198	52957231	7	34	4.857143	no	no	yes	no	no	no	no	no	no	yes
182194699	chr1	53033161	53033232	28	72	2.571429	no	no	yes	no	no	no	no	no	no	yes
182194701	chr1	53037382	53037423	22	42	1.909091	no	no	yes	no	no	no	no	no	yes	yes
182194704	chr1	53042412	53042452	22	41	1.909091	no	no	yes	no	no	no	no	yes	no	yes
182194715	chr1	53086957	53087017	29	61	2.103448	no	no	no	no	no	yes	no	yes	yes	no
182194725	chr1	53095716	53095759	19	44	2.368421	no	no	yes	no	no	no	no	no	no	yes
182194727	chr1	53096431	53096615	101	185	1.851485	no	no	yes	no	no	no	no	yes	no	yes
182194730	chr1	53096657	53096707	21	51	2.428571	no	no	yes	no	no	no	no	yes	no	yes
182194736	chr1	53098809	53098953	61	145	2.442623	no	no	yes	no	no	no	no	no	no	yes
182194739	chr1	53100018	53100056	19	39	2.210526	no	no	yes	no	no	no	no	yes	no	yes
182194740	chr1	53101208	53101268	30	61	2.064516	no	no	yes	no	no	no	no	no	no	yes
182194743	chr1	53113984	53114021	20	38	1.950000	yes	no	yes	no	no	no	no	yes	no	yes
182194745	chr1	53115869	53115900	8	32	4.000000	no	yes	yes	no	no	no	no	yes	no	yes
182194747	chr1	53124626	53124671	23	46	2.043478	no	no	yes	no	no	no	no	yes	yes	yes
182194748	chr1	53127478	53127519	22	42	2.000000	no	no	yes	no	no	no	no	no	yes	yes
182194766	chr1	53173623	53173682	26	60	2.307692	no	no	no	no	no	no	no	yes	yes	no

182194767	chr1	53182411	53182448	19	38	2.052632	no	no	yes	no	no	no	no	yes	yes	yes
182194775	chr1	53200500	53200545	24	46	1.916667	no	yes	yes	no	no	no	no	no	no	yes
182194789	chr1	53250571	53250649	35	79	2.285714	no	no	yes	no	no	yes	no	yes	no	yes
182194791	chr1	53256315	53256339	12	25	2.083333	no	no	yes	no	yes	no	no	yes	no	yes
182194792	chr1	53273272	53273321	14	50	3.571429	no	no	yes	no	yes	no	no	no	no	yes
182194793	chr1	53283423	53284323	28	901	34.107143	no	no	yes	no	no	no	yes	yes	yes	yes
182194794	chr1	53293316	53293361	24	46	1.916667	no	no	yes	no	no	no	no	yes	yes	yes
182194795	chr1	53296986	53297077	46	92	2.000000	no	no	yes	no	no	no	no	no	no	yes
182194796	chr1	53299100	53299208	35	109	3.114286	no	no	yes	no	no	no	no	no	no	yes
182194809	chr1	53342683	53342725	20	43	2.250000	no	no	no	no	no	no	no	no	no	no
182194854	chr1	53487070	53487117	17	48	2.941176	no	no	no	no	no	no	no	no	no	no
182194862	chr1	53502006	53502056	24	51	2.166667	no	no	no	no	no	no	no	yes	no	no
182194874	chr1	53516045	53516129	42	85	2.023809	no	no	yes	no	no	no	no	yes	yes	yes
182194877	chr1	53524625	53524660	12	36	3.000000	no	no	yes	no	no	no	no	no	no	yes
182194878	chr1	53525455	53525498	9	44	4.888889	no	no	yes	no	no	no	no	yes	no	yes
182194880	chr1	53530717	53530779	30	63	2.100000	no	no	yes	no	no	no	no	no	no	yes
182194887	chr1	53543227	53543269	21	43	2.047619	no	no	yes	no	no	no	no	yes	yes	yes
182194888	chr1	53546694	53546732	19	39	2.052632	no	no	yes	no	no	no	no	yes	no	yes
182194889	chr1	53551557	53551587	15	31	2.066667	no	no	yes	no	no	no	no	no	yes	yes
182194897	chr1	53556814	53557071	101	258	2.592233	no	no	yes	no	no	no	no	yes	yes	yes
182194899	chr1	53558773	53558806	14	34	2.428571	no	no	yes	no	no	no	no	no	no	yes
182194903	chr1	53579696	53579727	12	32	2.666667	no	no	yes	no	no	no	no	yes	no	yes
182194914	chr1	53628127	53628171	10	45	4.454545	no	no	yes	no	no	no	no	no	no	yes
182194915	chr1	53631958	53631994	10	37	3.636364	no	no	yes	no	no	no	no	no	no	yes
182194922	chr1	53632652	53632721	31	70	2.322581	no	no	yes	no	no	no	no	yes	no	yes

182194925	chr1	53633176	53633221	19	46	2.421053	no	no	yes	no	no	no	no	no	no	yes
182194926	chr1	53633233	53633257	12	25	2.083333	no	no	yes	no	no	no	no	no	no	yes
182194929	chr1	53638531	53638567	16	37	2.312500	no	no	yes	no	no	no	no	no	no	yes
182194935	chr1	53660262	53660301	21	40	2.000000	no	no	yes	no	no	no	no	no	no	yes
182194936	chr1	53663041	53663131	44	91	2.090909	no	no	yes	no	no	no	no	no	yes	yes
182194938	chr1	53684862	53684906	23	45	1.956522	no	no	yes	no	no	no	no	yes	yes	yes
182194942	chr1	53700482	53700537	23	56	2.434783	no	no	yes	no	no	no	no	yes	no	yes
182194956	chr1	53739159	53739200	9	42	4.666667	no	no	no	no	no	no	yes	yes	yes	no
182194967	chr1	53770124	53770310	102	187	1.833333	no	no	yes	no	no	yes	no	yes	no	yes
182194968	chr1	53770606	53770631	13	26	2.000000	no	no	yes	no	no	yes	no	no	no	yes
182194986	chr1	53810295	53810338	21	44	2.095238	no	no	yes	no	no	no	no	no	no	yes
182194988	chr1	53819099	53819140	18	42	2.315789	no	no	yes	no	no	no	no	no	no	yes
182194990	chr1	53822507	53822582	37	76	2.054054	no	no	yes	no	no	no	no	yes	no	yes
182194993	chr1	53828310	53828342	14	33	2.357143	no	no	yes	no	no	no	no	no	no	yes
182195000	chr1	53844133	53844178	16	46	2.875000	no	no	no	no	no	no	no	yes	no	no
182195003	chr1	53855878	53855951	38	74	1.947368	no	no	yes	no	no	no	no	yes	no	yes
182195005	chr1	53860855	53860886	16	32	2.000000	no	no	yes	no	no	no	no	yes	no	yes
182195006	chr1	53861594	53861629	16	36	2.250000	no	no	yes	no	no	no	no	no	no	yes
182195009	chr1	53864202	53864246	20	45	2.250000	no	no	yes	no	no	no	no	no	no	yes
182195010	chr1	53865157	53865206	23	50	2.260870	no	no	yes	no	no	yes	no	no	no	yes
182195018	chr1	53893766	53893807	21	42	2.142857	yes	no	yes	no	no	no	no	no	no	yes
182195020	chr1	53896635	53896662	12	28	2.333333	no	no	yes	no	no	no	no	yes	no	yes
182195028	chr1	53909874	53909908	17	35	2.058824	no	no	yes	no	no	no	no	yes	no	yes
182195029	chr1	53910229	53910266	19	38	2.000000	no	yes	no	no	no	yes	no	yes	yes	yes
182195036	chr1	53939469	53939500	16	32	2.000000	no	no	yes	no	no	yes	no	no	yes	yes

182195037	chr1	53943230	53943275	22	46	2.090909	no	no	yes	no	no	no	no	yes	no	yes
182195039	chr1	53945869	53945924	26	56	2.153846	yes	no	no	no	yes	no	yes	yes	yes	yes
182195040	chr1	53945921	53946072	82	152	1.865854	yes	no	no	no	yes	no	yes	yes	yes	yes
182195042	chr1	53958463	53958498	18	36	2.000000	no	no	yes	no	no	yes	no	no	no	yes
182195057	chr1	54001349	54001413	32	65	2.062500	no	no	no	no	no	no	no	yes	no	no
182195069	chr1	54017965	54018097	17	133	7.823529	no	no	yes	yes	no	no	no	yes	yes	yes
182195071	chr1	54023189	54023274	45	86	1.911111	no	no	no	no	no	no	no	no	no	no
182195077	chr1	54033097	54033142	19	46	2.473684	no	no	yes	no	yes	yes	no	no	no	yes
182195101	chr1	54143395	54143426	16	32	2.000000	no	yes	yes	no	no	yes	no	no	no	yes
182195104	chr1	54150649	54150688	21	40	1.952381	no	no	yes	no	no	no	no	no	no	yes
182195108	chr1	54173744	54173785	22	42	1.909091	no	yes	yes	no	yes	yes	no	yes	yes	yes
182195120	chr1	54230409	54230453	22	45	2.045455	no	no	yes	no	no	no	no	yes	no	yes
182195138	chr1	54239512	54239557	20	46	2.300000	no	no	yes	no	no	yes	no	yes	yes	yes
182195141	chr1	54240080	54240120	20	41	2.050000	no	no	yes	no	no	no	no	yes	no	yes
182195143	chr1	54250382	54250499	40	118	2.950000	no	no	yes	no	no	no	no	yes	yes	yes
182195144	chr1	54252519	54252570	22	52	2.363636	yes	no	yes	no	no	no	no	no	no	yes
182195148	chr1	54258765	54258793	15	29	1.933333	no	no	yes	no	no	no	no	yes	no	yes
182195152	chr1	54273315	54273348	16	34	2.125000	no	no	yes	no	no	no	no	yes	yes	yes
182195153	chr1	54276117	54276175	29	59	2.034483	no	no	yes	no	no	no	no	yes	yes	yes
182195162	chr1	54307191	54307240	17	50	2.888889	no	no	yes	no	no	no	no	no	no	yes
182195168	chr1	54321113	54321189	38	77	2.026316	no	no	yes	no	no	no	no	no	no	yes
182195170	chr1	54332918	54332948	16	31	1.937500	no	no	yes	no	no	no	no	yes	yes	yes
182195174	chr1	54363384	54363427	20	44	2.200000	no	no	yes	no	no	no	no	yes	no	yes
182195175	chr1	54365036	54365075	21	40	1.952381	no	no	yes	no	no	no	no	yes	no	yes
182195176	chr1	54369700	54369797	47	98	2.085106	no	no	yes	no	no	no	no	no	no	yes

182195185	chr1	54395048	54395092	23	45	1.956522	no	no	yes	no	no	no	no	yes	yes	yes
182195187	chr1	54397509	54397566	27	58	2.259259	no	no	yes	no	no	no	no	yes	yes	yes
182195188	chr1	54398709	54398745	14	37	2.642857	no	no	yes	no	no	no	no	no	no	yes
182195190	chr1	54403369	54403412	23	44	1.913043	no	no	yes	no	no	no	no	yes	yes	yes
182195195	chr1	54411081	54411113	15	33	2.200000	no	no	yes	no	no	no	no	yes	yes	yes
182195236	chr1	54476255	54476421	73	167	2.301370	no	no	no	no	no	no	no	yes	yes	no
182195253	chr1	54503866	54503927	23	62	2.739130	no	no	no	no	no	no	no	no	no	no
182195276	chr1	54531188	54531214	14	27	1.928571	no	no	no	no	no	no	no	no	no	no
182195282	chr1	54539702	54539848	63	147	2.343750	no	no	no	no	no	no	no	no	no	no
182195317	chr1	54576757	54576800	18	44	2.444444	no	no	yes	no	no	no	no	yes	no	yes
182195320	chr1	54581322	54581353	16	32	2.000000	no	no	yes	no	no	no	no	yes	yes	yes
182195321	chr1	54582811	54582845	14	35	2.500000	no	no	yes	no	no	no	no	yes	yes	yes
182195322	chr1	54584624	54584653	10	30	2.909091	no	no	yes	no	no	no	no	no	no	yes
182195324	chr1	54594263	54594298	17	36	2.294118	no	no	yes	no	no	no	no	no	no	yes
182195326	chr1	54597675	54597737	32	63	1.968750	no	yes	yes	no	no	no	no	no	yes	yes
182195338	chr1	54622750	54622799	25	50	2.000000	no	no	yes	no	no	no	no	yes	yes	yes
182195357	chr1	54669362	54669386	11	25	2.272727	no	no	yes	no	no	no	no	no	no	yes
182195371	chr1	54705865	54705899	17	35	2.058824	no	no	yes	no	no	no	no	no	yes	yes
182195376	chr1	54716062	54716095	17	34	2.058824	no	yes	yes	no	no	no	yes	yes	yes	yes
182195377	chr1	54718392	54718428	18	37	2.166667	no	no	yes	no	no	yes	no	no	no	yes
182195391	chr1	54792764	54792788	12	25	2.083333	yes	no	yes	no	no	no	no	yes	yes	yes
182195393	chr1	54796714	54796750	18	37	2.111111	no	no	yes	no	no	no	no	yes	no	yes
182195395	chr1	54817758	54817790	15	33	2.266667	no	no	yes	no	no	no	no	no	no	yes
182195402	chr1	54827420	54827459	21	40	1.904762	no	no	yes	no	no	no	no	no	no	yes
182195407	chr1	54848673	54848734	32	62	1.937500	no	no	no	no	no	yes	no	yes	no	no

182195408	chr1	54857317	54857352	19	36	1.947368	no	no	yes	no	no	no	no	yes	yes	yes
182195410	chr1	54866753	54866795	17	43	2.529412	no	no	yes	no	no	no	no	yes	no	yes
182195411	chr1	54868648	54868747	52	100	1.942308	no	no	yes	no	no	no	no	no	no	yes
182195413	chr1	54870823	54870887	31	65	2.096774	no	no	yes	no	no	yes	no	yes	no	yes
182195417	chr1	54887171	54887202	16	32	2.000000	yes	no	yes	no	yes	no	yes	yes	yes	yes
182195427	chr1	54922877	54922922	24	46	1.916667	no	no	no	no	no	no	no	yes	yes	no
182195444	chr1	54936451	54936503	24	53	2.208333	no	no	no	no	no	no	no	no	no	no
182195470	chr1	55010526	55010605	39	80	2.051282	no	yes	no	no	no	no	no	no	no	yes
182195472	chr1	55016215	55016257	18	43	2.388889	no	yes	no	no	no	no	no	yes	yes	yes
182195476	chr1	55019169	55019206	19	38	2.052632	no	no	no	no	no	no	no	yes	no	no
182195483	chr1	55056268	55056303	18	36	2.000000	no	no	yes	no	no	no	no	yes	no	yes
182195486	chr1	55066467	55066495	14	29	2.071429	no	yes	no	no	no	no	no	yes	no	yes
182195488	chr1	55070684	55070741	22	58	2.681818	no	no	yes	no	no	yes	no	yes	no	yes
182195495	chr1	55135725	55135778	25	54	2.160000	no	no	yes	no	no	no	no	no	no	yes
182195496	chr1	55141060	55141100	21	41	2.047619	no	no	yes	no	no	no	no	yes	no	yes
182195497	chr1	55151591	55151630	19	40	2.157895	no	no	yes	no	no	no	no	yes	no	yes
182195498	chr1	55155482	55155506	13	25	1.923077	no	no	yes	no	no	no	no	yes	no	yes
182195501	chr1	55173545	55173604	29	60	2.068965	no	no	yes	no	no	no	no	no	no	yes
182195503	chr1	55176061	55176108	15	48	3.250000	no	no	yes	no	no	no	no	yes	no	yes
182195504	chr1	55176428	55176535	53	108	2.037736	no	no	yes	yes	no	no	no	no	no	yes
182195519	chr1	55217370	55217426	21	57	2.772727	yes	no	no	no	no	no	no	yes	no	no
182195524	chr1	55220900	55220925	12	26	2.166667	no	no	yes	no	no	no	no	yes	no	yes
182195531	chr1	55245269	55245307	18	39	2.166667	no	no	yes	no	no	no	no	no	no	yes
182195554	chr1	55327772	55327807	17	36	2.176471	no	no	no	no	no	no	no	yes	no	no
182195571	chr1	55385598	55385642	24	45	1.916667	no	no	yes	no	no	no	no	yes	no	yes

182195577	chr1	55397054	55397079	13	26	2.000000	no	no	yes	no	no	no	no	no	no	yes
182195580	chr1	55400196	55400238	16	43	2.705882	no	no	yes	no	no	no	no	no	no	yes
182195586	chr1	55420706	55420789	40	84	2.100000	no	no	yes	no	no	no	no	no	no	yes
182195588	chr1	55426300	55426398	31	99	3.193548	no	no	yes	no	no	no	no	yes	yes	yes
182195592	chr1	55442080	55442117	19	38	2.157895	no	no	yes	no	no	no	no	yes	no	yes
182195598	chr1	55492727	55492757	16	31	2.000000	no	no	yes	no	no	no	no	yes	no	yes
182195602	chr1	55500787	55500840	26	54	2.076923	no	no	yes	no	no	no	no	no	yes	yes
182195603	chr1	55509050	55509222	27	173	6.407407	no	no	yes	no	no	no	no	yes	no	yes
182195616	chr1	55606209	55606252	22	44	2.045455	no	no	yes	no	no	no	no	no	no	yes
182195618	chr1	55618556	55618582	8	27	3.375000	no	no	yes	no	no	no	no	no	no	yes
182195621	chr1	55629216	55629251	14	36	2.571429	no	no	yes	no	no	no	no	no	no	yes
182195626	chr1	55658871	55658911	22	41	1.954545	no	no	yes	no	no	no	no	no	no	yes
182195627	chr1	55660009	55660042	17	34	2.000000	no	no	yes	no	no	no	no	no	no	yes
182195636	chr1	55662530	55662718	30	189	6.300000	no	no	yes	no	no	no	no	yes	no	yes
182195642	chr1	55662988	55663107	32	120	3.676471	no	no	yes	no	no	no	no	no	no	yes
182195649	chr1	55664558	55664618	27	61	2.259259	no	no	yes	no	no	no	no	no	no	yes
182195654	chr1	55665175	55665216	21	42	2.000000	no	no	yes	no	no	no	no	no	no	yes
182195660	chr1	55666198	55666359	41	162	3.975610	no	no	yes	no	no	no	no	no	yes	yes
182195677	chr1	55702482	55702529	23	48	2.086957	no	no	yes	no	no	no	no	yes	no	yes
182195682	chr1	55708421	55708456	18	36	2.000000	no	no	yes	no	no	no	no	yes	no	yes
182195684	chr1	55709902	55709966	21	65	3.095238	no	no	yes	no	no	no	no	no	no	yes
182195687	chr1	55728199	55728271	22	73	3.391304	no	no	yes	no	no	no	no	no	no	yes
182195689	chr1	55728302	55728417	19	116	6.190476	no	no	yes	no	no	no	no	no	no	yes
182195693	chr1	55728827	55728940	48	114	2.458333	no	no	yes	no	no	no	no	no	no	yes
182195703	chr1	55771955	55771989	16	35	2.187500	no	no	yes	no	no	no	no	no	no	yes

182195715	chr1	55815136	55815197	21	62	2.952381	no	no	yes	no	no	no	no	yes	yes	yes
182195716	chr1	55824744	55824778	17	35	2.058824	no	no	yes	no	no	no	no	no	no	yes
182195719	chr1	55836031	55836065	8	35	4.375000	no	no	yes	no	no	no	no	yes	no	yes
182195722	chr1	55856895	55856964	26	70	2.703704	no	no	yes	no	no	no	no	no	no	yes
182195724	chr1	55889853	55889886	16	34	2.125000	no	no	yes	no	no	no	no	no	no	yes
182195729	chr1	55900769	55900805	18	37	2.055556	no	no	yes	no	no	no	no	no	no	yes
182195734	chr1	55908367	55908404	20	38	1.900000	no	no	yes	no	no	no	no	no	no	yes
182195737	chr1	55920162	55920213	24	52	2.166667	no	no	yes	no	no	no	no	yes	yes	yes
182195742	chr1	55941368	55941696	183	329	1.803279	no	no	yes	no	no	no	no	no	no	yes
182195747	chr1	55976911	55976951	13	41	3.142857	no	no	yes	no	no	no	no	no	no	yes
182195751	chr1	55980059	55980087	14	29	2.071429	no	no	yes	no	no	no	no	no	no	yes
182195768	chr1	56006373	56006413	20	41	2.050000	no	no	yes	no	no	no	no	no	no	yes
182195769	chr1	56007239	56007274	15	36	2.400000	no	no	yes	no	no	no	no	yes	no	yes
182195772	chr1	56007313	56007365	14	53	3.785714	no	no	yes	no	no	no	no	yes	no	yes
182195778	chr1	56043351	56043384	17	34	2.000000	no	no	yes	no	no	no	no	no	no	yes
182195786	chr1	56059704	56059790	31	87	2.764706	no	no	yes	no	no	yes	no	no	no	yes
182195789	chr1	56067039	56067086	21	48	2.285714	no	no	yes	no	no	no	no	no	no	yes
182195791	chr1	56068540	56068587	22	48	2.181818	no	no	yes	no	no	no	no	no	yes	yes
182195798	chr1	56117327	56117362	16	36	2.250000	no	no	no	no	no	no	no	no	no	no
182195800	chr1	56119951	56119986	18	36	2.000000	no	no	no	no	no	no	no	no	no	no
182195803	chr1	56132133	56132164	15	32	2.133333	no	no	no	no	no	no	no	yes	no	no
182195804	chr1	56136713	56136770	31	58	1.903226	no	no	no	no	no	no	no	no	no	no
182195817	chr1	56155435	56155475	21	41	1.952381	no	no	yes	no	no	yes	no	yes	no	yes
182195823	chr1	56162892	56162919	14	28	2.000000	no	no	yes	no	no	no	no	no	no	yes
182195827	chr1	56170277	56170315	15	39	2.666667	no	no	yes	no	no	no	no	no	yes	yes

182195828	chr1	56183756	56183819	25	64	2.560000	no	no	yes	no	no	no	no	no	no	yes
182195832	chr1	56205536	56205573	20	38	1.900000	no	no	yes	no	no	no	no	no	no	yes
182195840	chr1	56228007	56228052	20	46	2.300000	no	no	yes	no	no	no	no	no	yes	yes
182195842	chr1	56248292	56248326	18	35	2.000000	no	yes	yes	no	yes	yes	no	no	no	yes
182195878	chr1	56378842	56378945	55	104	1.890909	no	no	yes	no	no	no	no	yes	no	yes
182195882	chr1	56390673	56390732	24	60	2.480000	no	no	yes	no	no	no	no	yes	no	yes
182195886	chr1	56398211	56398303	39	93	2.384615	no	no	yes	no	no	no	no	yes	yes	yes
182195891	chr1	56410563	56410625	22	63	2.863636	no	no	yes	no	no	no	no	yes	yes	yes
182195896	chr1	56416530	56416608	14	79	5.642857	no	yes	yes	no	no	yes	no	yes	yes	yes
182195907	chr1	56433097	56433136	22	40	1.909091	no	no	yes	no	no	no	no	yes	no	yes
182195910	chr1	56445185	56445218	14	34	2.428571	no	no	yes	no	no	no	no	yes	yes	yes
182195922	chr1	56477546	56477583	19	38	2.000000	no	no	yes	no	yes	no	yes	yes	yes	yes
182195923	chr1	56478156	56478208	18	53	2.944444	yes	no	yes	no	no	no	no	yes	no	yes
182195925	chr1	56480463	56480492	14	30	2.142857	no	no	yes	no	no	no	no	no	yes	yes
182195934	chr1	56521711	56521771	24	61	2.583333	no	no	yes	no	no	no	no	no	yes	yes
182195937	chr1	56540800	56540832	17	33	1.941176	no	no	yes	no	no	no	no	no	yes	yes
182195938	chr1	56545054	56545090	18	37	2.055556	no	no	yes	no	no	no	no	no	yes	yes
182195940	chr1	56548836	56548867	16	32	2.000000	no	no	yes	no	no	no	no	yes	yes	yes
182195946	chr1	56560272	56560323	26	52	2.000000	no	no	yes	no	no	no	no	yes	no	yes
182195948	chr1	56564959	56564999	20	41	2.100000	no	no	yes	no	no	no	no	yes	no	yes
182195949	chr1	56576794	56576830	18	37	2.166667	no	no	yes	no	no	no	no	yes	yes	yes
182195953	chr1	56587548	56587582	18	35	1.944444	no	no	yes	no	no	no	no	no	no	yes
182195973	chr1	56634117	56634154	19	38	2.000000	no	no	yes	no	no	no	no	no	no	yes
182195980	chr1	56644492	56644550	29	59	2.034483	yes	no	yes	no	no	no	no	yes	yes	yes
182195988	chr1	56653254	56653291	11	38	3.545455	no	no	yes	no	no	no	no	yes	no	yes

182195989	chr1	56658468	56658550	40	83	2.075000	no	no	yes	no	no	no	no	no	no	yes
182195994	chr1	56674713	56674786	25	74	2.923077	no	no	yes	no	no	no	no	no	no	yes
182196003	chr1	56718119	56718184	32	66	2.156250	no	no	no	no	no	yes	no	yes	no	no
182196004	chr1	56723052	56723097	24	46	1.916667	no	no	yes	no	no	no	no	no	no	yes
182196011	chr1	56743852	56743898	24	47	1.958333	no	no	yes	no	no	no	no	no	no	yes
182196014	chr1	56761770	56761816	22	47	2.181818	no	no	yes	no	no	no	no	yes	no	yes
182196019	chr1	56771899	56771957	31	59	1.967742	no	no	yes	no	no	no	no	yes	no	yes
182196020	chr1	56774548	56774586	21	39	1.904762	no	no	yes	no	no	no	no	yes	no	yes
182196025	chr1	56784785	56784817	16	33	2.062500	no	no	yes	no	no	no	no	no	no	yes
182196027	chr1	56790516	56790558	16	43	2.750000	no	no	yes	no	no	no	no	no	no	yes
182196029	chr1	56792822	56792870	20	49	2.500000	no	no	yes	no	no	no	no	yes	no	yes
182196038	chr1	56823430	56823460	15	31	2.066667	no	no	no	no	no	yes	no	yes	no	no
182196044	chr1	56846438	56846469	15	32	2.133333	no	no	no	no	no	no	no	no	no	no
182196045	chr1	56850779	56850826	23	48	2.173913	no	no	no	no	no	no	no	no	no	no
182196051	chr1	56863822	56863899	21	78	3.761905	no	no	yes	no	no	no	no	yes	no	yes
182196077	chr1	56983018	56983162	30	145	4.966667	no	no	yes	no	no	no	no	no	no	yes
182196080	chr1	56986652	56986689	20	38	1.950000	no	no	yes	no	no	no	no	yes	no	yes
182196081	chr1	56996580	56996610	16	31	1.937500	no	yes	yes	no	no	no	no	yes	no	yes
182196083	chr1	57009536	57009587	24	52	2.166667	no	no	yes	no	no	no	no	no	no	yes
182196085	chr1	57010723	57010752	12	30	2.500000	no	no	no	yes	yes	no	no	no	yes	yes
182196088	chr1	57037845	57037869	12	25	2.083333	no	no	yes	no	no	no	no	no	no	yes
182196090	chr1	57042828	57042877	18	50	2.789474	no	no	yes	no	no	no	no	no	no	yes
182196100	chr1	57085387	57085430	22	44	2.000000	no	no	yes	no	no	no	no	yes	no	yes
182196105	chr1	57102189	57102238	18	50	2.777778	no	no	yes	no	no	no	no	no	yes	yes
182196119	chr1	57152716	57152759	14	44	3.062500	no	no	yes	no	no	no	no	no	no	yes

182196127	chr1	57187846	57187881	19	36	1.947368	no	no	yes	no	no	no	no	no	no	yes
182196144	chr1	57236725	57236749	13	25	1.923077	no	no	yes	no	no	no	no	yes	no	yes
182196146	chr1	57243471	57243501	16	31	1.937500	no	no	yes	no	no	no	no	no	no	yes
182196148	chr1	57275120	57275160	14	41	2.928571	no	no	yes	no	no	no	no	no	no	yes
182196153	chr1	57290616	57290931	154	316	2.051948	no	no	yes	no	no	no	no	yes	no	yes
182196156	chr1	57307469	57307556	46	88	1.913043	yes	no	yes	no	no	no	no	yes	yes	yes
182196160	chr1	57309512	57309557	24	46	1.916667	no	no	yes	no	no	no	no	no	no	yes
182196161	chr1	57311017	57311052	12	36	3.000000	no	no	yes	no	no	no	no	yes	no	yes
182196163	chr1	57324552	57324597	22	46	2.090909	no	no	yes	no	no	no	no	no	no	yes
182196164	chr1	57329667	57329703	19	37	1.947368	no	no	yes	no	no	no	no	no	no	yes
182196174	chr1	57355150	57355193	19	44	2.315789	no	no	yes	no	no	no	no	no	no	yes
182196176	chr1	57355655	57355685	10	31	3.100000	no	no	yes	no	no	no	no	no	no	yes
182196185	chr1	57365413	57365465	28	53	1.964286	no	no	yes	no	no	no	no	no	no	yes
182196194	chr1	57396368	57396404	19	37	1.947368	no	no	yes	no	no	no	no	no	no	yes
182196198	chr1	57421893	57421926	15	34	2.266667	no	no	yes	no	no	no	no	yes	yes	yes
182196199	chr1	57422364	57422388	12	25	2.083333	no	no	yes	no	no	no	yes	yes	yes	yes
182196200	chr1	57424124	57424172	25	49	1.960000	yes	no	yes	no	no	no	yes	yes	yes	yes
182196214	chr1	57440525	57440590	24	66	2.791667	no	no	yes	no	no	no	no	yes	no	yes
182196218	chr1	57441246	57441372	56	127	2.275862	no	no	yes	no	no	no	no	no	no	yes
182196219	chr1	57443306	57443350	17	45	2.647059	no	no	yes	no	no	no	no	no	no	yes
182196239	chr1	57508906	57509093	31	188	6.096774	no	no	yes	no	no	no	no	no	no	yes
182196241	chr1	57515388	57515556	86	169	2.000000	no	no	yes	no	no	no	no	no	no	yes
182196252	chr1	57550600	57550628	12	29	2.416667	no	no	yes	no	no	no	no	yes	no	yes
182196253	chr1	57551119	57551158	20	40	2.000000	no	no	yes	no	no	no	no	no	no	yes
182196276	chr1	57595217	57595290	18	74	4.052631	no	no	yes	no	no	no	no	no	no	yes

182196277	chr1	57595283	57595321	14	39	2.928571	no	no	yes	no	no	no	no	no	no	yes
182196289	chr1	57625274	57625315	21	42	2.047619	no	no	yes	no	no	no	no	yes	yes	yes
182196290	chr1	57628618	57628658	20	41	2.050000	no	no	yes	no	no	no	no	no	no	yes
182196292	chr1	57638921	57639423	111	503	4.349206	no	no	yes	no	no	no	no	no	no	yes
182196295	chr1	57644565	57644615	24	51	2.125000	no	no	yes	no	no	no	no	no	no	yes
182196298	chr1	57655177	57655237	27	61	2.407408	no	no	yes	no	no	no	no	yes	no	yes
182196307	chr1	57675665	57675708	23	44	1.956522	no	no	yes	no	no	no	no	no	no	yes
182196330	chr1	57700096	57700136	20	41	2.050000	no	no	yes	no	no	no	no	no	no	yes
182196335	chr1	57722156	57722200	21	45	2.142857	no	no	yes	no	no	no	no	no	yes	yes
182196349	chr1	57753614	57753661	25	48	1.920000	no	no	yes	no	no	no	no	no	no	yes
182196373	chr1	57782969	57783061	46	93	2.021739	no	no	yes	no	no	no	no	no	no	yes
182196386	chr1	57813031	57813062	16	32	2.000000	no	no	yes	no	no	no	no	no	no	yes
182196392	chr1	57834572	57834627	26	56	2.153846	no	no	yes	no	no	no	no	no	no	yes
182196405	chr1	57871252	57871287	17	36	2.117647	no	no	yes	no	no	no	no	no	no	yes
182196416	chr1	57900128	57900160	17	33	1.941176	no	no	yes	no	no	no	no	no	no	yes
182196418	chr1	57911490	57911529	19	40	2.105263	no	no	yes	no	no	no	no	no	no	yes
182196425	chr1	57922326	57922369	20	44	2.200000	no	no	yes	no	no	no	no	yes	no	yes
182196427	chr1	57922366	57922416	16	51	3.187500	no	no	yes	no	no	no	no	yes	no	yes
182196428	chr1	57922942	57922977	17	36	2.176471	no	no	yes	no	no	no	no	no	no	yes
182196432	chr1	57944803	57944882	40	80	2.000000	no	no	yes	no	no	no	no	no	yes	yes
182196437	chr1	57955637	57955690	25	54	2.160000	no	no	yes	no	no	no	no	no	no	yes
182196438	chr1	57955780	57955821	22	42	2.000000	no	no	yes	no	no	no	no	no	no	yes
182196444	chr1	57972872	57972949	41	78	1.902439	no	no	yes	no	no	no	no	no	no	yes
182196445	chr1	57974489	57974524	8	36	4.500000	no	no	yes	no	no	no	no	no	no	yes
182196447	chr1	57974838	57974876	16	39	2.500000	no	no	yes	no	no	no	no	no	no	yes

182196448	chr1	57980137	57980174	19	38	2.000000	no	no	yes	no	no	no	no	no	no	yes
182196449	chr1	57981314	57981360	18	47	2.611111	no	no	yes	no	no	no	no	no	no	yes
182196458	chr1	57999999	58000069	24	71	2.958333	no	no	yes	no	no	no	no	yes	no	yes
182196461	chr1	58007220	58007255	16	36	2.250000	no	no	yes	no	no	no	no	yes	no	yes
182196462	chr1	58009736	58009836	52	101	1.942308	no	no	yes	no	no	no	no	no	no	yes
182196466	chr1	58018077	58018126	21	50	2.380952	no	no	yes	no	no	no	no	no	no	yes
182196467	chr1	58025486	58025646	87	161	1.850575	no	no	yes	no	no	no	no	no	no	yes
182196469	chr1	58030988	58031026	19	39	2.052632	no	no	yes	no	no	no	no	yes	no	yes
182196472	chr1	58045767	58045799	17	33	2.000000	no	no	yes	no	no	no	no	no	no	yes
182196473	chr1	58046444	58046475	16	32	2.000000	no	no	yes	no	no	no	no	no	no	yes
182196501	chr1	58117845	58117876	12	32	2.666667	no	no	yes	no	no	no	no	no	no	yes
182196511	chr1	58160730	58160754	12	25	2.083333	no	no	yes	no	no	no	no	no	no	yes
182196512	chr1	58161431	58161466	17	36	2.176471	no	no	yes	no	no	no	no	no	no	yes
182196543	chr1	58229694	58229725	11	32	2.909091	yes	no	yes	no	no	no	no	no	no	yes
182196548	chr1	58246874	58246925	24	52	2.250000	no	no	yes	no	no	no	no	yes	no	yes
182196564	chr1	58298019	58298047	13	29	2.230769	no	no	yes	no	no	no	no	no	no	yes
182196568	chr1	58310237	58310286	17	50	2.941176	no	no	yes	no	no	no	no	no	no	yes
182196571	chr1	58327308	58327336	12	29	2.416667	no	no	yes	no	no	no	no	no	no	yes
182196573	chr1	58333776	58333800	12	25	2.083333	no	no	yes	no	no	no	no	no	yes	yes
182196591	chr1	58392979	58393013	16	35	2.187500	no	no	yes	no	no	no	no	no	yes	yes
182196594	chr1	58420184	58420213	15	30	2.000000	no	no	yes	no	no	no	no	no	no	yes
182196601	chr1	58442118	58442167	24	50	2.083333	no	no	yes	no	no	no	no	yes	yes	yes
182196602	chr1	58448352	58448379	14	28	2.000000	no	no	yes	no	no	no	no	yes	no	yes
182196613	chr1	58487687	58487725	15	39	2.625000	no	no	yes	no	no	no	no	no	no	yes
182196617	chr1	58506874	58506898	13	25	1.923077	yes	no	yes	no	no	no	no	no	no	yes

182196621	chr1	58531598	58531633	17	36	2.117647	yes	no	yes	no	no	no	no	no	no	yes
182196625	chr1	58547496	58547537	21	42	2.000000	yes	no	yes	no	no	no	no	yes	yes	yes
182196637	chr1	58593269	58593330	24	62	2.625000	no	no	no	no	no	no	no	yes	no	no
182196639	chr1	58596803	58596860	25	58	2.320000	no	no	no	no	no	no	no	yes	yes	no
182196647	chr1	58608526	58608569	20	44	2.200000	no	no	no	no	no	no	no	no	yes	no
182196648	chr1	58613200	58613242	18	43	2.388889	no	no	no	no	no	no	no	no	no	no
182196666	chr1	58664213	58664247	11	35	3.181818	no	no	yes	no	no	no	no	no	no	yes
182196672	chr1	58688284	58688319	17	36	2.117647	no	no	yes	no	no	no	no	no	no	yes
182196692	chr1	58729238	58729417	30	180	6.133333	no	no	yes	no	no	no	no	no	no	yes
182196696	chr1	58729440	58729493	22	54	2.434783	no	no	yes	no	no	no	no	no	no	yes
182196699	chr1	58729584	58730405	34	822	25.371429	no	no	yes	no	no	no	no	yes	no	yes
182196731	chr1	58773810	58773834	12	25	2.083333	no	no	no	no	no	no	no	yes	no	no
182196740	chr1	58816560	58816596	18	37	2.055556	no	no	yes	no	no	yes	yes	yes	no	yes
182196753	chr1	58861148	58861416	98	269	2.762376	no	no	yes	no	no	no	no	yes	no	yes
182196756	chr1	58866800	58866877	34	78	2.294118	no	no	yes	no	no	no	no	yes	no	yes
182196759	chr1	58877447	58877496	26	50	1.923077	no	no	yes	no	no	no	no	no	no	yes
182196761	chr1	58891788	58891827	18	40	2.222222	no	no	yes	no	no	no	no	yes	no	yes
182196765	chr1	58896740	58896773	17	34	2.000000	yes	no	yes	no	yes	no	no	yes	yes	yes
182196769	chr1	58908418	58908465	25	48	1.920000	no	no	yes	no	no	no	no	no	no	yes
182196773	chr1	58917819	58917875	28	57	2.035714	no	no	yes	no	no	no	no	yes	no	yes
182196775	chr1	58917914	58917956	21	43	2.095238	no	no	yes	no	no	no	no	no	no	yes
182196776	chr1	58918744	58918816	27	73	2.703704	no	no	yes	no	no	no	no	no	no	yes
182196782	chr1	58968958	58968994	17	37	2.352941	no	no	yes	no	no	no	no	yes	no	yes
182196792	chr1	58997019	58997075	28	57	2.035714	no	no	yes	no	no	no	no	no	no	yes
182196793	chr1	58997066	58997111	24	46	1.916667	no	no	yes	no	no	no	no	no	yes	yes

182196799 chr1 59023254 59023279 13 26 2.000000 no no yes no no
yes no no no yes
182196804 chr1 59051138 59051175 15 38 2.562500 no no yes no no
no no yes no yes
182196811 chr1 59058969 59059012 18 44 2.444444 no no yes no no
no no yes no yes
182196821 chr1 59073205 59073242 15 38 2.533333 no no yes no no
no no no no yes
182196822 chr1 59076785 59076822 16 38 2.375000 no no yes no no
no no yes yes yes
182196823 chr1 59076864 59076965 35 102 2.914286 no no yes no
no no no yes yes yes
182196828 chr1 59104659 59104684 12 26 2.166667 no no yes no no
yes no yes no yes
182196830 chr1 59109733 59109771 18 39 2.222222 no no yes no no
no no yes no yes
182196833 chr1 59118511 59118551 20 41 2.050000 no no yes no no
no no no no yes
182196838 chr1 59133346 59133382 17 37 2.294118 no no yes no no
yes no no no yes
182196842 chr1 59138030 59138082 21 53 2.523809 no no yes no no
no no no yes yes
182196844 chr1 59146912 59146942 15 31 2.066667 yes no yes no no
no yes yes yes yes
182196849 chr1 59154659 59154744 42 86 2.047619 no no yes no no
no no no yes yes
182196875 chr1 59241485 59241522 9 38 4.100000 no no yes no no
yes no yes no yes
182196880 chr1 59250694 59250748 29 55 1.931034 no no yes no no
no no no no yes
182196894 chr1 59289420 59289462 22 43 1.954545 no no yes no
yes no no no no yes
182196896 chr1 59289714 59289753 21 40 1.952381 yes no yes no no
no no yes yes yes
182196897 chr1 59296638 59296773 16 136 8.500000 yes no no no
yes no yes yes yes yes
182196898 chr1 59297039 59297079 15 41 2.733333 yes yes yes no
yes no yes yes yes yes
182196899 chr1 59298568 59298646 38 79 2.078947 no no yes no no
no no yes yes yes
182196910 chr1 59337002 59337066 28 65 2.321429 no no yes no no
no no no no yes
182196916 chr1 59366423 59366452 12 30 2.500000 no no yes no no
no no yes yes yes
182196923 chr1 59401427 59401451 13 25 1.923077 no no yes no no
no no no no yes
182196924 chr1 59403986 59404015 16 30 1.937500 no no yes no no
no no no no yes
182196925 chr1 59406818 59406874 26 57 2.307692 no no yes no no
no no yes no yes

182196928	chr1	59421508	59421549	21	42	2.047619	no	no	yes	no	no	no	no	yes	no	yes
182196931	chr1	59426662	59426703	16	42	2.687500	no	no	yes	no	no	no	no	yes	yes	yes
182196936	chr1	59447160	59447192	15	33	2.200000	no	no	yes	no	no	no	no	no	yes	yes
182196937	chr1	59447193	59447229	15	37	2.466667	no	no	yes	no	no	no	no	no	yes	yes
182196938	chr1	59451307	59451335	15	29	1.933333	no	no	yes	no	no	no	no	no	no	yes
182196939	chr1	59451474	59451512	14	39	2.785714	no	no	yes	no	no	no	no	no	no	yes
182196942	chr1	59459437	59459480	22	44	2.000000	no	no	yes	no	no	no	no	no	no	yes
182196956	chr1	59482549	59482649	36	101	2.805556	no	no	yes	no	no	no	no	no	no	yes
182196959	chr1	59488498	59488567	28	70	2.482759	no	no	yes	no	no	no	no	yes	no	yes
182196967	chr1	59505179	59505210	16	32	2.000000	no	no	yes	no	no	no	no	no	no	yes
182196972	chr1	59516074	59516110	10	37	3.800000	no	no	yes	no	no	no	no	no	no	yes
182196974	chr1	59525786	59525826	16	41	2.562500	no	no	yes	no	no	no	no	no	no	yes
182196979	chr1	59542952	59542994	21	43	2.047619	no	no	yes	no	no	no	no	yes	no	yes
182197004	chr1	59667526	59667600	38	75	1.973684	no	no	yes	no	no	no	no	no	no	yes
182197007	chr1	59680245	59680288	22	44	2.000000	no	no	yes	no	no	no	no	yes	yes	yes
182197008	chr1	59680490	59680522	16	33	2.062500	no	no	yes	no	no	no	no	no	no	yes
182197009	chr1	59681131	59681175	22	45	2.045455	no	no	yes	no	no	yes	no	no	no	yes
182197013	chr1	59691608	59691657	26	50	2.000000	no	no	yes	no	no	no	no	yes	yes	yes
182197016	chr1	59693539	59693571	12	33	2.750000	no	no	yes	no	no	no	no	yes	yes	yes
182197021	chr1	59705358	59705423	25	66	2.760000	no	no	yes	no	no	no	no	no	yes	yes
182197023	chr1	59714709	59714753	21	45	2.190476	no	no	yes	no	no	no	no	yes	no	yes
182197024	chr1	59721207	59721261	27	55	2.037037	no	no	yes	no	no	no	no	no	no	yes
182197042	chr1	59771528	59771567	18	40	2.222222	no	no	yes	no	no	no	no	yes	no	yes
182197053	chr1	59814828	59814861	17	34	2.000000	yes	no	yes	no	no	no	yes	yes	no	yes
182197056	chr1	59825892	59825946	28	55	1.964286	no	no	yes	no	no	no	no	no	no	yes

182197057	chr1	59836006	59836038	16	33	2.062500	no	no	yes	no	no	yes	no	no	no	yes
182197061	chr1	59847503	59847539	16	37	2.312500	no	no	yes	no	no	no	no	no	no	yes
182197067	chr1	59858718	59858756	17	39	2.352941	no	no	yes	no	no	no	no	yes	no	yes
182197072	chr1	59861907	59862087	60	181	3.016667	no	no	yes	no	no	no	no	no	no	yes
182197073	chr1	59868040	59868064	13	25	1.923077	no	no	yes	no	no	no	no	no	no	yes
182197085	chr1	59915457	59915489	14	33	2.357143	no	no	yes	no	no	no	no	no	no	yes
182197090	chr1	59926773	59926811	16	39	2.437500	yes	no	no	no	no	no	yes	yes	yes	no
182197098	chr1	59949568	59949615	22	48	2.272727	no	no	no	no	no	no	no	no	yes	no
182197120	chr1	60011422	60011465	16	44	2.750000	yes	no	yes	no	no	no	no	no	no	yes
182197122	chr1	60025530	60025568	20	39	1.950000	no	no	yes	no	no	no	no	no	no	yes
182197123	chr1	60026459	60026498	16	40	2.529412	no	no	yes	no	no	no	no	no	no	yes
182197126	chr1	60033272	60033316	20	45	2.350000	no	no	yes	no	no	no	no	yes	yes	yes
182197127	chr1	60033617	60033645	13	29	2.230769	no	no	yes	yes	no	no	no	no	no	yes
182197129	chr1	60038692	60038736	23	45	1.956522	no	no	yes	no	no	no	no	no	no	yes
182197135	chr1	60054950	60055015	22	66	3.000000	no	no	yes	no	no	no	no	no	no	yes
182197136	chr1	60055246	60055280	18	35	1.944444	no	no	no	yes	no	no	no	no	no	yes
182197140	chr1	60064242	60064371	38	130	3.473684	no	no	yes	no	no	no	no	no	no	yes
182197146	chr1	60083869	60083949	40	81	2.025000	no	no	no	no	no	no	no	yes	no	no
182197150	chr1	60085661	60085963	71	303	4.220779	no	no	no	no	no	no	no	no	no	no
182197156	chr1	60092902	60092934	12	33	2.750000	no	no	no	no	no	no	no	no	no	no
182197159	chr1	60116168	60116194	13	27	2.076923	no	no	yes	no	no	no	no	no	no	yes
182197168	chr1	60159267	60159314	22	48	2.181818	no	no	no	no	no	no	no	no	no	no
182197169	chr1	60159700	60159748	24	49	2.041667	no	no	no	no	no	no	no	no	no	no
182197179	chr1	60195209	60195292	43	84	1.953488	no	no	no	no	no	no	no	no	no	no
182197196	chr1	60252328	60252370	17	43	2.588235	no	no	no	no	no	no	no	no	no	no

182197200	chr1	60270655	60271019	47	365	7.814815	no	no	no	no	no	no	no	no	no	no
182197211	chr1	60290615	60290659	23	45	1.956522	no	no	no	no	no	no	no	no	no	no
182197229	chr1	60345658	60345696	20	39	1.950000	no	no	no	no	no	no	no	yes	no	no
182197230	chr1	60350201	60350298	48	98	2.041667	no	no	no	no	no	no	no	no	no	no
182197231	chr1	60352114	60352145	16	32	2.000000	no	no	no	no	no	no	no	no	no	no
182197232	chr1	60357342	60357373	17	32	1.941176	no	no	no	no	no	no	no	no	no	no
182197234	chr1	60375488	60375618	63	131	2.079365	no	no	no	no	no	no	no	no	yes	no
182197239	chr1	60383722	60383747	13	26	2.000000	no	no	no	no	no	no	no	no	no	no
182197241	chr1	60401628	60401681	28	54	1.928571	no	no	no	no	no	no	no	no	yes	no
182197242	chr1	60407211	60407287	32	77	2.424242	no	no	no	no	no	no	no	no	no	no
182197244	chr1	60416274	60416307	16	34	2.125000	no	no	no	no	no	no	no	no	no	no
182197249	chr1	60443616	60443644	13	29	2.230769	no	no	no	no	no	no	no	no	no	no
182197257	chr1	60477747	60477955	105	209	1.990476	no	no	no	no	no	no	no	no	no	no
182197269	chr1	60514754	60514795	21	42	2.047619	no	no	no	no	no	yes	no	yes	no	no
182197277	chr1	60551789	60551823	18	35	1.944444	no	no	yes	no	no	no	no	no	no	yes
182197282	chr1	60553852	60553992	67	141	2.179105	no	no	yes	no	no	no	no	no	no	yes
182197289	chr1	60580550	60580600	9	51	5.400000	no	no	yes	no	no	no	no	yes	yes	yes
182197291	chr1	60582535	60582571	18	37	2.055556	no	no	yes	no	no	no	no	no	no	yes
182197292	chr1	60590756	60590786	14	31	2.214286	no	no	yes	no	no	no	no	no	no	yes
182197306	chr1	60619284	60619328	21	45	2.142857	no	no	yes	no	no	no	no	no	no	yes
182197311	chr1	60636701	60636774	32	74	2.312500	no	no	yes	no	no	no	no	no	no	yes
182197319	chr1	60666638	60666680	14	43	2.937500	no	no	yes	no	no	yes	no	no	no	yes
182197322	chr1	60668462	60668502	22	41	1.909091	no	no	yes	no	no	no	no	no	no	yes
182197329	chr1	60696998	60697035	20	38	1.900000	no	no	yes	no	no	no	no	no	no	yes
182197332	chr1	60716455	60716493	19	39	2.105263	no	no	yes	no	no	no	no	no	no	yes

182197337	chr1	60732665	60732750	42	86	2.047619	no	no	yes	no	no	no	no	yes	no	yes
182197352	chr1	60763302	60763369	35	68	1.942857	no	no	yes	no	no	no	no	yes	no	yes
182197362	chr1	60788212	60788248	18	37	2.111111	no	no	yes	no	no	no	no	no	no	yes
182197382	chr1	60891143	60891184	16	42	2.687500	no	no	yes	no	no	no	no	no	no	yes
182197384	chr1	60896040	60896084	19	45	2.368421	no	no	yes	no	no	no	no	no	yes	yes
182197388	chr1	60898745	60898825	40	81	2.050000	no	no	yes	no	no	no	no	yes	no	yes
182197389	chr1	60899187	60899227	20	41	2.050000	no	no	yes	no	no	no	no	yes	yes	yes
182197397	chr1	60916072	60916130	23	59	2.583333	no	no	yes	no	no	no	no	no	no	yes
182197403	chr1	60921520	60921568	20	49	2.550000	no	no	yes	no	no	no	no	yes	no	yes
182197404	chr1	60921564	60921605	18	42	2.333333	no	no	yes	no	no	no	no	yes	no	yes
182197414	chr1	60936465	60936518	20	54	2.700000	no	no	yes	no	no	no	no	yes	yes	yes
182197424	chr1	60977794	60977931	25	138	5.290323	no	no	yes	no	no	no	no	no	no	yes
182197430	chr1	60995528	60995586	30	59	1.966667	no	no	yes	no	no	no	no	yes	no	yes
182197453	chr1	61053481	61053517	17	37	2.176471	yes	no	yes	no	no	no	no	yes	yes	yes
182197457	chr1	61057022	61057070	18	49	2.722222	yes	no	yes	no	no	no	no	yes	yes	yes
182197458	chr1	61057291	61057315	9	25	2.777778	yes	no	yes	no	no	no	no	yes	yes	yes
182197463	chr1	61067812	61067854	14	43	3.142857	no	no	yes	no	no	no	no	yes	yes	yes
182197471	chr1	61071690	61071732	18	43	2.444444	no	no	yes	no	no	no	no	yes	no	yes
182197472	chr1	61072160	61072193	16	34	2.125000	no	no	yes	no	no	no	no	yes	no	yes
182197475	chr1	61077123	61077147	11	25	2.272727	yes	no	yes	no	no	no	yes	yes	yes	yes
182197478	chr1	61080953	61081001	25	49	1.960000	yes	no	yes	no	no	no	no	yes	yes	yes
182197483	chr1	61093321	61093387	35	67	1.971429	no	yes	yes	no	no	no	no	no	no	yes
182197484	chr1	61093613	61093670	27	58	2.148148	no	yes	yes	no	no	no	no	yes	no	yes
182197486	chr1	61106144	61106175	16	32	2.000000	no	yes	yes	no	no	no	no	no	no	yes
182197487	chr1	61106190	61106231	15	42	2.866667	no	yes	yes	no	no	no	no	no	no	yes

182197492	chr1	61117532	61117594	31	63	2.032258	no	yes	yes	no	no	no	no	yes	no	yes
182197497	chr1	61128710	61128749	21	40	1.904762	no	yes	yes	no	no	no	no	no	no	yes
182197500	chr1	61138419	61138490	34	72	2.117647	no	no	yes	no	no	no	no	yes	no	yes
182197504	chr1	61153939	61154015	38	77	2.026316	no	no	yes	no	no	no	no	yes	no	yes
182197509	chr1	61167149	61167193	23	45	2.043478	no	no	yes	no	no	no	no	no	yes	yes
182197515	chr1	61184116	61184176	19	61	3.210526	no	no	yes	no	no	no	no	yes	yes	yes
182197518	chr1	61192877	61192914	20	38	1.900000	no	no	yes	no	no	no	no	yes	yes	yes
182197527	chr1	61228943	61228987	23	45	1.956522	no	no	yes	no	no	no	no	no	no	yes
182197532	chr1	61239314	61239351	17	38	2.235294	no	no	yes	no	no	no	no	no	no	yes
182197533	chr1	61243908	61243940	17	33	2.000000	no	no	yes	no	no	no	no	no	no	yes
182197542	chr1	61283783	61283814	14	32	2.357143	no	no	yes	no	no	no	no	no	no	yes
182197544	chr1	61294893	61294922	14	30	2.142857	no	no	yes	no	no	no	no	yes	no	yes
182197547	chr1	61318886	61318935	25	50	2.040000	no	no	yes	no	no	no	no	yes	yes	yes
182197552	chr1	61327046	61327089	23	44	1.913043	no	no	yes	no	no	no	no	no	no	yes
182197555	chr1	61332556	61332593	20	38	1.900000	no	yes	yes	yes	yes	yes	no	yes	no	yes
182197567	chr1	61359893	61359943	25	51	2.080000	no	no	yes	no	no	no	no	no	no	yes
182197571	chr1	61380990	61381027	20	38	1.900000	no	no	yes	no	no	no	no	no	no	yes
182197578	chr1	61393228	61393311	12	84	7.000000	no	no	yes	no	no	no	no	yes	no	yes
182197580	chr1	61405420	61405451	15	32	2.133333	no	no	yes	no	no	no	no	no	no	yes
182197584	chr1	61423978	61424015	10	38	3.800000	no	no	yes	no	no	no	no	yes	no	yes
182197585	chr1	61425397	61425487	46	91	1.978261	no	no	yes	no	no	no	no	yes	no	yes
182197593	chr1	61456796	61456862	23	67	2.840000	no	yes	no	no	no	yes	no	no	no	yes
182197600	chr1	61462017	61462065	23	49	2.217391	no	yes	no	no	no	no	no	yes	no	yes
182197616	chr1	61485806	61485842	19	37	1.947368	no	no	yes	no	no	no	no	no	no	yes
182197617	chr1	61486562	61486591	14	30	2.142857	no	no	yes	no	no	no	no	no	yes	yes

182197618	chr1	61487115	61487139	13	25	1.923077	no	no	yes	no	no	no	no	yes	no	yes
182197638	chr1	61557700	61557785	42	86	2.047619	no	no	no	no	no	no	no	yes	no	no
182197640	chr1	61581080	61581136	25	57	2.320000	no	no	no	no	no	no	no	no	no	no
182197643	chr1	61586185	61586243	22	59	2.708333	no	no	no	no	no	no	no	no	no	no
182197646	chr1	61589557	61589606	22	50	2.272727	no	no	yes	no	no	no	no	yes	yes	yes
182197655	chr1	61625555	61625600	23	46	2.000000	no	no	no	no	no	no	no	no	no	no
182197659	chr1	61635147	61635176	14	30	2.142857	no	no	no	no	no	no	no	yes	no	no
182197665	chr1	61656925	61656955	15	31	2.066667	no	no	no	no	no	no	no	no	no	no
182197680	chr1	61692030	61692061	15	32	2.133333	no	no	yes	no	no	no	no	no	no	yes
182197684	chr1	61692432	61692558	58	127	2.278688	no	no	yes	no	no	no	no	yes	yes	yes
182197698	chr1	61704727	61704772	22	46	2.090909	no	no	yes	no	no	no	no	no	no	yes
182197711	chr1	61723191	61723227	15	37	2.533333	no	no	yes	no	no	no	no	yes	no	yes
182197725	chr1	61753164	61753200	19	37	1.947368	no	no	yes	no	no	no	no	yes	no	yes
182197733	chr1	61763974	61764019	23	46	2.000000	no	no	yes	no	no	no	no	yes	no	yes
182197753	chr1	61795148	61795179	17	32	1.941176	no	no	yes	no	no	no	no	no	no	yes
182197770	chr1	61835680	61835831	76	152	2.000000	no	no	yes	no	no	no	no	yes	no	yes
182197771	chr1	61839668	61839701	18	34	1.944444	no	no	yes	no	no	no	no	yes	no	yes
182197772	chr1	61841140	61841195	30	56	1.933333	no	no	yes	no	no	no	no	no	no	yes
182197773	chr1	61846107	61846142	17	36	2.117647	no	no	yes	no	no	no	no	no	no	yes
182197774	chr1	61848783	61848821	16	39	2.411765	no	no	yes	no	no	no	no	no	no	yes
182197776	chr1	61860067	61860100	17	34	2.000000	no	no	yes	no	no	no	no	yes	no	yes
182197781	chr1	61867833	61867864	13	32	2.538461	no	no	yes	no	no	no	no	no	no	yes
182197794	chr1	61887080	61887120	22	41	1.909091	no	no	yes	no	no	no	no	yes	no	yes
182197808	chr1	61935367	61935417	19	51	2.684211	no	no	yes	no	no	no	no	no	no	yes
182197818	chr1	61965345	61965418	39	74	1.923077	no	no	yes	no	no	no	no	no	no	yes

182197821	chr1	61974301	61974344	21	44	2.190476	no	no	yes	no	no	no	no	yes	no	yes
182197826	chr1	61980891	61980925	18	35	1.944444	no	no	yes	no	no	no	no	yes	no	yes
182197847	chr1	62037817	62037845	15	29	1.933333	yes	no	yes	no	no	no	no	no	no	yes
182197849	chr1	62041691	62041749	24	59	2.500000	no	no	yes	no	no	no	no	no	no	yes
182197862	chr1	62066327	62066364	21	38	1.904762	no	no	yes	no	no	no	no	yes	no	yes
182197874	chr1	62083038	62083106	33	69	2.090909	no	no	yes	no	no	no	no	yes	no	yes
182197878	chr1	62086245	62086367	62	123	1.983871	no	no	yes	no	no	no	no	no	no	yes
182197881	chr1	62088794	62088887	24	94	4.041667	no	no	yes	no	no	no	no	yes	no	yes
182197896	chr1	62108848	62108885	20	38	1.900000	no	no	yes	no	no	no	no	no	no	yes
182197904	chr1	62137384	62137687	54	304	5.629630	no	no	yes	no	no	no	no	no	yes	yes
182197908	chr1	62141025	62141066	17	42	2.470588	no	no	yes	no	no	no	no	no	no	yes
182197910	chr1	62144162	62144348	93	187	2.010753	no	no	yes	no	no	no	no	yes	yes	yes
182197917	chr1	62150497	62150536	21	40	1.904762	no	no	yes	no	no	no	no	no	no	yes
182197918	chr1	62153264	62153336	37	73	2.027027	no	no	yes	no	no	no	no	no	no	yes
182197919	chr1	62155356	62155438	40	83	2.075000	no	no	yes	no	no	no	no	no	no	yes
182197922	chr1	62157351	62157398	25	48	1.920000	no	no	yes	no	no	no	no	no	no	yes
182197950	chr1	62209935	62209987	27	53	1.962963	no	no	no	yes	no	no	no	yes	yes	yes
182197952	chr1	62210022	62210083	24	62	2.583333	no	no	no	yes	no	no	no	yes	yes	yes
182197977	chr1	62266493	62266636	51	144	2.862745	no	no	yes	no	no	no	no	yes	yes	yes
182197979	chr1	62272314	62272350	18	37	2.055556	no	no	yes	no	yes	no	no	yes	yes	yes
182197988	chr1	62283916	62283955	17	40	2.352941	no	no	yes	no	no	no	no	no	no	yes
182197989	chr1	62290112	62290145	12	34	2.833333	no	no	yes	no	no	no	no	no	no	yes
182197992	chr1	62321779	62321815	13	37	2.923077	no	no	no	no	no	no	no	yes	no	no
182198004	chr1	62349429	62349475	16	47	2.941176	no	no	no	no	no	no	no	yes	yes	no
182198022	chr1	62373812	62373884	37	73	1.972973	no	no	no	no	no	no	no	no	no	no

182198046	chr1	62435898	62435936	20	39	1.950000	yes	no	no	no	no	no	no	yes	yes	no
182198047	chr1	62441917	62441961	22	45	2.045455	no	no	yes	no	no	yes	no	yes	yes	yes
182198067	chr1	62505879	62505926	24	48	2.083333	no	no	yes	no	no	no	no	no	no	yes
182198069	chr1	62510407	62510445	16	39	2.562500	no	no	yes	no	no	no	no	no	no	yes
182198070	chr1	62511958	62512001	18	44	2.444444	yes	no	yes	no	no	no	no	no	no	yes
182198072	chr1	62513179	62513251	28	73	2.607143	no	no	yes	no	no	no	no	yes	no	yes
182198081	chr1	62531455	62531492	20	38	1.900000	no	no	yes	no	no	yes	no	no	no	yes
182198082	chr1	62551783	62551818	12	36	3.000000	no	no	yes	no	no	no	no	no	no	yes
182198083	chr1	62552085	62552116	15	32	2.133333	no	no	yes	no	no	no	no	no	no	yes
182198086	chr1	62553995	62554095	50	101	2.020000	no	no	yes	no	no	no	no	no	no	yes
182198093	chr1	62577029	62577073	21	45	2.285714	no	no	yes	no	no	no	no	no	no	yes
182198100	chr1	62604463	62604513	26	51	1.961538	no	yes	yes	no	no	no	no	no	no	yes
182198101	chr1	62605833	62605857	8	25	3.125000	no	yes	yes	no	no	no	no	no	no	yes
182198102	chr1	62612575	62612604	15	30	2.000000	no	no	yes	no	no	no	no	no	no	yes
182198105	chr1	62621105	62621157	25	53	2.280000	no	no	yes	no	no	no	no	yes	no	yes
182198111	chr1	62638527	62638676	16	150	8.800000	no	no	yes	no	no	no	no	no	no	yes
182198119	chr1	62663464	62663509	15	46	3.200000	yes	no	yes	no	no	no	no	no	no	yes
182198128	chr1	62677068	62677105	19	38	2.052632	no	no	yes	no	no	no	no	yes	yes	yes
182198132	chr1	62687374	62687426	26	53	2.038461	no	no	yes	no	no	yes	no	yes	yes	yes
182198139	chr1	62709785	62709829	22	45	2.045455	no	yes	no	no	no	no	no	no	no	yes
182198140	chr1	62711171	62711210	18	40	2.222222	no	no	no	no	no	yes	no	no	no	no
182198150	chr1	62721958	62722032	15	75	5.312500	no	no	no	no	no	no	no	no	no	no
182198161	chr1	62735995	62736029	16	35	2.187500	no	no	no	no	no	no	no	yes	yes	no
182198164	chr1	62743581	62743621	17	41	2.411765	no	no	no	no	no	no	no	no	no	no
182198166	chr1	62743606	62743686	33	81	2.484848	no	no	no	no	no	no	no	no	no	no

182198202	chr1	62792598	62792641	20	44	2.200000	no	no	yes	no	no	no	no	no	no	yes
182198206	chr1	62793809	62793849	12	41	3.583333	no	no	yes	no	no	no	no	no	no	yes
182198208	chr1	62797389	62797461	38	73	1.921053	no	no	yes	no	no	no	no	no	no	yes
182198214	chr1	62820893	62820938	24	46	2.000000	yes	no	yes	no	no	no	no	no	no	yes
182198216	chr1	62835485	62835520	18	36	2.000000	no	yes	yes	no	no	no	no	no	no	yes
182198227	chr1	62868080	62868125	21	46	2.238095	no	no	no	no	no	no	no	no	no	no
182198229	chr1	62873599	62873642	21	44	2.095238	no	no	no	no	no	no	no	no	no	no
182198237	chr1	62904709	62904748	20	40	2.000000	yes	no	no	no	no	no	no	no	no	no
182198242	chr1	62912114	62912146	16	33	2.062500	no	no	no	no	no	no	no	yes	yes	no
182198260	chr1	62975755	62975789	12	35	2.916667	no	no	no	no	yes	no	no	no	no	yes
182198262	chr1	62977620	62977669	23	50	2.217391	no	no	yes	no	no	no	no	no	yes	yes
182198290	chr1	63047184	63047227	23	44	1.913043	no	no	no	no	no	no	no	no	no	no
182198292	chr1	63053000	63053033	16	34	2.125000	no	no	no	no	no	no	no	no	no	no
182198298	chr1	63066173	63066213	20	41	2.050000	no	no	no	no	no	no	no	no	no	no
182198299	chr1	63068207	63068253	19	47	2.526316	no	no	no	no	no	no	no	yes	no	no
182198300	chr1	63076217	63076259	22	43	2.000000	no	no	no	no	no	no	no	no	no	no
182198305	chr1	63098108	63098187	17	80	4.764706	no	no	no	no	no	no	no	no	no	no
182198311	chr1	63129355	63129389	17	35	2.058824	no	no	no	no	no	no	no	yes	yes	no
182198312	chr1	63129378	63129431	24	54	2.250000	no	no	no	no	no	no	no	yes	yes	no
182198315	chr1	63139937	63139967	15	31	2.066667	no	no	yes	no	no	no	no	yes	yes	yes
182198317	chr1	63156056	63156086	16	31	1.937500	no	no	yes	no	no	no	no	no	no	yes
182198318	chr1	63157540	63157572	10	33	3.181818	no	no	yes	no	no	no	no	no	no	yes
182198322	chr1	63172681	63172750	34	70	2.147059	no	no	yes	no	no	no	no	yes	no	yes
182198327	chr1	63182324	63182431	54	108	2.037037	no	no	yes	no	no	no	no	no	no	yes
182198333	chr1	63182467	63182515	19	49	2.700000	no	no	yes	no	no	no	no	no	no	yes

182198341	chr1	63192008	63192045	20	38	1.950000	no	no	yes	no	no	no	no	no	no	yes
182198348	chr1	63221972	63222008	16	37	2.312500	no	no	yes	no	no	yes	no	no	no	yes
182198371	chr1	63281952	63281995	15	44	2.937500	no	no	yes	no	no	no	no	no	no	yes
182198386	chr1	63296436	63296475	14	40	2.928571	no	no	yes	no	no	no	no	no	no	yes
182198395	chr1	63323865	63323900	14	36	2.714286	yes	no	yes	yes	no	no	yes	yes	yes	yes
182198396	chr1	63324112	63324146	18	35	1.944444	yes	no	yes	yes	no	no	yes	yes	yes	yes
182198407	chr1	63352879	63352914	19	36	1.947368	no	no	no	no	no	no	no	yes	no	no
182198412	chr1	63372548	63372589	22	42	1.909091	no	no	yes	no	no	no	no	no	no	yes
182198432	chr1	63418273	63418305	15	33	2.266667	no	no	yes	no	no	no	no	no	no	yes
182198439	chr1	63438566	63438613	25	48	1.920000	no	no	no	no	no	yes	no	yes	no	no
182198440	chr1	63438761	63438789	14	29	2.142857	no	no	no	no	no	yes	no	no	no	no
182198447	chr1	63444564	63444685	54	122	2.290909	no	no	yes	no	no	no	no	no	no	yes
182198448	chr1	63445335	63445397	31	63	2.064516	no	no	yes	no	no	no	no	no	no	yes
182198449	chr1	63446475	63446499	13	25	1.923077	no	no	yes	no	no	yes	no	no	no	yes
182198468	chr1	63474907	63474941	16	35	2.187500	no	no	yes	no	no	no	no	no	no	yes
182198469	chr1	63485430	63485508	26	79	3.153846	no	no	yes	no	no	no	no	no	no	yes
182198475	chr1	63516348	63516380	11	33	2.916667	no	no	yes	no	no	no	no	no	no	yes
182198481	chr1	63520604	63520660	19	57	3.050000	no	no	yes	no	no	no	no	no	no	yes
182198483	chr1	63525066	63525102	18	37	2.111111	no	no	yes	no	no	no	no	yes	no	yes
182198484	chr1	63525440	63525476	19	37	2.000000	no	no	yes	no	no	no	no	no	no	yes
182198497	chr1	63567750	63567782	16	33	2.062500	no	no	yes	no	no	no	no	no	no	yes
182198508	chr1	63602595	63602643	23	49	2.173913	no	no	yes	no	no	no	no	yes	no	yes
182198519	chr1	63635803	63635830	14	28	2.000000	no	no	yes	no	no	no	no	yes	yes	yes
182198520	chr1	63642253	63642300	25	48	1.920000	no	no	yes	no	no	no	no	no	no	yes
182198521	chr1	63644143	63644185	23	43	1.913043	no	no	yes	no	no	no	no	yes	yes	yes

182198525	chr1	63663153	63663227	37	75	2.027027	no	no	no	no	no	no	no	yes	yes	no
182198527	chr1	63670072	63670129	18	58	3.222222	no	no	no	no	no	no	no	yes	no	no
182198558	chr1	63742109	63742151	22	43	2.000000	no	no	no	no	no	no	no	yes	no	no
182198560	chr1	63752077	63752106	15	30	2.000000	no	no	no	no	no	no	no	no	no	no
182198568	chr1	63774653	63774712	28	60	2.142857	yes	no	yes	no	no	no	yes	yes	yes	yes
182198576	chr1	63808025	63808097	38	73	1.973684	no	no	yes	no	no	no	no	no	no	yes
182198578	chr1	63813604	63813643	19	40	2.105263	no	no	yes	no	no	no	no	yes	no	yes
182198582	chr1	63823286	63823329	16	44	2.750000	no	no	yes	no	no	no	no	no	no	yes
182198587	chr1	63841019	63841047	12	29	2.416667	no	no	yes	no	no	no	no	no	no	yes
182198589	chr1	63850657	63850685	14	29	2.071429	no	no	yes	no	no	no	no	no	no	yes
182198592	chr1	63853764	63853795	17	32	1.941176	no	no	yes	no	no	no	no	no	no	yes
182198597	chr1	63898368	63898409	22	42	1.909091	no	no	yes	no	no	no	no	no	no	yes
182198604	chr1	63913274	63913313	20	40	2.000000	no	no	yes	no	no	no	no	no	no	yes
182198611	chr1	63962456	63962488	17	33	2.000000	no	no	yes	no	no	no	no	no	no	yes
182198614	chr1	63981993	63982030	18	38	2.111111	no	no	yes	no	no	no	no	no	no	yes
182198619	chr1	63992854	63992884	13	31	2.461539	no	no	yes	no	no	no	no	no	no	yes
182198620	chr1	64000862	64000900	18	39	2.166667	no	no	yes	no	no	no	no	no	no	yes
182198622	chr1	64007536	64007575	20	40	2.000000	no	no	yes	no	no	no	no	no	yes	yes
182198623	chr1	64013853	64013884	14	32	2.285714	no	no	yes	no	no	no	no	yes	no	yes
182198630	chr1	64027513	64027554	21	42	2.047619	no	no	yes	no	no	no	no	no	no	yes
182198633	chr1	64030865	64030906	18	42	2.333333	no	no	yes	no	no	no	no	no	no	yes
182198641	chr1	64079185	64079246	33	62	1.939394	no	no	yes	no	no	no	no	no	no	yes
182198645	chr1	64097872	64097906	17	35	2.117647	no	no	yes	no	no	no	no	yes	no	yes
182198656	chr1	64127404	64127592	104	189	1.836538	no	no	yes	no	no	no	no	no	no	yes
182198659	chr1	64157863	64157903	20	41	2.050000	no	no	yes	no	no	no	no	no	no	yes

182198660	chr1	64158765	64158796	15	32	2.133333	no	no	yes	no	no	no	no	no	no	yes
182198662	chr1	64166558	64166616	30	59	1.966667	no	no	yes	no	no	no	no	no	no	yes
182198663	chr1	64172246	64172275	15	30	2.000000	yes	no	yes	no	no	no	no	yes	yes	yes
182198664	chr1	64175015	64175081	21	67	3.238095	no	no	yes	no	no	no	no	no	no	yes
182198666	chr1	64194152	64194191	21	40	1.904762	no	no	no	no	no	no	no	yes	no	no
182198669	chr1	64199332	64199375	19	44	2.315789	no	no	no	no	no	no	no	yes	yes	no
182198670	chr1	64199666	64199695	16	30	1.937500	no	no	no	no	no	no	no	yes	no	no
182198682	chr1	64223065	64223091	11	27	2.454545	no	no	yes	no	no	no	no	no	no	yes
182198701	chr1	64321360	64321452	47	93	1.978723	no	no	no	no	no	no	no	no	yes	no
182198705	chr1	64322936	64322985	25	50	2.000000	no	no	no	no	no	no	no	no	no	no
182198749	chr1	64433386	64433465	41	80	1.951220	no	no	no	no	no	no	no	no	yes	no
182198757	chr1	64456326	64456366	20	41	2.050000	no	no	no	no	no	no	no	no	no	no
182198761	chr1	64462108	64462147	20	40	2.000000	no	no	no	no	no	no	no	yes	no	no
182198762	chr1	64466159	64466394	28	236	8.312500	no	no	no	no	no	no	no	yes	no	no
182198765	chr1	64466376	64466442	30	67	2.233333	no	no	no	no	no	no	no	yes	no	no
182198770	chr1	64466456	64466572	38	117	3.078947	no	no	no	no	no	no	no	yes	no	no
182198774	chr1	64471405	64471429	8	25	3.125000	no	no	yes	no	no	no	no	yes	yes	yes
182198776	chr1	64472200	64472280	42	81	2.023809	no	no	yes	no	no	no	no	yes	no	yes
182198785	chr1	64496746	64496771	12	26	2.166667	no	no	yes	no	no	no	no	no	yes	yes
182198795	chr1	64520913	64520950	18	38	2.111111	no	no	yes	no	no	no	no	yes	no	yes
182198796	chr1	64525718	64525759	22	42	1.909091	no	no	yes	no	no	no	no	no	no	yes
182198802	chr1	64541896	64542056	80	161	2.012500	no	no	yes	no	no	no	no	yes	no	yes
182198804	chr1	64542971	64543009	12	39	3.230769	no	no	yes	no	no	no	no	no	no	yes
182198812	chr1	64578985	64579021	19	37	1.947368	yes	no	yes	no	no	no	no	yes	no	yes
182198819	chr1	64601650	64601674	10	25	2.500000	no	no	yes	no	no	no	no	yes	yes	yes

182198825	chr1	64613351	64613394	8	44	5.750000	no	no	yes	no	no	no	no	no	yes	yes
182198831	chr1	64617389	64617414	13	26	2.000000	no	no	yes	no	no	no	no	no	no	yes
182198833	chr1	64621128	64621159	15	32	2.133333	no	no	yes	no	no	no	no	no	no	yes
182198834	chr1	64625938	64625974	18	37	2.111111	no	no	yes	no	no	no	no	no	no	yes
182198836	chr1	64633270	64633306	18	37	2.055556	no	no	yes	no	no	no	no	no	yes	yes
182198845	chr1	64680554	64680599	24	46	1.916667	no	no	yes	no	no	no	no	no	no	yes
182198846	chr1	64683433	64683484	26	52	2.038461	no	no	yes	no	no	no	no	yes	yes	yes
182198849	chr1	64687508	64687557	27	50	1.925926	no	no	yes	no	no	no	no	yes	no	yes
182198865	chr1	64739663	64739700	21	38	1.904762	no	no	no	no	no	no	no	no	no	no
182198868	chr1	64745166	64745203	20	38	1.950000	no	no	no	yes	yes	no	yes	yes	no	yes
182198873	chr1	64751748	64751788	19	41	2.157895	no	no	yes	no	no	no	no	no	no	yes
182198875	chr1	64758893	64758937	8	45	5.875000	no	no	yes	no	no	no	no	yes	no	yes
182198877	chr1	64759150	64759185	18	36	2.000000	no	no	yes	no	no	no	no	no	no	yes
182198899	chr1	64856933	64856963	15	31	2.133333	no	no	yes	no	no	no	no	yes	no	yes
182198903	chr1	64860827	64861049	120	223	1.858333	no	no	yes	no	no	no	no	no	no	yes
182198913	chr1	64900581	64900620	20	40	2.000000	no	no	yes	no	no	no	no	no	no	yes
182198920	chr1	64941901	64941929	12	29	2.416667	yes	no	yes	no	no	no	no	yes	yes	yes
182198924	chr1	64963321	64963354	17	34	2.000000	no	no	yes	no	no	no	no	no	no	yes
182198927	chr1	64969767	64969794	14	28	2.000000	no	no	yes	no	no	no	no	yes	yes	yes
182198934	chr1	64974121	64974159	20	39	1.950000	no	no	yes	no	yes	yes	no	yes	no	yes
182198950	chr1	65001600	65001679	35	80	2.285714	no	no	yes	no	no	no	no	yes	yes	yes
182198959	chr1	65014363	65014406	23	44	1.956522	no	no	yes	no	no	no	no	yes	no	yes
182198961	chr1	65014396	65014436	22	41	1.954545	no	no	yes	no	no	no	no	yes	no	yes
182198981	chr1	65045482	65045527	20	46	2.300000	no	no	yes	no	no	no	no	yes	yes	yes
182198987	chr1	65053395	65053447	23	53	2.304348	no	no	yes	no	no	no	no	no	no	yes

182198988	chr1	65054399	65054427	14	29	2.071429	no	no	yes	no	no	no	no	no	no	yes
182198996	chr1	65067870	65067911	14	42	3.000000	yes	no	no	no	no	no	yes	yes	yes	no
182198999	chr1	65073918	65073943	13	26	2.000000	no	no	no	no	no	no	no	yes	no	no
182199001	chr1	65077940	65078077	68	138	2.029412	no	no	no	no	yes	no	no	no	no	yes
182199019	chr1	65115821	65115850	15	30	2.000000	no	no	no	no	no	no	no	no	yes	no
182199038	chr1	65148141	65148189	18	49	2.722222	yes	no	yes	no	no	no	yes	yes	yes	yes
182199039	chr1	65149264	65149300	19	37	1.947368	no	no	yes	no	no	no	no	yes	yes	yes
182199040	chr1	65150020	65150123	52	104	2.000000	no	no	yes	no	no	no	no	yes	yes	yes
182199041	chr1	65152005	65152044	20	40	2.000000	no	no	yes	no	no	no	no	yes	yes	yes
182199083	chr1	65287091	65287133	20	43	2.150000	no	no	yes	no	no	no	no	yes	no	yes
182199094	chr1	65309296	65309328	17	33	1.941176	yes	no	yes	no	no	no	no	yes	yes	yes
182199101	chr1	65319904	65319941	20	38	1.900000	no	no	yes	no	no	no	no	yes	yes	yes
182199104	chr1	65347718	65347762	16	45	2.812500	no	no	yes	no	no	no	no	yes	no	yes
182199108	chr1	65351433	65351473	20	41	2.050000	no	no	yes	no	no	no	no	no	no	yes
182199120	chr1	65368731	65368783	25	53	2.160000	no	no	yes	no	no	no	no	yes	no	yes
182199126	chr1	65380307	65380345	18	39	2.166667	no	no	yes	no	no	yes	no	no	no	yes
182199132	chr1	65409830	65409871	22	42	1.909091	no	no	yes	no	no	no	no	yes	no	yes
182199138	chr1	65433794	65433824	14	31	2.214286	no	yes	yes	no	no	no	no	yes	no	yes
182199160	chr1	65507452	65507555	40	104	2.650000	no	no	yes	no	no	no	no	no	no	yes
182199173	chr1	65558423	65558471	24	49	2.041667	no	no	yes	no	no	no	no	yes	yes	yes
182199178	chr1	65570078	65570128	25	51	2.080000	no	no	yes	no	no	no	no	no	no	yes
182199191	chr1	65641872	65641924	26	53	2.076923	no	no	no	no	no	yes	no	no	yes	no
182199192	chr1	65641927	65641972	22	46	2.136364	no	no	no	no	no	yes	no	no	no	no
182199213	chr1	65680798	65680837	20	40	2.000000	no	no	no	no	no	no	no	no	no	no
182199215	chr1	65691089	65691176	20	88	4.400000	no	no	no	no	no	no	no	no	no	no

182199217	chr1	65694080	65694104	11	25	2.272727	no	no	no	no	no	no	no	yes	yes	no
182199220	chr1	65698862	65698895	16	34	2.125000	no	no	no	no	no	no	no	no	no	no
182199226	chr1	65721581	65721617	19	37	2.000000	no	no	no	no	no	no	no	yes	no	no
182199247	chr1	65824610	65824667	22	58	2.636364	no	no	yes	no	no	no	no	no	no	yes
182199253	chr1	65836618	65836686	22	69	3.136364	no	no	yes	no	no	no	no	no	no	yes
182199270	chr1	65887261	65887417	60	157	2.683333	no	no	yes	no	no	no	no	yes	no	yes
182199284	chr1	65911356	65911388	17	33	1.941176	no	no	yes	no	no	no	no	no	no	yes
182199288	chr1	65921746	65921778	15	33	2.200000	no	no	yes	no	no	no	no	yes	no	yes
182199291	chr1	65927406	65927535	37	130	3.621622	no	no	yes	no	no	no	no	no	no	yes
182199297	chr1	65951745	65951769	9	25	2.777778	no	no	yes	no	no	no	no	no	no	yes
182199301	chr1	65958823	65958869	24	47	1.958333	no	no	yes	no	no	no	no	yes	yes	yes
182199302	chr1	65961735	65961782	24	48	2.000000	no	no	yes	no	no	no	no	no	no	yes
182199305	chr1	65970953	65970995	22	43	1.954545	no	no	yes	no	no	no	no	no	yes	yes
182199308	chr1	65981204	65981255	27	52	1.925926	no	no	yes	no	no	no	no	no	no	yes
182199318	chr1	66045247	66045271	13	25	1.923077	no	no	yes	no	no	no	no	no	no	yes
182199320	chr1	66071031	66071076	23	46	2.043478	no	no	yes	no	no	no	no	yes	no	yes
182199327	chr1	66083743	66083781	19	39	2.052632	no	no	yes	no	no	no	no	no	no	yes
182199328	chr1	66089671	66089712	19	42	2.210526	no	no	yes	no	no	no	no	no	no	yes
182199336	chr1	66112668	66112704	19	37	1.947368	no	no	yes	no	no	no	no	yes	no	yes
182199339	chr1	66123496	66123564	25	69	2.880000	no	no	yes	no	no	no	no	no	no	yes
182199348	chr1	66141584	66141609	13	26	2.000000	no	no	yes	no	no	no	no	no	no	yes
182199351	chr1	66148270	66148308	21	39	1.952381	no	no	yes	no	no	no	no	no	no	yes
182199353	chr1	66152581	66152638	28	58	2.071429	no	no	yes	no	no	no	no	no	no	yes
182199356	chr1	66155596	66155632	14	37	2.642857	no	no	yes	no	no	no	no	no	no	yes
182199363	chr1	66178513	66178558	24	46	2.000000	no	no	yes	no	no	no	no	no	no	yes

182199365	chr1	66195744	66196102	178	359	2.016854	no	no	yes	no	no	no	no	no	no	yes
182199370	chr1	66215380	66215409	7	30	4.428571	no	no	yes	no	no	no	no	no	yes	yes
182199383	chr1	66263762	66263795	11	34	3.090909	no	no	yes	no	no	no	no	yes	no	yes
182199397	chr1	66293581	66293668	46	88	1.934783	no	no	yes	no	no	no	no	yes	yes	yes
182199398	chr1	66297612	66297638	14	27	1.928571	no	no	yes	no	no	no	no	no	no	yes
182199402	chr1	66339817	66339863	14	47	3.400000	no	no	yes	no	no	no	no	yes	no	yes
182199408	chr1	66346979	66347073	46	95	2.065217	no	no	yes	no	no	no	no	yes	yes	yes
182199410	chr1	66361412	66361449	20	38	1.950000	no	no	yes	no	no	no	no	no	no	yes
182199437	chr1	66440993	66441043	26	51	1.961538	no	no	no	no	no	no	no	no	no	no
182199438	chr1	66441032	66441086	28	55	2.035714	no	no	no	no	no	no	no	no	yes	no
182199441	chr1	66441125	66441239	58	115	1.982759	no	no	no	no	no	no	no	no	yes	no
182199445	chr1	66441308	66441393	30	86	2.933333	no	no	no	no	no	no	no	no	no	no
182199463	chr1	66445562	66445631	21	70	3.333333	no	no	no	no	no	no	no	yes	yes	no
182199465	chr1	66447153	66447185	15	33	2.200000	no	no	no	no	no	no	no	no	no	no
182199469	chr1	66479186	66479232	24	47	2.000000	no	no	no	no	no	no	no	no	no	no
182199470	chr1	66480041	66480089	24	49	2.125000	no	no	no	no	no	no	no	yes	no	no
182199472	chr1	66488752	66488793	17	42	2.529412	no	no	no	no	no	no	no	yes	yes	no
182199475	chr1	66506693	66506727	13	35	2.692308	no	no	no	no	no	no	no	yes	yes	no
182199484	chr1	66518853	66518884	16	32	2.000000	no	no	no	no	no	no	no	no	no	no
182199491	chr1	66560486	66560535	26	50	1.923077	no	no	yes	no	no	no	no	no	no	yes
182199492	chr1	66560676	66560703	14	28	2.000000	no	no	yes	no	no	no	no	no	no	yes
182199502	chr1	66594627	66594655	14	29	2.071429	no	no	yes	no	no	no	no	yes	no	yes
182199506	chr1	66608988	66609047	26	60	2.307692	no	no	yes	no	no	no	no	yes	yes	yes
182199507	chr1	66609380	66609410	16	31	1.937500	no	no	yes	no	no	no	no	no	yes	yes
182199510	chr1	66616229	66616278	17	50	2.941176	no	no	yes	no	no	no	no	no	no	yes

182199511	chr1	66616539	66616579	13	41	3.153846	no	no	yes	no	no	no	no	no	no	yes
182199515	chr1	66630987	66631046	19	60	3.047619	no	no	yes	no	no	no	no	yes	no	yes
182199517	chr1	66632713	66632756	22	44	2.000000	no	no	yes	no	no	no	no	no	no	yes
182199519	chr1	66637731	66637822	42	92	2.190476	no	no	yes	no	no	no	no	no	no	yes
182199531	chr1	66666908	66666947	21	40	2.000000	no	no	yes	no	yes	no	no	no	no	yes
182199534	chr1	66669545	66669583	17	39	2.294118	no	no	yes	no	no	no	no	no	yes	yes
182199546	chr1	66703747	66703799	23	53	2.333333	no	no	yes	no	no	no	no	yes	no	yes
182199549	chr1	66705042	66705066	13	25	1.923077	no	no	yes	no	no	yes	no	yes	no	yes
182199550	chr1	66705735	66705781	21	47	2.238095	no	no	yes	no	no	yes	no	yes	no	yes
182199556	chr1	66709989	66710014	12	26	2.166667	no	no	yes	no	no	no	no	no	no	yes
182199559	chr1	66717591	66717630	20	40	2.000000	no	no	yes	no	no	no	no	no	no	yes
182199564	chr1	66736111	66736159	26	49	1.923077	no	no	yes	no	no	no	no	no	no	yes
182199567	chr1	66736152	66736287	54	136	2.535714	no	no	yes	no	no	no	no	no	no	yes
182199570	chr1	66736322	66736660	27	339	12.437500	no	no	yes	no	no	no	no	yes	no	yes
182199574	chr1	66746906	66746960	23	55	2.375000	no	yes	no	no	no	no	no	no	no	yes
182199589	chr1	66792787	66792828	22	42	1.954545	no	no	no	no	no	no	no	no	no	no
182199592	chr1	66800362	66800397	17	36	2.117647	no	no	yes	no	no	no	no	no	no	yes
182199595	chr1	66809495	66809527	13	33	2.538461	no	no	no	no	no	no	no	no	no	no
182199608	chr1	66840235	66840268	17	34	2.117647	no	no	yes	no	no	no	no	no	no	yes
182199638	chr1	66904060	66904100	22	41	1.909091	no	no	yes	no	no	yes	no	no	no	yes
182199639	chr1	66906808	66906860	23	53	2.391304	no	no	yes	no	no	no	no	yes	no	yes
182199644	chr1	66924880	66924925	19	46	2.450000	yes	no	no	no	yes	no	no	yes	yes	yes
182199645	chr1	66925046	66925086	21	41	1.952381	yes	no	no	yes	yes	no	no	yes	no	yes
182199647	chr1	66936150	66936186	19	37	2.000000	no	no	yes	no	no	no	no	yes	no	yes
182199650	chr1	66939883	66939922	20	40	2.000000	no	no	yes	no	no	no	no	no	no	yes

182199652	chr1	66944457	66944486	14	30	2.142857	no	no	yes	no	no	no	no	yes	no	yes
182199653	chr1	66945092	66945128	17	37	2.235294	no	no	yes	no	no	no	no	yes	yes	yes
182199656	chr1	66954383	66954423	22	41	1.909091	no	no	yes	no	no	no	no	yes	yes	yes
182199659	chr1	66958125	66958161	15	37	2.466667	yes	yes	no	yes	no	no	no	no	no	yes
182199661	chr1	66961054	66961116	31	63	2.032258	no	no	yes	no	no	yes	no	no	yes	yes
182199662	chr1	66965374	66965412	14	39	2.857143	no	no	yes	no	no	no	no	no	no	yes
182199671	chr1	67029831	67029864	15	34	2.266667	no	no	yes	no	no	no	no	yes	no	yes
182199684	chr1	67076992	67077042	21	51	2.476191	no	no	no	no	no	no	no	no	no	no
182199695	chr1	67110759	67110796	17	38	2.235294	no	no	yes	no	no	no	no	no	no	yes
182199697	chr1	67135171	67135207	19	37	2.000000	yes	no	yes	no	no	no	no	yes	yes	yes
182199700	chr1	67158851	67159276	28	426	15.000000	no	no	yes	no	no	no	no	yes	no	yes
182199715	chr1	67218266	67218327	18	62	3.444444	no	no	yes	no	no	no	no	no	no	yes
182199719	chr1	67218583	67218642	19	60	3.157895	yes	no	yes	no	no	no	no	no	no	yes
182199722	chr1	67218977	67219028	22	52	2.363636	yes	no	yes	no	no	no	no	no	no	yes
182199744	chr1	67285408	67285433	12	26	2.166667	no	no	no	no	no	no	no	no	no	no
182199748	chr1	67303928	67303959	16	32	2.062500	no	no	no	no	no	no	no	no	no	no
182199752	chr1	67307092	67307121	14	30	2.142857	yes	no	no	no	no	no	no	no	no	no
182199757	chr1	67321923	67321955	17	33	1.941176	no	no	yes	no	no	no	no	no	no	yes
182199760	chr1	67333008	67333044	18	37	2.111111	no	no	yes	no	no	no	no	yes	no	yes
182199768	chr1	67342485	67342537	24	53	2.291667	no	no	yes	no	no	no	no	yes	yes	yes
182199772	chr1	67358582	67358617	16	36	2.312500	no	no	yes	no	no	no	no	no	no	yes
182199783	chr1	67398185	67398250	31	66	2.129032	no	no	no	no	no	no	no	no	no	no
182199788	chr1	67425573	67425611	20	39	1.950000	yes	no	yes	no	no	no	no	yes	yes	yes
182199795	chr1	67450354	67450384	14	31	2.214286	no	no	no	no	no	no	no	no	no	no
182199821	chr1	67504132	67504209	22	78	3.545455	no	no	no	no	no	no	no	yes	no	no

182199835	chr1	67535256	67535299	20	44	2.200000	no	no	no	no	no	no	no	yes	no	no
182199841	chr1	67551537	67551590	20	54	2.700000	no	no	no	no	no	no	no	no	yes	no
182199843	chr1	67555560	67555605	17	46	2.722222	no	no	no	no	no	no	no	yes	yes	no
182199847	chr1	67570916	67570959	15	44	3.000000	no	no	no	no	no	no	no	yes	no	no
182199852	chr1	67605066	67605101	19	36	1.947368	no	no	no	no	no	no	no	no	yes	no
182199877	chr1	67669669	67669698	14	30	2.142857	no	no	no	no	no	no	no	no	no	no
182199878	chr1	67670251	67670495	122	245	2.008197	no	no	no	no	no	no	no	no	no	no
182199879	chr1	67671086	67671132	19	47	2.473684	no	no	no	no	no	no	no	no	no	no
182199888	chr1	67685200	67685234	14	35	2.500000	yes	yes	no	no	yes	no	yes	yes	yes	yes
182199889	chr1	67688215	67688258	22	44	2.045455	no	yes	no	no	no	yes	no	yes	no	yes
182199901	chr1	67725375	67725399	13	25	1.923077	no	no	yes	no	no	no	no	yes	yes	yes
182199902	chr1	67726857	67726915	24	59	2.458333	no	no	yes	no	no	no	no	no	yes	yes
182199904	chr1	67762685	67762716	14	32	2.285714	no	no	yes	no	no	no	no	no	no	yes
182199914	chr1	67787382	67787484	50	103	2.100000	no	no	yes	no	no	no	no	yes	yes	yes
182199916	chr1	67790298	67790322	12	25	2.083333	no	no	yes	no	no	no	no	no	no	yes
182199917	chr1	67794849	67794891	8	43	5.375000	no	no	yes	no	no	no	no	no	no	yes
182199937	chr1	67834575	67834610	18	36	2.000000	no	no	yes	no	no	no	no	yes	yes	yes
182199940	chr1	67844422	67844463	22	42	1.909091	no	no	yes	no	no	no	no	no	no	yes
182199942	chr1	67846747	67846775	15	29	1.933333	no	no	yes	no	no	no	no	no	yes	yes
182199943	chr1	67849205	67849245	21	41	1.952381	no	no	yes	no	no	no	no	no	no	yes
182199945	chr1	67850116	67850144	15	29	1.933333	no	no	yes	no	no	no	no	yes	yes	yes
182199949	chr1	67858476	67858512	14	37	2.642857	no	no	yes	no	no	no	no	no	no	yes
182199960	chr1	67883671	67883711	21	41	2.000000	no	no	yes	no	no	no	no	no	yes	yes
182199991	chr1	67993132	67993173	19	42	2.315789	no	no	yes	no	no	no	no	no	no	yes
182199992	chr1	67993157	67993195	16	39	2.562500	no	no	yes	no	no	no	no	no	no	yes

182199993	chr1	67993184	67993274	42	91	2.279070	no	no	yes	no	no	no	no	no	no	yes
182200000	chr1	68013327	68013369	13	43	3.384615	no	no	yes	no	no	no	no	no	no	yes
182200013	chr1	68045402	68045432	15	31	2.133333	no	no	yes	no	no	yes	no	yes	yes	yes
182200044	chr1	68140310	68140345	18	36	2.000000	no	no	yes	no	no	no	no	no	no	yes
182200045	chr1	68154048	68154086	19	39	2.052632	no	no	yes	no	no	no	no	no	no	yes
182200047	chr1	68163192	68163235	17	44	2.705882	yes	no	yes	no	no	no	no	yes	no	yes
182200049	chr1	68183223	68183268	22	46	2.090909	no	no	yes	no	no	yes	no	no	no	yes
182200052	chr1	68196144	68196177	15	34	2.333333	no	no	yes	no	no	no	no	no	no	yes
182200055	chr1	68211341	68211393	21	53	2.523809	no	no	yes	no	no	no	no	yes	no	yes
182200059	chr1	68222897	68222938	13	42	3.307692	no	no	yes	no	no	no	no	no	no	yes
182200062	chr1	68227749	68227775	12	27	2.250000	no	no	yes	no	no	no	no	yes	no	yes
182200063	chr1	68228647	68228677	11	31	2.909091	no	no	yes	no	no	no	no	no	no	yes
182200069	chr1	68232582	68232633	20	52	2.600000	yes	no	yes	no	no	no	no	yes	yes	yes
182200088	chr1	68277626	68277658	16	33	2.062500	no	no	no	no	no	no	no	no	no	no
182200118	chr1	68331047	68331080	17	34	2.000000	no	no	no	no	no	no	no	yes	no	no
182200122	chr1	68338538	68338572	17	35	2.058824	no	no	no	no	no	no	no	yes	no	no
182200131	chr1	68357346	68357386	21	41	1.952381	no	no	no	no	no	no	no	no	no	no
182200132	chr1	68359803	68359832	14	30	2.142857	no	no	no	no	no	no	no	no	no	no
182200139	chr1	68383134	68383168	18	35	1.944444	no	no	no	no	no	no	no	yes	yes	no
182200155	chr1	68408654	68408712	29	59	2.034483	no	no	no	no	no	no	no	no	no	no
182200170	chr1	68435175	68435229	22	55	2.500000	no	no	yes	no	no	no	no	no	no	yes
182200172	chr1	68440780	68440820	19	41	2.157895	no	no	yes	no	no	no	no	no	no	yes
182200183	chr1	68497588	68497655	35	68	1.942857	yes	no	yes	no	no	no	no	yes	yes	yes
182200193	chr1	68517240	68517276	20	37	1.950000	no	no	yes	no	no	no	no	no	no	yes
182200196	chr1	68537790	68537835	24	46	2.000000	no	yes	no	no	no	no	no	no	no	yes

182200208	chr1	68589152	68589185	17	34	2.000000	no	no	no	no	no	no	no	no	no	no
182200214	chr1	68600823	68600874	25	52	2.080000	no	no	no	no	no	no	no	no	yes	no
182200215	chr1	68603878	68603922	22	45	2.136364	no	no	no	no	no	no	no	no	no	no
182200218	chr1	68621841	68621896	13	56	4.384615	no	no	no	no	no	no	no	no	no	no
182200220	chr1	68627241	68627282	21	42	2.000000	no	no	no	no	no	no	no	no	no	no
182200222	chr1	68629520	68629566	24	47	1.958333	no	no	no	no	no	no	no	no	no	no
182200227	chr1	68633768	68634146	190	379	2.031579	no	no	yes	no	no	no	no	yes	no	yes
182200232	chr1	68648428	68648492	16	65	4.117647	no	no	no	no	no	no	no	no	no	no
182200251	chr1	68674518	68674667	80	150	1.875000	no	no	no	no	no	no	no	no	no	no
182200254	chr1	68691123	68691191	18	69	3.842105	no	no	yes	no	no	no	no	no	no	yes
182200258	chr1	68721521	68721571	26	51	2.038461	no	no	yes	no	no	no	no	no	no	yes
182200271	chr1	68794384	68794430	21	47	2.285714	no	no	yes	no	no	no	no	no	no	yes
182200278	chr1	68815293	68815417	39	125	3.195122	no	no	yes	no	no	no	no	no	yes	yes
182200289	chr1	68896820	68896859	19	40	2.105263	no	no	yes	no	no	no	no	no	no	yes
182200296	chr1	68898633	68898674	18	42	2.388889	no	no	yes	no	no	no	no	no	no	yes
182200300	chr1	68898737	68898791	15	55	3.666667	no	no	yes	no	no	no	no	no	no	yes
182200301	chr1	68899882	68899935	18	54	3.105263	no	no	yes	no	no	no	no	no	no	yes
182200311	chr1	68919417	68919455	20	39	1.950000	no	no	yes	no	no	no	no	no	no	yes
182200312	chr1	68930037	68930148	52	112	2.153846	no	no	yes	no	no	no	no	no	no	yes
182200317	chr1	68971557	68971598	22	42	1.909091	no	no	no	no	no	no	no	no	no	no
182200318	chr1	68972599	68972645	16	47	2.941176	no	no	no	no	no	no	no	no	no	no
182200329	chr1	69000769	69000814	24	46	1.958333	no	no	yes	no	no	no	no	no	no	yes
182200331	chr1	69006813	69006838	12	26	2.166667	no	no	yes	no	no	no	no	no	no	yes
182200337	chr1	69018915	69018966	18	52	2.888889	no	no	yes	no	no	no	no	no	yes	yes
182200342	chr1	69023413	69023460	25	48	1.920000	no	yes	no	no	no	no	no	no	no	yes

182200359	chr1	69063658	69063708	24	51	2.125000	no	no	yes	no	no	no	no	no	no	yes
182200365	chr1	69087562	69087604	22	43	1.954545	no	yes	yes	no	no	no	no	yes	no	yes
182200378	chr1	69128851	69128890	22	40	1.909091	no	no	yes	no	no	no	no	no	no	yes
182200382	chr1	69136234	69136270	19	37	1.947368	no	no	yes	no	no	no	no	no	no	yes
182200383	chr1	69146902	69146933	15	32	2.133333	no	no	yes	no	no	no	no	no	no	yes
182200386	chr1	69159175	69159212	14	38	2.714286	no	no	yes	no	no	no	no	no	no	yes
182200393	chr1	69187471	69187519	16	49	3.062500	no	no	yes	no	no	no	no	no	no	yes
182200399	chr1	69193486	69193642	86	157	1.837209	no	no	yes	no	no	no	no	no	no	yes
182200400	chr1	69197120	69197155	14	36	2.642857	no	no	yes	no	no	no	no	no	no	yes
182200422	chr1	69231971	69232001	16	31	1.937500	no	no	yes	no	no	no	no	no	no	yes
182200434	chr1	69266545	69266626	38	82	2.315789	no	no	no	no	no	no	no	no	no	no
182200435	chr1	69267505	69267545	21	41	1.952381	no	no	no	no	no	no	no	no	no	no
182200440	chr1	69279080	69279142	21	63	3.000000	no	no	no	no	no	no	no	yes	no	no
182200460	chr1	69366081	69366132	26	52	2.000000	no	no	no	no	no	no	no	no	no	no
182200463	chr1	69376979	69377018	20	40	2.100000	no	no	no	no	no	no	no	yes	yes	no
182200466	chr1	69378857	69378907	22	51	2.363636	no	no	no	no	no	no	no	no	no	no
182200469	chr1	69378994	69379026	17	33	1.941176	no	no	no	no	no	no	no	no	no	no
182200473	chr1	69385711	69385782	37	72	2.027027	no	no	no	no	no	no	no	no	no	no
182200476	chr1	69397722	69397765	24	44	1.916667	no	no	no	no	no	no	no	no	no	no
182200501	chr1	69481461	69481501	21	41	1.952381	no	no	no	no	no	no	no	no	no	no
182200509	chr1	69498860	69498915	27	56	2.222222	no	no	no	no	no	no	no	yes	no	no
182200517	chr1	69518628	69518766	24	139	5.875000	no	no	no	no	no	no	no	no	no	no
182200523	chr1	69531247	69531272	11	26	2.363636	no	no	no	no	no	no	no	no	no	no
182200525	chr1	69531819	69531878	24	60	2.500000	no	no	no	no	no	no	no	no	no	no
182200527	chr1	69533319	69533355	17	37	2.176471	no	no	no	no	no	no	no	yes	no	no

182200534	chr1	69544314	69544361	24	48	2.000000	no	no	no	no	no	no	no	no	no	no
182200543	chr1	69572858	69572887	15	30	2.000000	no	no	yes	no	no	no	no	no	no	yes
182200544	chr1	69580349	69580386	20	38	1.900000	no	no	yes	no	no	no	no	no	no	yes
182200551	chr1	69604278	69604310	15	33	2.266667	no	no	yes	no	no	no	no	no	no	yes
182200558	chr1	69622814	69622862	24	49	2.041667	no	no	yes	no	no	no	no	no	no	yes
182200559	chr1	69630520	69630560	22	41	1.909091	no	no	yes	no	no	no	no	no	no	yes
182200560	chr1	69630887	69630912	11	26	2.363636	no	no	yes	no	no	no	no	no	no	yes
182200562	chr1	69640369	69640412	23	44	1.913043	no	no	yes	no	no	no	no	no	no	yes
182200564	chr1	69648044	69648090	16	47	3.000000	no	no	yes	no	no	no	no	no	no	yes
182200565	chr1	69649013	69649044	11	32	2.909091	no	no	yes	no	no	no	no	no	no	yes
182200566	chr1	69656946	69657007	28	62	2.321429	no	no	yes	no	no	no	no	no	no	yes
182200569	chr1	69657146	69657179	16	34	2.125000	no	no	yes	no	no	no	no	no	no	yes
182200570	chr1	69658060	69658095	17	36	2.235294	no	no	yes	no	no	no	no	no	yes	yes
182200585	chr1	69709809	69709978	88	170	1.943182	no	no	yes	no	yes	no	no	no	no	yes
182200589	chr1	69720800	69720839	11	40	3.818182	no	no	yes	no	no	no	no	no	no	yes
182200591	chr1	69722967	69723011	22	45	2.090909	no	no	yes	no	no	no	no	no	no	yes
182200592	chr1	69732270	69732310	21	41	2.000000	no	no	yes	no	no	no	no	no	no	yes
182200597	chr1	69755200	69755321	60	122	2.033333	no	no	yes	no	no	no	no	no	no	yes
182200599	chr1	69755784	69755812	15	29	1.933333	no	no	yes	no	no	no	no	yes	yes	yes
182200601	chr1	69757144	69757178	14	35	2.500000	no	no	yes	no	no	no	no	no	no	yes
182200608	chr1	69776639	69776857	112	219	1.955357	no	no	yes	no	no	no	no	yes	yes	yes
182200616	chr1	69787957	69788010	27	54	2.000000	no	no	yes	no	no	no	no	yes	no	yes
182200621	chr1	69814628	69814656	15	29	1.933333	no	no	yes	no	no	no	no	no	no	yes
182200626	chr1	69830399	69830423	10	25	2.500000	no	no	yes	no	no	no	no	no	no	yes
182200628	chr1	69840983	69841009	12	27	2.250000	no	no	yes	no	no	no	no	no	no	yes

182200629	chr1	69845802	69845833	14	32	2.357143	no	no	yes	no	no	no	no	yes	no	yes
182200634	chr1	69864328	69864363	12	36	3.000000	no	no	yes	no	no	no	no	yes	no	yes
182200637	chr1	69875117	69875146	15	30	2.000000	no	no	yes	no	no	yes	no	no	no	yes
182200640	chr1	69878859	69878917	25	59	2.346154	yes	no	yes	no	no	no	no	no	no	yes
182200649	chr1	69914539	69914603	34	65	1.911765	no	no	yes	no	no	no	no	no	no	yes
182200656	chr1	69919956	69919998	17	43	2.529412	no	yes	yes	no	no	yes	no	no	no	yes
182200667	chr1	69957026	69957093	35	68	1.942857	no	no	yes	no	no	no	no	no	no	yes
182200673	chr1	69966447	69966479	16	33	2.125000	no	no	yes	no	no	no	no	no	no	yes
182200681	chr1	69978287	69978342	20	56	2.800000	no	no	yes	no	no	no	no	no	no	yes
182200685	chr1	69990200	69990226	13	27	2.076923	no	no	yes	no	no	no	no	no	no	yes
182200687	chr1	70001343	70001389	22	47	2.181818	no	no	yes	no	no	no	no	no	no	yes
182200698	chr1	70034269	70034359	45	91	2.022222	no	no	yes	no	no	no	no	yes	no	yes
182200700	chr1	70047970	70048002	12	33	2.750000	no	no	yes	no	no	no	no	no	no	yes
182200701	chr1	70052439	70052467	14	29	2.071429	no	no	yes	no	no	no	no	no	no	yes
182200703	chr1	70056234	70056265	15	32	2.133333	no	no	yes	no	no	no	no	no	no	yes
182200705	chr1	70060974	70061160	94	187	2.031915	no	no	yes	no	no	no	no	no	no	yes
182200706	chr1	70062110	70062139	11	30	2.666667	no	no	yes	no	no	no	no	no	no	yes
182200718	chr1	70100767	70100802	18	36	2.000000	no	no	yes	no	no	no	no	no	no	yes
182200719	chr1	70103865	70103892	13	28	2.153846	no	no	yes	no	no	no	no	yes	no	yes
182200722	chr1	70108738	70108823	44	86	2.000000	no	no	yes	no	no	no	no	no	no	yes
182200725	chr1	70113528	70113592	33	65	1.969697	no	no	yes	no	no	no	no	yes	no	yes
182200726	chr1	70113599	70113645	24	47	1.958333	no	no	yes	no	no	no	no	no	no	yes
182200728	chr1	70115781	70115826	23	46	2.000000	no	no	yes	no	no	no	no	yes	no	yes
182200729	chr1	70115829	70115874	22	46	2.090909	no	no	yes	no	no	no	no	yes	no	yes
182200731	chr1	70119933	70119988	28	56	2.000000	no	no	yes	no	no	no	no	no	no	yes

182200732	chr1	70124207	70124244	20	38	1.900000	no	no	yes	no	no	yes	no	no	no	yes
182200733	chr1	70130611	70130645	17	35	2.058824	no	no	yes	no	no	no	no	no	no	yes
182200734	chr1	70131871	70131915	19	45	2.368421	no	no	yes	no	no	no	no	no	no	yes
182200737	chr1	70134156	70134186	12	31	2.583333	no	no	yes	no	no	no	no	yes	yes	yes
182200741	chr1	70155593	70155643	26	51	1.961538	no	no	yes	no	no	no	no	yes	no	yes
182200743	chr1	70156282	70156313	16	32	2.000000	no	no	yes	no	no	no	no	no	no	yes
182200746	chr1	70158990	70159046	28	57	2.071429	no	no	yes	no	no	no	no	no	no	yes
182200747	chr1	70166158	70166189	15	32	2.133333	no	no	yes	no	no	no	no	no	no	yes
182200749	chr1	70166895	70166942	25	48	1.960000	no	no	yes	no	no	no	no	no	no	yes
182200757	chr1	70189536	70189571	16	36	2.250000	no	no	yes	no	no	no	no	no	no	yes
182200764	chr1	70207615	70207656	22	42	1.909091	no	no	yes	no	no	no	no	yes	no	yes
182200784	chr1	70242346	70242388	22	43	1.954545	no	no	yes	no	no	no	no	no	yes	yes
182200790	chr1	70260905	70260945	20	41	2.100000	no	yes	no	no	no	yes	no	no	no	yes
182200805	chr1	70297008	70297052	22	45	2.045455	no	no	yes	no	no	no	no	no	no	yes
182200806	chr1	70301029	70301073	18	45	2.473684	no	no	yes	no	no	no	no	no	no	yes
182200808	chr1	70301164	70301229	24	66	2.692308	no	no	yes	no	no	no	no	no	no	yes
182200815	chr1	70317931	70318015	43	85	2.000000	no	no	yes	no	no	no	no	no	no	yes
182200823	chr1	70342608	70342654	18	47	2.611111	no	no	yes	no	no	no	no	yes	no	yes
182200830	chr1	70357621	70357650	13	30	2.307692	no	no	yes	no	no	no	no	no	no	yes
182200837	chr1	70386291	70386623	166	333	2.006024	no	no	no	no	no	yes	no	no	yes	no
182200839	chr1	70416272	70416300	15	29	1.933333	no	no	yes	no	no	no	no	yes	no	yes
182200842	chr1	70432992	70433019	13	28	2.153846	no	no	yes	no	no	no	no	no	no	yes
182200846	chr1	70443044	70443132	23	89	3.869565	no	no	no	no	no	no	no	no	no	no
182200854	chr1	70454541	70454580	14	40	2.857143	no	no	no	no	no	no	no	no	no	no
182200855	chr1	70455155	70455226	28	72	2.571429	no	no	no	no	no	no	no	no	no	no

182200870	chr1	70518968	70519004	19	37	1.947368	no	no	no	no	no	no	no	no	no	no
182200887	chr1	70567797	70567833	18	37	2.111111	no	no	no	no	no	no	no	no	no	no
182200900	chr1	70624665	70624799	37	135	3.575000	no	no	no	no	no	no	no	yes	no	no
182200905	chr1	70633378	70633408	15	31	2.066667	no	no	no	no	no	no	no	yes	no	no
182200907	chr1	70639785	70639821	19	37	1.947368	no	no	no	no	no	no	no	no	no	no
182200910	chr1	70646531	70647988	102	1458	14.203252	no	no	no	no	no	no	no	no	no	no
182200916	chr1	70648050	70648134	30	85	2.966667	no	no	no	no	no	no	no	no	no	no
182200930	chr1	70658055	70658122	23	68	2.956522	no	no	no	no	no	no	no	no	no	no
182200932	chr1	70667606	70667634	9	29	3.333333	no	no	no	no	no	no	no	yes	no	no
182200934	chr1	70671695	70671724	11	30	2.666667	no	no	no	no	no	no	no	no	no	no
182200935	chr1	70678012	70678056	23	45	1.956522	no	no	no	no	no	no	no	no	no	no
182200939	chr1	70697607	70697802	83	196	2.416667	no	no	no	no	no	no	no	yes	no	no
182200949	chr1	70712308	70712372	19	65	3.428571	no	no	yes	no	no	no	no	no	no	yes
182200970	chr1	70790757	70790871	59	115	2.000000	no	no	no	no	no	no	no	no	yes	no
182200975	chr1	70801557	70801641	44	85	2.000000	no	no	no	no	no	no	no	no	no	no
182200981	chr1	70801823	70801860	16	38	2.500000	no	no	no	no	no	no	no	no	no	no
182200988	chr1	70809817	70809857	21	41	1.952381	no	no	no	no	no	no	no	no	no	no
182200989	chr1	70812450	70812504	27	55	2.074074	no	no	no	no	no	no	no	yes	no	no
182201002	chr1	70861424	70861461	9	38	4.100000	no	no	yes	no	no	no	no	no	no	yes
182201004	chr1	70862719	70862756	20	38	1.950000	no	no	yes	no	no	no	no	no	no	yes
182201008	chr1	70874074	70874113	16	40	2.500000	no	no	yes	no	no	no	no	yes	no	yes
182201011	chr1	70884122	70884161	18	40	2.222222	no	no	yes	no	no	no	no	no	no	yes
182201016	chr1	70914067	70914120	23	54	2.375000	no	no	yes	no	no	no	no	no	no	yes
182201019	chr1	70920146	70920200	26	55	2.115385	no	no	yes	no	no	no	no	yes	no	yes
182201024	chr1	70933369	70933397	12	29	2.500000	no	no	yes	no	no	no	no	no	no	yes

182201027	chr1	70944047	70944104	30	58	1.933333	no	no	yes	no	no	no	no	no	no	yes
182201039	chr1	70989940	70989971	14	32	2.285714	no	no	yes	no	no	no	no	no	no	yes
182201045	chr1	71013215	71013357	77	143	1.857143	no	no	yes	no	no	no	no	no	no	yes
182201047	chr1	71018747	71018804	30	58	2.000000	no	no	yes	no	no	no	no	yes	no	yes
182201051	chr1	71022704	71022759	24	56	2.333333	no	no	yes	no	no	no	no	no	no	yes
182201056	chr1	71026640	71026684	23	45	1.956522	no	no	yes	no	no	no	no	no	no	yes
182201060	chr1	71032252	71032302	24	51	2.125000	no	no	yes	no	no	no	no	no	yes	yes
182201069	chr1	71059733	71059857	36	125	3.486486	no	no	yes	no	no	no	no	yes	no	yes
182201072	chr1	71078729	71078764	16	36	2.250000	no	no	yes	no	no	no	no	no	no	yes
182201076	chr1	71093852	71093893	21	42	2.000000	no	no	yes	no	no	no	no	yes	yes	yes
182201077	chr1	71095490	71095532	21	43	2.095238	no	no	yes	no	no	no	no	no	no	yes
182201078	chr1	71104696	71104758	33	63	1.909091	no	no	yes	no	no	no	no	yes	yes	yes
182201079	chr1	71105710	71105750	21	41	1.952381	no	no	yes	no	no	no	no	no	no	yes
182201080	chr1	71105895	71105943	25	49	1.960000	no	no	yes	no	no	no	no	no	no	yes
182201084	chr1	71118099	71118136	12	38	3.166667	no	no	yes	no	no	no	no	no	no	yes
182201086	chr1	71118386	71118447	21	62	3.000000	no	no	yes	no	no	no	no	no	no	yes
182201096	chr1	71159010	71159059	26	50	1.961538	no	no	yes	no	no	no	no	no	no	yes
182201100	chr1	71165255	71165313	26	59	2.269231	no	no	yes	no	no	no	no	no	no	yes
182201104	chr1	71191475	71191515	20	41	2.050000	no	no	yes	no	no	no	no	no	no	yes
182201110	chr1	71212233	71212272	18	40	2.222222	no	no	yes	no	no	no	no	no	no	yes
182201118	chr1	71236555	71236579	11	25	2.272727	no	no	yes	no	no	no	no	yes	no	yes
182201120	chr1	71237043	71237184	75	142	1.893333	no	yes	yes	no	no	yes	no	no	no	yes
182201130	chr1	71288531	71288565	13	35	2.692308	no	no	yes	no	no	no	no	no	no	yes
182201141	chr1	71327683	71327734	27	52	1.962963	no	no	yes	no	no	no	no	no	no	yes
182201150	chr1	71344644	71344684	20	41	2.100000	no	no	yes	no	no	no	no	yes	no	yes

182201153	chr1	71346555	71346607	22	53	2.454545	no	no	yes	no	no	no	no	no	no	yes
182201163	chr1	71368966	71369007	15	42	2.800000	no	no	yes	no	no	no	no	no	no	yes
182201165	chr1	71383632	71383666	16	35	2.187500	no	no	yes	no	no	no	no	yes	no	yes
182201172	chr1	71395970	71396000	16	31	2.000000	no	yes	yes	no	no	no	no	no	no	yes
182201175	chr1	71418641	71418684	20	44	2.200000	no	no	yes	no	no	no	no	no	no	yes
182201177	chr1	71428629	71428657	14	29	2.071429	no	no	yes	no	no	no	no	no	no	yes
182201182	chr1	71431058	71431099	14	42	3.000000	no	no	yes	no	no	no	no	no	no	yes
182201194	chr1	71464366	71464397	16	32	2.000000	no	no	yes	no	no	yes	no	no	yes	yes
182201195	chr1	71468052	71468090	16	39	2.562500	no	no	yes	no	no	no	no	no	no	yes
182201208	chr1	71500596	71500632	19	37	1.947368	no	no	yes	no	no	no	no	no	no	yes
182201216	chr1	71523148	71523246	51	99	1.941176	no	no	yes	no	no	no	no	no	no	yes
182201218	chr1	71523277	71523325	21	49	2.363636	no	no	yes	no	no	no	no	no	no	yes
182201222	chr1	71535401	71535441	19	41	2.157895	no	no	yes	no	no	no	no	no	no	yes
182201225	chr1	71552521	71552571	18	51	2.833333	no	no	yes	no	no	no	no	no	no	yes
182201226	chr1	71554276	71554323	24	48	2.125000	no	no	yes	no	no	no	no	no	no	yes
182201232	chr1	71568667	71568714	21	48	2.285714	no	no	yes	no	no	no	no	no	no	yes
182201234	chr1	71570930	71571024	47	95	2.021277	yes	yes	yes	no	no	no	no	no	no	yes
182201238	chr1	71583080	71583106	14	27	1.928571	no	no	yes	no	no	no	no	no	no	yes
182201244	chr1	71603117	71603192	38	76	2.000000	no	no	yes	no	no	no	no	yes	no	yes
182201245	chr1	71603269	71603306	19	38	2.000000	no	no	yes	no	no	no	no	yes	no	yes
182201247	chr1	71609108	71609146	19	39	2.105263	no	no	yes	no	no	no	no	no	no	yes
182201250	chr1	71614731	71614776	19	46	2.421053	no	no	yes	no	no	no	no	yes	no	yes
182201254	chr1	71642373	71642410	14	38	2.785714	no	no	yes	no	no	no	no	no	no	yes
182201260	chr1	71647520	71647554	17	35	2.058824	no	no	yes	no	no	no	no	no	no	yes
182201261	chr1	71658790	71658820	9	31	3.400000	no	no	yes	no	no	no	no	no	yes	yes

182201263	chr1	71659159	71659206	26	48	1.923077	no	no	yes	no	no	no	no	no	yes	yes
182201267	chr1	71675039	71675087	13	49	3.600000	no	no	yes	no	no	no	no	no	no	yes
182201285	chr1	71694536	71694562	12	27	2.250000	no	no	yes	no	no	no	no	no	no	yes
182201286	chr1	71695222	71695265	16	44	2.750000	no	no	yes	no	no	no	no	no	no	yes
182201290	chr1	71702591	71702624	17	34	2.000000	no	no	yes	no	no	no	no	no	no	yes
182201305	chr1	71739488	71739536	22	49	2.272727	no	no	yes	no	no	no	no	no	no	yes
182201306	chr1	71740717	71740749	14	33	2.428571	no	no	yes	no	no	no	no	no	yes	yes
182201323	chr1	71791563	71791607	19	45	2.421053	no	no	yes	no	no	no	no	no	no	yes
182201326	chr1	71804013	71804048	13	36	2.769231	no	no	yes	no	no	no	no	no	no	yes
182201330	chr1	71811572	71811596	11	25	2.272727	no	no	yes	no	no	no	no	no	no	yes
182201335	chr1	71829629	71829700	39	72	1.923077	no	no	yes	no	no	no	no	no	yes	yes
182201336	chr1	71830818	71830869	26	52	2.000000	no	no	yes	no	no	no	no	no	no	yes
182201337	chr1	71831154	71831183	15	30	2.000000	no	no	yes	no	no	no	no	no	no	yes
182201338	chr1	71835655	71835689	17	35	2.117647	no	no	yes	no	no	no	no	yes	yes	yes
182201348	chr1	71836887	71836949	33	63	1.969697	no	no	yes	no	no	no	no	no	no	yes
182201349	chr1	71837468	71837510	21	43	2.095238	yes	no	yes	no	no	no	no	no	no	yes
182201350	chr1	71845269	71845340	36	72	2.000000	no	no	yes	no	no	no	no	no	no	yes
182201355	chr1	71852859	71852894	18	36	2.000000	no	no	yes	no	no	no	no	no	no	yes
182201356	chr1	71852890	71852919	16	30	1.937500	no	no	yes	no	no	no	no	no	no	yes
182201360	chr1	71866191	71866234	23	44	1.913043	no	no	yes	no	no	no	no	no	no	yes
182201374	chr1	71897130	71897175	24	46	1.916667	no	no	yes	no	no	no	no	no	no	yes
182201377	chr1	71898678	71898771	47	94	2.000000	no	no	yes	no	no	no	no	no	no	yes
182201379	chr1	71898781	71898875	48	95	2.041667	no	no	yes	no	no	no	no	no	no	yes
182201382	chr1	71899314	71899339	13	26	2.000000	no	no	yes	no	no	no	no	no	no	yes
182201383	chr1	71905301	71905344	21	44	2.142857	no	no	yes	no	no	no	no	no	no	yes

182201384	chr1	71905343	71905413	18	71	4.105263	no	no	yes	no	no	no	no	no	no	yes
182201394	chr1	71927332	71927504	44	173	3.931818	no	no	yes	no	no	no	no	no	no	yes
182201399	chr1	71931148	71931183	18	36	2.000000	no	no	yes	no	no	no	no	no	no	yes
182201400	chr1	71932275	71932310	15	36	2.400000	no	no	yes	no	no	no	no	no	no	yes
182201401	chr1	71932508	71932540	17	33	1.941176	no	no	yes	no	no	no	no	no	no	yes
182201404	chr1	71936156	71936205	26	50	1.961538	no	no	yes	no	no	no	no	no	yes	yes
182201408	chr1	71942980	71943114	58	135	2.327586	no	no	yes	no	no	no	no	no	no	yes
182201412	chr1	71943246	71943309	23	64	2.782609	no	no	yes	no	no	no	no	no	no	yes
182201417	chr1	71973878	71973929	22	52	2.391304	no	no	yes	no	no	no	no	no	no	yes
182201427	chr1	72002942	72002984	20	43	2.200000	no	no	yes	no	no	no	no	no	no	yes
182201430	chr1	72010600	72010640	22	41	1.954545	no	no	yes	no	no	no	no	yes	no	yes
182201438	chr1	72017080	72017124	11	45	4.000000	no	no	yes	no	no	no	no	no	no	yes
182201445	chr1	72027540	72027578	14	39	2.785714	no	no	yes	no	no	no	no	no	no	yes
182201448	chr1	72041662	72041700	20	39	1.950000	no	no	yes	no	no	no	no	no	no	yes
182201453	chr1	72046591	72046621	16	31	1.937500	no	no	yes	no	no	no	no	no	no	yes
182201465	chr1	72085206	72085248	20	43	2.150000	no	no	yes	no	no	no	no	no	no	yes
182201471	chr1	72088567	72088594	12	28	2.333333	no	no	yes	no	no	no	no	no	no	yes
182201479	chr1	72107801	72107831	16	31	1.937500	no	no	yes	no	no	no	no	no	no	yes
182201482	chr1	72112374	72112412	16	39	2.411765	no	no	yes	no	no	no	no	no	no	yes
182201483	chr1	72113436	72113480	21	45	2.142857	no	no	yes	no	no	no	no	no	no	yes
182201484	chr1	72113462	72113517	16	56	3.333333	no	no	yes	no	no	no	no	no	no	yes
182201486	chr1	72114449	72114491	23	43	1.956522	no	no	yes	no	no	no	no	no	no	yes
182201494	chr1	72132523	72132552	15	30	2.066667	no	no	yes	no	no	no	no	no	no	yes
182201495	chr1	72133125	72133163	18	39	2.166667	no	no	yes	no	no	no	no	no	no	yes
182201507	chr1	72146071	72146109	16	39	2.437500	no	no	yes	no	no	no	no	no	no	yes

182201509	chr1	72151317	72151353	18	37	2.055556	no	no	yes	no	no	no	no	no	no	yes
182201511	chr1	72158481	72158550	34	70	2.058824	no	no	yes	no	no	no	no	no	no	yes
182201521	chr1	72182679	72182759	42	81	2.000000	no	no	yes	no	no	no	no	no	no	yes
182201522	chr1	72191412	72191453	22	42	1.954545	no	no	yes	no	no	no	no	yes	no	yes
182201523	chr1	72193650	72193691	20	42	2.200000	no	no	yes	no	no	no	no	no	no	yes
182201530	chr1	72211320	72211348	11	29	2.636364	no	no	yes	no	no	no	no	no	no	yes
182201534	chr1	72225899	72225929	16	31	2.000000	no	no	yes	no	no	no	no	no	no	yes
182201536	chr1	72241436	72241498	31	63	2.064516	no	no	yes	no	no	no	no	yes	no	yes
182201538	chr1	72261447	72261489	17	43	2.529412	no	no	yes	no	no	no	no	no	no	yes
182201539	chr1	72266341	72266387	25	47	1.920000	no	no	yes	no	no	no	no	no	no	yes
182201540	chr1	72266614	72266646	17	33	1.941176	no	no	yes	no	no	no	no	no	no	yes
182201546	chr1	72282867	72282917	21	51	2.476191	yes	no	no	no	no	no	no	yes	yes	no
182201552	chr1	72288460	72288495	16	36	2.250000	no	no	yes	no	no	no	no	no	no	yes
182201569	chr1	72331255	72331307	26	53	2.076923	no	no	yes	no	no	no	no	no	no	yes
182201579	chr1	72374395	72374456	15	62	4.133333	no	no	yes	no	no	no	no	yes	no	yes
182201581	chr1	72377667	72377750	42	84	2.000000	no	no	yes	no	no	no	no	no	no	yes
182201588	chr1	72393227	72393296	35	70	2.000000	no	no	yes	no	no	no	no	no	no	yes
182201590	chr1	72394816	72394862	19	47	2.526316	no	no	yes	no	no	no	no	no	no	yes
182201594	chr1	72405784	72405827	23	44	2.000000	no	no	yes	no	no	no	no	no	no	yes
182201595	chr1	72407154	72407195	22	42	1.909091	no	no	yes	no	no	no	no	no	no	yes
182201598	chr1	72410107	72410143	19	37	1.947368	no	no	yes	no	no	no	no	yes	yes	yes
182201609	chr1	72442614	72442665	23	52	2.260870	no	no	yes	no	no	no	no	no	no	yes
182201617	chr1	72468014	72468048	18	35	1.944444	no	no	yes	no	no	no	no	no	no	yes
182201621	chr1	72476053	72476083	14	31	2.285714	no	no	yes	no	no	no	no	no	no	yes
182201624	chr1	72478377	72478424	25	48	1.920000	no	no	yes	no	no	no	no	no	no	yes

182201625	chr1	72483456	72483495	21	40	1.952381	no	no	yes	no	no	no	no	yes	no	yes
182201640	chr1	72545115	72545150	16	36	2.250000	no	no	yes	no	no	no	no	no	no	yes
182201642	chr1	72551684	72551756	34	73	2.205882	no	no	yes	no	no	no	no	no	no	yes
182201645	chr1	72554141	72554309	58	169	2.868852	no	no	yes	no	no	no	no	no	no	yes
182201651	chr1	72566645	72566672	14	28	2.000000	no	no	yes	no	no	no	no	no	no	yes
182201653	chr1	72568826	72568870	23	45	2.000000	no	no	yes	no	no	no	no	no	no	yes
182201658	chr1	72627416	72627444	15	29	1.933333	no	no	yes	no	no	no	no	no	no	yes
182201665	chr1	72645726	72645763	18	38	2.111111	no	no	yes	no	no	no	no	no	no	yes
182201671	chr1	72652824	72652874	26	51	1.961538	no	no	yes	no	no	no	no	yes	no	yes
182201673	chr1	72654107	72654145	19	39	2.052632	no	no	yes	no	no	no	no	no	no	yes
182201694	chr1	72721220	72721304	30	85	2.900000	no	no	yes	no	no	no	no	no	no	yes
182201705	chr1	72734276	72734328	22	53	2.409091	no	no	yes	no	no	no	no	no	yes	yes
182201706	chr1	72735245	72735283	20	39	1.950000	no	no	yes	no	no	no	no	no	no	yes
182201708	chr1	72737066	72737129	25	64	2.600000	no	no	yes	no	no	no	no	no	no	yes
182201710	chr1	72744540	72744601	31	62	2.000000	no	no	yes	no	no	no	no	yes	no	yes
182201716	chr1	72778451	72778492	21	42	2.047619	no	no	yes	no	no	no	no	no	yes	yes
182201717	chr1	72783502	72783531	15	30	2.000000	no	no	yes	no	no	yes	no	no	no	yes
182201719	chr1	72788910	72788934	13	25	1.923077	no	no	yes	no	no	no	no	no	no	yes
182201720	chr1	72791453	72791482	16	30	1.937500	no	no	yes	no	no	yes	no	no	no	yes
182201721	chr1	72793020	72793044	7	25	3.571429	yes	no	yes	no	no	no	no	no	no	yes
182201733	chr1	72843486	72843604	18	119	6.833333	no	no	yes	no	no	no	no	no	no	yes
182201746	chr1	72876443	72876475	17	33	1.941176	no	no	yes	no	no	no	no	no	no	yes
182201758	chr1	72896826	72896884	29	59	2.068965	no	no	yes	no	no	no	no	yes	no	yes
182201761	chr1	72906810	72906846	19	37	1.947368	no	no	no	no	no	no	no	no	no	no
182201765	chr1	72924680	72924723	13	44	3.285714	no	no	no	no	no	no	no	yes	yes	no

182201770	chr1	72944570	72944621	15	52	3.666667	no	no	no	no	no	no	no	no	no	no
182201787	chr1	72971817	72971876	30	60	2.000000	no	no	no	no	no	no	no	no	no	no
182201800	chr1	73008994	73009049	24	56	2.320000	no	no	no	no	no	no	no	no	no	no
182201807	chr1	73032957	73033055	47	99	2.106383	no	no	no	no	no	no	no	no	yes	no
182201808	chr1	73035690	73035733	24	44	1.958333	no	no	no	no	no	no	no	no	no	no
182201809	chr1	73035945	73035997	26	53	2.115385	no	no	no	no	no	no	no	no	no	no
182201812	chr1	73042431	73042465	18	35	1.944444	no	no	no	no	no	no	no	no	no	no
182201814	chr1	73043749	73043786	9	38	4.100000	no	no	no	no	no	no	no	no	no	no
182201837	chr1	73123738	73123796	20	59	2.950000	no	no	no	no	no	no	no	no	no	no
182201841	chr1	73137048	73137134	46	87	1.913043	no	no	no	no	no	no	no	no	no	no
182201852	chr1	73182392	73182435	10	44	4.700000	no	no	no	no	no	no	no	no	no	no
182201858	chr1	73201280	73201532	32	253	7.710526	no	no	no	no	no	no	no	no	no	no
182201867	chr1	73220599	73220645	18	47	2.611111	no	no	no	no	no	no	no	no	no	no
182201868	chr1	73224644	73224681	17	38	2.235294	no	no	no	no	no	no	no	no	no	no
182201875	chr1	73249849	73249951	38	103	2.763158	no	no	no	no	no	no	no	no	no	no
182201879	chr1	73253791	73253856	34	66	2.000000	no	no	no	no	no	no	no	no	no	no
182201881	chr1	73253868	73253911	16	44	2.750000	no	no	no	no	no	no	no	no	no	no
182201888	chr1	73257892	73257940	22	49	2.272727	no	no	no	no	no	no	no	no	no	no
182201891	chr1	73257935	73258003	34	69	2.029412	no	no	no	no	no	no	no	no	no	no
182201892	chr1	73259995	73260029	15	35	2.375000	no	no	no	no	no	no	no	no	no	no
182201896	chr1	73260099	73260154	18	56	3.166667	no	no	no	no	no	no	no	no	no	no
182201908	chr1	73297238	73297277	22	40	1.909091	no	no	no	no	no	no	no	no	no	no
182201914	chr1	73305855	73305891	19	37	1.947368	yes	yes	no	no	no	no	no	no	no	yes
182201915	chr1	73305916	73305961	21	46	2.190476	yes	yes	no	no	no	no	no	no	no	yes
182201916	chr1	73305960	73306045	44	86	1.977273	yes	yes	no	no	no	no	no	no	no	yes

182201917	chr1	73306603	73306645	19	43	2.315789	yes	no	yes	no	no	no	no	no	no	yes
182201923	chr1	73318485	73318510	12	26	2.166667	no	no	yes	no	no	no	no	no	no	yes
182201937	chr1	73350935	73351150	106	216	2.037736	no	no	yes	no	no	no	no	no	no	yes
182201938	chr1	73352572	73352622	24	51	2.125000	no	no	yes	no	no	no	no	no	no	yes
182201944	chr1	73364352	73364387	12	36	3.083333	no	no	no	no	no	no	no	yes	no	no
182201948	chr1	73364450	73364504	27	55	2.074074	no	no	no	no	no	no	no	yes	no	no
182201957	chr1	73372223	73372388	70	166	2.408451	no	no	no	no	no	no	no	no	no	no
182201959	chr1	73388474	73388553	42	80	2.000000	no	no	no	no	no	no	no	no	no	no
182201960	chr1	73389189	73389220	15	32	2.133333	no	no	no	no	no	no	no	no	no	no
182201967	chr1	73390556	73390674	47	119	2.571429	no	no	no	no	no	no	no	no	no	no
182201971	chr1	73415617	73415645	13	29	2.230769	no	no	no	no	no	no	no	no	no	no
182201973	chr1	73418988	73419060	33	73	2.272727	no	no	no	no	no	no	no	no	no	no
182201980	chr1	73434508	73434567	27	60	2.222222	no	no	no	no	no	no	no	no	no	no
182201981	chr1	73436291	73436335	22	45	2.045455	no	no	no	no	no	no	no	no	no	no
182201982	chr1	73439355	73439392	7	38	5.250000	no	no	no	no	no	no	no	no	no	no
182201986	chr1	73441796	73441868	30	73	2.433333	no	no	no	no	no	no	no	no	no	no
182201996	chr1	73456045	73456082	18	38	2.111111	no	no	no	no	no	no	no	no	no	no
182201997	chr1	73456576	73456698	61	123	2.016393	no	no	no	no	no	no	no	no	no	no
182202004	chr1	73485889	73485927	20	39	2.000000	no	no	no	no	no	no	no	no	no	no
182202005	chr1	73492808	73492844	18	37	2.111111	no	no	no	no	no	no	no	yes	no	no
182202010	chr1	73521690	73521802	58	113	1.948276	no	no	no	no	no	no	no	no	no	no
182202024	chr1	73573275	73573301	14	27	1.928571	no	no	no	no	no	no	no	yes	yes	no
182202033	chr1	73616770	73616827	30	58	1.933333	no	no	no	no	no	no	no	no	no	no
182202037	chr1	73641201	73641226	12	26	2.166667	no	no	yes	no	no	no	no	no	no	yes
182202038	chr1	73655002	73655026	13	25	1.923077	no	no	yes	no	no	no	no	no	no	yes

182202044	chr1	73675021	73675058	20	38	1.900000	no	no	yes	no	no	no	no	yes	yes	yes
182202046	chr1	73677441	73677483	16	43	2.750000	no	no	yes	no	no	no	no	no	no	yes
182202047	chr1	73679417	73679459	20	43	2.150000	no	no	yes	no	no	no	no	no	no	yes
182202063	chr1	73742891	73742934	19	44	2.350000	no	no	no	no	no	no	no	no	no	no
182202069	chr1	73754608	73754661	14	54	3.857143	no	no	no	no	no	no	no	no	no	no
182202074	chr1	73765987	73766020	10	34	3.400000	no	no	no	no	no	no	no	no	no	no
182202075	chr1	73767359	73767392	17	34	2.058824	no	no	no	no	no	no	no	no	no	no
182202080	chr1	73776472	73776514	21	43	2.047619	no	no	no	no	no	no	no	no	no	no
182202084	chr1	73776867	73776903	18	37	2.055556	no	no	no	no	no	no	no	no	no	no
182202086	chr1	73801467	73801501	11	35	3.083333	no	no	yes	no	no	no	no	no	no	yes
182202087	chr1	73808219	73808271	27	53	1.962963	no	no	yes	no	no	no	no	no	no	yes
182202102	chr1	73828866	73828918	28	53	1.964286	no	no	yes	no	no	no	no	no	no	yes
182202105	chr1	73835254	73835312	17	59	3.529412	no	no	yes	no	no	no	no	no	no	yes
182202109	chr1	73844266	73844298	15	33	2.200000	no	no	yes	no	no	no	no	no	no	yes
182202111	chr1	73845455	73845496	21	42	2.095238	no	no	yes	no	no	no	no	no	no	yes
182202118	chr1	73879281	73879330	25	50	2.040000	no	no	yes	no	no	no	no	no	no	yes
182202120	chr1	73882627	73882736	41	110	2.780488	yes	no	yes	no	no	no	no	no	no	yes
182202122	chr1	73890655	73890775	58	121	2.086207	no	no	yes	no	no	no	no	no	no	yes
182202126	chr1	73898123	73898180	26	58	2.230769	no	no	yes	no	no	no	no	no	no	yes
182202129	chr1	73917440	73917659	30	220	7.333333	no	no	no	no	no	no	no	no	no	no
182202131	chr1	73917767	73917811	14	45	3.285714	no	no	no	no	no	no	no	no	no	no
182202136	chr1	73941955	73942036	43	82	1.930233	no	no	no	no	no	no	no	no	no	no
182202145	chr1	73963534	73963675	70	142	2.028571	no	no	no	no	no	no	no	no	no	no
182202149	chr1	73971733	73972123	21	391	18.959999	no	no	no	no	no	no	no	no	no	no
182202152	chr1	73976148	73976208	29	61	2.137931	no	no	no	no	no	no	no	no	no	no

182202170	chr1	74032168	74032215	25	48	1.960000	no	no	yes	no	no	no	no	no	no	yes
182202179	chr1	74054759	74054821	22	63	2.863636	no	no	yes	no	no	no	no	no	no	yes
182202191	chr1	74055131	74055178	20	48	2.400000	no	no	yes	no	no	no	no	no	no	yes
182202202	chr1	74068597	74068636	21	40	1.952381	no	no	yes	no	no	no	no	no	no	yes
182202205	chr1	74074018	74074058	18	41	2.315789	no	no	yes	no	no	no	no	no	no	yes
182202206	chr1	74080002	74080030	15	29	1.933333	no	no	yes	no	no	no	no	no	no	yes
182202211	chr1	74096422	74096475	26	54	2.076923	no	no	yes	no	no	no	no	no	no	yes
182202213	chr1	74106755	74106818	32	64	2.000000	no	no	yes	no	no	no	no	yes	no	yes
182202214	chr1	74107521	74107573	19	53	2.761905	no	no	yes	no	no	no	no	no	no	yes
182202217	chr1	74108094	74108118	13	25	1.923077	no	no	yes	no	no	no	no	yes	no	yes
182202218	chr1	74108198	74108233	13	36	2.769231	no	no	yes	no	no	no	no	yes	no	yes
182202219	chr1	74117354	74117396	22	43	1.954545	no	no	yes	no	no	no	no	no	no	yes
182202220	chr1	74118516	74118547	16	32	2.000000	no	no	yes	no	no	no	no	no	no	yes
182202226	chr1	74139101	74139169	26	69	2.653846	no	no	yes	no	no	no	no	no	no	yes
182202231	chr1	74157455	74157496	22	42	2.000000	no	no	yes	no	no	no	no	no	no	yes
182202237	chr1	74177660	74177686	14	27	1.928571	no	no	yes	no	no	no	no	no	no	yes
182202240	chr1	74186020	74186058	20	39	2.000000	no	no	yes	no	no	no	no	no	no	yes
182202242	chr1	74196160	74196242	45	83	1.933333	no	no	yes	no	no	no	no	no	no	yes
182202244	chr1	74209457	74209497	19	41	2.210526	no	no	yes	no	no	no	no	yes	no	yes
182202246	chr1	74216439	74216477	18	39	2.222222	no	no	yes	no	no	no	no	no	no	yes
182202249	chr1	74220520	74220574	29	55	1.965517	no	no	yes	no	no	no	no	no	no	yes
182202252	chr1	74229968	74230023	29	56	1.931034	no	no	yes	no	no	no	no	no	no	yes
182202253	chr1	74233293	74233324	12	32	2.666667	no	no	yes	no	no	no	no	no	no	yes
182202265	chr1	74275790	74275847	28	58	2.071429	no	no	yes	no	no	no	no	no	yes	yes
182202269	chr1	74311144	74311168	12	25	2.083333	no	no	yes	no	no	no	no	no	no	yes

182202271	chr1	74314334	74314366	16	33	2.125000	no	no	yes	no	no	no	no	yes	no	yes
182202274	chr1	74319485	74319526	16	42	2.625000	no	no	yes	no	no	no	no	no	no	yes
182202280	chr1	74327673	74327708	17	36	2.176471	no	no	yes	no	no	no	no	no	no	yes
182202281	chr1	74327779	74327842	25	64	2.576923	no	no	yes	no	no	no	no	no	no	yes
182202282	chr1	74345026	74345069	14	44	3.285714	no	no	yes	no	no	no	no	no	no	yes
182202288	chr1	74368699	74368793	32	95	2.968750	yes	no	yes	no	no	yes	no	yes	yes	yes
182202289	chr1	74372689	74372722	16	34	2.125000	no	yes	yes	no	no	no	no	no	no	yes
182202294	chr1	74397197	74397250	28	54	1.928571	no	no	yes	no	no	no	no	yes	no	yes
182202295	chr1	74415630	74415692	33	63	1.939394	no	no	yes	no	no	no	no	no	no	yes
182202300	chr1	74436216	74436265	24	50	2.083333	no	no	yes	no	no	no	no	yes	yes	yes
182202301	chr1	74438036	74438071	18	36	2.000000	yes	no	yes	no	no	no	no	no	no	yes
182202307	chr1	74473466	74473511	16	46	2.875000	no	no	yes	no	no	no	no	no	no	yes
182202309	chr1	74478437	74478469	14	33	2.357143	no	no	yes	no	no	no	no	no	no	yes
182202310	chr1	74484276	74484309	18	34	1.944444	no	no	yes	no	no	no	no	no	no	yes
182202315	chr1	74490471	74490508	19	38	2.052632	no	no	yes	no	no	no	no	no	no	yes
182202324	chr1	74512374	74512409	17	36	2.117647	no	no	yes	no	no	no	no	yes	no	yes
182202327	chr1	74531454	74531486	17	33	1.941176	no	no	yes	no	no	no	no	no	yes	yes
182202331	chr1	74547142	74547185	12	44	3.615385	no	no	no	no	no	no	no	no	no	no
182202353	chr1	74612281	74612320	18	40	2.277778	no	no	yes	no	no	yes	no	yes	no	yes
182202359	chr1	74650402	74650453	27	52	1.925926	no	no	yes	no	no	no	no	no	no	yes
182202361	chr1	74650888	74650940	26	53	2.038461	no	no	yes	no	no	no	no	no	no	yes
182202366	chr1	74657209	74657240	13	32	2.461539	no	no	yes	no	no	no	no	no	no	yes
182202369	chr1	74660612	74660720	58	109	1.931034	no	no	yes	no	no	no	no	no	no	yes
182202372	chr1	74691446	74691475	15	30	2.000000	no	no	no	no	no	no	no	no	no	no
182202375	chr1	74716266	74716301	13	36	2.785714	no	no	yes	no	no	no	no	no	no	yes

182202377	chr1	74731117	74731141	12	25	2.083333	no	no	yes	no	no	no	no	yes	no	yes
182202381	chr1	74732044	74732518	150	475	3.173333	yes	no	yes	no	no	no	no	yes	yes	yes
182202382	chr1	74733044	74733098	23	55	2.391304	yes	no	no	no	yes	no	no	yes	yes	yes
182202398	chr1	74747685	74747724	18	40	2.222222	yes	no	yes	no	no	no	no	no	no	yes
182202403	chr1	74747777	74747860	38	84	2.210526	yes	no	yes	no	no	no	no	yes	no	yes
182202409	chr1	74758378	74758723	38	346	9.105263	no	no	yes	no	no	no	no	yes	no	yes
182202418	chr1	74792323	74792368	24	46	1.958333	no	no	no	no	no	no	no	no	no	no
182202420	chr1	74797917	74797954	19	38	2.000000	no	no	no	no	no	no	no	no	no	no
182202423	chr1	74810314	74810350	19	37	1.947368	no	no	no	no	no	no	no	no	no	no
182202433	chr1	74810600	74810669	37	70	1.945946	no	no	no	no	no	no	no	no	no	no
182202443	chr1	74832101	74832142	23	42	1.913043	no	no	no	no	no	no	no	no	no	no
182202454	chr1	74854267	74854299	17	33	1.941176	no	no	no	no	no	no	no	no	no	no
182202474	chr1	74935660	74935706	23	47	2.043478	no	no	no	no	no	no	no	yes	yes	no
182202482	chr1	74959627	74959662	12	36	3.083333	no	no	no	no	no	no	no	no	no	no
182202484	chr1	74971301	74971333	14	33	2.357143	no	no	no	no	no	no	no	no	no	no
182202486	chr1	74973070	74973099	13	30	2.307692	no	no	no	no	no	no	no	no	no	no
182202488	chr1	74983235	74983301	32	67	2.093750	no	no	no	no	no	no	no	no	no	no
182202496	chr1	75013574	75013604	15	31	2.066667	no	no	no	no	no	no	no	no	no	no
182202516	chr1	75073120	75073169	25	50	2.000000	no	no	no	no	no	no	no	yes	no	no
182202517	chr1	75081135	75081170	16	36	2.250000	no	no	no	no	no	no	no	no	no	no
182202519	chr1	75083733	75083796	22	64	2.909091	no	no	no	no	no	no	no	yes	yes	no
182202531	chr1	75119236	75119263	14	28	2.000000	no	no	no	no	no	no	no	no	no	no
182202532	chr1	75121929	75122041	60	113	1.916667	no	no	no	no	no	yes	no	no	no	no
182202535	chr1	75125754	75125793	16	40	2.500000	no	no	no	no	no	no	no	no	no	no
182202538	chr1	75147244	75147275	16	32	2.000000	no	no	yes	no	no	no	no	no	no	yes

182202548	chr1	75189557	75189589	17	33	1.941176	no	no	no	no	no	no	no	yes	no	no
182202550	chr1	75201166	75201202	16	37	2.312500	no	no	no	no	no	yes	no	yes	no	no
182202552	chr1	75209205	75209242	20	38	1.950000	no	no	yes	no	no	no	no	yes	no	yes
182202561	chr1	75223045	75223084	21	40	1.904762	no	no	yes	no	no	no	no	no	no	yes
182202570	chr1	75276067	75276092	13	26	2.000000	no	no	yes	no	no	no	no	no	yes	yes
182202574	chr1	75280131	75280508	34	378	10.972973	no	no	yes	no	no	no	no	no	no	yes
182202587	chr1	75323781	75323808	13	28	2.153846	no	no	yes	no	no	no	no	no	no	yes
182202589	chr1	75326642	75326676	18	35	1.944444	no	no	yes	no	no	no	no	yes	no	yes
182202593	chr1	75330145	75330226	40	82	2.100000	no	no	yes	no	no	no	no	no	no	yes
182202596	chr1	75341479	75341534	15	56	3.750000	no	no	yes	no	no	no	no	yes	no	yes
182202606	chr1	75371538	75371580	15	43	2.812500	no	no	yes	no	no	no	no	no	no	yes
182202645	chr1	75530924	75530977	25	54	2.160000	no	no	yes	no	no	no	no	no	no	yes
182202654	chr1	75548348	75548399	26	52	2.000000	no	no	yes	no	no	no	no	no	no	yes
182202662	chr1	75564577	75564636	30	60	2.000000	no	no	yes	no	no	no	no	no	no	yes
182202671	chr1	75584558	75584593	11	36	3.272727	no	no	yes	no	no	no	no	no	no	yes
182202673	chr1	75588215	75588276	20	62	3.150000	no	no	yes	no	no	no	no	yes	no	yes
182202679	chr1	75605240	75605277	19	38	2.000000	no	no	yes	no	no	no	no	no	no	yes
182202685	chr1	75616114	75616158	18	45	2.500000	no	no	no	no	no	no	yes	yes	yes	no
182202689	chr1	75654815	75654865	24	51	2.166667	no	no	yes	no	no	no	no	yes	no	yes
182202699	chr1	75669685	75669720	18	36	2.000000	no	no	yes	no	no	no	no	no	no	yes
182202706	chr1	75695681	75695755	28	75	2.714286	no	no	yes	no	no	no	no	no	no	yes
182202721	chr1	75735525	75735557	17	33	2.000000	no	no	yes	no	no	no	no	no	no	yes
182202737	chr1	75765576	75765733	81	158	1.962963	no	no	yes	no	no	no	no	yes	no	yes
182202746	chr1	75790567	75790595	15	29	1.933333	yes	no	yes	no	no	yes	no	yes	yes	yes
182202758	chr1	75814690	75814744	18	55	3.166667	no	no	yes	no	no	no	no	no	no	yes

182202774	chr1	75884950	75885072	42	123	2.928571	no	no	yes	no	no	no	no	no	no	yes
182202780	chr1	75885204	75885274	35	71	2.057143	no	no	yes	no	no	no	no	no	no	yes
182202786	chr1	75886594	75886770	82	177	2.268293	no	no	yes	no	no	no	no	no	no	yes
182202790	chr1	75899790	75899818	12	29	2.416667	no	no	yes	no	no	no	no	no	no	yes
182202809	chr1	75930224	75930280	26	57	2.192308	yes	no	yes	no	no	no	no	no	no	yes
182202822	chr1	75962937	75962970	17	34	2.000000	no	no	yes	no	no	no	no	no	no	yes
182202823	chr1	75963205	75963250	18	46	2.555556	no	no	yes	no	no	no	no	no	no	yes
182202824	chr1	75967718	75967759	17	42	2.529412	no	no	yes	no	no	no	no	no	no	yes
182202826	chr1	75986740	75986810	33	71	2.181818	no	no	yes	no	no	no	no	yes	yes	yes
182202831	chr1	75996547	75996581	12	35	3.000000	no	no	yes	no	no	no	no	yes	no	yes
182202836	chr1	76004874	76004916	15	43	2.933333	no	no	yes	no	no	no	no	no	no	yes
182202838	chr1	76009628	76009718	44	91	2.068182	no	no	yes	no	no	no	no	yes	no	yes
182202845	chr1	76046342	76046392	23	51	2.260870	no	no	yes	no	no	no	no	no	no	yes
182202847	chr1	76047727	76047768	18	42	2.333333	no	no	yes	no	no	no	no	no	no	yes
182202849	chr1	76048945	76048996	24	52	2.166667	no	no	yes	no	no	no	no	no	no	yes
182202855	chr1	76066066	76066110	12	45	3.833333	no	yes	no	no	no	no	no	no	no	yes
182202861	chr1	76095802	76095830	15	29	2.000000	no	no	yes	no	no	no	no	yes	no	yes
182202862	chr1	76096549	76096611	33	63	2.000000	no	no	yes	no	no	no	no	no	no	yes
182202878	chr1	76119793	76119833	13	41	3.142857	no	no	yes	no	no	no	no	no	no	yes
182202882	chr1	76139356	76139422	16	67	4.187500	no	no	yes	no	no	no	no	no	no	yes
182202887	chr1	76150529	76150560	17	32	1.941176	no	no	yes	no	no	no	no	no	no	yes
182202888	chr1	76151203	76151254	26	52	2.000000	no	no	yes	no	no	no	no	no	no	yes
182202913	chr1	76223692	76223733	22	42	1.909091	no	no	yes	no	no	no	no	no	no	yes
182202917	chr1	76258390	76258422	16	33	2.062500	no	no	yes	no	no	no	no	yes	no	yes
182202918	chr1	76259835	76259876	22	42	1.954545	no	no	yes	no	no	no	no	no	no	yes

182202922	chr1	76270120	76270160	20	41	2.100000	no	no	yes	no	no	no	no	yes	no	yes
182202924	chr1	76275783	76275825	20	43	2.150000	no	no	yes	no	no	no	no	no	no	yes
182202929	chr1	76279971	76279995	11	25	2.272727	no	no	yes	no	no	no	no	no	no	yes
182202932	chr1	76285766	76285805	21	40	1.904762	no	no	yes	no	no	no	no	no	yes	yes
182202933	chr1	76288271	76288310	20	40	2.050000	no	no	yes	no	no	no	no	no	yes	yes
182202934	chr1	76292267	76292323	24	57	2.375000	no	no	yes	no	no	no	no	yes	no	yes
182202935	chr1	76303840	76303865	12	26	2.166667	no	no	yes	no	no	no	no	no	no	yes
182202936	chr1	76303885	76303942	28	58	2.071429	no	no	yes	no	no	no	no	no	no	yes
182202937	chr1	76304146	76304179	16	34	2.125000	no	no	yes	no	no	no	no	no	no	yes
182202939	chr1	76320914	76320957	12	44	3.916667	no	no	yes	no	no	no	no	no	no	yes
182202941	chr1	76325725	76325759	12	35	2.916667	no	no	yes	no	no	no	no	no	no	yes
182202944	chr1	76333540	76333583	21	44	2.095238	no	no	yes	no	no	no	no	no	no	yes
182202946	chr1	76340725	76340801	36	77	2.166667	no	no	yes	no	no	no	no	no	yes	yes
182202948	chr1	76346462	76346540	39	79	2.076923	no	no	yes	no	no	no	no	yes	no	yes
182202954	chr1	76370676	76370721	23	46	2.000000	no	no	yes	no	no	no	no	no	no	yes
182202960	chr1	76388964	76389006	21	43	2.047619	no	no	yes	no	no	no	no	no	no	yes
182202969	chr1	76417208	76417244	18	37	2.055556	no	no	yes	no	no	no	no	no	no	yes
182202970	chr1	76419415	76419453	19	39	2.052632	no	no	yes	no	no	no	no	no	no	yes
182202972	chr1	76419903	76419961	30	59	1.966667	no	no	yes	no	no	no	no	no	no	yes
182202976	chr1	76436552	76436591	19	40	2.157895	no	no	yes	no	no	no	no	no	no	yes
182202982	chr1	76468017	76468054	18	38	2.166667	no	no	yes	no	no	no	no	no	no	yes
182202983	chr1	76472298	76472326	15	29	1.933333	no	no	yes	no	no	no	no	no	no	yes
182202986	chr1	76488856	76488895	21	40	1.904762	no	no	yes	no	no	no	no	no	no	yes
182202999	chr1	76494469	76494769	96	301	3.112245	no	no	yes	no	no	no	no	yes	no	yes
182203001	chr1	76513677	76513711	8	35	4.500000	no	no	yes	no	no	no	no	no	no	yes

182203003	chr1	76517524	76517552	15	29	1.933333	no	no	yes	no	no	no	no	no	no	yes
182203012	chr1	76565403	76565461	31	59	1.967742	no	no	yes	no	no	no	no	no	yes	yes
182203015	chr1	76576931	76576969	12	39	3.250000	no	yes	yes	no	no	no	no	no	no	yes
182203017	chr1	76583578	76583657	39	80	2.051282	no	no	yes	no	no	no	no	yes	no	yes
182203026	chr1	76607909	76607950	18	42	2.444444	no	no	yes	no	no	no	no	yes	yes	yes
182203028	chr1	76611223	76611266	22	44	2.045455	no	no	yes	no	no	no	no	no	no	yes
182203030	chr1	76623626	76623674	24	49	2.041667	no	no	yes	no	no	no	no	no	no	yes
182203031	chr1	76627390	76627426	13	37	2.923077	no	no	yes	no	no	no	no	yes	no	yes
182203036	chr1	76641834	76641876	19	43	2.263158	no	no	no	no	no	no	no	no	no	no
182203039	chr1	76646406	76646650	122	245	2.008197	no	no	no	no	no	no	no	no	no	no
182203045	chr1	76666353	76666492	45	140	3.108696	no	no	no	no	no	no	no	yes	no	no
182203053	chr1	76691170	76691293	66	124	1.878788	no	no	no	no	no	no	no	yes	no	no
182203055	chr1	76706244	76706301	28	58	2.071429	no	no	no	no	no	no	no	no	no	no
182203059	chr1	76717747	76717787	18	41	2.277778	no	no	no	no	no	no	no	no	no	no
182203060	chr1	76725233	76725267	14	35	2.500000	no	no	no	no	no	no	no	yes	no	no
182203061	chr1	76727009	76727050	22	42	1.954545	no	no	no	no	no	no	no	no	no	no
182203066	chr1	76758223	76758256	11	34	3.090909	no	no	yes	no	no	no	no	no	no	yes
182203069	chr1	76763429	76763462	14	34	2.400000	no	no	yes	no	no	no	no	no	yes	yes
182203070	chr1	76773786	76773822	17	37	2.235294	no	no	yes	no	no	no	no	no	no	yes
182203073	chr1	76780413	76780468	18	56	3.166667	no	no	no	no	no	no	no	no	no	no
182203079	chr1	76812442	76812472	14	31	2.214286	no	no	no	no	no	no	no	no	no	no
182203080	chr1	76816387	76816436	26	50	1.923077	no	no	no	no	no	no	no	no	no	no
182203081	chr1	76825921	76825971	21	51	2.428571	no	no	no	no	no	no	no	no	no	no
182203085	chr1	76839719	76839754	18	36	2.000000	no	no	no	no	no	no	no	no	no	no
182203086	chr1	76845768	76845813	22	46	2.136364	no	no	no	no	no	no	no	no	no	no

182203090	chr1	76849456	76849511	24	56	2.333333	no	no	no	no	no	no	no	no	no	no
182203092	chr1	76849540	76849602	31	63	2.096774	no	no	no	no	no	no	no	no	no	no
182203098	chr1	76849863	76849911	11	49	4.333333	no	no	no	no	no	no	no	yes	no	no
182203104	chr1	76850213	76850273	27	61	2.296296	no	no	no	no	no	no	no	yes	no	no
182203106	chr1	76852468	76852550	30	83	2.766667	no	no	no	no	no	no	no	no	no	no
182203110	chr1	76852573	76852617	20	45	2.250000	no	no	no	no	no	no	no	no	no	no
182203111	chr1	76852650	76852684	18	35	1.944444	no	no	no	no	no	no	no	no	no	no
182203112	chr1	76862062	76862099	20	38	1.900000	no	no	no	no	no	no	no	no	no	no
182203116	chr1	76882883	76882955	38	73	1.921053	no	no	yes	no	no	no	no	no	no	yes
182203143	chr1	76991202	76991238	17	37	2.176471	no	no	yes	no	no	no	no	no	no	yes
182203146	chr1	77014171	77014198	13	28	2.153846	no	no	yes	no	no	no	no	yes	yes	yes
182203150	chr1	77016085	77016336	24	252	10.791667	no	no	yes	no	no	no	no	yes	yes	yes
182203151	chr1	77021163	77021224	28	62	2.250000	no	no	yes	no	no	no	no	yes	no	yes
182203153	chr1	77034707	77034740	18	34	1.944444	no	no	yes	no	no	no	no	no	no	yes
182203155	chr1	77062063	77062137	38	75	2.000000	no	no	yes	no	no	no	no	no	no	yes
182203160	chr1	77096882	77096940	27	59	2.222222	no	no	yes	no	no	no	no	yes	no	yes
182203164	chr1	77119873	77119910	12	38	3.250000	no	no	yes	no	no	no	no	no	no	yes
182203171	chr1	77135377	77135446	36	70	2.027778	no	no	yes	no	no	no	no	no	no	yes
182203173	chr1	77144896	77144930	17	35	2.058824	no	no	yes	no	no	no	no	no	no	yes
182203181	chr1	77156578	77156608	16	31	1.937500	no	no	yes	no	no	no	no	yes	no	yes
182203182	chr1	77169525	77169698	85	174	2.047059	yes	no	yes	no	no	no	no	no	no	yes
182203189	chr1	77192787	77192829	17	43	2.588235	no	no	yes	no	no	no	no	no	no	yes
182203192	chr1	77200462	77200636	95	175	1.873684	no	no	yes	no	no	no	no	yes	no	yes
182203207	chr1	77264006	77264047	22	42	2.000000	no	no	no	no	no	no	no	yes	no	no
182203209	chr1	77271123	77271151	14	29	2.071429	no	no	no	no	no	no	no	no	no	no

182203211	chr1	77289683	77289707	13	25	1.923077	no	no	yes	no	no	no	no	no	no	yes
182203213	chr1	77312778	77312809	13	32	2.538461	no	no	yes	no	no	no	no	yes	no	yes
182203215	chr1	77318606	77318634	14	29	2.071429	no	no	yes	no	no	no	no	no	yes	yes
182203217	chr1	77322906	77322948	21	43	2.047619	no	no	yes	no	no	no	no	no	yes	yes
182203232	chr1	77359480	77359508	14	29	2.071429	no	no	yes	no	no	no	no	no	no	yes
182203246	chr1	77404639	77404671	17	33	2.000000	no	no	yes	no	no	no	no	no	no	yes
182203259	chr1	77444195	77444739	33	545	16.941177	no	no	yes	no	no	no	no	no	no	yes
182203284	chr1	77506387	77506419	17	33	1.941176	no	no	yes	no	no	no	no	no	yes	yes
182203292	chr1	77529730	77529864	15	135	9.000000	no	no	yes	no	no	no	no	no	no	yes
182203302	chr1	77545228	77545269	20	42	2.200000	no	no	yes	no	no	no	no	yes	no	yes
182203310	chr1	77574092	77574196	22	105	4.772727	no	no	yes	no	no	no	no	yes	no	yes
182203313	chr1	77579939	77579970	10	32	3.400000	no	no	yes	no	yes	no	no	yes	no	yes
182203327	chr1	77636775	77636846	37	72	1.972973	no	no	yes	no	no	no	no	no	no	yes
182203335	chr1	77674093	77674131	21	39	1.904762	no	no	yes	no	no	no	no	yes	yes	yes
182203338	chr1	77683092	77683126	9	35	3.888889	yes	no	yes	no	no	no	yes	yes	yes	yes
182203349	chr1	77702901	77702947	11	47	4.363636	no	no	yes	no	no	no	no	no	no	yes
182203354	chr1	77709733	77709782	20	50	2.500000	no	no	yes	no	no	no	no	yes	no	yes
182203362	chr1	77737670	77737716	22	47	2.136364	no	no	yes	no	no	no	no	no	no	yes
182203363	chr1	77741817	77741865	26	49	1.961538	no	no	yes	no	no	no	no	no	no	yes
182203367	chr1	77753809	77753864	25	56	2.240000	no	no	yes	no	no	no	no	no	no	yes
182203368	chr1	77756635	77756674	21	40	1.904762	no	no	yes	no	no	no	no	no	no	yes
182203369	chr1	77760734	77760767	16	34	2.125000	yes	no	no	no	no	no	no	no	no	no
182203373	chr1	77762336	77762467	64	132	2.062500	no	no	no	no	no	no	no	yes	no	no
182203390	chr1	77780413	77780441	12	29	2.500000	no	no	yes	no	no	yes	no	yes	yes	yes
182203404	chr1	77817272	77817333	14	62	4.333333	no	no	yes	no	no	no	no	no	no	yes

182203410	chr1	77841456	77841489	14	34	2.428571	no	no	yes	no	no	no	no	no	yes	yes
182203414	chr1	77847469	77847539	30	71	2.366667	no	yes	yes	no	no	no	no	no	no	yes
182203416	chr1	77857693	77857717	11	25	2.272727	no	no	yes	no	no	no	no	yes	yes	yes
182203437	chr1	77881513	77881557	19	45	2.473684	no	no	no	no	yes	no	no	no	no	yes
182203444	chr1	77889916	77889958	22	43	1.954545	yes	no	yes	no	no	no	no	yes	yes	yes
182203466	chr1	77931643	77931692	24	50	2.083333	yes	yes	yes	no	no	no	no	no	no	yes
182203471	chr1	77941230	77941260	15	31	2.066667	yes	no	yes	no	no	no	no	yes	yes	yes
182203475	chr1	77960715	77960753	18	39	2.277778	no	no	yes	no	yes	no	no	yes	no	yes
182203478	chr1	77971404	77971607	104	204	2.000000	no	no	yes	no	no	no	no	yes	yes	yes
182203480	chr1	77978931	77978960	12	30	2.500000	yes	no	no	yes	no	no	no	yes	yes	yes
182203484	chr1	77985124	77985152	13	29	2.230769	no	no	yes	no	no	no	no	no	no	yes
182203488	chr1	77987914	77988015	30	102	3.354839	no	no	yes	no	no	no	no	no	no	yes
182203494	chr1	78002699	78002733	16	35	2.187500	no	no	yes	no	no	no	no	yes	yes	yes
182203495	chr1	78005373	78005414	22	42	1.954545	yes	no	yes	no	no	no	no	yes	yes	yes
182203500	chr1	78020762	78020802	21	41	2.000000	no	no	yes	no	no	no	no	yes	yes	yes
182203509	chr1	78034936	78034991	23	56	2.434783	no	no	yes	no	no	no	no	no	no	yes
182203510	chr1	78038776	78038823	19	48	2.526316	no	no	yes	no	no	no	no	yes	yes	yes
182203524	chr1	78067850	78067980	64	131	2.046875	no	no	yes	no	no	no	no	no	no	yes
182203528	chr1	78071539	78071615	25	77	3.115385	no	no	yes	no	no	no	no	no	no	yes
182203535	chr1	78098350	78098425	19	76	4.000000	no	no	yes	no	no	no	no	no	no	yes
182203545	chr1	78107099	78107139	22	41	1.909091	no	no	yes	no	no	no	no	no	no	yes
182203547	chr1	78115874	78115903	13	30	2.307692	no	no	yes	no	no	no	no	no	no	yes
182203552	chr1	78130784	78130817	16	34	2.125000	no	no	yes	no	no	no	no	yes	no	yes
182203559	chr1	78135450	78135480	12	31	2.666667	no	no	yes	no	no	no	no	no	no	yes
182203565	chr1	78164129	78164158	12	30	2.500000	no	no	no	no	no	no	no	no	no	no

182203586	chr1	78243519	78243562	19	44	2.315789	no	no	yes	no	no	no	no	no	no	yes
182203592	chr1	78262642	78262682	19	41	2.157895	no	no	yes	no	no	no	no	no	no	yes
182203608	chr1	78327285	78327323	14	39	2.687500	no	no	yes	no	no	no	no	no	no	yes
182203613	chr1	78331524	78331580	30	57	1.900000	no	no	yes	no	no	no	no	no	no	yes
182203615	chr1	78337300	78337353	15	54	3.500000	no	no	yes	no	no	no	no	no	no	yes
182203624	chr1	78355157	78355227	27	71	2.666667	no	no	yes	no	no	no	no	yes	no	yes
182203639	chr1	78378716	78378772	24	57	2.458333	no	no	yes	no	no	no	no	no	no	yes
182203640	chr1	78384458	78384498	19	41	2.157895	no	no	yes	no	no	no	no	no	no	yes
182203641	chr1	78386364	78386401	12	38	3.000000	no	no	yes	no	no	no	no	no	no	yes
182203643	chr1	78400273	78400311	15	39	2.600000	no	no	yes	no	no	no	no	no	no	yes
182203648	chr1	78415541	78415887	30	347	11.718750	no	no	yes	no	no	no	no	no	no	yes
182203655	chr1	78438536	78438629	48	94	1.958333	no	no	yes	no	no	no	no	no	no	yes
182203661	chr1	78453546	78453619	23	74	3.166667	no	no	yes	no	no	no	no	yes	no	yes
182203680	chr1	78528785	78528880	49	96	1.979592	no	no	yes	no	no	no	no	no	no	yes
182203684	chr1	78532387	78532553	62	167	2.693548	no	no	yes	no	no	no	no	no	no	yes
182203689	chr1	78572449	78572480	15	32	2.133333	no	no	no	no	no	no	no	no	no	no
182203692	chr1	78583670	78583701	15	32	2.200000	no	no	no	no	no	no	no	no	no	no
182203697	chr1	78596329	78596380	26	52	2.000000	no	no	no	no	no	no	no	no	no	no
182203701	chr1	78604912	78604964	29	53	1.931034	no	no	no	no	no	no	no	yes	no	no
182203704	chr1	78620259	78620452	91	194	2.131868	yes	yes	yes	no	yes	no	no	yes	yes	yes
182203711	chr1	78653123	78653165	20	43	2.150000	no	no	yes	no	no	no	no	no	no	yes
182203716	chr1	78668328	78668397	37	70	1.918919	no	no	no	no	no	no	no	yes	no	no
182203717	chr1	78669042	78669165	62	124	2.000000	no	no	no	no	no	no	no	no	no	no
182203719	chr1	78675165	78675196	8	32	4.250000	no	no	no	no	no	no	no	no	no	no
182203734	chr1	78705176	78705368	97	193	1.989691	no	no	no	no	no	no	no	no	yes	no

182203736	chr1	78707323	78707357	15	35	2.333333	no	no	no	no	no	no	no	no	no	no
182203740	chr1	78711515	78711556	16	42	2.705882	no	no	no	no	no	no	no	no	no	no
182203741	chr1	78716670	78716701	16	32	2.062500	no	no	no	no	no	no	no	no	no	no
182203754	chr1	78752247	78752271	12	25	2.083333	no	no	no	no	no	no	no	no	no	no
182203763	chr1	78798436	78798491	28	56	2.000000	no	no	no	no	no	no	no	no	no	no
182203764	chr1	78801071	78801117	20	47	2.400000	no	no	no	no	no	no	no	yes	no	no
182203767	chr1	78814530	78814568	19	39	2.052632	no	no	no	no	no	no	no	yes	no	no
182203768	chr1	78817481	78817532	27	52	1.962963	no	no	no	no	no	no	no	no	no	no
182203773	chr1	78836567	78836632	24	66	2.750000	no	no	no	no	no	no	no	no	no	no
182203783	chr1	78858886	78858910	11	25	2.272727	no	no	no	no	no	no	no	no	no	no
182203784	chr1	78859746	78859788	21	43	2.047619	no	no	no	no	no	no	no	no	no	no
182203789	chr1	78883887	78883991	32	105	3.281250	no	no	no	no	no	no	no	no	no	no
182203793	chr1	78912159	78912192	16	34	2.125000	no	no	yes	no	no	no	no	yes	yes	yes
182203795	chr1	78917384	78917434	27	51	1.925926	no	no	yes	no	yes	no	no	no	no	yes
182203796	chr1	78917444	78917481	19	38	2.052632	no	no	yes	no	yes	no	no	no	no	yes
182203802	chr1	78948405	78948435	16	31	1.937500	no	no	yes	no	no	no	no	no	no	yes
182203806	chr1	78961533	78961574	20	42	2.100000	no	no	yes	no	no	no	no	no	no	yes
182203808	chr1	78965991	78966029	20	39	1.950000	no	no	yes	no	no	no	no	no	no	yes
182203809	chr1	78973562	78973603	21	42	2.047619	no	no	yes	no	no	no	no	no	no	yes
182203810	chr1	78973599	78973673	30	75	2.516129	no	no	yes	no	no	no	no	no	no	yes
182203818	chr1	79005742	79005792	22	51	2.363636	no	no	yes	no	no	no	no	yes	no	yes
182203844	chr1	79042211	79042239	14	29	2.071429	no	no	yes	no	no	no	no	yes	yes	yes
182203847	chr1	79053311	79053352	20	42	2.150000	no	no	yes	no	no	no	no	yes	no	yes
182203850	chr1	79064408	79064447	21	40	1.904762	no	no	yes	no	no	no	no	no	no	yes
182203852	chr1	79067784	79067847	21	64	3.095238	no	no	yes	no	no	no	no	no	no	yes

182203858	chr1	79079379	79079423	18	45	2.555556	no	no	yes	no	no	no	no	no	no	yes
182203862	chr1	79090409	79090451	17	43	2.588235	no	no	yes	no	no	no	no	no	no	yes
182203875	chr1	79097310	79097337	11	28	2.545455	no	no	yes	no	no	no	no	no	no	yes
182203877	chr1	79102497	79102587	26	91	3.500000	no	no	yes	no	no	no	no	no	no	yes
182203882	chr1	79109680	79109717	20	38	1.900000	no	no	yes	no	no	no	no	yes	yes	yes
182203884	chr1	79129567	79129607	15	41	2.800000	no	no	yes	no	no	no	no	no	no	yes
182203896	chr1	79162952	79163027	40	76	1.900000	no	no	yes	no	no	no	no	no	no	yes
182203899	chr1	79172885	79172917	17	33	1.941176	no	no	yes	no	no	no	no	no	no	yes
182203902	chr1	79189481	79189511	13	31	2.384615	yes	no	yes	no	no	no	no	yes	yes	yes
182203905	chr1	79198832	79198899	34	68	2.000000	no	no	yes	no	no	no	no	yes	no	yes
182203906	chr1	79199188	79199232	19	45	2.368421	no	no	yes	no	no	no	no	yes	no	yes
182203911	chr1	79202354	79202401	23	48	2.086957	no	no	yes	no	no	no	no	no	no	yes
182203915	chr1	79209375	79209411	20	37	1.950000	no	no	yes	no	no	no	no	no	no	yes
182203916	chr1	79210201	79210238	19	38	2.000000	no	no	yes	no	no	no	no	no	no	yes
182203928	chr1	79235149	79235181	16	33	2.062500	no	no	yes	no	no	no	no	no	no	yes
182203929	chr1	79238359	79238407	20	49	2.428571	no	no	yes	no	no	no	no	no	no	yes
182203936	chr1	79259371	79259409	20	39	1.950000	no	no	yes	no	no	no	no	yes	no	yes
182203941	chr1	79279273	79279346	37	74	2.000000	no	no	yes	no	no	no	no	no	no	yes
182203944	chr1	79281801	79281838	16	38	2.375000	no	no	yes	no	no	no	no	yes	no	yes
182203946	chr1	79281852	79281890	14	39	2.785714	no	no	yes	no	no	no	no	yes	no	yes
182203951	chr1	79308399	79308435	14	37	2.642857	no	no	no	no	no	no	no	no	no	no
182203953	chr1	79316375	79316431	30	57	1.900000	no	no	no	no	no	no	no	no	no	no
182203961	chr1	79325397	79325433	16	37	2.312500	yes	no	yes	no	no	yes	no	no	no	yes
182203967	chr1	79327200	79327283	42	84	2.047619	no	no	yes	no	no	no	no	yes	no	yes
182203970	chr1	79337892	79338040	29	149	5.000000	no	no	yes	no	no	no	no	no	no	yes

182203971	chr1	79341474	79341515	21	42	2.000000	no	no	yes	no	no	no	no	no	no	yes
182203973	chr1	79352362	79352399	20	38	1.900000	no	no	no	no	no	no	no	no	no	no
182203985	chr1	79384811	79384848	21	38	1.904762	no	no	no	no	no	no	no	no	no	no
182203996	chr1	79429087	79429115	15	29	1.933333	no	no	no	no	no	no	no	no	no	no
182203999	chr1	79437198	79437237	16	40	2.500000	no	no	no	no	no	no	no	no	no	no
182204004	chr1	79455077	79455131	26	55	2.115385	no	no	no	no	no	no	no	no	no	no
182204005	chr1	79456329	79456371	20	43	2.200000	no	no	no	no	no	no	no	no	no	no
182204010	chr1	79485532	79485561	15	30	2.066667	no	no	no	no	no	no	no	no	no	no
182204015	chr1	79496401	79496429	15	29	1.933333	no	no	no	no	no	no	no	yes	no	no
182204016	chr1	79502824	79502860	12	37	3.083333	no	no	no	no	no	no	no	no	no	no
182204020	chr1	79520013	79520268	126	256	2.039683	yes	no	no	no	no	no	no	no	no	no
182204023	chr1	79524965	79525018	24	54	2.320000	no	no	no	no	no	no	no	no	no	no
182204033	chr1	79531485	79531525	20	41	2.050000	no	no	no	no	no	no	no	no	no	no
182204043	chr1	79571279	79572654	33	1376	41.434784	no	no	no	no	no	no	no	yes	no	no
182204046	chr1	79576284	79576320	14	37	2.642857	no	no	no	no	no	no	no	no	no	no
182204060	chr1	79614437	79614490	29	54	1.931034	no	no	no	no	no	no	no	yes	no	no
182204061	chr1	79616210	79616243	17	34	2.000000	no	no	no	no	no	no	no	no	no	no
182204062	chr1	79617371	79617451	36	81	2.250000	no	no	no	no	no	no	no	no	no	no
182204063	chr1	79618917	79618958	16	42	2.687500	no	no	no	no	no	no	no	no	no	no
182204072	chr1	79627125	79627162	8	38	4.750000	no	no	no	no	no	no	no	no	no	no
182204081	chr1	79649920	79649982	31	63	2.032258	no	no	no	no	no	no	no	no	no	no
182204086	chr1	79682623	79682652	14	30	2.214286	no	no	no	no	no	no	no	no	no	no
182204087	chr1	79682676	79682711	16	36	2.250000	no	no	no	no	no	no	no	no	no	no
182204088	chr1	79682725	79682756	16	32	2.000000	no	no	no	no	no	no	no	no	no	no
182204090	chr1	79683844	79683947	30	104	3.600000	no	no	no	no	no	no	no	yes	no	no

182204093	chr1	79683997	79684028	13	32	2.461539	no	no	no	no	no	no	no	no	no	no
182204094	chr1	79684116	79684149	16	34	2.187500	no	no	no	no	no	no	no	no	no	no
182204096	chr1	79686296	79686327	14	32	2.285714	no	no	no	no	no	no	no	no	no	no
182204111	chr1	79722804	79722882	35	79	2.257143	no	no	no	no	no	no	no	no	no	no
182204117	chr1	79729769	79729851	40	83	2.075000	no	no	no	no	no	no	no	no	no	no
182204120	chr1	79738109	79738170	34	62	1.911765	no	no	no	no	no	no	no	no	no	no
182204126	chr1	79753090	79753161	32	72	2.250000	no	no	no	no	no	no	no	no	no	no
182204137	chr1	79767644	79767698	25	55	2.240000	no	no	no	no	no	no	no	no	no	no
182204139	chr1	79771209	79771264	28	56	2.000000	no	no	no	no	no	no	no	no	no	no
182204148	chr1	79795086	79795131	13	46	3.615385	no	no	no	no	no	no	no	no	no	no
182204150	chr1	79804209	79804250	20	42	2.150000	no	no	no	no	no	no	no	no	no	no
182204152	chr1	79829539	79829578	21	40	1.904762	no	no	no	no	no	no	no	no	no	no
182204154	chr1	79841522	79841551	15	30	2.000000	no	no	no	no	no	no	no	no	no	no
182204155	chr1	79842653	79842688	18	36	2.055556	no	no	no	no	no	no	no	no	no	no
182204160	chr1	79871553	79871583	16	31	1.937500	no	no	no	no	no	no	no	no	no	no
182204164	chr1	79902564	79902597	16	34	2.187500	no	no	no	no	no	no	no	yes	no	no
182204168	chr1	79913993	79914034	22	42	1.909091	no	no	no	no	no	no	no	no	no	no
182204169	chr1	79916040	79916105	29	66	2.275862	no	no	no	no	no	no	no	no	no	no
182204182	chr1	79947645	79947685	12	41	3.500000	no	no	no	no	no	no	no	no	no	no
182204191	chr1	79969509	79969938	214	430	2.009346	no	no	yes	no	no	no	no	yes	no	yes
182204192	chr1	79972683	79972731	18	49	2.722222	no	no	yes	no	no	no	no	no	no	yes
182204200	chr1	79992932	79993019	42	88	2.095238	no	no	yes	no	no	no	no	no	no	yes
182204204	chr1	80008499	80008532	18	34	1.944444	no	no	yes	no	no	no	no	no	no	yes
182204206	chr1	80008752	80008802	23	51	2.260870	no	no	yes	no	no	no	no	no	no	yes
182204209	chr1	80011362	80011406	24	45	1.916667	no	no	yes	no	no	no	no	no	no	yes

182204210	chr1	80013320	80013360	22	41	1.909091	no	no	yes	no	no	no	no	no	no	yes
182204217	chr1	80032153	80032238	37	86	2.351351	no	no	yes	no	no	no	no	no	no	yes
182204237	chr1	80065719	80065768	25	50	2.000000	no	no	no	no	no	no	no	no	no	no
182204238	chr1	80066841	80066873	16	33	2.062500	no	no	no	no	no	no	no	no	no	no
182204241	chr1	80074515	80074593	39	79	2.025641	no	no	no	no	no	no	no	no	no	no
182204248	chr1	80099181	80099215	17	35	2.058824	no	no	no	no	no	no	no	no	no	no
182204259	chr1	80122529	80122570	20	42	2.150000	no	no	no	no	no	no	no	no	no	no
182204263	chr1	80132511	80132553	19	43	2.263158	no	no	no	no	no	no	no	no	no	no
182204266	chr1	80139996	80140024	14	29	2.071429	no	no	no	no	no	no	no	no	no	no
182204271	chr1	80146776	80146827	26	52	2.000000	no	no	no	no	no	no	no	no	no	no
182204274	chr1	80156758	80156794	20	37	1.950000	no	no	no	no	no	no	no	no	no	no
182204283	chr1	80192756	80192793	20	38	1.950000	no	no	no	no	no	no	no	no	no	no
182204284	chr1	80192905	80192938	16	34	2.125000	no	no	no	no	no	no	no	no	no	no
182204286	chr1	80195362	80195409	25	48	1.920000	no	no	no	no	no	no	no	no	no	no
182204287	chr1	80195568	80195608	19	41	2.210526	no	no	no	no	no	no	no	no	no	no
182204304	chr1	80234413	80234446	16	34	2.125000	no	no	no	no	no	no	no	no	no	no
182204317	chr1	80272150	80272185	18	36	2.000000	no	no	no	no	no	no	no	no	no	no
182204320	chr1	80283181	80283232	25	52	2.080000	yes	no	no	no	no	no	no	no	no	no
182204322	chr1	80296833	80296876	18	44	2.444444	no	no	no	no	no	no	no	no	no	no
182204323	chr1	80297731	80297763	17	33	1.941176	no	no	no	no	no	no	no	no	no	no
182204327	chr1	80323453	80323503	26	51	1.961538	no	no	no	no	no	no	no	no	no	no
182204333	chr1	80330402	80330994	31	593	19.545454	no	no	no	no	no	no	yes	no	no	no
182204337	chr1	80331252	80331344	43	93	2.162791	no	no	no	no	no	no	no	no	no	no
182204349	chr1	80361460	80361687	101	228	2.346535	no	no	no	no	no	no	no	yes	no	no
182204358	chr1	80392301	80392341	15	41	2.866667	no	no	no	no	no	no	no	yes	no	no

182204363	chr1	80414951	80415000	24	50	2.083333	no	no	no	no	no	no	no	no	no	no
182204367	chr1	80414983	80415024	20	42	2.100000	no	no	no	no	no	no	no	no	no	no
182204374	chr1	80422093	80422127	13	35	2.692308	no	no	no	no	no	no	no	no	no	no
182204376	chr1	80438901	80438932	17	32	1.941176	no	no	no	no	no	no	no	no	no	no
182204381	chr1	80449630	80449667	18	38	2.111111	no	no	no	no	no	no	no	yes	no	no
182204384	chr1	80462655	80462688	16	34	2.125000	no	no	no	no	no	no	no	no	no	no
182204386	chr1	80464038	80464071	16	34	2.125000	no	no	no	no	no	yes	no	no	no	no
182204405	chr1	80503488	80503522	16	35	2.187500	no	no	no	no	no	no	no	no	no	no
182204407	chr1	80503621	80503650	14	30	2.142857	no	no	no	no	no	no	no	no	no	no
182204408	chr1	80503767	80503797	16	31	1.937500	no	no	no	no	no	no	no	no	no	no
182204409	chr1	80504163	80504212	25	50	2.000000	no	no	no	no	no	no	no	no	no	no
182204419	chr1	80520986	80521087	51	102	2.000000	no	no	no	no	no	no	no	yes	no	no
182204422	chr1	80531765	80531837	35	73	2.085714	no	no	no	no	no	no	no	no	no	no
182204423	chr1	80532103	80532142	21	40	1.952381	no	no	no	no	no	no	no	no	yes	no
182204424	chr1	80533547	80533580	18	34	1.944444	no	no	no	no	no	no	no	yes	yes	no
182204426	chr1	80538975	80539022	23	48	2.130435	no	no	yes	no	no	no	no	no	no	yes
182204463	chr1	80644545	80644581	18	37	2.055556	no	no	yes	no	no	no	no	no	no	yes
182204464	chr1	80650141	80650171	16	31	1.937500	no	no	no	no	no	no	no	no	no	no
182204465	chr1	80662218	80662251	15	34	2.266667	no	no	no	no	no	no	no	no	no	no
182204468	chr1	80673633	80673685	29	53	1.931034	no	no	no	no	no	no	no	no	no	no
182204469	chr1	80676179	80676223	13	45	3.615385	no	no	no	no	no	no	no	no	no	no
182204480	chr1	80700161	80700499	149	339	2.340000	no	no	no	no	no	no	no	no	no	no
182204482	chr1	80701434	80701515	18	82	4.476191	no	no	no	no	no	no	no	yes	no	no
182204501	chr1	80749652	80749704	27	53	2.074074	no	no	no	no	no	no	no	no	no	no
182204504	chr1	80765795	80765828	18	34	1.944444	no	no	no	no	no	no	no	no	no	no

182204512	chr1	80817571	80817613	22	43	2.045455	no	no	no	no	no	no	no	no	no	no
182204518	chr1	80833110	80833153	23	44	1.913043	no	no	no	no	no	no	no	no	no	no
182204520	chr1	80840861	80840919	28	59	2.107143	no	no	no	no	no	no	no	no	no	no
182204521	chr1	80846598	80846640	18	43	2.444444	no	no	no	no	no	no	no	no	no	no
182204524	chr1	80852142	80852186	23	45	2.043478	no	no	no	no	no	no	no	no	no	no
182204531	chr1	80877717	80877772	26	56	2.192308	no	no	no	no	no	no	no	no	no	no
182204535	chr1	80898526	80898554	12	29	2.416667	no	no	no	no	no	no	no	no	no	no
182204538	chr1	80908749	80908791	18	43	2.368421	no	no	no	no	no	no	no	yes	no	no
182204545	chr1	80929640	80929681	22	42	1.954545	no	no	no	no	no	no	no	no	no	no
182204562	chr1	80975683	80975724	23	42	1.913043	no	no	no	no	no	no	no	no	no	no
182204563	chr1	80975716	80975753	15	38	2.600000	no	no	no	no	no	no	no	no	no	no
182204564	chr1	80976677	80976715	20	39	1.950000	no	no	no	no	no	no	no	no	no	no
182204572	chr1	80990195	80990230	18	36	2.111111	no	no	no	no	no	no	no	no	no	no
182204581	chr1	81026393	81026430	16	38	2.375000	no	no	no	no	no	no	no	no	no	no
182204594	chr1	81040744	81040787	20	44	2.200000	no	no	no	no	no	no	no	no	no	no
182204596	chr1	81042571	81042599	15	29	1.933333	no	no	no	no	no	no	no	yes	no	no
182204600	chr1	81047557	81047601	20	45	2.250000	no	no	no	no	no	no	no	yes	no	no
182204603	chr1	81057030	81057083	18	54	3.000000	no	no	no	no	no	no	no	no	no	no
182204605	chr1	81060450	81060559	56	110	1.982143	no	no	no	no	no	no	no	no	no	no
182204606	chr1	81064707	81064745	19	39	2.052632	no	no	no	no	no	no	no	no	no	no
182204617	chr1	81094844	81094953	60	110	1.916667	no	no	no	no	no	no	no	no	no	no
182204619	chr1	81101305	81101339	16	35	2.187500	no	no	no	no	no	no	no	no	no	no
182204625	chr1	81135744	81135784	19	41	2.157895	no	no	no	no	no	no	no	yes	yes	no
182204626	chr1	81144834	81144868	16	35	2.187500	no	no	no	no	no	no	no	no	no	no
182204636	chr1	81188751	81188787	20	37	1.950000	no	no	no	no	no	no	no	no	no	no

182204638	chr1	81198452	81198487	17	36	2.117647	no	no	no	no	no	no	no	yes	no	no
182204642	chr1	81218442	81218475	17	34	2.000000	no	no	yes	no	no	no	no	yes	yes	yes
182204644	chr1	81222888	81222918	14	31	2.214286	no	no	no	no	no	yes	no	no	no	no
182204648	chr1	81238293	81238344	22	52	2.363636	no	no	no	no	no	no	no	yes	yes	no
182204663	chr1	81270663	81270698	16	36	2.250000	no	no	no	no	no	no	no	no	no	no
182204664	chr1	81274022	81274057	16	36	2.250000	no	no	no	no	no	no	no	no	no	no
182204675	chr1	81292765	81292807	23	43	1.956522	no	no	no	no	no	no	no	no	no	no
182204685	chr1	81317805	81317873	36	69	1.916667	no	no	yes	no	no	no	no	yes	no	yes
182204692	chr1	81324541	81324596	28	56	2.071429	no	no	yes	no	no	no	no	no	no	yes
182204693	chr1	81327994	81328031	20	38	1.950000	no	no	yes	no	no	no	no	yes	yes	yes
182204712	chr1	81355963	81356003	21	41	2.000000	no	no	yes	no	no	no	no	no	no	yes
182204717	chr1	81363571	81363655	41	85	2.097561	no	no	yes	no	no	no	no	no	no	yes
182204718	chr1	81368718	81368778	27	61	2.259259	no	no	yes	no	no	no	no	no	no	yes
182204719	chr1	81374930	81374969	20	40	2.050000	no	no	yes	no	no	no	no	no	no	yes
182204727	chr1	81393815	81393851	10	37	4.000000	no	no	yes	no	no	no	no	no	no	yes
182204736	chr1	81407546	81407578	16	33	2.062500	no	no	yes	no	no	no	no	yes	no	yes
182204740	chr1	81426986	81427050	31	65	2.096774	no	yes	yes	no	no	no	no	no	no	yes
182204750	chr1	81449097	81449331	77	235	3.051948	no	no	yes	no	no	no	no	no	no	yes
182204759	chr1	81461259	81461302	22	44	2.000000	no	no	yes	no	no	no	no	no	no	yes
182204765	chr1	81464932	81464968	18	37	2.055556	no	no	yes	no	no	no	no	yes	no	yes
182204771	chr1	81482805	81482852	25	48	1.920000	no	no	yes	no	no	no	no	no	no	yes
182204772	chr1	81485503	81485538	18	36	2.000000	no	no	yes	no	no	no	no	no	no	yes
182204781	chr1	81509495	81509522	14	28	2.000000	no	no	yes	no	no	no	no	no	yes	yes
182204783	chr1	81513719	81513747	15	29	1.933333	no	no	yes	no	no	yes	no	no	no	yes
182204787	chr1	81521891	81521915	11	25	2.272727	no	no	yes	no	no	no	no	no	no	yes

182204793	chr1	81535395	81535441	12	47	3.916667	no	no	yes	no	no	no	no	no	yes	yes
182204794	chr1	81536612	81536651	17	40	2.411765	no	no	yes	no	no	no	no	yes	no	yes
182204799	chr1	81552608	81552645	20	38	1.900000	no	no	yes	no	no	no	no	yes	no	yes
182204825	chr1	81629994	81630032	16	39	2.437500	no	no	yes	no	no	no	no	no	no	yes
182204852	chr1	81713485	81713525	21	41	1.952381	no	no	yes	no	no	no	no	no	no	yes
182204853	chr1	81720635	81720671	15	37	2.466667	no	no	yes	no	no	no	no	yes	no	yes
182204854	chr1	81723197	81723386	94	190	2.021277	no	no	yes	no	no	yes	no	yes	no	yes
182204857	chr1	81729085	81729128	23	44	1.956522	no	no	yes	no	no	no	no	no	no	yes
182204859	chr1	81736453	81736509	24	57	2.375000	no	no	yes	no	no	no	no	no	no	yes
182204862	chr1	81746833	81746937	28	105	3.750000	no	no	yes	no	no	no	no	yes	no	yes
182204870	chr1	81749028	81749066	18	39	2.166667	no	no	yes	no	no	no	no	no	no	yes
182204871	chr1	81754136	81754218	43	83	1.930233	no	no	yes	no	no	no	no	yes	no	yes
182204880	chr1	81755122	81755201	18	80	4.400000	no	no	yes	no	no	no	no	no	no	yes
182204882	chr1	81755187	81755242	28	56	2.000000	no	no	yes	no	no	no	no	no	no	yes
182204892	chr1	81783823	81783857	18	35	1.944444	no	no	yes	no	no	no	no	no	no	yes
182204894	chr1	81790265	81790296	12	32	2.666667	no	no	yes	no	no	no	no	no	no	yes
182204896	chr1	81791880	81791908	15	29	1.933333	no	no	yes	no	no	no	no	yes	no	yes
182204897	chr1	81795474	81795506	16	33	2.125000	no	no	yes	no	no	no	no	no	no	yes
182204899	chr1	81797278	81797314	18	37	2.166667	no	no	yes	no	no	no	no	no	no	yes
182204903	chr1	81812394	81812469	36	76	2.111111	no	no	yes	no	no	no	no	yes	yes	yes
182204904	chr1	81822374	81822438	27	65	2.413793	no	no	yes	no	no	no	no	no	no	yes
182204908	chr1	81823321	81823395	28	75	2.750000	no	no	yes	no	no	no	no	yes	no	yes
182204911	chr1	81823920	81823960	18	41	2.333333	no	no	yes	no	no	no	no	no	no	yes
182204913	chr1	81839736	81839924	22	189	8.750000	no	no	yes	no	no	no	no	no	no	yes
182204917	chr1	81840612	81840691	40	80	2.000000	no	no	yes	no	no	no	no	yes	no	yes

182204919	chr1	81855527	81855562	15	36	2.400000	no	no	yes	no	no	no	no	no	no	yes
182204920	chr1	81856871	81856918	24	48	2.000000	no	no	yes	no	no	no	no	no	no	yes
182204922	chr1	81862233	81862265	17	33	2.000000	no	no	yes	no	no	no	no	no	no	yes
182204925	chr1	81874396	81874428	17	33	1.941176	no	no	yes	no	no	no	no	no	no	yes
182204928	chr1	81880037	81880091	27	55	2.037037	no	no	yes	no	no	no	no	no	no	yes
182204929	chr1	81883667	81883831	74	165	2.243243	no	no	yes	no	no	no	no	no	no	yes
182204931	chr1	81896682	81896718	17	37	2.235294	no	no	yes	no	no	no	no	no	no	yes
182204933	chr1	81903718	81903796	28	79	2.821429	no	no	yes	no	no	no	no	no	no	yes
182204941	chr1	81919094	81919134	22	41	1.954545	no	no	yes	no	no	no	no	no	no	yes
182204943	chr1	81955693	81955735	21	43	2.047619	yes	no	yes	no	no	no	no	no	no	yes
182204944	chr1	81958587	81958615	15	29	1.933333	no	no	yes	no	no	no	no	no	no	yes
182204958	chr1	82032027	82032069	21	43	2.190476	no	no	no	no	no	no	no	no	no	no
182204959	chr1	82041775	82041803	14	29	2.071429	no	no	no	no	no	no	no	no	no	no
182204961	chr1	82046201	82046459	37	259	7.000000	no	no	no	no	no	no	no	no	no	no
182204979	chr1	82106990	82107027	11	38	3.416667	no	no	no	no	no	no	no	yes	no	no
182204986	chr1	82127246	82127285	19	40	2.105263	no	no	no	no	no	no	no	no	yes	no
182204988	chr1	82145954	82145984	14	31	2.214286	no	no	no	no	no	no	no	no	no	no
182205004	chr1	82190051	82190127	39	77	1.974359	no	no	no	no	no	no	no	no	no	no
182205019	chr1	82268009	82268046	17	38	2.235294	no	no	yes	no	no	no	no	no	yes	yes
182205023	chr1	82293978	82294022	18	45	2.500000	no	no	yes	no	no	no	no	no	no	yes
182205031	chr1	82328961	82328994	17	34	2.000000	no	no	yes	no	no	no	no	no	no	yes
182205041	chr1	82362050	82362166	39	117	3.000000	no	no	yes	no	no	no	no	no	no	yes
182205046	chr1	82373527	82373598	20	72	3.650000	no	no	yes	no	no	no	no	yes	no	yes
182205050	chr1	82385363	82385479	58	117	2.017241	no	no	yes	no	no	no	no	yes	no	yes
182205052	chr1	82386016	82386049	16	34	2.125000	no	no	yes	no	no	no	no	no	no	yes

182205053	chr1	82388706	82388752	22	47	2.136364	no	no	yes	no	no	no	no	yes	no	yes
182205057	chr1	82402319	82402359	17	41	2.411765	no	no	yes	no	no	no	no	no	no	yes
182205058	chr1	82404256	82404290	18	35	1.944444	no	no	yes	no	no	no	no	no	no	yes
182205061	chr1	82407504	82407568	18	65	3.611111	no	no	yes	no	no	no	no	no	no	yes
182205081	chr1	82437729	82437757	13	29	2.230769	no	no	yes	no	no	no	no	no	no	yes
182205085	chr1	82448705	82448756	19	52	2.736842	no	no	yes	no	no	no	no	no	no	yes
182205090	chr1	82467038	82467069	14	32	2.285714	no	no	yes	no	no	no	no	yes	no	yes
182205091	chr1	82468495	82468528	17	34	2.000000	no	no	yes	no	no	no	no	no	no	yes
182205093	chr1	82476142	82476173	17	32	1.941176	no	no	yes	no	no	no	no	no	no	yes
182205098	chr1	82493398	82493438	20	41	2.200000	no	no	yes	no	no	no	no	no	no	yes
182205104	chr1	82503272	82503309	16	38	2.375000	no	no	yes	no	no	no	no	no	no	yes
182205107	chr1	82521245	82521284	21	40	1.904762	no	no	yes	no	no	no	no	no	no	yes
182205109	chr1	82525325	82525392	30	68	2.366667	no	no	yes	no	no	no	no	yes	no	yes
182205122	chr1	82558719	82558753	16	35	2.187500	no	no	yes	no	no	no	no	no	no	yes
182205128	chr1	82579388	82579431	23	44	1.913043	no	no	yes	no	no	no	no	no	no	yes
182205141	chr1	82637206	82637253	17	48	2.882353	no	no	yes	no	no	no	no	no	no	yes
182205142	chr1	82638229	82638268	18	40	2.222222	no	no	yes	no	no	no	no	no	no	yes
182205143	chr1	82638793	82638850	29	58	2.000000	no	no	yes	no	no	no	no	no	no	yes
182205146	chr1	82654141	82654169	12	29	2.500000	no	no	yes	no	no	no	no	no	no	yes
182205147	chr1	82656609	82656642	13	34	2.615385	no	no	yes	no	no	no	no	no	yes	yes
182205153	chr1	82680426	82680473	19	48	2.500000	no	no	yes	no	no	no	no	no	no	yes
182205155	chr1	82681854	82681897	22	44	2.000000	no	no	yes	no	no	no	no	no	no	yes
182205175	chr1	82745272	82745308	19	37	2.000000	no	no	yes	no	no	no	no	no	no	yes
182205183	chr1	82766597	82766632	18	36	2.055556	no	no	yes	no	no	no	no	no	no	yes
182205184	chr1	82774039	82774089	18	51	2.833333	no	no	yes	no	no	no	no	no	no	yes

182205185	chr1	82774867	82774912	23	46	2.000000	no	no	yes	no	no	no	no	no	no	yes
182205192	chr1	82787823	82787873	23	51	2.260870	no	no	yes	no	no	no	no	no	no	yes
182205194	chr1	82792601	82792629	15	29	1.933333	no	no	yes	no	no	no	no	no	no	yes
182205195	chr1	82792759	82792799	20	41	2.050000	no	no	yes	no	no	no	no	no	no	yes
182205209	chr1	82822501	82822525	10	25	2.500000	no	no	yes	no	no	no	no	no	no	yes
182205212	chr1	82834362	82834396	16	35	2.187500	no	no	yes	no	no	no	no	no	no	yes
182205220	chr1	82856616	82856644	11	29	2.727273	no	no	no	no	no	no	no	no	no	no
182205225	chr1	82866480	82866512	16	33	2.125000	no	no	no	no	no	no	no	no	no	no
182205231	chr1	82879705	82879748	21	44	2.095238	no	no	no	no	no	no	no	no	yes	no
182205240	chr1	82928614	82928651	19	38	2.105263	no	no	yes	no	no	no	no	no	no	yes
182205243	chr1	82934477	82934527	18	51	2.888889	no	no	yes	no	no	no	no	no	no	yes
182205245	chr1	82952576	82952638	22	63	2.909091	no	no	yes	no	no	no	no	no	no	yes
182205247	chr1	82953961	82954077	61	117	1.934426	no	no	yes	no	no	no	no	no	no	yes
182205248	chr1	82954191	82954225	17	35	2.058824	no	no	yes	no	no	no	no	no	no	yes
182205259	chr1	82987901	82987961	20	61	2.954545	no	no	yes	no	no	no	no	no	no	yes
182205261	chr1	82992522	82992582	25	61	2.480000	no	no	yes	no	no	no	no	no	no	yes
182205269	chr1	83016771	83016798	14	28	2.000000	no	no	yes	no	no	no	no	yes	no	yes
182205271	chr1	83018881	83018991	42	111	2.642857	no	no	yes	no	no	no	no	no	no	yes
182205276	chr1	83019015	83019270	127	256	2.062992	no	no	yes	no	no	no	no	no	no	yes
182205285	chr1	83034595	83034650	20	56	2.761905	no	no	yes	no	no	no	no	no	no	yes
182205291	chr1	83053548	83053605	30	58	1.966667	no	no	yes	no	no	no	no	no	yes	yes
182205299	chr1	83068069	83068260	103	192	1.922330	no	no	yes	no	no	no	no	no	no	yes
182205300	chr1	83071089	83071151	21	63	3.095238	no	no	yes	no	no	no	no	no	no	yes
182205301	chr1	83071589	83071715	64	127	1.984375	no	no	yes	no	no	no	no	no	no	yes
182205302	chr1	83072840	83072865	12	26	2.166667	no	no	yes	no	no	no	no	no	no	yes

182205303	chr1	83074005	83074035	12	31	2.583333	no	no	yes	no	no	no	no	yes	no	yes
182205305	chr1	83075166	83075200	16	35	2.250000	no	no	yes	no	no	no	no	yes	no	yes
182205308	chr1	83083140	83083177	17	38	2.294118	no	no	yes	no	no	no	no	no	no	yes
182205309	chr1	83083657	83083716	31	60	2.000000	no	no	yes	no	no	no	no	no	no	yes
182205329	chr1	83111528	83111571	14	44	3.285714	no	no	yes	no	no	no	no	no	no	yes
182205333	chr1	83115656	83115742	26	87	3.423077	no	no	yes	no	no	no	no	no	no	yes
182205339	chr1	83122251	83122293	23	43	1.913043	no	no	yes	no	no	no	no	no	yes	yes
182205355	chr1	83173957	83173998	19	42	2.263158	no	no	no	no	no	no	no	no	no	no
182205362	chr1	83209100	83209159	31	60	1.935484	no	no	no	no	no	no	no	no	no	no
182205386	chr1	83271010	83271049	21	40	1.952381	no	no	no	no	no	no	no	no	yes	no
182205391	chr1	83287807	83288017	107	211	1.971963	no	no	no	no	no	no	no	no	no	no
182205436	chr1	83417819	83417880	26	62	2.461539	no	no	yes	no	no	no	no	no	no	yes
182205448	chr1	83443967	83444014	25	48	1.960000	no	no	no	no	no	no	no	no	no	no
182205467	chr1	83504917	83504954	20	38	1.900000	no	no	no	no	no	no	no	no	no	no
182205478	chr1	83553183	83553212	13	30	2.307692	no	no	no	no	no	no	no	yes	no	no
182205496	chr1	83625262	83625296	11	35	3.083333	no	no	yes	no	no	no	no	no	no	yes
182205499	chr1	83636110	83636150	19	41	2.157895	no	no	yes	no	no	no	no	no	no	yes
182205502	chr1	83642074	83642112	14	39	2.785714	no	no	yes	no	no	no	no	yes	yes	yes
182205512	chr1	83670562	83670598	16	37	2.312500	no	no	yes	no	no	no	no	no	no	yes
182205515	chr1	83676269	83676335	27	67	2.481482	no	no	yes	no	no	no	no	yes	yes	yes
182205516	chr1	83683319	83683359	18	41	2.277778	no	no	yes	no	no	no	no	no	no	yes
182205518	chr1	83691640	83691670	14	31	2.285714	no	no	yes	no	no	no	no	no	no	yes
182205519	chr1	83696745	83696777	10	33	3.300000	no	no	yes	no	no	no	no	yes	no	yes
182205523	chr1	83721776	83721812	17	37	2.176471	no	no	yes	no	no	no	no	no	no	yes
182205524	chr1	83723153	83723183	12	31	2.583333	no	no	yes	no	no	no	no	no	no	yes

182205526	chr1	83731714	83731761	23	48	2.086957	no	no	yes	no	no	no	no	no	no	yes
182205534	chr1	83741146	83741184	19	39	2.052632	no	no	yes	no	no	no	no	no	no	yes
182205548	chr1	83795109	83795143	8	35	4.375000	no	no	yes	no	no	no	no	no	yes	yes
182205552	chr1	83807221	83807251	15	31	2.133333	no	no	yes	no	no	no	no	no	no	yes
182205553	chr1	83807641	83807669	14	29	2.071429	no	no	yes	no	no	no	no	no	no	yes
182205558	chr1	83831077	83831130	28	54	1.928571	no	no	yes	no	no	yes	no	no	no	yes
182205562	chr1	83837014	83837058	19	45	2.350000	no	no	yes	no	no	no	no	yes	no	yes
182205584	chr1	83892979	83893022	17	44	2.588235	no	no	yes	no	no	no	no	no	no	yes
182205585	chr1	83899551	83899588	12	38	3.166667	no	no	yes	no	no	no	no	no	no	yes
182205590	chr1	83928937	83929032	31	96	3.096774	no	no	yes	no	no	no	no	yes	no	yes
182205591	chr1	83929605	83929644	20	40	2.000000	no	no	yes	no	no	no	no	yes	no	yes
182205594	chr1	83956108	83956147	18	40	2.222222	no	no	yes	no	no	no	no	no	no	yes
182205597	chr1	83964619	83964669	24	51	2.125000	no	no	yes	no	no	no	no	no	no	yes
182205598	chr1	83964659	83964692	17	34	2.000000	no	no	yes	no	no	no	no	no	no	yes
182205609	chr1	84020180	84020214	18	35	1.944444	no	no	no	no	no	no	no	yes	no	no
182205610	chr1	84024692	84024727	17	36	2.176471	no	no	no	no	no	no	no	no	no	no
182205612	chr1	84027589	84027660	31	72	2.354839	no	no	no	no	no	no	no	yes	yes	no
182205623	chr1	84048909	84048950	22	42	2.000000	no	no	yes	no	no	no	no	yes	no	yes
182205633	chr1	84083391	84083417	14	27	1.928571	no	no	yes	no	no	no	no	yes	yes	yes
182205648	chr1	84133585	84133643	20	59	2.950000	no	no	yes	no	no	no	no	yes	no	yes
182205652	chr1	84142507	84142828	166	322	1.987952	no	no	yes	no	no	no	no	no	yes	yes
182205653	chr1	84142942	84142972	14	31	2.214286	no	no	yes	no	no	no	no	no	no	yes
182205657	chr1	84165401	84165432	14	32	2.357143	no	no	yes	no	no	no	no	yes	no	yes
182205666	chr1	84194272	84194303	15	32	2.200000	no	no	yes	no	no	no	no	no	yes	yes
182205668	chr1	84198889	84198940	27	52	1.925926	no	no	yes	no	no	no	no	no	no	yes

182205673	chr1	84207063	84207119	30	57	1.900000	no	no	yes	no	no	no	no	no	no	yes
182205674	chr1	84210246	84210277	14	32	2.285714	no	no	yes	no	no	no	no	no	no	yes
182205675	chr1	84210300	84210341	22	42	1.909091	no	no	yes	no	no	no	no	no	no	yes
182205677	chr1	84213012	84213041	15	30	2.000000	no	no	yes	no	no	no	no	no	no	yes
182205685	chr1	84258681	84258716	16	36	2.250000	no	no	no	no	no	no	no	yes	no	no
182205696	chr1	84291601	84291651	28	51	1.928571	no	no	no	no	no	no	no	no	no	no
182205699	chr1	84299658	84299698	8	41	5.125000	no	no	yes	no	no	no	no	yes	yes	yes
182205716	chr1	84354804	84354834	13	31	2.384615	no	no	yes	no	no	no	no	yes	no	yes
182205723	chr1	84378595	84378649	26	55	2.115385	no	no	yes	no	no	no	no	no	no	yes
182205738	chr1	84424968	84425002	14	35	2.466667	no	no	no	no	no	no	no	no	yes	no
182205746	chr1	84457665	84457689	13	25	1.923077	no	no	no	no	no	no	no	no	no	no
182205754	chr1	84496101	84496138	19	38	2.105263	no	no	yes	no	no	no	no	yes	no	yes
182205755	chr1	84511440	84511642	98	203	2.071429	no	no	yes	no	no	no	no	yes	yes	yes
182205786	chr1	84572211	84572243	10	33	3.300000	no	no	yes	no	no	no	no	no	no	yes
182205787	chr1	84574316	84574349	9	34	3.777778	yes	yes	no	yes	yes	no	yes	yes	yes	yes
182205788	chr1	84574483	84574537	19	55	2.850000	yes	no	yes	no	no	no	yes	yes	yes	yes
182205790	chr1	84581656	84581707	24	52	2.166667	no	no	yes	no	no	no	no	yes	yes	yes
182205791	chr1	84582177	84582212	16	36	2.250000	no	no	yes	no	no	no	no	no	no	yes
182205812	chr1	84646435	84646459	11	25	2.272727	no	yes	no	no	no	yes	no	no	no	yes
182205817	chr1	84670906	84670944	18	39	2.222222	no	no	yes	no	no	no	no	no	no	yes
182205820	chr1	84679053	84679105	26	53	2.038461	no	no	yes	no	no	no	no	yes	no	yes
182205822	chr1	84689749	84689804	29	56	1.931034	no	no	yes	no	no	no	no	yes	yes	yes
182205823	chr1	84690820	84690863	17	44	2.588235	yes	no	no	no	no	no	no	yes	no	no
182205824	chr1	84699091	84699124	17	34	2.000000	no	no	no	no	no	no	no	yes	no	no
182205836	chr1	84738756	84738791	14	36	2.571429	no	no	no	no	no	no	no	no	no	no

182205838	chr1	84738797	84738977	28	181	6.464286	no	no	no	no	no	no	no	no	no	no
182205842	chr1	84746399	84746436	18	38	2.111111	no	no	no	no	no	no	no	no	no	no
182205851	chr1	84756737	84756804	33	68	2.090909	no	no	no	no	no	no	no	yes	yes	no
182205857	chr1	84761235	84761263	15	29	1.933333	no	no	no	no	no	no	no	no	no	no
182205862	chr1	84770356	84770446	40	91	2.275000	no	no	no	no	no	no	no	yes	no	no
182205863	chr1	84776783	84776819	17	37	2.176471	no	no	no	no	no	no	no	yes	yes	no
182205865	chr1	84791748	84791792	10	45	4.636364	no	no	no	no	no	no	no	yes	no	no
182205870	chr1	84798013	84798066	26	54	2.076923	no	no	no	no	no	no	no	no	no	no
182205896	chr1	84851193	84851229	17	37	2.176471	no	no	yes	no	no	no	no	no	no	yes
182205915	chr1	84911334	84911389	21	56	2.714286	no	no	no	no	no	no	no	no	yes	no
182205916	chr1	84917612	84917656	21	45	2.142857	no	no	no	no	no	no	no	no	no	no
182205918	chr1	84918474	84918542	16	69	4.312500	no	no	no	no	no	no	no	no	no	no
182205925	chr1	84938287	84938390	52	104	2.000000	no	no	yes	no	no	no	no	no	no	yes
182205946	chr1	84988480	84988524	17	45	2.764706	no	no	yes	no	no	no	no	yes	no	yes
182205961	chr1	85018587	85018636	16	50	3.125000	no	yes	yes	no	yes	yes	no	no	no	yes
182205962	chr1	85025622	85025656	18	35	2.000000	no	yes	yes	no	yes	no	no	no	no	yes
182205963	chr1	85033039	85033075	17	37	2.176471	no	no	yes	no	no	no	no	no	yes	yes
182205966	chr1	85039200	85039228	14	29	2.071429	no	no	yes	no	no	no	no	no	no	yes
182205972	chr1	85053891	85053930	21	40	1.904762	no	no	yes	no	no	no	no	no	no	yes
182205977	chr1	85067238	85067265	14	28	2.000000	no	no	yes	no	no	no	no	yes	no	yes
182205986	chr1	85084357	85084393	17	37	2.294118	no	no	yes	no	no	no	no	no	no	yes
182205989	chr1	85095374	85095410	19	37	2.000000	no	no	yes	no	no	no	no	yes	no	yes
182205992	chr1	85096461	85096506	19	46	2.421053	no	no	yes	no	no	no	no	no	no	yes
182205997	chr1	85108981	85109013	15	33	2.200000	no	no	yes	no	no	no	no	yes	no	yes
182206001	chr1	85125223	85125267	23	45	1.956522	no	no	yes	no	no	no	no	no	no	yes

182206005	chr1	85132697	85132733	8	37	4.625000	no	no	yes	no	no	no	no	no	no	yes
182206025	chr1	85189117	85189276	77	160	2.077922	no	no	yes	no	no	no	no	yes	yes	yes
182206026	chr1	85194325	85194432	56	108	1.928571	no	no	yes	no	no	no	no	no	no	yes
182206031	chr1	85201189	85201217	15	29	1.933333	yes	no	no	no	no	no	yes	yes	yes	no
182206035	chr1	85211458	85211499	19	42	2.210526	no	no	no	no	no	no	no	yes	no	no
182206060	chr1	85277006	85277052	18	47	2.611111	yes	no	yes	no	no	no	no	yes	yes	yes
182206070	chr1	85303448	85303485	20	38	1.950000	no	no	yes	no	no	no	no	yes	yes	yes
182206077	chr1	85316370	85316402	15	33	2.200000	no	no	yes	no	no	no	no	yes	no	yes
182206088	chr1	85344545	85344613	29	69	2.379310	no	no	yes	no	no	no	no	yes	yes	yes
182206090	chr1	85353719	85353743	13	25	1.923077	no	no	yes	no	no	no	no	no	no	yes
182206093	chr1	85359130	85359337	105	208	1.980952	no	no	yes	no	no	no	no	yes	yes	yes
182206094	chr1	85359299	85359453	79	155	1.962025	no	no	yes	no	no	no	no	yes	yes	yes
182206103	chr1	85403189	85403245	12	57	4.750000	no	no	yes	no	no	no	no	yes	no	yes
182206111	chr1	85433070	85433124	22	55	2.500000	no	no	yes	no	no	no	no	no	no	yes
182206115	chr1	85434158	85434201	14	44	3.214286	no	no	yes	no	no	yes	no	yes	no	yes
182206116	chr1	85436489	85436529	21	41	2.000000	no	no	yes	no	no	no	no	no	yes	yes
182206121	chr1	85483252	85483277	13	26	2.000000	no	no	yes	no	no	no	no	no	no	yes
182206123	chr1	85487494	85487546	26	53	2.076923	no	no	yes	no	no	yes	no	no	no	yes
182206152	chr1	85549472	85549510	20	39	1.950000	no	no	yes	no	no	no	no	no	yes	yes
182206153	chr1	85550463	85550513	28	51	1.964286	no	no	yes	no	no	no	no	no	no	yes
182206156	chr1	85561655	85561703	24	49	2.041667	no	no	yes	no	no	no	no	no	no	yes
182206163	chr1	85568681	85568707	14	27	1.928571	no	no	yes	no	no	no	no	yes	no	yes
182206171	chr1	85580437	85580560	64	124	1.937500	yes	no	no	no	no	no	no	yes	yes	no
182206179	chr1	85597060	85597101	19	42	2.210526	no	no	no	no	no	no	no	no	yes	no
182206183	chr1	85615799	85615829	13	31	2.384615	no	no	no	no	no	no	no	yes	yes	no

182206185	chr1	85618886	85618918	16	33	2.187500	no	no	no	no	no	no	no	no	no	no
182206186	chr1	85620078	85620107	13	30	2.307692	no	no	no	no	no	no	no	no	no	no
182206190	chr1	85628952	85628980	14	29	2.071429	no	no	no	no	no	no	no	yes	yes	no
182206194	chr1	85634345	85634377	17	33	1.941176	no	no	no	no	no	no	no	no	no	no
182206204	chr1	85657255	85657283	14	29	2.071429	no	no	yes	no	no	no	no	yes	yes	yes
182206205	chr1	85660620	85660747	32	128	4.000000	no	no	yes	no	no	no	no	no	yes	yes
182206208	chr1	85700688	85700730	21	43	2.047619	no	no	yes	no	no	no	no	yes	yes	yes
182206211	chr1	85707850	85707878	15	29	1.933333	no	no	no	yes	no	no	no	yes	yes	yes
182206213	chr1	85718294	85718336	18	43	2.388889	no	no	no	no	no	no	no	no	no	no
182206227	chr1	85732597	85732639	12	43	3.666667	no	no	yes	no	no	no	no	no	no	yes
182206233	chr1	85743980	85744030	15	51	3.312500	no	no	yes	no	no	no	no	yes	no	yes
182206237	chr1	85762509	85762563	16	55	3.437500	no	no	yes	no	no	no	no	no	no	yes
182206250	chr1	85793435	85793474	20	40	2.000000	no	no	yes	no	no	no	no	no	no	yes
182206263	chr1	85809772	85809821	19	50	2.684211	no	no	yes	no	no	no	no	no	no	yes
182206270	chr1	85845009	85845037	15	29	1.933333	no	no	yes	no	no	no	no	no	no	yes
182206272	chr1	85848861	85848888	14	28	2.000000	no	no	yes	no	no	no	no	no	no	yes
182206279	chr1	85862418	85862447	13	30	2.307692	no	no	yes	no	no	no	no	no	no	yes
182206281	chr1	85875182	85875237	24	56	2.375000	no	no	yes	no	no	no	no	no	no	yes
182206287	chr1	85908012	85908049	19	38	2.000000	no	no	yes	no	no	no	no	no	no	yes
182206296	chr1	85918578	85918610	17	33	1.941176	no	no	yes	no	no	no	no	no	no	yes
182206298	chr1	85920946	85920976	15	31	2.066667	no	no	yes	no	no	no	no	no	no	yes
182206304	chr1	85962769	85962809	22	41	1.954545	no	no	yes	no	no	no	no	no	no	yes
182206309	chr1	85989308	85989346	20	39	2.050000	no	no	yes	no	no	no	no	no	no	yes
182206318	chr1	86000152	86000195	21	44	2.095238	no	no	yes	no	no	no	no	no	no	yes
182206323	chr1	86039978	86040026	20	49	2.450000	no	no	yes	no	no	no	no	no	no	yes

182206324	chr1	86042519	86042558	21	40	1.904762	no	no	yes	no	no	no	no	no	no	yes
182206328	chr1	86048168	86048220	17	53	3.176471	no	no	yes	no	no	no	no	no	no	yes
182206329	chr1	86055363	86055401	19	39	2.105263	no	no	yes	no	no	no	no	no	no	yes
182206332	chr1	86063076	86063124	26	49	1.961538	no	no	yes	no	no	no	no	no	no	yes
182206337	chr1	86067484	86067520	16	37	2.312500	no	no	yes	no	no	no	no	no	no	yes
182206339	chr1	86089114	86089162	19	49	2.578947	no	no	yes	no	no	no	no	no	no	yes
182206342	chr1	86092192	86092234	21	43	2.142857	no	no	yes	no	no	no	no	no	no	yes
182206344	chr1	86097367	86097413	25	47	1.920000	no	no	yes	no	no	no	no	yes	no	yes
182206352	chr1	86106799	86106828	13	30	2.307692	no	no	yes	no	no	no	no	no	no	yes
182206355	chr1	86110126	86110192	34	67	2.000000	no	no	yes	no	no	no	no	no	no	yes
182206361	chr1	86116144	86116177	16	34	2.125000	no	no	yes	no	no	no	no	no	no	yes
182206364	chr1	86127253	86127293	16	41	2.562500	no	no	yes	no	no	no	no	no	no	yes
182206369	chr1	86139211	86139249	14	39	2.785714	no	no	yes	no	no	no	no	yes	no	yes
182206373	chr1	86151035	86151137	51	103	2.019608	no	no	yes	no	no	no	no	yes	yes	yes
182206379	chr1	86171302	86171342	22	41	1.909091	no	no	no	no	no	no	no	no	no	no
182206381	chr1	86174682	86174713	17	32	1.941176	no	no	no	no	no	no	no	no	no	no
182206389	chr1	86205008	86205066	26	59	2.296296	no	no	no	no	no	no	no	no	no	no
182206391	chr1	86208439	86208479	10	41	4.200000	no	no	no	no	no	no	no	yes	yes	no
182206395	chr1	86214229	86214260	16	32	2.000000	no	no	no	no	no	no	no	no	no	no
182206410	chr1	86221770	86221803	16	34	2.125000	no	no	no	no	no	no	no	no	no	no
182206414	chr1	86235637	86235684	18	48	2.631579	no	no	no	no	no	no	no	no	no	no
182206416	chr1	86236746	86236792	12	47	3.916667	no	no	no	no	no	no	no	no	no	no
182206429	chr1	86286746	86286795	24	50	2.083333	no	no	no	no	no	no	no	no	no	no
182206430	chr1	86287385	86287434	23	50	2.260870	no	no	no	no	no	no	no	no	no	no
182206434	chr1	86321050	86321081	15	32	2.133333	yes	no	no	no	no	no	no	no	no	no

182206441	chr1	86370266	86370309	21	44	2.095238	no	no	yes	no	no	yes	no	no	no	yes
182206454	chr1	86426144	86426176	16	33	2.125000	no	no	yes	no	no	no	no	no	no	yes
182206456	chr1	86431027	86431076	26	50	1.961538	no	no	yes	no	no	no	no	no	no	yes
182206469	chr1	86488857	86488899	22	43	2.045455	no	no	yes	no	no	no	no	no	no	yes
182206477	chr1	86519683	86519728	24	46	1.916667	no	no	no	no	no	no	no	no	no	no
182206484	chr1	86528052	86528076	13	25	1.923077	no	no	no	no	no	no	no	no	no	no
182206485	chr1	86529413	86529468	20	56	2.800000	no	no	no	no	no	no	no	no	no	no
182206491	chr1	86537913	86537976	25	64	2.576923	no	no	no	no	no	no	no	no	no	no
182206506	chr1	86618088	86618121	11	34	3.181818	no	no	yes	no	no	no	no	no	no	yes
182206509	chr1	86639981	86640021	21	41	2.095238	no	no	yes	no	no	no	no	no	no	yes
182206510	chr1	86642803	86642861	31	59	1.903226	no	no	yes	no	no	no	no	no	no	yes
182206522	chr1	86691761	86691801	16	41	2.687500	no	no	yes	no	no	no	no	no	no	yes
182206524	chr1	86692569	86692598	7	30	4.285714	no	no	yes	no	no	no	no	no	no	yes
182206532	chr1	86713117	86713160	21	44	2.190476	no	no	yes	no	no	no	no	no	no	yes
182206536	chr1	86724905	86724986	34	82	2.529412	no	no	yes	no	no	no	no	no	no	yes
182206539	chr1	86728670	86728705	18	36	2.000000	no	no	yes	no	no	no	no	no	no	yes
182206544	chr1	86750689	86750720	15	32	2.200000	no	no	no	no	no	no	no	no	yes	no
182206551	chr1	86769201	86769248	23	48	2.086957	no	no	no	no	no	no	no	yes	no	no
182206617	chr1	86883448	86883522	25	75	3.160000	no	no	yes	no	no	no	no	no	no	yes
182206620	chr1	86889274	86889307	18	34	1.944444	no	no	yes	no	no	no	no	no	no	yes
182206639	chr1	86928419	86928454	18	36	2.055556	no	no	yes	no	no	no	no	no	no	yes
182206642	chr1	86942467	86942529	32	63	2.000000	no	no	yes	no	no	no	no	no	yes	yes
182206643	chr1	86944240	86944282	24	43	1.916667	no	no	yes	no	no	no	no	no	no	yes
182206646	chr1	86957120	86957172	23	53	2.304348	no	no	yes	no	no	no	no	no	no	yes
182206654	chr1	86969106	86969130	13	25	1.923077	no	no	yes	no	no	no	no	no	no	yes

182206657	chr1	86980647	86980711	32	65	2.031250	no	no	yes	no	no	no	no	no	no	yes
182206671	chr1	87016448	87016486	20	39	1.950000	no	no	yes	no	no	no	no	no	no	yes
182206672	chr1	87017670	87017845	58	176	3.016949	no	no	yes	no	no	no	no	no	no	yes
182206675	chr1	87028842	87028876	19	35	1.947368	no	no	yes	no	no	no	no	no	yes	yes
182206688	chr1	87072005	87072059	28	55	1.964286	no	no	yes	no	no	no	no	no	no	yes
182206689	chr1	87072237	87072289	25	53	2.160000	no	no	yes	no	no	no	no	no	no	yes
182206693	chr1	87084778	87084828	22	51	2.318182	no	no	yes	no	no	no	no	yes	no	yes
182206701	chr1	87106552	87106592	19	41	2.157895	no	yes	yes	no	no	no	no	no	no	yes
182206706	chr1	87127537	87127574	20	38	2.000000	no	no	yes	no	no	no	no	no	no	yes
182206707	chr1	87128474	87128506	16	33	2.062500	no	no	yes	no	no	no	no	no	no	yes
182206708	chr1	87133792	87133820	14	29	2.071429	no	yes	yes	no	no	yes	no	no	yes	yes
182206711	chr1	87158263	87158304	17	42	2.470588	no	no	yes	no	no	no	no	no	no	yes
182206712	chr1	87177699	87177731	17	33	1.941176	no	no	no	no	no	no	no	no	no	no
182206713	chr1	87180426	87180471	23	46	2.000000	no	no	no	no	no	no	no	yes	yes	no
182206721	chr1	87201158	87201200	22	43	2.000000	no	no	no	no	no	no	no	yes	yes	no
182206736	chr1	87260790	87260826	15	37	2.500000	no	no	yes	no	no	no	no	no	no	yes
182206745	chr1	87346746	87346777	15	32	2.200000	no	yes	no	no	no	no	no	yes	no	yes
182206746	chr1	87348234	87348281	24	48	2.000000	no	yes	no	no	no	no	no	yes	no	yes
182206749	chr1	87358124	87358169	21	46	2.190476	no	no	yes	no	no	no	no	yes	no	yes
182206755	chr1	87368063	87368105	21	43	2.095238	no	no	yes	no	no	no	no	no	no	yes
182206757	chr1	87370258	87370299	18	42	2.333333	no	no	yes	no	no	no	no	no	yes	yes
182206760	chr1	87384613	87384645	17	33	2.058824	no	no	no	no	no	no	no	no	no	no
182206766	chr1	87430647	87430694	20	48	2.380952	no	no	no	no	no	no	no	no	no	no
182206772	chr1	87442639	87442682	24	44	1.916667	no	no	no	no	no	no	no	no	no	no
182206777	chr1	87461016	87461062	15	47	3.133333	no	no	no	no	no	no	no	yes	no	no

182206788	chr1	87548504	87548568	28	65	2.464286	no	no	no	no	no	no	no	no	no	no
182206803	chr1	87592971	87593012	19	42	2.263158	no	no	no	no	no	no	no	no	no	no
182206811	chr1	87619082	87619120	20	39	1.950000	no	no	no	no	no	no	no	yes	no	no
182206820	chr1	87642280	87642329	23	50	2.173913	no	no	yes	no	no	no	no	yes	yes	yes
182206821	chr1	87647707	87647733	14	27	1.928571	no	no	yes	no	no	no	no	no	yes	yes
182206822	chr1	87651461	87651505	20	45	2.250000	no	no	yes	no	no	no	no	yes	no	yes
182206824	chr1	87658964	87659025	30	62	2.066667	no	no	yes	no	no	no	no	no	no	yes
182206826	chr1	87663796	87663827	12	32	2.666667	no	no	yes	no	no	no	no	no	no	yes
182206831	chr1	87679544	87679579	15	36	2.400000	no	no	yes	no	no	no	no	no	no	yes
182206834	chr1	87690614	87690653	17	40	2.352941	no	no	yes	no	no	no	no	no	no	yes
182206858	chr1	87764034	87764087	20	54	2.700000	no	no	yes	no	no	no	no	yes	no	yes
182206863	chr1	87791339	87791367	13	29	2.230769	no	no	yes	no	no	no	no	no	no	yes
182206864	chr1	87794273	87794305	17	33	1.941176	no	no	yes	no	no	no	no	yes	no	yes
182206866	chr1	87794691	87794731	20	41	2.050000	no	no	yes	no	no	no	no	yes	no	yes
182206872	chr1	87830385	87830425	20	41	2.050000	no	no	yes	no	no	no	no	yes	no	yes
182206880	chr1	87843592	87843636	20	45	2.250000	no	no	yes	no	no	no	no	no	no	yes
182206887	chr1	87855828	87855856	14	29	2.071429	no	no	yes	no	no	no	no	no	no	yes
182206889	chr1	87860822	87860853	17	32	1.941176	no	no	yes	no	no	no	no	no	no	yes
182206899	chr1	87872097	87872136	20	40	2.050000	no	no	yes	no	no	no	no	yes	no	yes
182206907	chr1	87880212	87880252	20	41	2.100000	no	no	yes	no	no	no	no	no	no	yes
182206914	chr1	87909469	87909505	19	37	2.000000	no	no	yes	no	no	no	no	no	no	yes
182206917	chr1	87918747	87918778	12	32	2.750000	no	no	yes	no	no	no	no	no	no	yes
182206920	chr1	87929168	87929220	25	53	2.120000	no	no	yes	no	no	no	no	no	no	yes
182206921	chr1	87932704	87932766	32	63	1.968750	no	no	yes	no	no	no	no	no	no	yes
182206930	chr1	87971293	87971334	19	42	2.210526	no	no	yes	no	no	no	no	no	no	yes

182206931	chr1	87971915	87971944	15	30	2.066667	no	no	yes	no	no	no	no	no	no	yes
182206933	chr1	87988305	87988400	34	96	2.823529	no	no	yes	no	no	no	no	no	yes	yes
182206937	chr1	88007766	88007837	31	72	2.387097	no	no	yes	no	no	no	no	no	no	yes
182206949	chr1	88042698	88042752	27	55	2.037037	no	no	yes	no	no	no	no	no	no	yes
182206950	chr1	88044018	88044050	14	33	2.357143	no	no	yes	no	no	no	no	yes	yes	yes
182206962	chr1	88093673	88093709	18	37	2.055556	no	no	yes	no	no	no	no	no	no	yes
182206965	chr1	88120185	88120238	27	54	2.000000	no	no	yes	no	no	no	no	no	no	yes
182206990	chr1	88190988	88191033	24	46	1.916667	no	no	yes	no	no	no	no	no	no	yes
182206994	chr1	88201616	88201647	14	32	2.357143	no	no	yes	no	no	no	no	no	no	yes
182207002	chr1	88246291	88246338	23	48	2.173913	no	no	yes	no	no	no	no	no	no	yes
182207005	chr1	88251939	88251976	17	38	2.352941	no	no	yes	no	no	no	no	no	no	yes
182207007	chr1	88253128	88253170	21	43	2.095238	no	no	yes	no	no	no	no	yes	no	yes
182207014	chr1	88279871	88280000	66	130	1.984848	no	no	yes	no	no	no	no	no	no	yes
182207030	chr1	88360446	88360579	68	134	1.985294	no	no	yes	no	no	no	no	no	no	yes
182207033	chr1	88374529	88374565	18	37	2.055556	no	no	yes	no	no	no	no	no	no	yes
182207042	chr1	88412917	88412947	16	31	1.937500	no	no	yes	no	no	no	no	yes	no	yes
182207043	chr1	88413172	88413207	18	36	2.111111	no	no	yes	no	no	no	no	no	no	yes
182207058	chr1	88440950	88441105	74	156	2.162162	no	no	yes	no	no	no	no	yes	no	yes
182207062	chr1	88447588	88447639	26	52	2.000000	no	no	yes	no	no	no	no	no	no	yes
182207065	chr1	88452769	88452797	15	29	1.933333	no	no	yes	no	no	no	no	no	no	yes
182207075	chr1	88469775	88469809	16	35	2.187500	no	no	yes	no	no	no	no	no	no	yes
182207076	chr1	88471536	88471573	18	38	2.166667	no	no	yes	no	no	yes	no	no	no	yes
182207081	chr1	88494038	88494092	28	55	2.000000	no	no	yes	no	no	no	no	no	no	yes
182207097	chr1	88570195	88570267	36	73	2.027778	no	no	yes	no	no	no	no	no	no	yes
182207098	chr1	88577238	88577272	17	35	2.058824	yes	no	yes	no	no	no	no	no	no	yes

182207103	chr1	88596297	88596321	11	25	2.272727	no	no	yes	no	no	no	no	no	no	yes
182207106	chr1	88596334	88596381	18	48	2.666667	no	no	yes	no	no	no	no	no	no	yes
182207111	chr1	88602832	88602881	22	50	2.272727	no	no	yes	no	no	no	no	no	no	yes
182207117	chr1	88604868	88604904	20	37	1.950000	no	no	yes	no	no	no	no	no	no	yes
182207119	chr1	88609803	88609853	27	51	1.925926	no	no	yes	no	no	no	no	no	no	yes
182207120	chr1	88609863	88609915	17	53	3.294118	no	no	yes	no	no	no	no	no	no	yes
182207123	chr1	88612419	88612454	9	36	4.000000	no	no	yes	no	no	no	no	yes	yes	yes
182207126	chr1	88628118	88628163	15	46	3.125000	no	no	yes	no	no	no	no	no	no	yes
182207138	chr1	88648087	88648129	23	43	1.913043	no	no	yes	no	no	no	no	no	no	yes
182207143	chr1	88670138	88670194	30	57	1.900000	no	no	yes	no	no	no	no	no	no	yes
182207145	chr1	88676240	88676299	31	60	1.967742	no	no	yes	no	no	no	no	no	no	yes
182207149	chr1	88684773	88684806	17	34	2.058824	no	no	yes	no	no	no	yes	yes	no	yes
182207150	chr1	88695269	88695294	10	26	2.600000	no	no	yes	no	no	no	no	yes	no	yes
182207157	chr1	88719735	88719782	24	48	2.000000	no	no	yes	no	no	no	no	no	no	yes
182207161	chr1	88743861	88743893	16	33	2.125000	no	no	yes	no	no	no	no	no	no	yes
182207163	chr1	88746068	88746122	8	55	7.250000	no	no	yes	no	no	no	no	no	no	yes
182207165	chr1	88751149	88751177	12	29	2.416667	no	no	yes	no	no	no	no	no	no	yes
182207170	chr1	88765615	88765667	26	53	2.038461	no	no	yes	no	no	no	no	no	no	yes
182207175	chr1	88788188	88788220	17	33	2.000000	no	no	yes	no	no	no	no	no	no	yes
182207178	chr1	88794924	88794976	27	53	1.962963	no	no	yes	no	no	no	no	no	no	yes
182207188	chr1	88828417	88828452	18	36	2.000000	yes	no	yes	no	no	no	no	no	no	yes
182207207	chr1	88875200	88875228	15	29	1.933333	no	no	yes	no	no	no	no	yes	no	yes
182207211	chr1	88891893	88891925	14	33	2.357143	yes	no	no	no	yes	no	no	yes	no	yes
182207233	chr1	88932385	88932434	16	50	3.250000	no	no	no	no	no	no	no	yes	no	no
182207235	chr1	88933384	88933422	20	39	1.950000	no	no	no	no	no	no	no	no	no	no

182207239	chr1	88951633	88951669	18	37	2.166667	no	no	yes	no	no	no	no	no	no	yes
182207240	chr1	88953650	88953687	20	38	1.950000	no	no	yes	no	no	no	no	no	no	yes
182207243	chr1	88955819	88955865	20	47	2.350000	no	no	yes	no	no	no	no	no	no	yes
182207244	chr1	88957654	88957701	23	48	2.086957	no	no	yes	no	no	no	no	no	no	yes
182207256	chr1	88978058	88978120	29	63	2.172414	no	no	yes	no	no	no	no	no	no	yes
182207261	chr1	88987537	88987574	19	38	2.000000	no	no	yes	no	no	no	no	no	no	yes
182207276	chr1	89006677	89006709	16	33	2.062500	no	yes	no	no	yes	yes	no	no	no	yes
182207281	chr1	89037730	89037844	57	115	2.017544	no	no	no	no	no	no	no	no	no	no
182207283	chr1	89040190	89040230	16	41	2.562500	no	no	no	no	no	no	no	yes	no	no
182207298	chr1	89070878	89070927	24	50	2.083333	no	no	no	no	no	no	no	no	no	no
182207320	chr1	89181018	89181104	34	87	2.558824	no	no	no	no	no	yes	no	no	no	no
182207322	chr1	89187295	89187347	20	53	2.619048	yes	no	yes	no	no	no	no	no	no	yes
182207324	chr1	89190434	89190471	20	38	2.000000	no	no	yes	no	no	no	no	yes	no	yes
182207325	chr1	89197401	89197474	26	74	2.793103	no	no	yes	no	no	no	no	no	no	yes
182207328	chr1	89202141	89202199	31	59	1.967742	no	no	yes	no	no	no	no	no	no	yes
182207337	chr1	89221708	89221746	19	39	2.052632	no	no	no	no	no	no	no	yes	no	no
182207338	chr1	89221909	89221974	33	66	2.000000	no	no	no	no	no	no	no	no	no	no
182207349	chr1	89257680	89257709	15	30	2.066667	no	no	no	no	no	no	no	no	no	no
182207350	chr1	89261652	89261695	21	44	2.142857	no	no	yes	no	no	no	no	no	yes	yes
182207351	chr1	89262882	89262922	16	41	2.687500	yes	no	yes	no	yes	no	no	yes	yes	yes
182207357	chr1	89279016	89279115	51	100	1.980392	no	no	no	no	no	no	no	no	yes	no
182207364	chr1	89295607	89295738	30	132	4.400000	no	no	no	no	no	no	no	yes	no	no
182207377	chr1	89358381	89358431	20	51	2.650000	no	no	no	no	no	no	no	yes	yes	no
182207409	chr1	89450418	89450466	23	49	2.130435	no	no	yes	no	no	no	no	no	no	yes
182207412	chr1	89455419	89455459	21	41	1.952381	no	no	yes	no	no	no	no	yes	yes	yes

182207413	chr1	89455555	89455623	34	69	2.088235	no	no	yes	no	no	no	no	yes	no	yes
182207417	chr1	89460802	89460839	20	38	1.900000	no	no	yes	no	no	no	no	yes	no	yes
182207419	chr1	89468906	89468952	20	47	2.350000	no	no	yes	no	no	no	no	no	no	yes
182207428	chr1	89504721	89504759	20	39	1.950000	no	no	yes	no	no	no	no	no	no	yes
182207430	chr1	89525483	89525530	20	48	2.400000	yes	no	yes	no	no	no	no	yes	yes	yes
182207434	chr1	89530974	89531010	19	37	1.947368	no	no	yes	no	no	no	no	yes	yes	yes
182207437	chr1	89540143	89540173	16	31	2.000000	no	no	yes	no	no	no	no	yes	no	yes
182207442	chr1	89548616	89548652	19	37	2.000000	no	no	yes	no	no	no	no	yes	yes	yes
182207443	chr1	89557734	89557784	24	51	2.125000	no	no	yes	no	no	no	no	no	no	yes
182207451	chr1	89568850	89568898	23	49	2.130435	no	no	yes	no	no	no	no	no	no	yes
182207455	chr1	89577819	89577862	22	44	2.000000	no	no	yes	no	no	no	no	yes	yes	yes
182207458	chr1	89603706	89603731	12	26	2.166667	no	no	yes	no	no	no	no	yes	no	yes
182207467	chr1	89637449	89637482	16	34	2.125000	no	no	yes	no	no	no	no	no	yes	yes
182207476	chr1	89671271	89671304	17	34	2.000000	no	no	yes	no	no	no	no	yes	no	yes
182207482	chr1	89685493	89685590	48	98	2.041667	no	no	yes	no	no	no	no	no	yes	yes
182207483	chr1	89693537	89693576	18	40	2.277778	no	no	yes	no	no	no	no	yes	yes	yes
182207499	chr1	89758748	89758793	20	46	2.350000	no	no	yes	no	no	no	no	yes	yes	yes
182207502	chr1	89763692	89763725	17	34	2.000000	no	yes	yes	no	no	no	no	yes	yes	yes
182207509	chr1	89772366	89772390	12	25	2.083333	no	no	yes	no	no	no	no	yes	yes	yes
182207515	chr1	89786369	89786399	15	31	2.066667	no	no	yes	no	no	no	no	no	yes	yes
182207523	chr1	89802357	89802394	20	38	1.900000	no	no	yes	no	no	no	no	no	yes	yes
182207529	chr1	89829459	89829508	22	50	2.363636	no	no	yes	no	no	no	no	yes	no	yes
182207530	chr1	89845218	89845259	20	42	2.150000	no	no	yes	no	no	no	no	no	no	yes
182207531	chr1	89849544	89849585	20	42	2.100000	no	no	yes	no	no	no	no	no	no	yes
182207532	chr1	89850931	89850969	16	39	2.562500	no	no	yes	no	no	no	no	yes	no	yes

182207542 chr1 89877595 89877648 27 54 2.000000 no no yes no no
no no yes yes yes
182207543 chr1 89880881 89880926 16 46 2.875000 no no yes no no
no no yes yes yes
182207544 chr1 89886898 89886930 15 33 2.266667 no no yes no no
no no no no yes
182207546 chr1 89896739 89896789 24 51 2.166667 no no yes no no
no no yes no yes
182207547 chr1 89900414 89900467 17 54 3.176471 no no yes no no
no no yes yes yes
182207566 chr1 89972748 89972785 16 38 2.500000 no no no no no
no no no no no
182207569 chr1 89979116 89979153 13 38 2.928571 no no no no no
no no no no no
182207581 chr1 89999338 89999372 18 35 1.944444 no no yes no no
no no yes yes yes
182207583 chr1 90017455 90017492 20 38 1.950000 no yes yes yes
no no no no no yes
182207587 chr1 90027443 90027486 15 44 2.933333 no yes no yes no
no no yes yes yes
182207601 chr1 90070398 90070450 18 53 2.894737 no no no no no
no no no no no
182207612 chr1 90099336 90099413 38 78 2.105263 no no no no no
no no no no no
182207619 chr1 90110642 90110688 18 47 2.666667 no no no no no
no no no no no
182207633 chr1 90162666 90162707 22 42 1.954545 no no no no no
no no no no no
182207641 chr1 90195395 90195430 10 36 3.545455 no no no no no
no no no no no
182207642 chr1 90196640 90196676 18 37 2.055556 no no no no no
no no no no no
182207643 chr1 90201007 90201035 15 29 2.000000 no no no no no
no no no yes no
182207660 chr1 90259676 90259713 11 38 3.454545 no no yes no no
no no no no yes
182207663 chr1 90262446 90262535 31 90 2.875000 no no yes no no
no no yes no yes
182207667 chr1 90273794 90273824 15 31 2.066667 no no yes no no
no no no yes yes
182207668 chr1 90274156 90274181 12 26 2.166667 no no yes no no
no no no no yes
182207679 chr1 90309950 90309990 19 41 2.157895 no no no no no
no no no no no
182207682 chr1 90325879 90325941 33 63 1.909091 no no no no no
no no no no no
182207690 chr1 90335453 90335485 16 33 2.062500 no no no no no
no no no no no
182207701 chr1 90378500 90379252 30 753 24.945946 no no no no
no no no no yes no

182207721	chr1	90428439	90428471	16	33	2.062500	no	no	no	no	no	no	no	no	no	no
182207722	chr1	90433607	90433634	12	28	2.333333	no	no	no	no	no	no	no	no	no	no
182207723	chr1	90434255	90434292	16	38	2.375000	no	no	no	no	no	no	no	no	no	no
182207724	chr1	90436640	90436666	13	27	2.076923	no	no	no	no	no	no	no	no	no	no
182207725	chr1	90437972	90438003	16	32	2.062500	no	no	no	no	no	no	no	no	no	no
182207727	chr1	90439913	90440007	31	95	3.064516	no	no	no	no	no	no	no	no	no	no
182207729	chr1	90442415	90442457	21	43	2.047619	no	no	no	no	no	no	no	no	no	no
182207738	chr1	90474628	90474668	20	41	2.050000	no	no	no	no	no	no	no	yes	no	no
182207742	chr1	90479309	90479390	30	82	2.733333	no	no	no	no	no	no	no	no	no	no
182207748	chr1	90498728	90498759	12	32	2.750000	no	no	no	no	no	no	no	no	no	no
182207749	chr1	90501922	90501967	24	46	1.916667	no	no	no	no	no	no	no	no	no	no
182207756	chr1	90527173	90527201	15	29	2.000000	no	no	yes	no	no	no	no	yes	yes	yes
182207757	chr1	90532923	90532992	36	70	1.944444	no	no	yes	no	no	no	no	yes	no	yes
182207759	chr1	90540421	90540453	12	33	2.750000	no	no	no	no	no	no	no	no	no	no
182207761	chr1	90543612	90543636	12	25	2.083333	no	no	no	no	no	no	no	no	no	no
182207766	chr1	90563571	90563630	17	60	3.421053	no	no	no	no	no	no	no	no	no	no
182207775	chr1	90604523	90604567	17	45	2.705882	no	no	no	no	no	no	no	no	no	no
182207805	chr1	90667593	90667644	26	52	2.000000	no	no	no	no	no	no	no	no	no	no
182207809	chr1	90671813	90671877	33	65	1.969697	no	no	no	no	no	no	no	no	no	no
182207825	chr1	90689982	90690010	13	29	2.230769	no	no	no	no	no	no	no	no	no	no
182207828	chr1	90700040	90700081	19	42	2.210526	no	no	no	no	no	no	no	no	no	no
182207829	chr1	90702658	90702692	16	35	2.187500	no	no	no	no	no	no	no	no	no	no
182207837	chr1	90716825	90716873	21	49	2.333333	no	no	no	yes	no	no	yes	yes	no	yes
182207844	chr1	90719905	90719946	19	42	2.263158	no	no	yes	no	no	no	yes	yes	yes	yes
182207849	chr1	90755941	90755984	19	44	2.368421	no	no	no	no	no	no	no	no	no	no

182207865	chr1	90811768	90811805	19	38	2.000000	no	no	yes	no	no	no	no	yes	yes	yes
182207879	chr1	90825964	90825998	17	35	2.058824	no	no	yes	no	no	no	no	yes	no	yes
182207884	chr1	90826222	90826336	50	115	2.288461	no	no	yes	no	no	no	no	yes	no	yes
182207889	chr1	90838475	90838552	22	78	3.727273	no	no	yes	no	no	no	no	no	no	yes
182207893	chr1	90847107	90847146	20	40	2.000000	no	no	yes	no	no	no	no	no	no	yes
182207907	chr1	90897452	90897484	17	33	2.000000	no	no	no	no	no	no	no	no	no	no
182207913	chr1	90932943	90932981	21	39	1.904762	no	no	yes	no	no	no	no	no	no	yes
182207917	chr1	90967524	90967562	19	39	2.052632	no	no	yes	no	no	no	no	no	no	yes
182207918	chr1	90968793	90968824	16	32	2.000000	no	no	yes	no	no	no	no	yes	no	yes
182207919	chr1	90973324	90973363	21	40	1.952381	no	no	yes	no	no	no	no	no	no	yes
182207920	chr1	90982793	90982825	16	33	2.062500	yes	no	yes	no	no	no	no	no	no	yes
182207927	chr1	91030404	91030449	23	46	2.000000	no	no	no	no	no	no	no	yes	no	no
182207933	chr1	91058955	91058998	20	44	2.200000	no	no	no	no	no	no	no	no	no	no
182207938	chr1	91075386	91075523	31	138	4.451613	no	no	no	no	no	no	no	yes	no	no
182207940	chr1	91078782	91078938	24	157	6.875000	no	no	no	no	no	no	no	no	no	no
182207950	chr1	91095523	91095591	30	69	2.290323	no	no	no	no	no	no	no	yes	no	no
182207958	chr1	91101008	91101042	18	35	2.000000	no	no	no	no	no	no	no	no	no	no
182207966	chr1	91130193	91130272	25	80	3.200000	no	no	no	no	no	no	no	no	no	no
182207974	chr1	91140691	91140744	22	54	2.454545	no	no	no	no	no	no	no	no	no	no
182207980	chr1	91154183	91154515	166	333	2.006024	no	no	no	no	no	no	no	no	no	no
182207987	chr1	91168562	91168732	28	171	6.250000	no	no	no	no	no	no	no	yes	no	no
182208005	chr1	91202251	91202282	17	32	1.941176	no	no	no	no	no	no	no	no	no	no
182208017	chr1	91236323	91236354	15	32	2.133333	no	no	no	no	no	no	no	no	no	no
182208018	chr1	91237020	91237062	21	43	2.047619	no	no	no	no	no	no	no	no	no	no
182208024	chr1	91261015	91261047	17	33	1.941176	yes	yes	no	no	yes	no	no	no	no	yes

182208026	chr1	91267260	91267290	16	31	1.937500	no	no	yes	no	no	no	no	no	no	yes
182208027	chr1	91272840	91272888	22	49	2.227273	no	no	yes	no	no	no	no	no	yes	yes
182208029	chr1	91276451	91276483	15	33	2.200000	no	no	yes	no	no	no	no	no	no	yes
182208052	chr1	91335596	91335704	37	109	2.945946	no	no	yes	no	no	no	no	yes	no	yes
182208054	chr1	91335972	91336181	88	210	2.382022	no	no	yes	no	no	no	no	yes	yes	yes
182208070	chr1	91351240	91351283	24	44	2.000000	no	no	yes	no	no	no	no	no	no	yes
182208074	chr1	91383976	91384007	13	32	2.461539	no	no	yes	no	no	no	no	no	no	yes
182208075	chr1	91384192	91384223	14	32	2.285714	no	no	yes	no	no	no	no	no	no	yes
182208078	chr1	91402856	91402892	19	37	1.947368	no	no	yes	no	no	no	no	no	no	yes
182208079	chr1	91403762	91403806	24	45	2.000000	no	no	yes	no	no	no	no	yes	no	yes
182208080	chr1	91403872	91403925	13	54	4.153846	no	no	yes	no	no	no	no	yes	no	yes
182208081	chr1	91404726	91404807	33	82	2.575758	yes	no	yes	no	yes	no	yes	yes	yes	yes
182208083	chr1	91408540	91408585	17	46	2.705882	no	no	no	no	no	no	no	yes	no	no
182208093	chr1	91437631	91437681	10	51	5.000000	no	no	no	no	no	no	no	no	no	no
182208107	chr1	91479976	91480013	20	38	1.950000	no	no	no	no	no	no	no	no	no	no
182208123	chr1	91549544	91549568	13	25	1.923077	no	no	no	no	no	no	no	no	yes	no
182208124	chr1	91549643	91549716	23	74	3.217391	no	no	no	no	no	no	no	no	no	no
182208126	chr1	91559532	91559600	16	69	4.312500	no	no	no	no	no	no	no	no	no	no
182208139	chr1	91578375	91578453	40	79	1.975000	no	no	no	no	no	no	no	yes	yes	no
182208141	chr1	91600795	91600922	64	128	2.000000	no	no	no	no	no	yes	no	yes	yes	no
182208144	chr1	91609295	91609360	29	66	2.275862	no	no	no	no	no	no	no	yes	no	no
182208145	chr1	91610296	91610329	11	34	2.923077	no	no	no	no	no	no	no	yes	yes	no
182208146	chr1	91612334	91612414	40	81	2.025000	no	no	no	no	no	no	no	no	yes	no
182208177	chr1	91717144	91717280	67	137	2.044776	no	no	yes	no	no	no	no	yes	no	yes
182208183	chr1	91728961	91729314	119	354	2.991597	no	no	yes	no	no	no	no	yes	no	yes

182208185	chr1	91729643	91729705	31	63	2.032258	no	no	yes	no	no	no	no	yes	yes	yes
182208187	chr1	91734079	91734103	8	25	3.125000	no	no	yes	no	no	no	no	yes	no	yes
182208193	chr1	91750881	91750933	25	53	2.120000	no	no	yes	no	no	no	no	no	no	yes
182208198	chr1	91778424	91778460	17	37	2.176471	no	no	yes	no	no	no	no	yes	no	yes
182208218	chr1	91848474	91848511	19	38	2.000000	no	no	yes	no	no	no	no	no	no	yes
182208229	chr1	91876096	91876125	11	30	2.727273	no	no	yes	no	no	no	no	yes	no	yes
182208230	chr1	91878669	91878695	14	27	1.928571	no	no	yes	no	no	no	no	yes	yes	yes
182208231	chr1	91879568	91879597	14	30	2.142857	no	no	yes	no	no	no	no	no	yes	yes
182208238	chr1	91886293	91886330	13	38	2.800000	yes	no	yes	no	no	no	yes	yes	yes	yes
182208239	chr1	91886897	91886946	26	50	1.923077	yes	no	yes	no	no	no	no	yes	yes	yes
182208241	chr1	91889289	91889367	39	79	2.025641	no	no	yes	no	no	no	no	no	yes	yes
182208244	chr1	91902591	91902628	20	38	1.900000	no	no	yes	no	no	no	no	yes	no	yes
182208247	chr1	91906439	91906476	20	38	1.900000	yes	no	no	no	no	no	no	yes	no	no
182208272	chr1	91925592	91925659	36	68	1.944444	no	no	no	no	no	no	no	yes	no	no
182208274	chr1	91925683	91925772	17	90	5.411765	no	no	no	no	no	no	no	yes	no	no
182208275	chr1	91925780	91925812	16	33	2.062500	no	no	no	no	no	no	no	yes	no	no
182208277	chr1	91927297	91927351	24	55	2.291667	no	no	no	no	no	no	no	yes	no	no
182208291	chr1	91939107	91939148	22	42	1.954545	no	no	no	no	no	no	no	no	no	no
182208296	chr1	91946786	91946825	16	40	2.500000	no	no	no	no	no	no	no	no	no	no
182208307	chr1	91969695	91969719	12	25	2.083333	no	no	yes	no	no	no	no	yes	no	yes
182208310	chr1	91984593	91984631	20	39	1.950000	no	no	yes	no	no	no	no	yes	no	yes
182208318	chr1	92004250	92004291	16	42	2.625000	no	no	yes	no	no	no	no	yes	no	yes
182208335	chr1	92032114	92032174	22	61	2.782609	yes	no	yes	no	no	no	no	no	yes	yes
182208339	chr1	92055617	92055645	15	29	1.933333	no	no	yes	no	no	no	no	no	no	yes
182208350	chr1	92078856	92078899	22	44	2.045455	no	no	no	no	no	no	no	no	yes	no

182208354	chr1	92089067	92089130	26	64	2.500000	no	no	yes	no	no	no	no	yes	no	yes
182208360	chr1	92103391	92103427	15	37	2.437500	no	no	yes	no	no	no	no	no	no	yes
182208370	chr1	92125647	92125693	23	47	2.130435	no	yes	yes	no	no	no	no	no	yes	yes
182208376	chr1	92136222	92136263	11	42	3.818182	no	no	yes	no	no	no	no	no	no	yes
182208379	chr1	92143927	92143959	16	33	2.062500	no	no	yes	no	no	no	no	no	no	yes
182208386	chr1	92168308	92168350	20	43	2.200000	yes	no	yes	no	no	no	no	no	no	yes
182208389	chr1	92183191	92183235	24	45	1.958333	no	no	yes	no	no	no	no	no	no	yes
182208398	chr1	92222300	92222394	26	95	3.678571	no	no	yes	no	no	no	no	no	no	yes
182208411	chr1	92250148	92250185	17	38	2.235294	no	no	yes	no	no	no	no	no	no	yes
182208416	chr1	92262333	92262379	19	47	2.473684	no	no	yes	no	no	yes	no	no	no	yes
182208417	chr1	92276246	92276289	23	44	1.913043	no	no	yes	no	no	no	no	no	no	yes
182208423	chr1	92310566	92310605	15	40	2.733333	no	no	yes	no	no	no	no	yes	no	yes
182208426	chr1	92316447	92316482	18	36	2.000000	no	no	yes	no	no	no	no	yes	no	yes
182208439	chr1	92356018	92356060	21	43	2.142857	no	no	yes	no	no	no	no	no	no	yes
182208440	chr1	92356116	92356158	22	43	2.045455	no	no	yes	no	no	no	no	no	no	yes
182208450	chr1	92386435	92386511	37	77	2.081081	no	no	yes	no	no	no	no	no	no	yes
182208485	chr1	92475937	92475978	22	42	1.909091	no	yes	no	no	no	no	no	yes	no	yes
182208492	chr1	92488466	92488503	20	38	1.900000	no	no	no	no	no	no	no	no	yes	no
182208493	chr1	92488895	92488951	24	57	2.400000	no	no	no	no	no	no	no	no	yes	no
182208501	chr1	92496053	92496090	19	38	2.052632	no	no	no	no	no	no	no	yes	no	no
182208515	chr1	92520887	92520967	40	81	2.025000	no	no	yes	no	no	no	no	no	no	yes
182208519	chr1	92526470	92526516	23	47	2.043478	no	no	yes	no	no	no	no	yes	no	yes
182208523	chr1	92529863	92529935	38	73	1.921053	no	no	yes	no	no	no	no	yes	no	yes
182208531	chr1	92555330	92555365	18	36	2.000000	no	no	yes	no	no	no	no	no	no	yes
182208543	chr1	92570871	92570953	42	83	1.976190	no	no	yes	no	no	no	no	no	no	yes

182208564	chr1	92639316	92639486	85	171	2.023530	no	no	yes	no	no	no	no	no	no	yes
182208582	chr1	92711418	92711457	20	40	2.000000	no	no	yes	no	no	no	no	yes	no	yes
182208596	chr1	92753730	92753773	13	44	3.384615	no	no	yes	no	no	no	no	no	yes	yes
182208612	chr1	92792654	92792703	27	50	1.925926	yes	no	no	no	no	no	no	yes	yes	no
182208619	chr1	92803842	92803872	16	31	1.937500	no	no	no	no	no	no	no	yes	no	no
182208633	chr1	92817189	92817226	18	38	2.111111	no	no	no	no	no	no	no	yes	no	no
182208643	chr1	92856737	92856769	16	33	2.187500	no	no	yes	no	no	no	no	yes	no	yes
182208651	chr1	92874159	92874189	16	31	1.937500	no	no	yes	no	no	no	no	no	no	yes
182208660	chr1	92912361	92912409	22	49	2.227273	no	no	yes	no	no	no	no	no	no	yes
182208669	chr1	92948625	92948792	91	168	1.901099	no	no	yes	no	no	no	no	no	no	yes
182208678	chr1	92967924	92967968	23	45	1.956522	no	no	no	no	no	no	no	no	no	no
182208679	chr1	92968964	92969012	24	49	2.083333	no	no	no	no	no	yes	no	yes	no	no
182208689	chr1	93000395	93000430	17	36	2.117647	no	no	no	no	no	no	no	yes	yes	no
182208690	chr1	93001994	93002031	20	38	1.900000	no	no	no	no	no	no	no	yes	yes	no
182208694	chr1	93005751	93005785	18	35	1.944444	no	no	no	no	no	no	no	no	no	no
182208715	chr1	93092317	93092356	20	40	2.000000	no	no	yes	no	no	no	no	no	no	yes
182208722	chr1	93117144	93117245	51	102	2.000000	no	no	yes	no	no	no	no	no	no	yes
182208729	chr1	93132972	93133014	23	43	1.956522	no	no	yes	no	no	no	no	no	no	yes
182208730	chr1	93143860	93143897	20	38	1.900000	no	no	no	no	no	no	no	yes	no	no
182208734	chr1	93151360	93151405	22	46	2.090909	no	yes	no	no	no	yes	no	yes	no	yes
182208735	chr1	93153361	93153398	13	38	2.800000	no	yes	no	no	no	yes	no	no	no	yes
182208745	chr1	93175370	93175408	20	39	1.950000	no	no	yes	no	no	no	no	no	no	yes
182208748	chr1	93184151	93184216	32	66	2.062500	no	no	yes	no	no	no	no	no	no	yes
182208749	chr1	93194411	93194451	8	41	5.250000	no	no	yes	no	no	yes	no	yes	no	yes
182208754	chr1	93197502	93197554	22	53	2.409091	no	no	yes	no	no	no	no	no	no	yes

182208757	chr1	93202431	93202464	13	34	2.615385	no	no	yes	no	no	no	no	no	no	yes
182208759	chr1	93207138	93207182	24	45	1.916667	no	no	no	yes	no	no	no	yes	no	yes
182208763	chr1	93231819	93231860	19	42	2.263158	no	no	yes	no	no	no	no	no	no	yes
182208773	chr1	93257266	93257300	16	35	2.250000	no	no	yes	no	no	no	no	no	no	yes
182208779	chr1	93258056	93258129	25	74	3.040000	no	no	yes	no	no	no	no	yes	no	yes
182208781	chr1	93260412	93260454	20	43	2.150000	no	no	yes	no	no	no	no	no	no	yes
182208782	chr1	93261586	93261654	35	69	2.000000	no	no	yes	no	no	yes	no	no	no	yes
182208783	chr1	93261827	93261860	8	34	4.375000	no	no	yes	no	no	yes	no	no	no	yes
182208784	chr1	93264899	93264933	19	35	1.947368	no	yes	yes	yes	no	no	no	no	no	yes
182208795	chr1	93298647	93298689	16	43	2.687500	no	no	yes	no	no	no	no	no	no	yes
182208810	chr1	93328047	93328100	27	54	2.037037	no	no	yes	no	no	no	no	no	no	yes
182208812	chr1	93333696	93333736	16	41	2.625000	yes	no	yes	no	no	no	no	no	no	yes
182208838	chr1	93415465	93415501	17	37	2.176471	no	no	no	no	no	no	no	no	yes	no
182208844	chr1	93420503	93420553	25	51	2.160000	no	no	no	no	no	no	no	no	no	no
182208867	chr1	93437415	93437443	15	29	1.933333	no	no	no	no	no	no	no	no	no	no
182208868	chr1	93443567	93443599	17	33	2.000000	no	no	no	no	no	no	no	yes	yes	no
182208873	chr1	93449251	93449306	24	56	2.333333	no	no	yes	no	no	no	no	yes	yes	yes
182208878	chr1	93457741	93457781	21	41	1.952381	no	no	yes	no	no	no	no	no	no	yes
182208885	chr1	93499933	93499961	15	29	1.933333	no	no	yes	no	no	no	no	yes	yes	yes
182208897	chr1	93530084	93530206	60	123	2.050000	no	no	yes	no	no	no	no	no	no	yes
182208899	chr1	93538113	93538167	26	55	2.153846	no	no	yes	no	no	no	no	yes	yes	yes
182208901	chr1	93561038	93561074	19	37	1.947368	no	no	no	no	no	yes	no	yes	no	no
182208906	chr1	93582210	93582301	45	92	2.044445	no	no	yes	yes	yes	yes	no	yes	yes	yes
182208911	chr1	93620210	93620243	16	34	2.125000	no	no	yes	no	no	no	no	yes	yes	yes
182208926	chr1	93670838	93670869	15	32	2.133333	no	no	yes	no	no	no	no	yes	yes	yes

182208927	chr1	93671454	93671482	15	29	1.933333	no	no	yes	no	no	no	no	yes	yes	yes
182208933	chr1	93694502	93694538	17	37	2.176471	no	no	yes	no	no	no	no	yes	no	yes
182208937	chr1	93724158	93724191	15	34	2.400000	no	no	yes	no	no	no	no	no	no	yes
182208942	chr1	93748645	93748740	48	96	2.000000	no	no	yes	no	no	no	no	no	no	yes
182208966	chr1	93827724	93827774	24	51	2.125000	no	no	yes	no	no	no	no	yes	no	yes
182208970	chr1	93842956	93843044	42	89	2.119048	no	no	yes	no	no	no	no	yes	yes	yes
182208973	chr1	93850130	93850192	26	63	2.423077	no	no	no	no	no	no	no	no	no	no
182208986	chr1	93892340	93892385	16	46	2.882353	no	no	yes	no	no	no	no	no	no	yes
182208987	chr1	93893641	93893682	21	42	2.047619	no	no	yes	no	no	yes	no	no	no	yes
182208989	chr1	93902097	93902135	17	39	2.352941	no	no	yes	no	no	no	no	no	no	yes
182208993	chr1	93909693	93909747	25	55	2.200000	yes	no	no	no	no	no	yes	yes	yes	no
182208996	chr1	93921492	93921524	17	33	2.058824	no	no	no	no	yes	no	no	no	yes	yes
182209007	chr1	93965905	93965934	13	30	2.307692	no	no	no	no	no	no	no	yes	yes	no
182209008	chr1	93972752	93972796	22	45	2.045455	no	no	no	no	no	no	no	no	no	no
182209013	chr1	93980423	93980474	22	52	2.363636	no	no	no	no	no	no	no	yes	yes	no
182209016	chr1	93984718	93984759	17	42	2.500000	no	no	no	no	no	no	no	no	no	no
182209021	chr1	93991118	93991160	18	43	2.500000	no	no	no	no	no	no	no	no	no	no
182209023	chr1	94006494	94006540	24	47	1.958333	no	no	yes	no	no	no	no	no	no	yes
182209028	chr1	94014212	94014392	98	181	1.887755	no	no	yes	no	no	no	no	yes	no	yes
182209044	chr1	94065739	94065892	77	154	2.000000	no	no	yes	no	no	no	no	no	no	yes
182209049	chr1	94093127	94093167	21	41	1.952381	no	no	yes	no	no	no	no	no	yes	yes
182209050	chr1	94095752	94095788	19	37	1.947368	no	no	yes	no	no	no	no	yes	yes	yes
182209053	chr1	94104950	94105018	30	69	2.300000	no	no	yes	no	no	no	no	yes	no	yes
182209057	chr1	94113648	94113680	12	33	2.750000	no	no	yes	no	no	no	no	no	yes	yes
182209059	chr1	94120875	94120919	13	45	3.538461	no	no	yes	no	no	no	no	yes	no	yes

182209066	chr1	94148005	94148040	18	36	2.000000	no	no	no	no	no	yes	no	no	no	no
182209070	chr1	94156859	94156890	15	32	2.133333	no	no	yes	no	no	no	no	no	no	yes
182209077	chr1	94185272	94185313	22	42	1.909091	no	no	yes	no	no	no	no	no	no	yes
182209083	chr1	94203212	94203249	20	38	1.900000	no	no	yes	no	no	no	no	no	no	yes
182209090	chr1	94221621	94221659	20	39	1.950000	no	no	yes	no	no	no	no	no	no	yes
182209092	chr1	94228264	94228292	15	29	2.000000	no	no	yes	no	no	no	no	no	no	yes
182209093	chr1	94228865	94228906	17	42	2.529412	no	no	yes	no	no	no	no	no	yes	yes
182209095	chr1	94237538	94237574	12	37	3.083333	no	no	yes	no	yes	no	yes	yes	yes	yes
182209106	chr1	94268791	94268830	22	40	1.909091	no	no	yes	no	no	no	no	no	no	yes
182209108	chr1	94280012	94280053	19	42	2.210526	no	no	yes	no	no	no	no	yes	no	yes
182209109	chr1	94282073	94282103	15	31	2.066667	no	no	yes	no	no	no	no	yes	yes	yes
182209112	chr1	94282609	94282641	17	33	1.941176	no	no	yes	no	no	no	no	no	no	yes
182209119	chr1	94295701	94295797	25	97	3.960000	no	no	yes	no	no	no	no	no	no	yes
182209126	chr1	94336178	94336215	19	38	2.000000	no	no	yes	no	no	yes	no	yes	no	yes
182209131	chr1	94344939	94345147	113	209	1.849558	no	no	yes	no	no	no	no	yes	yes	yes
182209167	chr1	94439659	94439696	20	38	1.950000	no	no	yes	no	no	no	no	no	no	yes
182209170	chr1	94451097	94451162	33	66	2.030303	no	no	yes	no	no	no	no	no	no	yes
182209176	chr1	94462182	94462292	55	111	2.054545	no	no	yes	no	no	no	no	yes	no	yes
182209177	chr1	94467097	94467126	13	30	2.307692	no	no	yes	no	no	no	no	no	no	yes
182209183	chr1	94506873	94506921	24	49	2.041667	no	no	yes	no	no	no	no	no	no	yes
182209185	chr1	94515829	94515874	17	46	2.705882	no	no	yes	no	no	no	no	yes	no	yes
182209190	chr1	94546983	94547042	29	60	2.068965	no	no	no	no	no	no	no	no	no	no
182209199	chr1	94582354	94582403	23	50	2.173913	no	no	no	no	no	no	no	no	yes	no
182209202	chr1	94585516	94585549	16	34	2.125000	no	no	no	no	no	yes	no	no	no	no
182209203	chr1	94588198	94588232	18	35	1.944444	no	no	yes	no	no	no	no	no	yes	yes

182209206	chr1	94596215	94596248	11	34	3.090909	no	no	no	no	no	no	no	yes	no	no
182209207	chr1	94601716	94601747	15	32	2.133333	no	no	no	no	no	no	no	no	yes	no
182209214	chr1	94635899	94635930	16	32	2.000000	no	no	yes	no	no	no	no	yes	no	yes
182209228	chr1	94684193	94684221	15	29	1.933333	no	no	yes	no	no	no	no	no	yes	yes
182209232	chr1	94697532	94697588	23	57	2.458333	no	no	yes	no	no	no	no	no	no	yes
182209237	chr1	94711168	94711199	12	32	2.666667	no	no	yes	no	no	no	no	no	no	yes
182209246	chr1	94748563	94748587	13	25	1.923077	no	no	yes	no	no	no	no	no	no	yes
182209250	chr1	94754469	94754510	17	42	2.444444	no	no	yes	no	no	no	no	no	no	yes
182209252	chr1	94766814	94766858	22	45	2.045455	no	no	yes	no	no	no	no	no	no	yes
182209260	chr1	94799969	94800034	33	66	2.030303	no	no	yes	no	no	no	no	no	no	yes
182209264	chr1	94824059	94824104	25	46	1.960000	yes	no	yes	no	no	no	no	no	no	yes
182209267	chr1	94849035	94849075	21	41	1.952381	no	no	yes	no	no	no	no	no	no	yes
182209275	chr1	94857516	94857552	13	37	2.923077	no	no	yes	no	no	no	no	yes	no	yes
182209284	chr1	94937028	94937052	13	25	1.923077	no	no	yes	no	no	no	no	yes	yes	yes
182209291	chr1	94946262	94946305	17	44	2.588235	no	no	yes	no	no	no	no	no	no	yes
182209299	chr1	94954201	94954247	24	47	1.958333	no	no	yes	no	no	no	no	no	yes	yes
182209303	chr1	94966439	94966480	21	42	2.000000	no	no	no	no	no	no	no	yes	no	no
182209304	chr1	94967866	94967892	13	27	2.076923	no	no	no	no	no	no	no	no	yes	no
182209306	chr1	94970446	94970529	46	84	1.913043	no	no	no	no	no	no	no	no	no	no
182209312	chr1	94985901	94985932	17	32	1.941176	no	no	yes	no	no	no	no	yes	yes	yes
182209316	chr1	94993233	94993357	65	125	1.984615	no	no	yes	no	no	no	no	no	no	yes
182209318	chr1	94993340	94993387	14	48	3.428571	no	no	yes	no	no	no	no	no	no	yes
182209328	chr1	95035197	95035239	16	43	2.812500	no	no	yes	no	no	yes	no	no	yes	yes
182209339	chr1	95045665	95045783	24	119	5.000000	no	no	yes	no	no	no	no	yes	no	yes
182209340	chr1	95045761	95045990	33	230	7.090909	no	no	yes	no	no	no	no	no	no	yes

182209342	chr1	95045982	95046031	23	50	2.173913	no	no	yes	no	no	no	no	no	no	yes
182209344	chr1	95049556	95049599	18	44	2.444444	no	no	yes	no	no	no	no	no	no	yes
182209380	chr1	95123134	95123167	18	34	1.944444	no	no	yes	no	no	no	no	yes	no	yes
182209389	chr1	95146978	95147011	18	34	1.944444	no	no	yes	no	no	no	no	yes	no	yes
182209392	chr1	95167269	95167336	34	68	2.000000	no	no	yes	no	no	no	no	yes	yes	yes
182209402	chr1	95184423	95184468	18	46	2.555556	no	no	yes	no	no	no	no	yes	no	yes
182209404	chr1	95186654	95186686	17	33	1.941176	no	no	yes	no	no	no	no	yes	no	yes
182209410	chr1	95226246	95226294	23	49	2.130435	no	no	yes	no	no	no	no	no	no	yes
182209411	chr1	95231472	95231506	15	35	2.333333	no	no	yes	no	no	no	no	no	no	yes
182209416	chr1	95242542	95242585	23	44	2.000000	no	no	yes	no	no	yes	no	yes	no	yes
182209417	chr1	95249836	95249985	39	150	3.846154	no	no	yes	no	no	no	no	yes	no	yes
182209423	chr1	95265225	95265509	148	285	1.932432	no	no	yes	no	no	no	no	no	yes	yes
182209425	chr1	95279457	95279599	29	143	4.931035	yes	no	no	no	no	no	no	no	no	no
182209426	chr1	95280078	95280122	22	45	2.045455	no	no	no	no	no	no	no	no	no	no
182209429	chr1	95304090	95304120	16	31	1.937500	no	no	no	no	no	no	no	no	no	no
182209437	chr1	95332886	95332914	14	29	2.071429	no	no	no	no	no	no	no	yes	yes	no
182209438	chr1	95332937	95332973	17	37	2.176471	no	no	no	no	no	no	no	yes	yes	no
182209441	chr1	95336230	95336286	31	57	1.935484	no	no	no	no	no	no	no	no	no	no
182209442	chr1	95346487	95346546	30	60	2.000000	no	no	no	no	no	no	no	no	no	no
182209449	chr1	95395768	95395794	14	27	1.928571	no	no	no	no	no	no	no	no	no	no
182209450	chr1	95396639	95396677	21	39	1.904762	no	no	no	no	no	no	no	yes	yes	no
182209460	chr1	95428425	95428478	27	54	2.037037	no	no	no	no	no	no	no	no	no	no
182209461	chr1	95431321	95431361	22	41	1.909091	no	no	no	no	no	no	no	yes	no	no
182209464	chr1	95436748	95436795	25	48	1.920000	no	no	no	no	no	no	no	no	no	no
182209470	chr1	95453974	95454026	10	53	5.083333	no	no	no	no	no	no	no	no	no	no

182209474	chr1	95472019	95472054	14	36	2.571429	no	no	no	no	no	no	no	no	no	no
182209477	chr1	95489596	95489809	106	214	2.018868	no	no	no	no	no	no	no	no	no	no
182209481	chr1	95513583	95513706	61	124	2.032787	no	no	yes	no	no	no	no	no	no	yes
182209484	chr1	95528354	95528413	25	60	2.400000	no	no	yes	no	no	no	no	no	no	yes
182209486	chr1	95534855	95534895	20	41	2.050000	no	no	yes	no	no	no	no	yes	no	yes
182209497	chr1	95586255	95586285	16	31	2.000000	no	no	yes	no	no	no	no	no	no	yes
182209505	chr1	95613680	95613731	24	52	2.166667	no	no	yes	no	no	no	no	no	no	yes
182209513	chr1	95633328	95633368	21	41	1.952381	no	no	yes	no	no	no	no	no	no	yes
182209514	chr1	95637223	95637268	18	46	2.555556	no	no	yes	no	no	no	no	yes	no	yes
182209539	chr1	95708184	95708231	25	48	1.920000	no	no	yes	no	no	no	no	no	no	yes
182209542	chr1	95728291	95728323	14	33	2.357143	no	no	yes	no	no	no	no	no	no	yes
182209543	chr1	95732005	95732053	25	49	2.000000	no	no	yes	no	no	no	no	no	no	yes
182209545	chr1	95732810	95732863	27	54	2.037037	no	no	yes	no	no	no	no	no	no	yes
182209550	chr1	95745706	95745754	19	49	2.578947	no	no	yes	no	no	no	no	yes	no	yes
182209557	chr1	95768551	95768583	13	33	2.538461	no	no	yes	no	no	no	no	no	no	yes
182209563	chr1	95781326	95781383	24	58	2.400000	no	no	yes	no	no	no	no	no	no	yes
182209570	chr1	95785524	95785563	18	40	2.277778	no	no	no	no	no	no	no	no	no	no
182209583	chr1	95820060	95820092	16	33	2.062500	no	no	no	no	no	no	no	no	no	no
182209607	chr1	95882126	95882155	15	30	2.066667	no	no	no	no	no	no	no	no	no	no
182209609	chr1	95889778	95889820	22	43	2.000000	no	no	no	no	no	no	no	yes	yes	no
182209618	chr1	95923022	95923062	21	41	1.952381	no	no	no	no	no	no	no	no	no	no
182209620	chr1	95943480	95943551	36	72	2.000000	no	no	yes	no	no	no	no	no	no	yes
182209636	chr1	95982541	95982611	35	71	2.028571	no	no	yes	no	no	no	no	no	no	yes
182209638	chr1	95988965	95989025	16	61	3.812500	no	no	yes	no	no	no	no	no	no	yes
182209641	chr1	95995150	95995185	15	36	2.400000	yes	no	yes	no	no	no	no	no	no	yes

182209644	chr1	96009767	96009799	16	33	2.125000	yes	no	yes	no	no	no	no	yes	yes	yes	
182209647	chr1	96014408	96014524	30	117	4.033333	no	no	yes	no	no	no	no	no	no	yes	
182209648	chr1	96017514	96017707	28	194	6.812500	no	no	yes	no	no	no	no	no	no	yes	
182209649	chr1	96017856	96017958	54	103	1.907407	no	no	yes	no	no	no	no	no	no	yes	
182209651	chr1	96021166	96021241	38	76	2.000000	no	no	yes	no	no	no	no	no	no	yes	
182209655	chr1	96043526	96043592	32	67	2.093750	no	no	no	no	no	no	no	no	no	no	
182209667	chr1	96109716	96109744	8	29	3.625000	no	no	no	no	no	no	no	no	no	no	
182209668	chr1	96111725	96111762	13	38	3.000000	no	no	no	no	no	no	no	no	no	no	
182209678	chr1	96134555	96134589	17	35	2.117647	no	no	no	no	no	no	no	no	no	no	
182209680	chr1	96136906	96136939	16	34	2.187500	no	no	no	no	no	no	no	no	no	no	
182209705	chr1	96162472	96162519	20	48	2.400000	no	no	no	no	no	no	no	no	no	no	
182209707	chr1	96165920	96165955	16	36	2.250000	no	no	no	no	no	no	no	no	no	no	
182209732	chr1	96204666	96204705	19	40	2.157895	no	no	no	no	no	no	no	no	no	no	
182209733	chr1	96212745	96212773	15	29	1.933333	no	no	no	no	no	no	no	no	no	no	
182209759	chr1	96282273	96282309	19	37	1.947368	no	no	yes	no	no	no	no	no	no	yes	
182209763	chr1	96303615	96303647	16	33	2.062500	no	no	yes	no	no	no	no	no	no	yes	
182209776	chr1	96351892	96351938	18	47	2.578947	no	no	yes	no	no	no	no	no	no	yes	
182209780	chr1	96354861	96354909	10	49	4.900000	no	no	yes	no	no	no	no	no	no	yes	
182209794	chr1	96405254	96405291	18	38	2.166667	no	no	no	no	no	no	no	no	no	no	
182209800	chr1	96424613	96424649	18	37	2.111111	no	no	no	no	no	no	no	no	no	no	
182209802	chr1	96427353	96427384	14	32	2.285714	no	no	no	no	no	no	no	no	no	no	
182209804	chr1	96441718	96441749	17	32	1.941176	no	no	no	no	no	no	no	no	no	no	
182209832	chr1	96534501	96534537	7	37	5.285714	no	no	no	no	no	no	no	no	yes	no	
182209835	chr1	96546119	96546165	24	47	1.958333	no	no	no	no	no	no	no	no	no	no	
182209837	chr1	96557274	96557308	17	35	2.058824	no	no	no	no	no	no	no	no	no	no	

182209840	chr1	96568415	96568511	32	97	3.031250	no	no	no	no	no	no	no	no	no	no
182209843	chr1	96578282	96578336	27	55	2.037037	no	no	no	no	no	no	no	no	no	no
182209848	chr1	96584077	96584119	17	43	2.705882	no	no	no	no	no	yes	no	yes	no	no
182209866	chr1	96636237	96636291	28	55	2.000000	no	no	no	no	no	no	no	no	no	no
182209867	chr1	96637299	96637352	24	54	2.250000	no	no	no	no	no	no	no	no	no	no
182209868	chr1	96642000	96642064	28	65	2.321429	no	no	no	no	no	no	no	no	no	no
182209870	chr1	96647012	96647053	18	42	2.333333	no	no	no	no	no	no	no	no	no	no
182209894	chr1	96704648	96704693	22	46	2.136364	no	no	no	no	no	no	no	no	no	no
182209908	chr1	96739190	96739240	18	51	2.833333	no	no	yes	no	no	no	no	no	no	yes
182209931	chr1	96764705	96764848	71	144	2.028169	no	no	yes	no	no	no	no	yes	yes	yes
182209933	chr1	96775880	96775945	22	66	3.045455	no	no	yes	no	no	no	no	no	no	yes
182209935	chr1	96778630	96778681	22	52	2.363636	no	no	yes	no	no	no	no	yes	no	yes
182209936	chr1	96786766	96786823	28	58	2.071429	no	no	yes	no	no	no	no	no	no	yes
182209938	chr1	96796746	96796770	12	25	2.083333	no	no	yes	no	no	no	no	yes	no	yes
182209941	chr1	96804838	96804868	12	31	2.583333	no	yes	no	yes	no	no	no	yes	yes	yes
182209944	chr1	96820279	96820318	20	40	2.000000	no	yes	no	no	no	no	no	yes	yes	yes
182209945	chr1	96821475	96821596	42	122	2.904762	no	yes	no	no	no	no	no	no	no	yes
182209958	chr1	96875542	96875580	20	39	2.000000	no	no	no	no	no	no	no	no	no	no
182209965	chr1	96894330	96894484	85	155	1.847059	no	no	no	no	no	no	no	no	no	no
182209974	chr1	96908125	96908187	11	63	5.909091	no	no	no	no	no	no	no	no	no	no
182209986	chr1	96948960	96948984	13	25	1.923077	no	no	no	no	no	no	no	no	no	no
182209991	chr1	96968974	96969014	21	41	1.952381	no	no	no	no	no	no	no	no	yes	no
182210011	chr1	97070235	97070278	23	44	1.956522	no	no	no	no	no	no	no	no	no	no
182210012	chr1	97072203	97072233	16	31	1.937500	no	no	no	no	no	no	no	no	no	no
182210013	chr1	97078836	97078876	21	41	1.952381	no	yes	no	no	no	no	no	no	no	yes

182210025	chr1	97116010	97116067	29	58	2.034483	no	no	yes	no	no	no	no	no	no	yes
182210031	chr1	97142833	97142864	12	32	2.615385	no	no	yes	no	no	no	no	no	no	yes
182210035	chr1	97148225	97148270	19	46	2.421053	no	no	yes	no	no	no	no	yes	yes	yes
182210041	chr1	97160013	97160048	17	36	2.117647	no	no	yes	no	no	no	no	no	no	yes
182210047	chr1	97173365	97173445	26	81	3.269231	no	no	yes	no	no	no	no	no	yes	yes
182210048	chr1	97173450	97173496	23	47	2.043478	no	no	yes	no	no	no	no	no	no	yes
182210051	chr1	97179692	97179738	25	47	2.000000	no	no	yes	no	no	no	no	no	no	yes
182210074	chr1	97228402	97228531	65	130	2.000000	no	no	yes	no	no	no	no	no	no	yes
182210075	chr1	97228801	97228845	23	45	2.000000	no	no	yes	no	no	no	no	no	no	yes
182210083	chr1	97236393	97236443	26	51	1.961538	no	no	yes	no	no	no	no	yes	no	yes
182210087	chr1	97243667	97243699	17	33	1.941176	no	no	yes	no	no	no	no	yes	no	yes
182210091	chr1	97255181	97255215	15	35	2.375000	no	no	yes	no	no	no	no	yes	yes	yes
182210100	chr1	97283604	97283637	15	34	2.266667	no	no	yes	no	no	no	no	no	no	yes
182210103	chr1	97308306	97308353	20	48	2.400000	no	no	yes	no	no	no	no	yes	yes	yes
182210109	chr1	97322417	97322453	18	37	2.111111	no	no	yes	no	no	no	no	no	no	yes
182210113	chr1	97330931	97330965	18	35	1.944444	no	no	yes	no	no	no	no	no	no	yes
182210117	chr1	97350307	97350345	18	39	2.166667	no	no	yes	no	no	no	no	no	yes	yes
182210127	chr1	97377684	97377727	22	44	2.090909	no	no	yes	no	no	no	no	yes	yes	yes
182210128	chr1	97381118	97381160	21	43	2.047619	no	no	yes	no	no	no	no	no	no	yes
182210143	chr1	97414086	97414142	29	57	2.034483	no	no	yes	no	no	no	no	no	no	yes
182210145	chr1	97415108	97415170	31	63	2.032258	no	no	yes	no	no	no	no	yes	yes	yes
182210146	chr1	97417296	97417338	18	43	2.388889	no	no	yes	no	no	no	no	no	no	yes
182210148	chr1	97424200	97424228	13	29	2.230769	no	no	yes	no	no	no	no	no	no	yes
182210150	chr1	97429853	97429890	15	38	2.533333	no	no	yes	no	no	no	no	no	no	yes
182210151	chr1	97436162	97436296	66	135	2.045455	no	no	yes	no	no	no	no	no	yes	yes

182210160	chr1	97460315	97460348	18	34	1.944444	no	no	yes	no	no	no	no	yes	no	yes
182210164	chr1	97475811	97475843	15	33	2.200000	no	no	yes	no	no	no	no	no	yes	yes
182210167	chr1	97485919	97485966	23	48	2.086957	no	no	yes	no	no	no	no	no	no	yes
182210171	chr1	97491064	97491116	28	53	1.964286	no	no	yes	no	no	no	no	no	no	yes
182210172	chr1	97491177	97491231	23	55	2.608696	no	no	yes	no	no	no	no	no	no	yes
182210176	chr1	97495521	97495558	18	38	2.111111	no	no	yes	no	no	no	no	no	no	yes
182210179	chr1	97499613	97499637	13	25	1.923077	no	no	yes	no	no	no	no	yes	no	yes
182210183	chr1	97505526	97505562	18	37	2.055556	no	no	yes	no	no	no	no	yes	no	yes
182210185	chr1	97511867	97511905	19	39	2.052632	no	no	yes	no	no	no	no	no	no	yes
182210189	chr1	97525790	97525999	113	210	1.867257	no	no	yes	no	no	no	no	yes	yes	yes
182210196	chr1	97539027	97539059	16	33	2.187500	no	no	yes	no	no	no	no	no	no	yes
182210198	chr1	97548117	97548150	15	34	2.266667	no	no	yes	no	no	no	no	no	no	yes
182210199	chr1	97553168	97553210	23	43	1.956522	no	no	yes	no	no	no	no	no	no	yes
182210212	chr1	97583605	97583641	19	37	2.000000	no	no	yes	no	no	no	no	no	no	yes
182210215	chr1	97585554	97585598	24	45	1.916667	yes	no	yes	no	no	no	no	no	no	yes
182210226	chr1	97610952	97611009	30	58	1.933333	no	no	yes	no	no	no	no	yes	no	yes
182210229	chr1	97612669	97612714	23	46	2.000000	no	no	yes	no	no	no	no	yes	no	yes
182210230	chr1	97613078	97613110	14	33	2.357143	no	no	yes	no	no	no	no	yes	no	yes
182210233	chr1	97618676	97618730	26	55	2.115385	no	no	yes	no	no	no	no	no	no	yes
182210234	chr1	97626294	97626333	21	40	1.952381	no	no	yes	no	no	no	no	yes	no	yes
182210235	chr1	97628802	97628837	12	36	3.000000	no	no	yes	no	no	no	no	no	no	yes
182210238	chr1	97636235	97636267	14	33	2.357143	no	no	yes	no	no	no	no	no	yes	yes
182210246	chr1	97659818	97659856	21	39	1.904762	no	no	yes	no	no	no	no	no	no	yes
182210250	chr1	97664294	97664349	24	56	2.416667	no	no	yes	no	no	no	no	no	no	yes
182210253	chr1	97665409	97665444	14	36	2.642857	no	no	yes	no	no	no	no	yes	no	yes

182210254	chr1	97668589	97668631	21	43	2.047619	no	no	yes	no	no	no	no	no	no	yes
182210255	chr1	97670994	97671041	15	48	3.200000	no	no	yes	no	no	no	no	yes	no	yes
182210257	chr1	97682986	97683026	22	41	1.954545	no	no	yes	no	no	no	no	no	no	yes
182210268	chr1	97702143	97702312	89	170	1.932584	no	no	yes	no	no	no	no	no	no	yes
182210271	chr1	97709349	97709449	53	101	1.924528	no	no	yes	no	no	no	no	no	no	yes
182210273	chr1	97718404	97718436	17	33	2.000000	no	no	yes	no	no	no	no	no	no	yes
182210278	chr1	97729193	97729229	18	37	2.055556	no	no	yes	no	no	no	no	no	no	yes
182210283	chr1	97747625	97747670	15	46	3.000000	no	no	yes	no	no	no	no	no	no	yes
182210288	chr1	97753808	97753855	22	48	2.181818	no	no	yes	no	no	no	no	no	no	yes
182210289	chr1	97768833	97768861	15	29	2.000000	no	no	yes	no	no	no	no	no	no	yes
182210290	chr1	97772930	97772967	19	38	2.000000	no	no	yes	no	no	no	no	no	no	yes
182210291	chr1	97776771	97776800	15	30	2.000000	no	no	yes	no	no	no	no	no	no	yes
182210308	chr1	97814990	97815033	19	44	2.368421	no	no	yes	no	no	no	no	no	no	yes
182210311	chr1	97818744	97818954	102	211	2.068627	no	no	yes	no	no	no	no	no	no	yes
182210314	chr1	97822386	97822429	19	44	2.350000	no	no	yes	no	no	no	no	no	no	yes
182210318	chr1	97828403	97828457	27	55	2.037037	no	no	yes	no	no	no	no	no	no	yes
182210320	chr1	97830759	97830791	13	33	2.500000	no	no	yes	no	no	no	no	no	no	yes
182210322	chr1	97843782	97843809	13	28	2.153846	no	no	yes	no	no	no	no	no	no	yes
182210323	chr1	97847083	97847141	21	59	2.809524	no	no	yes	no	no	no	no	no	no	yes
182210334	chr1	97928267	97928300	15	34	2.333333	no	no	yes	no	no	no	no	no	no	yes
182210357	chr1	98011185	98011210	13	26	2.000000	no	no	yes	no	no	no	no	no	no	yes
182210370	chr1	98046174	98046232	15	59	3.933333	yes	no	no	no	yes	no	no	yes	yes	yes
182210378	chr1	98057575	98057601	13	27	2.076923	no	no	no	no	no	no	no	no	no	no
182210379	chr1	98057706	98057757	16	52	3.312500	no	no	no	no	no	no	no	no	yes	no
182210394	chr1	98086481	98086610	47	130	2.978723	no	no	no	no	no	no	no	no	no	no

182210395	chr1	98091632	98091660	15	29	1.933333	no	no	no	no	no	no	no	no	no	no
182210400	chr1	98112620	98112660	9	41	4.666667	no	no	no	no	no	no	no	no	no	no
182210403	chr1	98121066	98121097	12	32	2.666667	no	no	no	no	no	no	no	yes	no	no
182210406	chr1	98130086	98130110	12	25	2.083333	no	no	no	no	no	no	no	no	no	no
182210409	chr1	98135464	98135492	14	29	2.071429	no	no	no	no	no	no	no	no	no	no
182210410	chr1	98143246	98143288	18	43	2.388889	no	no	no	no	no	no	no	no	no	no
182210412	chr1	98151835	98151906	36	72	2.000000	no	no	no	no	no	no	no	no	no	no
182210414	chr1	98166988	98167027	21	40	1.904762	no	no	no	no	no	no	no	no	no	no
182210415	chr1	98169961	98169993	17	33	1.941176	no	no	no	no	no	no	no	no	no	no
182210416	chr1	98179004	98179041	8	38	4.500000	no	no	no	no	no	no	no	no	no	no
182210422	chr1	98186357	98186423	32	67	2.093750	no	no	no	no	no	no	no	no	no	no
182210449	chr1	98283346	98283377	15	32	2.200000	no	no	no	no	no	no	no	no	no	no
182210477	chr1	98333557	98333721	82	165	2.012195	no	no	no	no	no	no	no	yes	yes	no
182210478	chr1	98334055	98334079	12	25	2.083333	no	no	no	no	no	no	no	yes	no	no
182210479	chr1	98337113	98337153	22	41	1.909091	no	no	no	no	no	no	no	yes	no	no
182210483	chr1	98357466	98357525	28	60	2.142857	no	no	no	no	no	no	no	no	no	no
182210516	chr1	98423765	98423816	24	52	2.250000	no	no	no	no	no	no	no	no	no	no
182210528	chr1	98456273	98456345	35	73	2.085714	no	no	no	no	no	no	no	no	no	no
182210533	chr1	98471595	98471630	17	36	2.117647	no	no	no	no	no	no	no	no	no	no
182210566	chr1	98530566	98530604	13	39	3.000000	no	no	no	no	no	no	no	no	no	no
182210572	chr1	98554971	98554997	11	27	2.454545	no	no	no	no	no	no	no	no	no	no
182210580	chr1	98597815	98597851	19	37	1.947368	no	no	no	no	no	no	no	no	no	no
182210581	chr1	98601040	98601068	12	29	2.416667	no	no	no	no	no	no	no	no	no	no
182210591	chr1	98633183	98633211	15	29	1.933333	no	no	no	no	no	no	no	no	no	no
182210615	chr1	98688866	98688895	14	30	2.142857	no	no	yes	no	no	no	no	yes	no	yes

182210619	chr1	98698036	98698076	15	41	2.800000	no	no	yes	no	no	no	no	no	no	yes
182210620	chr1	98701967	98702012	14	46	3.266667	no	no	yes	no	no	no	no	no	no	yes
182210622	chr1	98719891	98719935	20	45	2.350000	no	no	yes	no	no	no	no	no	no	yes
182210624	chr1	98719929	98719966	19	38	2.052632	no	no	yes	no	no	no	no	no	no	yes
182210625	chr1	98722673	98722866	97	194	2.000000	no	no	yes	no	no	no	no	no	no	yes
182210627	chr1	98732791	98732819	15	29	1.933333	no	no	yes	no	no	no	no	no	no	yes
182210630	chr1	98742508	98742578	34	71	2.088235	no	no	yes	no	no	no	no	no	no	yes
182210633	chr1	98749296	98749320	13	25	1.923077	no	no	yes	no	no	no	no	yes	yes	yes
182210636	chr1	98760300	98760347	20	48	2.400000	no	yes	no	no	no	no	no	no	no	yes
182210638	chr1	98772107	98772158	24	52	2.166667	no	no	no	no	no	no	no	no	no	no
182210645	chr1	98797961	98798005	15	45	2.937500	no	no	no	no	no	no	no	no	no	no
182210653	chr1	98817546	98817571	13	26	2.000000	no	no	no	no	no	no	no	no	no	no
182210655	chr1	98830548	98830580	12	33	2.750000	no	no	no	no	no	no	no	no	no	no
182210676	chr1	98885901	98885937	12	37	3.083333	no	no	no	no	no	no	no	no	no	no
182210681	chr1	98906202	98906239	15	38	2.533333	no	no	yes	no	no	no	no	no	no	yes
182210682	chr1	98906156	98906214	23	59	2.565217	no	no	yes	no	no	no	no	no	no	yes
182210689	chr1	98924884	98924918	18	35	1.944444	no	no	yes	no	no	no	no	no	no	yes
182210691	chr1	98931160	98931197	20	38	1.900000	no	no	yes	no	no	no	no	no	no	yes
182210696	chr1	98941993	98942051	26	59	2.269231	no	no	yes	no	no	no	no	no	no	yes
182210698	chr1	98942053	98942098	24	46	1.916667	no	no	yes	no	no	no	no	no	no	yes
182210699	chr1	98947573	98947615	20	43	2.150000	no	no	yes	no	no	no	no	no	no	yes
182210701	chr1	98955064	98955088	13	25	1.923077	no	no	yes	no	no	no	no	no	no	yes
182210702	chr1	98955713	98955773	31	61	1.967742	no	no	yes	no	no	no	no	no	no	yes
182210704	chr1	98973304	98973385	35	82	2.371428	no	no	yes	no	no	no	no	no	no	yes
182210705	chr1	98991397	98991422	12	26	2.166667	no	no	yes	no	no	no	no	yes	yes	yes

182210712	chr1	99017909	99018436	290	528	1.841379	no	no	yes	no	no	no	no	yes	yes	yes
182210720	chr1	99057448	99057488	20	41	2.050000	no	no	yes	no	no	no	no	no	no	yes
182210721	chr1	99060340	99060371	16	32	2.000000	no	no	yes	no	no	no	no	no	no	yes
182210726	chr1	99070123	99070151	15	29	1.933333	no	no	yes	no	no	no	no	no	no	yes
182210743	chr1	99084428	99084479	26	52	2.000000	no	no	yes	no	no	no	no	no	no	yes
182210758	chr1	99111785	99112120	178	336	1.960674	no	no	yes	no	no	no	no	yes	no	yes
182210761	chr1	99117394	99117465	36	72	2.000000	no	no	yes	no	no	no	no	no	no	yes
182210762	chr1	99117574	99117613	17	40	2.411765	no	no	yes	no	no	no	no	no	no	yes
182210763	chr1	99120166	99120203	16	38	2.375000	no	no	yes	no	no	no	no	no	no	yes
182210764	chr1	99130883	99130914	13	32	2.461539	no	no	yes	no	no	no	no	no	no	yes
182210769	chr1	99148545	99148580	18	36	2.000000	no	yes	yes	no	no	no	no	no	no	yes
182210781	chr1	99194312	99194337	12	26	2.166667	no	no	yes	no	no	no	no	no	no	yes
182210786	chr1	99212405	99212480	36	76	2.111111	no	no	yes	no	no	no	no	no	no	yes
182210791	chr1	99236824	99236860	9	37	4.111111	no	no	yes	no	no	no	no	yes	no	yes
182210792	chr1	99238500	99238706	104	207	2.009615	no	no	yes	no	no	no	no	no	no	yes
182210793	chr1	99249037	99249080	18	44	2.444444	no	no	no	no	no	no	no	no	no	no
182210798	chr1	99258926	99258982	29	57	2.000000	no	no	no	no	no	no	no	no	no	no
182210800	chr1	99268527	99268551	13	25	1.923077	no	no	yes	no	no	no	no	no	no	yes
182210803	chr1	99276183	99276207	12	25	2.083333	no	no	yes	no	no	no	no	no	no	yes
182210811	chr1	99284665	99284703	17	39	2.294118	no	no	yes	no	no	no	no	no	no	yes
182210812	chr1	99295504	99295552	23	49	2.130435	no	no	yes	no	no	no	no	no	no	yes
182210816	chr1	99317507	99317559	24	53	2.250000	no	no	no	no	no	no	no	no	no	no
182210829	chr1	99356348	99356398	25	51	2.040000	no	no	no	no	no	no	no	no	no	no
182210831	chr1	99373434	99373459	13	26	2.000000	no	no	no	no	no	no	no	yes	no	no
182210838	chr1	99377542	99377569	13	28	2.153846	no	no	no	no	no	no	no	no	yes	no

182210839	chr1	99377786	99377828	22	43	1.954545	no	no	no	no	no	no	no	no	no	no
182210867	chr1	99432872	99432911	19	40	2.105263	no	no	no	no	no	no	no	no	no	no
182210879	chr1	99493992	99494020	11	29	2.636364	yes	no	yes	no	no	no	no	yes	no	yes
182210880	chr1	99499565	99499597	17	33	1.941176	no	no	yes	no	no	no	no	no	no	yes
182210884	chr1	99502598	99502635	19	38	2.052632	no	no	yes	no	no	no	no	no	no	yes
182210895	chr1	99531448	99531496	10	49	4.900000	no	no	yes	no	no	no	no	no	no	yes
182210899	chr1	99548245	99548278	10	34	3.400000	no	no	yes	no	no	no	no	no	no	yes
182210900	chr1	99549832	99549891	30	60	2.000000	no	no	yes	no	no	no	no	no	no	yes
182210901	chr1	99553380	99553405	13	26	2.000000	no	no	yes	no	no	no	no	no	no	yes
182210912	chr1	99579678	99579718	15	41	2.812500	no	no	yes	no	no	no	no	yes	no	yes
182210925	chr1	99614141	99614181	12	41	3.500000	no	no	no	no	no	no	no	no	no	no
182210933	chr1	99623245	99623306	20	62	3.150000	no	no	no	no	no	no	no	no	no	no
182210937	chr1	99636444	99636495	20	52	2.600000	no	no	no	no	no	no	no	yes	no	no
182210938	chr1	99641778	99641806	13	29	2.230769	no	no	no	no	no	no	no	yes	no	no
182210940	chr1	99649471	99649502	15	32	2.133333	no	no	yes	no	no	no	no	yes	yes	yes
182210944	chr1	99654322	99654363	21	42	2.047619	no	no	yes	no	no	no	no	yes	no	yes
182210949	chr1	99659980	99660016	18	37	2.111111	no	no	yes	no	no	no	no	yes	yes	yes
182210950	chr1	99663593	99663623	12	31	2.666667	no	no	yes	no	no	no	no	no	no	yes
182210951	chr1	99666282	99666474	96	193	2.010417	no	no	yes	no	no	no	no	no	no	yes
182210967	chr1	99724868	99724959	31	92	2.857143	no	no	yes	no	no	no	no	no	no	yes
182210968	chr1	99725893	99725926	17	34	2.000000	no	no	yes	no	no	no	no	no	no	yes
182210969	chr1	99727262	99727309	20	48	2.380952	no	no	yes	no	no	no	no	yes	no	yes
182210971	chr1	99729075	99729164	43	90	2.116279	no	no	yes	no	no	no	no	yes	yes	yes
182210981	chr1	99762463	99762500	16	38	2.375000	no	no	yes	no	no	no	no	yes	no	yes
182210985	chr1	99776106	99776152	22	47	2.181818	no	no	no	no	no	no	no	yes	yes	no

182210993	chr1	99790783	99790821	20	39	1.950000	yes	no	no	no	no	no	no	yes	no	no
182211003	chr1	99811937	99811980	14	44	3.214286	no	no	no	no	no	no	no	yes	no	no
182211009	chr1	99815877	99815912	18	36	2.000000	no	no	no	no	no	no	no	no	yes	no
182211016	chr1	99822867	99822930	9	64	7.111111	no	no	no	no	no	no	no	no	no	no
182211030	chr1	99835180	99835218	20	39	1.950000	no	no	no	no	no	no	no	no	no	no
182211045	chr1	99869340	99869381	16	42	2.687500	no	no	yes	no	no	no	no	no	no	yes
182211046	chr1	99874500	99874529	10	30	3.000000	no	yes	yes	no	no	no	no	yes	no	yes
182211048	chr1	99891130	99891158	15	29	1.933333	no	no	yes	no	no	no	no	no	no	yes
182211049	chr1	99894524	99894561	20	38	1.900000	no	no	yes	no	no	no	no	no	no	yes
182211064	chr1	99922846	99922882	16	37	2.312500	no	yes	no	no	no	no	no	no	no	yes
182211083	chr1	99969892	99969932	21	41	2.000000	yes	no	no	no	yes	no	yes	yes	yes	yes
182211090	chr1	99988707	99988770	21	64	3.095238	no	no	yes	no	no	no	no	yes	no	yes
182211104	chr1	100018057	100018098	21	42	2.000000	no	no	yes	no	no	yes	no	no	no	yes
182211109	chr1	100033387	100033434	24	48	2.000000	no	yes	yes	no	no	no	no	no	no	yes
182211112	chr1	100041230	100041267	16	38	2.437500	no	no	yes	no	no	no	no	yes	no	yes
182211135	chr1	100082604	100082657	28	54	1.928571	no	yes	yes	no	no	yes	no	no	no	yes
182211136	chr1	100083890	100083943	26	54	2.076923	no	yes	yes	no	yes	yes	no	no	no	yes
182211141	chr1	100100269	100100298	8	30	3.750000	no	no	yes	no	no	yes	no	yes	no	yes
182211142	chr1	100109882	100109916	18	35	1.944444	no	no	yes	no	no	no	no	yes	no	yes
182211143	chr1	100110396	100110432	18	37	2.055556	no	no	no	yes	yes	no	no	no	no	yes
182211149	chr1	100119362	100119386	13	25	1.923077	no	no	yes	no	no	no	no	no	no	yes
182211151	chr1	100125273	100125306	14	34	2.428571	no	no	yes	no	no	no	no	no	no	yes
182211155	chr1	100138436	100138505	22	70	3.181818	no	no	yes	no	no	no	no	no	no	yes
182211183	chr1	100220983	100221016	17	34	2.000000	no	no	yes	no	no	no	no	no	no	yes
182211191	chr1	100238338	100238386	25	49	1.960000	no	no	yes	no	no	no	no	yes	no	yes

182211196	chr1	100238977	100239036	19	60	3.157895	no	no	yes	no	no	no	no	no	no	yes
182211200	chr1	100258115	100258168	26	54	2.076923	no	no	yes	no	no	yes	no	no	no	yes
182211202	chr1	100262155	100262218	14	64	4.642857	no	no	yes	no	yes	yes	no	no	no	yes
182211210	chr1	100292621	100292658	18	38	2.111111	no	yes	no	no	no	yes	no	yes	no	yes
182211211	chr1	100293230	100293260	16	31	1.937500	no	no	no	no	no	yes	no	no	no	no
182211215	chr1	100316471	100316506	17	36	2.117647	no	no	no	no	no	no	no	no	no	no
182211223	chr1	100332129	100332169	13	41	3.142857	no	no	no	no	yes	no	no	no	no	yes
182211225	chr1	100335815	100335855	17	41	2.529412	no	no	no	no	no	no	no	yes	no	no
182211231	chr1	100361102	100361155	23	54	2.391304	no	no	yes	no	no	no	no	yes	no	yes
182211240	chr1	100416153	100416193	18	41	2.263158	no	no	yes	no	no	no	no	no	yes	yes
182211247	chr1	100429688	100429788	32	101	3.187500	no	no	yes	no	no	no	no	no	no	yes
182211248	chr1	100430521	100430608	41	88	2.243902	no	no	yes	no	no	no	no	yes	yes	yes
182211249	chr1	100434268	100434302	18	35	1.944444	no	no	yes	no	no	no	no	yes	yes	yes
182211253	chr1	100445920	100445951	8	32	4.125000	no	no	yes	no	no	no	no	yes	yes	yes
182211256	chr1	100462275	100462308	18	34	1.944444	no	no	yes	no	no	yes	no	yes	yes	yes
182211258	chr1	100469091	100469142	25	52	2.080000	no	no	yes	no	no	no	no	no	no	yes
182211264	chr1	100491457	100491493	17	37	2.235294	no	no	yes	no	no	no	no	no	no	yes
182211272	chr1	100514899	100514937	18	39	2.166667	no	no	yes	no	no	no	no	no	no	yes
182211276	chr1	100530178	100530211	16	34	2.125000	no	no	no	no	no	no	no	yes	no	no
182211279	chr1	100545484	100545616	63	133	2.111111	no	no	no	no	no	no	no	yes	no	no
182211280	chr1	100546013	100546067	19	55	2.857143	no	no	no	no	no	no	no	no	no	no
182211298	chr1	100582138	100582168	8	31	3.875000	no	no	no	no	no	no	no	no	no	no
182211299	chr1	100582450	100582490	21	41	2.047619	no	no	no	no	no	no	no	no	no	no
182211302	chr1	100595192	100595408	108	217	2.009259	no	no	no	no	no	no	no	no	no	no
182211303	chr1	100596517	100596545	12	29	2.416667	no	no	no	no	no	no	no	no	no	no

182211305	chr1	100621787	100621823	19	37	1.947368	no	no	no	no	no	no	no	no	no	no
182211321	chr1	100684659	100684720	32	62	1.937500	no	no	no	no	no	no	no	no	no	no
182211325	chr1	100686643	100686713	38	71	1.921053	no	no	no	no	no	no	no	no	no	no
182211332	chr1	100687232	100687277	23	46	2.000000	no	no	no	no	no	no	no	no	no	no
182211342	chr1	100725115	100725162	24	48	2.000000	no	no	yes	no	no	no	no	no	no	yes
182211344	chr1	100729745	100729773	14	29	2.142857	no	no	yes	no	no	no	no	no	no	yes
182211345	chr1	100731035	100731064	16	30	1.937500	no	no	yes	no	no	no	no	yes	yes	yes
182211346	chr1	100737779	100737826	23	48	2.086957	no	yes	yes	no	no	no	no	no	yes	yes
182211358	chr1	100768865	100768922	27	58	2.222222	no	no	no	no	no	no	no	no	no	no
182211362	chr1	100788324	100788374	19	51	2.684211	no	no	no	no	no	no	no	no	no	no
182211364	chr1	100793341	100793371	15	31	2.133333	no	no	no	no	no	no	no	no	yes	no
182211377	chr1	100837291	100837331	19	41	2.315789	no	no	no	no	no	no	no	no	no	no
182211384	chr1	100853144	100853199	24	56	2.333333	no	no	no	no	no	no	no	yes	no	no
182211389	chr1	100860805	100860842	19	38	2.000000	no	no	no	no	no	no	no	yes	no	no
182211391	chr1	100870524	100870557	16	34	2.125000	no	no	no	no	no	no	no	yes	no	no
182211392	chr1	100872689	100872729	18	41	2.277778	no	yes	no	no	no	no	no	yes	yes	yes
182211393	chr1	100874979	100875034	27	56	2.148148	no	no	yes	no	no	no	no	no	no	yes
182211397	chr1	100898062	100898178	38	117	3.078947	no	no	yes	no	no	no	no	no	no	yes
182211398	chr1	100901955	100901984	13	30	2.307692	no	no	yes	no	no	no	no	no	yes	yes
182211399	chr1	100903061	100903095	17	35	2.117647	no	no	yes	no	no	no	no	no	no	yes
182211402	chr1	100920141	100920176	18	36	2.000000	no	no	yes	no	no	no	no	yes	yes	yes
182211405	chr1	100925899	100925933	17	35	2.058824	no	no	yes	no	no	no	no	no	yes	yes
182211406	chr1	100931570	100931598	13	29	2.230769	no	no	yes	no	no	no	no	yes	no	yes
182211408	chr1	100941336	100941367	12	32	2.666667	no	no	yes	no	yes	no	no	yes	yes	yes
182211409	chr1	100941766	100941833	32	68	2.125000	no	no	yes	no	yes	no	no	yes	no	yes

182211410	chr1	100954917	100954945	13	29	2.230769	no	no	yes	no	no	no	no	no	no	yes
182211416	chr1	100964223	100964394	95	172	1.873684	no	no	yes	no	no	no	no	no	no	yes
182211418	chr1	100975138	100975181	22	44	2.136364	no	yes	yes	no	no	no	no	no	no	yes
182211427	chr1	101001783	101001815	15	33	2.333333	no	no	yes	no	no	no	no	no	no	yes
182211428	chr1	101003682	101003722	17	41	2.470588	no	no	yes	no	no	no	no	yes	no	yes
182211437	chr1	101033322	101033380	16	59	3.555556	no	no	yes	no	no	no	no	no	no	yes
182211450	chr1	101072157	101072190	17	34	2.000000	yes	no	yes	no	no	no	no	yes	no	yes
182211455	chr1	101102203	101102238	18	36	2.000000	no	no	no	no	no	no	no	no	no	no
182211457	chr1	101105342	101105393	18	52	2.944444	no	no	no	no	no	no	no	no	no	no
182211485	chr1	101179505	101179541	15	37	2.533333	no	no	no	no	no	no	no	yes	no	no
182211494	chr1	101183188	101183228	22	41	2.000000	no	no	no	no	no	no	no	yes	no	no
182211519	chr1	101225004	101225170	72	167	2.373333	no	no	no	no	no	no	no	yes	no	no
182211527	chr1	101249049	101249098	23	50	2.173913	no	no	no	no	no	no	no	no	no	no
182211531	chr1	101253927	101253964	20	38	1.900000	no	no	no	no	no	no	no	yes	no	no
182211537	chr1	101262113	101262152	21	40	1.904762	no	no	no	no	no	no	no	yes	no	no
182211539	chr1	101274923	101274964	18	42	2.333333	no	no	no	no	no	no	no	no	no	no
182211540	chr1	101284994	101285022	15	29	1.933333	no	no	no	no	no	no	no	yes	yes	no
182211547	chr1	101292500	101292556	24	57	2.500000	no	no	no	no	no	no	no	yes	no	no
182211552	chr1	101325073	101325106	18	34	1.944444	no	no	yes	no	no	no	no	no	no	yes
182211555	chr1	101345701	101345744	13	44	3.285714	no	no	yes	no	no	no	no	no	no	yes
182211564	chr1	101369718	101369748	16	31	1.937500	no	no	yes	no	no	no	no	no	no	yes
182211570	chr1	101375085	101375123	15	39	2.600000	no	no	yes	no	no	no	no	yes	no	yes
182211577	chr1	101407616	101407669	24	54	2.291667	no	no	no	no	no	no	no	yes	no	no
182211579	chr1	101423038	101423074	14	37	2.642857	no	no	no	no	no	no	no	no	no	no
182211582	chr1	101423099	101423171	31	73	2.419355	no	no	no	no	no	no	no	no	no	no

182211625	chr1	101533190	101533255	31	66	2.129032	no	no	no	no	no	no	no	no	no	no
182211628	chr1	101544600	101544628	14	29	2.142857	no	no	no	no	no	no	no	no	no	no
182211638	chr1	101552392	101552561	82	170	2.156626	no	no	no	no	no	no	no	yes	no	no
182211646	chr1	101567180	101567260	43	81	1.930233	no	no	no	no	no	no	no	yes	no	no
182211649	chr1	101574017	101574064	25	48	1.920000	no	no	no	no	no	no	no	no	no	no
182211656	chr1	101619548	101619600	28	53	1.964286	no	no	no	no	no	no	no	no	no	no
182211660	chr1	101630322	101630375	28	54	1.928571	no	no	no	no	no	no	no	no	no	no
182211661	chr1	101637941	101637991	27	51	1.962963	no	no	no	no	no	no	no	no	no	no
182211689	chr1	101713010	101713066	18	57	3.277778	no	no	yes	no	no	no	no	no	no	yes
182211697	chr1	101730463	101730497	18	35	1.944444	no	no	yes	no	no	yes	no	no	no	yes
182211698	chr1	101731041	101731088	16	48	3.000000	no	yes	yes	no	no	no	no	no	no	yes
182211706	chr1	101776229	101776258	15	30	2.066667	no	no	yes	no	no	no	no	no	no	yes
182211708	chr1	101784174	101784241	34	68	2.000000	no	no	yes	no	no	no	no	no	no	yes
182211712	chr1	101796003	101796038	12	36	3.000000	no	no	no	no	no	no	no	no	no	no
182211713	chr1	101805657	101805801	79	145	1.873418	no	no	yes	no	no	no	no	no	no	yes
182211716	chr1	101826291	101826317	12	27	2.250000	no	no	yes	no	no	no	no	no	no	yes
182211720	chr1	101843716	101843752	19	37	1.947368	no	no	yes	no	no	no	no	no	no	yes
182211721	chr1	101843818	101843879	33	62	1.909091	no	no	yes	no	no	no	no	no	no	yes
182211725	chr1	101845142	101845194	17	53	3.117647	no	no	yes	no	no	no	no	no	no	yes
182211729	chr1	101857585	101857611	14	27	1.928571	no	no	yes	no	no	no	no	yes	no	yes
182211731	chr1	101867460	101867518	29	59	2.034483	no	no	yes	no	no	no	no	no	no	yes
182211733	chr1	101874470	101874508	20	39	1.950000	no	no	yes	no	no	no	no	no	no	yes
182211744	chr1	101887641	101887712	34	72	2.117647	no	no	yes	no	no	no	no	no	no	yes
182211752	chr1	101899883	101899951	35	69	1.971429	no	no	yes	no	no	no	no	no	no	yes
182211753	chr1	101901165	101901198	16	34	2.125000	no	no	yes	no	no	no	no	no	yes	yes

182211759	chr1	101940707	101940757	14	51	3.642857	no	no	yes	no	no	no	no	no	no	yes
182211763	chr1	101944072	101944105	17	34	2.000000	no	no	yes	no	no	no	no	no	no	yes
182211767	chr1	101948868	101948912	21	45	2.142857	no	no	yes	no	no	no	no	no	no	yes
182211771	chr1	101952283	101952407	64	125	2.000000	no	no	yes	no	no	no	no	no	no	yes
182211777	chr1	101994593	101994631	19	39	2.052632	no	no	yes	no	no	no	no	no	no	yes
182211778	chr1	101999841	101999878	20	38	1.900000	no	no	no	no	no	no	no	no	no	no
182211780	chr1	102005236	102005300	32	65	2.031250	no	no	no	no	no	no	no	no	no	no
182211783	chr1	102007295	102007335	21	41	1.952381	no	no	no	no	no	no	no	no	no	no
182211786	chr1	102029261	102029299	15	39	2.625000	no	no	no	no	no	no	no	no	no	no
182211787	chr1	102029284	102029325	19	42	2.263158	no	no	no	no	no	no	no	no	no	no
182211788	chr1	102034770	102034812	22	43	1.954545	no	no	no	no	no	no	no	no	no	no
182211793	chr1	102037824	102037865	16	42	2.625000	no	no	no	no	no	no	no	yes	no	no
182211805	chr1	102072762	102072843	41	82	2.000000	no	no	no	no	no	no	no	no	no	no
182211812	chr1	102093321	102093367	19	47	2.473684	no	no	no	no	no	no	no	no	no	no
182211816	chr1	102093919	102093948	15	30	2.000000	no	no	no	no	no	no	no	no	no	no
182211826	chr1	102116445	102116518	18	74	4.111111	no	no	no	no	no	no	no	no	no	no
182211829	chr1	102118573	102118646	26	74	2.851852	no	no	no	no	no	no	no	no	no	no
182211833	chr1	102138088	102138136	24	49	2.041667	no	no	no	no	no	no	no	no	no	no
182211834	chr1	102138252	102138304	27	53	1.962963	no	no	no	no	no	no	no	no	no	no
182211838	chr1	102148312	102148350	21	39	1.904762	no	no	no	no	no	no	no	no	no	no
182211840	chr1	102149007	102149036	15	30	2.000000	no	no	no	no	no	no	no	no	yes	no
182211841	chr1	102149265	102149302	13	38	2.857143	no	no	no	no	no	no	no	no	no	no
182211842	chr1	102154483	102154521	15	39	2.666667	no	no	no	no	no	no	no	no	no	no
182211844	chr1	102170795	102170827	9	33	3.700000	no	no	no	no	no	no	no	no	no	no
182211850	chr1	102171545	102171597	19	53	2.789474	no	no	no	no	no	no	no	no	no	no

182211855	chr1	102173458	102173505	12	48	4.000000	no	no	no	no	no	no	no	no	no	no
182211857	chr1	102181254	102181304	27	51	1.962963	no	no	no	no	no	no	no	no	no	no
182211879	chr1	102267204	102267237	16	34	2.125000	no	no	yes	no	no	no	no	no	no	yes
182211891	chr1	102291919	102291971	25	53	2.120000	no	no	yes	no	no	no	no	no	no	yes
182211903	chr1	102325239	102325267	13	29	2.230769	no	no	yes	no	no	no	no	no	no	yes
182211919	chr1	102377203	102377233	16	31	1.937500	no	no	yes	no	no	no	no	no	no	yes
182211922	chr1	102399063	102399092	15	30	2.000000	no	no	no	no	no	no	no	no	no	no
182211944	chr1	102467458	102467489	14	32	2.285714	no	no	no	no	no	no	no	no	no	no
182211946	chr1	102471984	102472017	16	34	2.125000	no	no	no	no	no	no	no	no	no	no
182211959	chr1	102494409	102494482	31	74	2.451613	no	no	no	no	no	no	no	no	no	no
182211960	chr1	102494468	102494524	25	57	2.320000	no	no	no	no	no	no	no	no	no	no
182211962	chr1	102497260	102497296	16	37	2.437500	no	no	no	no	no	no	no	no	no	no
182211963	chr1	102500152	102500185	18	34	1.944444	no	no	no	no	no	no	no	no	no	no
182211976	chr1	102548239	102548280	22	42	1.909091	no	no	no	no	no	no	no	no	no	no
182211978	chr1	102559120	102559151	14	32	2.285714	no	no	no	no	no	no	no	no	no	no
182211982	chr1	102578423	102578451	13	29	2.230769	no	no	no	no	no	no	no	no	no	no
182211987	chr1	102583383	102583558	24	176	7.230769	no	no	no	no	no	no	no	no	no	no
182212015	chr1	102638486	102638551	18	66	3.722222	no	no	no	no	no	no	no	no	no	no
182212019	chr1	102645434	102645573	67	140	2.089552	no	no	no	no	no	no	no	no	no	no
182212020	chr1	102649791	102649856	15	66	4.352941	no	no	no	no	no	no	no	no	no	no
182212029	chr1	102663899	102663952	29	54	1.965517	no	no	no	no	no	no	no	yes	no	no
182212039	chr1	102709819	102709855	19	37	2.052632	no	no	no	no	no	no	no	no	no	no
182212048	chr1	102718012	102718180	83	169	2.048193	no	no	no	no	no	no	no	no	no	no
182212057	chr1	102719053	102719111	19	59	3.157895	no	no	no	no	no	no	no	no	no	no
182212081	chr1	102754064	102754221	34	158	4.648649	no	no	no	no	no	no	no	no	no	no

182212092	chr1	102778292	102778329	17	38	2.235294	no	no	yes	no	no	no	no	no	no	yes
182212093	chr1	102785214	102785250	19	37	2.000000	no	no	yes	no	no	no	no	no	no	yes
182212106	chr1	102817977	102818020	22	44	2.090909	no	no	yes	no	no	no	no	no	no	yes
182212134	chr1	102888967	102889003	19	37	2.000000	no	no	yes	no	no	no	no	no	no	yes
182212136	chr1	102903803	102903866	32	64	2.000000	no	no	yes	no	no	no	no	no	no	yes
182212138	chr1	102905402	102905450	26	49	1.961538	no	no	yes	no	no	no	no	no	no	yes
182212140	chr1	102913274	102913320	24	47	2.000000	no	no	yes	no	no	no	no	no	no	yes
182212141	chr1	102914355	102914391	18	37	2.055556	no	yes	no	yes	no	no	no	no	no	yes
182212148	chr1	102940121	102940157	19	37	1.947368	no	no	yes	no	no	no	no	no	no	yes
182212152	chr1	102954194	102954234	22	41	1.909091	no	no	yes	no	no	no	no	no	no	yes
182212158	chr1	102982028	102982080	22	53	2.409091	no	no	yes	no	no	no	no	no	no	yes
182212159	chr1	102983094	102983157	29	64	2.233333	no	no	yes	no	no	no	no	no	no	yes
182212162	chr1	102998371	102998412	20	42	2.100000	no	no	yes	no	no	no	no	no	no	yes
182212166	chr1	103015748	103015788	18	41	2.388889	no	no	yes	no	no	no	no	no	no	yes
182212173	chr1	103028759	103028799	18	41	2.333333	no	no	yes	no	no	no	no	no	no	yes
182212174	chr1	103030542	103030578	15	37	2.466667	no	no	yes	no	no	yes	no	no	no	yes
182212181	chr1	103042919	103043271	78	353	4.535714	no	no	yes	no	no	no	no	no	no	yes
182212191	chr1	103071651	103071721	33	71	2.151515	no	no	yes	no	no	no	no	no	no	yes
182212194	chr1	103087199	103087227	15	29	1.933333	no	no	yes	no	no	no	no	no	no	yes
182212196	chr1	103091099	103091131	17	33	2.000000	no	no	yes	no	no	no	no	no	no	yes
182212199	chr1	103106637	103106665	13	29	2.230769	no	no	yes	no	no	no	no	yes	yes	yes
182212200	chr1	103108385	103108417	14	33	2.357143	yes	no	yes	no	yes	no	no	yes	no	yes
182212208	chr1	103155181	103155226	22	46	2.090909	no	no	no	no	no	no	no	no	no	no
182212217	chr1	103167086	103167119	10	34	3.272727	no	no	no	no	no	no	no	no	no	no
182212220	chr1	103168219	103168302	34	84	2.470588	no	no	no	no	no	no	no	no	no	no

182212224	chr1	103172906	103172954	25	49	1.960000	no	no	no	no	no	no	no	no	no	no
182212228	chr1	103173458	103173488	10	31	3.100000	no	no	no	no	no	no	no	no	no	no
182212234	chr1	103182138	103182988	34	851	25.117647	no	no	no	no	no	no	no	no	no	no
182212237	chr1	103183381	103183407	12	27	2.250000	no	no	no	no	no	no	no	no	no	no
182212241	chr1	103188815	103188890	39	76	1.948718	no	no	no	no	no	no	no	no	no	no
182212246	chr1	103208660	103208711	28	52	1.928571	no	no	no	no	no	no	no	no	no	no
182212250	chr1	103219868	103219988	40	121	3.025000	no	no	no	no	no	no	no	no	yes	no
182212262	chr1	103234324	103234365	20	42	2.100000	no	no	no	no	no	no	no	no	no	no
182212263	chr1	103235226	103235264	12	39	3.071429	no	no	no	no	no	no	no	no	no	no
182212270	chr1	103261657	103261710	26	54	2.076923	no	no	no	no	no	no	no	no	no	no
182212271	chr1	103265416	103265453	20	38	1.900000	no	no	no	no	no	no	no	no	no	no
182212274	chr1	103274715	103274774	27	60	2.296296	no	no	no	no	no	no	no	no	no	no
182212284	chr1	103303862	103303886	13	25	1.923077	no	no	no	no	no	no	no	no	no	no
182212291	chr1	103317362	103317395	18	34	1.944444	no	no	no	no	no	no	no	no	no	no
182212295	chr1	103320207	103320290	38	84	2.210526	no	no	no	no	no	no	no	no	no	no
182212305	chr1	103350550	103351357	13	808	61.190475	no	no	no	no	no	no	no	no	no	no
182212311	chr1	103361745	103361802	24	58	2.384615	no	no	no	no	no	no	no	no	no	no
182212318	chr1	103363194	103363293	36	100	2.777778	no	no	no	no	no	no	no	no	yes	no
182212339	chr1	103414775	103414862	45	88	1.977778	yes	no	no	no	no	no	no	no	yes	no
182212340	chr1	103417334	103417375	22	42	1.954545	no	no	yes	no	yes	no	no	yes	no	yes
182212342	chr1	103428382	103428410	11	29	2.636364	no	no	yes	no	no	no	no	no	no	yes
182212343	chr1	103430288	103430333	24	46	1.958333	no	no	yes	no	no	no	no	no	no	yes
182212349	chr1	103440986	103441028	16	43	2.687500	no	no	yes	no	no	no	no	no	no	yes
182212352	chr1	103453045	103453069	12	25	2.083333	no	no	yes	no	no	no	no	no	no	yes
182212353	chr1	103453143	103453190	26	48	1.923077	no	no	yes	no	no	no	no	no	no	yes

182212357	chr1	103457605	103457636	12	32	2.666667	no	no	yes	no	no	no	no	yes	no	yes
182212364	chr1	103471129	103471170	16	42	2.625000	no	no	yes	no	no	no	no	no	no	yes
182212367	chr1	103471383	103471438	28	56	2.071429	no	no	yes	no	no	no	no	no	no	yes
182212368	chr1	103482647	103482687	18	41	2.277778	no	no	yes	no	no	no	no	no	no	yes
182212370	chr1	103505816	103505985	86	170	2.011628	no	no	yes	no	no	no	no	no	no	yes
182212374	chr1	103525498	103525672	17	175	10.294118	yes	no	no	no	yes	no	yes	yes	yes	yes
182212376	chr1	103534928	103535007	37	80	2.216216	no	no	yes	no	no	no	no	no	yes	yes
182212387	chr1	103557151	103557196	8	46	5.750000	no	no	yes	no	no	no	no	no	yes	yes
182212389	chr1	103557854	103557898	24	45	1.916667	no	no	yes	no	no	no	no	no	no	yes
182212390	chr1	103561573	103561624	17	52	3.000000	yes	no	yes	no	no	no	no	no	no	yes
182212394	chr1	103568789	103568866	30	78	2.580645	yes	yes	yes	no	no	no	no	no	no	yes
182212408	chr1	103621109	103621154	24	46	1.916667	yes	no	yes	no	no	yes	no	no	no	yes
182212441	chr1	103766168	103766231	26	64	2.500000	no	no	no	no	no	no	no	no	no	no
182212444	chr1	103777314	103777397	42	84	2.047619	no	no	no	no	no	no	no	no	no	no
182212445	chr1	103778797	103778846	26	50	2.000000	no	no	no	no	no	no	no	no	no	no
182212448	chr1	103782461	103782494	15	34	2.266667	no	no	no	no	no	no	no	no	no	no
182212449	chr1	103785743	103786017	139	275	1.978417	no	no	no	no	no	no	no	no	no	no
182212452	chr1	103787771	103787815	16	45	2.764706	no	no	no	no	no	no	no	no	no	no
182212453	chr1	103791349	103791386	19	38	2.000000	no	no	no	no	no	no	no	no	no	no
182212467	chr1	103839551	103839593	20	43	2.200000	no	no	no	no	no	no	no	no	no	no
182212473	chr1	103864276	103864319	14	44	3.066667	no	no	no	no	no	no	no	no	no	no
182212505	chr1	103979483	103979525	22	43	1.954545	no	no	no	no	no	no	no	no	no	no
182212506	chr1	103979977	103980009	17	33	1.941176	no	no	no	no	no	no	no	no	no	no
182212507	chr1	103983880	103983914	16	35	2.250000	no	no	no	no	no	no	no	no	no	no
182212508	chr1	103984937	103984967	15	31	2.066667	no	no	no	no	no	no	no	no	no	no

182212510	chr1	103992063	103992169	53	107	2.018868	no	no	no	no	no	no	no	yes	yes	no
182212512	chr1	103999546	103999587	22	42	2.000000	no	no	no	no	no	no	no	no	no	no
182212523	chr1	104036672	104036728	23	57	2.478261	no	no	no	no	no	no	no	no	no	no
182212533	chr1	104064917	104064970	28	54	1.928571	no	no	no	no	no	no	no	no	no	no
182212544	chr1	104120054	104120100	19	47	2.473684	no	no	yes	no	no	no	no	no	no	yes
182212547	chr1	104126802	104127550	248	749	3.020161	no	yes	yes	no	no	yes	no	no	no	yes
182212548	chr1	104128550	104128589	20	40	2.050000	no	no	yes	no	no	no	no	yes	no	yes
182212551	chr1	104134423	104134455	15	33	2.200000	no	no	yes	no	no	no	no	no	no	yes
182212554	chr1	104141042	104141082	21	41	1.952381	no	no	yes	no	no	no	no	no	no	yes
182212568	chr1	104165261	104165482	113	222	2.026549	no	no	yes	no	no	no	no	no	no	yes
182212569	chr1	104168713	104168753	16	41	2.562500	no	no	yes	no	no	no	no	no	no	yes
182212576	chr1	104196919	104197015	45	97	2.155555	no	no	yes	no	no	no	no	no	no	yes
182212591	chr1	104223426	104223473	24	48	2.000000	no	no	yes	no	no	no	no	no	no	yes
182212597	chr1	104244571	104244632	31	62	2.000000	no	no	no	no	no	no	no	no	no	no
182212598	chr1	104249057	104249316	144	260	1.833333	no	no	no	no	no	no	no	no	no	no
182212601	chr1	104256324	104256361	20	38	1.900000	no	no	no	no	no	no	no	yes	no	no
182212602	chr1	104256361	104256409	23	49	2.130435	no	no	no	no	no	no	no	yes	no	no
182212619	chr1	104318736	104318764	14	29	2.071429	no	no	no	no	no	no	no	no	no	no
182212620	chr1	104319656	104319680	13	25	1.923077	no	no	no	no	no	no	no	no	no	no
182212631	chr1	104354434	104354470	16	37	2.312500	no	no	no	no	no	no	no	no	no	no
182212640	chr1	104403758	104403791	17	34	2.000000	no	no	no	no	no	no	no	no	no	no
182212642	chr1	104403789	104403842	23	54	2.347826	no	no	no	no	no	no	no	no	no	no
182212643	chr1	104403848	104403884	19	37	1.947368	no	no	no	no	no	no	no	no	no	no
182212645	chr1	104405462	104405494	17	33	1.941176	no	no	no	no	no	no	no	no	no	no
182212676	chr1	104497169	104497206	16	38	2.375000	no	no	no	no	no	no	no	no	no	no

182212712	chr1	104567060	104567111	16	52	3.312500	no	no	no	no	no	no	no	no	no	no
182212714	chr1	104570995	104571035	21	41	2.047619	no	no	no	no	no	no	no	no	no	no
182212721	chr1	104605087	104605130	12	44	3.666667	no	no	no	no	no	no	no	no	no	no
182212736	chr1	104651999	104652046	25	48	1.920000	no	no	no	no	no	no	no	no	no	no
182212738	chr1	104652630	104652700	28	71	2.535714	no	no	no	no	no	no	no	no	no	no
182212741	chr1	104675011	104675041	16	31	1.937500	no	no	no	no	no	no	no	no	no	no
182212746	chr1	104684802	104684834	15	33	2.266667	no	no	no	no	no	no	no	no	no	no
182212750	chr1	104700536	104700592	27	57	2.185185	no	no	no	no	no	no	no	no	no	no
182212756	chr1	104705095	104705248	77	154	2.000000	no	no	no	no	no	no	no	no	no	no
182212761	chr1	104712387	104712471	30	85	2.900000	no	no	no	no	no	no	no	no	no	no
182212777	chr1	104758067	104758108	12	42	3.428571	no	no	no	no	no	no	no	yes	yes	no
182212780	chr1	104758217	104758271	26	55	2.153846	no	no	no	no	no	no	no	yes	no	no
182212785	chr1	104769718	104769899	101	182	1.841584	no	no	no	no	no	no	no	no	no	no
182212788	chr1	104780418	104780459	15	42	2.800000	no	no	no	no	no	no	no	yes	no	no
182212798	chr1	104808736	104808840	34	105	3.088235	no	no	no	no	no	no	no	no	no	no
182212803	chr1	104824903	104824959	27	57	2.111111	no	no	no	no	no	no	no	no	no	no
182212810	chr1	104854933	104855003	28	71	2.535714	no	no	no	no	no	no	no	no	no	no
182212816	chr1	104861211	104861294	41	84	2.073171	no	no	no	no	no	no	no	no	no	no
182212824	chr1	104874183	104874242	30	60	2.000000	no	no	no	no	no	no	no	no	yes	no
182212841	chr1	104909449	104909493	24	45	1.916667	no	no	no	no	no	no	no	no	no	no
182212843	chr1	104910457	104910493	18	37	2.055556	no	no	no	no	no	no	no	no	no	no
182212848	chr1	104915164	104915205	19	42	2.263158	no	no	no	no	no	no	no	no	no	no
182212849	chr1	104921886	104921937	27	52	2.000000	no	no	no	no	no	no	no	yes	no	no
182212852	chr1	104943094	104943133	15	40	2.625000	no	no	no	no	no	no	no	no	no	no
182212855	chr1	104949383	104949416	12	34	2.769231	no	no	no	no	no	no	no	no	no	no

182212858	chr1	104953343	104953495	77	153	2.000000	no	no	no	no	no	no	no	no	no	no
182212859	chr1	104955721	104955752	11	32	2.909091	no	no	no	no	no	no	no	no	no	no
182212864	chr1	104967021	104967153	29	133	4.586207	no	no	no	no	no	no	no	yes	no	no
182212877	chr1	105000375	105000427	27	53	1.962963	no	no	yes	no	no	no	no	no	yes	yes
182212882	chr1	105006462	105006497	15	36	2.400000	no	no	yes	no	no	no	no	no	no	yes
182212892	chr1	105019990	105020018	15	29	1.933333	no	no	yes	no	no	no	no	no	no	yes
182212896	chr1	105024640	105024676	17	37	2.176471	no	no	yes	no	no	no	no	no	no	yes
182212908	chr1	105079860	105079895	19	36	1.947368	no	no	no	no	no	no	no	no	no	no
182212914	chr1	105085026	105085105	21	80	3.826087	no	no	no	no	no	no	no	no	no	no
182212918	chr1	105089859	105089897	19	39	2.052632	no	no	no	no	no	no	no	no	no	no
182212929	chr1	105108616	105108685	34	70	2.058824	no	no	no	no	no	no	no	no	no	no
182212946	chr1	105122025	105122072	23	48	2.086957	no	no	no	no	no	no	no	no	no	no
182212963	chr1	105182072	105182116	16	45	2.812500	no	no	no	no	no	no	no	no	no	no
182212964	chr1	105184062	105184100	19	39	2.052632	no	no	no	no	no	no	no	no	no	no
182212971	chr1	105222958	105223000	22	43	2.000000	no	no	no	no	no	no	no	no	no	no
182212979	chr1	105247355	105247387	17	33	1.941176	no	no	no	no	no	no	no	no	no	no
182212996	chr1	105276049	105276095	22	47	2.136364	no	no	no	no	no	no	no	no	no	no
182212998	chr1	105281143	105281181	19	39	2.052632	no	no	no	no	no	no	no	no	no	no
182213002	chr1	105284653	105284684	15	32	2.133333	no	no	no	no	no	no	no	no	no	no
182213018	chr1	105336142	105336207	35	66	1.942857	no	no	no	no	no	no	no	no	no	no
182213021	chr1	105336259	105336318	29	60	2.137931	no	no	no	no	no	no	no	no	no	no
182213032	chr1	105353598	105353639	14	42	3.214286	no	no	no	no	no	no	no	no	no	no
182213050	chr1	105376399	105376453	16	55	3.562500	no	no	no	no	no	no	no	no	no	no
182213053	chr1	105387788	105387826	16	39	2.437500	no	no	no	no	no	no	no	no	no	no
182213063	chr1	105414865	105414900	17	36	2.117647	no	no	no	no	no	no	no	no	no	no

182213064	chr1	105417999	105418031	17	33	1.941176	no	no	no	no	no	no	no	no	no	no
182213072	chr1	105454902	105454931	14	30	2.142857	no	no	no	no	no	no	no	no	no	no
182213073	chr1	105463524	105463564	19	41	2.210526	no	no	no	no	no	no	no	no	no	no
182213087	chr1	105482059	105482108	23	50	2.173913	no	no	no	no	no	no	no	yes	no	no
182213092	chr1	105501340	105501388	23	49	2.130435	no	no	no	no	no	no	no	no	no	no
182213105	chr1	105538993	105539024	11	32	2.909091	no	no	no	no	no	no	no	no	no	no
182213111	chr1	105556918	105556948	15	31	2.066667	no	no	no	no	no	no	no	no	yes	no
182213126	chr1	105595333	105595459	60	127	2.183333	no	no	yes	no	no	no	no	no	no	yes
182213134	chr1	105603940	105603975	18	36	2.000000	no	no	yes	no	no	no	no	no	no	yes
182213136	chr1	105608084	105608159	27	76	2.821429	no	no	yes	no	no	no	no	no	no	yes
182213145	chr1	105624494	105624585	29	92	3.233333	no	no	no	no	no	no	no	no	no	no
182213153	chr1	105650688	105650752	29	65	2.241379	no	no	no	no	no	no	no	no	no	no
182213154	chr1	105658001	105658033	16	33	2.125000	no	no	no	no	no	no	no	no	no	no
182213157	chr1	105665492	105665551	30	60	2.000000	no	no	no	no	no	no	no	no	no	no
182213161	chr1	105681626	105681699	38	74	1.947368	no	no	no	no	no	no	no	no	no	no
182213162	chr1	105684680	105684724	23	45	1.956522	no	no	no	no	no	no	no	yes	no	no
182213164	chr1	105694087	105694123	14	37	2.642857	no	no	no	no	no	no	no	no	no	no
182213168	chr1	105705908	105705936	15	29	1.933333	no	no	no	no	no	no	no	no	no	no
182213176	chr1	105732552	105732599	18	48	2.666667	no	no	no	no	no	no	no	no	no	no
182213179	chr1	105736345	105736389	13	45	3.538461	no	no	no	no	no	no	no	yes	no	no
182213184	chr1	105749470	105749498	15	29	1.933333	no	no	no	no	no	no	no	no	no	no
182213191	chr1	105751017	105751073	27	57	2.148148	no	no	no	no	no	no	no	no	no	no
182213204	chr1	105769123	105769436	37	314	8.513514	no	no	no	no	no	no	no	no	no	no
182213205	chr1	105771719	105771766	20	48	2.400000	no	no	no	no	no	no	no	no	no	no
182213208	chr1	105789380	105789417	20	38	1.900000	no	no	no	no	no	no	no	no	no	no

182213209	chr1	105792930	105792959	16	30	1.937500	no	no	no	no	no	no	no	no	no	no
182213212	chr1	105802732	105802765	14	34	2.428571	no	no	no	no	no	no	no	no	no	no
182213237	chr1	105858637	105858679	22	43	1.954545	no	no	no	no	no	no	no	no	no	no
182213240	chr1	105874200	105874248	24	49	2.166667	no	no	no	no	no	no	no	no	no	no
182213268	chr1	105953567	105953628	24	62	2.583333	yes	no	yes	no	yes	no	no	no	no	yes
182213278	chr1	105963189	105963225	19	37	2.052632	no	no	yes	no	no	no	no	no	no	yes
182213279	chr1	105963241	105963280	20	40	2.000000	no	no	yes	no	no	no	no	no	no	yes
182213284	chr1	105969167	105969226	19	60	3.000000	no	no	yes	no	no	no	no	no	no	yes
182213287	chr1	105979943	105979971	14	29	2.071429	no	no	yes	no	no	no	no	no	no	yes
182213321	chr1	106052005	106052055	26	51	1.961538	no	no	yes	no	no	no	no	no	no	yes
182213330	chr1	106068664	106068696	17	33	1.941176	no	no	yes	no	no	no	no	no	no	yes
182213331	chr1	106073723	106073898	89	176	1.977528	no	no	yes	no	no	no	no	yes	no	yes
182213335	chr1	106079916	106079958	21	43	2.047619	yes	no	yes	no	no	yes	no	no	no	yes
182213338	chr1	106091726	106091809	42	84	2.000000	no	no	yes	no	no	no	no	no	no	yes
182213339	chr1	106093996	106094039	24	44	1.916667	no	no	yes	no	no	no	no	no	no	yes
182213344	chr1	106100072	106100112	16	41	2.562500	no	no	yes	no	no	no	no	no	no	yes
182213351	chr1	106131205	106131259	26	55	2.115385	no	no	no	no	no	no	no	no	no	no
182213379	chr1	106275706	106275763	17	58	3.388889	no	no	no	no	no	no	no	yes	no	no
182213382	chr1	106283101	106283134	18	34	1.944444	no	no	no	no	no	no	no	yes	no	no
182213386	chr1	106286233	106286310	24	78	3.240000	no	no	no	no	no	no	no	no	no	no
182213389	chr1	106299789	106299829	21	41	2.047619	no	no	no	no	no	no	no	no	no	no
182213390	chr1	106300441	106300478	20	38	1.900000	no	no	no	no	no	no	no	no	no	no
182213395	chr1	106317583	106317625	23	43	1.913043	no	no	no	no	no	no	no	yes	yes	no
182213396	chr1	106321975	106322114	56	140	2.589286	no	no	no	no	no	no	no	no	no	no
182213400	chr1	106328498	106328539	20	42	2.150000	no	no	no	no	no	no	no	yes	no	no

182213403	chr1	106337152	106337190	20	39	2.000000	no	no	no	no	no	no	no	no	no	no
182213416	chr1	106383257	106383282	13	26	2.000000	no	no	no	no	no	no	no	no	no	no
182213421	chr1	106391667	106391700	17	34	2.058824	no	no	no	no	no	no	no	yes	no	no
182213429	chr1	106402124	106402169	17	46	2.705882	no	no	no	no	no	no	no	no	no	no
182213432	chr1	106402158	106402268	23	111	4.880000	no	no	no	no	no	no	no	no	no	no
182213449	chr1	106430105	106430145	22	41	1.909091	no	no	no	no	no	no	no	yes	no	no
182213469	chr1	106486934	106486966	17	33	1.941176	no	no	no	no	no	no	no	yes	no	no
182213472	chr1	106501742	106501766	13	25	1.923077	no	no	no	no	no	no	no	yes	yes	no
182213473	chr1	106509498	106509525	14	28	2.000000	no	no	no	no	no	no	no	no	no	no
182213475	chr1	106517018	106517050	16	33	2.062500	no	no	no	no	no	no	no	no	no	no
182213482	chr1	106520231	106520278	18	48	2.777778	no	no	no	no	no	no	no	yes	no	no
182213486	chr1	106521540	106521571	11	32	2.909091	no	no	no	no	no	no	no	no	no	no
182213496	chr1	106542218	106542262	24	45	1.916667	no	no	no	no	no	no	no	no	no	no
182213515	chr1	106583717	106583759	20	43	2.150000	no	no	no	no	no	no	no	no	no	no
182213524	chr1	106610334	106610370	18	37	2.111111	no	no	no	no	no	no	no	yes	yes	no
182213526	chr1	106617288	106617318	15	31	2.066667	no	no	no	no	no	no	no	no	yes	no
182213537	chr1	106642021	106642065	18	45	2.611111	no	no	no	no	no	no	no	no	no	no
182213547	chr1	106675748	106675790	23	43	1.956522	no	no	no	no	no	no	no	no	yes	no
182213549	chr1	106680400	106680439	16	40	2.562500	no	no	no	no	no	no	no	yes	yes	no
182213558	chr1	106723920	106723956	17	37	2.176471	no	no	no	no	no	no	no	no	no	no
182213559	chr1	106724492	106724531	20	40	2.000000	no	no	no	no	no	no	no	no	yes	no
182213570	chr1	106765386	106765455	27	70	2.571429	no	no	no	no	no	no	no	no	no	no
182213576	chr1	106800580	106800622	16	43	2.750000	no	no	no	no	no	no	no	yes	no	no
182213577	chr1	106804773	106804799	14	27	1.928571	no	no	no	no	yes	no	no	no	no	yes
182213579	chr1	106815146	106815182	14	37	2.666667	no	no	no	no	no	no	no	yes	no	no

182213601	chr1	106846143	106846187	17	45	2.647059	no	no	yes	no	no	yes	no	no	no	yes
182213616	chr1	106916408	106916437	14	30	2.142857	no	no	no	no	no	no	no	yes	no	no
182213618	chr1	106921732	106921770	20	39	1.950000	no	no	no	no	no	no	no	yes	no	no
182213623	chr1	106923746	106923786	20	41	2.150000	no	no	no	no	no	no	no	no	no	no
182213624	chr1	106923837	106923879	22	43	1.954545	no	no	no	no	no	no	no	no	no	no
182213633	chr1	106952268	106952308	19	41	2.157895	no	no	no	no	no	no	no	yes	no	no
182213642	chr1	106977964	106978004	19	41	2.157895	no	no	no	no	no	no	no	no	no	no
182213643	chr1	106980844	106980896	22	53	2.454545	no	no	no	no	no	no	no	no	no	no
182213646	chr1	106984773	106984872	31	100	3.419355	no	no	no	no	no	no	no	no	no	no
182213655	chr1	107027431	107027469	20	39	1.950000	no	no	no	no	no	no	no	no	no	no
182213677	chr1	107069411	107069455	23	45	2.000000	no	no	no	no	no	no	no	no	no	no
182213679	chr1	107069463	107069506	22	44	2.045455	no	no	no	no	no	no	no	no	no	no
182213682	chr1	107071477	107071503	14	27	1.928571	no	no	no	no	no	no	no	no	no	no
182213686	chr1	107088638	107088683	23	46	2.000000	no	no	no	no	no	no	no	no	no	no
182213695	chr1	107088771	107088850	30	80	2.733333	no	no	no	no	no	no	no	no	no	no
182213699	chr1	107088919	107088975	22	57	2.681818	no	no	no	no	no	no	no	no	no	no
182213701	chr1	107088963	107088989	14	27	1.928571	no	no	no	no	no	no	no	no	no	no
182213703	chr1	107089178	107089213	13	36	2.666667	no	no	no	no	no	no	no	yes	no	no
182213704	chr1	107093955	107093996	16	42	2.625000	no	no	no	no	no	no	no	yes	no	no
182213706	chr1	107096310	107096340	14	31	2.214286	no	no	no	no	no	no	no	no	yes	no
182213714	chr1	107120382	107120418	18	37	2.055556	no	no	no	no	no	no	no	no	no	no
182213716	chr1	107129407	107129454	19	48	2.600000	no	no	no	no	no	no	no	yes	no	no
182213718	chr1	107134903	107134940	20	38	1.950000	no	no	no	no	no	no	no	no	no	no
182213721	chr1	107140109	107140149	21	41	1.952381	yes	no	no	no	yes	no	no	yes	yes	yes
182213726	chr1	107151996	107152102	46	107	2.326087	no	no	yes	no	no	no	no	no	no	yes

182213744	chr1	107200308	107200340	17	33	2.000000	no	no	yes	no	no	no	no	no	yes	yes
182213754	chr1	107251740	107251789	21	50	2.454545	no	no	yes	no	no	no	no	yes	no	yes
182213756	chr1	107262139	107262171	16	33	2.062500	no	no	yes	no	no	no	no	no	no	yes
182213759	chr1	107272198	107272231	12	34	2.833333	no	no	yes	no	no	no	no	yes	no	yes
182213764	chr1	107289848	107289884	17	37	2.235294	no	no	yes	no	no	no	no	no	no	yes
182213765	chr1	107290138	107290175	19	38	2.000000	no	no	yes	no	no	no	no	no	no	yes
182213768	chr1	107296542	107296590	23	49	2.217391	no	no	yes	no	no	no	no	no	no	yes
182213770	chr1	107296601	107296637	19	37	1.947368	no	no	yes	no	no	no	no	no	no	yes
182213773	chr1	107296717	107296771	19	55	2.900000	no	no	yes	no	no	no	no	no	no	yes
182213779	chr1	107297133	107297239	44	107	2.533333	no	no	yes	no	no	no	no	no	no	yes
182213784	chr1	107342383	107342418	16	36	2.312500	no	no	yes	no	no	no	no	yes	no	yes
182213798	chr1	107380843	107380893	25	51	2.040000	no	no	yes	no	no	no	no	yes	no	yes
182213800	chr1	107385316	107385397	27	82	3.037037	no	no	yes	no	no	no	no	no	yes	yes
182213802	chr1	107386066	107386144	36	79	2.194444	no	no	yes	no	no	no	no	yes	no	yes
182213805	chr1	107410206	107410240	13	35	2.692308	no	yes	yes	no	no	no	no	no	no	yes
182213808	chr1	107417065	107417092	14	28	2.000000	no	no	yes	no	no	no	no	no	no	yes
182213809	chr1	107417845	107417879	17	35	2.058824	no	no	yes	no	no	no	no	no	no	yes
182213814	chr1	107466040	107466162	58	123	2.120690	no	no	yes	no	no	no	no	no	yes	yes
182213817	chr1	107489178	107489210	16	33	2.062500	no	no	no	no	no	no	no	no	no	no
182213818	chr1	107489528	107489592	33	65	1.969697	no	no	no	no	no	no	no	no	no	no
182213820	chr1	107502684	107502713	13	30	2.307692	no	no	no	no	no	no	no	yes	yes	no
182213827	chr1	107526961	107526990	15	30	2.000000	no	no	no	no	no	no	no	yes	yes	no
182213835	chr1	107550086	107550122	17	37	2.235294	no	no	no	no	no	no	no	no	no	no
182213837	chr1	107553147	107553179	15	33	2.200000	no	no	no	no	no	no	no	no	no	no
182213838	chr1	107555656	107555704	22	49	2.227273	no	no	no	no	no	no	no	yes	yes	no

182213840	chr1	107563159	107563195	16	37	2.437500	no	no	no	no	no	no	no	no	no	no
182213846	chr1	107588320	107588356	17	37	2.176471	no	no	yes	no	no	no	no	no	no	yes
182213853	chr1	107610234	107610526	158	293	1.854430	no	no	yes	no	no	no	no	yes	no	yes
182213861	chr1	107644925	107644983	19	59	3.105263	no	no	yes	no	no	no	no	no	no	yes
182213862	chr1	107647868	107647894	14	27	1.928571	no	no	yes	no	no	no	no	no	no	yes
182213865	chr1	107659710	107659743	17	34	2.000000	no	no	yes	no	no	no	no	no	no	yes
182213866	chr1	107667599	107667637	21	39	1.904762	no	no	yes	no	no	no	no	no	no	yes
182213868	chr1	107680500	107680551	25	52	2.080000	no	no	yes	no	no	no	no	yes	no	yes
182213873	chr1	107715278	107715319	22	42	2.000000	no	no	yes	no	no	no	no	no	no	yes
182213875	chr1	107723329	107723385	30	57	1.900000	no	no	yes	no	no	no	no	no	no	yes
182213880	chr1	107747793	107748185	197	393	2.000000	no	no	yes	no	no	no	no	yes	no	yes
182213899	chr1	107810903	107810944	21	42	2.047619	no	no	yes	no	no	no	no	no	no	yes
182213901	chr1	107818209	107818238	15	30	2.000000	no	no	yes	no	no	no	no	yes	no	yes
182213902	chr1	107818450	107818503	21	54	2.590909	no	no	yes	no	no	no	no	yes	yes	yes
182213908	chr1	107840182	107840226	23	45	2.000000	no	no	yes	no	no	no	no	no	no	yes
182213912	chr1	107863416	107863449	17	34	2.000000	no	no	yes	no	no	no	no	no	yes	yes
182213915	chr1	107884754	107884797	19	44	2.421053	no	no	yes	no	no	no	no	no	no	yes
182213926	chr1	107918655	107918700	23	46	2.000000	no	no	yes	no	no	no	no	no	no	yes
182213932	chr1	107937715	107937747	14	33	2.428571	no	no	yes	no	no	no	no	no	no	yes
182213936	chr1	107965284	107965320	13	37	2.923077	yes	no	yes	no	no	no	yes	yes	no	yes
182213941	chr1	107998642	107998697	22	56	2.545455	no	no	no	no	no	no	no	no	no	no
182213944	chr1	108001879	108001935	18	57	3.100000	no	no	no	no	no	no	no	no	no	no
182213945	chr1	108002215	108002267	20	53	2.619048	no	no	no	no	no	no	no	no	no	no
182213960	chr1	108057462	108057535	34	74	2.176471	no	no	yes	no	no	no	no	yes	no	yes
182213962	chr1	108073340	108073467	64	128	2.015625	no	no	yes	no	no	no	no	yes	yes	yes

182213979	chr1	108113989	108114044	25	56	2.280000	no	no	no	no	no	no	no	no	no	no
182213980	chr1	108115722	108115753	16	32	2.000000	no	no	no	no	no	no	no	no	no	no
182213981	chr1	108116379	108116419	19	41	2.210526	no	no	no	no	no	no	no	no	no	no
182214002	chr1	108182945	108182986	22	42	2.000000	no	no	yes	no	no	no	no	yes	yes	yes
182214003	chr1	108184300	108184333	17	34	2.000000	no	no	yes	no	no	no	no	yes	no	yes
182214020	chr1	108241348	108241414	24	67	2.791667	no	no	yes	no	no	no	no	no	no	yes
182214065	chr1	108344766	108344816	23	51	2.260870	no	no	no	no	no	no	no	yes	no	no
182214085	chr1	108383694	108383731	19	38	2.052632	no	no	yes	no	no	yes	no	no	no	yes
182214153	chr1	108495187	108495254	37	68	1.918919	no	no	no	no	no	yes	no	yes	no	no
182214180	chr1	108550570	108550678	40	109	2.707317	no	no	no	no	no	no	no	no	no	no
182214186	chr1	108550781	108550839	26	59	2.269231	no	no	no	no	no	no	no	no	no	no
182214191	chr1	108551031	108551063	16	33	2.125000	no	no	no	no	no	no	no	yes	no	no
182214199	chr1	108571748	108571795	19	48	2.526316	no	no	yes	no	no	no	no	no	no	yes
182214214	chr1	108595551	108595591	17	41	2.470588	no	no	yes	no	no	no	no	yes	yes	yes
182214216	chr1	108595579	108595799	93	221	2.404255	no	no	yes	no	no	no	no	yes	yes	yes
182214223	chr1	108636897	108636937	21	41	2.000000	no	yes	yes	no	no	no	no	no	no	yes
182214225	chr1	108647307	108647366	29	60	2.068965	no	no	no	no	no	yes	no	yes	no	no
182214232	chr1	108657975	108658122	54	148	2.740741	no	no	yes	no	no	no	no	no	yes	yes
182214238	chr1	108669005	108669046	16	42	2.750000	no	no	no	no	no	no	no	yes	no	no
182214252	chr1	108680870	108680921	15	52	3.600000	no	no	no	no	no	no	no	no	no	no
182214253	chr1	108682889	108682920	16	32	2.000000	no	no	no	no	no	no	no	no	no	no
182214263	chr1	108724761	108724796	12	36	3.000000	no	no	yes	no	no	no	no	no	no	yes
182214267	chr1	108732690	108732738	26	49	1.923077	no	no	yes	no	no	no	no	yes	yes	yes
182214272	chr1	108739116	108739159	22	44	2.000000	no	no	yes	no	no	no	no	no	no	yes
182214277	chr1	108756566	108756608	23	43	1.913043	no	no	yes	no	no	no	no	no	no	yes

182214283	chr1	108775067	108775126	30	60	2.066667	no	no	yes	no	no	no	no	no	no	yes
182214289	chr1	108793685	108793710	11	26	2.363636	no	no	yes	no	no	yes	no	no	no	yes
182214299	chr1	108856719	108856762	16	44	2.750000	yes	no	yes	no	no	no	no	no	no	yes
182214308	chr1	108882845	108882889	22	45	2.045455	no	no	yes	no	no	no	no	yes	no	yes
182214311	chr1	108897427	108897465	17	39	2.294118	yes	no	yes	no	no	yes	no	no	no	yes
182214313	chr1	108899254	108899295	22	42	1.954545	no	no	yes	no	no	yes	no	no	no	yes
182214314	chr1	108901302	108901376	38	75	1.973684	yes	no	yes	no	no	no	no	yes	yes	yes
182214315	chr1	108902011	108902037	14	27	1.928571	no	no	yes	no	no	no	no	no	no	yes
182214329	chr1	108931801	108931877	30	77	2.687500	no	yes	yes	no	no	yes	no	no	no	yes
182214332	chr1	108934523	108934562	20	40	2.000000	no	yes	yes	no	no	yes	no	no	no	yes
182214339	chr1	108956875	108956912	8	38	4.750000	no	no	yes	no	no	no	no	yes	yes	yes
182214347	chr1	108983799	108983856	11	58	5.272727	no	no	yes	no	no	no	no	yes	no	yes
182214348	chr1	108986671	108986724	27	54	2.000000	no	no	yes	yes	no	yes	no	no	no	yes
182214359	chr1	109005955	109005994	22	40	1.909091	no	no	yes	no	no	no	no	no	no	yes
182214366	chr1	109028751	109028779	14	29	2.071429	no	no	yes	no	no	no	no	no	no	yes
182214369	chr1	109030375	109030412	17	38	2.294118	no	no	yes	no	no	yes	no	no	no	yes
182214371	chr1	109042290	109042342	21	53	2.523809	yes	no	no	no	no	no	no	yes	no	no
182214403	chr1	109080377	109080421	23	45	2.043478	no	no	no	no	no	no	no	yes	yes	no
182214404	chr1	109080517	109080553	14	37	2.642857	no	no	no	no	no	no	no	yes	yes	no
182214413	chr1	109097135	109097170	16	36	2.250000	no	no	no	no	no	yes	no	no	no	no
182214471	chr1	109246599	109246623	13	25	1.923077	no	no	no	no	no	no	no	yes	no	no
182214475	chr1	109266004	109266055	10	52	5.300000	no	no	yes	no	no	no	no	yes	no	yes
182214477	chr1	109272916	109272946	12	31	2.583333	no	yes	no	yes	no	no	no	yes	no	yes
182214484	chr1	109298531	109298569	19	39	2.105263	no	no	yes	no	no	no	no	yes	no	yes
182214485	chr1	109300736	109300788	25	53	2.120000	no	no	yes	no	no	no	no	yes	yes	yes

182214486	chr1	109301247	109301283	18	37	2.166667	no	no	yes	no	no	no	no	yes	yes	yes
182214490	chr1	109302145	109302176	16	32	2.000000	no	no	yes	no	no	no	no	yes	no	yes
182214493	chr1	109309361	109309389	15	29	1.933333	no	no	no	no	no	yes	no	yes	yes	no
182214502	chr1	109348456	109348496	20	41	2.150000	no	no	yes	no	no	no	no	no	no	yes
182214508	chr1	109363463	109363499	16	37	2.312500	no	no	yes	no	no	no	no	no	no	yes
182214511	chr1	109370503	109370539	18	37	2.055556	yes	no	yes	no	no	no	no	yes	no	yes
182214514	chr1	109377149	109377186	17	38	2.235294	no	no	yes	no	no	no	no	no	yes	yes
182214521	chr1	109397928	109397970	22	43	2.000000	yes	no	no	no	no	no	yes	yes	yes	no
182214529	chr1	109426387	109426420	15	34	2.266667	yes	no	no	no	yes	no	yes	yes	no	yes
182214537	chr1	109449479	109449574	28	96	3.448276	no	no	no	no	no	no	no	no	no	no
182214547	chr1	109465238	109465275	19	38	2.052632	no	no	no	no	no	no	no	yes	no	no
182214554	chr1	109483873	109483925	29	53	1.931034	yes	no	no	no	yes	no	yes	yes	yes	yes
182214555	chr1	109483904	109483950	25	47	1.920000	yes	no	no	no	yes	no	yes	yes	yes	yes
182214560	chr1	109498944	109498998	17	55	3.235294	no	yes	yes	no	no	yes	no	yes	no	yes
182214563	chr1	109505514	109505552	16	39	2.437500	no	yes	no	no	no	no	no	no	no	yes
182214568	chr1	109521828	109521871	24	44	1.916667	no	no	no	no	no	no	no	yes	yes	no
182214575	chr1	109532115	109532154	16	40	2.500000	no	no	no	no	no	no	no	yes	yes	no
182214580	chr1	109534195	109534223	13	29	2.230769	no	no	no	no	no	no	no	yes	no	no
182214584	chr1	109549948	109550020	34	73	2.147059	no	no	yes	no	no	no	no	no	no	yes
182214590	chr1	109562078	109562121	18	44	2.500000	no	no	yes	no	no	no	no	no	no	yes
182214599	chr1	109604923	109604999	39	77	2.000000	no	yes	yes	no	no	no	no	no	no	yes
182214601	chr1	109605116	109605293	90	178	1.977778	no	yes	yes	no	no	no	no	no	no	yes
182214602	chr1	109605236	109605354	59	119	2.016949	no	yes	yes	no	no	no	no	no	no	yes
182214604	chr1	109619804	109619837	16	34	2.125000	yes	no	yes	no	yes	no	no	yes	yes	yes
182214605	chr1	109628596	109628640	21	45	2.142857	yes	yes	yes	no	yes	yes	no	yes	yes	yes

182214606	chr1	109630557	109630589	8	33	4.125000	yes	no	yes	no	no	yes	no	yes	yes	yes
182214614	chr1	109656206	109656264	30	59	1.966667	yes	yes	no	no	yes	no	yes	yes	yes	yes
182214620	chr1	109669575	109669625	21	51	2.476191	no	yes	yes	no	no	yes	no	yes	no	yes
182214632	chr1	109715242	109715289	25	48	1.960000	no	yes	yes	no	no	yes	no	no	no	yes
182214638	chr1	109737658	109737702	23	45	2.086957	no	no	yes	yes	yes	no	no	no	no	yes
182214641	chr1	109759006	109759047	22	42	1.954545	no	no	yes	no	no	no	no	yes	no	yes
182214643	chr1	109759207	109759241	14	35	2.500000	no	yes	yes	yes	yes	no	no	yes	no	yes
182214644	chr1	109759513	109759537	13	25	1.923077	no	no	yes	no	yes	no	no	yes	no	yes
182214645	chr1	109760701	109760738	17	38	2.235294	no	no	yes	no	no	yes	no	yes	no	yes
182214648	chr1	109774290	109774321	16	32	2.000000	no	no	yes	no	no	no	no	yes	no	yes
182214658	chr1	109807961	109807998	10	38	3.800000	no	no	no	no	no	no	no	yes	no	no
182214670	chr1	109849603	109849641	20	39	1.950000	no	no	yes	no	no	no	no	yes	yes	yes
182214676	chr1	109868271	109868305	18	35	1.944444	no	no	yes	no	no	no	no	yes	yes	yes
182214678	chr1	109878674	109878720	18	47	2.611111	no	no	no	no	no	no	no	no	yes	no
182214681	chr1	109896341	109896416	38	76	2.000000	no	yes	no	no	no	no	no	yes	no	yes
182214689	chr1	109907342	109907434	50	93	1.940000	no	no	no	no	no	no	no	yes	no	no
182214690	chr1	109907466	109907498	16	33	2.062500	no	no	no	no	no	no	no	yes	no	no
182214696	chr1	109926976	109927004	15	29	1.933333	no	no	yes	no	no	yes	no	yes	yes	yes
182214700	chr1	109944719	109944821	54	103	1.907407	no	no	no	no	no	no	no	yes	yes	no
182214705	chr1	109953981	109954036	19	56	2.947368	no	no	no	no	no	no	no	yes	no	no
182214709	chr1	109960626	109960655	15	30	2.000000	no	no	no	no	no	no	no	yes	yes	no
182214714	chr1	109978207	109978248	19	42	2.315789	no	no	no	no	no	no	no	yes	yes	no
182214716	chr1	109987578	109987659	40	82	2.050000	no	no	yes	no	no	no	no	yes	yes	yes
182214717	chr1	109992006	109992031	13	26	2.000000	no	no	yes	no	no	no	no	no	no	yes
182214718	chr1	109998296	109998328	13	33	2.538461	no	no	yes	no	no	no	no	no	no	yes

182214720	chr1	110010393	110010429	19	37	1.947368	no	no	yes	no	no	no	no	no	no	yes
182214729	chr1	110039530	110039568	19	39	2.052632	no	yes	yes	no	no	no	no	yes	no	yes
182214733	chr1	110061720	110061744	12	25	2.083333	no	yes	yes	no	no	no	no	no	yes	yes
182214735	chr1	110064006	110064043	14	38	2.714286	no	no	yes	no	no	no	no	no	no	yes
182214737	chr1	110066333	110066357	12	25	2.083333	no	no	yes	no	no	no	no	no	yes	yes
182214739	chr1	110078385	110078431	24	47	1.958333	no	no	no	no	no	no	no	no	no	no
182214742	chr1	110080110	110080243	39	134	3.452381	no	no	no	no	no	no	no	yes	no	no
182214743	chr1	110087386	110087839	46	454	9.800000	no	no	yes	no	no	no	no	yes	no	yes
182214748	chr1	110094344	110094378	19	35	1.947368	no	no	yes	no	no	no	no	yes	no	yes
182214752	chr1	110109525	110109602	41	78	1.902439	no	no	yes	no	no	no	no	no	no	yes
182214757	chr1	110120096	110120207	52	112	2.153846	no	no	no	no	no	no	no	yes	yes	no
182214764	chr1	110128201	110128241	16	41	2.562500	no	no	no	no	no	no	no	no	no	no
182214768	chr1	110142072	110142106	10	35	3.363636	no	no	no	no	no	no	no	no	no	no
182214774	chr1	110158242	110158276	15	35	2.400000	no	no	yes	no	no	no	no	no	no	yes
182214775	chr1	110161071	110161106	16	36	2.250000	no	no	yes	no	no	no	no	no	no	yes
182214776	chr1	110170620	110170660	16	41	2.562500	no	no	yes	no	no	no	no	no	yes	yes
182214785	chr1	110199840	110199976	54	137	2.537037	no	yes	no	no	no	no	no	yes	no	yes
182214788	chr1	110216493	110216523	15	31	2.133333	no	no	yes	no	no	no	no	no	no	yes
182214790	chr1	110240003	110240043	18	41	2.333333	no	yes	yes	no	no	no	no	yes	yes	yes
182214791	chr1	110240403	110240431	15	29	1.933333	no	yes	yes	no	no	no	no	yes	yes	yes
182214792	chr1	110243699	110243735	12	37	3.166667	no	yes	yes	no	no	no	no	yes	yes	yes
182214796	chr1	110254890	110254926	15	37	2.466667	no	no	yes	no	no	no	no	yes	yes	yes
182214806	chr1	110301904	110301928	13	25	1.923077	no	no	yes	no	no	no	no	no	no	yes
182214807	chr1	110312597	110312645	15	49	3.266667	no	no	yes	no	no	no	no	yes	no	yes
182214808	chr1	110317232	110317261	13	30	2.307692	no	no	yes	no	no	no	no	no	no	yes

182214812	chr1	110331786	110331826	21	41	1.952381	no	no	yes	no	no	no	no	no	no	yes
182214820	chr1	110362908	110362994	43	87	2.023256	no	yes	yes	no	no	yes	no	no	no	yes
182214827	chr1	110392755	110392812	30	58	1.933333	no	no	yes	no	no	no	no	yes	no	yes
182214849	chr1	110451712	110451753	19	42	2.210526	no	no	yes	no	no	no	no	no	no	yes
182214850	chr1	110452920	110452963	23	44	2.000000	no	no	yes	no	no	no	no	no	no	yes
182214851	chr1	110454173	110454230	30	58	2.000000	no	no	yes	no	no	no	no	yes	no	yes
182214852	chr1	110457029	110457099	38	71	1.921053	no	yes	yes	no	yes	no	no	no	no	yes
182214859	chr1	110471928	110471973	24	46	2.000000	yes	no	no	no	no	no	no	yes	no	no
182214861	chr1	110479372	110479412	13	41	3.230769	no	no	yes	no	no	no	no	no	no	yes
182214863	chr1	110489289	110489321	15	33	2.333333	no	no	yes	no	yes	yes	no	yes	no	yes
182214866	chr1	110503166	110503215	26	50	2.000000	no	no	no	no	no	no	no	no	yes	no
182214875	chr1	110513554	110513587	17	34	2.000000	no	no	no	no	no	no	no	yes	no	no
182214886	chr1	110543245	110543275	14	31	2.214286	no	no	yes	no	no	no	no	yes	no	yes
182214889	chr1	110569599	110569823	30	225	7.533333	no	no	yes	no	no	no	no	no	no	yes
182214891	chr1	110572197	110572243	23	47	2.043478	no	no	yes	no	no	no	no	yes	no	yes
182214892	chr1	110576656	110576781	15	126	8.466666	no	no	yes	no	no	no	no	yes	no	yes
182214898	chr1	110592428	110592463	16	36	2.312500	no	no	yes	no	no	no	no	no	no	yes
182214902	chr1	110596313	110596351	19	39	2.052632	no	yes	yes	no	no	yes	no	no	no	yes
182214903	chr1	110600399	110600440	17	42	2.470588	no	yes	yes	no	no	no	no	no	no	yes
182214906	chr1	110616556	110616587	16	32	2.000000	no	no	yes	no	no	no	no	yes	no	yes
182214913	chr1	110637299	110637330	16	32	2.000000	no	no	no	no	no	no	no	no	no	no
182214917	chr1	110642792	110642824	12	33	2.750000	no	no	no	no	no	no	no	no	no	no
182214918	chr1	110643288	110643320	17	33	1.941176	no	no	no	no	no	no	no	no	no	no
182214922	chr1	110652489	110652829	170	341	2.005882	no	no	no	no	no	yes	no	no	no	no
182214924	chr1	110661026	110661055	15	30	2.000000	no	no	no	no	no	no	no	no	no	no

182214925	chr1	110661054	110661092	12	39	3.000000	no	no	no	no	no	no	no	no	no	no
182214927	chr1	110702779	110702817	14	39	2.785714	no	no	no	no	no	no	no	no	no	no
182214933	chr1	110722305	110722351	23	47	2.130435	no	no	no	no	no	no	no	yes	no	no
182214943	chr1	110736763	110736810	22	48	2.181818	no	no	no	no	no	no	no	no	no	no
182214947	chr1	110748069	110748144	40	76	1.975000	no	no	no	no	no	no	no	no	no	no
182214948	chr1	110750814	110750849	17	36	2.117647	no	no	no	no	no	no	no	no	no	no
182214959	chr1	110781109	110781141	15	33	2.200000	no	no	no	no	no	no	no	yes	yes	no
182214964	chr1	110814248	110814275	13	28	2.153846	no	no	no	no	no	no	no	no	no	no
182214969	chr1	110831821	110831867	18	47	2.611111	no	no	no	no	no	no	no	no	no	no
182214970	chr1	110832244	110832276	11	33	3.090909	no	no	no	no	no	no	no	no	no	no
182214972	chr1	110844589	110844641	26	53	2.038461	no	no	no	no	no	no	no	no	no	no
182214973	chr1	110851901	110851940	19	40	2.105263	no	no	no	no	no	no	no	no	no	no
182214977	chr1	110878571	110878896	173	326	1.901734	no	no	yes	no	no	no	no	yes	yes	yes
182214984	chr1	110900876	110900916	21	41	1.952381	no	no	no	no	no	yes	no	no	no	no
182214999	chr1	110952879	110952906	10	28	2.800000	no	no	yes	no	yes	yes	no	yes	no	yes
182215010	chr1	110976529	110976559	16	31	1.937500	no	no	no	no	no	no	no	no	no	no
182215030	chr1	111052681	111052766	33	86	2.727273	no	no	no	no	no	no	no	yes	no	no
182215053	chr1	111139500	111139536	17	37	2.176471	yes	no	yes	no	yes	no	no	yes	yes	yes
182215054	chr1	111140499	111140551	25	53	2.120000	yes	yes	yes	no	no	no	yes	yes	no	yes
182215061	chr1	111164515	111164554	19	40	2.105263	no	no	yes	no	no	no	no	no	no	yes
182215064	chr1	111169408	111169496	43	89	2.069767	no	no	yes	no	no	no	no	no	no	yes
182215068	chr1	111224556	111224585	16	30	1.937500	no	no	yes	no	no	no	no	yes	no	yes
182215071	chr1	111232355	111232392	13	38	3.000000	no	no	yes	no	no	yes	no	yes	no	yes
182215073	chr1	111244858	111244887	14	30	2.142857	no	no	no	no	no	no	no	no	no	no
182215075	chr1	111245804	111245856	15	53	3.533333	no	no	no	no	no	no	no	no	yes	no

182215079	chr1	111279660	111279694	17	35	2.058824	yes	no	no	no	no	no	no	no	no	no
182215096	chr1	111324973	111325005	17	33	1.941176	yes	no	no	no	no	yes	no	no	no	no
182215099	chr1	111334014	111334050	19	37	2.000000	no	no	no	no	no	no	no	yes	no	no
182215102	chr1	111342447	111342475	15	29	1.933333	no	no	no	no	no	no	no	no	no	no
182215125	chr1	111414898	111415070	45	173	3.844445	no	no	no	yes	no	yes	no	yes	no	yes
182215141	chr1	111482016	111482050	17	35	2.058824	no	no	no	no	no	no	no	yes	no	no
182215151	chr1	111506917	111507007	38	91	2.394737	no	no	yes	no	no	no	no	no	no	yes
182215158	chr1	111534762	111534803	22	42	1.909091	no	no	yes	no	no	no	no	no	yes	yes
182215171	chr1	111544734	111545142	34	409	12.000000	no	no	yes	no	no	no	no	no	no	yes
182215174	chr1	111551715	111551771	21	57	2.761905	no	no	yes	no	no	no	no	yes	no	yes
182215202	chr1	111611100	111611132	15	33	2.200000	no	no	yes	no	no	no	no	no	yes	yes
182215205	chr1	111617031	111617076	18	46	2.555556	no	no	yes	no	no	no	no	yes	yes	yes
182215208	chr1	111625294	111625330	18	37	2.111111	no	no	yes	no	no	no	no	yes	yes	yes
182215211	chr1	111625749	111625827	37	79	2.189189	no	no	yes	no	no	no	no	yes	no	yes
182215225	chr1	111655225	111655275	24	51	2.166667	no	no	yes	no	no	no	no	yes	no	yes
182215227	chr1	111657150	111657187	7	38	5.571429	no	no	yes	no	no	no	no	yes	yes	yes
182215229	chr1	111663322	111663354	18	33	1.944444	no	no	yes	no	no	no	no	no	no	yes
182215232	chr1	111674268	111674312	24	45	1.916667	no	no	yes	no	no	no	no	no	no	yes
182215245	chr1	111697537	111697572	18	36	2.000000	no	no	yes	no	no	no	no	no	no	yes
182215262	chr1	111772422	111772448	14	27	1.928571	no	yes	no	no	no	no	no	no	no	yes
182215263	chr1	111775296	111775336	21	41	2.000000	no	yes	no	no	no	yes	no	no	no	yes
182215272	chr1	111815278	111815321	17	44	2.666667	no	no	yes	no	no	no	no	no	no	yes
182215276	chr1	111834345	111834396	22	52	2.409091	no	no	yes	no	no	no	no	no	no	yes
182215282	chr1	111847338	111847367	10	30	3.000000	no	no	yes	no	no	no	no	no	no	yes
182215283	chr1	111860991	111861093	50	103	2.060000	no	no	yes	no	no	no	no	yes	no	yes

182215289	chr1	111884664	111884705	21	42	2.000000	no	no	yes	no	no	no	no	yes	no	yes
182215301	chr1	111926144	111926234	45	91	2.022222	no	no	yes	no	no	no	no	no	no	yes
182215303	chr1	111948788	111948841	28	54	1.928571	no	no	yes	no	no	no	no	yes	yes	yes
182215311	chr1	111977005	111977051	14	47	3.357143	no	no	yes	no	no	no	no	yes	yes	yes
182215314	chr1	111985916	111985943	8	28	3.500000	no	no	yes	no	no	no	no	yes	no	yes
182215315	chr1	111989519	111989547	14	29	2.142857	yes	no	no	no	no	no	yes	yes	yes	no
182215317	chr1	111989724	111989778	12	55	4.583333	yes	yes	no	no	no	no	yes	yes	yes	yes
182215342	chr1	112092516	112092550	16	35	2.187500	no	no	no	no	no	no	no	no	no	no
182215346	chr1	112094807	112094836	14	30	2.142857	no	no	no	no	no	no	no	no	no	no
182215358	chr1	112124882	112124918	16	37	2.312500	no	no	no	no	no	no	no	no	no	no
182215360	chr1	112143022	112143057	18	36	2.000000	no	no	no	no	no	no	no	no	no	no
182215361	chr1	112143404	112143432	15	29	1.933333	no	no	no	no	no	no	no	no	no	no
182215363	chr1	112154201	112154233	16	33	2.125000	no	no	no	no	no	no	no	no	no	no
182215371	chr1	112182438	112182463	13	26	2.000000	no	no	yes	no	no	no	no	no	no	yes
182215392	chr1	112249507	112249547	19	41	2.210526	no	no	yes	no	no	no	no	no	no	yes
182215394	chr1	112250360	112250397	18	38	2.166667	no	no	yes	no	no	no	no	yes	no	yes
182215398	chr1	112267550	112267594	23	45	1.956522	no	no	yes	no	no	no	no	no	no	yes
182215399	chr1	112268639	112268696	30	58	1.933333	no	no	yes	no	no	no	no	yes	yes	yes
182215423	chr1	112305628	112305659	15	32	2.133333	no	no	yes	no	no	no	no	no	yes	yes
182215424	chr1	112308139	112308194	27	56	2.074074	no	no	yes	no	no	no	no	yes	yes	yes
182215426	chr1	112321918	112321950	17	33	1.941176	no	no	yes	no	no	no	no	yes	no	yes
182215428	chr1	112325889	112325929	22	41	1.954545	no	no	yes	no	no	no	no	yes	no	yes
182215430	chr1	112331683	112331730	14	48	3.500000	no	no	yes	no	no	no	no	yes	no	yes
182215431	chr1	112335130	112335166	19	37	1.947368	no	no	yes	no	no	no	no	no	no	yes
182215444	chr1	112369666	112369766	37	101	2.707317	no	no	no	no	no	no	no	yes	yes	no

182215458	chr1	112389552	112389583	15	32	2.200000	no	no	no	no	no	no	no	yes	no	no
182215466	chr1	112408195	112408242	24	48	2.000000	no	no	yes	no	no	no	no	no	no	yes
182215468	chr1	112409184	112409226	23	43	1.913043	no	no	yes	no	no	no	no	no	no	yes
182215469	chr1	112409270	112409334	28	65	2.310345	no	no	yes	no	no	no	no	no	no	yes
182215471	chr1	112409605	112409633	10	29	2.900000	no	no	yes	no	no	no	no	no	no	yes
182215472	chr1	112410637	112410949	156	313	2.006410	no	no	yes	no	no	no	no	yes	yes	yes
182215509	chr1	112496293	112496335	22	43	1.954545	no	no	yes	no	no	no	no	no	no	yes
182215512	chr1	112531775	112531836	32	62	1.937500	no	no	yes	no	no	no	no	no	no	yes
182215516	chr1	112540671	112540715	24	45	1.916667	no	no	yes	no	no	no	no	no	yes	yes
182215518	chr1	112541620	112541655	18	36	2.055556	no	no	yes	no	no	yes	no	no	no	yes
182215519	chr1	112541711	112541743	16	33	2.062500	no	no	yes	no	no	yes	no	no	no	yes
182215520	chr1	112542176	112542209	17	34	2.000000	yes	no	yes	no	no	no	no	no	no	yes
182215525	chr1	112550962	112551004	20	43	2.200000	no	no	yes	no	no	no	no	no	no	yes
182215552	chr1	112622698	112622735	20	38	1.900000	no	no	yes	no	no	no	no	yes	yes	yes
182215558	chr1	112638975	112639019	15	45	3.062500	no	no	yes	no	no	no	no	no	no	yes
182215567	chr1	112641446	112641499	24	54	2.250000	no	no	yes	no	no	no	no	no	no	yes
182215569	chr1	112644807	112644852	24	46	1.916667	no	no	yes	no	no	no	no	no	no	yes
182215570	chr1	112654975	112655006	13	32	2.461539	no	no	yes	no	no	no	no	no	no	yes
182215583	chr1	112715313	112715358	17	46	2.666667	yes	no	no	no	yes	no	yes	yes	no	yes
182215597	chr1	112743858	112743895	18	38	2.111111	no	no	no	no	no	no	yes	yes	yes	no
182215604	chr1	112757176	112757215	20	40	2.000000	no	no	no	no	no	no	no	no	no	no
182215733	chr1	112934685	112934735	27	51	1.962963	no	no	yes	no	no	no	no	yes	yes	yes
182215735	chr1	112936020	112936066	21	47	2.238095	yes	no	yes	no	no	no	no	yes	yes	yes
182215739	chr1	112950985	112951061	15	77	5.058824	no	no	yes	no	no	no	no	yes	no	yes
182215742	chr1	112955021	112955073	27	53	1.962963	no	no	yes	no	no	no	no	yes	no	yes

182215752	chr1	112964619	112964657	20	39	2.000000	no	no	yes	no	no	yes	no	no	no	yes
182215753	chr1	112966229	112966268	21	40	1.904762	no	no	yes	no	no	no	no	no	no	yes
182215759	chr1	112989217	112989250	16	34	2.125000	no	no	yes	no	no	no	no	no	no	yes
182215787	chr1	113043689	113043717	10	29	2.900000	no	no	yes	no	no	no	no	yes	yes	yes
182215795	chr1	113062900	113062954	22	55	2.500000	no	no	yes	no	no	no	no	no	no	yes
182215802	chr1	113064396	113064463	7	68	10.000000	no	no	yes	no	no	no	no	no	no	yes
182215804	chr1	113066752	113066823	20	72	3.600000	no	no	yes	no	no	no	no	yes	no	yes
182215808	chr1	113068167	113068203	18	37	2.166667	no	no	yes	no	no	no	no	no	no	yes
182215829	chr1	113088431	113088463	16	33	2.062500	no	no	yes	no	no	no	no	no	no	yes
182215835	chr1	113098500	113098553	24	54	2.250000	yes	no	yes	no	no	no	no	no	no	yes
182215844	chr1	113146984	113147018	18	35	1.944444	no	no	no	no	no	no	no	yes	yes	no
182215850	chr1	113169879	113169904	13	26	2.000000	no	no	no	no	no	yes	no	yes	no	no
182215860	chr1	113199845	113199910	21	66	3.142857	no	no	no	no	no	yes	no	yes	yes	no
182215865	chr1	113211796	113211840	24	45	1.958333	no	no	no	no	no	no	no	yes	yes	no
182215902	chr1	113319963	113320032	29	70	2.413793	no	no	no	no	no	no	no	no	no	no
182215919	chr1	113370784	113370812	14	29	2.071429	no	no	no	no	no	no	no	yes	yes	no
182215922	chr1	113377170	113377197	12	28	2.333333	no	no	no	no	no	no	no	no	no	no
182215923	chr1	113378188	113378249	31	62	2.000000	no	no	no	no	no	no	no	no	no	no
182215929	chr1	113393543	113393574	14	32	2.357143	no	no	yes	no	no	no	no	no	yes	yes
182215932	chr1	113395701	113395734	15	34	2.266667	no	no	yes	no	no	no	no	no	no	yes
182215934	chr1	113401548	113401585	13	38	2.857143	no	no	yes	no	no	no	no	yes	no	yes
182215936	chr1	113406122	113406150	15	29	1.933333	no	no	yes	no	no	no	no	yes	no	yes
182215937	chr1	113408522	113408578	26	57	2.192308	no	no	yes	no	no	no	no	no	no	yes
182215945	chr1	113429983	113430014	12	32	2.666667	no	no	yes	no	no	no	no	no	yes	yes
182215947	chr1	113440226	113440261	16	36	2.312500	no	no	yes	no	no	no	no	yes	no	yes

182215956	chr1	113481134	113481174	21	41	1.952381	no	no	yes	no	no	no	no	no	no	yes
182215964	chr1	113525558	113525601	21	44	2.095238	no	no	yes	no	no	no	no	no	no	yes
182215966	chr1	113540501	113540549	24	49	2.041667	no	no	yes	no	no	no	no	yes	yes	yes
182215967	chr1	113543923	113543963	20	41	2.100000	no	no	yes	no	no	no	no	no	no	yes
182215980	chr1	113582967	113583003	19	37	1.947368	no	no	yes	no	no	no	no	no	no	yes
182215993	chr1	113616643	113616690	16	48	2.941176	no	no	yes	no	no	no	no	no	no	yes
182216011	chr1	113655457	113655497	19	41	2.263158	no	no	yes	no	no	no	no	no	no	yes
182216016	chr1	113677733	113677774	16	42	2.625000	no	no	yes	no	no	no	no	yes	no	yes
182216037	chr1	113735436	113735461	12	26	2.166667	no	no	yes	no	no	no	no	no	no	yes
182216038	chr1	113741476	113741529	28	54	1.928571	no	no	yes	no	no	no	no	no	no	yes
182216041	chr1	113746505	113746540	17	36	2.176471	no	no	yes	no	no	no	no	no	no	yes
182216050	chr1	113782109	113782151	22	43	1.954545	no	no	yes	no	no	no	no	yes	no	yes
182216051	chr1	113783456	113783496	14	41	2.928571	no	no	yes	no	no	no	no	yes	no	yes
182216053	chr1	113790421	113790446	13	26	2.000000	no	no	yes	no	no	no	no	yes	no	yes
182216055	chr1	113792813	113792853	17	41	2.500000	no	no	yes	no	no	no	no	yes	no	yes
182216058	chr1	113802650	113802680	15	31	2.066667	no	no	yes	no	no	no	no	yes	no	yes
182216074	chr1	113831206	113831262	27	57	2.148148	no	no	yes	no	no	no	no	no	no	yes
182216087	chr1	113849407	113849468	26	62	2.384615	no	no	yes	no	no	no	no	yes	yes	yes
182216101	chr1	113867231	113867256	12	26	2.166667	no	no	yes	no	no	no	no	yes	no	yes
182216103	chr1	113869968	113870004	18	37	2.055556	no	yes	yes	no	no	no	no	yes	yes	yes
182216105	chr1	113874676	113874702	14	27	1.928571	no	no	yes	no	no	no	no	yes	yes	yes
182216112	chr1	113913689	113913724	18	36	2.055556	no	yes	no	no	no	no	no	no	no	yes
182216121	chr1	113929442	113929471	15	30	2.000000	yes	no	no	no	yes	no	yes	yes	yes	yes
182216122	chr1	113932036	113932067	17	32	2.000000	no	no	yes	no	no	no	no	yes	no	yes
182216136	chr1	113977353	113977377	11	25	2.272727	no	yes	no	no	no	no	no	yes	no	yes

182216137	chr1	113982468	113982501	13	34	2.571429	no	no	yes	no	no	yes	no	yes	yes	yes
182216139	chr1	113984696	113984745	25	50	2.000000	no	no	yes	no	no	no	no	yes	no	yes
182216143	chr1	114001782	114001816	17	35	2.058824	no	no	yes	no	yes	no	no	yes	no	yes
182216146	chr1	114028052	114028098	15	47	3.133333	no	no	yes	no	no	no	no	no	no	yes
182216160	chr1	114054371	114054399	15	29	1.933333	no	no	no	no	no	no	no	yes	no	no
182216163	chr1	114054467	114054507	21	41	1.952381	no	no	no	no	no	no	no	yes	no	no
182216164	chr1	114060755	114060785	14	31	2.214286	no	no	no	no	no	no	no	yes	no	no
182216167	chr1	114075174	114075201	14	28	2.000000	no	no	no	no	no	no	no	yes	yes	no
182216169	chr1	114086722	114086750	14	29	2.071429	no	no	no	no	no	no	no	no	no	no
182216173	chr1	114110613	114110650	18	38	2.222222	no	no	yes	no	no	no	no	no	no	yes
182216177	chr1	114133106	114133160	22	55	2.500000	no	no	yes	no	no	no	no	yes	no	yes
182216194	chr1	114200432	114200477	22	46	2.136364	no	no	no	no	no	no	no	yes	no	no
182216227	chr1	114320562	114320595	17	34	2.000000	no	no	no	no	no	no	no	no	no	no
182216230	chr1	114340317	114340341	12	25	2.083333	no	no	no	no	no	no	no	no	yes	no
182216237	chr1	114344619	114344647	15	29	1.933333	no	no	no	no	no	no	no	no	yes	no
182216240	chr1	114347393	114347483	21	91	4.333333	no	no	no	no	no	no	no	yes	yes	no
182216241	chr1	114352971	114353008	20	38	1.950000	yes	no	no	no	no	no	no	yes	yes	no
182216252	chr1	114387318	114387351	16	34	2.125000	no	no	no	no	no	no	no	yes	yes	no
182216256	chr1	114394097	114394136	22	40	1.909091	no	yes	no	no	no	no	no	yes	no	yes
182216272	chr1	114429747	114429859	55	113	2.054545	no	no	yes	no	no	no	no	no	no	yes
182216275	chr1	114435545	114435586	20	42	2.100000	no	no	yes	no	no	no	no	yes	yes	yes
182216279	chr1	114443032	114443074	19	43	2.315789	no	no	yes	no	no	no	no	yes	no	yes
182216281	chr1	114449695	114449727	16	33	2.125000	no	no	yes	no	no	no	no	yes	no	yes
182216286	chr1	114461216	114461324	48	109	2.395833	no	no	yes	no	no	yes	no	no	yes	yes
182216288	chr1	114471630	114471665	19	36	1.947368	no	no	yes	no	no	no	no	no	no	yes

182216310	chr1	114511105	114511137	16	33	2.125000	no	no	no	no	yes	no	yes	yes	no	yes
182216334	chr1	114549713	114549751	20	39	1.950000	no	no	no	no	no	no	no	no	no	no
182216339	chr1	114556346	114556373	12	28	2.333333	no	no	no	no	no	no	no	no	no	no
182216340	chr1	114556361	114556431	21	71	3.318182	no	no	no	no	no	no	no	no	no	no
182216341	chr1	114561823	114561909	44	87	1.977273	no	no	no	no	no	no	no	no	no	no
182216380	chr1	114639953	114639989	17	37	2.176471	no	no	yes	no	no	no	no	no	no	yes
182216382	chr1	114650339	114650377	19	39	2.052632	no	no	yes	no	no	no	no	no	no	yes
182216418	chr1	114722549	114722644	49	96	1.959184	no	no	yes	no	no	no	no	yes	no	yes
182216419	chr1	114724212	114724242	14	31	2.214286	no	no	yes	no	yes	no	no	no	yes	yes
182216426	chr1	114752992	114753048	26	57	2.192308	no	no	yes	no	no	no	no	yes	no	yes
182216429	chr1	114772012	114772062	26	51	1.961538	no	yes	no	no	no	yes	no	yes	no	yes
182216430	chr1	114773594	114773624	16	31	1.937500	no	yes	no	no	no	yes	no	yes	yes	yes
182216457	chr1	114812684	114812733	22	50	2.260870	no	no	no	no	no	no	no	no	no	no
182216458	chr1	114812728	114812781	29	54	1.931034	no	no	no	no	no	no	no	yes	no	no
182216459	chr1	114822244	114822284	21	41	1.952381	no	no	no	no	no	no	no	yes	no	no
182216462	chr1	114827810	114827860	25	51	2.080000	no	no	no	no	no	no	no	no	no	no
182216465	chr1	114848773	114848807	18	35	1.944444	no	yes	yes	no	no	no	no	no	no	yes
182216466	chr1	114851545	114851599	18	55	3.055556	no	no	no	no	no	yes	no	no	no	no
182216485	chr1	114893248	114893399	52	152	2.909091	no	no	yes	no	no	no	no	yes	no	yes
182216490	chr1	114915078	114915131	24	54	2.250000	no	no	yes	no	no	no	no	no	no	yes
182216495	chr1	114946172	114946216	20	45	2.350000	no	no	yes	no	no	no	no	no	no	yes
182216509	chr1	115005069	115005112	18	44	2.444444	no	no	no	no	no	no	no	yes	no	no
182216515	chr1	115029552	115029586	14	35	2.466667	yes	no	no	no	no	no	no	no	no	no
182216516	chr1	115032723	115032765	11	43	3.833333	yes	no	yes	no	no	no	no	yes	no	yes
182216518	chr1	115032763	115032804	17	42	2.588235	yes	no	yes	no	no	no	no	yes	no	yes

182216523	chr1	115079056	115079087	15	32	2.133333	no	no	yes	no	no	no	no	yes	no	yes
182216533	chr1	115160024	115160125	41	102	2.523809	no	no	no	no	no	no	no	yes	no	no
182216535	chr1	115166960	115166994	13	35	2.692308	no	no	no	no	no	no	no	yes	yes	no
182216537	chr1	115173161	115173203	21	43	2.047619	no	no	no	no	no	no	no	no	no	no
182216544	chr1	115182172	115182218	19	47	2.473684	no	no	no	no	no	no	no	yes	no	no
182216549	chr1	115182239	115182319	41	81	2.000000	no	no	no	no	no	no	no	yes	no	no
182216554	chr1	115185778	115185840	33	63	1.909091	no	no	no	no	no	no	no	no	no	no
182216559	chr1	115211638	115211677	21	40	1.904762	no	no	no	no	no	no	no	yes	yes	no
182216564	chr1	115231455	115231493	20	39	1.950000	no	no	no	no	no	no	no	no	no	no
182216573	chr1	115250336	115250374	20	39	1.950000	no	no	no	no	no	no	no	no	no	no
182216580	chr1	115264753	115264794	20	42	2.100000	no	no	no	no	no	no	no	no	no	no
182216581	chr1	115267218	115267247	15	30	2.000000	no	no	no	no	no	no	no	no	yes	no
182216584	chr1	115281014	115281046	14	33	2.357143	no	no	yes	no	no	no	no	no	yes	yes
182216587	chr1	115299903	115299975	37	73	1.972973	no	no	yes	no	no	no	no	no	yes	yes
182216590	chr1	115303450	115303536	24	87	3.518518	no	no	yes	no	no	no	no	yes	no	yes
182216595	chr1	115307317	115307360	22	44	2.045455	no	no	yes	no	no	no	no	no	yes	yes
182216598	chr1	115319387	115319434	26	48	1.923077	no	no	yes	no	no	no	no	yes	no	yes
182216613	chr1	115351017	115351076	21	60	2.857143	no	no	yes	no	no	no	no	no	no	yes
182216619	chr1	115359333	115359366	16	34	2.125000	no	no	yes	no	no	no	no	no	no	yes
182216627	chr1	115378005	115378045	21	41	1.952381	no	no	no	no	no	no	no	no	no	no
182216630	chr1	115383217	115383247	16	31	1.937500	no	no	no	no	no	no	no	no	no	no
182216639	chr1	115418635	115418671	19	37	1.947368	no	no	no	no	no	no	no	no	no	no
182216645	chr1	115449344	115449388	24	45	1.916667	no	no	no	no	no	no	no	no	no	no
182216646	chr1	115452674	115452700	12	27	2.250000	no	no	no	no	no	no	no	no	yes	no
182216652	chr1	115479118	115479171	20	54	2.700000	yes	no	no	no	no	no	no	yes	no	no

182216658	chr1	115500815	115500856	21	42	2.000000	no	no	no	no	no	no	no	no	no	no
182216664	chr1	115533425	115533462	20	38	1.900000	no	no	no	no	no	no	no	no	no	no
182216667	chr1	115541440	115541468	15	29	1.933333	no	no	no	no	no	no	no	no	no	no
182216668	chr1	115545649	115545673	9	25	2.777778	no	no	no	no	no	no	no	yes	no	no
182216673	chr1	115560288	115560341	18	54	3.055556	no	no	no	no	no	no	no	yes	yes	no
182216678	chr1	115564394	115564427	12	34	2.833333	yes	no	no	no	no	no	no	yes	yes	no
182216697	chr1	115615443	115615481	15	39	2.600000	no	no	no	no	no	no	no	yes	no	no
182216700	chr1	115644505	115644568	20	64	3.090909	no	no	yes	no	no	no	no	no	no	yes
182216702	chr1	115655986	115656029	23	44	2.000000	no	no	yes	no	no	no	no	yes	yes	yes
182216703	chr1	115656533	115656567	18	35	1.944444	no	no	yes	no	no	no	no	yes	yes	yes
182216716	chr1	115706137	115706203	22	67	3.136364	no	no	yes	no	no	no	no	yes	no	yes
182216717	chr1	115721526	115721561	16	36	2.250000	no	no	yes	no	no	no	no	no	yes	yes
182216721	chr1	115731407	115731442	18	36	2.000000	no	no	yes	no	no	no	no	no	no	yes
182216723	chr1	115742193	115742249	26	57	2.269231	no	no	yes	no	no	no	no	no	no	yes
182216730	chr1	115768746	115768781	12	36	3.000000	no	no	no	no	yes	no	no	yes	yes	yes
182216732	chr1	115772936	115772986	20	51	2.600000	no	no	no	no	no	no	no	no	no	no
182216739	chr1	115800072	115800168	47	97	2.063830	no	no	no	no	no	no	no	yes	no	no
182216744	chr1	115826061	115826101	20	41	2.100000	no	no	no	no	no	no	no	no	no	no
182216745	chr1	115835097	115835150	22	54	2.454545	no	no	no	no	no	no	no	yes	yes	no
182216746	chr1	115836718	115836747	15	30	2.000000	no	yes	no	no	no	no	no	no	no	yes
182216747	chr1	115838902	115838952	28	51	1.964286	no	no	yes	no	yes	no	yes	yes	yes	yes
182216748	chr1	115839583	115839620	20	38	1.900000	no	no	yes	no	yes	no	yes	yes	yes	yes
182216751	chr1	115850650	115850793	48	144	3.000000	no	no	no	no	no	no	no	yes	no	no
182216759	chr1	115871739	115871784	20	46	2.300000	no	no	no	no	no	no	no	no	no	no
182216761	chr1	115879925	115879987	18	63	3.500000	no	no	no	no	no	no	no	yes	no	no

182216772	chr1	115895465	115895522	27	58	2.148148	no	no	no	no	no	no	no	no	no	no
182216784	chr1	115951756	115951781	13	26	2.000000	no	no	no	no	no	no	no	yes	yes	no
182216788	chr1	115960984	115961043	22	60	2.782609	no	no	no	no	no	no	no	no	no	no
182216793	chr1	115976469	115976500	15	32	2.133333	yes	no	no	no	no	no	no	yes	yes	no
182216795	chr1	115989255	115989291	10	37	3.800000	no	no	yes	no	no	no	no	no	no	yes
182216797	chr1	115991731	115991756	8	26	3.250000	no	no	yes	no	no	no	no	no	no	yes
182216799	chr1	115993409	115993457	10	49	4.900000	no	no	yes	no	no	no	no	no	no	yes
182216815	chr1	116031073	116031099	14	27	1.928571	no	no	yes	no	no	no	no	no	no	yes
182216817	chr1	116039563	116039594	16	32	2.062500	no	no	yes	no	no	no	no	no	no	yes
182216821	chr1	116065638	116065680	19	43	2.263158	no	no	yes	no	no	no	no	no	no	yes
182216822	chr1	116069718	116069751	18	34	1.944444	no	yes	no	no	no	no	no	no	no	yes
182216830	chr1	116100560	116100612	21	53	2.500000	no	no	no	no	no	no	no	no	no	no
182216832	chr1	116100692	116100830	36	139	3.891892	no	no	no	no	no	no	no	no	no	no
182216834	chr1	116100862	116100897	18	36	2.000000	no	no	no	no	no	no	no	no	no	no
182216841	chr1	116111857	116111955	36	99	2.692308	no	no	yes	no	no	no	no	yes	yes	yes
182216842	chr1	116117034	116117080	18	47	2.611111	no	no	yes	no	no	no	no	no	no	yes
182216848	chr1	116125860	116125895	15	36	2.400000	no	no	yes	no	no	no	no	yes	yes	yes
182216849	chr1	116127296	116127346	26	51	1.961538	no	no	yes	no	no	no	no	no	yes	yes
182216857	chr1	116153769	116153998	56	230	4.125000	no	no	no	no	no	no	no	yes	no	no
182216861	chr1	116162065	116162093	14	29	2.071429	no	no	no	no	no	no	no	yes	yes	no
182216862	chr1	116168278	116168333	23	56	2.434783	no	no	no	no	no	no	yes	yes	no	no
182216865	chr1	116184588	116184616	15	29	1.933333	no	no	no	no	no	no	no	no	no	no
182216885	chr1	116232766	116232802	19	37	1.947368	no	no	no	no	no	no	no	yes	no	no
182216886	chr1	116234556	116234681	62	126	2.032258	no	no	no	no	no	no	no	yes	yes	no
182216892	chr1	116245608	116245664	20	57	2.900000	no	no	no	no	no	no	no	no	no	no

182216903	chr1	116274288	116274337	18	50	2.777778	no	no	no	no	no	no	no	no	no	no
182216906	chr1	116275048	116275166	57	119	2.157895	no	no	no	no	no	no	no	no	no	no
182216917	chr1	116299108	116299149	19	42	2.210526	no	no	no	no	no	no	no	no	no	no
182216919	chr1	116300103	116300132	16	30	1.937500	no	no	no	no	no	no	no	no	no	no
182216929	chr1	116322245	116322294	22	50	2.272727	no	no	no	no	no	no	no	yes	no	no
182216931	chr1	116325492	116325533	23	42	1.913043	no	no	no	no	no	no	no	yes	no	no
182216932	chr1	116332867	116332960	35	94	2.685714	no	no	no	no	no	no	no	yes	no	no
182216933	chr1	116334207	116334235	10	29	2.900000	no	no	no	no	no	no	no	yes	yes	no
182216955	chr1	116369959	116370001	21	43	2.047619	no	no	no	no	no	no	no	yes	no	no
182216956	chr1	116370063	116370101	19	39	2.052632	no	no	no	no	no	no	no	yes	no	no
182216959	chr1	116379374	116379414	19	41	2.157895	no	no	yes	no	no	no	no	yes	yes	yes
182216961	chr1	116387576	116387621	22	46	2.090909	no	no	yes	no	no	no	no	yes	yes	yes
182216962	chr1	116391950	116391999	19	50	2.750000	no	no	yes	no	yes	no	no	no	no	yes
182216968	chr1	116423152	116423311	80	160	2.012500	no	no	no	no	no	yes	no	no	no	no
182216969	chr1	116425804	116425844	19	41	2.157895	no	no	yes	no	no	no	no	no	no	yes
182216976	chr1	116436915	116437174	32	260	8.242424	no	no	yes	no	no	no	no	yes	no	yes
182216984	chr1	116444655	116444687	11	33	3.000000	no	no	yes	no	no	no	no	no	no	yes
182216985	chr1	116449182	116449219	16	38	2.411765	no	no	yes	no	no	no	no	yes	no	yes
182216987	chr1	116461488	116461518	16	31	1.937500	no	no	yes	no	no	no	no	yes	no	yes
182216997	chr1	116476618	116476659	23	42	1.913043	no	no	yes	no	no	no	no	yes	yes	yes
182217021	chr1	116571265	116571372	53	108	2.056604	yes	no	no	no	no	no	no	yes	yes	no
182217023	chr1	116581549	116581586	19	38	2.000000	no	no	yes	no	no	no	no	no	yes	yes
182217039	chr1	116649494	116649533	17	40	2.352941	no	no	yes	no	no	no	no	yes	no	yes
182217042	chr1	116667517	116667569	7	53	7.250000	no	no	yes	no	no	no	yes	yes	no	yes
182217044	chr1	116667830	116667855	12	26	2.166667	yes	no	no	no	no	no	yes	yes	yes	no

182217045	chr1	116670420	116670450	16	31	1.937500	no	no	no	no	no	no	no	yes	no	no
182217050	chr1	116671812	116671837	12	26	2.166667	no	yes	no	no	no	yes	no	no	no	yes
182217054	chr1	116678885	116678920	16	36	2.312500	no	no	no	no	no	no	no	yes	no	no
182217058	chr1	116678917	116678952	19	36	1.947368	no	no	no	no	no	no	no	yes	no	no
182217077	chr1	116740631	116740667	19	37	2.000000	no	no	no	no	yes	no	no	no	no	yes
182217078	chr1	116740771	116740819	25	49	2.000000	no	no	no	no	yes	no	no	no	yes	yes
182217082	chr1	116740813	116741082	103	270	2.640777	no	no	no	no	yes	no	no	yes	yes	yes
182217086	chr1	116748194	116748266	30	73	2.433333	no	no	no	no	no	no	no	yes	no	no
182217089	chr1	116765715	116765747	13	33	2.615385	no	no	yes	no	no	no	no	yes	no	yes
182217095	chr1	116783522	116783553	16	32	2.000000	no	no	no	no	no	no	no	no	no	no
182217117	chr1	116816191	116816247	30	57	1.966667	no	no	no	no	no	no	no	no	no	no
182217122	chr1	116826225	116826349	41	125	3.048780	no	no	no	no	no	no	no	yes	yes	no
182217132	chr1	116856401	116856445	22	45	2.090909	no	no	no	no	no	no	no	no	no	no
182217143	chr1	116888635	116888669	18	35	1.944444	no	no	no	no	no	no	no	no	no	no
182217144	chr1	116891635	116891663	15	29	1.933333	no	no	no	no	no	no	no	yes	yes	no
182217151	chr1	116903155	116903185	16	31	1.937500	no	no	no	no	no	no	no	no	yes	no
182217153	chr1	116909989	116910017	15	29	1.933333	yes	no	no	no	yes	no	yes	yes	yes	yes
182217155	chr1	116917476	116917514	20	39	1.950000	no	no	yes	no	no	no	no	yes	no	yes
182217171	chr1	117011397	117011429	17	33	2.000000	no	no	yes	no	no	no	no	yes	yes	yes
182217174	chr1	117016124	117016157	12	34	2.833333	no	no	yes	no	no	no	no	yes	no	yes
182217176	chr1	117024069	117024097	15	29	1.933333	no	no	yes	no	no	no	no	yes	yes	yes
182217179	chr1	117025996	117026030	18	35	1.944444	no	no	yes	no	yes	yes	no	yes	yes	yes
182217182	chr1	117029302	117029351	15	50	3.312500	no	no	yes	no	no	no	no	yes	no	yes
182217192	chr1	117057930	117057974	13	45	3.461539	no	no	yes	no	no	no	no	no	no	yes
182217194	chr1	117071805	117071853	25	49	1.960000	no	no	yes	no	no	no	no	no	no	yes

182217195	chr1	117086027	117086086	28	60	2.142857	no	no	yes	no	no	no	no	no	yes	yes
182217199	chr1	117089221	117089283	21	63	3.045455	no	no	yes	no	no	no	no	no	no	yes
182217200	chr1	117101699	117101729	13	31	2.461539	no	yes	yes	no	no	yes	no	no	no	yes
182217203	chr1	117102714	117102754	16	41	2.562500	no	yes	no	no	no	yes	no	yes	no	yes
182217205	chr1	117102740	117102817	28	78	2.785714	no	yes	no	no	no	yes	no	yes	no	yes
182217206	chr1	117111204	117111237	17	34	2.000000	no	yes	yes	no	no	yes	no	yes	no	yes
182217207	chr1	117120506	117120542	13	37	3.000000	no	no	yes	no	no	no	no	no	no	yes
182217208	chr1	117123452	117123481	15	30	2.000000	yes	no	no	no	no	no	no	no	no	no
182217209	chr1	117125383	117125432	17	50	2.944444	no	no	no	no	no	no	no	no	no	no
182217230	chr1	117167941	117168007	32	67	2.218750	no	no	yes	no	no	no	no	no	no	yes
182217234	chr1	117176450	117176495	23	46	2.000000	no	no	yes	no	no	no	no	no	no	yes
182217239	chr1	117181803	117181874	16	72	4.500000	no	no	yes	no	no	no	no	yes	yes	yes
182217241	chr1	117187904	117187983	40	80	2.000000	no	no	yes	no	no	no	no	yes	no	yes
182217275	chr1	117325965	117326006	21	42	2.095238	no	no	no	no	no	no	no	yes	no	no
182217278	chr1	117328458	117328510	27	53	1.962963	no	no	no	no	no	no	no	no	yes	no
182217282	chr1	117330628	117330664	17	37	2.235294	no	no	no	no	no	no	no	no	no	no
182217284	chr1	117332505	117332574	22	70	3.181818	no	no	no	no	no	no	no	no	no	no
182217285	chr1	117333925	117334032	56	108	1.946429	no	no	no	no	no	no	no	no	yes	no
182217301	chr1	117360879	117360957	18	79	4.388889	no	no	no	no	no	no	no	yes	no	no
182217308	chr1	117385713	117385798	43	86	2.000000	no	no	yes	no	no	no	no	yes	no	yes
182217310	chr1	117397566	117397603	19	38	2.052632	no	no	yes	no	no	no	no	no	no	yes
182217313	chr1	117413849	117413887	20	39	1.950000	no	no	yes	no	no	no	no	no	no	yes
182217314	chr1	117414454	117414491	17	38	2.294118	no	no	yes	no	no	no	no	no	no	yes
182217320	chr1	117424536	117424595	27	60	2.222222	no	no	yes	no	no	no	no	no	no	yes
182217324	chr1	117425081	117425185	58	105	1.896552	no	no	yes	no	no	no	no	yes	no	yes

182217339	chr1	117441147	117441178	14	32	2.285714	no	no	yes	no	no	no	no	no	no	yes
182217357	chr1	117499731	117499780	11	50	4.727273	no	no	yes	no	no	no	no	no	no	yes
182217363	chr1	117513619	117513682	33	64	1.969697	no	no	yes	no	no	no	no	no	no	yes
182217364	chr1	117513967	117514003	13	37	3.000000	no	no	yes	no	no	no	no	no	no	yes
182217367	chr1	117526904	117526936	11	33	3.181818	no	yes	no	no	no	no	no	no	no	yes
182217385	chr1	117583399	117583478	42	80	1.952381	no	no	no	no	no	no	no	no	no	no
182217394	chr1	117584721	117584756	18	36	2.000000	no	no	no	no	no	no	no	no	no	no
182217401	chr1	117621805	117621843	20	39	1.950000	no	no	yes	no	no	no	no	no	no	yes
182217403	chr1	117622320	117622382	20	63	3.250000	no	no	yes	no	no	no	no	yes	yes	yes
182217404	chr1	117625990	117626023	17	34	2.000000	no	yes	no	no	no	no	no	no	no	yes
182217420	chr1	117673255	117673293	12	39	3.250000	no	no	no	no	no	no	no	yes	yes	no
182217425	chr1	117678051	117678086	17	36	2.117647	no	no	no	no	no	no	no	yes	no	no
182217430	chr1	117693419	117693449	13	31	2.384615	no	no	no	no	no	no	no	yes	no	no
182217446	chr1	117740183	117740305	56	123	2.214286	no	no	no	no	no	no	no	yes	no	no
182217451	chr1	117775026	117775054	13	29	2.230769	no	no	no	no	no	no	no	no	no	no
182217453	chr1	117784148	117784180	17	33	1.941176	no	no	no	no	no	no	no	yes	yes	no
182217459	chr1	117806853	117806894	14	42	3.000000	no	no	no	no	no	no	no	no	no	no
182217461	chr1	117812432	117812475	20	44	2.250000	no	no	no	no	no	no	no	yes	no	no
182217478	chr1	117897898	117898047	76	150	1.986842	no	no	yes	no	no	no	no	no	no	yes
182217481	chr1	117912438	117912487	23	50	2.217391	no	no	yes	no	no	yes	no	no	no	yes
182217487	chr1	117956699	117956735	18	37	2.055556	no	no	yes	no	no	no	no	no	no	yes
182217502	chr1	118021185	118021213	15	29	1.933333	no	no	yes	no	no	no	no	no	no	yes
182217510	chr1	118037264	118037296	17	33	1.941176	no	no	yes	no	no	no	no	no	no	yes
182217520	chr1	118068141	118068171	15	31	2.066667	no	no	yes	no	no	no	no	no	no	yes
182217522	chr1	118075236	118075288	26	53	2.038461	no	no	yes	no	no	no	no	yes	yes	yes

182217529	chr1	118107351	118107394	23	44	1.956522	no	no	yes	no	no	no	no	yes	no	yes
182217532	chr1	118116795	118116829	17	35	2.058824	no	no	yes	no	no	no	no	yes	no	yes
182217539	chr1	118131091	118131121	16	31	2.000000	no	no	yes	no	no	no	no	no	no	yes
182217543	chr1	118132630	118132677	19	48	2.500000	no	no	yes	no	no	no	no	no	no	yes
182217547	chr1	118138524	118138560	10	37	3.700000	no	no	yes	no	no	no	no	no	no	yes
182217549	chr1	118145637	118145689	28	53	1.928571	no	no	yes	no	no	no	no	yes	no	yes
182217550	chr1	118147157	118147200	22	44	2.000000	no	no	yes	no	no	no	no	no	no	yes
182217554	chr1	118154850	118154879	16	30	1.937500	no	no	yes	no	no	no	no	yes	no	yes
182217555	chr1	118155002	118155043	21	42	2.000000	no	no	yes	no	no	no	no	no	yes	yes
182217559	chr1	118186393	118186423	16	31	1.937500	no	no	yes	no	no	no	no	no	no	yes
182217591	chr1	118246885	118246917	17	33	1.941176	no	no	no	no	no	no	no	no	yes	no
182217592	chr1	118256007	118256048	20	42	2.100000	no	no	no	no	no	no	no	no	no	no
182217594	chr1	118273708	118273767	30	60	2.033333	no	no	no	no	no	no	no	no	no	no
182217604	chr1	118317593	118317634	16	42	2.687500	no	no	no	no	no	no	no	no	no	no
182217610	chr1	118319082	118319174	47	93	1.978723	no	no	no	no	no	no	no	yes	no	no
182217611	chr1	118319134	118319473	64	340	5.304348	no	no	no	no	no	no	no	yes	no	no
182217620	chr1	118331588	118331616	15	29	1.933333	no	no	no	no	no	no	no	no	no	no
182217621	chr1	118339658	118339749	42	92	2.190476	no	no	no	no	no	no	no	yes	yes	no
182217624	chr1	118367503	118367544	19	42	2.263158	no	no	no	no	no	no	no	no	no	no
182217631	chr1	118382950	118382994	22	45	2.045455	no	no	no	no	no	no	no	no	no	no
182217647	chr1	118415622	118415666	21	45	2.142857	no	no	no	no	no	no	no	no	no	no
182217652	chr1	118430328	118430421	50	94	1.960000	no	no	no	no	no	no	no	yes	no	no
182217664	chr1	118470478	118470570	48	93	1.937500	no	no	no	no	no	no	no	no	no	no
182217669	chr1	118489634	118489662	15	29	1.933333	no	no	no	no	no	no	no	no	no	no
182217673	chr1	118503082	118503191	26	110	4.230769	no	no	no	no	no	no	no	no	no	no

182217686	chr1	118537016	118537067	22	52	2.363636	no	no	no	no	no	no	no	no	no	no
182217689	chr1	118558510	118558559	25	50	2.040000	no	no	no	no	no	no	no	no	no	no
182217690	chr1	118564614	118564651	18	38	2.166667	no	no	no	no	no	no	no	yes	yes	no
182217693	chr1	118583135	118583169	18	35	1.944444	no	no	no	no	no	no	no	no	no	no
182217710	chr1	118644825	118644849	11	25	2.272727	no	no	no	no	no	no	no	no	no	no
182217720	chr1	118683996	118684024	13	29	2.230769	no	no	no	no	no	no	no	no	no	no
182217726	chr1	118691859	118691895	15	37	2.437500	no	no	no	no	no	no	no	no	no	no
182217738	chr1	118714173	118714305	24	133	5.392857	no	no	no	no	no	yes	no	no	no	no
182217752	chr1	118739093	118739127	17	35	2.058824	no	no	no	no	no	no	no	no	no	no
182217753	chr1	118739124	118739173	20	50	2.500000	no	no	no	no	no	no	no	no	no	no
182217755	chr1	118746269	118746305	17	37	2.294118	no	no	no	no	no	no	no	no	no	no
182217758	chr1	118757242	118757280	19	39	2.052632	no	no	no	no	no	no	no	no	no	no
182217773	chr1	118815124	118815164	15	41	2.800000	no	no	no	no	no	no	no	no	no	no
182217774	chr1	118818579	118818616	17	38	2.235294	no	no	no	no	no	no	no	no	no	no
182217776	chr1	118823871	118823902	16	32	2.062500	no	no	no	no	no	no	no	no	no	no
182217783	chr1	118846268	118846315	24	48	2.083333	no	no	no	no	no	no	no	no	no	no
182217788	chr1	118866633	118866708	34	76	2.294118	no	no	no	no	no	no	no	no	no	no
182217790	chr1	118890427	118890455	9	29	3.222222	no	no	yes	no	no	no	no	no	yes	yes
182217792	chr1	118902075	118902123	17	49	2.882353	no	no	yes	no	no	no	no	no	no	yes
182217801	chr1	118933244	118933278	18	35	1.944444	no	no	yes	no	no	no	no	yes	yes	yes
182217804	chr1	118953299	118953332	18	34	2.000000	no	no	yes	no	no	no	no	no	no	yes
182217808	chr1	118979760	118979803	16	44	2.812500	no	no	yes	no	no	no	yes	yes	yes	yes
182217809	chr1	118979972	118979997	8	26	3.250000	no	no	yes	no	no	no	yes	yes	yes	yes
182217812	chr1	118984495	118984538	22	44	2.000000	no	no	yes	no	no	no	yes	yes	yes	yes
182217818	chr1	118994877	118994916	21	40	1.904762	no	no	no	no	no	no	no	no	no	no

182217819	chr1	118998494	118998543	19	50	2.619048	no	no	no	no	no	no	no	yes	no	no
182217823	chr1	119005302	119005342	20	41	2.050000	no	no	no	no	no	no	no	yes	no	no
182217826	chr1	119033881	119033920	20	40	2.000000	no	no	yes	no	no	yes	no	no	no	yes
182217827	chr1	119035537	119035583	22	47	2.272727	no	no	yes	no	no	no	no	no	no	yes
182217834	chr1	119039423	119039473	18	51	2.833333	no	no	yes	no	no	no	no	no	no	yes
182217842	chr1	119055762	119055807	23	46	2.000000	no	no	yes	no	no	no	no	no	no	yes
182217846	chr1	119056520	119056577	24	58	2.500000	no	no	yes	no	no	no	no	no	no	yes
182217847	chr1	119061725	119061757	17	33	1.941176	no	no	yes	no	no	no	no	no	no	yes
182217848	chr1	119067398	119067430	16	33	2.125000	no	no	yes	no	no	no	no	no	no	yes
182217852	chr1	119075583	119075620	21	38	1.952381	no	no	yes	no	no	no	no	no	no	yes
182217854	chr1	119094461	119094517	31	57	1.903226	no	no	yes	no	no	no	no	no	no	yes
182217859	chr1	119103934	119103997	21	64	3.090909	no	no	yes	no	no	no	no	no	no	yes
182217862	chr1	119104135	119104159	10	25	2.500000	no	no	yes	no	no	no	no	no	no	yes
182217877	chr1	119136089	119136125	19	37	2.000000	no	no	yes	no	no	no	no	no	no	yes
182217878	chr1	119146427	119146468	20	42	2.100000	no	yes	yes	no	no	no	no	no	no	yes
182217889	chr1	119164475	119164504	13	30	2.307692	no	no	yes	no	no	no	no	no	no	yes
182217925	chr1	119203640	119203672	7	33	4.714286	no	no	yes	no	no	no	no	no	no	yes
182217928	chr1	119216894	119216946	25	53	2.120000	no	no	yes	no	no	no	no	no	no	yes
182217930	chr1	119236093	119236197	41	105	2.560976	no	no	yes	no	no	no	no	yes	no	yes
182217943	chr1	119262232	119262269	18	38	2.111111	no	no	yes	no	no	no	no	no	no	yes
182217947	chr1	119264616	119264764	48	149	3.104167	no	no	yes	no	no	no	no	no	no	yes
182217952	chr1	119268269	119268312	15	44	3.000000	no	no	yes	no	no	no	no	no	no	yes
182217953	chr1	119274423	119274454	16	32	2.000000	no	no	yes	no	no	no	no	no	no	yes
182217954	chr1	119278097	119278131	17	35	2.058824	no	no	yes	no	no	no	no	yes	no	yes
182217962	chr1	119296908	119297010	50	103	2.060000	no	no	yes	no	no	no	no	no	yes	yes

182217979	chr1	119320315	119320370	28	56	2.035714	no	no	yes	no	no	no	no	no	no	yes
182217982	chr1	119327353	119327396	19	44	2.368421	yes	no	yes	no	no	no	no	yes	yes	yes
182217992	chr1	119375160	119375212	26	53	2.038461	no	no	yes	no	no	no	no	no	no	yes
182218009	chr1	119436653	119436703	26	51	2.000000	no	no	no	no	no	no	no	no	no	no
182218017	chr1	119451023	119451108	37	86	2.432432	no	no	no	no	no	no	no	no	no	no
182218021	chr1	119460549	119460579	16	31	1.937500	no	no	no	no	no	no	no	no	no	no
182218024	chr1	119466695	119466746	26	52	2.000000	yes	no	no	no	no	no	no	yes	yes	no
182218026	chr1	119467861	119467895	18	35	1.944444	no	no	yes	no	no	no	no	yes	no	yes
182218037	chr1	119508157	119508209	14	53	3.785714	no	no	yes	no	no	yes	no	no	no	yes
182218038	chr1	119508199	119508239	20	41	2.050000	no	no	yes	no	no	yes	no	no	no	yes
182218041	chr1	119517736	119517942	67	207	3.089552	no	no	no	no	no	no	no	yes	yes	no
182218042	chr1	119528878	119528911	17	34	2.058824	no	no	no	no	no	no	no	no	no	no
182218047	chr1	119539227	119539278	24	52	2.166667	no	no	yes	no	no	no	no	no	no	yes
182218048	chr1	119541628	119541666	19	39	2.105263	no	no	yes	no	no	no	no	yes	yes	yes
182218050	chr1	119542672	119542707	15	36	2.466667	no	no	yes	no	no	no	no	yes	yes	yes
182218052	chr1	119544609	119544673	31	65	2.096774	no	no	yes	no	no	no	no	yes	no	yes
182218066	chr1	119605160	119605186	12	27	2.250000	no	no	yes	no	no	no	no	yes	yes	yes
182218072	chr1	119622838	119623293	254	456	1.811024	no	no	no	yes	no	no	yes	yes	yes	yes
182218075	chr1	119623574	119623602	15	29	1.933333	no	no	no	yes	no	no	yes	no	no	yes
182218076	chr1	119631584	119631622	11	39	3.545455	no	no	yes	no	no	no	yes	yes	yes	yes
182218079	chr1	119642802	119642836	18	35	1.944444	no	no	yes	no	no	no	no	no	no	yes
182218082	chr1	119647168	119647198	16	31	1.937500	no	no	yes	no	no	no	no	yes	yes	yes
182218083	chr1	119648177	119648256	8	80	10.000000	yes	no	no	no	yes	no	yes	yes	yes	yes
182218085	chr1	119650198	119650248	25	51	2.040000	no	no	yes	no	no	no	no	no	no	yes
182218091	chr1	119683956	119683997	21	42	2.000000	no	no	yes	no	no	no	no	no	yes	yes

182218092	chr1	119686495	119686523	10	29	2.900000	no	no	yes	no	no	no	no	yes	yes	yes
182218101	chr1	119699112	119699144	17	33	2.000000	no	no	yes	no	no	no	no	yes	no	yes
182218103	chr1	119712454	119712480	13	27	2.076923	yes	no	yes	no	no	no	yes	yes	yes	yes
182218107	chr1	119723544	119723588	23	45	1.956522	no	no	yes	no	no	yes	no	yes	yes	yes
182218112	chr1	119736364	119736438	33	75	2.272727	yes	no	yes	no	no	yes	no	yes	no	yes
182218139	chr1	119812364	119812392	15	29	1.933333	yes	no	no	no	no	no	no	yes	no	no
182218142	chr1	119819994	119820036	20	43	2.250000	no	no	no	no	no	no	no	no	no	no
182218157	chr1	119887339	119887370	16	32	2.062500	no	no	no	no	no	yes	no	no	no	no
182218163	chr1	119894016	119894162	27	147	5.444445	no	no	no	yes	no	no	no	yes	yes	yes
182218167	chr1	119917031	119917120	28	90	3.172414	no	no	yes	no	no	no	no	yes	no	yes
182218171	chr1	119931974	119932028	18	55	3.055556	no	no	yes	no	no	no	no	no	no	yes
182218173	chr1	119934845	119934944	50	100	2.000000	no	no	yes	no	no	yes	no	no	no	yes
182218174	chr1	119938121	119938158	20	38	1.950000	yes	no	yes	no	no	no	no	no	no	yes
182218178	chr1	119949788	119949812	13	25	1.923077	no	no	yes	no	no	yes	no	no	no	yes
182218181	chr1	119958924	119959056	63	133	2.126984	no	no	yes	no	no	no	no	no	no	yes
182218183	chr1	119971959	119971987	9	29	3.222222	no	no	yes	no	no	no	no	yes	no	yes
182218186	chr1	119972100	119972150	21	51	2.428571	no	no	yes	no	no	no	no	yes	yes	yes
182218218	chr1	120132893	120132937	22	45	2.045455	no	no	no	no	no	no	no	no	yes	no
182218225	chr1	120159494	120159555	33	62	1.939394	no	no	yes	no	no	no	no	no	no	yes
182218313	chr1	120431302	120431381	38	80	2.105263	no	no	yes	no	no	yes	no	no	no	yes
182218343	chr1	120497968	120497999	14	32	2.357143	no	no	yes	no	no	no	no	yes	no	yes
182218344	chr1	120498040	120498680	28	641	22.892857	no	no	yes	no	no	no	no	yes	no	yes
182218358	chr1	120551235	120551271	19	37	1.947368	no	no	yes	no	no	no	no	no	no	yes
182218450	chr1	120823356	120828299	1540	4944	3.207692	no	no	yes	yes	no	no	no	no	no	yes
182218550	chr1	121059716	121059753	20	38	1.900000	yes	no	no	no	no	no	no	no	no	no

182218559	chr1	121095142	121095195	28	54	1.928571	no	yes	yes	no	no	yes	no	yes	no	yes
182218665	chr1	121395937	121396006	24	70	2.916667	yes	no	yes	no	no	no	yes	yes	yes	yes
182218668	chr1	121396080	121396161	33	82	2.470588	yes	no	yes	no	no	no	yes	yes	yes	yes
182218676	chr1	121396366	121396652	161	287	1.838509	yes	no	yes	no	no	yes	yes	yes	yes	yes
182218686	chr1	121407493	121407539	17	47	2.764706	no	no	yes	no	no	no	no	no	no	yes
182218700	chr1	121431505	121431581	37	77	2.108108	no	no	yes	no	no	no	no	yes	no	yes
182218704	chr1	121432180	121432289	24	110	4.518518	no	no	yes	no	no	no	no	no	no	yes
182218706	chr1	121441611	121441648	20	38	1.900000	no	no	yes	no	no	no	no	no	no	yes
182218709	chr1	121445285	121445316	16	32	2.000000	no	no	yes	no	no	no	no	no	no	yes
182218714	chr1	121459098	121459150	17	53	3.117647	no	no	yes	no	no	no	no	yes	no	yes
182218724	chr1	121494180	121494232	26	53	2.115385	no	no	no	no	no	yes	no	no	no	no
182218739	chr1	121517987	121518019	8	33	4.125000	no	no	no	no	no	yes	no	yes	yes	no
182218768	chr1	121742260	121775784	340	33525	98.484344	no	no	no	no	no	no	no	yes	no	no
182218774	chr1	121781191	121796538	1867	15348	8.044728	no	no	no	no	no	no	no	no	no	no
182218778	chr1	121803295	121806584	1608	3290	1.997076	no	no	no	no	no	no	no	no	no	no
182218780	chr1	121810456	121811107	343	652	1.915452	no	no	no	no	no	no	no	no	no	no
182218783	chr1	121813473	121823516	1866	10044	5.551872	no	no	no	no	no	no	no	no	no	no
182218798	chr1	121845942	121851570	1869	5629	3.021402	no	no	no	no	no	no	no	no	no	no
182218820	chr1	121930791	121935105	1866	4315	2.327974	no	no	no	no	no	no	no	no	no	no
182218824	chr1	121940636	121944269	1870	3634	1.956684	no	no	no	no	no	no	no	no	no	no
182218833	chr1	121958116	121963443	1866	5328	2.865096	no	no	no	no	no	no	no	no	no	no
182218834	chr1	121961880	121965119	1685	3240	1.934757	no	no	no	no	no	no	no	no	no	no
182218848	chr1	124953023	124953073	27	51	1.925926	no	no	no	no	no	no	no	no	no	no
182218856	chr1	124979800	124979841	21	42	2.047619	no	no	no	no	no	no	no	no	no	no
182218857	chr1	124980190	124980231	15	42	2.800000	no	no	no	no	no	no	no	no	no	no

182218859	chr1	124980248	124980280	16	33	2.062500	no	no	no	no	no	no	no	no	no	no
182218866	chr1	125013529	125015250	171	1722	10.092485	no	no	no	no	no	no	no	no	no	no
182218872	chr1	125051012	125051474	234	463	1.995726	no	no	no	no	no	no	no	no	no	no
182218876	chr1	125065056	125065090	13	35	2.692308	no	no	no	no	no	no	no	no	no	no
182218889	chr1	125069358	125069443	39	86	2.205128	no	no	no	no	no	no	no	no	no	no
182218940	chr1	125085860	125086168	161	309	1.944099	no	no	no	no	no	no	yes	no	yes	no
182218944	chr1	125086229	125086295	17	67	3.941176	no	no	no	no	no	no	yes	no	no	no
182218948	chr1	125086466	125086505	16	40	2.470588	no	no	no	no	no	no	yes	no	no	no
182218955	chr1	125087256	125087289	16	34	2.187500	no	no	no	no	no	no	yes	no	no	no
182218960	chr1	125087643	125087705	26	63	2.500000	no	no	no	no	no	no	yes	no	no	no
182218963	chr1	125087798	125087848	26	51	2.000000	no	no	no	no	no	no	yes	no	no	no
182218965	chr1	125087891	125087970	41	80	1.975610	no	no	no	no	no	no	yes	no	no	no
182218971	chr1	125097865	125097900	16	36	2.250000	no	no	no	no	no	no	no	no	no	no
182218981	chr1	125116430	125116476	21	47	2.285714	no	no	no	no	no	no	no	no	no	no
182218984	chr1	125118363	125118427	32	65	2.031250	no	no	no	no	no	no	yes	no	no	no
182218987	chr1	125118413	125118503	38	91	2.421053	no	no	no	no	no	no	yes	no	no	no
182219001	chr1	125119218	125119359	76	142	1.894737	no	no	no	no	no	no	yes	no	no	no
182219029	chr1	125149361	125149396	18	36	2.000000	no	no	no	no	no	no	no	no	no	no
182219035	chr1	125152949	125153033	39	85	2.205128	no	no	no	no	no	no	no	no	yes	no
182219039	chr1	125155715	125155951	114	237	2.078947	no	no	no	no	no	no	no	yes	no	no
182219042	chr1	125158409	125158448	21	40	1.904762	no	no	no	no	no	no	no	no	no	no
182219049	chr1	125167629	125167709	43	81	1.930233	no	no	no	no	no	no	no	yes	yes	no
182219053	chr1	125170694	125170776	42	83	1.976190	no	no	no	no	no	no	no	yes	no	no
182219099	chr1	143196753	143196783	16	31	1.937500	no	no	no	no	no	no	no	no	no	no
182219167	chr1	143233668	143233750	42	83	1.976190	no	no	no	no	no	no	no	yes	no	no

182219190	chr1	143245119	143245214	23	96	4.173913	no	no	no	no	no	no	no	no	yes	no
182219212	chr1	143258784	143258815	16	32	2.000000	no	no	no	no	no	no	no	yes	no	no
182219248	chr1	143274566	143274635	27	70	2.666667	no	no	no	no	no	no	no	no	no	no
182219256	chr1	143275630	143275674	24	45	1.958333	no	no	no	no	no	no	no	yes	yes	no
182219259	chr1	143276058	143276133	39	76	1.948718	no	no	no	no	no	no	no	no	no	no
182219419	chr1	143497104	143497159	27	56	2.074074	no	no	yes	no	no	no	yes	no	yes	yes
182219429	chr1	143497476	143497529	23	54	2.391304	no	no	yes	no	no	no	yes	yes	yes	yes
182219475	chr1	143548117	143548167	27	51	1.962963	no	no	yes	no	no	no	no	no	no	yes
182219476	chr1	143551384	143551429	16	46	2.875000	no	no	yes	no	no	no	no	no	no	yes
182219480	chr1	143568117	143568164	23	48	2.130435	no	no	yes	no	no	no	no	no	no	yes
182219488	chr1	143589099	143589144	22	46	2.090909	no	no	no	no	no	no	no	no	no	no
182219499	chr1	143609312	143609338	14	27	1.928571	no	no	yes	no	no	no	no	no	no	yes
182219504	chr1	143622758	143623036	24	279	11.833333	no	no	yes	no	no	no	no	no	no	yes
182219532	chr1	143651463	143651510	9	48	5.333333	no	no	no	no	no	yes	no	yes	no	no
182219536	chr1	143659471	143659507	10	37	3.700000	no	no	no	no	no	no	no	no	no	no
182219542	chr1	143687953	143687993	19	41	2.157895	no	no	no	no	no	no	no	no	no	no
182219584	chr1	143735759	143735844	42	86	2.047619	yes	no	no	no	no	no	no	yes	yes	no
182219617	chr1	143840878	143840961	40	84	2.150000	no	no	no	no	no	no	no	no	no	no
182219689	chr1	144076471	144076519	17	49	2.882353	no	no	no	no	no	no	yes	no	no	no
182219701	chr1	144076892	144076992	44	101	2.363636	no	no	no	no	no	no	yes	no	no	no
182219709	chr1	144077380	144077428	24	49	2.083333	no	no	no	no	no	no	yes	yes	yes	no
182219732	chr1	144101259	144101291	14	33	2.357143	no	no	no	no	no	no	no	no	no	no
182219941	chr1	144323485	144323613	58	129	2.241379	no	no	no	no	no	no	yes	yes	no	no
182219975	chr1	144412468	144412503	15	36	2.400000	yes	no	no	no	no	no	no	yes	no	no
182219978	chr1	144415839	144415863	10	25	2.500000	no	no	no	no	no	no	yes	no	no	no

182220011	chr1	144472158	144472185	14	28	2.000000	no	no	no	no	no	yes	no	no	no	no
182220036	chr1	144541944	144542120	28	177	6.300000	no	no	no	no	no	no	no	no	no	no
182220106	chr1	144642709	144643233	246	525	2.166667	yes	no	yes	no	no	no	yes	yes	no	yes
182220153	chr1	144735880	144735951	39	72	1.948718	no	no	yes	no	no	no	no	no	no	yes
182220175	chr1	144783607	144783643	19	37	2.000000	no	no	yes	no	no	no	no	no	no	yes
182220276	chr1	144850934	144851343	190	410	2.167539	no	no	yes	no	no	no	no	no	no	yes
182220301	chr1	144859417	144859455	16	39	2.562500	no	no	yes	no	no	no	no	no	no	yes
182220313	chr1	144882579	144882700	54	122	2.254545	no	no	yes	no	no	no	yes	yes	no	yes
182220319	chr1	144882831	144882908	40	78	1.975000	no	no	yes	no	no	no	yes	yes	no	yes
182220335	chr1	144883778	144884105	97	328	3.407767	no	no	yes	no	no	no	yes	no	no	yes
182220359	chr1	144941491	144941528	16	38	2.437500	no	no	yes	no	no	no	no	no	no	yes
182220664	chr1	145419034	145419184	48	151	3.145833	no	no	no	no	no	no	no	no	no	no
182220683	chr1	145451888	145451938	26	51	2.000000	no	no	no	no	no	no	no	yes	yes	no
182220729	chr1	145512433	145512473	16	41	2.562500	no	no	yes	no	no	no	no	no	no	yes
182220735	chr1	145519836	145519897	33	62	1.909091	no	no	yes	no	no	no	no	no	no	yes
182220738	chr1	145525709	145525798	35	90	2.571429	no	no	no	no	no	no	no	no	no	no
182220740	chr1	145525864	145525904	21	41	1.952381	no	no	no	no	no	no	no	yes	no	no
182220758	chr1	145572537	145572574	19	38	2.105263	no	no	no	no	yes	no	no	no	no	yes
182220797	chr1	145697346	145697383	14	38	2.785714	no	no	yes	no	no	no	no	yes	no	yes
182220831	chr1	145773794	145773832	20	39	1.950000	no	no	yes	no	yes	no	no	yes	no	yes
182220834	chr1	145788773	145788803	14	31	2.214286	no	no	yes	no	no	no	no	no	no	yes
182220839	chr1	145791150	145791210	30	61	2.033333	no	no	yes	no	no	no	no	no	no	yes
182220844	chr1	145801731	145801796	23	66	3.043478	no	no	yes	no	no	no	no	no	no	yes
182220853	chr1	145816722	145816759	17	38	2.294118	no	no	yes	no	no	no	no	no	no	yes
182220861	chr1	145858773	145858801	14	29	2.071429	no	no	yes	no	no	no	yes	yes	yes	yes

182220863	chr1	145868197	145868246	24	50	2.083333	no	no	yes	no	no	no	no	yes	no	yes
182220876	chr1	145895417	145895606	96	190	2.000000	no	no	yes	no	no	no	no	yes	no	yes
182220877	chr1	145898234	145898258	8	25	3.125000	no	no	yes	no	no	no	no	yes	no	yes
182220885	chr1	145909438	145909717	40	280	6.976744	no	no	yes	no	no	no	no	yes	no	yes
182220903	chr1	145944501	145944533	14	33	2.357143	no	no	yes	no	no	no	no	no	no	yes
182220907	chr1	145958880	145958915	18	36	2.000000	yes	no	yes	no	no	yes	no	yes	yes	yes
182220909	chr1	145965395	145965426	16	32	2.000000	yes	no	yes	no	no	yes	no	yes	no	yes
182220917	chr1	145974966	145975007	23	42	1.913043	no	yes	yes	yes	no	no	no	no	yes	yes
182220919	chr1	145978015	145978071	30	57	1.900000	no	yes	no	yes	no	yes	no	yes	no	yes
182220922	chr1	145989043	145989091	7	49	6.777778	no	no	no	no	no	no	no	yes	yes	no
182220925	chr1	145996802	145996841	9	40	4.444445	yes	no	no	no	no	no	no	yes	no	no
182220936	chr1	146023098	146023170	34	73	2.147059	no	no	yes	no	no	no	no	yes	no	yes
182220943	chr1	146037334	146037396	32	63	1.968750	yes	no	no	no	no	no	no	yes	yes	no
182220944	chr1	146037733	146037766	15	34	2.333333	yes	no	no	no	no	no	no	yes	no	no
182220949	chr1	146050493	146050543	24	51	2.166667	no	no	no	no	yes	no	no	yes	no	yes
182221081	chr1	146376700	146376732	16	33	2.062500	yes	no	yes	no	no	no	no	yes	yes	yes
182221126	chr1	146481634	146481717	42	84	2.000000	no	no	yes	no	no	no	no	no	no	yes
182221152	chr1	146551757	146551820	32	64	2.000000	no	no	yes	no	no	no	no	no	no	yes
182221216	chr1	146728280	146728318	12	39	3.416667	no	no	yes	no	no	no	no	no	no	yes
182221221	chr1	146736795	146736847	12	53	4.500000	no	no	yes	no	no	no	no	no	no	yes
182221224	chr1	146751650	146751681	16	32	2.000000	no	no	yes	no	no	no	no	no	no	yes
182221263	chr1	146880544	146880605	24	62	2.583333	no	no	yes	no	no	no	no	no	no	yes
182221281	chr1	146938524	146938710	88	187	2.125000	yes	no	no	no	no	no	yes	yes	yes	no
182221294	chr1	146957910	146957996	45	87	1.955556	yes	no	yes	no	no	no	no	no	no	yes
182221334	chr1	147072980	147073025	20	46	2.300000	no	no	no	no	no	no	no	yes	no	no

182221335	chr1	147078187	147078249	34	63	1.911765	no	no	no	no	no	no	no	yes	yes	no
182221336	chr1	147078558	147078593	18	36	2.000000	no	no	no	no	no	no	no	yes	yes	no
182221347	chr1	147110599	147110638	20	40	2.050000	no	no	yes	no	no	no	no	yes	no	yes
182221360	chr1	147147966	147148049	41	84	2.097561	no	no	no	no	no	no	no	no	no	no
182221361	chr1	147149184	147149220	11	37	3.363636	no	no	no	no	no	no	no	no	no	no
182221363	chr1	147161794	147161820	12	27	2.250000	no	no	yes	no	no	yes	no	no	no	yes
182221364	chr1	147169492	147169586	46	95	2.108696	no	no	yes	no	no	no	no	yes	no	yes
182221366	chr1	147180146	147180179	18	34	1.944444	no	no	yes	no	no	yes	no	no	no	yes
182221368	chr1	147185318	147185356	20	39	1.950000	no	no	yes	no	no	yes	no	no	no	yes
182221369	chr1	147196585	147196617	9	33	3.666667	no	no	yes	no	no	no	no	no	no	yes
182221372	chr1	147199107	147199148	22	42	1.909091	no	no	yes	no	no	no	no	yes	no	yes
182221378	chr1	147212651	147212692	22	42	2.090909	no	no	yes	no	no	yes	no	yes	no	yes
182221400	chr1	147263571	147263803	118	233	1.983051	no	no	yes	no	no	no	no	yes	no	yes
182221403	chr1	147287251	147287304	24	54	2.250000	no	no	yes	no	no	yes	no	yes	yes	yes
182221413	chr1	147341682	147341717	10	36	3.600000	no	no	yes	no	no	no	no	no	yes	yes
182221427	chr1	147416349	147416416	22	68	3.083333	no	no	yes	no	no	no	no	no	no	yes
182221435	chr1	147452677	147452713	17	37	2.176471	no	no	yes	no	no	no	no	yes	no	yes
182221455	chr1	147500772	147500856	32	85	2.656250	no	no	yes	no	no	no	no	yes	no	yes
182221459	chr1	147507547	147507589	14	43	3.071429	no	no	yes	no	no	no	no	yes	yes	yes
182221462	chr1	147519252	147519320	32	69	2.156250	no	no	no	no	no	no	no	no	no	no
182221465	chr1	147530551	147530604	27	54	2.000000	no	no	no	no	no	no	no	no	no	no
182221472	chr1	147544690	147544743	27	54	2.000000	no	no	yes	no	no	no	no	no	yes	yes
182221473	chr1	147550677	147550711	13	35	2.692308	no	no	yes	no	no	no	no	yes	yes	yes
182221477	chr1	147566101	147566133	15	33	2.200000	no	no	yes	no	no	no	no	no	no	yes
182221486	chr1	147599339	147599379	23	41	1.913043	yes	no	yes	no	no	no	no	yes	yes	yes

182221487	chr1	147599881	147599934	24	54	2.250000	yes	no	yes	no	no	no	yes	yes	yes	yes
182221490	chr1	147605337	147605369	17	33	2.058824	no	no	yes	no	no	no	no	no	no	yes
182221491	chr1	147617234	147617263	14	30	2.142857	no	no	yes	no	no	no	no	yes	no	yes
182221492	chr1	147625161	147625193	14	33	2.357143	no	yes	no	no	no	no	no	yes	no	yes
182221501	chr1	147689976	147690013	18	38	2.166667	no	no	no	no	no	yes	no	no	no	no
182221508	chr1	147718880	147718908	13	29	2.230769	no	no	no	no	no	no	no	yes	yes	no
182221514	chr1	147735513	147735548	17	36	2.176471	no	no	no	no	no	no	no	no	yes	no
182221518	chr1	147750955	147750981	13	27	2.076923	no	no	no	no	no	no	no	yes	yes	no
182221519	chr1	147757496	147757554	25	59	2.360000	no	yes	yes	no	no	yes	no	yes	yes	yes
182221520	chr1	147760853	147760992	42	140	3.357143	yes	no	yes	no	no	no	no	yes	yes	yes
182221522	chr1	147780678	147780728	25	51	2.040000	no	no	yes	no	no	no	no	no	yes	yes
182221532	chr1	147819488	147819530	14	43	3.071429	no	no	no	no	no	no	no	no	no	no
182221533	chr1	147826434	147826467	17	34	2.000000	no	no	no	no	no	no	no	no	no	no
182221547	chr1	147848244	147848274	16	31	1.937500	no	no	no	no	no	no	no	yes	no	no
182221552	chr1	147876061	147876104	21	44	2.095238	no	no	no	no	no	no	no	no	no	no
182221555	chr1	147883987	147884017	16	31	2.000000	no	no	no	no	no	no	no	no	no	no
182221556	chr1	147891861	147891907	15	47	3.133333	no	no	no	no	no	no	no	no	no	no
182221586	chr1	147958137	147958161	13	25	1.923077	no	no	yes	no	no	no	no	no	no	yes
182221598	chr1	148011610	148011651	18	42	2.333333	yes	no	yes	no	yes	no	no	no	no	yes
182221599	chr1	148013407	148013474	33	68	2.060606	no	yes	yes	no	no	yes	no	yes	yes	yes
182221608	chr1	148029049	148029122	31	74	2.468750	no	no	no	no	no	no	no	no	no	no
182221649	chr1	148105758	148109173	1558	3416	2.213291	no	yes	yes	yes	no	no	no	no	no	yes
182221720	chr1	148295012	148295268	79	257	3.237500	yes	yes	yes	no	no	no	no	no	no	yes
182221726	chr1	148310371	148310406	17	36	2.117647	no	no	yes	no	no	no	no	yes	yes	yes
182221750	chr1	148333391	148333474	31	84	2.774194	no	no	yes	no	no	no	no	yes	no	yes

182221755	chr1	148353089	148353118	15	30	2.000000	no	no	yes	no	no	no	no	yes	yes	yes
182221756	chr1	148353813	148353926	58	114	1.965517	no	no	yes	no	no	no	no	yes	yes	yes
182221766	chr1	148358861	148359019	58	159	2.844828	yes	no	yes	no	no	no	no	no	no	yes
182221780	chr1	148377241	148377283	23	43	1.956522	no	no	yes	no	no	no	no	no	no	yes
182221811	chr1	148434945	148435014	24	70	2.846154	yes	no	yes	no	yes	no	no	no	no	yes
182221812	chr1	148435785	148435809	13	25	1.923077	no	yes	yes	no	no	no	no	no	yes	yes
182221832	chr1	148522491	148522527	16	37	2.312500	yes	no	no	no	no	no	no	yes	yes	no
182221991	chr1	148956362	148956409	25	48	1.960000	no	no	yes	no	no	no	no	no	no	yes
182221993	chr1	148959659	148959715	19	57	3.100000	no	no	yes	no	no	no	no	no	no	yes
182222009	chr1	149023570	149023844	28	275	9.741936	yes	no	yes	no	no	no	no	yes	no	yes
182222021	chr1	149056083	149056128	20	46	2.300000	no	no	yes	no	no	no	no	no	no	yes
182222158	chr1	149477297	149477347	27	51	1.925926	yes	no	yes	no	no	no	no	no	no	yes
182222202	chr1	149521067	149521137	24	71	2.958333	no	no	yes	no	no	no	no	no	no	yes
182222326	chr1	149746255	149746287	17	33	1.941176	no	no	yes	no	no	no	no	no	no	yes
182222396	chr1	149917233	149917275	23	43	1.913043	no	no	yes	no	no	no	no	no	yes	yes
182222401	chr1	149931469	149931521	29	53	1.931034	yes	no	yes	no	yes	no	no	yes	yes	yes
182222402	chr1	149932617	149932641	9	25	2.777778	no	no	yes	no	no	no	no	yes	no	yes
182222413	chr1	149962541	149962581	20	41	2.050000	no	no	yes	no	yes	no	no	yes	no	yes
182222414	chr1	149962814	149962842	15	29	1.933333	no	no	yes	no	yes	no	no	no	yes	yes
182222422	chr1	149979647	149979698	14	52	3.785714	no	no	yes	no	no	no	no	yes	no	yes
182222428	chr1	149993551	149993584	12	34	2.769231	no	no	yes	no	no	no	no	yes	no	yes
182222430	chr1	149997017	149997053	18	37	2.055556	no	no	yes	no	no	no	no	yes	yes	yes
182222432	chr1	150003703	150003735	16	33	2.062500	no	no	yes	no	no	no	no	no	no	yes
182222435	chr1	150011303	150011343	21	41	1.952381	yes	no	no	no	no	no	no	yes	yes	no
182222436	chr1	150012202	150012226	11	25	2.272727	no	no	no	no	no	no	no	yes	yes	no

182222448	chr1	150016309	150016337	14	29	2.071429	no	no	no	no	no	no	no	yes	yes	no
182222459	chr1	150042702	150042783	43	82	1.953488	no	no	no	no	no	no	no	no	yes	no
182222470	chr1	150079136	150079169	18	34	1.944444	no	no	yes	no	no	no	no	no	no	yes
182222473	chr1	150090333	150090364	12	32	2.750000	no	no	yes	no	no	no	no	yes	yes	yes
182222477	chr1	150106604	150106647	12	44	3.666667	no	no	yes	no	no	no	no	yes	yes	yes
182222478	chr1	150112507	150112538	15	32	2.133333	no	no	yes	no	no	no	no	no	yes	yes
182222483	chr1	150121364	150121397	17	34	2.000000	no	no	yes	no	no	no	no	no	no	yes
182222490	chr1	150133732	150133788	30	57	1.900000	no	no	yes	no	no	no	no	no	yes	yes
182222495	chr1	150149217	150149268	26	52	2.000000	yes	no	no	no	yes	no	no	yes	yes	yes
182222496	chr1	150163412	150163437	12	26	2.166667	no	no	yes	no	no	no	no	yes	yes	yes
182222501	chr1	150189335	150189387	27	53	2.000000	no	no	no	no	no	no	no	no	no	no
182222512	chr1	150220070	150220143	37	74	2.000000	no	yes	no	no	no	yes	no	no	no	yes
182222517	chr1	150226681	150226727	15	47	3.133333	no	no	yes	yes	no	no	no	yes	yes	yes
182222535	chr1	150271606	150271642	14	37	2.642857	no	no	yes	no	no	no	no	no	yes	yes
182222553	chr1	150315886	150315933	20	48	2.400000	no	no	no	no	no	no	no	yes	yes	no
182222575	chr1	150367356	150367471	58	116	2.000000	no	no	yes	no	no	no	no	yes	no	yes
182222604	chr1	150408486	150408742	138	257	1.862319	no	no	yes	no	no	no	no	yes	no	yes
182222611	chr1	150417358	150417451	44	94	2.136364	no	no	yes	no	no	no	no	yes	no	yes
182222618	chr1	150428917	150428979	32	63	1.968750	no	no	yes	no	no	no	no	no	no	yes
182222630	chr1	150449545	150449609	32	65	2.031250	no	no	yes	no	no	no	no	yes	no	yes
182222651	chr1	150483179	150483212	17	34	2.000000	no	no	no	no	no	no	no	no	no	no
182222656	chr1	150486897	150486921	13	25	1.923077	yes	no	no	no	no	no	no	yes	yes	no
182222669	chr1	150506487	150506667	91	181	1.989011	no	no	yes	no	no	no	no	yes	yes	yes
182222671	chr1	150515287	150515316	13	30	2.307692	yes	no	no	no	no	no	no	yes	no	no
182222699	chr1	150549031	150549229	85	199	2.356322	yes	no	yes	no	no	no	yes	yes	yes	yes

182222718	chr1	150584409	150584437	14	29	2.071429	no	no	no	no	no	no	no	no	no	no
182222740	chr1	150601301	150601404	27	104	3.793103	yes	no	yes	no	no	no	no	yes	no	yes
182222747	chr1	150616761	150616801	21	41	1.952381	no	no	yes	no	no	no	no	yes	no	yes
182222755	chr1	150632388	150632427	19	40	2.105263	no	no	no	no	no	no	no	no	no	no
182222780	chr1	150682757	150682790	17	34	2.058824	no	no	yes	no	no	no	no	no	no	yes
182222817	chr1	150743085	150743123	20	39	1.950000	no	no	yes	no	no	no	no	no	no	yes
182222818	chr1	150743498	150744730	36	1233	34.490566	no	no	yes	no	no	no	no	no	no	yes
182222842	chr1	150784941	150784989	16	49	3.187500	no	no	no	no	no	no	no	no	no	no
182222844	chr1	150787917	150787975	30	59	2.000000	no	no	no	no	no	no	no	yes	yes	no
182222859	chr1	150832188	150832228	21	41	1.952381	no	no	yes	no	no	no	no	no	no	yes
182222867	chr1	150842322	150842362	21	41	1.952381	no	no	yes	no	no	no	no	yes	yes	yes
182222868	chr1	150842575	150842614	19	40	2.157895	no	no	yes	no	no	no	no	yes	no	yes
182222870	chr1	150846224	150846259	17	36	2.117647	no	no	yes	no	no	no	no	yes	no	yes
182222878	chr1	150854896	150854987	49	92	1.897959	no	no	yes	no	no	no	no	no	no	yes
182222888	chr1	150874704	150874744	21	41	1.952381	no	no	yes	no	no	no	no	yes	yes	yes
182222890	chr1	150878391	150878419	14	29	2.071429	no	no	no	no	no	no	no	no	yes	no
182222904	chr1	150897218	150897257	21	40	2.000000	no	no	yes	no	no	no	no	no	no	yes
182222947	chr1	150963455	150963490	18	36	2.000000	no	yes	yes	no	no	yes	no	yes	yes	yes
182222949	chr1	150964347	150964378	17	32	1.941176	yes	yes	no	yes	yes	yes	no	yes	no	yes
182222950	chr1	150965259	150965288	14	30	2.142857	no	yes	yes	no	yes	yes	no	no	no	yes
182222951	chr1	150971703	150971757	29	55	1.931034	no	no	yes	no	no	no	no	yes	yes	yes
182222954	chr1	150981950	150981995	23	46	2.000000	yes	no	no	no	no	no	no	yes	yes	no
182222956	chr1	150987040	150987076	14	37	2.666667	no	no	yes	no	no	no	no	no	no	yes
182222959	chr1	150988462	150988489	14	28	2.000000	no	no	yes	no	no	no	no	yes	yes	yes
182222969	chr1	151008570	151008609	20	40	2.000000	yes	yes	no	no	yes	no	yes	yes	no	yes

182222972	chr1	151019632	151019681	20	50	2.550000	no	no	yes	no	no	no	no	no	no	yes
182222984	chr1	151058108	151058147	17	40	2.411765	no	no	yes	no	yes	no	no	yes	yes	yes
182222986	chr1	151064957	151064993	18	37	2.055556	no	no	yes	no	no	no	no	yes	no	yes
182223009	chr1	151093588	151093633	22	46	2.181818	no	no	yes	no	no	no	no	no	no	yes
182223020	chr1	151108675	151108714	21	40	1.904762	no	no	yes	no	no	no	no	yes	no	yes
182223031	chr1	151122214	151122248	17	35	2.058824	no	yes	no	no	no	no	no	yes	no	yes
182223035	chr1	151148464	151148496	17	33	1.941176	no	no	no	no	no	no	no	no	no	no
182223043	chr1	151160587	151160622	18	36	2.000000	no	yes	yes	no	no	yes	no	no	yes	yes
182223046	chr1	151162444	151162478	16	35	2.187500	no	no	yes	no	no	no	no	no	no	yes
182223075	chr1	151218993	151219018	12	26	2.166667	no	no	yes	no	no	no	no	no	no	yes
182223109	chr1	151283346	151283375	15	30	2.000000	no	no	yes	no	no	no	no	yes	yes	yes
182223111	chr1	151292352	151292393	17	42	2.470588	no	yes	no	no	no	yes	no	yes	yes	yes
182223112	chr1	151292661	151292698	20	38	1.900000	no	yes	no	no	no	yes	no	yes	yes	yes
182223113	chr1	151296319	151296357	16	39	2.437500	no	no	yes	no	no	no	no	no	no	yes
182223129	chr1	151328670	151328730	32	61	1.937500	yes	no	no	no	no	yes	no	yes	yes	no
182223151	chr1	151361660	151361718	28	59	2.142857	no	no	no	no	no	no	no	no	no	no
182223161	chr1	151371964	151371999	16	36	2.250000	no	no	yes	no	no	no	no	yes	yes	yes
182223162	chr1	151373377	151373500	64	124	2.000000	yes	no	no	no	no	no	no	yes	yes	no
182223181	chr1	151407166	151407208	21	43	2.142857	no	no	yes	no	yes	yes	no	no	no	yes
182223182	chr1	151409147	151409171	12	25	2.083333	yes	no	yes	no	no	no	no	yes	yes	yes
182223191	chr1	151421181	151421566	193	386	2.010363	no	no	yes	no	no	no	no	no	no	yes
182223192	chr1	151429116	151429174	31	59	2.000000	yes	no	yes	no	no	yes	no	no	no	yes
182223206	chr1	151459362	151459400	20	39	1.950000	yes	no	no	no	yes	no	yes	yes	no	yes
182223207	chr1	151463394	151463434	21	41	1.952381	no	no	no	no	no	no	no	yes	no	no
182223209	chr1	151466874	151466919	14	46	3.200000	no	no	no	no	no	no	no	no	no	no

182223221	chr1	151487154	151487218	25	65	2.640000	no	no	no	no	no	no	no	yes	no	no
182223244	chr1	151529843	151529896	22	54	2.454545	no	no	yes	no	no	yes	no	no	no	yes
182223245	chr1	151531797	151531933	29	137	4.724138	no	no	yes	no	no	no	no	no	no	yes
182223259	chr1	151565983	151566024	18	42	2.333333	no	yes	yes	no	no	yes	no	yes	no	yes
182223264	chr1	151572485	151572517	14	33	2.357143	no	no	yes	no	no	no	no	no	no	yes
182223265	chr1	151581593	151581630	20	38	1.950000	no	no	yes	no	no	no	no	no	yes	yes
182223296	chr1	151612621	151612665	23	45	1.956522	no	no	yes	no	no	no	yes	yes	yes	yes
182223304	chr1	151629440	151629546	40	107	2.690476	no	no	yes	no	no	yes	no	no	no	yes
182223332	chr1	151654860	151654899	13	40	3.153846	no	no	yes	no	no	no	no	yes	no	yes
182223333	chr1	151659049	151659082	18	34	1.944444	no	no	yes	no	no	no	no	no	no	yes
182223335	chr1	151659523	151659736	109	214	1.963303	yes	yes	yes	no	no	no	no	yes	yes	yes
182223336	chr1	151660685	151660727	20	43	2.150000	no	yes	yes	no	no	yes	no	yes	no	yes
182223337	chr1	151661532	151661560	15	29	1.933333	no	yes	yes	no	no	yes	no	no	no	yes
182223340	chr1	151664079	151664114	18	36	2.111111	no	no	yes	no	no	no	no	no	no	yes
182223342	chr1	151664162	151664219	21	58	2.826087	no	no	yes	no	no	no	no	no	no	yes
182223353	chr1	151704498	151704529	16	32	2.000000	no	no	yes	no	no	no	no	yes	no	yes
182223355	chr1	151706342	151706366	12	25	2.083333	no	no	yes	yes	yes	no	no	no	yes	yes
182223356	chr1	151712902	151712952	18	51	2.888889	no	no	yes	no	no	no	no	no	yes	yes
182223357	chr1	151714023	151714051	10	29	2.900000	no	no	yes	no	no	no	no	yes	yes	yes
182223358	chr1	151722202	151722244	23	43	1.913043	no	no	yes	no	no	no	no	yes	yes	yes
182223365	chr1	151744616	151744661	24	46	1.916667	no	no	no	no	no	no	no	no	no	no
182223398	chr1	151820584	151820616	15	33	2.266667	no	no	yes	no	no	no	no	yes	no	yes
182223401	chr1	151825246	151825307	23	62	2.739130	no	no	yes	no	no	no	no	yes	yes	yes
182223402	chr1	151825517	151825550	17	34	2.000000	no	no	yes	no	no	no	no	yes	yes	yes
182223404	chr1	151831808	151831851	19	44	2.315789	yes	no	no	no	yes	no	no	yes	yes	yes

182223406	chr1	151840540	151840588	25	49	1.960000	yes	no	no	no	yes	no	no	yes	yes	yes
182223408	chr1	151847198	151847344	27	147	5.703704	no	yes	yes	yes	no	yes	no	no	no	yes
182223414	chr1	151859120	151859207	44	88	2.000000	no	no	no	no	no	no	no	yes	no	no
182223416	chr1	151861356	151861404	20	49	2.450000	no	no	no	no	no	no	no	yes	no	no
182223418	chr1	151861718	151861780	30	63	2.166667	no	no	no	no	no	no	no	yes	no	no
182223422	chr1	151863382	151863412	16	31	2.000000	no	no	no	no	no	no	no	yes	no	no
182223458	chr1	151941242	151941429	98	188	1.918367	no	no	no	no	no	no	no	yes	yes	no
182223463	chr1	151949257	151949302	16	46	2.882353	no	no	yes	no	no	yes	no	no	no	yes
182223474	chr1	151971114	151971155	22	42	1.909091	no	no	no	no	no	no	no	yes	yes	no
182223480	chr1	151982800	151982840	19	41	2.157895	no	no	no	no	no	yes	no	yes	no	no
182223492	chr1	152014226	152014256	14	31	2.214286	no	no	yes	no	no	no	no	yes	no	yes
182223495	chr1	152016807	152016875	23	69	2.884615	no	no	yes	no	no	no	no	yes	no	yes
182223506	chr1	152059282	152059449	88	168	1.909091	no	no	no	no	no	no	no	yes	no	no
182223514	chr1	152061294	152061700	226	407	1.809734	no	no	no	no	no	no	no	yes	no	no
182223524	chr1	152083343	152083629	28	287	10.517241	no	no	no	no	no	yes	no	no	no	no
182223530	chr1	152107882	152108324	244	443	1.848361	no	no	no	yes	no	no	yes	yes	yes	yes
182223538	chr1	152108695	152108911	120	217	1.833333	no	no	no	yes	no	no	yes	yes	yes	yes
182223541	chr1	152108882	152109100	120	219	1.825000	no	no	no	yes	no	no	yes	yes	yes	yes
182223544	chr1	152109598	152110502	90	905	10.088889	no	no	no	yes	no	no	yes	yes	yes	yes
182223552	chr1	152111882	152111934	27	53	1.962963	no	no	no	yes	no	no	yes	yes	yes	yes
182223554	chr1	152112633	152112668	18	36	2.055556	no	no	no	yes	no	no	yes	yes	yes	yes
182223575	chr1	152149500	152149531	16	32	2.000000	no	no	yes	no	no	no	no	no	no	yes
182223576	chr1	152154708	152154911	42	204	4.857143	no	yes	yes	yes	no	no	no	yes	no	yes
182223579	chr1	152155350	152156250	36	901	25.027779	no	no	yes	yes	no	no	no	yes	yes	yes
182223581	chr1	152156531	152156705	36	175	4.861111	no	no	yes	yes	no	no	no	yes	yes	yes

182223582	chr1	152157390	152157460	34	71	2.088235	no	no	yes	no	no	no	no	no	no	yes
182223584	chr1	152160524	152160566	18	43	2.388889	no	no	yes	no	no	no	no	yes	no	yes
182223585	chr1	152167427	152167458	13	32	2.461539	yes	no	yes	no	no	no	no	no	yes	yes
182223586	chr1	152172635	152172687	26	53	2.115385	no	no	yes	no	no	no	no	yes	no	yes
182223590	chr1	152194589	152194635	16	47	2.882353	no	no	yes	no	no	no	no	no	no	yes
182223592	chr1	152194639	152194699	30	61	2.100000	no	no	yes	no	no	no	no	no	no	yes
182223606	chr1	152197402	152197447	15	46	3.066667	yes	no	yes	no	no	no	no	yes	no	yes
182223624	chr1	152208931	152208965	18	35	1.944444	no	no	yes	no	no	no	no	no	no	yes
182223626	chr1	152212595	152212637	17	43	2.588235	no	yes	yes	no	no	no	no	no	no	yes
182223628	chr1	152213254	152221067	1410	7814	5.550354	no	no	yes	yes	no	no	yes	yes	yes	yes
182223630	chr1	152225267	152225298	16	32	2.000000	no	no	yes	no	no	no	no	no	no	yes
182223640	chr1	152237942	152237978	19	37	1.947368	no	no	yes	no	no	no	no	no	no	yes
182223645	chr1	152250634	152250706	33	73	2.212121	no	no	yes	no	no	no	no	no	no	yes
182223664	chr1	152257378	152257447	27	70	2.607143	no	no	yes	no	no	no	no	yes	no	yes
182223675	chr1	152294089	152294202	61	114	1.885246	no	no	yes	no	no	no	no	no	no	yes
182223680	chr1	152303180	152314028	972	10849	11.154872	yes	yes	yes	yes	no	no	no	yes	yes	yes
182223688	chr1	152328886	152328929	23	44	1.913043	no	no	yes	no	no	no	no	no	yes	yes
182223692	chr1	152344754	152344778	13	25	1.923077	no	no	yes	no	no	no	no	yes	yes	yes
182223694	chr1	152345200	152345238	11	39	3.416667	no	no	yes	no	no	no	no	no	no	yes
182223695	chr1	152350824	152354131	225	3308	14.576923	no	no	yes	yes	no	no	no	yes	yes	yes
182223698	chr1	152354133	152356528	231	2396	10.459575	no	no	yes	yes	no	no	no	yes	yes	yes
182223709	chr1	152364296	152364325	14	30	2.142857	no	yes	yes	no	no	yes	no	no	yes	yes
182223716	chr1	152390252	152390369	63	118	1.888889	no	no	yes	no	no	no	no	no	no	yes
182223718	chr1	152393695	152393726	15	32	2.200000	no	no	yes	no	no	no	no	no	no	yes
182223723	chr1	152410012	152410369	180	358	1.988889	no	no	yes	yes	no	no	no	yes	no	yes

182223726	chr1	152420692	152420735	21	44	2.142857	no	no	yes	no	no	no	no	no	no	yes
182223727	chr1	152427733	152427799	21	67	3.227273	no	no	yes	no	no	no	no	no	yes	yes
182223731	chr1	152458882	152458942	30	61	2.100000	no	no	no	no	no	no	no	yes	no	no
182223734	chr1	152463847	152463921	30	75	2.500000	no	no	no	no	no	no	no	no	yes	no
182223743	chr1	152475163	152475233	38	71	1.921053	no	no	no	no	no	no	no	no	no	no
182223745	chr1	152475330	152475389	23	60	2.695652	no	no	no	no	no	no	no	no	no	no
182223748	chr1	152480055	152480094	17	40	2.411765	no	no	no	no	no	no	no	yes	no	no
182223755	chr1	152501008	152501037	16	30	1.937500	no	no	no	no	no	no	no	no	no	no
182223760	chr1	152511571	152511654	24	84	3.500000	no	no	no	yes	no	no	no	no	no	yes
182223762	chr1	152511812	152511890	27	79	2.928571	no	no	no	yes	no	no	no	no	no	yes
182223772	chr1	152539350	152539390	19	41	2.157895	no	no	no	no	no	no	no	no	no	no
182223790	chr1	152627322	152627421	53	100	1.943396	no	no	no	no	no	no	no	no	no	no
182223792	chr1	152627615	152627647	17	33	1.941176	no	no	no	no	no	no	no	no	no	no
182223803	chr1	152647627	152647667	20	41	2.050000	no	no	no	no	no	no	no	no	no	no
182223805	chr1	152656449	152656503	23	55	2.391304	no	yes	no	no	no	yes	no	no	no	yes
182223817	chr1	152683853	152683901	17	49	2.882353	no	no	no	no	no	no	no	no	no	no
182223827	chr1	152709200	152709252	24	53	2.208333	no	no	no	yes	no	no	no	no	no	yes
182223832	chr1	152719663	152719747	36	85	2.361111	no	no	no	yes	no	no	no	no	no	yes
182223835	chr1	152719714	152719908	63	195	3.095238	no	no	no	yes	no	no	no	no	no	yes
182223837	chr1	152720807	152720849	20	43	2.200000	no	no	no	no	no	yes	no	no	no	no
182223846	chr1	152737839	152737891	21	53	2.523809	no	yes	no	no	no	yes	no	no	no	yes
182223847	chr1	152747281	152747338	30	58	1.966667	no	no	no	no	no	no	no	no	no	no
182223851	chr1	152748692	152748774	24	83	3.458333	no	no	no	no	no	no	no	no	no	no
182223854	chr1	152758903	152759005	34	103	3.029412	yes	no	no	no	no	no	no	no	no	no
182223855	chr1	152759781	152759845	27	65	2.407408	no	no	no	yes	no	no	no	yes	no	yes

182223859	chr1	152776419	152776474	28	56	2.035714	no	no	no	yes	no	no	no	no	no	yes
182223861	chr1	152776539	152776599	24	61	2.541667	no	no	no	yes	no	no	no	no	no	yes
182223863	chr1	152778125	152778177	16	53	3.312500	no	no	no	no	no	no	no	yes	no	no
182223868	chr1	152787335	152787401	27	67	2.592592	no	no	yes	yes	no	no	no	no	no	yes
182223875	chr1	152798811	152798855	18	45	2.555556	no	no	no	no	no	yes	no	no	no	no
182223885	chr1	152822174	152822224	21	51	2.409091	no	no	no	no	no	no	no	no	no	no
182223889	chr1	152825348	152825418	32	71	2.218750	no	no	no	no	no	no	no	no	no	no
182223895	chr1	152827505	152827585	39	81	2.102564	no	no	no	yes	no	no	no	no	no	yes
182223896	chr1	152827629	152827663	18	35	1.944444	no	no	no	yes	no	no	no	no	no	yes
182223903	chr1	152851916	152851965	22	50	2.272727	no	no	no	no	no	no	no	yes	yes	no
182223914	chr1	152884450	152884608	48	159	3.326531	no	no	no	yes	no	no	no	yes	yes	yes
182223915	chr1	152887851	152887893	22	43	1.954545	no	no	no	no	no	no	no	no	no	no
182223937	chr1	152934501	152934533	14	33	2.357143	no	no	yes	no	no	no	no	no	no	yes
182223939	chr1	152935637	152935666	12	30	2.583333	no	no	yes	no	no	no	no	no	no	yes
182223942	chr1	152943137	152943161	12	25	2.083333	no	no	yes	no	no	no	no	no	no	yes
182223944	chr1	152948591	152948785	21	195	9.285714	no	no	yes	yes	no	no	no	no	no	yes
182223946	chr1	152960745	152960775	9	31	3.444444	no	no	no	no	no	no	no	yes	no	no
182223952	chr1	152975845	152975885	12	41	3.500000	no	no	no	no	no	no	no	no	no	no
182223981	chr1	153041799	153041838	20	40	2.000000	yes	no	no	no	yes	no	no	yes	no	yes
182223994	chr1	153098712	153098769	30	58	1.966667	no	no	yes	no	no	no	no	no	no	yes
182224015	chr1	153164919	153164962	21	44	2.095238	no	no	no	no	no	no	no	no	no	no
182224019	chr1	153190155	153190201	24	47	2.000000	no	yes	no	no	no	no	no	yes	no	yes
182224020	chr1	153190952	153190986	16	35	2.250000	no	yes	no	no	no	no	no	yes	yes	yes
182224026	chr1	153207828	153207872	23	45	1.956522	no	no	no	no	no	no	no	no	no	no
182224027	chr1	153207876	153207904	15	29	1.933333	no	no	no	no	no	no	no	no	no	no

182224034	chr1	153238219	153238278	19	60	3.210526	no	no	no	no	no	no	no	no	no	no
182224035	chr1	153239892	153239933	23	42	1.913043	no	no	no	no	no	no	no	no	no	no
182224039	chr1	153243258	153244467	188	1210	6.440594	no	no	no	no	no	no	no	no	no	no
182224044	chr1	153245228	153245265	20	38	1.900000	no	no	no	no	no	no	no	yes	no	no
182224045	chr1	153246207	153246231	13	25	1.923077	no	no	no	no	no	no	no	no	no	no
182224050	chr1	153250613	153250923	164	311	1.951220	no	no	no	no	no	no	no	yes	no	no
182224056	chr1	153256521	153256581	16	61	3.937500	no	no	no	no	no	no	no	no	no	no
182224058	chr1	153256652	153257006	28	355	12.821428	no	no	no	no	no	no	no	no	no	no
182224063	chr1	153261103	153261137	18	35	1.944444	no	no	no	yes	no	no	yes	yes	no	yes
182224071	chr1	153261271	153261404	45	134	2.977778	no	no	no	yes	no	no	yes	yes	no	yes
182224093	chr1	153299289	153299332	20	44	2.200000	no	no	yes	no	no	no	no	no	no	yes
182224103	chr1	153340697	153340727	15	31	2.066667	no	no	yes	no	no	no	no	no	no	yes
182224111	chr1	153355743	153355776	7	34	4.857143	no	no	no	no	no	no	no	yes	yes	no
182224115	chr1	153360863	153360927	28	65	2.428571	no	yes	no	no	no	no	no	yes	yes	yes
182224120	chr1	153379779	153379846	29	68	2.333333	no	no	no	no	yes	yes	no	no	no	yes
182224121	chr1	153380507	153380541	18	35	1.944444	no	no	no	no	yes	no	no	no	no	yes
182224125	chr1	153396945	153396970	13	26	2.000000	yes	no	no	no	no	no	no	no	no	no
182224129	chr1	153421595	153421625	14	31	2.214286	no	yes	no	no	no	no	no	no	no	yes
182224133	chr1	153431088	153431370	64	283	4.609375	no	no	no	no	no	no	no	yes	yes	no
182224135	chr1	153436605	153436643	20	39	2.000000	no	no	no	no	no	yes	no	no	no	no
182224138	chr1	153441673	153441717	22	45	2.045455	no	no	no	no	no	no	no	yes	yes	no
182224156	chr1	153489782	153489901	23	120	5.217391	no	no	no	no	no	no	no	no	no	no
182224159	chr1	153492688	153492736	24	49	2.083333	no	no	no	no	no	no	no	no	no	no
182224166	chr1	153507290	153507330	20	41	2.200000	no	no	no	no	no	no	no	yes	yes	no
182224172	chr1	153514722	153514765	20	44	2.200000	no	no	no	no	no	no	no	no	yes	no

182224192	chr1	153566010	153566054	21	45	2.190476	yes	no	yes	no	no	no	no	yes	no	yes
182224214	chr1	153583917	153583972	27	56	2.074074	no	no	no	no	no	no	no	no	no	no
182224230	chr1	153611580	153611640	28	61	2.178571	no	no	yes	no	no	no	no	yes	yes	yes
182224244	chr1	153651159	153651229	19	71	3.750000	no	no	no	no	no	no	no	no	no	no
182224247	chr1	153652461	153652521	16	61	3.937500	no	no	no	no	no	no	no	no	no	no
182224267	chr1	153680175	153680211	11	37	3.454545	yes	no	yes	no	no	no	no	yes	yes	yes
182224309	chr1	153728683	153728722	21	40	1.904762	no	yes	no	yes	no	no	no	yes	yes	yes
182224315	chr1	153748566	153748598	14	33	2.357143	no	yes	yes	no	no	no	no	yes	no	yes
182224316	chr1	153748865	153748896	16	32	2.000000	no	no	yes	no	no	no	no	no	no	yes
182224321	chr1	153762566	153762590	12	25	2.083333	no	no	yes	no	no	no	no	no	no	yes
182224327	chr1	153771273	153771310	20	38	1.900000	no	no	yes	no	no	no	no	yes	yes	yes
182224333	chr1	153779500	153779531	15	32	2.133333	no	yes	yes	no	no	yes	no	no	no	yes
182224371	chr1	153852719	153852761	21	43	2.047619	no	no	yes	no	yes	no	no	no	no	yes
182224387	chr1	153881791	153881826	18	36	2.000000	no	no	yes	no	no	no	no	yes	yes	yes
182224404	chr1	153898701	153898739	20	39	1.950000	no	no	yes	no	no	yes	no	no	no	yes
182224406	chr1	153903637	153903686	22	50	2.272727	no	no	yes	no	no	no	no	no	no	yes
182224421	chr1	153922911	153922953	21	43	2.047619	yes	no	yes	no	yes	no	no	yes	no	yes
182224432	chr1	153953949	153953989	21	41	1.952381	yes	no	yes	no	no	yes	no	yes	no	yes
182224455	chr1	154003963	154004022	21	60	2.952381	no	no	yes	no	no	no	no	no	no	yes
182224490	chr1	154040423	154040461	21	39	1.904762	yes	no	yes	no	no	no	no	no	no	yes
182224493	chr1	154041630	154041675	22	46	2.090909	no	no	yes	no	no	no	no	no	no	yes
182224519	chr1	154079747	154079794	20	48	2.400000	no	yes	yes	no	no	no	no	yes	no	yes
182224555	chr1	154131387	154131415	13	29	2.230769	no	no	yes	no	no	no	no	yes	no	yes
182224570	chr1	154159066	154159107	19	42	2.315789	no	no	yes	no	no	no	no	yes	yes	yes
182224571	chr1	154162456	154162514	29	59	2.034483	no	yes	yes	no	no	no	no	yes	yes	yes

182224590	chr1	154222264	154222451	95	188	1.978947	no	no	yes	no	no	no	no	yes	yes	yes
182224600	chr1	154239291	154239316	13	26	2.000000	no	no	yes	no	no	no	no	no	no	yes
182224601	chr1	154239414	154239447	16	34	2.187500	no	no	yes	no	no	no	no	no	no	yes
182224611	chr1	154268962	154269006	20	45	2.250000	no	yes	yes	no	no	no	no	yes	no	yes
182224617	chr1	154281533	154281561	15	29	1.933333	no	no	no	no	no	no	no	yes	yes	no
182224647	chr1	154300588	154300635	21	48	2.285714	no	no	no	no	no	no	no	no	no	no
182224660	chr1	154321427	154321455	15	29	1.933333	no	no	yes	no	no	no	no	yes	no	yes
182224665	chr1	154348695	154348773	40	79	1.975000	no	no	yes	no	no	no	no	yes	yes	yes
182224684	chr1	154371979	154372036	27	58	2.148148	no	no	no	no	no	no	no	yes	yes	no
182224714	chr1	154426052	154426230	87	179	2.080460	no	no	yes	no	no	no	no	no	no	yes
182224716	chr1	154439846	154439878	16	33	2.062500	no	no	yes	no	no	no	no	yes	no	yes
182224727	chr1	154467125	154467155	16	31	1.937500	no	yes	no	no	no	yes	no	yes	yes	yes
182224730	chr1	154489353	154489394	20	42	2.100000	yes	no	yes	yes	no	no	no	yes	no	yes
182224733	chr1	154502078	154502113	18	36	2.055556	yes	no	no	no	yes	no	yes	yes	no	yes
182224747	chr1	154543889	154543942	26	54	2.076923	no	no	yes	no	no	no	no	yes	yes	yes
182224752	chr1	154558456	154558499	20	44	2.250000	no	no	yes	yes	no	no	yes	yes	yes	yes
182224759	chr1	154593523	154593548	12	26	2.166667	no	no	yes	no	no	no	no	yes	yes	yes
182224764	chr1	154601766	154602047	147	282	1.918367	no	no	no	yes	yes	yes	no	yes	no	yes
182224768	chr1	154607685	154607888	78	204	2.700000	no	no	yes	no	no	no	no	yes	yes	yes
182224772	chr1	154611632	154611661	15	30	2.000000	no	no	yes	no	no	no	no	yes	yes	yes
182224774	chr1	154618060	154618105	20	46	2.350000	no	no	yes	no	no	no	no	yes	no	yes
182224775	chr1	154618099	154618146	22	48	2.227273	no	no	yes	no	no	no	no	yes	no	yes
182224776	chr1	154627506	154627553	22	48	2.181818	no	no	yes	no	yes	no	yes	yes	yes	yes
182224818	chr1	154705712	154705740	12	29	2.416667	no	yes	yes	no	no	no	no	no	no	yes
182224839	chr1	154755788	154756323	254	536	2.155642	no	no	yes	no	no	no	no	yes	yes	yes

182224859	chr1	154799327	154799373	18	47	2.611111	no	no	yes	no	no	no	no	no	yes	yes
182224860	chr1	154802547	154802580	18	34	1.944444	no	no	yes	no	no	no	no	no	yes	yes
182224880	chr1	154880582	154880620	20	39	1.950000	no	no	no	no	no	no	no	no	no	no
182224885	chr1	154897310	154897649	70	340	4.736842	no	no	no	no	no	no	no	yes	no	no
182224902	chr1	154953769	154953804	18	36	2.000000	yes	no	yes	no	no	no	no	yes	yes	yes
182224905	chr1	154965509	154965548	22	40	1.909091	no	no	yes	yes	no	yes	no	yes	no	yes
182224906	chr1	154971779	154971812	17	34	2.000000	no	no	yes	no	no	no	no	yes	yes	yes
182224907	chr1	154973824	154973857	18	34	1.944444	yes	no	yes	no	no	no	yes	yes	yes	yes
182224924	chr1	155013563	155013604	22	42	1.909091	yes	no	yes	no	no	no	no	yes	yes	yes
182224930	chr1	155030369	155030408	14	40	2.857143	no	no	yes	no	no	no	no	no	no	yes
182224937	chr1	155046460	155046489	15	30	2.066667	yes	no	yes	yes	no	no	no	yes	no	yes
182224948	chr1	155078756	155078806	27	51	1.962963	yes	no	yes	no	no	no	yes	yes	yes	yes
182224982	chr1	155120438	155120497	29	60	2.068965	yes	yes	no	no	no	no	no	yes	yes	yes
182224998	chr1	155147318	155147449	31	132	4.375000	no	no	no	no	no	no	no	no	no	no
182225019	chr1	155167024	155167077	24	54	2.250000	no	no	no	no	no	no	no	yes	yes	no
182225020	chr1	155169842	155169883	19	42	2.315789	no	no	yes	yes	yes	no	no	yes	no	yes
182225021	chr1	155177507	155177553	21	47	2.238095	no	no	yes	no	no	no	no	yes	no	yes
182225024	chr1	155181950	155181985	11	36	3.363636	no	yes	yes	yes	no	no	no	yes	yes	yes
182225030	chr1	155188676	155192051	180	3376	18.909575	yes	no	yes	yes	yes	yes	yes	yes	yes	yes
182225048	chr1	155236793	155236838	18	46	2.555556	yes	no	yes	no	no	no	no	yes	no	yes
182225056	chr1	155250409	155250442	16	34	2.187500	no	no	yes	yes	yes	yes	yes	yes	yes	yes
182225065	chr1	155265039	155265176	34	138	4.058824	no	no	yes	no	no	no	no	yes	no	yes
182225083	chr1	155287829	155287870	23	42	1.913043	yes	yes	no	yes	no	no	no	yes	no	yes
182225085	chr1	155293705	155293804	50	100	2.000000	no	no	yes	no	no	no	no	no	no	yes
182225087	chr1	155295999	155296032	11	34	3.181818	no	no	yes	no	no	no	no	yes	no	yes

182225107	chr1	155336352	155336419	24	68	2.833333	no	yes	no	no	no	no	no	no	no	yes
182225111	chr1	155342940	155342968	14	29	2.071429	no	no	yes	no	no	yes	no	no	no	yes
182225121	chr1	155353708	155353775	34	68	2.000000	no	no	yes	no	no	no	no	no	yes	yes
182225122	chr1	155353791	155353835	22	45	2.090909	no	no	yes	no	no	no	no	no	yes	yes
182225155	chr1	155414611	155414647	19	37	1.947368	no	no	yes	no	no	no	no	no	no	yes
182225156	chr1	155415005	155415045	21	41	1.952381	no	no	yes	no	no	no	no	no	no	yes
182225161	chr1	155416843	155416879	9	37	4.111111	no	no	yes	no	no	no	no	yes	no	yes
182225187	chr1	155462562	155462587	12	26	2.166667	no	no	yes	no	no	no	no	yes	no	yes
182225189	chr1	155467233	155467279	23	47	2.043478	no	no	yes	no	no	no	no	yes	yes	yes
182225190	chr1	155469729	155469756	14	28	2.000000	no	no	yes	no	no	no	no	no	yes	yes
182225194	chr1	155476960	155476997	19	38	2.000000	no	no	yes	no	no	no	no	no	no	yes
182225204	chr1	155492505	155492555	21	51	2.428571	no	no	yes	no	no	no	no	no	no	yes
182225210	chr1	155502930	155502971	18	42	2.444444	no	no	yes	no	no	no	no	no	no	yes
182225214	chr1	155512165	155512193	13	29	2.230769	no	no	yes	no	no	no	no	no	no	yes
182225220	chr1	155531303	155531344	21	42	2.000000	no	no	yes	no	no	no	no	yes	no	yes
182225322	chr1	155721340	155721417	22	78	3.545455	yes	no	yes	no	no	no	no	no	no	yes
182225333	chr1	155730243	155730308	24	66	2.642857	no	no	yes	no	no	yes	no	no	no	yes
182225336	chr1	155736771	155736798	13	28	2.153846	yes	yes	yes	no	no	no	no	no	no	yes
182225339	chr1	155739579	155739622	23	44	2.000000	no	no	no	no	no	yes	no	yes	no	no
182225363	chr1	155772228	155772259	15	32	2.133333	no	no	yes	no	no	no	no	no	no	yes
182225378	chr1	155804435	155804465	16	31	1.937500	no	no	yes	no	no	no	no	no	no	yes
182225379	chr1	155809515	155810010	24	496	20.166666	no	no	yes	no	no	no	no	no	no	yes
182225385	chr1	155814252	155814295	21	44	2.095238	no	no	yes	no	no	no	no	no	no	yes
182225386	chr1	155815195	155815241	24	47	1.958333	no	no	yes	no	no	no	no	yes	no	yes
182225408	chr1	155849986	155850023	19	38	2.000000	no	no	yes	no	no	no	no	no	no	yes

182225411	chr1	155855535	155855606	28	72	2.678571	no	no	yes	no	no	no	no	no	no	yes
182225416	chr1	155859437	155859519	33	83	2.500000	yes	no	no	no	no	no	yes	yes	yes	no
182225424	chr1	155871728	155871767	22	40	1.909091	no	no	yes	no	no	no	no	yes	yes	yes
182225441	chr1	155910892	155910931	19	40	2.105263	yes	no	yes	no	yes	no	no	yes	yes	yes
182225444	chr1	155914988	155915129	73	142	1.945205	no	yes	yes	no	no	yes	no	yes	yes	yes
182225453	chr1	155932422	155932461	21	40	1.904762	no	no	yes	no	yes	no	no	yes	yes	yes
182225457	chr1	155938660	155938720	25	61	2.440000	no	no	no	no	no	no	no	yes	no	no
182225465	chr1	155952056	155952101	24	46	1.958333	yes	no	yes	no	no	yes	no	yes	no	yes
182225473	chr1	155978841	155978897	28	57	2.142857	yes	no	yes	no	no	no	no	yes	yes	yes
182225479	chr1	155995823	155995925	51	103	2.019608	no	no	yes	no	no	no	no	no	yes	yes
182225496	chr1	156009479	156009522	22	44	2.000000	no	yes	yes	no	yes	yes	no	yes	yes	yes
182225509	chr1	156031975	156032020	24	46	1.916667	no	no	yes	no	no	no	no	no	no	yes
182225527	chr1	156070524	156070568	23	45	2.043478	no	no	no	no	no	yes	no	yes	yes	no
182225528	chr1	156075241	156075281	16	41	2.562500	no	yes	no	no	no	no	no	yes	yes	yes
182225542	chr1	156090233	156090270	20	38	1.900000	no	no	yes	no	no	no	no	yes	yes	yes
182225550	chr1	156106614	156106666	22	53	2.409091	yes	yes	yes	no	no	no	yes	yes	no	yes
182225553	chr1	156113962	156113988	13	27	2.076923	yes	no	yes	no	no	no	no	yes	yes	yes
182225558	chr1	156125996	156126039	23	44	1.956522	yes	no	yes	no	no	no	no	yes	no	yes
182225575	chr1	156153048	156153095	24	48	2.000000	yes	no	yes	no	no	no	no	yes	yes	yes
182225576	chr1	156153771	156153799	15	29	2.000000	yes	no	yes	no	no	no	no	yes	no	yes
182225596	chr1	156177907	156177941	15	35	2.333333	no	no	no	no	no	yes	no	no	no	no
182225604	chr1	156189713	156189753	16	41	2.562500	no	no	no	no	no	no	no	no	no	no
182225605	chr1	156193110	156193149	21	40	1.904762	yes	no	no	no	no	no	no	yes	yes	no
182225616	chr1	156225518	156225557	20	40	2.000000	no	yes	yes	yes	no	no	no	yes	yes	yes
182225622	chr1	156243970	156244015	14	46	3.200000	no	yes	no	yes	yes	yes	no	yes	no	yes

182225630	chr1	156271242	156271284	18	43	2.500000	no	no	yes	no	no	no	no	no	no	yes
182225637	chr1	156277420	156277500	36	81	2.250000	no	no	yes	no	no	no	no	yes	no	yes
182225642	chr1	156289750	156289798	22	49	2.227273	no	no	yes	no	no	yes	no	no	no	yes
182225646	chr1	156290276	156290338	28	63	2.250000	no	yes	yes	no	yes	yes	no	no	no	yes
182225648	chr1	156296099	156296149	26	51	1.961538	yes	no	no	no	no	no	no	yes	yes	no
182225687	chr1	156335215	156335251	19	37	1.947368	no	no	yes	no	yes	no	no	yes	no	yes
182225688	chr1	156342139	156342181	21	43	2.047619	no	no	yes	no	no	no	no	no	no	yes
182225701	chr1	156366269	156366329	31	61	2.000000	no	no	yes	no	no	no	no	no	no	yes
182225708	chr1	156391791	156391815	12	25	2.083333	no	yes	no	no	no	no	no	yes	yes	yes
182225719	chr1	156401903	156401956	21	54	2.619048	no	no	no	no	no	no	no	yes	yes	no
182225721	chr1	156409252	156409289	18	38	2.111111	yes	no	yes	no	no	no	no	no	no	yes
182225727	chr1	156412549	156412584	18	36	2.000000	no	no	yes	no	yes	no	no	yes	no	yes
182225733	chr1	156436658	156436702	18	45	2.611111	no	no	yes	no	no	no	no	yes	yes	yes
182225746	chr1	156456672	156456697	10	26	2.600000	yes	no	no	no	yes	no	no	yes	yes	yes
182225759	chr1	156475301	156475352	27	52	1.925926	no	no	yes	no	no	no	no	yes	no	yes
182225760	chr1	156479991	156480016	13	26	2.000000	yes	no	yes	no	no	no	no	yes	yes	yes
182225761	chr1	156481260	156481299	16	40	2.500000	no	no	yes	no	no	no	no	yes	yes	yes
182225763	chr1	156498581	156498619	8	39	4.875000	no	no	yes	no	no	no	no	yes	yes	yes
182225785	chr1	156538745	156538777	15	33	2.200000	no	no	yes	no	no	no	no	no	no	yes
182225833	chr1	156615872	156615946	28	75	2.678571	yes	no	no	no	no	no	no	yes	no	no
182225844	chr1	156639744	156639776	17	33	1.941176	no	no	no	no	yes	no	no	yes	yes	yes
182225849	chr1	156646651	156646677	7	27	3.857143	yes	no	yes	no	yes	no	no	yes	no	yes
182225850	chr1	156647198	156647226	13	29	2.307692	no	no	yes	no	no	yes	no	yes	no	yes
182225852	chr1	156651485	156651513	12	29	2.416667	no	yes	yes	yes	no	no	no	yes	no	yes
182225857	chr1	156660542	156660579	20	38	1.900000	no	no	yes	no	no	no	no	yes	yes	yes

182225866	chr1	156668899	156668940	21	42	2.000000	no	yes	no	no	no	no	no	yes	yes	yes
182225892	chr1	156743698	156743733	15	36	2.400000	no	no	no	yes	yes	yes	no	yes	yes	yes
182225895	chr1	156747231	156747274	22	44	2.000000	no	no	yes	no	no	no	no	yes	yes	yes
182225897	chr1	156751707	156751784	29	78	2.724138	yes	no	yes	no	no	no	yes	yes	yes	yes
182225913	chr1	156780368	156780413	23	46	2.043478	no	no	yes	no	no	no	no	no	no	yes
182225914	chr1	156780395	156780449	18	55	3.000000	no	no	yes	no	no	no	no	no	no	yes
182225931	chr1	156800010	156800058	25	49	1.960000	no	no	yes	no	no	no	no	no	no	yes
182225933	chr1	156804328	156804368	18	41	2.388889	no	no	no	no	no	no	no	yes	no	no
182225943	chr1	156836724	156836853	26	130	5.000000	yes	no	yes	no	no	no	no	yes	no	yes
182225946	chr1	156847447	156847488	22	42	1.909091	no	no	yes	no	no	no	no	no	no	yes
182225947	chr1	156848880	156848912	17	33	1.941176	no	no	yes	no	no	no	yes	yes	yes	yes
182225953	chr1	156862694	156862719	11	26	2.363636	no	no	yes	no	no	no	no	yes	yes	yes
182225959	chr1	156871477	156871529	28	53	1.928571	no	no	yes	no	no	no	no	no	no	yes
182225961	chr1	156874311	156874373	22	63	2.909091	no	yes	yes	no	no	yes	no	no	no	yes
182225963	chr1	156883087	156883126	18	40	2.277778	no	no	no	no	no	no	no	yes	no	no
182225964	chr1	156883914	156883972	26	59	2.269231	no	no	no	no	no	no	no	yes	no	no
182225972	chr1	156892321	156892387	30	67	2.233333	no	no	no	no	no	no	no	yes	yes	no
182225978	chr1	156902233	156902259	14	27	1.928571	no	no	yes	no	yes	no	no	yes	yes	yes
182225979	chr1	156906546	156906582	14	37	2.642857	no	no	yes	no	no	no	no	yes	no	yes
182225981	chr1	156911024	156911299	127	276	2.217054	no	no	yes	no	no	yes	no	yes	no	yes
182225993	chr1	156935177	156935216	22	40	1.909091	no	yes	no	no	yes	yes	no	no	no	yes
182225999	chr1	156979156	156979180	12	25	2.083333	no	no	yes	no	no	no	no	no	no	yes
182226000	chr1	156982558	156982588	16	31	2.000000	no	no	yes	no	no	no	no	no	no	yes
182226005	chr1	156992578	156992630	26	53	2.038461	no	no	yes	no	no	no	no	yes	no	yes
182226007	chr1	157001756	157001792	20	37	1.950000	no	no	yes	no	no	no	no	no	no	yes

182226017	chr1	157026129	157026170	22	42	1.954545	no	no	yes	no	no	no	no	yes	yes	yes
182226026	chr1	157045568	157045612	24	45	1.958333	yes	no	no	no	no	no	yes	yes	yes	no
182226034	chr1	157056287	157056349	21	63	3.045455	no	no	no	no	no	no	no	yes	no	no
182226059	chr1	157107367	157107408	21	42	2.047619	no	no	yes	no	no	no	no	no	yes	yes
182226062	chr1	157126656	157127058	31	403	13.000000	no	no	yes	no	no	no	no	no	yes	yes
182226063	chr1	157127123	157127163	21	41	1.952381	no	no	yes	no	no	no	no	no	yes	yes
182226064	chr1	157127196	157127220	13	25	1.923077	no	no	yes	no	no	no	no	no	yes	yes
182226070	chr1	157178540	157178612	35	73	2.085714	no	no	yes	no	no	no	no	yes	yes	yes
182226074	chr1	157194475	157194539	32	65	2.093750	no	no	no	no	no	no	no	no	no	no
182226077	chr1	157199962	157200111	75	150	2.000000	no	no	no	no	no	no	no	no	no	no
182226082	chr1	157209938	157209973	17	36	2.176471	no	no	no	no	no	no	no	yes	no	no
182226090	chr1	157236921	157236963	14	43	3.071429	no	no	yes	no	no	no	no	no	no	yes
182226093	chr1	157249113	157249154	18	42	2.315789	no	no	no	no	no	no	no	no	no	no
182226100	chr1	157265599	157265676	40	78	1.950000	no	no	no	no	no	no	no	no	no	no
182226130	chr1	157418948	157418988	20	41	2.100000	no	no	no	no	no	no	no	no	no	no
182226133	chr1	157419198	157419270	23	73	3.217391	no	no	no	no	no	no	no	no	no	no
182226148	chr1	157485114	157485175	31	62	2.000000	no	no	no	no	no	no	no	no	no	no
182226152	chr1	157496202	157496231	16	30	1.937500	no	no	no	no	no	no	no	yes	yes	no
182226160	chr1	157516618	157516661	23	44	2.000000	yes	no	yes	no	no	no	no	no	no	yes
182226165	chr1	157549718	157549764	20	47	2.350000	no	no	yes	no	no	no	no	no	no	yes
182226172	chr1	157575831	157575859	14	29	2.071429	no	no	yes	no	no	no	no	yes	yes	yes
182226174	chr1	157581408	157581444	14	37	2.642857	yes	no	yes	no	no	no	no	yes	yes	yes
182226182	chr1	157611842	157611871	11	30	2.727273	no	no	no	no	no	no	no	no	no	no
182226185	chr1	157636607	157636647	19	41	2.157895	no	no	yes	no	no	no	no	no	no	yes
182226197	chr1	157671265	157671319	25	55	2.280000	no	no	no	no	no	no	no	no	no	no

182226200	chr1	157679051	157679212	81	162	2.012346	no	no	yes	no	yes	no	no	no	no	yes
182226208	chr1	157695887	157695926	20	40	2.000000	no	no	yes	no	no	no	no	no	no	yes
182226242	chr1	157839035	157839063	14	29	2.071429	no	no	yes	no	no	no	no	yes	yes	yes
182226247	chr1	157865660	157865725	27	66	2.444444	no	no	yes	no	no	no	no	no	no	yes
182226249	chr1	157873869	157873900	11	32	3.000000	no	no	yes	no	no	no	no	no	no	yes
182226255	chr1	157878735	157878791	30	57	1.900000	no	no	yes	no	no	no	no	yes	no	yes
182226262	chr1	157917032	157917113	41	82	2.000000	no	no	no	no	no	no	no	no	no	no
182226263	chr1	157923125	157923158	11	34	3.000000	no	no	no	no	no	no	no	no	no	no
182226268	chr1	157946640	157946673	17	34	2.000000	no	no	yes	no	no	no	no	no	no	yes
182226270	chr1	157954222	157954259	12	38	3.166667	no	no	no	no	no	no	no	no	no	no
182226279	chr1	157967513	157967874	34	362	10.571428	no	no	no	no	no	no	no	no	no	no
182226290	chr1	157993573	157993597	8	25	3.125000	yes	no	no	no	yes	no	no	yes	yes	yes
182226293	chr1	158009819	158009862	15	44	3.000000	no	no	yes	no	no	no	no	yes	yes	yes
182226295	chr1	158011996	158012022	14	27	1.928571	no	no	yes	no	no	no	no	yes	yes	yes
182226298	chr1	158031330	158031369	14	40	2.857143	no	no	yes	no	no	yes	no	no	no	yes
182226299	chr1	158032308	158032348	21	41	2.000000	no	no	yes	no	no	no	no	yes	yes	yes
182226302	chr1	158036215	158036250	12	36	3.000000	no	no	yes	no	no	no	no	yes	yes	yes
182226309	chr1	158077305	158077342	20	38	1.900000	no	no	yes	no	no	no	no	yes	no	yes
182226310	chr1	158078604	158078655	22	52	2.454545	no	no	yes	no	no	no	no	no	no	yes
182226311	chr1	158078780	158078814	18	35	1.944444	no	no	yes	no	no	no	no	yes	yes	yes
182226312	chr1	158081707	158081746	19	40	2.105263	no	no	yes	no	no	no	no	no	no	yes
182226313	chr1	158082580	158082638	26	59	2.269231	no	no	yes	no	no	no	no	no	no	yes
182226316	chr1	158093884	158093922	20	39	1.950000	no	no	yes	no	no	no	no	no	no	yes
182226327	chr1	158123981	158124029	23	49	2.173913	no	no	no	no	no	no	no	yes	no	no
182226333	chr1	158149162	158149202	20	41	2.050000	no	no	yes	no	no	no	no	yes	no	yes

182226335	chr1	158155011	158155042	16	32	2.000000	no	no	no	no	no	no	no	no	no	no
182226337	chr1	158160319	158160380	33	62	1.909091	no	no	no	no	no	no	no	no	no	no
182226338	chr1	158160724	158160774	26	51	1.961538	no	no	no	no	no	no	no	yes	yes	no
182226345	chr1	158196809	158196842	13	34	2.692308	yes	no	no	no	no	no	no	no	no	no
182226356	chr1	158246112	158246143	14	32	2.285714	no	no	no	no	no	no	no	no	no	no
182226362	chr1	158257577	158257608	15	32	2.133333	no	no	yes	no	no	no	no	yes	no	yes
182226386	chr1	158331979	158332034	16	56	3.500000	yes	no	no	no	no	no	no	no	yes	no
182226388	chr1	158332702	158332748	22	47	2.136364	no	no	no	no	no	no	no	no	no	no
182226403	chr1	158355134	158355162	15	29	1.933333	yes	no	yes	no	no	no	no	no	no	yes
182226416	chr1	158402464	158402499	16	36	2.250000	no	no	no	no	no	no	no	no	no	no
182226420	chr1	158424001	158424042	21	42	2.047619	no	no	yes	no	no	no	no	no	no	yes
182226433	chr1	158473698	158473743	14	46	3.466667	no	no	yes	no	no	yes	no	yes	no	yes
182226436	chr1	158473768	158473932	35	165	4.694445	no	no	yes	no	no	yes	no	yes	no	yes
182226438	chr1	158473921	158473952	10	32	3.200000	no	no	yes	no	no	yes	no	yes	no	yes
182226446	chr1	158508613	158508657	23	45	2.000000	no	no	no	no	no	no	no	no	no	no
182226449	chr1	158520114	158520185	28	72	2.655172	no	no	no	no	no	no	no	no	yes	no
182226466	chr1	158569020	158569057	19	38	2.000000	no	no	yes	no	no	no	no	no	no	yes
182226467	chr1	158573264	158573344	36	81	2.277778	no	no	no	no	no	no	no	no	no	no
182226475	chr1	158585887	158585929	20	43	2.150000	no	no	no	no	no	no	no	no	no	no
182226478	chr1	158605941	158605981	21	41	1.952381	yes	no	yes	no	no	no	no	no	no	yes
182226480	chr1	158613146	158613176	15	31	2.066667	no	no	yes	no	no	yes	no	no	no	yes
182226495	chr1	158637471	158637717	124	247	1.991935	no	no	yes	no	no	no	no	no	no	yes
182226498	chr1	158647029	158647065	18	37	2.111111	no	no	yes	no	no	no	no	no	no	yes
182226499	chr1	158651479	158651522	20	44	2.200000	no	no	yes	no	no	no	no	no	no	yes
182226502	chr1	158656242	158656296	29	55	1.931034	no	no	yes	no	no	yes	no	no	no	yes

182226506	chr1	158666132	158666289	84	158	1.880952	no	no	yes	no	no	no	no	yes	no	yes
182226508	chr1	158666503	158666531	14	29	2.071429	no	no	yes	no	no	no	no	no	no	yes
182226512	chr1	158670766	158670805	18	40	2.222222	no	no	yes	no	no	no	no	no	no	yes
182226513	chr1	158683668	158683706	19	39	2.052632	no	no	yes	no	no	no	no	yes	no	yes
182226514	chr1	158686355	158686398	23	44	1.956522	no	no	yes	no	no	no	no	yes	yes	yes
182226531	chr1	158718290	158718328	19	39	2.052632	yes	no	yes	no	no	no	no	no	no	yes
182226538	chr1	158736971	158737011	21	41	1.952381	no	no	no	no	no	no	no	no	no	no
182226557	chr1	158779426	158779454	14	29	2.142857	no	no	yes	no	no	no	no	no	no	yes
182226559	chr1	158785756	158785797	18	42	2.333333	no	no	no	no	no	no	no	no	no	no
182226573	chr1	158841731	158841764	11	34	3.090909	no	no	yes	no	no	no	no	no	no	yes
182226574	chr1	158844733	158844788	27	56	2.111111	no	no	yes	no	no	no	no	no	no	yes
182226577	chr1	158862767	158862821	27	55	2.037037	no	no	no	no	no	no	no	no	no	no
182226578	chr1	158872287	158872324	20	38	1.950000	no	no	no	no	no	no	no	yes	no	no
182226581	chr1	158881864	158881976	56	113	2.017857	no	no	yes	no	no	no	no	yes	no	yes
182226586	chr1	158901410	158901518	30	109	3.633333	no	no	no	no	no	no	no	no	no	no
182226587	chr1	158901589	158901631	16	43	2.750000	no	no	no	no	no	no	no	no	no	no
182226589	chr1	158917553	158917597	23	45	2.000000	no	no	no	no	no	no	no	no	no	no
182226594	chr1	158932673	158932702	9	30	3.333333	no	no	yes	no	no	no	no	no	no	yes
182226597	chr1	158971267	158971369	53	103	1.943396	no	no	yes	no	no	no	no	no	no	yes
182226609	chr1	159020725	159020775	26	51	1.961538	no	no	yes	no	no	yes	no	no	no	yes
182226617	chr1	159038354	159038395	21	42	2.000000	no	no	yes	no	no	no	no	no	no	yes
182226626	chr1	159065554	159065616	31	63	2.032258	no	no	yes	no	no	yes	no	no	no	yes
182226627	chr1	159067467	159067497	15	31	2.066667	no	no	yes	no	no	no	no	no	yes	yes
182226629	chr1	159083600	159083631	16	32	2.000000	no	no	yes	no	no	no	no	no	no	yes
182226630	chr1	159083650	159083697	25	48	1.920000	no	no	yes	no	no	no	no	no	no	yes

182226650	chr1	159142353	159142378	13	26	2.000000	no	no	yes	no	no	no	no	yes	no	yes
182226655	chr1	159170901	159171214	157	314	2.006369	yes	no	no	no	no	no	no	yes	yes	no
182226660	chr1	159193090	159193117	12	28	2.333333	no	no	yes	no	no	no	no	no	no	yes
182226661	chr1	159198809	159198868	20	60	3.000000	no	no	yes	no	no	no	no	no	no	yes
182226667	chr1	159202069	159202123	20	55	2.750000	no	yes	yes	no	no	yes	no	yes	no	yes
182226668	chr1	159204582	159204698	57	117	2.052632	yes	no	yes	no	yes	no	no	yes	yes	yes
182226674	chr1	159226610	159226634	12	25	2.083333	no	no	no	no	no	no	no	yes	no	no
182226687	chr1	159279298	159279333	19	36	2.000000	no	no	no	no	yes	no	no	yes	no	yes
182226693	chr1	159289646	159289692	25	47	1.960000	yes	no	no	no	no	no	no	yes	no	no
182226694	chr1	159290385	159290443	29	59	2.034483	no	no	yes	no	no	no	no	no	no	yes
182226696	chr1	159295470	159295516	24	47	2.000000	no	no	yes	no	no	no	no	no	no	yes
182226705	chr1	159370227	159370267	21	41	1.952381	no	no	yes	no	no	no	no	no	no	yes
182226706	chr1	159373570	159373621	28	52	1.928571	no	no	yes	no	no	no	no	no	no	yes
182226715	chr1	159408822	159408869	25	48	1.960000	no	no	yes	no	no	no	no	yes	no	yes
182226721	chr1	159424354	159424394	16	41	2.562500	no	no	yes	no	no	no	no	no	no	yes
182226723	chr1	159424438	159424477	21	40	1.904762	no	no	yes	no	no	no	no	no	no	yes
182226728	chr1	159444883	159444958	37	76	2.054054	no	no	yes	no	no	no	no	yes	no	yes
182226738	chr1	159469760	159469803	21	44	2.142857	yes	no	yes	no	no	no	no	no	no	yes
182226740	chr1	159471338	159471369	15	32	2.133333	no	no	yes	no	no	no	no	no	no	yes
182226744	chr1	159491900	159491946	20	47	2.350000	no	no	no	no	no	no	no	no	no	no
182226756	chr1	159510698	159510766	30	69	2.300000	no	no	no	no	no	no	no	no	no	no
182226772	chr1	159559778	159559809	16	32	2.000000	no	no	no	no	no	no	no	no	no	no
182226776	chr1	159577622	159577680	21	59	2.818182	no	no	no	no	no	no	no	no	no	no
182226778	chr1	159583034	159583063	14	30	2.214286	yes	no	no	no	no	no	no	no	no	no
182226779	chr1	159586904	159586941	20	38	1.900000	yes	no	no	no	no	no	no	no	no	no

182226781	chr1	159594312	159594350	20	39	2.000000	no	no	no	no	no	no	no	yes	no	no
182226784	chr1	159598938	159598973	18	36	2.000000	no	yes	no	no	no	no	no	yes	no	yes
182226802	chr1	159643410	159643499	34	90	2.638889	no	no	no	no	no	no	no	yes	no	no
182226807	chr1	159647778	159647804	12	27	2.250000	no	no	no	no	no	no	no	yes	yes	no
182226810	chr1	159650652	159650720	35	69	2.028571	no	no	no	no	no	no	no	no	no	no
182226814	chr1	159652883	159652933	26	51	1.961538	no	no	no	no	no	no	no	no	no	no
182226830	chr1	159694249	159694288	20	40	2.050000	no	no	no	no	no	no	no	no	no	no
182226831	chr1	159694355	159694395	17	41	2.529412	no	no	no	no	no	no	no	no	no	no
182226832	chr1	159695383	159695420	16	38	2.437500	no	no	no	no	no	no	no	no	no	no
182226837	chr1	159706490	159706528	20	39	2.000000	yes	no	no	no	no	no	no	yes	no	no
182226838	chr1	159707445	159707493	25	49	2.040000	no	no	no	no	no	no	no	yes	no	no
182226839	chr1	159708113	159708141	15	29	1.933333	no	no	no	no	no	no	no	yes	no	no
182226840	chr1	159713406	159713503	48	98	2.041667	no	yes	yes	no	yes	no	no	yes	no	yes
182226849	chr1	159746705	159746737	17	33	1.941176	no	no	no	no	no	no	no	yes	no	no
182226853	chr1	159755096	159755314	89	219	2.516484	no	no	no	no	no	no	no	yes	no	no
182226859	chr1	159778825	159778858	16	34	2.125000	no	no	no	no	no	no	no	no	no	no
182226863	chr1	159792561	159792606	18	46	2.555556	no	no	no	no	no	no	no	no	no	no
182226867	chr1	159806347	159806468	56	122	2.196429	no	no	yes	no	no	no	no	no	no	yes
182226868	chr1	159807386	159807437	17	52	3.058824	no	no	yes	no	no	no	no	no	no	yes
182226871	chr1	159809538	159809581	24	44	1.958333	no	no	no	yes	no	yes	no	no	no	yes
182226882	chr1	159834402	159834431	7	30	4.428571	no	no	yes	no	no	yes	no	yes	no	yes
182226883	chr1	159840259	159840316	30	58	1.933333	no	yes	yes	no	no	no	no	no	no	yes
182226886	chr1	159859103	159859140	15	38	2.533333	no	no	yes	no	no	no	no	no	yes	yes
182226887	chr1	159859143	159859183	20	41	2.050000	no	no	yes	no	no	no	no	no	yes	yes
182226890	chr1	159866066	159866102	18	37	2.055556	no	yes	yes	no	no	no	no	no	no	yes

182226903	chr1	159906883	159906914	11	32	2.909091	no	no	no	no	no	no	no	no	no	no
182226904	chr1	159908966	159909007	19	42	2.210526	no	no	no	no	no	no	no	yes	yes	no
182226908	chr1	159939670	159939709	21	40	1.904762	no	no	yes	no	no	no	no	yes	yes	yes
182226912	chr1	159956497	159956523	14	27	1.928571	no	no	no	no	no	no	no	yes	no	no
182226930	chr1	159993899	159993943	23	45	1.956522	no	no	no	no	no	no	no	yes	no	no
182226931	chr1	159993954	159994039	33	86	2.606061	no	no	no	no	no	no	no	yes	no	no
182226936	chr1	160005645	160005683	19	39	2.052632	no	no	yes	no	no	no	no	no	no	yes
182226944	chr1	160038884	160038944	31	61	1.967742	no	yes	yes	no	yes	no	no	no	no	yes
182226949	chr1	160047127	160047177	20	51	2.550000	no	no	yes	no	no	no	no	yes	yes	yes
182226960	chr1	160085946	160085983	21	38	1.952381	no	no	yes	no	no	no	no	yes	no	yes
182226961	chr1	160089195	160089241	22	47	2.136364	no	yes	no	no	no	no	no	no	no	yes
182226963	chr1	160089932	160089985	21	54	2.619048	no	yes	no	no	no	no	no	no	no	yes
182226964	chr1	160090715	160090775	19	61	3.315789	no	no	no	no	no	yes	no	no	no	no
182226969	chr1	160101099	160101135	17	37	2.176471	no	no	no	no	no	no	no	yes	yes	no
182226972	chr1	160108693	160108717	12	25	2.083333	no	no	no	no	no	no	no	yes	yes	no
182226973	chr1	160109374	160109405	15	32	2.200000	no	no	no	no	no	no	no	yes	yes	no
182226981	chr1	160116938	160116979	14	42	3.000000	no	no	yes	no	no	no	no	yes	yes	yes
182226985	chr1	160135324	160135352	15	29	1.933333	no	no	yes	no	no	no	no	no	no	yes
182226999	chr1	160155457	160155513	31	57	2.000000	no	no	yes	no	no	no	no	no	no	yes
182227003	chr1	160161214	160161252	20	39	1.950000	no	no	yes	no	no	no	no	yes	no	yes
182227014	chr1	160196419	160196443	12	25	2.083333	no	no	yes	no	no	no	no	yes	no	yes
182227018	chr1	160203125	160203320	96	196	2.062500	no	no	yes	no	no	no	no	no	no	yes
182227023	chr1	160205393	160205434	18	42	2.333333	yes	no	no	no	yes	no	yes	yes	yes	yes
182227025	chr1	160220784	160220822	18	39	2.166667	yes	no	yes	no	yes	yes	no	yes	no	yes
182227027	chr1	160229478	160229517	10	40	4.200000	no	no	yes	no	no	no	no	no	no	yes

182227043	chr1	160243271	160243307	17	37	2.235294	no	no	yes	no	no	no	no	yes	no	yes
182227045	chr1	160244190	160244220	14	31	2.214286	no	no	yes	no	no	no	no	yes	no	yes
182227056	chr1	160267441	160267487	25	47	1.960000	yes	no	yes	no	no	no	no	yes	no	yes
182227066	chr1	160325753	160325793	19	41	2.263158	no	no	yes	no	no	yes	no	yes	no	yes
182227071	chr1	160352354	160352403	24	50	2.083333	no	no	yes	no	no	no	no	no	no	yes
182227073	chr1	160362672	160362715	23	44	2.000000	no	no	no	no	no	no	no	yes	yes	no
182227076	chr1	160368688	160368724	19	37	1.947368	no	no	yes	no	no	no	no	yes	yes	yes
182227080	chr1	160375625	160375666	24	42	1.916667	no	no	no	no	no	no	no	yes	yes	no
182227093	chr1	160400585	160400615	15	31	2.133333	no	no	no	no	yes	no	yes	yes	no	yes
182227100	chr1	160439662	160439711	26	50	1.961538	no	no	no	no	no	no	no	no	no	no
182227101	chr1	160439998	160440044	13	47	3.714286	no	no	no	no	no	no	no	yes	no	no
182227105	chr1	160448629	160448653	13	25	1.923077	no	no	no	no	no	no	no	no	no	no
182227110	chr1	160451759	160451791	11	33	3.090909	no	no	no	no	no	no	no	yes	yes	no
182227117	chr1	160481462	160481525	28	64	2.285714	no	no	no	no	no	no	no	yes	no	no
182227125	chr1	160489036	160489077	22	42	1.954545	no	no	yes	no	no	no	no	yes	no	yes
182227126	chr1	160496817	160496852	18	36	2.000000	no	no	yes	no	no	no	no	no	no	yes
182227131	chr1	160537276	160537306	16	31	1.937500	no	no	yes	no	no	no	no	no	no	yes
182227135	chr1	160549412	160549445	14	34	2.466667	no	no	yes	no	no	no	no	yes	yes	yes
182227141	chr1	160580068	160580119	28	52	1.964286	yes	no	no	no	no	no	no	yes	no	no
182227149	chr1	160586641	160586713	32	73	2.281250	no	no	no	no	no	no	no	no	no	no
182227171	chr1	160647161	160647193	17	33	2.000000	yes	no	no	no	yes	no	no	yes	yes	yes
182227175	chr1	160655060	160655116	19	57	3.000000	no	no	no	no	no	no	no	no	no	no
182227178	chr1	160656896	160656936	19	41	2.210526	no	no	no	no	no	no	no	yes	yes	no
182227183	chr1	160666406	160666449	20	44	2.200000	no	no	no	no	no	no	no	yes	yes	no
182227190	chr1	160677326	160677731	33	406	12.363636	no	no	yes	no	no	no	no	yes	yes	yes

182227192	chr1	160679569	160679605	18	37	2.111111	no	no	yes	no	no	yes	no	yes	no	yes
182227193	chr1	160680739	160680822	42	84	2.000000	no	yes	yes	no	no	no	no	yes	yes	yes
182227196	chr1	160704860	160704902	21	43	2.095238	no	no	yes	no	no	no	no	yes	no	yes
182227213	chr1	160744593	160744631	19	39	2.052632	no	no	yes	no	no	no	no	yes	yes	yes
182227214	chr1	160749423	160749465	22	43	1.954545	yes	no	yes	no	no	no	no	yes	no	yes
182227223	chr1	160770474	160770545	36	72	2.000000	no	no	no	no	no	no	no	no	no	no
182227224	chr1	160771286	160771318	15	33	2.200000	no	no	no	no	no	no	no	no	yes	no
182227226	chr1	160780083	160780196	37	114	3.081081	yes	no	no	no	yes	no	no	yes	yes	yes
182227232	chr1	160796067	160796116	24	50	2.083333	yes	no	no	no	yes	no	no	yes	yes	yes
182227233	chr1	160796108	160796143	18	36	2.000000	yes	no	no	no	yes	no	no	yes	yes	yes
182227236	chr1	160798057	160798098	19	42	2.315789	no	no	yes	no	no	no	no	yes	yes	yes
182227249	chr1	160837414	160837472	29	59	2.034483	no	no	yes	no	no	no	no	yes	no	yes
182227250	chr1	160842177	160842211	17	35	2.117647	no	no	yes	no	no	no	no	no	no	yes
182227254	chr1	160855826	160855877	25	52	2.080000	no	no	yes	no	no	no	no	yes	yes	yes
182227257	chr1	160859580	160859612	17	33	1.941176	no	no	yes	no	no	no	no	no	no	yes
182227260	chr1	160867274	160867299	12	26	2.166667	no	no	no	no	no	no	no	no	no	no
182227261	chr1	160868091	160868141	23	51	2.217391	no	no	no	no	no	no	no	no	no	no
182227271	chr1	160875744	160875830	24	87	3.750000	no	no	no	no	no	yes	no	yes	yes	no
182227274	chr1	160883657	160883729	24	73	3.041667	no	no	yes	no	no	no	no	no	yes	yes
182227276	chr1	160888756	160888805	19	50	2.631579	no	no	no	no	no	no	no	no	no	no
182227281	chr1	160901811	160901852	20	42	2.200000	no	no	no	no	no	no	no	no	no	no
182227287	chr1	160905150	160905202	18	53	2.944444	no	no	no	no	no	no	no	yes	yes	no
182227289	chr1	160907025	160907099	37	75	2.027027	no	no	no	no	no	no	no	no	no	no
182227313	chr1	160936140	160936186	23	47	2.086957	no	no	yes	no	no	yes	no	yes	yes	yes
182227316	chr1	160943264	160943305	21	42	2.000000	no	no	yes	no	no	no	no	yes	no	yes

182227322	chr1	160954907	160954936	15	30	2.000000	yes	no	no	no	no	no	no	no	yes	no
182227328	chr1	160962937	160962991	26	55	2.153846	no	no	no	no	no	no	no	yes	no	no
182227365	chr1	161019059	161019090	16	32	2.000000	no	no	yes	no	no	no	no	yes	yes	yes
182227366	chr1	161019261	161019293	16	33	2.062500	no	no	yes	no	no	no	no	yes	yes	yes
182227383	chr1	161049054	161049108	29	55	2.000000	no	yes	no	yes	yes	no	no	no	no	yes
182227388	chr1	161053656	161053680	12	25	2.083333	no	no	yes	no	no	yes	no	no	yes	yes
182227407	chr1	161067137	161067167	15	31	2.133333	no	no	yes	no	no	no	no	yes	yes	yes
182227411	chr1	161073342	161073394	27	53	2.000000	no	no	yes	no	no	no	no	yes	yes	yes
182227413	chr1	161080728	161080754	13	27	2.076923	no	no	yes	no	no	no	no	yes	yes	yes
182227414	chr1	161089123	161089164	22	42	1.909091	no	no	yes	no	no	no	no	yes	yes	yes
182227452	chr1	161148826	161148869	23	44	1.913043	no	no	no	no	no	no	no	no	no	no
182227460	chr1	161175641	161175678	19	38	2.000000	no	no	yes	no	no	yes	no	yes	no	yes
182227465	chr1	161179366	161179416	23	51	2.217391	no	no	no	no	no	no	no	no	yes	no
182227469	chr1	161189432	161189457	13	26	2.000000	no	yes	no	no	no	no	no	yes	yes	yes
182227485	chr1	161233898	161233982	20	85	4.250000	no	no	yes	no	no	no	no	yes	no	yes
182227522	chr1	161286927	161286960	16	34	2.125000	no	no	no	no	no	no	no	no	no	no
182227530	chr1	161299527	161299573	14	47	3.357143	no	no	no	no	no	no	no	yes	yes	no
182227542	chr1	161321938	161321988	21	51	2.428571	no	no	yes	no	no	no	no	no	no	yes
182227547	chr1	161328851	161328889	21	39	1.904762	no	no	yes	no	no	yes	no	no	no	yes
182227553	chr1	161343661	161343700	20	40	2.000000	no	no	yes	no	no	no	no	no	no	yes
182227562	chr1	161370566	161370595	14	30	2.142857	no	yes	yes	no	no	no	no	no	no	yes
182227573	chr1	161390239	161390284	16	46	2.875000	no	no	no	no	no	no	yes	yes	yes	no
182227581	chr1	161416784	161416835	24	52	2.208333	no	no	yes	no	no	no	no	no	no	yes
182227602	chr1	161428662	161428705	22	44	2.000000	no	no	yes	no	no	no	no	yes	yes	yes
182227658	chr1	161481736	161481786	20	51	2.550000	no	no	no	no	no	no	no	yes	yes	no

182227662	chr1	161489175	161489206	10	32	3.200000	no	no	no	no	no	no	no	no	no	no
182227666	chr1	161493293	161493427	70	135	2.028571	no	no	no	no	no	no	no	no	no	no
182227667	chr1	161496248	161496294	13	47	3.500000	no	no	no	no	no	no	no	no	no	no
182227668	chr1	161497187	161497228	19	42	2.210526	no	no	no	no	no	no	no	yes	no	no
182227678	chr1	161511079	161511104	12	26	2.166667	no	no	yes	no	no	yes	no	yes	yes	yes
182227681	chr1	161524437	161524466	10	30	3.000000	yes	no	yes	no	no	yes	yes	yes	yes	yes
182227683	chr1	161531245	161531292	24	48	2.000000	no	no	yes	no	no	no	no	yes	yes	yes
182227688	chr1	161545580	161545731	76	152	2.000000	no	no	yes	no	yes	no	no	yes	yes	yes
182227712	chr1	161631968	161631994	8	27	3.375000	yes	no	no	no	no	no	no	yes	no	no
182227715	chr1	161645634	161645671	18	38	2.166667	no	no	no	no	no	no	no	yes	no	no
182227733	chr1	161691966	161691996	16	31	1.937500	no	no	no	no	no	no	no	yes	yes	no
182227734	chr1	161717427	161717468	22	42	1.954545	no	no	no	no	no	no	no	yes	no	no
182227741	chr1	161739555	161739593	20	39	1.950000	no	no	no	no	no	no	no	yes	no	no
182227749	chr1	161753272	161753313	20	42	2.150000	no	yes	yes	no	no	yes	no	no	no	yes
182227751	chr1	161758591	161758627	19	37	2.000000	no	no	no	no	no	no	no	no	no	no
182227763	chr1	161777835	161777910	40	76	1.900000	no	no	yes	no	no	no	no	no	no	yes
182227765	chr1	161781323	161781453	65	131	2.030769	no	no	yes	no	no	no	no	no	no	yes
182227771	chr1	161795535	161795578	21	44	2.142857	no	no	yes	no	no	no	no	no	no	yes
182227782	chr1	161815651	161815677	12	27	2.250000	no	no	yes	no	no	no	no	no	no	yes
182227786	chr1	161835494	161835570	38	77	2.026316	no	no	yes	no	no	no	no	no	no	yes
182227787	chr1	161840411	161840454	23	44	1.913043	no	yes	yes	no	no	no	no	yes	no	yes
182227792	chr1	161853171	161853207	17	37	2.176471	no	no	yes	no	no	no	no	no	no	yes
182227793	chr1	161857020	161857048	13	29	2.230769	no	no	yes	no	no	no	no	no	no	yes
182227794	chr1	161857242	161857385	71	144	2.056338	no	no	yes	no	no	no	no	no	no	yes
182227798	chr1	161863631	161863659	15	29	1.933333	no	no	yes	no	no	no	no	no	no	yes

182227800	chr1	161869278	161869345	25	68	2.769231	no	no	yes	no	no	no	no	yes	no	yes
182227807	chr1	161900364	161900411	23	48	2.086957	no	no	yes	no	no	no	no	no	yes	yes
182227817	chr1	161921684	161921749	34	66	1.941176	no	no	yes	no	no	no	no	no	yes	yes
182227818	chr1	161921972	161922012	21	41	1.952381	no	no	yes	no	no	no	no	yes	no	yes
182227819	chr1	161931836	161931868	16	33	2.062500	no	no	yes	no	no	no	no	yes	no	yes
182227823	chr1	161960723	161960755	18	33	1.944444	no	yes	no	no	no	no	no	no	no	yes
182227827	chr1	161971623	161971671	22	49	2.227273	no	no	no	no	no	no	no	no	no	no
182227830	chr1	161974232	161974285	20	54	2.700000	no	no	no	no	no	no	no	yes	yes	no
182227834	chr1	161982041	161982073	14	33	2.357143	no	no	no	no	no	no	no	yes	no	no
182227835	chr1	161985151	161985183	12	33	2.750000	no	no	yes	no	yes	no	no	yes	no	yes
182227836	chr1	161994157	161994194	15	38	2.600000	no	no	yes	no	no	no	no	yes	yes	yes
182227838	chr1	162003635	162003673	20	39	1.950000	no	no	yes	no	no	no	no	yes	no	yes
182227840	chr1	162006919	162006956	10	38	4.000000	no	no	yes	no	no	no	no	no	no	yes
182227841	chr1	162011195	162011223	12	29	2.416667	no	no	yes	no	no	no	no	no	yes	yes
182227843	chr1	162023846	162023881	14	36	2.571429	yes	no	no	no	yes	no	yes	yes	yes	yes
182227847	chr1	162038913	162038948	18	36	2.000000	yes	no	no	no	no	no	no	yes	no	no
182227854	chr1	162060732	162060763	16	32	2.062500	no	no	no	no	no	no	no	no	yes	no
182227867	chr1	162112306	162112332	14	27	1.928571	no	no	yes	no	no	no	no	no	no	yes
182227868	chr1	162115977	162116042	34	66	2.000000	no	no	yes	no	no	no	no	no	yes	yes
182227872	chr1	162120053	162120149	45	97	2.177778	no	no	yes	no	no	no	no	yes	no	yes
182227875	chr1	162131254	162131283	14	30	2.142857	no	no	yes	no	no	no	no	yes	yes	yes
182227876	chr1	162145649	162145680	13	32	2.538461	no	no	yes	no	no	no	no	yes	yes	yes
182227880	chr1	162155368	162155410	17	43	2.529412	no	no	yes	no	no	no	no	no	no	yes
182227882	chr1	162155885	162155917	16	33	2.062500	no	no	yes	no	no	no	no	no	no	yes
182227890	chr1	162172697	162172742	22	46	2.227273	no	no	yes	no	no	no	no	yes	no	yes

182227891	chr1	162172781	162172811	15	31	2.066667	no	no	yes	no	no	no	no	yes	yes	yes
182227900	chr1	162220500	162220533	16	34	2.125000	no	no	yes	no	no	no	no	no	no	yes
182227907	chr1	162226451	162226498	22	48	2.227273	no	no	yes	no	no	no	no	yes	no	yes
182227911	chr1	162234420	162234485	30	66	2.200000	no	no	yes	no	no	no	no	no	no	yes
182227923	chr1	162271173	162271334	55	162	2.945455	no	no	yes	no	no	no	no	yes	no	yes
182227928	chr1	162299248	162299311	32	64	2.000000	no	no	yes	no	no	no	no	yes	yes	yes
182227932	chr1	162315184	162315215	13	32	2.428571	no	no	yes	no	no	no	no	no	no	yes
182227933	chr1	162316103	162316134	16	32	2.000000	no	no	yes	no	no	yes	no	yes	no	yes
182227938	chr1	162349644	162349710	35	67	1.914286	no	no	yes	no	no	no	no	yes	no	yes
182227939	chr1	162350626	162350658	17	33	1.941176	no	no	yes	no	no	no	no	yes	no	yes
182227940	chr1	162355492	162355523	16	32	2.000000	no	no	yes	no	no	no	no	no	no	yes
182227941	chr1	162357076	162357104	12	29	2.416667	no	no	no	yes	no	no	no	no	no	yes
182227946	chr1	162365056	162365106	20	51	2.550000	yes	no	yes	no	yes	no	no	no	no	yes
182227948	chr1	162378678	162378721	22	44	2.045455	yes	no	yes	no	no	no	no	no	no	yes
182227953	chr1	162400098	162400161	31	64	2.064516	no	no	yes	no	no	no	no	no	no	yes
182227961	chr1	162418355	162418410	18	56	3.111111	no	no	no	no	no	no	no	no	no	no
182227972	chr1	162446584	162446651	34	68	2.000000	no	no	no	no	no	yes	no	no	yes	no
182227976	chr1	162456889	162456924	13	36	2.846154	no	no	no	no	no	no	no	yes	no	no
182227993	chr1	162467449	162467480	16	32	2.000000	no	no	no	no	no	no	no	yes	no	no
182227994	chr1	162467639	162467678	21	40	1.904762	no	no	no	no	no	no	no	yes	no	no
182227997	chr1	162472352	162472385	17	34	2.058824	no	no	no	no	no	no	no	no	no	no
182228003	chr1	162487480	162487528	24	49	2.041667	no	no	no	no	no	no	no	yes	yes	no
182228005	chr1	162487880	162487978	24	99	4.125000	no	no	no	no	no	no	no	yes	no	no
182228009	chr1	162502228	162502270	22	43	2.000000	no	no	yes	no	no	no	no	yes	no	yes
182228015	chr1	162516658	162516705	22	48	2.181818	no	no	yes	no	no	no	no	yes	no	yes

182228017	chr1	162530099	162530138	15	40	2.625000	no	no	no	no	no	yes	no	no	no	no
182228033	chr1	162555207	162555245	20	39	2.000000	no	no	no	no	no	no	no	no	no	no
182228037	chr1	162560347	162560384	19	38	2.000000	no	no	no	no	yes	no	no	yes	yes	yes
182228045	chr1	162600934	162600966	13	33	2.615385	no	no	no	no	no	no	no	no	no	no
182228046	chr1	162610628	162610665	16	38	2.437500	no	no	no	no	no	no	no	no	yes	no
182228051	chr1	162627497	162627550	23	54	2.333333	no	no	no	no	no	no	no	yes	no	no
182228060	chr1	162634503	162634539	19	37	1.947368	no	no	yes	no	no	no	no	yes	yes	yes
182228072	chr1	162666212	162666241	14	30	2.142857	no	no	yes	no	no	no	no	yes	yes	yes
182228073	chr1	162666914	162666965	22	52	2.363636	no	no	yes	no	no	no	no	no	no	yes
182228075	chr1	162667044	162667088	24	45	1.958333	no	no	yes	no	no	no	no	no	no	yes
182228077	chr1	162668828	162668907	42	80	1.904762	no	no	yes	no	no	no	no	no	no	yes
182228084	chr1	162680617	162680702	43	86	2.000000	no	no	yes	no	no	no	no	yes	yes	yes
182228086	chr1	162682744	162682791	23	48	2.130435	no	no	yes	no	no	no	no	yes	no	yes
182228092	chr1	162698347	162698426	42	80	1.904762	no	no	yes	no	no	no	no	yes	yes	yes
182228093	chr1	162700007	162700063	27	57	2.111111	no	no	yes	no	no	no	no	no	yes	yes
182228094	chr1	162706130	162706156	13	27	2.076923	no	no	yes	no	no	no	no	yes	yes	yes
182228099	chr1	162721664	162721702	20	39	1.950000	no	no	yes	no	no	no	no	no	yes	yes
182228101	chr1	162727041	162727078	12	38	3.250000	no	no	yes	no	no	no	no	no	no	yes
182228105	chr1	162727100	162727274	27	175	6.481482	no	no	yes	no	no	no	no	no	no	yes
182228111	chr1	162727342	162727461	62	120	1.935484	no	no	yes	no	no	no	no	no	no	yes
182228114	chr1	162727507	162727560	28	54	1.928571	no	no	yes	no	no	no	no	no	no	yes
182228116	chr1	162727962	162728152	31	191	5.577778	no	no	yes	no	no	no	no	no	yes	yes
182228117	chr1	162728238	162728278	12	41	3.500000	no	no	yes	no	no	no	no	yes	no	yes
182228130	chr1	162753603	162753641	17	39	2.294118	no	no	yes	no	no	yes	no	yes	yes	yes
182228131	chr1	162760417	162760625	26	209	8.115385	no	no	yes	no	no	no	no	no	no	yes

182228137	chr1	162767283	162767325	21	43	2.047619	yes	yes	no	yes	no	no	no	no	no	yes
182228139	chr1	162768235	162768275	21	41	1.952381	no	no	yes	no	no	no	no	no	no	yes
182228140	chr1	162771587	162771624	19	38	2.105263	no	no	yes	no	no	yes	no	no	no	yes
182228151	chr1	162796523	162796583	33	61	1.909091	yes	no	yes	no	no	no	no	no	yes	yes
182228158	chr1	162837657	162837703	18	47	2.666667	no	no	yes	no	no	no	no	no	no	yes
182228165	chr1	162841159	162841196	15	38	2.533333	no	no	yes	no	no	no	no	no	yes	yes
182228171	chr1	162862528	162862559	16	32	2.000000	no	no	yes	no	no	no	no	no	yes	yes
182228173	chr1	162872546	162872585	21	40	1.952381	no	no	no	no	no	no	no	no	yes	no
182228181	chr1	162900271	162900380	26	110	4.230769	no	no	no	no	no	no	no	no	no	no
182228183	chr1	162901838	162901864	13	27	2.076923	no	no	no	no	no	no	no	no	no	no
182228195	chr1	162964773	162964809	18	37	2.055556	no	no	no	no	no	no	no	no	no	no
182228197	chr1	162991573	162991615	22	43	1.954545	no	no	no	no	no	no	no	no	yes	no
182228205	chr1	163019819	163019854	19	36	1.947368	no	no	no	no	no	no	no	no	no	no
182228206	chr1	163020168	163020202	16	35	2.187500	no	no	no	no	no	no	no	no	yes	no
182228210	chr1	163037456	163037942	94	487	5.350000	no	no	no	no	no	no	no	yes	no	no
182228213	chr1	163051687	163051724	20	38	1.900000	no	no	no	no	no	no	no	no	no	no
182228221	chr1	163061370	163061398	14	29	2.071429	no	no	no	no	no	no	no	no	no	no
182228229	chr1	163082726	163082831	53	106	2.000000	no	no	no	no	no	no	no	no	yes	no
182228233	chr1	163096602	163096916	157	315	2.006369	no	no	no	no	no	no	no	no	no	no
182228237	chr1	163105062	163105152	28	91	3.206897	no	no	no	no	no	no	no	yes	no	no
182228248	chr1	163127779	163127823	21	45	2.142857	no	no	yes	no	no	no	no	no	no	yes
182228259	chr1	163154950	163154994	20	45	2.250000	no	no	yes	no	no	no	no	no	yes	yes
182228262	chr1	163159784	163159822	17	39	2.352941	no	no	yes	no	no	no	no	no	no	yes
182228264	chr1	163159874	163159925	22	52	2.454545	no	no	yes	no	no	no	no	no	no	yes
182228266	chr1	163167968	163168010	22	43	2.000000	no	no	yes	no	no	no	no	no	yes	yes

182228269	chr1	163179676	163179709	16	34	2.187500	no	no	yes	no	no	no	no	no	no	yes
182228278	chr1	163198148	163198185	15	38	2.600000	no	no	yes	no	no	no	no	no	no	yes
182228288	chr1	163232516	163232553	16	38	2.375000	no	no	yes	no	no	no	no	no	yes	yes
182228301	chr1	163257314	163257393	42	80	1.952381	no	no	yes	no	no	no	no	no	no	yes
182228303	chr1	163259872	163259901	8	30	3.750000	no	yes	yes	no	no	no	no	no	no	yes
182228313	chr1	163314526	163314562	17	37	2.235294	no	no	yes	no	no	no	no	no	no	yes
182228317	chr1	163350398	163350441	23	44	1.956522	no	no	yes	no	no	no	no	no	no	yes
182228324	chr1	163370077	163370101	12	25	2.083333	no	no	no	no	no	no	no	no	no	no
182228336	chr1	163408438	163408519	39	82	2.128205	no	no	no	no	no	no	no	yes	no	no
182228346	chr1	163435742	163435777	13	36	2.769231	no	no	no	no	no	no	no	no	no	no
182228350	chr1	163452391	163452428	17	38	2.294118	no	no	no	no	no	no	no	no	no	no
182228352	chr1	163459489	163459518	15	30	2.000000	no	no	no	no	no	no	no	no	no	no
182228353	chr1	163462014	163462049	18	36	2.000000	no	no	no	no	no	no	no	no	no	no
182228360	chr1	163477224	163477260	16	37	2.312500	no	no	no	no	no	no	no	no	no	no
182228373	chr1	163525173	163525211	20	39	1.950000	no	no	no	no	no	no	no	no	no	no
182228378	chr1	163530429	163530492	21	64	3.047619	no	no	no	no	no	no	no	no	yes	no
182228386	chr1	163552087	163552386	39	300	7.717949	no	no	no	no	no	no	no	no	no	no
182228387	chr1	163552380	163552444	34	65	1.941176	no	no	no	no	no	no	no	no	no	no
182228390	chr1	163560985	163561019	8	35	4.375000	no	no	no	no	no	no	no	no	no	no
182228401	chr1	163601527	163601585	31	59	1.903226	no	no	no	no	no	no	no	no	no	no
182228404	chr1	163607179	163607231	27	53	2.000000	no	no	no	no	no	no	no	no	no	no
182228420	chr1	163657568	163657602	17	35	2.117647	no	no	no	no	no	no	no	no	no	no
182228422	chr1	163662732	163662757	12	26	2.166667	no	no	no	no	no	no	no	no	no	no
182228423	chr1	163663728	163663763	17	36	2.117647	no	no	no	no	no	no	no	no	no	no
182228429	chr1	163678636	163678681	23	46	2.000000	no	no	no	no	no	no	no	no	no	no

182228430	chr1	163686727	163686778	24	52	2.166667	no	no	no	no	no	no	no	no	no	no
182228431	chr1	163688552	163688584	12	33	2.750000	no	no	no	no	no	no	no	no	no	no
182228433	chr1	163700009	163700047	20	39	2.000000	no	no	no	no	no	no	no	no	no	no
182228434	chr1	163704328	163704361	16	34	2.125000	no	no	no	no	no	no	no	no	no	no
182228443	chr1	163710886	163710970	24	85	3.583333	no	no	no	no	no	no	no	yes	no	no
182228450	chr1	163714857	163714904	21	48	2.333333	no	no	no	no	no	no	no	no	no	no
182228451	chr1	163714885	163715653	38	769	20.842106	no	no	no	no	no	no	no	no	no	no
182228468	chr1	163765211	163765281	18	71	4.055555	no	no	no	no	no	no	no	no	no	no
182228469	chr1	163765302	163765334	15	33	2.200000	no	no	no	no	no	no	no	no	no	no
182228476	chr1	163769874	163769952	39	79	2.025641	yes	no	no	no	no	no	no	no	yes	no
182228478	chr1	163774551	163774578	10	28	2.800000	no	no	no	no	no	no	no	no	yes	no
182228484	chr1	163787093	163787148	27	56	2.074074	no	no	no	no	no	no	no	no	no	no
182228492	chr1	163802900	163802975	29	76	2.724138	no	no	no	no	no	no	no	no	no	no
182228495	chr1	163808095	163808135	20	41	2.050000	no	no	no	no	no	no	no	no	yes	no
182228502	chr1	163823180	163823218	18	39	2.166667	no	no	no	no	no	no	no	yes	yes	no
182228507	chr1	163833500	163833536	18	37	2.111111	no	no	no	no	no	no	no	no	no	no
182228508	chr1	163833819	163833863	20	45	2.250000	no	no	no	no	no	no	no	no	no	no
182228510	chr1	163839642	163839692	23	51	2.291667	no	no	no	no	no	no	no	no	no	no
182228516	chr1	163862646	163862671	12	26	2.166667	no	no	no	no	no	no	no	no	no	no
182228533	chr1	163986145	163986235	47	91	1.936170	no	no	no	no	no	no	no	no	no	no
182228534	chr1	163987569	163987594	13	26	2.000000	no	no	no	no	no	no	no	no	no	no
182228550	chr1	164042644	164042676	17	33	2.000000	no	no	no	no	no	no	no	no	no	no
182228551	chr1	164046976	164047018	18	43	2.388889	no	no	no	no	no	no	no	no	no	no
182228556	chr1	164055154	164055186	17	33	2.000000	no	no	no	no	no	no	no	no	no	no
182228559	chr1	164057707	164057779	33	73	2.212121	no	no	no	no	no	no	no	yes	no	no

182228574	chr1	164108619	164108648	15	30	2.000000	no	no	no	no	no	no	no	yes	yes	no
182228576	chr1	164131361	164131403	21	43	2.095238	no	no	no	no	no	no	no	no	no	no
182228581	chr1	164140511	164140554	16	44	2.750000	no	no	no	no	no	no	no	no	no	no
182228584	chr1	164152024	164152055	15	32	2.133333	no	no	no	no	no	no	no	no	no	no
182228599	chr1	164200725	164200761	15	37	2.533333	no	no	no	no	no	no	no	no	no	no
182228602	chr1	164200841	164200953	60	113	1.916667	no	no	no	no	no	no	no	no	no	no
182228628	chr1	164302210	164302265	17	56	3.352941	no	no	no	no	no	no	no	no	no	no
182228630	chr1	164306168	164306201	16	34	2.187500	no	no	no	no	no	no	no	no	no	no
182228633	chr1	164324571	164324617	16	47	2.937500	no	no	no	no	no	no	no	no	no	no
182228640	chr1	164339191	164339225	18	35	1.944444	no	no	no	no	no	no	no	no	no	no
182228652	chr1	164357308	164357367	18	60	3.333333	no	yes	no	no	no	yes	no	no	no	yes
182228656	chr1	164365017	164365060	23	44	1.913043	no	no	no	no	no	no	no	no	no	no
182228680	chr1	164431786	164431822	17	37	2.176471	no	no	no	no	no	no	no	no	no	no
182228699	chr1	164453673	164453783	30	111	3.774194	no	no	no	no	no	no	no	no	no	no
182228707	chr1	164474901	164474939	15	39	2.600000	no	no	no	no	no	no	no	yes	no	no
182228712	chr1	164477884	164477944	31	61	1.967742	no	no	no	no	no	no	no	no	no	no
182228730	chr1	164523087	164523190	34	104	3.058824	no	no	no	no	no	no	no	no	no	no
182228750	chr1	164557520	164557565	22	46	2.090909	no	no	yes	no	no	no	no	yes	yes	yes
182228757	chr1	164576860	164576893	18	34	1.944444	no	yes	yes	no	no	no	no	yes	yes	yes
182228770	chr1	164641242	164641271	15	30	2.000000	no	yes	yes	no	no	no	no	yes	yes	yes
182228772	chr1	164651234	164651306	26	73	2.807692	yes	no	yes	no	no	no	no	no	no	yes
182228774	chr1	164660993	164661038	23	46	2.000000	no	no	yes	no	no	no	no	yes	yes	yes
182228778	chr1	164664474	164664504	15	31	2.066667	no	no	yes	no	no	no	no	yes	yes	yes
182228779	chr1	164667349	164667383	14	35	2.500000	no	no	yes	no	no	no	no	yes	no	yes
182228780	chr1	164670712	164670747	17	36	2.117647	no	no	yes	no	no	no	no	no	yes	yes

182228786	chr1	164692187	164692250	31	64	2.064516	no	no	yes	no	no	no	no	no	yes	yes
182228790	chr1	164722602	164722636	18	35	1.944444	no	no	yes	no	no	no	no	no	yes	yes
182228792	chr1	164729358	164729390	13	33	2.500000	no	no	yes	no	no	no	no	yes	no	yes
182228794	chr1	164741161	164741200	13	40	3.076923	no	no	yes	no	no	no	no	no	no	yes
182228803	chr1	164776809	164776848	19	40	2.105263	no	no	yes	no	no	no	no	no	yes	yes
182228806	chr1	164780869	164780894	12	26	2.166667	no	no	yes	no	no	no	no	yes	no	yes
182228807	chr1	164781892	164781947	25	56	2.240000	no	no	yes	no	no	no	no	no	yes	yes
182228808	chr1	164786566	164786602	19	37	2.000000	no	no	yes	no	no	no	no	no	yes	yes
182228810	chr1	164792605	164792629	9	25	2.777778	no	yes	yes	yes	no	no	no	no	yes	yes
182228815	chr1	164804001	164804037	18	37	2.166667	no	no	yes	no	no	no	no	no	yes	yes
182228816	chr1	164812669	164812717	23	49	2.130435	yes	no	yes	no	no	no	no	no	no	yes
182228819	chr1	164824512	164824554	22	43	1.954545	no	no	yes	no	no	no	no	yes	no	yes
182228820	chr1	164825844	164825921	37	78	2.108108	no	no	yes	no	no	no	no	no	yes	yes
182228821	chr1	164827772	164827836	28	65	2.321429	yes	no	yes	no	no	no	no	no	no	yes
182228829	chr1	164844369	164844412	16	44	2.750000	no	no	yes	no	no	no	no	no	yes	yes
182228851	chr1	164924909	164924972	32	64	2.000000	no	no	no	no	no	no	no	no	no	no
182228853	chr1	164927559	164927634	32	76	2.437500	no	no	no	no	no	no	no	no	yes	no
182228861	chr1	164941497	164941634	58	138	2.379310	no	no	no	no	no	no	no	no	no	no
182228862	chr1	164941621	164941668	20	48	2.500000	no	no	no	no	no	no	no	no	no	no
182228884	chr1	165038030	165038074	25	45	1.920000	no	no	no	no	no	no	no	no	no	no
182228885	chr1	165051368	165051400	14	33	2.357143	no	no	no	no	no	no	no	no	no	no
182228898	chr1	165114985	165115023	16	39	2.437500	no	no	no	no	no	no	no	yes	yes	no
182228899	chr1	165115115	165115155	18	41	2.277778	no	no	no	no	no	no	no	yes	yes	no
182228902	chr1	165115910	165116316	57	407	7.133333	no	no	no	no	no	no	no	no	yes	no
182228903	chr1	165123032	165123069	20	38	1.900000	no	no	no	no	no	no	no	no	no	no

182228904	chr1	165127146	165127173	14	28	2.000000	no	no	no	no	no	no	no	no	no	no
182228910	chr1	165138838	165138877	20	40	2.000000	no	no	no	no	no	no	no	no	no	no
182228914	chr1	165150254	165150472	95	219	2.368421	no	no	no	no	no	no	no	yes	no	no
182228924	chr1	165193951	165193986	18	36	2.000000	no	no	no	no	no	no	no	no	no	no
182228934	chr1	165261241	165261291	27	51	1.962963	no	no	yes	no	no	no	no	no	no	yes
182228948	chr1	165283475	165283527	28	53	1.964286	no	no	yes	no	no	no	no	yes	no	yes
182228949	chr1	165287362	165287392	14	31	2.214286	no	no	yes	no	no	no	no	no	no	yes
182228959	chr1	165333728	165333770	16	43	2.687500	no	no	yes	no	no	no	no	no	no	yes
182228963	chr1	165354152	165354208	29	57	2.000000	no	no	yes	no	no	no	no	yes	yes	yes
182228967	chr1	165376795	165376830	16	36	2.250000	no	no	no	no	no	no	no	yes	no	no
182228971	chr1	165393196	165393225	7	30	4.285714	no	no	no	no	no	no	no	yes	yes	no
182228974	chr1	165402719	165402792	26	74	2.846154	no	no	yes	no	no	no	no	yes	no	yes
182228977	chr1	165403026	165403072	24	47	1.958333	no	no	yes	no	no	no	no	yes	no	yes
182228982	chr1	165412577	165412610	17	34	2.000000	no	no	yes	no	no	no	no	no	yes	yes
182228985	chr1	165432368	165432399	15	32	2.133333	no	no	yes	no	no	no	no	no	no	yes
182228988	chr1	165450656	165450704	23	49	2.173913	no	no	no	no	no	no	no	no	no	no
182228991	chr1	165456471	165456520	24	50	2.083333	no	no	no	no	no	no	no	yes	no	no
182228999	chr1	165480332	165480390	27	59	2.259259	no	no	yes	no	no	no	no	no	no	yes
182229005	chr1	165481933	165482025	42	93	2.279070	no	no	yes	no	no	no	no	yes	no	yes
182229007	chr1	165484565	165484605	16	41	2.562500	no	no	yes	no	no	no	no	no	no	yes
182229008	chr1	165485648	165485688	22	41	1.909091	no	no	yes	no	no	no	no	yes	no	yes
182229009	chr1	165495724	165495752	14	29	2.071429	no	no	yes	no	no	no	no	yes	no	yes
182229017	chr1	165524413	165524449	19	37	2.000000	no	no	yes	no	no	yes	no	no	no	yes
182229018	chr1	165529645	165529698	25	54	2.160000	yes	no	yes	no	no	no	no	yes	no	yes
182229021	chr1	165529707	165529969	144	263	1.847222	yes	no	yes	no	no	no	no	yes	no	yes

182229032	chr1	165549307	165549348	22	42	1.909091	no	no	yes	no	no	no	no	no	no	yes
182229044	chr1	165579385	165579420	16	36	2.250000	no	no	yes	no	no	no	no	yes	no	yes
182229050	chr1	165582869	165582905	18	37	2.055556	yes	no	no	no	no	yes	no	no	no	no
182229057	chr1	165583430	165583481	24	52	2.166667	no	no	no	no	no	yes	no	no	no	no
182229061	chr1	165596651	165596693	22	43	2.000000	no	no	no	no	no	no	no	no	no	no
182229066	chr1	165605258	165605361	52	104	2.000000	no	no	yes	no	no	no	no	yes	no	yes
182229069	chr1	165610187	165610232	16	46	2.937500	no	no	yes	no	no	no	no	no	no	yes
182229070	chr1	165612776	165612817	20	42	2.100000	no	no	yes	no	no	no	no	yes	no	yes
182229071	chr1	165616479	165616512	18	34	1.944444	no	no	yes	no	no	no	no	yes	yes	yes
182229080	chr1	165629989	165630019	16	31	1.937500	no	no	no	no	no	no	no	yes	yes	no
182229082	chr1	165634743	165634807	18	65	3.611111	no	no	yes	no	no	no	no	yes	yes	yes
182229085	chr1	165649318	165649352	16	35	2.187500	yes	yes	yes	no	no	yes	no	yes	yes	yes
182229098	chr1	165675304	165675340	14	37	2.714286	yes	no	yes	no	no	no	no	no	no	yes
182229108	chr1	165690169	165690231	29	63	2.206897	no	no	yes	no	no	no	no	no	no	yes
182229114	chr1	165698593	165698637	17	45	2.647059	yes	no	no	no	yes	no	no	yes	yes	yes
182229121	chr1	165708138	165708223	26	86	3.307692	yes	no	yes	no	no	no	no	no	no	yes
182229125	chr1	165718532	165718577	23	46	2.000000	no	no	no	no	no	no	no	yes	yes	no
182229127	chr1	165725025	165725075	16	51	3.312500	no	yes	no	no	no	no	no	no	no	yes
182229130	chr1	165731809	165731842	17	34	2.000000	no	no	yes	no	no	no	no	no	no	yes
182229141	chr1	165756814	165756864	18	51	2.750000	no	no	yes	no	no	no	no	no	no	yes
182229148	chr1	165761973	165762288	28	316	11.285714	no	no	yes	no	no	no	no	yes	no	yes
182229152	chr1	165787369	165787419	24	51	2.125000	no	no	yes	no	no	no	no	no	no	yes
182229181	chr1	165823722	165823775	28	54	1.928571	no	no	yes	no	no	no	no	yes	yes	yes
182229194	chr1	165859395	165859419	13	25	1.923077	no	no	yes	no	no	no	no	no	no	yes
182229212	chr1	165913891	165913938	23	48	2.086957	no	no	no	no	no	no	no	yes	yes	no

182229221	chr1	165955634	165955721	43	88	2.046512	no	no	no	no	no	no	no	no	no	no
182229222	chr1	165958173	165958267	35	95	2.914286	no	no	no	no	no	no	no	yes	no	no
182229238	chr1	165989131	165989194	34	64	1.911765	no	no	no	no	no	no	no	yes	no	no
182229245	chr1	166017726	166017805	40	80	2.000000	no	no	no	no	no	no	no	yes	no	no
182229246	chr1	166022764	166022799	13	36	2.846154	no	yes	no	no	no	no	no	no	no	yes
182229253	chr1	166025601	166025645	21	45	2.190476	no	no	no	no	no	no	no	yes	no	no
182229261	chr1	166041593	166041630	20	38	1.900000	yes	no	no	no	no	no	no	no	no	no
182229262	chr1	166045919	166045951	15	33	2.200000	no	no	no	no	no	no	no	no	no	no
182229265	chr1	166049348	166049403	15	56	3.733333	no	no	no	no	no	no	no	no	no	no
182229276	chr1	166085147	166085188	20	42	2.100000	no	no	yes	no	no	no	no	no	no	yes
182229277	chr1	166086925	166086964	15	40	2.666667	no	no	yes	no	no	no	no	yes	no	yes
182229285	chr1	166119066	166119107	21	42	2.095238	no	no	yes	no	no	no	no	yes	yes	yes
182229286	chr1	166128253	166128282	16	30	1.937500	no	no	yes	no	no	no	no	yes	no	yes
182229289	chr1	166139810	166139834	13	25	1.923077	no	no	yes	no	no	no	no	yes	no	yes
182229294	chr1	166170570	166170594	12	25	2.083333	no	no	yes	no	no	no	no	yes	no	yes
182229300	chr1	166192435	166192499	20	65	3.181818	no	no	yes	no	no	no	no	no	no	yes
182229332	chr1	166300289	166300326	20	38	1.900000	no	no	yes	no	no	no	no	no	no	yes
182229360	chr1	166351572	166351608	17	37	2.235294	no	no	no	no	no	no	no	no	no	no
182229363	chr1	166359113	166359139	13	27	2.076923	no	no	no	no	no	no	no	no	no	no
182229371	chr1	166385884	166385930	24	47	2.000000	no	no	no	no	no	no	no	yes	yes	no
182229374	chr1	166388434	166388491	23	58	2.521739	no	no	yes	no	no	no	no	no	no	yes
182229389	chr1	166451115	166451159	24	45	1.916667	no	no	yes	no	no	no	no	no	no	yes
182229396	chr1	166463461	166464179	26	719	27.466667	no	no	no	no	no	no	no	yes	no	no
182229409	chr1	166519817	166519854	20	38	1.900000	no	no	no	no	no	no	no	no	no	no
182229423	chr1	166575239	166575364	63	126	2.000000	no	no	yes	no	no	no	no	no	no	yes

182229426	chr1	166586369	166586404	16	36	2.250000	no	no	no	no	no	no	no	yes	no	no
182229427	chr1	166597287	166597335	23	49	2.217391	no	no	no	no	no	no	no	no	no	no
182229428	chr1	166597739	166597773	11	35	3.166667	no	no	no	no	no	no	no	no	yes	no
182229429	chr1	166601673	166601708	17	36	2.117647	no	no	no	no	no	no	no	no	no	no
182229433	chr1	166603028	166603062	18	35	2.000000	yes	no	no	no	no	no	no	no	no	no
182229438	chr1	166623802	166623841	21	40	1.952381	no	no	yes	no	no	no	no	yes	no	yes
182229439	chr1	166624036	166624075	22	40	1.909091	no	no	yes	no	no	no	no	yes	no	yes
182229442	chr1	166632763	166632801	20	39	1.950000	no	no	no	no	no	no	no	yes	no	no
182229443	chr1	166636311	166636351	22	41	1.954545	no	no	no	no	no	no	no	no	no	no
182229447	chr1	166650739	166650783	24	45	1.916667	no	no	no	no	no	no	no	no	no	no
182229452	chr1	166654465	166654510	22	46	2.136364	no	no	no	no	no	no	no	no	no	no
182229454	chr1	166659300	166659342	12	43	3.583333	no	no	no	no	no	no	no	no	no	no
182229456	chr1	166665342	166665371	13	30	2.307692	yes	no	no	no	no	no	no	no	no	no
182229460	chr1	166670510	166670566	21	57	2.714286	no	no	yes	no	no	no	no	no	no	yes
182229461	chr1	166677369	166677498	47	130	2.897959	no	no	yes	no	no	no	no	yes	no	yes
182229465	chr1	166680593	166680628	17	36	2.117647	no	no	yes	no	no	no	no	no	no	yes
182229466	chr1	166687047	166687081	12	35	3.083333	no	no	no	no	no	no	no	no	no	no
182229474	chr1	166704595	166704653	22	59	2.681818	no	no	no	no	no	no	no	yes	no	no
182229481	chr1	166740287	166740327	20	41	2.150000	no	no	no	no	no	no	no	no	no	no
182229483	chr1	166742213	166742243	16	31	1.937500	no	no	no	no	no	no	no	no	no	no
182229488	chr1	166766484	166766530	22	47	2.136364	no	no	yes	no	no	no	no	no	no	yes
182229497	chr1	166808075	166808107	17	33	1.941176	no	no	no	no	no	no	no	no	no	no
182229519	chr1	166881371	166881408	15	38	2.533333	no	no	no	no	no	no	no	no	no	no
182229525	chr1	166900108	166900293	95	186	2.063158	no	no	yes	no	no	no	no	no	no	yes
182229526	chr1	166902391	166902487	46	97	2.108696	no	no	yes	no	no	no	no	yes	yes	yes

182229531	chr1	166943580	166943609	13	30	2.307692	no	no	yes	no	no	no	no	no	no	yes
182229533	chr1	166946094	166946156	32	63	1.968750	no	no	yes	no	no	no	no	no	yes	yes
182229535	chr1	166964658	166964682	12	25	2.083333	no	no	yes	no	no	no	no	yes	no	yes
182229539	chr1	166975574	166975619	20	46	2.285714	yes	no	no	no	yes	no	yes	yes	yes	yes
182229555	chr1	167060555	167060591	16	37	2.312500	no	no	yes	no	no	no	no	yes	no	yes
182229560	chr1	167064787	167064851	33	65	1.969697	no	no	yes	no	no	no	no	yes	yes	yes
182229563	chr1	167067030	167067075	22	46	2.090909	no	no	yes	no	no	no	no	yes	yes	yes
182229565	chr1	167068081	167068117	17	37	2.235294	no	no	yes	no	no	no	no	no	no	yes
182229567	chr1	167090436	167090485	25	50	2.000000	yes	no	yes	no	no	no	no	yes	no	yes
182229574	chr1	167110672	167110716	21	45	2.142857	no	no	yes	no	no	no	no	yes	yes	yes
182229576	chr1	167114797	167114835	14	39	2.687500	no	no	yes	no	no	no	no	yes	yes	yes
182229580	chr1	167126357	167126392	13	36	2.846154	no	no	yes	yes	no	no	no	no	no	yes
182229581	chr1	167135127	167135164	18	38	2.111111	no	no	yes	no	no	no	no	no	no	yes
182229582	chr1	167138001	167138038	20	38	1.900000	no	no	yes	no	no	no	no	yes	no	yes
182229583	chr1	167139313	167139356	16	44	2.750000	no	no	yes	no	no	no	no	yes	yes	yes
182229594	chr1	167155150	167155193	16	44	2.750000	no	no	yes	no	no	no	no	no	no	yes
182229600	chr1	167175519	167175560	20	42	2.200000	no	no	no	no	yes	yes	no	no	no	yes
182229616	chr1	167210740	167210771	11	32	2.909091	no	no	no	no	no	no	no	no	no	no
182229621	chr1	167236408	167236464	28	57	2.142857	no	no	yes	no	no	no	no	yes	no	yes
182229624	chr1	167238269	167238300	14	32	2.357143	no	no	yes	no	no	no	no	no	no	yes
182229625	chr1	167239170	167239214	23	45	1.956522	no	no	yes	no	no	no	no	yes	no	yes
182229628	chr1	167247856	167247891	16	36	2.312500	no	no	yes	no	no	no	no	yes	no	yes
182229631	chr1	167278815	167278850	16	36	2.312500	no	no	yes	no	no	no	no	no	no	yes
182229638	chr1	167330603	167330659	29	57	1.965517	no	no	yes	no	no	no	no	yes	yes	yes
182229647	chr1	167348918	167348949	16	32	2.000000	no	no	yes	no	no	no	no	no	no	yes

182229653	chr1	167357341	167357387	20	47	2.350000	yes	no	yes	no	no	no	no	yes	yes	yes
182229660	chr1	167383150	167383174	13	25	1.923077	yes	no	yes	no	no	no	no	no	no	yes
182229662	chr1	167391536	167391562	12	27	2.250000	no	no	yes	no	no	no	no	yes	no	yes
182229667	chr1	167402686	167402742	26	57	2.230769	no	no	yes	no	no	no	no	no	no	yes
182229674	chr1	167416077	167416139	32	63	1.968750	no	yes	no	yes	no	yes	no	no	no	yes
182229676	chr1	167421761	167421800	22	40	1.909091	no	yes	no	no	no	no	no	yes	no	yes
182229681	chr1	167434906	167435361	111	456	4.114035	yes	no	yes	no	yes	no	no	no	no	yes
182229684	chr1	167438119	167438144	12	26	2.166667	no	no	yes	no	no	no	no	no	no	yes
182229691	chr1	167456636	167456668	17	33	2.000000	no	no	yes	no	no	yes	no	no	yes	yes
182229698	chr1	167472780	167472810	15	31	2.066667	no	no	yes	no	no	no	no	no	no	yes
182229703	chr1	167493361	167493403	22	43	2.045455	no	no	yes	no	no	no	no	yes	no	yes
182229705	chr1	167506213	167506334	26	122	4.642857	no	no	yes	no	no	no	no	no	no	yes
182229706	chr1	167510211	167510253	23	43	1.956522	no	no	yes	no	no	no	no	yes	yes	yes
182229715	chr1	167542140	167542171	12	32	2.666667	no	yes	yes	no	no	no	no	yes	yes	yes
182229717	chr1	167550816	167550844	15	29	1.933333	no	no	yes	no	no	no	no	yes	yes	yes
182229754	chr1	167646506	167646544	18	39	2.166667	no	no	yes	no	no	no	no	yes	no	yes
182229757	chr1	167663340	167663364	13	25	1.923077	no	no	yes	no	no	no	no	yes	yes	yes
182229758	chr1	167667104	167667178	38	75	2.000000	no	no	yes	no	no	no	no	no	no	yes
182229764	chr1	167696214	167696245	15	32	2.133333	no	no	yes	no	no	no	no	no	no	yes
182229770	chr1	167705053	167705099	16	47	2.937500	no	yes	no	no	no	no	no	no	no	yes
182229771	chr1	167706433	167706467	18	35	2.000000	no	yes	no	no	no	yes	no	no	yes	yes
182229774	chr1	167719580	167719639	31	60	1.935484	no	no	no	no	no	no	no	yes	yes	no
182229795	chr1	167776262	167776300	20	39	1.950000	no	no	yes	no	no	no	no	yes	yes	yes
182229811	chr1	167809102	167809152	20	51	2.650000	no	no	no	no	no	yes	no	no	no	no
182229815	chr1	167820320	167820363	19	44	2.368421	yes	no	yes	no	no	no	yes	yes	yes	yes

182229817	chr1	167839495	167839526	17	32	1.941176	no	no	yes	no	no	no	no	no	no	yes
182229819	chr1	167843334	167843378	18	45	2.500000	no	no	yes	no	no	no	no	yes	no	yes
182229839	chr1	167910457	167910497	18	41	2.277778	no	no	yes	no	no	no	no	yes	no	yes
182229841	chr1	167919093	167919163	25	71	2.777778	no	no	yes	no	no	yes	no	no	no	yes
182229847	chr1	167935412	167935446	18	35	2.000000	yes	no	yes	no	no	no	no	yes	yes	yes
182229868	chr1	167993940	167994019	24	80	3.320000	no	no	yes	no	no	yes	no	yes	no	yes
182229872	chr1	168002030	168002069	18	40	2.222222	no	no	yes	no	no	no	no	no	no	yes
182229880	chr1	168008952	168009004	21	53	2.571429	no	no	yes	no	no	no	no	no	no	yes
182229885	chr1	168015630	168015662	15	33	2.333333	yes	no	yes	no	no	no	no	yes	no	yes
182229886	chr1	168018495	168018535	21	41	2.095238	no	no	yes	no	no	no	no	no	no	yes
182229889	chr1	168039561	168039624	31	64	2.129032	no	no	yes	no	no	no	no	no	no	yes
182229893	chr1	168046735	168046780	21	46	2.190476	no	no	yes	no	no	no	no	no	no	yes
182229896	chr1	168066139	168066184	22	46	2.181818	no	no	yes	no	no	no	no	no	no	yes
182229897	chr1	168066821	168066864	23	44	1.913043	no	no	yes	no	no	no	no	no	no	yes
182229902	chr1	168074033	168074068	17	36	2.117647	no	no	yes	no	no	no	no	yes	no	yes
182229905	chr1	168090484	168090533	16	50	3.125000	yes	no	yes	no	yes	no	no	yes	no	yes
182229919	chr1	168136943	168136983	21	41	1.952381	yes	no	yes	no	no	no	no	yes	yes	yes
182229920	chr1	168140785	168140860	40	76	1.975000	no	no	no	no	no	no	no	no	no	no
182229926	chr1	168146099	168146208	20	110	5.500000	no	no	no	no	no	no	no	yes	no	no
182229928	chr1	168146254	168146291	19	38	2.000000	no	no	no	no	no	no	no	yes	no	no
182229929	chr1	168146348	168146387	19	40	2.105263	no	no	no	no	no	no	no	yes	no	no
182229942	chr1	168164643	168164677	17	35	2.058824	no	no	no	no	no	no	no	no	no	no
182229949	chr1	168167854	168167882	14	29	2.142857	no	no	no	no	no	no	no	yes	yes	no
182229952	chr1	168176333	168176369	18	37	2.055556	no	no	no	no	no	no	no	no	no	no
182229982	chr1	168220798	168220849	27	52	1.925926	no	no	no	no	no	no	no	yes	yes	no

182229985	chr1	168232418	168232450	17	33	1.941176	no	no	yes	no	no	no	no	no	yes	yes
182229989	chr1	168261094	168261142	21	49	2.333333	no	no	no	no	no	no	no	no	yes	no
182229992	chr1	168283540	168283588	25	49	2.040000	no	no	yes	no	no	no	no	no	no	yes
182229993	chr1	168284069	168284127	30	59	1.966667	no	no	yes	no	no	no	no	no	no	yes
182230005	chr1	168302671	168302712	19	42	2.210526	no	no	yes	no	no	no	no	no	no	yes
182230006	chr1	168304860	168304900	18	41	2.277778	no	no	yes	no	no	no	no	no	yes	yes
182230010	chr1	168310758	168310834	36	77	2.222222	no	no	yes	no	no	no	no	no	no	yes
182230012	chr1	168311048	168311105	30	58	1.966667	no	no	yes	no	no	no	no	no	no	yes
182230015	chr1	168316814	168316926	56	113	2.017857	yes	no	no	no	no	no	no	yes	no	no
182230018	chr1	168320535	168320598	32	64	2.000000	no	no	yes	no	no	no	no	yes	yes	yes
182230035	chr1	168347461	168347498	20	38	1.950000	no	no	yes	no	no	no	no	no	no	yes
182230052	chr1	168405367	168405455	39	89	2.307692	no	no	yes	no	no	no	no	no	no	yes
182230055	chr1	168408822	168408874	26	53	2.038461	no	no	yes	no	no	no	no	yes	no	yes
182230067	chr1	168449640	168449672	17	33	1.941176	no	no	yes	no	yes	yes	no	no	no	yes
182230071	chr1	168457943	168457984	20	42	2.100000	no	no	yes	no	no	no	no	no	no	yes
182230077	chr1	168471606	168471642	18	37	2.055556	no	no	yes	no	no	no	no	no	no	yes
182230091	chr1	168521666	168521707	21	42	2.000000	no	no	no	no	no	no	no	no	yes	no
182230095	chr1	168523307	168523356	12	50	4.166667	no	no	no	no	no	no	no	yes	yes	no
182230111	chr1	168591982	168592031	21	50	2.523809	no	no	no	no	no	no	no	yes	no	no
182230117	chr1	168601819	168601874	18	56	3.111111	no	no	no	no	no	no	no	no	no	no
182230119	chr1	168604374	168604434	28	61	2.178571	no	no	no	no	no	no	no	no	no	no
182230124	chr1	168633620	168633666	16	47	2.882353	no	no	no	no	no	no	no	yes	no	no
182230125	chr1	168636628	168636664	16	37	2.312500	no	no	no	no	no	no	no	yes	no	no
182230130	chr1	168641814	168641931	52	118	2.288461	no	no	no	no	no	no	no	no	no	no
182230131	chr1	168642162	168642190	15	29	1.933333	no	no	no	no	no	no	no	no	no	no

182230137	chr1	168666322	168666362	15	41	2.800000	no	no	no	no	no	no	no	no	no	no
182230138	chr1	168666689	168666789	50	101	2.020000	no	no	no	no	no	no	no	no	no	no
182230153	chr1	168716219	168716258	12	40	3.333333	no	no	yes	no	no	no	no	no	no	yes
182230159	chr1	168725322	168725362	18	41	2.333333	no	no	yes	no	no	no	no	yes	no	yes
182230176	chr1	168776845	168776901	18	57	3.166667	no	no	yes	no	no	no	no	no	no	yes
182230178	chr1	168784331	168784374	17	44	2.555556	no	no	yes	no	no	no	no	no	no	yes
182230187	chr1	168791971	168792012	22	42	1.909091	no	no	yes	no	no	no	no	no	no	yes
182230196	chr1	168807747	168807778	16	32	2.000000	no	no	yes	no	no	no	no	yes	no	yes
182230208	chr1	168829123	168829193	34	71	2.176471	no	no	yes	no	no	no	no	no	no	yes
182230216	chr1	168835024	168835132	57	109	1.947368	no	no	yes	no	no	no	no	yes	no	yes
182230223	chr1	168845595	168845630	18	36	2.055556	no	no	yes	no	no	no	no	no	no	yes
182230246	chr1	168908880	168908926	22	47	2.136364	no	no	yes	no	no	no	no	no	no	yes
182230252	chr1	168921308	168921356	23	49	2.130435	no	no	yes	no	no	no	no	yes	no	yes
182230253	chr1	168921891	168921942	20	52	2.600000	no	no	yes	no	no	no	no	yes	yes	yes
182230259	chr1	168932999	168933228	117	230	1.965812	no	no	yes	no	no	no	no	no	no	yes
182230265	chr1	168944477	168944570	46	94	2.043478	no	no	yes	no	no	no	no	no	no	yes
182230271	chr1	168959965	168960021	29	57	1.965517	no	no	yes	no	no	no	no	no	no	yes
182230272	chr1	168965354	168965385	12	32	2.666667	no	no	yes	no	no	no	no	no	no	yes
182230281	chr1	168978668	168978693	7	26	3.714286	no	no	yes	no	no	no	no	no	no	yes
182230293	chr1	169015909	169015954	15	46	3.066667	no	no	yes	no	no	no	no	yes	no	yes
182230294	chr1	169017422	169017454	17	33	1.941176	no	no	yes	no	no	no	no	no	no	yes
182230296	chr1	169020364	169020400	18	37	2.055556	no	no	yes	no	no	no	no	no	no	yes
182230312	chr1	169055590	169055730	70	141	2.014286	no	no	yes	no	no	no	no	no	no	yes
182230314	chr1	169061858	169061906	21	49	2.333333	no	no	yes	no	no	no	no	yes	no	yes
182230321	chr1	169073482	169073626	72	145	2.013889	no	no	yes	no	no	no	no	yes	yes	yes

182230334	chr1	169095655	169095690	16	36	2.312500	no	no	yes	no	no	no	no	no	no	yes
182230335	chr1	169106125	169106151	14	27	1.928571	yes	no	yes	no	no	no	yes	yes	no	yes
182230338	chr1	169114016	169114079	32	64	2.000000	no	no	yes	no	no	no	no	yes	yes	yes
182230350	chr1	169139126	169139188	20	63	3.150000	no	no	yes	no	no	no	no	yes	yes	yes
182230357	chr1	169173281	169173322	23	42	1.913043	no	no	yes	no	no	no	no	no	no	yes
182230358	chr1	169173618	169173668	18	51	2.833333	no	no	yes	no	no	no	no	no	no	yes
182230369	chr1	169210666	169210705	18	40	2.277778	no	no	yes	no	no	no	no	yes	no	yes
182230370	chr1	169212223	169212247	12	25	2.083333	no	no	yes	no	no	no	no	yes	no	yes
182230372	chr1	169212260	169212298	12	39	3.153846	no	no	yes	no	no	no	no	yes	no	yes
182230374	chr1	169221641	169221672	16	32	2.062500	no	no	yes	no	no	no	no	no	no	yes
182230381	chr1	169233898	169233980	45	83	1.911111	no	no	yes	no	no	no	no	no	no	yes
182230394	chr1	169267794	169267834	18	41	2.277778	no	no	yes	no	no	no	no	no	no	yes
182230395	chr1	169271746	169271776	16	31	1.937500	no	no	yes	no	no	no	no	yes	no	yes
182230396	chr1	169272121	169272158	15	38	2.600000	no	no	yes	no	no	no	no	no	no	yes
182230401	chr1	169305250	169305285	18	36	2.000000	no	no	yes	no	no	no	no	no	no	yes
182230405	chr1	169317952	169317987	18	36	2.000000	no	no	yes	no	no	no	no	no	no	yes
182230409	chr1	169319439	169319477	17	39	2.294118	no	no	yes	no	no	no	no	no	no	yes
182230418	chr1	169342488	169343033	60	546	8.985915	no	no	yes	no	no	no	no	no	no	yes
182230422	chr1	169348666	169348707	13	42	3.230769	no	no	yes	no	no	no	no	yes	yes	yes
182230424	chr1	169349463	169349530	32	68	2.125000	no	no	yes	no	no	no	no	no	no	yes
182230431	chr1	169354744	169354808	32	65	2.062500	no	no	yes	no	no	no	no	no	no	yes
182230440	chr1	169367155	169367186	12	32	2.666667	yes	no	yes	no	no	no	no	yes	no	yes
182230448	chr1	169395802	169395841	19	40	2.105263	no	no	yes	no	no	no	no	no	no	yes
182230449	chr1	169396538	169396584	16	47	2.882353	no	yes	yes	no	no	yes	no	no	no	yes
182230459	chr1	169423186	169423227	22	42	1.909091	no	no	yes	no	no	no	no	no	no	yes

182230465	chr1	169435991	169436022	17	32	1.941176	no	no	yes	no	no	no	no	no	no	yes
182230475	chr1	169512062	169512094	17	33	1.941176	no	yes	no	no	no	no	no	yes	no	yes
182230476	chr1	169516454	169516495	18	42	2.333333	yes	no	yes	no	no	no	no	no	no	yes
182230480	chr1	169536232	169536261	15	30	2.000000	no	no	yes	no	no	no	no	no	no	yes
182230485	chr1	169542304	169542407	51	104	2.039216	no	no	no	yes	no	no	no	no	no	yes
182230489	chr1	169561892	169561927	15	36	2.400000	no	no	yes	no	no	no	no	yes	no	yes
182230493	chr1	169561946	169561990	21	45	2.190476	no	no	yes	no	no	no	no	yes	no	yes
182230505	chr1	169593915	169594187	136	273	2.007353	no	no	yes	no	no	no	no	no	no	yes
182230509	chr1	169605787	169605827	21	41	1.952381	no	no	yes	no	no	no	no	no	no	yes
182230514	chr1	169620730	169620900	86	171	1.988372	no	no	yes	no	no	no	no	yes	no	yes
182230519	chr1	169620960	169621447	134	488	3.628572	no	no	yes	no	no	no	no	yes	no	yes
182230523	chr1	169629124	169629306	90	183	2.033333	no	no	yes	no	no	no	no	no	no	yes
182230559	chr1	169699670	169699708	17	39	2.294118	no	no	yes	no	no	no	no	yes	yes	yes
182230560	chr1	169706334	169706395	31	62	2.000000	no	no	yes	no	no	no	no	yes	no	yes
182230561	chr1	169706905	169706939	18	35	1.944444	no	no	yes	no	no	no	no	yes	yes	yes
182230562	chr1	169711812	169711844	17	33	1.941176	yes	no	yes	no	no	no	no	yes	yes	yes
182230564	chr1	169711969	169712005	15	37	2.500000	yes	no	yes	no	no	no	no	yes	no	yes
182230573	chr1	169737177	169737217	22	41	1.909091	no	no	yes	no	no	no	no	yes	no	yes
182230577	chr1	169737374	169737504	29	131	4.468750	no	no	yes	no	no	no	no	no	no	yes
182230578	chr1	169737979	169738009	15	31	2.066667	no	no	yes	no	no	no	no	no	no	yes
182230582	chr1	169750384	169750419	14	36	2.571429	no	no	yes	no	no	no	no	yes	no	yes
182230587	chr1	169763866	169763903	18	38	2.111111	no	no	yes	no	no	no	no	no	yes	yes
182230592	chr1	169787167	169787201	7	35	5.000000	no	no	yes	no	no	no	no	yes	yes	yes
182230593	chr1	169787841	169787872	16	32	2.000000	no	no	yes	no	no	no	no	no	no	yes
182230594	chr1	169788786	169788819	16	34	2.187500	no	no	yes	no	no	no	no	no	no	yes

182230595	chr1	169797906	169797933	12	28	2.333333	no	no	yes	no	no	no	no	no	no	yes
182230598	chr1	169811651	169811890	24	240	9.884615	no	no	yes	no	no	no	no	yes	no	yes
182230612	chr1	169889354	169889393	21	40	1.904762	yes	no	yes	no	no	no	no	no	yes	yes
182230617	chr1	169909336	169909366	15	31	2.066667	no	no	no	no	no	no	no	yes	no	no
182230622	chr1	169920973	169921007	17	35	2.117647	no	no	no	no	no	yes	no	no	no	no
182230623	chr1	169922966	169923010	24	45	1.958333	no	no	yes	no	no	no	no	no	no	yes
182230624	chr1	169929322	169929361	15	40	2.666667	no	no	yes	no	no	no	no	yes	no	yes
182230625	chr1	169932278	169932315	20	38	1.900000	no	no	yes	no	no	no	no	yes	no	yes
182230628	chr1	169937647	169937708	27	62	2.407408	no	no	yes	no	no	no	no	no	no	yes
182230629	chr1	169937681	169937742	28	62	2.214286	no	no	yes	no	no	no	no	no	no	yes
182230630	chr1	169937719	169937771	18	53	2.894737	no	no	yes	no	no	no	no	no	no	yes
182230632	chr1	169956038	169956067	9	30	3.444444	no	no	yes	no	no	no	no	no	no	yes
182230633	chr1	169971018	169971066	22	49	2.227273	no	no	yes	no	no	no	no	no	no	yes
182230634	chr1	169994670	169994707	21	38	1.904762	no	no	yes	no	no	no	no	no	no	yes
182230635	chr1	169994740	169994772	17	33	1.941176	no	no	yes	no	no	no	no	no	no	yes
182230642	chr1	170018557	170018620	34	64	1.970588	no	no	yes	no	no	no	no	no	no	yes
182230646	chr1	170023859	170023891	15	33	2.200000	yes	no	yes	no	no	yes	no	no	no	yes
182230647	chr1	170023987	170024041	20	55	2.750000	yes	no	yes	no	no	yes	no	no	no	yes
182230650	chr1	170032591	170032645	27	55	2.074074	yes	no	yes	no	no	no	no	no	yes	yes
182230658	chr1	170083673	170083721	22	49	2.272727	no	no	yes	no	no	no	no	no	no	yes
182230663	chr1	170102163	170102210	21	48	2.333333	no	no	no	no	no	no	no	no	yes	no
182230665	chr1	170114800	170114853	18	54	3.000000	yes	no	no	no	no	no	no	yes	no	no
182230668	chr1	170134279	170134307	14	29	2.071429	no	no	no	no	no	no	no	no	yes	no
182230678	chr1	170157311	170157343	17	33	1.941176	yes	no	yes	no	no	no	no	no	no	yes
182230681	chr1	170165727	170165764	15	38	2.600000	no	no	yes	no	no	no	no	no	no	yes

182230690	chr1	170181264	170181325	31	62	2.000000	no	no	yes	no	no	no	no	no	no	yes
182230696	chr1	170202317	170202343	14	27	1.928571	no	no	yes	no	no	no	no	no	no	yes
182230702	chr1	170214781	170214856	30	76	2.533333	no	no	yes	no	no	no	no	yes	yes	yes
182230706	chr1	170219126	170219154	13	29	2.230769	no	no	yes	no	no	no	no	yes	no	yes
182230713	chr1	170234111	170234258	55	148	2.690909	yes	no	yes	no	no	no	no	no	no	yes
182230716	chr1	170244390	170244427	19	38	2.000000	no	no	yes	no	no	no	no	yes	yes	yes
182230717	chr1	170248518	170248550	15	33	2.200000	no	no	yes	no	no	no	no	yes	no	yes
182230728	chr1	170271713	170271752	20	40	2.050000	no	no	yes	no	yes	yes	no	yes	no	yes
182230729	chr1	170272159	170272189	15	31	2.066667	no	no	yes	no	no	no	no	no	no	yes
182230739	chr1	170305738	170305779	22	42	2.000000	no	no	no	no	no	no	no	yes	no	no
182230757	chr1	170397133	170397164	12	32	2.666667	no	no	no	no	no	no	no	yes	yes	no
182230758	chr1	170399634	170399683	27	50	1.925926	no	no	no	no	no	no	no	no	no	no
182230760	chr1	170405365	170405416	27	52	1.925926	no	no	no	no	no	no	no	no	no	no
182230761	chr1	170410760	170410791	12	32	2.666667	no	no	no	no	no	no	no	yes	no	no
182230775	chr1	170440271	170440303	18	33	1.944444	no	no	no	no	no	no	no	no	no	no
182230784	chr1	170486787	170486841	21	55	2.619048	no	no	yes	no	no	no	no	no	no	yes
182230786	chr1	170497732	170497760	13	29	2.230769	no	no	yes	no	no	no	no	no	no	yes
182230799	chr1	170537043	170537137	51	95	1.941176	no	no	yes	no	no	no	no	yes	no	yes
182230808	chr1	170567235	170567267	16	33	2.062500	no	no	no	no	no	no	no	no	no	no
182230815	chr1	170583364	170583425	18	62	3.444444	no	no	no	no	no	no	no	yes	yes	no
182230820	chr1	170605190	170605227	14	38	2.666667	no	no	yes	no	no	no	no	no	no	yes
182230837	chr1	170660719	170660761	15	43	3.000000	no	no	no	no	no	no	no	yes	yes	no
182230844	chr1	170674317	170674354	20	38	1.900000	no	no	yes	no	no	no	no	yes	no	yes
182230845	chr1	170677822	170677865	18	44	2.444444	no	no	yes	no	no	no	no	no	no	yes
182230847	chr1	170683445	170683487	16	43	2.750000	no	no	yes	no	no	no	no	no	no	yes

182230851	chr1	170691511	170691569	24	59	2.480000	no	no	yes	no	no	no	no	yes	no	yes
182230857	chr1	170694085	170694128	23	44	1.913043	no	no	yes	no	no	no	no	no	yes	yes
182230858	chr1	170697508	170697572	32	65	2.062500	no	no	yes	no	no	no	no	yes	no	yes
182230863	chr1	170702779	170702821	21	43	2.095238	no	no	yes	no	no	no	no	no	no	yes
182230867	chr1	170726853	170726905	28	53	1.928571	no	yes	yes	no	no	no	no	yes	no	yes
182230871	chr1	170737126	170737182	29	57	1.965517	no	yes	no	no	no	yes	no	no	no	yes
182230874	chr1	170753227	170753283	26	57	2.307692	no	no	yes	no	no	yes	no	no	no	yes
182230878	chr1	170769899	170769937	21	39	1.904762	no	no	yes	no	no	no	no	no	yes	yes
182230885	chr1	170779122	170779148	14	27	1.928571	no	no	yes	no	no	no	no	yes	no	yes
182230888	chr1	170786256	170786296	19	41	2.157895	no	no	yes	no	no	no	no	no	no	yes
182230898	chr1	170816885	170816937	27	53	1.962963	no	no	yes	no	no	no	no	no	no	yes
182230910	chr1	170866689	170866728	21	40	1.904762	no	no	yes	no	no	no	no	no	no	yes
182230918	chr1	170895311	170895342	16	32	2.062500	no	no	yes	no	no	no	no	no	no	yes
182230921	chr1	170898236	170898292	19	57	3.000000	no	no	yes	no	no	no	no	yes	no	yes
182230931	chr1	170915282	170915348	32	67	2.125000	no	no	yes	no	no	no	no	no	no	yes
182230933	chr1	170920370	170920678	125	309	2.468750	no	no	yes	no	no	no	no	no	no	yes
182230938	chr1	170926074	170926115	18	42	2.388889	no	no	yes	no	no	no	no	no	no	yes
182230947	chr1	170953778	170953814	9	37	4.000000	no	no	yes	no	no	no	no	no	no	yes
182230956	chr1	170969351	170969401	16	51	3.250000	no	no	yes	no	no	no	no	no	no	yes
182230966	chr1	171009018	171009062	16	45	2.812500	no	no	yes	no	no	no	no	no	no	yes
182230972	chr1	171037191	171037228	19	38	2.000000	no	no	yes	no	no	no	no	no	no	yes
182230973	chr1	171037249	171037285	19	37	2.000000	no	no	yes	no	no	no	no	no	no	yes
182230992	chr1	171096683	171096796	30	114	3.903226	no	no	yes	no	no	no	no	no	no	yes
182230996	chr1	171109436	171109467	16	32	2.000000	no	no	yes	no	no	no	no	no	no	yes
182230998	chr1	171114848	171114921	37	74	2.000000	no	no	yes	no	no	no	no	no	yes	yes

182231004	chr1	171134159	171134191	11	33	3.000000	no	no	yes	no	no	no	no	no	no	yes
182231010	chr1	171166191	171166248	29	58	2.000000	no	no	yes	no	no	no	no	no	no	yes
182231015	chr1	171174986	171175052	23	67	2.875000	no	no	yes	no	no	no	no	yes	no	yes
182231017	chr1	171181746	171181892	49	147	3.020408	no	no	yes	no	no	no	no	yes	yes	yes
182231019	chr1	171189500	171189524	13	25	1.923077	no	no	yes	no	no	no	no	no	no	yes
182231021	chr1	171210718	171210967	82	250	3.048780	no	yes	yes	no	no	no	no	yes	no	yes
182231031	chr1	171231662	171231693	16	32	2.000000	no	no	yes	no	no	no	no	no	no	yes
182231032	chr1	171232877	171232907	16	31	1.937500	no	no	yes	no	no	no	no	no	no	yes
182231036	chr1	171251403	171251472	32	70	2.218750	no	no	yes	no	no	no	no	yes	yes	yes
182231039	chr1	171263209	171263267	31	59	1.935484	no	no	yes	no	no	no	no	no	no	yes
182231052	chr1	171288147	171288240	47	94	2.000000	no	no	no	no	no	no	no	yes	yes	no
182231065	chr1	171333660	171333695	18	36	2.055556	yes	no	yes	no	no	yes	no	no	no	yes
182231072	chr1	171354703	171354746	23	44	1.956522	no	no	no	no	no	no	no	no	no	no
182231106	chr1	171450825	171450963	69	139	2.014493	no	no	no	no	no	no	no	no	no	no
182231117	chr1	171461910	171461975	18	66	3.500000	no	no	no	no	no	no	no	no	no	no
182231125	chr1	171475134	171475174	22	41	1.909091	no	no	no	no	no	no	no	no	no	no
182231142	chr1	171521857	171521919	33	63	1.969697	yes	no	yes	no	no	no	no	yes	no	yes
182231157	chr1	171540656	171540692	18	37	2.055556	no	no	no	yes	no	no	no	no	no	yes
182231160	chr1	171548393	171548463	34	71	2.147059	no	no	yes	no	no	no	no	no	no	yes
182231171	chr1	171565398	171565422	13	25	1.923077	no	no	yes	no	no	no	no	yes	yes	yes
182231172	chr1	171575550	171575595	22	46	2.090909	no	no	yes	no	no	no	no	yes	no	yes
182231177	chr1	171589781	171589827	22	47	2.136364	no	no	yes	no	no	no	no	yes	no	yes
182231186	chr1	171618885	171618926	22	42	1.909091	no	no	yes	no	no	no	no	no	no	yes
182231190	chr1	171620069	171620113	13	45	3.538461	no	no	yes	no	no	no	no	no	no	yes
182231199	chr1	171648613	171648677	35	65	1.942857	no	no	yes	no	no	no	no	no	no	yes

182231200	chr1	171649022	171649066	24	45	1.916667	no	no	yes	no	no	no	no	no	no	yes
182231210	chr1	171667145	171667323	90	179	2.000000	no	no	no	no	no	no	no	no	no	no
182231217	chr1	171682525	171682581	11	57	5.083333	no	no	no	no	no	yes	no	yes	no	no
182231231	chr1	171698751	171698894	56	144	2.655172	no	no	no	no	no	no	no	no	no	no
182231233	chr1	171701049	171701092	14	44	3.000000	no	yes	no	no	no	yes	no	no	no	yes
182231242	chr1	171730773	171730828	27	56	2.074074	no	no	yes	no	no	no	no	no	no	yes
182231260	chr1	171806449	171806493	15	45	3.000000	no	no	yes	no	no	no	no	no	no	yes
182231262	chr1	171806672	171806801	67	130	1.985075	no	no	yes	no	no	no	no	no	no	yes
182231274	chr1	171834527	171834559	17	33	2.000000	no	no	no	no	no	no	no	no	no	no
182231276	chr1	171841395	171841428	16	34	2.187500	yes	no	no	no	no	no	yes	yes	yes	no
182231277	chr1	171841929	171841966	17	38	2.235294	no	no	yes	no	no	no	yes	yes	yes	yes
182231278	chr1	171843130	171843173	20	44	2.250000	no	no	yes	no	no	no	no	yes	no	yes
182231282	chr1	171870046	171870074	15	29	1.933333	no	no	yes	no	no	no	no	no	no	yes
182231286	chr1	171871630	171871954	158	325	2.063291	no	no	yes	no	no	no	no	yes	no	yes
182231287	chr1	171874303	171874336	11	34	3.000000	no	no	yes	no	no	no	no	no	no	yes
182231295	chr1	171914855	171914892	18	38	2.111111	no	no	yes	no	no	no	no	no	yes	yes
182231296	chr1	171915661	171915747	39	87	2.230769	no	no	yes	no	no	no	no	yes	yes	yes
182231298	chr1	171917857	171917904	25	48	1.920000	no	no	yes	no	no	no	no	no	no	yes
182231299	chr1	171920818	171920846	15	29	1.933333	no	no	yes	no	no	no	no	yes	yes	yes
182231317	chr1	171963719	171963775	26	57	2.192308	no	no	yes	no	no	no	no	no	no	yes
182231324	chr1	171982696	171982739	22	44	2.000000	no	no	yes	no	no	no	no	yes	no	yes
182231332	chr1	172008976	172009029	25	54	2.160000	no	no	yes	no	no	no	no	no	no	yes
182231343	chr1	172038250	172038301	25	52	2.080000	no	no	yes	no	no	no	no	no	no	yes
182231355	chr1	172073419	172073474	18	56	3.277778	no	no	yes	no	no	no	no	no	no	yes
182231356	chr1	172083882	172083924	14	43	3.142857	no	no	yes	no	no	no	no	yes	no	yes

182231358	chr1	172092621	172092655	17	35	2.058824	no	no	yes	no	no	no	no	no	no	yes
182231363	chr1	172106409	172106449	12	41	3.583333	no	no	yes	no	no	no	no	no	no	yes
182231364	chr1	172106594	172106628	17	35	2.117647	no	no	yes	no	no	no	no	no	no	yes
182231372	chr1	172138238	172138277	19	40	2.105263	yes	no	yes	no	no	yes	no	yes	yes	yes
182231378	chr1	172147775	172147832	27	58	2.148148	no	no	yes	no	no	no	no	yes	yes	yes
182231383	chr1	172161177	172161224	25	48	1.920000	no	no	yes	no	no	no	no	no	no	yes
182231391	chr1	172174041	172174083	14	43	3.142857	no	no	yes	no	no	no	no	no	no	yes
182231406	chr1	172236588	172236629	12	42	3.666667	no	no	yes	no	no	no	no	no	no	yes
182231413	chr1	172247544	172247607	33	64	1.939394	no	no	yes	no	no	no	no	no	no	yes
182231416	chr1	172249979	172250018	20	40	2.000000	no	no	yes	no	no	no	no	no	no	yes
182231417	chr1	172250297	172250328	15	32	2.133333	no	no	yes	no	no	no	no	no	no	yes
182231424	chr1	172263329	172263364	14	36	2.571429	no	no	yes	no	no	no	no	no	no	yes
182231425	chr1	172269036	172269083	23	48	2.086957	no	no	yes	no	no	no	no	yes	yes	yes
182231440	chr1	172329749	172329779	15	31	2.133333	no	no	yes	no	no	no	no	no	no	yes
182231456	chr1	172381488	172381549	30	62	2.100000	yes	no	yes	no	no	no	no	yes	no	yes
182231457	chr1	172387014	172387044	10	31	3.100000	yes	no	yes	no	no	no	no	yes	no	yes
182231460	chr1	172400412	172400487	27	76	2.814815	no	no	yes	no	no	no	no	yes	no	yes
182231463	chr1	172418438	172418479	22	42	1.909091	no	yes	yes	no	no	yes	no	yes	no	yes
182231470	chr1	172444753	172444793	21	41	1.952381	yes	no	yes	no	no	no	no	yes	yes	yes
182231471	chr1	172455310	172455350	17	41	2.588235	no	no	yes	no	no	no	no	no	no	yes
182231474	chr1	172468778	172469367	294	590	2.006803	no	yes	no	no	no	yes	no	no	no	yes
182231489	chr1	172504137	172504174	17	38	2.294118	no	no	no	no	no	no	no	no	no	no
182231492	chr1	172512983	172513032	26	50	1.961538	no	no	no	no	no	no	no	yes	yes	no
182231493	chr1	172513442	172513533	34	92	2.705882	no	no	no	no	no	no	no	yes	no	no
182231496	chr1	172519998	172520048	28	51	1.964286	no	no	no	no	no	no	no	no	no	no

182231500	chr1	172529505	172529536	16	32	2.000000	no	no	no	no	no	no	no	yes	no	no
182231511	chr1	172561744	172561786	15	43	2.866667	no	no	yes	no	no	no	no	no	no	yes
182231522	chr1	172580154	172580202	23	49	2.173913	no	no	yes	no	no	no	no	yes	no	yes
182231525	chr1	172585914	172585947	12	34	2.833333	no	no	yes	yes	no	no	no	yes	no	yes
182231529	chr1	172613080	172613132	17	53	3.055556	no	no	no	no	no	yes	no	yes	yes	no
182231533	chr1	172624429	172624479	18	51	2.888889	no	no	no	no	no	no	no	no	no	no
182231542	chr1	172624921	172624957	13	37	2.923077	no	no	no	no	no	no	no	yes	no	no
182231547	chr1	172628790	172628823	16	34	2.125000	no	no	no	no	no	no	no	no	no	no
182231554	chr1	172651247	172651287	20	41	2.050000	no	no	no	no	no	no	no	yes	yes	no
182231558	chr1	172659336	172659403	27	68	2.518518	no	no	no	yes	no	no	no	yes	yes	yes
182231565	chr1	172668685	172668772	41	88	2.146342	no	no	no	no	no	no	no	no	no	no
182231566	chr1	172672011	172672068	28	58	2.071429	no	no	no	no	no	no	no	yes	no	no
182231569	chr1	172680180	172680214	10	35	3.363636	no	no	no	no	no	no	no	no	no	no
182231570	chr1	172685435	172685524	45	90	2.000000	no	no	no	no	no	no	no	yes	no	no
182231579	chr1	172694176	172694260	40	85	2.175000	no	no	no	no	no	no	no	no	no	no
182231583	chr1	172705839	172705863	13	25	1.923077	no	no	no	no	no	no	no	no	no	no
182231584	chr1	172710602	172710632	14	31	2.214286	no	no	no	no	no	no	no	yes	no	no
182231589	chr1	172711089	172711132	17	44	2.666667	no	no	no	no	no	no	no	no	no	no
182231591	chr1	172719676	172719716	21	41	1.952381	no	no	no	no	no	no	no	yes	no	no
182231593	chr1	172721574	172721615	21	42	2.000000	no	no	no	no	no	no	no	no	no	no
182231596	chr1	172724796	172724875	42	80	1.904762	no	no	no	no	no	no	no	yes	yes	no
182231599	chr1	172732542	172732661	64	120	1.906250	no	no	no	no	no	no	no	yes	no	no
182231603	chr1	172755467	172755496	14	30	2.142857	no	no	no	no	no	no	no	yes	no	no
182231606	chr1	172772793	172772827	16	35	2.187500	no	no	no	no	no	no	no	no	no	no
182231624	chr1	172839391	172839420	16	30	1.937500	no	no	yes	no	no	no	no	no	no	yes

182231628	chr1	172841923	172841953	14	31	2.214286	no	no	yes	no	no	no	no	no	no	yes
182231641	chr1	172874565	172874601	13	37	2.846154	no	no	yes	no	no	no	no	no	no	yes
182231644	chr1	172884666	172884726	27	61	2.333333	no	no	yes	no	no	no	no	no	yes	yes
182231648	chr1	172893637	172893688	22	52	2.363636	no	no	yes	no	no	no	no	yes	yes	yes
182231653	chr1	172901714	172901741	12	28	2.333333	no	no	yes	no	no	no	no	no	no	yes
182231654	chr1	172907406	172907453	23	48	2.086957	no	yes	yes	no	no	no	no	yes	yes	yes
182231656	chr1	172909730	172909790	31	61	1.967742	no	no	yes	no	no	no	no	yes	yes	yes
182231657	chr1	172917472	172917508	12	37	3.083333	no	no	yes	no	no	no	no	no	no	yes
182231659	chr1	172917746	172917791	23	46	2.000000	no	no	yes	no	no	no	no	yes	no	yes
182231660	chr1	172921365	172921401	13	37	2.846154	no	no	yes	no	no	no	no	no	no	yes
182231670	chr1	172947220	172947272	19	53	2.750000	no	no	yes	no	no	no	no	yes	yes	yes
182231675	chr1	172969541	172969580	16	40	2.500000	no	no	yes	no	no	no	no	yes	no	yes
182231677	chr1	172971790	172971836	16	47	2.937500	no	no	yes	no	no	no	no	no	no	yes
182231685	chr1	172979284	172979316	15	33	2.200000	no	no	yes	no	no	no	no	no	no	yes
182231692	chr1	172994936	172994979	21	44	2.095238	no	no	yes	no	no	no	no	no	no	yes
182231706	chr1	173048664	173048699	19	36	1.947368	no	no	yes	no	no	no	no	yes	yes	yes
182231711	chr1	173062563	173062671	36	109	3.027778	no	no	yes	no	no	no	no	yes	yes	yes
182231718	chr1	173086879	173086910	15	32	2.133333	no	no	no	no	no	no	no	no	no	no
182231730	chr1	173120950	173120990	22	41	1.909091	no	no	no	no	no	no	no	no	no	no
182231732	chr1	173125625	173125668	15	44	3.000000	no	no	no	no	no	no	no	no	no	no
182231734	chr1	173139349	173139381	17	33	1.941176	no	no	no	no	no	no	no	yes	yes	no
182231743	chr1	173163855	173163887	12	33	2.833333	no	no	no	no	no	no	no	no	no	no
182231747	chr1	173205695	173205737	20	43	2.150000	yes	no	yes	no	yes	no	no	yes	yes	yes
182231754	chr1	173240126	173240166	16	41	2.625000	no	no	no	no	no	no	no	no	no	no
182231769	chr1	173254835	173254863	15	29	1.933333	no	no	no	no	no	no	no	yes	no	no

182231772	chr1	173270850	173270920	35	71	2.028571	no	no	no	no	no	no	no	yes	yes	no
182231775	chr1	173299135	173299163	13	29	2.230769	no	no	no	no	no	no	no	yes	yes	no
182231777	chr1	173300319	173300353	17	35	2.058824	no	no	no	no	no	no	no	yes	yes	no
182231783	chr1	173312395	173312442	26	48	1.923077	no	no	no	no	no	no	no	no	no	no
182231785	chr1	173316734	173316852	59	119	2.016949	no	no	no	no	no	no	no	no	no	no
182231796	chr1	173362529	173362570	22	42	2.000000	no	no	no	no	yes	no	no	no	no	yes
182231822	chr1	173403582	173403613	16	32	2.000000	no	no	no	no	no	no	no	no	no	no
182231831	chr1	173422212	173422236	13	25	1.923077	no	no	yes	no	no	no	no	yes	no	yes
182231833	chr1	173438705	173438742	20	38	1.900000	no	no	yes	no	no	no	no	no	no	yes
182231839	chr1	173443555	173443597	22	43	1.954545	yes	no	yes	no	no	no	no	no	no	yes
182231844	chr1	173451716	173451770	23	55	2.375000	no	no	yes	no	no	no	no	no	no	yes
182231853	chr1	173477158	173477231	22	74	3.409091	yes	no	yes	no	yes	no	yes	yes	yes	yes
182231861	chr1	173488319	173488432	57	114	2.017544	no	yes	yes	no	no	yes	no	yes	yes	yes
182231868	chr1	173526703	173526799	48	97	2.020833	no	no	yes	yes	yes	no	no	no	no	yes
182231869	chr1	173536784	173536820	16	37	2.375000	no	no	yes	no	no	no	no	yes	no	yes
182231893	chr1	173599095	173599123	15	29	1.933333	no	no	yes	no	no	no	no	yes	no	yes
182231905	chr1	173612250	173612362	16	113	6.944445	no	no	yes	no	no	no	no	no	no	yes
182231919	chr1	173636748	173636785	18	38	2.166667	no	no	yes	no	no	no	no	yes	no	yes
182231920	chr1	173644329	173644370	22	42	1.909091	no	no	yes	no	no	no	no	no	no	yes
182231921	chr1	173646563	173646606	18	44	2.500000	no	no	yes	no	no	no	no	no	no	yes
182231927	chr1	173663466	173663528	28	63	2.250000	no	no	yes	no	no	no	no	yes	yes	yes
182231942	chr1	173693373	173693406	16	34	2.125000	no	no	no	no	no	no	no	no	no	no
182231944	chr1	173702664	173702704	11	41	4.000000	no	no	no	no	no	no	no	yes	no	no
182231948	chr1	173711980	173712022	21	43	2.095238	no	no	no	no	no	no	no	no	no	no
182231954	chr1	173742601	173742684	30	84	2.800000	no	no	yes	no	no	yes	no	yes	no	yes

182231962	chr1	173754192	173754228	19	37	1.947368	no	no	yes	no	no	no	no	yes	no	yes
182231963	chr1	173758305	173758364	30	60	2.000000	no	no	yes	no	no	no	no	yes	no	yes
182231973	chr1	173780996	173781034	14	39	2.785714	yes	no	yes	no	no	no	no	no	yes	yes
182231979	chr1	173785075	173785116	21	42	2.000000	no	no	yes	no	no	no	no	no	no	yes
182231997	chr1	173826937	173827001	34	65	1.911765	no	yes	yes	no	no	no	no	no	no	yes
182232000	chr1	173832195	173832221	14	27	1.928571	no	no	yes	no	no	no	no	no	no	yes
182232003	chr1	173832218	173832319	49	102	2.081633	no	no	yes	no	no	no	no	no	no	yes
182232005	chr1	173834719	173834794	38	76	2.078947	no	no	yes	no	no	no	no	no	no	yes
182232006	chr1	173837077	173837119	20	43	2.200000	no	no	yes	no	no	no	no	no	no	yes
182232014	chr1	173854374	173854414	19	41	2.157895	no	yes	yes	no	no	yes	no	no	no	yes
182232016	chr1	173865683	173865755	35	73	2.085714	yes	no	yes	no	yes	yes	no	yes	yes	yes
182232019	chr1	173875187	173875243	25	57	2.320000	no	no	yes	no	no	no	no	no	no	yes
182232022	chr1	173891044	173891090	22	47	2.181818	no	yes	no	no	no	no	no	no	no	yes
182232025	chr1	173911260	173911308	18	49	2.722222	no	no	yes	no	no	yes	no	no	no	yes
182232033	chr1	173931773	173931817	23	45	1.956522	no	yes	no	no	no	no	no	no	yes	yes
182232048	chr1	173952149	173952195	15	47	3.133333	no	no	yes	no	no	no	no	yes	yes	yes
182232055	chr1	173956770	173956806	19	37	1.947368	no	no	yes	no	no	no	no	no	no	yes
182232062	chr1	173980589	173980628	17	40	2.411765	no	no	yes	no	no	yes	no	no	no	yes
182232081	chr1	174022136	174022175	18	40	2.222222	yes	no	no	no	yes	no	yes	yes	no	yes
182232086	chr1	174026753	174026804	23	52	2.260870	no	no	no	no	no	no	no	yes	no	no
182232101	chr1	174044196	174044251	24	56	2.333333	no	no	no	no	no	no	no	no	no	no
182232102	chr1	174048933	174048975	22	43	1.954545	no	no	no	no	no	no	no	no	no	no
182232139	chr1	174111118	174111143	13	26	2.000000	no	no	no	no	yes	no	no	no	no	yes
182232145	chr1	174134439	174134561	37	123	3.219512	no	no	yes	no	no	no	no	yes	no	yes
182232146	chr1	174136116	174136504	23	389	17.625000	no	no	yes	no	no	no	no	no	no	yes

182232156	chr1	174152279	174152313	17	35	2.058824	no	no	yes	no	no	no	no	yes	no	yes
182232162	chr1	174168314	174168347	15	34	2.266667	no	no	yes	no	no	no	no	no	no	yes
182232173	chr1	174198600	174198670	37	71	1.972973	no	no	yes	no	no	no	no	yes	no	yes
182232178	chr1	174219054	174219098	17	45	2.764706	no	no	yes	no	no	no	no	yes	yes	yes
182232190	chr1	174253722	174253757	16	36	2.250000	no	no	yes	no	no	no	no	no	no	yes
182232194	chr1	174260743	174260796	28	54	1.928571	no	no	yes	no	no	no	no	no	no	yes
182232195	chr1	174265742	174265773	17	32	1.941176	no	no	yes	no	no	no	no	no	no	yes
182232197	chr1	174271109	174271150	22	42	1.909091	no	no	yes	no	no	no	no	no	no	yes
182232198	chr1	174272524	174272557	18	34	1.944444	no	no	yes	no	no	no	no	no	no	yes
182232201	chr1	174277858	174277891	15	34	2.266667	no	no	yes	no	no	no	no	no	no	yes
182232203	chr1	174280716	174280764	20	49	2.450000	no	no	yes	no	no	no	no	no	no	yes
182232219	chr1	174305727	174305760	14	34	2.428571	no	no	yes	no	no	no	no	no	no	yes
182232232	chr1	174342163	174342209	23	47	2.043478	no	no	yes	no	no	no	no	no	no	yes
182232261	chr1	174371579	174371628	27	50	1.925926	no	no	yes	no	no	no	no	no	no	yes
182232272	chr1	174399499	174399533	15	35	2.333333	no	no	yes	no	no	no	no	yes	no	yes
182232277	chr1	174414449	174414487	20	39	1.950000	no	no	yes	no	no	no	no	yes	yes	yes
182232278	chr1	174418610	174418649	19	40	2.105263	no	no	yes	no	no	no	no	no	no	yes
182232305	chr1	174499170	174499206	17	37	2.176471	no	no	yes	no	no	no	no	no	no	yes
182232306	chr1	174499231	174499272	15	42	2.800000	no	no	yes	no	no	no	no	no	no	yes
182232312	chr1	174517816	174517867	25	52	2.120000	no	no	yes	no	no	no	no	no	no	yes
182232324	chr1	174536661	174536696	14	36	2.642857	no	no	yes	no	no	no	no	no	no	yes
182232331	chr1	174566305	174566344	19	40	2.157895	no	no	yes	no	no	no	no	no	no	yes
182232333	chr1	174568691	174568728	16	38	2.375000	no	no	yes	no	no	no	no	no	yes	yes
182232337	chr1	174573192	174573304	50	113	2.260000	no	no	yes	no	no	no	no	no	no	yes
182232353	chr1	174608966	174609081	56	116	2.071429	no	no	yes	no	no	no	no	no	no	yes

182232359	chr1	174640288	174640340	25	53	2.120000	no	no	yes	no	no	no	no	no	yes	yes
182232360	chr1	174640465	174640522	18	58	3.157895	no	no	yes	no	no	no	no	yes	no	yes
182232361	chr1	174640514	174640539	13	26	2.000000	no	no	yes	no	no	no	no	yes	no	yes
182232362	chr1	174640543	174640573	15	31	2.066667	no	no	yes	no	no	no	no	yes	yes	yes
182232364	chr1	174641433	174641504	35	72	2.114286	no	no	yes	no	no	no	no	no	no	yes
182232366	chr1	174642675	174642723	19	49	2.550000	no	no	yes	no	no	no	no	yes	no	yes
182232367	chr1	174642728	174642782	16	55	3.437500	no	no	yes	no	no	no	no	yes	no	yes
182232375	chr1	174665337	174665410	22	74	3.291667	no	no	yes	no	no	no	no	no	no	yes
182232384	chr1	174690535	174690578	22	44	2.000000	no	no	yes	no	no	no	no	yes	no	yes
182232393	chr1	174718111	174718159	24	49	2.041667	no	no	yes	no	no	no	no	no	no	yes
182232399	chr1	174739194	174739227	18	34	1.944444	no	no	yes	no	no	no	no	no	no	yes
182232405	chr1	174742053	174742227	40	175	4.341464	no	no	yes	no	no	no	no	no	yes	yes
182232422	chr1	174772920	174772996	40	77	1.925000	no	no	yes	no	no	no	no	yes	no	yes
182232436	chr1	174799933	174799996	10	64	6.400000	no	no	yes	no	yes	no	no	yes	no	yes
182232439	chr1	174807398	174807451	27	54	2.037037	no	no	yes	no	no	no	no	no	yes	yes
182232443	chr1	174813530	174813555	13	26	2.000000	no	no	yes	no	no	no	no	no	no	yes
182232448	chr1	174829711	174829743	17	33	1.941176	no	no	yes	no	no	no	no	no	no	yes
182232465	chr1	174871489	174871522	13	34	2.615385	no	no	yes	no	no	no	no	no	yes	yes
182232467	chr1	174874203	174874242	19	40	2.105263	yes	no	yes	no	no	no	no	yes	yes	yes
182232472	chr1	174879771	174879810	20	40	2.000000	no	no	yes	no	no	no	no	no	no	yes
182232474	chr1	174880556	174880603	20	48	2.400000	no	no	yes	no	no	no	no	yes	no	yes
182232476	chr1	174885240	174885276	17	37	2.176471	no	no	yes	no	no	no	no	no	no	yes
182232477	chr1	174889089	174889126	19	38	2.000000	no	no	yes	no	no	no	no	no	no	yes
182232493	chr1	174925849	174925907	31	59	1.903226	no	no	yes	no	no	no	no	yes	no	yes
182232502	chr1	174952142	174952209	25	68	2.720000	no	no	yes	no	no	no	no	yes	no	yes

182232506	chr1	174971571	174971600	14	30	2.142857	no	no	yes	no	no	no	no	yes	yes	yes
182232509	chr1	174972887	174972957	36	71	1.972222	no	no	yes	no	no	no	no	no	no	yes
182232514	chr1	174974773	174974937	81	165	2.049383	no	no	yes	no	no	no	no	yes	yes	yes
182232515	chr1	174976035	174976062	13	28	2.153846	no	yes	yes	no	no	no	no	yes	yes	yes
182232517	chr1	174979335	174979381	14	47	3.357143	no	no	yes	no	no	no	no	no	no	yes
182232520	chr1	174998920	174999028	55	109	1.981818	yes	no	no	no	yes	no	no	yes	yes	yes
182232521	chr1	175004700	175004747	25	48	2.000000	no	yes	no	yes	yes	no	no	no	no	yes
182232540	chr1	175053315	175053362	18	48	2.631579	no	no	no	no	no	no	no	no	no	no
182232549	chr1	175078379	175078427	24	49	2.125000	no	no	yes	no	no	no	no	yes	no	yes
182232582	chr1	175118956	175119003	17	48	2.789474	no	no	yes	no	no	no	no	no	no	yes
182232584	chr1	175124836	175124872	16	37	2.375000	no	no	yes	no	no	no	no	no	no	yes
182232592	chr1	175143501	175143532	16	32	2.000000	no	no	yes	no	no	no	no	no	no	yes
182232596	chr1	175149552	175149655	40	104	2.650000	no	no	no	no	no	no	no	no	no	no
182232599	chr1	175153483	175154165	136	683	4.992857	no	no	no	no	no	no	no	yes	yes	no
182232603	chr1	175157720	175157750	15	31	2.066667	no	yes	no	no	no	no	no	no	no	yes
182232611	chr1	175194213	175194245	8	33	4.250000	no	no	no	no	no	no	no	yes	yes	no
182232612	chr1	175196519	175196557	21	39	1.904762	no	no	no	no	no	no	no	no	no	no
182232614	chr1	175209054	175209092	19	39	2.052632	no	no	no	no	no	no	no	yes	yes	no
182232615	chr1	175217203	175217260	29	58	2.000000	no	no	no	no	no	no	no	no	no	no
182232634	chr1	175250902	175250937	14	36	2.571429	no	no	no	no	no	no	no	no	no	no
182232635	chr1	175254782	175254819	18	38	2.111111	no	no	no	no	no	no	no	no	no	no
182232646	chr1	175287949	175287993	22	45	2.045455	no	no	no	no	no	no	no	no	yes	no
182232649	chr1	175297577	175297621	22	45	2.045455	no	no	no	no	no	yes	no	no	yes	no
182232653	chr1	175311488	175311526	21	39	1.904762	no	no	yes	no	no	no	no	no	yes	yes
182232661	chr1	175314872	175314951	26	80	3.076923	no	no	yes	no	no	yes	no	yes	no	yes

182232671	chr1	175340872	175340912	16	41	2.562500	no	no	yes	no	no	no	no	no	no	yes
182232679	chr1	175372330	175372379	25	50	2.040000	no	no	yes	no	no	no	no	no	yes	yes
182232680	chr1	175374372	175374440	31	69	2.225806	no	no	yes	no	no	no	no	yes	yes	yes
182232681	chr1	175374434	175374490	28	57	2.107143	no	no	yes	no	no	no	no	yes	yes	yes
182232686	chr1	175387468	175387506	18	39	2.277778	no	no	yes	no	no	no	no	no	no	yes
182232688	chr1	175394712	175394759	25	48	1.920000	no	no	yes	no	no	no	no	no	no	yes
182232691	chr1	175406099	175406131	17	33	2.000000	no	no	yes	no	no	no	no	no	no	yes
182232693	chr1	175415834	175415860	13	27	2.076923	no	no	yes	no	no	no	no	no	no	yes
182232696	chr1	175426735	175426802	26	68	2.615385	no	no	yes	no	no	no	no	no	no	yes
182232698	chr1	175426796	175426992	35	197	5.500000	no	no	yes	no	no	no	no	no	no	yes
182232709	chr1	175446910	175446950	20	41	2.150000	no	no	yes	no	no	no	no	yes	no	yes
182232710	chr1	175449274	175449324	22	51	2.318182	no	no	yes	no	no	no	no	yes	no	yes
182232711	chr1	175454086	175454138	26	53	2.038461	no	no	yes	no	no	no	no	no	no	yes
182232712	chr1	175458128	175458192	31	65	2.096774	no	no	yes	no	no	no	no	yes	no	yes
182232714	chr1	175459129	175459157	15	29	1.933333	no	no	yes	no	no	no	no	yes	no	yes
182232716	chr1	175459176	175459251	38	76	2.000000	no	no	yes	no	no	no	no	yes	no	yes
182232719	chr1	175460869	175460905	16	37	2.312500	no	no	yes	no	no	no	no	no	yes	yes
182232721	chr1	175460946	175460997	22	52	2.363636	no	no	yes	no	no	no	no	no	yes	yes
182232722	chr1	175465851	175465883	16	33	2.062500	no	no	yes	no	no	no	no	no	no	yes
182232728	chr1	175484444	175484488	20	45	2.250000	no	no	yes	no	no	no	no	yes	no	yes
182232740	chr1	175515154	175515193	20	40	2.050000	no	no	yes	no	no	no	no	no	no	yes
182232742	chr1	175518681	175518742	33	62	1.909091	no	no	yes	no	no	no	no	no	no	yes
182232744	chr1	175528542	175528588	24	47	2.041667	no	no	yes	no	no	no	no	no	no	yes
182232746	chr1	175543456	175543493	17	38	2.235294	no	no	yes	no	no	no	no	yes	yes	yes
182232747	chr1	175547001	175547031	12	31	2.583333	no	no	yes	no	no	no	no	no	no	yes

182232758	chr1	175585755	175585833	40	79	2.000000	no	no	yes	no	no	no	no	yes	no	yes
182232761	chr1	175599147	175599187	19	41	2.210526	no	no	yes	no	no	no	no	yes	no	yes
182232763	chr1	175626650	175626685	17	36	2.117647	no	no	yes	no	no	no	no	no	no	yes
182232764	chr1	175629217	175629279	29	63	2.172414	no	no	yes	no	no	no	no	yes	no	yes
182232767	chr1	175635845	175635889	24	45	1.916667	no	no	yes	no	no	no	no	no	no	yes
182232768	chr1	175636403	175636468	29	66	2.310345	no	no	yes	no	no	no	no	yes	no	yes
182232771	chr1	175650718	175651229	21	512	24.115385	no	no	yes	no	no	no	no	no	no	yes
182232778	chr1	175661720	175661762	17	43	2.611111	no	no	yes	no	no	no	no	yes	no	yes
182232792	chr1	175746878	175746918	20	41	2.050000	no	no	no	no	no	no	no	no	no	no
182232794	chr1	175748114	175748162	18	49	2.722222	no	no	no	no	no	no	no	no	no	no
182232817	chr1	175819933	175819970	20	38	1.900000	no	no	no	no	no	no	no	no	no	no
182232821	chr1	175840531	175840560	14	30	2.142857	no	no	no	no	no	no	no	yes	yes	no
182232825	chr1	175862723	175862771	21	49	2.333333	no	no	no	no	no	no	no	no	no	no
182232827	chr1	175871097	175871138	22	42	2.000000	no	no	no	no	no	no	no	no	no	no
182232830	chr1	175873055	175873091	17	37	2.235294	no	no	no	no	no	no	no	yes	no	no
182232831	chr1	175877759	175877787	7	29	4.142857	no	no	yes	no	no	no	no	no	no	yes
182232832	chr1	175878762	175878813	27	52	1.925926	no	no	yes	no	no	no	no	yes	yes	yes
182232833	chr1	175879121	175879149	14	29	2.142857	no	yes	no	no	no	no	no	yes	no	yes
182232834	chr1	175884503	175884539	17	37	2.176471	no	no	no	no	no	no	no	no	yes	no
182232839	chr1	175906279	175906342	26	64	2.461539	no	no	yes	no	no	no	no	no	no	yes
182232851	chr1	175946819	175946857	19	39	2.052632	no	no	yes	no	no	no	no	no	no	yes
182232863	chr1	175974258	175974296	19	39	2.105263	no	no	yes	no	no	no	no	no	no	yes
182232875	chr1	176018291	176018336	21	46	2.238095	no	no	yes	no	no	no	no	yes	no	yes
182232881	chr1	176028553	176028591	18	39	2.222222	no	no	yes	no	no	no	no	yes	no	yes
182232899	chr1	176060454	176060499	25	46	1.920000	no	no	yes	no	no	no	no	no	no	yes

182232915	chr1	176110642	176110687	16	46	2.937500	no	no	yes	no	no	no	no	no	no	yes
182232916	chr1	176117435	176117464	14	30	2.142857	no	no	yes	no	no	no	no	yes	no	yes
182232920	chr1	176121218	176121250	17	33	2.000000	no	no	yes	no	no	no	no	no	no	yes
182232924	chr1	176132539	176133296	39	758	19.466667	no	no	yes	no	no	no	no	no	yes	yes
182232926	chr1	176138565	176138592	14	28	2.000000	no	no	yes	no	no	no	no	no	yes	yes
182232927	chr1	176138677	176138714	19	38	2.000000	no	no	yes	no	no	no	no	no	no	yes
182232944	chr1	176168783	176168823	22	41	1.954545	no	no	yes	no	no	no	no	no	no	yes
182232947	chr1	176177767	176177802	17	36	2.117647	no	no	yes	no	no	no	no	yes	yes	yes
182232960	chr1	176195092	176195159	17	68	4.055555	no	no	yes	no	no	no	no	no	no	yes
182232968	chr1	176209217	176209401	31	185	5.937500	no	no	yes	no	no	no	no	no	no	yes
182232997	chr1	176276297	176276325	15	29	1.933333	no	no	yes	no	no	no	no	no	no	yes
182233007	chr1	176300883	176301022	58	140	2.406780	no	no	yes	no	no	no	no	no	no	yes
182233013	chr1	176306260	176306295	15	36	2.466667	yes	no	yes	no	no	no	no	no	no	yes
182233021	chr1	176330153	176330228	40	76	1.900000	no	no	yes	no	no	no	no	no	no	yes
182233030	chr1	176363639	176363677	20	39	1.950000	no	no	yes	no	no	no	no	no	no	yes
182233066	chr1	176439281	176439333	27	53	1.962963	no	no	yes	no	no	no	no	no	no	yes
182233081	chr1	176500475	176500545	24	71	2.923077	no	no	yes	no	no	no	no	no	no	yes
182233085	chr1	176501051	176501114	34	64	2.000000	no	no	yes	no	no	no	no	no	no	yes
182233094	chr1	176513336	176513368	11	33	3.000000	no	no	yes	no	no	no	no	no	no	yes
182233096	chr1	176525381	176525424	10	44	4.400000	no	no	yes	no	no	no	no	no	no	yes
182233098	chr1	176528103	176528179	31	77	2.484848	no	no	yes	no	no	no	no	no	no	yes
182233122	chr1	176602138	176602185	24	48	2.000000	no	no	yes	no	no	no	no	no	no	yes
182233124	chr1	176607038	176607077	15	40	2.666667	no	no	yes	no	no	no	no	no	no	yes
182233127	chr1	176610189	176610280	36	92	2.555556	no	no	yes	no	no	no	no	yes	no	yes
182233130	chr1	176629427	176629491	33	65	1.969697	no	no	yes	no	no	no	no	no	no	yes

182233140	chr1	176642223	176642280	28	58	2.071429	no	no	yes	no	no	no	no	no	no	yes
182233158	chr1	176677677	176677714	16	38	2.375000	no	no	yes	no	no	no	no	no	no	yes
182233161	chr1	176677760	176677820	30	61	2.066667	no	no	yes	no	no	no	no	no	no	yes
182233167	chr1	176696269	176696377	53	109	2.056604	no	no	yes	no	no	no	no	yes	yes	yes
182233180	chr1	176731636	176731737	32	102	3.111111	no	no	yes	no	no	no	no	no	no	yes
182233183	chr1	176736389	176736436	26	48	1.923077	no	no	yes	no	no	no	no	yes	no	yes
182233186	chr1	176736476	176736538	33	63	1.939394	no	no	yes	no	no	no	no	no	no	yes
182233191	chr1	176754456	176754591	67	136	2.029851	no	no	yes	no	no	no	no	no	no	yes
182233192	chr1	176759877	176759913	18	37	2.055556	no	no	yes	no	no	no	no	no	no	yes
182233195	chr1	176766325	176766383	23	59	2.600000	no	no	yes	no	no	no	no	no	no	yes
182233197	chr1	176772111	176772203	38	93	2.447368	no	no	yes	no	no	no	no	no	no	yes
182233201	chr1	176777819	176777861	18	43	2.388889	no	no	yes	no	no	no	no	yes	no	yes
182233209	chr1	176794749	176794796	24	48	2.000000	no	no	yes	no	no	no	no	yes	no	yes
182233214	chr1	176800967	176801028	31	62	2.000000	no	no	yes	no	no	no	no	yes	no	yes
182233220	chr1	176820802	176820839	18	38	2.166667	no	no	yes	no	no	no	no	no	yes	yes
182233227	chr1	176860605	176860630	13	26	2.000000	no	no	yes	no	no	yes	no	no	no	yes
182233230	chr1	176865584	176865636	27	53	1.962963	no	no	yes	no	no	no	no	no	no	yes
182233243	chr1	176922884	176922917	18	34	1.944444	no	no	yes	no	no	no	no	no	no	yes
182233244	chr1	176927789	176927821	17	33	2.000000	no	no	yes	no	no	no	no	no	no	yes
182233247	chr1	176935748	176935806	22	59	2.681818	no	no	yes	no	no	yes	no	no	no	yes
182233254	chr1	176952230	176952263	17	34	2.000000	no	no	yes	no	no	no	no	no	no	yes
182233257	chr1	176967843	176968015	41	173	4.219512	no	no	yes	no	no	no	no	no	no	yes
182233259	chr1	176970693	176970729	16	37	2.312500	no	no	yes	no	no	no	no	no	no	yes
182233264	chr1	176990521	176990562	20	42	2.100000	no	no	yes	no	no	no	no	no	no	yes
182233274	chr1	176997510	176997536	13	27	2.076923	no	no	yes	no	no	no	no	no	yes	yes

182233282	chr1	177010237	177010265	14	29	2.071429	no	no	yes	no	no	no	no	no	no	yes
182233284	chr1	177011649	177011726	32	78	2.470588	no	no	yes	no	no	no	no	yes	no	yes
182233290	chr1	177031187	177031225	15	39	2.562500	no	no	yes	no	no	no	no	no	no	yes
182233293	chr1	177035410	177035438	15	29	1.933333	no	no	yes	no	no	no	no	no	no	yes
182233301	chr1	177047342	177047385	22	44	2.000000	no	no	yes	no	no	no	no	no	no	yes
182233305	chr1	177058810	177058855	14	46	3.285714	no	no	yes	no	no	no	no	no	no	yes
182233306	chr1	177059680	177059712	13	33	2.615385	no	no	yes	no	no	no	no	no	no	yes
182233313	chr1	177082103	177082143	21	41	1.952381	no	no	yes	no	no	no	no	no	no	yes
182233319	chr1	177145663	177145696	16	34	2.125000	no	no	yes	no	no	no	no	yes	yes	yes
182233329	chr1	177164720	177164761	12	42	3.583333	yes	no	no	no	yes	no	no	yes	yes	yes
182233334	chr1	177179568	177179618	20	51	2.550000	no	no	yes	no	no	no	no	no	no	yes
182233335	chr1	177182112	177182164	23	53	2.304348	no	no	yes	no	no	no	no	yes	no	yes
182233344	chr1	177218932	177218973	21	42	2.000000	no	no	yes	no	no	no	no	no	no	yes
182233347	chr1	177242068	177242092	13	25	1.923077	no	no	yes	no	no	no	no	no	no	yes
182233377	chr1	177326650	177326686	19	37	1.947368	no	no	no	no	no	no	no	no	no	no
182233390	chr1	177354934	177354969	17	36	2.117647	no	no	yes	no	no	no	no	no	no	yes
182233401	chr1	177397229	177397271	22	43	2.000000	no	no	yes	no	no	no	no	no	no	yes
182233405	chr1	177408174	177408204	16	31	1.937500	no	no	yes	no	no	no	no	no	no	yes
182233413	chr1	177415555	177415653	27	99	3.666667	no	no	yes	no	no	no	no	no	no	yes
182233415	chr1	177422899	177422931	8	33	4.125000	no	no	yes	no	no	no	no	no	no	yes
182233420	chr1	177435179	177435207	15	29	2.000000	no	no	yes	no	no	no	no	no	no	yes
182233421	chr1	177435375	177435410	18	36	2.000000	no	no	yes	no	no	no	no	no	no	yes
182233441	chr1	177501222	177501293	26	72	2.769231	no	no	yes	no	no	no	no	no	no	yes
182233444	chr1	177501827	177501882	24	56	2.375000	no	no	yes	no	no	no	no	no	no	yes
182233451	chr1	177518926	177518980	27	55	2.074074	no	no	yes	no	no	no	no	no	no	yes

182233453	chr1	177525502	177525559	26	58	2.269231	no	no	yes	no	no	no	no	yes	no	yes
182233456	chr1	177527470	177527506	20	37	1.900000	no	no	yes	no	no	no	no	no	no	yes
182233457	chr1	177527542	177527579	19	38	2.000000	no	no	yes	no	no	no	no	no	no	yes
182233472	chr1	177569212	177569293	40	82	2.050000	yes	no	yes	no	no	no	no	yes	no	yes
182233474	chr1	177570527	177570558	16	32	2.062500	no	no	yes	no	no	no	no	no	no	yes
182233475	chr1	177572606	177572637	15	32	2.200000	yes	no	yes	no	no	no	no	no	no	yes
182233476	chr1	177572957	177572996	20	40	2.100000	no	no	yes	no	no	no	no	no	no	yes
182233477	chr1	177575098	177575129	8	32	4.000000	no	no	yes	no	no	no	no	no	no	yes
182233513	chr1	177734432	177734483	25	52	2.080000	no	no	yes	no	no	no	no	yes	no	yes
182233516	chr1	177747456	177747504	25	49	1.960000	no	no	yes	no	no	no	no	no	no	yes
182233520	chr1	177756563	177756589	14	27	1.928571	no	no	yes	no	no	no	no	yes	no	yes
182233523	chr1	177772049	177772094	23	46	2.043478	no	no	yes	no	no	no	no	no	no	yes
182233525	chr1	177779141	177779202	26	62	2.384615	no	no	yes	no	no	no	no	yes	no	yes
182233527	chr1	177809457	177809498	22	42	1.909091	no	no	yes	no	no	no	no	no	no	yes
182233529	chr1	177820219	177820316	26	98	3.807692	no	no	yes	no	no	no	no	yes	yes	yes
182233533	chr1	177827960	177828066	55	107	1.945455	no	no	yes	no	no	no	no	no	no	yes
182233536	chr1	177836260	177836298	19	39	2.052632	no	no	yes	no	no	no	no	no	no	yes
182233537	chr1	177853489	177853548	30	60	2.000000	no	no	yes	no	no	no	no	yes	no	yes
182233540	chr1	177855275	177855308	17	34	2.000000	no	no	yes	no	no	no	no	yes	no	yes
182233552	chr1	177902513	177902542	14	30	2.214286	no	no	yes	no	no	no	no	no	yes	yes
182233560	chr1	177926879	177926904	13	26	2.000000	no	no	yes	no	no	yes	no	yes	yes	yes
182233561	chr1	177933430	177933462	16	33	2.187500	no	no	yes	no	no	no	no	no	no	yes
182233562	chr1	177940145	177940169	12	25	2.083333	no	no	yes	no	no	no	no	no	no	yes
182233564	chr1	177947693	177947803	25	111	4.480000	no	no	yes	no	no	no	no	yes	yes	yes
182233573	chr1	177966315	177966367	26	53	2.038461	no	no	yes	no	no	no	no	yes	yes	yes

182233576	chr1	177978787	177978844	26	58	2.230769	no	no	yes	no	no	no	no	yes	no	yes
182233580	chr1	177998670	177998775	50	106	2.120000	no	no	yes	no	no	no	no	no	no	yes
182233581	chr1	177999158	177999187	15	30	2.000000	no	no	yes	no	no	no	no	no	yes	yes
182233582	chr1	178009882	178009924	21	43	2.047619	no	no	yes	no	no	no	no	no	no	yes
182233583	chr1	178011463	178011488	13	26	2.000000	no	no	yes	no	no	no	no	yes	no	yes
182233587	chr1	178028762	178028804	10	43	4.400000	no	no	yes	no	no	no	no	no	no	yes
182233604	chr1	178071989	178072027	14	39	2.785714	no	no	no	no	no	no	no	no	yes	no
182233610	chr1	178092021	178092054	17	34	2.000000	no	no	no	no	yes	no	no	yes	yes	yes
182233613	chr1	178093768	178093825	18	58	3.222222	yes	no	no	no	yes	no	no	yes	yes	yes
182233614	chr1	178096943	178096973	14	31	2.214286	no	no	yes	no	no	no	no	no	no	yes
182233632	chr1	178143146	178143180	17	35	2.117647	no	no	yes	no	no	no	no	no	no	yes
182233634	chr1	178143931	178143959	14	29	2.071429	no	no	yes	no	no	no	no	yes	yes	yes
182233644	chr1	178165188	178165267	30	80	2.666667	no	no	yes	no	no	no	no	yes	no	yes
182233645	chr1	178166326	178166362	17	37	2.176471	no	no	yes	no	no	no	no	yes	no	yes
182233648	chr1	178176639	178176670	8	32	4.000000	no	no	yes	no	no	no	no	no	no	yes
182233649	chr1	178176967	178176999	14	33	2.357143	no	no	yes	no	no	no	no	no	no	yes
182233655	chr1	178193563	178193602	20	40	2.100000	no	no	yes	no	no	yes	no	no	no	yes
182233659	chr1	178195434	178195476	22	43	1.954545	yes	no	yes	no	no	no	no	yes	no	yes
182233667	chr1	178225631	178225688	31	58	1.903226	no	no	yes	no	no	no	no	no	no	yes
182233682	chr1	178278976	178279028	27	53	1.962963	no	no	yes	no	no	no	no	no	yes	yes
182233684	chr1	178281258	178281288	15	31	2.066667	no	no	yes	no	no	no	no	no	yes	yes
182233689	chr1	178291494	178291522	13	29	2.307692	no	no	yes	no	no	no	no	yes	no	yes
182233692	chr1	178296029	178296170	26	142	5.461538	no	no	yes	no	no	no	no	no	no	yes
182233695	chr1	178298915	178298947	17	33	1.941176	no	no	yes	no	no	no	no	no	no	yes
182233696	chr1	178301078	178301123	20	46	2.285714	no	no	yes	no	no	no	no	no	no	yes

182233699	chr1	178321386	178321414	12	29	2.500000	no	no	yes	no	no	no	no	no	no	yes
182233702	chr1	178333717	178333751	16	35	2.187500	no	no	yes	no	no	no	no	no	no	yes
182233712	chr1	178381746	178381855	57	110	1.929825	no	no	yes	no	no	no	no	no	no	yes
182233713	chr1	178381919	178381975	28	57	2.035714	no	no	yes	no	no	no	no	no	no	yes
182233714	chr1	178383680	178383723	18	44	2.444444	no	no	yes	no	no	no	no	no	no	yes
182233715	chr1	178391651	178391795	72	145	2.013889	no	no	yes	no	no	no	no	no	no	yes
182233722	chr1	178412946	178413040	32	95	2.969697	no	no	yes	no	no	yes	no	yes	no	yes
182233726	chr1	178418050	178418078	13	29	2.230769	no	no	yes	no	no	no	no	yes	yes	yes
182233729	chr1	178428561	178428599	13	39	3.000000	no	no	yes	no	no	no	no	no	no	yes
182233736	chr1	178442445	178442488	23	44	1.913043	no	no	yes	no	no	no	no	no	no	yes
182233737	chr1	178444682	178444714	16	33	2.062500	no	no	yes	no	no	no	no	no	no	yes
182233739	chr1	178448526	178448564	20	39	1.950000	no	no	yes	no	no	no	no	no	no	yes
182233740	chr1	178449862	178449898	19	37	1.947368	no	no	yes	no	no	no	no	no	no	yes
182233741	chr1	178459327	178459390	33	64	1.969697	no	no	yes	no	no	no	no	no	no	yes
182233752	chr1	178491137	178491180	23	44	1.913043	no	no	yes	no	no	no	no	no	yes	yes
182233755	chr1	178496939	178496982	11	44	4.000000	yes	no	yes	no	yes	no	no	no	no	yes
182233759	chr1	178505574	178506010	16	437	27.312500	no	no	no	no	no	no	no	no	no	no
182233762	chr1	178540318	178540354	16	37	2.312500	no	no	yes	no	no	no	no	yes	yes	yes
182233763	chr1	178546541	178546576	14	36	2.642857	no	no	yes	no	no	no	no	no	no	yes
182233769	chr1	178570849	178570904	26	56	2.192308	no	no	no	no	no	no	no	no	no	no
182233801	chr1	178653669	178653701	14	33	2.357143	no	no	no	no	no	no	no	yes	no	no
182233818	chr1	178686600	178686646	21	47	2.285714	no	no	no	no	no	no	no	yes	yes	no
182233819	chr1	178688141	178688182	21	42	2.000000	no	no	no	no	no	no	no	no	no	no
182233847	chr1	178726888	178726916	14	29	2.071429	yes	no	yes	no	no	no	no	yes	yes	yes
182233853	chr1	178737034	178737063	16	30	1.937500	no	no	yes	no	no	no	no	no	no	yes

182233859	chr1	178780941	178780974	13	34	2.692308	no	no	yes	no	no	no	no	no	no	yes
182233862	chr1	178788775	178788924	47	150	3.137255	no	no	yes	no	no	no	no	no	no	yes
182233863	chr1	178790592	178790645	28	54	1.928571	no	no	yes	no	no	no	no	no	no	yes
182233874	chr1	178855800	178855832	17	33	1.941176	no	no	yes	no	no	no	no	no	no	yes
182233876	chr1	178855979	178856015	19	37	1.947368	no	no	yes	no	no	no	no	no	no	yes
182233883	chr1	178875933	178875971	19	39	2.105263	no	no	yes	no	no	no	no	no	no	yes
182233899	chr1	178931566	178931601	19	36	1.947368	no	no	no	no	no	no	no	no	yes	no
182233902	chr1	178942659	178942704	18	46	2.555556	no	no	no	no	no	no	no	yes	yes	no
182233905	chr1	178959071	178959103	16	33	2.062500	no	no	no	no	no	no	no	yes	no	no
182233926	chr1	179012735	179012763	15	29	1.933333	no	no	no	no	no	no	no	no	no	no
182233932	chr1	179028109	179028162	27	54	2.000000	no	no	yes	no	no	no	no	yes	yes	yes
182233953	chr1	179052156	179052217	31	62	2.000000	no	no	yes	no	no	no	no	yes	no	yes
182233956	chr1	179055546	179055578	13	33	2.615385	no	no	yes	no	no	no	no	no	no	yes
182233963	chr1	179071385	179071414	14	30	2.142857	no	no	yes	no	no	no	no	yes	no	yes
182233974	chr1	179120916	179120956	20	41	2.050000	no	no	yes	no	no	no	no	yes	yes	yes
182233976	chr1	179122590	179122646	16	57	3.529412	no	no	yes	no	no	no	no	no	no	yes
182233979	chr1	179134424	179134459	11	36	3.272727	yes	no	yes	no	no	no	no	no	no	yes
182233999	chr1	179159759	179159809	25	51	2.120000	no	no	yes	no	no	no	no	yes	no	yes
182234002	chr1	179160148	179160199	22	52	2.454545	no	no	yes	no	no	no	no	no	no	yes
182234009	chr1	179174522	179174577	28	56	2.000000	no	no	yes	no	no	no	no	no	no	yes
182234015	chr1	179182887	179182925	19	39	2.052632	yes	no	yes	no	no	no	no	yes	no	yes
182234026	chr1	179213280	179213337	30	58	1.933333	no	no	yes	no	no	no	no	no	no	yes
182234037	chr1	179229624	179229667	12	44	3.666667	yes	no	no	no	yes	no	yes	yes	yes	yes
182234039	chr1	179229712	179229739	7	28	4.000000	yes	no	no	no	no	no	yes	yes	yes	no
182234086	chr1	179313495	179313595	32	101	3.156250	no	no	yes	no	no	no	no	no	no	yes

182234092	chr1	179337464	179337592	63	129	2.047619	no	no	yes	no	no	no	no	no	yes	yes
182234097	chr1	179342297	179342354	30	58	2.000000	no	no	yes	no	no	yes	no	yes	no	yes
182234106	chr1	179349625	179349661	17	37	2.176471	no	no	yes	no	no	no	no	yes	no	yes
182234109	chr1	179353047	179353100	18	54	3.052632	no	no	yes	no	no	no	no	no	no	yes
182234122	chr1	179394654	179394685	15	32	2.133333	no	no	yes	no	no	no	no	no	no	yes
182234123	chr1	179396769	179396805	19	37	1.947368	no	no	yes	no	no	no	no	no	no	yes
182234166	chr1	179503297	179503338	22	42	1.909091	no	no	yes	no	no	no	no	no	no	yes
182234173	chr1	179528240	179528277	20	38	1.900000	no	no	yes	no	no	no	no	no	yes	yes
182234181	chr1	179541639	179541733	40	95	2.475000	no	no	yes	no	no	no	no	no	no	yes
182234183	chr1	179550047	179550078	14	32	2.357143	yes	no	yes	no	no	yes	no	no	no	yes
182234184	chr1	179550141	179550183	20	43	2.200000	yes	no	yes	no	no	yes	no	no	no	yes
182234194	chr1	179586295	179586328	14	34	2.428571	yes	no	no	no	no	no	yes	yes	yes	no
182234196	chr1	179586449	179586487	17	39	2.294118	yes	no	no	no	no	no	yes	yes	yes	no
182234197	chr1	179587531	179587556	11	26	2.363636	no	yes	no	no	no	no	no	no	no	yes
182234204	chr1	179614289	179614383	29	95	3.275862	no	no	yes	no	no	no	no	no	no	yes
182234223	chr1	179706211	179706252	20	42	2.150000	no	no	no	no	no	no	no	yes	yes	no
182234229	chr1	179721693	179721727	18	35	1.944444	no	no	no	no	no	no	no	no	no	no
182234233	chr1	179731795	179731869	24	75	3.125000	no	no	yes	no	no	no	no	yes	yes	yes
182234242	chr1	179768302	179768351	23	50	2.217391	no	no	yes	no	no	no	no	no	no	yes
182234244	chr1	179771701	179771737	16	37	2.375000	no	no	yes	no	no	no	no	no	no	yes
182234248	chr1	179783755	179783868	61	114	1.918033	no	no	yes	no	no	no	no	no	no	yes
182234264	chr1	179857463	179857514	26	52	2.000000	no	no	yes	no	no	no	no	no	yes	yes
182234267	chr1	179864543	179864591	24	49	2.041667	no	yes	yes	no	no	yes	no	yes	yes	yes
182234269	chr1	179871051	179871132	40	82	2.050000	no	no	yes	no	no	no	no	yes	yes	yes
182234271	chr1	179880274	179880326	21	53	2.500000	no	no	no	no	no	no	no	yes	no	no

182234283	chr1	179897433	179897487	29	55	1.965517	no	no	yes	no	no	no	no	no	no	yes
182234285	chr1	179900017	179900075	18	59	3.210526	yes	no	yes	no	no	no	no	no	no	yes
182234332	chr1	180022184	180022221	17	38	2.235294	no	no	yes	no	no	no	no	no	no	yes
182234337	chr1	180030349	180030391	20	43	2.150000	no	no	yes	no	no	no	no	no	no	yes
182234350	chr1	180067932	180068002	35	71	2.028571	no	no	yes	no	no	no	no	no	no	yes
182234351	chr1	180070405	180070439	16	35	2.187500	no	no	yes	no	no	no	no	yes	no	yes
182234353	chr1	180074255	180074293	18	39	2.166667	no	no	yes	no	no	no	no	no	no	yes
182234360	chr1	180085472	180085545	22	74	3.363636	yes	no	yes	no	no	no	no	yes	no	yes
182234362	chr1	180092517	180092551	18	35	1.944444	no	no	yes	no	no	no	no	no	no	yes
182234382	chr1	180137108	180137150	9	43	4.777778	no	no	no	no	no	no	no	yes	yes	no
182234384	chr1	180138767	180139027	57	261	4.539682	no	no	no	no	no	no	no	no	no	no
182234386	chr1	180139028	180139081	19	54	3.000000	no	no	no	no	no	no	no	no	no	no
182234395	chr1	180154769	180154809	14	41	2.928571	yes	no	no	no	no	no	yes	yes	yes	no
182234414	chr1	180229990	180230032	20	43	2.150000	yes	no	no	no	no	no	yes	yes	yes	no
182234417	chr1	180240067	180240102	17	36	2.117647	no	no	yes	no	no	no	no	yes	no	yes
182234423	chr1	180244981	180245019	20	39	2.000000	no	no	yes	no	no	no	no	yes	yes	yes
182234424	chr1	180245393	180245419	10	27	2.700000	no	no	yes	no	no	no	no	yes	no	yes
182234428	chr1	180251064	180251119	17	56	3.352941	no	no	yes	no	no	no	no	yes	no	yes
182234438	chr1	180267211	180267260	24	50	2.083333	no	no	yes	no	no	no	no	yes	yes	yes
182234439	chr1	180286009	180286050	20	42	2.100000	no	no	yes	no	no	yes	no	no	no	yes
182234451	chr1	180345947	180345989	18	43	2.444444	no	no	yes	no	no	no	no	no	no	yes
182234457	chr1	180373685	180373730	22	46	2.090909	no	no	yes	no	no	no	no	no	no	yes
182234466	chr1	180400158	180400191	16	34	2.125000	no	no	yes	no	no	no	no	no	no	yes
182234476	chr1	180458975	180459011	13	37	2.923077	no	no	yes	no	no	no	no	yes	no	yes
182234488	chr1	180489381	180489416	19	36	1.947368	no	no	yes	no	no	no	no	no	yes	yes

182234490	chr1	180500856	180500912	29	57	1.965517	no	no	yes	no	no	no	no	yes	no	yes
182234496	chr1	180514568	180514641	23	74	3.217391	no	no	no	no	no	no	no	yes	no	no
182234501	chr1	180527646	180527676	16	31	1.937500	no	no	no	no	no	no	no	yes	no	no
182234510	chr1	180553175	180553211	17	37	2.235294	no	no	yes	no	no	no	no	no	no	yes
182234514	chr1	180572786	180572841	29	56	2.000000	no	no	no	no	no	no	no	yes	no	no
182234519	chr1	180577254	180577290	13	37	2.923077	no	no	no	no	no	no	no	no	no	no
182234522	chr1	180590788	180590816	14	29	2.071429	no	no	no	no	no	no	no	no	no	no
182234523	chr1	180595302	180595340	19	39	2.052632	no	no	no	no	no	no	no	yes	no	no
182234527	chr1	180599809	180599839	16	31	1.937500	no	no	no	no	no	no	no	yes	no	no
182234528	chr1	180600357	180600439	45	83	1.911111	no	no	no	no	no	no	no	yes	yes	no
182234532	chr1	180635937	180635975	19	39	2.052632	no	no	yes	no	no	no	no	yes	no	yes
182234534	chr1	180638789	180638832	21	44	2.095238	no	no	yes	no	no	no	no	yes	no	yes
182234548	chr1	180674715	180674789	37	75	2.027027	no	no	yes	no	no	no	no	yes	yes	yes
182234557	chr1	180687850	180687967	56	118	2.107143	no	no	yes	no	no	no	no	no	no	yes
182234588	chr1	180768498	180768527	15	30	2.000000	no	no	yes	no	no	no	no	no	yes	yes
182234592	chr1	180776418	180776458	21	41	1.952381	no	no	yes	no	no	no	no	yes	no	yes
182234599	chr1	180788930	180788985	26	56	2.153846	no	no	yes	no	no	no	no	no	no	yes
182234601	chr1	180801723	180801761	21	39	1.904762	no	no	yes	no	no	no	no	no	no	yes
182234608	chr1	180819499	180819538	21	40	1.952381	no	no	yes	no	no	no	no	no	no	yes
182234614	chr1	180838487	180838518	11	32	2.909091	no	no	yes	no	no	no	no	yes	no	yes
182234619	chr1	180857226	180857258	17	33	1.941176	no	no	yes	no	no	no	no	no	no	yes
182234620	chr1	180862402	180862436	15	35	2.333333	no	no	yes	no	no	no	no	yes	yes	yes
182234621	chr1	180864631	180864664	14	34	2.428571	no	no	yes	no	no	no	no	yes	no	yes
182234630	chr1	180897013	180897049	14	37	2.642857	no	no	no	no	no	no	no	no	no	no
182234641	chr1	180920699	180920741	22	43	1.954545	no	no	yes	no	no	no	no	yes	yes	yes

182234643	chr1	180928241	180928275	7	35	5.000000	yes	no	yes	no	yes	no	no	no	no	yes
182234648	chr1	180936976	180937000	7	25	3.571429	no	no	yes	no	no	no	no	yes	no	yes
182234658	chr1	180986125	180986149	12	25	2.083333	no	no	yes	no	no	no	no	no	no	yes
182234662	chr1	181011833	181011891	17	59	3.300000	no	no	yes	no	no	no	no	yes	yes	yes
182234664	chr1	181012067	181012114	17	48	2.882353	no	no	yes	no	no	no	no	yes	no	yes
182234670	chr1	181026896	181026932	16	37	2.312500	no	no	no	no	no	no	no	no	no	no
182234700	chr1	181105496	181105524	11	29	2.727273	no	no	no	no	no	no	yes	yes	yes	no
182234725	chr1	181159744	181159794	27	51	1.925926	no	no	no	no	no	no	no	yes	yes	no
182234727	chr1	181168569	181168604	19	36	1.947368	no	no	no	no	no	no	no	no	no	no
182234731	chr1	181175490	181175528	20	39	1.950000	no	no	yes	no	no	no	no	yes	no	yes
182234736	chr1	181203959	181203987	13	29	2.307692	no	no	no	no	no	no	no	no	yes	no
182234746	chr1	181243705	181243803	49	99	2.061224	no	no	no	no	no	no	no	no	no	no
182234774	chr1	181300449	181300485	19	37	2.000000	no	no	no	no	no	no	no	no	no	no
182234778	chr1	181318298	181318347	24	50	2.083333	no	no	yes	no	no	no	yes	yes	yes	yes
182234783	chr1	181335346	181335373	14	28	2.000000	no	no	yes	no	no	no	no	no	no	yes
182234788	chr1	181349645	181349781	42	137	3.261905	no	no	yes	no	no	no	no	yes	yes	yes
182234791	chr1	181360785	181360834	25	50	2.040000	no	no	yes	no	no	no	no	yes	yes	yes
182234792	chr1	181361064	181361099	7	36	5.428571	no	no	yes	no	no	no	no	yes	yes	yes
182234804	chr1	181385686	181385722	15	37	2.533333	no	no	yes	no	no	no	no	yes	no	yes
182234811	chr1	181400297	181400339	22	43	2.000000	no	no	yes	no	no	no	no	no	no	yes
182234820	chr1	181444722	181444748	12	27	2.250000	no	no	yes	no	no	no	no	yes	no	yes
182234821	chr1	181445454	181445494	20	41	2.100000	no	no	yes	no	no	no	no	yes	no	yes
182234822	chr1	181448856	181448908	23	53	2.304348	no	no	yes	no	no	no	no	no	no	yes
182234830	chr1	181470389	181470426	9	38	4.222222	no	no	yes	no	no	no	no	no	no	yes
182234831	chr1	181479518	181479549	16	32	2.000000	no	no	yes	no	no	no	no	yes	no	yes

182234833	chr1	181482603	181482646	22	44	2.136364	yes	no	yes	no	no	no	yes	yes	yes
182234837	chr1	181490618	181490660	19	43	2.368421	no	no	yes	no	no	no	no	yes	no	yes
182234838	chr1	181490645	181490677	11	33	3.000000	no	no	yes	no	no	no	no	no	no	yes
182234840	chr1	181492925	181492992	23	68	3.000000	no	no	yes	no	no	no	no	no	no	yes
182234841	chr1	181497385	181497426	21	42	2.047619	no	no	yes	no	no	no	no	no	no	yes
182234843	chr1	181507498	181507600	53	103	1.943396	no	no	yes	no	no	no	no	no	yes	yes
182234850	chr1	181546410	181546450	22	41	1.909091	no	no	yes	no	no	no	no	no	no	yes
182234851	chr1	181547193	181547334	52	142	2.730769	no	no	yes	no	no	no	no	yes	no	yes
182234856	chr1	181583057	181583099	18	43	2.400000	no	no	yes	no	no	no	no	no	no	yes
182234858	chr1	181584913	181584971	27	59	2.185185	no	no	yes	no	no	no	no	no	no	yes
182234877	chr1	181675228	181675258	14	31	2.214286	no	no	yes	no	no	no	no	yes	no	yes
182234878	chr1	181675389	181675417	15	29	1.933333	no	no	yes	no	no	no	no	yes	no	yes
182234879	chr1	181678533	181678570	19	38	2.000000	no	no	yes	no	no	no	no	no	no	yes
182234883	chr1	181696645	181696676	16	32	2.000000	no	no	yes	no	no	no	no	yes	yes	yes
182234887	chr1	181727601	181727634	16	34	2.125000	no	no	yes	no	no	no	no	no	no	yes
182234890	chr1	181732502	181732548	9	47	5.222222	no	no	no	yes	no	no	no	yes	no	yes
182234892	chr1	181732588	181732613	12	26	2.166667	no	no	yes	yes	no	no	no	yes	no	yes
182234893	chr1	181734393	181734930	75	538	7.213333	no	no	yes	no	no	no	no	no	no	yes
182234897	chr1	181781951	181782025	36	75	2.083333	no	no	yes	no	no	no	no	yes	no	yes
182234898	chr1	181814255	181814283	11	29	2.636364	no	no	no	no	no	yes	no	no	no	no
182234899	chr1	181816085	181816118	17	34	2.000000	no	no	no	no	no	no	no	no	no	no
182234930	chr1	181918682	181918713	14	32	2.285714	no	no	no	no	no	no	no	no	no	no
182234931	chr1	181931271	181931302	15	32	2.133333	no	no	no	no	no	no	no	no	no	no
182234943	chr1	181952928	181952963	14	36	2.642857	no	no	no	no	no	no	no	no	no	no
182234952	chr1	181983192	181983237	13	46	3.692308	no	no	yes	no	no	no	no	no	no	yes

182234955	chr1	181995374	181995419	21	46	2.190476	no	no	yes	no	no	no	no	no	yes	yes
182234968	chr1	182030651	182030713	32	63	2.000000	no	no	no	no	no	no	no	yes	no	no
182234971	chr1	182049322	182049357	16	36	2.250000	no	no	no	no	no	no	no	no	no	no
182234973	chr1	182054849	182054878	8	30	3.750000	no	yes	no	no	no	no	no	yes	yes	yes
182234974	chr1	182062091	182062120	15	30	2.000000	yes	no	no	no	no	yes	no	yes	no	no
182234975	chr1	182067741	182067787	24	47	2.000000	no	no	yes	no	no	no	no	no	no	yes
182234977	chr1	182081752	182081812	32	61	1.968750	no	no	no	no	no	no	no	no	no	no
182234985	chr1	182103854	182103903	16	50	3.125000	no	no	yes	no	no	no	no	no	no	yes
182234988	chr1	182103949	182103989	20	41	2.050000	no	no	yes	no	no	no	no	no	no	yes
182235004	chr1	182156102	182156144	23	43	1.913043	no	no	yes	no	no	no	no	no	yes	yes
182235010	chr1	182183335	182183376	22	42	1.909091	yes	no	yes	no	no	no	no	yes	no	yes
182235019	chr1	182194740	182194788	15	49	3.187500	no	no	yes	no	no	no	no	no	no	yes
182235022	chr1	182208908	182208944	19	37	1.947368	no	no	yes	no	no	no	no	yes	no	yes
182235023	chr1	182209189	182209221	16	33	2.062500	no	no	yes	no	no	no	no	no	yes	yes
182235024	chr1	182210720	182210761	21	42	2.000000	no	no	yes	no	no	no	no	yes	no	yes
182235025	chr1	182211061	182211089	15	29	1.933333	no	no	yes	no	no	no	no	yes	no	yes
182235026	chr1	182213206	182213247	21	42	2.000000	no	no	yes	no	no	no	no	no	no	yes
182235037	chr1	182236684	182236739	19	56	2.947368	no	no	yes	no	no	no	no	yes	no	yes
182235041	chr1	182237147	182237197	27	51	1.925926	no	no	yes	no	no	no	no	yes	yes	yes
182235043	chr1	182238389	182238451	33	63	1.939394	no	no	yes	no	no	no	no	no	no	yes
182235044	chr1	182238763	182238789	14	27	1.928571	no	no	yes	no	no	no	no	no	no	yes
182235054	chr1	182259416	182259467	26	52	2.000000	no	no	yes	no	no	no	no	yes	yes	yes
182235059	chr1	182269581	182269628	19	48	2.578947	no	no	yes	no	no	no	no	yes	yes	yes
182235066	chr1	182279452	182279492	15	41	2.800000	no	no	yes	no	no	no	no	yes	yes	yes
182235072	chr1	182293445	182293500	23	56	2.434783	yes	no	yes	no	no	no	no	yes	yes	yes

182235079	chr1	182295826	182295912	26	87	3.321429	no	no	yes	no	no	no	no	yes	no	yes
182235088	chr1	182298301	182298353	26	53	2.038461	no	no	yes	no	no	no	no	yes	yes	yes
182235093	chr1	182299942	182299994	15	53	3.533333	no	no	yes	no	no	no	no	yes	no	yes
182235099	chr1	182311009	182311063	26	55	2.115385	yes	no	yes	no	no	no	no	yes	no	yes
182235112	chr1	182329565	182329595	13	31	2.384615	no	no	no	no	no	yes	no	yes	yes	no
182235113	chr1	182332299	182332352	28	54	1.928571	no	no	no	no	no	no	no	yes	yes	no
182235121	chr1	182336654	182336683	13	30	2.307692	no	no	no	no	no	yes	no	yes	yes	no
182235127	chr1	182354198	182354241	17	44	2.611111	no	no	no	no	no	no	no	no	no	no
182235143	chr1	182377365	182377395	13	31	2.461539	no	no	no	no	no	no	no	yes	yes	no
182235153	chr1	182411404	182411444	19	41	2.210526	no	no	yes	no	no	no	no	no	no	yes
182235156	chr1	182424763	182424798	19	36	1.947368	no	no	yes	no	no	no	no	no	no	yes
182235162	chr1	182435732	182435760	13	29	2.230769	no	no	yes	no	no	no	no	no	no	yes
182235164	chr1	182436713	182436745	17	33	1.941176	no	no	yes	no	no	no	no	no	no	yes
182235174	chr1	182446635	182446672	14	38	2.714286	no	no	yes	no	no	no	no	yes	no	yes
182235175	chr1	182446740	182446986	43	247	6.069767	yes	no	yes	no	no	no	no	yes	no	yes
182235183	chr1	182460163	182460289	62	127	2.048387	no	no	yes	no	no	yes	no	yes	yes	yes
182235214	chr1	182551643	182551679	19	37	2.000000	no	no	yes	no	no	no	no	no	no	yes
182235220	chr1	182576344	182576386	22	43	2.045455	no	no	no	yes	no	yes	no	no	no	yes
182235240	chr1	182664316	182664346	15	31	2.066667	no	no	yes	no	no	no	no	yes	no	yes
182235241	chr1	182675063	182675123	30	61	2.066667	no	no	yes	no	no	no	no	yes	yes	yes
182235242	chr1	182679636	182679684	25	49	2.000000	no	no	yes	no	no	no	no	yes	no	yes
182235244	chr1	182684769	182684809	19	41	2.263158	yes	no	no	no	no	no	no	no	no	no
182235250	chr1	182701487	182701530	21	44	2.095238	no	no	no	no	no	no	no	no	yes	no
182235276	chr1	182743787	182743835	16	49	3.187500	no	no	no	no	no	no	no	yes	no	no
182235284	chr1	182750760	182750844	30	85	2.833333	no	no	no	no	no	no	no	no	no	no

182235288	chr1	182767543	182767573	13	31	2.384615	no	no	no	no	no	no	no	yes	no	no
182235294	chr1	182781733	182781770	19	38	2.000000	no	no	no	no	no	no	no	yes	no	no
182235298	chr1	182790469	182790606	70	138	1.971429	no	no	yes	no	no	no	no	yes	yes	yes
182235300	chr1	182794586	182794650	32	65	2.062500	no	no	yes	no	no	no	no	yes	no	yes
182235302	chr1	182799281	182799331	26	51	1.961538	no	no	yes	no	no	no	no	no	no	yes
182235306	chr1	182813449	182813487	20	39	1.950000	no	no	yes	no	no	no	no	no	no	yes
182235307	chr1	182813666	182813703	19	38	2.000000	no	no	yes	no	no	no	no	no	no	yes
182235310	chr1	182823807	182823930	63	124	2.000000	no	no	yes	no	no	no	no	no	no	yes
182235312	chr1	182838276	182838333	26	58	2.230769	no	no	yes	no	no	no	no	yes	no	yes
182235314	chr1	182839120	182839168	17	49	2.882353	yes	no	yes	no	no	no	no	yes	yes	yes
182235315	chr1	182845004	182845044	22	41	1.909091	no	no	yes	no	no	no	no	yes	yes	yes
182235317	chr1	182861116	182861169	16	54	3.277778	no	no	yes	no	no	yes	no	yes	no	yes
182235326	chr1	182869483	182869565	40	83	2.075000	no	no	yes	no	no	no	no	yes	yes	yes
182235327	chr1	182869579	182869611	15	33	2.200000	no	no	yes	no	no	no	no	yes	yes	yes
182235328	chr1	182874926	182874962	18	37	2.055556	no	no	yes	yes	no	no	no	no	no	yes
182235330	chr1	182883707	182883766	26	60	2.370370	no	no	yes	no	no	no	no	no	no	yes
182235333	chr1	182887178	182887221	15	44	2.933333	no	yes	no	yes	no	no	no	yes	yes	yes
182235346	chr1	182905743	182905780	18	38	2.166667	no	no	yes	no	no	no	no	yes	no	yes
182235374	chr1	182949724	182949773	26	50	1.923077	no	no	yes	no	no	yes	no	no	no	yes
182235390	chr1	182990528	182990660	33	133	4.030303	no	no	no	no	no	no	no	yes	yes	no
182235394	chr1	183003827	183003873	21	47	2.238095	no	no	no	no	no	no	no	no	no	no
182235401	chr1	183030766	183030809	21	44	2.095238	no	no	yes	no	no	no	no	yes	yes	yes
182235405	chr1	183050989	183051025	19	37	2.052632	no	no	yes	no	no	no	no	no	no	yes
182235406	chr1	183051986	183052068	43	83	1.953488	no	no	yes	no	no	no	no	yes	no	yes
182235409	chr1	183069312	183069388	39	77	1.974359	no	no	yes	no	no	no	no	no	no	yes

182235413	chr1	183104187	183104225	20	39	1.950000	no	no	yes	no	no	no	no	no	no	yes
182235416	chr1	183111515	183111551	17	37	2.235294	no	no	yes	no	no	yes	no	no	no	yes
182235418	chr1	183127126	183127150	11	25	2.272727	no	no	yes	no	no	no	no	no	no	yes
182235422	chr1	183149358	183150266	30	909	28.977272	no	no	no	no	no	no	no	yes	yes	no
182235429	chr1	183172767	183172817	14	51	3.600000	no	no	no	no	no	no	no	no	no	no
182235432	chr1	183173075	183173191	54	117	2.166667	no	no	no	no	no	no	no	no	no	no
182235434	chr1	183184995	183185030	18	36	2.000000	no	no	no	no	no	no	no	no	no	no
182235435	chr1	183185594	183185632	20	39	1.950000	yes	no	no	no	no	no	no	yes	no	no
182235442	chr1	183199482	183199510	13	29	2.307692	no	no	yes	no	no	no	no	no	no	yes
182235447	chr1	183208375	183208414	21	40	1.904762	no	no	yes	no	no	no	no	no	yes	yes
182235450	chr1	183232919	183232965	22	47	2.136364	no	no	yes	no	no	no	no	no	yes	yes
182235454	chr1	183234325	183234357	14	33	2.357143	no	no	yes	no	no	no	no	yes	yes	yes
182235456	chr1	183244368	183244430	12	63	5.153846	no	yes	no	no	no	yes	no	yes	no	yes
182235462	chr1	183252216	183252374	39	159	4.102564	yes	yes	no	no	no	yes	no	yes	no	yes
182235480	chr1	183277588	183277626	11	39	3.545455	no	no	yes	no	no	no	no	yes	no	yes
182235486	chr1	183305142	183305208	31	67	2.187500	yes	no	yes	no	no	no	no	no	yes	yes
182235487	chr1	183305291	183305325	17	35	2.058824	yes	no	yes	no	no	no	no	no	yes	yes
182235493	chr1	183319604	183319646	16	43	2.705882	no	no	yes	no	no	no	no	no	no	yes
182235494	chr1	183319637	183319683	24	47	1.958333	no	no	yes	no	no	no	no	no	no	yes
182235500	chr1	183328451	183328475	13	25	1.923077	no	no	yes	no	no	no	no	yes	yes	yes
182235501	chr1	183328967	183329010	9	44	5.333333	no	no	yes	no	no	no	no	no	no	yes
182235503	chr1	183333928	183334017	45	90	2.022222	no	no	yes	no	no	no	no	no	yes	yes
182235505	chr1	183344130	183344165	12	36	3.000000	no	no	yes	no	no	no	no	yes	no	yes
182235517	chr1	183374781	183374818	19	38	2.157895	no	no	yes	no	no	yes	no	yes	no	yes
182235526	chr1	183390933	183391005	36	73	2.027778	no	no	yes	no	no	no	no	no	no	yes

182235528	chr1	183413558	183413594	19	37	1.947368	no	no	yes	no	no	no	no	yes	no	yes
182235535	chr1	183430470	183430525	23	56	2.434783	no	no	no	no	no	no	no	yes	no	no
182235537	chr1	183436888	183436929	21	42	2.000000	no	no	no	no	no	no	no	yes	no	no
182235558	chr1	183472355	183472391	18	37	2.055556	yes	no	no	no	yes	no	no	yes	yes	yes
182235559	chr1	183477420	183477799	32	380	11.941176	no	no	yes	no	no	no	no	yes	yes	yes
182235561	chr1	183477801	183477841	18	41	2.277778	no	no	yes	no	no	no	no	yes	yes	yes
182235569	chr1	183504995	183505035	17	41	2.470588	no	no	yes	no	no	no	no	yes	no	yes
182235570	chr1	183509609	183509654	24	46	1.916667	no	no	yes	no	no	no	no	no	no	yes
182235571	chr1	183512597	183512638	20	42	2.200000	no	no	yes	no	no	no	no	no	yes	yes
182235572	chr1	183523935	183523976	16	42	2.687500	no	no	yes	no	no	no	no	no	no	yes
182235574	chr1	183525982	183526007	13	26	2.000000	no	no	yes	no	no	no	no	no	no	yes
182235575	chr1	183531118	183531159	22	42	1.954545	no	no	yes	no	no	no	no	no	no	yes
182235577	chr1	183535933	183535976	22	44	2.045455	no	no	yes	no	no	no	no	no	no	yes
182235578	chr1	183536245	183536287	14	43	3.071429	no	no	yes	no	no	no	no	no	no	yes
182235588	chr1	183584920	183584948	14	29	2.071429	no	no	yes	no	no	no	no	yes	no	yes
182235592	chr1	183606393	183606424	16	32	2.000000	no	no	no	no	no	no	no	yes	no	no
182235593	chr1	183606432	183606475	14	44	3.066667	no	no	no	no	no	no	no	no	no	no
182235599	chr1	183627322	183627361	20	40	2.000000	no	yes	yes	no	yes	no	no	no	yes	yes
182235601	chr1	183631662	183631697	15	36	2.400000	no	no	yes	no	yes	no	no	no	no	yes
182235602	chr1	183633308	183633336	14	29	2.071429	yes	no	yes	no	yes	no	no	yes	yes	yes
182235609	chr1	183675296	183675327	17	32	1.941176	no	no	yes	no	no	no	no	no	no	yes
182235620	chr1	183691815	183691846	16	32	2.125000	no	no	yes	no	no	no	no	no	yes	yes
182235624	chr1	183698100	183698141	22	42	1.954545	no	no	yes	no	no	no	no	yes	yes	yes
182235626	chr1	183715361	183715396	15	36	2.400000	no	no	yes	no	no	no	no	no	no	yes
182235627	chr1	183715381	183715422	21	42	2.000000	no	no	yes	no	no	no	no	no	no	yes

182235632	chr1	183733617	183733657	20	41	2.100000	no	no	yes	no	no	no	no	no	yes	yes
182235633	chr1	183734980	183735016	19	37	1.947368	no	no	yes	no	no	no	no	yes	yes	yes
182235634	chr1	183736644	183736684	15	41	2.866667	no	no	yes	no	no	no	no	no	yes	yes
182235641	chr1	183755205	183755249	22	45	2.045455	yes	no	yes	no	no	no	no	no	no	yes
182235642	chr1	183755398	183755426	15	29	1.933333	yes	no	yes	no	no	no	no	no	no	yes
182235645	chr1	183771294	183771325	17	32	1.941176	no	no	yes	no	no	no	no	no	no	yes
182235649	chr1	183782998	183783036	19	39	2.105263	no	no	yes	no	no	no	no	no	no	yes
182235661	chr1	183827345	183827381	19	37	1.947368	no	no	yes	no	no	no	no	no	no	yes
182235662	chr1	183829551	183829589	18	39	2.222222	no	no	yes	no	no	no	no	no	no	yes
182235673	chr1	183887148	183887204	20	57	2.809524	no	no	yes	no	no	no	no	no	yes	yes
182235675	chr1	183895419	183895471	28	53	1.964286	no	no	yes	no	no	no	no	yes	no	yes
182235679	chr1	183901654	183901701	25	48	1.960000	no	no	yes	no	no	no	no	no	no	yes
182235688	chr1	183934919	183934961	17	43	2.529412	no	no	yes	no	no	yes	no	no	no	yes
182235689	chr1	183936206	183936234	14	29	2.071429	no	yes	yes	no	yes	no	no	yes	no	yes
182235691	chr1	183952987	183953022	16	36	2.250000	no	no	yes	no	no	no	no	no	no	yes
182235692	chr1	183955647	183955727	36	81	2.250000	no	no	yes	no	no	no	no	no	no	yes
182235699	chr1	183976359	183976399	15	41	2.800000	no	no	yes	no	no	no	no	no	no	yes
182235704	chr1	183995717	183995754	19	38	2.000000	no	no	yes	no	no	no	no	yes	no	yes
182235705	chr1	183996984	183997024	20	41	2.050000	no	no	yes	no	no	no	no	yes	yes	yes
182235710	chr1	184004803	184004842	10	40	4.100000	no	no	yes	no	no	no	no	no	no	yes
182235712	chr1	184010357	184010403	23	47	2.086957	no	no	yes	no	no	no	no	no	no	yes
182235714	chr1	184027707	184027741	16	35	2.187500	no	no	yes	no	no	no	no	no	no	yes
182235716	chr1	184036602	184036637	18	36	2.055556	no	no	yes	no	no	no	yes	yes	yes	yes
182235719	chr1	184048409	184048448	18	40	2.277778	no	no	no	no	no	no	no	no	no	no
182235722	chr1	184054096	184054136	20	41	2.100000	yes	no	yes	no	no	no	no	yes	yes	yes

182235723	chr1	184058530	184058562	17	33	2.000000	no	no	yes	no	no	no	no	no	no	yes
182235724	chr1	184061649	184061692	19	44	2.350000	no	no	yes	no	no	no	no	yes	no	yes
182235726	chr1	184084422	184084491	23	70	3.043478	no	no	yes	no	no	no	no	no	no	yes
182235727	chr1	184085815	184085862	15	48	3.125000	no	no	yes	no	no	no	no	yes	yes	yes
182235732	chr1	184097925	184097964	21	40	1.952381	no	no	yes	no	no	yes	no	yes	no	yes
182235733	chr1	184103301	184103336	18	36	2.000000	no	no	yes	no	no	no	no	no	no	yes
182235734	chr1	184103920	184103949	16	30	1.937500	no	no	yes	no	no	no	no	no	no	yes
182235740	chr1	184110751	184110784	18	34	1.944444	no	no	yes	no	no	no	no	yes	no	yes
182235748	chr1	184111075	184111132	30	58	1.933333	no	no	yes	no	no	no	no	yes	no	yes
182235755	chr1	184113137	184113187	27	51	1.962963	no	no	yes	no	no	no	no	no	no	yes
182235768	chr1	184142877	184142927	25	51	2.040000	no	no	yes	no	no	no	no	yes	no	yes
182235792	chr1	184235641	184235682	11	42	3.750000	no	no	no	no	no	no	no	no	no	no
182235796	chr1	184257632	184257678	23	47	2.043478	no	no	no	no	no	no	no	no	no	no
182235803	chr1	184292130	184292158	14	29	2.071429	no	no	no	no	no	no	no	no	no	no
182235805	chr1	184293264	184293291	14	28	2.000000	no	no	no	no	no	no	no	no	no	no
182235814	chr1	184321420	184321455	18	36	2.055556	no	no	no	no	no	no	no	yes	no	no
182235817	chr1	184327820	184327920	31	101	3.212121	no	no	no	no	no	no	no	yes	no	no
182235823	chr1	184353898	184353929	16	32	2.000000	no	no	no	no	no	no	no	no	no	no
182235824	chr1	184354088	184354131	21	44	2.095238	no	no	no	no	no	no	no	no	no	no
182235829	chr1	184358520	184358600	41	81	1.975610	no	no	no	no	no	no	no	no	no	no
182235833	chr1	184380231	184380264	8	34	4.250000	no	no	no	no	no	no	no	no	no	no
182235840	chr1	184387268	184387313	23	46	2.043478	yes	no	no	no	yes	no	yes	yes	no	yes
182235845	chr1	184401724	184401757	17	34	2.000000	no	no	yes	no	no	no	no	no	no	yes
182235849	chr1	184427894	184427932	20	39	1.950000	no	no	yes	no	no	no	no	yes	yes	yes
182235853	chr1	184456566	184456659	47	94	2.000000	no	no	yes	no	no	no	no	no	no	yes

182235855	chr1	184462554	184462583	15	30	2.000000	no	no	yes	no	no	no	no	yes	yes	yes
182235859	chr1	184468031	184468075	22	45	2.045455	no	no	yes	no	no	no	no	yes	yes	yes
182235862	chr1	184474959	184475001	12	43	3.583333	no	no	yes	no	no	no	no	no	no	yes
182235871	chr1	184505858	184505890	13	33	2.538461	no	no	yes	no	no	no	no	no	no	yes
182235872	chr1	184506977	184507018	17	42	2.470588	no	no	yes	no	no	no	no	no	no	yes
182235873	chr1	184520699	184520738	15	40	2.666667	no	no	yes	no	no	no	no	yes	no	yes
182235876	chr1	184524598	184524638	21	41	2.000000	no	no	yes	no	no	no	no	yes	no	yes
182235878	chr1	184530397	184530450	15	54	3.600000	no	no	yes	no	no	no	no	yes	no	yes
182235902	chr1	184596511	184596539	14	29	2.071429	no	no	yes	no	no	no	no	no	no	yes
182235908	chr1	184607598	184607765	26	168	6.692307	no	no	yes	no	yes	no	no	no	no	yes
182235909	chr1	184609685	184609709	13	25	1.923077	no	no	yes	no	yes	no	no	no	no	yes
182235912	chr1	184624736	184624776	21	41	2.000000	no	yes	yes	no	no	no	no	yes	yes	yes
182235915	chr1	184638759	184638800	15	42	2.800000	no	no	yes	no	no	no	no	no	no	yes
182235917	chr1	184640756	184640816	29	61	2.172414	no	no	yes	no	no	no	no	no	no	yes
182235926	chr1	184652409	184652458	25	50	2.080000	no	no	yes	no	no	no	no	no	no	yes
182235930	chr1	184656572	184656614	16	43	2.647059	no	no	yes	no	no	no	no	no	no	yes
182235931	chr1	184657887	184657947	27	61	2.259259	no	no	yes	no	no	no	no	no	no	yes
182235936	chr1	184660043	184660067	11	25	2.272727	no	no	yes	no	no	no	no	yes	no	yes
182235946	chr1	184691607	184691642	19	36	1.947368	no	yes	no	no	no	no	no	yes	yes	yes
182235951	chr1	184701859	184701891	14	33	2.357143	no	no	yes	no	no	no	no	yes	no	yes
182235954	chr1	184718302	184718333	16	32	2.000000	no	no	yes	no	no	no	no	no	no	yes
182235958	chr1	184739226	184739282	29	57	1.965517	no	no	yes	no	no	no	no	no	no	yes
182235962	chr1	184739285	184739323	21	39	1.904762	no	no	yes	no	no	no	no	no	no	yes
182235967	chr1	184743475	184743510	15	36	2.400000	no	no	yes	no	no	no	no	yes	no	yes
182235972	chr1	184751152	184751194	18	43	2.444444	no	no	yes	no	no	no	no	no	no	yes

182235973	chr1	184756011	184756042	7	32	4.375000	no	no	no	no	no	no	no	yes	no	no
182235988	chr1	184778359	184778391	17	33	1.941176	no	no	no	no	no	no	no	no	no	no
182235992	chr1	184793930	184793960	16	31	1.937500	no	yes	yes	no	no	yes	no	yes	yes	yes
182235999	chr1	184809596	184809712	30	117	3.843750	no	no	yes	no	no	no	no	no	no	yes
182236003	chr1	184818505	184818545	20	41	2.050000	no	no	yes	no	no	no	no	no	no	yes
182236004	chr1	184825449	184825483	18	35	2.000000	no	no	yes	no	no	no	no	no	no	yes
182236011	chr1	184858177	184858381	105	205	2.009524	no	no	yes	no	no	no	no	yes	no	yes
182236012	chr1	184859033	184859069	18	37	2.055556	no	no	yes	no	no	no	no	no	no	yes
182236023	chr1	184874396	184874439	18	44	2.444444	no	no	yes	no	no	no	no	no	no	yes
182236027	chr1	184899383	184899452	24	70	2.958333	no	no	yes	no	no	no	no	no	no	yes
182236028	chr1	184899555	184899589	18	35	1.944444	no	no	yes	no	no	no	no	no	no	yes
182236043	chr1	184949024	184949049	13	26	2.000000	no	no	yes	no	no	no	no	no	no	yes
182236054	chr1	184989040	184989075	14	36	2.571429	no	no	no	no	no	no	no	no	no	no
182236066	chr1	185038737	185038810	38	74	1.973684	no	no	no	no	no	no	no	no	no	no
182236087	chr1	185128919	185128961	18	43	2.388889	no	no	yes	no	no	no	no	no	no	yes
182236094	chr1	185144042	185144118	36	77	2.138889	no	no	yes	no	no	no	no	no	no	yes
182236104	chr1	185185762	185185797	18	36	2.000000	no	no	yes	no	no	no	no	no	no	yes
182236105	chr1	185187441	185187480	20	40	2.050000	no	no	yes	no	no	no	no	no	no	yes
182236108	chr1	185206900	185206985	44	86	1.954545	no	no	yes	no	no	no	no	yes	no	yes
182236119	chr1	185229744	185229768	13	25	1.923077	no	no	yes	no	no	no	no	no	no	yes
182236120	chr1	185232070	185232122	26	53	2.038461	no	no	yes	no	no	no	no	no	no	yes
182236121	chr1	185242723	185242763	20	41	2.100000	no	no	yes	no	no	no	no	no	no	yes
182236125	chr1	185248721	185248751	16	31	1.937500	no	no	yes	no	no	no	no	yes	no	yes
182236134	chr1	185269260	185269337	40	78	1.950000	no	no	yes	no	no	no	no	yes	no	yes
182236137	chr1	185274234	185274342	33	109	3.128205	no	no	yes	no	no	no	no	no	no	yes

182236140	chr1	185281004	185281053	24	50	2.083333	no	yes	yes	no	no	no	no	yes	yes	yes
182236141	chr1	185281266	185281319	27	54	2.000000	no	yes	yes	no	no	no	no	yes	yes	yes
182236143	chr1	185286837	185286877	10	41	4.100000	no	no	yes	no	no	no	no	yes	no	yes
182236144	chr1	185306620	185306651	16	32	2.000000	yes	no	yes	no	yes	yes	no	no	no	yes
182236145	chr1	185307256	185307304	18	49	2.722222	yes	no	yes	no	no	yes	no	no	yes	yes
182236147	chr1	185314744	185314820	40	77	1.925000	no	no	yes	no	no	no	no	yes	yes	yes
182236159	chr1	185347590	185347618	15	29	1.933333	no	no	no	no	no	no	no	no	no	no
182236161	chr1	185370252	185370312	30	61	2.033333	no	no	no	no	no	no	no	no	no	no
182236163	chr1	185371823	185371855	16	33	2.062500	no	no	no	no	no	no	no	no	no	no
182236171	chr1	185394762	185394805	24	44	1.916667	no	no	no	no	no	no	no	no	no	no
182236173	chr1	185399369	185399433	32	65	2.031250	no	no	no	no	no	no	no	yes	yes	no
182236174	chr1	185402205	185402241	20	37	1.950000	no	no	no	no	no	no	no	no	no	no
182236177	chr1	185409320	185409361	22	42	1.954545	no	no	no	no	no	no	no	yes	no	no
182236180	chr1	185420830	185421002	47	173	3.680851	no	no	no	no	no	no	no	yes	yes	no
182236184	chr1	185431957	185432005	16	49	3.250000	no	no	no	no	no	no	no	no	no	no
182236189	chr1	185445459	185445490	16	32	2.062500	no	no	no	no	no	no	no	no	no	no
182236190	chr1	185446625	185446650	13	26	2.000000	no	no	no	no	no	no	no	no	no	no
182236196	chr1	185468165	185468532	183	368	2.010929	no	no	no	no	no	no	no	no	no	no
182236202	chr1	185480313	185480394	32	82	2.562500	no	no	no	no	no	no	no	no	no	no
182236203	chr1	185484461	185484500	19	40	2.157895	no	no	no	no	no	no	no	yes	no	no
182236216	chr1	185515394	185515476	41	83	2.024390	no	no	no	no	no	no	no	no	no	no
182236224	chr1	185532953	185532992	14	40	2.857143	no	no	no	no	no	no	no	no	yes	no
182236239	chr1	185598037	185598074	15	38	2.533333	no	no	yes	no	no	no	no	yes	no	yes
182236250	chr1	185620266	185620395	24	130	5.333333	no	no	yes	no	no	no	no	no	no	yes
182236277	chr1	185657576	185657633	23	58	2.652174	no	no	no	no	no	yes	no	yes	no	no

182236292	chr1	185676370	185676404	18	35	1.944444	no	no	no	no	no	no	no	no	no	no
182236297	chr1	185687556	185687615	27	60	2.259259	no	no	no	no	no	no	no	yes	no	no
182236299	chr1	185692779	185692806	14	28	2.000000	no	no	no	no	no	no	no	no	no	no
182236307	chr1	185717558	185717589	14	32	2.428571	no	no	no	no	no	no	no	yes	no	no
182236320	chr1	185776096	185776141	22	46	2.136364	no	no	yes	no	no	no	no	no	no	yes
182236324	chr1	185786977	185787016	21	40	1.904762	no	no	yes	no	no	no	no	no	no	yes
182236326	chr1	185789613	185789637	7	25	3.571429	no	no	yes	no	no	no	no	yes	no	yes
182236344	chr1	185821010	185821054	21	45	2.142857	no	no	yes	no	no	no	no	no	no	yes
182236355	chr1	185862094	185862128	17	35	2.117647	no	no	yes	no	no	no	no	yes	no	yes
182236359	chr1	185868727	185868767	19	41	2.210526	no	no	yes	no	no	no	no	no	no	yes
182236363	chr1	185893490	185893527	13	38	2.928571	no	no	yes	no	no	no	no	yes	no	yes
182236369	chr1	185897201	185897288	43	88	2.046512	no	no	yes	no	no	no	no	yes	yes	yes
182236373	chr1	185908819	185908858	20	40	2.050000	no	no	yes	no	no	no	no	no	no	yes
182236378	chr1	185935628	185935663	19	36	1.947368	no	no	yes	no	no	no	no	yes	no	yes
182236379	chr1	185936662	185936696	16	35	2.187500	no	no	yes	no	no	no	no	no	no	yes
182236386	chr1	185959654	185959688	18	35	1.944444	no	no	yes	no	no	no	no	yes	no	yes
182236387	chr1	185969740	185969764	12	25	2.083333	no	no	yes	no	no	no	no	yes	yes	yes
182236392	chr1	185985215	185985246	17	32	1.941176	no	no	yes	no	no	no	no	no	no	yes
182236397	chr1	186005094	186005576	137	483	3.688406	no	no	yes	no	no	no	no	yes	no	yes
182236402	chr1	186010173	186010219	18	47	2.611111	no	no	yes	no	no	no	no	no	no	yes
182236404	chr1	186017269	186017344	39	76	1.974359	no	no	yes	no	no	no	no	no	yes	yes
182236409	chr1	186036903	186036948	24	46	2.000000	no	no	yes	no	no	no	no	yes	no	yes
182236410	chr1	186049236	186049627	209	392	1.894737	no	no	yes	no	no	no	no	no	no	yes
182236424	chr1	186091367	186091603	125	237	1.896000	no	no	yes	no	no	no	no	no	yes	yes
182236425	chr1	186092069	186092101	16	33	2.062500	no	no	yes	no	no	no	no	no	no	yes

182236427	chr1	186092739	186092782	20	44	2.200000	no	no	yes	no	no	no	no	no	no	yes
182236429	chr1	186108745	186108774	12	30	2.500000	no	no	yes	no	no	no	no	yes	no	yes
182236432	chr1	186129097	186129148	22	52	2.375000	no	no	yes	no	no	no	no	no	no	yes
182236447	chr1	186185087	186185125	21	39	1.904762	no	no	yes	no	no	no	no	no	no	yes
182236453	chr1	186193212	186193352	47	141	2.940000	no	no	no	no	no	no	no	yes	no	no
182236457	chr1	186201297	186201349	22	53	2.409091	no	no	no	no	no	no	no	yes	no	no
182236464	chr1	186238873	186238897	10	25	2.500000	no	no	no	no	no	no	no	yes	no	no
182236475	chr1	186269679	186269709	12	31	2.583333	no	no	no	no	no	no	no	no	no	no
182236488	chr1	186292320	186292359	22	40	1.909091	no	no	no	no	no	no	no	no	no	no
182236490	chr1	186303692	186303739	22	48	2.181818	no	no	yes	no	no	no	no	yes	no	yes
182236496	chr1	186307755	186308260	207	506	2.444444	no	no	no	yes	no	no	no	no	no	yes
182236499	chr1	186308577	186308618	21	42	2.000000	no	no	no	yes	no	no	no	no	yes	yes
182236503	chr1	186319419	186319451	12	33	2.833333	no	no	yes	no	no	no	no	no	no	yes
182236508	chr1	186326972	186327344	37	373	10.439024	yes	no	yes	no	no	no	no	yes	no	yes
182236512	chr1	186328620	186328712	45	93	2.066667	no	no	yes	no	no	no	no	no	no	yes
182236518	chr1	186352594	186352634	20	41	2.050000	no	no	yes	no	no	no	no	yes	no	yes
182236525	chr1	186389408	186389450	18	43	2.388889	no	no	yes	no	no	no	no	no	no	yes
182236527	chr1	186391000	186391025	10	26	2.600000	no	no	yes	no	no	no	no	yes	no	yes
182236530	chr1	186396812	186396853	22	42	2.000000	no	no	yes	no	no	no	no	yes	no	yes
182236543	chr1	186408489	186408544	30	56	1.933333	no	no	yes	no	no	no	no	no	no	yes
182236545	chr1	186410296	186410333	19	38	2.000000	no	no	yes	no	no	no	no	no	no	yes
182236571	chr1	186489702	186489796	39	95	2.435897	no	no	yes	no	no	no	no	no	no	yes
182236575	chr1	186494192	186494244	22	53	2.409091	no	no	yes	no	no	no	no	no	no	yes
182236577	chr1	186494923	186494962	13	40	3.000000	no	no	yes	no	no	no	no	yes	yes	yes
182236586	chr1	186516799	186516833	17	35	2.058824	no	no	yes	no	no	no	no	no	no	yes

182236595	chr1	186557502	186557540	14	39	2.800000	no	no	no	no	no	no	no	yes	no	no
182236610	chr1	186617896	186617924	15	29	1.933333	no	no	no	no	no	no	no	yes	no	no
182236612	chr1	186629202	186629231	15	30	2.000000	no	no	no	no	no	no	no	yes	yes	no
182236617	chr1	186643420	186643453	16	34	2.125000	no	no	no	no	no	no	no	no	no	no
182236634	chr1	186667252	186667299	23	48	2.086957	no	no	no	no	no	no	no	no	no	no
182236639	chr1	186677415	186677465	20	51	2.550000	no	no	yes	no	no	yes	no	yes	no	yes
182236645	chr1	186700204	186700237	7	34	4.857143	no	no	no	no	no	no	no	no	no	no
182236649	chr1	186713814	186713860	22	47	2.136364	no	no	no	no	no	no	no	no	no	no
182236654	chr1	186721166	186721226	30	61	2.033333	no	no	no	no	no	no	no	no	no	no
182236670	chr1	186795381	186796372	495	992	2.004040	no	no	no	no	no	no	no	no	no	no
182236677	chr1	186807736	186807783	25	48	1.920000	no	no	no	no	no	no	no	no	no	no
182236689	chr1	186836099	186836134	19	36	1.947368	no	no	yes	no	no	no	no	yes	no	yes
182236694	chr1	186838280	186838328	25	49	1.960000	no	no	yes	no	no	no	no	no	no	yes
182236698	chr1	186848518	186848550	15	33	2.266667	no	no	yes	no	no	no	no	yes	no	yes
182236704	chr1	186857085	186857175	39	91	2.375000	no	no	yes	no	no	no	no	no	no	yes
182236716	chr1	186870484	186870943	230	460	2.008696	no	yes	yes	yes	no	no	no	yes	yes	yes
182236719	chr1	186875658	186875704	15	47	3.133333	no	no	yes	no	no	no	no	no	no	yes
182236722	chr1	186878086	186878376	39	291	7.538462	no	no	yes	no	no	no	no	no	no	yes
182236727	chr1	186891141	186891173	7	33	4.857143	no	no	yes	no	no	no	no	no	no	yes
182236741	chr1	186934464	186934566	48	103	2.145833	no	no	yes	no	no	no	no	no	no	yes
182236743	chr1	186936608	186936636	14	29	2.071429	no	no	yes	no	no	no	no	no	no	yes
182236745	chr1	186943521	186943558	19	38	2.000000	no	no	yes	no	no	no	no	no	no	yes
182236747	chr1	186948722	186948752	16	31	1.937500	no	no	yes	no	no	no	no	yes	yes	yes
182236759	chr1	186973905	186973931	12	27	2.250000	no	no	yes	no	no	no	no	no	no	yes
182236760	chr1	186979780	186979810	15	31	2.066667	no	no	yes	no	no	no	no	no	no	yes

182236763	chr1	186995422	186995453	14	32	2.357143	no	no	no	no	no	no	no	no	no	no
182236774	chr1	187036272	187036331	18	60	3.333333	no	no	no	no	no	no	no	yes	no	no
182236779	chr1	187052434	187052464	15	31	2.066667	no	no	no	no	no	no	no	no	no	no
182236793	chr1	187078350	187078378	15	29	1.933333	no	no	yes	no	no	no	no	no	no	yes
182236805	chr1	187112983	187113017	12	35	2.916667	no	no	yes	no	no	no	no	no	no	yes
182236806	chr1	187120159	187120188	8	30	3.750000	no	no	yes	no	no	no	no	no	yes	yes
182236807	chr1	187127201	187127229	15	29	2.000000	no	no	yes	no	no	no	no	no	no	yes
182236818	chr1	187144546	187144599	26	54	2.076923	no	no	yes	no	no	no	no	no	no	yes
182236827	chr1	187147716	187147767	27	52	1.962963	no	no	yes	no	no	no	no	no	no	yes
182236841	chr1	187191832	187191873	21	42	2.047619	no	no	yes	no	no	no	no	no	no	yes
182236845	chr1	187202858	187202899	19	42	2.263158	no	no	yes	no	no	no	no	no	no	yes
182236850	chr1	187213339	187213372	15	34	2.266667	no	no	yes	no	no	no	no	no	no	yes
182236854	chr1	187222400	187222445	18	46	2.578947	no	no	yes	no	no	no	no	no	no	yes
182236858	chr1	187249631	187249674	14	44	3.214286	no	no	yes	no	no	no	no	no	no	yes
182236870	chr1	187280224	187280248	13	25	1.923077	no	no	yes	no	no	no	no	no	yes	yes
182236877	chr1	187308096	187308128	17	33	1.941176	no	no	yes	no	no	no	no	no	no	yes
182236878	chr1	187308956	187308990	17	35	2.058824	no	no	yes	no	no	no	no	no	no	yes
182236881	chr1	187322359	187322400	16	42	2.625000	no	no	yes	no	no	no	no	no	no	yes
182236882	chr1	187327720	187327752	17	33	1.941176	no	no	yes	no	no	no	no	no	yes	yes
182236891	chr1	187393280	187393311	15	32	2.133333	no	no	yes	no	no	no	no	no	no	yes
182236893	chr1	187395650	187395690	20	41	2.100000	no	no	yes	no	no	no	no	no	no	yes
182236895	chr1	187402133	187402166	14	34	2.428571	no	no	yes	no	no	no	no	no	no	yes
182236906	chr1	187432841	187432903	18	63	3.611111	no	no	yes	no	no	no	no	no	no	yes
182236911	chr1	187447553	187447581	14	29	2.071429	no	no	yes	no	no	no	no	no	no	yes
182236912	chr1	187455552	187455591	20	40	2.000000	no	no	yes	no	no	no	no	no	no	yes

182236913	chr1	187456465	187456500	18	36	2.000000	no	no	yes	no	no	no	no	yes	no	yes
182236916	chr1	187471063	187471107	21	45	2.190476	no	no	yes	no	no	no	no	no	no	yes
182236917	chr1	187473341	187473419	38	79	2.078947	no	no	yes	no	no	no	no	yes	no	yes
182236920	chr1	187482511	187482632	65	122	1.923077	no	no	yes	no	no	no	no	no	no	yes
182236926	chr1	187511406	187511453	24	48	2.000000	no	no	yes	no	no	no	no	no	no	yes
182236933	chr1	187536111	187536172	26	62	2.461539	no	no	yes	no	no	no	no	no	no	yes
182236938	chr1	187546773	187546798	13	26	2.000000	no	no	yes	no	no	no	no	no	no	yes
182236952	chr1	187563458	187563511	27	54	2.000000	no	yes	yes	no	no	no	no	yes	no	yes
182236957	chr1	187574922	187575023	48	102	2.125000	no	no	yes	no	no	no	no	no	no	yes
182236967	chr1	187597512	187597566	29	55	1.931034	no	no	yes	no	no	no	no	no	no	yes
182236969	chr1	187603687	187603767	17	81	4.823529	no	no	yes	no	no	no	no	no	no	yes
182236981	chr1	187627619	187627656	15	38	2.666667	no	no	yes	no	no	no	no	yes	yes	yes
182236990	chr1	187639452	187639490	12	39	3.250000	no	no	yes	no	no	no	no	yes	no	yes
182236991	chr1	187646955	187646993	20	39	1.950000	no	no	yes	no	no	no	no	yes	yes	yes
182236995	chr1	187656702	187656726	13	25	1.923077	no	no	yes	no	no	no	no	no	no	yes
182236999	chr1	187666380	187666577	33	198	5.970588	no	no	yes	no	no	no	no	yes	no	yes
182237000	chr1	187667838	187667879	19	42	2.210526	no	no	yes	no	no	no	no	yes	no	yes
182237001	chr1	187669748	187669790	23	43	1.913043	no	no	yes	no	no	no	no	no	no	yes
182237013	chr1	187694042	187694082	21	41	1.952381	no	no	no	no	no	no	no	no	no	no
182237017	chr1	187697764	187697845	38	82	2.210526	no	no	no	no	no	no	no	no	no	no
182237025	chr1	187716642	187716737	48	96	2.000000	no	no	no	no	no	no	no	no	no	no
182237038	chr1	187754782	187754813	16	32	2.062500	no	no	no	no	no	no	no	no	no	no
182237045	chr1	187760904	187760972	36	69	1.916667	no	no	no	no	no	no	no	no	no	no
182237057	chr1	187797734	187797805	36	72	2.055556	no	no	no	no	no	no	no	no	no	no
182237058	chr1	187798764	187798792	14	29	2.071429	no	no	no	no	no	no	no	no	no	no

182237059	chr1	187799606	187799653	25	48	1.920000	no	no	no	no	no	no	no	no	no	no
182237062	chr1	187809542	187809593	20	52	2.600000	no	no	no	no	no	no	no	yes	no	no
182237067	chr1	187833050	187833122	37	73	1.972973	no	no	no	no	no	no	no	no	no	no
182237075	chr1	187852363	187852431	35	69	1.971429	no	no	no	no	no	no	no	yes	no	no
182237083	chr1	187868610	187868649	12	40	3.285714	no	no	no	no	no	no	no	yes	no	no
182237087	chr1	187885097	187885131	17	35	2.058824	no	no	no	no	no	no	no	no	no	no
182237096	chr1	187951343	187951379	19	37	1.947368	no	no	no	no	no	no	no	no	no	no
182237105	chr1	187963077	187963135	27	59	2.185185	no	no	no	no	no	no	no	no	no	no
182237107	chr1	187975920	187975972	24	53	2.208333	no	no	no	no	no	no	no	no	no	no
182237108	chr1	187977127	187977177	18	51	2.944444	no	no	no	no	no	no	no	no	no	no
182237116	chr1	187993426	187993485	30	60	2.000000	no	no	no	no	no	no	no	no	no	no
182237118	chr1	187994587	187994628	15	42	2.800000	no	no	no	no	no	no	no	yes	no	no
182237122	chr1	188001603	188001639	16	37	2.375000	no	no	no	no	no	no	no	no	no	no
182237131	chr1	188022943	188023063	40	121	3.000000	no	no	yes	no	no	no	no	yes	yes	yes
182237139	chr1	188050688	188050741	28	54	1.964286	no	no	yes	no	no	no	no	yes	no	yes
182237143	chr1	188062498	188062536	9	39	4.333333	no	no	yes	no	no	no	no	yes	no	yes
182237159	chr1	188089995	188090057	23	63	2.791667	no	no	yes	no	no	no	no	no	no	yes
182237160	chr1	188094369	188094404	14	36	2.571429	no	no	yes	no	no	no	no	no	no	yes
182237164	chr1	188114577	188114605	13	29	2.230769	no	no	yes	no	no	no	no	no	no	yes
182237176	chr1	188144508	188144536	12	29	2.416667	no	yes	no	no	no	no	no	no	no	yes
182237177	chr1	188149014	188149062	17	49	2.882353	no	no	no	no	no	no	no	no	no	no
182237179	chr1	188160833	188160871	20	39	1.950000	no	no	no	no	no	no	no	yes	no	no
182237180	chr1	188163634	188163670	14	37	2.600000	no	no	no	no	no	no	no	no	no	no
182237181	chr1	188171045	188171085	22	41	1.954545	no	no	no	no	no	no	no	yes	yes	no
182237184	chr1	188180686	188180804	42	119	2.880952	no	no	no	no	no	no	no	no	no	no

182237188	chr1	188186977	188187032	24	56	2.320000	no	no	no	no	no	no	no	no	no	no
182237197	chr1	188201497	188201526	14	30	2.142857	no	no	no	no	no	no	no	no	no	no
182237210	chr1	188213248	188213281	12	34	2.846154	no	no	no	no	no	no	no	no	no	no
182237216	chr1	188231705	188231766	23	62	2.695652	no	no	no	no	no	no	no	no	no	no
182237223	chr1	188237139	188237183	19	45	2.400000	no	no	no	no	no	no	no	no	no	no
182237254	chr1	188271739	188271786	23	48	2.086957	no	no	no	no	no	no	no	yes	no	no
182237261	chr1	188273939	188273971	16	33	2.062500	no	no	no	no	no	no	no	yes	no	no
182237262	chr1	188273956	188274006	25	51	2.120000	no	no	no	no	no	no	no	yes	no	no
182237273	chr1	188311056	188311096	18	41	2.333333	no	no	no	no	no	no	no	no	no	no
182237274	chr1	188321411	188321482	34	72	2.117647	no	no	no	no	no	no	no	no	no	no
182237279	chr1	188327836	188327896	30	61	2.033333	no	no	no	no	no	no	no	no	no	no
182237294	chr1	188361159	188361207	17	49	2.882353	no	no	no	no	no	no	no	no	no	no
182237295	chr1	188363558	188363606	25	49	2.080000	no	no	no	no	no	no	no	yes	no	no
182237297	chr1	188372467	188372520	20	54	2.700000	no	no	no	no	no	no	no	no	no	no
182237301	chr1	188384708	188384746	21	39	1.952381	no	no	no	no	no	no	no	no	no	no
182237314	chr1	188424801	188424833	15	33	2.200000	no	no	no	no	no	no	no	no	no	no
182237316	chr1	188424911	188424996	17	86	5.000000	no	no	no	no	no	no	no	no	no	no
182237319	chr1	188428218	188428248	12	31	2.583333	no	no	no	no	no	no	no	no	no	no
182237324	chr1	188446853	188447044	76	192	2.519480	no	no	no	no	no	no	no	yes	no	no
182237327	chr1	188452889	188452918	14	30	2.142857	no	no	no	no	no	no	no	no	no	no
182237328	chr1	188454242	188454268	14	27	1.928571	no	no	no	no	no	no	no	no	no	no
182237330	chr1	188471535	188471570	16	36	2.312500	no	no	no	no	no	no	no	no	no	no
182237334	chr1	188473944	188473968	12	25	2.083333	no	no	no	no	no	no	no	yes	no	no
182237339	chr1	188493470	188493498	15	29	1.933333	no	no	no	no	no	no	no	yes	no	no
182237349	chr1	188509441	188509468	14	28	2.000000	no	no	yes	no	no	no	no	no	no	yes

182237350	chr1	188512010	188512064	20	55	2.800000	no	no	yes	no	no	no	no	no	no	yes
182237351	chr1	188514471	188514522	25	52	2.080000	no	no	yes	no	no	no	no	no	no	yes
182237359	chr1	188532120	188532152	17	33	1.941176	no	no	yes	no	no	no	no	no	no	yes
182237360	chr1	188532214	188532373	84	160	1.916667	no	no	yes	no	no	no	no	no	no	yes
182237367	chr1	188555218	188555254	15	37	2.466667	no	no	no	no	no	no	no	no	no	no
182237369	chr1	188556044	188556090	20	47	2.380952	no	no	no	no	no	no	no	no	no	no
182237375	chr1	188566038	188566110	38	73	1.921053	no	no	no	no	no	no	no	no	no	no
182237378	chr1	188568610	188568656	25	47	1.920000	no	no	no	no	no	no	no	no	no	no
182237379	chr1	188568684	188568732	23	49	2.173913	no	no	no	no	no	no	no	no	no	no
182237384	chr1	188586530	188586575	22	46	2.090909	no	no	no	no	no	no	no	no	no	no
182237387	chr1	188593393	188593427	18	35	1.944444	no	no	no	no	no	no	no	yes	no	no
182237389	chr1	188600112	188600175	31	64	2.064516	no	no	no	no	no	no	no	no	no	no
182237394	chr1	188605075	188605116	22	42	1.954545	no	no	no	no	no	no	no	no	no	no
182237397	chr1	188607283	188607419	26	137	5.500000	no	no	no	no	no	no	no	no	no	no
182237402	chr1	188633290	188633323	17	34	2.000000	no	no	no	no	no	no	no	no	no	no
182237404	chr1	188638887	188638927	18	41	2.277778	no	no	no	no	no	no	no	no	no	no
182237405	chr1	188649578	188649625	26	48	1.923077	no	no	no	no	no	no	no	no	no	no
182237406	chr1	188651200	188651249	25	50	2.000000	no	no	no	no	no	no	no	no	no	no
182237407	chr1	188653671	188653732	32	62	1.937500	no	no	no	no	no	no	no	no	no	no
182237409	chr1	188654657	188654708	24	52	2.291667	no	no	no	no	no	no	no	no	no	no
182237413	chr1	188659526	188659584	31	59	1.903226	no	no	no	no	no	no	no	no	yes	no
182237417	chr1	188673157	188673197	21	41	1.952381	no	no	no	no	no	no	no	no	no	no
182237426	chr1	188680172	188680233	23	62	2.708333	no	no	no	no	no	no	no	no	no	no
182237429	chr1	188688194	188688242	22	49	2.227273	no	no	no	no	no	no	no	no	no	no
182237437	chr1	188697164	188697298	39	135	3.487805	no	no	no	no	no	no	no	yes	no	no

182237445	chr1	188716969	188717009	13	41	3.153846	no	no	yes	no	no	no	no	no	no	yes
182237451	chr1	188748929	188748979	24	51	2.125000	no	no	no	no	no	no	no	no	no	no
182237457	chr1	188766806	188766853	20	48	2.523809	no	no	no	no	no	no	no	no	no	no
182237459	chr1	188777424	188777461	14	38	2.714286	no	no	no	no	no	no	no	no	no	no
182237461	chr1	188781087	188781135	22	49	2.227273	no	no	no	no	no	no	no	yes	no	no
182237477	chr1	188807941	188807985	24	45	1.875000	no	no	no	no	no	no	no	no	no	no
182237521	chr1	188868582	188868620	20	39	2.050000	no	no	no	no	no	yes	no	no	no	no
182237531	chr1	188886173	188886205	15	33	2.200000	no	no	yes	no	no	no	no	no	no	yes
182237546	chr1	188916302	188916342	16	41	2.562500	no	no	yes	no	no	no	no	no	no	yes
182237553	chr1	188967252	188967280	14	29	2.071429	no	no	yes	no	no	no	no	no	no	yes
182237554	chr1	188967663	188967699	18	37	2.055556	no	no	yes	no	no	no	no	no	no	yes
182237560	chr1	188988341	188988384	16	44	2.750000	no	no	yes	no	no	no	no	no	no	yes
182237561	chr1	188994010	188994051	21	42	2.095238	no	no	yes	no	no	no	no	no	no	yes
182237562	chr1	189004178	189004225	26	48	1.923077	no	no	yes	no	no	no	no	no	no	yes
182237569	chr1	189010567	189010601	18	35	1.944444	no	no	yes	no	no	no	no	no	no	yes
182237581	chr1	189028282	189028314	16	33	2.187500	no	no	yes	no	no	no	no	no	no	yes
182237583	chr1	189030641	189030693	22	53	2.409091	no	no	yes	no	no	no	no	no	no	yes
182237585	chr1	189031609	189031667	27	59	2.185185	no	no	yes	no	no	no	no	no	no	yes
182237588	chr1	189045025	189045073	25	49	1.960000	no	no	no	no	no	no	no	no	no	no
182237592	chr1	189059575	189059663	34	89	2.617647	no	no	no	no	no	no	no	no	no	no
182237593	chr1	189063838	189063967	61	130	2.129032	no	no	no	no	no	no	no	yes	no	no
182237594	chr1	189064330	189064378	25	49	1.960000	no	no	no	no	no	no	no	yes	no	no
182237598	chr1	189068858	189068942	34	85	2.472222	no	no	no	no	no	no	no	no	no	no
182237603	chr1	189077582	189077618	16	37	2.375000	no	no	no	no	no	no	no	no	no	no
182237605	chr1	189077763	189077801	19	39	2.105263	no	no	no	no	no	no	no	no	no	no

182237610	chr1	189107851	189107879	13	29	2.230769	no	no	no	no	no	no	no	no	no	no
182237614	chr1	189120520	189120553	18	34	1.944444	no	no	no	no	no	no	no	no	no	no
182237618	chr1	189128806	189128838	17	33	1.941176	no	no	no	no	no	no	no	no	no	no
182237622	chr1	189128932	189128999	35	68	1.971429	no	no	no	no	no	no	no	no	no	no
182237641	chr1	189177855	189177899	16	45	2.812500	no	no	no	no	no	no	no	no	no	no
182237644	chr1	189179228	189179271	21	44	2.238095	no	no	no	no	no	no	no	no	no	no
182237646	chr1	189190270	189190381	58	112	1.931034	no	no	no	no	no	no	no	no	no	no
182237653	chr1	189216756	189216793	20	38	1.900000	no	no	no	no	no	no	no	no	no	no
182237656	chr1	189222279	189222330	24	52	2.166667	no	no	no	no	no	no	no	no	no	no
182237669	chr1	189231872	189231926	22	55	2.500000	no	no	no	no	no	no	no	no	no	no
182237674	chr1	189232615	189232666	10	52	5.500000	no	no	no	no	no	no	no	no	no	no
182237675	chr1	189238611	189238644	16	34	2.125000	no	no	no	no	no	no	no	no	no	no
182237676	chr1	189238719	189238769	26	51	1.961538	no	no	no	no	no	no	no	no	no	no
182237703	chr1	189295733	189295781	22	49	2.272727	no	no	no	no	no	no	no	no	no	no
182237708	chr1	189311467	189311502	16	36	2.312500	no	no	no	no	no	no	no	no	no	no
182237709	chr1	189320773	189320820	24	48	2.083333	no	no	no	no	no	no	no	no	no	no
182237711	chr1	189324531	189324610	36	80	2.222222	no	no	no	no	no	no	no	no	no	no
182237715	chr1	189332255	189332296	16	42	2.588235	no	no	no	no	no	no	no	no	no	no
182237716	chr1	189333339	189333371	16	33	2.062500	no	no	no	no	no	no	no	no	no	no
182237721	chr1	189341706	189341769	27	64	2.407408	no	no	no	no	no	no	no	no	no	no
182237727	chr1	189352612	189352653	23	42	1.913043	no	no	no	no	no	no	no	no	no	no
182237731	chr1	189363219	189363320	33	102	3.090909	no	no	no	no	no	no	no	no	no	no
182237732	chr1	189366146	189366180	18	35	1.944444	no	no	no	no	no	no	no	no	no	no
182237734	chr1	189375774	189375806	14	33	2.500000	no	no	no	no	no	no	no	no	no	no
182237742	chr1	189388683	189388722	18	40	2.277778	no	no	no	no	no	no	no	yes	no	no

182237767	chr1	189427544	189427583	16	40	2.562500	no	no	no	no	no	no	no	no	no	no
182237771	chr1	189428810	189428847	19	38	2.105263	no	no	no	no	no	no	no	no	no	no
182237773	chr1	189440168	189440468	155	301	1.954839	no	no	no	no	no	no	no	no	no	no
182237775	chr1	189450433	189450476	21	44	2.095238	no	no	no	no	no	no	no	no	no	no
182237780	chr1	189455628	189455672	22	45	2.136364	no	no	no	no	no	no	no	no	no	no
182237784	chr1	189468661	189468702	19	42	2.263158	no	no	no	no	no	no	no	no	no	no
182237788	chr1	189474777	189474844	34	68	2.058824	no	no	no	no	no	no	no	no	no	no
182237789	chr1	189478002	189478055	24	54	2.333333	no	no	no	no	no	no	no	no	no	no
182237791	chr1	189482182	189482214	16	33	2.062500	no	no	no	no	no	no	no	no	no	no
182237799	chr1	189499199	189499241	23	43	1.956522	no	no	no	no	no	no	no	no	no	no
182237800	chr1	189499461	189499499	20	39	1.950000	no	no	no	no	no	no	no	no	no	no
182237803	chr1	189504061	189504089	14	29	2.071429	no	no	no	no	no	no	no	no	no	no
182237805	chr1	189507853	189507887	18	35	1.944444	no	no	no	no	no	no	no	no	no	no
182237807	chr1	189511615	189511675	11	61	5.500000	no	no	no	no	no	no	no	yes	no	no
182237815	chr1	189533727	189533772	20	46	2.300000	no	no	no	no	no	no	no	no	no	no
182237819	chr1	189544610	189544672	31	63	2.032258	no	no	no	no	no	no	no	no	no	no
182237825	chr1	189554184	189554237	22	54	2.454545	no	no	no	no	no	no	no	no	no	no
182237836	chr1	189562889	189562949	31	61	2.032258	no	no	no	no	no	no	no	no	no	no
182237840	chr1	189571235	189571291	26	57	2.307692	no	no	no	no	no	no	no	no	no	no
182237851	chr1	189580786	189580815	15	30	2.000000	no	no	no	no	no	no	no	no	no	no
182237852	chr1	189585015	189585046	12	32	2.666667	no	no	no	no	no	no	no	yes	no	no
182237857	chr1	189592568	189592605	16	38	2.375000	no	no	no	no	no	no	no	no	no	no
182237858	chr1	189593453	189593502	25	50	2.000000	no	no	no	no	no	no	no	no	no	no
182237861	chr1	189597333	189597407	35	75	2.142857	no	no	no	no	no	no	no	yes	yes	no
182237862	chr1	189597463	189597500	19	38	2.052632	no	no	no	no	no	no	no	no	no	no

182237869	chr1	189604106	189604144	21	39	1.952381	no	no	no	no	no	no	no	no	no	no
182237870	chr1	189604525	189604581	27	57	2.148148	no	no	no	no	no	no	no	no	no	no
182237890	chr1	189640660	189640692	15	33	2.200000	no	no	no	no	no	no	no	no	no	no
182237897	chr1	189654420	189654458	20	39	2.000000	no	no	no	no	no	no	no	no	no	no
182237901	chr1	189672926	189672991	34	66	1.941176	no	no	no	no	no	no	no	no	yes	no
182237908	chr1	189691678	189691723	25	46	1.920000	no	no	no	no	no	no	no	no	no	no
182237912	chr1	189697616	189697649	18	34	1.944444	no	no	no	no	no	no	no	no	no	no
182237915	chr1	189699930	189699975	25	46	1.920000	no	no	no	no	no	no	no	no	no	no
182237918	chr1	189706805	189706891	28	87	3.107143	no	no	no	no	no	no	no	no	yes	no
182237922	chr1	189732881	189732911	16	31	1.937500	no	no	no	no	no	no	no	no	no	no
182237925	chr1	189744922	189745032	52	111	2.192308	no	no	no	no	no	no	no	no	no	no
182237926	chr1	189745792	189745833	21	42	2.000000	no	no	no	no	no	no	no	no	no	no
182237933	chr1	189765460	189765490	15	31	2.066667	no	no	no	no	no	no	no	no	no	no
182237940	chr1	189783207	189783231	12	25	2.083333	no	no	yes	no	no	no	no	no	no	yes
182237949	chr1	189799892	189799921	14	30	2.142857	no	no	yes	no	no	no	no	no	no	yes
182237952	chr1	189807771	189807807	12	37	3.000000	no	no	yes	no	no	no	no	no	no	yes
182237963	chr1	189831629	189831662	18	34	1.944444	no	no	no	no	no	no	no	no	no	no
182237964	chr1	189832317	189832349	18	33	1.944444	no	no	no	no	no	no	no	no	no	no
182237975	chr1	189867347	189867409	32	63	1.968750	yes	no	no	no	no	yes	no	no	no	no
182237976	chr1	189867543	189867591	17	49	2.888889	yes	no	no	no	no	yes	no	no	no	no
182238007	chr1	189998238	189998276	17	39	2.294118	no	no	no	no	no	no	no	no	no	no
182238010	chr1	190000940	190000971	16	32	2.000000	no	no	no	no	no	no	no	no	no	no
182238011	chr1	190007391	190007456	18	66	3.722222	no	no	no	no	no	no	no	no	no	no
182238020	chr1	190018163	190018187	13	25	1.923077	no	no	no	no	no	no	no	no	no	no
182238022	chr1	190018691	190018778	39	88	2.256410	no	no	no	no	no	no	no	no	no	no

182238038	chr1	190053813	190053855	23	43	1.956522	no	no	no	no	no	no	no	yes	yes	no
182238039	chr1	190054623	190054652	15	30	2.000000	no	no	no	no	no	no	no	no	no	no
182238041	chr1	190063998	190064039	22	42	1.954545	no	no	no	no	no	no	no	no	no	no
182238051	chr1	190086960	190086991	15	32	2.133333	no	no	no	no	no	no	no	no	no	no
182238055	chr1	190101549	190101593	14	45	3.133333	no	no	yes	no	no	no	no	no	no	yes
182238056	chr1	190107208	190107318	57	111	1.947368	no	no	yes	no	no	no	no	no	no	yes
182238057	chr1	190109132	190109163	17	32	1.941176	no	no	yes	no	no	no	no	yes	no	yes
182238065	chr1	190125314	190125360	17	47	2.764706	no	no	yes	no	no	no	no	no	no	yes
182238066	chr1	190127162	190127197	18	36	2.000000	no	no	yes	no	no	no	no	yes	no	yes
182238072	chr1	190150193	190150263	33	71	2.151515	no	no	yes	no	no	no	no	no	no	yes
182238073	chr1	190152139	190152179	19	41	2.210526	no	no	yes	no	no	no	no	yes	no	yes
182238075	chr1	190161382	190161429	21	48	2.333333	no	no	yes	no	no	no	no	no	no	yes
182238079	chr1	190195215	190195337	41	123	3.000000	no	no	yes	no	no	no	no	no	no	yes
182238084	chr1	190203399	190203498	30	100	3.333333	no	no	yes	no	no	no	no	no	no	yes
182238086	chr1	190204266	190204307	13	42	3.230769	no	no	yes	no	no	no	no	no	no	yes
182238091	chr1	190206960	190207007	17	48	2.833333	no	no	yes	no	no	no	no	no	no	yes
182238092	chr1	190207934	190207963	14	30	2.142857	no	no	yes	no	no	no	no	yes	yes	yes
182238099	chr1	190235343	190235371	14	29	2.071429	no	no	yes	no	no	no	no	no	no	yes
182238104	chr1	190247853	190247877	13	25	1.923077	no	no	yes	no	no	no	no	yes	no	yes
182238112	chr1	190282377	190282414	20	38	1.900000	no	no	yes	no	no	no	no	no	no	yes
182238115	chr1	190284918	190284996	39	79	2.025641	no	no	yes	no	no	no	no	yes	no	yes
182238123	chr1	190302234	190302295	31	62	2.064516	no	no	yes	no	no	no	no	no	no	yes
182238126	chr1	190308132	190308277	72	146	2.027778	no	no	yes	no	no	no	no	yes	no	yes
182238128	chr1	190318755	190319069	169	315	1.863905	no	no	yes	no	no	no	no	no	no	yes
182238133	chr1	190333534	190333574	19	41	2.210526	no	no	yes	no	no	no	no	no	no	yes

182238146	chr1	190373453	190373504	26	52	2.000000	no	no	yes	no	no	no	no	yes	no	yes
182238147	chr1	190374281	190374325	8	45	5.625000	no	no	yes	no	no	no	no	yes	no	yes
182238159	chr1	190386234	190386286	25	53	2.153846	no	no	yes	no	no	no	no	yes	yes	yes
182238162	chr1	190400527	190400553	13	27	2.076923	no	no	yes	no	no	no	no	yes	no	yes
182238163	chr1	190401927	190402002	30	76	2.531250	no	no	yes	no	no	no	no	no	no	yes
182238164	chr1	190402323	190402364	22	42	1.954545	no	no	yes	no	no	no	no	yes	no	yes
182238168	chr1	190407971	190408017	23	47	2.043478	no	no	yes	no	no	no	no	no	no	yes
182238174	chr1	190412867	190412903	19	37	2.000000	no	no	yes	no	no	no	no	no	no	yes
182238177	chr1	190415207	190415241	18	35	1.944444	no	no	yes	no	no	no	no	yes	yes	yes
182238178	chr1	190417571	190417607	15	37	2.466667	no	no	yes	no	no	no	no	no	no	yes
182238180	chr1	190419705	190419765	30	61	2.066667	no	no	yes	no	no	no	no	no	no	yes
182238181	chr1	190420670	190420708	19	39	2.052632	no	no	yes	no	no	no	no	no	no	yes
182238183	chr1	190421590	190421658	34	69	2.058824	no	no	yes	no	no	no	no	no	no	yes
182238184	chr1	190424065	190424117	16	53	3.312500	no	no	yes	no	no	no	no	no	no	yes
182238192	chr1	190442671	190442813	65	143	2.200000	no	no	yes	no	no	no	no	no	no	yes
182238199	chr1	190446089	190446125	18	37	2.111111	no	no	yes	no	no	no	no	no	no	yes
182238205	chr1	190456073	190456104	16	32	2.062500	no	no	yes	no	no	no	no	yes	no	yes
182238210	chr1	190467818	190467842	13	25	1.923077	no	no	yes	no	no	no	no	no	no	yes
182238211	chr1	190471006	190471031	13	26	2.000000	no	no	yes	no	no	no	no	no	yes	yes
182238212	chr1	190472436	190472473	20	38	1.900000	no	no	yes	no	no	no	no	no	no	yes
182238215	chr1	190477229	190477266	20	38	1.900000	no	no	yes	no	no	yes	no	yes	no	yes
182238224	chr1	190495630	190495672	21	43	2.047619	no	no	no	no	no	no	no	no	no	no
182238229	chr1	190512695	190512746	12	52	4.142857	no	no	no	no	no	no	no	no	no	no
182238233	chr1	190520165	190520206	21	42	2.047619	no	no	no	no	no	no	no	yes	no	no
182238236	chr1	190521677	190521774	30	98	3.300000	no	no	no	no	no	no	no	no	no	no

182238238	chr1	190521831	190521861	16	31	1.937500	no	no	no	no	no	no	no	no	no	no
182238240	chr1	190529849	190529886	17	38	2.235294	no	no	no	no	no	no	no	no	no	no
182238241	chr1	190531840	190531893	28	54	1.964286	no	no	no	no	no	no	no	no	no	no
182238246	chr1	190542443	190542474	13	32	2.461539	no	no	no	no	no	no	no	no	no	no
182238271	chr1	190636556	190636749	94	194	2.074468	no	no	yes	no	no	no	no	no	no	yes
182238272	chr1	190638785	190638829	21	45	2.190476	no	no	yes	no	no	no	no	no	no	yes
182238280	chr1	190657906	190657945	21	40	1.904762	no	no	yes	no	no	no	no	no	no	yes
182238287	chr1	190703534	190703571	19	38	2.000000	no	no	yes	no	no	no	no	no	no	yes
182238288	chr1	190705562	190705591	15	30	2.000000	no	no	yes	no	no	no	no	no	no	yes
182238290	chr1	190708631	190708664	18	34	2.000000	no	no	yes	no	no	no	no	yes	no	yes
182238293	chr1	190720376	190720422	23	47	2.043478	no	no	yes	no	no	no	no	no	no	yes
182238296	chr1	190729399	190729441	22	43	1.954545	no	no	yes	no	no	no	no	yes	no	yes
182238299	chr1	190729476	190729518	22	43	2.000000	no	no	yes	no	no	no	no	yes	no	yes
182238303	chr1	190729729	190729768	19	40	2.105263	no	no	yes	no	no	no	no	yes	no	yes
182238305	chr1	190729759	190729812	26	54	2.115385	no	no	yes	no	no	no	no	yes	no	yes
182238306	chr1	190742485	190742534	25	50	2.040000	no	no	yes	no	no	no	no	no	no	yes
182238311	chr1	190759002	190759049	23	48	2.086957	no	no	yes	no	no	no	no	no	no	yes
182238313	chr1	190764645	190764676	16	32	2.062500	no	no	yes	no	no	no	no	no	no	yes
182238323	chr1	190794571	190794619	21	49	2.428571	no	no	yes	no	no	no	no	no	no	yes
182238326	chr1	190797487	190797537	20	51	2.550000	yes	yes	yes	no	no	no	no	yes	no	yes
182238328	chr1	190801225	190801268	24	44	1.916667	no	yes	no	no	no	no	no	yes	no	yes
182238337	chr1	190840037	190840062	12	26	2.166667	no	no	no	no	no	no	no	no	no	no
182238338	chr1	190841822	190841899	24	78	3.250000	no	no	no	no	no	no	no	yes	no	no
182238342	chr1	190874323	190874355	14	33	2.428571	no	no	no	no	no	no	no	no	no	no
182238347	chr1	190888214	190888246	16	33	2.062500	no	no	yes	no	no	no	no	no	no	yes

182238349	chr1	190895997	190896047	18	51	2.833333	no	no	yes	no	no	no	no	no	no	yes
182238354	chr1	190896354	190896406	27	53	1.962963	no	no	yes	no	no	no	no	no	no	yes
182238360	chr1	190921104	190921451	180	348	1.938889	no	no	yes	no	no	no	no	no	no	yes
182238362	chr1	190922367	190922431	34	65	1.970588	no	no	yes	no	no	no	no	no	no	yes
182238367	chr1	190936914	190936954	18	41	2.277778	no	no	yes	no	no	no	no	no	no	yes
182238376	chr1	190948151	190948208	23	58	2.500000	no	no	yes	no	no	no	no	no	no	yes
182238378	chr1	190957978	190958006	15	29	1.933333	no	no	yes	no	no	no	no	yes	no	yes
182238380	chr1	190958971	190959013	19	43	2.315789	no	no	yes	no	no	no	no	no	no	yes
182238387	chr1	190966771	190966860	29	90	3.103448	no	no	yes	no	no	no	no	no	no	yes
182238392	chr1	190977952	190977987	18	36	2.000000	no	no	yes	no	no	no	no	no	no	yes
182238401	chr1	190982868	190983006	36	139	3.837838	no	no	yes	no	no	no	no	no	no	yes
182238403	chr1	190983219	190983251	14	33	2.357143	no	no	yes	no	no	no	no	no	no	yes
182238405	chr1	190987315	190987355	22	41	1.909091	no	no	yes	no	no	no	no	no	no	yes
182238433	chr1	191054118	191054155	19	38	2.000000	no	no	yes	no	no	no	no	no	no	yes
182238436	chr1	191066701	191066742	15	42	2.866667	no	no	yes	no	no	no	no	no	no	yes
182238440	chr1	191071542	191071584	22	43	2.000000	no	no	yes	no	no	no	no	no	no	yes
182238441	chr1	191074844	191074875	16	32	2.000000	no	no	yes	no	no	no	no	yes	no	yes
182238452	chr1	191096535	191096660	37	126	3.358974	no	no	yes	no	no	no	no	no	no	yes
182238453	chr1	191097109	191097161	13	53	4.071429	no	no	yes	no	no	no	no	no	no	yes
182238463	chr1	191120206	191120331	63	126	2.000000	no	no	yes	no	no	no	no	no	no	yes
182238473	chr1	191133813	191133859	23	47	2.043478	no	no	yes	no	no	no	no	no	no	yes
182238489	chr1	191161034	191161071	16	38	2.437500	no	no	no	no	no	no	no	yes	no	no
182238492	chr1	191162518	191162571	27	54	2.074074	no	no	no	no	no	no	no	no	no	no
182238496	chr1	191170173	191170212	19	40	2.105263	no	no	no	no	no	no	no	no	no	no
182238508	chr1	191195029	191195227	99	199	2.010101	no	no	no	no	no	no	no	no	no	no

182238509	chr1	191196566	191196619	26	54	2.076923	no	no	no	no	no	no	no	no	no	no
182238510	chr1	191196933	191197031	34	99	2.911765	no	no	no	no	no	no	no	no	no	no
182238519	chr1	191220712	191220749	17	38	2.294118	no	no	no	no	no	yes	no	no	no	no
182238526	chr1	191234628	191234728	15	101	7.000000	no	no	no	no	no	no	no	no	no	no
182238541	chr1	191318639	191318698	31	60	1.935484	no	no	no	no	no	no	no	no	no	no
182238543	chr1	191329929	191329955	14	27	1.928571	no	no	no	no	no	no	no	no	no	no
182238547	chr1	191338683	191338717	15	35	2.400000	no	no	no	no	no	no	no	no	no	no
182238556	chr1	191357795	191357827	14	33	2.428571	no	no	no	no	no	no	no	no	no	no
182238567	chr1	191380622	191380664	22	43	1.954545	no	no	no	no	no	no	no	no	yes	no
182238572	chr1	191404794	191404829	17	36	2.117647	no	no	no	no	no	no	no	no	yes	no
182238584	chr1	191441651	191441676	13	26	2.000000	no	no	no	no	no	no	no	no	no	no
182238594	chr1	191476532	191476577	22	46	2.090909	no	no	no	no	no	no	no	no	no	no
182238599	chr1	191476803	191476853	26	51	1.961538	no	no	no	no	no	no	no	no	no	no
182238600	chr1	191477498	191477539	22	42	1.954545	no	no	no	no	no	no	no	no	no	no
182238608	chr1	191494742	191494788	18	47	2.666667	no	no	no	no	no	no	no	no	no	no
182238610	chr1	191504412	191504443	15	32	2.133333	no	no	no	no	no	no	no	no	no	no
182238611	chr1	191507634	191507674	12	41	3.416667	no	no	no	no	no	no	no	no	no	no
182238614	chr1	191509234	191509312	43	79	1.976744	no	no	no	no	no	no	no	no	no	no
182238620	chr1	191518455	191518492	19	38	2.000000	no	no	no	no	no	no	no	no	no	no
182238630	chr1	191548783	191548827	24	45	1.958333	no	no	no	no	no	no	no	no	no	no
182238642	chr1	191586195	191586235	12	41	3.416667	no	no	no	no	no	no	no	no	no	no
182238661	chr1	191610412	191610502	42	91	2.166667	no	no	no	no	no	no	no	no	no	no
182238665	chr1	191627395	191627435	21	41	1.952381	no	no	no	no	no	no	no	no	no	no
182238667	chr1	191627947	191627991	15	45	3.000000	no	no	no	no	no	no	no	no	no	no
182238670	chr1	191628362	191628427	21	66	3.190476	no	no	no	no	no	no	no	no	no	no

182238679	chr1	191640058	191640540	241	483	2.004149	no	no	no	no	no	no	no	no	no	no
182238680	chr1	191641100	191641140	18	41	2.277778	no	no	no	no	no	no	no	no	no	no
182238681	chr1	191642726	191642767	22	42	1.909091	no	no	no	no	no	no	no	no	no	no
182238682	chr1	191646372	191646409	20	38	1.950000	no	no	no	no	no	no	no	no	no	no
182238684	chr1	191649016	191649129	33	114	3.454545	no	no	no	no	no	no	no	no	no	no
182238702	chr1	191708901	191708932	15	32	2.133333	no	no	no	no	no	no	no	yes	no	no
182238705	chr1	191718596	191718696	28	101	3.551724	no	no	no	no	no	no	no	no	no	no
182238707	chr1	191718685	191718743	31	59	1.903226	no	no	no	no	no	no	no	no	no	no
182238714	chr1	191733919	191733951	17	33	2.000000	no	no	no	no	no	no	no	no	no	no
182238715	chr1	191737029	191737064	15	36	2.400000	no	no	no	no	no	no	no	no	no	no
182238739	chr1	191817263	191817993	366	731	2.002732	no	no	no	no	no	no	no	yes	no	no
182238741	chr1	191819786	191819877	39	92	2.375000	no	no	no	no	no	no	no	no	no	no
182238744	chr1	191820593	191820628	16	36	2.250000	no	no	no	no	no	no	no	no	no	no
182238745	chr1	191822085	191822118	15	34	2.333333	no	no	no	no	no	no	no	yes	no	no
182238764	chr1	191845193	191845234	18	42	2.388889	no	no	yes	no	no	no	no	no	no	yes
182238771	chr1	191866726	191866755	15	30	2.000000	no	no	yes	no	no	no	no	no	no	yes
182238774	chr1	191878034	191878058	13	25	1.923077	no	no	yes	no	no	no	no	no	no	yes
182238775	chr1	191878266	191878297	14	32	2.285714	no	no	yes	no	no	no	no	no	no	yes
182238777	chr1	191886150	191886182	17	33	1.941176	no	no	yes	no	no	no	no	yes	no	yes
182238782	chr1	191916893	191916924	16	32	2.000000	no	no	yes	no	no	no	no	no	no	yes
182238783	chr1	191923361	191923393	17	33	2.058824	no	no	yes	no	no	no	no	no	no	yes
182238791	chr1	191944294	191944327	15	34	2.333333	no	no	yes	no	no	no	no	no	no	yes
182238798	chr1	191961674	191961708	18	35	1.944444	no	no	yes	no	no	no	no	no	no	yes
182238800	chr1	191969442	191969529	23	88	3.826087	no	no	yes	no	no	no	no	yes	yes	yes
182238805	chr1	191999088	191999120	17	33	1.941176	no	no	yes	no	no	no	no	no	no	yes

182238810	chr1	192004016	192004052	17	37	2.176471	no	no	yes	no	no	no	no	no	no	yes
182238814	chr1	192011569	192011631	23	63	2.875000	no	no	no	no	no	yes	no	yes	no	no
182238818	chr1	192020503	192020546	23	44	1.913043	no	no	no	no	no	no	no	no	no	no
182238823	chr1	192030615	192030645	13	31	2.384615	no	no	no	no	no	no	no	no	no	no
182238824	chr1	192036504	192036538	17	35	2.058824	no	no	no	no	no	no	no	no	no	no
182238829	chr1	192055324	192055364	14	41	2.866667	no	no	no	no	no	no	no	no	no	no
182238837	chr1	192083827	192083862	14	36	2.533333	no	no	no	no	no	no	no	no	no	no
182238839	chr1	192084845	192084887	16	43	2.687500	no	no	no	no	no	no	no	no	no	no
182238858	chr1	192122844	192122884	16	41	2.750000	no	no	no	no	no	no	no	yes	no	no
182238859	chr1	192123145	192123183	20	39	1.950000	no	no	no	no	no	no	no	yes	no	no
182238872	chr1	192159478	192159513	14	36	2.571429	no	no	yes	no	no	no	no	yes	no	yes
182238880	chr1	192179079	192179110	16	32	2.062500	no	no	yes	no	no	no	no	no	no	yes
182238883	chr1	192196792	192196818	13	27	2.076923	no	no	yes	no	no	no	no	no	no	yes
182238885	chr1	192197683	192197709	14	27	1.928571	no	no	yes	no	no	no	no	no	no	yes
182238890	chr1	192205905	192205932	13	28	2.153846	no	no	yes	no	no	no	no	no	no	yes
182238905	chr1	192222910	192222976	34	67	2.029412	no	no	yes	no	no	no	no	no	no	yes
182238914	chr1	192257297	192257377	36	81	2.250000	no	no	yes	no	no	no	no	yes	no	yes
182238916	chr1	192257367	192257446	38	80	2.105263	no	no	yes	no	no	no	no	yes	no	yes
182238917	chr1	192260675	192260729	17	55	3.235294	no	no	yes	no	no	no	no	no	no	yes
182238926	chr1	192296571	192296608	19	38	2.157895	no	no	yes	no	no	no	no	no	no	yes
182238930	chr1	192297660	192297689	15	30	2.000000	no	no	yes	no	no	no	no	no	no	yes
182238935	chr1	192315025	192315049	13	25	1.923077	no	no	yes	no	no	no	no	no	no	yes
182238939	chr1	192324649	192324694	23	46	2.000000	no	no	yes	no	no	no	no	no	no	yes
182238940	chr1	192328388	192328429	20	42	2.100000	no	no	yes	no	no	no	no	yes	no	yes
182238941	chr1	192329411	192329452	21	42	2.000000	no	no	yes	no	no	no	no	no	no	yes

182238942	chr1	192331229	192331262	18	34	1.944444	no	no	yes	no	no	no	no	no	no	yes
182238947	chr1	192342648	192342678	16	31	2.000000	no	no	yes	no	no	no	no	no	no	yes
182238948	chr1	192348948	192348974	10	27	2.700000	no	no	yes	no	no	no	no	no	no	yes
182238951	chr1	192351667	192351794	34	128	3.722222	no	no	yes	no	no	no	no	yes	no	yes
182238954	chr1	192357421	192357456	18	36	2.000000	no	no	yes	no	no	no	no	yes	no	yes
182238966	chr1	192387340	192387372	17	33	1.941176	no	no	yes	no	no	yes	no	yes	no	yes
182238970	chr1	192396893	192396930	20	38	1.900000	no	no	yes	no	no	no	no	no	no	yes
182238971	chr1	192399728	192399756	12	29	2.416667	no	no	yes	no	no	no	no	no	no	yes
182238972	chr1	192400077	192400118	21	42	2.000000	no	no	yes	no	no	no	no	yes	no	yes
182238973	chr1	192401197	192401249	26	53	2.038461	no	no	yes	no	no	no	no	no	no	yes
182238977	chr1	192409495	192409531	18	37	2.055556	no	no	yes	no	no	no	no	no	no	yes
182239003	chr1	192488527	192488580	22	54	2.454545	no	no	yes	no	no	no	no	yes	yes	yes
182239031	chr1	192526839	192526867	15	29	1.933333	no	no	yes	no	no	no	no	no	no	yes
182239032	chr1	192536630	192536665	16	36	2.250000	no	no	yes	no	no	no	no	yes	no	yes
182239033	chr1	192545486	192545569	44	84	1.909091	no	no	yes	no	no	no	no	yes	no	yes
182239046	chr1	192565775	192565821	24	47	1.958333	no	no	yes	no	no	no	no	yes	no	yes
182239047	chr1	192565972	192566009	11	38	3.454545	no	no	yes	no	no	no	no	yes	no	yes
182239052	chr1	192591504	192591532	15	29	2.000000	no	no	yes	no	no	no	no	no	no	yes
182239053	chr1	192595344	192595478	16	135	8.277778	no	no	yes	no	no	no	no	yes	yes	yes
182239058	chr1	192605721	192605811	46	91	2.043478	no	no	yes	no	no	no	no	yes	yes	yes
182239068	chr1	192611345	192611376	14	32	2.285714	no	no	yes	no	no	no	no	no	no	yes
182239072	chr1	192626813	192626846	16	34	2.125000	no	no	yes	no	no	no	no	yes	yes	yes
182239074	chr1	192630165	192630207	22	43	1.954545	no	no	yes	no	no	no	no	yes	no	yes
182239075	chr1	192636600	192636633	16	34	2.125000	no	no	yes	no	no	no	no	yes	no	yes
182239076	chr1	192638701	192638762	32	62	1.937500	no	no	yes	no	no	no	no	no	no	yes

182239081	chr1	192641372	192641423	24	52	2.166667	no	no	yes	no	no	no	no	no	no	yes
182239085	chr1	192655723	192655795	29	73	2.517241	no	no	yes	no	no	no	no	no	no	yes
182239086	chr1	192655874	192655913	16	40	2.562500	no	no	yes	no	no	no	no	no	yes	yes
182239093	chr1	192679126	192679170	17	45	2.647059	no	no	yes	no	no	no	no	yes	no	yes
182239098	chr1	192691839	192691871	15	33	2.200000	no	no	yes	no	no	no	no	yes	yes	yes
182239100	chr1	192698494	192698551	21	58	2.761905	no	no	yes	no	no	no	no	yes	no	yes
182239102	chr1	192700446	192700493	17	48	2.941176	no	no	yes	no	no	no	no	no	no	yes
182239105	chr1	192703107	192703135	13	29	2.230769	no	no	yes	no	no	no	no	no	no	yes
182239106	chr1	192704194	192704233	17	40	2.352941	no	no	yes	no	no	no	no	yes	no	yes
182239111	chr1	192710163	192710214	27	52	2.000000	no	no	yes	no	no	no	no	no	no	yes
182239114	chr1	192725375	192725419	18	45	2.555556	no	no	yes	no	no	yes	no	no	no	yes
182239139	chr1	192786726	192786773	21	48	2.285714	no	no	yes	no	no	no	no	no	yes	yes
182239148	chr1	192806213	192806305	48	93	1.958333	no	no	yes	no	no	no	no	yes	no	yes
182239157	chr1	192832250	192832329	37	80	2.162162	no	no	yes	no	no	no	no	yes	no	yes
182239194	chr1	192881945	192881997	21	53	2.571429	no	no	yes	no	no	no	no	no	no	yes
182239211	chr1	192921173	192921223	26	51	1.961538	no	no	yes	no	no	no	no	no	no	yes
182239216	chr1	192944517	192944566	22	50	2.272727	no	no	yes	no	no	no	no	no	no	yes
182239218	chr1	192950599	192950633	18	35	1.944444	no	no	yes	no	no	no	no	no	no	yes
182239219	chr1	192953328	192953376	16	49	3.125000	no	no	yes	no	no	no	no	yes	no	yes
182239220	chr1	192955523	192955553	16	31	1.937500	yes	no	yes	no	no	no	no	no	no	yes
182239229	chr1	192972464	192972505	21	42	2.000000	no	no	no	no	no	no	no	yes	no	no
182239237	chr1	192992290	192992328	21	39	1.952381	no	no	no	no	no	no	no	no	yes	no
182239239	chr1	192995805	192995848	22	44	2.000000	no	no	no	no	no	no	no	no	no	no
182239245	chr1	193012485	193012602	59	118	2.000000	no	yes	no	no	no	no	no	no	no	yes
182239252	chr1	193060355	193060380	11	26	2.363636	yes	no	yes	no	no	yes	no	yes	yes	yes

182239254	chr1	193066912	193067113	101	202	2.049505	no	no	yes	no	no	no	no	yes	no	yes
182239256	chr1	193070250	193070283	16	34	2.125000	no	no	yes	no	no	no	no	yes	yes	yes
182239267	chr1	193096034	193096099	26	66	2.500000	no	yes	no	no	no	yes	no	yes	no	yes
182239270	chr1	193105271	193105301	9	31	3.444444	no	no	yes	yes	no	no	yes	yes	no	yes
182239281	chr1	193121408	193121482	40	75	1.925000	yes	no	no	no	no	no	no	yes	no	no
182239292	chr1	193148607	193148639	16	33	2.125000	no	no	yes	no	no	no	no	no	no	yes
182239294	chr1	193154118	193154184	35	67	1.942857	no	no	yes	no	no	yes	no	no	no	yes
182239299	chr1	193178296	193178331	19	36	1.947368	no	no	yes	no	no	yes	no	no	no	yes
182239300	chr1	193179666	193179696	16	31	1.937500	no	yes	yes	no	no	no	no	no	no	yes
182239301	chr1	193193718	193193758	19	41	2.263158	no	no	yes	no	no	no	no	no	no	yes
182239306	chr1	193205099	193205146	21	48	2.380952	no	no	yes	no	no	no	no	no	no	yes
182239308	chr1	193209941	193209978	20	38	1.900000	no	no	yes	no	no	no	no	no	yes	yes
182239311	chr1	193224343	193224697	29	355	12.516129	no	no	yes	no	no	no	no	yes	no	yes
182239333	chr1	193270809	193270843	16	35	2.187500	no	no	no	no	no	no	no	no	yes	no
182239337	chr1	193282734	193282760	14	27	1.928571	no	no	no	no	no	no	no	no	no	no
182239338	chr1	193287720	193287752	17	33	1.941176	no	no	no	no	no	no	no	no	no	no
182239343	chr1	193301241	193301287	20	47	2.400000	no	no	no	no	no	no	no	no	no	no
182239345	chr1	193302458	193302490	17	33	1.941176	no	no	no	no	no	no	no	no	no	no
182239346	chr1	193303038	193303134	32	97	3.031250	no	no	no	no	no	no	no	no	no	no
182239356	chr1	193342988	193343040	22	53	2.409091	no	no	yes	no	no	no	no	no	yes	yes
182239365	chr1	193352794	193352863	35	70	2.000000	no	no	yes	no	no	no	no	no	no	yes
182239366	chr1	193353910	193353941	15	32	2.133333	no	no	yes	no	no	no	no	no	no	yes
182239369	chr1	193358214	193358300	36	87	2.472222	no	no	yes	no	no	no	no	yes	no	yes
182239389	chr1	193404034	193404079	17	46	2.941176	no	no	no	no	no	no	no	no	no	no
182239399	chr1	193434859	193434890	13	32	2.461539	no	no	no	no	no	no	no	yes	no	no

182239411	chr1	193444582	193444639	26	58	2.230769	no	no	no	no	no	no	no	no	no	no
182239423	chr1	193478850	193478881	14	32	2.285714	no	no	yes	no	no	no	no	no	no	yes
182239425	chr1	193498607	193498702	48	96	2.000000	no	no	yes	no	no	no	no	no	no	yes
182239427	chr1	193507984	193508024	18	41	2.277778	no	no	yes	no	no	no	no	no	yes	yes
182239439	chr1	193545497	193545560	32	64	2.000000	no	no	yes	no	no	no	no	no	no	yes
182239452	chr1	193569402	193569457	27	56	2.074074	no	no	yes	no	no	no	no	yes	no	yes
182239456	chr1	193588101	193588293	28	193	7.035714	no	no	yes	no	no	no	no	yes	no	yes
182239463	chr1	193591591	193591622	17	32	2.000000	no	no	yes	no	no	no	no	no	no	yes
182239467	chr1	193610012	193610045	13	34	2.769231	no	no	yes	no	no	no	no	no	no	yes
182239469	chr1	193612004	193612041	17	38	2.294118	no	no	yes	no	no	no	no	no	no	yes
182239473	chr1	193631717	193631771	29	55	1.931034	no	no	yes	no	no	no	no	no	no	yes
182239478	chr1	193636744	193636781	20	38	1.900000	no	no	yes	no	no	no	no	no	no	yes
182239481	chr1	193646679	193646718	21	40	1.904762	no	no	yes	no	no	no	no	no	no	yes
182239485	chr1	193658172	193658211	18	40	2.222222	no	no	yes	no	no	no	no	no	yes	yes
182239486	chr1	193662262	193662286	11	25	2.272727	no	no	yes	no	no	no	no	yes	yes	yes
182239489	chr1	193674174	193674247	29	74	2.551724	no	no	yes	no	no	no	no	no	no	yes
182239493	chr1	193697577	193697618	16	42	2.625000	no	no	yes	no	no	no	no	yes	no	yes
182239494	chr1	193698356	193698391	19	36	1.947368	no	no	yes	no	no	no	no	no	yes	yes
182239496	chr1	193707616	193707650	17	35	2.117647	no	no	yes	no	no	no	no	yes	yes	yes
182239501	chr1	193750033	193750077	24	45	1.958333	no	no	yes	no	no	no	no	no	no	yes
182239509	chr1	193763140	193763172	17	33	1.941176	no	no	yes	no	no	no	no	yes	yes	yes
182239510	chr1	193764959	193764990	12	32	2.666667	no	no	yes	no	no	no	no	yes	no	yes
182239522	chr1	193816037	193816078	14	42	3.142857	no	no	yes	no	no	no	no	yes	yes	yes
182239530	chr1	193848504	193848543	22	40	1.954545	no	no	yes	no	no	no	no	no	no	yes
182239538	chr1	193862971	193863015	24	45	1.916667	no	no	yes	no	no	no	no	no	no	yes

182239539	chr1	193865664	193865690	13	27	2.076923	no	no	yes	no	no	no	no	yes	no	yes
182239541	chr1	193869511	193869610	26	100	3.814815	no	no	yes	no	no	no	no	no	no	yes
182239548	chr1	193895132	193895160	14	29	2.071429	no	no	yes	no	no	no	no	no	no	yes
182239550	chr1	193902918	193902972	26	55	2.115385	no	no	yes	no	no	no	no	no	no	yes
182239555	chr1	193905170	193905204	15	35	2.333333	no	no	yes	no	no	no	no	no	no	yes
182239558	chr1	193919610	193919661	26	52	2.076923	no	no	yes	no	no	no	no	no	no	yes
182239560	chr1	193934924	193934953	9	30	3.444444	no	no	yes	no	no	no	no	no	no	yes
182239581	chr1	193954634	193954667	11	34	3.083333	no	no	yes	no	no	no	no	no	no	yes
182239582	chr1	193964174	193964220	16	47	2.833333	no	no	yes	no	no	no	no	no	no	yes
182239586	chr1	193966284	193966335	26	52	2.000000	no	no	yes	no	no	no	no	no	no	yes
182239587	chr1	193969922	193969955	17	34	2.058824	no	no	yes	no	no	no	no	no	yes	yes
182239591	chr1	193978256	193978286	11	31	2.750000	no	no	yes	no	no	no	no	no	no	yes
182239595	chr1	193985918	193986083	82	166	2.024390	no	no	yes	no	no	no	no	no	no	yes
182239602	chr1	194006047	194006086	19	40	2.105263	no	no	yes	no	no	no	no	no	no	yes
182239619	chr1	194063535	194063592	31	58	1.935484	no	no	yes	no	no	no	no	no	no	yes
182239651	chr1	194142208	194142259	27	52	1.925926	no	no	yes	no	no	no	no	no	no	yes
182239682	chr1	194232793	194232830	20	38	1.900000	no	no	no	no	no	no	no	no	no	no
182239683	chr1	194232817	194232851	16	35	2.187500	no	no	no	no	no	no	no	no	no	no
182239684	chr1	194238510	194238560	22	51	2.363636	no	no	no	no	no	no	no	no	no	no
182239690	chr1	194240163	194240267	50	105	2.140000	no	no	no	no	no	no	no	no	no	no
182239697	chr1	194262415	194262454	17	40	2.352941	no	no	no	no	no	no	no	no	no	no
182239698	chr1	194266154	194266183	9	30	3.200000	no	no	no	no	no	no	no	no	no	no
182239705	chr1	194277079	194277125	25	47	1.960000	no	no	no	no	no	no	no	no	no	no
182239712	chr1	194280412	194280452	18	41	2.333333	no	no	no	no	no	no	no	yes	no	no
182239714	chr1	194284317	194284349	16	33	2.062500	no	no	no	no	no	no	no	no	no	no

182239716	chr1	194291038	194291086	25	49	2.000000	no	no	no	no	no	no	no	yes	no	no
182239720	chr1	194292758	194292799	20	42	2.200000	no	no	no	no	no	no	no	no	no	no
182239724	chr1	194303499	194303958	37	460	12.309524	no	no	no	no	no	no	no	no	no	no
182239730	chr1	194331209	194331259	15	51	3.400000	no	no	no	no	no	no	no	no	no	no
182239737	chr1	194334678	194334727	26	50	1.923077	no	no	no	no	no	no	no	no	no	no
182239740	chr1	194339026	194339055	16	30	1.937500	no	no	no	no	no	no	no	no	no	no
182239750	chr1	194372756	194372855	51	100	2.019608	no	no	no	no	no	no	no	yes	no	no
182239751	chr1	194378124	194378154	9	31	3.444444	no	no	no	no	no	no	no	no	no	no
182239754	chr1	194378428	194378457	12	30	2.500000	no	no	no	no	no	no	no	no	no	no
182239757	chr1	194380279	194380326	24	48	2.000000	no	no	no	no	no	no	no	no	no	no
182239759	chr1	194380737	194380771	18	35	2.000000	no	no	no	no	no	no	no	no	no	no
182239783	chr1	194412018	194412050	17	33	1.941176	no	no	no	no	no	no	no	no	no	no
182239796	chr1	194442044	194442078	17	35	2.117647	no	no	no	no	no	no	no	no	no	no
182239798	chr1	194449788	194449875	44	88	2.000000	no	no	no	no	no	no	no	no	no	no
182239802	chr1	194473319	194473361	22	43	1.954545	no	no	no	no	no	no	no	no	no	no
182239803	chr1	194478716	194478795	40	80	2.050000	no	no	no	no	no	no	no	no	no	no
182239810	chr1	194481415	194481468	25	54	2.160000	no	no	no	no	no	no	no	no	no	no
182239813	chr1	194482309	194482336	14	28	2.000000	no	no	no	no	no	no	no	no	no	no
182239817	chr1	194491553	194491583	15	31	2.066667	no	no	no	no	no	no	no	no	no	no
182239844	chr1	194530465	194530584	57	120	2.105263	no	no	no	no	no	no	no	no	no	no
182239857	chr1	194552627	194552667	18	41	2.277778	no	no	no	no	no	no	no	no	no	no
182239861	chr1	194564309	194564335	14	27	1.928571	no	no	no	no	no	no	no	no	yes	no
182239865	chr1	194569969	194570001	12	33	2.750000	no	no	no	no	no	no	no	no	no	no
182239867	chr1	194570042	194570123	42	82	1.952381	no	no	no	no	no	no	no	no	no	no
182239883	chr1	194593800	194593864	20	65	3.238095	no	no	no	no	no	no	no	no	no	no

182239884	chr1	194596269	194596298	15	30	2.000000	no	no	no	no	no	no	no	no	no	no
182239910	chr1	194629898	194629956	24	59	2.541667	no	no	no	no	no	no	no	yes	no	no
182239927	chr1	194688925	194688952	14	28	2.000000	no	no	no	no	no	no	no	no	no	no
182239929	chr1	194698084	194698117	18	34	1.944444	no	no	no	no	no	no	no	no	no	no
182239932	chr1	194704766	194704805	21	40	1.952381	no	no	no	no	no	no	no	no	no	no
182239936	chr1	194715506	194715541	17	36	2.117647	no	no	no	no	no	no	no	no	no	no
182239938	chr1	194729626	194729668	23	43	1.956522	no	no	no	no	no	no	no	no	no	no
182239940	chr1	194733213	194733269	27	57	2.111111	no	no	no	no	no	no	no	yes	no	no
182239941	chr1	194733557	194733600	22	44	2.000000	no	no	no	no	no	no	no	no	no	no
182239947	chr1	194756025	194756078	18	54	3.166667	no	no	no	no	no	no	no	yes	no	no
182239950	chr1	194768529	194768586	29	58	2.000000	no	no	no	no	no	no	no	yes	yes	no
182239971	chr1	194819491	194819521	12	31	2.583333	no	no	yes	no	no	no	no	no	no	yes
182239973	chr1	194823547	194823589	17	43	2.529412	no	no	yes	no	no	no	no	yes	no	yes
182239994	chr1	194882122	194882153	15	32	2.200000	no	no	yes	no	no	no	no	no	no	yes
182239995	chr1	194882568	194882714	75	147	1.986667	no	no	yes	no	no	no	no	no	no	yes
182239999	chr1	194893139	194893184	8	46	6.125000	no	no	yes	no	no	no	no	no	no	yes
182240003	chr1	194897888	194897936	25	49	1.960000	no	no	yes	no	no	no	no	no	no	yes
182240010	chr1	194920214	194920249	11	36	3.363636	no	no	yes	no	no	no	no	no	no	yes
182240013	chr1	194928161	194928249	46	89	1.934783	no	no	yes	no	no	no	no	no	no	yes
182240016	chr1	194957440	194957486	21	47	2.238095	no	no	no	no	no	no	no	no	no	no
182240019	chr1	194962437	194962496	31	60	1.935484	no	no	no	no	no	no	no	no	no	no
182240020	chr1	194970284	194970323	20	40	2.000000	no	no	no	no	no	no	no	no	no	no
182240033	chr1	194990631	194990656	13	26	2.000000	no	no	no	no	no	no	no	yes	yes	no
182240038	chr1	195012749	195012775	14	27	1.928571	no	no	no	no	no	no	no	yes	no	no
182240056	chr1	195035618	195035650	16	33	2.062500	no	no	no	no	no	no	no	no	no	no

182240057	chr1	195039657	195039696	21	40	1.904762	no	no	no	no	no	no	no	no	no	no
182240062	chr1	195044961	195045008	19	48	2.631579	no	no	no	no	no	no	no	no	no	no
182240070	chr1	195058270	195058328	31	59	1.903226	no	no	no	no	no	no	no	no	no	no
182240074	chr1	195066780	195066810	16	31	1.937500	no	no	no	no	no	no	no	no	no	no
182240077	chr1	195074067	195074479	52	413	8.000000	no	no	no	no	no	no	no	no	no	no
182240080	chr1	195075451	195075491	22	41	1.954545	no	no	no	no	no	no	no	yes	no	no
182240083	chr1	195077370	195077407	18	38	2.111111	no	no	no	no	no	no	no	no	no	no
182240088	chr1	195113167	195113193	13	27	2.076923	no	no	no	no	no	no	no	no	no	no
182240089	chr1	195114576	195114608	17	33	1.941176	no	no	no	no	no	no	no	no	no	no
182240092	chr1	195127421	195127477	19	57	3.000000	no	no	no	no	no	no	no	no	no	no
182240093	chr1	195132191	195132245	25	55	2.200000	no	no	no	no	no	no	no	no	no	no
182240096	chr1	195132699	195132740	20	42	2.100000	no	no	no	no	no	no	no	no	no	no
182240110	chr1	195146151	195146198	21	48	2.333333	no	no	no	no	no	no	no	no	no	no
182240111	chr1	195146906	195146945	21	40	1.904762	no	no	no	no	no	no	no	no	no	no
182240114	chr1	195150901	195150974	37	74	2.108108	no	no	no	no	no	no	no	no	no	no
182240121	chr1	195151749	195151799	27	51	1.925926	no	no	no	no	no	no	no	no	no	no
182240125	chr1	195157591	195157630	16	40	2.500000	no	no	no	no	no	no	no	no	no	no
182240126	chr1	195157669	195157707	17	39	2.294118	no	no	no	no	no	no	no	no	no	no
182240127	chr1	195163816	195163850	16	35	2.187500	no	no	no	no	no	no	no	no	no	no
182240128	chr1	195163841	195163872	15	32	2.133333	no	no	no	no	no	no	no	no	no	no
182240129	chr1	195166464	195166505	22	42	2.000000	no	no	no	no	no	no	no	no	no	no
182240135	chr1	195184819	195184851	17	33	1.941176	no	no	no	no	no	no	no	no	no	no
182240136	chr1	195187684	195187712	12	29	2.416667	no	no	no	no	no	no	no	no	no	no
182240140	chr1	195196726	195196754	12	29	2.416667	no	no	no	no	no	no	no	no	no	no
182240146	chr1	195223244	195223331	43	88	2.046512	no	no	no	no	no	no	no	no	no	no

182240152	chr1	195233835	195233860	13	26	2.000000	no	no	no	no	no	no	no	no	no	no
182240156	chr1	195245948	195245981	16	34	2.125000	no	no	no	no	no	no	no	yes	yes	no
182240157	chr1	195246282	195246336	29	55	1.965517	no	no	no	no	no	no	no	yes	no	no
182240163	chr1	195262090	195262119	14	30	2.142857	no	no	no	no	no	no	no	no	no	no
182240175	chr1	195292375	195292410	18	36	2.000000	no	no	no	no	no	no	no	no	no	no
182240176	chr1	195295866	195295958	46	93	2.021739	no	no	no	no	no	no	no	no	no	no
182240178	chr1	195303086	195303151	33	66	2.000000	no	no	no	no	no	no	no	no	no	no
182240180	chr1	195305849	195305899	26	51	2.000000	no	no	no	no	no	no	no	no	no	no
182240183	chr1	195315857	195315893	16	37	2.375000	no	no	no	no	no	no	no	no	no	no
182240188	chr1	195335894	195335935	20	42	2.100000	no	no	no	no	no	no	no	no	yes	no
182240190	chr1	195339352	195339422	16	71	4.437500	no	no	no	no	no	no	no	no	no	no
182240191	chr1	195340391	195340438	24	48	2.041667	no	no	no	no	no	no	no	no	no	no
182240196	chr1	195361385	195361427	16	43	2.687500	no	no	no	no	no	no	no	no	no	no
182240200	chr1	195383099	195383181	26	83	3.192308	no	no	no	no	no	no	no	no	no	no
182240203	chr1	195383202	195383276	26	75	2.884615	no	no	no	no	no	no	no	no	no	no
182240208	chr1	195394657	195394716	31	60	2.032258	no	no	no	no	no	no	no	yes	yes	no
182240213	chr1	195401371	195401426	15	56	3.800000	no	no	no	no	no	no	no	no	no	no
182240223	chr1	195409943	195409981	12	39	3.333333	no	no	no	no	no	no	no	no	no	no
182240225	chr1	195413491	195413605	56	115	2.053571	no	no	no	no	no	no	no	no	no	no
182240231	chr1	195436625	195436670	16	46	2.875000	no	no	no	no	no	no	no	no	no	no
182240234	chr1	195451620	195451667	21	48	2.333333	no	no	no	no	no	no	no	no	no	no
182240237	chr1	195470659	195470695	19	37	2.000000	no	no	no	no	no	no	no	no	no	no
182240240	chr1	195473366	195473407	18	42	2.444444	no	no	no	no	no	no	no	no	no	no
182240242	chr1	195478712	195478749	19	38	2.000000	no	no	no	no	no	no	no	no	no	no
182240247	chr1	195501299	195501331	18	33	1.944444	no	no	no	no	no	no	no	no	no	no

182240250	chr1	195509340	195509368	14	29	2.071429	no	no	no	no	no	no	no	no	no	no
182240251	chr1	195515208	195515253	22	46	2.090909	no	no	no	no	no	no	no	no	no	no
182240252	chr1	195524748	195524778	15	31	2.066667	no	no	no	no	no	no	no	no	no	no
182240260	chr1	195537290	195537322	16	33	2.062500	no	no	no	no	no	no	no	no	yes	no
182240261	chr1	195539899	195540053	78	155	1.987180	no	no	no	no	no	no	no	no	no	no
182240262	chr1	195543191	195543238	22	48	2.181818	no	no	no	no	no	no	no	no	no	no
182240266	chr1	195547874	195547906	12	33	2.692308	no	no	no	no	no	no	no	no	no	no
182240274	chr1	195549686	195549849	91	164	1.846154	no	no	no	no	no	no	no	no	no	no
182240285	chr1	195571552	195572072	25	521	21.920000	no	no	no	no	no	no	no	yes	no	no
182240307	chr1	195604619	195604664	22	46	2.090909	no	no	no	no	no	no	no	yes	no	no
182240308	chr1	195606522	195606555	16	34	2.187500	no	no	no	no	no	no	no	yes	no	no
182240309	chr1	195608308	195608340	17	33	2.000000	no	no	no	no	no	no	no	no	no	no
182240310	chr1	195609882	195609924	18	43	2.388889	no	no	no	no	no	no	no	no	no	no
182240311	chr1	195611265	195611299	18	35	1.944444	no	no	no	no	no	no	no	no	no	no
182240317	chr1	195621082	195621165	31	84	2.647059	no	no	no	no	no	no	no	no	no	no
182240320	chr1	195626081	195626134	28	54	1.928571	no	no	no	no	no	no	no	no	no	no
182240325	chr1	195634564	195634594	16	31	1.937500	no	no	no	no	no	no	no	no	no	no
182240326	chr1	195635916	195635949	15	34	2.333333	no	no	no	no	no	no	no	no	no	no
182240327	chr1	195638009	195638043	17	35	2.058824	no	no	no	no	no	no	no	no	no	no
182240337	chr1	195662129	195662163	15	35	2.333333	no	no	no	no	no	no	no	no	no	no
182240340	chr1	195680921	195680967	25	47	1.920000	no	no	no	no	no	no	no	no	no	no
182240341	chr1	195685156	195685180	13	25	1.923077	no	no	no	no	no	no	no	no	no	no
182240352	chr1	195720971	195721277	152	307	2.059211	no	no	no	no	no	no	no	yes	no	no
182240364	chr1	195729873	195729951	26	79	3.038461	no	no	no	no	no	no	no	no	no	no
182240365	chr1	195730892	195730927	17	36	2.176471	no	no	no	no	no	no	no	no	no	no

182240376	chr1	195765733	195765767	16	35	2.187500	no	no	no	no	no	no	no	no	no	no
182240379	chr1	195773271	195773299	14	29	2.071429	no	no	no	no	no	no	no	no	no	no
182240380	chr1	195776940	195776976	19	37	2.000000	no	no	no	no	no	no	no	no	no	no
182240382	chr1	195780010	195780042	16	33	2.125000	no	no	no	no	no	no	no	no	no	no
182240383	chr1	195781376	195781406	15	31	2.133333	no	no	no	no	no	no	no	no	no	no
182240384	chr1	195783226	195783265	18	40	2.222222	no	no	no	no	no	no	no	no	no	no
182240399	chr1	195816141	195816182	19	42	2.210526	no	no	no	no	no	no	no	no	no	no
182240407	chr1	195825810	195825841	15	32	2.133333	no	no	no	no	no	no	no	no	no	no
182240408	chr1	195827622	195827680	27	59	2.185185	no	no	no	no	no	no	no	no	no	no
182240411	chr1	195832440	195832474	18	35	1.944444	no	no	no	no	no	no	no	yes	no	no
182240421	chr1	195832947	195832975	15	29	1.933333	no	no	no	no	no	no	no	yes	no	no
182240427	chr1	195839183	195839215	14	33	2.428571	no	no	no	no	no	no	no	no	no	no
182240433	chr1	195842167	195842203	16	37	2.312500	no	no	no	no	no	no	no	no	no	no
182240436	chr1	195847452	195847502	21	51	2.476191	no	no	no	no	no	no	no	yes	no	no
182240441	chr1	195872204	195872240	19	37	1.947368	no	no	no	no	no	no	no	no	no	no
182240443	chr1	195883293	195883336	22	44	2.000000	no	no	no	no	no	no	no	no	no	no
182240451	chr1	195908083	195908107	12	25	2.083333	no	no	no	no	no	no	no	yes	no	no
182240455	chr1	195911114	195911241	20	128	6.272727	no	no	no	no	no	no	no	no	no	no
182240467	chr1	195933218	195933288	36	71	1.972222	no	no	no	no	no	no	no	no	no	no
182240474	chr1	195970131	195970164	15	34	2.266667	no	no	no	no	no	no	no	no	no	no
182240484	chr1	196008208	196008244	19	37	1.947368	no	no	no	no	no	no	no	yes	no	no
182240488	chr1	196019851	196019963	60	113	1.883333	no	no	no	no	no	no	no	no	no	no
182240491	chr1	196019957	196020055	13	99	7.666667	no	no	no	no	no	no	no	no	no	no
182240493	chr1	196033598	196033637	21	40	1.904762	no	no	no	no	no	no	no	no	no	no
182240498	chr1	196049415	196049450	19	36	2.000000	no	no	yes	no	no	no	no	no	no	yes

182240499	chr1	196059109	196059141	16	33	2.125000	no	no	yes	no	no	no	no	yes	no	yes
182240501	chr1	196075531	196075578	23	48	2.130435	no	no	no	no	no	no	no	no	no	no
182240512	chr1	196123396	196123431	14	36	2.642857	no	no	no	no	no	no	no	no	no	no
182240514	chr1	196131062	196131095	14	34	2.428571	no	no	no	no	no	no	no	no	no	no
182240518	chr1	196142746	196142778	14	33	2.357143	no	no	no	no	no	no	no	no	yes	no
182240524	chr1	196147718	196147770	26	53	2.038461	no	no	no	no	no	no	no	yes	no	no
182240529	chr1	196150702	196150747	15	46	3.000000	no	no	no	no	no	no	no	no	no	no
182240535	chr1	196175335	196175376	14	42	3.071429	no	no	no	no	no	no	no	no	no	no
182240555	chr1	196214876	196214904	15	29	1.933333	no	no	no	no	no	no	no	no	no	no
182240556	chr1	196215964	196215997	16	34	2.125000	no	no	no	no	no	no	no	no	no	no
182240560	chr1	196237055	196237101	23	47	2.043478	no	no	yes	no	no	no	no	no	no	yes
182240569	chr1	196270712	196270758	16	47	2.937500	no	no	yes	no	no	no	no	no	no	yes
182240577	chr1	196286915	196286956	15	42	3.000000	no	no	yes	no	no	no	no	no	no	yes
182240587	chr1	196295699	196295811	58	113	1.948276	no	no	yes	no	no	no	no	yes	no	yes
182240589	chr1	196310240	196310274	17	35	2.058824	no	no	yes	no	no	no	no	no	no	yes
182240591	chr1	196315388	196315414	14	27	1.928571	no	no	yes	no	no	no	no	yes	no	yes
182240592	chr1	196325731	196325759	15	29	1.933333	no	no	yes	no	no	no	no	no	no	yes
182240600	chr1	196329983	196330078	50	96	1.960000	no	no	yes	no	no	no	no	no	no	yes
182240616	chr1	196373461	196373543	42	83	1.976190	no	no	yes	no	no	no	no	yes	no	yes
182240624	chr1	196410884	196410935	9	52	5.888889	no	no	yes	no	no	no	no	yes	no	yes
182240626	chr1	196416577	196416605	12	29	2.416667	no	no	yes	no	no	no	no	no	no	yes
182240630	chr1	196422438	196422470	16	33	2.125000	no	no	yes	no	no	no	no	no	no	yes
182240633	chr1	196427211	196427265	28	55	1.964286	no	no	yes	no	no	no	no	no	no	yes
182240646	chr1	196451624	196451674	24	51	2.125000	no	no	yes	no	no	no	no	no	no	yes
182240647	chr1	196452422	196452454	18	33	1.944444	no	no	yes	no	no	no	no	no	no	yes

182240650	chr1	196458188	196458223	15	36	2.466667	no	no	yes	no	no	no	no	no	no	yes
182240651	chr1	196461358	196461411	26	54	2.076923	no	no	yes	no	no	no	no	yes	no	yes
182240661	chr1	196492096	196492171	37	76	2.054054	no	no	yes	no	no	no	no	no	no	yes
182240676	chr1	196532585	196532620	17	36	2.176471	no	no	yes	no	no	no	no	no	no	yes
182240679	chr1	196541982	196542032	26	51	1.961538	no	no	yes	no	no	no	no	no	no	yes
182240681	chr1	196548103	196548139	17	37	2.235294	no	no	yes	no	no	no	no	no	no	yes
182240683	chr1	196549405	196549449	22	45	2.045455	no	no	yes	no	no	no	no	no	no	yes
182240685	chr1	196552228	196552261	17	34	2.000000	no	no	yes	no	no	no	no	no	no	yes
182240686	chr1	196558911	196558955	20	45	2.300000	no	no	yes	no	no	no	no	no	no	yes
182240693	chr1	196569221	196569248	13	28	2.153846	no	no	yes	no	no	no	no	yes	no	yes
182240697	chr1	196573636	196573701	34	66	1.970588	no	no	yes	no	no	no	no	no	no	yes
182240701	chr1	196580220	196580254	17	35	2.117647	no	no	yes	no	no	no	no	no	no	yes
182240702	chr1	196582873	196582932	23	60	2.608696	yes	no	yes	no	no	no	no	no	no	yes
182240708	chr1	196592478	196592559	34	82	2.411765	no	no	yes	no	no	no	no	yes	no	yes
182240722	chr1	196595239	196595291	18	53	2.947368	no	no	yes	no	no	no	no	no	no	yes
182240726	chr1	196598258	196598309	25	52	2.080000	no	no	yes	no	no	no	no	no	no	yes
182240728	chr1	196604694	196604751	29	58	2.068965	no	no	yes	no	no	no	no	no	no	yes
182240732	chr1	196616890	196616919	14	30	2.214286	no	no	no	no	no	no	no	no	no	no
182240735	chr1	196627826	196627897	30	72	2.433333	no	no	no	no	no	no	no	no	no	no
182240738	chr1	196629820	196629874	28	55	2.035714	no	no	no	no	no	no	no	no	no	no
182240746	chr1	196654549	196654594	24	46	1.958333	no	no	yes	no	no	no	no	yes	yes	yes
182240751	chr1	196657980	196658048	33	69	2.090909	no	no	yes	no	no	no	no	yes	no	yes
182240756	chr1	196665353	196665385	17	33	1.941176	no	no	yes	no	no	no	no	no	no	yes
182240761	chr1	196682749	196682785	16	37	2.312500	no	no	yes	no	no	no	no	no	no	yes
182240762	chr1	196692260	196692297	19	38	2.000000	no	no	yes	no	no	no	no	no	no	yes

182240767	chr1	196702258	196702345	43	88	2.069767	no	no	yes	no	no	yes	no	yes	no	yes
182240773	chr1	196714471	196714543	34	73	2.147059	no	no	yes	no	no	no	no	no	no	yes
182240779	chr1	196728958	196728992	12	35	2.916667	no	no	yes	no	no	no	no	no	no	yes
182240830	chr1	196877723	196877758	18	36	2.000000	no	no	yes	no	no	no	no	yes	no	yes
182240833	chr1	196885425	196885597	30	173	5.848485	no	no	no	no	no	yes	no	no	no	no
182240835	chr1	196889069	196889105	18	37	2.055556	no	no	yes	no	no	no	no	yes	no	yes
182240840	chr1	196907090	196907143	16	54	3.375000	no	no	yes	no	no	no	no	no	no	yes
182240852	chr1	196922688	196922731	13	44	3.384615	no	no	no	no	no	no	no	no	no	no
182240876	chr1	196965860	196965925	28	66	2.464286	no	no	no	no	no	no	no	no	no	no
182240878	chr1	196969696	196969736	19	41	2.210526	yes	no	no	no	no	no	no	no	no	no
182240880	chr1	196978733	196978778	24	46	1.916667	no	no	yes	no	no	no	no	no	yes	yes
182240883	chr1	196984197	196984226	11	30	2.727273	no	no	yes	no	no	no	no	no	no	yes
182240887	chr1	196996849	196996900	7	52	8.000000	no	no	yes	no	no	no	no	no	no	yes
182240894	chr1	197006260	197006298	20	39	1.950000	no	no	yes	no	no	no	no	no	no	yes
182240900	chr1	197007832	197007870	20	39	2.000000	no	no	yes	no	no	no	no	yes	no	yes
182240901	chr1	197007900	197007938	20	39	1.950000	no	no	yes	no	no	no	no	yes	no	yes
182240905	chr1	197010518	197010558	20	41	2.050000	no	no	no	no	no	yes	no	yes	no	no
182240910	chr1	197017829	197017859	15	31	2.066667	no	no	no	no	no	no	no	yes	yes	no
182240918	chr1	197029790	197029862	39	73	1.974359	no	no	no	no	no	no	no	yes	yes	no
182240923	chr1	197040475	197040509	18	35	1.944444	no	no	yes	no	no	no	no	no	no	yes
182240929	chr1	197059709	197059750	21	42	2.000000	no	no	yes	no	no	no	no	no	no	yes
182240930	chr1	197061701	197061742	22	42	1.954545	no	no	yes	no	no	no	no	no	no	yes
182240939	chr1	197089682	197089732	25	51	2.040000	no	no	yes	no	no	no	no	yes	no	yes
182240947	chr1	197144915	197144946	17	32	1.941176	no	no	yes	no	no	no	no	yes	yes	yes
182240949	chr1	197145918	197146056	73	139	1.958904	no	no	yes	no	no	no	no	yes	yes	yes

182240951	chr1	197154342	197154391	25	50	2.000000	no	yes	no	no	no	no	no	no	no	yes
182240952	chr1	197161442	197161476	13	35	2.714286	no	no	yes	no	no	no	no	no	no	yes
182240953	chr1	197162572	197162600	11	29	2.636364	no	no	yes	no	no	no	no	no	no	yes
182240954	chr1	197164658	197164990	34	333	10.270270	no	no	yes	no	no	no	no	yes	no	yes
182240972	chr1	197224102	197224162	30	61	2.033333	no	no	no	no	no	yes	no	yes	no	no
182240981	chr1	197260172	197260211	15	40	2.666667	no	no	no	no	no	no	no	no	no	no
182240982	chr1	197260329	197260380	25	52	2.080000	no	no	no	no	no	no	no	no	no	no
182240986	chr1	197270825	197270873	25	49	2.040000	no	no	yes	no	no	no	no	no	yes	yes
182240987	chr1	197276184	197276228	15	45	3.000000	no	no	yes	no	no	no	no	no	no	yes
182240989	chr1	197283642	197283672	16	31	1.937500	no	no	yes	no	no	no	no	no	no	yes
182240996	chr1	197290841	197290883	22	43	2.000000	no	no	yes	no	no	no	no	no	no	yes
182241006	chr1	197308994	197309027	17	34	2.000000	no	no	yes	no	no	no	no	yes	no	yes
182241010	chr1	197312711	197312963	102	253	2.495238	no	no	yes	no	no	no	no	no	no	yes
182241021	chr1	197341168	197341222	19	55	3.000000	no	no	yes	no	no	no	no	no	no	yes
182241032	chr1	197364056	197364112	29	57	1.965517	no	yes	yes	no	no	yes	no	no	no	yes
182241034	chr1	197365581	197365624	15	44	2.933333	no	no	yes	no	no	no	no	no	no	yes
182241038	chr1	197381117	197381158	20	42	2.100000	no	no	yes	no	no	no	no	no	no	yes
182241040	chr1	197385396	197385436	21	41	1.952381	no	no	yes	no	no	no	no	no	no	yes
182241043	chr1	197387267	197387356	37	90	2.421053	no	no	yes	no	no	no	no	no	no	yes
182241049	chr1	197400669	197400747	36	79	2.222222	no	no	yes	no	no	no	no	yes	no	yes
182241059	chr1	197408233	197408348	58	116	2.000000	no	no	yes	no	no	no	no	no	no	yes
182241061	chr1	197414256	197414287	14	32	2.357143	no	no	yes	no	no	no	no	yes	no	yes
182241070	chr1	197428889	197428921	15	33	2.200000	no	no	yes	no	no	no	no	no	no	yes
182241087	chr1	197485596	197485631	17	36	2.117647	no	no	no	no	no	no	no	yes	no	no
182241095	chr1	197501991	197502035	18	45	2.473684	no	no	no	no	no	no	no	no	no	no

182241103	chr1	197503110	197503160	24	51	2.125000	no	no	no	no	no	no	no	no	no	no
182241118	chr1	197553120	197553170	14	51	3.600000	no	no	yes	no	no	no	no	no	no	yes
182241124	chr1	197566973	197567013	13	41	3.230769	no	no	yes	no	no	no	no	yes	no	yes
182241134	chr1	197593011	197593054	20	44	2.200000	no	no	yes	no	no	no	no	no	no	yes
182241139	chr1	197600678	197600737	20	60	2.913043	no	no	yes	no	no	no	no	no	no	yes
182241140	chr1	197605218	197605304	45	87	1.977778	no	no	yes	no	no	yes	no	no	no	yes
182241142	chr1	197619630	197619662	17	33	1.941176	no	no	yes	no	no	no	no	yes	no	yes
182241143	chr1	197621276	197621317	19	42	2.210526	no	no	yes	no	no	no	no	yes	no	yes
182241145	chr1	197638834	197638875	20	42	2.100000	no	no	yes	no	no	no	no	no	no	yes
182241146	chr1	197641355	197641395	22	41	1.909091	no	no	yes	no	no	no	no	no	no	yes
182241150	chr1	197662077	197662127	24	51	2.125000	no	no	yes	no	no	no	no	no	no	yes
182241155	chr1	197686585	197686610	12	26	2.166667	yes	no	yes	no	no	no	no	no	yes	yes
182241156	chr1	197695530	197695599	35	70	2.000000	no	no	yes	no	no	no	no	no	no	yes
182241157	chr1	197696035	197696089	26	55	2.115385	no	no	yes	no	no	no	no	no	yes	yes
182241158	chr1	197701565	197701601	17	37	2.176471	no	no	yes	no	no	no	no	no	no	yes
182241164	chr1	197707572	197707708	73	137	1.945205	no	no	yes	no	no	no	no	no	no	yes
182241180	chr1	197755353	197755401	24	49	2.041667	no	no	yes	no	no	no	no	no	no	yes
182241186	chr1	197769565	197769628	32	64	2.000000	no	no	yes	no	no	no	no	yes	yes	yes
182241190	chr1	197777777	197777813	19	37	1.947368	no	no	no	no	no	no	no	yes	no	no
182241231	chr1	197853520	197853557	15	38	2.533333	no	no	no	no	no	no	no	no	no	no
182241243	chr1	197879156	197879199	24	44	1.916667	no	no	no	no	no	no	no	yes	no	no
182241256	chr1	197901221	197901257	17	37	2.176471	no	no	no	no	no	no	no	no	yes	no
182241258	chr1	197914481	197914511	13	31	2.384615	no	no	yes	no	no	no	no	yes	no	yes
182241260	chr1	197921802	197921838	19	37	2.000000	no	no	yes	no	no	no	no	yes	yes	yes
182241262	chr1	197924244	197924271	13	28	2.153846	no	no	yes	no	no	no	no	yes	no	yes

182241263	chr1	197926209	197926243	17	35	2.058824	no	no	yes	no	no	no	no	yes	no	yes
182241274	chr1	197950680	197950761	41	82	2.000000	no	no	no	no	no	no	no	no	no	no
182241276	chr1	197961984	197962016	14	33	2.428571	no	no	no	no	no	no	no	yes	no	no
182241279	chr1	197962505	197962632	70	128	1.900000	no	no	no	no	no	no	no	no	no	no
182241287	chr1	197980064	197980174	21	111	5.285714	no	no	no	no	no	no	no	no	no	no
182241290	chr1	197992337	197992381	22	45	2.136364	no	no	no	no	no	no	no	no	no	no
182241301	chr1	198013305	198013337	17	33	1.941176	no	no	no	no	no	no	no	no	no	no
182241302	chr1	198018432	198018492	28	61	2.178571	no	no	no	no	no	no	no	yes	no	no
182241322	chr1	198084419	198084468	25	50	2.000000	no	no	no	no	no	no	no	yes	yes	no
182241324	chr1	198086671	198086706	16	36	2.250000	no	no	no	no	no	no	no	no	no	no
182241327	chr1	198125226	198125255	14	30	2.142857	no	no	no	no	no	no	no	no	no	no
182241328	chr1	198125608	198125644	11	37	3.363636	no	no	no	no	no	no	no	no	yes	no
182241333	chr1	198143072	198143119	21	48	2.285714	no	no	no	no	no	no	no	yes	no	no
182241336	chr1	198144362	198144527	91	166	1.824176	no	no	no	no	no	no	no	no	yes	no
182241341	chr1	198160341	198160387	23	47	2.043478	no	no	yes	no	no	no	no	yes	yes	yes
182241344	chr1	198176792	198176837	23	46	2.043478	no	no	yes	no	no	no	no	no	yes	yes
182241355	chr1	198209047	198209122	30	76	2.566667	no	no	yes	no	no	no	no	no	no	yes
182241359	chr1	198221922	198221953	15	32	2.133333	no	no	yes	no	no	no	no	no	no	yes
182241372	chr1	198252623	198252749	55	127	2.385965	no	no	yes	no	no	no	no	no	no	yes
182241374	chr1	198255484	198255519	16	36	2.250000	no	no	yes	no	no	no	no	yes	no	yes
182241377	chr1	198265797	198265884	46	88	1.913043	no	no	yes	no	no	no	no	yes	yes	yes
182241382	chr1	198287085	198287122	12	38	3.076923	no	no	yes	no	no	no	no	no	no	yes
182241384	chr1	198293840	198293889	25	50	2.000000	no	no	yes	no	no	no	no	yes	no	yes
182241386	chr1	198297291	198297327	19	37	2.052632	no	no	yes	no	no	no	no	no	no	yes
182241387	chr1	198321831	198321873	22	43	2.045455	no	yes	no	no	no	no	no	no	no	yes

182241388	chr1	198323861	198323940	40	80	2.000000	no	no	no	no	no	no	no	no	no	no
182241395	chr1	198354713	198354840	63	128	2.031746	no	no	no	no	no	no	no	no	no	no
182241397	chr1	198366913	198366941	14	29	2.071429	no	no	no	no	no	no	no	no	no	no
182241399	chr1	198368208	198368315	41	108	2.634146	no	no	no	no	no	no	no	no	no	no
182241402	chr1	198376829	198376854	13	26	2.000000	no	no	no	no	no	no	no	no	no	no
182241404	chr1	198382251	198382281	16	31	1.937500	no	no	no	no	no	no	no	no	yes	no
182241406	chr1	198392648	198392742	43	95	2.209302	no	no	no	no	no	no	no	yes	no	no
182241413	chr1	198397862	198397989	64	128	2.000000	no	no	no	no	no	no	no	yes	no	no
182241421	chr1	198418425	198418453	14	29	2.071429	no	no	no	no	no	no	no	no	no	no
182241424	chr1	198438732	198438761	15	30	2.000000	no	no	no	no	no	no	no	no	no	no
182241426	chr1	198440810	198440872	24	63	2.555556	no	no	no	no	no	no	no	yes	no	no
182241433	chr1	198458654	198458735	29	82	2.827586	no	no	yes	no	no	no	no	no	no	yes
182241437	chr1	198468265	198468305	18	41	2.388889	no	no	yes	no	no	no	no	yes	no	yes
182241447	chr1	198492630	198492809	81	180	2.246914	no	no	yes	no	no	no	no	yes	no	yes
182241449	chr1	198495833	198495870	20	38	1.950000	no	no	yes	no	no	no	no	yes	yes	yes
182241450	chr1	198497275	198497338	32	64	2.000000	no	no	yes	no	no	no	no	no	yes	yes
182241455	chr1	198508142	198508389	64	248	3.867647	no	no	yes	no	no	no	no	no	no	yes
182241459	chr1	198516990	198517045	25	56	2.240000	no	no	yes	no	no	no	no	no	no	yes
182241465	chr1	198542411	198542451	20	41	2.150000	no	no	no	no	no	no	no	no	yes	no
182241470	chr1	198555871	198555984	18	114	6.300000	no	no	no	no	no	no	no	no	no	no
182241485	chr1	198583575	198583646	30	72	2.483871	no	no	no	no	no	no	no	no	no	no
182241490	chr1	198609375	198609434	29	60	2.068965	no	no	no	no	no	no	no	no	no	no
182241501	chr1	198631491	198631531	18	41	2.277778	no	no	no	no	no	no	no	no	no	no
182241515	chr1	198674380	198674427	16	48	3.000000	no	no	yes	no	no	no	no	no	no	yes
182241524	chr1	198694755	198694816	30	62	2.100000	no	yes	yes	no	no	no	no	yes	no	yes

182241525	chr1	198696484	198696528	22	45	2.136364	yes	no	yes	no	no	no	no	yes	yes	yes
182241529	chr1	198707580	198707610	16	31	1.937500	no	no	yes	no	no	yes	no	no	no	yes
182241530	chr1	198709587	198709628	22	42	1.954545	no	no	yes	no	no	no	no	no	no	yes
182241535	chr1	198725444	198725472	14	29	2.071429	no	no	yes	no	no	no	no	yes	no	yes
182241543	chr1	198735462	198735497	18	36	2.055556	no	no	yes	no	no	yes	no	no	no	yes
182241545	chr1	198742032	198742067	17	36	2.176471	no	no	yes	no	no	no	no	yes	yes	yes
182241547	chr1	198743579	198743618	20	40	2.000000	no	no	yes	no	no	no	no	no	no	yes
182241556	chr1	198775536	198775560	11	25	2.272727	no	no	no	no	no	no	no	no	no	no
182241558	chr1	198781122	198781183	27	62	2.296296	no	no	yes	no	no	no	no	no	no	yes
182241561	chr1	198785332	198785402	35	71	2.142857	no	no	yes	no	no	no	no	no	no	yes
182241564	chr1	198785407	198785495	47	89	1.957447	no	no	yes	no	no	no	no	no	no	yes
182241572	chr1	198841424	198841465	22	42	1.954545	no	no	yes	no	no	no	no	no	yes	yes
182241573	chr1	198841816	198842125	29	310	10.793103	no	no	yes	no	no	no	no	no	no	yes
182241576	chr1	198844942	198844989	20	48	2.400000	no	no	yes	no	no	no	no	yes	no	yes
182241584	chr1	198863505	198863534	9	30	3.300000	no	no	yes	no	no	no	no	yes	no	yes
182241590	chr1	198868710	198868811	23	102	4.565217	no	no	yes	no	no	no	no	no	no	yes
182241600	chr1	198876254	198876293	21	40	1.952381	no	no	yes	no	no	no	no	yes	no	yes
182241606	chr1	198887087	198887126	19	40	2.157895	no	no	yes	no	no	no	no	yes	no	yes
182241611	chr1	198953380	198953408	14	29	2.071429	no	no	no	no	no	no	no	no	no	no
182241617	chr1	198984608	198984659	25	52	2.120000	no	no	yes	no	no	no	no	no	no	yes
182241618	chr1	198993413	198993442	16	30	1.937500	no	no	no	no	no	no	no	no	no	no
182241625	chr1	198996858	198996989	42	132	3.238095	no	no	no	no	no	no	no	no	no	no
182241637	chr1	199055640	199055761	54	122	2.296296	no	no	yes	no	no	no	no	no	no	yes
182241639	chr1	199058018	199058064	22	47	2.136364	no	no	yes	no	no	no	no	no	yes	yes
182241641	chr1	199062611	199062656	24	46	1.958333	no	no	yes	no	no	no	no	no	no	yes

182241642	chr1	199070603	199070698	46	96	2.086957	no	no	yes	no	no	no	no	no	no	yes
182241652	chr1	199085724	199085786	26	63	2.423077	no	no	no	no	no	no	no	yes	yes	no
182241655	chr1	199085860	199085912	25	53	2.153846	no	no	no	no	no	no	no	yes	no	no
182241658	chr1	199088481	199088540	29	60	2.068965	no	no	no	no	no	no	no	no	no	no
182241677	chr1	199112544	199112586	15	43	3.000000	no	no	no	no	no	no	no	no	no	no
182241689	chr1	199145625	199145653	15	29	1.933333	no	no	no	no	no	no	no	no	no	no
182241692	chr1	199149270	199149314	20	45	2.300000	no	no	yes	no	no	no	no	no	no	yes
182241704	chr1	199165252	199165316	34	65	1.911765	no	no	yes	no	no	no	no	no	yes	yes
182241707	chr1	199170226	199170279	20	54	2.800000	no	no	yes	no	no	no	no	no	yes	yes
182241717	chr1	199205968	199206012	16	45	2.812500	no	no	yes	no	no	no	no	yes	yes	yes
182241719	chr1	199211090	199211123	18	34	1.944444	no	no	yes	no	no	no	no	yes	no	yes
182241723	chr1	199225893	199225929	19	37	1.947368	no	no	yes	no	no	no	no	no	yes	yes
182241724	chr1	199227437	199227488	28	52	1.928571	no	no	yes	no	no	no	no	no	yes	yes
182241750	chr1	199281370	199281495	63	126	2.000000	no	no	yes	no	no	no	no	no	no	yes
182241751	chr1	199292230	199292258	15	29	2.000000	no	no	yes	no	no	no	no	no	yes	yes
182241756	chr1	199307552	199307628	38	77	2.052632	no	no	yes	no	no	no	no	yes	no	yes
182241757	chr1	199308380	199308406	14	27	1.928571	no	no	yes	no	no	no	no	yes	yes	yes
182241774	chr1	199367722	199367772	26	51	1.961538	no	no	yes	no	no	no	no	no	no	yes
182241775	chr1	199371697	199371728	13	32	2.461539	yes	no	yes	no	no	no	no	no	no	yes
182241778	chr1	199394627	199394667	17	41	2.470588	no	no	no	no	no	no	no	no	yes	no
182241780	chr1	199398620	199398658	12	39	3.153846	no	no	no	no	no	no	no	no	no	no
182241784	chr1	199401763	199401798	18	36	2.000000	no	no	no	no	no	no	no	no	no	no
182241798	chr1	199435894	199435941	21	48	2.285714	no	no	no	no	no	no	no	no	no	no
182241801	chr1	199460560	199460605	24	46	1.958333	no	no	no	no	no	no	no	no	no	no
182241807	chr1	199466549	199466656	52	108	2.153846	no	no	no	no	no	no	no	no	no	no

182241809	chr1	199467375	199467408	14	34	2.428571	no	no	no	no	no	no	no	no	no	no
182241829	chr1	199512856	199512901	24	46	1.916667	no	no	no	no	no	no	no	no	no	no
182241831	chr1	199513123	199513190	32	68	2.187500	no	no	no	no	no	no	no	no	no	no
182241835	chr1	199513175	199513218	19	44	2.368421	no	no	no	no	no	no	no	no	no	no
182241836	chr1	199516797	199516830	18	34	1.944444	no	no	no	no	no	no	no	yes	no	no
182241854	chr1	199559089	199559117	15	29	2.000000	no	no	no	no	no	no	no	no	no	no
182241857	chr1	199559547	199559724	87	178	2.183908	no	no	no	no	no	no	no	no	no	no
182241866	chr1	199581765	199581802	7	38	5.428571	no	no	no	no	no	no	no	no	yes	no
182241869	chr1	199595891	199595976	43	86	2.000000	no	no	no	no	no	no	no	no	no	no
182241876	chr1	199625748	199625785	20	38	1.950000	no	no	no	no	no	no	no	no	no	no
182241879	chr1	199642369	199642410	19	42	2.315789	no	no	no	no	no	no	no	no	no	no
182241885	chr1	199655249	199655348	48	100	2.083333	no	no	no	no	no	no	no	no	no	no
182241889	chr1	199675800	199675863	24	64	2.666667	no	no	no	no	no	no	no	yes	no	no
182241891	chr1	199675865	199675932	32	68	2.125000	no	no	no	no	no	no	no	yes	no	no
182241902	chr1	199719654	199719693	20	40	2.050000	no	no	no	no	no	no	no	yes	no	no
182241903	chr1	199719729	199719780	21	52	2.523809	no	no	no	no	no	no	no	yes	no	no
182241905	chr1	199719774	199719830	27	57	2.185185	no	no	no	no	no	no	no	yes	no	no
182241906	chr1	199723366	199723400	18	35	1.944444	no	no	no	no	no	no	no	no	yes	no
182241907	chr1	199727862	199727907	20	46	2.350000	no	no	no	no	no	no	no	yes	no	no
182241909	chr1	199734632	199734685	22	54	2.454545	no	no	no	no	no	no	no	no	yes	no
182241910	chr1	199735766	199735803	19	38	2.052632	no	no	no	no	no	no	no	no	yes	no
182241911	chr1	199738232	199738279	25	48	1.920000	no	no	no	no	no	no	no	yes	no	no
182241912	chr1	199745280	199745339	17	60	3.705882	no	no	no	no	no	no	no	no	no	no
182241915	chr1	199762924	199763104	88	181	2.056818	no	no	no	no	no	no	no	yes	no	no
182241922	chr1	199775617	199775687	31	71	2.290323	no	no	no	no	no	no	no	no	yes	no

182241925	chr1	199782499	199782664	83	166	2.012048	no	no	no	no	no	no	no	yes	no	no
182241928	chr1	199794791	199794907	43	117	2.818182	no	no	no	no	no	no	no	yes	no	no
182241936	chr1	199828055	199828091	19	37	2.000000	no	no	no	no	no	no	no	no	no	no
182241941	chr1	199841626	199841691	28	66	2.379310	no	no	no	no	no	no	no	no	no	no
182241947	chr1	199858045	199858109	31	65	2.129032	no	no	no	no	no	no	no	yes	no	no
182241953	chr1	199860512	199860547	18	36	2.055556	no	no	no	no	no	no	no	no	no	no
182241964	chr1	199880890	199880938	15	49	3.400000	no	no	no	no	no	no	no	yes	no	no
182241967	chr1	199889889	199889934	21	46	2.238095	no	no	no	no	no	no	no	no	no	no
182241974	chr1	199899519	199899667	26	149	5.821429	no	no	no	no	no	no	no	no	no	no
182241993	chr1	199926508	199926573	27	66	2.428571	no	no	no	no	no	no	no	no	no	no
182241995	chr1	199927092	199927130	19	39	2.052632	no	no	no	no	no	no	no	no	no	no
182242019	chr1	199958057	199958099	23	43	1.913043	no	no	no	no	no	no	no	no	no	no
182242040	chr1	199996829	199996870	19	42	2.263158	no	no	no	no	no	no	no	no	no	no
182242047	chr1	200004222	200004258	15	37	2.466667	no	no	no	no	no	no	no	yes	no	no
182242060	chr1	200019119	200019158	20	40	2.000000	no	no	no	no	no	no	no	no	no	no
182242076	chr1	200024840	200024886	18	47	2.611111	no	no	no	no	no	no	no	no	no	no
182242077	chr1	200027327	200027358	15	32	2.200000	yes	no	no	no	no	no	no	yes	no	no
182242078	chr1	200029215	200029243	15	29	1.933333	no	no	yes	yes	no	no	no	yes	yes	yes
182242082	chr1	200039174	200039212	17	39	2.411765	no	no	yes	no	no	no	no	yes	yes	yes
182242084	chr1	200056176	200056211	11	36	3.166667	no	no	yes	no	no	no	no	no	no	yes
182242096	chr1	200075487	200075522	17	36	2.117647	no	no	yes	no	no	no	no	yes	yes	yes
182242105	chr1	200089981	200090036	16	56	3.470588	no	no	yes	no	no	no	no	no	no	yes
182242110	chr1	200113189	200113289	50	101	2.020000	no	no	yes	no	no	no	no	yes	yes	yes
182242124	chr1	200138198	200138234	19	37	1.947368	no	no	yes	no	no	no	no	yes	no	yes
182242130	chr1	200145979	200146015	16	37	2.312500	no	no	yes	no	no	no	no	no	no	yes

182242131	chr1	200146116	200146156	19	41	2.157895	no	no	yes	no	no	no	no	no	no	yes
182242132	chr1	200146827	200146879	27	53	1.962963	no	no	yes	no	no	yes	no	no	no	yes
182242133	chr1	200146926	200146963	14	38	2.714286	no	no	yes	no	no	yes	no	no	no	yes
182242134	chr1	200147749	200147789	12	41	3.416667	no	no	yes	no	yes	no	yes	yes	no	yes
182242137	chr1	200163266	200163309	20	44	2.200000	no	no	yes	no	no	no	no	no	no	yes
182242145	chr1	200182490	200182518	15	29	1.933333	no	no	no	no	no	no	no	no	no	no
182242152	chr1	200206464	200206575	51	112	2.192308	no	no	no	no	no	no	no	yes	yes	no
182242160	chr1	200213506	200213550	20	45	2.250000	yes	no	no	no	no	no	no	yes	yes	no
182242161	chr1	200213576	200213627	12	52	4.333333	yes	no	no	no	no	no	no	yes	yes	no
182242168	chr1	200227468	200227511	20	44	2.200000	no	no	no	no	no	no	no	no	no	no
182242173	chr1	200232412	200232461	26	50	1.923077	no	no	no	no	no	no	no	yes	no	no
182242174	chr1	200232701	200232743	21	43	2.095238	no	no	no	no	no	no	no	no	no	no
182242176	chr1	200246042	200246089	24	48	2.000000	no	no	no	no	no	no	no	yes	no	no
182242185	chr1	200283677	200283713	9	37	4.111111	no	no	yes	no	no	no	no	no	no	yes
182242192	chr1	200296964	200296993	13	30	2.307692	no	no	yes	no	no	no	no	yes	no	yes
182242218	chr1	200365910	200366095	93	186	2.000000	no	no	yes	no	no	yes	no	yes	yes	yes
182242226	chr1	200381712	200381763	22	52	2.500000	no	no	yes	no	no	no	no	yes	yes	yes
182242229	chr1	200384372	200384409	12	38	3.076923	no	no	yes	no	no	no	no	no	no	yes
182242235	chr1	200398699	200398805	51	107	2.176471	no	no	yes	no	no	no	no	yes	no	yes
182242236	chr1	200404322	200404369	18	48	2.666667	no	no	yes	no	no	yes	no	no	no	yes
182242238	chr1	200406439	200406568	65	130	2.061538	no	yes	yes	no	no	yes	no	yes	yes	yes
182242240	chr1	200406698	200406735	12	38	3.250000	no	yes	yes	no	no	yes	no	yes	no	yes
182242242	chr1	200407266	200407392	67	127	1.925373	no	no	yes	yes	no	no	no	yes	yes	yes
182242244	chr1	200409611	200409656	21	46	2.190476	no	no	yes	yes	no	no	no	yes	yes	yes
182242245	chr1	200414634	200414679	18	46	2.555556	no	no	yes	no	no	no	no	no	no	yes

182242246	chr1	200415548	200415577	15	30	2.000000	no	yes	yes	no	no	yes	no	yes	yes	yes
182242248	chr1	200418749	200418778	15	30	2.000000	no	no	yes	no	no	no	no	no	no	yes
182242267	chr1	200438425	200438450	13	26	2.000000	no	no	yes	no	no	no	no	yes	no	yes
182242269	chr1	200446472	200446522	16	51	3.187500	no	no	yes	no	no	no	no	no	yes	yes
182242272	chr1	200458245	200458287	22	43	1.954545	yes	no	yes	no	no	no	no	no	no	yes
182242274	chr1	200468060	200468095	18	36	2.000000	no	no	yes	no	no	no	no	no	no	yes
182242279	chr1	200479617	200479817	79	201	2.569620	no	no	yes	no	no	yes	no	yes	yes	yes
182242285	chr1	200481392	200481425	16	34	2.125000	no	no	yes	no	no	no	no	yes	yes	yes
182242293	chr1	200490329	200490385	28	57	2.035714	no	no	no	no	no	no	no	yes	yes	no
182242294	chr1	200490750	200490776	13	27	2.076923	no	no	no	no	no	no	no	yes	no	no
182242302	chr1	200498384	200498414	14	31	2.214286	no	no	no	no	no	no	no	yes	yes	no
182242304	chr1	200498958	200499047	44	90	2.045455	no	no	no	no	no	no	no	no	no	no
182242315	chr1	200505607	200505645	13	39	2.928571	no	no	no	no	no	no	no	yes	no	no
182242332	chr1	200524912	200524947	11	36	3.363636	no	no	no	no	no	no	no	no	no	no
182242337	chr1	200535345	200535382	18	38	2.222222	no	no	no	no	no	no	no	yes	yes	no
182242338	chr1	200536905	200536933	15	29	1.933333	no	no	no	no	no	no	no	no	no	no
182242351	chr1	200559482	200559541	31	60	1.935484	no	no	yes	no	no	no	no	yes	no	yes
182242363	chr1	200599983	200600014	17	32	1.941176	no	no	yes	no	no	no	no	no	no	yes
182242364	chr1	200605148	200605334	62	187	3.016129	no	no	yes	yes	no	no	no	no	yes	yes
182242365	chr1	200605415	200605547	66	133	2.015152	no	no	yes	no	no	no	no	no	no	yes
182242368	chr1	200616696	200616744	24	49	2.083333	no	no	yes	no	no	no	no	no	no	yes
182242369	chr1	200621838	200621878	12	41	3.416667	no	no	no	no	no	no	no	yes	no	no
182242387	chr1	200654391	200654443	28	53	1.928571	no	no	yes	no	yes	no	no	yes	yes	yes
182242394	chr1	200673230	200673266	14	37	2.714286	no	no	yes	no	no	no	no	yes	no	yes
182242406	chr1	200690657	200690696	22	40	1.909091	no	no	yes	no	no	no	no	no	no	yes

182242409	chr1	200693794	200693831	9	38	4.222222	no	yes	no	no	no	no	no	no	no	yes
182242435	chr1	200749456	200749524	34	69	2.029412	no	no	yes	no	no	no	no	no	no	yes
182242440	chr1	200759995	200760041	21	47	2.238095	no	no	yes	no	no	no	no	yes	yes	yes
182242442	chr1	200765909	200765954	23	46	2.000000	no	no	yes	no	no	no	no	no	yes	yes
182242443	chr1	200766870	200766905	18	36	2.000000	no	no	yes	no	no	no	no	no	no	yes
182242447	chr1	200778101	200778129	15	29	1.933333	no	no	yes	no	no	no	no	no	no	yes
182242464	chr1	200817148	200817272	30	125	4.366667	no	no	yes	no	no	no	no	yes	yes	yes
182242468	chr1	200827718	200827757	19	40	2.105263	no	no	yes	no	no	no	no	yes	no	yes
182242478	chr1	200868778	200868845	29	68	2.413793	no	no	no	no	no	no	no	no	no	no
182242484	chr1	200887580	200887634	23	55	2.391304	no	no	no	no	no	no	no	yes	yes	no
182242486	chr1	200898481	200898519	7	39	5.714286	no	no	yes	no	no	no	no	yes	yes	yes
182242487	chr1	200899357	200899389	10	33	3.500000	no	no	yes	no	no	no	no	yes	no	yes
182242488	chr1	200911010	200911185	26	176	6.807693	yes	no	yes	no	no	no	no	no	yes	yes
182242507	chr1	200974032	200974072	16	41	2.588235	no	yes	yes	no	no	no	no	yes	no	yes
182242510	chr1	200982560	200982595	17	36	2.117647	no	no	yes	no	no	no	no	yes	no	yes
182242513	chr1	200992775	200992812	18	38	2.166667	no	no	yes	no	no	no	no	yes	no	yes
182242519	chr1	201010866	201010891	11	26	2.363636	no	no	yes	no	no	no	no	yes	no	yes
182242524	chr1	201023729	201023769	11	41	3.727273	yes	no	no	no	no	no	yes	yes	yes	no
182242527	chr1	201025046	201025074	13	29	2.230769	no	no	yes	no	no	no	no	yes	yes	yes
182242534	chr1	201038161	201038208	15	48	3.125000	no	no	yes	no	no	no	no	yes	no	yes
182242540	chr1	201048080	201048124	24	45	1.958333	no	no	yes	no	no	no	no	no	no	yes
182242541	chr1	201055905	201055936	14	32	2.357143	no	no	yes	no	no	no	no	yes	yes	yes
182242546	chr1	201069529	201069553	12	25	2.083333	no	no	no	yes	no	no	no	no	no	yes
182242547	chr1	201077603	201077631	15	29	2.000000	no	no	yes	no	no	no	no	no	no	yes
182242548	chr1	201087681	201087710	15	30	2.066667	no	no	yes	no	no	no	no	yes	no	yes

182242552	chr1	201103417	201103449	13	33	2.538461	no	no	yes	no	no	no	no	yes	yes	yes
182242555	chr1	201111923	201112012	28	90	3.250000	no	no	yes	no	no	no	no	yes	no	yes
182242562	chr1	201130998	201131041	23	44	1.913043	no	no	no	no	no	no	no	no	no	no
182242565	chr1	201131265	201131309	23	45	1.956522	no	no	no	no	no	no	no	yes	no	no
182242569	chr1	201139198	201139238	19	41	2.210526	no	no	yes	no	no	no	no	yes	yes	yes
182242576	chr1	201168321	201168345	11	25	2.272727	no	no	yes	no	no	no	no	no	no	yes
182242577	chr1	201170847	201170878	16	32	2.000000	no	no	yes	no	no	no	yes	yes	yes	yes
182242583	chr1	201178435	201178508	31	74	2.375000	no	no	no	no	no	no	no	no	no	no
182242584	chr1	201180174	201180222	22	49	2.227273	no	no	no	no	no	no	no	yes	no	no
182242588	chr1	201189208	201189273	31	66	2.129032	no	no	no	no	no	no	no	no	no	no
182242593	chr1	201200415	201200456	18	42	2.333333	no	no	yes	no	no	no	no	no	no	yes
182242601	chr1	201216350	201216385	16	36	2.250000	no	no	yes	no	no	no	no	yes	no	yes
182242602	chr1	201217677	201217729	26	53	2.038461	no	no	yes	no	no	no	no	yes	no	yes
182242611	chr1	201219962	201220142	89	181	2.078652	no	no	yes	no	no	no	no	yes	no	yes
182242636	chr1	201319406	201319444	20	39	1.950000	no	no	yes	no	no	no	no	no	no	yes
182242637	chr1	201319652	201319701	21	50	2.380952	no	no	yes	no	no	no	no	no	yes	yes
182242638	chr1	201320221	201320263	22	43	2.045455	no	no	yes	no	no	yes	no	yes	no	yes
182242640	chr1	201336868	201336901	16	34	2.187500	no	no	no	no	no	no	no	no	no	no
182242648	chr1	201376943	201376983	20	41	2.100000	no	no	yes	no	no	no	no	yes	yes	yes
182242649	chr1	201382501	201382578	20	78	3.857143	yes	no	yes	no	no	yes	no	yes	yes	yes
182242653	chr1	201418844	201418875	16	32	2.000000	no	no	yes	no	no	no	no	no	no	yes
182242672	chr1	201467732	201467767	18	36	2.000000	no	no	yes	no	no	no	no	yes	yes	yes
182242682	chr1	201497488	201497521	17	34	2.000000	yes	no	yes	no	no	no	no	no	no	yes
182242683	chr1	201501554	201501605	24	52	2.208333	no	no	yes	no	no	no	no	yes	no	yes
182242685	chr1	201509515	201509549	17	35	2.058824	yes	no	yes	no	no	no	no	no	yes	yes

182242698	chr1	201537490	201537553	15	64	4.187500	no	no	yes	no	no	no	no	yes	yes	yes
182242699	chr1	201540971	201541007	17	37	2.176471	no	no	yes	no	no	no	no	yes	yes	yes
182242704	chr1	201563361	201563397	17	37	2.176471	no	no	yes	no	no	no	no	yes	yes	yes
182242706	chr1	201568930	201568957	14	28	2.000000	no	no	yes	no	no	no	no	yes	no	yes
182242719	chr1	201609853	201609881	15	29	1.933333	no	no	no	no	no	no	no	yes	yes	no
182242721	chr1	201612837	201612879	16	43	2.687500	no	no	no	no	no	no	no	yes	yes	no
182242728	chr1	201641919	201641985	33	67	2.060606	no	no	yes	no	no	no	no	yes	no	yes
182242738	chr1	201642753	201642793	22	41	1.909091	no	no	yes	no	no	no	no	yes	no	yes
182242743	chr1	201643376	201643404	13	29	2.230769	no	no	yes	no	no	no	no	yes	no	yes
182242749	chr1	201651395	201651437	19	43	2.315789	no	no	yes	no	no	no	no	yes	yes	yes
182242751	chr1	201654384	201654420	14	37	2.642857	no	no	yes	no	no	no	no	no	no	yes
182242752	chr1	201655256	201655285	7	30	4.285714	no	no	yes	no	no	no	no	yes	yes	yes
182242753	chr1	201666775	201666807	16	33	2.062500	no	no	yes	no	no	no	no	no	no	yes
182242755	chr1	201670533	201670579	21	47	2.380952	no	no	yes	no	no	no	no	yes	no	yes
182242758	chr1	201670872	201670968	48	97	2.041667	no	no	yes	no	no	no	no	yes	no	yes
182242765	chr1	201689854	201690808	109	955	8.747826	no	no	yes	no	no	no	no	yes	no	yes
182242767	chr1	201696176	201696231	16	56	3.500000	no	no	yes	no	no	no	no	yes	yes	yes
182242771	chr1	201703398	201703441	21	44	2.095238	no	no	yes	no	no	no	no	yes	yes	yes
182242775	chr1	201720782	201720816	17	35	2.058824	no	no	yes	no	no	no	no	yes	yes	yes
182242777	chr1	201727117	201727167	26	51	1.961538	no	no	yes	no	no	no	no	yes	no	yes
182242784	chr1	201740140	201740177	19	38	2.000000	no	no	yes	no	no	no	yes	yes	no	yes
182242810	chr1	201834660	201834691	15	32	2.133333	no	no	yes	no	no	no	no	yes	no	yes
182242819	chr1	201886989	201887054	33	66	2.000000	no	no	no	no	no	no	no	yes	no	no
182242821	chr1	201888106	201888151	20	46	2.285714	yes	no	no	no	no	no	no	yes	no	no
182242823	chr1	201888187	201888218	16	32	2.000000	yes	no	no	no	no	no	no	yes	yes	no

182242824	chr1	201888654	201888686	17	33	1.941176	yes	yes	no	no	no	no	no	yes	yes	yes
182242840	chr1	201923932	201924030	15	99	6.562500	no	no	yes	no	no	no	no	yes	no	yes
182242857	chr1	201949209	201949237	14	29	2.071429	no	no	no	no	no	no	no	yes	no	no
182242861	chr1	201954748	201954807	28	60	2.142857	yes	no	no	no	no	no	no	yes	yes	no
182242868	chr1	201966865	201966992	16	128	7.666667	no	no	yes	no	no	no	no	yes	no	yes
182242876	chr1	201977857	201977921	21	65	3.095238	yes	no	no	no	no	no	no	no	no	no
182242878	chr1	201982569	201982620	21	52	2.571429	yes	no	yes	no	no	no	yes	yes	yes	yes
182242887	chr1	202001198	202001235	18	38	2.111111	yes	no	yes	no	yes	no	no	no	yes	yes
182242888	chr1	202003554	202003692	69	139	2.014493	no	no	yes	no	no	yes	no	no	no	yes
182242889	chr1	202005135	202005160	12	26	2.166667	no	no	yes	no	no	yes	no	no	no	yes
182242894	chr1	202013533	202013591	21	59	2.818182	yes	no	yes	no	no	yes	no	yes	yes	yes
182242907	chr1	202041221	202041262	21	42	2.000000	no	no	no	no	no	no	no	yes	yes	no
182242910	chr1	202046161	202046198	18	38	2.111111	no	no	no	no	no	no	no	yes	no	no
182242948	chr1	202097429	202097464	9	36	4.000000	no	no	no	no	no	yes	no	no	no	no
182242953	chr1	202101587	202101638	13	52	4.076923	no	no	no	no	no	no	no	yes	no	no
182242957	chr1	202107839	202107875	18	37	2.111111	no	no	no	no	no	no	no	yes	no	no
182242963	chr1	202129616	202129670	24	55	2.291667	no	yes	no	no	no	no	no	no	yes	yes
182242964	chr1	202132415	202132452	21	38	1.904762	no	yes	no	no	no	yes	no	yes	yes	yes
182242970	chr1	202144869	202144923	24	55	2.291667	yes	no	no	no	no	no	yes	yes	yes	no
182242973	chr1	202156245	202156277	17	33	1.941176	no	no	yes	no	no	no	no	yes	no	yes
182242974	chr1	202161009	202161058	15	50	3.333333	yes	no	yes	no	yes	no	no	yes	yes	yes
182242975	chr1	202167900	202167924	7	25	3.571429	yes	no	no	no	no	no	no	no	yes	no
182242983	chr1	202173624	202173679	23	56	2.434783	no	no	yes	no	no	no	no	yes	yes	yes
182242989	chr1	202182655	202182710	16	56	3.500000	yes	no	yes	no	no	no	no	yes	yes	yes
182242991	chr1	202186198	202186244	8	47	5.777778	yes	no	yes	no	no	no	no	no	no	yes

182242992	chr1	202187008	202187042	7	35	5.000000	no	yes	yes	no	no	no	no	yes	no	yes
182242993	chr1	202188774	202188821	21	48	2.285714	no	no	yes	no	no	no	no	yes	yes	yes
182243006	chr1	202203536	202203571	12	36	3.000000	yes	no	yes	no	no	no	no	yes	yes	yes
182243019	chr1	202215102	202215137	18	36	2.000000	no	no	yes	no	no	no	no	yes	no	yes
182243035	chr1	202251844	202251881	20	38	1.950000	no	no	yes	no	no	no	no	yes	no	yes
182243059	chr1	202332946	202332978	16	33	2.125000	no	no	yes	no	no	no	no	no	no	yes
182243066	chr1	202343161	202343212	18	52	2.842105	no	no	no	no	no	no	no	no	no	no
182243069	chr1	202343890	202343921	15	32	2.133333	no	no	no	no	no	no	no	yes	yes	no
182243093	chr1	202391561	202392154	30	594	19.799999	no	no	yes	no	no	no	no	no	no	yes
182243100	chr1	202402361	202402393	17	33	1.941176	no	no	yes	no	no	no	no	yes	yes	yes
182243107	chr1	202430927	202430961	11	35	3.083333	no	no	yes	no	no	no	no	no	yes	yes
182243111	chr1	202447937	202447977	21	41	1.952381	yes	no	yes	no	no	no	no	yes	no	yes
182243129	chr1	202496518	202496543	13	26	2.000000	yes	no	yes	no	no	no	no	no	no	yes
182243139	chr1	202511028	202511069	19	42	2.315789	no	no	yes	no	no	no	no	no	no	yes
182243150	chr1	202529829	202529859	15	31	2.066667	no	no	yes	no	no	no	no	no	no	yes
182243155	chr1	202547116	202547153	20	38	1.900000	no	no	yes	no	no	no	no	yes	yes	yes
182243162	chr1	202568632	202568677	20	46	2.400000	no	no	yes	no	no	yes	no	yes	yes	yes
182243165	chr1	202596192	202596287	47	96	2.042553	no	yes	no	no	no	no	no	yes	no	yes
182243171	chr1	202616098	202616133	18	36	2.055556	no	no	yes	no	no	no	no	yes	yes	yes
182243177	chr1	202624203	202624248	22	46	2.181818	no	no	yes	no	no	no	no	yes	yes	yes
182243183	chr1	202629435	202629478	21	44	2.095238	no	no	yes	no	no	no	no	yes	yes	yes
182243184	chr1	202637354	202637394	13	41	3.071429	no	no	yes	no	no	no	no	yes	no	yes
182243189	chr1	202647815	202647844	13	30	2.307692	no	no	yes	no	no	no	no	no	no	yes
182243191	chr1	202661305	202661344	14	40	2.928571	no	no	yes	no	no	no	no	yes	yes	yes
182243207	chr1	202714785	202714821	20	37	2.000000	no	no	no	no	no	no	no	no	no	no

182243211	chr1	202739436	202739468	16	33	2.125000	no	no	yes	no	no	no	no	no	no	yes
182243218	chr1	202746646	202746671	13	26	2.000000	yes	no	yes	no	yes	no	no	yes	no	yes
182243228	chr1	202772995	202773034	15	40	2.625000	no	yes	yes	yes	no	yes	no	no	no	yes
182243243	chr1	202795806	202795952	73	147	2.013699	yes	no	yes	no	no	no	no	yes	no	yes
182243250	chr1	202811291	202811330	21	40	1.952381	yes	yes	no	no	no	no	yes	yes	yes	yes
182243260	chr1	202824722	202824772	25	51	2.120000	yes	no	yes	no	no	no	no	yes	no	yes
182243274	chr1	202863167	202863201	17	35	2.117647	no	no	yes	no	no	no	no	yes	yes	yes
182243286	chr1	202886317	202886358	20	42	2.150000	no	no	yes	no	no	no	no	yes	yes	yes
182243305	chr1	202928665	202928712	21	48	2.380952	yes	no	no	no	no	no	no	no	no	no
182243311	chr1	202939872	202939912	20	41	2.050000	no	no	no	no	no	yes	no	yes	no	no
182243316	chr1	202953675	202953715	19	41	2.157895	no	no	yes	no	no	no	no	yes	yes	yes
182243317	chr1	202967261	202967300	17	40	2.411765	yes	no	no	no	yes	no	yes	yes	yes	yes
182243327	chr1	202978723	202978752	14	30	2.142857	no	no	no	no	no	no	no	no	no	no
182243350	chr1	203012111	203012272	87	162	1.862069	no	no	yes	no	no	no	no	yes	yes	yes
182243351	chr1	203013446	203013498	24	53	2.208333	no	no	yes	no	no	no	no	yes	no	yes
182243355	chr1	203035828	203035881	28	54	1.964286	no	no	yes	no	no	no	no	yes	yes	yes
182243371	chr1	203102828	203102856	11	29	2.636364	no	no	yes	no	no	no	no	no	no	yes
182243380	chr1	203146118	203146212	46	95	2.108696	no	no	yes	no	no	no	no	no	yes	yes
182243385	chr1	203167124	203167269	36	146	4.055555	no	yes	no	no	no	yes	no	yes	no	yes
182243399	chr1	203213524	203213548	13	25	1.923077	no	no	yes	no	no	no	no	yes	no	yes
182243412	chr1	203240202	203240234	8	33	4.125000	no	no	yes	no	no	yes	no	yes	no	yes
182243429	chr1	203274928	203274970	17	43	2.529412	no	no	yes	no	no	no	no	yes	yes	yes
182243443	chr1	203309531	203309569	19	39	2.052632	no	yes	no	no	no	no	no	yes	yes	yes
182243451	chr1	203320627	203320666	21	40	1.904762	no	no	no	no	no	no	no	yes	yes	no
182243455	chr1	203326754	203326791	18	38	2.111111	no	no	no	no	no	no	no	yes	yes	no

182243456	chr1	203327647	203327678	16	32	2.062500	no	no	no	no	no	no	yes	yes	no	no
182243462	chr1	203356248	203356299	27	52	1.925926	no	no	no	no	no	no	no	no	no	no
182243478	chr1	203439932	203439975	19	44	2.315789	no	no	no	no	no	no	no	yes	no	no
182243479	chr1	203442486	203442544	29	59	2.137931	no	no	no	no	no	no	no	no	no	no
182243481	chr1	203455480	203455509	14	30	2.142857	no	no	no	no	no	no	no	no	no	no
182243492	chr1	203469261	203469287	14	27	1.928571	no	no	no	no	no	no	no	no	no	no
182243493	chr1	203469925	203469950	12	26	2.166667	no	no	no	no	no	no	no	no	yes	no
182243495	chr1	203473305	203473339	18	35	1.944444	no	no	no	no	no	no	no	no	no	no
182243498	chr1	203475672	203475734	29	63	2.206897	yes	no	no	no	no	no	no	yes	yes	no
182243503	chr1	203483278	203483318	12	41	3.416667	no	no	no	yes	no	no	no	no	yes	yes
182243510	chr1	203503272	203503308	18	37	2.055556	no	no	yes	no	no	no	no	no	no	yes
182243511	chr1	203505917	203505955	20	39	1.950000	no	no	yes	no	no	no	no	yes	yes	yes
182243513	chr1	203515845	203515886	22	42	1.954545	no	no	no	no	no	no	no	yes	no	no
182243515	chr1	203519213	203519300	45	88	1.955556	no	no	no	no	no	no	no	yes	yes	no
182243516	chr1	203524620	203524663	22	44	2.045455	no	no	no	no	no	no	no	yes	yes	no
182243523	chr1	203527510	203527564	15	55	3.625000	no	no	no	no	no	no	no	yes	no	no
182243528	chr1	203529223	203529734	187	512	2.751323	no	no	no	no	no	no	no	yes	yes	no
182243529	chr1	203530632	203530664	18	33	1.944444	no	no	no	no	no	no	no	yes	no	no
182243554	chr1	203569865	203569893	15	29	1.933333	no	no	no	no	no	no	no	yes	yes	no
182243560	chr1	203576351	203576409	30	59	1.966667	no	no	no	no	no	no	no	no	no	no
182243566	chr1	203583821	203584111	105	291	2.757009	no	no	no	no	no	no	no	yes	yes	no
182243573	chr1	203599897	203599927	16	31	1.937500	no	no	no	no	no	no	no	no	yes	no
182243575	chr1	203603935	203604182	24	248	10.333333	no	no	no	no	no	no	no	yes	no	no
182243576	chr1	203604265	203604316	25	52	2.080000	no	no	no	no	no	no	no	yes	no	no
182243578	chr1	203607424	203607455	16	32	2.000000	no	no	no	no	no	no	no	yes	yes	no

182243583	chr1	203621669	203621709	12	41	3.384615	no	no	no	no	no	no	no	yes	yes	no
182243594	chr1	203657642	203657672	15	31	2.066667	no	no	yes	no	yes	no	no	yes	yes	yes
182243603	chr1	203682081	203682222	71	142	2.000000	yes	no	yes	no	no	no	no	yes	yes	yes
182243612	chr1	203687242	203687321	31	80	2.687500	no	no	yes	no	no	no	no	yes	no	yes
182243630	chr1	203735881	203735909	12	29	2.416667	no	no	yes	no	no	no	no	no	yes	yes
182243631	chr1	203743554	203743593	15	40	2.666667	no	yes	no	no	no	no	no	no	yes	yes
182243632	chr1	203744376	203744420	16	45	2.764706	no	no	no	no	no	yes	no	yes	yes	no
182243649	chr1	203764736	203764783	23	48	2.130435	yes	no	no	no	no	no	no	yes	no	no
182243674	chr1	203804452	203804518	33	67	2.060606	no	no	yes	no	no	no	no	yes	yes	yes
182243681	chr1	203819133	203819189	30	57	1.900000	no	no	yes	no	no	yes	no	no	no	yes
182243683	chr1	203826304	203826339	18	36	2.000000	no	no	yes	no	no	no	no	no	no	yes
182243686	chr1	203831377	203831418	19	42	2.210526	no	no	yes	no	no	no	no	no	no	yes
182243695	chr1	203843221	203843254	16	34	2.125000	no	no	yes	no	no	no	no	no	no	yes
182243711	chr1	203902102	203902135	12	34	2.833333	no	no	no	no	no	no	no	yes	no	no
182243743	chr1	203935655	203935686	17	32	1.941176	no	no	no	no	no	no	no	no	yes	no
182243760	chr1	203971550	203971575	13	26	2.000000	no	no	no	no	no	no	no	no	no	no
182243764	chr1	203988440	203988481	22	42	1.954545	no	no	no	no	no	no	no	no	no	no
182243770	chr1	204001808	204001870	32	63	2.000000	no	no	yes	no	no	no	no	yes	no	yes
182243785	chr1	204022348	204022420	23	73	3.166667	no	no	no	no	no	no	no	no	no	no
182243786	chr1	204024211	204024247	12	37	3.083333	no	no	no	no	no	no	no	yes	no	no
182243789	chr1	204031255	204031284	13	30	2.307692	no	no	no	no	no	no	no	no	yes	no
182243794	chr1	204035387	204035424	19	38	2.000000	no	no	yes	no	no	no	no	no	no	yes
182243800	chr1	204044550	204044601	26	52	2.000000	no	no	no	no	no	no	no	no	no	no
182243825	chr1	204066089	204066216	58	128	2.206897	no	no	no	no	yes	no	no	no	no	yes
182243829	chr1	204075321	204075356	16	36	2.250000	no	no	yes	no	no	no	yes	yes	yes	yes

182243832	chr1	204082095	204082145	17	51	3.117647	no	no	yes	no	no	no	no	yes	no	yes
182243842	chr1	204111913	204111957	23	45	1.956522	yes	no	yes	no	no	no	no	no	no	yes
182243848	chr1	204129258	204129299	22	42	1.909091	no	no	no	no	no	no	no	yes	yes	no
182243856	chr1	204160176	204160216	19	41	2.210526	no	no	yes	no	no	no	no	yes	no	yes
182243865	chr1	204194978	204195015	20	38	1.900000	no	no	yes	no	no	no	no	no	yes	yes
182243871	chr1	204203775	204203857	27	83	3.074074	no	no	yes	no	no	no	no	no	no	yes
182243878	chr1	204218771	204218801	16	31	1.937500	no	no	no	no	no	yes	no	yes	yes	no
182243891	chr1	204250417	204250494	36	78	2.194444	no	no	yes	no	no	no	no	yes	no	yes
182243898	chr1	204281533	204281603	32	71	2.312500	no	no	yes	no	no	no	no	no	yes	yes
182243903	chr1	204292459	204292497	20	39	1.950000	no	no	yes	no	no	no	no	yes	no	yes
182243905	chr1	204292479	204292642	58	164	2.844828	no	no	yes	no	no	no	no	yes	yes	yes
182243906	chr1	204292952	204293002	27	51	1.925926	no	no	yes	no	no	no	no	yes	yes	yes
182243907	chr1	204302521	204302552	15	32	2.133333	no	no	yes	no	no	no	no	yes	no	yes
182243914	chr1	204324198	204324227	15	30	2.000000	no	no	yes	no	no	no	no	yes	no	yes
182243918	chr1	204336635	204336668	16	34	2.125000	no	no	yes	no	no	no	no	no	yes	yes
182243928	chr1	204370378	204370425	23	48	2.086957	yes	no	yes	no	no	no	no	yes	no	yes
182243954	chr1	204420531	204420571	22	41	1.954545	no	no	yes	no	no	no	no	yes	yes	yes
182243957	chr1	204436637	204436709	37	73	1.972973	no	no	yes	no	no	yes	no	yes	no	yes
182243961	chr1	204466609	204466648	21	40	1.952381	no	no	yes	no	no	no	no	yes	yes	yes
182243962	chr1	204471059	204471733	25	675	27.000000	no	no	yes	no	no	no	no	no	no	yes
182243964	chr1	204483079	204483113	16	35	2.187500	no	no	yes	no	no	no	no	no	no	yes
182243965	chr1	204485372	204485413	20	42	2.100000	no	no	yes	no	no	no	no	no	no	yes
182243969	chr1	204494223	204494265	23	43	1.913043	no	no	yes	no	no	no	yes	yes	yes	yes
182243973	chr1	204504074	204504119	9	46	5.222222	no	no	no	no	no	no	no	yes	no	no
182243978	chr1	204509577	204509647	33	71	2.151515	no	no	no	no	no	no	no	yes	yes	no

182243985	chr1	204518365	204518404	14	40	2.857143	no	no	yes	no	no	no	no	yes	yes	yes
182243989	chr1	204539373	204539417	23	45	1.956522	no	no	yes	no	no	no	no	no	no	yes
182243991	chr1	204545956	204545984	15	29	1.933333	no	no	yes	no	no	no	no	yes	no	yes
182243992	chr1	204548856	204548888	15	33	2.266667	no	no	yes	no	no	no	no	no	no	yes
182243994	chr1	204554876	204554906	15	31	2.066667	no	yes	no	no	no	yes	no	no	no	yes
182244014	chr1	204603519	204603557	20	39	1.950000	no	no	yes	no	no	no	no	no	no	yes
182244018	chr1	204608123	204608167	16	45	2.812500	no	no	yes	no	no	no	no	no	no	yes
182244019	chr1	204624707	204624737	15	31	2.066667	no	no	yes	no	no	no	no	yes	no	yes
182244029	chr1	204655153	204655207	26	55	2.115385	no	no	yes	no	no	no	no	no	no	yes
182244033	chr1	204660196	204660268	24	73	3.041667	no	no	yes	no	no	no	no	no	no	yes
182244036	chr1	204661899	204661937	17	39	2.352941	no	no	yes	no	no	no	no	no	no	yes
182244041	chr1	204683456	204683485	15	30	2.000000	no	no	yes	no	no	no	no	yes	no	yes
182244047	chr1	204710026	204710053	12	28	2.333333	no	no	no	no	no	no	no	no	no	no
182244048	chr1	204711743	204711783	19	41	2.157895	no	no	no	no	no	no	no	yes	no	no
182244053	chr1	204724568	204724607	20	40	2.000000	no	no	no	no	no	no	no	no	no	no
182244063	chr1	204750276	204750385	35	110	3.142857	no	no	no	no	no	no	no	yes	yes	no
182244068	chr1	204755967	204756048	34	82	2.400000	no	no	no	no	no	no	no	no	yes	no
182244072	chr1	204757827	204757902	33	76	2.363636	no	no	no	no	no	no	no	no	no	no
182244073	chr1	204759228	204759265	19	38	2.000000	no	no	no	no	no	no	no	no	no	no
182244085	chr1	204789688	204789725	17	38	2.235294	no	no	no	no	no	no	no	no	no	no
182244087	chr1	204790745	204790799	13	55	4.285714	no	no	no	no	no	no	no	no	no	no
182244091	chr1	204796378	204796893	190	516	2.730964	no	no	no	no	no	no	no	yes	no	no
182244101	chr1	204816359	204816390	15	32	2.133333	no	no	no	no	no	no	no	no	no	no
182244105	chr1	204830574	204830655	18	82	4.550000	no	no	yes	no	no	no	no	yes	no	yes
182244106	chr1	204832938	204832980	20	43	2.150000	no	no	yes	no	no	no	no	yes	yes	yes

182244108	chr1	204836141	204836182	18	42	2.333333	no	no	yes	no	no	no	no	no	yes	yes
182244127	chr1	204883959	204883993	13	35	2.769231	no	no	yes	no	no	no	no	no	no	yes
182244130	chr1	204893677	204893707	15	31	2.066667	no	no	yes	no	no	no	no	no	yes	yes
182244131	chr1	204894686	204894736	26	51	1.961538	no	no	yes	no	no	no	no	yes	yes	yes
182244132	chr1	204896496	204896526	14	31	2.214286	no	no	yes	no	no	no	no	yes	no	yes
182244138	chr1	204923710	204923738	13	29	2.230769	no	no	yes	no	no	no	no	yes	no	yes
182244141	chr1	204927580	204927615	15	36	2.466667	no	no	yes	no	no	no	no	yes	no	yes
182244142	chr1	204928962	204928998	18	37	2.055556	yes	no	yes	no	no	no	no	no	yes	yes
182244144	chr1	204929685	204929750	29	66	2.275862	no	no	yes	no	no	no	no	yes	no	yes
182244147	chr1	204947846	204947884	20	39	2.050000	no	no	yes	no	no	yes	no	yes	yes	yes
182244164	chr1	204997287	204997323	19	37	2.000000	no	yes	yes	yes	no	no	no	yes	yes	yes
182244168	chr1	205007440	205007481	22	42	1.909091	yes	no	yes	no	no	no	no	no	no	yes
182244172	chr1	205013997	205014032	14	36	2.571429	no	no	yes	no	no	no	no	yes	yes	yes
182244173	chr1	205015275	205015328	21	54	2.571429	no	no	yes	no	no	no	no	no	no	yes
182244180	chr1	205026698	205026741	21	44	2.190476	no	no	no	no	no	no	no	no	no	no
182244200	chr1	205090848	205090879	9	32	3.555556	yes	no	yes	no	no	no	no	no	no	yes
182244224	chr1	205146524	205146568	20	45	2.250000	no	yes	no	no	no	no	no	no	no	yes
182244230	chr1	205159797	205159834	18	38	2.111111	no	no	yes	no	no	yes	no	no	no	yes
182244239	chr1	205175927	205175953	14	27	1.928571	no	no	yes	no	no	no	no	yes	yes	yes
182244247	chr1	205188685	205188718	17	34	2.000000	no	no	yes	no	no	no	no	yes	no	yes
182244252	chr1	205202065	205202154	25	90	3.607143	yes	no	yes	no	no	no	no	yes	yes	yes
182244254	chr1	205203484	205203596	31	113	3.677419	no	no	yes	no	no	yes	no	yes	yes	yes
182244256	chr1	205205295	205205327	17	33	2.058824	no	no	yes	no	no	no	no	yes	yes	yes
182244259	chr1	205209632	205209667	18	36	2.000000	no	no	yes	no	no	no	no	yes	no	yes
182244261	chr1	205212130	205212176	22	47	2.136364	yes	no	no	no	no	no	no	yes	no	no

182244264	chr1	205227994	205228036	22	43	2.090909	yes	no	no	no	yes	no	yes	yes	no	yes
182244266	chr1	205229538	205229586	22	49	2.363636	yes	no	yes	no	no	no	no	yes	yes	yes
182244269	chr1	205231602	205231644	22	43	1.954545	no	no	yes	no	no	no	no	no	no	yes
182244271	chr1	205241801	205241837	15	37	2.466667	no	yes	no	yes	no	no	yes	yes	no	yes
182244272	chr1	205247813	205247853	21	41	1.952381	no	no	yes	no	no	no	no	yes	yes	yes
182244286	chr1	205286735	205286771	10	37	3.700000	no	no	no	no	no	no	no	yes	yes	no
182244295	chr1	205310849	205310881	14	33	2.357143	no	no	yes	no	no	no	no	yes	yes	yes
182244307	chr1	205361459	205361496	18	38	2.222222	no	no	no	no	no	no	no	no	no	no
182244325	chr1	205423604	205423635	15	32	2.200000	no	no	yes	no	no	no	no	yes	no	yes
182244330	chr1	205437056	205437092	18	37	2.166667	no	yes	yes	no	yes	no	no	no	yes	yes
182244334	chr1	205456706	205456766	32	61	1.906250	yes	no	yes	no	yes	no	yes	yes	yes	yes
182244345	chr1	205482911	205482943	16	33	2.062500	no	no	no	no	no	no	no	yes	no	no
182244347	chr1	205485033	205485227	30	195	6.454545	no	no	no	no	no	no	no	yes	no	no
182244352	chr1	205491866	205491898	17	33	1.941176	no	no	no	no	no	no	no	yes	yes	no
182244358	chr1	205506359	205506407	23	49	2.130435	no	no	yes	no	no	no	no	yes	yes	yes
182244362	chr1	205522936	205522976	18	41	2.277778	yes	no	yes	no	yes	no	no	no	no	yes
182244363	chr1	205525258	205525305	14	48	3.428571	no	no	yes	no	no	yes	no	no	no	yes
182244373	chr1	205551396	205551480	45	85	1.911111	no	no	no	no	no	no	no	no	no	no
182244383	chr1	205580254	205580320	32	67	2.093750	no	no	yes	no	no	no	no	no	no	yes
182244384	chr1	205591892	205591926	16	35	2.187500	yes	no	yes	no	no	no	no	yes	no	yes
182244387	chr1	205613569	205613630	32	62	2.000000	no	yes	yes	no	no	no	no	yes	yes	yes
182244404	chr1	205651113	205651499	148	387	2.608974	no	no	no	no	no	no	no	no	no	no
182244408	chr1	205651812	205651978	28	167	6.107143	no	no	no	no	no	no	no	no	no	no
182244410	chr1	205658147	205658187	17	41	2.411765	no	yes	no	no	yes	no	no	yes	no	yes
182244419	chr1	205682854	205682895	22	42	1.909091	no	no	no	no	no	no	no	yes	no	no

182244424	chr1	205694070	205694118	16	49	3.000000	no	no	no	no	no	no	no	no	no	no
182244433	chr1	205711196	205711256	32	61	1.937500	no	no	no	no	no	no	no	yes	yes	no
182244435	chr1	205720725	205720755	16	31	1.937500	yes	no	yes	no	no	no	no	yes	no	yes
182244446	chr1	205733601	205733661	32	61	1.906250	no	no	yes	no	no	no	no	no	no	yes
182244447	chr1	205740815	205740860	23	46	2.000000	no	no	yes	no	no	no	no	no	no	yes
182244451	chr1	205749181	205749218	18	38	2.111111	no	no	yes	no	no	no	yes	yes	yes	yes
182244464	chr1	205790712	205790762	24	51	2.166667	no	yes	no	no	yes	yes	no	no	no	yes
182244469	chr1	205830642	205830898	64	257	4.015625	yes	no	yes	no	no	no	no	no	no	yes
182244477	chr1	205839988	205840012	12	25	2.083333	no	no	yes	no	no	no	no	no	no	yes
182244504	chr1	205913450	205913486	18	37	2.055556	no	yes	no	no	yes	no	no	yes	yes	yes
182244506	chr1	205923851	205923877	12	27	2.250000	no	no	yes	no	no	no	no	no	yes	yes
182244510	chr1	205940400	205940440	16	41	2.562500	no	no	yes	no	no	no	no	yes	no	yes
182244513	chr1	205943753	205943789	18	37	2.055556	yes	no	no	no	no	no	no	yes	no	no
182244530	chr1	205980806	205980907	32	102	3.117647	no	no	yes	no	no	no	no	yes	yes	yes
182244532	chr1	205981364	205982054	35	691	19.885714	no	no	yes	no	no	no	no	yes	yes	yes
182244533	chr1	205984851	205984889	13	39	2.928571	no	no	yes	no	no	no	no	no	no	yes
182244535	chr1	205985737	205985843	27	107	3.962963	no	no	yes	no	no	no	no	no	no	yes
182244539	chr1	205989114	205989170	24	57	2.541667	no	no	yes	no	no	no	no	yes	no	yes
182244549	chr1	206011214	206011240	13	27	2.076923	no	no	yes	no	no	no	no	yes	yes	yes
182244554	chr1	206019884	206019937	28	54	2.071429	no	no	yes	no	no	no	no	yes	no	yes
182244567	chr1	206040193	206040244	27	52	2.000000	no	no	yes	no	no	no	no	yes	no	yes
182244571	chr1	206044273	206044323	21	51	2.476191	no	no	yes	no	no	no	no	no	no	yes
182244579	chr1	206060350	206060389	22	40	1.909091	yes	no	yes	no	no	no	no	yes	no	yes
182244593	chr1	206085329	206085371	18	43	2.388889	no	no	yes	no	no	no	no	no	no	yes
182244598	chr1	206093099	206093130	16	32	2.062500	no	no	yes	no	no	no	no	no	no	yes

182244602	chr1	206105526	206105561	13	36	2.846154	no	no	no	no	no	no	no	yes	yes	no
182244603	chr1	206110271	206110303	12	33	2.750000	no	no	no	yes	yes	no	no	yes	no	yes
182244604	chr1	206114035	206114075	19	41	2.210526	no	no	yes	no	no	no	no	no	yes	yes
182244615	chr1	206140067	206140093	13	27	2.076923	no	no	no	no	no	no	no	no	yes	no
182244626	chr1	206174757	206174786	14	30	2.142857	no	no	no	no	no	yes	no	yes	no	no
182244649	chr1	206235238	206235292	21	55	2.619048	no	no	yes	no	no	no	no	no	no	yes
182244715	chr1	206461859	206461915	19	57	3.105263	no	yes	no	no	no	yes	no	yes	no	yes
182244718	chr1	206468189	206468224	17	36	2.117647	no	no	no	no	no	no	no	yes	yes	no
182244725	chr1	206486282	206487039	102	758	7.364486	no	no	yes	no	no	no	no	yes	no	yes
182244726	chr1	206488072	206488114	22	43	1.954545	no	no	yes	no	no	no	no	yes	no	yes
182244729	chr1	206489214	206489293	34	80	2.411765	no	no	yes	no	no	no	no	no	no	yes
182244740	chr1	206507562	206507590	15	29	1.933333	yes	no	no	no	yes	no	yes	yes	yes	yes
182244742	chr1	206513365	206513400	18	36	2.000000	no	no	yes	no	no	no	no	yes	yes	yes
182244752	chr1	206531468	206531500	14	33	2.428571	no	no	yes	no	no	no	no	no	no	yes
182244773	chr1	206608408	206608539	66	132	2.000000	yes	no	yes	no	no	yes	no	yes	no	yes
182244776	chr1	206618540	206618568	14	29	2.071429	no	no	no	no	no	no	no	no	no	no
182244785	chr1	206637167	206637232	33	66	2.000000	no	no	yes	no	no	no	no	yes	yes	yes
182244787	chr1	206642919	206642958	20	40	2.000000	no	no	yes	no	no	no	no	yes	no	yes
182244790	chr1	206645400	206645450	21	51	2.428571	no	no	yes	no	no	no	no	no	no	yes
182244796	chr1	206651526	206651562	19	37	1.947368	no	yes	yes	no	no	no	no	yes	yes	yes
182244797	chr1	206657855	206657887	13	33	2.538461	no	no	yes	no	no	no	no	yes	yes	yes
182244798	chr1	206659553	206659583	16	31	1.937500	no	no	yes	no	no	no	no	yes	yes	yes
182244801	chr1	206663940	206663968	15	29	1.933333	no	no	yes	no	no	no	no	yes	yes	yes
182244812	chr1	206684718	206684747	13	30	2.307692	yes	no	no	no	no	yes	no	yes	yes	no
182244815	chr1	206686135	206686160	13	26	2.000000	no	no	yes	no	no	no	yes	yes	no	yes

182244822	chr1	206706840	206706935	45	96	2.133333	no	no	yes	no	no	no	no	no	no	yes
182244830	chr1	206739417	206739449	15	33	2.200000	no	no	no	no	no	no	no	yes	yes	no
182244831	chr1	206741559	206741590	15	32	2.133333	no	no	no	no	no	no	no	yes	yes	no
182244853	chr1	206781842	206782069	26	228	8.846154	no	no	yes	no	no	no	no	yes	no	yes
182244874	chr1	206864385	206864438	21	54	2.545455	yes	no	no	no	no	no	no	no	no	no
182244875	chr1	206873842	206873881	21	40	1.904762	no	no	no	no	no	no	no	yes	yes	no
182244878	chr1	206875363	206875419	30	57	1.966667	no	no	no	no	no	no	no	yes	no	no
182244885	chr1	206894500	206894534	7	35	5.000000	no	no	no	no	no	no	no	no	yes	no
182244887	chr1	206898419	206898499	41	81	2.000000	no	no	yes	no	no	no	no	yes	yes	yes
182244889	chr1	206900682	206900713	14	32	2.285714	no	no	yes	no	no	no	no	yes	yes	yes
182244891	chr1	206906322	206906384	24	63	2.666667	no	yes	yes	no	no	no	no	no	no	yes
182244893	chr1	206907081	206907129	8	49	6.375000	yes	no	yes	no	no	yes	no	yes	yes	yes
182244895	chr1	206913463	206913504	19	42	2.263158	no	no	yes	no	no	yes	no	yes	yes	yes
182244901	chr1	206923761	206923785	11	25	2.272727	yes	no	no	no	no	no	no	yes	yes	no
182244902	chr1	206923867	206923971	52	105	2.076923	yes	no	no	no	no	no	no	yes	yes	no
182244906	chr1	206924569	206924740	93	172	1.903226	no	no	no	no	no	no	no	yes	yes	no
182244909	chr1	206927082	206927127	22	46	2.090909	no	no	no	no	no	no	no	yes	no	no
182244916	chr1	206960567	206960649	30	83	2.766667	no	yes	yes	yes	yes	no	no	yes	no	yes
182244917	chr1	206961389	206961432	21	44	2.095238	yes	no	yes	no	no	no	no	yes	no	yes
182244926	chr1	207001691	207001726	18	36	2.000000	no	no	no	no	no	no	no	yes	yes	no
182244928	chr1	207008134	207008163	14	30	2.142857	no	no	no	no	no	no	no	yes	yes	no
182244931	chr1	207023056	207023089	17	34	2.117647	no	no	no	yes	yes	no	no	yes	yes	yes
182244932	chr1	207023503	207023532	15	30	2.000000	no	no	yes	no	no	no	no	yes	no	yes
182244936	chr1	207048533	207048742	107	210	1.962617	no	yes	yes	no	no	no	no	yes	no	yes
182244946	chr1	207086044	207086086	23	43	1.913043	no	no	no	no	no	no	no	yes	yes	no

182244947	chr1	207086127	207086176	26	50	2.000000	no	no	no	no	no	no	no	yes	yes	no
182244948	chr1	207096374	207096440	33	67	2.030303	no	yes	yes	no	no	no	no	no	no	yes
182244951	chr1	207101966	207102025	29	60	2.103448	no	no	no	no	no	no	no	yes	yes	no
182244959	chr1	207126258	207126304	22	47	2.136364	no	no	yes	no	no	yes	no	no	no	yes
182244964	chr1	207149560	207149594	18	35	1.944444	no	no	no	no	no	yes	no	no	no	no
182244968	chr1	207174926	207174962	17	37	2.176471	no	no	yes	no	no	no	no	no	yes	yes
182244975	chr1	207205735	207205777	22	43	1.954545	no	no	no	no	no	no	no	yes	yes	no
182244976	chr1	207206278	207206309	15	32	2.133333	no	no	no	no	no	no	no	no	no	no
182244983	chr1	207228404	207228451	24	48	2.000000	no	yes	no	no	no	no	no	yes	no	yes
182244994	chr1	207290340	207290401	30	62	2.066667	no	no	yes	no	no	no	no	no	yes	yes
182244995	chr1	207293940	207293977	20	38	1.900000	no	no	yes	no	no	no	no	no	no	yes
182245009	chr1	207320663	207320715	14	53	3.785714	yes	no	no	no	no	no	no	yes	yes	no
182245014	chr1	207323198	207323261	14	64	4.642857	no	no	yes	no	no	no	no	yes	no	yes
182245016	chr1	207324002	207324094	45	93	2.066667	yes	no	yes	no	no	no	no	yes	yes	yes
182245017	chr1	207325954	207325998	24	45	1.916667	yes	no	yes	no	no	no	no	yes	no	yes
182245023	chr1	207357554	207357596	22	43	2.000000	no	no	yes	no	no	no	no	no	no	yes
182245037	chr1	207385904	207385932	14	29	2.071429	no	no	yes	no	no	no	no	no	no	yes
182245041	chr1	207394778	207394815	19	38	2.000000	no	no	no	no	no	no	no	no	no	no
182245044	chr1	207404955	207404996	22	42	2.045455	no	no	yes	no	no	no	no	yes	no	yes
182245062	chr1	207443856	207443903	23	48	2.086957	no	no	no	no	no	no	no	no	no	no
182245067	chr1	207462752	207462777	12	26	2.166667	no	no	yes	no	no	no	no	no	no	yes
182245068	chr1	207471124	207471202	36	79	2.194444	no	no	yes	no	no	no	no	yes	yes	yes
182245111	chr1	207581371	207581463	39	93	2.357143	no	no	yes	no	no	no	no	yes	no	yes
182245113	chr1	207581527	207581566	19	40	2.263158	no	no	yes	no	no	no	no	yes	no	yes
182245116	chr1	207595232	207595261	15	30	2.000000	no	no	yes	no	no	no	no	no	no	yes

182245129	chr1	207613756	207613793	18	38	2.111111	no	no	yes	no	no	no	no	yes	no	yes
182245130	chr1	207616459	207616493	18	35	1.944444	no	no	yes	no	no	no	no	no	no	yes
182245139	chr1	207622079	207622130	26	52	2.000000	no	no	yes	no	no	no	no	no	no	yes
182245163	chr1	207712918	207713080	82	163	1.987805	no	no	yes	no	no	no	no	no	yes	yes
182245174	chr1	207750883	207750922	17	40	2.352941	no	no	no	no	no	no	no	yes	yes	no
182245175	chr1	207758213	207758247	19	35	1.947368	no	no	yes	no	no	no	no	yes	no	yes
182245177	chr1	207767661	207767692	16	32	2.000000	yes	yes	yes	no	no	yes	no	no	yes	yes
182245178	chr1	207767847	207767908	31	62	2.032258	yes	yes	yes	no	no	yes	no	no	no	yes
182245180	chr1	207768249	207768299	24	51	2.125000	no	yes	yes	no	no	no	no	no	no	yes
182245183	chr1	207776496	207776527	16	32	2.000000	no	no	yes	no	no	no	no	yes	no	yes
182245191	chr1	207807759	207807805	10	47	4.636364	no	no	yes	no	yes	no	no	yes	yes	yes
182245192	chr1	207808197	207808225	14	29	2.071429	no	no	yes	no	yes	no	no	yes	yes	yes
182245195	chr1	207824024	207824074	8	51	6.375000	no	no	yes	no	no	no	no	yes	yes	yes
182245198	chr1	207845518	207845559	12	42	3.583333	no	no	yes	no	no	no	no	no	no	yes
182245199	chr1	207846741	207846917	30	177	5.966667	no	no	yes	no	no	no	no	no	no	yes
182245207	chr1	207897076	207897100	12	25	2.083333	no	no	yes	no	no	no	no	yes	yes	yes
182245208	chr1	207897185	207897243	30	59	2.033333	no	no	yes	no	no	no	no	yes	no	yes
182245216	chr1	207922378	207922403	12	26	2.166667	no	no	no	no	no	no	no	yes	yes	no
182245217	chr1	207925950	207925991	19	42	2.315789	no	no	no	no	no	no	no	yes	no	no
182245231	chr1	207990550	207990589	16	40	2.500000	no	no	no	no	no	no	no	no	no	no
182245233	chr1	208004623	208004729	27	107	3.962963	no	no	no	no	no	no	no	no	no	no
182245240	chr1	208029972	208030011	19	40	2.105263	no	no	yes	no	yes	no	no	yes	no	yes
182245246	chr1	208077054	208077084	14	31	2.214286	no	no	yes	no	no	no	no	yes	no	yes
182245247	chr1	208083169	208083234	31	66	2.129032	no	no	yes	no	no	no	no	no	no	yes
182245251	chr1	208089655	208089690	15	36	2.466667	no	no	yes	no	no	no	no	no	no	yes

182245254	chr1	208109007	208109051	12	45	3.750000	no	no	yes	no	no	no	no	no	yes	yes
182245257	chr1	208130364	208130416	23	53	2.304348	no	no	yes	no	no	no	no	yes	no	yes
182245258	chr1	208143561	208143592	8	32	4.125000	no	no	yes	no	no	no	no	no	no	yes
182245259	chr1	208145325	208145368	22	44	2.000000	no	no	yes	no	no	no	no	no	yes	yes
182245265	chr1	208155736	208155784	22	49	2.227273	no	no	yes	no	no	no	no	no	no	yes
182245270	chr1	208179238	208179280	21	43	2.047619	no	no	yes	no	no	no	no	yes	no	yes
182245279	chr1	208215524	208215561	12	38	3.166667	no	no	yes	no	no	no	no	yes	no	yes
182245282	chr1	208219267	208219338	21	72	3.428571	no	no	yes	no	no	no	no	yes	yes	yes
182245284	chr1	208237022	208237212	97	191	2.010309	no	no	yes	no	no	no	no	yes	no	yes
182245298	chr1	208265843	208265891	24	49	2.041667	no	no	no	no	no	yes	no	yes	no	no
182245306	chr1	208277268	208277306	19	39	2.052632	no	no	no	no	no	no	no	no	no	no
182245310	chr1	208299211	208299249	12	39	3.250000	no	no	no	no	no	no	no	no	no	no
182245312	chr1	208301386	208301422	14	37	2.714286	no	no	no	no	no	no	no	no	no	no
182245315	chr1	208312976	208313000	12	25	2.083333	no	no	no	no	no	no	no	no	yes	no
182245318	chr1	208322337	208322393	30	57	1.900000	no	no	no	no	no	no	no	no	no	no
182245325	chr1	208341263	208341321	23	59	2.565217	no	no	no	no	no	no	no	yes	yes	no
182245336	chr1	208370980	208371028	22	49	2.304348	no	no	no	no	no	no	no	no	no	no
182245337	chr1	208390832	208390860	13	29	2.230769	no	no	no	no	no	no	no	no	no	no
182245353	chr1	208448750	208448789	21	40	1.952381	no	no	no	no	no	no	no	no	no	no
182245354	chr1	208451625	208451653	8	29	3.625000	no	no	no	no	no	no	no	yes	no	no
182245359	chr1	208459190	208459229	15	40	2.666667	no	no	no	no	no	no	no	no	no	no
182245370	chr1	208530111	208530215	54	105	1.944444	no	no	no	no	no	no	no	no	no	no
182245373	chr1	208530793	208530863	31	71	2.290323	no	no	no	no	no	no	no	no	no	no
182245379	chr1	208543208	208543317	56	110	1.964286	no	no	no	no	no	no	no	no	no	no
182245384	chr1	208563063	208563108	24	46	1.916667	no	no	no	no	no	no	no	no	no	no

182245387	chr1	208571513	208571562	19	50	2.631579	no	no	no	no	no	no	no	yes	no	no
182245388	chr1	208588091	208588122	15	32	2.133333	no	no	no	no	no	no	no	no	no	no
182245401	chr1	208613797	208613919	65	123	1.923077	no	no	no	no	yes	no	no	no	no	yes
182245402	chr1	208614045	208614087	22	43	1.954545	no	no	no	no	yes	no	no	no	no	yes
182245407	chr1	208642901	208643025	59	125	2.118644	no	no	no	no	no	no	no	no	no	no
182245408	chr1	208644758	208644828	16	71	4.437500	no	no	no	no	no	no	no	no	no	no
182245428	chr1	208680480	208680533	26	54	2.076923	no	no	no	no	no	no	no	yes	no	no
182245430	chr1	208688755	208688884	30	130	4.433333	no	no	no	no	no	no	no	no	no	no
182245442	chr1	208714983	208715018	13	36	2.769231	no	no	no	no	no	no	no	no	no	no
182245463	chr1	208755569	208755619	16	51	3.187500	no	no	no	no	no	no	no	no	no	no
182245487	chr1	208824460	208824520	15	61	4.133333	no	no	no	no	no	no	no	no	no	no
182245492	chr1	208839480	208839535	16	56	3.500000	no	no	no	no	no	no	no	no	no	no
182245493	chr1	208843849	208843916	34	68	2.000000	no	no	no	no	no	no	no	no	no	no
182245496	chr1	208844587	208844864	157	278	1.821656	no	no	no	no	no	no	no	no	no	no
182245504	chr1	208854897	208854930	18	34	1.944444	no	no	no	no	no	no	no	no	no	no
182245516	chr1	208874183	208874219	19	37	2.000000	no	no	no	no	no	no	no	no	yes	no
182245530	chr1	208908054	208908090	19	37	2.000000	no	no	no	no	no	no	no	no	yes	no
182245534	chr1	208926341	208926386	16	46	2.875000	no	no	no	no	no	no	no	no	no	no
182245565	chr1	209049217	209049272	19	56	2.947368	no	no	no	no	no	no	no	no	yes	no
182245568	chr1	209065969	209066003	18	35	1.944444	no	no	no	no	no	no	no	no	no	no
182245572	chr1	209077157	209077194	18	38	2.111111	no	no	no	no	no	no	no	yes	no	no
182245575	chr1	209078978	209079013	15	36	2.466667	no	no	no	no	no	no	no	yes	yes	no
182245584	chr1	209106139	209106183	21	45	2.238095	no	no	no	no	no	no	no	yes	yes	no
182245590	chr1	209123724	209123771	22	48	2.181818	no	no	no	no	no	no	no	no	no	no
182245610	chr1	209137289	209137335	23	47	2.130435	no	no	no	no	no	no	no	no	yes	no

182245611	chr1	209139948	209139999	27	52	1.925926	no	no	no	no	no	no	no	yes	no	no
182245614	chr1	209140090	209140152	33	63	1.909091	no	no	no	no	no	no	no	yes	no	no
182245619	chr1	209143420	209143458	21	39	1.904762	no	no	no	no	no	no	no	no	no	no
182245623	chr1	209165093	209165152	27	60	2.222222	no	no	yes	no	no	no	no	no	no	yes
182245625	chr1	209173546	209173574	13	29	2.230769	no	no	yes	no	no	yes	no	no	no	yes
182245646	chr1	209219607	209219637	15	31	2.066667	no	no	yes	no	no	no	no	yes	yes	yes
182245648	chr1	209219714	209219787	35	74	2.142857	no	no	yes	no	no	no	no	no	no	yes
182245650	chr1	209219836	209219899	19	64	3.368421	no	no	yes	no	no	no	no	no	no	yes
182245652	chr1	209220947	209220987	12	41	3.416667	no	no	yes	no	no	no	no	no	no	yes
182245659	chr1	209222759	209222797	20	39	2.000000	no	no	yes	no	no	no	no	yes	no	yes
182245660	chr1	209223638	209223751	56	114	2.053571	no	no	yes	no	no	no	no	no	no	yes
182245662	chr1	209230352	209230438	42	87	2.071429	no	no	yes	no	no	no	no	yes	yes	yes
182245664	chr1	209240669	209240701	10	33	3.181818	no	no	yes	no	no	no	no	no	no	yes
182245671	chr1	209247529	209247701	33	173	5.228571	no	no	yes	no	no	no	no	no	no	yes
182245682	chr1	209293103	209293150	23	48	2.130435	no	no	no	no	no	no	no	no	no	no
182245685	chr1	209295937	209295971	18	35	1.944444	no	no	no	no	no	no	no	no	no	no
182245693	chr1	209351205	209351242	20	38	2.000000	no	no	no	no	no	no	no	no	no	no
182245701	chr1	209398146	209398199	28	54	1.928571	no	no	no	no	no	no	no	yes	yes	no
182245704	chr1	209421019	209421048	16	30	1.937500	no	no	no	no	no	no	no	no	no	no
182245708	chr1	209432260	209432290	15	31	2.066667	no	yes	yes	no	no	yes	no	yes	yes	yes
182245721	chr1	209448551	209448593	15	43	2.812500	no	no	no	no	no	no	no	no	no	no
182245729	chr1	209459751	209459799	21	49	2.333333	no	no	no	no	no	no	no	no	no	no
182245734	chr1	209509245	209509277	17	33	2.000000	no	no	no	no	no	no	no	no	no	no
182245739	chr1	209516928	209516980	21	53	2.523809	no	no	no	no	no	no	no	no	yes	no
182245750	chr1	209529293	209529436	64	144	2.281250	no	no	yes	no	no	no	no	yes	no	yes

182245752	chr1	209529398	209529492	49	95	1.959184	no	no	yes	no	no	no	no	yes	no	yes
182245753	chr1	209529572	209529623	22	52	2.363636	no	no	yes	no	no	no	no	yes	yes	yes
182245760	chr1	209551175	209551219	20	45	2.300000	no	no	yes	no	no	no	no	no	no	yes
182245762	chr1	209551278	209551313	15	36	2.400000	no	no	yes	no	no	no	no	no	no	yes
182245763	chr1	209554859	209554903	23	45	2.043478	no	no	yes	no	no	no	no	yes	yes	yes
182245765	chr1	209562377	209562414	16	38	2.375000	no	no	yes	no	no	no	no	yes	no	yes
182245771	chr1	209576077	209576102	12	26	2.166667	no	no	no	no	no	no	no	no	no	no
182245778	chr1	209623256	209623330	36	75	2.083333	no	no	yes	no	no	yes	no	yes	yes	yes
182245782	chr1	209638809	209638841	15	33	2.200000	no	no	yes	no	no	no	no	yes	yes	yes
182245787	chr1	209655820	209655860	12	41	3.416667	no	no	no	no	no	no	no	yes	no	no
182245788	chr1	209658029	209658061	16	33	2.062500	no	no	no	no	no	no	no	yes	yes	no
182245794	chr1	209674156	209674239	42	84	2.000000	no	no	yes	no	no	no	no	yes	yes	yes
182245797	chr1	209677915	209677944	13	30	2.307692	no	no	yes	no	no	no	no	no	no	yes
182245798	chr1	209683339	209683460	63	122	1.936508	no	no	yes	no	no	no	no	no	no	yes
182245799	chr1	209683558	209683589	16	32	2.000000	no	no	yes	no	no	no	no	yes	no	yes
182245801	chr1	209698120	209698218	24	99	4.037037	no	no	yes	no	no	no	no	yes	no	yes
182245810	chr1	209725815	209725847	17	33	1.941176	no	no	yes	no	no	no	no	yes	no	yes
182245818	chr1	209760502	209760554	25	53	2.120000	no	no	yes	no	no	yes	no	no	no	yes
182245823	chr1	209771496	209771523	12	28	2.333333	no	no	yes	no	no	no	no	yes	yes	yes
182245826	chr1	209792376	209792408	15	33	2.200000	no	yes	no	yes	yes	no	no	yes	yes	yes
182245831	chr1	209799610	209799638	15	29	1.933333	no	no	yes	no	no	no	no	yes	yes	yes
182245832	chr1	209802191	209802222	9	32	3.666667	yes	no	yes	no	no	no	no	yes	yes	yes
182245837	chr1	209820343	209820371	15	29	1.933333	no	no	no	no	no	no	no	no	no	no
182245840	chr1	209835536	209835568	15	33	2.200000	no	no	yes	no	no	no	no	no	no	yes
182245853	chr1	209877531	209877567	15	37	2.466667	no	no	no	no	no	no	no	yes	no	no

182245868	chr1	209922966	209923015	13	50	4.000000	no	no	yes	no	no	no	no	yes	no	yes
182245873	chr1	209936500	209936533	17	34	2.000000	no	no	yes	no	no	no	no	yes	no	yes
182245876	chr1	209939728	209939752	8	25	3.125000	no	no	yes	no	no	no	no	no	yes	yes
182245879	chr1	209961399	209961429	16	31	1.937500	no	no	yes	no	no	no	no	no	no	yes
182245896	chr1	210003864	210003904	21	41	2.000000	no	no	yes	no	no	no	no	no	no	yes
182245897	chr1	210004471	210004511	20	41	2.050000	no	no	yes	no	no	no	no	yes	no	yes
182245898	chr1	210009911	210009987	23	77	3.347826	no	no	yes	no	no	no	no	no	no	yes
182245899	chr1	210017726	210017784	28	59	2.107143	no	no	yes	no	no	no	no	no	no	yes
182245902	chr1	210023879	210024094	116	216	1.870690	no	no	yes	no	no	no	no	no	no	yes
182245904	chr1	210026737	210026772	17	36	2.117647	no	no	yes	no	no	no	no	no	no	yes
182245911	chr1	210065542	210065577	12	36	3.000000	no	no	yes	no	no	no	no	no	no	yes
182245916	chr1	210110310	210110353	22	44	2.000000	no	no	yes	no	no	no	no	no	no	yes
182245920	chr1	210136319	210136387	29	69	2.433333	no	no	yes	no	no	no	no	no	no	yes
182245928	chr1	210150868	210150900	17	33	2.000000	no	no	yes	no	no	no	no	no	no	yes
182245932	chr1	210161666	210161703	17	38	2.352941	no	yes	no	no	no	yes	no	no	no	yes
182245935	chr1	210166604	210166657	18	54	2.947368	no	yes	no	no	no	no	no	no	no	yes
182245939	chr1	210169924	210169974	24	51	2.125000	no	yes	no	no	no	no	no	no	no	yes
182245943	chr1	210201856	210201896	16	41	2.588235	no	no	no	no	no	no	no	no	yes	no
182245950	chr1	210207746	210207866	59	121	2.084746	no	no	no	no	no	no	no	no	no	no
182245956	chr1	210222251	210222286	19	36	1.947368	no	no	no	no	no	no	no	yes	no	no
182245962	chr1	210241515	210241555	14	41	2.928571	no	no	yes	no	no	no	no	no	no	yes
182245964	chr1	210244069	210244105	19	37	1.947368	no	yes	yes	no	no	no	no	no	no	yes
182245967	chr1	210259829	210259866	20	38	1.900000	no	no	no	no	no	no	no	no	yes	no
182245968	chr1	210263091	210263119	14	29	2.071429	no	no	no	no	no	no	no	no	yes	no
182245976	chr1	210275761	210275793	14	33	2.357143	no	no	no	no	no	no	no	no	no	no

182245981	chr1	210304744	210304779	18	36	2.000000	no	no	no	no	no	yes	no	no	no	no
182245996	chr1	210337191	210337290	50	100	2.000000	no	no	yes	no	no	no	no	no	no	yes
182245997	chr1	210338827	210338861	18	35	2.000000	no	no	yes	no	no	no	no	no	no	yes
182246008	chr1	210374328	210374381	28	54	1.928571	yes	no	yes	no	no	no	no	yes	yes	yes
182246009	chr1	210377292	210377331	21	40	1.952381	no	no	yes	no	no	no	no	yes	no	yes
182246014	chr1	210407261	210407297	12	37	3.000000	no	no	yes	no	no	no	no	yes	no	yes
182246017	chr1	210411805	210411845	10	41	4.200000	no	no	yes	no	no	no	no	no	yes	yes
182246023	chr1	210458524	210458559	18	36	2.055556	no	no	yes	no	no	no	no	no	no	yes
182246024	chr1	210459517	210459577	33	61	1.939394	no	no	yes	no	no	no	no	no	no	yes
182246025	chr1	210465850	210466211	24	362	15.346154	no	no	yes	no	no	no	no	yes	no	yes
182246029	chr1	210495216	210495259	19	44	2.421053	no	no	yes	no	no	no	no	no	no	yes
182246035	chr1	210517923	210517957	17	35	2.058824	no	no	yes	no	no	no	no	no	no	yes
182246040	chr1	210533249	210533449	100	201	2.010000	no	no	yes	no	no	no	no	no	no	yes
182246042	chr1	210535676	210535700	12	25	2.083333	no	no	yes	no	no	no	no	no	no	yes
182246046	chr1	210561343	210561380	18	38	2.111111	no	no	yes	no	no	no	no	no	no	yes
182246059	chr1	210609205	210609239	18	35	1.944444	no	no	yes	no	no	no	no	no	no	yes
182246061	chr1	210628467	210628508	16	42	2.625000	no	no	yes	no	no	no	no	no	yes	yes
182246068	chr1	210648673	210648704	16	32	2.000000	no	no	yes	no	no	no	no	no	no	yes
182246072	chr1	210656240	210656287	25	48	1.960000	no	no	yes	no	no	no	no	no	no	yes
182246075	chr1	210679515	210679552	15	38	2.533333	no	yes	yes	no	no	no	no	no	no	yes
182246076	chr1	210679930	210679979	23	50	2.217391	no	yes	yes	no	no	no	no	no	no	yes
182246077	chr1	210686549	210686580	17	32	1.941176	no	no	yes	no	no	no	no	no	no	yes
182246078	chr1	210690202	210690261	25	60	2.400000	no	no	yes	no	no	no	no	no	yes	yes
182246082	chr1	210717004	210717041	19	38	2.052632	no	no	yes	no	no	no	no	no	no	yes
182246094	chr1	210740684	210740708	13	25	1.923077	no	no	yes	no	no	no	no	no	no	yes

182246096	chr1	210742208	210742274	35	67	1.914286	no	no	yes	no	no	no	no	no	no	yes
182246097	chr1	210752646	210752683	18	38	2.111111	no	no	yes	no	no	no	no	no	no	yes
182246122	chr1	210861613	210861670	24	58	2.416667	no	no	yes	no	yes	no	no	no	no	yes
182246130	chr1	210892626	210892658	16	33	2.062500	no	no	yes	no	no	no	no	no	no	yes
182246131	chr1	210896065	210896104	21	40	1.904762	no	no	yes	no	no	no	no	no	no	yes
182246132	chr1	210906351	210906429	39	79	2.025641	no	no	yes	no	no	no	no	yes	yes	yes
182246133	chr1	210909996	210910377	96	382	3.900990	no	no	yes	no	no	no	no	yes	yes	yes
182246144	chr1	210938428	210938470	23	43	1.956522	no	no	yes	no	no	no	no	yes	no	yes
182246152	chr1	210971819	210971864	23	46	2.000000	no	no	yes	no	no	no	no	no	no	yes
182246155	chr1	210981567	210981602	15	36	2.400000	no	no	yes	no	no	no	no	no	no	yes
182246156	chr1	210981891	210981927	16	37	2.437500	no	no	yes	no	no	no	no	no	no	yes
182246161	chr1	210989668	210989701	17	34	2.000000	no	no	yes	no	no	no	no	no	no	yes
182246162	chr1	210990387	210990412	13	26	2.000000	no	no	yes	no	no	no	no	yes	yes	yes
182246172	chr1	211016127	211016268	35	142	4.057143	no	no	yes	no	no	no	no	no	no	yes
182246194	chr1	211092979	211093016	20	38	1.900000	no	no	yes	no	no	no	no	yes	yes	yes
182246196	chr1	211097441	211097487	24	47	1.958333	no	no	yes	no	no	no	no	no	no	yes
182246198	chr1	211106179	211106210	17	32	1.941176	no	no	yes	no	no	no	no	no	yes	yes
182246199	chr1	211108002	211108037	18	36	2.000000	no	no	yes	no	no	yes	no	no	no	yes
182246246	chr1	211201616	211201655	21	40	1.952381	no	no	yes	no	no	no	no	no	no	yes
182246253	chr1	211209974	211210013	21	40	1.952381	no	no	no	no	no	no	no	yes	no	no
182246272	chr1	211234867	211234906	17	40	2.352941	no	no	no	no	no	no	no	yes	no	no
182246279	chr1	211248857	211248881	12	25	2.083333	no	no	no	no	no	no	no	yes	no	no
182246281	chr1	211249374	211249407	15	34	2.333333	no	no	no	no	no	no	no	no	no	no
182246301	chr1	211284015	211284068	28	54	2.000000	no	no	yes	no	no	no	no	no	no	yes
182246308	chr1	211288405	211288475	36	71	1.972222	yes	no	yes	no	no	no	no	no	no	yes

182246313	chr1	211288477	211288576	54	100	1.907407	yes	no	yes	no	no	no	no	no	no	yes
182246317	chr1	211307014	211307044	16	31	1.937500	no	no	yes	no	no	no	no	no	no	yes
182246319	chr1	211307485	211307539	22	55	2.500000	no	no	yes	no	no	no	no	no	no	yes
182246323	chr1	211310372	211310408	15	37	2.533333	no	no	yes	no	no	no	no	no	no	yes
182246324	chr1	211310465	211310512	21	48	2.318182	no	no	yes	no	no	no	no	no	no	yes
182246328	chr1	211326755	211326781	14	27	1.928571	yes	yes	yes	no	no	no	yes	yes	yes	yes
182246332	chr1	211339713	211339742	11	30	2.727273	no	no	yes	no	no	no	no	yes	yes	yes
182246333	chr1	211341141	211341180	19	40	2.105263	no	no	yes	no	no	no	no	yes	yes	yes
182246340	chr1	211358893	211358967	34	75	2.228571	no	yes	yes	no	no	no	no	yes	no	yes
182246344	chr1	211381098	211381128	14	31	2.214286	no	no	yes	no	no	no	no	no	no	yes
182246357	chr1	211397243	211397271	10	29	3.000000	no	yes	yes	no	no	no	no	no	no	yes
182246362	chr1	211429537	211429567	16	31	1.937500	no	no	yes	no	no	no	no	no	no	yes
182246364	chr1	211431426	211431455	12	30	2.500000	no	no	yes	no	no	no	no	no	no	yes
182246388	chr1	211485458	211485491	16	34	2.187500	no	no	yes	no	no	no	no	no	yes	yes
182246395	chr1	211499025	211499252	121	228	1.892562	no	no	no	no	no	no	no	yes	no	no
182246404	chr1	211527020	211527048	15	29	1.933333	no	no	no	no	no	no	no	yes	no	no
182246409	chr1	211537391	211537429	20	39	1.950000	no	no	no	no	no	no	no	yes	yes	no
182246414	chr1	211542010	211542060	22	51	2.363636	no	no	no	no	no	no	no	yes	yes	no
182246422	chr1	211565294	211565327	11	34	3.181818	no	no	no	no	no	no	no	no	no	no
182246424	chr1	211578976	211579014	13	39	3.000000	no	no	no	no	yes	no	yes	yes	yes	yes
182246425	chr1	211579074	211579106	16	33	2.125000	no	no	no	no	yes	no	yes	yes	no	yes
182246426	chr1	211588624	211588659	12	36	3.000000	no	no	no	no	no	no	no	yes	no	no
182246439	chr1	211608713	211608753	15	41	2.800000	no	no	no	no	no	no	no	yes	no	no
182246442	chr1	211610294	211610326	15	33	2.200000	no	no	no	no	no	no	no	yes	no	no
182246448	chr1	211621207	211621242	16	36	2.250000	no	no	no	no	no	no	no	no	no	no

182246464	chr1	211647855	211647907	19	53	3.052632	no	no	yes	no	no	no	no	yes	yes	yes
182246494	chr1	211700771	211700798	10	28	2.800000	no	no	no	no	no	no	no	no	no	no
182246498	chr1	211725019	211725153	64	135	2.107692	no	no	yes	no	no	no	no	yes	yes	yes
182246502	chr1	211749357	211749418	23	62	2.695652	no	yes	no	no	no	yes	no	no	no	yes
182246503	chr1	211762208	211762233	11	26	2.363636	no	no	yes	no	no	no	no	no	no	yes
182246524	chr1	211829332	211829367	18	36	2.055556	yes	no	yes	no	no	no	no	yes	yes	yes
182246564	chr1	211903458	211903519	15	62	4.133333	no	no	no	no	no	no	no	yes	no	no
182246570	chr1	211913571	211913639	24	69	2.875000	no	no	no	no	no	no	no	no	yes	no
182246574	chr1	211923162	211923213	25	52	2.080000	no	no	no	no	no	no	no	yes	yes	no
182246579	chr1	211932669	211932697	11	29	2.636364	no	no	no	no	no	no	no	yes	no	no
182246581	chr1	211938859	211938893	18	35	1.944444	no	no	no	no	no	no	no	yes	yes	no
182246582	chr1	211945946	211945970	13	25	1.923077	no	no	yes	no	no	no	no	no	no	yes
182246583	chr1	211953056	211953097	20	42	2.150000	no	no	yes	no	no	no	no	yes	yes	yes
182246591	chr1	211980858	211980897	21	40	1.904762	no	no	yes	no	no	no	no	no	no	yes
182246593	chr1	211980928	211980982	28	55	1.964286	no	no	yes	no	no	no	no	no	no	yes
182246594	chr1	211994093	211994140	20	48	2.400000	no	no	yes	no	no	no	no	no	no	yes
182246600	chr1	212012872	212012921	26	50	1.923077	no	no	yes	no	no	no	no	no	no	yes
182246607	chr1	212028389	212028428	19	40	2.105263	no	no	yes	no	no	no	no	no	no	yes
182246619	chr1	212102961	212103014	25	54	2.160000	no	yes	yes	no	no	no	no	yes	no	yes
182246623	chr1	212119160	212119196	16	37	2.312500	no	no	no	no	no	no	no	no	no	no
182246659	chr1	212186554	212186649	32	96	3.000000	no	no	yes	no	no	no	no	yes	no	yes
182246667	chr1	212202700	212202840	31	141	4.371428	no	no	no	no	no	no	no	yes	yes	no
182246669	chr1	212204191	212204232	19	42	2.210526	no	no	no	no	no	no	no	no	no	no
182246687	chr1	212224353	212224395	16	43	2.687500	no	no	no	no	no	yes	no	yes	no	no
182246703	chr1	212254949	212254989	12	41	3.416667	no	no	no	no	no	no	no	yes	yes	no

182246747	chr1	212339653	212339688	17	36	2.117647	no	no	yes	no	no	no	no	no	no	yes
182246748	chr1	212340207	212340247	20	41	2.050000	no	no	yes	no	no	no	no	no	no	yes
182246750	chr1	212346206	212346300	31	95	3.193548	no	no	yes	no	no	no	no	no	no	yes
182246761	chr1	212366219	212366259	21	41	1.952381	no	no	yes	no	no	no	no	no	no	yes
182246768	chr1	212375106	212375187	21	82	3.666667	no	no	yes	no	no	yes	no	no	no	yes
182246771	chr1	212381703	212381841	68	139	2.044118	no	no	yes	no	no	no	no	yes	yes	yes
182246775	chr1	212406671	212406715	23	45	1.956522	no	no	yes	no	no	no	no	no	no	yes
182246782	chr1	212445452	212445504	26	53	2.038461	no	no	yes	no	no	no	no	yes	no	yes
182246797	chr1	212503659	212503699	21	41	1.952381	yes	no	no	no	no	no	no	no	no	no
182246806	chr1	212528278	212528310	15	33	2.266667	no	no	yes	no	no	no	no	no	no	yes
182246809	chr1	212530661	212530692	15	32	2.133333	no	no	yes	no	no	no	no	no	no	yes
182246811	chr1	212537226	212537250	11	25	2.272727	no	no	no	no	no	no	no	no	no	no
182246822	chr1	212556421	212556472	18	52	2.944444	no	no	no	no	no	yes	no	no	no	no
182246824	chr1	212561103	212561146	19	44	2.368421	no	no	yes	no	no	no	no	yes	yes	yes
182246826	chr1	212565030	212565069	21	40	1.952381	yes	no	no	no	no	no	no	yes	no	no
182246830	chr1	212586293	212586324	17	32	2.000000	no	no	yes	no	no	no	no	yes	no	yes
182246831	chr1	212587626	212587669	24	44	1.833333	no	no	yes	no	no	no	no	yes	no	yes
182246835	chr1	212596084	212596125	19	42	2.210526	no	no	yes	no	no	no	no	yes	yes	yes
182246842	chr1	212625712	212625746	12	35	2.916667	no	no	no	yes	yes	no	no	yes	no	yes
182246843	chr1	212629439	212629474	12	36	3.083333	no	no	no	no	no	no	no	yes	no	no
182246852	chr1	212640997	212641039	23	43	1.956522	no	no	no	no	no	no	no	no	no	no
182246853	chr1	212641270	212641356	41	87	2.121951	no	no	no	no	no	no	no	yes	no	no
182246861	chr1	212653241	212653273	17	33	1.941176	no	no	no	no	no	yes	no	yes	yes	no
182246863	chr1	212657301	212657398	33	98	3.060606	no	no	yes	no	no	no	no	yes	no	yes
182246882	chr1	212709236	212709292	30	57	1.900000	no	no	no	no	no	no	no	no	no	no

182246885	chr1	212720731	212720764	17	34	2.058824	no	no	no	no	no	no	no	no	no	no
182246898	chr1	212728327	212728358	17	32	1.941176	no	yes	yes	no	no	no	no	no	no	yes
182246900	chr1	212730728	212730762	15	35	2.333333	no	yes	yes	no	no	no	no	yes	no	yes
182246903	chr1	212739656	212739699	22	44	2.000000	no	no	yes	no	no	no	no	yes	no	yes
182246911	chr1	212755727	212755779	28	53	2.000000	no	no	yes	no	no	no	no	no	no	yes
182246926	chr1	212776706	212776761	24	56	2.320000	no	no	yes	no	no	no	no	yes	no	yes
182246979	chr1	212839108	212839150	15	43	2.866667	no	no	yes	no	no	no	no	no	no	yes
182246991	chr1	212854918	212854959	16	42	2.687500	no	no	yes	no	no	yes	no	no	no	yes
182246994	chr1	212854956	212855024	37	69	1.918919	no	no	yes	no	no	yes	no	no	no	yes
182246995	chr1	212855782	212855837	20	56	2.950000	yes	no	yes	no	yes	yes	no	no	no	yes
182246997	chr1	212862049	212862102	15	54	3.600000	no	no	yes	no	no	no	no	yes	no	yes
182246999	chr1	212872598	212872649	20	52	2.700000	no	no	yes	no	no	no	no	no	no	yes
182247004	chr1	212890690	212890742	24	53	2.208333	no	no	yes	no	no	no	no	no	no	yes
182247014	chr1	212923191	212923237	24	47	1.958333	no	no	no	no	no	no	no	yes	no	no
182247020	chr1	212936003	212936041	20	39	1.950000	no	no	no	no	no	no	no	yes	no	no
182247022	chr1	212944693	212944725	17	33	1.941176	no	no	no	no	no	no	no	yes	no	no
182247027	chr1	212964832	212964870	20	39	1.950000	no	no	yes	no	no	no	no	yes	no	yes
182247028	chr1	212981037	212981072	10	36	3.600000	no	no	yes	no	no	no	no	yes	yes	yes
182247029	chr1	212982747	212982785	21	39	1.952381	no	no	yes	no	no	no	no	yes	no	yes
182247037	chr1	212995865	212995911	25	47	1.920000	no	no	yes	no	no	no	no	yes	no	yes
182247040	chr1	213006796	213006828	15	33	2.266667	no	no	yes	no	yes	no	no	no	no	yes
182247052	chr1	213041323	213041349	14	27	1.928571	no	no	no	no	no	no	no	yes	no	no
182247067	chr1	213076174	213076216	21	43	2.142857	no	no	yes	no	no	no	no	yes	no	yes
182247074	chr1	213102064	213102093	15	30	2.000000	no	no	yes	no	no	no	no	no	no	yes
182247077	chr1	213121503	213121538	16	36	2.375000	no	no	yes	no	no	no	no	no	no	yes

182247079	chr1	213128229	213128270	10	42	4.400000	no	no	yes	no	no	no	no	yes	no	yes
182247080	chr1	213129543	213129583	21	41	1.952381	no	yes	no	yes	yes	no	no	no	yes	yes
182247081	chr1	213134313	213134342	16	30	1.937500	no	no	yes	no	no	no	no	no	no	yes
182247083	chr1	213137311	213137347	19	37	1.947368	no	no	yes	no	no	no	no	yes	no	yes
182247106	chr1	213169758	213169786	8	29	3.625000	no	no	yes	no	no	no	no	yes	no	yes
182247112	chr1	213179057	213179091	18	35	2.000000	no	no	yes	no	no	no	no	no	no	yes
182247115	chr1	213182719	213182796	34	78	2.305556	no	no	yes	no	no	no	no	yes	no	yes
182247116	chr1	213184395	213184432	9	38	4.200000	no	no	yes	no	no	no	no	no	no	yes
182247129	chr1	213220653	213220704	28	52	1.928571	no	no	yes	no	no	no	no	no	no	yes
182247131	chr1	213229305	213229346	22	42	1.909091	no	no	yes	no	no	no	no	no	yes	yes
182247132	chr1	213230924	213230956	12	33	2.833333	no	no	yes	no	no	no	no	no	no	yes
182247133	chr1	213232072	213232103	12	32	2.666667	no	no	yes	no	no	no	no	yes	yes	yes
182247134	chr1	213237954	213237980	14	27	1.928571	no	no	yes	no	no	no	no	no	no	yes
182247135	chr1	213250890	213250942	27	53	2.000000	no	no	yes	no	no	no	no	no	no	yes
182247138	chr1	213262191	213262220	9	30	3.333333	no	no	yes	no	no	no	no	no	no	yes
182247150	chr1	213293733	213293794	30	62	2.100000	no	no	no	no	no	no	no	no	no	no
182247152	chr1	213294845	213294895	25	51	2.080000	no	no	no	no	no	no	no	no	no	no
182247154	chr1	213302835	213302881	21	47	2.238095	no	no	no	no	no	no	no	no	no	no
182247160	chr1	213326191	213326228	19	38	2.000000	no	no	no	no	no	no	no	no	no	no
182247190	chr1	213456281	213456320	20	40	2.000000	no	no	no	no	no	no	no	no	no	no
182247191	chr1	213466949	213466987	19	39	2.052632	no	no	no	no	no	no	no	yes	no	no
182247192	chr1	213470001	213470029	12	29	2.416667	no	no	no	no	no	no	no	yes	no	no
182247194	chr1	213476037	213476075	9	39	4.333333	no	no	no	no	no	no	no	yes	yes	no
182247210	chr1	213559528	213559584	30	57	1.900000	no	no	yes	no	no	no	no	no	no	yes
182247216	chr1	213576203	213576240	20	38	1.900000	no	no	yes	no	no	no	no	no	no	yes

182247226	chr1	213601330	213601394	29	65	2.266667	no	no	no	no	no	no	no	no	no	no
182247239	chr1	213646846	213646898	28	53	1.964286	no	no	no	no	no	no	no	no	no	no
182247242	chr1	213664581	213664615	15	35	2.333333	no	no	no	no	no	no	no	no	no	no
182247244	chr1	213678142	213678172	14	31	2.214286	no	no	no	no	no	no	no	no	no	no
182247249	chr1	213720910	213720955	23	46	2.043478	no	no	no	no	no	no	no	no	no	no
182247256	chr1	213740603	213741018	27	416	15.379311	no	no	yes	no	no	no	no	no	no	yes
182247259	chr1	213749069	213749155	43	87	2.023256	no	no	yes	no	no	no	no	no	no	yes
182247260	chr1	213749648	213749678	16	31	1.937500	no	no	yes	no	no	no	no	yes	no	yes
182247261	chr1	213751342	213751374	8	33	4.125000	no	no	yes	no	no	no	no	no	yes	yes
182247266	chr1	213764300	213764325	12	26	2.166667	no	no	yes	no	no	no	no	yes	no	yes
182247271	chr1	213779007	213779043	19	37	2.000000	no	no	yes	no	no	no	no	no	no	yes
182247273	chr1	213782630	213782675	24	46	1.916667	no	no	yes	no	no	no	no	no	no	yes
182247275	chr1	213789733	213789759	14	27	1.928571	no	no	yes	no	no	no	no	yes	no	yes
182247277	chr1	213793866	213793899	15	34	2.266667	no	no	yes	no	no	no	no	yes	yes	yes
182247278	chr1	213803599	213803623	10	25	2.500000	no	no	no	no	no	no	no	yes	no	no
182247285	chr1	213823727	213823770	15	44	3.000000	no	no	yes	no	no	no	no	no	no	yes
182247294	chr1	213856490	213856565	29	76	2.655172	no	no	yes	no	no	no	no	yes	no	yes
182247308	chr1	213885165	213885195	16	31	1.937500	no	no	yes	no	no	no	no	no	no	yes
182247311	chr1	213895205	213895235	15	31	2.066667	yes	no	yes	no	no	yes	no	yes	no	yes
182247317	chr1	213904955	213905009	25	55	2.240000	no	no	yes	no	no	no	no	yes	no	yes
182247319	chr1	213905049	213905114	18	66	3.631579	no	no	yes	no	no	no	no	yes	no	yes
182247324	chr1	213908766	213908840	39	75	1.923077	no	no	yes	no	no	no	no	yes	no	yes
182247326	chr1	213919949	213919986	18	38	2.111111	no	no	yes	no	no	no	no	no	no	yes
182247331	chr1	213928505	213928540	18	36	2.000000	no	no	yes	no	no	no	no	no	no	yes
182247332	chr1	213931563	213931619	30	57	1.900000	no	no	yes	no	no	no	no	yes	no	yes

182247333	chr1	213934670	213934701	17	32	1.941176	no	no	yes	no	no	no	no	yes	no	yes
182247337	chr1	213957395	213957447	27	53	1.962963	no	no	yes	no	no	no	no	no	no	yes
182247338	chr1	213957869	213957911	22	43	1.954545	no	no	yes	no	no	no	no	no	no	yes
182247346	chr1	213978012	213978052	22	41	1.954545	no	no	yes	no	no	no	no	yes	no	yes
182247347	chr1	213980271	213980299	15	29	1.933333	yes	no	yes	no	no	no	yes	yes	no	yes
182247351	chr1	213984724	213984762	18	39	2.166667	no	no	yes	no	yes	no	no	yes	no	yes
182247373	chr1	214045096	214045156	20	61	3.050000	no	no	no	no	no	no	no	yes	no	no
182247374	chr1	214045488	214045531	21	44	2.095238	no	no	no	no	no	no	no	no	no	no
182247378	chr1	214053314	214053459	72	146	2.027778	no	no	yes	no	no	no	no	no	no	yes
182247382	chr1	214056381	214056415	17	35	2.058824	no	no	yes	no	no	no	no	no	no	yes
182247404	chr1	214152743	214152780	15	38	2.533333	no	no	yes	no	no	no	no	yes	no	yes
182247405	chr1	214156773	214156886	59	114	1.932203	no	no	yes	no	no	no	no	yes	yes	yes
182247412	chr1	214166174	214166209	12	36	3.000000	no	no	yes	no	no	no	no	yes	yes	yes
182247413	chr1	214167589	214167620	12	32	2.666667	no	no	yes	no	no	no	no	yes	no	yes
182247414	chr1	214171576	214171604	14	29	2.142857	no	no	yes	no	no	no	no	no	no	yes
182247415	chr1	214178426	214178464	20	39	2.000000	no	no	yes	no	no	no	no	yes	no	yes
182247419	chr1	214187792	214187838	7	47	7.000000	yes	no	no	no	no	no	no	yes	no	no
182247422	chr1	214187874	214187930	21	57	2.761905	yes	no	no	no	no	no	no	yes	no	no
182247423	chr1	214189009	214189056	22	48	2.181818	no	no	no	no	no	no	no	yes	no	no
182247424	chr1	214194292	214194335	22	44	2.000000	no	no	no	no	no	no	no	no	no	no
182247426	chr1	214210121	214210170	25	50	2.000000	no	no	no	no	no	no	no	no	no	no
182247430	chr1	214215017	214215061	17	45	2.705882	no	no	no	no	no	no	no	yes	no	no
182247434	chr1	214235308	214235350	19	43	2.263158	no	no	no	no	no	no	no	yes	yes	no
182247435	chr1	214241534	214241564	16	31	2.000000	no	no	no	no	no	no	no	yes	no	no
182247443	chr1	214258417	214258469	17	53	3.176471	no	no	no	no	no	no	no	no	yes	no

182247451	chr1	214281443	214281481	20	39	1.950000	no	no	yes	no	no	no	yes	yes	yes	yes
182247452	chr1	214281570	214281605	13	36	2.769231	no	no	yes	no	no	no	yes	yes	yes	yes
182247453	chr1	214281927	214281961	18	35	1.944444	no	no	yes	no	no	no	no	yes	yes	yes
182247459	chr1	214320051	214320101	25	51	2.120000	no	no	yes	no	no	no	no	yes	yes	yes
182247461	chr1	214342706	214342764	17	59	3.470588	no	no	no	no	no	no	no	yes	yes	no
182247465	chr1	214357493	214357534	17	42	2.470588	no	yes	no	no	yes	yes	no	no	no	yes
182247467	chr1	214361308	214361344	18	37	2.055556	no	no	yes	no	no	no	no	yes	yes	yes
182247472	chr1	214382694	214382734	16	41	2.562500	no	no	yes	no	no	no	no	no	no	yes
182247473	chr1	214382766	214382803	20	38	1.900000	no	no	yes	no	no	no	no	no	no	yes
182247474	chr1	214385476	214385575	28	100	3.571429	no	no	yes	no	no	no	no	no	no	yes
182247475	chr1	214385694	214385727	16	34	2.125000	no	no	yes	no	no	no	no	yes	yes	yes
182247487	chr1	214413963	214414005	21	43	2.047619	no	no	yes	no	no	no	no	no	no	yes
182247493	chr1	214442713	214442743	15	31	2.066667	no	no	yes	no	no	no	no	no	no	yes
182247501	chr1	214453630	214453664	18	35	1.944444	no	no	yes	no	no	no	no	yes	yes	yes
182247508	chr1	214460472	214460625	72	154	2.138889	no	no	yes	no	no	no	no	yes	yes	yes
182247513	chr1	214487220	214487260	19	41	2.157895	no	no	yes	no	no	no	no	no	no	yes
182247530	chr1	214539699	214539729	15	31	2.133333	no	no	yes	no	no	no	no	yes	no	yes
182247531	chr1	214542107	214542199	30	93	3.031250	no	no	yes	no	no	no	no	no	yes	yes
182247534	chr1	214544793	214544899	31	107	3.451613	no	no	yes	no	no	no	no	yes	no	yes
182247535	chr1	214546449	214546481	17	33	1.941176	no	no	yes	no	no	no	no	yes	yes	yes
182247536	chr1	214547400	214547424	11	25	2.272727	no	no	yes	no	no	no	no	yes	yes	yes
182247538	chr1	214553989	214554013	13	25	1.923077	no	no	no	no	no	no	no	no	yes	no
182247572	chr1	214667230	214667267	18	38	2.111111	no	no	no	no	no	no	no	no	no	no
182247573	chr1	214670764	214670795	15	32	2.133333	no	no	no	no	no	no	no	yes	yes	no
182247577	chr1	214676827	214676856	14	30	2.142857	no	no	no	no	no	no	no	yes	no	no

182247583	chr1	214683115	214683551	86	437	5.093023	no	no	no	no	no	no	no	no	no	no
182247587	chr1	214685516	214685579	26	64	2.538461	no	no	no	no	no	no	no	yes	no	no
182247595	chr1	214719932	214720167	79	236	2.987342	no	no	no	no	no	no	no	yes	no	no
182247596	chr1	214728576	214728608	16	33	2.062500	no	no	no	no	no	no	no	no	no	no
182247603	chr1	214744411	214744444	12	34	2.769231	no	no	no	no	no	no	no	no	no	no
182247605	chr1	214756262	214756330	33	69	2.121212	no	no	no	no	no	no	no	no	no	no
182247608	chr1	214767369	214767503	45	135	3.021739	no	no	no	no	no	no	no	no	no	no
182247609	chr1	214770598	214770638	12	41	3.416667	no	no	no	no	no	no	no	no	no	no
182247628	chr1	214848788	214848824	11	37	3.250000	no	no	no	no	no	no	no	no	no	no
182247650	chr1	214885792	214885854	14	63	4.500000	no	no	no	no	no	no	no	no	no	no
182247653	chr1	214906362	214906390	15	29	1.933333	no	no	no	no	no	no	no	no	no	no
182247656	chr1	214907740	214907803	32	64	2.093750	no	no	no	no	no	no	no	no	no	no
182247658	chr1	214908395	214908598	100	204	2.040000	no	no	no	no	no	no	no	no	no	no
182247664	chr1	214917164	214917231	32	68	2.125000	no	no	no	no	no	no	no	yes	no	no
182247669	chr1	214917714	214917758	21	45	2.238095	no	no	no	no	no	no	no	no	no	no
182247670	chr1	214920670	214920696	13	27	2.076923	no	no	no	no	no	no	no	no	no	no
182247673	chr1	214933367	214933415	24	49	2.125000	no	no	no	no	no	no	no	yes	no	no
182247676	chr1	214935446	214935475	16	30	1.937500	no	no	no	no	no	no	no	no	yes	no
182247678	chr1	214949338	214949378	20	41	2.050000	no	no	yes	no	no	no	no	no	no	yes
182247682	chr1	214983527	214983560	11	34	3.000000	no	no	yes	no	no	no	no	yes	no	yes
182247684	chr1	214986401	214986469	34	69	2.029412	no	no	yes	no	no	no	no	no	no	yes
182247687	chr1	214995237	214995289	24	53	2.291667	no	no	no	no	no	no	no	no	no	no
182247689	chr1	214997047	214997106	31	60	2.000000	no	no	no	no	no	no	no	no	no	no
182247697	chr1	215020991	215021030	19	40	2.157895	no	no	yes	no	no	no	no	no	no	yes
182247703	chr1	215029510	215029656	49	147	2.943396	no	no	yes	no	no	no	no	no	no	yes

182247706	chr1	215042197	215042311	61	115	1.885246	no	no	yes	no	no	no	no	no	no	yes
182247711	chr1	215056842	215056979	68	138	2.044118	no	no	yes	no	no	no	no	no	yes	yes
182247712	chr1	215059036	215059071	18	36	2.000000	no	no	yes	no	no	no	no	yes	no	yes
182247714	chr1	215059055	215059159	36	105	2.916667	no	no	yes	no	no	no	no	yes	no	yes
182247717	chr1	215064307	215064371	26	65	2.500000	no	no	yes	no	no	no	no	no	no	yes
182247719	chr1	215074736	215074766	14	31	2.214286	no	no	yes	no	no	no	no	no	no	yes
182247721	chr1	215075778	215075811	18	34	1.944444	no	no	yes	no	no	no	no	no	no	yes
182247733	chr1	215113883	215113916	17	34	2.000000	no	no	yes	no	no	no	no	yes	yes	yes
182247735	chr1	215120435	215120467	17	33	1.941176	no	no	yes	no	no	no	no	no	no	yes
182247737	chr1	215128777	215128821	15	45	3.133333	no	no	yes	no	no	no	no	yes	yes	yes
182247745	chr1	215146977	215147016	21	40	1.904762	no	no	yes	no	no	no	no	yes	no	yes
182247746	chr1	215147447	215147478	17	32	1.941176	no	no	yes	no	no	no	no	no	yes	yes
182247748	chr1	215150730	215150777	21	48	2.285714	no	no	yes	no	no	no	no	no	no	yes
182247750	chr1	215158613	215158712	50	100	2.000000	no	no	yes	no	no	no	no	no	no	yes
182247759	chr1	215192967	215193252	108	286	2.666667	no	no	yes	no	no	no	no	no	no	yes
182247760	chr1	215195720	215195751	17	32	1.941176	no	no	yes	no	no	yes	no	yes	no	yes
182247770	chr1	215213636	215213688	26	53	2.038461	no	no	yes	no	no	no	no	no	no	yes
182247780	chr1	215231330	215231364	14	35	2.500000	no	no	yes	no	no	no	no	no	no	yes
182247786	chr1	215259265	215259296	15	32	2.133333	no	no	no	no	no	no	no	no	yes	no
182247790	chr1	215264796	215264897	36	102	2.800000	no	no	no	no	no	no	no	no	no	no
182247796	chr1	215283078	215283103	13	26	2.000000	no	no	no	no	no	no	no	no	no	no
182247799	chr1	215292555	215292591	19	37	1.947368	no	no	no	no	no	no	no	no	no	no
182247807	chr1	215305352	215305385	17	34	2.058824	no	no	no	no	no	no	no	no	no	no
182247810	chr1	215308854	215308895	19	42	2.421053	no	no	no	no	no	no	no	no	no	no
182247819	chr1	215350607	215350712	55	106	1.963636	no	no	no	no	no	no	no	no	no	no

182247824	chr1	215362947	215362981	16	35	2.187500	no	no	no	no	no	no	no	no	no	no
182247826	chr1	215371968	215372438	40	471	12.175000	no	no	no	no	no	no	no	no	no	no
182247833	chr1	215373204	215373247	22	44	2.000000	no	no	no	no	no	no	no	no	no	no
182247836	chr1	215373242	215373361	27	120	4.666667	no	no	no	no	no	no	no	no	no	no
182247840	chr1	215392008	215392051	23	44	2.000000	no	no	no	no	no	no	no	yes	no	no
182247862	chr1	215449510	215449569	17	60	3.555556	no	no	no	no	no	no	no	no	no	no
182247868	chr1	215449670	215449711	19	42	2.263158	no	no	no	no	no	no	no	no	no	no
182247870	chr1	215460296	215460341	19	46	2.526316	no	no	no	no	no	no	no	no	no	no
182247875	chr1	215482136	215482187	26	52	2.000000	no	no	no	no	no	no	no	yes	yes	no
182247884	chr1	215515481	215515533	20	53	2.619048	no	no	no	no	no	no	no	yes	no	no
182247887	chr1	215527498	215527574	38	77	2.131579	no	no	no	no	no	no	no	no	no	no
182247899	chr1	215563295	215563321	14	27	1.928571	no	no	no	no	no	no	no	no	no	no
182247904	chr1	215575692	215575735	19	44	2.315789	no	no	yes	no	no	no	no	no	no	yes
182247910	chr1	215591189	215591250	32	62	2.000000	no	no	yes	no	no	no	no	yes	yes	yes
182247916	chr1	215615000	215615053	24	54	2.250000	no	no	yes	no	no	no	no	no	no	yes
182247920	chr1	215625150	215625264	57	115	2.017544	no	yes	no	no	no	no	no	no	no	yes
182247934	chr1	215671796	215671888	45	93	2.066667	no	no	yes	no	no	no	no	yes	yes	yes
182247940	chr1	215683718	215683758	20	41	2.200000	no	no	yes	no	no	no	no	no	no	yes
182247948	chr1	215712601	215712627	12	27	2.250000	no	no	yes	no	no	no	no	no	no	yes
182247949	chr1	215723273	215723313	15	41	2.733333	no	no	yes	no	no	no	no	no	no	yes
182247958	chr1	215727181	215727283	42	103	2.500000	no	no	yes	no	no	no	no	no	no	yes
182247964	chr1	215738450	215738614	81	165	2.049383	no	no	yes	no	no	no	no	no	no	yes
182247967	chr1	215742408	215742486	29	79	2.741935	no	no	yes	no	no	no	no	no	no	yes
182247968	chr1	215743107	215743132	13	26	2.000000	no	no	yes	no	no	no	no	no	no	yes
182247971	chr1	215743420	215743484	32	65	2.031250	no	no	yes	no	no	no	no	no	no	yes

182247974	chr1	215754510	215754549	20	40	2.050000	no	no	yes	no	no	no	no	yes	no	yes
182247985	chr1	215774531	215774560	12	30	2.500000	no	no	yes	no	no	no	no	no	no	yes
182248002	chr1	215812716	215812755	18	40	2.222222	no	no	yes	no	no	no	no	no	no	yes
182248015	chr1	215865821	215865909	32	89	2.757576	no	no	yes	no	no	no	no	no	no	yes
182248021	chr1	215875855	215875963	46	109	2.416667	no	no	yes	no	no	no	no	yes	no	yes
182248026	chr1	215893251	215893276	12	26	2.166667	no	no	yes	no	no	no	no	no	no	yes
182248030	chr1	215901797	215901836	20	40	2.000000	no	no	yes	no	no	yes	no	no	no	yes
182248032	chr1	215905037	215905073	19	37	1.947368	no	no	yes	no	no	no	no	yes	no	yes
182248034	chr1	215907832	215907904	23	73	3.125000	no	no	yes	no	no	no	no	no	no	yes
182248035	chr1	215910283	215910309	14	27	1.928571	no	no	yes	no	no	no	no	no	no	yes
182248040	chr1	215938082	215938142	28	61	2.178571	no	no	yes	no	no	no	no	yes	no	yes
182248041	chr1	215941264	215941300	19	37	1.947368	no	no	yes	no	no	no	no	no	no	yes
182248042	chr1	215947845	215947882	17	38	2.235294	no	no	yes	no	no	no	no	no	no	yes
182248045	chr1	215965225	215965265	18	41	2.277778	no	no	yes	no	no	no	no	no	no	yes
182248058	chr1	216019395	216019420	11	26	2.363636	no	no	yes	no	no	no	no	no	no	yes
182248062	chr1	216029984	216030661	37	678	19.210526	no	no	yes	no	no	no	no	yes	no	yes
182248067	chr1	216044363	216044399	17	37	2.176471	no	no	yes	no	no	no	no	no	no	yes
182248077	chr1	216059106	216059143	17	38	2.235294	no	no	yes	no	no	no	no	no	no	yes
182248079	chr1	216059549	216059584	16	36	2.312500	no	no	yes	no	no	no	no	no	no	yes
182248080	chr1	216063115	216063145	16	31	1.937500	no	no	yes	no	no	no	no	yes	no	yes
182248090	chr1	216097844	216098139	48	296	6.166667	no	no	yes	no	no	no	no	yes	no	yes
182248097	chr1	216106536	216106581	20	46	2.350000	no	no	yes	no	no	no	no	yes	no	yes
182248104	chr1	216117850	216117915	30	66	2.200000	no	no	yes	no	no	no	no	no	no	yes
182248107	chr1	216119418	216119449	15	32	2.133333	no	no	yes	no	no	no	no	yes	no	yes
182248114	chr1	216132390	216132426	18	37	2.055556	no	no	yes	no	no	no	no	no	no	yes

182248121	chr1	216142049	216142088	18	40	2.277778	no	no	yes	no	no	no	no	no	no	yes
182248122	chr1	216144250	216144282	11	33	3.000000	no	no	yes	no	no	no	no	yes	no	yes
182248134	chr1	216191939	216191975	16	37	2.375000	no	no	yes	no	no	no	no	no	no	yes
182248142	chr1	216209533	216209587	27	55	2.037037	no	no	yes	no	no	no	no	no	no	yes
182248145	chr1	216227209	216227244	17	36	2.117647	no	no	yes	no	no	no	no	no	no	yes
182248155	chr1	216253868	216253913	23	46	2.000000	no	no	yes	no	no	no	no	no	no	yes
182248161	chr1	216280420	216280459	21	40	1.952381	no	no	yes	no	no	no	no	no	no	yes
182248168	chr1	216304061	216304105	24	45	1.958333	no	no	yes	no	no	no	no	no	no	yes
182248173	chr1	216326513	216326553	20	41	2.100000	no	no	yes	no	no	no	no	no	no	yes
182248174	chr1	216328819	216328855	18	37	2.055556	no	no	yes	no	no	no	no	no	no	yes
182248179	chr1	216359116	216359147	16	32	2.000000	no	no	yes	no	no	no	no	no	no	yes
182248180	chr1	216366590	216366627	19	38	2.000000	no	no	yes	no	no	no	no	no	no	yes
182248184	chr1	216377689	216377715	10	27	2.700000	no	no	yes	no	no	no	no	no	no	yes
182248201	chr1	216416968	216417001	17	34	2.000000	no	no	yes	no	no	no	no	yes	yes	yes
182248202	chr1	216419839	216419865	13	27	2.076923	no	no	yes	no	no	no	no	no	no	yes
182248221	chr1	216506447	216506513	35	67	1.914286	no	yes	no	no	no	yes	no	yes	no	yes
182248227	chr1	216515251	216515292	22	42	1.909091	no	no	yes	no	no	no	no	no	no	yes
182248236	chr1	216535798	216535843	24	46	1.916667	no	no	yes	no	no	no	no	yes	yes	yes
182248248	chr1	216573221	216573252	14	32	2.285714	no	no	yes	no	no	no	no	yes	no	yes
182248249	chr1	216579458	216579488	16	31	1.937500	no	no	yes	no	no	no	no	no	no	yes
182248256	chr1	216611596	216611634	21	39	1.952381	no	no	yes	no	no	no	no	no	no	yes
182248259	chr1	216618469	216618510	22	42	2.045455	no	no	yes	no	no	no	no	no	no	yes
182248260	chr1	216630052	216630092	21	41	1.952381	no	no	yes	no	no	no	no	no	no	yes
182248261	chr1	216630213	216630250	20	38	1.900000	no	no	yes	no	no	no	no	yes	no	yes
182248267	chr1	216658324	216658348	12	25	2.083333	no	no	yes	no	no	no	no	yes	no	yes

182248269	chr1	216665894	216665944	26	51	1.961538	no	no	yes	no	no	no	no	no	yes	yes
182248278	chr1	216693237	216693307	25	71	2.880000	no	no	yes	no	no	no	no	no	no	yes
182248281	chr1	216709292	216709354	30	63	2.100000	no	no	yes	no	no	no	no	yes	no	yes
182248282	chr1	216717178	216717249	35	72	2.057143	no	no	yes	no	no	no	no	no	no	yes
182248283	chr1	216720767	216720796	14	30	2.142857	no	no	yes	no	no	no	no	yes	no	yes
182248290	chr1	216732742	216732773	7	32	4.571429	no	no	yes	no	no	no	no	yes	no	yes
182248292	chr1	216741176	216741330	27	155	6.000000	no	no	yes	no	no	no	no	no	no	yes
182248297	chr1	216757783	216757810	12	28	2.333333	no	no	yes	no	no	no	no	no	no	yes
182248311	chr1	216798133	216798169	19	37	2.052632	no	no	yes	no	no	no	no	no	no	yes
182248340	chr1	216891432	216891463	16	32	2.000000	no	no	yes	no	no	no	no	no	no	yes
182248341	chr1	216893424	216893470	25	47	1.920000	no	no	yes	no	no	no	no	no	no	yes
182248351	chr1	216915369	216915405	16	37	2.375000	no	no	yes	no	no	no	no	yes	no	yes
182248357	chr1	216926856	216926898	7	43	6.142857	no	no	yes	no	no	no	no	no	yes	yes
182248368	chr1	216962559	216962601	12	43	3.583333	no	no	yes	no	no	no	no	yes	no	yes
182248370	chr1	216969410	216969438	12	29	2.416667	no	no	yes	no	no	no	no	no	no	yes
182248371	chr1	216969890	216969949	30	60	2.000000	no	no	yes	no	no	no	no	no	no	yes
182248372	chr1	216972059	216972090	16	32	2.000000	no	no	yes	no	no	no	no	no	no	yes
182248376	chr1	216983169	216983209	14	41	3.000000	no	no	yes	no	no	no	no	no	no	yes
182248379	chr1	216993761	216993816	28	56	2.000000	no	no	yes	no	no	no	no	no	no	yes
182248386	chr1	217003554	217003615	22	62	2.909091	no	no	yes	no	no	no	no	yes	no	yes
182248388	chr1	217006049	217006090	19	42	2.210526	no	no	yes	no	no	no	no	no	no	yes
182248391	chr1	217007715	217007757	20	43	2.200000	no	no	yes	no	no	no	no	no	no	yes
182248394	chr1	217014785	217014823	20	39	2.000000	no	no	yes	no	no	no	no	no	no	yes
182248410	chr1	217059029	217059073	20	45	2.238095	no	no	yes	no	no	no	no	no	no	yes
182248412	chr1	217059220	217059270	21	51	2.476191	no	no	yes	no	no	no	no	no	no	yes

182248415	chr1	217064170	217064209	21	40	1.952381	no	no	yes	no	no	no	no	no	no	yes
182248420	chr1	217070989	217071040	25	52	2.240000	no	no	yes	no	no	no	no	no	no	yes
182248422	chr1	217079119	217079161	12	43	3.583333	yes	no	yes	no	no	no	no	yes	yes	yes
182248442	chr1	217137253	217137318	30	66	2.200000	no	no	yes	no	no	no	no	yes	yes	yes
182248445	chr1	217156372	217156414	22	43	2.000000	no	no	no	no	no	no	no	yes	no	no
182248451	chr1	217162923	217162976	26	54	2.076923	no	no	no	no	no	no	no	no	no	no
182248458	chr1	217183357	217183382	13	26	2.000000	no	no	no	no	no	no	no	no	no	no
182248460	chr1	217194198	217194229	14	32	2.285714	no	no	no	no	no	no	no	no	no	no
182248471	chr1	217226657	217226682	13	26	2.000000	no	no	no	no	no	no	no	no	yes	no
182248479	chr1	217239075	217239232	59	158	2.688524	no	no	no	no	no	no	no	no	no	no
182248486	chr1	217240115	217240163	24	49	2.041667	no	no	no	no	no	no	no	no	no	no
182248489	chr1	217261391	217261424	17	34	2.117647	no	no	no	no	no	no	no	no	no	no
182248490	chr1	217261486	217261513	13	28	2.153846	no	no	no	no	no	no	no	no	no	no
182248495	chr1	217276138	217276189	21	52	2.666667	no	no	no	no	no	no	no	yes	yes	no
182248505	chr1	217316689	217316724	17	36	2.117647	no	no	no	no	no	no	no	no	no	no
182248506	chr1	217316744	217316801	28	58	2.107143	no	no	no	no	no	no	no	no	no	no
182248511	chr1	217325740	217325773	16	34	2.125000	no	no	no	no	no	no	no	no	yes	no
182248512	chr1	217336350	217336382	17	33	2.000000	no	no	no	no	no	no	no	yes	no	no
182248520	chr1	217352746	217352798	26	53	2.076923	no	no	no	no	no	no	no	no	no	no
182248534	chr1	217402999	217403030	16	32	2.000000	no	no	no	no	no	no	no	no	yes	no
182248535	chr1	217403315	217403351	15	37	2.500000	no	no	no	no	no	no	no	yes	no	no
182248537	chr1	217405938	217405974	12	37	3.250000	no	no	no	no	no	no	no	no	no	no
182248542	chr1	217406786	217406846	24	61	2.541667	no	no	no	no	no	no	no	no	no	no
182248545	chr1	217422346	217422378	17	33	1.941176	no	no	no	no	no	no	no	yes	yes	no
182248554	chr1	217453654	217453707	28	54	2.035714	no	no	yes	no	no	no	no	yes	no	yes

182248555	chr1	217458673	217458730	31	58	1.903226	no	no	yes	no	no	no	no	no	no	yes
182248570	chr1	217500154	217500214	30	61	2.066667	no	no	yes	no	no	no	no	no	no	yes
182248576	chr1	217514568	217514600	17	33	1.941176	no	no	yes	no	no	no	no	no	no	yes
182248592	chr1	217552158	217552208	24	51	2.125000	no	no	yes	no	no	no	no	yes	no	yes
182248594	chr1	217559073	217559101	12	29	2.416667	no	no	yes	no	no	no	no	no	no	yes
182248596	chr1	217561073	217561099	12	27	2.250000	no	no	yes	no	no	no	no	no	no	yes
182248606	chr1	217591198	217591279	27	82	2.965517	no	no	yes	no	no	no	no	yes	no	yes
182248607	chr1	217599796	217599839	23	44	1.913043	no	no	yes	no	no	no	no	no	no	yes
182248610	chr1	217629220	217629250	12	31	2.583333	no	no	yes	no	no	no	no	yes	no	yes
182248618	chr1	217640043	217640094	10	52	5.272727	no	no	yes	no	no	no	no	no	no	yes
182248635	chr1	217688512	217688545	17	34	2.000000	no	no	yes	no	no	no	no	no	no	yes
182248646	chr1	217701281	217701359	34	79	2.323529	no	no	yes	no	no	no	no	no	no	yes
182248655	chr1	217718681	217718719	16	39	2.437500	no	no	yes	no	no	no	no	yes	no	yes
182248661	chr1	217746044	217746100	24	57	2.360000	no	no	yes	no	no	no	no	no	no	yes
182248663	chr1	217746344	217746372	13	29	2.230769	no	no	yes	no	no	no	no	yes	no	yes
182248670	chr1	217760222	217760286	32	65	2.031250	no	no	yes	no	no	no	no	no	no	yes
182248676	chr1	217772888	217773097	19	210	11.052631	no	no	yes	no	no	no	no	no	no	yes
182248678	chr1	217773474	217773504	16	31	1.937500	no	no	yes	no	no	no	no	no	no	yes
182248683	chr1	217780100	217780132	8	33	4.375000	no	no	yes	no	no	no	no	yes	yes	yes
182248685	chr1	217781620	217781650	16	31	1.937500	no	no	yes	no	no	no	no	no	no	yes
182248688	chr1	217788133	217788210	39	78	2.000000	no	no	yes	no	no	no	no	no	no	yes
182248693	chr1	217812407	217812498	50	92	1.940000	no	no	yes	no	no	no	no	yes	no	yes
182248694	chr1	217813802	217813833	16	32	2.000000	no	no	yes	no	no	no	no	no	no	yes
182248700	chr1	217824905	217824958	25	54	2.160000	no	no	yes	no	no	no	no	no	no	yes
182248704	chr1	217830669	217830696	13	28	2.153846	no	no	yes	no	no	no	no	no	no	yes

182248705	chr1	217833357	217833398	21	42	2.095238	no	no	yes	no	no	no	no	yes	no	yes
182248707	chr1	217841269	217841297	15	29	2.000000	no	no	yes	no	no	no	no	no	no	yes
182248713	chr1	217859103	217859225	63	123	2.015873	no	no	yes	no	no	no	no	no	no	yes
182248717	chr1	217859326	217859362	16	37	2.375000	no	no	yes	no	no	no	no	no	no	yes
182248722	chr1	217871410	217871444	17	35	2.058824	no	yes	no	no	no	no	no	no	no	yes
182248724	chr1	217879285	217879313	15	29	1.933333	no	no	no	no	no	no	no	no	no	no
182248726	chr1	217883069	217883106	16	38	2.437500	no	no	no	no	no	no	no	no	no	no
182248732	chr1	217919609	217919651	21	43	2.047619	no	yes	yes	no	no	no	no	no	no	yes
182248733	chr1	217920892	217920920	14	29	2.071429	no	no	no	no	no	yes	no	no	no	no
182248735	chr1	217924738	217924792	27	55	2.037037	no	no	no	no	no	no	no	no	no	no
182248738	chr1	217938098	217938127	15	30	2.000000	no	no	no	no	no	no	no	no	no	no
182248743	chr1	217954063	217954113	23	51	2.217391	no	no	no	no	no	no	no	yes	yes	no
182248751	chr1	217983175	217983224	24	50	2.166667	no	no	no	no	no	no	no	yes	yes	no
182248753	chr1	217987376	217987438	26	63	2.392857	no	no	no	no	no	no	no	no	no	no
182248756	chr1	217995417	217995456	21	40	1.904762	no	no	no	no	no	no	no	no	no	no
182248764	chr1	218035126	218035159	18	34	1.944444	no	no	no	no	no	no	no	no	yes	no
182248773	chr1	218051995	218052032	20	38	1.900000	no	no	yes	no	no	no	no	yes	no	yes
182248780	chr1	218063109	218063148	21	40	2.000000	no	no	no	no	no	no	no	no	no	no
182248781	chr1	218066194	218066226	16	33	2.125000	no	no	no	no	no	no	no	no	no	no
182248791	chr1	218123575	218123733	87	159	1.827586	no	no	no	no	no	no	no	no	no	no
182248794	chr1	218127919	218127964	22	46	2.136364	no	no	no	no	no	no	no	yes	no	no
182248795	chr1	218127958	218128006	25	49	2.000000	no	no	no	no	no	no	no	yes	no	no
182248796	chr1	218129355	218129391	19	37	2.000000	yes	no	no	no	no	no	no	no	no	no
182248809	chr1	218152780	218152820	12	41	3.416667	no	no	no	no	no	no	no	no	no	no
182248810	chr1	218170764	218171033	145	270	1.875862	no	no	no	no	no	no	no	yes	no	no

182248817	chr1	218188883	218188920	16	38	2.375000	no	no	no	no	no	no	no	no	no	no
182248821	chr1	218192414	218192438	12	25	2.083333	no	no	no	no	no	no	no	no	no	no
182248822	chr1	218203445	218203612	84	168	2.000000	no	no	no	no	no	no	no	no	no	no
182248864	chr1	218345793	218345824	15	32	2.133333	yes	no	no	no	yes	no	yes	yes	yes	yes
182248869	chr1	218373380	218373476	36	97	2.641026	no	no	yes	no	no	no	no	yes	yes	yes
182248879	chr1	218379441	218379501	31	61	1.967742	no	no	yes	no	no	no	no	yes	no	yes
182248897	chr1	218440640	218440692	27	53	2.074074	no	no	yes	no	no	no	no	no	no	yes
182248898	chr1	218441390	218441418	12	29	2.416667	no	yes	no	no	no	no	no	yes	no	yes
182248902	chr1	218461729	218461832	41	104	2.619048	no	no	yes	no	no	no	no	no	no	yes
182248906	chr1	218461887	218461929	18	43	2.388889	no	no	yes	no	no	no	no	no	no	yes
182248907	chr1	218461925	218461966	21	42	2.000000	no	no	yes	no	no	no	no	no	no	yes
182248929	chr1	218501012	218501043	15	32	2.133333	no	no	yes	no	no	no	no	no	yes	yes
182248934	chr1	218520414	218520455	19	42	2.210526	no	no	yes	no	no	no	no	no	no	yes
182248936	chr1	218525720	218525767	25	48	1.920000	no	yes	no	no	no	no	no	yes	yes	yes
182248940	chr1	218542910	218542948	20	39	2.000000	no	no	no	no	no	no	no	no	no	no
182248952	chr1	218570062	218570102	16	41	2.562500	no	no	no	no	no	no	no	no	no	no
182248985	chr1	218620266	218620305	15	40	2.666667	no	no	no	no	no	no	no	no	no	no
182248995	chr1	218664660	218664705	22	46	2.136364	no	no	no	no	no	no	no	yes	no	no
182249006	chr1	218680395	218680419	10	25	2.500000	no	no	no	no	no	no	no	no	no	no
182249016	chr1	218714559	218714629	30	71	2.366667	no	no	no	no	no	no	no	no	no	no
182249025	chr1	218732197	218732234	18	38	2.111111	no	no	no	no	no	no	no	no	no	no
182249029	chr1	218774333	218774364	16	32	2.125000	no	no	no	no	no	no	no	no	no	no
182249031	chr1	218779738	218779772	18	35	2.055556	no	no	no	no	no	no	no	yes	yes	no
182249036	chr1	218792589	218792639	25	51	2.040000	no	no	no	no	no	no	no	no	no	no
182249042	chr1	218826451	218826502	18	52	2.888889	no	no	no	no	no	no	no	no	no	no

182249044	chr1	218833860	218833968	48	109	2.265306	no	no	no	no	no	no	no	no	no	no
182249047	chr1	218835389	218835487	48	99	2.062500	no	no	no	no	no	no	no	yes	no	no
182249056	chr1	218863225	218863268	16	44	2.705882	no	no	no	no	no	no	no	no	no	no
182249060	chr1	218868360	218868393	12	34	2.833333	no	no	no	no	no	no	no	yes	yes	no
182249062	chr1	218885275	218885316	21	42	2.000000	no	no	no	no	no	no	no	no	yes	no
182249063	chr1	218887710	218887738	12	29	2.416667	no	no	no	no	no	no	no	no	yes	no
182249066	chr1	218888892	218888936	18	45	2.666667	no	no	no	no	no	no	no	no	no	no
182249072	chr1	218894699	218894822	63	124	1.968254	no	no	no	no	no	no	no	no	yes	no
182249075	chr1	218906357	218906412	28	56	2.035714	no	no	no	no	no	no	no	no	no	no
182249076	chr1	218907449	218907486	20	38	1.900000	no	no	no	no	no	no	no	no	no	no
182249081	chr1	218923232	218923275	12	44	3.615385	no	no	no	no	no	no	no	no	no	no
182249082	chr1	218923901	218923934	15	34	2.266667	no	no	no	no	no	no	no	no	no	no
182249085	chr1	218926239	218926263	13	25	1.923077	no	no	no	no	no	no	no	yes	no	no
182249089	chr1	218929083	218929161	42	79	1.928571	no	no	no	no	no	no	no	no	no	no
182249100	chr1	218944975	218945008	16	34	2.125000	no	no	no	no	no	no	no	yes	no	no
182249104	chr1	218950437	218950477	19	41	2.157895	no	no	no	no	no	no	no	no	no	no
182249105	chr1	218955394	218955433	20	40	2.100000	no	no	no	no	no	no	no	yes	no	no
182249109	chr1	218959917	218959952	17	36	2.117647	no	no	no	no	no	no	no	yes	yes	no
182249110	chr1	218964873	218964910	20	38	1.900000	no	no	no	no	no	no	no	no	no	no
182249111	chr1	218982188	218982212	13	25	1.923077	no	no	no	no	no	yes	no	no	no	no
182249115	chr1	218991438	218991479	22	42	1.909091	no	no	yes	no	no	no	no	no	no	yes
182249147	chr1	219064157	219064201	10	45	4.636364	no	no	yes	no	no	no	no	yes	yes	yes
182249148	chr1	219065308	219065340	17	33	1.941176	no	no	yes	no	no	no	no	yes	no	yes
182249157	chr1	219076271	219076295	11	25	2.272727	no	no	yes	no	no	no	no	no	no	yes
182249175	chr1	219129869	219129915	20	47	2.350000	no	no	yes	no	no	no	no	no	no	yes

182249189	chr1	219201941	219202027	45	87	1.977778	no	no	yes	no	no	no	no	yes	yes	yes
182249191	chr1	219205575	219205616	16	42	2.687500	no	no	yes	no	no	no	no	no	no	yes
182249195	chr1	219230459	219230503	23	45	1.956522	no	no	no	no	no	no	no	no	no	no
182249200	chr1	219241656	219241684	14	29	2.071429	no	no	no	no	no	no	no	no	no	no
182249206	chr1	219245512	219245554	23	43	1.956522	no	no	no	no	no	no	no	no	yes	no
182249216	chr1	219275718	219275799	43	82	1.976744	no	no	no	no	no	no	no	no	no	no
182249217	chr1	219277413	219277439	12	27	2.250000	no	no	no	no	no	no	no	yes	yes	no
182249223	chr1	219291881	219291917	19	37	1.947368	no	no	no	no	no	no	no	no	no	no
182249227	chr1	219311019	219311062	22	44	2.045455	no	no	no	no	no	no	no	yes	yes	no
182249228	chr1	219313003	219313033	15	31	2.066667	no	no	no	no	no	no	no	yes	no	no
182249232	chr1	219326294	219326323	15	30	2.000000	no	no	no	no	no	no	no	yes	no	no
182249235	chr1	219338889	219338929	13	41	3.230769	no	no	no	no	no	no	no	no	no	no
182249239	chr1	219349620	219349656	16	37	2.437500	no	no	no	no	no	no	no	no	no	no
182249254	chr1	219394240	219394273	16	34	2.125000	no	no	no	no	no	no	no	no	no	no
182249264	chr1	219419624	219419659	15	36	2.400000	no	no	yes	no	no	no	no	no	no	yes
182249267	chr1	219424024	219424063	14	40	2.750000	no	no	yes	no	no	no	no	no	no	yes
182249275	chr1	219446974	219447018	16	45	2.764706	no	no	yes	no	no	no	no	no	no	yes
182249284	chr1	219472269	219472376	53	108	2.037736	no	no	no	no	no	no	no	no	no	no
182249287	chr1	219479683	219479726	22	44	2.090909	no	no	no	no	no	no	no	no	no	no
182249314	chr1	219563332	219563371	16	40	2.500000	no	no	no	no	no	no	no	no	no	no
182249318	chr1	219570151	219570201	23	51	2.304348	no	no	no	no	no	no	no	yes	no	no
182249322	chr1	219572534	219572575	12	42	3.500000	no	no	no	no	no	no	no	no	no	no
182249331	chr1	219589700	219589805	50	106	2.120000	no	no	no	no	no	no	no	no	yes	no
182249351	chr1	219653416	219653504	44	89	2.022727	no	no	no	no	no	no	no	yes	no	no
182249352	chr1	219655039	219655079	21	41	1.952381	no	no	no	no	no	no	no	no	no	no

182249356	chr1	219657975	219658012	16	38	2.375000	no	no	no	no	no	no	no	yes	no	no
182249358	chr1	219661303	219661350	22	48	2.181818	no	no	no	no	no	no	no	yes	yes	no
182249359	chr1	219662925	219662958	9	34	3.777778	no	no	no	no	no	no	no	yes	no	no
182249361	chr1	219665156	219665204	20	49	2.450000	no	no	no	no	no	no	no	yes	no	no
182249363	chr1	219680908	219680944	19	37	1.947368	no	no	no	no	no	no	no	no	no	no
182249364	chr1	219687006	219687037	16	32	2.000000	no	no	yes	no	no	no	no	no	yes	yes
182249366	chr1	219698696	219698726	15	31	2.133333	no	no	yes	no	no	no	no	no	no	yes
182249377	chr1	219746682	219746712	15	31	2.066667	no	no	yes	no	no	no	no	no	no	yes
182249378	chr1	219746907	219746944	18	38	2.166667	no	no	yes	no	no	no	no	no	no	yes
182249398	chr1	219821707	219821731	12	25	2.083333	no	no	yes	no	no	no	no	no	no	yes
182249402	chr1	219836708	219836738	15	31	2.066667	no	no	yes	no	no	no	no	yes	no	yes
182249408	chr1	219846092	219846134	22	43	1.954545	no	no	yes	no	no	no	no	no	no	yes
182249415	chr1	219901427	219901453	13	27	2.076923	no	no	yes	no	no	no	no	yes	no	yes
182249419	chr1	219917292	219917324	16	33	2.062500	no	no	yes	no	no	no	no	no	yes	yes
182249433	chr1	219935201	219935243	22	43	2.000000	no	no	yes	no	no	no	no	no	yes	yes
182249452	chr1	219971812	219971940	50	129	2.547170	no	no	yes	no	no	no	no	no	no	yes
182249453	chr1	219981685	219981719	18	35	1.944444	no	no	yes	no	no	yes	no	no	no	yes
182249455	chr1	219991316	219991348	16	33	2.062500	no	no	yes	no	no	no	no	yes	yes	yes
182249460	chr1	220006371	220006413	21	43	2.047619	no	no	yes	no	no	no	no	no	no	yes
182249473	chr1	220043209	220043246	9	38	4.300000	no	no	yes	no	no	no	no	yes	yes	yes
182249476	chr1	220046710	220046740	15	31	2.066667	yes	no	no	no	no	no	yes	yes	yes	no
182249478	chr1	220052693	220052762	36	70	1.972222	no	no	no	no	no	no	no	no	no	no
182249481	chr1	220063973	220064013	20	41	2.150000	no	no	yes	no	no	no	no	yes	no	yes
182249500	chr1	220087093	220087128	16	36	2.250000	no	no	yes	no	no	no	no	no	yes	yes
182249507	chr1	220100380	220100509	62	130	2.096774	no	no	yes	yes	no	no	no	no	no	yes

182249515	chr1	220125413	220125460	25	48	2.040000	no	no	yes	no	no	no	no	no	no	yes
182249526	chr1	220187090	220187145	27	56	2.074074	no	no	yes	no	no	no	no	no	no	yes
182249528	chr1	220226592	220226632	21	41	1.952381	no	no	yes	no	no	no	no	yes	yes	yes
182249529	chr1	220228595	220228630	18	36	2.055556	no	no	yes	no	no	no	no	no	no	yes
182249530	chr1	220229015	220229056	17	42	2.470588	no	no	yes	no	no	no	no	no	no	yes
182249531	chr1	220229230	220229290	32	61	1.906250	no	no	yes	no	no	no	no	no	no	yes
182249532	chr1	220233068	220233116	23	49	2.130435	no	no	yes	no	no	no	no	no	no	yes
182249533	chr1	220241699	220241738	18	40	2.222222	no	no	yes	no	no	no	no	no	no	yes
182249543	chr1	220265476	220265516	12	41	3.416667	no	no	yes	no	no	yes	no	no	no	yes
182249548	chr1	220288362	220288400	21	39	1.952381	no	no	no	no	no	no	no	no	no	no
182249550	chr1	220296096	220296131	13	36	2.769231	no	no	no	no	no	no	no	yes	no	no
182249552	chr1	220300502	220300545	12	44	3.750000	no	no	no	no	no	no	no	yes	yes	no
182249572	chr1	220327544	220327590	25	47	1.920000	no	no	no	no	no	no	no	yes	no	no
182249587	chr1	220377899	220377923	13	25	1.923077	no	no	no	no	no	no	no	no	no	no
182249597	chr1	220391186	220391229	23	44	1.956522	no	no	no	no	no	no	no	no	no	no
182249600	chr1	220396695	220396724	15	30	2.000000	no	no	no	no	no	no	no	no	no	no
182249605	chr1	220413908	220413961	27	54	2.000000	no	no	no	no	no	no	no	no	no	no
182249608	chr1	220422403	220422432	14	30	2.142857	no	no	no	no	no	no	no	yes	yes	no
182249609	chr1	220426586	220426865	149	280	1.906040	no	no	no	no	no	no	no	yes	no	no
182249613	chr1	220439912	220439946	18	35	1.944444	no	no	no	no	no	no	no	no	no	no
182249618	chr1	220447519	220447558	15	40	2.666667	yes	no	no	no	no	no	no	no	no	no
182249621	chr1	220447837	220447866	15	30	2.000000	yes	no	no	no	no	no	no	no	no	no
182249625	chr1	220459182	220459239	30	58	1.933333	no	no	no	no	no	no	no	yes	yes	no
182249648	chr1	220523357	220523389	17	33	1.941176	no	no	no	no	no	no	no	no	no	no
182249657	chr1	220542882	220542943	31	62	2.032258	no	no	yes	no	no	no	no	no	no	yes

182249667	chr1	220575617	220575657	18	41	2.277778	no	no	yes	no	no	no	no	no	no	yes
182249672	chr1	220588312	220588351	22	40	1.909091	no	no	yes	no	no	no	no	no	no	yes
182249673	chr1	220588500	220588531	15	32	2.200000	no	no	yes	no	no	no	no	no	no	yes
182249680	chr1	220604771	220604879	53	109	2.056604	no	no	yes	no	no	no	no	no	no	yes
182249688	chr1	220614555	220614596	22	42	1.909091	no	no	yes	no	no	no	no	no	no	yes
182249689	chr1	220618013	220618065	28	53	1.928571	no	no	yes	no	no	no	no	no	no	yes
182249693	chr1	220634891	220634920	14	30	2.142857	no	no	yes	no	no	no	no	no	no	yes
182249696	chr1	220665757	220665825	36	69	1.916667	no	no	no	no	no	no	no	no	no	no
182249699	chr1	220673671	220673729	26	59	2.269231	no	no	no	no	no	no	no	no	no	no
182249700	chr1	220675040	220675097	28	58	2.071429	no	no	no	no	no	no	no	no	no	no
182249709	chr1	220698275	220698300	12	26	2.166667	no	yes	no	no	no	no	no	no	no	yes
182249723	chr1	220735225	220735261	19	37	2.000000	no	no	no	no	no	no	no	no	no	no
182249728	chr1	220735305	220735369	30	65	2.225806	no	no	no	no	no	no	no	no	no	no
182249749	chr1	220781212	220781255	21	44	2.095238	no	no	yes	no	no	no	no	no	no	yes
182249750	chr1	220782245	220782279	18	35	1.944444	no	no	yes	no	no	no	no	no	no	yes
182249752	chr1	220789066	220789138	32	73	2.281250	no	no	yes	no	no	no	no	yes	yes	yes
182249755	chr1	220813765	220813793	15	29	2.000000	no	yes	yes	no	no	no	no	yes	no	yes
182249758	chr1	220820083	220820120	15	38	2.600000	no	no	yes	no	no	yes	no	no	no	yes
182249763	chr1	220835032	220835067	18	36	2.000000	no	no	yes	no	no	no	no	no	yes	yes
182249776	chr1	220871479	220871535	23	57	2.478261	no	no	yes	no	no	no	no	no	no	yes
182249779	chr1	220880229	220880270	15	42	2.800000	yes	yes	yes	yes	no	no	yes	yes	yes	yes
182249783	chr1	220888668	220888702	15	35	2.333333	no	no	no	no	no	no	no	yes	yes	no
182249784	chr1	220890840	220890876	15	37	2.533333	no	no	no	no	no	no	no	yes	yes	no
182249785	chr1	220893823	220893859	14	37	2.642857	no	no	no	no	no	no	no	yes	yes	no
182249787	chr1	220916330	220916391	29	62	2.137931	no	no	no	no	no	no	no	yes	no	no

182249788	chr1	220926942	220926981	17	40	2.352941	no	no	no	no	no	no	no	no	no	no
182249789	chr1	220931535	220931566	7	32	4.714286	no	no	no	no	no	no	no	no	no	no
182249822	chr1	220975159	220975207	26	49	1.923077	no	no	no	no	no	no	no	no	no	no
182249824	chr1	220975870	220975898	13	29	2.230769	no	no	no	no	no	no	no	no	no	no
182249829	chr1	220984800	220984842	21	43	2.095238	no	no	no	no	no	no	no	yes	no	no
182249831	chr1	220991782	220991813	16	32	2.125000	no	no	no	no	no	no	no	no	yes	no
182249833	chr1	220993698	220993774	33	77	2.333333	no	no	no	no	no	no	no	yes	no	no
182249845	chr1	221010464	221010515	26	52	2.038461	no	no	no	no	no	no	no	no	no	no
182249846	chr1	221015870	221015924	16	55	3.437500	no	no	no	no	no	no	no	yes	no	no
182249849	chr1	221020525	221020562	17	38	2.235294	no	no	no	no	no	no	no	no	no	no
182249863	chr1	221065437	221065491	28	55	2.000000	no	no	no	no	no	no	no	yes	no	no
182249869	chr1	221085231	221085282	23	52	2.260870	no	no	no	no	no	no	no	no	no	no
182249879	chr1	221101454	221101484	16	31	1.937500	no	no	no	no	no	no	no	no	yes	no
182249880	chr1	221103773	221103808	9	36	4.000000	no	no	no	no	no	no	no	no	no	no
182249881	chr1	221103872	221103918	22	47	2.136364	no	no	no	no	no	no	no	no	no	no
182249884	chr1	221104164	221104247	39	84	2.153846	no	no	no	no	no	no	no	no	no	no
182249891	chr1	221136427	221136471	16	45	2.812500	no	no	no	no	no	no	no	no	no	no
182249892	chr1	221143752	221143822	38	71	1.921053	no	no	no	no	no	no	no	no	yes	no
182249903	chr1	221160383	221160460	41	78	1.975610	no	no	no	no	no	no	no	no	no	no
182249909	chr1	221172803	221172840	20	38	1.900000	no	no	no	no	no	no	no	yes	yes	no
182249910	chr1	221173388	221173436	25	49	2.040000	no	no	no	no	no	no	no	yes	no	no
182249914	chr1	221185062	221185095	16	34	2.125000	no	no	no	no	no	no	no	no	no	no
182249917	chr1	221189270	221189311	15	42	2.800000	no	no	no	no	no	no	no	no	no	no
182249923	chr1	221216089	221216129	22	41	1.954545	no	no	no	no	no	no	no	no	no	no
182249924	chr1	221219832	221219875	23	44	1.913043	no	no	no	no	no	no	no	no	yes	no

182249926	chr1	221221678	221221783	52	106	2.038461	no	no	no	no	no	no	no	no	no	no
182249928	chr1	221230908	221230935	14	28	2.000000	no	no	no	no	no	no	no	yes	yes	no
182249929	chr1	221231367	221231391	13	25	1.923077	no	no	no	no	no	no	no	yes	no	no
182249938	chr1	221234565	221234601	19	37	1.947368	no	no	no	no	no	no	no	no	no	no
182249950	chr1	221268666	221269032	186	367	2.000000	no	no	no	no	no	no	no	yes	no	no
182249951	chr1	221269084	221269124	21	41	2.000000	no	no	no	no	no	no	no	no	no	no
182249958	chr1	221290964	221291107	74	144	1.945946	no	no	no	no	no	no	no	yes	yes	no
182249966	chr1	221303292	221303327	18	36	2.000000	no	no	no	no	no	no	no	yes	no	no
182249967	chr1	221306455	221306491	19	37	1.947368	no	no	no	no	no	no	no	no	no	no
182249973	chr1	221336497	221336544	23	48	2.130435	yes	no	no	no	no	no	no	yes	no	no
182249974	chr1	221338865	221338905	14	41	2.928571	no	no	no	no	no	no	no	no	no	no
182249981	chr1	221376237	221376274	18	38	2.111111	no	no	no	no	no	no	no	no	yes	no
182249982	chr1	221383022	221383055	17	34	2.058824	no	no	no	no	no	no	no	yes	no	no
182249985	chr1	221393026	221393057	16	32	2.000000	no	no	no	no	no	no	no	no	no	no
182249993	chr1	221410742	221410778	17	37	2.235294	no	no	no	no	no	no	no	yes	no	no
182249995	chr1	221416284	221416330	16	47	2.937500	no	no	no	no	no	no	no	yes	yes	no
182249996	chr1	221419545	221419570	13	26	2.000000	no	no	no	no	no	no	no	yes	yes	no
182250014	chr1	221474906	221474974	21	69	3.428571	no	no	no	no	no	no	no	no	no	no
182250015	chr1	221475146	221475191	21	46	2.190476	no	no	no	no	no	no	no	no	no	no
182250025	chr1	221496261	221496323	15	63	4.055555	no	no	no	no	no	no	no	no	no	no
182250036	chr1	221526775	221526944	33	170	5.166667	no	no	no	no	no	no	no	no	no	no
182250040	chr1	221533210	221533256	22	47	2.136364	no	no	no	no	no	no	no	no	no	no
182250043	chr1	221541750	221541818	36	69	1.944444	no	no	no	no	no	no	no	no	no	no
182250045	chr1	221546002	221546040	14	39	2.928571	no	no	no	no	no	no	no	no	no	no
182250063	chr1	221612283	221612408	41	126	3.073171	no	no	no	no	no	no	no	yes	yes	no

182250070	chr1	221634202	221634264	23	63	2.750000	no	no	no	no	no	no	no	yes	no	no
182250097	chr1	221699985	221700098	8	114	14.250000	no	no	no	no	no	no	no	yes	no	no
182250098	chr1	221700092	221700234	16	143	9.000000	no	no	no	no	no	no	no	yes	no	no
182250099	chr1	221703139	221703176	20	38	1.950000	no	no	yes	no	no	no	no	no	yes	yes
182250102	chr1	221712118	221712158	21	41	1.952381	yes	no	yes	no	no	no	no	yes	no	yes
182250106	chr1	221726198	221726237	19	40	2.105263	no	no	yes	no	no	no	no	yes	yes	yes
182250107	chr1	221730812	221730849	8	38	4.600000	no	no	yes	no	no	no	no	no	no	yes
182250109	chr1	221732304	221732348	21	45	2.142857	no	no	yes	no	no	no	no	yes	yes	yes
182250110	chr1	221741004	221741065	30	62	2.066667	no	no	yes	no	no	no	no	yes	yes	yes
182250116	chr1	221749666	221749696	15	31	2.066667	no	no	no	no	no	no	no	yes	no	no
182250117	chr1	221754282	221754378	49	97	2.020408	no	no	no	no	no	no	no	yes	yes	no
182250119	chr1	221766108	221766146	18	39	2.166667	no	no	no	no	no	no	no	yes	no	no
182250125	chr1	221773284	221773334	14	51	3.642857	no	no	no	no	no	no	no	yes	no	no
182250131	chr1	221792651	221792688	20	38	1.900000	no	no	no	no	no	no	no	no	yes	no
182250143	chr1	221843372	221843443	18	72	4.000000	no	no	no	no	no	no	no	yes	no	no
182250150	chr1	221858671	221858708	17	38	2.294118	no	no	no	no	no	no	no	no	no	no
182250157	chr1	221881312	221881353	7	42	5.777778	no	no	yes	no	no	no	no	no	no	yes
182250159	chr1	221885784	221885836	10	53	5.300000	no	no	yes	no	no	no	no	no	no	yes
182250166	chr1	221887024	221887055	16	32	2.000000	no	no	yes	no	no	no	no	yes	yes	yes
182250170	chr1	221897126	221897168	20	43	2.150000	no	no	yes	no	no	no	no	yes	yes	yes
182250171	chr1	221897849	221897892	23	44	2.000000	no	no	yes	no	no	no	no	yes	yes	yes
182250175	chr1	221905689	221905744	16	56	3.500000	no	no	yes	no	no	no	no	no	yes	yes
182250187	chr1	221941130	221941217	42	88	2.095238	no	no	yes	no	no	no	no	yes	no	yes
182250196	chr1	221971648	221971690	21	43	2.095238	no	no	yes	no	no	no	no	no	no	yes
182250218	chr1	222012328	222012367	21	40	2.000000	no	no	yes	no	no	no	no	no	no	yes

182250238	chr1	222076889	222076943	26	55	2.115385	no	no	no	no	no	no	no	no	no	no
182250255	chr1	222120953	222120993	20	41	2.050000	no	no	yes	no	no	no	no	no	no	yes
182250261	chr1	222137137	222137173	16	37	2.312500	no	no	yes	no	no	no	no	yes	yes	yes
182250272	chr1	222153783	222153827	16	45	2.823529	no	no	yes	no	no	no	no	yes	no	yes
182250283	chr1	222184687	222184718	16	32	2.000000	no	no	yes	no	no	no	no	no	no	yes
182250289	chr1	222196794	222196839	21	46	2.190476	no	no	yes	no	no	no	no	no	no	yes
182250292	chr1	222205374	222205408	18	35	2.000000	no	no	yes	no	no	no	no	no	no	yes
182250297	chr1	222211227	222211278	24	52	2.166667	no	no	yes	no	no	no	no	no	no	yes
182250300	chr1	222242171	222242204	16	34	2.125000	no	no	yes	no	no	no	no	no	no	yes
182250301	chr1	222242227	222242262	16	36	2.250000	no	no	yes	no	no	no	no	no	no	yes
182250305	chr1	222250216	222250245	15	30	2.066667	no	no	yes	no	no	no	no	no	no	yes
182250310	chr1	222271419	222271459	19	41	2.263158	no	no	yes	no	no	no	no	no	no	yes
182250312	chr1	222273001	222273044	22	44	2.000000	no	no	yes	no	no	no	no	no	no	yes
182250314	chr1	222282256	222282290	18	35	1.944444	no	no	yes	no	no	no	no	no	no	yes
182250315	chr1	222292768	222292799	15	32	2.133333	no	no	yes	no	no	no	no	no	no	yes
182250316	chr1	222302382	222302430	25	49	2.040000	no	no	yes	no	no	no	no	no	no	yes
182250317	chr1	222311442	222311495	28	54	1.928571	no	no	yes	no	no	no	no	no	yes	yes
182250318	chr1	222313076	222313116	20	41	2.050000	no	no	yes	no	no	no	no	no	no	yes
182250319	chr1	222313999	222314045	16	47	3.000000	no	no	yes	no	no	no	no	no	no	yes
182250322	chr1	222323407	222323443	19	37	1.947368	no	no	yes	no	no	no	no	no	no	yes
182250325	chr1	222332189	222332307	59	119	2.016949	no	no	yes	no	no	no	no	yes	no	yes
182250327	chr1	222333209	222333247	12	39	3.250000	no	no	yes	no	no	no	no	no	no	yes
182250333	chr1	222360531	222360588	30	58	1.966667	no	no	yes	no	no	no	no	yes	no	yes
182250339	chr1	222401158	222401204	23	47	2.043478	no	no	no	no	no	no	no	no	no	no
182250340	chr1	222404918	222404968	21	51	2.428571	no	no	no	no	no	no	no	yes	yes	no

182250347	chr1	222434798	222434839	21	42	2.095238	no	no	no	no	no	no	no	no	no	no
182250357	chr1	222471991	222474061	826	2071	2.547648	no	no	no	no	no	no	no	yes	yes	no
182250370	chr1	222486830	222486903	21	74	3.714286	yes	no	yes	no	no	no	no	no	no	yes
182250402	chr1	222523379	222523410	16	32	2.000000	no	no	yes	no	no	no	no	no	no	yes
182250405	chr1	222530495	222530530	18	36	2.000000	no	no	yes	no	no	no	no	no	no	yes
182250422	chr1	222558512	222558548	18	37	2.111111	no	yes	yes	no	no	no	no	no	no	yes
182250423	chr1	222558885	222559038	77	154	2.000000	no	no	yes	no	no	no	no	no	no	yes
182250425	chr1	222587648	222587754	54	107	1.981481	no	no	yes	no	no	no	no	no	no	yes
182250430	chr1	222598265	222598434	85	170	2.011765	no	no	no	no	no	no	no	no	no	no
182250435	chr1	222614151	222614214	19	64	3.368421	no	no	no	no	no	no	no	no	no	no
182250436	chr1	222616799	222616848	24	50	2.083333	no	no	no	no	no	no	no	yes	yes	no
182250437	chr1	222617486	222617513	13	28	2.153846	yes	no	no	no	no	no	no	yes	no	no
182250438	chr1	222617883	222618053	12	171	14.916667	yes	no	no	no	no	no	no	yes	yes	no
182250442	chr1	222618258	222618291	15	34	2.266667	no	no	yes	no	no	no	yes	yes	no	yes
182250446	chr1	222645523	222645550	12	28	2.333333	no	no	yes	no	no	no	no	no	no	yes
182250447	chr1	222654194	222654226	15	33	2.200000	no	no	yes	no	no	no	no	no	no	yes
182250448	chr1	222660450	222660476	14	27	1.928571	no	no	yes	no	yes	yes	no	no	no	yes
182250452	chr1	222676915	222676952	14	38	2.714286	yes	no	yes	no	no	no	no	no	no	yes
182250455	chr1	222682572	222682611	18	40	2.222222	no	no	yes	no	no	no	no	yes	no	yes
182250463	chr1	222690691	222690743	24	53	2.333333	no	no	yes	no	no	no	no	no	no	yes
182250464	chr1	222696136	222696175	13	40	3.076923	no	no	yes	no	no	no	no	no	no	yes
182250467	chr1	222712514	222712542	14	29	2.071429	yes	no	yes	no	yes	no	yes	yes	yes	yes
182250468	chr1	222715580	222715621	21	42	2.000000	no	no	yes	no	no	no	no	no	no	yes
182250471	chr1	222718774	222718873	50	100	2.040000	no	no	yes	no	no	no	no	no	yes	yes
182250481	chr1	222756910	222756952	17	43	2.529412	no	no	yes	no	no	no	no	no	no	yes

182250482	chr1	222758653	222758689	19	37	1.947368	no	no	yes	no	no	no	no	no	no	yes
182250490	chr1	222803320	222803347	14	28	2.000000	no	no	no	no	no	no	no	no	no	no
182250498	chr1	222814960	222814988	11	29	2.636364	yes	no	no	no	no	no	no	yes	yes	no
182250500	chr1	222818367	222818436	33	70	2.121212	no	no	yes	no	no	no	no	no	yes	yes
182250501	chr1	222829771	222829807	20	37	1.950000	no	no	yes	no	no	no	no	yes	no	yes
182250502	chr1	222847739	222847779	20	41	2.050000	no	no	yes	no	no	no	no	yes	no	yes
182250505	chr1	222852325	222852382	19	58	3.105263	no	no	yes	no	no	no	no	yes	no	yes
182250507	chr1	222853050	222853090	20	41	2.050000	no	no	yes	no	no	no	no	no	no	yes
182250516	chr1	222868445	222868518	36	74	2.083333	no	no	yes	no	no	no	no	no	no	yes
182250519	chr1	222878824	222878871	22	48	2.181818	no	no	yes	no	no	no	no	no	no	yes
182250523	chr1	222898118	222898162	21	45	2.142857	no	no	yes	no	no	no	no	yes	yes	yes
182250531	chr1	222912565	222912600	10	36	3.700000	no	no	yes	no	no	no	no	no	yes	yes
182250532	chr1	222928590	222928622	12	33	2.692308	no	no	yes	no	no	no	no	yes	no	yes
182250533	chr1	222934647	222934685	19	39	2.052632	no	no	yes	no	no	no	no	no	no	yes
182250543	chr1	222949487	222949524	20	38	1.900000	no	no	yes	no	no	no	no	no	no	yes
182250545	chr1	222951934	222951988	29	55	1.965517	no	no	yes	no	no	no	no	no	yes	yes
182250546	chr1	222952173	222952209	19	37	1.947368	no	no	yes	no	no	no	no	no	no	yes
182250547	chr1	222957906	222957937	16	32	2.000000	no	no	yes	no	no	no	no	no	no	yes
182250552	chr1	222966098	222966122	13	25	1.923077	no	no	yes	no	no	no	no	no	no	yes
182250570	chr1	222988566	222988634	34	69	2.058824	no	no	yes	no	no	no	no	yes	no	yes
182250571	chr1	222990565	222990646	42	82	1.976190	no	yes	yes	yes	no	no	no	no	no	yes
182250573	chr1	223008166	223008202	7	37	5.285714	no	no	no	no	no	no	no	no	yes	no
182250576	chr1	223022928	223022952	11	25	2.272727	no	no	no	no	no	no	no	yes	yes	no
182250580	chr1	223031358	223031513	79	156	1.974684	no	no	no	no	no	no	no	yes	yes	no
182250583	chr1	223038477	223038531	17	55	3.235294	no	no	no	no	no	no	no	yes	yes	no

182250593	chr1	223060234	223060258	12	25	2.083333	no	no	no	no	no	no	no	yes	no	no
182250604	chr1	223092415	223092453	11	39	3.545455	no	no	no	no	yes	no	no	no	no	yes
182250608	chr1	223103764	223103799	17	36	2.176471	yes	no	no	no	no	no	no	yes	no	no
182250610	chr1	223113787	223113818	16	32	2.000000	no	no	yes	no	no	no	no	no	no	yes
182250624	chr1	223137206	223137256	27	51	1.925926	no	no	yes	no	yes	no	no	no	no	yes
182250629	chr1	223143976	223144017	18	42	2.388889	yes	no	no	no	no	no	no	yes	yes	no
182250635	chr1	223160853	223160917	28	65	2.321429	no	no	no	no	no	no	no	yes	no	no
182250652	chr1	223189348	223189777	16	430	26.875000	no	no	no	no	no	no	no	yes	no	no
182250667	chr1	223199392	223199472	26	81	3.074074	no	no	no	no	no	no	no	yes	yes	no
182250668	chr1	223199472	223199527	29	56	2.000000	no	no	no	no	no	no	no	yes	no	no
182250676	chr1	223212239	223212271	16	33	2.062500	no	no	no	no	no	no	no	no	yes	no
182250678	chr1	223225560	223225593	17	34	2.000000	no	no	yes	no	no	no	no	no	no	yes
182250679	chr1	223225910	223225947	19	38	2.000000	no	no	yes	no	no	no	no	no	no	yes
182250681	chr1	223258175	223258207	16	33	2.062500	no	no	yes	no	no	no	no	no	yes	yes
182250685	chr1	223293648	223294034	214	387	1.845794	no	no	yes	no	no	no	no	yes	no	yes
182250689	chr1	223307640	223307679	19	40	2.105263	no	no	yes	no	no	no	no	no	no	yes
182250696	chr1	223323181	223323285	41	105	2.571429	no	no	yes	no	no	no	no	yes	no	yes
182250701	chr1	223329633	223329807	94	175	1.872340	no	no	yes	no	no	no	no	yes	no	yes
182250704	chr1	223329817	223329912	40	96	2.400000	no	no	yes	no	no	no	no	yes	no	yes
182250710	chr1	223352923	223352970	20	48	2.400000	no	no	yes	no	no	no	no	yes	no	yes
182250713	chr1	223364347	223364381	19	35	1.947368	yes	no	no	no	no	no	yes	yes	yes	no
182250724	chr1	223379115	223379297	91	183	2.021978	no	no	no	no	no	no	no	yes	no	no
182250725	chr1	223379760	223379802	21	43	2.047619	no	no	no	no	no	no	no	no	no	no
182250740	chr1	223476083	223476111	15	29	2.000000	no	no	no	no	no	no	no	no	no	no
182250742	chr1	223476637	223476681	18	45	2.526316	no	no	no	no	no	no	no	no	no	no

182250750	chr1	223487197	223487257	17	61	3.705882	no	no	no	no	no	no	no	no	yes	no
182250755	chr1	223497880	223497912	11	33	3.000000	no	no	no	no	no	no	no	no	no	no
182250764	chr1	223507860	223507903	23	44	1.913043	no	no	no	no	no	no	no	yes	no	no
182250769	chr1	223533927	223533978	28	52	1.928571	no	no	no	no	no	no	no	no	no	no
182250778	chr1	223547257	223547282	13	26	2.000000	no	no	yes	no	no	no	no	yes	no	yes
182250779	chr1	223555638	223555681	23	44	1.913043	no	no	yes	no	no	no	no	no	no	yes
182250780	chr1	223557254	223557291	13	38	3.000000	no	no	yes	no	no	no	no	yes	no	yes
182250784	chr1	223558786	223558831	24	46	1.916667	no	no	yes	no	no	no	no	yes	no	yes
182250786	chr1	223558828	223558868	17	41	2.388889	no	no	yes	no	no	no	no	yes	no	yes
182250788	chr1	223613822	223613862	19	41	2.157895	no	no	yes	no	no	no	no	no	no	yes
182250798	chr1	223635542	223636907	91	1366	15.010989	no	no	yes	no	no	no	no	yes	no	yes
182250799	chr1	223642213	223642300	46	88	1.934783	no	no	yes	no	no	no	no	yes	no	yes
182250800	chr1	223654129	223654182	26	54	2.076923	no	no	yes	no	no	no	no	yes	yes	yes
182250802	chr1	223655018	223655044	8	27	3.375000	yes	no	yes	no	no	no	no	yes	yes	yes
182250803	chr1	223655646	223655678	17	33	1.941176	no	no	yes	no	no	no	no	yes	no	yes
182250810	chr1	223671006	223671046	19	41	2.157895	no	no	no	no	no	no	no	yes	yes	no
182250838	chr1	223712900	223712933	17	34	2.000000	no	no	yes	no	no	no	yes	yes	yes	yes
182250847	chr1	223733959	223734013	18	55	3.055556	no	no	yes	no	no	no	no	no	yes	yes
182250851	chr1	223748636	223748740	51	105	2.058824	no	no	yes	no	no	no	yes	yes	yes	yes
182250852	chr1	223751101	223751129	14	29	2.071429	yes	no	yes	no	no	no	no	yes	no	yes
182250853	chr1	223753094	223753134	19	41	2.210526	no	no	yes	no	no	no	no	yes	no	yes
182250863	chr1	223761333	223761395	31	63	2.032258	no	no	yes	no	no	no	no	yes	no	yes
182250868	chr1	223776924	223776957	16	34	2.187500	no	no	no	no	no	yes	no	yes	yes	no
182250869	chr1	223781193	223781222	15	30	2.000000	no	no	yes	no	no	no	no	yes	yes	yes
182250877	chr1	223797735	223797767	16	33	2.062500	no	no	yes	no	no	no	no	no	no	yes

182250882	chr1	223817201	223817399	18	199	11.055555	no	no	yes	no	no	no	no	yes	yes	yes
182250887	chr1	223841459	223841531	37	73	1.972973	no	no	yes	no	no	no	no	yes	yes	yes
182250888	chr1	223845977	223846012	18	36	2.000000	yes	no	no	no	no	no	yes	yes	yes	no
182250894	chr1	223876519	223876846	28	328	11.931034	no	no	no	no	no	no	no	yes	no	no
182250903	chr1	223897950	223898004	16	55	3.437500	no	no	no	no	no	no	no	no	no	no
182250954	chr1	224011302	224011384	42	83	1.976190	no	no	no	no	no	yes	no	yes	no	no
182250963	chr1	224043255	224043313	29	59	2.068965	no	no	no	no	no	no	no	yes	no	no
182250969	chr1	224044009	224044056	24	48	2.083333	no	no	no	no	no	no	no	no	no	no
182251012	chr1	224108633	224108713	36	81	2.243243	no	no	no	no	no	no	no	no	no	no
182251052	chr1	224189480	224189516	15	37	2.600000	yes	no	yes	no	no	no	no	no	no	yes
182251053	chr1	224191059	224191088	9	30	3.333333	no	no	yes	no	no	no	no	no	no	yes
182251064	chr1	224204729	224204769	10	41	4.200000	no	no	no	no	no	no	no	yes	no	no
182251067	chr1	224210074	224210113	16	40	2.500000	no	no	yes	no	no	no	no	no	no	yes
182251089	chr1	224269567	224269605	19	39	2.105263	no	no	yes	no	no	no	no	yes	no	yes
182251099	chr1	224276912	224277015	21	104	5.000000	no	no	yes	no	no	no	no	yes	no	yes
182251100	chr1	224277072	224277243	93	172	1.849462	no	no	yes	no	no	no	no	yes	yes	yes
182251101	chr1	224277264	224277321	20	58	2.900000	no	no	yes	no	no	no	no	yes	yes	yes
182251106	chr1	224296451	224296495	19	45	2.473684	no	no	yes	no	no	no	no	no	no	yes
182251132	chr1	224378517	224378919	49	403	8.224490	no	yes	yes	no	no	no	no	yes	yes	yes
182251138	chr1	224400092	224400127	18	36	2.111111	no	no	yes	no	no	no	no	yes	yes	yes
182251145	chr1	224414454	224414509	19	56	3.157895	no	no	yes	no	no	no	no	no	no	yes
182251147	chr1	224414499	224414527	15	29	2.000000	no	no	yes	no	no	no	no	no	no	yes
182251149	chr1	224434035	224434075	21	41	1.952381	yes	no	yes	yes	no	no	no	yes	yes	yes
182251152	chr1	224434464	224434515	22	52	2.409091	yes	no	yes	no	yes	no	no	yes	yes	yes
182251155	chr1	224434525	224434559	15	35	2.333333	yes	no	yes	no	yes	no	no	yes	yes	yes

182251174	chr1	224485443	224485480	19	38	2.000000	no	no	yes	no	no	no	no	yes	no	yes
182251175	chr1	224487871	224487907	19	37	1.947368	no	no	yes	no	no	no	no	no	no	yes
182251184	chr1	224509319	224509378	24	60	2.541667	no	no	yes	no	no	no	no	no	no	yes
182251189	chr1	224531278	224531316	17	39	2.294118	no	no	yes	no	no	no	no	no	no	yes
182251203	chr1	224549080	224549105	13	26	2.000000	no	no	yes	no	no	no	no	yes	no	yes
182251208	chr1	224549240	224549518	140	279	2.014286	no	no	yes	no	no	no	no	no	no	yes
182251209	chr1	224549635	224549802	59	168	2.847458	no	no	yes	no	no	no	no	no	no	yes
182251211	chr1	224549877	224550017	72	141	1.958333	no	no	yes	no	no	no	no	no	no	yes
182251216	chr1	224550351	224550463	57	113	1.982456	no	no	yes	no	no	no	no	no	no	yes
182251218	chr1	224551437	224551620	73	184	2.547945	no	no	yes	no	no	no	no	yes	no	yes
182251219	chr1	224551573	224551696	59	124	2.101695	no	no	yes	no	no	no	no	yes	no	yes
182251221	chr1	224551834	224552018	77	185	2.402597	no	no	yes	no	no	no	no	no	no	yes
182251226	chr1	224552909	224553137	78	229	2.901235	no	no	yes	no	no	no	no	no	no	yes
182251228	chr1	224553165	224553336	92	172	1.869565	no	no	yes	no	no	no	no	no	no	yes
182251230	chr1	224573706	224573742	17	37	2.235294	yes	no	yes	no	no	no	no	no	no	yes
182251238	chr1	224617544	224617575	15	32	2.133333	no	no	yes	no	no	no	yes	yes	yes	yes
182251243	chr1	224649932	224649993	27	62	2.333333	no	no	yes	no	no	no	no	no	yes	yes
182251251	chr1	224698897	224698934	18	38	2.111111	no	no	yes	no	no	no	no	yes	no	yes
182251252	chr1	224699975	224700080	43	106	2.488372	no	no	yes	no	no	no	no	no	no	yes
182251257	chr1	224708217	224708252	17	36	2.117647	no	no	yes	no	no	no	no	yes	yes	yes
182251258	chr1	224709149	224709184	18	36	2.000000	no	no	yes	no	no	no	no	no	no	yes
182251265	chr1	224753290	224753356	33	67	2.030303	no	no	no	no	no	no	no	yes	no	no
182251271	chr1	224789219	224789268	16	50	3.176471	no	no	no	no	no	no	no	yes	no	no
182251272	chr1	224793349	224793396	24	48	2.000000	no	no	no	no	no	no	no	yes	yes	no
182251274	chr1	224809547	224809575	15	29	1.933333	no	no	yes	no	no	no	no	no	yes	yes

182251284	chr1	224852945	224853033	8	89	11.125000	no	no	yes	no	no	no	no	yes	yes	yes
182251285	chr1	224858778	224858825	25	48	2.000000	no	no	yes	no	no	no	no	no	no	yes
182251302	chr1	224918132	224918273	71	142	2.000000	no	no	yes	no	no	no	no	no	no	yes
182251310	chr1	224966501	224966525	12	25	2.083333	yes	no	yes	no	no	no	no	no	no	yes
182251311	chr1	224971114	224971150	15	37	2.466667	no	no	yes	no	no	no	no	no	no	yes
182251314	chr1	224982399	224982478	31	80	2.612903	no	no	yes	no	no	no	no	yes	no	yes
182251321	chr1	225003804	225003858	28	55	1.964286	no	no	yes	no	no	no	no	no	no	yes
182251331	chr1	225023255	225023298	19	44	2.368421	no	no	yes	no	no	no	no	yes	no	yes
182251332	chr1	225024430	225024519	45	90	2.022222	no	yes	yes	no	no	yes	no	no	no	yes
182251350	chr1	225073633	225073707	30	75	2.666667	no	no	yes	no	no	no	no	yes	no	yes
182251355	chr1	225077852	225077885	17	34	2.000000	no	no	yes	no	no	no	no	no	no	yes
182251358	chr1	225082567	225082608	21	42	2.000000	no	no	yes	yes	no	no	no	no	no	yes
182251359	chr1	225083731	225083766	17	36	2.117647	no	no	yes	no	no	no	no	no	no	yes
182251374	chr1	225152985	225153041	26	57	2.192308	no	no	yes	no	no	no	no	no	no	yes
182251382	chr1	225165989	225166043	27	55	2.037037	no	no	yes	no	no	no	no	no	no	yes
182251391	chr1	225208145	225208208	31	64	2.064516	no	no	yes	no	no	no	no	yes	no	yes
182251398	chr1	225240295	225240337	21	43	2.047619	yes	no	yes	no	no	no	no	no	no	yes
182251399	chr1	225252466	225252520	27	55	2.037037	no	no	yes	no	no	no	no	no	no	yes
182251403	chr1	225263192	225263264	34	73	2.147059	no	no	yes	no	no	no	no	yes	no	yes
182251404	chr1	225266518	225266582	32	65	2.062500	no	no	yes	no	no	no	no	no	no	yes
182251405	chr1	225272768	225272799	17	32	1.941176	no	no	yes	no	no	no	no	no	no	yes
182251413	chr1	225277075	225277136	22	62	2.909091	no	no	yes	no	no	no	no	no	no	yes
182251423	chr1	225301304	225301330	12	27	2.250000	no	no	yes	no	no	no	no	no	no	yes
182251428	chr1	225302089	225302152	29	64	2.206897	no	no	yes	no	no	no	no	no	no	yes
182251430	chr1	225308002	225308038	17	37	2.235294	no	no	yes	no	no	no	no	no	yes	yes

182251431	chr1	225308105	225308157	26	53	2.076923	no	no	yes	no	no	no	no	no	no	yes
182251436	chr1	225320719	225320751	16	33	2.062500	no	no	yes	no	no	no	no	no	no	yes
182251447	chr1	225335182	225335240	26	59	2.269231	no	no	yes	no	no	no	no	yes	yes	yes
182251449	chr1	225335213	225335499	96	287	2.990000	no	no	yes	no	no	no	no	yes	yes	yes
182251464	chr1	225343004	225343038	18	35	1.944444	no	no	yes	no	no	no	no	no	yes	yes
182251466	chr1	225345774	225345799	13	26	2.000000	no	no	yes	no	no	no	no	no	no	yes
182251468	chr1	225359481	225359519	19	39	2.052632	no	no	yes	no	no	no	no	no	no	yes
182251474	chr1	225374177	225374214	19	38	2.000000	no	no	yes	no	no	no	no	yes	no	yes
182251479	chr1	225407794	225407843	24	50	2.083333	no	no	yes	no	no	no	no	yes	no	yes
182251480	chr1	225412310	225412350	20	41	2.100000	yes	no	yes	no	no	no	no	no	no	yes
182251482	chr1	225427780	225427816	16	37	2.312500	no	no	yes	no	yes	no	yes	yes	yes	yes
182251494	chr1	225486000	225486034	17	35	2.058824	no	no	no	no	no	yes	no	yes	no	no
182251497	chr1	225489565	225489611	15	47	3.187500	no	yes	no	no	no	no	no	no	no	yes
182251500	chr1	225496318	225496347	15	30	2.000000	no	yes	no	no	yes	yes	no	yes	no	yes
182251502	chr1	225509062	225509093	16	32	2.000000	yes	no	yes	no	no	no	no	yes	no	yes
182251503	chr1	225510232	225510269	19	38	2.000000	no	no	yes	no	no	no	no	no	no	yes
182251507	chr1	225519328	225519406	18	79	4.388889	no	no	no	yes	yes	yes	no	yes	yes	yes
182251513	chr1	225522411	225522449	18	39	2.166667	no	no	yes	no	no	no	no	yes	no	yes
182251514	chr1	225535197	225535233	15	37	2.533333	no	no	yes	no	no	no	no	no	no	yes
182251516	chr1	225544833	225544872	14	40	2.933333	no	no	yes	no	no	no	no	no	no	yes
182251528	chr1	225577894	225577925	16	32	2.000000	no	no	yes	no	no	no	no	no	no	yes
182251539	chr1	225593072	225593107	17	36	2.117647	no	no	yes	no	no	no	no	yes	no	yes
182251546	chr1	225610042	225610081	21	40	1.952381	no	no	yes	no	no	no	no	yes	yes	yes
182251555	chr1	225616560	225616596	19	37	1.947368	no	no	yes	no	no	no	no	no	no	yes
182251556	chr1	225619214	225619252	20	39	1.950000	no	no	yes	no	no	no	no	no	no	yes

182251558	chr1	225627878	225627950	33	73	2.212121	no	no	yes	no	no	no	no	yes	no	yes
182251563	chr1	225647823	225647887	34	65	1.911765	no	no	yes	no	no	no	no	no	yes	yes
182251576	chr1	225668158	225668281	67	124	1.925373	no	no	no	no	no	no	no	yes	no	no
182251587	chr1	225693229	225693326	26	98	3.769231	no	no	no	no	no	no	no	yes	yes	no
182251595	chr1	225707105	225707283	30	179	5.966667	no	no	yes	no	no	no	no	yes	yes	yes
182251600	chr1	225712050	225712091	19	42	2.210526	no	no	yes	no	no	no	no	no	no	yes
182251656	chr1	225823652	225823682	16	31	1.937500	no	no	yes	no	no	no	no	yes	no	yes
182251669	chr1	225845467	225845509	22	43	1.954545	no	yes	yes	no	no	yes	no	yes	no	yes
182251670	chr1	225848951	225848992	21	42	2.047619	yes	no	no	yes	yes	no	no	yes	no	yes
182251672	chr1	225849654	225849807	84	154	1.833333	yes	no	yes	no	no	no	no	yes	yes	yes
182251674	chr1	225859108	225859136	12	29	2.500000	no	no	yes	no	no	yes	no	no	no	yes
182251685	chr1	225888339	225888387	21	49	2.333333	no	no	yes	no	no	no	no	yes	yes	yes
182251686	chr1	225889185	225889227	11	43	3.833333	yes	no	yes	no	no	no	no	yes	yes	yes
182251690	chr1	225892783	225892843	30	61	2.066667	no	no	yes	no	no	no	no	no	no	yes
182251702	chr1	225911862	225911898	18	37	2.055556	yes	no	yes	no	no	no	no	yes	yes	yes
182251706	chr1	225920372	225920410	20	39	2.000000	no	yes	yes	no	yes	yes	no	yes	no	yes
182251707	chr1	225920754	225920785	14	32	2.285714	no	no	yes	no	no	yes	no	yes	yes	yes
182251709	chr1	225924685	225924736	22	52	2.347826	yes	no	no	no	no	no	yes	yes	yes	no
182251715	chr1	225928573	225928627	28	55	1.964286	no	no	no	no	no	no	no	yes	yes	no
182251727	chr1	225943065	225943229	78	165	2.115385	no	no	no	no	no	no	no	yes	no	no
182251728	chr1	225944949	225945029	42	81	2.000000	no	no	no	no	no	no	no	yes	yes	no
182251779	chr1	226062525	226062561	20	37	1.950000	yes	no	yes	no	no	no	yes	yes	yes	yes
182251796	chr1	226098275	226098309	18	35	1.944444	no	no	no	no	no	no	no	yes	yes	no
182251805	chr1	226109598	226109634	18	37	2.055556	no	no	no	no	no	no	yes	yes	yes	no
182251809	chr1	226110257	226110297	21	41	1.952381	no	no	no	no	no	no	yes	yes	yes	no

182251810	chr1	226110590	226110625	18	36	2.000000	no	no	no	no	no	no	yes	yes	yes	no
182251811	chr1	226111694	226111738	21	45	2.142857	no	no	no	no	no	no	no	yes	yes	no
182251823	chr1	226118618	226118649	16	32	2.062500	no	no	no	no	no	no	no	yes	no	no
182251831	chr1	226134204	226134406	23	203	9.086957	no	no	no	no	no	no	no	no	no	no
182251847	chr1	226161546	226161632	46	87	1.934783	no	no	no	yes	yes	no	no	no	no	yes
182251859	chr1	226199996	226200031	10	36	3.600000	no	no	no	no	no	no	no	yes	no	no
182251870	chr1	226222171	226222208	20	38	1.900000	no	no	no	no	no	no	no	no	no	no
182251871	chr1	226223264	226223300	16	37	2.312500	yes	no	no	no	no	no	no	yes	yes	no
182251873	chr1	226228837	226228874	19	38	2.000000	no	no	no	no	no	no	no	yes	no	no
182251874	chr1	226230931	226231026	34	96	2.717949	no	no	no	no	no	yes	no	no	no	no
182251890	chr1	226276696	226276720	13	25	1.923077	no	no	yes	no	no	no	no	yes	no	yes
182251895	chr1	226284884	226284915	12	32	2.666667	no	no	yes	no	no	no	no	no	no	yes
182251901	chr1	226298995	226299038	18	44	2.555556	no	no	yes	no	no	no	no	no	no	yes
182251902	chr1	226300945	226300992	25	48	2.040000	no	no	yes	no	no	no	no	no	no	yes
182251911	chr1	226318257	226318283	13	27	2.076923	no	no	no	no	yes	no	no	yes	no	yes
182251931	chr1	226350675	226350734	31	60	1.967742	no	no	no	no	no	no	no	yes	yes	no
182251932	chr1	226361597	226361741	78	145	1.858974	no	no	yes	no	yes	yes	no	no	no	yes
182251953	chr1	226438520	226438554	18	35	1.944444	yes	no	no	no	no	no	no	yes	no	no
182251957	chr1	226454482	226454524	20	43	2.150000	no	no	no	no	no	no	no	yes	yes	no
182251981	chr1	226509023	226509086	34	64	1.911765	no	no	no	no	no	no	no	no	no	no
182251985	chr1	226511344	226511372	15	29	1.933333	no	no	no	no	no	no	no	yes	no	no
182251986	chr1	226511670	226511723	29	54	1.931034	no	no	no	no	no	no	no	yes	no	no
182252000	chr1	226567823	226567861	17	39	2.294118	no	no	yes	no	no	no	no	no	no	yes
182252004	chr1	226587564	226587602	21	39	1.904762	no	no	yes	no	no	no	no	no	no	yes
182252007	chr1	226622680	226622825	19	146	7.684210	no	no	no	no	no	no	no	no	no	no

182252014	chr1	226632508	226632541	13	34	2.615385	no	yes	no	no	no	no	no	no	no	yes
182252021	chr1	226636910	226636983	29	74	2.620690	no	no	yes	no	no	no	no	yes	yes	yes
182252023	chr1	226636952	226637090	37	139	3.707317	no	no	yes	no	no	no	no	yes	yes	yes
182252024	chr1	226643803	226643837	18	35	1.944444	no	no	yes	no	no	no	no	yes	yes	yes
182252027	chr1	226649336	226649368	17	33	1.941176	no	no	yes	no	no	no	no	yes	no	yes
182252039	chr1	226660633	226660688	23	56	2.434783	yes	no	yes	no	no	no	no	yes	yes	yes
182252041	chr1	226667932	226667975	12	44	3.666667	yes	no	yes	no	no	no	no	yes	yes	yes
182252042	chr1	226669782	226669812	16	31	1.937500	no	no	yes	no	no	no	no	no	no	yes
182252050	chr1	226687120	226687148	15	29	1.933333	no	no	yes	no	no	no	no	yes	no	yes
182252051	chr1	226688114	226688149	18	36	2.000000	no	no	yes	no	no	no	no	yes	yes	yes
182252056	chr1	226696547	226696587	20	41	2.100000	no	no	yes	no	no	no	no	no	no	yes
182252061	chr1	226708216	226708263	20	48	2.380952	no	no	yes	no	no	no	no	yes	no	yes
182252062	chr1	226711056	226711094	15	39	2.666667	no	no	yes	no	no	no	no	yes	no	yes
182252065	chr1	226712966	226713008	16	43	2.687500	no	no	yes	no	no	no	no	yes	yes	yes
182252067	chr1	226716354	226716402	26	49	1.961538	no	no	yes	no	no	no	no	yes	no	yes
182252076	chr1	226733154	226733185	12	32	2.666667	no	no	yes	no	no	no	no	yes	yes	yes
182252078	chr1	226740898	226740940	20	43	2.200000	no	no	no	no	no	no	no	yes	no	no
182252079	chr1	226741746	226741830	42	85	2.047619	no	no	no	no	no	no	no	no	no	no
182252083	chr1	226751688	226751721	12	34	2.769231	no	no	no	no	no	no	no	no	no	no
182252103	chr1	226785591	226785638	23	48	2.130435	no	no	no	no	no	no	no	yes	no	no
182252105	chr1	226787067	226787100	11	34	3.090909	no	no	no	no	no	no	no	yes	no	no
182252116	chr1	226797603	226798412	39	810	21.076923	no	no	no	no	no	no	no	yes	yes	no
182252118	chr1	226799548	226799584	18	37	2.111111	no	no	no	no	no	no	no	yes	yes	no
182252121	chr1	226819665	226819760	48	96	2.000000	no	no	no	no	no	no	no	yes	yes	no
182252122	chr1	226827330	226827370	21	41	1.952381	yes	no	no	no	no	no	no	no	yes	no

182252123	chr1	226827914	226827954	12	41	3.384615	no	no	yes	no	no	no	no	yes	no	yes
182252139	chr1	226867750	226867799	25	50	2.000000	no	no	no	no	no	no	no	yes	yes	no
182252141	chr1	226870546	226870587	22	42	1.909091	yes	no	yes	no	no	no	yes	yes	yes	yes
182252143	chr1	226871005	226871037	17	33	1.941176	yes	yes	yes	no	no	no	yes	yes	yes	yes
182252144	chr1	226877921	226877954	17	34	2.000000	no	no	yes	no	no	no	no	yes	yes	yes
182252146	chr1	226880493	226880633	22	141	6.230769	yes	no	yes	no	yes	no	no	yes	yes	yes
182252147	chr1	226883426	226883466	20	41	2.050000	no	no	yes	no	no	no	no	yes	no	yes
182252159	chr1	226959598	226959639	17	42	2.470588	no	no	yes	no	no	no	no	yes	yes	yes
182252163	chr1	226963223	226963256	17	34	2.000000	no	no	yes	no	no	no	no	no	yes	yes
182252164	chr1	226968822	226968867	25	46	1.920000	no	no	yes	no	no	no	no	yes	no	yes
182252165	chr1	226972256	226972285	15	30	2.000000	no	no	yes	no	no	no	no	yes	no	yes
182252170	chr1	226976090	226976448	151	359	2.418301	yes	no	yes	no	no	no	yes	yes	no	yes
182252173	chr1	226977826	226977866	21	41	1.952381	no	no	yes	no	no	yes	no	yes	no	yes
182252177	chr1	226978124	226978171	24	48	2.000000	no	no	yes	no	no	yes	no	yes	no	yes
182252185	chr1	226978649	226978780	71	132	1.859155	no	no	yes	no	no	no	no	yes	no	yes
182252188	chr1	226978716	226978888	58	173	2.919355	no	no	yes	no	no	no	no	yes	yes	yes
182252189	chr1	226978929	226978975	24	47	1.958333	no	no	yes	no	no	no	no	yes	yes	yes
182252194	chr1	226991953	226992047	50	95	1.980000	yes	yes	no	no	no	no	no	yes	no	yes
182252206	chr1	227048532	227048575	19	44	2.315789	no	no	yes	no	no	no	no	no	no	yes
182252207	chr1	227048887	227048946	27	60	2.296296	no	no	yes	no	no	no	no	no	no	yes
182252208	chr1	227054796	227054833	20	38	1.950000	no	no	yes	no	no	no	no	no	no	yes
182252211	chr1	227063087	227063138	18	52	2.944444	no	no	yes	no	no	no	no	no	no	yes
182252212	chr1	227063827	227063864	19	38	2.000000	no	no	yes	no	no	no	no	no	no	yes
182252215	chr1	227072983	227073014	15	32	2.133333	no	no	yes	no	no	no	no	yes	yes	yes
182252218	chr1	227088581	227088622	20	42	2.200000	no	no	yes	no	no	no	no	no	no	yes

182252222	chr1	227124371	227124406	17	36	2.117647	no	yes	yes	no	no	no	no	no	no	yes
182252224	chr1	227124408	227124444	17	37	2.176471	no	yes	yes	no	no	no	no	no	no	yes
182252225	chr1	227124562	227124600	18	39	2.166667	no	yes	yes	no	no	no	no	yes	yes	yes
182252226	chr1	227125883	227125929	19	47	2.526316	no	no	yes	no	no	no	no	no	no	yes
182252248	chr1	227156419	227156490	32	72	2.250000	no	no	yes	no	no	no	no	no	no	yes
182252250	chr1	227162862	227162941	34	80	2.352941	no	no	yes	no	no	no	no	no	no	yes
182252255	chr1	227162972	227162999	12	28	2.333333	no	no	yes	no	no	no	no	no	no	yes
182252259	chr1	227163035	227163217	46	183	4.060000	no	no	yes	no	no	no	no	no	no	yes
182252261	chr1	227164687	227164729	16	43	2.687500	no	no	yes	no	no	no	no	no	no	yes
182252270	chr1	227206259	227206352	47	94	2.000000	no	no	yes	no	no	no	no	no	no	yes
182252277	chr1	227213413	227213490	25	78	3.120000	no	no	yes	no	no	no	no	no	no	yes
182252286	chr1	227235390	227235446	26	57	2.192308	no	no	yes	no	no	yes	no	yes	no	yes
182252294	chr1	227259755	227259782	14	28	2.000000	no	no	yes	no	no	no	no	yes	no	yes
182252295	chr1	227263278	227263315	17	38	2.235294	no	no	yes	no	no	no	no	no	no	yes
182252314	chr1	227287592	227287624	17	33	2.000000	no	no	yes	no	no	no	no	no	no	yes
182252320	chr1	227311795	227311842	25	48	1.920000	no	no	yes	no	no	no	no	yes	yes	yes
182252321	chr1	227316036	227316061	13	26	2.000000	no	no	yes	no	no	no	no	yes	no	yes
182252323	chr1	227317634	227317672	21	39	1.904762	yes	no	no	no	yes	no	no	yes	yes	yes
182252324	chr1	227328864	227328907	22	44	2.000000	no	no	no	no	no	no	no	yes	yes	no
182252344	chr1	227366182	227366288	24	107	4.458333	no	no	no	no	no	no	no	yes	no	no
182252364	chr1	227395012	227395059	11	48	4.333333	no	no	yes	no	no	no	no	yes	no	yes
182252367	chr1	227410555	227410586	8	32	4.000000	no	no	yes	no	no	yes	no	yes	no	yes
182252376	chr1	227428115	227428150	19	36	1.947368	no	no	yes	no	no	no	no	no	yes	yes
182252377	chr1	227429140	227429175	10	36	3.454545	no	no	yes	no	no	no	no	yes	no	yes
182252382	chr1	227437305	227437534	46	230	4.920000	no	no	no	no	no	no	no	yes	no	no

182252384	chr1	227446264	227446292	15	29	1.933333	no	no	no	no	no	no	no	no	no	no
182252385	chr1	227448208	227448248	18	41	2.277778	no	no	no	no	no	no	no	yes	yes	no
182252391	chr1	227467883	227467912	16	30	1.937500	no	no	no	no	no	no	no	no	no	no
182252403	chr1	227535107	227535152	17	46	2.823529	no	no	no	no	no	no	no	no	no	no
182252405	chr1	227538689	227538721	17	33	1.941176	no	no	no	no	no	no	no	yes	no	no
182252406	chr1	227547759	227547799	17	41	2.411765	no	no	no	no	no	no	no	yes	no	no
182252407	chr1	227549077	227549120	20	44	2.200000	no	no	no	no	no	no	no	yes	no	no
182252408	chr1	227556148	227556188	12	41	3.266667	no	no	no	no	no	no	no	no	no	no
182252416	chr1	227567679	227567723	14	45	3.133333	no	no	yes	no	no	no	no	no	no	yes
182252418	chr1	227571124	227571165	19	42	2.210526	no	no	yes	no	no	no	no	yes	no	yes
182252430	chr1	227597763	227597812	22	50	2.272727	no	no	yes	no	no	yes	no	no	no	yes
182252433	chr1	227607661	227607699	20	39	1.950000	no	no	yes	no	no	no	no	yes	no	yes
182252439	chr1	227607732	227607766	18	35	1.944444	no	no	yes	no	no	no	no	no	no	yes
182252455	chr1	227659109	227659141	12	33	2.833333	no	yes	yes	no	no	no	no	yes	no	yes
182252456	chr1	227659127	227659167	20	41	2.100000	no	yes	yes	no	no	no	no	yes	no	yes
182252459	chr1	227671351	227671384	17	34	2.000000	no	no	yes	no	no	no	no	no	no	yes
182252463	chr1	227679173	227679209	19	37	1.947368	no	no	no	no	no	no	no	no	no	no
182252471	chr1	227692305	227692339	17	35	2.058824	no	no	no	no	no	no	no	no	no	no
182252476	chr1	227694925	227694991	33	67	2.030303	no	no	no	no	no	no	no	no	no	no
182252508	chr1	227735887	227736105	64	219	3.417910	yes	no	yes	no	no	no	no	yes	yes	yes
182252526	chr1	227742963	227743002	20	40	2.050000	no	no	yes	no	no	yes	no	yes	no	yes
182252527	chr1	227743413	227743454	17	42	2.470588	no	no	yes	no	no	yes	no	yes	no	yes
182252531	chr1	227748583	227748803	112	221	1.973214	no	no	yes	no	no	yes	no	yes	yes	yes
182252534	chr1	227749741	227749848	50	108	2.200000	no	no	yes	no	no	no	no	yes	no	yes
182252539	chr1	227765186	227765215	14	30	2.142857	no	no	yes	no	no	no	no	yes	yes	yes

182252541	chr1	227767545	227767614	36	70	2.027778	no	no	yes	no	no	no	no	yes	yes	yes
182252548	chr1	227782459	227782571	54	113	2.092592	no	no	no	no	no	no	no	yes	no	no
182252549	chr1	227788379	227788418	20	40	2.000000	yes	no	yes	no	no	no	yes	yes	yes	yes
182252555	chr1	227791356	227791473	54	118	2.185185	no	yes	no	no	no	no	no	yes	yes	yes
182252557	chr1	227792294	227792357	22	64	2.909091	no	no	no	no	no	yes	no	no	yes	no
182252573	chr1	227815936	227815994	30	59	2.000000	no	no	yes	no	no	no	no	yes	yes	yes
182252576	chr1	227816341	227816377	19	37	1.947368	no	no	yes	no	no	no	yes	no	no	yes
182252595	chr1	227858869	227858908	21	40	1.904762	no	no	no	no	no	no	no	no	no	no
182252603	chr1	227867317	227867362	12	46	3.916667	no	no	no	no	no	no	no	no	no	no
182252606	chr1	227883836	227883923	22	88	4.000000	no	no	no	no	no	no	no	no	yes	no
182252608	chr1	227888776	227888838	30	63	2.100000	no	no	no	no	no	no	no	yes	yes	no
182252621	chr1	227899043	227899083	19	41	2.210526	no	no	no	no	no	no	no	no	yes	no
182252629	chr1	227900517	227900846	124	330	2.661290	no	no	no	no	no	no	no	yes	yes	no
182252638	chr1	227903665	227903880	108	216	2.000000	no	no	no	no	no	no	no	no	yes	no
182252639	chr1	227904913	227904945	17	33	2.000000	no	no	no	no	no	no	no	no	no	no
182252645	chr1	227906648	227906833	76	186	2.480520	no	no	no	no	no	no	no	yes	yes	no
182252651	chr1	227907835	227907886	25	52	2.080000	no	no	no	no	no	no	no	no	no	no
182252655	chr1	227909450	227909555	53	106	2.037736	no	no	no	no	no	no	no	yes	no	no
182252665	chr1	227915896	227915927	15	32	2.133333	no	no	no	no	no	no	no	yes	yes	no
182252666	chr1	227916297	227916329	13	33	2.538461	no	no	no	no	no	no	no	yes	yes	no
182252674	chr1	227921075	227921570	272	496	1.823529	no	yes	no	yes	no	no	no	no	no	yes
182252680	chr1	227935083	227935268	105	186	1.809524	no	no	yes	no	no	no	no	yes	yes	yes
182252682	chr1	227935217	227935325	31	109	3.562500	no	no	yes	no	no	no	no	yes	yes	yes
182252693	chr1	227941277	227941553	69	277	4.014493	no	no	yes	no	no	yes	no	yes	yes	yes
182252694	chr1	227946612	227946649	17	38	2.235294	no	no	yes	no	no	no	no	yes	yes	yes

182252695	chr1	227946838	227946874	18	37	2.111111	no	no	yes	no	no	no	yes	yes	yes	yes
182252711	chr1	227964807	227964848	19	42	2.210526	no	no	no	no	no	no	no	no	no	no
182252712	chr1	227965336	227965593	31	258	8.562500	no	no	no	no	no	no	no	no	no	no
182252726	chr1	227971830	227971924	49	95	1.938776	no	no	no	no	no	no	no	yes	yes	no
182252730	chr1	227973689	227973814	63	126	2.000000	no	no	no	no	no	no	yes	yes	no	no
182252740	chr1	227992998	227993040	19	43	2.368421	no	no	no	no	no	no	no	yes	no	no
182252741	chr1	227993398	227993459	29	62	2.241379	no	no	no	no	no	no	no	no	no	no
182252743	chr1	227998578	227998609	15	32	2.200000	no	no	no	no	no	no	no	yes	no	no
182252746	chr1	228006897	228006938	19	42	2.210526	yes	no	no	no	no	no	yes	yes	no	no
182252748	chr1	228008521	228008558	16	38	2.500000	no	no	yes	no	no	no	yes	yes	yes	yes
182252749	chr1	228009343	228009373	14	31	2.214286	no	no	yes	no	no	no	no	yes	yes	yes
182252756	chr1	228011673	228011697	13	25	1.923077	no	no	yes	no	no	no	no	yes	yes	yes
182252760	chr1	228023426	228023616	100	191	1.910000	no	no	yes	no	no	no	no	yes	no	yes
182252769	chr1	228031593	228031642	24	50	2.166667	no	no	yes	no	no	no	no	yes	yes	yes
182252770	chr1	228031656	228031711	26	56	2.192308	no	no	yes	no	no	no	no	yes	yes	yes
182252783	chr1	228042722	228042756	17	35	2.058824	no	no	yes	no	no	no	no	yes	no	yes
182252790	chr1	228046718	228046780	28	63	2.250000	no	no	yes	no	no	no	no	yes	no	yes
182252794	chr1	228055505	228055560	21	56	2.636364	no	no	yes	no	no	no	no	no	no	yes
182252803	chr1	228083626	228083661	17	36	2.117647	no	no	yes	no	no	no	yes	yes	yes	yes
182252804	chr1	228094589	228094651	33	63	1.909091	no	no	yes	no	no	no	no	yes	yes	yes
182252806	chr1	228101386	228101460	37	75	2.054054	no	no	no	yes	yes	yes	no	yes	yes	yes
182252809	chr1	228109563	228109624	28	62	2.392857	yes	no	no	no	no	no	yes	yes	no	no
182252813	chr1	228110093	228110137	20	45	2.300000	yes	no	no	no	no	no	no	yes	yes	no
182252825	chr1	228138787	228138822	15	36	2.400000	no	no	no	no	no	no	no	yes	no	no
182252826	chr1	228140305	228140352	26	48	1.923077	yes	yes	yes	yes	yes	no	yes	yes	no	yes

182252828	chr1	228144061	228144114	26	54	2.153846	yes	no	yes	no	no	no	no	yes	yes	yes
182252838	chr1	228157118	228157188	31	71	2.290323	no	no	yes	no	no	no	no	yes	no	yes
182252840	chr1	228158658	228158709	26	52	2.000000	no	no	no	yes	no	no	yes	yes	no	yes
182252843	chr1	228164517	228164554	20	38	1.900000	no	no	yes	no	no	no	no	yes	no	yes
182252849	chr1	228174112	228174515	16	404	25.250000	yes	no	yes	no	no	no	yes	yes	yes	yes
182252863	chr1	228207512	228207673	88	162	1.852273	yes	no	yes	no	no	no	no	yes	yes	yes
182252865	chr1	228207595	228207839	113	245	2.221239	yes	no	yes	no	no	no	no	yes	yes	yes
182252866	chr1	228213716	228213747	12	32	2.666667	yes	no	yes	no	no	no	yes	yes	yes	yes
182252867	chr1	228213884	228213923	21	40	1.904762	yes	no	yes	no	no	no	yes	yes	no	yes
182252875	chr1	228226336	228226374	21	39	1.904762	no	no	yes	no	no	no	no	yes	no	yes
182252880	chr1	228238534	228238570	19	37	1.947368	no	no	yes	no	yes	no	no	yes	no	yes
182252890	chr1	228238932	228238972	18	41	2.277778	no	no	yes	no	yes	no	no	yes	no	yes
182252891	chr1	228239879	228239970	44	92	2.090909	no	no	yes	no	no	no	no	no	yes	yes
182252899	chr1	228271931	228271980	21	50	2.380952	no	no	yes	yes	yes	no	no	yes	no	yes
182252905	chr1	228279615	228279727	46	113	2.521739	no	no	yes	no	no	no	no	yes	no	yes
182252908	chr1	228281784	228281868	32	85	2.666667	no	no	yes	no	no	no	no	no	yes	yes
182252909	chr1	228285989	228286019	16	31	1.937500	yes	no	yes	no	no	yes	no	no	no	yes
182252910	chr1	228289308	228289430	26	123	4.730769	no	no	yes	no	no	yes	no	yes	no	yes
182252911	chr1	228289778	228289810	17	33	2.058824	no	no	yes	no	no	no	no	no	yes	yes
182252914	chr1	228308756	228308809	20	54	2.800000	yes	no	yes	no	no	no	no	yes	no	yes
182252919	chr1	228310217	228310258	22	42	1.909091	no	no	yes	no	no	no	no	yes	no	yes
182252923	chr1	228310363	228310427	22	65	2.916667	no	no	yes	no	no	no	no	yes	no	yes
182252930	chr1	228320249	228320345	51	97	1.960784	no	no	yes	no	no	no	no	yes	no	yes
182252944	chr1	228344316	228344358	23	43	1.956522	no	no	yes	no	no	no	no	yes	no	yes
182252959	chr1	228351617	228351723	39	107	2.769231	no	no	yes	no	no	yes	no	no	no	yes

182252960	chr1	228360137	228360175	18	39	2.166667	no	yes	yes	yes	yes	no	no	yes	no	yes
182252964	chr1	228363096	228363142	16	47	2.833333	no	no	yes	no	no	no	no	yes	no	yes
182252967	chr1	228366661	228366699	17	39	2.294118	no	no	yes	no	yes	no	no	yes	yes	yes
182252968	chr1	228369529	228369755	69	227	3.267606	yes	no	yes	no	no	no	no	yes	yes	yes
182252976	chr1	228376419	228376447	13	29	2.230769	no	no	yes	no	no	yes	no	yes	yes	yes
182252977	chr1	228377820	228377848	14	29	2.142857	no	no	yes	no	no	yes	no	yes	no	yes
182252979	chr1	228378055	228378204	37	150	4.135135	no	no	yes	no	no	yes	no	yes	yes	yes
182252990	chr1	228397217	228397257	20	41	2.100000	yes	no	yes	no	yes	yes	no	yes	yes	yes
182252991	chr1	228399013	228399076	24	64	2.666667	no	no	yes	no	no	no	no	yes	no	yes
182252998	chr1	228401334	228401393	30	60	2.033333	no	no	yes	no	no	no	no	yes	no	yes
182253000	chr1	228406655	228406700	23	46	2.086957	yes	no	no	no	yes	no	yes	yes	no	yes
182253006	chr1	228407057	228407179	31	123	4.096774	yes	no	no	no	no	yes	no	yes	yes	no
182253013	chr1	228426978	228427142	20	165	8.450000	no	no	no	no	no	no	no	yes	no	no
182253029	chr1	228450834	228450905	37	72	1.972973	no	no	no	no	no	no	no	no	no	no
182253033	chr1	228452541	228452643	55	103	1.909091	no	no	no	no	no	no	no	yes	yes	no
182253042	chr1	228453768	228453804	16	37	2.312500	no	no	no	no	no	no	no	yes	yes	no
182253047	chr1	228462002	228462040	19	39	2.105263	yes	no	no	no	no	no	no	no	no	no
182253061	chr1	228484062	228484101	21	40	2.000000	no	no	no	no	no	no	no	no	no	no
182253062	chr1	228484463	228484491	15	29	1.933333	no	no	no	no	no	no	no	no	no	no
182253067	chr1	228486758	228486829	34	72	2.147059	yes	no	no	no	yes	no	yes	yes	yes	yes
182253069	chr1	228487075	228487132	31	58	1.903226	yes	no	no	no	yes	no	yes	yes	yes	yes
182253070	chr1	228487156	228487216	30	61	2.033333	yes	no	no	no	no	no	yes	yes	yes	no
182253073	chr1	228487333	228487379	23	47	2.043478	yes	no	no	no	no	no	yes	yes	yes	no
182253074	chr1	228487985	228488024	17	40	2.352941	yes	no	yes	no	no	no	no	yes	yes	yes
182253077	chr1	228494180	228494222	23	43	1.913043	no	yes	no	no	no	yes	no	yes	no	yes

182253078	chr1	228498666	228498705	16	40	2.529412	no	no	no	no	no	no	no	yes	no	no
182253080	chr1	228505197	228505228	16	32	2.000000	no	no	no	no	no	no	no	no	no	no
182253090	chr1	228515258	228515300	18	43	2.500000	no	no	no	no	no	no	no	yes	no	no
182253097	chr1	228518224	228518304	40	81	2.025000	no	no	no	no	no	no	no	yes	no	no
182253102	chr1	228531210	228531619	224	410	1.839286	no	no	no	no	no	no	no	yes	no	no
182253105	chr1	228531811	228532046	105	236	2.257143	no	no	no	no	no	no	no	yes	no	no
182253109	chr1	228544053	228544098	24	46	1.916667	no	no	no	no	no	no	no	yes	no	no
182253110	chr1	228544978	228545157	99	180	1.858586	no	no	no	no	no	no	no	yes	no	no
182253114	chr1	228546755	228547174	229	420	1.847162	no	no	no	no	no	no	no	no	no	no
182253120	chr1	228556813	228556856	12	44	3.666667	no	no	no	no	no	yes	no	yes	no	no
182253213	chr1	228647262	228647306	19	45	2.368421	no	no	no	no	no	yes	yes	yes	yes	no
182253222	chr1	228665536	228665606	30	71	2.400000	no	no	no	no	no	no	no	no	no	no
182253240	chr1	228724873	228724914	19	42	2.210526	no	no	no	no	no	no	no	no	no	no
182253241	chr1	228729426	228729468	22	43	1.954545	no	no	no	no	no	no	no	no	no	no
182253242	chr1	228732455	228732507	27	53	2.000000	no	no	no	no	no	no	no	no	no	no
182253243	chr1	228740397	228740446	26	50	1.923077	no	no	yes	no	no	no	no	no	no	yes
182253246	chr1	228764577	228764609	17	33	1.941176	no	no	no	no	no	no	no	no	no	no
182253247	chr1	228767201	228767267	32	67	2.156250	no	no	no	no	no	no	no	no	no	no
182253251	chr1	228772953	228772991	16	39	2.437500	no	no	no	no	no	no	no	yes	yes	no
182253253	chr1	228776258	228776291	14	34	2.428571	yes	no	no	no	no	no	no	no	no	no
182253264	chr1	228802610	228802648	14	39	2.785714	no	no	no	no	no	no	no	yes	yes	no
182253265	chr1	228804744	228804796	22	53	2.409091	no	no	no	no	no	no	no	yes	no	no
182253281	chr1	228852091	228852170	42	80	1.952381	no	no	no	no	no	no	no	no	no	no
182253283	chr1	228857924	228857964	23	41	1.913043	yes	no	no	no	no	no	no	yes	no	no
182253287	chr1	228871172	228871208	12	37	3.083333	no	no	no	no	no	no	no	no	no	no

182253294	chr1	228886449	228886498	20	50	2.500000	no	no	no	no	no	no	no	no	no	no
182253299	chr1	228895889	228895941	28	53	2.000000	no	no	no	no	no	no	no	no	no	no
182253307	chr1	228912675	228912699	13	25	1.923077	no	no	no	no	no	no	no	no	no	no
182253308	chr1	228922258	228922290	17	33	1.941176	no	no	no	no	no	no	no	no	no	no
182253310	chr1	228922639	228922671	17	33	1.941176	no	no	no	no	no	no	no	yes	no	no
182253311	chr1	228926969	228927200	78	232	2.974359	no	no	no	no	no	no	no	no	no	no
182253312	chr1	228931895	228931964	35	70	2.000000	no	no	no	no	no	no	no	yes	yes	no
182253318	chr1	228960639	228960681	21	43	2.047619	no	no	no	no	no	no	no	no	no	no
182253321	chr1	228965601	228965690	34	90	2.638889	no	no	no	no	no	no	no	no	no	no
182253324	chr1	228975021	228975094	36	74	2.055556	no	no	no	no	no	no	no	no	yes	no
182253361	chr1	229042194	229042246	9	53	6.000000	no	yes	no	no	no	no	no	yes	yes	yes
182253376	chr1	229078604	229078633	14	30	2.142857	no	no	no	no	no	no	no	no	no	no
182253377	chr1	229088090	229088247	32	158	4.937500	no	no	no	no	yes	yes	no	no	yes	yes
182253378	chr1	229090663	229090709	23	47	2.086957	no	no	yes	no	no	no	no	no	yes	yes
182253381	chr1	229095457	229095541	43	85	2.023256	yes	no	yes	no	no	no	no	yes	no	yes
182253392	chr1	229110449	229110484	17	36	2.117647	no	no	yes	no	no	no	no	yes	no	yes
182253399	chr1	229149368	229149432	34	65	1.911765	no	no	yes	no	no	no	no	yes	yes	yes
182253427	chr1	229213081	229213134	28	54	1.964286	no	no	no	no	no	no	no	no	no	no
182253429	chr1	229218391	229218447	30	57	1.933333	no	no	no	no	no	no	no	yes	no	no
182253435	chr1	229220855	229220894	21	40	1.904762	no	no	no	no	no	no	no	yes	no	no
182253466	chr1	229259768	229259803	18	36	2.055556	no	no	yes	no	no	no	no	no	no	yes
182253473	chr1	229277000	229277098	43	99	2.418605	no	no	yes	no	no	no	no	no	no	yes
182253479	chr1	229288108	229288228	27	121	4.464286	no	no	yes	no	no	no	no	yes	no	yes
182253480	chr1	229295399	229295435	19	37	1.947368	no	no	yes	no	no	no	no	no	yes	yes
182253489	chr1	229330017	229330141	62	125	2.016129	no	no	yes	no	no	no	no	yes	yes	yes

182253497 chr1 229342114 229342155 15 42 2.800000 no no no yes
yes no no yes yes yes
182253498 chr1 229343499 229343728 102 230 2.254902 yes no no no
no no no yes yes no
182253504 chr1 229362729 229362763 17 35 2.058824 no no no no
no no no no no no
182253510 chr1 229378988 229379029 22 42 1.954545 no no no no
no no no yes yes no
182253525 chr1 229400533 229400575 22 43 1.954545 no no no no
no no no no no no
182253526 chr1 229404214 229404281 37 68 1.918919 no no no no
no no no yes no no
182253536 chr1 229420378 229420426 12 49 4.000000 no no no no
no no no yes no no
182253547 chr1 229431937 229431971 18 35 2.000000 no no yes no
no no yes yes no yes
182253561 chr1 229457575 229457615 21 41 1.952381 no no yes no
no no no no no yes
182253562 chr1 229457644 229457680 20 37 1.950000 no no yes no
no no no no no yes
182253563 chr1 229458044 229458081 20 38 1.900000 no no yes no
no no no no no yes
182253566 chr1 229467950 229467990 20 41 2.100000 no no yes no
no no no no no yes
182253567 chr1 229468987 229469028 21 42 2.000000 no no yes no
no no no no no yes
182253577 chr1 229519367 229519470 52 104 2.000000 yes no yes no
no no no no no yes
182253611 chr1 229578311 229578392 42 82 2.000000 no no no no
no no no yes no no
182253615 chr1 229585387 229585571 100 185 1.890000 no no no no
no no no yes yes no
182253622 chr1 229593739 229593777 15 39 2.600000 no yes no no
no no no no no yes
182253627 chr1 229618381 229618542 80 162 2.025000 no no yes no
no no no no no yes
182253628 chr1 229620023 229620058 15 36 2.400000 no no yes no
no no no yes no yes
182253631 chr1 229622580 229622612 11 33 3.000000 no no yes no
no no no no no yes
182253632 chr1 229625725 229625769 20 45 2.250000 yes no yes no
no no yes yes yes yes
182253662 chr1 229704691 229704715 10 25 2.500000 no no no no
no yes no no no no
182253664 chr1 229711660 229711694 8 35 4.222222 no no no no no
no no no no no
182253673 chr1 229731183 229731232 20 50 2.500000 no no no no
no no no yes no no
182253692 chr1 229787854 229787902 21 49 2.333333 no no no no
no no no yes no no

182253701	chr1	229796924	229796951	14	28	2.000000	no	no	no	no	no	no	no	no	no	no
182253710	chr1	229808439	229808507	36	69	1.972222	no	no	no	no	no	no	no	yes	no	no
182253721	chr1	229812279	229812376	53	98	1.905660	no	no	no	no	no	yes	no	yes	no	no
182253729	chr1	229825241	229825276	17	36	2.117647	no	no	yes	no	no	no	no	no	no	yes
182253730	chr1	229829446	229829517	34	72	2.205882	no	no	yes	no	no	no	no	no	no	yes
182253738	chr1	229838339	229838383	22	45	2.045455	no	no	yes	no	no	no	no	yes	no	yes
182253765	chr1	229900679	229901102	47	424	9.021276	no	no	no	no	no	no	no	no	no	no
182253776	chr1	229923951	229923992	21	42	2.000000	no	no	no	no	no	no	no	no	no	no
182253787	chr1	229946879	229946911	15	33	2.200000	no	no	no	no	no	no	no	yes	no	no
182253789	chr1	229954417	229954449	12	33	2.750000	no	no	no	no	no	no	no	no	no	no
182253800	chr1	229972214	229972271	27	58	2.222222	no	no	no	no	no	no	no	yes	no	no
182253807	chr1	229993425	229993462	12	38	3.000000	no	no	no	no	no	no	no	yes	no	no
182253811	chr1	230012137	230012168	13	32	2.538461	no	no	no	no	no	no	no	yes	no	no
182253825	chr1	230050769	230050865	47	97	2.106383	no	no	no	no	no	no	no	no	no	no
182253829	chr1	230079355	230079387	17	33	1.941176	no	no	yes	no	no	no	no	no	yes	yes
182253834	chr1	230108176	230108211	16	36	2.250000	no	no	yes	no	no	no	no	yes	yes	yes
182253837	chr1	230122624	230122679	20	56	2.800000	no	no	yes	no	no	no	no	yes	yes	yes
182253838	chr1	230127193	230127349	78	157	2.012820	no	no	yes	no	no	no	no	yes	yes	yes
182253839	chr1	230142201	230142275	37	75	2.027027	no	no	yes	no	no	no	no	yes	yes	yes
182253840	chr1	230145712	230145753	17	42	2.444444	no	no	yes	no	no	no	no	yes	yes	yes
182253846	chr1	230176110	230176239	65	130	2.000000	no	no	yes	no	no	no	no	yes	yes	yes
182253852	chr1	230186150	230186202	16	53	3.312500	no	no	yes	no	no	no	no	yes	yes	yes
182253853	chr1	230188969	230189033	18	65	3.666667	no	no	yes	no	no	no	no	no	yes	yes
182253854	chr1	230193452	230193535	26	84	3.230769	no	no	yes	no	no	no	no	no	no	yes
182253855	chr1	230198297	230198532	39	236	6.025000	no	no	yes	no	no	no	no	no	no	yes

182253858	chr1	230212536	230212578	21	43	2.047619	no	no	yes	no	no	no	no	yes	yes	yes
182253859	chr1	230226153	230226177	11	25	2.272727	no	no	yes	no	no	no	no	yes	yes	yes
182253868	chr1	230254516	230254544	15	29	1.933333	yes	no	yes	no	no	no	no	yes	yes	yes
182253872	chr1	230260179	230260210	11	32	2.909091	no	no	yes	no	no	no	no	no	no	yes
182253873	chr1	230265357	230265398	22	42	1.954545	no	yes	yes	yes	no	yes	no	no	no	yes
182253877	chr1	230272741	230272787	15	47	3.133333	yes	no	yes	no	no	no	no	no	no	yes
182253879	chr1	230274950	230274993	17	44	2.588235	no	no	yes	no	no	no	no	no	no	yes
182253888	chr1	230275883	230275925	19	43	2.263158	no	no	yes	no	no	no	no	yes	no	yes
182253892	chr1	230285318	230285401	16	84	5.250000	no	no	no	no	no	no	no	yes	no	no
182253904	chr1	230293401	230293444	22	44	2.000000	no	no	no	no	no	no	no	yes	yes	no
182253906	chr1	230296471	230296510	20	40	2.000000	no	no	no	no	no	no	no	no	no	no
182253909	chr1	230308212	230308243	15	32	2.133333	no	no	no	no	no	no	no	yes	yes	no
182253911	chr1	230312261	230312293	16	33	2.062500	no	no	no	no	no	no	no	no	no	no
182253912	chr1	230313222	230313278	29	57	2.000000	no	no	no	no	no	no	no	no	no	no
182253915	chr1	230314143	230314167	13	25	1.923077	no	no	no	no	no	yes	no	no	no	no
182253916	chr1	230314670	230314716	24	47	1.958333	no	yes	no	no	no	no	no	no	no	yes
182253922	chr1	230315921	230316180	100	260	2.620000	no	yes	no	no	no	no	no	yes	no	yes
182253924	chr1	230316145	230316241	32	97	3.093750	no	yes	no	no	no	no	no	yes	no	yes
182253930	chr1	230332007	230332053	20	47	2.350000	no	no	yes	no	no	no	no	no	no	yes
182253934	chr1	230335025	230335965	373	941	2.556430	no	no	yes	no	no	no	no	yes	no	yes
182253935	chr1	230339172	230339211	20	40	2.000000	no	no	yes	no	no	no	no	yes	no	yes
182253946	chr1	230375159	230375195	19	37	1.947368	no	no	yes	no	no	no	no	no	no	yes
182253949	chr1	230376546	230376570	13	25	1.923077	no	no	yes	no	no	no	no	no	yes	yes
182253952	chr1	230393088	230393132	19	45	2.350000	no	no	yes	no	no	no	no	yes	no	yes
182253957	chr1	230394533	230394860	113	328	2.886956	no	no	yes	no	no	no	no	yes	no	yes

182253964	chr1	230395035	230395221	46	187	3.909091	no	no	yes	no	no	no	no	yes	yes	yes
182253970	chr1	230395198	230395823	57	626	11.016129	no	no	yes	no	no	no	no	yes	yes	yes
182253982	chr1	230423054	230423089	17	36	2.117647	no	no	yes	no	no	no	no	yes	no	yes
182253985	chr1	230426223	230426267	9	45	5.000000	yes	no	no	no	yes	no	yes	yes	no	yes
182254002	chr1	230485376	230485424	17	49	2.941176	no	no	no	no	no	no	no	yes	no	no
182254009	chr1	230494660	230494783	44	124	2.844445	no	no	no	no	no	no	no	yes	no	no
182254010	chr1	230494775	230494814	20	40	2.050000	no	no	no	no	no	no	no	yes	no	no
182254012	chr1	230499286	230499332	23	47	2.086957	no	no	no	no	no	no	no	yes	no	no
182254025	chr1	230552056	230552087	17	32	1.941176	no	no	no	no	no	no	no	no	no	no
182254028	chr1	230566380	230566509	26	130	5.000000	no	no	no	no	no	no	no	no	no	no
182254030	chr1	230574502	230574549	23	48	2.130435	no	no	no	no	no	no	no	yes	no	no
182254031	chr1	230574532	230574586	29	55	2.034483	no	no	no	no	no	no	no	yes	no	no
182254039	chr1	230582184	230582218	17	35	2.058824	no	no	no	no	no	no	no	yes	no	no
182254046	chr1	230590450	230590498	26	49	1.923077	no	no	no	no	no	no	no	no	no	no
182254064	chr1	230644572	230644617	17	46	2.705882	no	no	yes	no	no	no	no	yes	no	yes
182254068	chr1	230653517	230653616	38	100	2.634146	no	no	yes	no	no	no	no	no	no	yes
182254070	chr1	230662996	230663037	21	42	2.142857	no	no	yes	no	no	no	no	no	no	yes
182254073	chr1	230667231	230667496	131	266	2.030534	no	no	yes	no	no	no	no	no	no	yes
182254074	chr1	230668252	230668284	16	33	2.125000	no	no	yes	no	no	no	no	yes	no	yes
182254075	chr1	230671722	230671750	14	29	2.071429	no	no	yes	no	no	no	no	no	no	yes
182254077	chr1	230677588	230677631	23	44	1.913043	no	no	yes	no	no	no	no	yes	no	yes
182254078	chr1	230690680	230690708	13	29	2.307692	no	no	yes	no	no	yes	no	yes	no	yes
182254080	chr1	230691174	230691219	24	46	1.916667	no	no	yes	no	no	yes	no	no	no	yes
182254084	chr1	230716319	230716377	30	59	1.966667	no	no	yes	no	no	no	no	no	yes	yes
182254088	chr1	230735022	230735074	26	53	2.038461	no	no	yes	no	no	no	no	no	no	yes

182254090	chr1	230736912	230736948	16	37	2.312500	no	no	yes	no	no	no	no	no	yes	yes
182254093	chr1	230746217	230746268	23	52	2.260870	no	no	yes	no	no	no	no	no	no	yes
182254098	chr1	230756461	230756500	22	40	1.909091	no	no	yes	no	no	no	no	no	no	yes
182254101	chr1	230760837	230760909	35	73	2.085714	no	no	yes	no	no	no	no	yes	no	yes
182254112	chr1	230778875	230778917	20	43	2.150000	no	no	yes	no	no	no	no	yes	no	yes
182254117	chr1	230799010	230799048	20	39	2.000000	no	no	yes	no	no	no	no	yes	no	yes
182254121	chr1	230808904	230808961	10	58	5.636364	no	no	no	no	no	no	no	yes	no	no
182254126	chr1	230827395	230827424	14	30	2.214286	no	no	no	no	no	no	no	yes	yes	no
182254134	chr1	230851330	230851360	16	31	1.937500	no	no	yes	no	no	no	no	yes	yes	yes
182254143	chr1	230874444	230874490	20	47	2.400000	no	no	yes	no	no	no	no	no	yes	yes
182254156	chr1	230943150	230943214	26	65	2.500000	no	no	yes	no	no	no	no	yes	no	yes
182254157	chr1	230943455	230943489	15	35	2.333333	no	no	yes	no	no	no	no	yes	no	yes
182254158	chr1	230943554	230943602	15	49	3.333333	no	no	yes	no	no	no	no	yes	no	yes
182254159	chr1	230950276	230950302	13	27	2.076923	no	no	yes	no	no	no	no	no	no	yes
182254164	chr1	230977524	230977565	21	42	2.000000	no	no	yes	no	no	no	no	yes	yes	yes
182254170	chr1	230990286	230990318	14	33	2.357143	no	no	yes	no	no	no	no	yes	no	yes
182254172	chr1	230999917	230999953	18	37	2.166667	no	no	yes	no	no	yes	no	no	no	yes
182254176	chr1	231011205	231011259	24	55	2.333333	no	no	no	no	no	no	no	yes	no	no
182254179	chr1	231015668	231015696	14	29	2.071429	no	no	no	no	no	no	no	yes	yes	no
182254185	chr1	231039978	231040012	17	35	2.058824	no	no	no	yes	no	no	yes	yes	yes	yes
182254187	chr1	231040719	231040897	99	179	1.828283	yes	no	no	no	no	no	yes	yes	yes	no
182254196	chr1	231047351	231047659	73	309	4.232877	no	no	no	no	no	no	no	yes	no	no
182254199	chr1	231047675	231047728	20	54	2.750000	no	no	no	no	no	no	no	yes	no	no
182254209	chr1	231052279	231052653	25	375	15.480000	no	no	no	no	no	no	no	yes	no	no
182254214	chr1	231064614	231064697	39	84	2.307692	no	no	no	no	no	no	no	yes	no	no

182254218	chr1	231073000	231073038	20	39	1.950000	no	no	no	no	no	no	no	no	no	no
182254220	chr1	231075223	231075266	13	44	3.384615	no	no	no	no	no	no	no	yes	no	no
182254229	chr1	231092275	231092334	29	60	2.068965	no	no	no	no	no	no	no	yes	no	no
182254242	chr1	231125139	231125175	18	37	2.055556	no	no	no	no	no	no	no	no	yes	no
182254253	chr1	231141348	231141390	21	43	2.142857	no	no	no	no	no	no	no	no	yes	no
182254258	chr1	231153850	231153889	12	40	3.333333	no	no	no	no	no	no	no	yes	yes	no
182254265	chr1	231161254	231161291	20	38	2.000000	yes	no	no	no	no	no	yes	yes	yes	no
182254271	chr1	231187682	231187716	14	35	2.500000	yes	no	yes	no	no	no	no	yes	yes	yes
182254275	chr1	231192180	231192225	20	46	2.300000	no	no	yes	no	no	no	no	no	no	yes
182254282	chr1	231202915	231202968	19	54	2.947368	no	no	yes	no	no	no	no	yes	yes	yes
182254292	chr1	231222369	231222411	22	43	1.954545	no	no	no	no	no	yes	no	no	no	no
182254295	chr1	231226899	231226928	16	30	1.937500	yes	no	yes	no	no	no	no	no	no	yes
182254297	chr1	231249456	231249480	13	25	1.923077	no	no	yes	no	no	no	no	yes	no	yes
182254315	chr1	231299065	231299099	14	35	2.571429	no	no	no	no	no	no	no	yes	no	no
182254316	chr1	231299083	231299118	17	36	2.117647	no	no	no	no	no	no	no	yes	no	no
182254317	chr1	231300080	231300105	13	26	2.000000	no	no	no	no	no	no	no	no	yes	no
182254320	chr1	231300602	231300661	31	60	1.935484	no	no	no	no	no	no	no	yes	yes	no
182254326	chr1	231304720	231304755	15	36	2.466667	no	no	no	no	no	no	no	yes	no	no
182254330	chr1	231306137	231306187	14	51	3.714286	no	no	no	no	no	no	no	yes	no	no
182254335	chr1	231307586	231307617	14	32	2.285714	no	no	no	no	no	no	no	no	no	no
182254336	chr1	231307794	231307833	15	40	2.733333	no	no	no	no	no	no	no	no	no	no
182254338	chr1	231316760	231316799	18	40	2.277778	no	no	no	no	no	no	no	no	no	no
182254363	chr1	231412241	231412273	17	33	1.941176	no	no	yes	no	no	no	no	yes	no	yes
182254384	chr1	231461933	231461967	17	35	2.058824	no	no	yes	no	no	no	no	no	no	yes
182254385	chr1	231463441	231463499	19	59	3.105263	no	no	yes	no	no	no	no	no	no	yes

182254388	chr1	231469909	231469994	43	86	2.023256	no	no	yes	no	no	no	no	no	no	yes
182254389	chr1	231470208	231470248	22	41	1.909091	no	no	yes	no	no	no	no	no	no	yes
182254398	chr1	231510857	231510885	14	29	2.142857	no	no	yes	no	no	no	no	no	no	yes
182254399	chr1	231511051	231511119	33	69	2.121212	no	no	yes	no	no	no	no	yes	no	yes
182254412	chr1	231549009	231549046	14	38	2.714286	no	no	yes	no	no	no	no	yes	no	yes
182254415	chr1	231558733	231558767	16	35	2.187500	no	no	yes	no	no	no	no	yes	no	yes
182254416	chr1	231558766	231558796	16	31	1.937500	no	no	yes	no	no	no	no	yes	no	yes
182254422	chr1	231562171	231562210	21	40	1.952381	no	no	yes	no	no	yes	no	no	no	yes
182254423	chr1	231566074	231566145	28	72	2.571429	no	yes	yes	no	no	no	no	no	no	yes
182254445	chr1	231625921	231625949	15	29	1.933333	yes	no	yes	no	no	no	no	yes	yes	yes
182254450	chr1	231652303	231652358	20	56	2.850000	no	no	yes	no	no	no	no	no	no	yes
182254456	chr1	231683572	231683618	15	47	3.133333	no	no	yes	no	no	no	no	no	yes	yes
182254463	chr1	231705920	231705954	17	35	2.176471	no	no	yes	no	no	no	no	no	no	yes
182254466	chr1	231711302	231711339	18	38	2.111111	no	no	yes	no	no	no	no	no	no	yes
182254473	chr1	231732542	231732566	13	25	1.923077	no	no	yes	no	no	no	no	no	no	yes
182254474	chr1	231732927	231733000	21	74	3.523809	no	no	yes	no	no	no	no	no	no	yes
182254487	chr1	231759246	231759274	15	29	1.933333	no	no	yes	no	no	no	no	no	no	yes
182254493	chr1	231778669	231778714	20	46	2.350000	no	no	yes	no	no	no	no	no	no	yes
182254503	chr1	231789944	231789987	23	44	1.913043	no	no	yes	no	no	no	no	no	no	yes
182254509	chr1	231799882	231799940	24	59	2.440000	no	no	yes	no	no	no	no	yes	no	yes
182254514	chr1	231807312	231807347	15	36	2.437500	no	no	yes	no	no	no	no	yes	yes	yes
182254515	chr1	231814096	231814131	19	36	1.947368	yes	no	yes	no	yes	no	no	yes	yes	yes
182254518	chr1	231816868	231816911	10	44	4.500000	no	no	yes	no	yes	no	no	no	no	yes
182254522	chr1	231816955	231816986	16	32	2.000000	no	no	yes	no	yes	no	no	no	no	yes
182254523	chr1	231823341	231823380	19	40	2.105263	no	no	yes	no	yes	no	no	no	no	yes

182254532 chr1 231850646 231850686 16 41 2.625000 no no yes no
no no no no no yes
182254533 chr1 231852891 231852935 22 45 2.090909 no no yes no
no no no no no yes
182254535 chr1 231853638 231853716 39 79 2.025641 no no yes no
no no no no no yes
182254539 chr1 231868319 231868357 20 39 2.000000 no no yes no
no no no no no yes
182254543 chr1 231875261 231875378 60 118 1.966667 no no yes no
no no no no no yes
182254549 chr1 231895387 231895419 17 33 1.941176 no no yes no
no no no no no yes
182254570 chr1 231966707 231966842 68 136 2.000000 no no yes no
no no no yes yes yes
182254582 chr1 231994683 231994714 15 32 2.133333 no no yes no
no no no no no yes
182254607 chr1 232037648 232038061 192 414 2.197917 no yes no no
no no no yes yes yes
182254615 chr1 232078011 232078063 25 53 2.120000 no no no no
no no no yes no no
182254616 chr1 232083567 232083600 15 34 2.266667 no no no no
no no no no no no
182254620 chr1 232093732 232093765 17 34 2.058824 no no no no
no no no no no no
182254650 chr1 232182131 232182170 22 40 1.909091 no no yes no
no no no yes no yes
182254655 chr1 232196685 232196717 7 33 4.857143 no no no no no
no no no no
182254668 chr1 232213129 232213198 24 70 2.916667 no no no no
no no no no yes no
182254674 chr1 232225050 232225092 21 43 2.095238 no no no no
no no no yes no no
182254676 chr1 232228515 232228579 28 65 2.310345 no no no no
no no no yes no no
182254683 chr1 232239130 232239185 23 56 2.434783 no no no no
no no no no no no
182254685 chr1 232249883 232249917 18 35 1.944444 no no no no
no no no yes yes no
182254689 chr1 232262052 232262088 17 37 2.176471 no no no no
no no no no no no
182254694 chr1 232270936 232270968 16 33 2.062500 no no no no
no no no no no no
182254699 chr1 232287119 232287153 11 35 3.250000 no no no no
no no no no no no
182254702 chr1 232300623 232300744 39 122 3.128205 no no no no
no no no no yes no
182254704 chr1 232302985 232303137 77 153 1.987013 no no no no
no no no yes no no
182254730 chr1 232367945 232367985 22 41 1.909091 no no no no
no no no no no no

182254739	chr1	232377859	232377890	15	32	2.133333	no	no	no	no	no	no	no	no	no	no
182254744	chr1	232384119	232384187	21	69	3.333333	no	no	no	no	no	no	no	yes	yes	no
182254746	chr1	232384446	232384503	29	58	2.068965	no	no	no	no	no	no	no	yes	yes	no
182254750	chr1	232400151	232400235	43	85	1.976744	no	no	yes	no	no	no	no	no	no	yes
182254751	chr1	232406577	232406764	62	188	3.032258	no	no	yes	no	no	no	no	yes	no	yes
182254752	chr1	232412794	232412909	46	116	2.521739	no	no	yes	no	no	no	no	yes	no	yes
182254754	chr1	232415679	232416150	76	472	6.234568	yes	no	yes	no	yes	no	no	yes	yes	yes
182254758	chr1	232452334	232452497	81	164	2.024691	no	no	yes	no	no	no	no	no	yes	yes
182254760	chr1	232460724	232460779	29	56	1.965517	no	no	yes	no	no	no	no	no	no	yes
182254766	chr1	232473607	232473636	12	30	2.500000	no	no	yes	no	no	no	no	yes	yes	yes
182254775	chr1	232544154	232544189	11	36	3.363636	no	no	yes	no	no	no	no	no	no	yes
182254776	chr1	232547567	232547606	11	40	3.727273	no	no	yes	no	no	no	no	no	no	yes
182254777	chr1	232547766	232547819	17	54	3.111111	no	no	yes	no	no	no	no	yes	no	yes
182254779	chr1	232550811	232550848	18	38	2.111111	no	no	yes	no	no	no	no	no	no	yes
182254783	chr1	232564098	232564279	90	182	2.022222	no	no	no	no	no	no	no	no	yes	no
182254788	chr1	232595445	232595477	14	33	2.357143	no	no	no	no	no	no	no	no	yes	no
182254795	chr1	232617054	232617096	10	43	4.300000	no	no	no	no	no	no	no	no	no	no
182254797	chr1	232627280	232627350	33	71	2.151515	no	no	no	no	no	no	no	no	yes	no
182254799	chr1	232629707	232629741	17	35	2.058824	no	no	no	no	no	no	yes	yes	no	no
182254802	chr1	232634698	232634738	19	41	2.157895	no	no	no	no	no	no	no	yes	no	no
182254808	chr1	232637048	232637160	36	113	3.138889	no	no	no	no	no	no	no	yes	no	no
182254811	chr1	232638236	232638341	55	106	1.963636	no	no	no	no	no	no	no	no	no	no
182254817	chr1	232659997	232660116	39	120	3.076923	no	no	no	no	no	no	no	yes	yes	no
182254819	chr1	232664948	232664997	26	50	1.961538	no	no	no	no	no	no	no	yes	yes	no
182254825	chr1	232685876	232685977	48	102	2.145833	no	no	no	no	no	no	no	yes	no	no

182254836	chr1	232701568	232701614	22	47	2.136364	no	no	no	no	no	no	no	yes	yes	no
182254838	chr1	232704960	232704992	16	33	2.125000	no	no	no	no	no	no	no	yes	yes	no
182254839	chr1	232707753	232707786	15	34	2.266667	no	no	no	no	no	no	no	no	no	no
182254845	chr1	232725258	232725457	92	200	2.184783	no	no	no	no	no	no	no	yes	yes	no
182254854	chr1	232752468	232752533	34	66	1.941176	no	no	no	no	no	no	no	yes	no	no
182254859	chr1	232759030	232759070	20	41	2.100000	no	no	no	no	no	no	no	no	no	no
182254879	chr1	232791865	232791897	17	33	1.941176	no	no	no	no	no	no	no	yes	no	no
182254882	chr1	232793004	232793339	37	336	9.135135	no	no	no	no	no	no	no	yes	no	no
182254892	chr1	232820954	232821003	9	50	5.777778	no	no	no	no	no	no	no	no	no	no
182254893	chr1	232822276	232822423	73	148	2.027397	no	no	no	no	no	no	no	no	no	no
182254912	chr1	232851948	232851997	25	50	2.000000	no	no	yes	no	no	no	no	no	no	yes
182254913	chr1	232858951	232859001	14	51	3.642857	no	no	yes	no	no	no	no	no	no	yes
182254919	chr1	232874167	232874257	42	91	2.166667	no	no	yes	no	no	no	no	no	yes	yes
182254920	chr1	232888910	232888943	16	34	2.187500	no	no	yes	no	no	no	no	yes	no	yes
182254931	chr1	232916337	232916432	48	96	2.000000	yes	no	no	no	no	no	no	no	no	no
182254938	chr1	232942504	232942593	40	90	2.250000	no	no	no	no	no	no	no	no	no	no
182254940	chr1	232942634	232942676	16	43	2.687500	no	no	no	no	no	no	no	no	no	no
182254943	chr1	232962483	232962511	15	29	1.933333	no	yes	yes	no	no	no	no	no	no	yes
182254945	chr1	232966901	232966936	18	36	2.000000	no	yes	yes	no	no	no	no	yes	yes	yes
182254947	chr1	232971395	232971465	32	71	2.242424	no	no	yes	no	no	no	no	yes	no	yes
182254948	chr1	232973101	232973340	120	240	2.000000	no	no	yes	no	no	no	no	yes	no	yes
182254949	chr1	232976658	232976809	38	152	4.000000	no	yes	yes	no	no	yes	no	no	no	yes
182254950	chr1	232976834	232976887	27	54	2.000000	no	yes	yes	no	no	yes	no	no	no	yes
182254954	chr1	233008248	233008286	14	39	2.857143	no	no	yes	no	no	no	no	no	no	yes
182254960	chr1	233035314	233035362	26	49	1.923077	no	no	yes	no	no	no	no	no	no	yes

182254963	chr1	233054008	233054058	25	51	2.040000	no	no	yes	no	no	no	no	no	no	yes
182254973	chr1	233081739	233081773	16	35	2.187500	no	no	yes	no	no	no	no	yes	yes	yes
182254974	chr1	233081765	233081914	73	150	2.054795	no	no	yes	no	no	no	no	yes	yes	yes
182254978	chr1	233089280	233089363	42	84	2.000000	no	no	yes	no	no	yes	no	no	no	yes
182254998	chr1	233134560	233134622	24	63	2.625000	no	no	yes	no	no	yes	no	no	no	yes
182255019	chr1	233174047	233174290	136	244	1.875000	no	no	yes	no	no	no	no	yes	no	yes
182255020	chr1	233174375	233174404	12	30	2.500000	no	no	yes	no	no	no	no	no	no	yes
182255021	chr1	233176329	233176369	17	41	2.529412	no	no	yes	no	no	no	no	no	no	yes
182255023	chr1	233185594	233185633	19	40	2.105263	no	no	yes	no	no	no	no	no	no	yes
182255025	chr1	233192981	233193022	17	42	2.470588	no	no	yes	no	no	no	no	no	no	yes
182255035	chr1	233203535	233203581	25	47	1.960000	no	no	yes	no	no	no	no	no	no	yes
182255044	chr1	233226944	233226988	20	45	2.250000	no	no	yes	no	no	no	no	no	no	yes
182255046	chr1	233229779	233229814	18	36	2.000000	no	no	yes	no	no	no	no	no	no	yes
182255050	chr1	233240039	233240068	12	30	2.500000	no	no	yes	no	no	no	no	no	no	yes
182255052	chr1	233246080	233246284	20	205	10.250000	no	no	yes	no	no	no	no	yes	no	yes
182255054	chr1	233271249	233271273	13	25	1.923077	no	no	yes	no	no	no	no	no	no	yes
182255055	chr1	233278127	233278154	13	28	2.153846	no	no	yes	no	no	no	no	no	no	yes
182255059	chr1	233290140	233290171	17	32	1.941176	yes	no	yes	no	no	no	no	yes	no	yes
182255063	chr1	233295575	233295613	12	39	3.333333	no	no	no	no	yes	no	yes	yes	no	yes
182255070	chr1	233299984	233300009	12	26	2.166667	no	no	no	no	no	no	no	no	no	no
182255081	chr1	233330932	233330975	10	44	4.400000	no	no	yes	no	no	no	no	no	no	yes
182255082	chr1	233335111	233335148	19	38	2.000000	no	no	yes	no	no	no	no	no	no	yes
182255093	chr1	233371784	233371868	40	85	2.125000	no	no	yes	no	no	no	no	no	no	yes
182255097	chr1	233383068	233383128	28	61	2.250000	no	yes	no	no	no	no	no	no	no	yes
182255109	chr1	233396437	233396469	12	33	2.750000	no	no	no	no	no	no	no	yes	no	no

182255131	chr1	233465173	233465211	20	39	1.950000	no	no	no	no	no	no	no	yes	no	no
182255142	chr1	233515469	233515500	12	32	2.666667	no	no	no	no	no	no	no	no	yes	no
182255146	chr1	233536893	233536989	14	97	6.928571	no	no	yes	no	no	no	no	no	no	yes
182255154	chr1	233557223	233557257	14	35	2.500000	no	no	no	no	no	no	no	yes	no	no
182255165	chr1	233588231	233588274	21	44	2.095238	no	no	no	no	no	no	no	yes	yes	no
182255171	chr1	233594404	233594915	35	512	14.434783	no	no	no	no	no	no	no	yes	no	no
182255173	chr1	233606924	233607000	38	77	2.026316	no	no	no	no	no	no	no	yes	yes	no
182255177	chr1	233616701	233616795	51	95	1.901961	no	no	yes	no	no	no	no	no	no	yes
182255179	chr1	233619918	233619947	8	30	3.750000	no	no	yes	no	no	no	no	yes	no	yes
182255181	chr1	233624430	233624492	20	63	3.150000	no	no	yes	no	no	yes	no	yes	yes	yes
182255182	chr1	233627470	233627501	14	32	2.285714	no	no	yes	no	no	no	no	yes	yes	yes
182255183	chr1	233629236	233629264	8	29	3.625000	yes	no	yes	no	no	no	no	no	no	yes
182255195	chr1	233661539	233661570	17	32	2.000000	no	no	yes	no	no	no	no	yes	no	yes
182255196	chr1	233661910	233661945	17	36	2.117647	no	yes	yes	no	no	no	no	no	no	yes
182255200	chr1	233672555	233672671	20	117	5.681818	no	no	no	no	no	yes	no	yes	no	no
182255201	chr1	233676335	233676397	21	63	3.095238	no	no	no	no	no	no	no	yes	no	no
182255213	chr1	233709546	233709618	35	73	2.142857	no	no	no	no	no	no	no	yes	no	no
182255216	chr1	233709767	233709950	101	184	1.871287	no	no	no	no	no	no	no	yes	no	no
182255218	chr1	233722807	233722851	16	45	2.812500	no	no	no	no	no	no	no	yes	no	no
182255237	chr1	233762802	233762864	31	63	2.032258	no	no	no	no	no	no	no	no	no	no
182255242	chr1	233778532	233778655	65	124	1.923077	no	no	no	no	no	no	no	no	no	no
182255245	chr1	233781519	233781549	12	31	2.583333	no	no	no	no	no	no	no	no	no	no
182255251	chr1	233797121	233797149	14	29	2.071429	no	no	no	no	no	no	no	no	no	no
182255255	chr1	233800429	233800466	16	38	2.437500	no	no	no	no	no	no	no	no	yes	no
182255259	chr1	233806502	233806578	36	77	2.138889	no	no	no	no	no	no	no	no	no	no

182255268	chr1	233834828	233834866	17	39	2.470588	no	no	no	no	no	no	no	no	no	no
182255278	chr1	233839195	233839244	25	50	2.000000	no	no	no	no	no	no	no	no	no	no
182255279	chr1	233839386	233839430	24	45	1.958333	no	no	no	no	no	no	no	no	no	no
182255283	chr1	233855430	233855461	12	32	2.666667	no	no	yes	no	no	no	no	no	no	yes
182255294	chr1	233874305	233874354	9	50	5.555555	no	no	no	no	no	no	no	yes	no	no
182255316	chr1	233934279	233934317	20	39	1.950000	no	no	yes	no	no	no	no	yes	no	yes
182255320	chr1	233939491	233939649	76	159	2.092105	no	no	yes	no	no	no	no	no	no	yes
182255328	chr1	233947280	233947323	23	44	1.913043	no	no	yes	no	no	no	no	no	no	yes
182255334	chr1	233950465	233950531	35	67	1.914286	no	no	yes	no	no	no	no	yes	no	yes
182255350	chr1	233992862	233992920	29	59	2.034483	no	no	yes	no	no	no	no	no	no	yes
182255352	chr1	234030006	234030039	11	34	3.090909	no	no	yes	no	no	no	no	no	no	yes
182255358	chr1	234049343	234049387	20	45	2.300000	no	no	yes	no	no	no	no	no	no	yes
182255381	chr1	234088162	234088234	31	73	2.343750	no	no	yes	no	no	no	no	no	no	yes
182255384	chr1	234097911	234099025	76	1115	15.271605	no	no	yes	no	no	no	no	yes	no	yes
182255390	chr1	234109987	234110060	38	74	1.947368	no	no	yes	no	no	no	no	yes	no	yes
182255393	chr1	234114849	234114886	18	38	2.111111	no	no	yes	no	no	no	no	yes	yes	yes
182255398	chr1	234120857	234120889	16	33	2.062500	no	no	yes	no	no	no	no	yes	no	yes
182255401	chr1	234134262	234134300	10	39	3.818182	no	no	yes	no	no	no	no	yes	no	yes
182255416	chr1	234164273	234164331	30	59	1.966667	no	no	yes	no	no	no	no	yes	no	yes
182255422	chr1	234176314	234176345	16	32	2.000000	no	no	yes	no	no	no	no	no	no	yes
182255431	chr1	234207381	234207434	19	54	2.842105	no	no	yes	no	no	no	no	yes	no	yes
182255432	chr1	234209258	234209292	17	35	2.058824	no	no	yes	no	no	no	no	yes	no	yes
182255435	chr1	234217609	234217677	33	69	2.090909	no	no	yes	no	no	no	no	yes	yes	yes
182255439	chr1	234217780	234217900	65	121	1.892308	no	no	yes	no	no	no	no	yes	yes	yes
182255449	chr1	234240108	234240151	22	44	2.000000	no	no	yes	no	no	no	no	yes	no	yes

182255451	chr1	234240656	234240713	28	58	2.071429	no	no	yes	no	no	no	no	yes	no	yes
182255456	chr1	234257863	234257898	17	36	2.117647	no	no	yes	no	no	no	no	yes	no	yes
182255459	chr1	234264143	234264176	17	34	2.000000	no	no	yes	no	no	no	no	no	no	yes
182255470	chr1	234269150	234269209	24	60	2.480000	no	no	yes	no	no	no	no	no	no	yes
182255471	chr1	234273631	234273660	14	30	2.142857	no	no	yes	no	no	no	no	no	no	yes
182255475	chr1	234299900	234299942	19	43	2.368421	no	no	yes	no	no	no	no	no	no	yes
182255481	chr1	234308578	234308733	51	156	3.058824	no	no	yes	no	no	no	no	no	no	yes
182255485	chr1	234317956	234317998	21	43	2.142857	no	no	yes	no	no	no	no	no	yes	yes
182255491	chr1	234320246	234320344	44	99	2.295455	no	no	yes	no	no	no	no	yes	no	yes
182255493	chr1	234323550	234323591	19	42	2.210526	no	yes	no	no	no	no	no	yes	no	yes
182255494	chr1	234323718	234323756	12	39	3.250000	no	yes	no	no	no	no	no	yes	no	yes
182255497	chr1	234326096	234326132	7	37	5.428571	no	no	no	no	no	no	no	yes	no	no
182255499	chr1	234333133	234333257	62	125	2.016129	no	no	no	no	no	no	no	no	no	no
182255507	chr1	234350335	234350386	22	52	2.363636	no	no	no	no	no	no	no	no	no	no
182255513	chr1	234365079	234365125	22	47	2.136364	no	no	yes	no	no	no	no	yes	no	yes
182255530	chr1	234393921	234393958	17	38	2.352941	no	no	yes	no	no	no	no	yes	no	yes
182255538	chr1	234399394	234399422	14	29	2.071429	no	no	yes	no	no	yes	no	yes	no	yes
182255544	chr1	234426310	234426350	20	41	2.050000	no	no	yes	no	no	no	no	no	no	yes
182255549	chr1	234444118	234444160	16	43	2.687500	no	no	yes	no	no	no	no	no	no	yes
182255550	chr1	234446634	234446681	24	48	2.041667	no	no	yes	no	no	no	no	no	no	yes
182255555	chr1	234451094	234451123	16	30	1.937500	no	no	yes	no	no	no	no	no	no	yes
182255557	chr1	234457861	234457920	29	60	2.068965	no	no	yes	no	no	no	no	no	no	yes
182255561	chr1	234480408	234480441	16	34	2.125000	no	no	no	no	no	no	no	yes	no	no
182255582	chr1	234517202	234517244	10	43	4.300000	no	no	no	no	no	no	no	yes	yes	no
182255588	chr1	234531885	234531913	14	29	2.071429	yes	no	no	no	no	no	no	yes	yes	no

182255596	chr1	234542415	234542481	23	67	2.913043	no	no	no	no	no	no	no	yes	yes	no
182255599	chr1	234546919	234546969	22	51	2.318182	no	no	no	no	no	no	no	yes	no	no
182255606	chr1	234564421	234564464	22	44	2.000000	yes	no	no	no	no	no	no	yes	yes	no
182255616	chr1	234609614	234609655	21	42	2.000000	yes	no	no	no	no	yes	yes	yes	yes	no
182255617	chr1	234609765	234609803	17	39	2.333333	yes	no	no	no	no	yes	yes	yes	yes	no
182255621	chr1	234616980	234617025	24	46	1.958333	no	no	no	no	no	no	no	yes	yes	no
182255624	chr1	234617843	234617909	30	67	2.233333	no	no	no	no	no	no	no	yes	no	no
182255628	chr1	234618326	234618361	18	36	2.000000	no	no	no	no	no	no	no	yes	yes	no
182255633	chr1	234640444	234640517	38	74	2.000000	no	no	no	no	no	no	no	yes	no	no
182255677	chr1	234709834	234709959	55	126	2.290909	no	no	yes	no	no	no	no	yes	yes	yes
182255685	chr1	234738029	234738084	16	56	3.500000	no	no	no	no	no	no	no	yes	yes	no
182255693	chr1	234761580	234761676	25	97	3.880000	no	no	no	no	no	no	no	yes	yes	no
182255699	chr1	234772818	234772849	15	32	2.133333	no	no	no	no	no	no	no	yes	no	no
182255707	chr1	234784082	234784167	42	86	2.071429	no	no	no	no	no	no	no	no	no	no
182255740	chr1	234873104	234873156	24	53	2.208333	no	no	yes	no	no	no	no	yes	no	yes
182255745	chr1	234903066	234903110	24	45	1.916667	no	no	yes	no	no	no	no	no	yes	yes
182255746	chr1	234907013	234907063	18	51	2.842105	no	no	yes	no	no	no	no	yes	yes	yes
182255747	chr1	234911973	234912045	36	73	2.027778	no	no	yes	no	no	no	no	yes	yes	yes
182255750	chr1	234922963	234923235	66	273	4.196970	no	no	yes	no	no	no	no	no	no	yes
182255752	chr1	234927550	234927606	23	57	2.521739	no	no	yes	no	no	no	no	yes	no	yes
182255753	chr1	234930692	234930875	98	184	1.877551	no	no	yes	no	no	no	no	yes	no	yes
182255769	chr1	234961913	234961955	17	43	2.529412	no	no	yes	no	yes	no	no	yes	no	yes
182255771	chr1	234965178	234965212	16	35	2.187500	yes	no	yes	no	no	no	no	yes	yes	yes
182255774	chr1	234970562	234970610	20	49	2.550000	yes	no	no	no	no	no	no	yes	yes	no
182255776	chr1	234971552	234972511	506	960	1.926877	no	no	no	no	no	no	no	yes	yes	no

182255778	chr1	234980450	234980482	16	33	2.062500	no	no	yes	no	no	no	no	yes	yes	yes
182255779	chr1	234981412	234981529	59	118	2.000000	yes	no	no	no	no	no	no	yes	no	no
182255791	chr1	235004928	235004957	15	30	2.000000	no	no	no	no	no	no	no	yes	no	no
182255793	chr1	235012306	235012344	15	39	2.600000	no	no	no	no	no	no	no	yes	no	no
182255828	chr1	235073872	235073978	55	107	1.945455	no	no	yes	no	no	no	no	yes	yes	yes
182255835	chr1	235080440	235080522	32	83	2.617647	no	no	no	no	no	no	no	yes	no	no
182255845	chr1	235096322	235096387	24	66	2.750000	no	no	no	no	no	yes	no	yes	yes	no
182255860	chr1	235122234	235122268	12	35	3.000000	no	no	yes	no	no	no	no	yes	no	yes
182255863	chr1	235126056	235126109	14	54	4.000000	no	no	yes	no	no	no	no	yes	no	yes
182255886	chr1	235183760	235183784	11	25	2.272727	no	no	yes	no	no	no	no	yes	no	yes
182255897	chr1	235202011	235202051	21	41	1.952381	no	no	yes	no	no	no	no	no	no	yes
182255899	chr1	235211329	235211368	12	40	3.333333	no	no	yes	no	no	no	no	no	no	yes
182255903	chr1	235215451	235215491	17	41	2.388889	no	no	yes	no	no	no	no	no	no	yes
182255905	chr1	235217405	235217468	18	64	3.555556	no	no	yes	no	no	no	no	no	yes	yes
182255909	chr1	235221404	235221458	30	55	1.933333	no	no	yes	no	no	no	no	no	no	yes
182255928	chr1	235245631	235245675	24	45	1.916667	no	no	yes	no	no	no	no	no	no	yes
182255929	chr1	235246285	235246319	18	35	1.944444	no	no	yes	no	no	no	no	yes	yes	yes
182255933	chr1	235250192	235250245	27	54	2.037037	no	no	yes	no	no	no	no	no	no	yes
182255934	chr1	235250487	235250631	72	145	2.013889	no	no	yes	no	no	no	no	yes	yes	yes
182255961	chr1	235298455	235298617	35	163	4.657143	no	no	yes	no	no	no	no	yes	no	yes
182255963	chr1	235298885	235298982	43	98	2.279070	no	no	yes	no	no	no	no	yes	yes	yes
182255967	chr1	235302145	235302198	27	54	2.000000	no	no	yes	no	no	no	no	yes	yes	yes
182255972	chr1	235307557	235307583	14	27	1.928571	no	no	yes	no	no	no	no	yes	yes	yes
182255982	chr1	235315539	235315636	49	98	2.000000	no	no	yes	no	no	no	no	yes	no	yes
182255987	chr1	235331055	235331122	32	68	2.250000	no	no	yes	no	no	no	no	yes	no	yes

182256012	chr1	235363153	235363257	41	105	2.558140	no	no	yes	no	no	yes	no	no	no	yes
182256059	chr1	235428594	235428635	18	42	2.368421	yes	no	yes	no	no	no	no	no	no	yes
182256063	chr1	235428942	235428984	8	43	5.375000	yes	no	yes	no	no	no	no	no	no	yes
182256068	chr1	235436783	235436816	17	34	2.000000	no	no	yes	no	no	yes	no	no	no	yes
182256071	chr1	235441943	235441975	15	33	2.200000	no	no	yes	no	no	yes	no	yes	no	yes
182256074	chr1	235447894	235447922	14	29	2.071429	no	yes	yes	no	no	no	no	no	yes	yes
182256075	chr1	235450237	235450275	18	39	2.166667	no	yes	no	yes	no	no	no	no	no	yes
182256077	chr1	235457322	235457352	16	31	2.000000	no	no	yes	no	no	no	no	no	no	yes
182256081	chr1	235464461	235464510	25	50	2.040000	no	yes	yes	no	no	yes	no	yes	yes	yes
182256090	chr1	235502669	235502713	23	45	1.956522	no	no	yes	no	no	no	no	yes	yes	yes
182256091	chr1	235504090	235504127	19	38	2.000000	no	no	yes	no	no	no	yes	yes	yes	yes
182256098	chr1	235518158	235518200	22	43	2.045455	no	no	no	no	no	no	no	no	yes	no
182256104	chr1	235523402	235523477	36	76	2.111111	no	no	no	no	no	no	no	no	no	no
182256106	chr1	235527338	235527414	38	77	2.026316	no	no	no	no	no	no	no	yes	yes	no
182256107	chr1	235528597	235529124	176	528	3.000000	no	no	no	no	no	no	no	no	no	no
182256119	chr1	235557160	235557210	25	51	2.040000	no	no	yes	no	no	no	no	no	no	yes
182256129	chr1	235570853	235570924	28	72	2.571429	no	no	yes	no	no	no	no	no	no	yes
182256138	chr1	235586383	235586423	16	41	2.562500	no	no	yes	no	no	no	no	yes	yes	yes
182256146	chr1	235587445	235587662	121	218	1.876033	no	no	yes	no	no	no	no	no	no	yes
182256149	chr1	235587652	235587706	19	55	2.894737	no	no	yes	no	no	no	no	no	no	yes
182256185	chr1	235636546	235636580	14	35	2.500000	no	no	yes	no	no	no	no	yes	yes	yes
182256199	chr1	235677420	235677448	15	29	1.933333	no	no	yes	no	no	no	no	no	no	yes
182256200	chr1	235680119	235680159	15	41	2.800000	no	no	yes	no	no	no	no	no	no	yes
182256209	chr1	235728723	235728753	8	31	3.875000	yes	no	yes	no	no	no	no	yes	no	yes
182256225	chr1	235780832	235780863	15	32	2.133333	no	no	yes	no	no	no	no	yes	no	yes

182256237	chr1	235812649	235812677	15	29	2.000000	no	no	yes	no	no	no	no	no	no	yes
182256242	chr1	235818054	235818092	20	39	1.950000	no	no	yes	no	no	no	no	no	no	yes
182256243	chr1	235818108	235818134	12	27	2.250000	no	no	yes	no	no	no	no	no	no	yes
182256246	chr1	235855695	235855752	24	58	2.500000	no	no	yes	no	no	no	no	yes	no	yes
182256248	chr1	235856118	235856154	19	37	1.947368	no	no	yes	no	no	no	no	yes	yes	yes
182256253	chr1	235858763	235858875	57	113	2.000000	no	no	yes	no	no	no	no	yes	yes	yes
182256265	chr1	235890749	235890860	37	112	3.027027	no	no	no	no	no	no	no	no	no	no
182256268	chr1	235892807	235892842	18	36	2.000000	no	no	no	no	no	no	no	yes	yes	no
182256285	chr1	235914745	235914819	35	75	2.171429	yes	no	no	no	no	no	no	yes	no	no
182256289	chr1	235917881	235917919	20	39	2.000000	no	no	no	no	no	no	no	yes	no	no
182256294	chr1	235922829	235922857	14	29	2.071429	no	no	no	no	no	no	no	no	yes	no
182256308	chr1	235943746	235943783	18	38	2.111111	no	no	no	no	yes	no	no	yes	yes	yes
182256309	chr1	235945022	235945055	13	34	2.615385	no	no	no	no	no	no	no	yes	no	no
182256315	chr1	235957200	235957257	23	58	2.521739	no	no	no	no	no	yes	no	no	no	no
182256316	chr1	235959236	235959272	18	37	2.055556	no	no	yes	no	no	no	no	no	yes	yes
182256327	chr1	235986852	235986985	63	134	2.126984	no	no	yes	no	no	no	no	yes	yes	yes
182256328	chr1	235987735	235987765	15	31	2.066667	no	no	yes	no	no	no	no	no	no	yes
182256334	chr1	236003057	236003221	27	165	6.111111	no	no	yes	no	no	no	no	yes	no	yes
182256338	chr1	236011714	236011891	34	178	5.105263	no	no	yes	no	no	no	no	yes	yes	yes
182256345	chr1	236031981	236032063	41	83	2.024390	no	no	yes	no	no	no	no	no	no	yes
182256348	chr1	236038659	236038909	34	251	7.382353	no	no	yes	no	no	no	no	yes	no	yes
182256369	chr1	236068406	236068444	21	39	1.952381	no	no	no	no	no	no	no	yes	no	no
182256382	chr1	236074749	236074780	17	32	1.941176	no	no	no	no	no	no	no	yes	no	no
182256388	chr1	236085711	236085751	17	41	2.444444	no	no	no	no	no	no	no	yes	no	no
182256393	chr1	236086075	236086114	21	40	1.904762	no	no	no	no	no	no	no	yes	no	no

182256394	chr1	236086145	236086303	84	159	1.904762	no	no	no	no	no	no	no	yes	no	no
182256409	chr1	236101705	236101941	98	237	2.394231	no	no	no	no	no	no	no	yes	no	no
182256417	chr1	236120687	236120795	35	109	3.054054	no	no	no	no	no	no	no	yes	yes	no
182256418	chr1	236120947	236120987	12	41	3.416667	no	no	no	no	no	no	no	yes	yes	no
182256434	chr1	236142385	236142446	20	62	3.047619	yes	no	no	no	no	no	yes	yes	yes	no
182256447	chr1	236155928	236155982	20	55	2.850000	no	no	yes	no	no	no	no	yes	yes	yes
182256450	chr1	236161485	236161530	23	46	2.000000	no	no	yes	no	no	no	no	yes	yes	yes
182256462	chr1	236173303	236173377	40	75	1.925000	no	no	yes	no	no	no	no	yes	no	yes
182256465	chr1	236177305	236177339	18	35	1.944444	no	no	yes	no	no	no	no	yes	no	yes
182256468	chr1	236186205	236186356	42	152	3.555556	no	no	yes	no	no	no	no	no	no	yes
182256473	chr1	236217353	236217403	27	51	1.888889	no	yes	yes	no	no	no	no	yes	no	yes
182256474	chr1	236219446	236219481	18	36	2.000000	no	no	yes	no	no	yes	no	no	no	yes
182256481	chr1	236239790	236239906	36	117	3.250000	no	no	yes	no	no	no	no	yes	no	yes
182256483	chr1	236245303	236245506	28	204	7.357143	no	no	yes	no	no	no	no	no	no	yes
182256485	chr1	236250135	236250175	20	41	2.050000	no	no	yes	no	no	no	no	no	no	yes
182256489	chr1	236252550	236252588	20	39	1.950000	no	no	yes	no	no	no	no	no	no	yes
182256500	chr1	236305762	236305808	24	47	1.958333	no	no	no	no	no	no	no	yes	no	no
182256539	chr1	236363705	236363745	21	41	1.952381	no	no	yes	no	no	no	no	yes	yes	yes
182256571	chr1	236411493	236411550	28	58	2.071429	no	no	yes	no	no	no	no	no	no	yes
182256588	chr1	236436784	236436809	13	26	2.000000	no	no	yes	no	no	no	no	no	no	yes
182256617	chr1	236493412	236493667	26	256	10.071428	no	no	yes	no	no	no	no	yes	no	yes
182256624	chr1	236495510	236495559	26	50	1.923077	no	no	yes	no	no	no	no	yes	yes	yes
182256631	chr1	236503032	236503081	24	50	2.125000	no	no	no	no	no	yes	no	yes	no	no
182256637	chr1	236511059	236511091	17	33	1.941176	no	no	no	no	no	no	no	yes	no	no
182256641	chr1	236513600	236513639	21	40	2.000000	no	no	no	no	no	no	no	no	no	no

182256642	chr1	236513669	236513709	15	41	2.800000	no	no	no	no	no	no	no	no	no	no
182256647	chr1	236513806	236513852	24	47	1.958333	no	no	no	no	no	no	no	no	no	no
182256661	chr1	236533902	236534009	44	108	2.454545	no	no	yes	no	no	no	no	yes	yes	yes
182256663	chr1	236534252	236534289	20	38	1.900000	no	no	yes	no	no	no	no	yes	no	yes
182256664	chr1	236551844	236551885	22	42	1.954545	no	yes	yes	no	no	yes	no	yes	no	yes
182256667	chr1	236560211	236560266	24	56	2.375000	no	no	yes	no	no	no	no	yes	no	yes
182256669	chr1	236565070	236565103	9	34	3.888889	no	no	yes	no	no	no	no	yes	no	yes
182256675	chr1	236571765	236571799	16	35	2.187500	no	no	yes	no	no	no	no	no	no	yes
182256676	chr1	236572026	236572070	24	45	1.916667	no	no	yes	no	no	no	no	no	no	yes
182256684	chr1	236611512	236611556	24	45	1.916667	no	no	no	no	no	no	no	no	no	no
182256685	chr1	236613167	236613197	16	31	1.937500	no	no	no	no	no	no	no	yes	no	no
182256695	chr1	236635824	236635877	20	54	2.700000	no	no	no	no	no	no	no	yes	no	no
182256696	chr1	236637224	236637264	21	41	1.952381	no	no	no	no	no	no	no	no	no	no
182256698	chr1	236638382	236638427	13	46	3.538461	no	no	no	no	no	no	no	no	no	no
182256703	chr1	236644436	236644480	21	45	2.238095	no	no	no	no	no	no	no	no	no	no
182256710	chr1	236648796	236648862	30	67	2.225806	no	no	no	no	no	no	no	yes	no	no
182256714	chr1	236652719	236652834	61	116	1.901639	no	no	no	no	no	no	no	yes	no	no
182256715	chr1	236654802	236654956	8	155	19.375000	no	no	no	no	no	no	no	no	no	no
182256726	chr1	236671859	236671891	14	33	2.428571	no	no	yes	no	no	no	no	yes	no	yes
182256727	chr1	236673969	236674026	18	58	3.222222	no	no	yes	no	no	no	no	yes	no	yes
182256741	chr1	236701403	236701435	16	33	2.125000	no	no	yes	no	no	no	no	yes	no	yes
182256746	chr1	236708515	236708557	21	43	2.047619	no	no	yes	no	no	no	no	no	no	yes
182256748	chr1	236713594	236715065	67	1472	21.970150	no	no	yes	no	no	no	no	no	no	yes
182256752	chr1	236728997	236729057	31	61	1.967742	no	no	yes	no	no	no	no	no	no	yes
182256763	chr1	236755632	236757289	49	1658	33.836735	no	no	yes	no	no	no	no	no	no	yes

182256768	chr1	236766421	236766451	16	31	1.937500	no	no	no	no	no	no	no	no	no	no
182256771	chr1	236781606	236781647	22	42	1.909091	no	no	no	no	no	no	no	no	no	no
182256772	chr1	236788697	236788747	18	51	2.944444	no	no	no	no	no	no	no	no	no	no
182256776	chr1	236792099	236792127	15	29	1.933333	no	no	no	no	no	no	no	no	no	no
182256783	chr1	236794387	236794496	55	110	2.000000	yes	no	no	no	no	no	no	yes	yes	no
182256784	chr1	236797484	236797584	49	101	2.061224	no	no	yes	no	no	no	no	no	no	yes
182256787	chr1	236835008	236835068	29	61	2.103448	yes	no	yes	no	no	no	no	yes	no	yes
182256794	chr1	236861073	236861121	25	49	1.960000	no	no	yes	no	no	no	no	no	no	yes
182256796	chr1	236873876	236873918	19	43	2.263158	no	no	yes	no	no	no	no	no	no	yes
182256799	chr1	236875619	236875738	61	120	2.098361	no	no	yes	no	no	no	no	no	no	yes
182256801	chr1	236888824	236888858	9	35	3.700000	no	no	yes	no	no	no	no	no	yes	yes
182256823	chr1	236947029	236947101	36	73	2.027778	no	no	no	no	no	no	no	no	yes	no
182256833	chr1	236951043	236951281	79	239	3.025000	no	no	no	no	no	no	no	no	no	no
182256834	chr1	236954172	236954202	16	31	1.937500	no	no	no	no	no	no	no	no	no	no
182256886	chr1	237051154	237051219	31	66	2.129032	no	no	yes	no	no	no	no	yes	yes	yes
182256892	chr1	237054168	237054308	55	141	2.581818	no	no	yes	no	no	no	no	yes	no	yes
182256895	chr1	237055920	237056838	23	919	40.434784	no	no	yes	no	no	no	no	yes	no	yes
182256899	chr1	237064714	237064797	44	84	1.909091	no	no	yes	no	no	no	no	yes	no	yes
182256904	chr1	237088069	237088099	16	31	1.937500	no	no	yes	no	no	no	no	no	no	yes
182256908	chr1	237097998	237098029	16	32	2.125000	no	no	yes	no	no	no	no	no	no	yes
182256917	chr1	237115171	237115218	16	48	2.888889	no	no	yes	no	no	no	no	yes	yes	yes
182256919	chr1	237116509	237116566	30	58	1.933333	no	no	yes	no	no	no	no	no	no	yes
182256929	chr1	237121041	237121073	16	33	2.062500	no	yes	yes	no	no	no	no	no	no	yes
182256932	chr1	237125773	237125955	101	183	1.841584	no	no	yes	no	no	no	no	no	no	yes
182256954	chr1	237154836	237154879	22	44	2.000000	no	no	yes	no	no	no	no	yes	no	yes

182256958	chr1	237163342	237163378	16	37	2.375000	no	no	yes	no	no	no	no	yes	yes	yes
182256971	chr1	237181537	237181585	24	49	2.083333	no	no	yes	no	no	no	no	no	yes	yes
182256976	chr1	237194585	237194629	19	45	2.368421	no	no	yes	no	no	no	no	no	no	yes
182256977	chr1	237195173	237195203	9	31	3.444444	no	no	yes	no	no	no	no	no	no	yes
182256978	chr1	237197414	237197457	23	44	1.913043	no	no	yes	no	no	no	no	yes	no	yes
182256988	chr1	237217030	237217067	20	38	1.950000	no	no	yes	no	no	no	no	no	no	yes
182256994	chr1	237237247	237237288	17	42	2.529412	no	no	yes	no	no	no	no	no	no	yes
182257007	chr1	237266740	237266938	66	199	3.015152	no	no	yes	no	no	no	no	no	no	yes
182257009	chr1	237272526	237272570	19	45	2.450000	no	no	yes	no	no	no	no	no	no	yes
182257012	chr1	237273919	237273985	30	67	2.322581	no	no	yes	no	no	no	no	no	no	yes
182257020	chr1	237275135	237275180	24	46	1.916667	no	no	yes	no	no	no	no	no	no	yes
182257022	chr1	237279476	237279516	21	41	2.047619	no	no	yes	no	no	no	no	no	no	yes
182257023	chr1	237281933	237282095	49	163	3.326531	no	no	yes	no	no	no	no	no	no	yes
182257030	chr1	237284574	237284617	12	44	3.750000	no	no	yes	no	no	no	no	no	no	yes
182257040	chr1	237322981	237323016	12	36	3.000000	no	no	yes	no	no	no	no	no	no	yes
182257042	chr1	237325838	237325864	12	27	2.250000	no	no	yes	no	no	no	no	no	no	yes
182257053	chr1	237364468	237364517	25	50	2.120000	no	no	yes	no	no	no	no	no	no	yes
182257058	chr1	237371542	237371580	19	39	2.052632	no	no	yes	no	no	no	no	yes	no	yes
182257059	chr1	237373548	237373612	31	65	2.096774	no	no	yes	no	no	no	no	no	no	yes
182257061	chr1	237378399	237378430	15	32	2.133333	no	no	yes	no	no	no	no	no	no	yes
182257067	chr1	237384479	237384511	16	33	2.062500	no	no	yes	no	no	no	no	no	yes	yes
182257068	chr1	237385203	237385240	20	38	1.900000	no	no	yes	no	no	no	no	no	no	yes
182257072	chr1	237400640	237400688	22	49	2.227273	no	no	yes	no	no	no	no	no	no	yes
182257074	chr1	237413375	237413415	22	41	1.909091	no	no	yes	no	no	no	no	no	no	yes
182257076	chr1	237419099	237419135	19	37	1.947368	no	no	yes	no	no	no	no	no	no	yes

182257077	chr1	237420847	237420951	52	105	2.038461	no	no	yes	no	no	no	no	no	no	yes
182257079	chr1	237423311	237423342	12	32	2.666667	no	no	yes	no	no	no	no	no	no	yes
182257084	chr1	237430103	237430159	24	57	2.541667	no	no	yes	no	no	no	no	no	no	yes
182257086	chr1	237430181	237430217	18	37	2.055556	no	no	yes	no	no	no	no	no	no	yes
182257091	chr1	237437865	237437891	14	27	1.928571	no	no	yes	no	no	no	no	no	no	yes
182257092	chr1	237446611	237446641	16	31	1.937500	no	no	yes	no	no	no	no	no	no	yes
182257098	chr1	237447859	237447906	23	48	2.173913	no	no	yes	no	no	no	no	yes	no	yes
182257101	chr1	237450982	237451020	17	39	2.294118	no	no	yes	no	no	no	no	no	no	yes
182257113	chr1	237452490	237452559	19	70	3.650000	no	no	yes	no	no	no	no	no	no	yes
182257114	chr1	237456042	237456089	21	48	2.333333	no	no	yes	no	no	no	no	no	no	yes
182257115	chr1	237464335	237464387	13	53	4.307693	no	no	yes	no	no	no	no	yes	no	yes
182257123	chr1	237469890	237469928	20	39	1.950000	no	no	yes	no	no	no	no	no	no	yes
182257125	chr1	237470412	237470482	31	71	2.290323	yes	no	yes	no	no	no	no	no	no	yes
182257132	chr1	237474227	237474271	18	45	2.500000	no	no	yes	no	no	no	no	no	no	yes
182257164	chr1	237532966	237533016	19	51	2.684211	no	no	yes	no	no	no	no	no	no	yes
182257168	chr1	237541300	237541344	23	45	1.956522	no	no	yes	no	no	no	no	no	no	yes
182257171	chr1	237546327	237546377	25	51	2.040000	no	no	yes	no	no	no	no	no	no	yes
182257174	chr1	237549530	237549567	11	38	3.636364	no	no	yes	no	no	no	no	no	no	yes
182257179	chr1	237561387	237561488	51	102	2.019608	no	no	yes	no	no	no	no	no	yes	yes
182257191	chr1	237588031	237588062	15	32	2.133333	no	no	yes	no	no	no	no	no	no	yes
182257192	chr1	237591082	237591237	15	156	10.400000	no	no	yes	no	no	no	no	yes	no	yes
182257203	chr1	237630992	237631035	22	44	2.000000	no	no	yes	no	no	no	no	no	no	yes
182257206	chr1	237643921	237643952	16	32	2.000000	no	no	yes	no	no	no	no	no	no	yes
182257209	chr1	237646770	237646839	36	70	1.944444	no	no	yes	no	no	no	no	no	no	yes
182257210	chr1	237668273	237668317	23	45	2.000000	no	no	yes	no	no	no	no	no	no	yes

182257221	chr1	237674612	237674659	20	48	2.400000	yes	no	yes	no	no	no	no	no	no	yes
182257224	chr1	237686453	237686489	18	37	2.055556	no	no	yes	no	no	no	no	no	yes	yes
182257226	chr1	237687588	237687618	16	31	1.937500	no	no	yes	no	no	no	no	no	no	yes
182257228	chr1	237691135	237691172	19	38	2.000000	no	no	yes	no	no	no	no	no	no	yes
182257234	chr1	237698458	237698524	34	67	1.970588	no	no	yes	no	no	no	no	yes	no	yes
182257237	chr1	237703590	237703628	17	39	2.294118	no	no	yes	no	no	no	no	no	no	yes
182257244	chr1	237717779	237717818	19	40	2.105263	no	no	yes	no	no	no	no	no	yes	yes
182257247	chr1	237726999	237727071	36	73	2.027778	no	no	yes	no	no	no	no	no	no	yes
182257251	chr1	237737057	237737114	25	58	2.320000	no	no	yes	no	no	no	no	yes	no	yes
182257252	chr1	237753660	237753685	11	26	2.363636	no	no	yes	no	no	no	no	no	yes	yes
182257254	chr1	237762216	237762244	13	29	2.230769	no	no	yes	no	no	no	no	no	yes	yes
182257258	chr1	237788524	237788665	71	142	2.000000	no	no	yes	no	no	no	no	no	no	yes
182257263	chr1	237789506	237789595	30	90	3.000000	no	no	yes	no	no	no	no	no	no	yes
182257265	chr1	237793320	237793360	18	41	2.277778	no	no	yes	no	no	no	no	yes	no	yes
182257272	chr1	237796600	237796671	36	72	2.000000	no	no	yes	no	no	no	no	no	no	yes
182257275	chr1	237802804	237802840	17	37	2.176471	no	no	yes	no	no	yes	no	no	no	yes
182257287	chr1	237860094	237860129	17	36	2.176471	no	no	no	no	no	no	no	yes	no	no
182257290	chr1	237865812	237865853	17	42	2.470588	no	no	yes	no	no	no	no	no	no	yes
182257299	chr1	237883630	237883763	20	134	6.619048	no	no	yes	no	no	no	no	no	no	yes
182257301	chr1	237883957	237884096	10	140	14.000000	no	no	yes	no	no	no	no	no	no	yes
182257302	chr1	237888636	237888690	29	55	1.965517	no	no	yes	no	no	no	no	no	no	yes
182257304	chr1	237893905	237893953	24	49	2.041667	no	no	yes	no	no	no	no	no	no	yes
182257316	chr1	237933025	237933068	20	44	2.200000	no	no	no	no	no	no	no	yes	yes	no
182257321	chr1	237946905	237947000	14	96	7.142857	no	no	no	no	no	no	no	no	no	no
182257324	chr1	237946993	237947055	28	63	2.250000	no	no	no	no	no	yes	no	no	no	no

182257342	chr1	237974942	237975055	39	114	3.025641	no	no	no	no	no	no	no	no	no	no
182257343	chr1	237977796	237977828	17	33	2.000000	no	no	no	no	no	no	no	no	no	no
182257347	chr1	238007142	238007183	20	42	2.100000	no	no	no	no	no	no	no	yes	no	no
182257351	chr1	238020145	238020179	17	35	2.117647	no	no	no	no	no	no	no	no	no	no
182257352	chr1	238022219	238022263	21	45	2.142857	no	no	no	no	no	no	no	no	no	no
182257358	chr1	238028434	238028474	16	41	2.588235	no	no	no	no	no	no	no	no	no	no
182257370	chr1	238051875	238051939	25	65	2.640000	no	no	no	no	no	no	no	no	no	no
182257379	chr1	238090527	238090558	14	32	2.285714	no	no	no	no	no	no	no	no	no	no
182257380	chr1	238095028	238095090	32	63	1.968750	no	no	no	no	no	no	no	no	no	no
182257385	chr1	238111917	238111946	12	30	2.500000	no	no	no	no	no	no	no	yes	no	no
182257393	chr1	238123314	238123342	13	29	2.230769	no	no	no	no	no	no	no	yes	no	no
182257397	chr1	238127670	238127743	29	74	2.620690	no	no	no	no	no	no	no	no	no	no
182257398	chr1	238135056	238135087	15	32	2.133333	no	no	no	no	no	no	no	no	no	no
182257406	chr1	238157620	238157672	13	53	4.214286	no	no	no	no	no	no	no	no	no	no
182257412	chr1	238180747	238180790	18	44	2.500000	no	no	no	no	no	no	no	no	no	no
182257415	chr1	238186591	238186689	41	99	2.441860	no	no	no	no	no	no	no	no	no	no
182257417	chr1	238186726	238186774	24	49	2.041667	no	no	no	no	no	no	no	no	no	no
182257422	chr1	238189210	238189250	17	41	2.411765	no	no	no	no	no	no	no	no	no	no
182257423	chr1	238189600	238189648	24	49	2.041667	no	no	no	no	no	no	no	no	no	no
182257432	chr1	238222563	238222610	25	48	1.920000	no	no	no	no	no	no	no	no	no	no
182257433	chr1	238222635	238222678	20	44	2.200000	no	no	no	no	no	no	no	no	no	no
182257437	chr1	238232052	238232132	39	81	2.076923	no	no	no	no	no	no	no	no	yes	no
182257439	chr1	238240559	238240583	12	25	2.083333	no	no	yes	no	no	no	no	no	no	yes
182257465	chr1	238315965	238315995	15	31	2.066667	no	no	yes	no	no	no	no	no	no	yes
182257467	chr1	238323065	238323117	26	53	2.038461	no	no	yes	no	no	no	no	no	no	yes

182257470	chr1	238324957	238325126	31	170	5.545455	no	no	yes	no	no	yes	no	no	no	yes
182257474	chr1	238330284	238330330	17	47	2.777778	no	no	yes	no	no	no	no	no	no	yes
182257479	chr1	238348801	238348837	13	37	2.785714	no	no	yes	no	no	no	no	no	no	yes
182257484	chr1	238351586	238351648	27	63	2.321429	no	no	yes	no	no	no	no	no	no	yes
182257489	chr1	238360394	238360442	25	49	1.960000	no	no	yes	no	no	no	no	no	no	yes
182257491	chr1	238361063	238361110	22	48	2.181818	no	no	yes	no	no	no	no	no	no	yes
182257498	chr1	238370892	238371024	16	133	8.312500	no	no	yes	no	no	no	no	yes	no	yes
182257500	chr1	238371418	238371454	16	37	2.312500	no	no	yes	no	no	no	no	no	no	yes
182257503	chr1	238374747	238374794	18	48	2.777778	no	no	yes	no	no	no	no	no	no	yes
182257518	chr1	238392200	238392372	87	173	1.988506	no	no	yes	no	no	no	no	yes	no	yes
182257519	chr1	238393947	238393990	23	44	1.913043	no	no	yes	no	no	no	no	no	no	yes
182257526	chr1	238416612	238416658	24	47	1.958333	no	no	no	no	no	no	no	no	no	no
182257533	chr1	238421256	238421318	31	63	2.064516	no	no	no	no	no	no	no	no	no	no
182257536	chr1	238437809	238437848	10	40	3.909091	no	no	no	no	no	no	no	no	no	no
182257546	chr1	238448166	238448198	17	33	2.000000	no	no	no	no	no	no	no	no	no	no
182257560	chr1	238496764	238496865	48	102	2.145833	no	no	yes	no	no	no	no	no	no	yes
182257563	chr1	238508605	238508646	20	42	2.150000	no	no	yes	no	no	no	no	no	no	yes
182257570	chr1	238531975	238532017	23	43	1.956522	no	no	yes	no	no	no	no	no	no	yes
182257575	chr1	238550411	238550454	23	44	1.913043	no	no	no	no	no	no	no	no	no	no
182257585	chr1	238571143	238571182	16	40	2.500000	no	no	no	no	no	no	no	no	no	no
182257592	chr1	238579102	238579175	39	74	1.923077	no	no	no	no	no	no	no	no	no	no
182257594	chr1	238579167	238579508	32	342	10.937500	no	no	no	no	no	no	no	no	no	no
182257604	chr1	238580947	238581058	37	112	3.000000	no	no	no	no	no	no	no	no	no	no
182257610	chr1	238605389	238605426	20	38	1.900000	no	no	no	no	no	no	no	no	no	no
182257614	chr1	238612912	238612976	27	65	2.481482	no	no	no	no	no	no	no	yes	no	no

182257618	chr1	238619813	238619863	25	51	2.080000	no	no	no	no	no	no	no	yes	yes	no
182257623	chr1	238630677	238630747	28	71	2.535714	no	no	no	no	no	no	no	no	no	no
182257624	chr1	238637322	238637357	14	36	2.571429	no	no	no	no	no	no	no	no	no	no
182257629	chr1	238641537	238641578	17	42	2.529412	no	no	no	no	no	no	no	no	no	no
182257645	chr1	238668892	238668924	17	33	1.941176	no	no	no	no	no	no	no	no	no	no
182257647	chr1	238670203	238670276	24	74	3.083333	no	no	no	no	no	no	no	yes	no	no
182257652	chr1	238691168	238691303	67	136	2.029851	no	no	no	no	no	no	no	no	no	no
182257655	chr1	238699501	238699547	25	47	1.920000	no	no	no	no	no	no	no	no	no	no
182257661	chr1	238728180	238728228	24	49	2.041667	no	no	no	no	no	no	no	no	no	no
182257672	chr1	238781999	238782040	22	42	1.954545	no	no	no	no	no	no	no	no	no	no
182257676	chr1	238801081	238801109	14	29	2.142857	no	no	no	no	no	no	no	no	no	no
182257691	chr1	238836968	238837081	40	114	2.850000	no	no	no	no	no	no	no	no	no	no
182257708	chr1	238875884	238875915	14	32	2.285714	no	no	no	no	no	no	no	no	no	no
182257725	chr1	238909048	238909094	24	47	2.083333	no	no	no	no	no	no	no	no	no	no
182257745	chr1	238960035	238960113	39	79	2.025641	no	no	no	no	no	no	no	yes	no	no
182257756	chr1	238985702	238985731	14	30	2.142857	no	no	no	no	no	no	no	no	no	no
182257758	chr1	238992648	238992807	54	160	2.981482	no	no	no	no	no	no	no	no	yes	no
182257761	chr1	239005687	239005740	24	54	2.375000	no	no	no	no	no	no	no	no	no	no
182257762	chr1	239010956	239010980	10	25	2.500000	no	no	no	no	no	no	no	no	no	no
182257764	chr1	239017794	239017848	25	55	2.200000	no	no	no	no	no	no	no	yes	no	no
182257765	chr1	239028056	239028080	13	25	1.923077	no	no	no	no	no	no	no	yes	no	no
182257781	chr1	239067554	239067621	34	68	2.000000	no	no	no	no	no	no	no	yes	no	no
182257782	chr1	239067702	239067745	14	44	3.142857	no	no	no	no	no	no	no	yes	no	no
182257786	chr1	239091138	239091219	29	82	3.000000	no	no	no	no	no	no	no	no	no	no
182257789	chr1	239099698	239099769	24	72	3.041667	no	no	no	no	no	no	no	no	no	no

182257804	chr1	239157075	239157112	16	38	2.375000	no	no	no	no	no	no	no	no	no	no
182257806	chr1	239158086	239158143	26	58	2.222222	no	no	no	no	no	no	no	no	no	no
182257808	chr1	239162783	239162814	8	32	4.125000	no	no	no	no	no	no	no	no	no	no
182257809	chr1	239166544	239166579	16	36	2.250000	no	no	no	no	no	no	no	no	no	no
182257811	chr1	239167409	239167486	41	78	1.951220	no	no	no	no	no	no	no	no	no	no
182257813	chr1	239172876	239172909	16	34	2.125000	no	no	no	no	no	no	no	yes	yes	no
182257830	chr1	239191961	239191997	18	37	2.111111	no	no	no	no	no	no	no	no	no	no
182257835	chr1	239198737	239198816	38	80	2.157895	no	no	no	no	no	no	no	no	no	no
182257837	chr1	239202391	239202447	26	57	2.192308	no	no	no	no	no	no	no	yes	no	no
182257848	chr1	239229824	239229862	19	39	2.157895	no	no	no	no	no	no	no	no	no	no
182257849	chr1	239230387	239230456	36	70	1.944444	no	no	no	no	no	no	no	no	no	no
182257855	chr1	239243933	239243992	25	60	2.384615	no	no	no	no	no	no	no	no	no	no
182257870	chr1	239259466	239259538	31	73	2.354839	no	no	no	no	no	no	no	no	yes	no
182257887	chr1	239299183	239299240	30	58	1.933333	no	no	no	no	no	no	no	no	no	no
182257891	chr1	239299447	239299503	26	57	2.192308	no	no	no	no	no	no	no	no	no	no
182257905	chr1	239335842	239335878	15	37	2.466667	no	no	no	no	no	no	no	no	no	no
182257906	chr1	239344346	239344379	17	34	2.000000	no	no	no	no	no	no	no	no	no	no
182257917	chr1	239367057	239367085	13	29	2.230769	no	no	no	no	no	no	no	no	no	no
182257923	chr1	239377911	239377977	22	67	3.090909	no	no	no	no	no	no	no	no	no	no
182257930	chr1	239397691	239397771	25	81	3.269231	no	no	yes	no	no	no	no	no	no	yes
182257936	chr1	239405596	239405633	20	38	1.900000	no	no	yes	no	no	no	no	yes	yes	yes
182257941	chr1	239418454	239418489	14	36	2.642857	no	no	yes	no	no	no	no	no	no	yes
182257945	chr1	239431979	239432025	23	47	2.043478	no	no	yes	no	no	no	no	no	yes	yes
182257946	chr1	239443651	239443785	68	135	2.000000	no	no	yes	no	no	no	no	no	no	yes
182257950	chr1	239450807	239450837	15	31	2.066667	no	no	yes	no	no	no	no	yes	no	yes

182257953	chr1	239463700	239463733	11	34	3.090909	no	no	yes	no	no	no	no	no	no	yes
182257961	chr1	239498419	239498459	21	41	1.952381	no	no	yes	no	no	no	no	no	no	yes
182257962	chr1	239498908	239498948	20	41	2.050000	no	no	yes	no	no	no	no	no	no	yes
182257964	chr1	239512603	239512656	25	54	2.200000	no	no	yes	no	no	no	no	no	no	yes
182257966	chr1	239516026	239516066	18	41	2.277778	no	no	yes	no	no	no	no	no	yes	yes
182257975	chr1	239545130	239545172	23	43	1.913043	no	no	yes	no	no	no	no	no	no	yes
182257982	chr1	239552133	239552473	166	341	2.120482	no	no	yes	no	no	no	no	no	no	yes
182257987	chr1	239568660	239568727	23	68	3.086957	no	no	yes	no	no	no	no	no	no	yes
182257991	chr1	239574782	239574819	20	38	1.900000	no	no	yes	no	no	no	no	no	no	yes
182257997	chr1	239577373	239577418	18	46	2.555556	no	no	yes	no	no	no	no	yes	no	yes
182258000	chr1	239580759	239580797	14	39	2.733333	no	no	yes	no	no	no	no	no	no	yes
182258003	chr1	239586234	239586272	17	39	2.294118	no	no	yes	no	no	no	no	no	no	yes
182258005	chr1	239589791	239589823	17	33	1.941176	no	no	yes	no	no	no	no	no	no	yes
182258006	chr1	239597663	239597712	26	50	1.923077	no	no	yes	no	no	no	no	no	no	yes
182258020	chr1	239607019	239607056	15	38	2.533333	no	no	yes	no	no	yes	no	yes	no	yes
182258021	chr1	239613647	239613687	14	41	2.928571	no	no	yes	no	no	no	no	no	no	yes
182258022	chr1	239615668	239615765	51	98	1.921569	no	no	yes	no	no	no	no	no	no	yes
182258035	chr1	239657391	239657438	24	48	2.000000	no	no	yes	no	no	no	no	yes	yes	yes
182258052	chr1	239685796	239686074	144	279	2.000000	no	no	yes	no	no	no	no	yes	no	yes
182258055	chr1	239688225	239688261	16	37	2.375000	no	no	yes	no	no	no	no	no	no	yes
182258079	chr1	239706541	239706832	154	292	1.922078	no	no	yes	no	no	yes	no	yes	yes	yes
182258082	chr1	239732171	239732972	26	802	30.961538	no	no	yes	no	no	no	no	no	no	yes
182258083	chr1	239736837	239736862	13	26	2.000000	no	no	yes	no	no	no	no	yes	no	yes
182258084	chr1	239740125	239740176	22	52	2.363636	no	no	yes	no	no	no	no	yes	yes	yes
182258088	chr1	239756841	239756876	13	36	2.846154	no	no	yes	no	no	no	no	no	no	yes

182258093	chr1	239768480	239768508	15	29	1.933333	no	no	yes	no	no	no	no	yes	no	yes
182258094	chr1	239771963	239772006	23	44	1.913043	no	no	yes	no	no	no	no	yes	no	yes
182258095	chr1	239774870	239774907	19	38	2.000000	no	no	yes	no	no	no	no	no	yes	yes
182258097	chr1	239779025	239779072	25	48	2.000000	no	no	yes	no	no	no	no	no	no	yes
182258100	chr1	239788849	239788910	33	62	1.939394	no	no	yes	no	no	no	no	no	no	yes
182258103	chr1	239801802	239801847	11	46	4.363636	no	no	yes	no	no	no	no	no	yes	yes
182258105	chr1	239806337	239806435	41	99	2.439024	no	no	yes	no	no	no	no	yes	yes	yes
182258108	chr1	239811069	239811107	17	39	2.294118	no	no	yes	no	no	no	no	yes	no	yes
182258114	chr1	239845345	239845373	12	29	2.416667	no	no	yes	no	no	no	no	yes	no	yes
182258115	chr1	239847537	239847577	15	41	2.733333	no	no	yes	no	no	no	no	no	no	yes
182258120	chr1	239849025	239849063	19	39	2.105263	no	no	yes	no	no	no	no	no	no	yes
182258122	chr1	239857969	239857997	13	29	2.230769	no	no	yes	no	no	no	no	yes	no	yes
182258129	chr1	239880817	239880845	14	29	2.071429	no	no	yes	no	no	no	no	yes	no	yes
182258136	chr1	239899355	239899435	34	81	2.441176	no	no	yes	no	no	no	no	no	no	yes
182258140	chr1	239899672	239899713	22	42	1.909091	no	no	yes	no	no	no	no	no	no	yes
182258156	chr1	239950468	239950493	12	26	2.166667	no	no	no	no	no	no	no	no	no	no
182258163	chr1	239970994	239971026	15	33	2.200000	no	no	no	no	no	no	no	no	no	no
182258174	chr1	239998188	239998231	23	44	1.913043	no	no	no	no	no	no	yes	yes	yes	no
182258189	chr1	240054003	240054054	26	52	2.115385	no	no	yes	no	no	no	no	no	no	yes
182258197	chr1	240071244	240071277	16	34	2.125000	no	no	yes	no	no	no	no	no	no	yes
182258198	chr1	240082930	240083293	15	364	24.555555	no	no	yes	no	no	no	no	no	no	yes
182258201	chr1	240087129	240087243	38	115	3.026316	no	no	yes	no	no	no	no	no	no	yes
182258207	chr1	240113931	240113962	16	32	2.062500	no	no	yes	no	no	no	no	no	no	yes
182258212	chr1	240129927	240129968	23	42	1.913043	no	no	yes	no	no	no	no	no	no	yes
182258213	chr1	240136104	240136156	26	53	2.038461	no	no	yes	no	no	no	no	no	no	yes

182258216	chr1	240141293	240141329	13	37	2.846154	no	no	yes	no	no	no	no	no	no	yes
182258225	chr1	240157568	240157623	22	56	2.545455	no	no	yes	no	no	no	no	no	no	yes
182258227	chr1	240159887	240159956	32	70	2.187500	no	no	yes	no	no	no	no	no	no	yes
182258236	chr1	240167516	240167553	14	38	2.714286	no	no	yes	no	no	no	no	no	no	yes
182258239	chr1	240179508	240179540	15	33	2.200000	no	no	yes	no	no	no	no	no	no	yes
182258243	chr1	240184922	240185219	28	298	10.433333	no	no	yes	no	no	no	no	yes	yes	yes
182258251	chr1	240206381	240206418	20	38	1.900000	no	no	yes	no	no	no	no	no	no	yes
182258252	chr1	240207539	240208435	33	897	27.181818	no	no	no	yes	no	yes	yes	no	no	yes
182258253	chr1	240210306	240210338	15	33	2.200000	no	no	yes	no	no	no	no	yes	yes	yes
182258259	chr1	240215556	240215602	21	47	2.285714	no	no	yes	no	no	no	no	no	no	yes
182258264	chr1	240233622	240233741	42	120	2.857143	no	no	yes	no	no	no	no	no	no	yes
182258267	chr1	240244278	240244360	28	83	2.964286	yes	no	yes	no	no	no	no	no	no	yes
182258270	chr1	240248376	240248445	29	70	2.448276	no	no	yes	no	no	no	no	no	no	yes
182258276	chr1	240276957	240276993	19	37	2.000000	no	no	yes	no	no	no	no	no	no	yes
182258278	chr1	240280273	240280301	15	29	1.933333	no	no	yes	no	no	no	no	no	no	yes
182258283	chr1	240292284	240292329	24	46	1.916667	no	no	yes	no	no	no	no	no	no	yes
182258297	chr1	240333346	240333410	33	65	1.969697	no	no	yes	no	no	no	no	yes	no	yes
182258298	chr1	240333560	240333592	16	33	2.125000	no	no	yes	no	no	no	no	no	no	yes
182258301	chr1	240339515	240339540	11	26	2.363636	no	no	yes	no	no	no	no	no	no	yes
182258303	chr1	240351859	240351944	18	86	4.777778	no	no	yes	no	no	no	no	yes	yes	yes
182258306	chr1	240361324	240361361	20	38	1.900000	no	no	yes	no	no	no	no	no	no	yes
182258308	chr1	240363349	240363423	39	75	1.923077	no	no	yes	no	no	no	no	yes	yes	yes
182258312	chr1	240365147	240365325	84	179	2.130952	no	no	yes	no	no	no	no	no	no	yes
182258321	chr1	240385588	240385612	12	25	2.083333	no	no	yes	no	no	no	no	no	no	yes
182258326	chr1	240405961	240406389	31	429	13.838710	no	no	yes	no	no	no	yes	no	yes	yes

182258339	chr1	240435521	240435558	14	38	2.714286	no	no	yes	no	no	no	no	no	no	yes
182258341	chr1	240440294	240440368	38	75	1.973684	no	no	yes	no	no	no	no	no	no	yes
182258351	chr1	240465121	240465205	41	85	2.097561	no	no	yes	no	no	no	no	yes	yes	yes
182258354	chr1	240468239	240468343	22	105	4.954545	no	no	yes	no	no	no	no	yes	no	yes
182258375	chr1	240536568	240536772	51	205	4.019608	no	no	yes	no	no	no	no	no	no	yes
182258391	chr1	240573606	240573655	25	50	2.000000	no	no	yes	no	no	no	no	no	no	yes
182258396	chr1	240578048	240578089	17	42	2.529412	no	no	yes	no	no	no	no	no	no	yes
182258401	chr1	240579196	240579240	21	45	2.190476	no	no	yes	no	no	no	no	no	no	yes
182258405	chr1	240585911	240585940	14	30	2.142857	no	no	yes	no	no	no	no	no	no	yes
182258416	chr1	240603466	240603526	11	61	5.545455	no	no	yes	no	no	no	no	yes	no	yes
182258419	chr1	240603773	240603861	38	89	2.473684	no	no	yes	no	no	no	no	no	no	yes
182258421	chr1	240609048	240609097	24	50	2.083333	no	no	yes	no	no	no	no	yes	no	yes
182258431	chr1	240626629	240626682	28	54	1.964286	no	no	no	no	no	no	no	no	no	no
182258432	chr1	240633164	240633207	23	44	1.956522	no	no	no	no	no	no	no	no	no	no
182258466	chr1	240693169	240693198	14	30	2.214286	no	no	no	no	no	no	no	yes	yes	no
182258511	chr1	240765986	240766087	34	102	2.921053	no	no	yes	no	no	no	no	yes	yes	yes
182258512	chr1	240766149	240766179	16	31	1.937500	no	no	yes	no	no	no	no	no	yes	yes
182258513	chr1	240770850	240770895	23	46	2.000000	no	yes	yes	no	yes	no	no	no	no	yes
182258523	chr1	240784955	240785154	99	200	2.030303	no	no	yes	no	no	no	no	no	yes	yes
182258532	chr1	240806501	240806532	16	32	2.000000	no	no	yes	no	no	no	no	yes	no	yes
182258534	chr1	240806532	240806589	12	58	4.846154	no	no	yes	no	no	no	no	yes	no	yes
182258535	chr1	240808996	240809034	19	39	2.052632	no	no	yes	no	no	no	no	no	no	yes
182258538	chr1	240813407	240813451	18	45	2.500000	no	no	yes	no	no	no	no	no	no	yes
182258540	chr1	240817521	240817555	17	35	2.058824	no	no	yes	no	no	no	no	no	no	yes
182258542	chr1	240833727	240833796	35	70	2.000000	no	no	yes	no	no	no	no	no	no	yes

182258544	chr1	240837951	240837986	19	36	1.947368	no	no	yes	no	no	no	no	no	no	yes
182258555	chr1	240855374	240855417	18	44	2.444444	no	no	yes	no	no	no	no	no	no	yes
182258559	chr1	240867998	240868158	79	161	2.101266	no	no	yes	no	no	no	no	no	no	yes
182258565	chr1	240877264	240877320	25	57	2.280000	no	no	yes	no	no	no	no	no	no	yes
182258566	chr1	240877312	240877357	14	46	3.200000	no	no	yes	no	no	no	no	no	no	yes
182258574	chr1	240890512	240890588	32	77	2.406250	no	no	yes	no	no	no	no	no	no	yes
182258579	chr1	240907417	240907475	22	59	2.695652	no	no	yes	no	no	no	no	yes	no	yes
182258580	chr1	240908399	240908490	48	92	1.916667	no	no	yes	no	no	no	no	no	no	yes
182258581	chr1	240910003	240910027	9	25	2.777778	no	no	yes	no	no	no	no	yes	no	yes
182258583	chr1	240913564	240913628	31	65	2.096774	no	no	yes	no	no	no	no	no	no	yes
182258603	chr1	240958008	240958046	20	39	1.950000	no	no	yes	no	no	no	no	no	no	yes
182258607	chr1	240966391	240966508	58	118	2.034483	no	no	yes	no	no	no	no	no	no	yes
182258609	chr1	240967998	240968039	15	42	2.866667	no	no	yes	no	no	no	no	yes	no	yes
182258628	chr1	241001393	241001432	21	40	1.952381	no	no	yes	no	no	no	no	no	no	yes
182258636	chr1	241015403	241015439	18	37	2.111111	no	no	yes	no	no	no	no	no	no	yes
182258639	chr1	241019956	241020001	17	46	2.705882	no	no	yes	no	no	no	no	no	no	yes
182258642	chr1	241023055	241023190	72	136	1.944444	no	no	yes	no	no	no	no	no	no	yes
182258645	chr1	241029851	241029886	17	36	2.117647	no	no	yes	no	no	no	no	yes	yes	yes
182258653	chr1	241043400	241043432	16	33	2.125000	no	no	yes	no	no	no	no	no	no	yes
182258668	chr1	241065234	241065330	37	97	2.589744	no	no	yes	no	no	no	no	no	no	yes
182258676	chr1	241082162	241082191	9	30	3.444444	no	no	yes	no	no	no	no	yes	no	yes
182258693	chr1	241101602	241101632	13	31	2.384615	no	no	yes	no	no	no	no	no	no	yes
182258697	chr1	241105116	241105231	57	116	2.070175	no	no	yes	no	no	no	no	yes	no	yes
182258702	chr1	241118238	241118277	20	40	2.100000	no	no	yes	no	no	no	no	no	yes	yes
182258713	chr1	241139164	241139230	35	67	1.914286	no	no	yes	no	no	no	no	yes	no	yes

182258724	chr1	241160400	241160570	57	171	2.982759	no	no	yes	no	no	no	no	no	no	yes
182258728	chr1	241161379	241161522	74	144	2.040540	no	no	yes	no	no	no	no	yes	no	yes
182258740	chr1	241187564	241187620	28	57	2.071429	no	no	yes	no	no	no	no	no	no	yes
182258763	chr1	241221905	241221965	21	61	2.904762	no	no	yes	no	no	no	no	no	no	yes
182258764	chr1	241223110	241223153	17	44	2.611111	no	no	yes	no	no	no	no	no	no	yes
182258767	chr1	241234170	241234202	17	33	1.941176	no	no	yes	no	no	no	no	no	no	yes
182258783	chr1	241255681	241255774	33	94	2.909091	no	no	yes	no	no	no	no	no	no	yes
182258784	chr1	241256210	241256255	18	46	2.611111	no	no	yes	no	no	no	no	no	no	yes
182258796	chr1	241274314	241274362	25	49	1.960000	no	no	yes	no	no	no	no	no	no	yes
182258807	chr1	241306285	241306389	44	105	2.386364	yes	no	yes	no	no	no	no	yes	no	yes
182258814	chr1	241306494	241306669	97	176	1.855670	yes	no	yes	no	no	no	no	yes	no	yes
182258822	chr1	241319701	241319729	15	29	1.933333	no	no	yes	no	no	no	no	no	no	yes
182258823	chr1	241322188	241322243	28	56	2.000000	no	no	yes	no	no	no	no	no	no	yes
182258833	chr1	241357320	241357350	9	31	3.444444	yes	yes	no	no	no	no	yes	yes	yes	yes
182258834	chr1	241357515	241357543	15	29	1.933333	yes	no	yes	no	no	no	no	yes	yes	yes
182258836	chr1	241358156	241358319	81	164	2.024691	yes	yes	yes	no	no	no	no	no	yes	yes
182258840	chr1	241368590	241368620	16	31	1.937500	no	no	no	no	no	no	no	no	no	no
182258841	chr1	241371760	241371785	12	26	2.166667	no	no	no	no	no	no	no	no	no	no
182258853	chr1	241413213	241413250	18	38	2.166667	yes	no	no	no	no	no	no	yes	no	no
182258866	chr1	241460286	241460317	15	32	2.133333	no	no	yes	no	no	no	no	no	no	yes
182258878	chr1	241490176	241490227	25	52	2.080000	no	no	no	no	no	no	no	no	no	no
182258879	chr1	241490355	241490398	18	44	2.555556	no	no	no	no	no	no	no	no	no	no
182258883	chr1	241491700	241491763	28	64	2.285714	no	no	no	no	no	no	no	no	yes	no
182258888	chr1	241504345	241504380	19	36	1.947368	no	no	yes	no	no	no	no	no	no	yes
182258889	chr1	241506101	241506143	21	43	2.047619	no	no	no	yes	no	no	no	yes	no	yes

182258891	chr1	241508841	241508869	13	29	2.230769	no	no	yes	no	no	no	no	no	no	yes
182258895	chr1	241509014	241509104	18	91	5.000000	no	no	yes	no	no	no	no	no	no	yes
182258902	chr1	241526426	241526465	21	40	2.047619	no	no	no	no	no	no	no	no	no	no
182258940	chr1	241596634	241596667	16	34	2.125000	no	no	yes	no	no	yes	no	yes	yes	yes
182258950	chr1	241608282	241608306	13	25	1.923077	no	no	yes	no	no	no	no	yes	no	yes
182258951	chr1	241611081	241611130	25	50	2.000000	no	no	yes	no	no	no	no	yes	yes	yes
182258952	chr1	241614678	241614862	45	185	4.111111	no	no	yes	no	no	no	no	no	yes	yes
182258958	chr1	241623953	241624337	64	385	6.046875	no	no	yes	no	no	no	no	yes	no	yes
182258977	chr1	241686082	241686120	18	39	2.222222	no	no	yes	no	no	no	no	no	no	yes
182258980	chr1	241694239	241694271	17	33	2.000000	no	no	yes	no	no	no	no	yes	no	yes
182258982	chr1	241696195	241696233	15	39	2.529412	no	no	yes	no	no	no	no	yes	yes	yes
182258990	chr1	241715298	241715343	23	46	2.000000	no	no	yes	no	no	no	no	yes	no	yes
182258993	chr1	241725428	241725464	18	37	2.055556	no	no	yes	no	no	no	no	yes	no	yes
182259001	chr1	241760695	241760746	20	52	2.600000	no	no	yes	no	no	no	no	no	no	yes
182259003	chr1	241770869	241770908	18	40	2.277778	no	no	yes	no	no	no	no	no	no	yes
182259008	chr1	241776003	241776031	14	29	2.142857	no	no	yes	no	no	no	no	yes	no	yes
182259009	chr1	241783839	241783881	22	43	1.954545	no	no	yes	no	no	no	no	yes	no	yes
182259012	chr1	241806863	241806899	19	37	2.052632	no	no	no	no	no	no	no	yes	no	no
182259018	chr1	241810448	241810485	17	38	2.294118	no	no	no	no	no	no	no	yes	no	no
182259019	chr1	241817703	241817743	19	41	2.157895	no	no	no	no	no	no	no	yes	yes	no
182259020	chr1	241817864	241818057	105	194	1.847619	no	no	no	no	no	no	no	yes	no	no
182259026	chr1	241829511	241829618	50	108	2.160000	no	no	no	no	no	no	no	yes	yes	no
182259030	chr1	241842287	241842343	25	57	2.280000	no	no	no	no	no	no	no	no	no	no
182259040	chr1	241857575	241857613	18	39	2.166667	no	no	yes	no	no	no	no	no	no	yes
182259056	chr1	241902943	241903270	25	328	13.120000	no	no	no	no	no	no	no	no	yes	no

182259059	chr1	241909391	241909426	15	36	2.400000	no	no	no	no	no	no	no	yes	no	no
182259075	chr1	241940979	241941014	16	36	2.250000	no	no	no	no	no	no	no	no	no	no
182259103	chr1	241985905	241985952	22	48	2.181818	no	no	no	no	no	no	no	yes	yes	no
182259107	chr1	241998850	241998896	19	47	2.473684	no	no	yes	no	no	no	no	yes	no	yes
182259140	chr1	242096650	242096703	27	54	2.037037	no	no	yes	no	no	no	no	no	no	yes
182259145	chr1	242112165	242112190	12	26	2.166667	no	no	yes	no	no	no	no	no	no	yes
182259153	chr1	242124876	242124928	21	53	2.571429	no	no	yes	no	no	no	no	yes	no	yes
182259157	chr1	242133584	242133620	16	37	2.312500	no	no	yes	no	no	no	no	yes	no	yes
182259166	chr1	242144151	242144302	76	152	2.013158	no	no	yes	no	no	no	no	no	no	yes
182259167	chr1	242146658	242146693	18	36	2.111111	yes	no	yes	no	no	no	no	no	no	yes
182259171	chr1	242165320	242165427	52	108	2.076923	no	no	yes	no	no	no	no	no	no	yes
182259178	chr1	242191270	242191297	14	28	2.000000	no	no	yes	no	no	no	no	no	no	yes
182259180	chr1	242192565	242192600	16	36	2.312500	no	no	yes	no	no	no	no	no	no	yes
182259182	chr1	242196569	242196601	12	33	2.750000	no	no	yes	no	no	no	no	no	no	yes
182259190	chr1	242207737	242207992	26	256	10.000000	no	no	yes	no	no	no	no	no	no	yes
182259200	chr1	242228002	242228040	19	39	2.105263	no	no	yes	no	no	no	no	no	no	yes
182259202	chr1	242228647	242228722	40	76	1.950000	no	no	yes	no	no	no	no	no	yes	yes
182259207	chr1	242231213	242231246	13	34	2.615385	no	no	yes	no	no	no	no	yes	no	yes
182259225	chr1	242275340	242275387	23	48	2.086957	no	no	yes	no	no	no	no	no	no	yes
182259234	chr1	242293323	242293359	9	37	4.444445	no	no	yes	no	no	no	no	no	no	yes
182259252	chr1	242328298	242328531	126	234	1.952381	no	no	yes	no	no	no	no	yes	no	yes
182259256	chr1	242343767	242343798	16	32	2.000000	no	no	yes	no	no	no	no	no	no	yes
182259258	chr1	242351206	242351236	15	31	2.066667	no	no	yes	no	no	no	no	no	no	yes
182259259	chr1	242362375	242362413	20	39	1.950000	no	no	yes	no	no	no	no	no	no	yes
182259261	chr1	242370429	242370461	16	33	2.062500	no	no	yes	no	no	no	no	no	no	yes

182259269	chr1	242393208	242393757	24	550	23.500000	no	no	yes	no	no	no	no	no	no	yes
182259285	chr1	242446240	242446284	22	45	2.090909	no	no	yes	no	no	no	no	no	no	yes
182259287	chr1	242452991	242453015	13	25	1.923077	no	no	yes	no	no	no	no	no	no	yes
182259306	chr1	242502645	242502702	18	58	3.222222	no	no	yes	no	no	no	no	no	no	yes
182259307	chr1	242506650	242506687	20	38	1.950000	no	no	yes	no	no	no	no	no	no	yes
182259316	chr1	242535518	242535554	12	37	3.076923	no	no	no	no	no	no	no	no	no	no
182259318	chr1	242538687	242538755	34	69	2.029412	no	no	no	no	no	no	no	yes	no	no
182259327	chr1	242564252	242564292	22	41	1.909091	no	no	no	no	no	no	no	no	no	no
182259333	chr1	242593087	242593118	16	32	2.062500	no	no	no	no	no	no	no	no	no	no
182259342	chr1	242612765	242612836	29	72	2.482759	no	no	no	no	no	no	no	no	no	no
182259344	chr1	242614126	242614167	20	42	2.100000	no	no	no	no	no	no	no	no	no	no
182259346	chr1	242614877	242614941	20	65	3.285714	no	no	no	no	no	no	no	yes	no	no
182259350	chr1	242623440	242623480	21	41	1.952381	no	no	no	no	no	no	no	no	no	no
182259354	chr1	242635500	242635541	17	42	2.470588	no	no	no	no	no	no	no	no	no	no
182259355	chr1	242635955	242635995	21	41	1.952381	no	no	no	no	no	no	no	no	yes	no
182259390	chr1	242695387	242695421	11	35	3.181818	no	no	no	no	no	no	no	no	no	no
182259391	chr1	242695705	242695742	19	38	2.105263	no	no	no	no	no	no	no	no	no	no
182259397	chr1	242712586	242712623	18	38	2.111111	no	no	no	no	no	no	no	yes	no	no
182259398	chr1	242717624	242717664	21	41	1.952381	no	no	no	no	no	no	no	no	no	no
182259407	chr1	242733702	242733840	31	139	4.483871	no	no	no	no	no	no	no	yes	no	no
182259411	chr1	242737269	242737305	17	37	2.176471	no	no	no	no	no	no	no	no	no	no
182259422	chr1	242755820	242755874	26	55	2.115385	no	no	no	no	no	no	no	yes	no	no
182259434	chr1	242784380	242784427	24	48	2.000000	no	no	no	no	no	no	no	no	yes	no
182259453	chr1	242818606	242818633	14	28	2.000000	no	no	no	no	no	no	no	no	no	no
182259459	chr1	242856419	242856525	12	107	8.785714	no	no	no	no	no	no	no	yes	no	no

182259467	chr1	242882785	242882835	22	51	2.318182	no	no	no	no	yes	no	no	no	no	yes
182259510	chr1	242990032	242990064	17	33	1.941176	no	no	no	no	no	no	no	no	no	no
182259513	chr1	243001611	243001664	28	54	1.928571	no	no	no	no	no	no	no	yes	no	no
182259537	chr1	243055252	243055276	13	25	1.923077	no	no	no	no	yes	no	no	no	no	yes
182259582	chr1	243203783	243203819	19	37	2.052632	no	no	yes	no	no	no	no	no	no	yes
182259591	chr1	243220407	243220474	31	68	2.225806	no	no	yes	no	no	no	no	no	no	yes
182259595	chr1	243234145	243234245	48	101	2.145833	no	no	yes	no	no	no	no	yes	yes	yes
182259606	chr1	243258078	243258123	23	46	2.000000	no	no	yes	no	no	no	no	yes	no	yes
182259609	chr1	243263164	243263236	39	73	1.923077	no	no	yes	no	no	no	no	yes	no	yes
182259610	chr1	243263793	243263829	19	37	2.000000	no	no	yes	no	no	no	no	yes	no	yes
182259612	chr1	243268860	243269033	88	174	1.977273	yes	no	yes	no	no	no	no	yes	yes	yes
182259627	chr1	243307256	243307287	15	32	2.133333	yes	no	yes	no	no	yes	no	yes	yes	yes
182259628	chr1	243308365	243308405	21	41	1.952381	no	no	yes	no	no	no	no	no	no	yes
182259629	chr1	243327260	243327556	30	297	10.400000	no	no	yes	no	no	no	no	no	no	yes
182259641	chr1	243338981	243339144	54	164	3.037037	no	no	yes	no	no	no	no	no	no	yes
182259643	chr1	243345775	243345813	18	39	2.166667	yes	no	yes	no	no	no	no	yes	no	yes
182259648	chr1	243354922	243355057	71	136	1.915493	no	no	yes	no	no	no	no	yes	yes	yes
182259652	chr1	243361974	243362429	57	456	8.000000	no	no	yes	no	no	no	no	no	no	yes
182259655	chr1	243372898	243372977	12	80	6.833333	no	no	yes	no	no	no	no	no	no	yes
182259657	chr1	243379548	243379581	12	34	2.833333	no	no	yes	no	no	no	no	no	no	yes
182259659	chr1	243380079	243380116	15	38	2.533333	no	no	yes	no	no	no	no	no	no	yes
182259664	chr1	243391871	243391895	12	25	2.083333	no	no	yes	no	no	no	no	yes	no	yes
182259665	chr1	243391908	243391986	39	79	2.025641	no	no	yes	no	no	no	no	yes	no	yes
182259668	chr1	243415129	243415157	12	29	2.416667	no	no	yes	no	no	no	no	no	no	yes
182259675	chr1	243454869	243454945	24	77	3.208333	no	no	yes	no	no	no	no	yes	yes	yes

182259676	chr1	243458379	243458410	15	32	2.133333	no	no	yes	no	no	no	no	no	no	yes
182259681	chr1	243472522	243472594	37	73	2.000000	no	no	yes	no	no	no	no	yes	yes	yes
182259682	chr1	243475086	243475167	41	82	2.024390	no	no	yes	no	no	no	no	yes	yes	yes
182259687	chr1	243481781	243481814	13	34	2.692308	no	no	yes	no	no	no	no	yes	yes	yes
182259690	chr1	243486793	243486893	51	101	1.980392	yes	no	yes	no	no	no	no	yes	yes	yes
182259701	chr1	243527983	243528025	21	43	2.047619	no	no	yes	no	no	no	no	no	no	yes
182259706	chr1	243535120	243535233	56	114	2.125000	no	no	yes	no	no	no	no	no	no	yes
182259709	chr1	243551802	243551839	18	38	2.111111	no	no	yes	no	no	no	no	no	no	yes
182259711	chr1	243563437	243563475	20	39	1.950000	no	no	yes	no	no	no	no	no	no	yes
182259720	chr1	243587770	243587817	24	48	2.041667	no	no	yes	no	no	no	no	no	no	yes
182259733	chr1	243613940	243613986	24	47	1.958333	no	no	yes	no	no	no	no	no	no	yes
182259739	chr1	243636156	243636198	20	43	2.150000	no	no	yes	no	no	no	no	no	no	yes
182259740	chr1	243636237	243636269	17	33	2.000000	no	no	yes	no	no	no	no	no	no	yes
182259746	chr1	243663434	243663493	18	60	3.388889	no	no	yes	no	no	no	no	no	no	yes
182259749	chr1	243677527	243677566	16	40	2.500000	no	no	yes	no	no	no	no	no	no	yes
182259752	chr1	243694732	243694770	20	39	1.950000	no	no	yes	no	no	yes	no	yes	no	yes
182259755	chr1	243701093	243701132	21	40	1.904762	no	no	yes	no	no	no	no	no	no	yes
182259760	chr1	243718971	243719015	15	45	2.882353	no	no	yes	no	no	no	no	yes	yes	yes
182259763	chr1	243726020	243726080	29	61	2.103448	no	no	yes	no	no	no	no	no	yes	yes
182259768	chr1	243750900	243750941	21	42	2.095238	no	no	yes	no	no	no	no	no	no	yes
182259769	chr1	243752788	243752823	18	36	2.000000	no	no	yes	no	no	no	no	yes	no	yes
182259775	chr1	243773184	243773229	23	46	2.000000	no	no	yes	no	no	no	no	no	no	yes
182259777	chr1	243780547	243780609	21	63	3.047619	no	no	yes	no	no	no	no	no	no	yes
182259779	chr1	243783559	243783601	22	43	1.954545	no	no	yes	no	no	no	no	no	no	yes
182259780	chr1	243784661	243784732	38	72	1.921053	no	no	yes	no	no	no	no	no	no	yes

182259792	chr1	243834509	243834543	12	35	3.000000	no	no	yes	no	no	no	no	yes	no	yes
182259795	chr1	243848214	243848272	26	59	2.296296	no	no	yes	no	no	no	no	yes	no	yes
182259809	chr1	243871370	243871411	22	42	1.954545	no	no	no	no	no	no	no	no	no	no
182259812	chr1	243890219	243890248	16	30	1.937500	no	no	no	no	no	no	no	no	no	no
182259827	chr1	243912444	243912564	57	121	2.189655	no	no	no	no	no	no	no	no	no	no
182259835	chr1	243939408	243939463	28	56	2.000000	no	no	yes	no	no	no	no	yes	yes	yes
182259837	chr1	243941068	243941269	67	202	3.014925	no	no	yes	no	no	no	no	no	yes	yes
182259853	chr1	243969018	243969046	15	29	1.933333	no	no	yes	no	no	no	no	no	yes	yes
182259856	chr1	243979527	243979761	60	235	3.934426	no	no	yes	no	no	no	yes	no	yes	yes
182259857	chr1	243980143	243980185	19	43	2.421053	no	no	yes	no	no	no	no	no	no	yes
182259858	chr1	243980212	243980267	28	56	2.035714	no	no	yes	no	no	no	no	no	no	yes
182259861	chr1	243988590	243988646	29	57	1.965517	no	no	yes	no	no	no	no	no	no	yes
182259869	chr1	244002663	244002698	15	36	2.400000	no	no	yes	no	no	no	no	no	no	yes
182259877	chr1	244038973	244039021	24	49	2.125000	no	no	yes	no	no	no	no	no	no	yes
182259886	chr1	244048596	244048661	29	66	2.275862	yes	no	no	no	no	no	yes	yes	no	no
182259888	chr1	244048672	244048699	12	28	2.333333	yes	no	no	no	no	no	yes	yes	no	no
182259889	chr1	244048802	244048833	14	32	2.357143	yes	no	no	no	no	no	yes	yes	yes	no
182259899	chr1	244083349	244083398	26	50	1.961538	no	no	yes	no	no	no	no	no	yes	yes
182259907	chr1	244090043	244090076	15	34	2.266667	no	yes	yes	no	no	no	no	yes	no	yes
182259909	chr1	244095421	244095557	45	137	3.044445	no	no	no	no	no	no	no	yes	no	no
182259910	chr1	244104210	244104250	20	41	2.050000	no	no	no	no	no	yes	no	yes	no	no
182259911	chr1	244105933	244105971	18	39	2.277778	no	no	no	no	no	no	no	no	no	no
182259913	chr1	244123008	244123044	19	37	1.947368	no	no	no	no	no	no	no	yes	no	no
182259914	chr1	244124901	244125020	60	120	2.000000	no	no	no	no	no	no	no	yes	no	no
182259916	chr1	244137654	244137685	12	32	2.666667	no	no	no	no	no	no	no	yes	no	no

182259919	chr1	244140532	244140719	87	188	2.172414	no	no	no	no	no	no	no	yes	no	no
182259930	chr1	244170361	244170431	28	71	2.535714	no	no	no	no	no	no	no	no	no	no
182259931	chr1	244170911	244170942	15	32	2.200000	no	no	no	no	no	no	no	no	no	no
182259932	chr1	244175168	244175213	7	46	6.571429	no	no	no	no	no	no	no	yes	yes	no
182259934	chr1	244175851	244175908	19	58	3.052632	no	no	no	no	no	no	no	no	no	no
182259935	chr1	244177512	244177558	21	47	2.238095	no	no	no	no	no	no	no	no	no	no
182259939	chr1	244186160	244186218	28	59	2.107143	no	no	yes	no	no	no	no	no	no	yes
182259942	chr1	244190551	244190802	50	252	5.040000	no	no	no	no	no	no	no	no	no	no
182259948	chr1	244212868	244212897	13	30	2.307692	no	no	no	no	no	no	no	yes	yes	no
182259949	chr1	244222744	244222775	17	32	1.941176	no	no	no	no	no	no	no	no	no	no
182259952	chr1	244232384	244232421	17	38	2.352941	no	no	no	no	no	no	no	yes	no	no
182259954	chr1	244233264	244233318	27	55	2.074074	no	no	no	no	no	no	no	yes	no	no
182259969	chr1	244276509	244276552	22	44	2.090909	no	no	no	no	no	no	no	yes	yes	no
182259971	chr1	244281188	244281228	22	41	1.954545	no	no	no	no	no	no	no	yes	no	no
182259972	chr1	244288290	244288316	14	27	1.928571	no	no	no	no	no	no	no	no	no	no
182259994	chr1	244323629	244323655	12	27	2.250000	no	no	no	no	no	no	no	yes	no	no
182260009	chr1	244332687	244332768	31	82	2.687500	no	no	no	no	no	no	no	yes	no	no
182260018	chr1	244358629	244358667	19	39	2.105263	no	no	yes	no	no	no	no	no	no	yes
182260030	chr1	244386955	244386997	22	43	2.000000	no	no	yes	no	no	no	no	yes	no	yes
182260046	chr1	244401468	244401504	9	37	4.100000	no	no	yes	no	no	no	no	yes	no	yes
182260048	chr1	244405920	244405958	19	39	2.157895	no	no	yes	no	no	no	no	no	no	yes
182260049	chr1	244419568	244419615	21	48	2.285714	yes	no	yes	no	no	no	no	yes	no	yes
182260050	chr1	244421031	244421073	21	43	2.047619	no	no	yes	no	no	no	no	no	no	yes
182260055	chr1	244435511	244435666	38	156	4.048780	no	no	yes	no	no	no	no	yes	yes	yes
182260064	chr1	244474399	244474441	20	43	2.150000	no	no	yes	no	no	no	no	no	no	yes

182260066	chr1	244474522	244474583	31	62	2.064516	no	no	yes	no	no	no	no	no	no	yes
182260072	chr1	244486252	244487314	59	1063	18.016949	no	no	yes	no	no	no	no	no	no	yes
182260076	chr1	244503043	244503075	15	33	2.200000	no	no	yes	no	no	no	no	yes	yes	yes
182260083	chr1	244515817	244515875	22	59	2.727273	no	no	yes	no	no	no	no	no	no	yes
182260094	chr1	244552822	244552871	19	50	2.600000	no	no	yes	no	no	yes	no	yes	no	yes
182260101	chr1	244565712	244565771	8	60	7.444445	no	no	yes	no	no	no	no	no	no	yes
182260118	chr1	244584008	244584070	26	63	2.423077	no	no	yes	no	no	no	no	no	no	yes
182260128	chr1	244636508	244636586	39	79	2.025641	no	yes	yes	no	no	no	no	yes	no	yes
182260132	chr1	244648640	244648711	34	72	2.147059	no	no	no	no	no	no	no	yes	no	no
182260143	chr1	244688614	244688648	18	35	1.944444	no	no	yes	no	no	no	no	no	no	yes
182260144	chr1	244692057	244692093	17	37	2.176471	no	no	yes	no	no	yes	no	no	no	yes
182260148	chr1	244707821	244707859	19	39	2.052632	no	yes	no	no	no	no	no	no	yes	yes
182260169	chr1	244742763	244742815	28	53	1.964286	no	no	no	no	no	no	no	no	no	no
182260195	chr1	244769340	244769379	21	40	1.904762	no	no	no	no	no	no	no	no	no	no
182260208	chr1	244803708	244803776	34	69	2.029412	no	no	no	no	no	no	no	no	no	no
182260215	chr1	244807261	244807332	38	72	1.921053	no	no	no	no	no	no	no	yes	no	no
182260234	chr1	244837558	244837589	16	32	2.000000	no	no	yes	no	no	no	no	yes	yes	yes
182260235	chr1	244849797	244849833	13	37	2.846154	yes	no	yes	no	yes	yes	no	yes	no	yes
182260236	chr1	244851830	244851888	31	59	1.903226	no	yes	yes	no	yes	no	no	yes	yes	yes
182260279	chr1	244916164	244916195	16	32	2.000000	no	no	no	no	no	no	no	no	no	no
182260282	chr1	244918708	244918852	20	145	7.250000	no	no	no	no	no	no	yes	yes	yes	no
182260283	chr1	244922842	244922902	14	61	4.357143	no	no	no	no	no	no	no	yes	no	no
182260293	chr1	244937049	244937243	18	195	11.000000	no	no	no	no	no	no	no	yes	no	no
182260316	chr1	244966677	244966769	45	93	2.155555	no	no	no	no	no	no	no	no	no	no
182260321	chr1	244970621	244970690	23	70	3.086957	no	no	yes	no	yes	yes	yes	yes	yes	yes

182260326	chr1	244973187	244973238	25	52	2.080000	no	no	yes	no	no	no	no	yes	yes	yes
182260327	chr1	244973948	244973989	22	42	1.909091	no	no	yes	no	no	no	no	yes	no	yes
182260335	chr1	244980463	244980490	14	28	2.000000	no	no	yes	no	no	no	no	yes	no	yes
182260348	chr1	245014636	245014675	20	40	2.000000	no	no	yes	no	no	no	no	no	no	yes
182260361	chr1	245044938	245044970	17	33	1.941176	no	no	yes	no	no	yes	no	yes	no	yes
182260364	chr1	245052016	245052093	39	78	2.000000	no	no	yes	no	no	no	no	yes	yes	yes
182260371	chr1	245081101	245081166	24	66	2.791667	no	no	yes	no	no	no	no	no	no	yes
182260375	chr1	245090205	245090255	20	51	2.550000	no	no	yes	no	no	no	no	yes	no	yes
182260376	chr1	245090324	245090350	14	27	1.928571	no	no	yes	no	no	no	no	yes	no	yes
182260384	chr1	245102848	245103736	89	889	10.033708	no	no	yes	no	no	no	no	yes	no	yes
182260388	chr1	245106482	245106516	18	35	2.000000	yes	no	yes	no	no	no	no	no	no	yes
182260389	chr1	245106506	245106545	16	40	2.687500	yes	no	yes	no	no	no	no	no	no	yes
182260404	chr1	245134139	245134180	19	42	2.210526	yes	no	no	no	no	no	no	yes	no	no
182260408	chr1	245140372	245140443	36	72	2.000000	no	no	no	no	no	no	no	no	no	no
182260409	chr1	245142319	245142367	24	49	2.041667	no	no	no	no	no	no	no	yes	yes	no
182260428	chr1	245148561	245148602	21	42	2.047619	no	no	no	no	no	no	no	no	no	no
182260430	chr1	245156413	245156458	22	46	2.181818	no	no	no	yes	no	no	yes	yes	no	yes
182260431	chr1	245156522	245156561	21	40	1.904762	no	no	no	yes	no	no	yes	yes	yes	yes
182260436	chr1	245168292	245168334	20	43	2.150000	no	no	yes	no	no	no	no	yes	yes	yes
182260441	chr1	245175902	245176006	38	105	2.763158	no	no	yes	no	no	no	no	no	no	yes
182260449	chr1	245199304	245199329	11	26	2.363636	no	no	yes	no	no	no	no	yes	yes	yes
182260458	chr1	245220411	245220455	7	45	6.428571	no	no	yes	no	no	no	no	no	no	yes
182260493	chr1	245271409	245271453	14	45	3.200000	no	no	yes	no	no	no	no	yes	no	yes
182260502	chr1	245283199	245283341	30	143	4.766667	yes	no	yes	no	no	no	no	yes	no	yes
182260506	chr1	245288792	245288827	19	36	1.947368	no	no	yes	no	no	no	no	no	no	yes

182260518	chr1	245309829	245309872	14	44	3.142857	no	no	yes	no	no	no	no	yes	no	yes
182260526	chr1	245311399	245311455	30	57	1.966667	no	no	yes	no	no	no	no	yes	yes	yes
182260529	chr1	245323842	245324238	111	397	3.589286	no	no	yes	no	no	no	no	yes	no	yes
182260541	chr1	245330459	245330606	76	148	1.973684	no	no	yes	no	no	no	no	no	yes	yes
182260545	chr1	245335739	245335773	16	35	2.187500	no	no	yes	no	no	no	yes	yes	no	yes
182260549	chr1	245344097	245344137	19	41	2.210526	no	no	yes	no	no	no	no	yes	no	yes
182260556	chr1	245351363	245351516	38	154	4.052631	no	no	yes	no	no	no	no	yes	no	yes
182260561	chr1	245361624	245361661	20	38	1.900000	no	no	yes	no	no	no	no	no	no	yes
182260570	chr1	245383206	245383345	69	140	2.028986	no	no	yes	no	no	no	no	no	no	yes
182260571	chr1	245384186	245384243	26	58	2.230769	no	no	yes	no	no	no	no	yes	yes	yes
182260576	chr1	245398720	245398787	31	68	2.187500	no	no	yes	no	no	no	no	no	no	yes
182260579	chr1	245412658	245412700	18	43	2.388889	no	no	yes	no	no	no	no	yes	no	yes
182260580	chr1	245416826	245416858	17	33	1.941176	no	no	yes	no	no	no	no	no	yes	yes
182260582	chr1	245426177	245426212	14	36	2.642857	no	no	yes	no	no	no	no	no	no	yes
182260591	chr1	245441326	245441360	18	35	1.944444	no	no	yes	no	no	no	no	no	no	yes
182260592	chr1	245442644	245444236	29	1593	55.060608	no	no	yes	no	no	no	no	no	yes	yes
182260595	chr1	245450851	245450922	37	72	2.000000	no	no	yes	no	no	no	no	no	no	yes
182260605	chr1	245464844	245464872	14	29	2.071429	no	no	yes	no	no	no	no	yes	no	yes
182260607	chr1	245469197	245469309	55	113	2.072727	no	no	yes	no	no	no	no	yes	yes	yes
182260610	chr1	245479645	245479680	18	36	2.055556	no	no	yes	no	no	no	no	no	no	yes
182260611	chr1	245484620	245484669	25	50	2.000000	no	no	yes	no	no	no	no	no	no	yes
182260615	chr1	245486637	245486672	14	36	2.714286	no	no	yes	no	no	no	no	no	no	yes
182260623	chr1	245504304	245504409	56	106	1.892857	no	no	yes	no	no	no	no	no	no	yes
182260628	chr1	245513133	245513161	12	29	2.416667	no	no	yes	no	no	no	no	yes	yes	yes
182260631	chr1	245520767	245520835	34	69	2.029412	no	no	yes	no	no	no	no	yes	yes	yes

182260632	chr1	245526857	245526910	23	54	2.391304	no	no	yes	no	no	no	no	no	no	yes
182260639	chr1	245546634	245546666	17	33	1.941176	no	no	yes	no	no	no	no	no	no	yes
182260641	chr1	245553524	245553565	14	42	3.071429	no	no	yes	no	no	no	no	no	no	yes
182260642	chr1	245556112	245556140	9	29	3.222222	no	no	yes	no	no	no	no	yes	no	yes
182260654	chr1	245570816	245570851	15	36	2.400000	no	no	yes	no	no	no	no	no	no	yes
182260655	chr1	245572607	245572633	14	27	1.928571	no	no	yes	no	no	no	no	yes	no	yes
182260659	chr1	245577510	245578061	41	552	13.547619	no	no	yes	no	no	no	yes	yes	no	yes
182260665	chr1	245605566	245605607	16	42	2.625000	no	no	yes	no	no	no	no	yes	yes	yes
182260668	chr1	245614858	245615030	34	173	5.088235	no	no	yes	no	yes	no	no	yes	yes	yes
182260671	chr1	245616087	245616157	31	71	2.290323	yes	no	yes	no	no	no	no	no	no	yes
182260672	chr1	245617026	245617058	15	33	2.266667	no	no	yes	no	no	no	no	no	no	yes
182260674	chr1	245618249	245619296	84	1048	12.976745	no	no	yes	no	no	no	no	no	no	yes
182260687	chr1	245650003	245650037	18	35	1.944444	no	no	yes	no	no	no	no	no	no	yes
182260694	chr1	245662012	245662066	25	55	2.240000	no	no	yes	no	no	no	no	no	no	yes
182260697	chr1	245662178	245662209	16	32	2.062500	no	no	yes	no	no	no	no	no	no	yes
182260704	chr1	245673011	245673458	31	448	15.000000	no	no	yes	no	no	yes	yes	yes	yes	yes
182260711	chr1	245680432	245680523	44	92	2.090909	no	no	yes	no	no	no	no	yes	yes	yes
182260712	chr1	245682395	245682752	61	358	5.868853	no	no	yes	no	no	no	no	yes	no	yes
182260713	chr1	245688119	245688158	21	40	1.904762	no	no	no	yes	no	yes	yes	yes	yes	yes
182260714	chr1	245688846	245689007	81	162	2.000000	no	no	yes	no	no	no	yes	yes	yes	yes
182260715	chr1	245692602	245692659	28	58	2.071429	no	no	yes	no	no	no	no	no	no	yes
182260716	chr1	245695736	245695999	67	264	4.014925	no	no	yes	no	no	no	no	no	no	yes
182260717	chr1	245698648	245698707	21	60	2.857143	no	no	yes	no	no	no	no	yes	no	yes
182260721	chr1	245715508	245715533	13	26	2.000000	no	no	no	no	no	no	no	yes	yes	no
182260738	chr1	245753542	245753692	77	151	1.961039	no	no	yes	no	no	no	no	yes	no	yes

182260742	chr1	245767725	245767860	68	136	2.058824	no	no	yes	no	no	no	no	no	yes	yes
182260747	chr1	245773916	245773950	18	35	1.944444	no	no	yes	no	no	no	no	no	no	yes
182260755	chr1	245779571	245779598	9	28	3.111111	no	no	yes	no	no	no	no	no	no	yes
182260758	chr1	245788009	245788319	38	311	8.184211	no	no	yes	no	no	no	no	yes	yes	yes
182260761	chr1	245795804	245795908	51	105	2.058824	no	no	yes	no	no	no	no	no	no	yes
182260764	chr1	245799732	245799947	106	216	2.037736	no	no	yes	no	no	no	no	no	yes	yes
182260777	chr1	245814805	245814885	36	81	2.305556	yes	no	yes	no	no	no	no	yes	yes	yes
182260780	chr1	245825395	245825460	31	66	2.129032	no	no	yes	no	no	no	no	yes	no	yes
182260781	chr1	245825464	245825556	47	93	2.000000	no	no	yes	no	no	no	no	yes	yes	yes
182260784	chr1	245847910	245848022	58	113	2.034483	no	no	yes	no	no	no	no	no	no	yes
182260788	chr1	245865126	245865172	10	47	5.100000	no	no	yes	no	no	no	no	yes	no	yes
182260790	chr1	245865178	245865239	31	62	2.000000	no	no	yes	no	no	no	no	yes	no	yes
182260792	chr1	245872332	245872506	85	175	2.070588	no	no	yes	no	no	no	no	no	no	yes
182260793	chr1	245875774	245875810	19	37	2.000000	no	no	yes	no	no	no	no	yes	yes	yes
182260795	chr1	245879897	245880137	32	241	7.531250	no	no	yes	no	no	no	no	yes	no	yes
182260816	chr1	245967353	245967382	14	30	2.142857	no	no	yes	no	no	no	no	no	no	yes
182260817	chr1	245974466	245977071	24	2606	108.583336	no	no	yes	no	no	no	yes	no	no	yes
182260820	chr1	245992483	245993007	47	525	11.191489	no	no	yes	no	no	no	no	no	no	yes
182260824	chr1	246000351	246000389	19	39	2.210526	no	no	yes	no	no	no	no	yes	no	yes
182260825	chr1	246001937	246001963	13	27	2.076923	no	no	yes	no	no	no	no	yes	yes	yes
182260827	chr1	246006282	246006324	17	43	2.529412	no	no	yes	no	no	no	no	no	no	yes
182260828	chr1	246011392	246011469	37	78	2.108108	no	no	yes	no	no	no	no	no	yes	yes
182260829	chr1	246011912	246012072	53	161	3.037736	no	no	yes	no	no	no	no	yes	no	yes
182260834	chr1	246024294	246025342	32	1049	33.000000	yes	no	yes	no	no	no	no	yes	yes	yes
182260841	chr1	246046629	246046681	26	53	2.038461	no	no	yes	no	no	no	no	no	no	yes

182260843	chr1	246057258	246057299	20	42	2.100000	no	no	yes	no	no	no	no	no	no	yes
182260850	chr1	246071729	246072472	57	744	13.105263	no	no	yes	no	no	no	no	yes	yes	yes
182260853	chr1	246075221	246075256	16	36	2.250000	no	no	yes	no	no	no	no	yes	no	yes
182260855	chr1	246081182	246081206	13	25	1.923077	no	no	yes	no	no	no	no	yes	no	yes
182260861	chr1	246096740	246096797	19	58	3.052632	yes	no	yes	no	no	no	no	yes	no	yes
182260867	chr1	246127466	246127490	13	25	1.923077	no	no	yes	no	no	no	no	no	no	yes
182260871	chr1	246135668	246135730	24	63	2.625000	no	no	yes	no	no	no	no	yes	no	yes
182260873	chr1	246139885	246139999	54	115	2.129630	no	no	yes	no	no	no	no	yes	yes	yes
182260878	chr1	246161011	246161053	22	43	1.954545	no	no	yes	no	no	no	no	yes	yes	yes
182260884	chr1	246180159	246180235	24	77	3.240000	no	no	yes	no	no	yes	no	no	no	yes
182260885	chr1	246180262	246180382	31	121	3.967742	no	no	yes	no	no	no	no	no	no	yes
182260891	chr1	246199769	246200684	63	916	15.015873	no	no	yes	no	no	no	no	yes	yes	yes
182260903	chr1	246232442	246234620	49	2179	44.959183	no	no	yes	no	no	no	no	yes	no	yes
182260909	chr1	246246820	246246868	25	49	2.040000	no	no	yes	no	no	no	no	yes	no	yes
182260910	chr1	246249064	246249097	18	34	1.888889	no	no	yes	no	no	no	no	no	no	yes
182260911	chr1	246251400	246252297	31	898	28.967741	no	no	yes	no	no	no	yes	no	no	yes
182260933	chr1	246282508	246282543	19	36	1.947368	no	no	yes	no	no	no	no	no	no	yes
182260935	chr1	246291866	246292532	29	667	23.000000	no	no	yes	no	no	no	no	yes	no	yes
182260937	chr1	246296409	246296441	17	33	2.000000	no	no	yes	no	no	no	no	no	no	yes
182260939	chr1	246308455	246308503	23	49	2.130435	no	no	yes	no	no	no	no	yes	no	yes
182260941	chr1	246308863	246309022	54	160	2.981482	no	no	yes	no	no	no	no	yes	no	yes
182260942	chr1	246311030	246311065	18	36	2.000000	no	no	yes	no	no	no	no	yes	no	yes
182260947	chr1	246324967	246325317	29	351	12.275862	no	no	yes	no	no	yes	no	yes	yes	yes
182260952	chr1	246344955	246344998	20	44	2.200000	no	no	yes	no	no	no	no	no	no	yes
182260966	chr1	246365241	246365287	24	47	2.000000	no	no	yes	no	no	no	no	no	no	yes

182260967	chr1	246369330	246369369	18	40	2.277778	no	no	yes	no	no	no	no	no	no	yes
182260979	chr1	246385303	246385360	28	58	2.071429	no	no	yes	no	no	no	no	no	no	yes
182260980	chr1	246385408	246385460	17	53	3.055556	no	no	yes	no	no	no	no	no	no	yes
182260981	chr1	246387836	246389890	62	2055	32.953125	no	no	yes	no	no	no	no	no	no	yes
182260991	chr1	246395323	246395348	13	26	2.000000	no	no	yes	no	no	no	no	no	no	yes
182260992	chr1	246395580	246395837	28	258	9.321428	no	no	yes	no	no	no	no	yes	no	yes
182260994	chr1	246400028	246400060	15	33	2.200000	no	no	yes	no	no	no	no	no	no	yes
182260995	chr1	246404634	246404686	22	53	2.409091	no	no	yes	no	no	no	no	no	no	yes
182261001	chr1	246429759	246430206	56	448	8.000000	no	no	yes	no	no	no	no	no	no	yes
182261004	chr1	246435703	246435820	31	118	3.806452	no	no	yes	no	no	no	no	yes	yes	yes
182261006	chr1	246455559	246455596	18	38	2.111111	no	no	yes	no	no	no	no	yes	yes	yes
182261007	chr1	246457050	246457096	24	47	1.958333	no	no	yes	no	no	no	no	no	no	yes
182261016	chr1	246462178	246462328	31	151	4.870968	no	no	yes	no	no	no	no	no	no	yes
182261024	chr1	246470556	246470648	45	93	2.111111	no	no	yes	no	no	no	no	yes	no	yes
182261028	chr1	246473660	246473697	19	38	2.000000	no	no	yes	no	no	no	no	yes	no	yes
182261029	chr1	246478335	246479230	47	896	19.062500	no	no	yes	no	no	no	no	yes	no	yes
182261034	chr1	246481644	246481757	46	114	2.489362	no	no	yes	no	no	no	no	no	no	yes
182261035	chr1	246482319	246482356	20	38	1.900000	no	no	yes	no	no	no	no	yes	no	yes
182261036	chr1	246483932	246485337	52	1406	27.038462	yes	no	yes	no	no	no	no	yes	yes	yes
182261041	chr1	246493638	246493891	65	254	4.000000	no	no	yes	no	no	no	no	no	no	yes
182261046	chr1	246519876	246522091	36	2216	61.555557	no	no	yes	no	no	no	no	yes	no	yes
182261056	chr1	246542237	246542280	23	44	1.913043	no	no	yes	no	no	no	no	no	no	yes
182261058	chr1	246542855	246542893	18	39	2.166667	no	no	yes	no	no	no	no	yes	no	yes
182261069	chr1	246586202	246586260	32	59	1.906250	no	no	yes	no	no	no	no	no	no	yes
182261075	chr1	246595903	246595937	18	35	1.944444	no	no	yes	no	no	no	no	no	yes	yes

182261087	chr1	246619811	246620934	54	1124	20.966103	no	no	yes	no	no	no	yes	no	no	yes
182261094	chr1	246636819	246636874	29	56	1.931034	no	no	yes	no	no	no	no	no	no	yes
182261096	chr1	246642811	246642868	12	58	4.769231	no	no	yes	no	no	no	no	yes	no	yes
182261098	chr1	246658700	246658741	17	42	2.470588	no	no	yes	no	no	no	no	yes	yes	yes
182261099	chr1	246658722	246658763	22	42	1.909091	no	no	yes	no	no	no	no	yes	yes	yes
182261101	chr1	246669766	246669876	57	111	2.000000	no	no	no	no	no	no	no	no	no	no
182261110	chr1	246694937	246695045	49	109	2.224490	no	no	no	no	no	no	no	yes	no	no
182261116	chr1	246705336	246705502	79	167	2.113924	no	no	no	no	no	no	no	yes	yes	no
182261141	chr1	246725211	246725343	34	133	3.911765	no	no	yes	no	no	no	no	yes	yes	yes
182261145	chr1	246735328	246735370	23	43	1.913043	no	no	yes	no	no	no	no	yes	yes	yes
182261156	chr1	246745158	246745217	22	60	2.727273	no	no	yes	no	no	no	no	no	no	yes
182261160	chr1	246758487	246758605	39	119	3.051282	no	no	yes	no	no	no	no	no	no	yes
182261161	chr1	246761027	246761206	91	180	2.000000	no	no	yes	no	no	no	no	yes	yes	yes
182261162	chr1	246762338	246762390	27	53	1.962963	no	no	yes	no	no	no	no	yes	no	yes
182261163	chr1	246764856	246765254	44	399	9.068182	no	no	yes	no	no	no	no	yes	no	yes
182261164	chr1	246768252	246768371	40	120	3.024390	no	no	no	no	no	yes	no	yes	no	no
182261165	chr1	246772389	246772564	63	176	2.793651	no	yes	yes	no	no	no	no	no	yes	yes
182261170	chr1	246782946	246783650	54	705	13.017858	yes	yes	yes	no	yes	no	no	yes	yes	yes
182261171	chr1	246785471	246785540	36	70	1.944444	no	no	yes	no	no	no	no	yes	yes	yes
182261172	chr1	246785645	246786006	30	362	12.066667	no	no	yes	no	yes	no	no	yes	yes	yes
182261174	chr1	246792742	246792897	30	156	5.129032	no	no	yes	no	no	yes	no	no	yes	yes
182261183	chr1	246794062	246794119	28	58	2.107143	no	no	yes	no	no	no	no	no	yes	yes
182261187	chr1	246800821	246801009	65	189	2.893939	no	no	yes	no	no	no	no	no	yes	yes
182261188	chr1	246802334	246802379	20	46	2.333333	no	no	yes	no	no	no	no	no	no	yes
182261192	chr1	246809568	246809731	59	164	2.779661	no	no	yes	no	no	no	no	yes	yes	yes

182261196	chr1	246814084	246814141	30	58	1.933333	no	no	yes	no	no	no	no	yes	yes	yes
182261197	chr1	246817942	246818014	30	73	2.451613	yes	no	yes	no	no	no	no	yes	yes	yes
182261213	chr1	246838800	246838999	85	200	2.447059	no	no	yes	no	no	yes	no	yes	yes	yes
182261214	chr1	246845199	246845352	38	154	4.052631	no	no	yes	no	no	no	no	no	no	yes
182261215	chr1	246849996	246850029	18	34	1.944444	yes	no	no	yes	yes	no	no	no	no	yes
182261216	chr1	246852350	246852427	39	78	2.000000	no	no	yes	no	no	no	no	no	no	yes
182261218	chr1	246856375	246856426	28	52	1.928571	no	no	yes	no	no	no	no	no	no	yes
182261220	chr1	246856543	246856603	30	61	2.033333	no	no	yes	no	no	no	no	no	yes	yes
182261222	chr1	246866374	246866419	20	46	2.350000	no	no	yes	no	no	no	no	no	yes	yes
182261225	chr1	246873347	246873716	73	370	5.067567	no	no	yes	no	no	no	no	no	no	yes
182261228	chr1	246877393	246877435	23	43	1.913043	yes	no	yes	no	no	no	no	yes	no	yes
182261229	chr1	246877901	246877936	14	36	2.571429	yes	no	yes	no	no	no	no	no	no	yes
182261237	chr1	246903868	246903910	22	43	2.045455	no	no	yes	no	no	no	no	no	no	yes
182261238	chr1	246904550	246904590	21	41	2.000000	no	no	yes	no	no	no	no	yes	no	yes
182261241	chr1	246910988	246911030	21	43	2.142857	no	no	yes	no	no	no	no	no	no	yes
182261243	chr1	246919803	246919832	11	30	2.727273	no	no	yes	no	no	no	no	yes	no	yes
182261245	chr1	246923006	246923051	23	46	2.000000	no	no	yes	no	no	no	no	no	no	yes
182261265	chr1	246958911	246959001	44	91	2.068182	no	no	yes	no	no	no	no	no	no	yes
182261271	chr1	246974143	246974195	22	53	2.500000	no	no	yes	no	no	no	no	no	no	yes
182261274	chr1	246975876	246975953	26	78	3.000000	no	no	yes	no	no	no	no	no	no	yes
182261326	chr1	247073335	247073478	70	144	2.071429	no	no	yes	no	no	no	no	yes	no	yes
182261328	chr1	247085754	247085786	13	33	2.692308	no	no	yes	no	no	no	no	yes	no	yes
182261329	chr1	247087137	247087172	16	36	2.312500	no	no	no	no	no	no	no	no	no	no
182261338	chr1	247115870	247118988	46	3119	67.914894	yes	no	no	no	no	no	yes	yes	yes	no
182261340	chr1	247120248	247120552	61	305	5.000000	no	yes	no	no	no	yes	no	yes	no	yes

182261345	chr1	247128851	247130024	62	1174	18.951612	no	no	yes	no	no	no	no	no	no	yes
182261353	chr1	247175617	247175664	13	48	3.692308	no	no	no	no	no	yes	no	yes	yes	no
182261354	chr1	247176044	247176079	14	36	2.571429	no	no	no	no	no	yes	no	no	no	no
182261372	chr1	247199609	247199773	82	165	2.012195	no	no	yes	no	no	no	no	no	yes	yes
182261374	chr1	247202618	247202643	13	26	2.000000	yes	no	yes	no	no	no	no	no	no	yes
182261390	chr1	247258637	247258673	9	37	4.200000	no	no	no	no	no	no	no	yes	no	no
182261408	chr1	247276361	247276389	15	29	1.933333	no	no	no	no	no	no	no	no	no	no
182261414	chr1	247277092	247277134	15	43	2.866667	no	no	no	no	no	no	no	yes	no	no
182261422	chr1	247283926	247283961	17	36	2.117647	no	no	no	no	no	no	no	no	no	no
182261428	chr1	247294243	247294312	36	70	1.944444	yes	no	no	no	no	no	no	yes	no	no
182261436	chr1	247314427	247314466	14	40	2.928571	yes	no	yes	no	no	no	no	no	no	yes
182261439	chr1	247321594	247321629	17	36	2.117647	no	no	yes	no	no	no	no	no	no	yes
182261445	chr1	247327717	247327877	80	161	2.025000	no	no	yes	no	no	yes	no	no	no	yes
182261446	chr1	247329507	247329541	17	35	2.058824	yes	no	no	yes	yes	no	no	yes	yes	yes
182261448	chr1	247336513	247336582	35	70	2.000000	no	no	no	no	no	no	no	yes	no	no
182261450	chr1	247339677	247340196	56	520	9.321428	no	no	yes	no	no	no	no	yes	no	yes
182261461	chr1	247363122	247363327	29	206	7.206897	no	no	no	no	no	no	no	yes	no	no
182261466	chr1	247371465	247371595	43	131	3.046512	no	no	no	no	no	no	no	yes	yes	no
182261471	chr1	247373854	247373883	7	30	4.285714	no	no	no	no	no	no	no	yes	yes	no
182261478	chr1	247393835	247393882	23	48	2.086957	no	yes	no	no	no	yes	no	no	no	yes
182261482	chr1	247398435	247398900	39	466	11.948718	no	no	no	no	no	no	no	yes	no	no
182261487	chr1	247404163	247404192	15	30	2.066667	no	no	no	no	no	no	no	yes	no	no
182261490	chr1	247408641	247408669	15	29	1.933333	no	no	no	no	no	no	no	yes	no	no
182261493	chr1	247416615	247416753	63	139	2.222222	no	no	yes	no	no	no	no	yes	yes	yes
182261495	chr1	247427462	247428335	73	874	12.000000	no	no	yes	no	no	no	no	no	no	yes

182261499	chr1	247433579	247434081	42	503	11.976191	no	no	yes	no	no	no	no	yes	no	yes
182261500	chr1	247436494	247436570	33	77	2.333333	no	no	yes	no	no	no	no	no	yes	yes
182261502	chr1	247437502	247437562	31	61	1.967742	no	no	yes	no	no	no	no	yes	yes	yes
182261504	chr1	247440022	247440144	18	123	6.833333	no	no	yes	no	no	no	no	yes	no	yes
182261511	chr1	247455491	247455534	22	44	2.000000	no	no	yes	no	no	no	no	no	no	yes
182261513	chr1	247458338	247458759	40	422	10.340000	yes	no	no	no	no	no	no	no	no	no
182261517	chr1	247468388	247468419	14	32	2.285714	no	no	no	no	no	no	no	no	no	no
182261522	chr1	247470982	247471864	52	883	16.980770	no	no	no	no	no	no	no	yes	yes	no
182261525	chr1	247480299	247480328	14	30	2.142857	no	no	no	no	no	no	no	no	no	no
182261530	chr1	247487786	247487820	18	35	1.944444	yes	no	no	no	no	no	no	yes	no	no
182261531	chr1	247494068	247494111	15	44	3.000000	no	no	no	no	no	no	no	no	no	no
182261534	chr1	247505321	247505348	14	28	2.000000	no	no	no	no	no	no	no	no	no	no
182261538	chr1	247521048	247521090	23	43	1.913043	no	no	yes	no	no	no	no	no	no	yes
182261557	chr1	247553518	247553559	15	42	2.933333	no	no	yes	no	no	no	no	no	no	yes
182261568	chr1	247590529	247590560	15	32	2.133333	no	no	yes	no	no	no	no	no	no	yes
182261571	chr1	247602196	247602250	23	55	2.434783	no	no	yes	no	no	no	no	no	no	yes
182261577	chr1	247620537	247620576	20	40	2.000000	no	yes	yes	no	no	yes	no	no	no	yes
182261578	chr1	247620876	247620913	19	38	2.105263	no	yes	yes	no	no	yes	no	no	no	yes
182261579	chr1	247621286	247621318	17	33	2.058824	no	yes	yes	no	no	yes	no	no	no	yes
182261584	chr1	247639109	247639198	44	90	2.045455	yes	no	yes	no	no	no	no	yes	no	yes
182261586	chr1	247656036	247656107	37	72	1.945946	no	no	yes	no	no	no	no	yes	no	yes
182261587	chr1	247656153	247656189	11	37	3.636364	no	no	yes	no	no	no	no	yes	no	yes
182261597	chr1	247670719	247670778	21	60	2.782609	no	no	yes	no	no	yes	no	no	no	yes
182261599	chr1	247677539	247677598	30	60	2.000000	no	no	yes	no	no	no	no	yes	no	yes
182261605	chr1	247694454	247694544	43	91	2.139535	no	no	yes	no	no	no	no	yes	no	yes

182261609	chr1	247700702	247700811	26	110	4.222222	no	no	yes	no	no	no	no	no	no	yes
182261615	chr1	247700920	247700984	23	65	2.791667	no	no	yes	no	no	no	no	no	no	yes
182261617	chr1	247700966	247701203	25	238	9.615385	no	no	yes	no	no	no	no	no	no	yes
182261620	chr1	247725485	247725546	32	62	2.000000	no	no	yes	no	no	no	no	no	no	yes
182261621	chr1	247735996	247736148	76	153	2.013158	no	no	yes	no	no	no	no	no	no	yes
182261623	chr1	247740162	247740251	45	90	2.022222	no	no	yes	no	no	yes	no	no	no	yes
182261624	chr1	247740322	247740360	19	39	2.052632	no	no	yes	no	no	yes	no	no	no	yes
182261625	chr1	247741146	247741174	10	29	3.000000	no	no	yes	no	no	no	no	no	no	yes
182261626	chr1	247742175	247742219	23	45	2.086957	no	no	yes	no	no	no	no	no	no	yes
182261627	chr1	247742242	247742536	100	295	3.050000	no	no	yes	no	no	no	no	yes	yes	yes
182261629	chr1	247743211	247743247	15	37	2.466667	no	no	yes	no	no	no	no	no	no	yes
182261630	chr1	247746271	247746301	16	31	2.000000	yes	yes	yes	no	no	no	no	no	no	yes
182261637	chr1	247761582	247761645	29	64	2.275862	no	no	no	no	no	yes	no	no	no	no
182261642	chr1	247772974	247773010	18	37	2.055556	no	no	yes	no	no	no	no	no	no	yes
182261645	chr1	247774830	247774897	33	68	2.090909	no	no	yes	no	yes	no	no	no	no	yes
182261656	chr1	247805687	247805747	22	61	2.818182	no	no	no	no	no	no	no	yes	no	no
182261660	chr1	247824579	247824617	18	39	2.222222	yes	no	no	no	no	no	no	yes	no	no
182261661	chr1	247825529	247825612	16	84	5.312500	no	no	no	no	no	no	no	yes	yes	no
182261672	chr1	247840302	247840359	29	58	2.000000	no	no	no	no	no	yes	no	yes	no	no
182261675	chr1	247846722	247846768	17	47	2.684211	no	no	no	no	no	no	no	no	no	no
182261677	chr1	247850051	247850127	38	77	2.052632	no	no	no	no	no	no	no	yes	no	no
182261678	chr1	247851918	247851963	14	46	3.200000	no	no	no	no	no	no	no	no	no	no
182261686	chr1	247859779	247859847	27	69	2.592592	no	no	yes	no	no	no	no	no	no	yes
182261687	chr1	247861238	247861285	18	48	2.666667	no	no	yes	no	no	no	no	no	no	yes
182261689	chr1	247868053	247868164	57	112	1.964912	no	no	yes	yes	no	no	no	no	yes	yes

182261691	chr1	247869548	247869650	51	103	2.019608	no	no	yes	no	no	no	no	yes	no	yes
182261692	chr1	247870261	247871078	75	818	10.906667	no	no	yes	no	no	no	no	no	no	yes
182261701	chr1	247894398	247894650	54	253	4.685185	yes	no	no	no	no	no	no	no	no	no
182261710	chr1	247906257	247906303	21	47	2.238095	no	no	no	no	no	no	no	yes	yes	no
182261714	chr1	247910405	247910551	77	147	1.974026	no	no	no	no	no	no	no	no	no	no
182261723	chr1	247935186	247935230	18	45	2.500000	no	yes	no	no	no	yes	no	no	no	yes
182261728	chr1	247937029	247937224	96	196	2.041667	no	no	no	no	yes	no	yes	yes	yes	yes
182261730	chr1	247937456	247937695	91	240	2.637363	yes	no	no	no	yes	no	no	yes	yes	yes
182261733	chr1	247940124	247940158	14	35	2.500000	no	no	no	no	no	yes	no	no	no	no
182261734	chr1	247940434	247940473	21	40	2.000000	no	no	no	no	no	yes	no	no	no	no
182261736	chr1	247941650	247941694	21	45	2.142857	no	no	no	no	no	no	no	no	no	no
182261738	chr1	247953515	247953562	24	48	2.000000	no	no	no	no	no	no	no	yes	no	no
182261749	chr1	247968132	247968168	19	37	1.947368	no	no	no	no	no	no	no	no	no	no
182261750	chr1	247982994	247983035	19	42	2.263158	no	no	yes	no	no	no	no	no	no	yes
182261751	chr1	247983055	247983130	36	76	2.166667	no	no	yes	no	no	no	no	no	no	yes
182261755	chr1	248001513	248001608	31	96	3.030303	no	no	no	no	no	no	no	no	no	no
182261757	chr1	248002018	248002054	18	37	2.055556	no	no	no	no	no	no	no	no	no	no
182261761	chr1	248019708	248019758	26	51	1.961538	no	no	yes	no	no	no	no	no	no	yes
182261770	chr1	248031131	248031204	37	74	2.000000	no	no	yes	no	no	no	no	no	no	yes
182261782	chr1	248081122	248081153	9	32	3.555556	no	no	no	no	no	no	no	no	no	no
182261790	chr1	248103011	248103190	36	180	5.111111	no	no	no	no	no	no	no	no	no	no
182261793	chr1	248113605	248113639	19	35	1.947368	no	no	no	no	no	no	no	no	no	no
182261796	chr1	248113751	248113791	19	41	2.157895	no	no	no	no	no	no	no	no	no	no
182261801	chr1	248117764	248117819	30	56	2.000000	no	no	no	no	no	no	no	no	no	no
182261807	chr1	248136780	248136889	17	110	6.444445	no	no	no	no	no	no	no	no	no	no

182261809	chr1	248141388	248141528	72	141	1.958333	no	no	no	no	no	no	no	no	no	no
182261813	chr1	248162212	248162258	22	47	2.136364	no	no	no	no	no	no	no	no	no	no
182261814	chr1	248164191	248165364	17	1174	68.208336	no	no	no	no	no	no	no	yes	no	no
182261817	chr1	248187690	248188862	65	1173	18.000000	no	no	no	no	no	no	no	yes	no	no
182261818	chr1	248198545	248198570	12	26	2.166667	no	no	yes	no	no	no	no	no	no	yes
182261819	chr1	248198674	248199032	73	359	4.958904	no	no	yes	no	no	no	no	yes	no	yes
182261842	chr1	248306428	248306935	17	508	29.666666	no	no	no	no	no	no	no	no	no	no
182261843	chr1	248311115	248311162	23	48	2.086957	no	no	no	no	no	no	no	no	no	no
182261846	chr1	248325073	248325114	21	42	2.000000	yes	no	no	no	no	no	no	no	no	no
182261850	chr1	248346472	248346531	24	60	2.583333	no	no	no	no	no	no	no	no	no	no
182261851	chr1	248346606	248346637	17	32	1.941176	no	no	no	no	no	no	no	no	no	no
182261852	chr1	248352826	248352865	12	40	3.142857	no	no	no	no	no	no	no	no	no	no
182261856	chr1	248354964	248355009	20	46	2.300000	no	no	no	no	no	no	no	yes	no	no
182261858	chr1	248361581	248361748	84	168	2.000000	yes	no	no	yes	no	no	no	no	no	yes
182261862	chr1	248380017	248380187	60	171	2.838710	no	no	yes	no	no	no	no	no	no	yes
182261863	chr1	248382979	248384677	68	1699	24.985294	no	no	yes	no	no	no	no	no	no	yes
182261865	chr1	248385653	248385737	42	85	2.047619	no	no	yes	no	no	no	no	yes	yes	yes
182261867	chr1	248408054	248409124	53	1071	20.207546	no	no	no	no	no	yes	no	yes	no	no
182261874	chr1	248446418	248446613	100	196	1.960000	no	no	yes	no	yes	no	no	yes	yes	yes
182261886	chr1	248464585	248464730	18	146	8.000000	no	no	no	no	no	no	no	no	no	no
182261902	chr1	248522580	248522879	100	300	3.000000	no	yes	no	yes	no	yes	no	yes	yes	yes
182261920	chr1	248599539	248599613	35	75	2.142857	no	no	no	no	no	no	no	no	yes	no
182261926	chr1	248641766	248641804	21	39	1.904762	no	no	yes	no	no	no	no	yes	yes	yes
182261930	chr1	248645443	248645659	115	217	1.886957	yes	no	no	no	no	no	no	no	no	no
182261932	chr1	248671297	248672143	51	847	16.607843	no	no	no	no	no	no	no	no	no	no

182261936	chr1	248674753	248676206	60	1454	24.028986	no	no	no	no	no	no	no	yes	no	no
182261944	chr1	248679443	248679484	17	42	2.444444	no	no	no	no	no	no	no	no	no	no
182261945	chr1	248680634	248680770	70	137	1.957143	no	no	no	no	no	yes	no	no	no	no
182261947	chr1	248683538	248683663	63	126	2.047619	no	no	yes	no	no	no	no	no	no	yes
182261952	chr1	248692331	248692372	21	42	2.047619	no	no	yes	no	no	no	no	yes	yes	yes
182261955	chr1	248693187	248693300	57	114	2.000000	no	no	yes	no	no	no	no	yes	no	yes
182261959	chr1	248702816	248703158	193	343	1.818653	no	no	yes	no	no	no	no	yes	no	yes
182261973	chr1	248740625	248740858	48	234	4.875000	no	no	yes	no	no	no	no	no	yes	yes
182261974	chr1	248742157	248743072	35	916	26.027779	no	no	yes	no	no	no	no	no	yes	yes
182261976	chr1	248744466	248744664	84	199	2.369048	no	no	yes	no	no	no	no	no	no	yes
182261977	chr1	248746565	248748300	31	1736	55.705883	no	no	yes	yes	no	no	yes	yes	yes	yes
182261983	chr1	248754597	248754638	20	42	2.150000	no	no	yes	no	no	no	no	yes	no	yes
182261984	chr1	248754818	248754870	20	53	2.700000	no	no	yes	no	no	no	no	no	yes	yes
182261993	chr1	248791096	248791136	20	41	2.050000	no	no	no	no	no	no	no	yes	yes	no
182261998	chr1	248802810	248803878	41	1069	26.073172	no	no	no	no	no	no	no	yes	yes	no
182261999	chr1	248804737	248804776	17	40	2.352941	no	no	no	no	no	no	no	yes	yes	no
182262000	chr1	248812481	248812568	44	88	2.000000	no	no	yes	no	no	no	no	yes	yes	yes
182262001	chr1	248812784	248812811	13	28	2.153846	no	no	yes	no	no	no	no	no	no	yes
182262004	chr1	248827951	248827991	22	41	1.909091	no	no	no	no	no	no	no	no	no	no
182262011	chr1	248840542	248841190	54	649	12.055555	no	no	yes	no	no	no	no	yes	yes	yes
182262014	chr1	248841369	248841566	66	198	3.000000	no	no	yes	no	no	no	no	yes	yes	yes
182262015	chr1	248844698	248844729	16	32	2.062500	no	no	yes	no	no	no	no	no	no	yes
182262017	chr1	248853796	248853868	24	73	3.000000	no	no	yes	no	no	no	no	yes	yes	yes
182262020	chr1	248861827	248861897	36	71	1.972222	no	no	yes	no	no	no	no	no	yes	yes
182262039	chr1	248890960	248890996	16	37	2.375000	no	no	no	no	no	no	no	yes	yes	no

182262044	chr1	248895360	248895390	16	31	1.937500	no	no	no	no	no	no	no	no	no	no
182262046	chr1	248904333	248904371	20	39	1.950000	no	no	no	no	no	no	no	yes	no	no
182262049	chr1	248918551	248918581	16	31	1.937500	no	yes	no	no	no	no	no	yes	no	yes
182262064	chr10	10733	10794	29	62	2.137931	no	no	no	no	no	no	no	no	no	no
182262087	chr10	70245	70288	19	44	2.315789	no	no	yes	no	no	no	no	no	no	yes
182262098	chr10	74696	74724	13	29	2.307692	yes	no	no	no	no	no	no	yes	no	no
182262106	chr10	87488	87525	18	38	2.111111	no	no	no	no	no	no	no	yes	yes	no
182262107	chr10	88805	89054	20	250	12.500000	no	no	no	no	no	no	no	yes	no	no
182262116	chr10	108499	108529	15	31	2.066667	no	no	no	no	no	no	no	no	no	no
182262134	chr10	148212	148468	28	257	9.178572	no	no	yes	no	yes	no	no	yes	yes	yes
182262136	chr10	154719	154755	18	37	2.055556	no	no	yes	no	no	yes	no	yes	no	yes
182262147	chr10	174454	175057	50	604	12.080000	no	no	yes	no	no	no	no	yes	no	yes
182262153	chr10	196934	197327	78	394	5.051282	yes	no	yes	no	no	no	no	no	yes	yes
182262161	chr10	206041	206077	16	37	2.375000	no	no	yes	no	no	no	no	yes	no	yes
182262163	chr10	215185	215433	123	249	2.024390	no	no	yes	no	no	no	no	yes	no	yes
182262166	chr10	259168	259205	20	38	1.900000	no	no	no	no	no	no	no	no	no	no
182262167	chr10	264277	265325	59	1049	17.847458	no	no	no	no	no	no	no	yes	yes	no
182262170	chr10	272009	272041	13	33	2.571429	no	no	no	no	no	no	no	yes	no	no
182262171	chr10	277181	277207	12	27	2.250000	no	yes	no	no	no	yes	no	yes	no	yes
182262173	chr10	277994	278297	78	304	3.915663	no	no	yes	no	no	no	no	yes	yes	yes
182262174	chr10	279362	279407	22	46	2.090909	no	no	yes	no	no	no	no	yes	no	yes
182262175	chr10	286504	286540	14	37	2.642857	no	no	yes	no	no	no	no	no	no	yes
182262176	chr10	294310	294348	17	39	2.294118	no	no	yes	no	no	no	no	no	no	yes
182262182	chr10	300535	300872	36	338	9.444445	no	no	yes	no	no	no	yes	yes	no	yes
182262184	chr10	301959	301999	19	41	2.157895	no	no	yes	no	no	no	no	no	no	yes

182262187	chr10	305242	305284	20	43	2.200000	no	no	yes	no	no	no	no	yes	no	yes
182262188	chr10	305296	305347	28	52	1.964286	no	no	yes	no	no	no	no	yes	no	yes
182262189	chr10	307774	308124	58	351	6.051724	no	no	yes	no	no	no	no	yes	no	yes
182262190	chr10	321532	323485	49	1954	39.843136	no	no	yes	no	no	no	yes	no	yes	yes
182262191	chr10	329627	329666	16	40	2.500000	yes	no	yes	no	no	no	no	yes	yes	yes
182262203	chr10	337928	338014	46	87	1.913043	no	no	yes	no	no	no	no	no	no	yes
182262205	chr10	338750	338947	41	198	4.562500	no	no	yes	no	no	no	no	yes	yes	yes
182262207	chr10	341340	341621	70	282	4.028572	no	no	yes	no	no	no	yes	no	yes	yes
182262212	chr10	347980	348054	33	75	2.272727	no	no	yes	no	no	no	yes	no	yes	yes
182262217	chr10	358597	358821	41	225	5.265306	no	no	yes	no	no	no	no	no	no	yes
182262218	chr10	361318	361415	48	98	2.062500	no	no	yes	no	no	no	no	yes	yes	yes
182262219	chr10	365821	365965	72	145	2.013889	no	no	yes	no	no	no	no	no	no	yes
182262220	chr10	367894	368294	58	401	6.913793	no	no	yes	no	no	no	yes	no	no	yes
182262224	chr10	371138	371213	18	76	4.157895	no	no	yes	no	no	no	no	no	no	yes
182262226	chr10	371625	371754	43	130	3.023256	no	no	yes	no	no	no	no	no	no	yes
182262227	chr10	372660	372842	35	183	5.228571	no	no	yes	no	no	no	no	yes	yes	yes
182262228	chr10	374101	374136	18	36	2.000000	no	no	yes	no	no	no	no	no	yes	yes
182262229	chr10	376231	376355	44	125	2.840909	no	no	yes	no	no	no	yes	no	yes	yes
182262236	chr10	379829	380504	34	676	19.882353	no	no	yes	no	no	no	no	yes	yes	yes
182262237	chr10	383926	384005	41	80	1.951220	no	no	yes	no	no	no	no	no	no	yes
182262239	chr10	384895	385217	52	323	6.211538	no	no	yes	no	no	no	no	yes	yes	yes
182262243	chr10	387435	387722	66	288	4.253334	no	no	yes	no	no	no	no	yes	no	yes
182262247	chr10	388953	389219	68	267	4.088235	no	no	yes	no	no	no	no	no	yes	yes
182262254	chr10	392698	392739	16	42	2.625000	no	no	yes	no	no	no	no	yes	no	yes
182262256	chr10	392879	392915	14	37	2.642857	no	no	yes	no	no	no	no	yes	no	yes

182262264	chr10	396810	396872	31	63	2.161290	no	no	yes	no	no	no	no	yes	yes	yes
182262265	chr10	400472	400764	81	293	3.617284	no	no	yes	no	no	no	no	no	no	yes
182262268	chr10	400764	404172	81	3409	42.160492	no	no	yes	no	no	no	no	yes	yes	yes
182262269	chr10	411580	411763	37	184	4.972973	no	no	yes	no	no	no	no	yes	yes	yes
182262270	chr10	411962	412018	22	57	2.565217	no	no	yes	no	no	no	no	yes	no	yes
182262271	chr10	413641	413928	96	288	3.000000	no	no	yes	yes	no	no	yes	yes	yes	yes
182262277	chr10	416309	416344	18	36	2.000000	no	no	yes	no	no	no	no	yes	yes	yes
182262278	chr10	417594	418363	38	770	20.710526	no	no	yes	no	no	no	no	yes	yes	yes
182262281	chr10	423467	423728	121	262	2.213115	no	no	yes	no	no	no	no	yes	no	yes
182262283	chr10	424903	425694	38	792	21.000000	no	no	yes	no	no	no	no	yes	yes	yes
182262286	chr10	429201	429569	22	369	17.043478	no	no	yes	no	no	yes	no	no	no	yes
182262287	chr10	436435	437487	59	1053	18.033333	yes	no	yes	no	no	no	no	yes	yes	yes
182262292	chr10	439220	439373	37	154	4.270270	no	no	yes	no	no	no	no	yes	no	yes
182262293	chr10	444041	444704	111	664	5.981982	no	no	yes	no	no	no	no	yes	yes	yes
182262296	chr10	445168	446440	214	1273	5.959276	no	no	yes	no	no	no	no	no	no	yes
182262297	chr10	447137	449233	50	2097	41.960785	no	no	yes	no	no	no	no	yes	yes	yes
182262299	chr10	450117	450401	145	285	1.965517	no	no	yes	no	no	no	no	yes	yes	yes
182262300	chr10	451966	452032	33	67	2.030303	no	no	yes	no	no	no	no	no	yes	yes
182262302	chr10	454346	454403	27	58	2.148148	no	no	yes	no	no	no	no	no	no	yes
182262306	chr10	454404	454872	196	469	2.441624	no	no	yes	no	no	no	no	yes	no	yes
182262307	chr10	455245	456483	57	1239	21.758621	no	no	yes	no	no	no	no	yes	yes	yes
182262309	chr10	458434	458851	70	418	6.057143	no	no	yes	no	no	no	no	yes	yes	yes
182262313	chr10	468984	469214	63	231	3.692308	no	no	yes	no	no	no	no	no	no	yes
182262315	chr10	471390	471462	38	73	1.947368	no	no	yes	no	no	no	no	no	no	yes
182262316	chr10	473347	473689	56	343	6.303571	no	no	yes	no	no	no	no	yes	no	yes

182262333	chr10	478408	478846	61	439	7.322581	no	no	yes	no	no	no	no	yes	yes	yes
182262337	chr10	479801	480520	44	720	16.000000	no	no	yes	no	no	no	yes	no	yes	yes
182262341	chr10	482322	482467	78	146	1.897436	no	no	yes	no	no	no	no	yes	yes	yes
182262342	chr10	483686	483719	11	34	3.000000	no	no	yes	no	no	no	no	no	no	yes
182262344	chr10	489540	490229	49	690	14.040000	no	no	yes	no	no	no	no	yes	yes	yes
182262346	chr10	491904	492076	40	173	4.341464	no	no	yes	no	no	no	no	no	no	yes
182262348	chr10	494522	494556	17	35	2.117647	no	no	yes	no	no	no	no	yes	yes	yes
182262349	chr10	494554	494679	63	126	2.000000	no	no	yes	no	no	no	no	yes	yes	yes
182262350	chr10	495699	496842	80	1144	14.511628	no	no	yes	no	no	no	no	yes	no	yes
182262353	chr10	499304	499347	23	44	1.913043	no	no	yes	no	no	no	no	no	yes	yes
182262355	chr10	502901	503267	94	367	3.904255	no	no	yes	no	no	no	no	no	no	yes
182262356	chr10	504522	504642	40	121	3.000000	no	no	yes	no	no	no	no	no	no	yes
182262358	chr10	505500	505862	108	363	3.379630	no	no	yes	no	no	no	no	yes	yes	yes
182262362	chr10	506990	507560	219	571	2.614350	no	no	yes	no	no	no	no	yes	yes	yes
182262364	chr10	509751	509865	60	115	1.933333	no	no	yes	no	no	no	no	no	yes	yes
182262366	chr10	513441	513565	66	125	1.893939	no	no	yes	no	no	no	no	yes	yes	yes
182262372	chr10	516350	516740	20	391	19.857143	no	no	yes	no	no	no	no	yes	yes	yes
182262376	chr10	516729	516854	63	126	2.047619	no	no	yes	no	no	no	no	yes	yes	yes
182262377	chr10	522817	523815	64	999	16.015625	no	no	yes	no	no	no	no	yes	no	yes
182262381	chr10	528279	528455	27	177	6.555555	no	no	yes	no	no	no	no	yes	no	yes
182262382	chr10	531204	531326	63	123	1.952381	no	no	yes	no	no	no	no	no	no	yes
182262383	chr10	532520	532793	18	274	15.444445	no	no	yes	no	no	no	no	yes	no	yes
182262385	chr10	537141	537262	61	122	2.000000	no	no	yes	no	no	no	no	no	no	yes
182262388	chr10	538940	539146	111	207	1.864865	no	no	yes	no	no	no	no	yes	yes	yes
182262389	chr10	540977	541152	23	176	7.695652	no	no	yes	no	no	no	no	no	yes	yes

182262391	chr10	541358	541843	226	486	2.176991	no	no	yes	no	no	no	no	yes	yes	yes
182262392	chr10	542594	542945	70	352	5.000000	no	no	yes	no	no	no	no	yes	no	yes
182262394	chr10	542998	543079	41	82	2.048780	no	no	yes	no	no	no	no	yes	no	yes
182262395	chr10	544003	544255	67	253	3.776119	no	no	yes	no	no	no	no	yes	yes	yes
182262397	chr10	547393	547532	58	140	2.413793	no	no	yes	no	no	no	no	yes	yes	yes
182262400	chr10	548443	548577	63	135	2.142857	no	no	yes	no	no	no	no	yes	yes	yes
182262403	chr10	549605	549685	39	81	2.076923	no	no	yes	no	no	no	no	no	yes	yes
182262404	chr10	550896	551136	81	241	2.963415	no	no	yes	no	no	no	no	no	yes	yes
182262405	chr10	553637	554292	60	656	11.000000	no	no	yes	no	no	no	no	yes	yes	yes
182262407	chr10	556042	556373	111	332	2.973684	no	no	yes	no	no	no	no	no	no	yes
182262409	chr10	557698	557737	21	40	1.904762	no	no	yes	no	no	no	no	no	no	yes
182262416	chr10	557773	557901	68	129	1.926471	no	no	yes	no	no	no	no	yes	yes	yes
182262419	chr10	559323	559350	11	28	2.545455	no	no	yes	no	no	no	no	no	yes	yes
182262420	chr10	559963	560008	23	46	2.043478	no	no	yes	no	no	no	no	no	no	yes
182262421	chr10	562615	562659	22	45	2.090909	no	no	yes	no	no	no	no	no	no	yes
182262423	chr10	566158	566274	59	117	1.983051	no	no	yes	no	no	no	no	yes	no	yes
182262426	chr10	577793	577836	19	44	2.315789	no	no	yes	no	no	no	no	no	no	yes
182262439	chr10	580252	580288	12	37	3.076923	no	no	yes	no	no	no	no	no	no	yes
182262442	chr10	584363	585090	143	728	5.006536	no	no	yes	no	no	no	yes	no	yes	yes
182262443	chr10	586897	587287	60	391	6.524590	no	no	yes	no	no	no	no	yes	yes	yes
182262444	chr10	587567	588298	46	732	15.913043	no	no	yes	no	no	no	no	yes	yes	yes
182262445	chr10	594349	594626	35	278	7.942857	no	no	yes	no	no	no	no	yes	yes	yes
182262447	chr10	597473	597501	15	29	1.933333	no	no	yes	no	no	no	no	no	no	yes
182262448	chr10	600425	600609	61	185	3.015873	no	no	yes	no	no	no	no	no	yes	yes
182262451	chr10	603886	603995	50	110	2.215686	no	no	yes	no	no	no	no	no	no	yes

182262452	chr10	605330	605364	18	35	2.000000	no	no	yes	no	no	no	no	no	no	yes
182262453	chr10	606329	606680	62	352	5.666667	no	no	yes	no	no	no	yes	no	no	yes
182262461	chr10	622257	622403	67	147	2.260870	no	no	yes	no	no	no	no	no	yes	yes
182262463	chr10	623171	623401	115	231	2.008696	no	no	yes	no	no	no	yes	yes	yes	yes
182262468	chr10	623453	623719	123	267	2.178862	no	no	yes	no	no	no	no	yes	yes	yes
182262469	chr10	623734	623811	28	78	2.785714	no	no	yes	no	no	no	no	no	yes	yes
182262470	chr10	626403	626499	26	97	3.703704	no	no	yes	no	no	no	no	yes	yes	yes
182262471	chr10	626697	626811	57	115	2.017544	no	no	yes	no	no	no	no	no	no	yes
182262472	chr10	628555	628635	38	81	2.131579	yes	no	yes	no	no	no	no	yes	yes	yes
182262473	chr10	629510	629744	77	235	3.051948	no	no	yes	no	no	no	no	yes	yes	yes
182262474	chr10	632298	633003	54	706	13.074074	no	no	yes	no	no	no	yes	yes	no	yes
182262475	chr10	633352	633484	33	133	4.030303	no	no	yes	no	no	no	yes	yes	yes	yes
182262479	chr10	639082	639518	196	437	2.234694	no	no	yes	no	no	no	yes	no	yes	yes
182262480	chr10	639968	640150	64	183	2.859375	no	no	yes	no	no	no	yes	no	yes	yes
182262481	chr10	640579	640792	18	214	11.888889	no	no	yes	no	no	yes	yes	no	no	yes
182262483	chr10	644505	644835	176	331	1.892045	no	no	yes	no	no	no	no	yes	no	yes
182262484	chr10	647000	649424	45	2425	54.066666	yes	no	yes	no	no	no	no	yes	no	yes
182262485	chr10	650309	650402	46	94	2.043478	no	yes	yes	yes	no	no	no	yes	no	yes
182262487	chr10	655383	655624	61	242	3.967213	no	no	yes	no	no	no	no	no	yes	yes
182262490	chr10	655740	656352	78	613	8.217949	no	no	yes	no	no	no	no	no	yes	yes
182262498	chr10	660097	660542	45	446	10.000000	no	no	yes	no	no	no	no	yes	yes	yes
182262499	chr10	666587	666652	15	66	4.400000	no	yes	yes	no	no	no	no	yes	no	yes
182262501	chr10	667533	667565	16	33	2.062500	no	yes	yes	no	no	no	no	yes	yes	yes
182262510	chr10	668018	668307	156	290	1.916667	no	yes	yes	no	no	no	no	yes	no	yes
182262517	chr10	668644	668678	13	35	2.692308	no	yes	yes	no	no	no	no	yes	no	yes

182262528	chr10	671255	672352	26	1098	40.935482	no	no	yes	no	no	no	yes	yes	yes	yes
182262535	chr10	679664	679785	49	122	2.489796	no	no	yes	no	no	no	no	yes	yes	yes
182262537	chr10	680854	681715	58	862	15.017241	no	no	yes	no	no	no	no	yes	yes	yes
182262548	chr10	689128	689334	108	207	1.935185	no	no	yes	no	no	no	yes	yes	yes	yes
182262549	chr10	689671	689717	23	47	2.130435	yes	no	no	no	no	no	yes	yes	yes	no
182262558	chr10	695826	696161	151	336	2.238411	no	no	no	no	no	no	no	no	yes	no
182262562	chr10	696469	697045	90	577	6.500000	no	no	no	no	no	no	no	yes	yes	no
182262566	chr10	697673	697783	58	111	1.913793	no	no	no	no	no	no	no	yes	yes	no
182262568	chr10	700160	700748	130	589	4.535211	no	no	no	no	no	no	no	yes	no	no
182262575	chr10	715323	715374	25	52	2.080000	no	no	no	no	no	no	no	no	no	no
182262579	chr10	717731	718501	70	771	11.042857	no	no	no	no	no	no	no	no	yes	no
182262580	chr10	723183	724196	78	1014	12.987342	no	no	no	no	no	no	no	yes	yes	no
182262581	chr10	725691	725736	23	46	2.000000	no	no	no	no	no	no	no	no	yes	no
182262582	chr10	726344	726929	46	586	12.638298	no	no	no	no	no	no	yes	no	yes	no
182262587	chr10	728775	729061	125	287	2.296000	no	no	no	no	no	no	no	no	no	no
182262593	chr10	730958	731274	46	317	6.913043	no	no	no	no	no	no	no	no	no	no
182262596	chr10	735395	738791	23	3397	145.222229	no	no	no	no	no	no	no	no	no	no
182262601	chr10	739516	739582	34	67	1.970588	no	no	no	no	no	no	no	yes	no	no
182262603	chr10	744073	744521	18	449	24.944445	no	no	yes	no	no	no	no	no	no	yes
182262607	chr10	751913	752655	57	743	13.017241	no	no	no	no	no	no	no	yes	no	no
182262608	chr10	756539	756575	14	37	2.642857	no	no	no	no	no	no	no	no	no	no
182262609	chr10	757032	757636	29	605	20.896551	no	no	no	no	no	no	no	no	no	no
182262613	chr10	766080	766921	40	842	21.049999	no	no	no	no	no	no	no	no	no	no
182262616	chr10	770237	770513	95	277	2.926316	no	no	no	no	no	no	no	no	no	no
182262617	chr10	773830	773932	22	103	4.681818	no	no	no	no	no	no	no	no	no	no

182262618	chr10	774251	774334	42	84	2.000000	no	no	no	no	no	no	no	no	no	no
182262620	chr10	777248	777986	49	739	15.000000	no	no	no	no	no	no	no	no	no	no
182262622	chr10	779420	779839	35	420	12.000000	no	no	no	no	no	no	no	yes	no	no
182262625	chr10	779754	779932	89	179	2.011236	no	no	no	no	no	no	no	no	no	no
182262627	chr10	780922	780964	14	43	3.071429	no	no	no	no	no	no	no	no	no	no
182262628	chr10	783732	784524	61	793	13.000000	no	no	no	no	no	no	no	yes	yes	no
182262629	chr10	786536	786566	14	31	2.214286	no	no	no	no	no	no	no	no	no	no
182262631	chr10	790292	790385	44	94	2.136364	no	no	no	no	no	no	no	yes	yes	no
182262632	chr10	791353	791385	13	33	2.615385	no	no	no	no	no	no	no	yes	yes	no
182262635	chr10	791764	791801	20	38	1.900000	no	no	no	no	no	no	no	yes	yes	no
182262639	chr10	791808	791961	38	154	3.952381	no	no	no	no	no	no	no	yes	yes	no
182262642	chr10	793649	794317	16	669	41.812500	no	no	no	no	no	no	yes	no	no	no
182262645	chr10	805498	805535	14	38	2.714286	no	no	no	no	no	no	yes	yes	yes	no
182262648	chr10	814104	814145	16	42	2.687500	no	no	yes	no	no	yes	no	no	no	yes
182262650	chr10	819941	820168	85	228	2.666667	no	no	yes	no	yes	no	no	no	no	yes
182262652	chr10	823173	823202	12	30	2.500000	no	no	yes	no	no	no	no	yes	yes	yes
182262660	chr10	842587	842630	23	44	2.000000	no	no	yes	no	no	no	no	no	no	yes
182262667	chr10	871945	872010	33	66	2.000000	no	no	yes	no	no	no	no	yes	yes	yes
182262669	chr10	880759	880787	14	29	2.071429	no	no	yes	no	no	no	no	no	no	yes
182262677	chr10	896026	896063	18	38	2.111111	no	no	yes	no	no	no	no	no	yes	yes
182262682	chr10	900025	900062	20	38	1.950000	no	no	yes	no	no	no	no	no	no	yes
182262689	chr10	905661	905725	19	65	3.421053	no	no	yes	no	no	no	no	yes	yes	yes
182262694	chr10	929213	929254	14	42	3.000000	no	no	yes	no	no	no	no	yes	no	yes
182262699	chr10	931104	931134	16	31	1.937500	no	no	yes	no	no	no	no	yes	yes	yes
182262708	chr10	932209	932249	21	41	2.000000	yes	no	no	no	no	no	yes	yes	yes	no

182262716 chr10 936240 936282 20 43 2.200000 no no yes no no no
no yes yes yes
182262725 chr10 943745 943790 15 46 3.000000 no no no no no no no
no no no
182262734 chr10 952360 953703 52 1344 25.846153 no no no no no no
no no no no
182262735 chr10 954220 954317 31 98 3.161290 no no no no no no no
no no no
182262736 chr10 954750 955132 26 383 14.730769 no no no no no no
no yes no no
182262737 chr10 955877 955945 30 69 2.281250 no no no no no no no
yes yes no
182262738 chr10 959327 959970 16 644 39.000000 no no no no no no
yes yes no no
182262747 chr10 975671 977012 41 1342 33.146343 no no yes no no no
yes yes no yes
182262755 chr10 991294 991329 17 36 2.117647 no no yes no no no
no yes no yes
182262757 chr10 997354 997449 50 96 1.940000 no no yes no no yes
no yes yes yes
182262760 chr10 1006643 1006723 42 81 1.928571 no no yes no no no
no yes no yes
182262761 chr10 1008740 1008849 54 110 2.037037 no no yes no no no
no yes no yes
182262762 chr10 1009271 1009402 55 132 2.436364 no no yes no no no
no yes yes yes
182262763 chr10 1010038 1010274 78 237 3.038461 no no yes no no no
no yes yes yes
182262765 chr10 1010856 1011243 190 388 2.062827 no no yes no no yes
no yes yes yes
182262766 chr10 1011386 1011571 59 186 3.062500 yes no yes no no yes
no yes no yes
182262767 chr10 1011671 1011700 15 30 2.000000 yes no yes no no no
no yes no yes
182262769 chr10 1014971 1015001 14 31 2.214286 no no yes no no no
no no no yes
182262770 chr10 1015347 1015731 33 385 11.325000 no no yes no no no
no no no yes
182262773 chr10 1016758 1016958 40 201 5.025000 no no yes no no no
no yes no yes
182262776 chr10 1020563 1020646 36 84 2.361111 no no yes no no yes
no yes yes yes
182262781 chr10 1030470 1030516 23 47 2.043478 no no yes no no no
no no no yes
182262782 chr10 1030494 1030668 57 175 3.033898 no no yes no no no
no no no yes
182262783 chr10 1031576 1031617 16 42 2.647059 no no yes no no no
no no no yes
182262789 chr10 1035852 1035880 9 29 3.222222 yes yes yes no no no
no yes no yes

182262790	chr10	1038636	1039267	63	632	10.047619	no	yes	yes	no	no	yes	no	yes	yes	yes
182262792	chr10	1042859	1042991	44	133	3.022727	no	no	yes	no	no	no	no	yes	no	yes
182262795	chr10	1046462	1047563	30	1102	36.292683	no	no	yes	no	no	no	no	yes	yes	yes
182262798	chr10	1056729	1056753	13	25	1.923077	yes	no	yes	no	yes	no	yes	yes	yes	yes
182262801	chr10	1070461	1070496	12	36	2.923077	no	no	yes	no	no	no	no	no	no	yes
182262803	chr10	1074512	1074612	52	101	1.942308	no	no	yes	no	no	no	no	yes	yes	yes
182262804	chr10	1075648	1075680	16	33	2.062500	no	no	yes	no	no	no	no	no	no	yes
182262806	chr10	1082759	1082803	14	45	3.214286	no	no	yes	no	no	no	no	no	no	yes
182262807	chr10	1082895	1083056	55	162	2.945455	no	no	yes	no	no	no	no	no	no	yes
182262809	chr10	1086757	1086815	18	59	3.277778	no	no	yes	no	no	no	no	no	no	yes
182262816	chr10	1094716	1095443	43	728	17.209303	no	no	yes	no	no	no	no	yes	no	yes
182262819	chr10	1099674	1100014	67	341	5.026667	no	no	yes	no	no	no	no	yes	no	yes
182262822	chr10	1101942	1102340	33	399	12.272727	no	no	yes	no	no	no	yes	yes	yes	yes
182262825	chr10	1107490	1108035	89	546	5.990000	no	no	yes	no	no	no	no	yes	yes	yes
182262831	chr10	1117846	1118604	58	759	13.155172	no	no	yes	no	no	no	no	yes	yes	yes
182262832	chr10	1121527	1121571	18	45	2.500000	no	no	yes	no	no	no	no	no	yes	yes
182262833	chr10	1123771	1123917	73	147	2.041096	yes	yes	yes	no	no	no	no	no	no	yes
182262835	chr10	1127654	1127745	48	92	1.916667	no	no	yes	no	no	no	no	yes	yes	yes
182262836	chr10	1128104	1128172	34	69	2.058824	no	no	yes	no	no	no	no	yes	no	yes
182262839	chr10	1128937	1128971	17	35	2.058824	no	no	yes	no	no	no	no	yes	yes	yes
182262848	chr10	1141815	1142043	70	229	3.253521	no	no	no	no	no	no	no	no	no	no
182262851	chr10	1149732	1149831	33	100	3.030303	no	no	no	no	no	no	no	yes	no	no
182262854	chr10	1150441	1150497	30	57	1.900000	no	no	no	no	no	no	no	yes	no	no
182262857	chr10	1152737	1152985	83	249	3.011765	no	no	no	no	no	no	no	yes	yes	no
182262866	chr10	1158402	1158452	20	51	2.650000	no	no	no	no	no	no	no	yes	no	no

182262869	chr10	1160670	1160900	57	231	4.070176	no	yes	yes	no	no	no	no	yes	no	yes
182262870	chr10	1163391	1163561	42	171	4.071429	no	yes	yes	no	no	no	yes	yes	no	yes
182262875	chr10	1164576	1165062	121	487	4.056000	no	yes	yes	no	no	yes	no	yes	no	yes
182262881	chr10	1165888	1166023	68	136	2.000000	no	no	yes	no	no	yes	no	yes	no	yes
182262884	chr10	1169766	1169857	33	92	2.787879	no	no	yes	no	no	no	no	no	no	yes
182262885	chr10	1170960	1170987	14	28	2.000000	no	no	yes	no	no	no	no	yes	no	yes
182262888	chr10	1171354	1171382	13	29	2.230769	no	no	yes	no	no	no	no	yes	yes	yes
182262891	chr10	1174053	1174342	135	290	2.148148	no	no	yes	no	no	no	no	no	yes	yes
182262893	chr10	1175966	1176206	79	241	3.050633	no	no	yes	no	no	no	no	no	no	yes
182262894	chr10	1179718	1179786	35	69	1.971429	no	yes	yes	no	no	no	no	no	no	yes
182262895	chr10	1179879	1179998	53	120	2.264151	no	yes	yes	no	no	no	no	no	no	yes
182262897	chr10	1180161	1180509	129	349	2.713178	no	yes	yes	no	no	no	no	no	yes	yes
182262898	chr10	1182339	1182529	41	191	4.658536	no	yes	yes	no	no	yes	no	yes	no	yes
182262899	chr10	1187330	1187430	35	101	2.885714	yes	no	yes	no	no	no	no	no	no	yes
182262900	chr10	1189714	1189961	27	248	9.185185	no	no	yes	no	no	no	no	yes	no	yes
182262901	chr10	1190059	1190214	80	156	1.975000	no	no	yes	no	no	no	no	yes	no	yes
182262902	chr10	1190969	1191160	38	192	5.052631	no	no	yes	no	no	no	no	no	no	yes
182262907	chr10	1195600	1195729	65	130	2.000000	no	no	yes	no	no	no	no	yes	no	yes
182262908	chr10	1197380	1197428	24	49	2.041667	no	no	yes	no	no	no	no	yes	no	yes
182262911	chr10	1201552	1201940	69	389	5.628572	no	yes	yes	no	yes	no	no	no	no	yes
182262912	chr10	1205333	1205478	36	146	4.055555	no	no	yes	no	no	no	no	yes	no	yes
182262913	chr10	1205851	1206023	31	173	5.575758	no	no	yes	no	no	no	no	yes	no	yes
182262917	chr10	1206333	1206496	57	164	2.862069	no	no	yes	no	no	no	yes	no	yes	yes
182262919	chr10	1206440	1206566	40	127	3.200000	no	no	yes	no	no	no	yes	no	yes	yes
182262924	chr10	1209355	1209471	60	117	1.950000	yes	no	yes	no	no	no	no	yes	yes	yes

182262929	chr10	1210764	1210788	12	25	2.083333	no	no	yes	no	no	no	no	yes	no	yes
182262937	chr10	1212591	1212907	44	317	7.200000	no	no	yes	no	no	no	no	yes	yes	yes
182262945	chr10	1213152	1213273	67	122	1.910448	no	no	yes	no	no	no	no	yes	no	yes
182262951	chr10	1213914	1214240	127	327	2.637795	no	no	yes	no	no	no	no	no	yes	yes
182262955	chr10	1214099	1214581	208	483	2.403846	no	no	yes	no	no	no	no	no	no	yes
182262958	chr10	1215852	1216054	60	203	3.416667	no	no	yes	no	yes	no	no	no	yes	yes
182262968	chr10	1231306	1231378	37	73	1.972973	no	no	yes	no	no	no	no	no	no	yes
182262969	chr10	1231723	1231795	38	73	1.921053	no	no	yes	no	no	no	no	no	no	yes
182262972	chr10	1232130	1232218	29	89	3.000000	no	no	yes	no	no	no	no	yes	no	yes
182262974	chr10	1232345	1232381	11	37	3.363636	no	no	yes	no	no	no	no	yes	yes	yes
182262978	chr10	1232785	1232849	34	65	1.941176	no	no	yes	no	no	no	no	yes	yes	yes
182262983	chr10	1234978	1236433	760	1456	1.917105	no	no	yes	no	no	no	no	no	no	yes
182262986	chr10	1242732	1242813	42	82	1.952381	no	no	yes	no	no	no	no	no	yes	yes
182262987	chr10	1248147	1248506	129	360	2.790698	no	no	yes	no	no	no	no	yes	yes	yes
182262991	chr10	1253945	1254046	51	102	2.039216	no	no	yes	no	no	no	no	yes	no	yes
182262997	chr10	1263810	1263853	23	44	1.913043	no	no	yes	no	no	no	no	no	no	yes
182262998	chr10	1265525	1266144	68	620	9.147058	no	no	yes	no	no	no	no	yes	yes	yes
182263000	chr10	1280543	1280797	23	255	11.086957	no	no	yes	no	no	no	no	yes	yes	yes
182263002	chr10	1292566	1292614	24	49	2.041667	no	no	yes	no	yes	no	no	no	no	yes
182263008	chr10	1293337	1293381	18	45	2.555556	yes	no	yes	no	no	no	no	yes	yes	yes
182263009	chr10	1297230	1297298	34	69	2.029412	no	no	yes	no	no	no	no	no	yes	yes
182263013	chr10	1310371	1310445	24	75	3.125000	no	no	yes	no	no	no	no	yes	yes	yes
182263017	chr10	1327733	1327771	15	39	2.733333	no	no	yes	no	no	no	no	no	yes	yes
182263018	chr10	1327968	1327997	13	30	2.307692	no	no	yes	no	no	no	no	no	yes	yes
182263030	chr10	1340064	1341905	42	1842	43.857143	no	no	yes	no	no	no	yes	yes	no	yes

182263032 chr10 1345016 1345147 44 132 3.000000 no no yes no no no
no no yes yes
182263038 chr10 1348959 1348992 14 34 2.428571 no no yes no no no
no no no yes
182263041 chr10 1355938 1355966 15 29 1.933333 no no yes no no no
no no no yes
182263045 chr10 1368815 1368964 58 150 2.724138 no no yes no no no
no yes yes yes
182263050 chr10 1373750 1374011 33 262 7.939394 no no yes no no no
yes no no yes
182263065 chr10 1377244 1377513 102 270 2.730769 no no yes no no no
no yes yes yes
182263072 chr10 1385114 1385153 21 40 1.904762 no no yes no no no
no no no yes
182263074 chr10 1395663 1395705 18 43 2.388889 no no yes no no no
no no yes yes
182263075 chr10 1396564 1398283 41 1720 41.951218 no no yes no no no
yes yes no yes
182263077 chr10 1405734 1406088 60 355 5.983333 no no yes no no no
no no no yes
182263078 chr10 1409179 1409296 37 118 3.216216 no no yes no no no
no no no yes
182263085 chr10 1409545 1409762 109 218 2.009174 no no yes no no no
no no no yes
182263087 chr10 1409989 1410316 109 328 3.017699 no no yes no no no
no no no yes
182263097 chr10 1417179 1417258 23 80 3.478261 no no yes no no no
no no no yes
182263099 chr10 1423569 1424209 20 641 32.049999 no no yes no no no
no yes yes yes
182263101 chr10 1428847 1428903 18 57 3.166667 no no yes no no no
no no yes yes
182263105 chr10 1432684 1432722 15 39 2.666667 no no yes no no no
no yes no yes
182263107 chr10 1434105 1434228 62 124 2.000000 no no yes no no no
no no no yes
182263108 chr10 1435402 1435563 83 162 1.963855 no no yes no no no
no no yes yes
182263112 chr10 1438147 1440227 92 2081 22.845362 no no yes no no no
no no no yes
182263119 chr10 1449998 1450060 33 63 1.909091 no no yes no no no
no yes no yes
182263123 chr10 1451679 1451787 21 109 5.238095 no no yes no no no
no yes yes yes
182263124 chr10 1458837 1458880 16 44 2.812500 no no yes no no no
no no no yes
182263130 chr10 1464078 1464866 66 789 11.954545 no no yes no no no
yes yes yes yes
182263132 chr10 1470071 1470126 26 56 2.153846 no no yes no no no
no no no yes

182263134	chr10	1472318	1472473	56	156	2.785714	no	no	yes	no	no	no	no	yes	yes	yes
182263135	chr10	1473920	1474070	76	151	1.986842	no	no	yes	no	no	no	no	no	yes	yes
182263137	chr10	1476130	1476172	23	43	1.913043	no	no	yes	no	no	no	no	yes	no	yes
182263139	chr10	1478562	1478938	30	377	12.633333	no	no	yes	no	no	no	no	no	no	yes
182263142	chr10	1485008	1485108	48	101	2.104167	no	no	yes	no	no	no	no	yes	no	yes
182263144	chr10	1485232	1485326	48	95	1.979167	no	no	yes	no	no	no	no	yes	no	yes
182263147	chr10	1486961	1486989	13	29	2.230769	no	no	yes	no	no	no	no	yes	no	yes
182263148	chr10	1489210	1489394	62	185	2.983871	no	no	yes	no	no	no	no	yes	yes	yes
182263150	chr10	1495681	1495726	24	46	1.916667	no	no	yes	no	no	no	no	yes	no	yes
182263165	chr10	1505606	1505797	49	192	3.918367	no	no	yes	no	no	no	no	yes	yes	yes
182263172	chr10	1516494	1516524	14	31	2.214286	no	no	yes	no	no	no	no	no	no	yes
182263174	chr10	1519767	1519863	35	97	2.771429	no	no	yes	no	no	no	no	no	no	yes
182263178	chr10	1525795	1525831	16	37	2.312500	yes	no	yes	no	no	no	no	yes	yes	yes
182263183	chr10	1526209	1526270	9	62	6.888889	yes	no	yes	no	no	no	no	yes	no	yes
182263184	chr10	1527610	1527647	17	38	2.235294	no	no	yes	no	no	no	no	no	no	yes
182263185	chr10	1529042	1529378	36	337	9.444445	no	no	yes	no	no	no	no	no	no	yes
182263189	chr10	1530829	1530982	49	154	3.142857	no	no	yes	no	no	no	no	no	no	yes
182263191	chr10	1532126	1532247	65	122	1.876923	no	no	yes	no	no	no	no	no	yes	yes
182263192	chr10	1540426	1542980	44	2555	58.955555	no	no	yes	no	no	no	yes	yes	no	yes
182263193	chr10	1543998	1544038	16	41	2.562500	no	no	yes	no	no	no	no	no	yes	yes
182263197	chr10	1547169	1547836	73	668	9.142858	no	no	yes	no	no	no	no	yes	no	yes
182263201	chr10	1549154	1549261	54	108	2.055556	no	no	yes	no	no	no	no	no	no	yes
182263204	chr10	1552500	1552738	128	239	1.867188	no	no	yes	no	no	no	no	no	yes	yes
182263208	chr10	1558189	1558838	59	650	11.000000	no	no	yes	no	no	no	no	no	yes	yes
182263210	chr10	1560425	1560585	38	161	4.236842	no	no	yes	no	no	no	no	yes	no	yes

182263215	chr10	1568532	1568584	20	53	2.666667	no	no	yes	no	no	no	no	no	no	yes
182263222	chr10	1575002	1575052	26	51	1.961538	no	no	yes	no	no	no	no	no	no	yes
182263225	chr10	1577134	1577447	78	314	4.012658	no	no	yes	no	no	no	no	yes	no	yes
182263226	chr10	1578694	1578732	20	39	1.950000	no	no	yes	no	no	no	no	no	yes	yes
182263227	chr10	1578743	1578782	22	40	1.909091	no	no	yes	no	no	no	no	no	yes	yes
182263232	chr10	1589416	1589547	60	132	2.216667	no	no	yes	no	no	no	no	no	yes	yes
182263233	chr10	1592231	1593427	63	1197	19.000000	no	no	yes	no	no	no	no	no	no	yes
182263237	chr10	1598519	1598585	35	67	1.942857	no	no	yes	no	no	no	no	no	no	yes
182263240	chr10	1602620	1602769	36	150	4.289474	no	no	yes	no	no	no	no	yes	no	yes
182263264	chr10	1610802	1610921	64	120	1.875000	no	no	yes	no	no	no	no	yes	no	yes
182263266	chr10	1616350	1617831	92	1482	15.990292	no	no	yes	no	no	no	no	no	yes	yes
182263273	chr10	1626016	1626453	63	438	6.952381	no	no	yes	no	no	no	no	yes	yes	yes
182263279	chr10	1632271	1632339	31	69	2.225806	no	no	yes	no	no	no	no	yes	no	yes
182263292	chr10	1639192	1639281	18	90	5.000000	no	no	yes	no	no	no	no	yes	yes	yes
182263298	chr10	1640636	1640686	20	51	2.550000	no	no	yes	no	no	no	no	no	no	yes
182263314	chr10	1649781	1649821	22	41	1.954545	no	no	yes	no	no	no	no	no	no	yes
182263315	chr10	1653486	1653670	37	185	5.000000	no	no	yes	no	no	no	no	no	no	yes
182263323	chr10	1659700	1660133	121	434	3.577236	no	no	yes	no	no	no	no	no	yes	yes
182263337	chr10	1669943	1669975	10	33	3.500000	no	no	yes	no	no	no	no	yes	yes	yes
182263338	chr10	1672003	1672143	49	141	2.877551	no	no	yes	no	no	no	no	no	no	yes
182263339	chr10	1672141	1672166	13	26	2.000000	no	no	yes	no	no	no	no	no	no	yes
182263344	chr10	1672531	1672723	105	193	1.847619	no	no	yes	no	no	no	no	yes	yes	yes
182263346	chr10	1674863	1675728	33	866	26.235294	no	no	yes	no	no	no	no	yes	no	yes
182263348	chr10	1680081	1680257	44	177	4.022727	no	no	yes	no	no	no	no	yes	no	yes
182263349	chr10	1684881	1685035	74	155	2.108108	no	no	yes	no	no	no	no	no	no	yes

182263355	chr10	1695666	1695724	30	59	2.000000	no	no	yes	no	no	no	no	yes	no	yes
182263357	chr10	1695862	1695910	18	49	2.722222	no	no	yes	no	no	no	no	yes	yes	yes
182263358	chr10	1696149	1696180	16	32	2.000000	no	no	yes	no	no	no	no	yes	no	yes
182263362	chr10	1699162	1701939	42	2778	65.978722	no	no	yes	no	no	no	yes	no	no	yes
182263363	chr10	1706884	1706955	23	72	3.173913	no	no	yes	no	no	no	no	yes	yes	yes
182263364	chr10	1711240	1711323	28	84	3.000000	no	no	yes	no	no	no	no	no	no	yes
182263366	chr10	1717005	1717102	16	98	6.375000	no	no	yes	no	no	no	no	yes	yes	yes
182263382	chr10	1752191	1752550	145	360	2.517241	no	no	no	no	no	no	no	yes	no	no
182263384	chr10	1756721	1756763	16	43	2.687500	no	no	no	no	no	no	no	no	no	no
182263387	chr10	1762601	1762639	18	39	2.222222	no	no	no	no	no	no	no	yes	no	no
182263390	chr10	1766008	1766421	71	414	5.776316	no	no	no	no	no	no	no	yes	yes	no
182263400	chr10	1780180	1780469	57	290	5.068965	no	no	no	no	no	no	no	no	no	no
182263404	chr10	1782507	1782631	62	125	2.016129	no	no	no	no	no	no	no	no	no	no
182263405	chr10	1785721	1785990	136	270	1.985294	no	no	no	no	no	no	no	yes	yes	no
182263410	chr10	1798181	1798220	21	40	1.904762	no	no	no	no	no	no	no	no	no	no
182263411	chr10	1800213	1800611	80	399	4.963415	no	no	no	no	no	no	no	no	no	no
182263413	chr10	1801111	1801254	75	144	1.920000	no	no	no	no	no	no	no	yes	no	no
182263415	chr10	1801323	1801389	34	67	1.970588	no	no	no	no	no	no	no	yes	no	no
182263416	chr10	1801472	1801508	20	37	1.950000	no	no	no	no	no	no	no	no	no	no
182263418	chr10	1807090	1807131	13	42	3.230769	no	no	no	no	no	no	no	no	no	no
182263419	chr10	1809268	1809367	50	100	2.000000	no	no	no	no	no	no	no	yes	yes	no
182263423	chr10	1820505	1820552	19	48	2.550000	no	no	no	no	no	no	no	no	no	no
182263425	chr10	1831267	1831391	62	125	2.016129	no	no	no	no	no	no	no	no	no	no
182263432	chr10	1834924	1834970	20	47	2.428571	no	no	no	no	no	no	no	no	no	no
182263436	chr10	1839306	1839533	107	228	2.130841	no	no	no	no	no	no	no	yes	no	no

182263442	chr10	1841837	1842069	108	233	2.175926	no	no	no	no	no	no	no	yes	no	no
182263444	chr10	1842331	1842380	26	50	1.923077	no	no	no	no	no	no	no	no	no	no
182263463	chr10	1870492	1870842	184	351	1.940217	no	no	no	no	no	no	no	no	no	no
182263467	chr10	1871658	1871978	166	321	1.963855	no	no	no	no	no	no	no	yes	no	no
182263470	chr10	1873571	1873623	27	53	1.962963	no	no	no	no	no	no	no	no	no	no
182263472	chr10	1890197	1890227	15	31	2.133333	no	no	no	no	no	no	no	no	no	no
182263475	chr10	1896363	1896402	19	40	2.105263	no	no	no	no	no	no	no	yes	yes	no
182263481	chr10	1900632	1900672	21	41	1.952381	no	no	no	no	no	no	no	no	no	no
182263482	chr10	1900975	1901039	34	65	1.970588	no	no	no	no	no	no	no	no	no	no
182263488	chr10	1906166	1906542	93	377	3.855856	no	no	no	no	no	no	no	no	no	no
182263490	chr10	1907617	1907667	22	51	2.363636	no	no	no	no	no	no	no	no	no	no
182263491	chr10	1909402	1909451	11	50	4.636364	no	no	no	no	no	no	no	no	no	no
182263493	chr10	1912109	1912148	21	40	1.904762	no	no	no	no	no	no	no	no	no	no
182263494	chr10	1913271	1913344	36	74	2.055556	no	no	no	no	no	no	no	no	no	no
182263498	chr10	1914976	1915022	20	47	2.333333	no	no	no	no	no	no	no	no	no	no
182263500	chr10	1915979	1916038	26	60	2.307692	no	no	no	no	no	no	no	no	no	no
182263501	chr10	1925273	1925312	17	40	2.352941	no	no	no	no	no	no	no	no	no	no
182263502	chr10	1926056	1926088	17	33	2.000000	no	no	no	no	no	no	no	no	no	no
182263509	chr10	1934161	1934229	34	69	2.029412	no	no	no	no	no	no	no	no	no	no
182263511	chr10	1936479	1936530	22	52	2.454545	no	no	no	no	no	no	no	no	no	no
182263514	chr10	1942561	1942828	67	268	4.000000	no	no	no	no	no	no	no	no	yes	no
182263515	chr10	1944463	1944830	53	368	7.037736	no	no	no	no	no	no	no	yes	no	no
182263518	chr10	1949233	1949325	46	93	2.043478	no	no	no	no	no	no	no	no	no	no
182263522	chr10	1962647	1962686	21	40	1.904762	no	no	no	no	no	no	no	no	no	no
182263527	chr10	1965323	1965383	32	61	1.906250	no	no	no	no	no	no	no	no	yes	no

182263529	chr10	1971468	1971780	61	313	5.131147	no	no	no	no	no	no	no	no	no	no
182263531	chr10	1979511	1979617	39	107	2.769231	no	no	no	no	no	no	no	no	no	no
182263533	chr10	1983142	1983205	28	64	2.285714	no	no	no	no	no	no	no	no	yes	no
182263534	chr10	1984238	1985039	88	802	9.076923	no	no	no	no	no	no	no	no	yes	no
182263535	chr10	1987590	1987687	48	98	2.041667	no	no	no	no	no	no	no	no	no	no
182263546	chr10	1991773	1991866	43	94	2.209302	no	no	yes	no	no	no	no	yes	no	yes
182263550	chr10	1993480	1993531	24	52	2.166667	no	no	yes	no	no	no	no	yes	no	yes
182263553	chr10	1995154	1995455	147	302	2.108844	no	no	yes	no	no	no	no	no	no	yes
182263556	chr10	1998577	1998640	33	64	2.030303	no	no	yes	no	no	no	no	no	no	yes
182263563	chr10	2013304	2013419	60	116	1.983333	no	no	no	no	yes	no	no	no	no	yes
182263564	chr10	2014554	2014595	21	42	2.000000	yes	no	no	no	yes	no	no	no	no	yes
182263572	chr10	2022456	2022613	70	158	2.253521	no	no	no	no	no	no	no	no	no	no
182263575	chr10	2024918	2025127	41	210	5.121951	no	no	no	no	no	no	no	no	yes	no
182263583	chr10	2048537	2049063	102	527	5.133333	no	no	no	no	no	no	no	yes	yes	no
182263588	chr10	2054667	2054697	16	31	1.937500	no	no	no	no	no	no	no	yes	no	no
182263589	chr10	2058678	2058729	21	52	2.523809	no	no	no	no	no	no	no	yes	no	no
182263596	chr10	2075006	2075167	27	162	6.000000	no	no	yes	no	no	yes	no	no	yes	yes
182263599	chr10	2083673	2083852	37	180	4.864865	no	no	no	no	no	no	no	no	yes	no
182263604	chr10	2085771	2085806	14	36	2.571429	no	no	no	no	no	no	no	yes	no	no
182263608	chr10	2089765	2089810	22	46	2.136364	no	no	no	no	no	no	no	no	no	no
182263609	chr10	2092260	2092458	59	199	3.372881	no	no	no	no	no	no	no	no	no	no
182263620	chr10	2101161	2101195	18	35	1.944444	no	no	no	no	no	no	no	no	yes	no
182263623	chr10	2115407	2115457	27	51	1.962963	no	no	no	no	no	no	no	no	no	no
182263627	chr10	2143243	2143436	39	194	4.974359	no	no	no	no	no	no	no	yes	no	no
182263640	chr10	2156500	2156662	85	163	1.941176	no	no	yes	no	no	no	no	no	no	yes

182263643	chr10	2166568	2166649	26	82	3.269231	no	no	yes	no	no	no	no	no	no	yes
182263645	chr10	2174764	2174788	13	25	1.923077	no	no	yes	no	no	no	no	no	no	yes
182263661	chr10	2203692	2203734	23	43	1.956522	no	no	no	no	no	no	no	no	no	no
182263670	chr10	2230422	2230452	16	31	1.937500	no	no	no	no	no	no	no	no	no	no
182263675	chr10	2231195	2231278	41	84	2.073171	no	no	no	no	no	no	no	no	no	no
182263678	chr10	2231726	2232643	56	918	16.392857	no	no	no	no	no	no	no	yes	yes	no
182263684	chr10	2236158	2236196	19	39	2.052632	no	no	no	no	no	no	no	no	no	no
182263695	chr10	2245708	2245756	22	49	2.227273	no	no	no	no	no	no	no	yes	no	no
182263696	chr10	2245746	2245783	18	38	2.111111	no	no	no	no	no	no	no	yes	no	no
182263700	chr10	2245864	2245925	32	62	2.000000	no	no	no	no	no	no	no	yes	no	no
182263703	chr10	2255801	2255846	16	46	3.125000	no	no	no	no	no	no	no	no	no	no
182263705	chr10	2258807	2258832	13	26	2.000000	no	no	no	no	no	no	no	no	no	no
182263708	chr10	2265111	2265135	11	25	2.272727	no	no	no	no	no	no	no	yes	no	no
182263710	chr10	2265143	2265204	22	62	2.909091	no	no	no	no	no	no	no	yes	no	no
182263721	chr10	2281444	2281486	16	43	2.647059	no	no	no	no	no	no	no	no	no	no
182263722	chr10	2282493	2282530	20	38	1.950000	no	no	no	no	no	no	no	yes	no	no
182263724	chr10	2284421	2284526	34	106	3.147059	no	no	no	no	no	no	no	no	no	no
182263725	chr10	2284812	2284844	17	33	1.941176	no	no	no	no	no	no	no	no	no	no
182263726	chr10	2290344	2291180	138	837	6.034965	no	no	no	no	no	no	no	no	no	no
182263728	chr10	2292963	2293006	22	44	2.000000	no	no	no	no	no	no	no	no	no	no
182263734	chr10	2315053	2315293	40	241	6.025000	yes	no	no	no	yes	no	yes	yes	yes	yes
182263735	chr10	2317502	2317581	40	80	2.000000	no	no	no	no	no	no	no	no	no	no
182263736	chr10	2317915	2318048	70	134	1.914286	no	no	no	no	no	no	no	no	no	no
182263737	chr10	2319267	2319305	20	39	1.950000	no	no	no	no	no	no	no	no	no	no
182263743	chr10	2328218	2328271	25	54	2.200000	no	no	no	no	no	no	no	yes	no	no

182263745	chr10	2328408	2328455	25	48	1.920000	no	no	no	no	no	no	no	yes	no	no
182263748	chr10	2328558	2328590	13	33	2.615385	no	no	no	no	no	no	no	yes	no	no
182263752	chr10	2340845	2340885	20	41	2.050000	no	no	no	no	no	no	no	no	no	no
182263756	chr10	2353807	2353845	21	39	1.904762	no	no	no	no	no	no	no	no	no	no
182263759	chr10	2354049	2354472	26	424	16.656250	no	no	no	no	no	no	no	no	no	no
182263763	chr10	2358004	2358112	54	109	2.018518	no	no	no	no	no	no	no	no	no	no
182263765	chr10	2359952	2360013	24	62	2.583333	no	no	no	no	no	no	no	yes	no	no
182263771	chr10	2367948	2368154	115	207	1.808696	no	no	no	no	no	yes	no	no	no	no
182263772	chr10	2371574	2372141	38	568	15.025641	no	no	yes	no	no	no	no	no	yes	yes
182263775	chr10	2373650	2373944	138	295	2.150000	no	no	yes	no	no	no	no	yes	no	yes
182263780	chr10	2374225	2374386	89	162	1.842697	no	no	yes	no	no	no	no	yes	no	yes
182263782	chr10	2374369	2374449	36	81	2.277778	no	no	yes	no	no	no	no	yes	no	yes
182263784	chr10	2374558	2374633	26	76	2.961539	no	no	yes	no	no	no	no	yes	no	yes
182263787	chr10	2388239	2388331	39	93	2.410256	no	no	yes	no	no	no	no	yes	no	yes
182263789	chr10	2392246	2392680	39	435	10.886364	no	no	yes	no	no	no	no	yes	yes	yes
182263792	chr10	2396980	2397035	28	56	2.000000	no	no	yes	no	no	no	no	no	no	yes
182263793	chr10	2400110	2400693	28	584	20.857143	no	no	yes	no	no	no	no	yes	no	yes
182263795	chr10	2401303	2401357	20	55	2.850000	no	no	yes	no	no	no	no	yes	no	yes
182263800	chr10	2404072	2404186	46	115	2.500000	no	no	yes	no	no	no	no	no	no	yes
182263802	chr10	2408216	2408249	16	34	2.125000	no	no	yes	no	no	no	no	no	no	yes
182263804	chr10	2410320	2410356	8	37	4.625000	no	no	yes	no	no	no	no	yes	no	yes
182263806	chr10	2412283	2412342	31	60	2.000000	no	no	yes	no	no	no	no	no	no	yes
182263811	chr10	2425355	2425615	130	261	2.007692	no	no	yes	no	no	no	no	no	no	yes
182263819	chr10	2430836	2430919	33	84	2.545455	no	no	yes	no	no	no	no	yes	no	yes
182263828	chr10	2436283	2436365	32	83	2.558824	no	no	yes	no	no	no	no	no	no	yes

182263830 chr10 2437780 2437818 18 39 2.222222 no no yes no no no
no no no yes
182263834 chr10 2454317 2454350 13 34 2.692308 no no yes no no no
no no no yes
182263838 chr10 2469930 2470000 35 71 2.028571 no no yes no no no
no no yes yes
182263841 chr10 2475588 2475623 16 36 2.312500 no no yes no no no
no no no yes
182263842 chr10 2477526 2477663 67 138 2.059701 no no yes no no no
no yes no yes
182263844 chr10 2481579 2481821 36 243 6.805555 no no yes no no no
no no no yes
182263845 chr10 2482065 2482105 22 41 1.909091 no no yes no no no
no no no yes
182263862 chr10 2501302 2501932 63 631 10.015873 yes yes yes no yes yes
yes no yes yes
182263863 chr10 2503537 2503803 39 267 6.850000 no no yes no no no
no yes no yes
182263869 chr10 2507831 2507969 56 139 2.482143 no no yes no no no
no no yes yes
182263870 chr10 2508007 2508046 12 40 3.333333 no no yes no no no
no no no yes
182263877 chr10 2511512 2511561 26 50 1.923077 no no yes no no no
no no no yes
182263880 chr10 2512778 2512855 41 78 1.902439 no no yes no no no
no no no yes
182263885 chr10 2513262 2513543 129 282 2.181818 no no yes no no no
no yes yes yes
182263891 chr10 2516983 2517042 20 60 3.000000 no no yes no no no
no no no yes
182263894 chr10 2518945 2518976 9 32 3.555556 no no yes no no no
no no no yes
182263896 chr10 2521069 2523611 28 2543 93.885712 no no yes no no no
yes yes yes yes
182263899 chr10 2536314 2536538 40 225 5.625000 no no yes no no no
no yes yes yes
182263916 chr10 2567081 2567125 20 45 2.250000 no yes yes no no no
no no no yes
182263920 chr10 2572002 2572054 24 53 2.208333 no no yes no no yes
no no no yes
182263923 chr10 2578820 2578879 26 60 2.307692 no no yes no no no
no yes yes yes
182263924 chr10 2579785 2579832 21 48 2.380952 no no yes no no no
no no no yes
182263928 chr10 2583357 2584363 40 1007 25.270834 no no yes no no no
no no no yes
182263934 chr10 2592406 2592483 38 78 2.052632 no no yes no no no
no yes no yes
182263935 chr10 2599224 2599263 18 40 2.222222 no no yes no no no
no no no yes

182263946	chr10	2610724	2610763	13	40	3.000000	no	no	yes	no	no	no	no	yes	no	yes
182263953	chr10	2626218	2626344	42	127	3.023256	no	no	no	no	no	no	no	no	no	no
182263955	chr10	2633809	2633852	20	44	2.200000	no	no	no	no	no	no	no	yes	yes	no
182263957	chr10	2636253	2636296	23	44	1.913043	no	no	no	no	no	no	no	no	no	no
182263962	chr10	2647546	2647626	20	81	4.050000	no	no	no	no	no	no	no	no	no	no
182263965	chr10	2649651	2649700	24	50	2.083333	no	no	no	no	no	no	no	no	no	no
182263966	chr10	2653434	2653479	23	46	2.043478	no	no	no	no	no	no	no	yes	no	no
182263967	chr10	2656394	2656782	49	389	8.102041	no	no	no	no	no	no	no	no	no	no
182263972	chr10	2661220	2661258	19	39	2.105263	no	no	no	no	no	no	no	no	no	no
182263973	chr10	2661730	2661762	15	33	2.200000	no	no	no	no	no	no	no	no	no	no
182263976	chr10	2667011	2667246	80	236	3.000000	no	no	no	no	no	no	no	no	no	no
182263977	chr10	2668941	2668965	12	25	2.083333	no	no	no	no	no	no	no	no	no	no
182263980	chr10	2669713	2669855	71	143	2.042253	no	no	no	no	no	no	no	yes	no	no
182263982	chr10	2683643	2683680	14	38	2.714286	no	no	no	no	no	no	no	no	no	no
182263993	chr10	2694904	2694930	13	27	2.076923	no	no	no	no	no	no	no	no	no	no
182263995	chr10	2706096	2706137	16	42	2.625000	no	no	no	no	no	no	no	no	no	no
182264005	chr10	2731192	2731233	23	42	1.956522	no	no	no	no	no	no	no	no	no	no
182264008	chr10	2736998	2737031	15	34	2.266667	no	no	no	no	no	no	no	no	no	no
182264015	chr10	2747215	2747294	40	80	2.000000	no	no	no	no	no	no	no	yes	yes	no
182264025	chr10	2755967	2756097	66	131	2.015152	no	no	no	no	no	no	no	no	no	no
182264030	chr10	2756771	2756819	26	49	1.961538	no	no	no	no	no	no	no	no	no	no
182264033	chr10	2767190	2767248	30	59	1.966667	no	no	no	no	no	no	no	yes	no	no
182264039	chr10	2780579	2780689	56	111	2.000000	no	no	no	no	no	no	no	no	no	no
182264041	chr10	2783399	2783494	22	96	4.363636	no	no	no	no	no	no	no	yes	no	no
182264042	chr10	2789434	2789463	14	30	2.142857	no	no	no	no	no	no	no	no	no	no

182264056	chr10	2815884	2815930	23	47	2.043478	no	no	no	no	no	no	no	no	no	no
182264058	chr10	2825265	2825301	12	37	3.076923	no	no	no	no	no	no	no	no	no	no
182264066	chr10	2829847	2830500	292	654	2.245734	no	no	no	no	no	no	no	no	no	no
182264069	chr10	2845812	2845842	16	31	1.937500	no	no	no	no	no	no	no	no	no	no
182264072	chr10	2849791	2849817	14	27	1.928571	no	no	no	no	no	no	no	no	no	no
182264077	chr10	2865642	2866045	31	404	13.032258	no	no	no	no	no	no	no	yes	no	no
182264078	chr10	2866033	2866067	11	35	3.181818	no	no	no	no	no	no	no	no	no	no
182264083	chr10	2871653	2872127	203	475	2.389163	no	no	no	no	no	no	no	yes	yes	no
182264093	chr10	2875781	2875821	21	41	1.952381	no	no	no	no	no	no	no	yes	no	no
182264095	chr10	2884685	2884717	13	33	2.538461	no	no	no	no	no	no	no	no	no	no
182264099	chr10	2888241	2888921	273	681	2.539568	no	no	no	no	no	no	no	yes	yes	no
182264106	chr10	2902385	2902450	32	66	2.062500	no	no	no	no	no	no	no	no	no	no
182264109	chr10	2906832	2907313	204	482	2.431373	no	no	no	no	no	no	no	yes	yes	no
182264111	chr10	2913199	2913315	40	117	2.925000	no	no	no	no	no	no	no	no	yes	no
182264112	chr10	2915188	2915253	8	66	8.250000	no	no	no	no	no	no	no	no	no	no
182264113	chr10	2915749	2915820	19	72	3.789474	no	no	no	no	no	no	no	yes	yes	no
182264116	chr10	2924571	2924665	45	95	2.111111	no	no	no	no	no	no	no	no	yes	no
182264119	chr10	2927308	2927410	54	103	1.925926	no	no	no	no	no	no	no	yes	yes	no
182264125	chr10	2934066	2934139	37	74	2.000000	no	no	no	no	no	no	no	yes	yes	no
182264135	chr10	2960890	2960940	26	51	1.961538	no	no	no	no	no	no	no	no	no	no
182264136	chr10	2963299	2963346	12	48	4.000000	no	no	no	no	no	no	no	yes	no	no
182264144	chr10	2970479	2970552	33	74	2.242424	no	no	no	no	no	no	no	yes	yes	no
182264146	chr10	2970595	2970656	18	62	3.500000	no	no	no	no	no	no	no	yes	no	no
182264150	chr10	2971698	2971750	17	53	3.117647	no	no	no	no	no	no	no	no	no	no
182264154	chr10	2977749	2977783	18	35	1.944444	no	no	no	no	no	no	no	no	no	no

182264163	chr10	2983996	2984095	22	100	4.681818	no	no	no	no	no	no	no	no	no	no
182264167	chr10	2987185	2987220	15	36	2.533333	no	no	no	no	no	no	no	yes	no	no
182264178	chr10	2988805	2988912	46	108	2.416667	no	no	no	no	no	no	no	no	no	no
182264187	chr10	3001794	3002360	185	567	3.040609	no	no	no	no	no	no	no	no	no	no
182264190	chr10	3018131	3018155	13	25	1.923077	no	no	no	no	no	no	no	no	no	no
182264204	chr10	3046465	3046660	38	196	5.153846	no	no	yes	no	no	no	no	yes	yes	yes
182264207	chr10	3048492	3048677	37	186	5.027027	no	no	no	no	no	no	no	yes	yes	no
182264208	chr10	3050289	3050325	18	37	2.111111	no	no	no	no	no	no	no	yes	yes	no
182264211	chr10	3053485	3053815	18	331	18.388889	no	no	no	no	no	no	no	yes	no	no
182264225	chr10	3073263	3073299	19	37	1.947368	no	no	yes	no	no	no	no	yes	yes	yes
182264230	chr10	3078212	3078254	22	43	2.000000	no	no	yes	no	no	no	no	yes	no	yes
182264231	chr10	3079913	3079995	38	83	2.184211	no	no	yes	no	no	no	no	yes	no	yes
182264234	chr10	3081738	3081828	47	91	2.000000	no	no	yes	no	no	no	no	yes	yes	yes
182264236	chr10	3084687	3085847	24	1161	47.799999	no	no	yes	no	no	no	no	no	yes	yes
182264241	chr10	3092930	3093072	49	143	2.959184	no	no	yes	no	no	no	no	yes	yes	yes
182264246	chr10	3106231	3106497	132	267	2.060150	no	no	yes	no	no	yes	yes	yes	no	yes
182264249	chr10	3106579	3107006	218	428	2.045871	no	no	yes	no	no	no	no	no	no	yes
182264250	chr10	3107786	3107812	10	27	2.700000	no	no	yes	no	no	yes	no	yes	yes	yes
182264255	chr10	3110834	3110902	36	69	1.916667	no	no	yes	no	no	no	no	yes	yes	yes
182264263	chr10	3115210	3115608	47	399	8.489362	no	no	yes	no	no	no	no	no	no	yes
182264266	chr10	3123819	3123852	16	34	2.250000	no	no	yes	no	no	no	no	yes	no	yes
182264267	chr10	3123924	3124004	38	81	2.131579	no	no	yes	no	no	no	no	yes	no	yes
182264271	chr10	3143518	3143642	61	125	2.065574	no	yes	yes	no	yes	no	no	yes	yes	yes
182264277	chr10	3167277	3167313	12	37	3.083333	no	no	yes	no	no	no	no	yes	no	yes
182264278	chr10	3167536	3167706	61	171	2.803279	no	no	yes	no	no	no	no	yes	no	yes

182264279	chr10	3168345	3168376	16	32	2.062500	no	no	yes	no	no	yes	no	no	no	yes
182264284	chr10	3178865	3178899	18	35	1.944444	no	no	no	no	no	no	no	yes	yes	no
182264291	chr10	3184731	3184788	30	58	1.933333	no	no	no	no	no	no	no	no	no	no
182264292	chr10	3184827	3184862	16	36	2.312500	no	no	no	no	no	no	no	no	no	no
182264293	chr10	3186664	3186706	23	43	1.913043	no	no	no	no	no	no	no	no	no	no
182264295	chr10	3191412	3191447	16	36	2.375000	no	no	no	no	no	no	no	no	no	no
182264302	chr10	3199422	3199479	17	58	3.388889	no	no	no	no	no	no	no	yes	no	no
182264306	chr10	3205910	3206397	64	488	7.625000	no	no	yes	no	no	no	no	no	no	yes
182264318	chr10	3215922	3216113	75	192	2.560000	no	no	no	no	no	no	no	no	no	no
182264323	chr10	3220868	3220899	14	32	2.285714	no	no	no	no	no	no	no	yes	no	no
182264324	chr10	3220945	3221005	23	61	2.652174	no	no	no	no	no	no	no	yes	yes	no
182264330	chr10	3224282	3224320	15	39	2.600000	no	no	yes	no	no	no	no	yes	no	yes
182264331	chr10	3224515	3224556	18	42	2.333333	no	no	yes	no	no	no	no	yes	no	yes
182264336	chr10	3230572	3230990	38	419	11.078947	no	no	no	no	no	no	no	no	no	no
182264338	chr10	3231435	3231983	30	549	18.548388	no	no	no	no	no	yes	no	yes	no	no
182264340	chr10	3231969	3232015	21	47	2.238095	no	no	no	no	no	yes	no	yes	no	no
182264341	chr10	3232184	3232226	19	43	2.263158	no	no	no	no	no	yes	no	yes	yes	no
182264342	chr10	3234905	3234937	17	33	2.000000	no	no	yes	no	no	no	no	no	no	yes
182264343	chr10	3240847	3240875	14	29	2.071429	no	no	yes	no	no	no	no	yes	yes	yes
182264344	chr10	3241233	3241275	22	43	2.000000	no	no	yes	no	no	no	no	no	yes	yes
182264345	chr10	3241967	3242023	31	57	1.935484	no	no	yes	no	no	no	no	yes	yes	yes
182264348	chr10	3243006	3243079	22	74	3.363636	no	no	yes	no	no	no	no	no	no	yes
182264349	chr10	3244961	3245026	30	66	2.200000	no	no	yes	no	no	no	no	no	no	yes
182264350	chr10	3251727	3252734	44	1008	22.977272	no	no	yes	no	no	no	no	no	no	yes
182264356	chr10	3253887	3254492	116	606	5.224138	no	no	yes	no	no	no	no	yes	no	yes

182264364	chr10	3276709	3276791	37	83	2.243243	no	no	yes	no	no	no	no	no	no	yes
182264366	chr10	3278638	3278793	76	156	2.052632	yes	no	no	no	no	no	no	no	no	no
182264368	chr10	3281042	3281074	17	33	1.941176	no	no	no	no	no	no	no	no	no	no
182264378	chr10	3292308	3292339	16	32	2.062500	no	no	no	no	no	no	no	no	no	no
182264387	chr10	3313945	3315285	29	1341	46.967743	no	no	no	no	no	no	no	yes	yes	no
182264388	chr10	3316607	3316636	15	30	2.000000	no	no	no	no	no	no	no	no	no	no
182264389	chr10	3317813	3318699	68	887	13.000000	no	no	no	no	no	no	yes	yes	yes	no
182264394	chr10	3321284	3321310	12	27	2.250000	no	no	no	no	no	no	no	no	yes	no
182264403	chr10	3350792	3350884	46	93	2.021739	no	no	no	no	no	no	no	yes	yes	no
182264411	chr10	3357266	3357305	20	40	2.000000	no	no	no	no	no	no	no	no	no	no
182264413	chr10	3360174	3360329	53	156	2.943396	no	no	no	no	no	no	no	no	no	no
182264415	chr10	3367246	3367288	7	43	6.250000	no	no	no	no	no	no	no	no	no	no
182264421	chr10	3373262	3373835	82	574	7.000000	no	no	no	no	no	no	no	yes	yes	no
182264429	chr10	3378222	3378263	21	42	2.000000	no	no	no	no	no	no	no	no	no	no
182264432	chr10	3385080	3385280	108	201	1.879630	no	no	no	no	no	no	no	no	no	no
182264435	chr10	3390871	3390920	26	50	1.923077	no	no	no	no	no	no	no	no	no	no
182264437	chr10	3391661	3391689	11	29	2.727273	no	no	no	no	no	no	no	yes	no	no
182264438	chr10	3391686	3391721	18	36	2.000000	no	no	no	no	no	no	no	yes	no	no
182264443	chr10	3397980	3398071	44	92	2.204545	no	no	yes	no	no	no	no	no	yes	yes
182264444	chr10	3398339	3398390	24	52	2.166667	no	no	yes	no	no	no	no	no	yes	yes
182264450	chr10	3407009	3407120	51	112	2.196079	no	no	no	no	no	no	no	no	no	no
182264451	chr10	3408244	3408446	50	203	4.060000	no	no	no	no	no	no	no	yes	yes	no
182264453	chr10	3409837	3409879	23	43	1.913043	no	no	no	no	no	no	no	no	no	no
182264455	chr10	3411146	3411385	15	240	15.882353	no	no	no	no	no	no	no	yes	yes	no
182264457	chr10	3412477	3412620	36	144	4.000000	no	no	no	no	no	no	no	no	no	no

182264458	chr10	3413988	3414022	15	35	2.333333	no	no	no	no	no	no	no	no	no	no
182264465	chr10	3419581	3419622	22	42	1.909091	no	no	no	no	no	no	no	yes	no	no
182264471	chr10	3420051	3420078	10	28	2.800000	no	no	no	no	no	no	no	yes	yes	no
182264475	chr10	3424278	3424325	25	48	2.000000	no	no	no	no	no	no	no	yes	no	no
182264480	chr10	3434223	3434274	25	52	2.080000	no	no	no	no	yes	no	no	no	yes	yes
182264494	chr10	3456230	3456301	37	72	1.945946	no	no	yes	no	no	no	no	no	no	yes
182264495	chr10	3456271	3458931	46	2661	57.891304	no	no	yes	no	no	no	yes	no	yes	yes
182264496	chr10	3462170	3462522	69	353	5.188406	no	no	yes	no	no	no	no	no	no	yes
182264497	chr10	3462476	3462575	47	100	2.148936	no	no	yes	no	no	no	no	no	no	yes
182264500	chr10	3466697	3466793	50	97	1.940000	no	no	yes	no	no	no	no	no	no	yes
182264503	chr10	3473629	3473666	18	38	2.111111	no	no	yes	no	no	no	no	yes	yes	yes
182264505	chr10	3474490	3474557	35	68	1.942857	no	no	yes	no	no	yes	no	no	no	yes
182264506	chr10	3483643	3483781	68	139	2.102941	no	no	yes	no	no	no	no	yes	yes	yes
182264511	chr10	3492103	3492174	24	72	3.000000	no	no	yes	no	no	no	no	no	no	yes
182264513	chr10	3500850	3501006	78	157	2.012820	yes	no	yes	no	no	yes	no	yes	no	yes
182264516	chr10	3512394	3512423	14	30	2.142857	no	no	no	no	no	no	no	yes	yes	no
182264517	chr10	3516370	3516406	11	37	3.250000	no	no	no	no	no	no	no	yes	yes	no
182264518	chr10	3519214	3519327	23	114	4.956522	no	no	no	no	no	no	no	yes	no	no
182264519	chr10	3519709	3519776	33	68	2.060606	no	no	no	no	no	no	no	no	no	no
182264521	chr10	3528528	3528554	13	27	2.076923	no	no	no	no	no	no	no	no	yes	no
182264523	chr10	3530612	3530848	59	237	4.016949	no	no	no	no	no	no	no	yes	yes	no
182264531	chr10	3532531	3532580	25	50	2.000000	no	no	yes	no	no	no	no	yes	no	yes
182264533	chr10	3533762	3533858	35	97	2.771429	no	no	yes	no	no	no	no	yes	yes	yes
182264534	chr10	3534066	3534096	12	31	2.583333	no	no	yes	no	no	no	no	yes	no	yes
182264543	chr10	3556322	3556749	71	428	6.042253	yes	no	no	no	no	no	yes	yes	yes	no

182264547	chr10	3564149	3564215	34	67	1.970588	no	no	no	no	no	no	no	no	no	no
182264550	chr10	3568959	3569030	37	72	1.972973	no	no	no	no	no	no	no	no	no	no
182264551	chr10	3569067	3569113	16	47	2.937500	no	no	no	no	no	no	no	no	no	no
182264552	chr10	3569734	3570265	177	532	3.005650	no	no	no	no	no	no	no	yes	yes	no
182264561	chr10	3587258	3587497	121	240	1.983471	no	no	no	no	no	no	no	yes	no	no
182264562	chr10	3593711	3593768	26	58	2.307692	no	no	no	no	no	no	no	yes	no	no
182264565	chr10	3594675	3594716	17	42	2.470588	no	no	no	no	no	no	no	no	no	no
182264573	chr10	3615948	3615979	17	32	1.941176	no	no	no	no	no	no	no	no	no	no
182264579	chr10	3648636	3648829	106	194	1.830189	no	no	no	no	no	no	no	yes	yes	no
182264584	chr10	3652801	3652858	31	58	1.935484	no	no	no	no	no	no	no	yes	no	no
182264588	chr10	3656835	3656972	70	138	1.985714	no	no	no	no	no	no	no	no	no	no
182264596	chr10	3663573	3663860	94	288	3.063830	no	no	no	no	no	no	no	no	no	no
182264597	chr10	3666297	3666388	46	92	2.000000	no	no	no	no	no	no	no	yes	yes	no
182264603	chr10	3677294	3677347	28	54	1.928571	no	no	no	no	no	no	no	no	no	no
182264608	chr10	3678889	3679188	152	300	2.000000	no	no	no	no	no	no	no	yes	no	no
182264610	chr10	3679674	3679715	20	42	2.150000	no	no	no	no	no	no	no	yes	no	no
182264618	chr10	3706435	3706534	23	100	4.240000	no	no	no	no	no	no	no	yes	no	no
182264619	chr10	3710970	3711020	21	51	2.428571	no	no	no	no	no	no	no	yes	no	no
182264620	chr10	3711016	3711114	45	99	2.288889	no	no	no	no	no	no	no	yes	no	no
182264626	chr10	3719781	3719816	8	36	4.500000	no	no	no	no	no	no	no	yes	no	no
182264628	chr10	3733035	3733064	15	30	2.000000	no	no	no	no	no	no	no	yes	no	no
182264635	chr10	3742913	3742963	12	51	4.250000	no	no	no	no	no	no	no	no	no	no
182264649	chr10	3759456	3759493	20	38	1.950000	no	no	yes	no	no	no	no	yes	no	yes
182264655	chr10	3768946	3768992	25	47	1.920000	no	no	no	no	no	yes	no	no	no	no
182264658	chr10	3773271	3773390	20	120	5.954545	no	no	no	no	no	no	no	yes	no	no

182264661	chr10	3776920	3776952	16	33	2.062500	no	yes	yes	no	no	no	no	yes	yes	yes
182264669	chr10	3785784	3785928	37	145	3.918919	yes	no	no	no	no	no	yes	yes	yes	no
182264672	chr10	3791574	3791627	17	54	3.176471	no	no	no	no	no	no	no	yes	no	no
182264675	chr10	3791621	3791684	29	64	2.241379	no	no	no	no	no	no	no	yes	no	no
182264677	chr10	3796999	3797031	16	33	2.125000	no	no	no	no	no	no	no	no	no	no
182264689	chr10	3818209	3818319	57	111	1.947368	no	no	no	no	no	no	no	no	no	no
182264692	chr10	3824811	3824845	16	35	2.187500	no	no	no	no	no	no	no	no	no	no
182264695	chr10	3827139	3827285	34	147	4.352941	no	no	no	no	no	no	no	no	no	no
182264697	chr10	3827871	3828018	46	148	3.208333	no	no	no	no	no	no	no	yes	yes	no
182264701	chr10	3828543	3828575	12	33	2.750000	no	no	no	no	no	no	no	yes	yes	no
182264703	chr10	3836535	3836563	13	29	2.307692	no	no	no	no	no	no	no	yes	yes	no
182264708	chr10	3849092	3849156	24	65	2.720000	no	no	no	no	no	no	no	yes	no	no
182264710	chr10	3850572	3850604	16	33	2.062500	no	no	no	no	no	no	no	yes	yes	no
182264715	chr10	3859953	3860025	26	73	2.750000	no	no	no	no	no	no	no	no	no	no
182264721	chr10	3875988	3876033	9	46	5.111111	no	no	no	no	no	no	no	yes	no	no
182264723	chr10	3878547	3878594	24	48	2.041667	no	no	no	no	no	no	no	no	no	no
182264726	chr10	3884527	3884600	38	74	1.947368	no	no	no	no	no	no	no	yes	no	no
182264738	chr10	3903528	3903564	19	37	2.000000	no	no	no	no	no	no	no	yes	no	no
182264744	chr10	3920542	3920999	232	458	1.991379	no	no	yes	no	no	no	no	no	yes	yes
182264747	chr10	3932485	3932594	27	110	4.034483	no	no	yes	no	no	no	no	no	no	yes
182264755	chr10	3946919	3946961	21	43	2.047619	no	no	no	no	no	no	no	yes	no	no
182264764	chr10	3949896	3949964	34	69	2.029412	no	no	no	no	no	no	no	yes	yes	no
182264766	chr10	3957804	3957931	64	128	2.000000	no	no	no	no	no	no	no	no	no	no
182264768	chr10	3973948	3974006	29	59	2.068965	no	no	no	no	no	no	no	no	no	no
182264771	chr10	3974688	3974742	21	55	2.666667	no	no	no	no	no	no	no	no	no	no

182264774	chr10	3982633	3982788	74	156	2.106667	no	no	no	no	no	no	no	no	yes	no
182264775	chr10	3988763	3988795	17	33	1.941176	no	no	no	no	no	no	no	no	no	no
182264777	chr10	3992431	3992475	20	45	2.250000	no	no	no	no	no	no	no	yes	no	no
182264781	chr10	4000800	4000840	20	41	2.150000	no	no	no	no	no	no	no	no	no	no
182264786	chr10	4011910	4012168	37	259	7.000000	no	no	no	no	no	no	no	yes	no	no
182264793	chr10	4013082	4013141	25	60	2.400000	no	no	no	no	no	no	no	yes	no	no
182264795	chr10	4013179	4013219	21	41	2.000000	no	no	no	no	no	no	no	yes	no	no
182264803	chr10	4018004	4018099	47	96	2.063830	no	no	no	no	no	no	no	yes	no	no
182264805	chr10	4020665	4020720	28	56	2.000000	no	no	no	no	no	no	no	no	no	no
182264808	chr10	4028348	4028390	21	43	2.047619	no	yes	yes	no	no	no	no	no	yes	yes
182264812	chr10	4031226	4031337	26	112	4.357143	no	no	yes	no	no	no	no	no	no	yes
182264813	chr10	4031699	4031735	19	37	1.947368	no	yes	yes	no	no	no	no	no	no	yes
182264817	chr10	4055426	4055458	16	33	2.125000	no	no	yes	no	no	no	no	no	no	yes
182264823	chr10	4071074	4071151	40	78	1.950000	no	no	yes	no	no	no	no	no	no	yes
182264834	chr10	4093752	4093811	29	60	2.068965	no	no	no	no	no	no	no	yes	no	no
182264836	chr10	4097196	4097266	20	71	3.550000	no	no	no	no	no	no	no	no	no	no
182264857	chr10	4138265	4138296	17	32	1.941176	no	no	no	no	no	no	no	yes	yes	no
182264864	chr10	4157960	4157989	16	30	1.937500	no	no	no	no	no	no	no	no	no	no
182264869	chr10	4176891	4177130	49	240	4.880000	no	no	no	no	no	no	no	yes	yes	no
182264875	chr10	4177127	4177244	54	118	2.222222	no	no	no	no	no	no	no	yes	yes	no
182264878	chr10	4189286	4189326	22	41	2.000000	no	no	no	no	no	no	no	no	yes	no
182264884	chr10	4199684	4199948	44	265	6.022727	no	no	no	no	no	no	no	no	yes	no
182264886	chr10	4201617	4201653	19	37	2.052632	no	no	no	no	yes	no	no	no	no	yes
182264892	chr10	4213239	4213282	21	44	2.095238	no	no	yes	no	no	no	no	no	no	yes
182264893	chr10	4214081	4214112	16	32	2.062500	no	no	yes	no	no	no	no	yes	no	yes

182264894	chr10	4217777	4217818	21	42	2.000000	no	no	yes	no	no	no	no	yes	yes	yes
182264898	chr10	4220458	4220560	42	103	2.452381	no	no	yes	no	no	no	no	yes	no	yes
182264904	chr10	4236359	4236410	22	52	2.363636	no	no	yes	no	no	yes	no	no	no	yes
182264908	chr10	4247140	4247164	12	25	2.083333	no	no	no	no	no	no	no	yes	yes	no
182264909	chr10	4247908	4247943	18	36	2.000000	no	no	no	no	no	no	no	no	no	no
182264916	chr10	4262605	4262670	34	66	1.941176	no	no	no	no	no	no	no	yes	yes	no
182264918	chr10	4266723	4266763	16	41	2.562500	no	no	no	no	no	no	no	yes	yes	no
182264922	chr10	4277789	4277833	22	45	2.045455	no	no	no	no	no	no	no	no	no	no
182264926	chr10	4294724	4294776	10	53	5.300000	no	no	no	no	no	no	no	no	no	no
182264928	chr10	4294766	4294808	15	43	2.933333	no	no	no	no	no	no	no	no	no	no
182264935	chr10	4295528	4295652	48	125	2.645833	no	no	no	no	no	no	no	yes	no	no
182264944	chr10	4320242	4320281	21	40	1.952381	no	no	no	no	no	no	no	yes	no	no
182264945	chr10	4326474	4326525	25	52	2.120000	no	no	no	no	no	no	no	no	no	no
182264948	chr10	4330375	4330410	18	36	2.000000	no	no	no	no	no	no	no	no	no	no
182264951	chr10	4332609	4332682	40	74	1.925000	no	no	no	no	no	no	no	yes	no	no
182264968	chr10	4347129	4347229	50	101	2.040000	no	no	no	no	no	no	no	no	no	no
182264972	chr10	4352998	4353029	16	32	2.062500	no	no	no	no	no	no	no	no	no	no
182264974	chr10	4360425	4360464	20	40	2.000000	no	no	no	no	no	no	no	no	no	no
182264978	chr10	4380326	4380366	17	41	2.388889	no	no	no	no	no	no	no	no	no	no
182264980	chr10	4380368	4380411	23	44	2.000000	no	no	no	no	no	no	no	no	no	no
182264981	chr10	4381368	4381413	25	46	1.920000	no	no	no	no	no	no	no	no	no	no
182264984	chr10	4395283	4395328	21	46	2.190476	no	no	yes	no	no	no	no	no	no	yes
182264985	chr10	4396164	4396219	26	56	2.153846	no	no	yes	no	no	no	no	no	no	yes
182264994	chr10	4414995	4415085	32	91	2.843750	no	no	no	no	no	yes	no	no	no	no
182264998	chr10	4430635	4430664	15	30	2.000000	no	no	no	no	no	no	no	no	no	no

182265005	chr10	4449095	4449229	27	135	5.074074	no	no	no	no	no	yes	no	no	no	no
182265010	chr10	4460354	4460413	27	60	2.222222	no	no	no	no	no	no	no	yes	no	no
182265018	chr10	4470513	4470549	16	37	2.312500	no	no	no	no	no	no	no	yes	yes	no
182265020	chr10	4480772	4480803	15	32	2.133333	no	no	no	no	no	no	no	no	no	no
182265022	chr10	4483613	4483737	26	125	4.884615	no	no	no	no	no	no	no	yes	yes	no
182265024	chr10	4490327	4490363	19	37	1.947368	no	no	no	no	no	no	no	no	yes	no
182265028	chr10	4493799	4493939	74	141	1.905405	no	no	no	no	no	no	no	no	no	no
182265029	chr10	4495859	4496203	25	345	14.148149	no	no	no	no	no	no	no	no	no	no
182265034	chr10	4509995	4510089	34	95	2.794118	no	no	yes	no	no	no	no	no	no	yes
182265039	chr10	4513727	4513842	23	116	5.041667	no	no	yes	no	no	no	no	no	no	yes
182265042	chr10	4519554	4519598	20	45	2.250000	no	no	no	no	no	no	no	yes	no	no
182265044	chr10	4519761	4519804	15	44	2.933333	no	no	no	no	no	no	no	no	no	no
182265050	chr10	4539495	4539531	17	37	2.176471	no	no	no	no	no	no	no	no	no	no
182265051	chr10	4542043	4542105	14	63	4.500000	no	no	no	no	no	no	no	no	no	no
182265052	chr10	4543644	4543672	15	29	1.933333	no	no	no	no	no	no	no	no	no	no
182265062	chr10	4550368	4550407	16	40	2.562500	no	no	no	no	no	no	no	no	no	no
182265066	chr10	4571754	4571816	23	63	2.720000	no	no	no	no	no	no	no	no	no	no
182265072	chr10	4594580	4594796	108	217	2.009259	no	no	no	no	no	no	no	no	no	no
182265074	chr10	4606227	4606268	19	42	2.210526	no	no	no	no	no	no	no	no	no	no
182265078	chr10	4608298	4608335	20	38	1.900000	no	no	no	no	no	no	no	no	no	no
182265083	chr10	4622762	4622804	20	43	2.250000	no	no	no	no	no	no	no	yes	no	no
182265084	chr10	4625277	4625315	20	39	2.000000	no	no	no	no	no	no	no	no	yes	no
182265086	chr10	4630599	4630678	40	80	2.000000	no	no	no	no	no	no	no	no	no	no
182265094	chr10	4691000	4691040	19	41	2.263158	no	no	no	no	no	no	no	yes	no	no
182265100	chr10	4692135	4692317	47	183	3.823529	no	no	no	no	no	no	no	yes	yes	no

182265108	chr10	4707891	4707990	51	100	1.960784	no	no	no	no	no	no	no	no	no	no
182265109	chr10	4709906	4709933	14	28	2.000000	no	no	no	no	no	no	no	no	no	no
182265114	chr10	4714313	4714369	23	57	2.565217	no	no	no	no	no	no	no	no	no	no
182265116	chr10	4714835	4714875	18	41	2.277778	no	no	no	no	no	no	no	no	no	no
182265118	chr10	4725135	4725203	34	69	2.029412	no	no	no	no	no	no	no	no	no	no
182265124	chr10	4738633	4738747	33	115	3.470588	no	no	no	no	no	no	no	yes	no	no
182265126	chr10	4747067	4747145	35	79	2.257143	no	no	no	no	no	yes	no	no	no	no
182265132	chr10	4754173	4754230	28	58	2.071429	no	no	yes	no	no	no	no	yes	no	yes
182265146	chr10	4803602	4803638	18	37	2.055556	no	no	yes	no	no	no	no	no	no	yes
182265155	chr10	4815436	4816095	110	660	6.000000	no	no	yes	no	no	no	no	yes	yes	yes
182265158	chr10	4816680	4816721	16	42	2.750000	no	no	yes	no	no	no	no	yes	yes	yes
182265166	chr10	4818750	4818774	10	25	2.500000	no	no	yes	no	no	no	no	no	no	yes
182265168	chr10	4826288	4826322	8	35	4.500000	yes	yes	yes	no	yes	no	yes	yes	no	yes
182265169	chr10	4832725	4832763	15	39	2.600000	yes	no	yes	no	no	no	no	no	no	yes
182265181	chr10	4875380	4875439	28	60	2.142857	no	no	yes	no	no	no	no	no	no	yes
182265182	chr10	4890550	4890575	13	26	2.000000	no	no	yes	no	no	no	no	yes	no	yes
182265185	chr10	4890605	4890662	31	58	1.903226	no	no	yes	no	no	no	no	yes	no	yes
182265186	chr10	4890652	4890691	17	40	2.333333	no	no	yes	no	no	no	no	yes	no	yes
182265188	chr10	4901366	4901397	13	32	2.461539	no	no	no	no	no	no	no	no	no	no
182265194	chr10	4914882	4914927	20	46	2.400000	no	no	no	no	no	no	no	yes	no	no
182265195	chr10	4914925	4914966	14	42	2.875000	no	no	no	no	no	no	no	no	no	no
182265208	chr10	4921970	4922059	27	90	3.370370	no	no	no	no	no	no	no	no	no	no
182265211	chr10	4924618	4924813	72	196	2.729730	no	no	no	no	no	no	no	yes	no	no
182265220	chr10	4942786	4942829	21	44	2.142857	no	no	no	no	no	no	no	yes	yes	no
182265228	chr10	4954640	4954737	49	98	2.000000	no	no	no	no	no	no	no	no	no	no

182265236	chr10	4994099	4994134	18	36	2.000000	no	no	yes	no	no	no	no	yes	no	yes
182265241	chr10	4997734	4997766	11	33	3.090909	no	no	yes	no	no	yes	no	yes	no	yes
182265244	chr10	5008626	5008671	23	46	2.086957	no	no	yes	no	no	no	no	yes	yes	yes
182265275	chr10	5094165	5094201	20	37	1.900000	yes	no	yes	no	no	no	no	yes	no	yes
182265279	chr10	5105282	5105330	24	49	2.041667	no	yes	yes	no	no	no	no	yes	no	yes
182265285	chr10	5108657	5108696	20	40	2.000000	no	no	no	no	no	yes	no	yes	no	no
182265302	chr10	5162173	5162205	12	33	2.750000	no	no	yes	no	no	no	no	no	no	yes
182265308	chr10	5214015	5214047	15	33	2.200000	no	no	yes	no	no	no	no	no	no	yes
182265327	chr10	5283965	5284015	22	51	2.409091	no	no	yes	no	no	no	no	yes	yes	yes
182265329	chr10	5285669	5285767	51	99	2.000000	no	no	yes	no	no	no	no	no	yes	yes
182265344	chr10	5305286	5305364	37	79	2.135135	yes	no	no	no	no	no	no	yes	no	no
182265348	chr10	5311757	5311812	13	56	4.214286	no	no	no	no	no	no	no	no	no	no
182265349	chr10	5311797	5311878	33	82	2.484848	no	no	no	no	no	no	no	no	no	no
182265352	chr10	5316332	5316368	19	37	2.052632	no	no	no	no	no	no	no	no	no	no
182265354	chr10	5318307	5318499	76	193	2.539474	no	no	no	no	no	no	no	yes	no	no
182265357	chr10	5327185	5327215	9	31	3.444444	no	no	no	no	no	no	no	no	no	no
182265358	chr10	5327728	5327759	12	32	2.666667	no	no	no	no	no	no	no	yes	no	no
182265366	chr10	5335944	5336010	24	67	2.760000	no	no	no	no	no	no	no	yes	no	no
182265378	chr10	5363009	5363100	20	92	4.600000	no	no	no	no	no	no	no	yes	no	no
182265382	chr10	5371111	5371252	77	142	1.922078	no	no	yes	no	no	no	no	yes	no	yes
182265398	chr10	5394988	5395032	21	45	2.142857	no	no	yes	no	no	no	no	yes	no	yes
182265410	chr10	5412814	5412911	33	98	2.969697	no	no	yes	yes	no	no	yes	yes	yes	yes
182265413	chr10	5412998	5413040	20	43	2.150000	no	no	yes	no	no	no	yes	yes	no	yes
182265429	chr10	5452106	5452144	17	39	2.294118	yes	no	yes	no	no	no	no	yes	no	yes
182265431	chr10	5463238	5463296	27	59	2.185185	no	no	no	no	no	no	no	yes	no	no

182265437	chr10	5479985	5480203	44	219	4.977273	no	no	no	no	no	no	no	yes	yes	no
182265438	chr10	5480950	5481126	43	177	4.139535	no	no	no	no	no	no	no	yes	no	no
182265439	chr10	5484742	5484784	20	43	2.150000	no	no	no	no	no	no	no	yes	no	no
182265442	chr10	5493974	5494193	82	220	2.764706	no	no	no	no	no	no	no	yes	yes	no
182265446	chr10	5503261	5503306	24	46	1.916667	no	no	no	no	no	no	no	yes	yes	no
182265450	chr10	5503303	5503465	74	163	2.202703	no	no	no	no	no	no	no	yes	yes	no
182265453	chr10	5503931	5504160	20	230	11.142858	no	no	no	no	no	no	no	yes	yes	no
182265455	chr10	5506844	5506910	35	67	1.914286	no	no	no	no	no	no	no	no	yes	no
182265456	chr10	5507998	5508070	37	73	1.972973	no	no	no	no	no	no	no	yes	yes	no
182265457	chr10	5508472	5508543	37	72	1.945946	no	no	no	no	no	no	no	no	yes	no
182265458	chr10	5515422	5515458	16	37	2.312500	no	no	no	no	yes	no	no	no	no	yes
182265465	chr10	5539430	5539600	85	171	2.011765	no	no	no	no	no	no	no	yes	yes	no
182265472	chr10	5551819	5551870	20	52	2.600000	no	no	yes	no	yes	no	no	yes	yes	yes
182265475	chr10	5558058	5558163	35	106	3.028571	no	no	no	no	no	no	no	no	yes	no
182265483	chr10	5563319	5563402	38	84	2.263158	no	no	no	no	yes	no	no	yes	yes	yes
182265505	chr10	5602152	5602454	44	303	6.886364	no	no	yes	no	no	no	no	yes	no	yes
182265520	chr10	5616220	5616573	19	354	19.000000	no	no	yes	no	no	yes	yes	yes	no	yes
182265524	chr10	5617809	5617895	24	87	3.625000	no	no	yes	no	no	no	no	yes	yes	yes
182265527	chr10	5629800	5629844	21	45	2.142857	no	no	yes	no	no	no	no	yes	yes	yes
182265528	chr10	5631318	5631361	19	44	2.315789	yes	no	yes	no	no	no	no	yes	no	yes
182265532	chr10	5635833	5635857	12	25	2.083333	no	no	no	no	no	no	no	no	yes	no
182265533	chr10	5644832	5644877	23	46	2.043478	no	no	yes	no	no	no	no	no	no	yes
182265534	chr10	5644967	5645002	16	36	2.250000	no	no	yes	no	no	no	no	no	no	yes
182265535	chr10	5645244	5645279	14	36	2.571429	no	no	yes	no	no	no	no	yes	no	yes
182265546	chr10	5666584	5666628	23	45	2.000000	yes	no	no	no	yes	no	yes	yes	yes	yes

182265548	chr10	5666994	5667032	18	39	2.166667	yes	no	no	no	no	no	no	yes	yes	no
182265549	chr10	5667027	5667070	15	44	3.066667	yes	no	no	no	no	no	no	yes	yes	no
182265551	chr10	5667112	5667160	25	49	2.040000	yes	no	no	no	no	no	no	yes	yes	no
182265561	chr10	5684551	5684594	23	44	1.913043	yes	no	no	no	no	no	yes	yes	yes	no
182265576	chr10	5716889	5716940	22	52	2.363636	no	no	yes	no	no	no	no	no	yes	yes
182265581	chr10	5732068	5732198	67	131	1.955224	no	no	yes	no	no	no	no	no	yes	yes
182265582	chr10	5734296	5734463	83	168	2.024096	no	no	yes	no	no	no	no	no	no	yes
182265584	chr10	5739384	5739521	69	138	2.000000	no	no	yes	no	no	no	no	no	no	yes
182265597	chr10	5794843	5794888	22	46	2.090909	no	no	yes	yes	no	yes	no	no	no	yes
182265602	chr10	5799767	5799807	21	41	2.000000	no	no	yes	no	no	no	no	no	no	yes
182265603	chr10	5813172	5813207	14	36	2.571429	yes	no	yes	no	no	no	yes	yes	no	yes
182265608	chr10	5823093	5823120	13	28	2.153846	no	no	yes	no	no	no	no	no	no	yes
182265613	chr10	5828172	5828284	56	113	2.017857	no	no	yes	no	no	no	no	no	no	yes
182265617	chr10	5841511	5841551	21	41	1.952381	no	no	yes	no	no	no	no	yes	no	yes
182265649	chr10	5897367	5897420	26	54	2.076923	no	no	yes	no	no	no	no	yes	no	yes
182265652	chr10	5899871	5899927	29	57	2.000000	no	no	yes	no	no	no	no	yes	no	yes
182265658	chr10	5916475	5916745	98	271	2.735294	yes	no	yes	no	no	yes	no	yes	no	yes
182265661	chr10	5923556	5923598	18	43	2.444444	yes	yes	yes	no	no	no	no	no	no	yes
182265662	chr10	5925188	5925222	17	35	2.058824	yes	no	yes	no	no	yes	no	no	no	yes
182265677	chr10	5969237	5969368	46	132	2.872340	no	no	yes	no	no	no	no	yes	no	yes
182265686	chr10	5985231	5985316	29	86	2.965517	no	no	no	no	no	no	no	yes	no	no
182265691	chr10	5998467	5998517	16	51	3.187500	no	no	no	no	no	no	no	no	no	no
182265699	chr10	6000960	6001441	193	482	2.512820	no	no	no	no	no	no	no	yes	yes	no
182265713	chr10	6024585	6024682	26	98	3.714286	no	no	yes	no	no	no	no	no	yes	yes
182265719	chr10	6045867	6045898	15	32	2.133333	no	no	yes	no	no	no	no	yes	yes	yes

182265723	chr10	6048685	6048764	39	80	2.076923	no	no	yes	no	no	no	no	no	yes	yes
182265731	chr10	6065941	6065975	17	35	2.058824	no	no	no	no	no	no	no	yes	yes	no
182265736	chr10	6075592	6075745	17	154	9.058824	no	no	no	no	no	no	no	no	no	no
182265740	chr10	6088185	6088227	20	43	2.150000	yes	no	no	no	no	no	no	yes	no	no
182265742	chr10	6088989	6089021	17	33	1.941176	yes	no	no	no	no	no	no	yes	yes	no
182265748	chr10	6090986	6091070	28	85	3.035714	no	no	yes	no	no	no	no	no	no	yes
182265753	chr10	6113436	6113496	32	61	1.906250	yes	yes	yes	no	no	yes	no	no	no	yes
182265759	chr10	6127450	6127732	44	283	6.431818	no	no	no	no	no	no	no	no	no	no
182265768	chr10	6139863	6139963	27	101	3.777778	no	no	no	no	no	no	no	yes	no	no
182265771	chr10	6140197	6140288	45	92	2.066667	no	no	no	no	no	no	no	yes	yes	no
182265776	chr10	6153948	6153985	19	38	2.000000	no	no	yes	no	no	no	no	yes	yes	yes
182265781	chr10	6163671	6163712	20	42	2.100000	yes	no	yes	no	no	no	yes	yes	yes	yes
182265784	chr10	6167494	6167525	10	32	3.200000	no	no	yes	no	no	no	no	yes	no	yes
182265785	chr10	6170629	6170660	15	32	2.133333	no	no	yes	no	no	no	no	yes	yes	yes
182265786	chr10	6170659	6170735	38	77	2.131579	no	no	yes	no	no	no	no	yes	yes	yes
182265792	chr10	6177980	6178020	15	41	2.733333	no	no	yes	no	no	no	no	yes	yes	yes
182265794	chr10	6183610	6183666	29	57	1.965517	no	no	yes	no	no	no	no	no	no	yes
182265805	chr10	6200658	6200698	18	41	2.333333	no	no	yes	no	no	no	no	yes	yes	yes
182265806	chr10	6201433	6201486	24	54	2.250000	no	no	yes	no	no	no	yes	yes	no	yes
182265808	chr10	6203885	6203926	20	42	2.100000	no	no	yes	no	no	no	yes	yes	yes	yes
182265819	chr10	6215314	6215449	38	136	3.564103	no	no	yes	yes	no	no	yes	yes	no	yes
182265821	chr10	6216845	6216926	41	82	2.000000	no	no	yes	no	no	no	no	yes	no	yes
182265823	chr10	6220403	6220447	22	45	2.090909	yes	no	yes	no	no	no	no	no	yes	yes
182265826	chr10	6227181	6227216	18	36	2.000000	no	no	yes	no	no	yes	no	yes	no	yes
182265829	chr10	6231757	6231921	63	165	2.634921	no	no	yes	no	no	yes	no	yes	no	yes

182265850	chr10	6275391	6275444	24	54	2.250000	no	no	yes	no	no	no	no	yes	yes	yes
182265862	chr10	6293928	6293989	31	62	2.032258	no	no	yes	no	yes	no	no	no	no	yes
182265880	chr10	6319641	6319690	18	50	2.700000	no	no	yes	no	no	no	no	yes	no	yes
182265882	chr10	6319769	6319811	21	43	2.095238	no	no	yes	no	no	no	no	no	no	yes
182265890	chr10	6338033	6338143	60	111	1.900000	no	no	yes	no	no	no	no	no	no	yes
182265896	chr10	6340405	6340449	16	45	2.812500	no	no	yes	no	no	no	no	no	no	yes
182265897	chr10	6343491	6343533	22	43	2.000000	no	no	yes	no	no	no	no	yes	no	yes
182265911	chr10	6392099	6392145	23	47	2.043478	no	no	no	no	no	no	no	no	no	no
182265912	chr10	6392298	6392329	17	32	1.941176	no	no	no	no	no	no	no	no	no	no
182265927	chr10	6417975	6418033	28	59	2.107143	no	no	no	no	no	no	no	no	no	no
182265932	chr10	6431420	6431456	18	37	2.055556	no	no	yes	no	no	no	no	yes	no	yes
182265941	chr10	6476824	6476953	64	130	2.031250	no	no	yes	no	no	no	no	yes	no	yes
182265952	chr10	6500398	6500455	28	58	2.107143	no	no	yes	no	no	no	no	no	no	yes
182265956	chr10	6505386	6505525	72	140	2.000000	no	no	yes	no	no	no	no	yes	no	yes
182265959	chr10	6509969	6510089	39	121	3.179487	no	no	yes	no	no	no	no	no	no	yes
182265965	chr10	6519000	6519047	24	48	2.000000	no	no	yes	no	no	no	no	no	no	yes
182265968	chr10	6523833	6524000	84	168	2.000000	no	no	yes	no	no	no	no	no	no	yes
182265969	chr10	6532436	6532586	49	151	3.081633	no	no	yes	no	no	no	no	no	yes	yes
182265980	chr10	6580257	6580329	9	73	7.545455	yes	no	no	no	yes	no	yes	yes	yes	yes
182265981	chr10	6580435	6580474	21	40	1.904762	yes	yes	no	no	no	no	yes	yes	yes	yes
182265989	chr10	6625887	6625957	37	71	1.972973	no	no	no	no	no	yes	no	no	no	no
182266003	chr10	6645454	6645493	20	40	2.000000	no	no	no	no	no	no	no	no	no	no
182266017	chr10	6675039	6675078	20	40	2.000000	no	no	no	no	no	no	no	no	no	no
182266019	chr10	6678401	6678576	88	176	2.000000	no	no	no	no	no	no	no	yes	yes	no
182266020	chr10	6678962	6679003	22	42	1.909091	no	no	no	no	no	no	no	yes	no	no

182266030	chr10	6698471	6698506	18	36	2.000000	no	no	yes	no	no	no	no	yes	yes	yes
182266031	chr10	6702024	6702065	18	42	2.333333	no	no	yes	no	no	no	no	yes	yes	yes
182266044	chr10	6729440	6729478	18	39	2.166667	no	no	yes	no	no	no	no	yes	no	yes
182266046	chr10	6738425	6738472	20	48	2.400000	no	yes	yes	no	no	no	no	yes	yes	yes
182266047	chr10	6738931	6738968	19	38	2.000000	no	yes	yes	no	no	yes	no	yes	no	yes
182266051	chr10	6744028	6744060	15	33	2.200000	no	no	yes	no	no	no	no	no	no	yes
182266057	chr10	6768985	6769023	20	39	1.950000	no	no	yes	no	no	no	no	no	no	yes
182266065	chr10	6781012	6781057	20	46	2.350000	no	no	yes	no	no	no	no	yes	yes	yes
182266072	chr10	6785941	6786014	39	74	2.025641	no	no	yes	no	no	no	no	yes	yes	yes
182266075	chr10	6793614	6793667	23	54	2.434783	no	no	yes	no	no	no	no	yes	no	yes
182266087	chr10	6813432	6813492	21	61	2.952381	no	no	yes	no	no	no	no	yes	yes	yes
182266091	chr10	6834772	6834813	20	42	2.100000	no	no	yes	no	no	yes	no	no	no	yes
182266094	chr10	6834934	6835691	24	758	31.692308	no	no	yes	no	no	yes	no	no	no	yes
182266097	chr10	6835731	6835776	20	46	2.300000	no	no	yes	no	no	no	no	no	no	yes
182266104	chr10	6835962	6836051	47	90	1.936170	no	no	yes	no	no	no	no	no	no	yes
182266107	chr10	6836079	6836171	41	93	2.317073	no	no	yes	no	no	no	no	no	yes	yes
182266114	chr10	6837001	6837047	17	47	2.882353	no	no	yes	no	no	no	no	no	no	yes
182266117	chr10	6837157	6837232	40	76	1.925000	no	no	yes	no	no	no	no	no	no	yes
182266122	chr10	6837306	6837356	27	51	1.925926	no	no	yes	no	no	no	no	no	no	yes
182266130	chr10	6843830	6843925	45	96	2.133333	no	no	yes	no	no	yes	no	yes	yes	yes
182266137	chr10	6867067	6867126	32	60	1.906250	no	no	yes	no	no	no	no	no	no	yes
182266139	chr10	6871245	6871312	34	68	2.000000	no	no	yes	no	no	no	no	yes	yes	yes
182266149	chr10	6894552	6894684	32	133	4.156250	no	no	yes	no	no	no	no	yes	no	yes
182266164	chr10	6923862	6923945	21	84	3.875000	no	no	yes	no	no	no	no	no	no	yes
182266185	chr10	6966065	6966106	20	42	2.100000	no	no	yes	no	no	no	no	yes	no	yes

182266195	chr10	7004000	7004280	101	281	2.821782	no	no	yes	no	no	no	no	yes	yes	yes
182266198	chr10	7015905	7015938	17	34	2.058824	no	no	yes	no	no	no	no	yes	no	yes
182266205	chr10	7034253	7034384	66	132	2.000000	no	no	yes	no	no	no	no	no	no	yes
182266206	chr10	7038926	7038982	22	57	2.590909	no	no	yes	no	no	no	no	no	no	yes
182266219	chr10	7055309	7055354	21	46	2.190476	no	no	yes	no	no	no	no	yes	no	yes
182266221	chr10	7064360	7064406	21	47	2.285714	no	no	yes	no	no	no	no	yes	no	yes
182266229	chr10	7085715	7085745	16	31	1.937500	no	no	yes	no	no	no	no	yes	yes	yes
182266230	chr10	7089400	7089481	23	82	3.565217	no	no	yes	no	no	no	no	no	no	yes
182266232	chr10	7096381	7096425	20	45	2.250000	no	yes	yes	no	no	no	no	no	no	yes
182266237	chr10	7098560	7098632	35	73	2.142857	no	no	yes	no	no	yes	no	yes	no	yes
182266239	chr10	7099011	7099160	74	150	2.027027	no	no	yes	no	no	no	no	no	no	yes
182266240	chr10	7101720	7101747	14	28	2.000000	no	no	yes	no	no	no	no	no	no	yes
182266247	chr10	7125386	7125410	12	25	2.083333	no	no	no	no	no	no	no	no	no	no
182266248	chr10	7131536	7131564	15	29	1.933333	no	no	no	no	no	no	no	no	no	no
182266251	chr10	7135957	7136029	22	73	3.318182	no	no	no	no	no	no	no	no	no	no
182266252	chr10	7136530	7136989	57	460	8.070175	no	no	no	no	no	no	no	yes	no	no
182266254	chr10	7143535	7143588	26	54	2.076923	no	no	no	no	no	no	no	no	no	no
182266255	chr10	7147597	7148294	41	698	17.024391	no	no	no	no	no	no	no	yes	no	no
182266262	chr10	7152059	7152134	39	76	1.948718	no	no	no	no	no	no	no	no	no	no
182266265	chr10	7158043	7158109	35	67	1.914286	no	no	no	no	no	yes	no	no	no	no
182266270	chr10	7168543	7168596	23	54	2.347826	no	no	yes	no	no	no	no	yes	yes	yes
182266272	chr10	7179462	7179500	20	39	1.950000	no	no	yes	no	no	no	no	yes	no	yes
182266277	chr10	7201800	7201880	39	81	2.076923	no	no	yes	no	no	no	no	no	no	yes
182266280	chr10	7210807	7210835	12	29	2.416667	no	no	yes	no	no	no	no	yes	no	yes
182266281	chr10	7213679	7213851	34	173	5.117647	no	no	yes	no	no	no	no	no	no	yes

182266284	chr10	7221704	7221746	13	43	3.187500	no	no	yes	no	no	no	no	no	no	yes
182266292	chr10	7239896	7239923	8	28	3.500000	no	no	yes	no	no	no	no	yes	no	yes
182266293	chr10	7246393	7246430	19	38	2.000000	no	no	yes	no	no	no	no	yes	no	yes
182266296	chr10	7259882	7259938	27	57	2.111111	no	no	yes	no	no	no	no	yes	no	yes
182266301	chr10	7282968	7282995	14	28	2.000000	no	no	yes	no	no	no	no	no	no	yes
182266307	chr10	7294564	7294682	42	119	2.833333	no	no	yes	no	no	yes	no	no	no	yes
182266310	chr10	7298598	7298660	30	63	2.100000	no	no	yes	no	no	no	no	yes	no	yes
182266320	chr10	7313169	7313206	19	38	2.000000	no	no	yes	no	no	no	no	no	no	yes
182266323	chr10	7316875	7316917	22	43	2.090909	no	no	yes	no	no	no	no	no	yes	yes
182266330	chr10	7330677	7330713	18	37	2.166667	no	no	yes	no	no	no	no	no	no	yes
182266332	chr10	7333736	7333786	26	51	1.961538	no	no	yes	no	no	no	no	no	no	yes
182266341	chr10	7350211	7350259	23	49	2.130435	no	no	yes	no	no	no	no	no	no	yes
182266342	chr10	7352013	7352046	16	34	2.125000	no	no	yes	no	no	no	no	yes	yes	yes
182266351	chr10	7367270	7367389	39	120	3.076923	no	no	yes	no	no	no	no	no	no	yes
182266363	chr10	7378017	7378597	99	581	5.813084	no	no	yes	no	no	no	no	yes	no	yes
182266365	chr10	7378725	7378826	30	102	3.333333	no	no	yes	no	no	no	no	yes	yes	yes
182266381	chr10	7456803	7456841	18	39	2.166667	no	no	yes	no	no	no	no	no	no	yes
182266386	chr10	7462683	7462723	14	41	3.000000	no	no	yes	no	no	no	no	no	no	yes
182266389	chr10	7475980	7476020	18	41	2.277778	no	no	no	no	no	no	no	yes	no	no
182266407	chr10	7505542	7505588	22	47	2.181818	no	no	no	no	no	no	no	yes	no	no
182266433	chr10	7527560	7527594	14	35	2.571429	no	no	no	no	no	no	no	yes	no	no
182266438	chr10	7532898	7532922	13	25	1.923077	yes	no	no	no	no	no	no	yes	yes	no
182266445	chr10	7545693	7545720	13	28	2.153846	no	no	no	no	no	no	no	no	no	no
182266446	chr10	7547656	7547757	35	102	2.914286	no	no	no	no	no	no	no	no	no	no
182266451	chr10	7556866	7556897	9	32	3.555556	no	no	no	no	no	no	no	yes	no	no

182266453 chr10 7564267 7564348 28 82 2.928571 no no yes no no no
no no no yes
182266465 chr10 7565472 7565876 219 405 1.894977 no no yes no no yes
no yes no yes
182266472 chr10 7578854 7578913 32 60 1.906250 yes no yes no no no
no no no yes
182266482 chr10 7593328 7593880 33 553 16.787878 no no yes no no no
no yes yes yes
182266484 chr10 7595426 7595559 67 134 2.000000 no no yes no no no
no no no yes
182266486 chr10 7597503 7597679 59 177 3.000000 no no yes no no no
no no no yes
182266487 chr10 7607927 7607954 13 28 2.153846 no no yes no no no
no yes no yes
182266488 chr10 7609561 7609626 31 66 2.161290 no no yes no no no
no no no yes
182266492 chr10 7613394 7613420 12 27 2.250000 no no yes no no no
no no no yes
182266493 chr10 7614669 7614723 18 55 3.052632 no no yes no no no
no no no yes
182266494 chr10 7622702 7622739 18 38 2.111111 yes no yes no no no
no no no yes
182266498 chr10 7626296 7626347 22 52 2.363636 no no yes no no no
no yes yes yes
182266499 chr10 7626816 7626849 16 34 2.187500 no no yes no no no
no no no yes
182266503 chr10 7628591 7628717 63 127 2.015873 no no yes no no no
no yes no yes
182266505 chr10 7635574 7635627 25 54 2.200000 no no yes no no no
no yes yes yes
182266506 chr10 7641535 7641574 19 40 2.105263 yes no yes no no no
no yes no yes
182266517 chr10 7649232 7649333 48 102 2.166667 no no yes no no no
no yes no yes
182266522 chr10 7666982 7667344 29 363 12.655172 yes no no no yes no
yes yes yes yes
182266523 chr10 7667521 7667568 21 48 2.333333 yes no no no no no
no yes yes no
182266524 chr10 7671781 7671836 23 56 2.478261 no no no no no no
no no no no
182266530 chr10 7672836 7673127 30 292 9.709678 no no no no no no
no yes yes no
182266542 chr10 7687694 7687863 84 170 2.023809 no no no no no no
no yes yes no
182266544 chr10 7697683 7697771 42 89 2.119048 no no no no no no
no no yes no
182266550 chr10 7723794 7723840 24 47 2.041667 no no yes no no no
no yes no yes
182266558 chr10 7733397 7733638 135 242 1.807407 no no yes no no yes
no no no yes

182266562	chr10	7737365	7737447	37	83	2.263158	no	no	yes	no	no	no	no	yes	no	yes
182266567	chr10	7743880	7743924	22	45	2.181818	no	no	yes	no	no	no	no	no	no	yes
182266571	chr10	7749413	7749473	30	61	2.033333	no	yes	no	no	no	yes	no	no	no	yes
182266575	chr10	7757589	7757625	15	37	2.466667	no	no	yes	no	no	no	no	yes	no	yes
182266576	chr10	7765523	7765562	21	40	1.952381	no	no	yes	no	no	no	no	no	no	yes
182266577	chr10	7765568	7765606	14	39	2.733333	no	no	yes	no	no	no	no	no	no	yes
182266579	chr10	7769503	7769604	51	102	2.000000	no	no	yes	no	no	no	no	no	no	yes
182266589	chr10	7802401	7802476	38	76	2.000000	no	yes	yes	yes	no	no	no	yes	yes	yes
182266590	chr10	7803238	7803273	16	36	2.250000	no	no	yes	no	no	no	no	no	no	yes
182266615	chr10	7864942	7864966	13	25	1.923077	no	no	yes	no	no	no	no	no	no	yes
182266617	chr10	7868263	7868533	46	271	5.916667	no	no	yes	no	no	no	no	yes	yes	yes
182266622	chr10	7871338	7871384	20	47	2.350000	no	no	yes	no	no	no	no	yes	no	yes
182266631	chr10	7911322	7911365	22	44	2.045455	no	no	yes	no	no	no	no	no	no	yes
182266644	chr10	7953724	7955082	68	1359	20.014492	no	no	yes	no	no	no	no	yes	yes	yes
182266648	chr10	7957268	7957311	21	44	2.095238	no	no	yes	no	no	no	no	yes	yes	yes
182266651	chr10	7959403	7959480	39	78	2.000000	no	no	yes	no	no	no	no	yes	no	yes
182266653	chr10	7964454	7964485	9	32	3.555556	no	no	no	yes	no	no	no	yes	no	yes
182266675	chr10	7997915	7997957	22	43	1.954545	no	no	yes	no	no	no	no	no	no	yes
182266682	chr10	8023811	8023985	82	175	2.134146	no	no	no	no	no	no	no	yes	no	no
182266702	chr10	8064272	8064323	22	52	2.363636	no	no	yes	no	no	no	no	yes	yes	yes
182266709	chr10	8074500	8074546	24	47	2.000000	no	yes	no	no	no	yes	no	yes	no	yes
182266715	chr10	8092421	8092459	18	39	2.222222	no	no	no	no	no	no	no	yes	no	no
182266721	chr10	8098926	8098987	33	62	1.909091	no	no	no	no	no	no	no	no	no	no
182266723	chr10	8107872	8107913	22	42	1.909091	no	no	no	no	no	no	no	no	no	no
182266724	chr10	8108707	8108746	20	40	2.050000	no	no	no	no	no	no	no	no	no	no

182266744	chr10	8138608	8138681	38	74	1.973684	no	no	no	no	no	no	no	yes	yes	no
182266745	chr10	8141678	8141707	11	30	2.666667	no	no	no	no	no	no	no	no	no	no
182266748	chr10	8156769	8157018	66	250	3.823529	no	no	no	no	no	no	no	yes	no	no
182266751	chr10	8158079	8158119	21	41	1.952381	no	no	no	no	no	no	no	no	no	no
182266752	chr10	8159815	8159859	23	45	1.956522	no	no	no	no	no	no	no	yes	no	no
182266755	chr10	8169940	8170007	35	68	1.942857	no	no	no	no	no	no	no	no	no	no
182266757	chr10	8170914	8170981	14	68	4.750000	no	no	no	no	no	no	no	no	no	no
182266762	chr10	8178338	8178372	18	35	1.944444	no	no	no	no	no	no	no	no	no	no
182266766	chr10	8191754	8191790	16	37	2.312500	no	no	no	no	no	no	no	no	no	no
182266769	chr10	8193857	8193926	35	70	2.000000	no	no	no	no	no	no	no	yes	no	no
182266775	chr10	8196286	8196336	24	51	2.125000	no	no	no	no	no	no	no	no	no	no
182266792	chr10	8220170	8220207	21	38	1.904762	no	no	no	no	no	no	no	no	no	no
182266796	chr10	8233363	8233433	33	71	2.151515	no	no	no	no	no	no	no	yes	no	no
182266798	chr10	8235815	8235855	19	41	2.157895	no	no	no	no	no	no	no	no	yes	no
182266806	chr10	8243107	8243166	16	60	3.722222	no	no	no	no	no	no	no	no	no	no
182266808	chr10	8251421	8251468	26	48	1.923077	no	no	no	no	no	no	no	no	no	no
182266821	chr10	8265393	8265417	10	25	2.500000	no	no	yes	no	no	no	no	no	no	yes
182266822	chr10	8266993	8267022	15	30	2.066667	no	no	yes	no	no	no	no	yes	no	yes
182266825	chr10	8270962	8270995	13	34	2.615385	no	no	no	no	no	no	no	no	no	no
182266838	chr10	8286848	8286881	9	34	3.777778	no	no	no	no	no	no	no	no	no	no
182266841	chr10	8293721	8293778	28	58	2.142857	no	no	no	no	no	no	no	yes	no	no
182266844	chr10	8295536	8295665	46	130	2.833333	no	no	no	no	no	no	no	yes	no	no
182266850	chr10	8296805	8296866	25	62	2.480000	no	no	no	no	no	no	no	yes	yes	no
182266864	chr10	8306453	8306520	12	68	5.833333	no	no	no	no	no	no	no	yes	yes	no
182266865	chr10	8307229	8307263	14	35	2.571429	no	no	no	no	no	no	no	no	yes	no

182266866	chr10	8309540	8309566	13	27	2.076923	no	no	no	no	no	no	no	no	no	no
182266868	chr10	8315748	8315791	22	44	2.000000	no	no	no	no	no	no	no	yes	yes	no
182266870	chr10	8325224	8325261	20	38	1.900000	no	no	no	no	no	no	no	no	yes	no
182266882	chr10	8336357	8336400	19	44	2.368421	no	no	no	no	no	no	no	no	no	no
182266884	chr10	8338961	8339012	25	52	2.080000	no	no	no	no	no	no	no	no	no	no
182266890	chr10	8352003	8352032	14	30	2.142857	no	no	no	no	no	no	no	no	no	no
182266891	chr10	8353655	8353699	22	45	2.136364	no	no	no	no	no	no	no	no	no	no
182266899	chr10	8357813	8357844	16	32	2.000000	no	no	no	no	no	no	no	no	no	no
182266905	chr10	8362543	8363156	20	614	30.730770	no	no	no	no	no	no	no	no	no	no
182266926	chr10	8390697	8390738	12	42	3.500000	no	no	no	no	no	no	no	no	no	no
182266964	chr10	8498437	8498482	25	46	1.920000	no	no	no	no	no	no	no	yes	no	no
182266975	chr10	8501143	8501203	28	61	2.178571	no	no	no	no	no	no	no	no	no	no
182266976	chr10	8503618	8503646	15	29	1.933333	no	no	no	no	no	no	no	no	no	no
182266985	chr10	8525615	8525803	96	189	1.979167	no	no	no	no	no	no	no	yes	no	no
182267000	chr10	8554571	8554610	20	40	2.000000	no	no	no	no	no	no	no	yes	no	no
182267005	chr10	8562762	8562985	25	224	8.838710	no	no	no	no	no	no	no	no	no	no
182267013	chr10	8580994	8581064	37	71	1.918919	no	no	no	no	no	no	no	no	yes	no
182267022	chr10	8594817	8594841	13	25	1.923077	no	no	no	no	no	no	no	yes	yes	no
182267032	chr10	8616974	8617002	12	29	2.416667	no	no	no	no	no	no	no	no	no	no
182267033	chr10	8617278	8617343	34	66	1.941176	no	no	no	no	no	no	no	yes	no	no
182267041	chr10	8639449	8639492	12	44	3.666667	no	no	no	no	no	no	no	no	no	no
182267042	chr10	8646831	8646866	12	36	3.000000	no	no	no	no	no	no	no	no	no	no
182267043	chr10	8647203	8647260	27	58	2.148148	no	no	no	no	no	no	no	no	no	no
182267044	chr10	8650359	8650401	19	43	2.263158	no	no	no	no	no	no	no	no	no	no
182267048	chr10	8661217	8661290	20	74	3.700000	no	no	no	no	no	no	no	no	no	no

182267054	chr10	8663449	8663490	20	42	2.150000	no	no	no	no	no	no	no	yes	no	no
182267060	chr10	8679128	8679175	18	48	2.777778	no	no	no	no	no	no	no	no	no	no
182267067	chr10	8688113	8688154	16	42	2.750000	no	no	no	no	no	no	no	no	no	no
182267072	chr10	8698578	8698641	32	64	2.031250	no	no	no	no	no	no	no	yes	no	no
182267080	chr10	8723573	8723615	22	43	2.000000	yes	no	no	no	no	no	no	no	no	no
182267081	chr10	8723887	8723968	40	82	2.050000	yes	no	no	no	no	no	no	no	no	no
182267085	chr10	8732123	8732173	24	51	2.166667	no	no	no	no	no	no	no	no	no	no
182267086	chr10	8737028	8737059	17	32	1.941176	no	no	no	no	no	no	no	no	no	no
182267087	chr10	8738256	8738291	18	36	2.000000	no	no	no	no	no	no	no	no	no	no
182267092	chr10	8747561	8747590	14	30	2.142857	no	no	no	no	no	no	no	yes	no	no
182267096	chr10	8765474	8765518	18	45	2.555556	no	no	no	no	no	no	no	no	no	no
182267100	chr10	8770149	8770174	13	26	2.000000	no	no	no	no	no	no	no	no	no	no
182267106	chr10	8791044	8791084	12	41	3.307692	no	no	no	no	no	no	no	no	no	no
182267109	chr10	8800043	8800118	25	76	3.040000	no	no	no	no	no	no	no	no	no	no
182267112	chr10	8807145	8807223	40	79	1.975000	no	no	no	no	no	no	no	no	no	no
182267113	chr10	8817107	8817147	19	41	2.210526	no	no	no	no	no	no	no	no	no	no
182267118	chr10	8825321	8825389	33	69	2.090909	no	no	no	no	no	no	no	no	no	no
182267120	chr10	8828512	8828549	20	38	1.900000	no	no	no	no	no	no	no	no	no	no
182267126	chr10	8840312	8840385	36	74	2.055556	no	no	no	no	no	no	no	no	no	no
182267138	chr10	8855972	8856090	24	119	4.777778	no	no	no	no	no	no	no	no	no	no
182267145	chr10	8867244	8867319	38	76	2.052632	no	no	no	no	no	no	no	yes	yes	no
182267184	chr10	8972504	8972535	16	32	2.000000	no	no	yes	no	no	no	no	no	no	yes
182267190	chr10	8999865	8999898	16	34	2.187500	no	no	no	no	no	no	no	no	no	no
182267194	chr10	9003580	9003612	17	33	1.941176	no	no	no	no	no	no	no	no	no	no
182267218	chr10	9064314	9064393	42	80	1.904762	no	no	no	no	no	no	no	no	no	no

182267228	chr10	9074794	9074920	18	127	7.142857	no	no	no	no	no	no	no	no	no	no
182267230	chr10	9085749	9085794	16	46	2.937500	no	no	no	no	no	no	no	no	no	no
182267231	chr10	9089193	9089234	21	42	2.047619	no	no	no	no	no	no	no	no	no	no
182267233	chr10	9094557	9094597	21	41	2.000000	no	no	no	no	no	no	no	no	no	no
182267249	chr10	9112976	9113037	27	62	2.407408	no	no	no	no	no	no	no	no	no	no
182267250	chr10	9113660	9113720	12	61	5.230769	no	no	no	no	no	no	no	no	no	no
182267251	chr10	9117614	9117667	20	54	2.666667	no	no	no	no	no	no	no	no	no	no
182267253	chr10	9117845	9117874	11	30	2.727273	no	no	no	no	no	no	no	no	no	no
182267256	chr10	9134010	9134052	20	43	2.150000	no	no	no	no	no	no	no	no	no	no
182267261	chr10	9140007	9140044	20	38	1.950000	no	no	no	no	no	no	no	no	no	no
182267266	chr10	9149067	9149100	16	34	2.187500	no	no	no	no	no	no	no	yes	no	no
182267268	chr10	9150253	9150295	14	43	3.071429	no	no	no	no	no	no	no	yes	no	no
182267272	chr10	9165386	9165432	21	47	2.238095	no	no	no	no	no	no	no	no	no	no
182267275	chr10	9196429	9196486	28	58	2.071429	no	no	no	no	no	no	no	yes	no	no
182267280	chr10	9199752	9199814	28	63	2.250000	no	no	no	no	no	no	no	no	no	no
182267283	chr10	9206808	9206863	27	56	2.074074	no	no	no	no	no	no	no	no	no	no
182267287	chr10	9215285	9215392	24	108	4.461538	no	no	no	no	no	no	no	no	no	no
182267296	chr10	9235411	9235440	15	30	2.000000	no	no	no	no	no	no	no	no	no	no
182267307	chr10	9246024	9246088	35	65	1.914286	no	no	no	no	no	no	no	yes	no	no
182267311	chr10	9257746	9257789	20	44	2.200000	no	no	no	no	no	no	no	yes	no	no
182267312	chr10	9260344	9260397	28	54	1.928571	no	no	no	no	no	no	no	no	no	no
182267333	chr10	9287648	9287732	45	85	1.955556	no	no	no	no	no	yes	no	yes	no	no
182267334	chr10	9289998	9290053	29	56	1.931034	no	no	no	no	no	no	no	yes	yes	no
182267336	chr10	9294958	9295020	21	63	3.000000	no	no	yes	no	no	yes	no	yes	yes	yes
182267337	chr10	9295950	9295989	20	40	2.050000	no	no	no	no	yes	yes	no	yes	no	yes

182267338	chr10	9302957	9302988	16	32	2.000000	no	no	yes	no	no	no	no	no	no	yes
182267340	chr10	9315483	9315520	18	38	2.111111	no	no	no	no	no	no	no	yes	no	no
182267360	chr10	9357672	9357701	15	30	2.000000	no	no	no	no	no	no	no	no	yes	no
182267362	chr10	9360110	9360145	16	36	2.250000	no	no	no	no	no	no	no	no	no	no
182267390	chr10	9392783	9392821	20	39	1.950000	no	no	no	no	no	no	no	no	no	no
182267391	chr10	9396210	9396438	56	229	4.089286	no	no	no	no	no	no	no	no	yes	no
182267400	chr10	9412435	9412475	21	41	1.952381	no	no	no	no	no	no	no	no	no	no
182267402	chr10	9422957	9422999	22	43	2.000000	no	no	no	no	no	no	no	no	no	no
182267403	chr10	9436458	9436499	19	42	2.210526	no	no	no	no	no	no	no	no	no	no
182267410	chr10	9471688	9471730	21	43	2.190476	no	no	no	no	no	no	no	no	no	no
182267414	chr10	9476924	9477003	39	80	2.051282	no	no	no	no	no	no	no	no	no	no
182267415	chr10	9477073	9477123	14	51	3.600000	no	no	no	no	no	no	no	no	no	no
182267420	chr10	9504605	9504683	18	79	4.388889	no	no	no	no	no	no	no	yes	no	no
182267421	chr10	9504911	9505061	32	151	4.718750	no	no	no	no	no	no	no	no	no	no
182267425	chr10	9507447	9507524	26	78	2.962963	no	no	no	no	no	no	no	yes	no	no
182267427	chr10	9509894	9509922	15	29	1.933333	no	no	no	no	no	no	no	no	no	no
182267430	chr10	9515726	9515776	27	51	1.962963	no	no	no	no	no	no	no	no	no	no
182267433	chr10	9515801	9515843	17	43	2.588235	no	no	no	no	no	no	no	no	no	no
182267434	chr10	9517838	9517935	24	98	4.333333	no	no	no	no	no	no	no	yes	yes	no
182267462	chr10	9581404	9581432	14	29	2.071429	no	no	no	no	no	no	no	yes	no	no
182267465	chr10	9585868	9585950	32	83	2.593750	no	no	no	no	no	no	no	no	no	no
182267498	chr10	9641760	9641831	32	72	2.250000	no	no	no	no	no	no	no	no	no	no
182267504	chr10	9654932	9655480	33	549	16.439024	no	no	no	no	no	no	no	no	no	no
182267512	chr10	9684879	9684923	18	45	2.500000	no	no	no	no	no	no	no	no	no	no
182267519	chr10	9704798	9704836	12	39	3.250000	no	no	no	no	no	no	no	no	no	no

182267526	chr10	9714652	9714690	20	39	1.950000	no	no	no	no	no	no	no	no	no	no
182267528	chr10	9721672	9721713	22	42	1.954545	no	no	no	no	no	yes	no	no	no	no
182267530	chr10	9734352	9734380	15	29	1.933333	no	no	no	no	no	no	no	no	yes	no
182267541	chr10	9748715	9748802	30	88	3.200000	no	no	no	no	no	no	no	no	no	no
182267543	chr10	9748809	9748834	12	26	2.166667	no	no	no	no	no	no	no	no	no	no
182267544	chr10	9749180	9749255	38	76	2.000000	no	no	no	no	no	no	no	no	no	no
182267559	chr10	9768052	9768092	18	41	2.277778	no	no	yes	no	no	no	no	yes	no	yes
182267566	chr10	9785944	9786069	64	126	2.000000	no	no	yes	no	no	no	no	yes	no	yes
182267567	chr10	9789730	9789772	20	43	2.200000	no	no	yes	no	no	no	no	no	no	yes
182267574	chr10	9797437	9797556	43	120	2.818182	no	no	yes	no	no	no	no	no	no	yes
182267577	chr10	9815162	9815394	130	233	1.869231	no	no	yes	no	no	no	no	no	no	yes
182267585	chr10	9828269	9828359	31	91	2.906250	no	no	yes	no	no	no	no	no	no	yes
182267586	chr10	9830777	9830821	22	45	2.045455	no	no	yes	no	no	no	no	no	no	yes
182267602	chr10	9862654	9862722	37	69	1.918919	no	no	yes	no	no	no	no	yes	no	yes
182267603	chr10	9865199	9865230	16	32	2.000000	no	no	yes	no	no	no	no	no	no	yes
182267608	chr10	9880366	9880705	167	340	2.083832	no	no	no	no	no	no	no	yes	no	no
182267613	chr10	9904281	9904316	12	36	3.083333	no	no	no	no	no	no	no	no	no	no
182267619	chr10	9932760	9932803	22	44	2.000000	no	no	no	no	no	no	no	no	no	no
182267629	chr10	9953158	9953195	19	38	2.000000	no	no	no	no	no	no	no	no	no	no
182267638	chr10	9981089	9981115	13	27	2.076923	no	no	no	no	no	no	no	no	yes	no
182267641	chr10	9988070	9988120	23	51	2.217391	no	no	no	no	no	no	no	no	no	no
182267646	chr10	10004878	10004902	12	25	2.083333	no	no	no	no	no	no	no	no	no	no
182267647	chr10	10005507	10005542	15	36	2.466667	no	no	no	no	no	no	no	no	no	no
182267651	chr10	10005598	10005671	34	74	2.235294	no	no	no	no	no	no	no	no	no	no
182267652	chr10	10006358	10006406	24	49	2.041667	no	no	no	no	no	no	no	no	no	no

182267655	chr10	10019285	10019366	34	82	2.470588	no	no	no	no	no	no	no	no	no	no
182267658	chr10	10023183	10023218	16	36	2.312500	no	no	no	no	no	no	no	no	no	no
182267666	chr10	10033374	10033529	52	156	2.964286	no	no	no	no	no	no	no	no	no	no
182267667	chr10	10033656	10033698	23	43	1.913043	no	no	no	no	no	no	no	no	no	no
182267672	chr10	10037236	10037311	23	76	3.200000	no	no	no	no	no	no	no	no	no	no
182267686	chr10	10090380	10090414	17	35	2.058824	no	no	no	no	no	no	no	no	no	no
182267687	chr10	10091049	10091095	17	47	2.722222	no	no	no	no	no	no	no	no	yes	no
182267701	chr10	10127419	10127460	21	42	2.000000	no	no	no	no	no	no	no	no	no	no
182267708	chr10	10134923	10134963	17	41	2.470588	no	no	no	no	no	no	no	no	no	no
182267711	chr10	10147913	10147966	21	54	2.571429	no	no	no	no	no	no	no	no	no	no
182267727	chr10	10166397	10166459	32	63	1.968750	no	no	no	no	no	no	no	no	no	no
182267728	chr10	10174109	10174152	14	44	3.142857	yes	no	no	no	no	no	no	no	no	no
182267733	chr10	10177835	10177933	26	99	3.925926	no	no	no	no	no	no	no	yes	no	no
182267741	chr10	10184191	10184233	21	43	2.095238	no	no	no	no	no	no	no	no	no	no
182267748	chr10	10193066	10193114	21	49	2.333333	no	no	no	no	no	no	no	yes	no	no
182267761	chr10	10224701	10224749	24	49	2.041667	no	no	no	no	no	no	no	no	no	no
182267762	chr10	10231900	10231951	27	52	1.925926	yes	no	no	no	no	no	no	no	no	no
182267768	chr10	10256798	10256873	29	76	2.793103	no	no	no	no	no	yes	no	no	no	no
182267774	chr10	10266133	10267327	30	1195	39.833332	no	no	no	no	no	no	yes	yes	no	no
182267777	chr10	10268456	10268486	16	31	1.937500	no	no	no	no	no	no	no	no	no	no
182267780	chr10	10279117	10279153	18	37	2.055556	no	no	no	no	no	no	no	no	no	no
182267795	chr10	10310470	10310501	16	32	2.000000	no	no	no	no	no	no	no	no	yes	no
182267798	chr10	10318200	10318238	20	39	1.950000	no	no	no	no	no	no	no	no	no	no
182267810	chr10	10347836	10347973	72	138	1.930556	no	no	no	no	no	no	no	no	no	no
182267820	chr10	10360142	10360180	20	39	1.950000	no	no	no	no	no	no	no	no	no	no

182267825	chr10	10366557	10366653	49	97	1.979592	yes	no	no	no	no	no	no	no	no	no
182267828	chr10	10376024	10376088	33	65	1.969697	no	no	yes	no	no	no	no	no	no	yes
182267836	chr10	10397263	10397303	21	41	1.952381	no	no	yes	no	no	no	no	no	no	yes
182267839	chr10	10404536	10404594	28	59	2.178571	no	no	yes	no	no	yes	no	no	no	yes
182267840	chr10	10404578	10404651	38	74	2.000000	no	no	yes	no	no	yes	no	no	no	yes
182267842	chr10	10409041	10409692	20	652	32.650002	no	no	yes	no	no	no	no	no	no	yes
182267847	chr10	10424140	10424177	18	38	2.111111	no	no	yes	no	no	no	no	no	no	yes
182267849	chr10	10441614	10441666	16	53	3.352941	no	no	yes	no	no	no	no	no	no	yes
182267858	chr10	10462146	10462176	14	31	2.214286	no	no	yes	no	no	no	no	no	no	yes
182267860	chr10	10462605	10462653	25	49	1.960000	yes	no	no	no	no	no	no	yes	no	no
182267862	chr10	10479500	10479553	23	54	2.347826	no	no	no	no	no	no	no	yes	yes	no
182267870	chr10	10490171	10490207	18	37	2.055556	no	no	no	no	no	no	no	no	yes	no
182267871	chr10	10491880	10491913	17	34	2.000000	no	no	no	no	no	no	no	no	no	no
182267878	chr10	10514613	10514638	12	26	2.166667	no	no	no	no	no	no	no	yes	yes	no
182267879	chr10	10517632	10517706	36	75	2.083333	no	no	no	no	no	no	no	no	yes	no
182267885	chr10	10545655	10545708	27	54	2.037037	no	no	no	no	no	no	no	no	no	no
182267889	chr10	10562782	10562829	22	48	2.181818	no	no	no	no	no	no	no	no	no	no
182267901	chr10	10594704	10594987	140	284	2.028571	no	no	no	no	no	no	no	no	no	no
182267930	chr10	10735250	10735336	39	87	2.225000	no	no	no	no	no	no	no	no	no	no
182267943	chr10	10765901	10765964	20	64	3.190476	no	no	no	no	no	no	no	no	no	no
182267944	chr10	10767208	10767244	12	37	3.083333	no	no	no	no	no	no	no	no	no	no
182267949	chr10	10773940	10773968	14	29	2.071429	no	no	no	no	no	no	no	no	no	no
182267952	chr10	10778332	10778360	12	29	2.416667	no	no	no	no	no	no	no	no	no	no
182267961	chr10	10794372	10794413	22	42	1.909091	no	no	yes	no	no	no	no	yes	no	yes
182267976	chr10	10865120	10865151	17	32	1.941176	no	no	yes	no	no	no	no	no	no	yes

182267980	chr10	10882180	10882229	24	50	2.125000	no	no	yes	no	no	no	no	yes	yes	yes
182267981	chr10	10882752	10882785	14	34	2.500000	no	no	yes	no	no	no	no	no	no	yes
182267984	chr10	10884859	10884888	14	30	2.142857	no	no	yes	no	no	no	no	no	no	yes
182267987	chr10	10890182	10890221	22	40	1.909091	no	no	yes	no	no	no	no	no	no	yes
182267989	chr10	10892143	10892181	19	39	2.052632	no	no	yes	no	no	no	no	yes	no	yes
182267997	chr10	10904724	10904789	28	66	2.357143	no	no	yes	no	no	no	no	no	no	yes
182268010	chr10	10913950	10913984	17	35	2.058824	no	no	yes	no	no	no	no	yes	no	yes
182268012	chr10	10920530	10920590	24	61	2.541667	no	no	yes	no	no	no	no	no	no	yes
182268023	chr10	10939076	10939263	62	188	3.032258	no	no	yes	no	no	no	no	no	no	yes
182268025	chr10	10953292	10953318	13	27	2.076923	no	no	yes	no	no	no	no	no	no	yes
182268028	chr10	10962071	10962141	37	71	2.000000	no	no	yes	no	no	no	no	no	no	yes
182268030	chr10	10962222	10962250	8	29	3.625000	no	no	yes	no	no	no	no	no	no	yes
182268040	chr10	10988967	10989000	17	34	2.000000	no	no	yes	no	no	no	no	no	no	yes
182268050	chr10	11030372	11030643	34	272	8.000000	no	no	yes	no	yes	no	no	no	yes	yes
182268053	chr10	11034351	11034547	96	197	2.062500	no	no	yes	no	no	no	no	no	no	yes
182268055	chr10	11040899	11040934	18	36	2.000000	no	no	yes	no	no	no	no	no	no	yes
182268056	chr10	11042412	11042480	32	69	2.187500	no	no	yes	no	no	no	no	no	yes	yes
182268058	chr10	11049959	11050001	20	43	2.150000	no	no	yes	no	no	no	no	yes	yes	yes
182268067	chr10	11104200	11104239	20	40	2.000000	no	no	yes	no	no	no	no	no	no	yes
182268069	chr10	11107746	11107787	22	42	1.909091	no	no	yes	no	no	no	no	yes	no	yes
182268070	chr10	11107794	11107844	19	51	2.684211	no	no	yes	no	no	no	no	yes	no	yes
182268071	chr10	11107856	11107931	17	76	4.388889	no	no	yes	no	no	no	no	no	no	yes
182268076	chr10	11112805	11113060	65	256	3.938462	no	no	yes	no	no	no	no	yes	yes	yes
182268077	chr10	11116344	11116412	36	69	1.944444	no	no	yes	no	no	no	no	no	no	yes
182268081	chr10	11142857	11143131	44	275	6.244444	no	no	yes	no	no	no	no	yes	yes	yes

182268082	chr10	11148162	11148241	41	80	1.951220	no	no	yes	no	no	no	no	yes	yes	yes
182268084	chr10	11162140	11162326	44	187	4.250000	no	no	yes	no	no	no	no	yes	no	yes
182268085	chr10	11168349	11168380	15	32	2.133333	no	no	yes	no	no	yes	no	yes	yes	yes
182268086	chr10	11173052	11173088	13	37	2.846154	no	no	yes	no	no	no	no	yes	yes	yes
182268090	chr10	11195517	11195545	14	29	2.071429	no	no	yes	no	no	no	no	yes	no	yes
182268096	chr10	11232628	11232662	14	35	2.571429	no	no	yes	no	no	no	no	yes	no	yes
182268097	chr10	11232717	11232794	24	78	3.250000	no	no	yes	no	no	no	no	yes	no	yes
182268099	chr10	11235123	11235187	26	65	2.615385	no	no	yes	no	no	no	no	yes	yes	yes
182268103	chr10	11254877	11254944	20	68	3.400000	no	no	yes	no	no	no	no	yes	no	yes
182268104	chr10	11255010	11255055	23	46	2.000000	no	no	yes	no	no	no	no	no	no	yes
182268111	chr10	11282149	11282182	14	34	2.428571	no	no	yes	no	no	no	no	yes	yes	yes
182268120	chr10	11283840	11284544	26	705	27.678572	no	no	yes	no	no	no	no	yes	yes	yes
182268124	chr10	11284726	11284944	120	219	1.858333	no	no	yes	no	no	no	no	yes	no	yes
182268131	chr10	11301431	11301464	18	34	1.944444	no	no	yes	no	no	no	yes	no	yes	yes
182268135	chr10	11301494	11301543	22	50	2.260870	no	no	yes	no	no	no	yes	no	yes	yes
182268138	chr10	11323066	11323095	15	30	2.000000	no	no	yes	no	no	no	no	yes	no	yes
182268140	chr10	11344879	11344907	13	29	2.230769	no	no	no	no	no	no	no	yes	yes	no
182268143	chr10	11355190	11355256	21	67	3.190476	no	no	no	no	no	no	no	yes	no	no
182268145	chr10	11365234	11367067	36	1834	50.945946	no	no	no	no	no	no	no	yes	yes	no
182268146	chr10	11369301	11369757	38	457	12.131579	no	no	no	no	no	no	no	no	yes	no
182268157	chr10	11400840	11400875	18	36	2.000000	no	no	no	no	no	no	no	yes	yes	no
182268159	chr10	11405374	11405409	17	36	2.117647	no	no	no	no	no	no	no	no	no	no
182268160	chr10	11405834	11405863	14	30	2.214286	no	no	no	no	no	no	no	no	no	no
182268167	chr10	11433720	11433788	32	69	2.156250	no	no	no	no	no	no	no	yes	yes	no
182268169	chr10	11437005	11437038	17	34	2.058824	no	no	no	no	no	no	no	no	no	no

182268172	chr10	11439284	11439326	21	43	2.047619	no	no	no	no	no	no	no	no	no	no
182268173	chr10	11439990	11440038	23	49	2.130435	no	no	no	no	no	no	no	no	no	no
182268174	chr10	11444690	11444806	61	117	1.934426	no	no	no	no	no	no	no	no	yes	no
182268181	chr10	11463954	11463992	15	39	2.600000	no	no	yes	no	no	no	no	no	no	yes
182268182	chr10	11463983	11464023	22	41	1.863636	no	no	yes	no	no	no	no	no	no	yes
182268187	chr10	11478502	11478547	24	46	1.916667	no	no	yes	no	no	no	no	no	no	yes
182268195	chr10	11496751	11496828	25	78	3.120000	no	no	yes	no	no	no	no	no	no	yes
182268208	chr10	11531802	11531840	19	39	2.052632	no	no	yes	no	no	no	no	no	no	yes
182268211	chr10	11543079	11543123	23	45	2.000000	no	no	yes	no	no	no	no	yes	no	yes
182268214	chr10	11547913	11547967	14	55	4.000000	no	no	yes	no	no	no	no	yes	no	yes
182268220	chr10	11558139	11558178	21	40	1.904762	no	no	yes	no	no	no	no	yes	no	yes
182268224	chr10	11577938	11577971	17	34	2.000000	no	no	yes	no	no	no	no	no	no	yes
182268232	chr10	11611705	11611745	20	41	2.150000	yes	yes	no	no	yes	no	yes	yes	yes	yes
182268262	chr10	11704660	11704715	29	56	1.965517	no	no	no	no	no	no	no	no	no	no
182268268	chr10	11716944	11716986	22	43	1.954545	no	no	no	no	no	no	no	yes	yes	no
182268270	chr10	11721179	11721224	10	46	4.500000	no	no	no	no	no	no	no	yes	no	no
182268274	chr10	11742312	11742382	20	71	3.550000	yes	yes	no	no	yes	no	yes	yes	yes	yes
182268275	chr10	11742460	11742484	10	25	2.500000	no	yes	no	no	yes	no	yes	yes	yes	yes
182268281	chr10	11769792	11769884	25	93	3.720000	no	no	no	no	no	no	no	no	no	no
182268282	chr10	11770249	11770283	19	35	1.947368	no	no	no	no	no	no	no	no	yes	no
182268298	chr10	11804948	11805027	39	80	2.051282	no	no	no	no	no	no	no	no	no	no
182268301	chr10	11821246	11821302	30	57	1.933333	no	no	no	no	no	no	no	yes	no	no
182268324	chr10	11882509	11882555	25	47	1.960000	no	no	yes	no	no	no	no	yes	no	yes
182268326	chr10	11885226	11885659	39	434	11.179487	no	no	yes	no	no	no	no	yes	yes	yes
182268328	chr10	11886796	11886828	15	33	2.200000	no	no	yes	no	no	no	no	yes	yes	yes

182268329	chr10	11887734	11887780	20	47	2.350000	no	no	yes	no	no	no	no	yes	yes	yes
182268334	chr10	11894875	11894973	47	99	2.127660	yes	no	no	no	no	no	yes	yes	yes	no
182268336	chr10	11902969	11903002	16	34	2.125000	no	no	no	no	no	no	no	no	no	no
182268343	chr10	11920340	11920382	17	43	2.529412	no	yes	no	no	no	no	no	no	no	yes
182268347	chr10	11926935	11926986	26	52	2.000000	no	no	yes	no	no	no	no	no	no	yes
182268348	chr10	11934942	11935005	24	64	2.666667	no	no	yes	no	no	no	no	yes	yes	yes
182268361	chr10	11971214	11971252	21	39	1.952381	no	no	yes	no	no	no	no	no	no	yes
182268362	chr10	11985119	11985152	16	34	2.187500	no	no	yes	no	no	no	no	no	no	yes
182268363	chr10	11985722	11985763	20	42	2.100000	no	no	yes	no	no	no	no	no	no	yes
182268379	chr10	12042879	12042908	16	30	1.937500	yes	no	yes	no	no	no	yes	yes	yes	yes
182268411	chr10	12075870	12075911	13	42	3.307692	no	no	yes	no	no	no	no	no	no	yes
182268415	chr10	12085560	12085594	12	35	3.000000	no	no	yes	no	no	no	no	no	no	yes
182268417	chr10	12086176	12086208	11	33	3.000000	no	no	yes	no	no	no	no	no	no	yes
182268438	chr10	12124693	12124719	13	27	2.076923	no	no	no	no	no	no	no	no	no	no
182268440	chr10	12129643	12129682	21	40	1.904762	yes	no	no	no	yes	no	yes	yes	yes	yes
182268442	chr10	12132850	12132986	69	137	1.985507	no	no	yes	no	no	no	no	no	no	yes
182268443	chr10	12133901	12133941	19	41	2.157895	no	no	yes	no	no	no	no	no	no	yes
182268445	chr10	12135880	12135916	18	37	2.055556	yes	no	yes	no	no	no	no	no	yes	yes
182268458	chr10	12170036	12170102	23	67	3.000000	no	yes	yes	no	no	yes	no	no	no	yes
182268462	chr10	12195584	12195627	18	44	2.421053	yes	no	yes	no	no	no	no	yes	yes	yes
182268474	chr10	12224275	12224311	17	37	2.176471	no	no	yes	no	no	no	no	no	no	yes
182268490	chr10	12244065	12244092	13	28	2.153846	no	no	yes	no	no	yes	no	yes	yes	yes
182268492	chr10	12246846	12247542	34	697	20.487179	yes	no	yes	no	yes	no	no	yes	yes	yes
182268498	chr10	12264506	12264538	16	33	2.062500	no	no	no	no	no	no	no	yes	yes	no
182268549	chr10	12349458	12349506	9	49	5.400000	yes	no	no	no	yes	no	yes	yes	yes	yes

182268551	chr10	12349498	12349542	23	45	2.000000	yes	no	no	no	yes	no	yes	yes	yes	yes
182268552	chr10	12349749	12349809	25	61	2.440000	no	yes	no	no	yes	no	yes	yes	yes	yes
182268578	chr10	12401020	12401344	44	325	7.488889	no	no	yes	no	no	no	no	no	no	yes
182268583	chr10	12410704	12410746	20	43	2.150000	no	no	yes	no	no	no	no	no	no	yes
182268588	chr10	12416158	12416193	18	36	2.000000	no	no	yes	no	no	yes	no	yes	no	yes
182268589	chr10	12418886	12418942	30	57	1.900000	no	no	yes	no	no	no	no	no	yes	yes
182268598	chr10	12432530	12432715	73	186	2.554054	no	no	yes	no	no	no	no	yes	no	yes
182268618	chr10	12471236	12471272	17	37	2.235294	no	no	yes	no	no	no	no	yes	yes	yes
182268623	chr10	12495086	12495129	15	44	2.933333	no	no	yes	no	no	no	no	no	no	yes
182268626	chr10	12502032	12502073	16	42	2.625000	no	no	yes	no	no	no	no	yes	yes	yes
182268628	chr10	12503111	12503142	16	32	2.000000	no	no	yes	no	no	no	no	yes	no	yes
182268629	chr10	12503174	12503213	20	40	2.100000	no	no	yes	no	no	no	no	yes	no	yes
182268632	chr10	12504740	12504805	34	66	1.941176	no	no	yes	no	no	no	no	no	no	yes
182268667	chr10	12536907	12536939	15	33	2.200000	no	no	yes	no	no	no	no	no	no	yes
182268670	chr10	12544699	12544918	20	220	11.000000	no	no	yes	no	no	no	no	yes	no	yes
182268673	chr10	12554113	12554166	27	54	2.000000	no	no	yes	no	no	no	no	no	no	yes
182268676	chr10	12557657	12557759	40	103	2.575000	no	no	yes	no	no	no	no	no	no	yes
182268680	chr10	12564072	12564097	12	26	2.166667	no	yes	yes	no	no	no	no	yes	no	yes
182268686	chr10	12568202	12568233	14	32	2.285714	no	no	yes	no	no	yes	no	no	no	yes
182268694	chr10	12573321	12573347	12	27	2.250000	no	no	yes	no	no	no	no	no	no	yes
182268695	chr10	12573599	12573639	19	41	2.263158	no	no	yes	no	no	no	no	no	no	yes
182268712	chr10	12615484	12615582	44	99	2.318182	no	no	yes	no	no	no	no	yes	yes	yes
182268721	chr10	12616104	12616141	14	38	2.714286	no	no	yes	no	no	no	no	yes	no	yes
182268736	chr10	12655622	12655652	14	31	2.214286	no	no	yes	no	no	no	no	no	no	yes
182268743	chr10	12676952	12676990	20	39	1.950000	no	no	yes	no	no	no	no	no	no	yes

182268748	chr10	12687556	12687615	27	60	2.222222	no	no	yes	no	no	no	no	no	no	yes
182268761	chr10	12721879	12721916	17	38	2.294118	no	no	yes	no	no	no	no	no	no	yes
182268774	chr10	12749526	12749578	27	53	1.962963	no	no	yes	no	no	no	no	yes	no	yes
182268779	chr10	12775000	12776122	33	1123	34.060608	no	no	yes	no	no	no	no	no	no	yes
182268784	chr10	12790055	12790130	37	76	2.054054	no	no	yes	no	no	no	no	no	no	yes
182268798	chr10	12820731	12820780	25	50	2.000000	no	no	yes	no	no	no	no	yes	yes	yes
182268799	chr10	12824953	12824998	24	46	1.916667	no	no	yes	no	no	no	no	no	no	yes
182268800	chr10	12827105	12827147	22	43	2.000000	no	no	yes	no	no	yes	no	yes	no	yes
182268823	chr10	12889013	12889043	8	31	3.875000	no	no	yes	no	no	no	no	no	no	yes
182268826	chr10	12891825	12891859	17	35	2.058824	no	no	yes	no	no	no	no	no	no	yes
182268827	chr10	12894205	12894255	17	51	3.000000	no	no	yes	no	no	no	no	no	no	yes
182268835	chr10	12894422	12894458	14	37	2.642857	no	no	yes	no	no	no	no	no	no	yes
182268839	chr10	12939621	12939694	37	74	2.000000	no	no	yes	no	no	no	no	no	yes	yes
182268844	chr10	12966172	12966278	36	107	2.972222	no	no	yes	no	no	yes	no	no	no	yes
182268849	chr10	12969667	12969726	31	60	2.032258	no	no	yes	no	no	no	no	no	no	yes
182268851	chr10	12971225	12971288	30	64	2.133333	no	no	yes	no	no	no	no	no	yes	yes
182268852	chr10	12973664	12973812	50	149	2.980000	no	no	yes	no	no	no	no	yes	no	yes
182268861	chr10	12994426	12994450	7	25	3.571429	no	no	yes	no	no	no	no	no	no	yes
182268862	chr10	12996729	12996757	12	29	2.416667	no	no	yes	no	no	no	no	no	no	yes
182268863	chr10	13013068	13013103	19	36	1.947368	no	no	yes	no	no	no	no	no	no	yes
182268894	chr10	13089221	13089314	28	94	3.500000	no	no	yes	no	no	no	no	yes	no	yes
182268901	chr10	13099554	13099609	29	56	1.931034	yes	no	no	no	yes	no	no	yes	yes	yes
182268903	chr10	13100038	13100073	16	36	2.250000	yes	no	yes	no	no	no	yes	yes	yes	yes
182268908	chr10	13113673	13113708	17	36	2.176471	no	no	yes	no	no	no	no	yes	yes	yes
182268912	chr10	13123200	13123228	15	29	1.933333	no	no	yes	no	no	yes	no	no	no	yes

182268915	chr10	13131712	13131752	21	41	2.000000	no	no	yes	no	no	no	no	no	no	yes
182268927	chr10	13156428	13156468	18	41	2.277778	no	no	no	no	no	yes	no	no	no	no
182268937	chr10	13172806	13172845	18	40	2.222222	no	no	yes	no	no	yes	no	no	no	yes
182268940	chr10	13180871	13180911	22	41	1.954545	no	no	yes	no	no	no	no	yes	no	yes
182268949	chr10	13218868	13218898	16	31	2.000000	no	no	no	no	no	no	no	no	no	no
182268950	chr10	13219284	13219322	18	39	2.166667	no	no	no	no	no	no	no	no	no	no
182268973	chr10	13255166	13255206	20	41	2.050000	no	no	no	no	no	no	no	yes	no	no
182268975	chr10	13255668	13255700	16	33	2.062500	no	no	no	no	no	no	no	no	no	no
182268983	chr10	13271909	13271972	26	64	2.461539	no	no	no	no	no	no	no	yes	yes	no
182268987	chr10	13283602	13283638	15	37	2.533333	no	no	yes	no	no	yes	no	yes	no	yes
182268989	chr10	13287016	13287047	16	32	2.062500	no	no	yes	no	no	no	no	no	no	yes
182269004	chr10	13299490	13299521	15	32	2.133333	no	no	yes	no	yes	no	yes	yes	yes	yes
182269023	chr10	13347491	13347519	10	29	2.900000	yes	no	yes	no	no	no	yes	yes	yes	yes
182269024	chr10	13347728	13347773	23	46	2.130435	yes	no	yes	no	no	no	yes	yes	yes	yes
182269055	chr10	13393163	13393194	15	32	2.133333	no	no	yes	no	no	no	no	no	no	yes
182269059	chr10	13411503	13411544	22	42	1.954545	no	no	no	no	no	no	no	no	no	no
182269074	chr10	13478729	13478757	15	29	1.933333	no	no	yes	no	no	no	no	no	no	yes
182269079	chr10	13500557	13500657	49	101	2.061224	no	no	yes	no	yes	no	no	yes	no	yes
182269082	chr10	13503788	13503819	12	32	2.666667	yes	no	yes	no	no	no	no	yes	no	yes
182269085	chr10	13528546	13528617	15	72	4.866667	no	yes	no	no	yes	no	yes	yes	yes	yes
182269110	chr10	13655737	13655789	18	53	3.000000	no	yes	yes	no	no	yes	no	no	no	yes
182269111	chr10	13657134	13657174	9	41	4.400000	yes	no	yes	yes	no	no	yes	yes	yes	yes
182269115	chr10	13674804	13674830	14	27	1.928571	yes	no	yes	no	no	yes	no	yes	no	yes
182269124	chr10	13686254	13686293	21	40	2.000000	no	no	yes	no	no	no	no	yes	no	yes
182269126	chr10	13693800	13693829	16	30	1.937500	no	no	yes	no	yes	yes	no	yes	yes	yes

182269133	chr10	13712148	13712196	16	49	3.058824	no	no	yes	no	no	yes	no	no	yes	yes
182269138	chr10	13726010	13726047	19	38	2.000000	no	no	yes	no	no	no	no	yes	no	yes
182269148	chr10	13761000	13761029	11	30	2.727273	no	no	yes	no	no	no	no	yes	no	yes
182269153	chr10	13766201	13766253	25	53	2.200000	no	no	yes	no	no	no	no	yes	no	yes
182269181	chr10	13822870	13822901	15	32	2.133333	no	no	yes	no	no	no	no	yes	no	yes
182269197	chr10	13862924	13862965	17	42	2.470588	no	no	yes	no	no	no	no	no	yes	yes
182269211	chr10	13868211	13868271	30	61	2.066667	no	no	yes	no	no	no	no	no	no	yes
182269212	chr10	13876889	13876941	29	53	1.931034	no	no	yes	no	no	no	no	no	no	yes
182269231	chr10	13913701	13913740	18	40	2.222222	no	no	yes	no	no	no	no	no	no	yes
182269232	chr10	13918422	13918451	12	30	2.500000	no	no	yes	no	no	no	no	no	no	yes
182269233	chr10	13919547	13919591	21	45	2.238095	no	no	yes	no	no	no	no	no	no	yes
182269244	chr10	13943311	13943345	18	35	1.944444	no	no	yes	no	no	no	no	no	no	yes
182269251	chr10	13958898	13958935	19	38	2.000000	no	no	yes	no	no	no	no	no	no	yes
182269264	chr10	13973768	13973799	16	32	2.000000	no	no	yes	no	no	no	no	yes	no	yes
182269269	chr10	13975547	13975670	58	124	2.135593	no	no	yes	no	no	no	no	yes	no	yes
182269277	chr10	13988635	13988672	14	38	2.714286	no	no	yes	no	no	no	no	no	no	yes
182269289	chr10	14016243	14016305	30	63	2.100000	no	no	yes	no	no	no	no	yes	yes	yes
182269300	chr10	14035705	14035752	14	48	3.500000	no	no	yes	no	no	no	no	yes	yes	yes
182269312	chr10	14045936	14046034	47	99	2.170213	no	no	yes	no	no	no	no	no	no	yes
182269321	chr10	14061091	14061163	34	73	2.147059	no	no	yes	no	no	no	no	no	no	yes
182269325	chr10	14068856	14068936	30	81	2.700000	no	no	yes	no	no	no	no	yes	no	yes
182269339	chr10	14097648	14097709	25	62	2.600000	no	no	yes	no	no	no	no	no	no	yes
182269345	chr10	14097940	14098172	34	233	6.511111	no	no	yes	no	no	no	no	no	no	yes
182269346	chr10	14100148	14100180	15	33	2.200000	no	no	yes	no	no	no	no	yes	yes	yes
182269356	chr10	14112429	14112469	21	41	2.047619	no	no	yes	no	no	no	no	no	no	yes

182269359	chr10	14123247	14123284	19	38	2.000000	no	no	yes	no	no	no	no	no	yes	yes
182269365	chr10	14134406	14134495	39	90	2.307692	no	no	yes	no	no	no	no	yes	no	yes
182269367	chr10	14141447	14141498	20	52	2.650000	no	no	yes	no	no	no	no	yes	no	yes
182269368	chr10	14144316	14144347	15	32	2.133333	no	no	yes	no	no	no	no	no	no	yes
182269370	chr10	14145592	14145624	14	33	2.357143	no	no	yes	no	no	no	no	no	no	yes
182269374	chr10	14157840	14157868	15	29	1.933333	no	no	yes	no	no	no	no	yes	no	yes
182269379	chr10	14162631	14162670	19	40	2.105263	no	no	yes	no	no	no	no	yes	no	yes
182269411	chr10	14216210	14216250	21	41	1.952381	no	no	yes	no	no	no	no	no	no	yes
182269427	chr10	14246381	14246523	74	143	1.932432	no	no	yes	no	no	no	no	yes	no	yes
182269431	chr10	14247498	14247553	22	56	2.545455	no	no	yes	no	no	no	no	no	yes	yes
182269435	chr10	14271958	14272003	16	46	2.823529	no	no	yes	no	no	no	no	no	no	yes
182269438	chr10	14285947	14285993	24	47	1.958333	no	no	yes	no	no	no	no	no	no	yes
182269440	chr10	14317680	14317715	15	36	2.400000	no	no	yes	no	no	no	no	no	no	yes
182269442	chr10	14318734	14318765	15	32	2.133333	no	no	yes	no	no	no	no	no	no	yes
182269455	chr10	14346200	14346243	22	44	2.000000	no	no	yes	no	no	no	no	no	no	yes
182269457	chr10	14347815	14347866	26	52	2.076923	no	no	yes	no	no	no	no	no	no	yes
182269478	chr10	14409879	14410010	56	132	2.428571	no	no	yes	no	no	no	no	yes	yes	yes
182269479	chr10	14410016	14410062	24	47	1.958333	no	no	yes	no	no	no	no	yes	yes	yes
182269482	chr10	14410275	14410343	33	69	2.181818	no	no	yes	no	no	no	no	yes	no	yes
182269502	chr10	14448789	14448818	13	30	2.307692	no	no	yes	no	no	no	no	no	yes	yes
182269506	chr10	14449157	14449469	148	313	2.128378	no	no	yes	no	no	no	no	yes	no	yes
182269514	chr10	14451906	14451941	17	36	2.117647	no	no	yes	no	no	no	no	no	no	yes
182269536	chr10	14503441	14503480	16	40	2.500000	no	no	no	no	no	no	no	no	yes	no
182269538	chr10	14505260	14505327	33	68	2.060606	no	no	no	no	no	no	no	no	no	no
182269543	chr10	14533511	14533568	23	58	2.521739	yes	no	yes	no	no	no	no	yes	yes	yes

182269549	chr10	14558264	14558314	18	51	2.833333	no	no	yes	no	no	no	no	yes	yes	yes
182269552	chr10	14568310	14568728	56	419	7.642857	no	no	yes	no	no	no	no	yes	yes	yes
182269554	chr10	14568778	14568821	21	44	2.095238	no	no	yes	no	no	no	no	yes	no	yes
182269557	chr10	14585405	14585437	18	33	1.944444	no	no	yes	no	no	no	no	yes	yes	yes
182269569	chr10	14606951	14607007	30	57	1.933333	no	no	yes	no	no	no	no	yes	yes	yes
182269570	chr10	14607752	14607798	24	47	2.000000	no	no	yes	no	no	no	no	yes	yes	yes
182269572	chr10	14613443	14613479	20	37	1.950000	yes	no	yes	no	no	no	no	yes	yes	yes
182269575	chr10	14627399	14627441	21	43	2.047619	no	no	yes	no	no	no	no	no	no	yes
182269584	chr10	14654533	14654577	23	45	2.086957	no	no	yes	no	no	no	no	yes	yes	yes
182269585	chr10	14658398	14658426	11	29	2.636364	no	no	yes	no	no	no	no	yes	yes	yes
182269590	chr10	14672027	14672053	13	27	2.076923	no	no	yes	no	no	no	no	no	no	yes
182269599	chr10	14688439	14688479	18	41	2.388889	no	no	yes	no	no	no	no	yes	yes	yes
182269620	chr10	14723033	14723076	13	44	3.384615	no	no	yes	no	no	yes	no	no	no	yes
182269621	chr10	14724135	14724164	15	30	2.000000	yes	no	yes	no	no	no	no	no	no	yes
182269634	chr10	14732970	14733031	27	62	2.296296	no	no	yes	no	no	no	no	no	no	yes
182269635	chr10	14733013	14733065	20	53	2.800000	no	no	yes	no	no	no	no	no	no	yes
182269638	chr10	14738664	14738771	55	108	1.981818	no	no	yes	no	no	no	no	no	no	yes
182269647	chr10	14765885	14765921	15	37	2.466667	no	no	yes	no	no	no	no	no	no	yes
182269651	chr10	14775282	14775320	20	39	1.950000	yes	no	no	no	no	no	no	yes	no	no
182269656	chr10	14792484	14792508	13	25	1.923077	no	no	no	no	no	no	no	no	no	
182269665	chr10	14825648	14825702	28	55	2.000000	no	no	yes	no	no	no	no	no	no	yes
182269678	chr10	14867288	14867346	29	59	2.034483	no	no	yes	yes	no	no	no	no	no	yes
182269681	chr10	14870718	14870750	17	33	2.000000	no	no	yes	no	no	no	no	no	no	yes
182269683	chr10	14879581	14879611	15	31	2.066667	yes	no	yes	no	no	no	yes	yes	yes	yes
182269704	chr10	14927579	14927636	25	58	2.320000	no	no	yes	no	no	yes	no	yes	yes	yes

182269712	chr10	14942537	14942575	18	39	2.166667	no	no	yes	no	no	no	no	yes	no	yes
182269783	chr10	15024550	15024596	20	47	2.350000	no	no	no	no	no	no	no	no	yes	no
182269807	chr10	15078827	15078863	19	37	2.052632	no	no	yes	no	no	no	no	no	no	yes
182269814	chr10	15089121	15089163	22	43	1.954545	yes	no	no	no	no	no	no	yes	no	no
182269816	chr10	15089666	15089704	16	39	2.437500	yes	no	no	no	no	no	no	no	no	no
182269818	chr10	15091616	15091650	16	35	2.187500	no	no	no	no	no	no	no	yes	no	no
182269822	chr10	15092837	15092873	19	37	2.000000	no	no	no	no	no	yes	no	yes	no	no
182269831	chr10	15125655	15125685	12	31	2.583333	no	no	yes	no	no	no	no	no	no	yes
182269842	chr10	15131759	15131801	21	43	2.047619	no	no	yes	no	no	no	no	no	no	yes
182269846	chr10	15136224	15136291	20	68	3.304348	no	no	yes	no	no	no	no	yes	no	yes
182269857	chr10	15136607	15136636	9	30	3.333333	no	no	yes	no	no	no	no	yes	no	yes
182269862	chr10	15143854	15143885	12	32	2.750000	no	no	yes	no	no	no	no	no	no	yes
182269867	chr10	15150883	15150966	38	84	2.236842	no	no	yes	no	no	no	no	yes	no	yes
182269869	chr10	15154994	15155055	30	62	2.066667	no	no	yes	no	yes	no	no	no	no	yes
182269879	chr10	15168275	15168323	25	49	2.000000	no	no	yes	no	no	no	yes	yes	yes	yes
182269882	chr10	15168721	15168794	23	74	3.375000	yes	no	no	no	no	no	no	yes	yes	no
182269906	chr10	15201424	15201455	17	32	1.941176	no	no	no	no	no	no	no	no	no	no
182269917	chr10	15243623	15243655	14	33	2.428571	no	no	yes	no	no	no	no	no	no	yes
182269919	chr10	15246086	15246230	72	145	2.013889	no	no	yes	no	no	no	no	yes	no	yes
182269926	chr10	15264814	15264907	31	94	2.969697	no	no	yes	no	no	no	no	no	no	yes
182269936	chr10	15298008	15298045	19	38	2.000000	no	no	yes	no	no	no	no	no	no	yes
182269942	chr10	15303227	15303281	29	55	1.931034	no	no	yes	no	no	no	no	no	no	yes
182269945	chr10	15318224	15318260	18	37	2.055556	no	no	yes	no	no	no	no	yes	no	yes
182269947	chr10	15326196	15326220	13	25	1.923077	no	no	yes	no	no	no	no	yes	yes	yes
182269961	chr10	15353834	15353872	19	39	2.105263	no	no	yes	no	no	no	no	yes	no	yes

182269963	chr10	15356198	15356261	26	64	2.461539	no	no	yes	no	no	no	no	no	no	yes
182269976	chr10	15376841	15376878	20	38	1.900000	no	no	no	no	no	no	no	no	no	no
182269994	chr10	15417859	15417898	19	40	2.157895	no	no	no	no	no	no	no	no	no	no
182270010	chr10	15436640	15436685	24	46	1.958333	no	no	no	no	no	no	no	yes	no	no
182270013	chr10	15437800	15437860	26	61	2.346154	no	no	no	no	no	no	no	no	no	no
182270031	chr10	15491821	15491866	19	46	2.421053	no	no	no	no	no	no	no	yes	no	no
182270045	chr10	15531302	15531344	20	43	2.150000	no	no	yes	no	no	no	no	no	no	yes
182270047	chr10	15531376	15531420	16	45	2.812500	no	no	yes	no	no	no	no	no	no	yes
182270071	chr10	15574770	15574822	27	53	1.962963	no	no	yes	no	no	no	no	no	no	yes
182270079	chr10	15600330	15600392	17	63	3.823529	no	no	yes	no	no	no	no	no	no	yes
182270081	chr10	15612831	15612856	13	26	2.000000	no	no	yes	no	no	no	no	no	no	yes
182270083	chr10	15614157	15614243	45	87	1.933333	no	no	yes	no	no	no	no	no	no	yes
182270084	chr10	15614224	15614264	22	41	1.954545	no	no	yes	no	no	no	no	no	no	yes
182270093	chr10	15630321	15630356	14	36	2.714286	no	no	yes	no	no	no	no	yes	no	yes
182270096	chr10	15640786	15640826	21	41	2.047619	no	no	yes	no	no	no	no	no	yes	yes
182270099	chr10	15648497	15648537	15	41	2.800000	no	no	yes	no	no	no	no	no	no	yes
182270102	chr10	15661505	15661534	10	30	3.000000	no	no	yes	no	no	no	no	yes	yes	yes
182270104	chr10	15663285	15663319	14	35	2.466667	no	no	yes	no	no	no	no	no	no	yes
182270105	chr10	15663308	15663385	41	78	1.902439	no	no	yes	no	no	no	no	no	no	yes
182270108	chr10	15678183	15678211	13	29	2.307692	no	no	yes	no	no	no	no	no	no	yes
182270111	chr10	15685867	15685924	30	58	1.933333	no	no	yes	no	no	no	no	no	no	yes
182270114	chr10	15693940	15693968	14	29	2.142857	no	no	yes	no	no	no	no	no	no	yes
182270125	chr10	15694652	15694716	30	65	2.200000	no	no	yes	no	no	no	no	no	no	yes
182270127	chr10	15704387	15704417	16	31	1.937500	no	no	yes	no	no	no	no	yes	no	yes
182270129	chr10	15712814	15712860	22	47	2.181818	no	no	yes	no	no	no	no	no	no	yes

182270140	chr10	15743593	15743634	21	42	2.047619	no	no	no	no	no	no	no	no	yes	no
182270141	chr10	15748009	15748125	38	117	3.078947	no	no	no	no	no	no	no	yes	no	no
182270142	chr10	15748539	15748580	15	42	2.800000	no	no	no	no	no	no	no	yes	no	no
182270156	chr10	15762255	15762323	29	69	2.379310	no	no	no	no	no	no	no	no	no	no
182270169	chr10	15769706	15769734	15	29	1.933333	no	no	no	no	no	no	no	no	no	no
182270175	chr10	15777828	15777852	12	25	2.083333	no	no	no	no	no	yes	no	yes	no	no
182270178	chr10	15787317	15787347	10	31	3.100000	no	no	yes	no	no	no	no	no	no	yes
182270179	chr10	15790255	15790294	19	40	2.105263	no	no	yes	no	no	no	no	yes	yes	yes
182270193	chr10	15820579	15820614	16	36	2.250000	no	no	yes	no	no	no	no	no	no	yes
182270196	chr10	15834909	15834951	20	43	2.150000	no	no	yes	no	no	no	no	no	no	yes
182270199	chr10	15856497	15856536	18	40	2.222222	no	no	yes	no	no	no	no	yes	no	yes
182270208	chr10	15880875	15880925	24	51	2.166667	no	no	no	no	no	no	no	no	no	no
182270234	chr10	15934427	15934463	19	37	1.947368	no	no	no	no	no	no	no	no	no	no
182270239	chr10	15941210	15941240	16	31	1.937500	no	no	no	no	no	no	no	no	no	no
182270242	chr10	15941265	15941477	30	213	7.433333	no	no	no	no	no	no	no	no	no	no
182270260	chr10	16006980	16007026	18	47	2.611111	no	no	no	no	no	no	no	no	no	no
182270261	chr10	16007170	16007241	35	72	2.057143	no	no	no	no	no	no	no	no	no	no
182270263	chr10	16011112	16011259	78	148	1.923077	no	no	no	no	no	no	no	no	no	no
182270273	chr10	16027414	16027462	23	49	2.130435	no	no	no	no	no	no	no	no	no	no
182270280	chr10	16054235	16054279	20	45	2.250000	no	no	no	no	no	no	no	no	no	no
182270289	chr10	16077889	16077917	14	29	2.071429	no	no	no	no	no	no	no	no	no	no
182270297	chr10	16093311	16093349	15	39	2.666667	no	no	no	no	no	no	no	no	no	no
182270311	chr10	16136014	16136058	25	45	1.920000	no	no	no	no	no	no	no	no	no	no
182270312	chr10	16144054	16144090	15	37	2.466667	no	no	no	no	no	no	no	no	no	no
182270316	chr10	16156755	16156826	38	72	1.921053	no	no	no	no	no	no	no	no	no	no

182270317	chr10	16157552	16157587	17	36	2.176471	no	no	no	no	no	no	no	no	no	no
182270336	chr10	16233293	16233320	13	28	2.153846	no	no	no	no	no	no	no	no	no	no
182270337	chr10	16234194	16234249	28	56	2.107143	no	no	no	no	no	no	no	no	no	no
182270350	chr10	16272525	16272580	22	56	2.545455	no	no	no	no	no	no	no	no	no	no
182270351	chr10	16280102	16280142	20	41	2.050000	no	no	yes	no	no	no	no	no	no	yes
182270353	chr10	16284999	16285036	16	38	2.375000	no	no	yes	no	no	no	no	no	no	yes
182270361	chr10	16295749	16295809	32	61	1.906250	no	yes	no	no	no	no	no	yes	no	yes
182270365	chr10	16297028	16297087	18	60	3.388889	no	no	no	no	no	no	no	yes	no	no
182270379	chr10	16302895	16302932	20	38	1.900000	no	no	no	no	no	no	no	yes	no	no
182270391	chr10	16329627	16329655	11	29	2.636364	no	no	no	no	no	no	no	no	no	no
182270407	chr10	16361276	16361338	27	63	2.321429	no	no	no	no	no	no	no	no	no	no
182270425	chr10	16403784	16403853	12	70	5.833333	no	no	no	no	no	no	no	no	no	no
182270426	chr10	16405059	16405089	16	31	1.937500	no	no	no	no	no	no	no	no	no	no
182270428	chr10	16409485	16409529	17	45	2.666667	no	no	no	no	no	no	no	no	yes	no
182270430	chr10	16413106	16413133	8	28	3.500000	no	no	no	no	no	no	no	yes	yes	no
182270433	chr10	16425677	16425707	14	31	2.214286	no	no	no	no	no	no	no	yes	yes	no
182270434	chr10	16428231	16428304	19	74	3.894737	no	no	no	no	no	no	no	no	no	no
182270440	chr10	16437243	16437276	17	34	2.117647	no	no	yes	no	no	no	yes	yes	yes	yes
182270442	chr10	16439642	16439684	17	43	2.647059	no	no	yes	no	no	no	no	yes	no	yes
182270449	chr10	16454582	16454627	21	46	2.238095	no	no	yes	no	no	no	no	no	no	yes
182270454	chr10	16461270	16461305	15	36	2.400000	no	no	yes	no	no	no	no	no	no	yes
182270458	chr10	16483109	16483142	17	34	2.058824	no	no	yes	no	no	no	no	yes	no	yes
182270472	chr10	16504029	16504074	22	46	2.090909	no	no	yes	no	no	no	no	no	no	yes
182270473	chr10	16504239	16504272	18	34	1.944444	no	no	yes	no	no	no	no	no	no	yes
182270474	chr10	16504380	16504421	20	42	2.200000	no	no	yes	no	no	no	no	no	no	yes

182270480	chr10	16513394	16513434	15	41	2.733333	no	yes	no	no	no	yes	no	no	no	yes
182270482	chr10	16523948	16523983	11	36	3.363636	no	yes	yes	no	no	no	no	no	no	yes
182270483	chr10	16530552	16530589	19	38	2.000000	no	no	yes	no	no	no	no	no	no	yes
182270490	chr10	16538686	16538732	24	47	1.958333	no	no	yes	no	no	no	no	no	no	yes
182270492	chr10	16539645	16539703	28	59	2.178571	no	yes	no	no	no	no	no	no	no	yes
182270494	chr10	16550840	16550883	21	44	2.095238	no	no	no	no	no	no	no	no	no	no
182270497	chr10	16561121	16561159	20	39	1.950000	no	no	no	no	no	no	no	no	no	no
182270508	chr10	16579797	16579833	19	37	1.947368	no	no	no	no	no	no	no	no	no	no
182270510	chr10	16582549	16582588	15	40	2.666667	no	no	no	no	no	no	no	no	no	no
182270520	chr10	16592077	16592210	67	134	2.000000	no	yes	no	no	no	yes	no	no	no	yes
182270521	chr10	16592223	16592258	18	36	2.000000	no	yes	no	no	no	yes	no	no	no	yes
182270527	chr10	16640118	16640174	31	57	1.903226	no	no	yes	no	no	no	no	no	no	yes
182270529	chr10	16645854	16646059	28	206	7.333333	no	no	yes	no	no	no	no	no	no	yes
182270533	chr10	16648885	16648913	15	29	2.000000	no	no	yes	no	no	no	no	no	yes	yes
182270549	chr10	16722791	16723033	26	243	9.218750	no	no	yes	no	no	no	no	no	no	yes
182270552	chr10	16729477	16729532	29	56	1.931034	no	no	yes	no	no	no	no	yes	yes	yes
182270558	chr10	16756882	16756926	23	45	2.043478	no	no	yes	no	no	no	no	yes	no	yes
182270563	chr10	16764702	16765149	226	448	1.995575	no	no	yes	no	no	no	no	no	no	yes
182270565	chr10	16768121	16768153	17	33	1.941176	no	no	yes	no	no	yes	no	yes	no	yes
182270569	chr10	16783657	16783688	12	32	2.666667	no	no	yes	no	no	no	no	no	no	yes
182270571	chr10	16785412	16785526	30	115	3.781250	no	no	yes	no	no	no	no	yes	no	yes
182270572	chr10	16790000	16790040	20	41	2.100000	no	no	yes	no	no	no	no	no	yes	yes
182270581	chr10	16826244	16826278	17	35	2.058824	no	no	yes	no	no	no	no	yes	no	yes
182270586	chr10	16833633	16833670	9	38	4.200000	no	no	yes	no	no	no	no	no	yes	yes
182270587	chr10	16835886	16835929	23	44	1.913043	no	no	yes	no	no	no	no	no	yes	yes

182270602 chr10 16855039 16855105 26 67 2.592592 no no yes no
no no no yes no yes
182270606 chr10 16857322 16857359 19 38 2.000000 no no yes no
no no no yes no yes
182270616 chr10 16883888 16883916 15 29 1.933333 no no yes no
no no no yes no yes
182270632 chr10 16903686 16903777 49 92 1.918367 no no yes no
no no no yes no yes
182270633 chr10 16906910 16906943 16 34 2.125000 no no yes no
no no no no no yes
182270637 chr10 16910024 16910077 29 54 1.931034 no no yes no
no no no yes yes yes
182270641 chr10 16930093 16930118 12 26 2.166667 no no yes no
no no no no no yes
182270649 chr10 16948786 16948811 13 26 2.000000 no no yes no
no no no no no yes
182270651 chr10 16950624 16950691 29 68 2.344828 no no yes no
no no no yes yes yes
182270666 chr10 16989353 16989395 16 43 2.687500 no no yes no
no no no no no yes
182270670 chr10 17003515 17003566 17 52 2.947368 no no yes no
no no no yes no yes
182270673 chr10 17008172 17008464 103 293 2.923810 no no yes no
no no no yes yes yes
182270694 chr10 17041468 17041500 17 33 1.941176 no no yes no
no no no no no yes
182270695 chr10 17043728 17043840 21 113 5.380952 no no yes yes
no no no no no yes
182270702 chr10 17044395 17044419 13 25 1.923077 no no yes no
no no no no no yes
182270717 chr10 17100850 17100909 29 60 2.068965 no no yes no
no no no yes yes yes
182270721 chr10 17104698 17104759 33 62 1.939394 no no yes no
no no no no no yes
182270722 chr10 17104769 17104814 14 46 3.200000 no no yes no
no no no no no yes
182270731 chr10 17130957 17130988 16 32 2.000000 no no no no no
no no yes no no
182270737 chr10 17150996 17151033 20 38 1.900000 no no yes no
no yes no no no yes
182270738 chr10 17152899 17152940 18 42 2.388889 no yes yes no
no yes no no no yes
182270739 chr10 17155300 17155337 20 38 1.900000 no no yes no
no no no no no yes
182270742 chr10 17159976 17160007 9 32 3.777778 no no yes no no
no no no no yes
182270744 chr10 17163259 17163354 50 96 1.920000 no no yes no
no no no yes no yes
182270749 chr10 17177950 17177979 15 30 2.000000 no no yes no
no no no yes no yes

182270752	chr10	17186974	17187009	18	36	2.055556	no	no	yes	no	no	no	no	no	no	yes
182270754	chr10	17201445	17201479	14	35	2.500000	yes	no	yes	no	no	no	yes	yes	yes	yes
182270757	chr10	17209040	17209115	37	76	2.108108	no	no	no	no	no	no	no	no	no	no
182270777	chr10	17271686	17271742	30	57	1.900000	no	no	no	no	no	no	no	no	no	no
182270786	chr10	17281444	17281489	24	46	1.958333	no	no	no	no	no	no	no	yes	no	no
182270792	chr10	17283901	17284064	22	164	7.400000	no	no	no	no	no	no	no	no	no	no
182270793	chr10	17288377	17288511	36	135	3.750000	no	no	no	no	no	no	no	no	no	no
182270799	chr10	17303274	17303359	44	86	2.045455	no	no	no	no	no	no	no	no	no	no
182270803	chr10	17304070	17304354	140	285	2.042857	no	no	no	no	no	no	no	no	no	no
182270804	chr10	17304379	17304415	14	37	2.714286	no	no	no	no	no	no	no	no	no	no
182270805	chr10	17304647	17304882	127	236	1.858268	no	no	no	no	no	no	no	no	no	no
182270830	chr10	17325397	17325519	35	123	3.571429	no	no	yes	no	no	no	no	yes	no	yes
182270832	chr10	17331739	17331783	23	45	2.000000	no	no	yes	no	no	no	no	no	no	yes
182270876	chr10	17422124	17422162	21	39	1.904762	no	no	yes	no	no	no	no	no	no	yes
182270879	chr10	17429520	17429547	14	28	2.000000	no	no	yes	no	no	no	no	yes	yes	yes
182270905	chr10	17518199	17518499	43	301	7.000000	no	no	no	no	no	no	no	no	no	no
182270925	chr10	17554885	17555111	30	227	7.870968	no	no	no	no	no	no	no	yes	no	no
182270941	chr10	17576367	17576404	20	38	1.900000	yes	no	no	no	no	no	no	no	no	no
182270942	chr10	17577983	17578023	21	41	1.952381	no	yes	no	no	no	no	no	yes	no	yes
182270949	chr10	17591601	17591626	13	26	2.000000	no	no	yes	no	no	no	no	no	no	yes
182270959	chr10	17624697	17624753	19	57	3.000000	no	no	no	no	no	no	no	yes	no	no
182270970	chr10	17632327	17632376	23	50	2.173913	no	no	no	no	no	no	no	no	no	no
182270975	chr10	17641566	17641599	18	34	2.000000	no	no	no	no	yes	no	no	no	no	yes
182270979	chr10	17653359	17653394	18	36	2.000000	no	no	yes	no	no	no	no	yes	no	yes
182270985	chr10	17671744	17671775	15	32	2.133333	no	no	yes	no	no	no	no	yes	no	yes

182270987	chr10	17699752	17699797	23	46	2.000000	no	no	yes	no	no	no	no	no	no	yes
182270990	chr10	17704759	17704790	14	32	2.357143	no	no	yes	no	no	no	no	no	no	yes
182270993	chr10	17731498	17731533	17	36	2.176471	no	no	no	no	no	no	no	no	no	no
182271002	chr10	17762625	17762681	30	57	1.900000	no	no	yes	no	no	no	no	yes	no	yes
182271004	chr10	17772265	17772306	22	42	1.909091	no	no	yes	no	no	no	no	no	no	yes
182271007	chr10	17776181	17776219	19	39	2.157895	no	no	yes	no	no	no	no	no	no	yes
182271015	chr10	17800349	17800421	27	73	2.678571	no	yes	no	no	no	no	no	no	no	yes
182271022	chr10	17817286	17817411	63	126	2.000000	no	no	yes	no	no	no	no	no	no	yes
182271029	chr10	17843314	17843385	29	72	2.482759	no	no	yes	no	no	no	no	no	no	yes
182271032	chr10	17854318	17854389	22	72	3.363636	no	no	yes	no	no	no	no	yes	no	yes
182271043	chr10	17871266	17871302	19	37	1.947368	no	no	yes	no	no	no	no	yes	no	yes
182271044	chr10	17872586	17872730	72	145	2.013889	no	no	yes	no	no	no	no	yes	no	yes
182271045	chr10	17873224	17873261	19	38	2.052632	no	no	yes	no	no	no	no	yes	no	yes
182271046	chr10	17876597	17876629	14	33	2.357143	no	no	yes	no	no	no	no	no	no	yes
182271053	chr10	17886345	17886411	26	67	2.576923	no	no	yes	no	no	no	no	no	no	yes
182271062	chr10	17899739	17899775	19	37	2.052632	no	no	yes	no	no	no	no	no	no	yes
182271063	chr10	17900182	17900236	27	55	2.037037	no	no	yes	no	no	no	no	no	no	yes
182271064	chr10	17900271	17900302	13	32	2.428571	no	no	yes	no	no	no	no	no	no	yes
182271076	chr10	17914324	17914638	178	315	1.825843	no	no	no	no	no	no	no	yes	no	no
182271078	chr10	17914784	17914814	13	31	2.384615	no	no	no	no	no	no	no	no	no	no
182271087	chr10	17919320	17919475	79	156	2.025316	no	no	no	no	no	no	no	no	no	no
182271091	chr10	17936445	17936498	27	54	2.000000	no	no	no	no	no	no	no	yes	no	no
182271100	chr10	17956268	17956508	45	241	5.533333	no	no	yes	no	no	no	no	yes	no	yes
182271107	chr10	17990318	17990362	16	45	2.812500	no	no	yes	no	no	no	no	yes	yes	yes
182271108	chr10	17990363	17990395	15	33	2.200000	no	no	yes	no	no	no	no	yes	yes	yes

182271109	chr10	17990533	17990566	17	34	2.000000	no	no	yes	no	no	no	no	yes	yes	yes
182271113	chr10	17997182	17997249	32	68	2.156250	no	no	yes	no	no	no	no	no	no	yes
182271115	chr10	17998918	17999014	34	97	2.852941	no	no	yes	no	no	no	no	yes	no	yes
182271117	chr10	18003732	18003822	41	91	2.214286	no	no	yes	no	no	no	no	no	no	yes
182271118	chr10	18013197	18013233	19	37	1.947368	no	no	yes	no	no	no	no	no	no	yes
182271126	chr10	18040061	18040100	20	40	2.100000	no	no	yes	no	no	no	no	no	no	yes
182271139	chr10	18071399	18071431	16	33	2.062500	no	no	no	no	no	no	no	yes	no	no
182271141	chr10	18073309	18073365	29	57	1.965517	no	no	no	no	no	no	no	no	no	no
182271143	chr10	18080298	18080346	20	49	2.476191	no	no	no	no	no	no	no	no	no	no
182271152	chr10	18087090	18087146	27	57	2.111111	no	no	no	no	no	no	no	no	no	no
182271154	chr10	18100558	18100582	13	25	1.923077	no	no	no	no	no	no	no	no	no	no
182271155	chr10	18101136	18101178	20	43	2.200000	no	no	no	no	no	no	no	no	no	no
182271162	chr10	18111560	18111612	24	53	2.208333	no	no	no	no	no	no	no	no	no	no
182271176	chr10	18121551	18121925	158	375	2.397516	no	no	no	no	no	no	no	yes	yes	no
182271181	chr10	18125179	18125210	15	32	2.133333	no	no	no	no	no	no	no	no	no	no
182271184	chr10	18131682	18131732	18	51	2.833333	no	no	no	no	no	no	no	no	no	no
182271188	chr10	18140419	18140463	24	45	1.958333	yes	no	no	no	no	no	yes	yes	yes	no
182271200	chr10	18156055	18156096	20	42	2.100000	no	no	yes	no	no	no	no	yes	no	yes
182271203	chr10	18166220	18166249	15	30	2.000000	no	no	yes	no	no	no	no	no	no	yes
182271207	chr10	18171630	18171660	14	31	2.214286	no	no	yes	no	no	no	no	no	yes	yes
182271209	chr10	18174171	18174226	28	56	2.000000	no	no	yes	no	no	no	no	yes	no	yes
182271212	chr10	18174249	18174361	55	113	2.109091	no	no	yes	no	no	no	no	yes	no	yes
182271225	chr10	18200487	18200537	18	51	3.000000	no	no	yes	no	no	no	no	no	no	yes
182271232	chr10	18225510	18225556	24	47	1.958333	no	no	yes	no	no	no	no	no	no	yes
182271244	chr10	18242071	18242143	32	73	2.281250	no	no	yes	no	no	no	no	no	no	yes

182271249	chr10	18271141	18271202	31	62	2.000000	no	no	yes	no	no	no	no	no	no	yes
182271250	chr10	18271433	18271469	18	37	2.055556	no	no	yes	no	no	no	no	no	no	yes
182271254	chr10	18273762	18273786	13	25	1.923077	no	no	yes	no	no	no	no	no	no	yes
182271266	chr10	18299068	18299136	19	69	3.631579	no	no	yes	no	no	no	no	no	no	yes
182271268	chr10	18303620	18303650	15	31	2.066667	no	no	yes	no	no	no	no	no	no	yes
182271304	chr10	18391334	18391363	10	30	3.000000	no	no	yes	no	no	no	no	no	no	yes
182271307	chr10	18398984	18399021	14	38	2.857143	no	no	yes	no	no	no	no	yes	no	yes
182271351	chr10	18511811	18511888	40	78	1.950000	no	no	yes	no	no	no	no	yes	no	yes
182271391	chr10	18621823	18621861	18	39	2.166667	no	no	yes	no	no	no	no	no	no	yes
182271394	chr10	18623131	18623177	24	47	1.958333	no	no	yes	no	no	no	no	no	no	yes
182271398	chr10	18647490	18647539	24	50	2.083333	no	no	yes	no	no	no	no	no	no	yes
182271402	chr10	18658610	18658637	13	28	2.153846	yes	no	yes	no	no	no	no	yes	yes	yes
182271409	chr10	18678149	18678207	32	59	1.906250	no	yes	no	no	no	no	no	no	no	yes
182271410	chr10	18679421	18679448	12	28	2.333333	no	yes	no	no	no	no	no	no	no	yes
182271414	chr10	18689362	18689390	9	29	3.222222	no	no	no	no	no	no	no	yes	yes	no
182271415	chr10	18690074	18690172	48	99	2.062500	no	no	no	no	no	no	no	yes	no	no
182271442	chr10	18711348	18711387	18	40	2.222222	no	no	yes	no	no	no	no	no	no	yes
182271447	chr10	18720888	18720973	37	86	2.368421	no	no	yes	no	no	no	no	no	no	yes
182271453	chr10	18741434	18741723	50	290	5.800000	no	no	yes	no	no	no	no	yes	no	yes
182271456	chr10	18746550	18746825	139	276	2.014389	no	yes	yes	no	no	no	no	no	yes	yes
182271457	chr10	18747904	18747932	15	29	1.933333	no	yes	no	no	no	yes	no	no	no	yes
182271464	chr10	18773914	18773961	25	48	1.960000	no	no	no	no	no	no	no	no	no	no
182271465	chr10	18775451	18775490	21	40	1.952381	no	no	no	no	no	no	no	no	no	no
182271482	chr10	18810761	18810810	24	50	2.083333	no	no	no	no	no	no	no	no	no	no
182271494	chr10	18822993	18823034	13	42	3.230769	no	no	no	no	no	no	no	no	no	no

182271495	chr10	18828608	18828636	15	29	1.933333	no	no	no	no	no	no	no	no	no	no
182271497	chr10	18838290	18838332	21	43	2.047619	no	no	no	no	no	no	no	no	no	no
182271501	chr10	18865917	18865949	14	33	2.357143	no	no	no	no	no	no	no	yes	no	no
182271508	chr10	18882546	18882585	19	40	2.105263	no	no	no	no	no	no	no	no	no	no
182271512	chr10	18895844	18895876	13	33	2.538461	no	no	no	no	no	no	no	no	no	no
182271514	chr10	18895934	18895984	24	51	2.125000	no	no	no	no	no	no	no	no	no	no
182271525	chr10	18926386	18926452	32	67	2.181818	no	no	no	no	no	no	no	no	no	no
182271526	chr10	18926600	18926644	20	45	2.300000	no	no	no	no	no	no	no	no	yes	no
182271531	chr10	18933278	18933314	18	37	2.055556	no	no	no	no	no	no	no	no	no	no
182271544	chr10	18960475	18960507	16	33	2.062500	no	no	no	no	no	no	no	no	no	no
182271552	chr10	18992984	18993016	17	33	2.000000	no	no	no	no	no	no	no	no	no	no
182271557	chr10	19002167	19002227	18	61	3.500000	no	no	no	no	no	no	no	no	no	no
182271563	chr10	19007557	19007609	21	53	2.500000	no	no	no	no	no	no	no	yes	no	no
182271568	chr10	19027668	19027710	17	43	2.500000	no	no	no	no	no	no	no	no	no	no
182271574	chr10	19027997	19028056	25	60	2.400000	no	no	no	no	no	no	no	no	no	no
182271585	chr10	19040903	19040961	31	59	1.903226	no	no	no	no	no	no	no	no	no	no
182271588	chr10	19045215	19045246	16	32	2.062500	no	no	no	no	no	no	no	no	no	no
182271593	chr10	19058823	19058857	18	35	1.944444	no	no	yes	no	no	no	no	no	no	yes
182271596	chr10	19066891	19066940	25	50	2.040000	no	no	yes	no	no	no	no	no	no	yes
182271601	chr10	19076247	19076314	23	68	2.958333	no	no	yes	no	no	no	no	no	no	yes
182271603	chr10	19082402	19082431	16	30	1.937500	no	no	yes	no	no	no	no	yes	no	yes
182271613	chr10	19102160	19102191	15	32	2.133333	no	no	yes	no	no	no	no	no	no	yes
182271614	chr10	19102200	19102246	24	47	1.958333	no	no	yes	no	no	no	no	no	no	yes
182271623	chr10	19116182	19116225	18	44	2.473684	no	no	yes	no	no	no	no	no	no	yes
182271647	chr10	19165800	19165997	99	198	2.010101	no	no	yes	yes	no	no	no	yes	no	yes

182271648	chr10	19169485	19169532	24	48	2.041667	no	no	yes	no	no	no	no	no	yes	yes
182271650	chr10	19171482	19171708	38	227	6.078948	no	no	yes	no	no	no	no	no	no	yes
182271668	chr10	19204416	19204449	11	34	3.083333	no	no	yes	no	no	no	no	yes	yes	yes
182271686	chr10	19260269	19260306	16	38	2.375000	no	no	yes	no	no	no	no	no	no	yes
182271696	chr10	19271983	19272024	22	42	1.954545	no	no	yes	no	no	no	no	no	no	yes
182271697	chr10	19273174	19273215	18	42	2.333333	no	no	yes	no	no	no	no	no	no	yes
182271703	chr10	19280715	19280750	18	36	2.000000	no	no	yes	no	no	no	no	no	no	yes
182271704	chr10	19282798	19282823	12	26	2.166667	no	no	yes	no	no	no	no	no	no	yes
182271706	chr10	19292364	19292405	20	42	2.150000	yes	no	yes	no	no	no	no	no	no	yes
182271707	chr10	19298435	19298471	18	37	2.055556	no	no	yes	no	no	no	no	yes	no	yes
182271708	chr10	19298462	19298495	10	34	3.400000	no	no	yes	no	no	no	no	yes	no	yes
182271717	chr10	19306566	19306597	11	32	2.909091	no	no	yes	no	no	no	no	no	no	yes
182271719	chr10	19312755	19312803	11	49	4.636364	no	no	yes	no	no	no	no	no	no	yes
182271722	chr10	19314297	19314333	15	37	2.533333	no	no	yes	no	no	no	no	no	no	yes
182271730	chr10	19316060	19316162	34	103	3.029412	no	no	yes	no	no	no	no	no	yes	yes
182271732	chr10	19319908	19319945	18	38	2.111111	no	no	yes	no	no	no	no	no	no	yes
182271738	chr10	19337060	19337091	13	32	2.538461	no	no	yes	no	no	no	no	no	no	yes
182271739	chr10	19338512	19338541	15	30	2.066667	no	no	yes	no	no	no	no	no	no	yes
182271742	chr10	19341117	19341153	19	37	1.947368	no	no	yes	no	no	no	no	no	no	yes
182271753	chr10	19359383	19359422	19	40	2.157895	no	no	yes	no	no	no	no	yes	no	yes
182271761	chr10	19369249	19369282	15	34	2.266667	no	no	yes	no	no	no	no	no	no	yes
182271765	chr10	19369652	19369733	36	82	2.277778	no	no	yes	no	no	no	no	no	no	yes
182271778	chr10	19380537	19380582	24	46	1.916667	no	no	yes	no	no	no	no	no	no	yes
182271784	chr10	19386686	19386728	22	43	1.954545	no	no	yes	no	no	no	no	no	no	yes
182271785	chr10	19388743	19388785	21	43	2.047619	no	no	yes	no	no	no	no	no	no	yes

182271796	chr10	19423388	19423422	12	35	3.000000	no	no	yes	no	no	no	no	no	no	yes
182271798	chr10	19425498	19425531	15	34	2.333333	no	no	yes	no	no	no	no	no	no	yes
182271800	chr10	19434584	19434618	18	35	1.944444	no	no	yes	no	no	no	no	no	no	yes
182271803	chr10	19437239	19437275	18	37	2.111111	no	no	yes	no	no	no	no	no	no	yes
182271811	chr10	19463145	19463176	12	32	2.666667	no	no	yes	no	no	no	no	no	no	yes
182271817	chr10	19490424	19490489	31	66	2.129032	no	no	yes	no	no	yes	no	yes	no	yes
182271820	chr10	19504598	19504635	20	38	1.900000	no	no	yes	no	no	no	no	no	no	yes
182271823	chr10	19505090	19505136	24	47	1.958333	no	no	yes	no	no	no	no	no	no	yes
182271828	chr10	19516684	19516732	21	49	2.380952	no	no	yes	no	no	no	no	yes	no	yes
182271829	chr10	19516751	19516798	21	48	2.333333	no	no	yes	no	no	no	no	yes	no	yes
182271831	chr10	19518419	19518462	18	44	2.421053	no	no	yes	no	no	no	no	yes	no	yes
182271832	chr10	19519109	19519205	46	97	2.108696	no	no	yes	no	no	no	no	yes	no	yes
182271839	chr10	19535468	19535587	46	120	2.625000	no	no	yes	no	no	no	no	no	no	yes
182271848	chr10	19567103	19567132	13	30	2.307692	no	no	yes	no	no	no	no	no	no	yes
182271851	chr10	19569624	19569664	20	41	2.150000	no	no	yes	no	no	no	no	no	no	yes
182271860	chr10	19577735	19577777	22	43	1.954545	no	no	yes	no	no	no	no	no	no	yes
182271861	chr10	19580926	19580951	11	26	2.363636	no	no	yes	no	no	no	no	no	yes	yes
182271863	chr10	19587695	19587887	95	193	2.031579	no	no	yes	no	no	no	no	no	no	yes
182271868	chr10	19590249	19590413	50	165	3.326923	no	no	yes	no	no	no	no	no	no	yes
182271869	chr10	19590425	19590452	12	28	2.333333	no	no	yes	no	no	no	no	no	no	yes
182271874	chr10	19604955	19604998	14	44	3.133333	no	no	yes	no	no	no	no	no	no	yes
182271881	chr10	19643096	19643130	18	35	1.944444	no	no	yes	no	no	no	no	no	no	yes
182271882	chr10	19653647	19653785	70	139	1.985714	no	no	yes	no	no	no	no	no	no	yes
182271893	chr10	19671933	19671965	16	33	2.062500	no	no	yes	no	no	no	no	no	no	yes
182271896	chr10	19688988	19689013	13	26	2.000000	no	no	yes	no	no	no	no	no	no	yes

182271904	chr10	19699278	19699328	9	51	5.666667	no	no	yes	no	no	no	no	yes	no	yes
182271908	chr10	19701262	19701338	30	77	2.580645	no	no	yes	no	no	no	no	no	no	yes
182271912	chr10	19701813	19701855	23	43	1.956522	no	no	yes	no	no	no	no	yes	no	yes
182271914	chr10	19706000	19706037	17	38	2.235294	no	no	yes	no	no	no	no	no	no	yes
182271918	chr10	19707611	19707639	14	29	2.071429	no	no	yes	no	no	no	no	no	yes	yes
182271919	chr10	19707988	19708019	13	32	2.538461	no	no	yes	no	no	no	no	no	no	yes
182271923	chr10	19715064	19715184	57	121	2.122807	no	no	yes	no	no	no	no	no	no	yes
182271931	chr10	19720099	19720147	25	49	1.960000	no	no	yes	no	no	no	no	no	no	yes
182271932	chr10	19721334	19721365	16	32	2.062500	no	no	yes	no	no	yes	no	no	yes	yes
182271934	chr10	19729389	19729449	31	61	2.064516	yes	no	yes	no	no	no	no	no	no	yes
182271935	chr10	19729510	19729610	43	101	2.348837	yes	no	yes	no	no	no	no	no	no	yes
182271940	chr10	19733172	19733218	23	47	2.086957	no	no	yes	no	no	no	no	no	no	yes
182271953	chr10	19757394	19757431	20	38	1.900000	no	no	yes	no	no	no	no	yes	no	yes
182271956	chr10	19760051	19760196	42	146	3.434783	no	no	yes	no	no	no	no	no	no	yes
182271964	chr10	19780916	19780958	22	43	1.954545	no	no	yes	no	no	no	no	no	no	yes
182271965	chr10	19784146	19784192	25	47	1.920000	no	no	yes	no	no	no	no	no	no	yes
182271970	chr10	19790945	19790985	20	41	2.050000	no	no	no	no	no	yes	no	no	no	no
182271982	chr10	19825556	19825589	16	34	2.125000	no	no	yes	no	no	no	no	no	no	yes
182271983	chr10	19825946	19825994	25	49	2.000000	no	no	yes	no	no	no	no	yes	yes	yes
182271989	chr10	19843312	19843347	15	36	2.400000	no	no	yes	no	no	no	no	yes	no	yes
182271991	chr10	19848975	19849007	11	33	3.000000	no	no	yes	no	no	no	no	no	no	yes
182272003	chr10	19877162	19877251	41	90	2.268293	no	no	yes	no	no	no	no	yes	no	yes
182272005	chr10	19878131	19878172	16	42	2.687500	no	no	yes	no	no	no	no	yes	no	yes
182272016	chr10	19898828	19898898	37	71	1.918919	no	no	yes	no	no	no	no	no	yes	yes
182272022	chr10	19908732	19908783	25	52	2.080000	no	no	yes	no	no	no	no	no	yes	yes

182272023	chr10	19909598	19909636	20	39	2.000000	no	no	yes	no	no	no	no	no	yes	yes
182272025	chr10	19913916	19913961	24	46	1.916667	no	no	yes	no	no	no	no	yes	no	yes
182272040	chr10	19933774	19933927	70	154	2.228571	no	no	yes	no	no	no	no	yes	no	yes
182272043	chr10	19935609	19935643	18	35	2.000000	no	no	yes	no	no	no	no	no	no	yes
182272045	chr10	19943692	19943736	22	45	2.090909	no	no	yes	no	no	no	no	no	no	yes
182272050	chr10	19960809	19960850	19	42	2.210526	no	no	yes	no	no	no	no	no	no	yes
182272052	chr10	19966327	19966447	25	121	4.920000	no	no	yes	no	no	no	no	no	no	yes
182272064	chr10	19981143	19981184	15	42	2.800000	no	no	yes	no	no	no	no	no	no	yes
182272065	chr10	19983247	19983283	18	37	2.111111	no	no	yes	no	no	no	no	no	no	yes
182272066	chr10	19986541	19986579	18	39	2.166667	no	no	yes	no	no	no	no	yes	no	yes
182272070	chr10	20000488	20000566	39	79	2.025641	no	no	yes	no	no	no	no	yes	no	yes
182272071	chr10	20007338	20007374	17	37	2.235294	no	no	yes	no	no	no	no	yes	yes	yes
182272075	chr10	20014293	20014337	23	45	1.956522	no	no	yes	no	no	no	no	yes	yes	yes
182272077	chr10	20017592	20017624	15	33	2.200000	no	no	yes	no	no	no	no	yes	yes	yes
182272079	chr10	20020565	20020602	20	38	1.900000	no	no	yes	no	no	no	no	no	no	yes
182272086	chr10	20026798	20027125	32	328	10.250000	no	no	yes	no	no	no	no	no	no	yes
182272095	chr10	20046184	20046224	19	41	2.210526	no	no	yes	no	no	no	no	no	no	yes
182272099	chr10	20052723	20052768	17	46	2.705882	no	no	yes	no	no	no	no	no	no	yes
182272102	chr10	20054909	20054945	14	37	2.642857	no	no	yes	no	no	no	no	no	no	yes
182272113	chr10	20068639	20068681	22	43	1.954545	no	no	yes	no	no	no	no	no	no	yes
182272118	chr10	20085785	20085821	19	37	2.000000	no	no	yes	no	no	no	no	no	no	yes
182272123	chr10	20097902	20097979	18	78	4.388889	no	no	yes	no	no	no	no	no	no	yes
182272145	chr10	20140900	20140931	15	32	2.133333	no	no	yes	no	no	no	no	no	no	yes
182272146	chr10	20141881	20141917	19	37	1.947368	no	no	yes	no	no	no	no	no	no	yes
182272148	chr10	20146322	20146372	25	51	2.120000	no	no	yes	no	no	no	no	yes	no	yes

182272151	chr10	20148136	20148175	11	40	3.538461	no	no	yes	no	no	no	no	no	no	yes
182272153	chr10	20151965	20152001	18	37	2.111111	no	no	yes	no	no	no	no	no	no	yes
182272154	chr10	20158354	20158401	16	48	3.000000	no	no	yes	no	no	no	no	no	yes	yes
182272160	chr10	20163419	20163464	21	46	2.190476	no	no	yes	no	no	no	no	yes	no	yes
182272164	chr10	20184345	20184392	19	48	2.650000	no	no	yes	no	no	no	no	no	no	yes
182272169	chr10	20189481	20189581	38	101	2.657895	no	no	yes	no	no	no	no	no	no	yes
182272178	chr10	20216097	20216130	16	34	2.125000	no	no	yes	no	no	no	no	yes	no	yes
182272185	chr10	20233127	20233160	17	34	2.000000	no	no	yes	no	no	no	no	no	yes	yes
182272192	chr10	20244528	20244560	18	33	1.944444	no	no	yes	no	no	no	no	no	no	yes
182272196	chr10	20252857	20252910	27	54	2.000000	no	no	yes	no	no	no	no	no	no	yes
182272205	chr10	20272429	20272469	21	41	1.952381	no	no	yes	no	no	no	no	yes	no	yes
182272211	chr10	20274098	20274144	16	47	3.000000	no	no	yes	no	no	no	no	yes	no	yes
182272213	chr10	20282022	20282051	14	30	2.142857	no	yes	no	no	no	no	no	no	no	yes
182272216	chr10	20287245	20287286	22	42	1.954545	no	yes	no	no	no	no	no	no	no	yes
182272218	chr10	20319766	20319802	16	37	2.312500	no	no	no	no	no	no	no	no	no	no
182272234	chr10	20350144	20350183	21	40	1.904762	no	no	yes	no	no	no	no	no	no	yes
182272247	chr10	20379401	20379432	16	32	2.000000	no	no	no	no	no	no	no	no	no	no
182272251	chr10	20400856	20400908	29	53	1.827586	no	no	no	no	no	no	no	no	no	no
182272256	chr10	20405363	20405424	22	62	2.818182	no	no	no	no	no	no	no	yes	no	no
182272259	chr10	20413392	20413443	27	52	2.037037	no	no	no	no	no	no	no	no	no	no
182272273	chr10	20448710	20448758	19	49	2.578947	no	no	no	no	no	no	no	no	no	no
182272284	chr10	20476960	20477112	82	153	1.914634	no	no	no	no	no	no	no	no	no	no
182272298	chr10	20534979	20535031	26	53	2.038461	no	no	no	no	no	no	no	no	no	no
182272314	chr10	20550747	20550778	12	32	2.750000	no	no	no	no	no	no	no	yes	no	no
182272330	chr10	20594356	20594392	20	37	1.950000	no	no	no	no	no	no	no	no	yes	no

182272341	chr10	20632497	20632541	20	45	2.250000	no	no	no	no	no	no	no	no	no	no
182272343	chr10	20644315	20644355	14	41	3.000000	no	no	no	no	no	no	no	no	yes	no
182272351	chr10	20667911	20667953	20	43	2.200000	no	no	no	no	no	no	no	no	no	no
182272355	chr10	20691032	20691104	36	73	2.027778	no	no	no	no	no	no	no	no	no	no
182272356	chr10	20708790	20708833	21	44	2.095238	no	no	no	no	no	no	no	no	yes	no
182272361	chr10	20715858	20715915	29	58	2.103448	no	no	no	no	no	no	no	yes	no	no
182272377	chr10	20770473	20770505	17	33	2.000000	no	no	no	no	no	no	no	no	no	no
182272384	chr10	20799509	20799566	30	58	2.000000	no	no	yes	no	no	no	no	no	no	yes
182272392	chr10	20822390	20822425	16	36	2.250000	no	no	yes	no	no	no	no	no	no	yes
182272394	chr10	20822551	20822676	56	126	2.298246	no	no	yes	no	no	no	no	no	no	yes
182272399	chr10	20822653	20822714	25	62	2.480000	no	no	yes	no	no	no	no	no	no	yes
182272400	chr10	20823751	20823779	12	29	2.500000	no	no	yes	no	no	no	no	no	no	yes
182272402	chr10	20848161	20848195	18	35	1.944444	no	no	yes	no	no	no	no	no	no	yes
182272418	chr10	20893345	20893373	14	29	2.071429	no	no	yes	no	no	no	no	no	no	yes
182272422	chr10	20896387	20896419	15	33	2.266667	no	no	yes	no	no	no	no	no	no	yes
182272427	chr10	20911339	20911411	36	73	2.027778	no	no	yes	no	no	no	no	no	no	yes
182272428	chr10	20913839	20913882	23	44	1.913043	no	no	yes	no	no	no	no	no	no	yes
182272443	chr10	20955846	20955877	17	32	1.941176	no	no	yes	no	no	no	no	yes	no	yes
182272444	chr10	20959274	20959311	16	38	2.437500	no	no	yes	no	no	no	no	yes	no	yes
182272448	chr10	20966989	20967041	16	53	3.222222	no	no	yes	no	no	no	no	no	no	yes
182272450	chr10	20980278	20980339	28	62	2.300000	no	no	yes	no	no	no	no	no	no	yes
182272451	chr10	20980310	20980389	39	80	2.102564	no	no	yes	no	no	no	no	no	no	yes
182272452	chr10	20983334	20983382	25	49	1.960000	no	no	yes	no	no	no	no	no	no	yes
182272453	chr10	20985271	20985354	41	84	2.048780	no	no	yes	no	no	no	no	yes	no	yes
182272454	chr10	20985419	20985501	41	83	2.024390	no	no	yes	no	no	no	no	yes	yes	yes

182272460	chr10	21008865	21008906	11	42	4.000000	no	no	yes	no	no	no	no	no	no	yes
182272463	chr10	21008899	21008928	16	30	1.937500	no	no	yes	no	no	no	no	no	no	yes
182272465	chr10	21011629	21011694	10	66	6.307693	no	no	yes	no	no	no	no	no	no	yes
182272467	chr10	21013404	21013432	15	29	1.933333	no	no	yes	no	no	no	no	no	no	yes
182272471	chr10	21030211	21030297	42	87	2.071429	no	yes	yes	no	no	no	no	yes	yes	yes
182272472	chr10	21031983	21032014	7	32	4.500000	no	no	yes	no	no	no	no	no	no	yes
182272475	chr10	21034658	21034692	17	35	2.058824	no	no	yes	no	no	no	no	no	no	yes
182272481	chr10	21061166	21061207	22	42	1.954545	no	no	yes	no	no	no	no	yes	no	yes
182272485	chr10	21061235	21061272	19	38	2.000000	no	no	yes	no	no	no	no	yes	no	yes
182272492	chr10	21071183	21071234	18	52	2.894737	no	no	yes	no	no	no	no	no	no	yes
182272503	chr10	21098183	21098225	16	43	2.687500	no	no	yes	no	no	no	no	yes	yes	yes
182272508	chr10	21118809	21118900	45	92	2.066667	no	no	yes	no	no	no	no	no	no	yes
182272509	chr10	21119419	21119543	32	125	3.777778	no	no	yes	no	no	no	no	no	no	yes
182272516	chr10	21140699	21140737	17	39	2.294118	no	no	yes	no	no	no	no	no	no	yes
182272517	chr10	21142988	21143023	16	36	2.312500	no	no	yes	no	no	no	no	no	no	yes
182272522	chr10	21154730	21154765	18	36	2.000000	no	no	yes	no	no	no	no	no	no	yes
182272529	chr10	21174127	21174170	23	44	1.913043	yes	no	yes	no	yes	no	yes	yes	yes	yes
182272541	chr10	21204421	21204461	18	41	2.277778	no	no	no	no	no	no	no	yes	no	no
182272545	chr10	21211709	21211741	16	33	2.125000	no	no	no	no	no	no	no	yes	yes	no
182272591	chr10	21293493	21293548	23	56	2.434783	no	no	no	no	no	no	no	yes	yes	no
182272593	chr10	21298025	21298068	23	44	1.913043	no	no	no	no	no	no	no	yes	no	no
182272607	chr10	21306839	21306878	21	40	1.904762	no	no	no	no	no	no	no	no	no	no
182272624	chr10	21334009	21334034	13	26	2.000000	no	no	no	no	no	no	no	no	yes	no
182272646	chr10	21371407	21371461	27	55	2.111111	no	no	yes	no	no	no	no	yes	no	yes
182272708	chr10	21489904	21489948	23	45	2.000000	no	no	no	no	no	no	no	no	no	no

182272710	chr10	21503257	21503290	17	34	2.000000	no	no	no	no	no	no	no	yes	no	no
182272722	chr10	21525616	21525647	16	32	2.000000	yes	no	yes	no	yes	no	no	yes	yes	yes
182272725	chr10	21534411	21534449	9	39	4.444445	yes	no	yes	no	yes	no	yes	yes	no	yes
182272727	chr10	21534907	21534949	22	43	2.000000	no	no	yes	no	no	no	yes	yes	yes	yes
182272729	chr10	21536363	21536398	17	36	2.117647	no	no	yes	no	no	no	no	no	no	yes
182272730	chr10	21536497	21536540	20	44	2.200000	no	no	yes	no	no	no	no	no	yes	yes
182272734	chr10	21544184	21544224	19	41	2.157895	no	no	yes	no	no	no	no	no	no	yes
182272760	chr10	21584749	21584811	31	63	2.032258	no	no	yes	no	no	no	no	yes	no	yes
182272763	chr10	21584847	21584883	16	37	2.312500	no	no	yes	no	no	no	no	yes	no	yes
182272766	chr10	21589885	21589926	12	42	3.583333	no	no	yes	no	no	no	no	yes	no	yes
182272784	chr10	21629268	21629319	26	52	2.038461	no	no	yes	no	no	no	no	no	no	yes
182272806	chr10	21686408	21686439	9	32	3.666667	no	no	yes	no	no	no	no	no	no	yes
182272818	chr10	21706213	21706241	14	29	2.071429	no	no	yes	no	no	no	no	yes	no	yes
182272821	chr10	21717917	21717974	10	58	5.818182	no	no	yes	no	no	no	no	yes	no	yes
182272823	chr10	21733695	21733786	45	92	2.044445	no	yes	yes	yes	no	no	no	yes	no	yes
182272827	chr10	21776087	21776154	30	68	2.258065	no	no	yes	no	no	no	no	no	no	yes
182272836	chr10	21800739	21800771	11	33	3.000000	no	no	yes	no	no	no	no	no	no	yes
182272838	chr10	21803862	21803894	17	33	1.941176	no	no	yes	no	no	no	no	yes	no	yes
182272840	chr10	21806114	21806188	24	75	3.291667	no	no	yes	no	no	no	no	yes	no	yes
182272845	chr10	21809549	21809588	18	40	2.222222	no	no	yes	no	no	no	no	no	no	yes
182272854	chr10	21823511	21823576	32	66	2.062500	no	no	yes	no	no	no	no	yes	no	yes
182272871	chr10	21859573	21859641	17	69	4.058824	no	no	yes	no	no	no	no	no	no	yes
182272894	chr10	21907978	21908286	113	309	2.710744	no	no	yes	no	no	no	no	no	no	yes
182272896	chr10	21909359	21909397	20	39	2.000000	no	no	yes	no	no	no	no	no	no	yes
182272904	chr10	21912151	21912191	21	41	2.000000	no	no	yes	no	no	no	no	no	no	yes

182272908	chr10	21942493	21942523	11	31	2.818182	no	no	yes	no	no	no	no	yes	no	yes
182272912	chr10	21947137	21947186	24	50	2.083333	no	no	yes	no	no	no	no	yes	no	yes
182272915	chr10	21956509	21956569	32	61	1.906250	no	no	yes	no	no	no	no	no	no	yes
182272921	chr10	21963205	21963242	19	38	2.000000	no	no	yes	no	no	no	no	no	yes	yes
182272926	chr10	21974436	21974489	27	54	2.000000	no	no	yes	no	no	no	no	yes	no	yes
182272929	chr10	21991604	21991636	17	33	1.941176	no	no	yes	no	no	no	no	no	no	yes
182272930	chr10	21993083	21993122	21	40	1.952381	no	no	yes	no	no	no	no	yes	yes	yes
182272942	chr10	22040695	22040737	20	43	2.150000	no	no	no	no	no	no	no	no	no	no
182272946	chr10	22058142	22058174	17	33	1.941176	no	no	no	no	no	no	no	no	no	no
182272962	chr10	22105865	22105896	14	32	2.285714	no	no	no	no	no	no	no	yes	no	no
182272963	chr10	22106321	22106353	17	33	1.941176	no	no	no	no	no	no	no	no	no	no
182272965	chr10	22109276	22109308	16	33	2.062500	no	no	no	no	no	no	no	no	no	no
182272982	chr10	22152351	22152399	22	49	2.227273	no	no	no	no	no	no	no	yes	no	no
182272983	chr10	22152630	22152667	16	38	2.437500	no	no	no	no	no	no	no	yes	no	no
182272985	chr10	22157062	22157112	24	51	2.125000	no	no	no	no	no	no	no	yes	yes	no
182273006	chr10	22245654	22245706	27	53	1.962963	no	no	no	no	no	no	no	yes	yes	no
182273011	chr10	22263332	22263369	14	38	2.714286	no	no	no	no	no	no	no	no	yes	no
182273012	chr10	22264358	22264383	12	26	2.166667	no	no	no	no	no	no	no	no	no	no
182273025	chr10	22286057	22286085	15	29	1.933333	no	no	no	no	no	no	no	no	no	no
182273029	chr10	22294563	22294656	47	94	2.000000	no	no	no	no	no	no	no	no	no	no
182273031	chr10	22300259	22300781	30	523	17.406250	no	no	no	no	no	no	no	no	no	no
182273033	chr10	22301915	22301946	14	32	2.285714	no	no	no	no	no	no	no	no	no	no
182273038	chr10	22315923	22315961	21	39	1.952381	yes	no	no	no	no	no	no	yes	yes	no
182273040	chr10	22321137	22321163	14	27	1.928571	yes	no	yes	no	yes	yes	yes	yes	yes	yes
182273046	chr10	22321733	22321771	21	39	1.952381	yes	no	yes	no	no	no	yes	yes	no	yes

182273048	chr10	22321841	22321945	58	105	1.931034	yes	no	yes	no	no	no	yes	yes	no	yes
182273051	chr10	22321993	22322036	20	44	2.350000	yes	no	yes	no	no	no	yes	yes	yes	yes
182273056	chr10	22325552	22325653	45	102	2.311111	yes	no	yes	no	no	no	yes	yes	yes	yes
182273059	chr10	22346356	22346390	10	35	3.500000	no	no	yes	no	no	no	no	yes	yes	yes
182273065	chr10	22367408	22367453	17	46	2.722222	no	no	yes	no	no	no	no	yes	no	yes
182273073	chr10	22391296	22391324	14	29	2.071429	yes	no	yes	no	no	no	no	yes	no	yes
182273082	chr10	22436956	22436986	16	31	1.937500	no	no	yes	no	no	no	yes	yes	yes	yes
182273083	chr10	22437234	22437278	22	45	2.045455	no	no	yes	no	no	no	yes	yes	yes	yes
182273084	chr10	22441087	22441119	14	33	2.357143	no	no	yes	no	yes	no	no	no	no	yes
182273090	chr10	22468400	22468443	23	44	1.913043	no	no	yes	no	no	no	no	yes	no	yes
182273097	chr10	22489381	22489513	57	133	2.333333	no	no	no	no	no	no	no	yes	no	no
182273098	chr10	22493010	22493051	13	42	3.142857	no	no	no	no	no	no	no	no	no	no
182273104	chr10	22503487	22503544	28	58	2.071429	no	no	no	no	no	no	no	yes	no	no
182273105	chr10	22504626	22504671	24	46	1.958333	no	no	no	no	no	no	no	yes	no	no
182273107	chr10	22517560	22517591	16	32	2.000000	no	no	no	no	no	no	no	no	no	no
182273113	chr10	22536644	22536685	15	42	2.800000	no	yes	no	no	no	yes	no	yes	no	yes
182273115	chr10	22538325	22538391	35	67	1.914286	no	no	yes	no	no	no	no	no	no	yes
182273117	chr10	22542591	22542615	13	25	1.923077	no	no	yes	no	no	no	no	yes	no	yes
182273118	chr10	22549110	22549157	24	48	2.000000	no	no	yes	no	no	no	no	no	no	yes
182273120	chr10	22556705	22556747	22	43	1.954545	no	no	yes	no	no	no	no	no	no	yes
182273122	chr10	22562029	22562074	23	46	2.000000	no	no	yes	no	no	no	no	yes	no	yes
182273131	chr10	22582684	22582738	26	55	2.153846	no	no	yes	no	no	no	no	yes	no	yes
182273134	chr10	22599042	22599135	49	94	1.938776	no	no	yes	no	no	no	no	yes	yes	yes
182273142	chr10	22607247	22607286	21	40	1.952381	no	no	yes	no	no	yes	no	yes	no	yes
182273144	chr10	22615927	22615974	24	48	2.000000	no	no	yes	no	no	no	no	yes	yes	yes

182273147	chr10	22627716	22627752	19	37	1.947368	no	no	yes	no	no	no	no	yes	yes	yes
182273148	chr10	22633361	22633399	20	39	1.950000	no	no	yes	no	no	no	no	yes	yes	yes
182273158	chr10	22655869	22655897	14	29	2.071429	no	no	yes	no	no	no	no	yes	yes	yes
182273165	chr10	22667934	22667977	8	44	5.500000	no	no	yes	no	no	no	no	yes	yes	yes
182273170	chr10	22677123	22677154	15	32	2.200000	no	no	yes	no	no	no	no	no	no	yes
182273177	chr10	22709661	22709698	17	38	2.235294	no	no	yes	no	no	no	no	yes	no	yes
182273178	chr10	22710600	22710635	16	36	2.250000	no	no	yes	no	no	no	no	yes	yes	yes
182273187	chr10	22720195	22720220	13	26	2.000000	no	no	no	no	no	no	no	no	no	no
182273188	chr10	22721039	22721072	15	34	2.333333	no	no	no	no	no	no	no	yes	no	no
182273189	chr10	22721241	22721280	21	40	1.904762	no	no	no	no	no	no	no	no	no	no
182273192	chr10	22725965	22725995	11	31	2.818182	no	no	no	no	no	no	no	no	no	no
182273197	chr10	22752675	22752721	24	47	1.958333	no	no	no	no	no	no	no	yes	no	no
182273205	chr10	22763162	22763198	19	37	2.000000	no	no	no	no	no	no	no	no	no	no
182273210	chr10	22777295	22777385	47	91	1.978723	no	no	no	no	no	no	no	no	no	no
182273211	chr10	22777401	22777439	18	39	2.222222	no	no	no	no	no	no	no	no	no	no
182273227	chr10	22803059	22803087	14	29	2.071429	no	no	no	no	no	no	no	no	yes	no
182273237	chr10	22834462	22834498	16	37	2.312500	no	no	no	no	no	no	no	no	yes	no
182273240	chr10	22841179	22841221	21	43	2.095238	no	no	no	no	no	no	no	no	no	no
182273245	chr10	22854735	22854777	20	43	2.150000	no	no	no	no	no	no	no	yes	no	no
182273254	chr10	22906098	22906138	18	41	2.315789	no	no	no	no	no	no	no	no	no	no
182273272	chr10	22967976	22968020	23	45	2.000000	no	no	yes	no	no	no	no	yes	no	yes
182273277	chr10	22980503	22980547	23	45	1.956522	no	no	yes	no	no	no	no	no	no	yes
182273283	chr10	22986636	22986676	18	41	2.333333	no	no	yes	no	no	no	no	no	no	yes
182273285	chr10	22990959	22991023	26	65	2.615385	no	no	yes	no	no	no	no	yes	no	yes
182273290	chr10	23008351	23008383	16	33	2.125000	no	yes	yes	yes	no	yes	no	no	no	yes

182273302	chr10	23034098	23034190	29	93	3.206897	no	no	yes	no	no	no	no	no	no	yes
182273305	chr10	23037563	23037587	13	25	1.923077	no	yes	yes	no	no	no	no	yes	no	yes
182273313	chr10	23064043	23064085	20	43	2.150000	no	no	yes	no	no	no	no	yes	yes	yes
182273314	chr10	23064073	23064104	17	32	2.000000	no	no	yes	no	no	no	no	no	yes	yes
182273316	chr10	23072127	23072252	62	126	2.032258	no	no	yes	no	no	no	no	yes	yes	yes
182273317	chr10	23075272	23075313	23	42	1.913043	no	no	yes	no	no	no	no	no	no	yes
182273326	chr10	23095421	23095461	20	41	2.100000	yes	no	yes	no	no	no	no	yes	yes	yes
182273365	chr10	23154636	23154825	28	190	7.000000	no	no	no	no	no	no	no	yes	no	no
182273371	chr10	23169515	23169585	32	71	2.218750	no	no	no	no	no	no	no	yes	no	no
182273374	chr10	23175900	23175937	20	38	1.950000	no	no	no	no	no	no	no	yes	no	no
182273380	chr10	23184412	23184604	92	193	2.097826	no	no	no	no	no	no	no	no	no	no
182273381	chr10	23188615	23188652	18	38	2.222222	no	no	no	no	no	no	no	no	no	no
182273385	chr10	23192955	23192992	17	38	2.235294	yes	no	yes	yes	no	no	yes	yes	yes	yes
182273387	chr10	23197524	23197559	18	36	2.000000	no	no	no	no	no	no	no	yes	yes	no
182273389	chr10	23210263	23210294	16	32	2.000000	no	no	yes	no	no	no	no	no	no	yes
182273406	chr10	23251277	23251322	21	46	2.190476	no	no	yes	no	no	no	no	no	no	yes
182273407	chr10	23255548	23255574	8	27	3.375000	no	no	yes	no	no	no	no	no	no	yes
182273408	chr10	23257918	23257948	16	31	1.937500	yes	no	yes	no	no	no	no	yes	no	yes
182273409	chr10	23258954	23259002	24	49	2.041667	no	no	yes	no	no	yes	no	no	no	yes
182273415	chr10	23276835	23276875	18	41	2.277778	no	no	yes	no	no	no	no	no	no	yes
182273425	chr10	23294362	23294419	20	58	3.000000	no	no	yes	no	no	no	no	yes	yes	yes
182273426	chr10	23295033	23295075	22	43	1.954545	no	no	yes	no	no	no	no	yes	no	yes
182273431	chr10	23303087	23303124	18	38	2.111111	no	no	yes	no	no	no	no	no	no	yes
182273433	chr10	23303478	23303556	21	79	3.904762	no	no	yes	no	no	no	no	no	no	yes
182273443	chr10	23319912	23319942	16	31	1.937500	no	no	yes	no	no	no	no	no	no	yes

182273453	chr10	23330950	23331001	23	52	2.304348	no	no	yes	no	no	no	no	yes	yes	yes
182273459	chr10	23331582	23331629	17	48	2.823529	no	no	yes	no	no	no	no	yes	yes	yes
182273467	chr10	23344149	23344181	17	33	1.941176	yes	no	yes	no	no	no	no	yes	yes	yes
182273482	chr10	23366414	23366493	26	80	3.076923	no	no	no	no	no	no	no	no	yes	no
182273497	chr10	23394761	23394799	20	39	1.950000	no	no	no	no	no	no	no	yes	yes	no
182273498	chr10	23394798	23394829	16	32	2.000000	no	no	no	no	no	no	no	yes	yes	no
182273502	chr10	23407223	23407258	16	36	2.250000	no	no	no	no	no	no	no	no	no	no
182273515	chr10	23419560	23419591	15	32	2.133333	no	no	no	no	no	no	no	no	no	no
182273525	chr10	23428292	23428344	18	53	2.944444	no	no	no	no	no	no	no	no	no	no
182273531	chr10	23441558	23441595	11	38	3.333333	no	yes	no	no	no	no	no	yes	no	yes
182273535	chr10	23449265	23449296	14	32	2.285714	no	no	yes	no	no	no	no	yes	no	yes
182273542	chr10	23457189	23457246	28	58	2.071429	no	no	yes	no	no	no	no	no	no	yes
182273557	chr10	23486943	23486987	22	45	2.045455	no	no	yes	no	no	no	no	no	no	yes
182273560	chr10	23487719	23487753	18	35	1.944444	no	no	yes	no	no	no	no	no	no	yes
182273572	chr10	23497660	23497708	20	49	2.550000	no	no	yes	no	no	no	no	no	no	yes
182273582	chr10	23518436	23518592	56	157	2.803571	no	no	yes	no	no	no	no	no	no	yes
182273584	chr10	23521913	23521937	13	25	1.923077	no	no	yes	no	no	no	no	yes	yes	yes
182273591	chr10	23542223	23542255	13	33	2.538461	no	no	no	no	no	no	no	no	yes	no
182273596	chr10	23554830	23554874	14	45	3.133333	no	no	no	no	no	no	no	no	no	no
182273607	chr10	23602431	23602495	22	65	2.954545	no	no	no	no	no	no	no	no	no	no
182273610	chr10	23610267	23610293	14	27	1.928571	no	no	no	no	no	no	no	no	no	no
182273611	chr10	23617115	23617167	25	53	2.200000	no	no	no	no	no	no	no	no	no	no
182273613	chr10	23617161	23617246	23	86	3.629630	no	no	no	no	no	no	no	no	no	no
182273619	chr10	23635191	23635233	22	43	1.954545	no	no	no	no	no	no	no	no	no	no
182273635	chr10	23687894	23687927	17	34	2.000000	no	no	no	no	no	no	no	no	no	no

182273636	chr10	23690835	23690873	18	39	2.166667	no	no	no	no	no	no	no	no	no	no
182273647	chr10	23718852	23718887	17	36	2.117647	no	no	yes	no	no	no	no	no	no	yes
182273678	chr10	23785827	23785864	15	38	2.500000	no	no	yes	no	no	no	no	no	no	yes
182273702	chr10	23818293	23818372	30	80	2.677419	no	no	yes	no	no	no	no	no	no	yes
182273728	chr10	23897768	23897810	17	43	2.588235	no	no	yes	no	no	no	no	no	no	yes
182273729	chr10	23899888	23899938	25	51	2.080000	no	no	yes	no	no	no	no	no	no	yes
182273746	chr10	23926793	23926823	15	31	2.066667	no	no	yes	no	no	no	no	yes	no	yes
182273752	chr10	23939877	23939913	18	37	2.111111	no	no	yes	no	no	no	no	no	no	yes
182273754	chr10	23939922	23939978	15	57	3.705882	no	no	yes	no	no	no	no	no	no	yes
182273758	chr10	23946275	23946355	35	81	2.314286	no	no	yes	no	no	no	no	no	no	yes
182273776	chr10	24014954	24014986	16	33	2.062500	no	no	yes	no	no	no	no	no	no	yes
182273790	chr10	24042239	24042272	17	34	2.000000	no	no	yes	no	yes	no	no	yes	no	yes
182273796	chr10	24070478	24070508	9	31	3.444444	no	no	yes	no	no	no	no	no	no	yes
182273801	chr10	24079982	24080018	19	37	1.947368	no	no	yes	no	no	no	no	no	no	yes
182273816	chr10	24106035	24106081	14	47	3.250000	no	no	yes	no	no	no	no	no	no	yes
182273820	chr10	24113236	24113278	21	43	2.095238	no	no	yes	no	no	no	no	no	no	yes
182273829	chr10	24161585	24161617	8	33	4.125000	no	no	yes	no	no	no	no	yes	no	yes
182273831	chr10	24166198	24166234	17	37	2.176471	no	no	yes	no	no	no	no	no	no	yes
182273851	chr10	24199927	24199955	14	29	2.071429	no	no	yes	no	no	no	no	no	no	yes
182273852	chr10	24200444	24200475	16	32	2.000000	no	no	yes	no	no	no	no	no	no	yes
182273879	chr10	24243653	24243830	91	178	1.956044	no	no	yes	no	no	no	no	no	no	yes
182273885	chr10	24258492	24258527	18	36	2.000000	no	no	yes	no	no	no	no	no	no	yes
182273896	chr10	24307937	24307975	20	39	1.950000	no	no	yes	no	no	no	no	no	yes	yes
182273901	chr10	24318153	24318185	16	33	2.187500	no	no	yes	no	no	no	no	no	no	yes
182273906	chr10	24326958	24326993	17	36	2.117647	no	no	yes	no	no	no	no	no	no	yes

182273916	chr10	24340579	24340624	19	46	2.421053	no	no	yes	no	no	no	no	no	yes	yes
182273917	chr10	24347956	24347991	12	36	3.083333	no	no	yes	no	no	no	no	no	no	yes
182273919	chr10	24354728	24354777	24	50	2.125000	no	no	yes	no	no	no	no	no	no	yes
182273930	chr10	24371560	24371593	18	34	1.944444	no	no	yes	no	no	no	no	yes	yes	yes
182273932	chr10	24382483	24382520	17	38	2.294118	no	no	yes	no	no	no	no	no	no	yes
182273956	chr10	24438904	24438945	20	42	2.100000	no	no	yes	no	no	no	no	yes	yes	yes
182273958	chr10	24453253	24453279	12	27	2.250000	no	no	yes	no	no	no	no	yes	no	yes
182273962	chr10	24462125	24462173	25	49	1.960000	no	no	yes	no	no	no	no	no	no	yes
182273980	chr10	24501067	24501101	16	35	2.187500	no	no	yes	no	no	no	no	no	no	yes
182273990	chr10	24524945	24525025	24	81	3.375000	no	no	yes	no	no	no	no	yes	yes	yes
182273998	chr10	24541882	24541974	35	93	2.657143	yes	no	yes	no	no	no	no	yes	yes	yes
182274001	chr10	24552546	24552595	20	50	2.500000	no	no	no	no	no	no	no	yes	yes	no
182274005	chr10	24557897	24557945	25	49	1.960000	no	no	no	no	no	no	no	no	no	no
182274006	chr10	24558459	24558493	16	35	2.187500	no	no	no	no	no	no	no	yes	yes	no
182274007	chr10	24560354	24560399	13	46	3.615385	no	no	no	no	no	no	no	no	no	no
182274015	chr10	24573643	24573843	50	201	3.962963	no	no	no	no	no	no	no	yes	no	no
182274019	chr10	24586505	24586561	27	57	2.111111	no	no	yes	no	no	no	no	no	no	yes
182274020	chr10	24587464	24587513	21	50	2.363636	no	no	yes	no	no	no	no	no	no	yes
182274022	chr10	24590736	24590765	14	30	2.142857	yes	no	yes	no	no	yes	no	no	no	yes
182274023	chr10	24591840	24591886	21	47	2.238095	no	no	yes	no	no	no	no	yes	no	yes
182274031	chr10	24627655	24627688	18	34	1.944444	no	no	yes	no	no	no	no	no	no	yes
182274037	chr10	24639869	24639912	20	44	2.200000	yes	no	yes	no	no	no	no	no	no	yes
182274069	chr10	24691772	24691808	20	37	1.900000	no	no	yes	no	no	no	no	no	no	yes
182274080	chr10	24708372	24708408	19	37	2.000000	no	no	yes	no	no	no	no	yes	no	yes
182274083	chr10	24710240	24710302	32	63	2.062500	no	no	yes	no	no	no	no	no	no	yes

182274086	chr10	24711154	24711192	20	39	1.950000	no	no	yes	no	no	no	no	no	no	yes
182274091	chr10	24723953	24723990	15	38	2.600000	yes	no	no	no	no	no	yes	yes	yes	no
182274101	chr10	24739981	24740039	26	59	2.269231	no	no	no	no	no	no	no	no	no	no
182274132	chr10	24767641	24767727	37	87	2.384615	no	no	no	no	no	no	no	no	no	no
182274156	chr10	24799780	24799820	21	41	1.952381	no	no	no	no	no	no	no	yes	no	no
182274168	chr10	24843789	24843831	11	43	4.090909	no	no	no	no	no	no	no	no	no	no
182274174	chr10	24857879	24857922	23	44	1.913043	no	no	yes	no	no	no	no	no	no	yes
182274175	chr10	24861141	24861182	23	42	1.913043	no	no	yes	no	no	no	no	no	no	yes
182274177	chr10	24865634	24865672	20	39	1.950000	no	no	yes	no	no	no	no	yes	no	yes
182274182	chr10	24866780	24866845	28	66	2.344828	yes	no	yes	no	no	no	no	yes	yes	yes
182274184	chr10	24866988	24867020	12	33	2.692308	yes	no	yes	no	no	no	no	yes	yes	yes
182274187	chr10	24872689	24872749	19	61	3.315789	no	no	yes	no	no	no	no	yes	no	yes
182274189	chr10	24875765	24875806	22	42	1.909091	no	no	yes	no	no	no	no	no	no	yes
182274196	chr10	24883015	24883066	28	52	1.964286	no	no	yes	no	no	no	no	no	no	yes
182274197	chr10	24886907	24886939	16	33	2.125000	no	no	yes	no	no	no	no	no	no	yes
182274200	chr10	24893257	24893308	18	52	2.888889	no	no	yes	no	no	no	no	yes	yes	yes
182274204	chr10	24897298	24897330	12	33	2.750000	no	no	yes	no	no	no	no	yes	yes	yes
182274205	chr10	24899967	24900002	18	36	2.000000	no	no	yes	no	no	no	no	yes	no	yes
182274206	chr10	24900645	24900684	18	40	2.222222	no	no	yes	no	no	no	no	yes	no	yes
182274209	chr10	24906184	24906214	11	31	2.818182	no	no	yes	no	no	no	no	no	no	yes
182274231	chr10	24958090	24958169	40	80	2.000000	no	no	yes	no	no	no	no	yes	yes	yes
182274235	chr10	24966029	24966072	23	44	2.000000	no	no	yes	no	no	no	no	no	no	yes
182274238	chr10	24967174	24967230	30	57	1.900000	no	no	yes	no	no	no	no	no	no	yes
182274241	chr10	24967738	24967779	17	42	2.470588	no	no	yes	no	no	no	no	yes	no	yes
182274249	chr10	24984102	24984146	21	45	2.142857	no	yes	yes	no	no	yes	no	no	yes	yes

182274254	chr10	24994212	24994259	22	48	2.272727	no	no	yes	no	no	no	no	yes	no	yes
182274259	chr10	24998268	24998307	18	40	2.277778	no	no	yes	no	no	no	no	yes	no	yes
182274271	chr10	25029855	25029891	19	37	1.947368	no	no	yes	no	no	no	no	yes	no	yes
182274275	chr10	25037703	25037728	12	26	2.166667	no	no	yes	no	no	no	no	yes	yes	yes
182274281	chr10	25064643	25064685	19	43	2.263158	no	no	yes	no	no	no	no	no	yes	yes
182274284	chr10	25073378	25073419	16	42	2.750000	no	no	yes	no	no	no	no	no	no	yes
182274287	chr10	25078095	25078135	13	41	3.142857	no	no	yes	no	no	no	no	no	no	yes
182274302	chr10	25115104	25115483	81	380	4.802469	no	no	yes	no	no	no	no	yes	no	yes
182274305	chr10	25131621	25131686	32	66	2.062500	no	no	yes	no	no	no	no	no	no	yes
182274320	chr10	25158992	25159022	16	31	1.937500	no	no	yes	no	yes	no	no	no	no	yes
182274331	chr10	25183078	25183119	22	42	1.909091	no	no	yes	no	no	no	no	no	yes	yes
182274333	chr10	25185221	25185264	15	44	2.937500	no	no	yes	no	no	no	no	no	no	yes
182274336	chr10	25191600	25191661	14	62	4.571429	no	no	yes	no	no	no	no	yes	no	yes
182274342	chr10	25213933	25213973	19	41	2.157895	no	no	yes	no	no	no	no	no	no	yes
182274345	chr10	25237899	25237950	26	52	2.115385	no	no	yes	no	no	no	no	yes	no	yes
182274350	chr10	25241994	25242033	22	40	1.909091	no	no	yes	no	no	no	no	no	no	yes
182274362	chr10	25306990	25307016	14	27	1.928571	no	no	yes	no	no	no	no	no	no	yes
182274379	chr10	25328172	25328215	10	44	4.400000	no	no	yes	no	no	no	no	no	no	yes
182274380	chr10	25333960	25334008	24	49	2.041667	no	no	yes	no	no	no	no	no	no	yes
182274391	chr10	25352534	25352575	17	42	2.470588	no	no	yes	no	no	no	no	yes	no	yes
182274393	chr10	25365243	25365295	27	53	1.962963	no	no	yes	no	no	no	no	no	no	yes
182274404	chr10	25423063	25423104	22	42	1.909091	no	no	yes	no	no	no	no	no	no	yes
182274410	chr10	25441188	25441216	13	29	2.230769	no	no	yes	no	no	no	no	no	no	yes
182274413	chr10	25444394	25444471	33	78	2.441176	no	no	yes	no	no	no	no	yes	no	yes
182274415	chr10	25444513	25444667	77	155	2.025974	no	no	yes	no	no	no	no	yes	no	yes

182274424	chr10	25466308	25466344	18	37	2.055556	no	yes	yes	no	no	no	no	yes	no	yes
182274428	chr10	25500090	25500150	19	61	3.210526	no	no	yes	no	no	no	no	no	no	yes
182274434	chr10	25536197	25536244	25	48	1.960000	no	no	yes	no	no	no	no	no	no	yes
182274439	chr10	25553298	25553328	16	31	1.937500	no	no	yes	no	no	no	no	no	no	yes
182274441	chr10	25555001	25555058	28	58	2.071429	no	no	yes	no	no	no	no	no	no	yes
182274454	chr10	25590663	25590697	18	35	1.944444	no	no	yes	no	no	no	no	yes	no	yes
182274455	chr10	25593790	25593822	17	33	1.941176	yes	no	yes	no	no	no	no	no	no	yes
182274459	chr10	25596508	25596589	30	82	2.733333	no	no	yes	no	no	no	no	no	no	yes
182274461	chr10	25597399	25597615	119	217	1.823529	no	no	yes	no	no	no	no	yes	no	yes
182274467	chr10	25629317	25629361	21	45	2.142857	no	no	no	no	no	no	no	yes	no	no
182274472	chr10	25641250	25641283	16	34	2.125000	no	no	no	no	no	no	no	no	no	no
182274474	chr10	25652603	25652634	16	32	2.000000	yes	no	yes	no	no	no	no	yes	no	yes
182274494	chr10	25688919	25688996	42	78	1.904762	no	no	yes	no	no	yes	no	yes	no	yes
182274496	chr10	25690038	25690094	30	57	1.966667	no	no	yes	no	no	no	no	no	no	yes
182274498	chr10	25696232	25696415	91	184	2.021978	no	no	yes	no	no	no	no	yes	yes	yes
182274500	chr10	25697039	25697070	15	32	2.133333	no	no	yes	no	no	no	no	yes	no	yes
182274523	chr10	25726417	25726477	31	61	2.032258	no	no	yes	no	no	no	no	yes	no	yes
182274534	chr10	25741115	25741199	41	85	2.121951	no	no	no	no	no	no	no	no	no	no
182274545	chr10	25753894	25753920	8	27	3.375000	no	no	no	no	no	no	no	yes	yes	no
182274549	chr10	25765284	25765371	46	88	1.913043	no	no	no	no	no	no	no	no	no	no
182274563	chr10	25791307	25791345	14	39	2.857143	no	no	no	no	no	no	no	no	no	no
182274566	chr10	25791425	25791462	16	38	2.375000	no	no	no	no	no	no	no	no	no	no
182274572	chr10	25802175	25802207	17	33	1.941176	no	no	no	no	no	no	no	no	no	no
182274573	chr10	25803868	25803900	16	33	2.062500	no	no	no	no	no	no	no	no	no	no
182274591	chr10	25876353	25876488	70	136	1.942857	no	no	no	no	no	no	no	yes	no	no

182274596	chr10	25886009	25886033	11	25	2.272727	no	no	no	no	no	no	no	no	no	no
182274600	chr10	25892644	25892676	12	33	2.750000	no	no	no	no	no	no	no	no	no	no
182274603	chr10	25913791	25913836	24	46	1.916667	no	no	no	no	no	no	no	no	no	no
182274604	chr10	25913824	25913886	30	63	2.166667	no	no	no	no	no	no	no	no	no	no
182274608	chr10	25918191	25918227	18	37	2.055556	no	no	no	no	no	no	no	no	no	no
182274626	chr10	25964351	25964379	11	29	2.636364	no	no	yes	no	no	no	no	no	no	yes
182274639	chr10	26030779	26030810	11	32	2.909091	no	no	yes	no	no	no	no	yes	no	yes
182274643	chr10	26034822	26034878	18	57	3.210526	no	no	yes	no	no	no	no	no	no	yes
182274652	chr10	26050530	26050566	18	37	2.055556	no	no	yes	no	no	no	no	no	no	yes
182274656	chr10	26051574	26051639	32	66	2.125000	no	no	yes	no	no	no	no	no	no	yes
182274667	chr10	26075580	26075859	28	280	10.258064	no	no	yes	no	no	no	no	no	no	yes
182274674	chr10	26108218	26108266	23	49	2.130435	no	no	yes	no	no	no	no	no	no	yes
182274675	chr10	26112857	26112902	20	46	2.400000	no	no	yes	no	no	no	no	yes	no	yes
182274677	chr10	26114084	26114122	16	39	2.437500	no	no	yes	no	no	no	no	no	yes	yes
182274678	chr10	26122677	26122737	29	61	2.172414	no	no	yes	no	no	no	no	no	no	yes
182274682	chr10	26124864	26124911	20	48	2.380952	no	no	yes	no	no	no	no	no	no	yes
182274683	chr10	26146335	26146359	10	25	2.500000	no	no	yes	no	no	no	no	yes	no	yes
182274684	chr10	26147232	26147294	21	63	3.000000	no	no	yes	no	no	no	no	no	no	yes
182274695	chr10	26174853	26174877	12	25	2.083333	no	no	yes	no	no	no	no	no	no	yes
182274696	chr10	26175191	26175248	20	58	2.900000	no	no	yes	no	no	no	no	yes	no	yes
182274707	chr10	26211550	26211578	15	29	2.000000	no	no	yes	no	no	no	no	yes	no	yes
182274710	chr10	26230242	26230281	16	40	2.500000	no	no	yes	no	no	no	no	no	no	yes
182274727	chr10	26281683	26281709	11	27	2.454545	no	no	yes	no	no	no	no	no	no	yes
182274730	chr10	26303215	26303246	15	32	2.200000	no	yes	yes	no	no	no	no	no	no	yes
182274744	chr10	26328071	26328099	15	29	1.933333	no	no	no	no	no	no	no	no	no	no

182274748	chr10	26331267	26331319	18	53	2.947368	no	no	no	no	no	no	no	yes	yes	no
182274771	chr10	26393486	26393645	47	160	3.437500	no	no	no	no	no	no	no	no	no	no
182274775	chr10	26397932	26397964	17	33	1.941176	no	no	no	no	no	no	no	no	no	no
182274778	chr10	26402604	26402632	11	29	2.636364	no	no	no	no	no	no	no	no	no	no
182274784	chr10	26413604	26413641	20	38	1.900000	no	no	no	no	no	no	no	yes	yes	no
182274790	chr10	26426038	26426085	25	48	1.960000	no	no	no	no	no	no	no	yes	no	no
182274795	chr10	26439194	26439226	8	33	4.125000	no	no	yes	no	no	no	no	yes	yes	yes
182274796	chr10	26440095	26440125	15	31	2.066667	no	no	yes	no	no	no	no	yes	no	yes
182274802	chr10	26450340	26450426	41	87	2.146342	no	no	yes	no	no	no	no	no	no	yes
182274803	chr10	26453102	26453145	22	44	2.136364	no	no	yes	no	no	no	no	no	no	yes
182274813	chr10	26469562	26469599	13	38	2.866667	no	no	yes	no	no	no	no	no	no	yes
182274815	chr10	26470799	26470905	36	107	2.972222	no	no	yes	no	no	no	no	yes	yes	yes
182274821	chr10	26479428	26479461	16	34	2.125000	no	no	yes	no	no	no	no	no	yes	yes
182274827	chr10	26486074	26486118	18	45	2.526316	no	no	yes	no	no	no	no	no	no	yes
182274828	chr10	26487577	26487606	13	30	2.384615	no	no	yes	no	no	no	no	yes	yes	yes
182274839	chr10	26508574	26508634	28	61	2.178571	yes	no	yes	no	no	no	no	yes	no	yes
182274842	chr10	26515723	26515886	33	164	4.969697	no	no	yes	no	no	no	no	no	yes	yes
182274845	chr10	26526249	26526343	37	95	2.567568	no	no	yes	no	no	no	no	no	no	yes
182274848	chr10	26534433	26534469	17	37	2.176471	no	no	yes	no	no	no	no	yes	no	yes
182274852	chr10	26539419	26539514	41	96	2.341463	no	no	yes	no	no	no	no	no	no	yes
182274871	chr10	26567197	26567248	21	52	2.476191	no	yes	no	yes	no	yes	yes	yes	yes	yes
182274874	chr10	26577032	26577075	20	44	2.200000	no	no	no	no	no	yes	no	no	no	no
182274883	chr10	26581087	26586813	1842	5727	3.084595	no	no	yes	no	no	no	no	yes	yes	yes
182274887	chr10	26594300	26594324	13	25	1.923077	no	yes	yes	no	no	yes	no	no	no	yes
182274906	chr10	26642937	26643014	17	78	4.647059	yes	no	no	no	no	no	no	yes	no	no

182274911	chr10	26644701	26648308	1837	3608	1.995645	yes	yes	yes	no	no	yes	no	yes	yes	yes
182274918	chr10	26660011	26660066	23	56	2.521739	no	no	no	no	no	no	no	no	no	no
182274926	chr10	26674944	26675041	48	98	2.041667	no	no	yes	no	no	no	no	yes	yes	yes
182274934	chr10	26690972	26691013	12	42	3.500000	no	no	yes	no	no	no	no	yes	yes	yes
182274935	chr10	26697952	26697979	11	28	2.545455	yes	no	yes	no	no	no	yes	yes	yes	yes
182274936	chr10	26699964	26700022	30	59	1.966667	no	no	yes	no	no	no	no	yes	yes	yes
182274937	chr10	26703598	26703624	14	27	1.928571	no	no	yes	no	no	no	no	no	no	yes
182274941	chr10	26720932	26720972	20	41	2.050000	no	no	yes	no	no	no	no	no	yes	yes
182274943	chr10	26723421	26723654	78	234	2.987500	no	no	yes	no	no	no	no	yes	no	yes
182274954	chr10	26742949	26742977	12	29	2.416667	no	no	yes	no	no	no	no	yes	yes	yes
182274958	chr10	26749235	26749272	20	38	1.950000	no	no	yes	no	no	no	no	no	no	yes
182274960	chr10	26754157	26754193	18	37	2.111111	no	no	yes	no	no	no	no	no	no	yes
182274967	chr10	26769338	26769384	23	47	2.086957	no	no	yes	no	no	no	no	no	no	yes
182274974	chr10	26803937	26804008	37	72	1.945946	no	no	yes	no	no	no	no	yes	no	yes
182274977	chr10	26811722	26811855	32	134	4.500000	no	no	yes	no	no	no	no	no	no	yes
182274990	chr10	26846209	26846266	23	58	2.608696	no	no	yes	no	no	no	no	no	no	yes
182274993	chr10	26854445	26854484	20	40	2.000000	no	no	yes	no	no	no	no	yes	no	yes
182274998	chr10	26860600	26860641	12	42	3.500000	no	no	yes	no	no	no	yes	yes	yes	yes
182275012	chr10	26876373	26876401	12	29	2.416667	no	no	no	no	no	no	no	no	no	no
182275015	chr10	26878105	26878161	24	57	2.500000	no	no	no	no	no	no	no	no	no	no
182275017	chr10	26885977	26886010	16	34	2.187500	no	no	no	no	no	no	no	yes	no	no
182275024	chr10	26891147	26891301	84	155	1.880952	no	no	no	no	no	no	no	yes	yes	no
182275034	chr10	26911589	26911637	23	49	2.173913	no	no	no	no	no	no	no	no	no	no
182275035	chr10	26913925	26913965	18	41	2.333333	no	no	no	no	no	no	no	yes	no	no
[truncated: 595,853 more chars]
